# A comprehensive meta-analysis on safety outcomes reveals the novel potentials of SGLT2is, especially preventing respiratory diseases

**DOI:** 10.3389/fendo.2024.1376446

**Published:** 2024-04-29

**Authors:** YuJun Cai, HaiTao Zou, Rong Hu, Hao Chen, GuoHuan Yang, LiDan Gong

**Affiliations:** ^1^ Department of Respiratory and Critical Care Medicine, Dianjiang People’s Hospital of Chongqing, Chongqing, China; ^2^ Department of Pharmacy, Dianjiang People’s Hospital of Chongqing, Chongqing, China

**Keywords:** SGLT2is, respiratory diseases, NSCLC, anemia, back pain, gastrointestinal diseases

## Background

Due to the cardiovascular and renal benefits of sodium-glucose cotransporter-2 inhibitors (SGLT2is) in patients with type 2 diabetes (T2D), those with heart failure (HF), and those with chronic kidney disease (CKD), SGLT2is have been recommended in these populations to prevent cardiorenal events (1–3). Two recent reviews (4, 5) mainly summarizing the results of mechanistic studies have reported that, apart from exerting the glucose-lowering and cardiorenal protection effects, SGLT2is can also decrease oxidative stress and endoplasmic reticulum stress, diminish proinflammatory and profibrotic pathways, stimulate mitochondrial biogenesis, restore mitochondrial health, and regulate the mTOR pathway. The aforementioned biological activities of SGLT2is seem to suggest the possibility that this drug class could be used for the prevention/treatment of other diseases except T2D, HF, and CKD. A comprehensive meta-analysis conducted by Qiu et al. (6), including a total of nine large-scale randomized controlled trials (RCTs) of SGLT2is, has evaluated 1,009 safety outcomes from 24 different body systems and, accordingly, has revealed some novel potentials of SGLT2is, such as the possible benefits of SGLT2is against some respiratory diseases including chronic obstructive pulmonary disease (COPD), asthma, and pneumonia. Nowadays, a great number of novel large-scale RCTs of SGLT2is are available, such as NCT03594110 [EMPA-KIDNEY] (7), NCT03619213 [DELIVER] (8), NCT03057951 [EMPEROR-Preserved] (9), NCT03521934 [SOLOIST-WHF] (10), and NCT03315143 [SCORED] (11). Therefore, we have carried out an updated meta-analysis to confirm the findings of Qiu et al. and examine the possibility of the novel applications of SGLT2is.

## Methods

This meta-analysis was performed according to the Preferred Reporting Items for Systematic Reviews and Meta-Analyses (PRISMA) statement (12). PubMed and ClinicalTrials.gov were searched to identify relevant papers published before September 8, 2023. We searched PubMed using the following search strategy: (Sodium-Glucose Transporter 2 Inhibitors[Mesh] OR “Sodium glucose transporter 2 inhibitor*”[TIAB] OR “Sodium glucose cotransporter 2 inhibitor*”[TIAB] OR “Sodium glucose co-transporter 2 inhibitor*”[TIAB] OR SGLT2*[TIAB] OR Gliflozin*[tiab] OR “Empagliflozin”[tiab] OR “Empagliflozin”[Supplementary Concept] OR “Dapagliflozin”[tiab] OR “Dapagliflozin”[Supplementary Concept] OR “Canagliflozin”[Mesh] OR “Canagliflozin”[tiab] OR “ertugliflozin”[tiab] OR “ertugliflozin”[Supplementary Concept] OR “sotagliflozin”[tiab] OR “LX4211”[tiab]) AND ((randomized controlled trial [pt] OR controlled clinical trial [pt] OR randomized [tiab] OR randomly [tiab] OR drug therapy [sh] OR placebo [tiab] OR trial [tiab] OR groups [tiab]) NOT (animals [mh] NOT humans [mh])).

In this meta-analysis, we included those RCTs that enrolled >500 subjects, compared SGLT2is with placebo or a non-SGLT2is drug, and reported various serious adverse events (SAEs) regarding various body systems. The outcomes of interest were the various SAEs which included trials reported at the website of ClinicalTrials.gov as long as they were reported by at least four of the included trials. We collected all of the outcome data from ClinicalTrials.gov. We conducted meta-analyses on eligible outcomes using a random-effects model (when *I* ^2^ ≥50%) or a fixed-effects model (when *I* ^2^ <50%). Risk ratio (RR) with its 95% confidence interval (CI) was regarded as effect size. *P <*0.05 was considered as statistically significant. All statistical analyses were done using the Stata/MP 16.0 software.

## Results

As shown in **Supplementary Figure S1** (flow diagram), we included a total of 26 papers reporting 27 large RCTs in total. These included trials that had their only ClinicalTrials.gov Identifiers: NCT03594110 [EMPA-KIDNEY] (7), NCT03619213 [DELIVER] (8), NCT03057951 [EMPEROR-Preserved] (9), NCT03521934 [SOLOIST-WHF] (10), NCT03315143 [SCORED] (11), NCT03242252 (13), NCT02033889 (14), NCT04157751 (15), NCT02384941 (16), NCT01042977 (17), NCT01031680 (18), NCT01164501 (19), NCT01106677 (20), NCT01177813 (21), NCT01106651 (22), NCT00528879 (23), NCT04350593 (24), NCT00673231 (25), NCT02065791 (26), NCT01032629 [CANVAS] (27), NCT01989754 [CANVAS-R] (27), NCT03057977 (28), NCT03036150 (29), NCT01986881 (30), NCT01131676 (31), NCT03036124 (32), and NCT01730534 (33). The detailed characteristics of the included 27 trials are provided in **Supplementary Table S1**, and these trials involved 100,995 subjects in total. We performed meta-analyses on a total of 1,080 kinds of diseases deriving from 25 kinds of body systems (**Supplementary Table S2**): blood and lymphatic system disorders (involving 17 diseases), cardiac disorders (involving 79 diseases), congenital, familial, and genetic disorders (involving two diseases), ear and labyrinth disorders (involving nine diseases), endocrine disorders (involving six diseases), eye disorders (involving 20 diseases), gastrointestinal disorders (involving 104 diseases), general disorders (involving 31 diseases), hepatobiliary disorders (involving 27 diseases), immune system disorders (involving four diseases), infections and infestations (involving 158 diseases), injury, poisoning, and procedural complications (involving 102 diseases), investigations (involving 16 diseases), metabolism and nutrition disorders (involving 32 diseases), musculoskeletal and connective tissue disorders (involving 51 diseases), benign, malignant, and unspecified neoplasms (involving 131 diseases), nervous system disorders (involving 100 diseases), product issues (involving five diseases), psychiatric disorders (involving 12 diseases), renal and urinary disorders (involving 39 diseases), reproductive system and breast disorders (involving 15 diseases), respiratory, thoracic, and mediastinal disorders (involving 47 diseases), skin and subcutaneous tissue disorders (involving 15 diseases), surgical and medical procedures (involving four diseases), and vascular disorders (involving 54 diseases).

**Figure 1:**
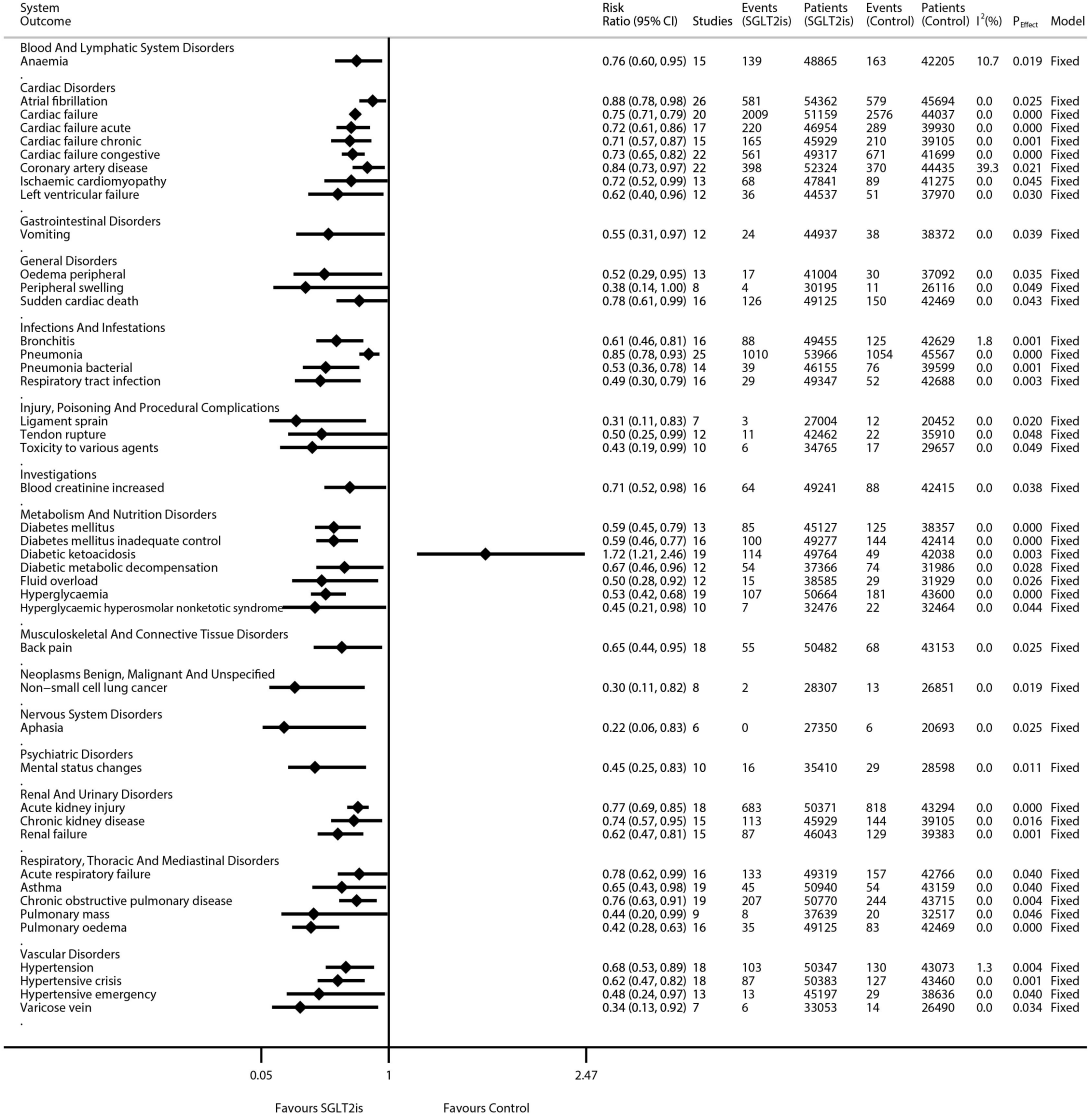
Meta-analyses of SGLT2is and 44 kinds of diseases (statistically significant findings).

The results of the meta-analyses on 1,080 kinds of diseases are detailed in **Supplementary Figure S2** (forest plots) and are summarized in **Supplementary Table S2**. Moreover, the statistically significant results were extracted and collated into **Figure 1**. SGLT2is were significantly associated with the decreased risks of eight kinds of cardiac disorders [e.g., atrial fibrillation (RR 0.88, 95% CI 0.78–0.98; *P* = 0.025; *N* of studies = 26; *I* ^2^ = 0.0%), cardiac failure (RR 0.75, 95% CI 0.71–0.79; *P* = 0.000; *N* of studies = 20; *I* ^2^ = 0.0%), and coronary artery disease (RR 0.84, 95% CI 0.73–0.97; *P* = 0.021; *N* of studies = 22; *I* ^2^ = 39.3%)], four kinds of vascular disorders [e.g., hypertension (RR 0.68, 95% CI 0.53–0.89; *P* = 0.004; *N* of studies = 18; *I* ^2^ = 1.3%), hypertensive crisis (RR 0.62, 95% CI 0.47–0.82; *P* = 0.001; *N* of studies = 18; *I* ^2^ = 0.0%), and hypertensive emergency (RR 0.48, 95% CI 0.24–0.97; *P* = 0.040; *N* of studies = 13; *I* ^2^ = 0.0%)], three kinds of renal disorders [e.g., acute kidney injury (RR 0.77, 95% CI 0.69–0.85; *P* = 0.000; *N* of studies = 18; *I* ^2^ = 0.0%) and CKD (RR 0.74, 95% CI 0.57–0.95; *P* = 0.016; *N* of studies = 15; *I* ^2^ = 0.0%)], and six kinds of metabolism disorders [e.g., diabetes mellitus (RR 0.59, 95% CI 0.45–0.79; *P* = 0.000; *N* of studies = 13; *I* ^2^ = 0.0%), hyperglycemia (RR 0.53, 95% CI 0.42–0.68; *P* = 0.000; *N* of studies = 19; *I* ^2^ = 0.0%), and hyperglycemic hyperosmolar nonketotic syndrome (RR 0.45, 95% CI 0.21–0.98; *P* = 0.044; *N* of studies = 10; *I* ^2^ = 0.0%)]. SGLT2is were significantly associated with the decreased risks of five kinds of respiratory disorders [e.g., acute respiratory failure (RR 0.78, 95% CI 0.62–0.99; *P* = 0.040; *N* of studies = 16; *I* ^2^ = 0.0%), asthma (RR 0.65, 95% CI 0.43–0.98; *P* = 0.040; *N* of studies = 19; *I* ^2^ = 0.0%), and COPD (RR 0.76, 95% CI 0.63–0.91; *P* = 0.004; *N* of studies = 19; *I* ^2^ = 0.0%)], and four kinds of infectious disorders [e.g., bronchitis (RR 0.61, 95% CI 0.46–0.81; *P* = 0.001; *N* of studies = 16; *I* ^2^ = 1.8%) and pneumonia (RR 0.85, 95% CI 0.78–0.93; *P* = 0.000; *N* of studies = 25; *I* ^2^ = 0.0%)]. SGLT2is were significantly associated with decreased risks of non-small cell lung cancer (NSCLC) (RR 0.30, 95% CI 0.11–0.82; *P* = 0.019; *N* of studies = 8; *I* ^2^ = 0.0%), aphasia (RR 0.22, 95% CI 0.06–0.83; *P* = 0.025; *N* of studies = 6; *I* ^2^ = 0.0%), mental status changes (RR 0.45, 95% CI 0.25–0.83; *P* = 0.011; *N* of studies = 10; *I* ^2^ = 0.0%), anemia (RR 0.76, 95% CI 0.60–0.95; *P* = 0.019; *N* of studies = 15; *I* ^2^ = 10.7%), and back pain (RR 0.65, 95% CI 0.44–0.95; *P* = 0.025; *N* of studies = 18; *I* ^2^ = 0.0%). SGLT2is were significantly associated with an increased risk of diabetic ketoacidosis (RR 1.72, 95% CI 1.21–2.46; *P* = 0.003; *N* of studies = 19; *I* ^2^ = 0.0%). Moreover, SGLT2is were not significantly associated with the incidences of 1,036 kinds of diseases, e.g., intestinal obstruction (RR 0.91, 95% CI 0.53–1.54; *P* = 0.716; *N* of studies = 17; *I* ^2^ = 0.0%), acute pancreatitis (RR 0.92, 95% CI 0.63–1.34; *P* = 0.676; *N* of studies = 17; *I* ^2^ = 0.0%), gastrointestinal hemorrhage (RR 0.88, 95% CI 0.67–1.16; *P* = 0.376; *N* of studies = 16; *I* ^2^ = 0.0%), upper gastrointestinal hemorrhage (RR 0.75, 95% CI 0.50–1.12; *P* = 0.161; *N* of studies = 14; *I* ^2^ = 0.5%), ischemic colitis (RR 1.11, 95% CI 0.53–2.36; *P* = 0.777; *N* of studies = 13; *I* ^2^ = 0.0%), and gastric ulcer (RR 0.95, 95% CI 0.57–1.61; *P* = 0.859; *N* of studies = 13; *I* ^2^ = 0%).

## Discussion

Up to now, this is the most comprehensive meta-analysis that assessed the safety outcomes of SGLT2is, given assessing 1,080 kinds of SAEs deriving from 25 kinds of body systems in total. Accordingly, we produced several key findings as follows: First, SGLT2is were significantly associated with the decreased risks of several kinds of cardiovascular disorders (e.g., cardiac failure, atrial fibrillation, and hypertensive crisis), renal disorders (e.g., acute kidney injury, and CKD), and metabolism disorders (e.g., diabetes mellitus, and hyperglycemia), but with an increased risk of diabetic ketoacidosis. These findings again confirm the known characteristics of SGLT2is in such a way that SGLT2is can exert the protective effects against relevant cardiorenal diseases and T2D but lead to common adverse effects, i.e., diabetic ketoacidosis. Second, SGLT2is were significantly associated with the decreased risks of several kinds of respiratory disorders (e.g., acute respiratory failure, asthma, and COPD), and infectious disorders (e.g., bronchitis and pneumonia). Similarly, a cohort study (34) showed that SGLT2is had a significant association with the lower risks of adverse respiratory events including respiratory failure, pulmonary edema, and pneumonia. These findings may suggest that SGLT2is could be used for the prevention of relevant respiratory and infectious diseases. Third, SGLT2is were significantly associated with a lower incidence of NSCLC. Similarly, two recent studies (35, 36) showed the benefits of SGLT2is against NSCLC. These may suggest a new application area of SGLT2is: as an adjuvant drug for the treatment of NSCLC. Fourth, our meta-analysis identified that SGLT2is were associated with lower incidences of aphasia and mental status changes. Meanwhile, Liebers et al. proposed the hypothesis that ketogenesis induced by SGLT2is could be an effective treatment for depressive disorders (37). Fifth, our meta-analysis identified that SGLT2is were associated with a lower incidence of anemia. Meanwhile, a newly published article (38) illustrated the possible mechanisms for the alleviation of anemia by SGLT2is. Sixth, our meta-analysis identified that SGLT2is were associated with a lower incidence of back pain, which may be due to the anti-inflammation and anti-oxidative stress activities of SGLT2is. Lastly, in this meta-analysis SGLT2is were not observed to have an association with the incidences of 1,036 kinds of diseases including various gastrointestinal diseases (e.g., intestinal obstruction, pancreatitis acute, and upper gastrointestinal hemorrhage). These findings suggest that SGLT2is are generally safe for various body systems, including the gastrointestinal system.

Compared with the meta-analysis of Qiu et al. (6), including nine trials involving a total of 33,124 subjects, our meta-analysis included more trials (27 trials) and involved more subjects (100,995 subjects). This suggested that our meta-analysis had a more sufficient statistical power to produce more credible findings. Moreover, Qiu et al. performed analyses only on the outcomes that were reported by at least three of the included trials, whereas we performed analyses only on the outcomes that were reported by at least four of the included trials. This suggested that we further reduced the possibility of the bias of meta-analysis results deriving from the limited number of included studies. On the contrary, this meta-analysis has two main weaknesses. First, just like the meta-analysis of Qiu et al. (6), our meta-analysis also evaluated a great number of outcomes, and therefore it was likely to lead to false positive findings. Second, the outcomes we performed analyses on in this study were the various SAEs reported by the included trials, not the primary outcomes of the included trials. Therefore, our findings in this study should be considered as exploratory but not confirmatory, and further verification is needed.

In conclusion, our meta-analysis confirms the benefits of SGLT2is against relevant cardiorenal diseases and T2D again, reveals the general safety of SGLT2is for various body systems including the gastrointestinal system, and, more importantly, suggests some novel potentials of SGLT2is: for the prevention/treatment of relevant respiratory and infectious diseases, NSCLC, depressive disorders, anemia, and back pain.

## Author contributions

YC: Conceptualization, Formal analysis, Writing – original draft, Writing – review & editing. HZ: Formal analysis, Writing – original draft, Writing – review & editing. RH: Formal analysis, Writing – original draft, Writing – review & editing. HC: Data curation, Writing – original draft, Writing – review & editing. GY: Data curation, Writing – original draft, Writing – review & editing. LG: Conceptualization, Writing – original draft, Writing – review & editing.

## Supplementary material

Supplementary Table 2 Meta-analyses of SGLT2is and 1,080 kinds of diseases.
