# Supplementary material for: A comprehensive meta-analysis on safety outcomes reveals the novel potentials of SGLT2is, especially preventing respiratory diseases
Source: Front Endocrinol (Lausanne). 2024 Apr 29;15:1376446. doi: 10.3389/fendo.2024.1376446 (PMC11089104; doi:10.3389/fendo.2024.1376446)
Supplement: Supplementary Table 2 — Meta-analyses of SGLT2is and 1,080 kinds of diseases. [file DataSheet_1.docx]

Full-text articles excluded (n = 233):

Sample size (n = 115)

Study type (n = 61)

Control (n = 39)

Intervention (n = 18)

Outcome (n = 0)

26 articles reporting 27 trials included in quantitative synthesis

(meta-analysis)

26 articles reporting 27 trials included in qualitative synthesis

(n = 21)

Full-text articles assessed for eligibility
(n = 259)

Records excluded
(n = 7912)

Records screened
(n = 8171)

Records after duplicates removed
(n = 8171)

Additional records identified through other sources
(n = 0)

## Identification

## Eligibility

## Included

## Screening

Records identified through database searching
(n = 8171)

Flow diagram of study selection
