# Supplementary material for: A comprehensive meta-analysis on safety outcomes reveals the novel potentials of SGLT2is, especially preventing respiratory diseases
Source: Front Endocrinol (Lausanne). 2024 Apr 29;15:1376446. doi: 10.3389/fendo.2024.1376446 (PMC11089104; doi:10.3389/fendo.2024.1376446)
Supplement: Supplementary file 2 [file DataSheet_2.pdf]

Trial	SGLT2is	Control	Risk Ratio (95% CI)	Events, Treatment	Events, Control	% Weight
NCT01032629	Canagliflozin	Placebo	0.56 (0.22, 1.45)	9/2886	8/1441	6.07
NCT01131676	Empagliflozin	Placebo	0.62 (0.29, 1.33)	15/4687	12/2333	9.56
NCT01164501	Empagliflozin	Placebo	0.76 (0.05, 12.13)	1/419	1/319	0.72
NCT01730534	Dapagliflozin	Placebo	0.71 (0.40, 1.27)	20/8574	28/8569	16.70
NCT01986881	Ertugliflozin	Placebo	1.50 (0.41, 5.53)	9/5493	3/2745	3.22
NCT01989754	Canagliflozin	Placebo	1.67 (0.61, 4.58)	10/2904	6/2903	5.37
NCT02065791	Canagliflozin	Placebo	0.70 (0.27, 1.83)	7/2200	10/2197	5.90
NCT03036124	Dapagliflozin	Placebo	0.27 (0.08, 0.98)	3/2368	11/2368	3.37
NCT03036150	Dapagliflozin	Placebo	0.88 (0.32, 2.41)	7/2149	8/2149	5.35
NCT03057951	Empagliflozin	Placebo	0.63 (0.36, 1.12)	19/2996	30/2989	16.73
NCT03057977	Empagliflozin	Placebo	1.67 (0.61, 4.58)	10/1863	6/1863	5.37
NCT03315143	Sotagliflozin	Placebo	1.50 (0.42, 5.31)	6/5291	4/5286	3.43
NCT03521934	Sotagliflozin	Placebo	2.52 (0.49, 12.96)	5/605	2/611	2.05
NCT03594110	Empagliflozin	Placebo	0.20 (0.04, 0.91)	2/3304	10/3305	2.38
NCT03619213	Dapagliflozin	Placebo	0.67 (0.35, 1.25)	16/3126	24/3127	13.79
Overall (I-squared = 10.7%, p = 0.334)			0.76 (0.60, 0.95)	139/48865	163/42205	100.00

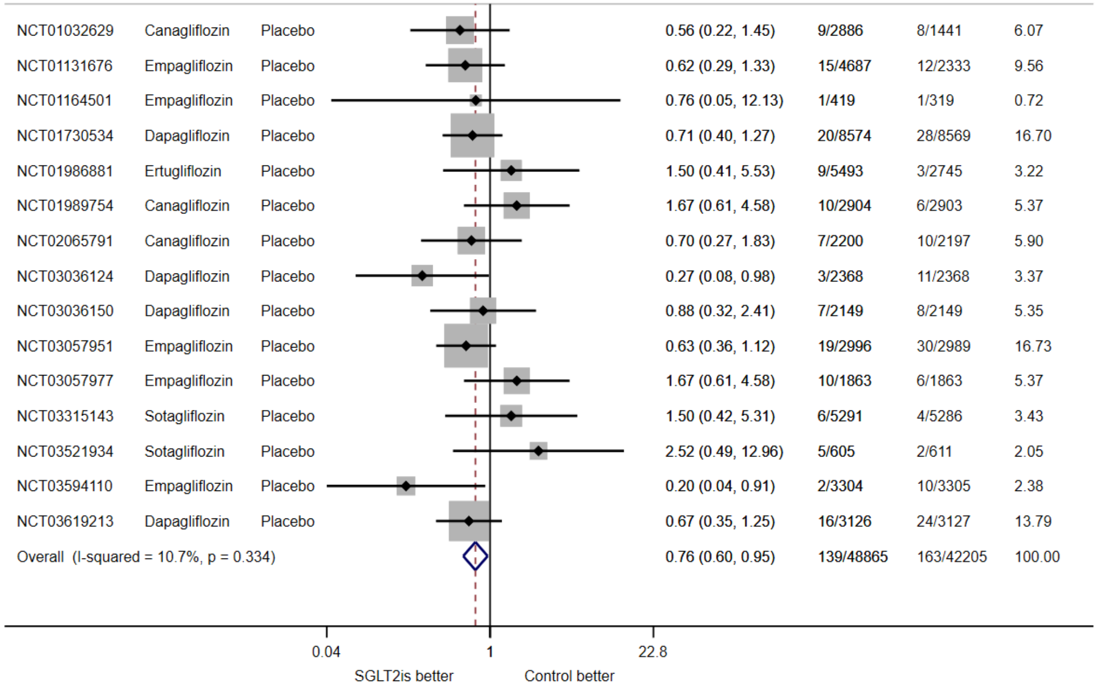

System 1-Blood And Lymphatic System Disorders-Figure S1 Meta-analysis of SGLT2is and Anaemia

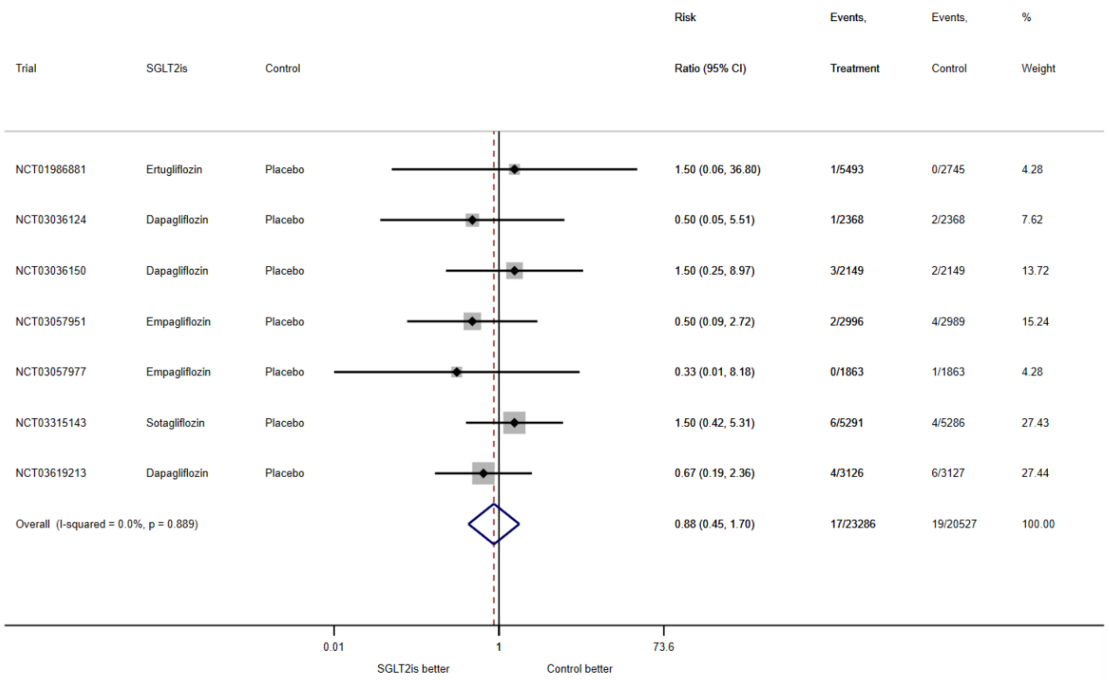

System 1-Blood And Lymphatic System Disorders-Figure S2 Meta-analysis of SGLT2is and Blood loss anaemia

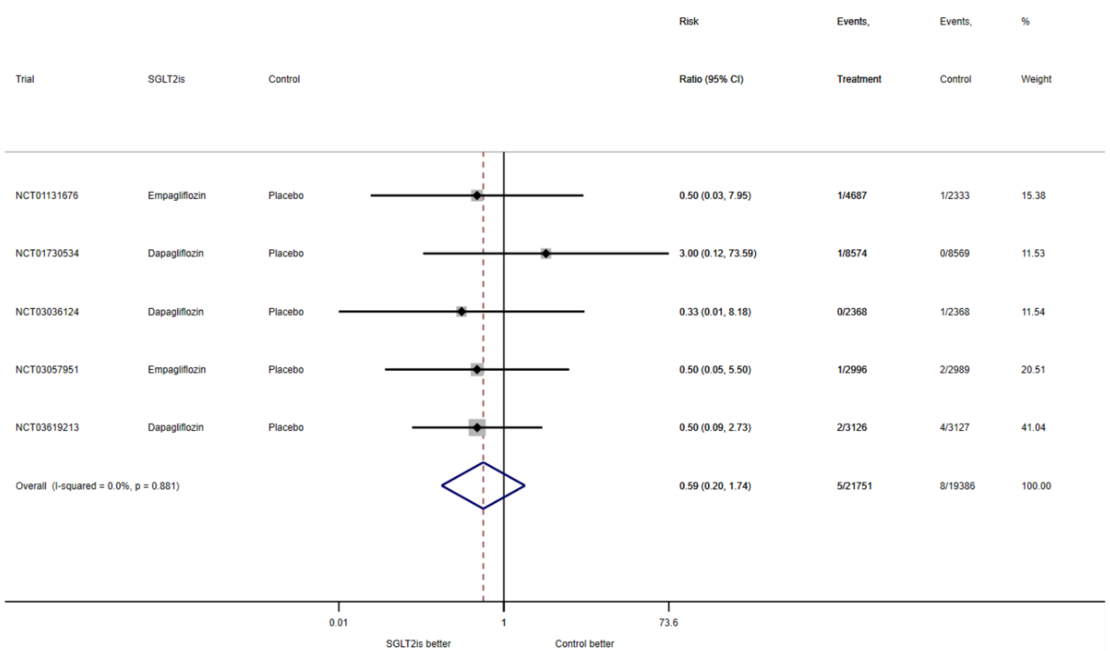

System 1-Blood And Lymphatic System Disorders-Figure S3 Meta-analysis of SGLT2is and Coagulopathy

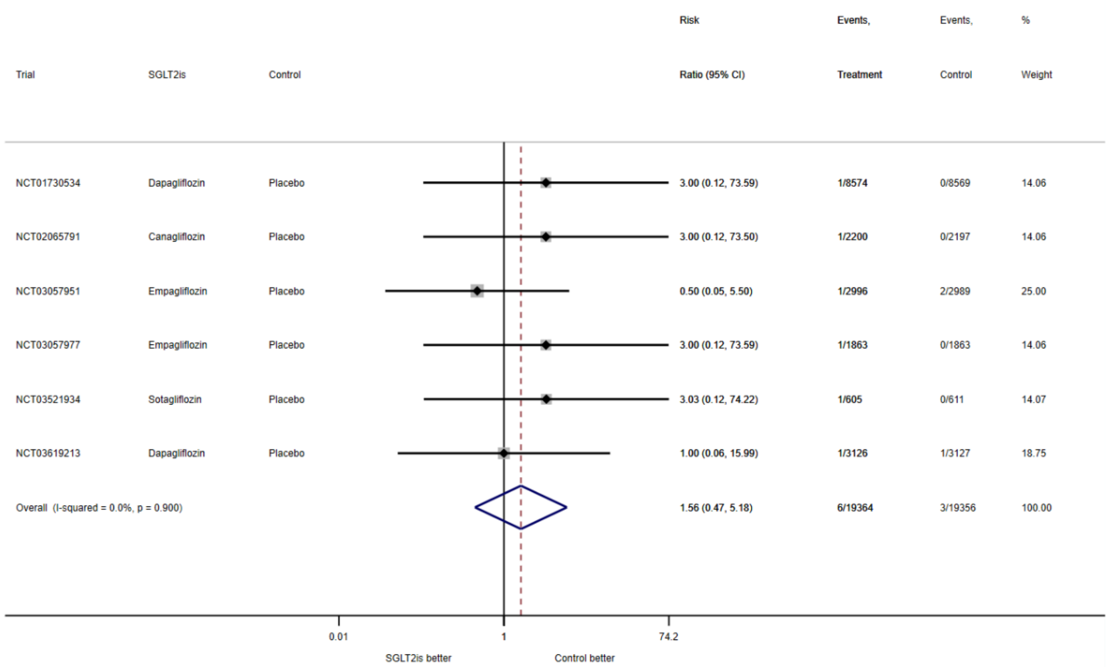

System 1-Blood And Lymphatic System Disorders-Figure S4 Meta-analysis of SGLT2is and Disseminated intravascular coagulation

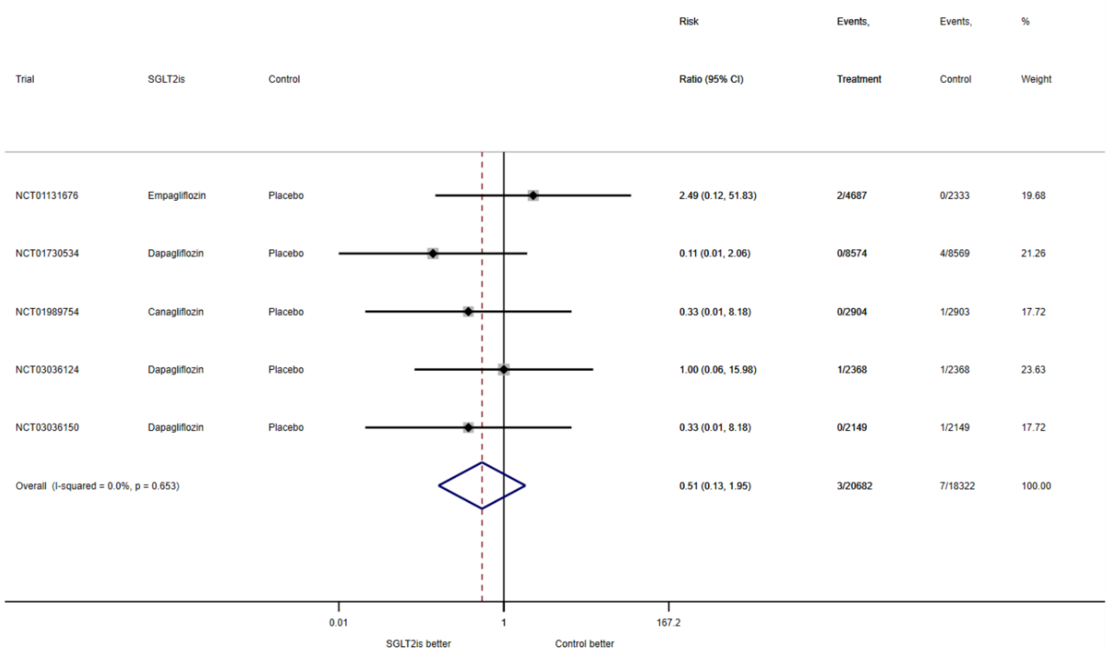

System 1-Blood And Lymphatic System Disorders-Figure S5 Meta-analysis of SGLT2is and Febrile neutropenia

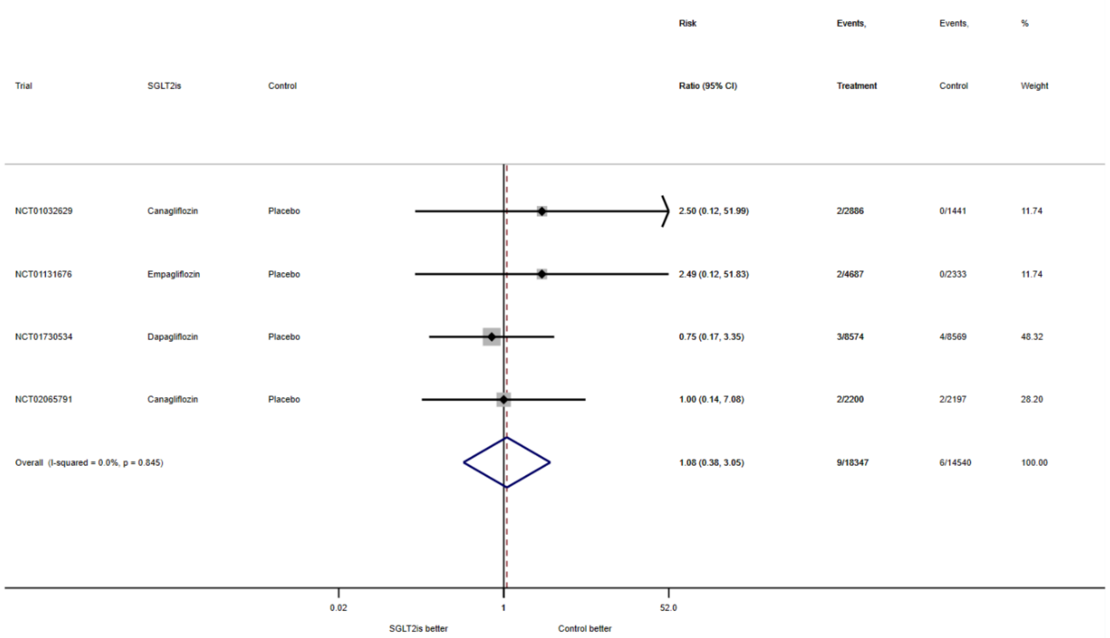

System 1-Blood And Lymphatic System Disorders-Figure S6 Meta-analysis of SGLT2is and Haemorrhagic anaemia

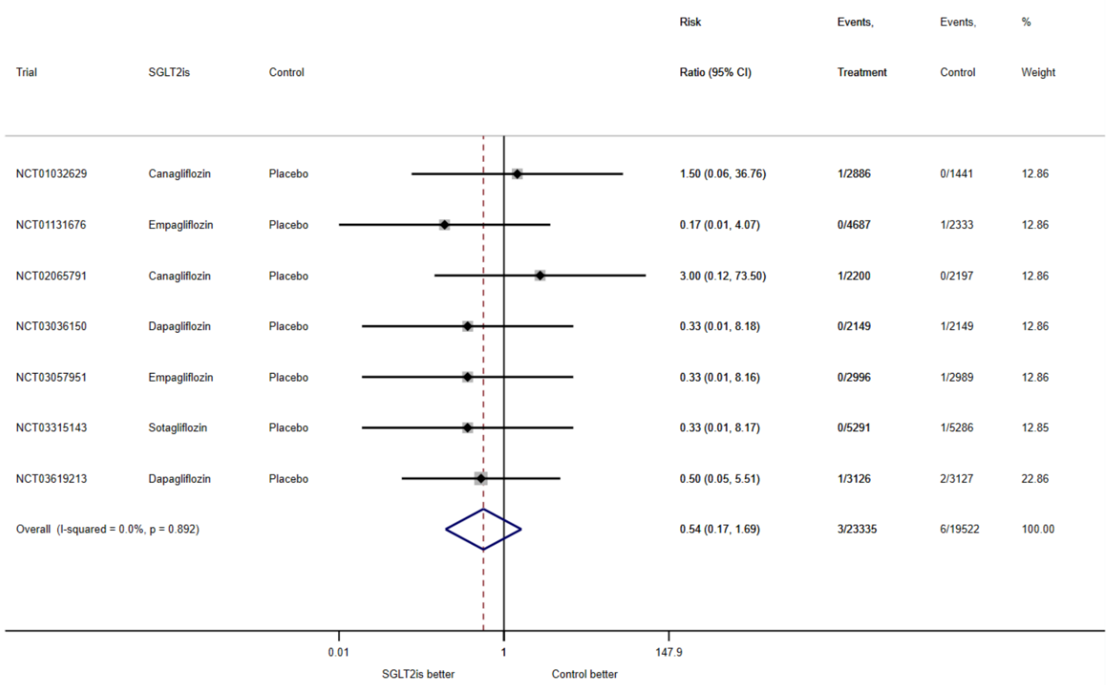

System 1-Blood And Lymphatic System Disorders-Figure S7 Meta-analysis of SGLT2is and Hypochromic anaemia

Trial	SGLT2is	Control	Risk Ratio (95% CI)	Events, Treatment	Events, Control	% Weight
NCT01032629	Canagliflozin	Placebo	3.50 (0.18, 67.64)	3/2886	0/1441	1.98
NCT01131676	Empagliflozin	Placebo	1.24 (0.24, 6.41)	5/4687	2/2333	6.47
NCT01730534	Dapagliflozin	Placebo	0.67 (0.32, 1.38)	12/8574	18/8569	32.62
NCT01986881	Ertugliflozin	Placebo	0.75 (0.13, 4.48)	3/5493	2/2745	5.43
NCT01989754	Canagliflozin	Placebo	0.50 (0.05, 5.51)	1/2904	2/2903	3.02
NCT02065791	Canagliflozin	Placebo	4.99 (0.24, 103.94)	2/2200	0/2197	1.88
NCT03036124	Dapagliflozin	Placebo	5.00 (0.24, 104.09)	2/2368	0/2368	1.88
NCT03036150	Dapagliflozin	Placebo	0.17 (0.02, 1.38)	1/2149	6/2149	3.88
NCT03057951	Empagliflozin	Placebo	0.50 (0.19, 1.33)	6/2996	12/2989	18.14
NCT03057977	Empagliflozin	Placebo	3.00 (0.12, 73.59)	1/1863	0/1863	1.70
NCT03315143	Sotagliflozin	Placebo	3.00 (0.61, 14.84)	6/5291	2/5286	6.79
NCT03521934	Sotagliflozin	Placebo	3.03 (0.12, 74.22)	1/605	0/611	1.70
NCT03594110	Empagliflozin	Placebo	0.50 (0.09, 2.73)	2/3304	4/3305	6.03
NCT03619213	Dapagliflozin	Placebo	0.60 (0.14, 2.51)	3/3126	5/3127	8.49
Overall (I-squared = 0.0%, p = 0.543)			0.79 (0.52, 1.20)	48/48446	53/41886	100.00

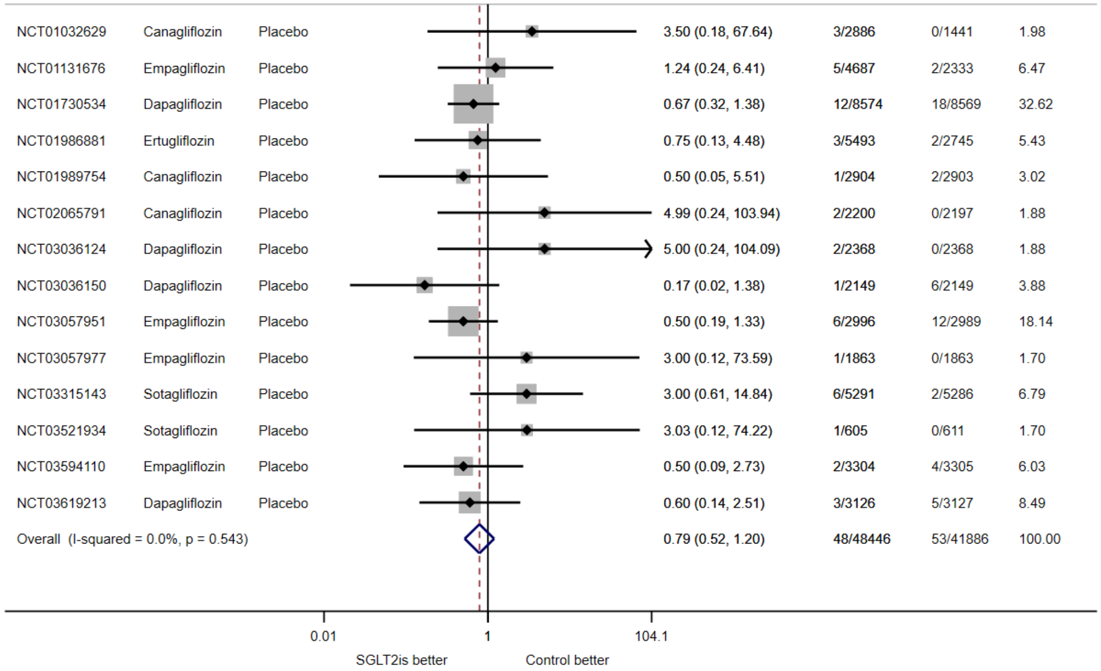

System 1-Blood And Lymphatic System Disorders-Figure S8 Meta-analysis of SGLT2is and Iron deficiency anaemia

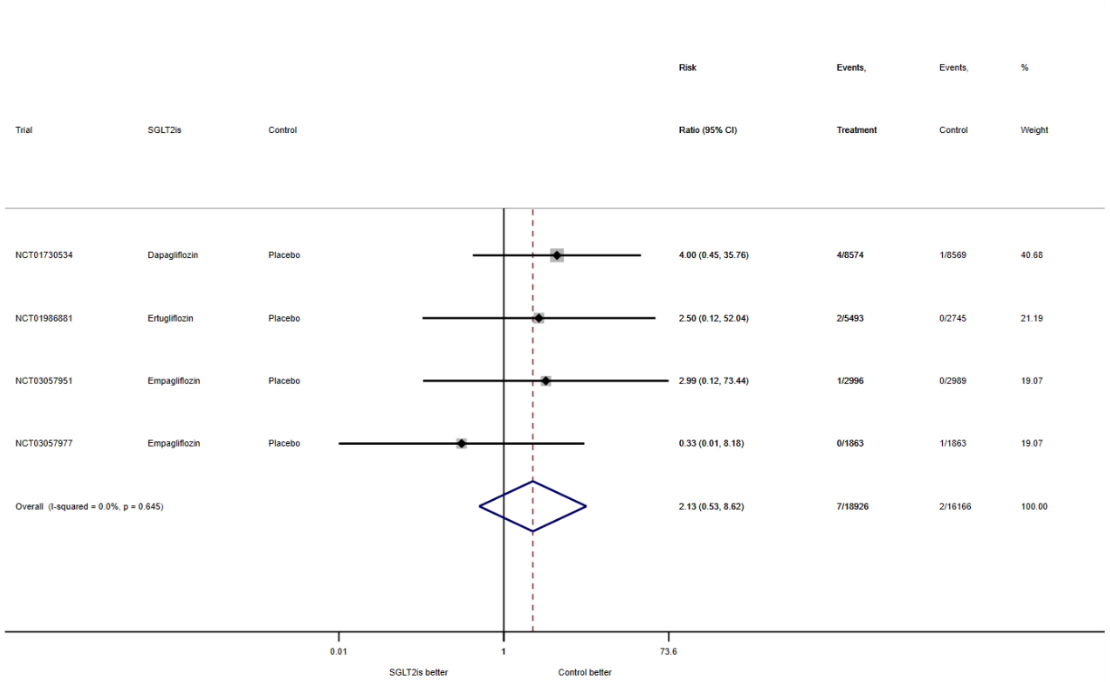

System 1-Blood And Lymphatic System Disorders-Figure S9 Meta-analysis of SGLT2is and Leukocytosis

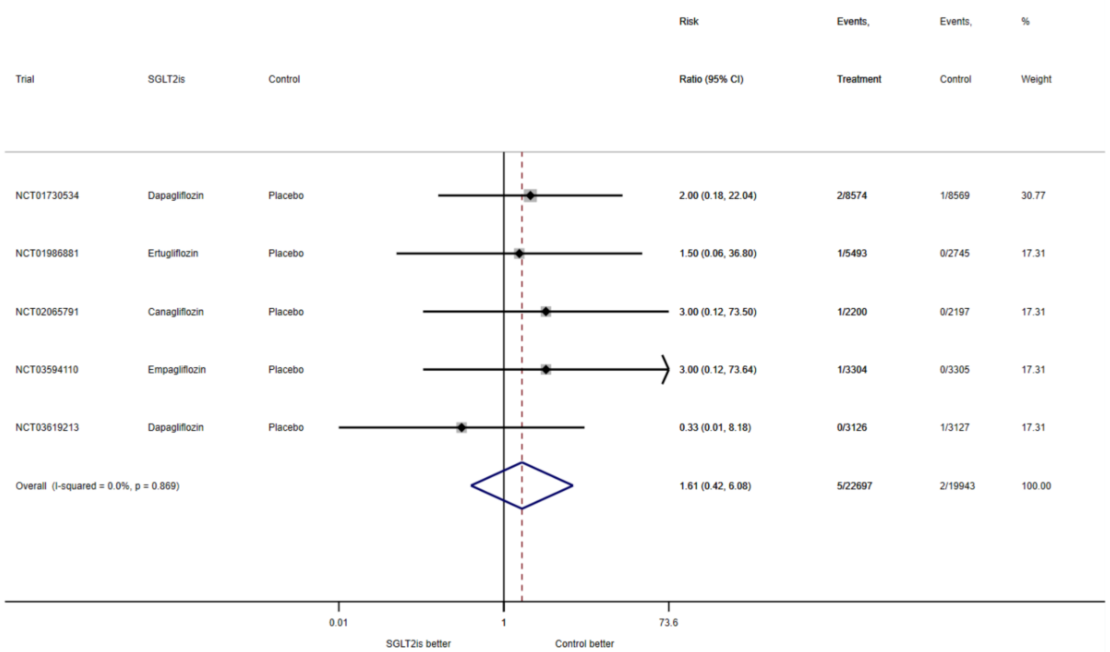

System 1-Blood And Lymphatic System Disorders-Figure S10 Meta-analysis of SGLT2is and Lymphadenopathy

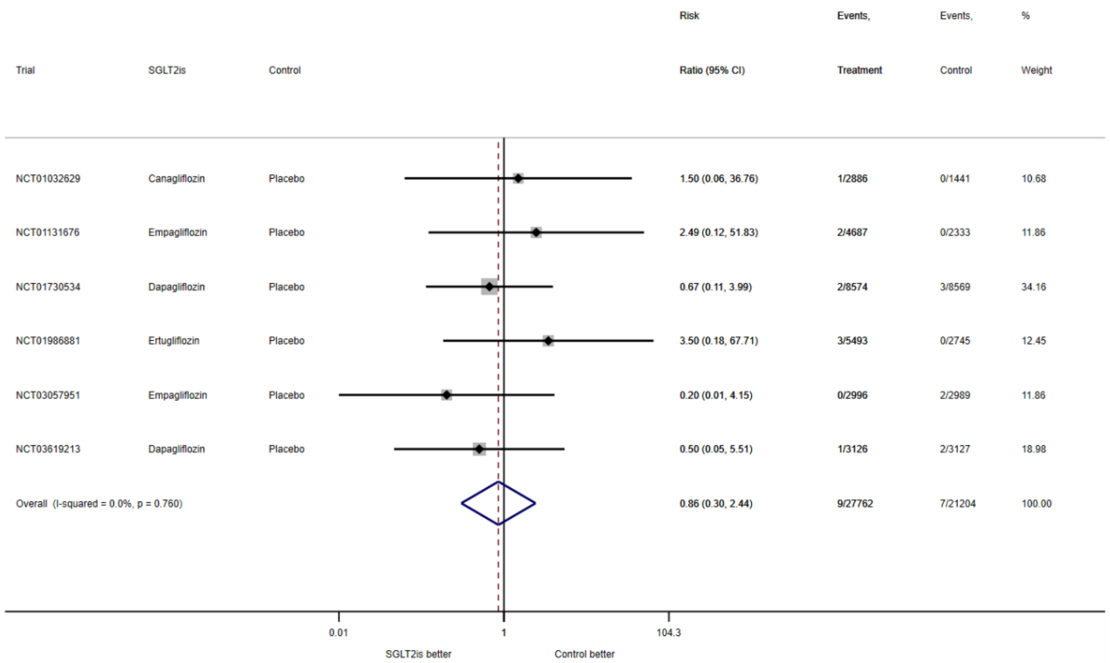

System 1-Blood And Lymphatic System Disorders-Figure S11 Meta-analysis of SGLT2is and Microcytic anaemia

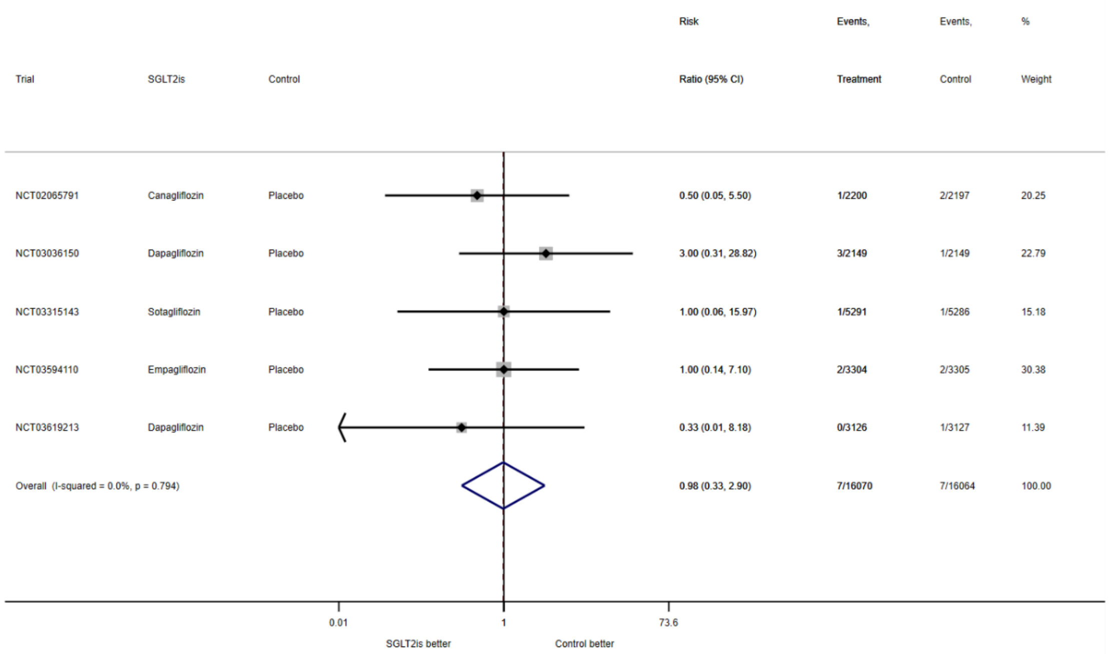

System 1-Blood And Lymphatic System Disorders-Figure S12 Meta-analysis of SGLT2is and Nephrogenic anaemia

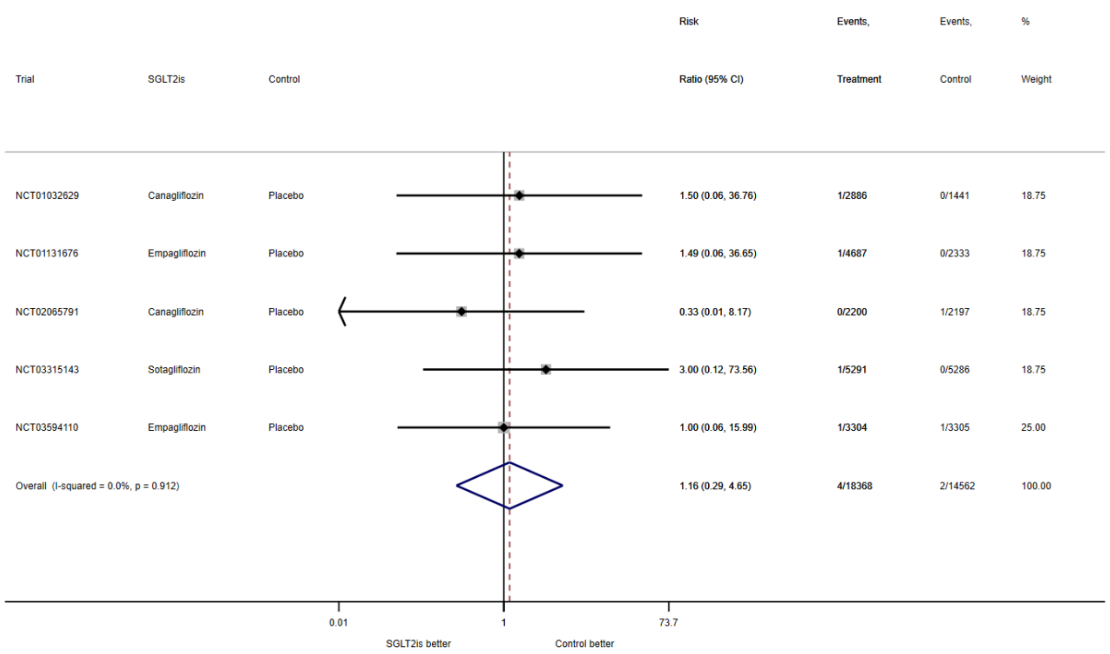

System 1-Blood And Lymphatic System Disorders-Figure S13 Meta-analysis of SGLT2is and Normochromic normocytic anaemia

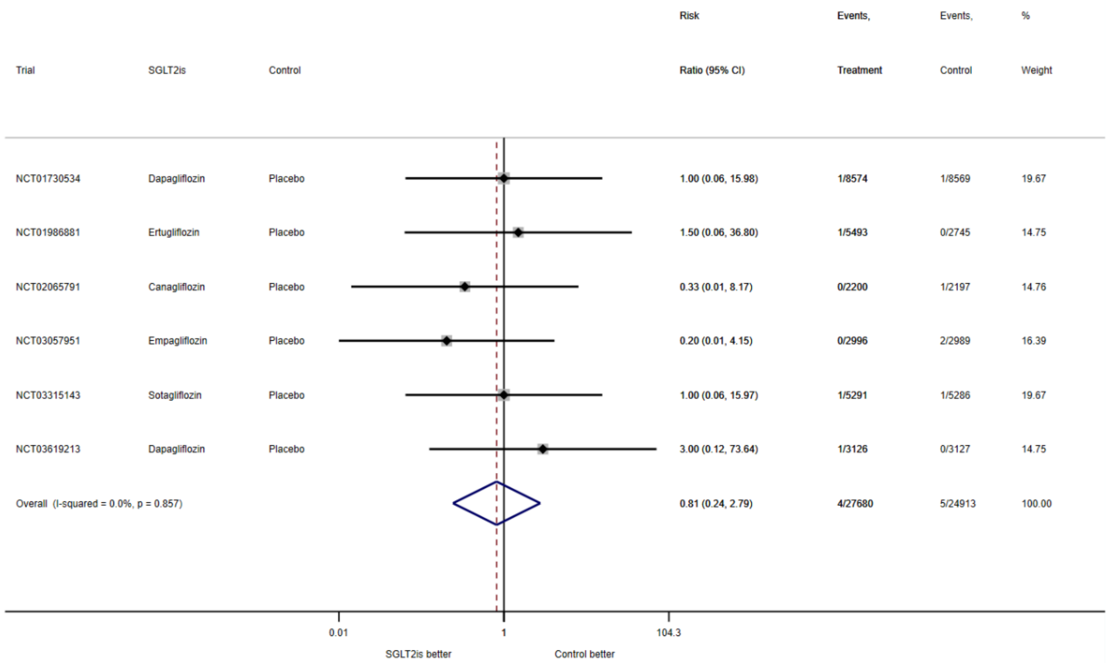

System 1-Blood And Lymphatic System Disorders-Figure S14 Meta-analysis of SGLT2is and Normocytic anaemia

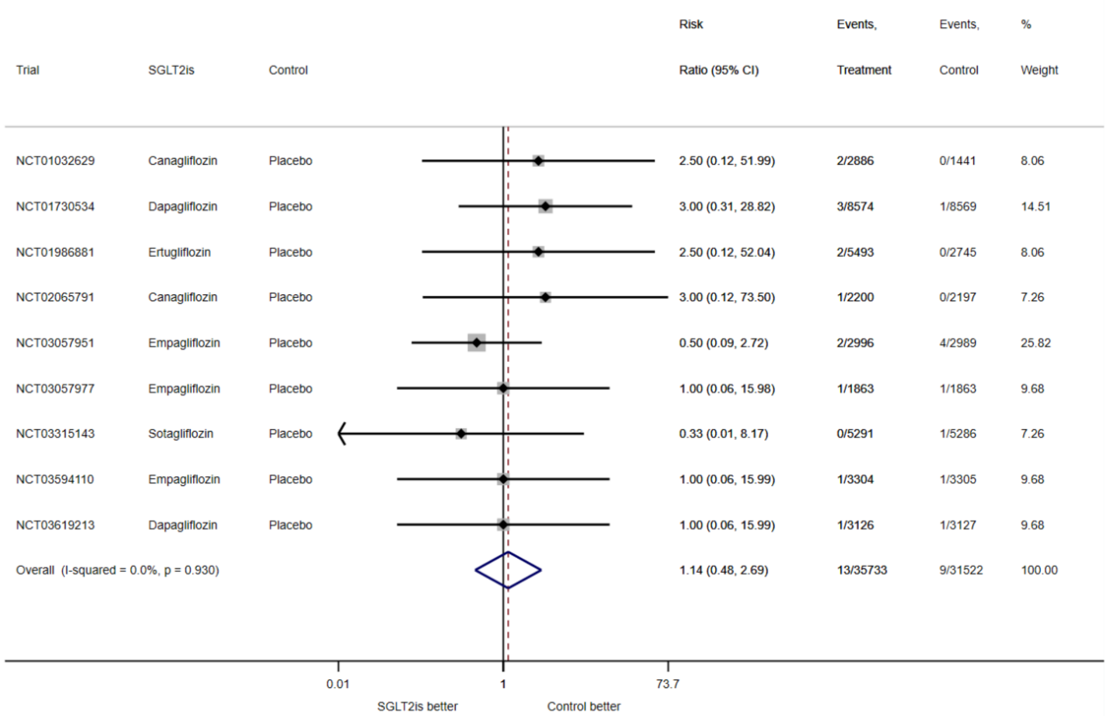

System 1-Blood And Lymphatic System Disorders-Figure S15 Meta-analysis of SGLT2is and Pancytopenia

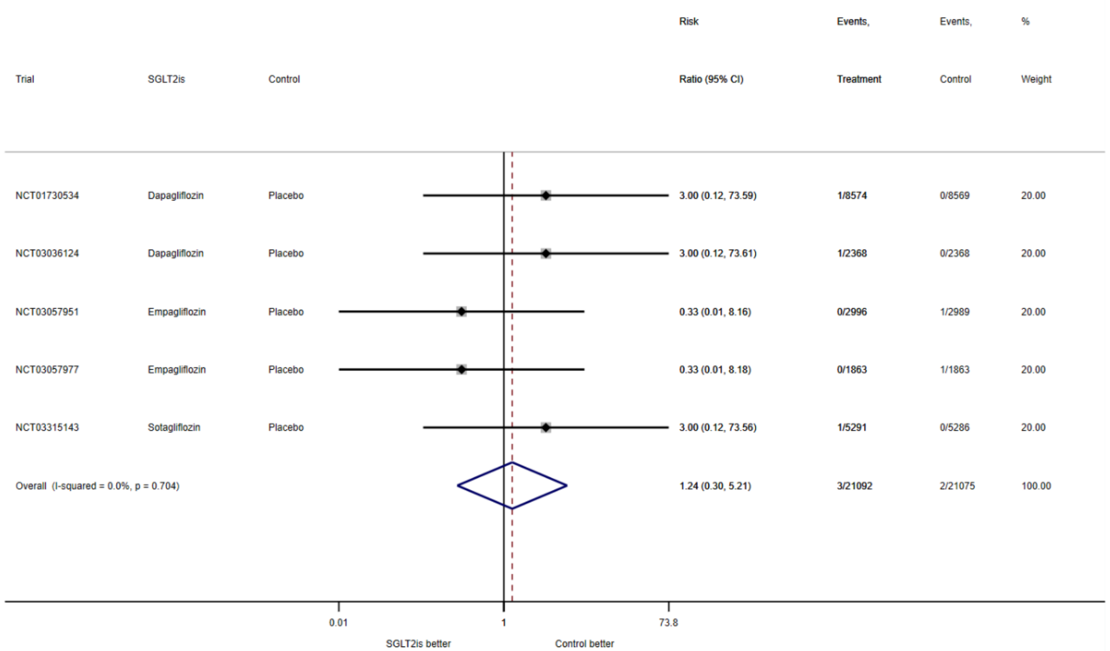

System 1-Blood And Lymphatic System Disorders-Figure S16 Meta-analysis of SGLT2is and Splenic infarction

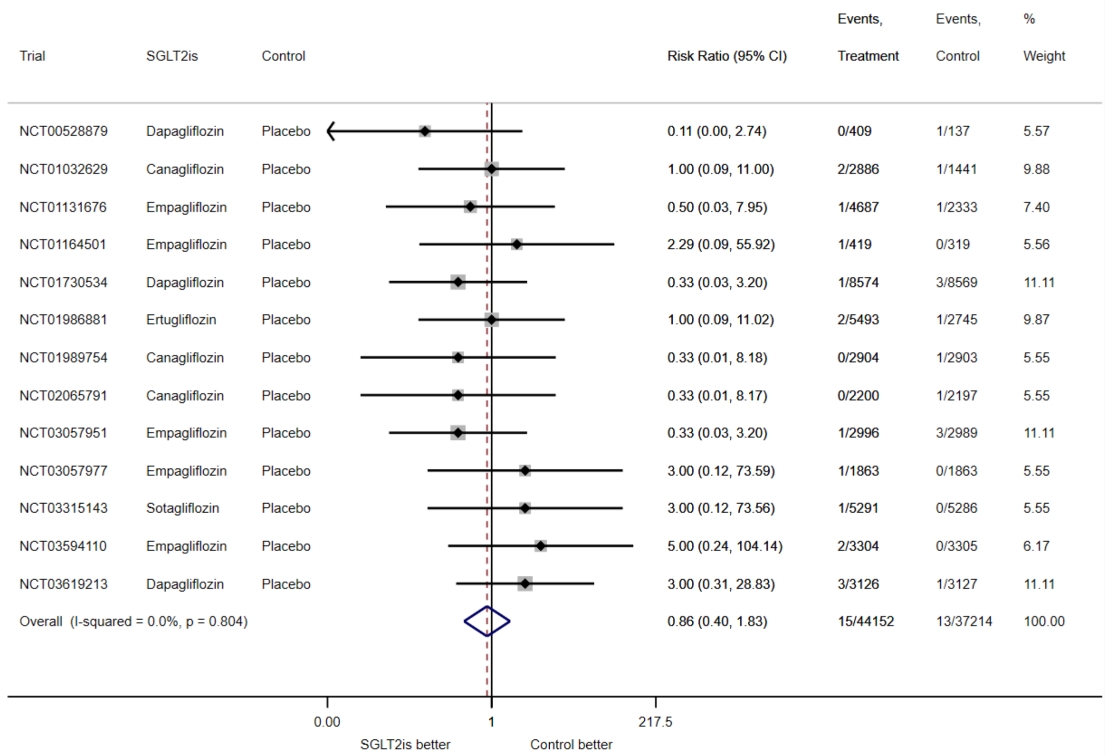

System 1-Blood And Lymphatic System Disorders-Figure S17 Meta-analysis of SGLT2is and Thrombocytopenia

Trial	SGLT2is	Control	Risk Ratio (95% CI)	Events, Treatment	Events, Control	% Weight
NCT01031680	Dapagliflozin	Placebo	3.01 (0.12, 73.77)	1/460	0/462	0.82
NCT01032629	Canagliflozin	Placebo	0.37 (0.13, 1.08)	6/2886	8/1441	7.52
NCT01042977	Dapagliflozin	Placebo	5.01 (0.24, 104.09)	2/482	0/483	0.91
NCT01131676	Empagliflozin	Placebo	1.19 (0.57, 2.49)	24/4687	10/2333	15.49
NCT01164501	Empagliflozin	Placebo	2.29 (0.09, 55.92)	1/419	0/319	0.82
NCT01730534	Dapagliflozin	Placebo	0.55 (0.30, 0.99)	17/8574	31/8569	24.05
NCT01986881	Ertugliflozin	Placebo	0.94 (0.40, 2.21)	15/5493	8/2745	11.43
NCT01989754	Canagliflozin	Placebo	0.36 (0.12, 1.14)	4/2904	11/2903	6.42
NCT02065791	Canagliflozin	Placebo	1.50 (0.61, 3.66)	12/2200	8/2197	10.53
NCT03036124	Dapagliflozin	Placebo	1.67 (0.40, 6.97)	5/2368	3/2368	4.10
NCT03036150	Dapagliflozin	Placebo	1.00 (0.14, 7.09)	2/2149	2/2149	2.19
NCT03057951	Empagliflozin	Placebo	3.99 (0.85, 18.78)	8/2996	2/2989	3.50
NCT03057977	Empagliflozin	Placebo	0.14 (0.01, 2.76)	0/1863	3/1863	0.96
NCT03315143	Sotagliflozin	Placebo	0.87 (0.32, 2.41)	7/5291	8/5286	8.17
NCT03521934	Sotagliflozin	Placebo	0.34 (0.01, 8.25)	0/605	1/611	0.82
NCT03594110	Empagliflozin	Placebo	3.00 (0.12, 73.64)	1/3304	0/3305	0.82
NCT03619213	Dapagliflozin	Placebo	2.00 (0.18, 22.05)	2/3126	1/3127	1.46
NCT01106677	Canagliflozin	Sitagliptin	(Excluded)	0/735	0/366	0.00
Overall (I-squared = 14.6%, p = 0.282)			0.87 (0.65, 1.16)	107/50542	96/43516	100.00

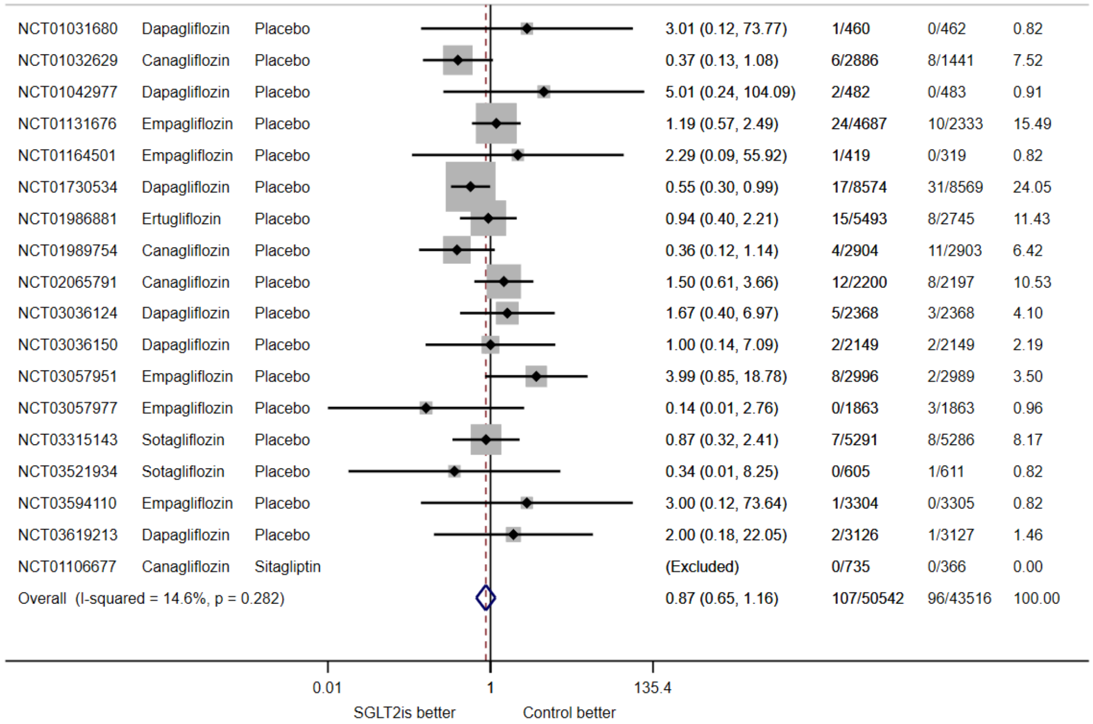

System 2-Cardiac Disorders-Figure S1 Meta-analysis of SGLT2is and Acute coronary syndrome

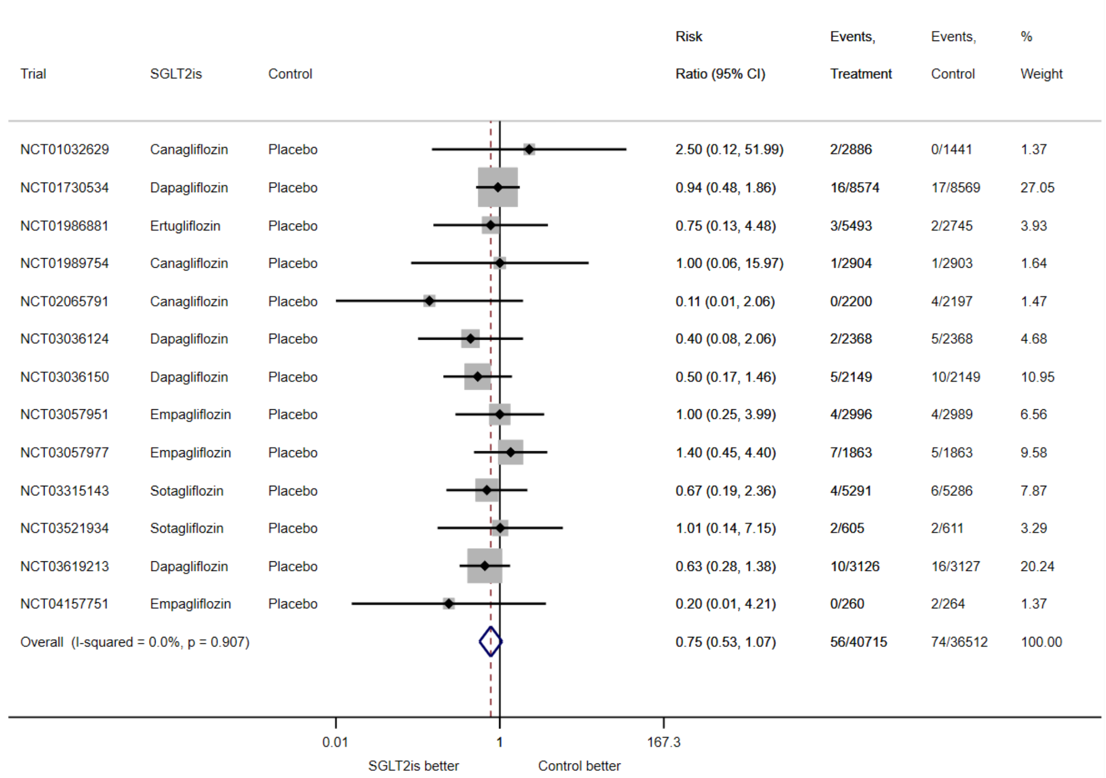

System 2-Cardiac Disorders-Figure S2 Meta-analysis of SGLT2is and Acute left ventricular failure

Trial	SGLT2is	Control	Risk Ratio (95% CI)	Events, Treatment	Events, Control	% Weight
NCT00528879	Dapagliflozin	Placebo	0.05 (0.00, 0.93)	0/409	3/137	0.11
NCT01031680	Dapagliflozin	Placebo	1.00 (0.06, 16.01)	1/460	1/462	0.13
NCT01032629	Canagliflozin	Placebo	0.90 (0.42, 1.94)	18/2886	10/1441	1.67
NCT01042977	Dapagliflozin	Placebo	0.33 (0.01, 8.18)	0/482	1/483	0.10
NCT01106651	Canagliflozin	Placebo	0.99 (0.09, 10.90)	2/477	1/237	0.17
NCT01106677	Canagliflozin	Sitagliptin	0.17 (0.01, 4.07)	0/735	1/366	0.10
NCT01131676	Empagliflozin	Placebo	0.95 (0.65, 1.37)	80/4687	42/2333	7.24
NCT01164501	Empagliflozin	Placebo	0.15 (0.01, 3.16)	0/419	2/319	0.11
NCT01177813	Empagliflozin	Sitagliptin or Placebo	3.03 (0.12, 74.27)	1/447	0/452	0.10
NCT01730534	Dapagliflozin	Placebo	1.14 (0.96, 1.35)	265/8574	233/8569	32.95
NCT01986881	Ertugliflozin	Placebo	0.88 (0.66, 1.16)	128/5493	73/2745	12.31
NCT01989754	Canagliflozin	Placebo	1.23 (0.74, 2.06)	32/2904	26/2903	3.74
NCT02033889	Ertugliflozin	Placebo/Glimepiride	0.51 (0.03, 8.07)	1/412	1/209	0.13
NCT02065791	Canagliflozin	Placebo	1.00 (0.64, 1.56)	38/2200	38/2197	4.99
NCT02384941	Sotagliflozin	Placebo	0.51 (0.07, 3.60)	2/525	2/268	0.26
NCT03036124	Dapagliflozin	Placebo	0.97 (0.62, 1.53)	37/2368	38/2368	4.92
NCT03036150	Dapagliflozin	Placebo	0.70 (0.44, 1.09)	32/2149	46/2149	4.96
NCT03057951	Empagliflozin	Placebo	1.04 (0.70, 1.54)	50/2996	48/2989	6.43
NCT03057977	Empagliflozin	Placebo	1.33 (0.73, 2.45)	24/1863	18/1863	2.68
NCT03242252	Sotagliflozin	Placebo	0.99 (0.18, 5.35)	4/527	2/260	0.35
NCT03315143	Sotagliflozin	Placebo	0.81 (0.56, 1.19)	48/5291	59/5286	6.90
NCT03521934	Sotagliflozin	Placebo	1.35 (0.30, 5.99)	4/605	3/611	0.44
NCT03594110	Empagliflozin	Placebo	1.20 (0.37, 3.93)	6/3304	5/3305	0.71
NCT03619213	Dapagliflozin	Placebo	1.00 (0.71, 1.41)	63/3126	63/3127	8.30
NCT04157751	Empagliflozin	Placebo	0.34 (0.01, 8.27)	0/260	1/264	0.10
NCT04350593	Dapagliflozin	Placebo	1.00 (0.06, 16.03)	1/613	1/616	0.13
Overall (I-squared = 0.0%, p = 0.870)			1.00 (0.90, 1.10)	837/54212	718/45959	100.00

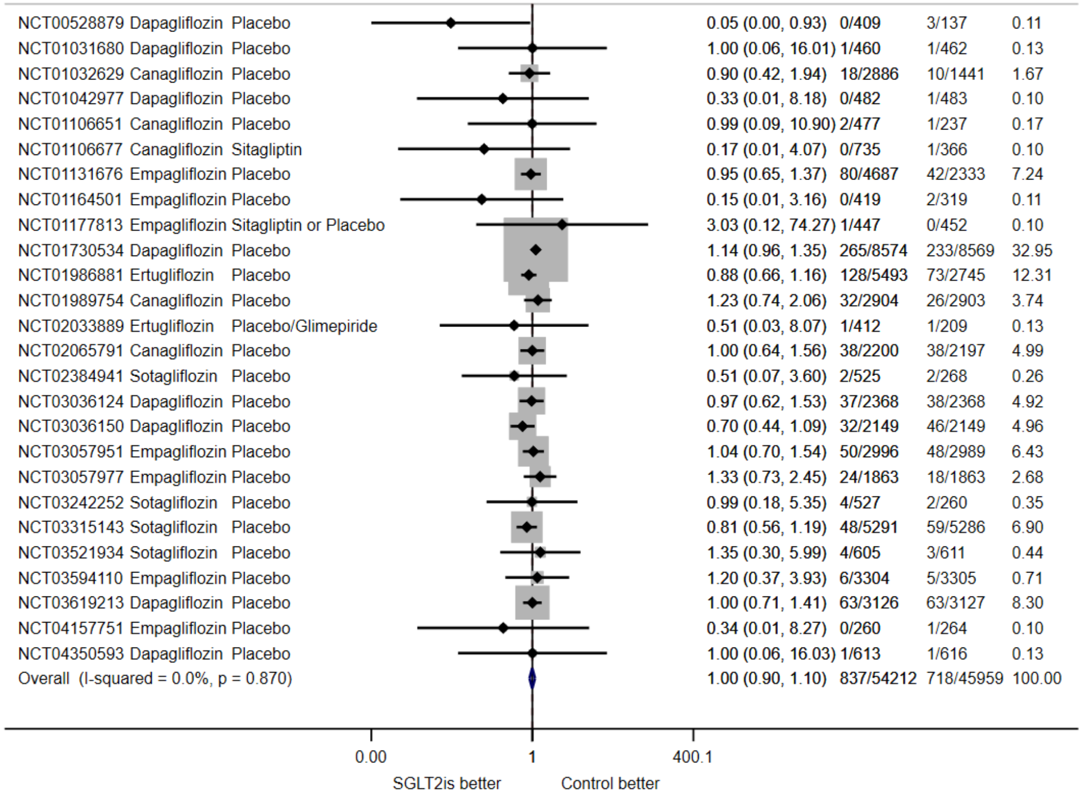

System 2-Cardiac Disorders-Figure S3 Meta-analysis of SGLT2is and Acute myocardial infarction

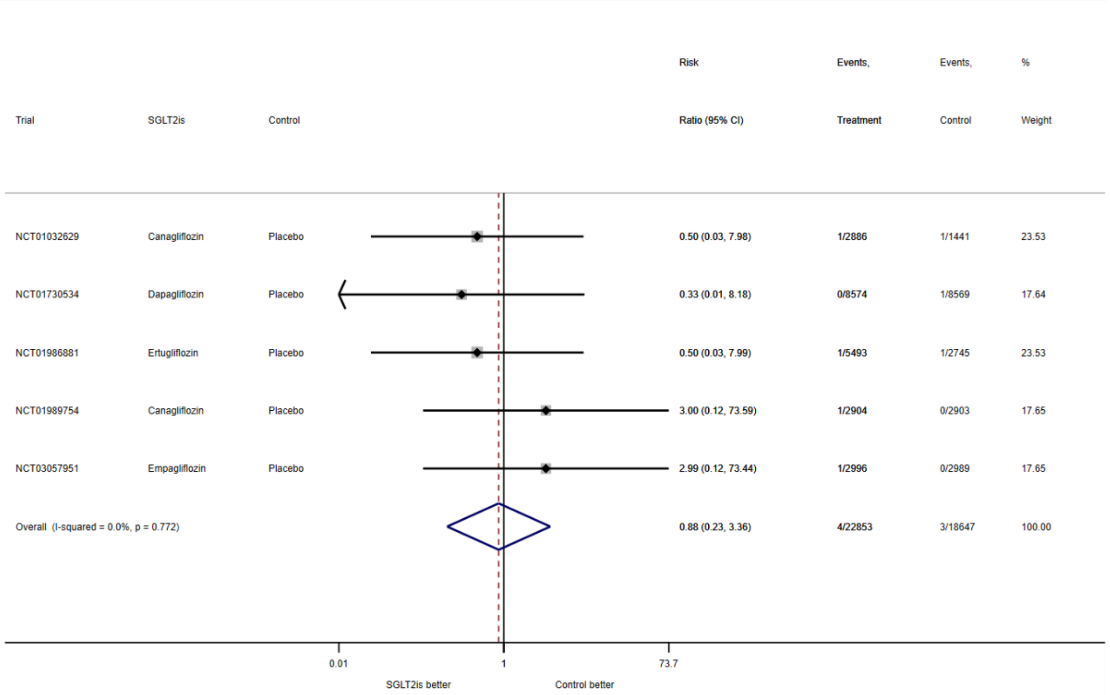

System 2-Cardiac Disorders-Figure S4 Meta-analysis of SGLT2is and Adams-stokes syndrome

Trial	SGLT2is	Control	Risk Ratio (95% CI)	Events, Treatment	Events, Control	% Weight
NCT00528879	Dapagliflozin	Placebo	0.33 (0.02, 5.32)	1/409	1/137	0.22
NCT00673231	Dapagliflozin	Placebo	1.62 (0.08, 33.61)	2/610	0/197	0.18
NCT01031680	Dapagliflozin	Placebo	3.01 (0.12, 73.77)	1/460	0/462	0.17
NCT01032629	Canagliflozin	Placebo	0.94 (0.57, 1.53)	45/2886	24/1441	7.01
NCT01042977	Dapagliflozin	Placebo	1.00 (0.06, 15.97)	1/482	1/483	0.22
NCT01106651	Canagliflozin	Placebo	0.50 (0.03, 7.91)	1/477	1/237	0.22
NCT01131676	Empagliflozin	Placebo	1.21 (0.81, 1.83)	78/4687	32/2333	10.15
NCT01730534	Dapagliflozin	Placebo	0.97 (0.78, 1.21)	154/8574	159/8569	35.11
NCT01986881	Ertugliflozin	Placebo	0.82 (0.57, 1.16)	80/5493	49/2745	13.61
NCT01989754	Canagliflozin	Placebo	0.67 (0.39, 1.14)	22/2904	33/2903	5.87
NCT02033889	Ertugliflozin	Placebo/Glimepiride	0.17 (0.01, 4.14)	0/412	1/209	0.17
NCT02065791	Canagliflozin	Placebo	1.50 (0.76, 2.94)	21/2200	14/2197	3.73
NCT03036124	Dapagliflozin	Placebo	1.00 (0.45, 2.22)	12/2368	12/2368	2.66
NCT03036150	Dapagliflozin	Placebo	0.44 (0.14, 1.44)	4/2149	9/2149	1.22
NCT03057951	Empagliflozin	Placebo	1.21 (0.70, 2.10)	28/2996	23/2989	5.61
NCT03057977	Empagliflozin	Placebo	0.60 (0.22, 1.65)	6/1863	10/1863	1.66
NCT03242252	Sotagliflozin	Placebo	0.49 (0.03, 7.86)	1/527	1/260	0.22
NCT03315143	Sotagliflozin	Placebo	0.72 (0.39, 1.32)	18/5291	25/5286	4.63
NCT03521934	Sotagliflozin	Placebo	1.35 (0.30, 5.99)	4/605	3/611	0.76
NCT03594110	Empagliflozin	Placebo	0.42 (0.18, 0.96)	8/3304	19/3305	2.49
NCT03619213	Dapagliflozin	Placebo	0.95 (0.50, 1.80)	18/3126	19/3127	4.10
Overall (I-squared = 0.0%, p = 0.718)			0.92 (0.81, 1.05)	505/51823	436/43871	100.00

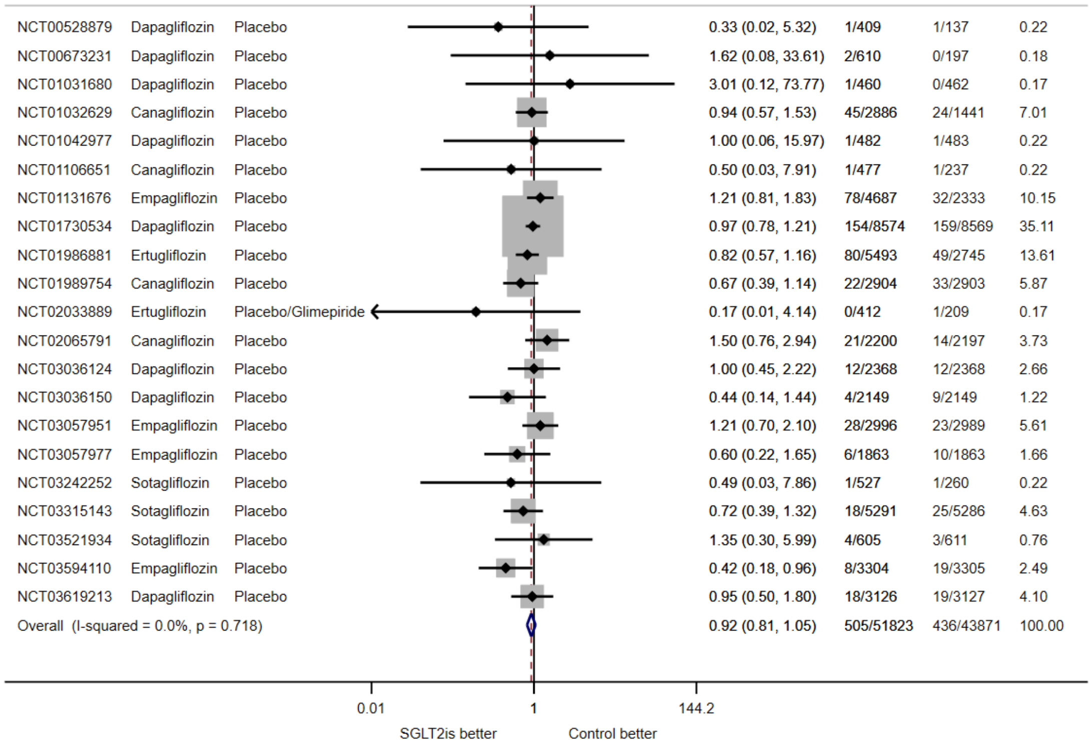

System 2-Cardiac Disorders-Figure S5 Meta-analysis of SGLT2is and Angina pectoris

Trial	SGLT2is	Control	Risk Ratio (95% CI)	Events, Treatment	Events, Control	% Weight
NCT00528879	Dapagliflozin	Placebo	0.33 (0.02, 5.32)	1/409	1/137	0.12
NCT01031680	Dapagliflozin	Placebo	0.33 (0.03, 3.21)	1/460	3/462	0.19
NCT01032629	Canagliflozin	Placebo	1.30 (0.78, 2.17)	52/2886	20/1441	3.64
NCT01042977	Dapagliflozin	Placebo	1.50 (0.25, 8.96)	3/482	2/483	0.30
NCT01106651	Canagliflozin	Placebo	0.50 (0.07, 3.51)	2/477	2/237	0.25
NCT01106677	Canagliflozin	Sitagliptin	0.17 (0.01, 4.07)	0/735	1/366	0.09
NCT01131676	Empagliflozin	Placebo	0.89 (0.69, 1.15)	155/4687	87/2333	14.36
NCT01164501	Empagliflozin	Placebo	0.15 (0.01, 3.16)	0/419	2/319	0.10
NCT01730534	Dapagliflozin	Placebo	1.01 (0.85, 1.19)	271/8574	269/8569	34.63
NCT01986881	Ertugliflozin	Placebo	0.81 (0.63, 1.06)	145/5493	89/2745	14.13
NCT01989754	Canagliflozin	Placebo	1.00 (0.65, 1.53)	42/2904	42/2903	5.29
NCT02033889	Ertugliflozin	Placebo/Glimepiride	3.56 (0.18, 68.59)	3/412	0/209	0.11
NCT02065791	Canagliflozin	Placebo	0.59 (0.32, 1.06)	17/2200	29/2197	2.69
NCT02384941	Sotagliflozin	Placebo	0.17 (0.01, 4.17)	0/525	1/268	0.09
NCT03036124	Dapagliflozin	Placebo	0.70 (0.40, 1.22)	21/2368	30/2368	3.10
NCT03036150	Dapagliflozin	Placebo	0.64 (0.33, 1.24)	14/2149	22/2149	2.14
NCT03057951	Empagliflozin	Placebo	1.13 (0.65, 1.97)	26/2996	23/2989	3.06
NCT03057977	Empagliflozin	Placebo	1.22 (0.51, 2.94)	11/1863	9/1863	1.24
NCT03242252	Sotagliflozin	Placebo	5.44 (0.30, 97.96)	5/527	0/260	0.11
NCT03315143	Sotagliflozin	Placebo	0.85 (0.57, 1.26)	44/5291	52/5286	5.97
NCT03521934	Sotagliflozin	Placebo	8.08 (1.01, 64.40)	8/605	1/611	0.22
NCT03594110	Empagliflozin	Placebo	1.22 (0.51, 2.95)	11/3304	9/3305	1.23
NCT03619213	Dapagliflozin	Placebo	0.79 (0.55, 1.15)	49/3126	62/3127	6.92
NCT01177813	Empagliflozin	Sitagliptin or Placebo	(Excluded)	0/447	0/452	0.00
Overall (I-squared = 1.4%, p = 0.441)			0.92 (0.83, 1.01)	881/53339	756/45079	100.00

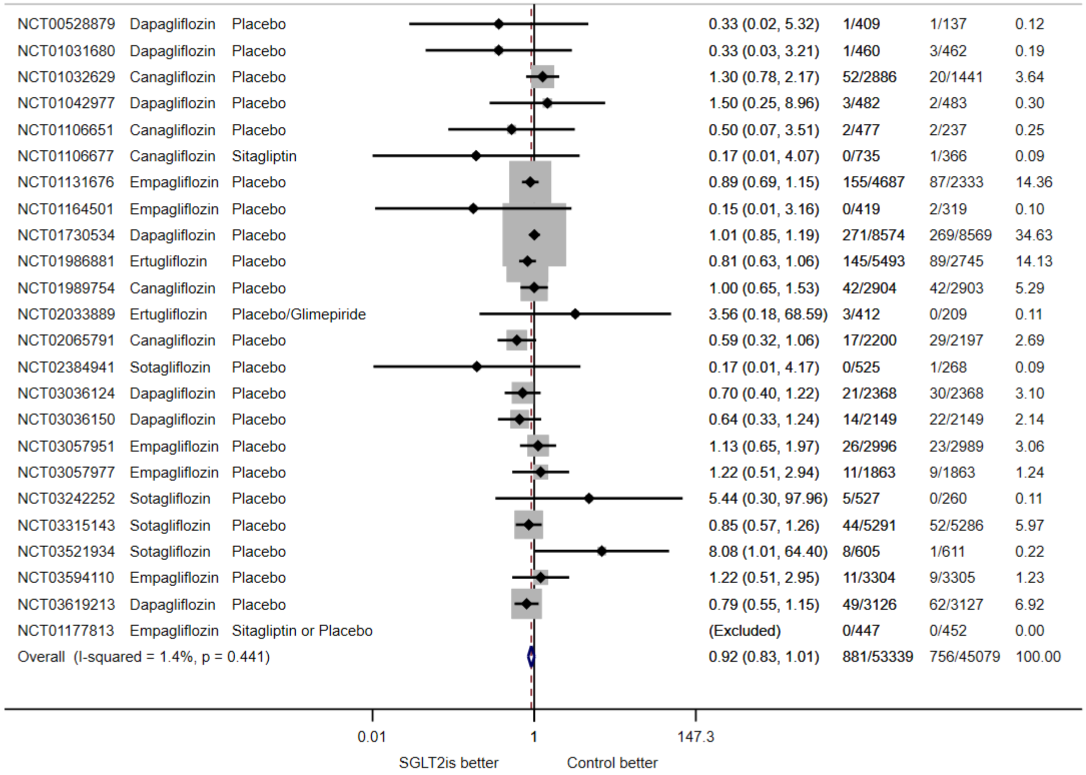

System 2-Cardiac Disorders-Figure S6 Meta-analysis of SGLT2is and Angina unstable

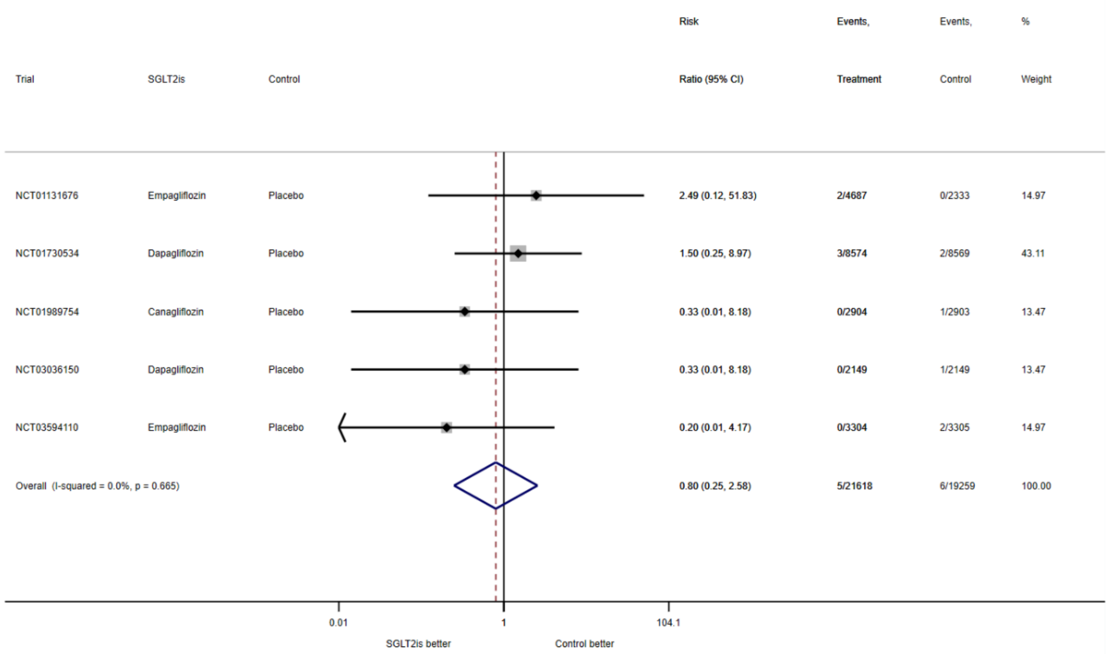

System 2-Cardiac Disorders-Figure S7 Meta-analysis of SGLT2is and Aortic valve disease

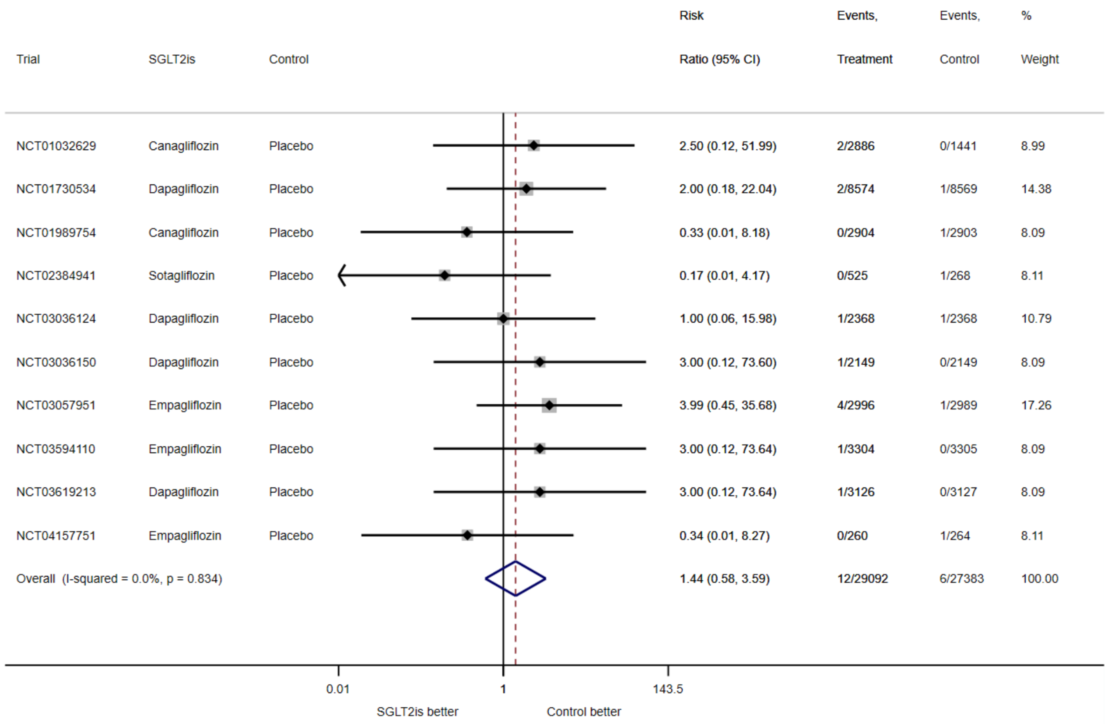

System 2-Cardiac Disorders-Figure S8 Meta-analysis of SGLT2is and Aortic valve incompetence

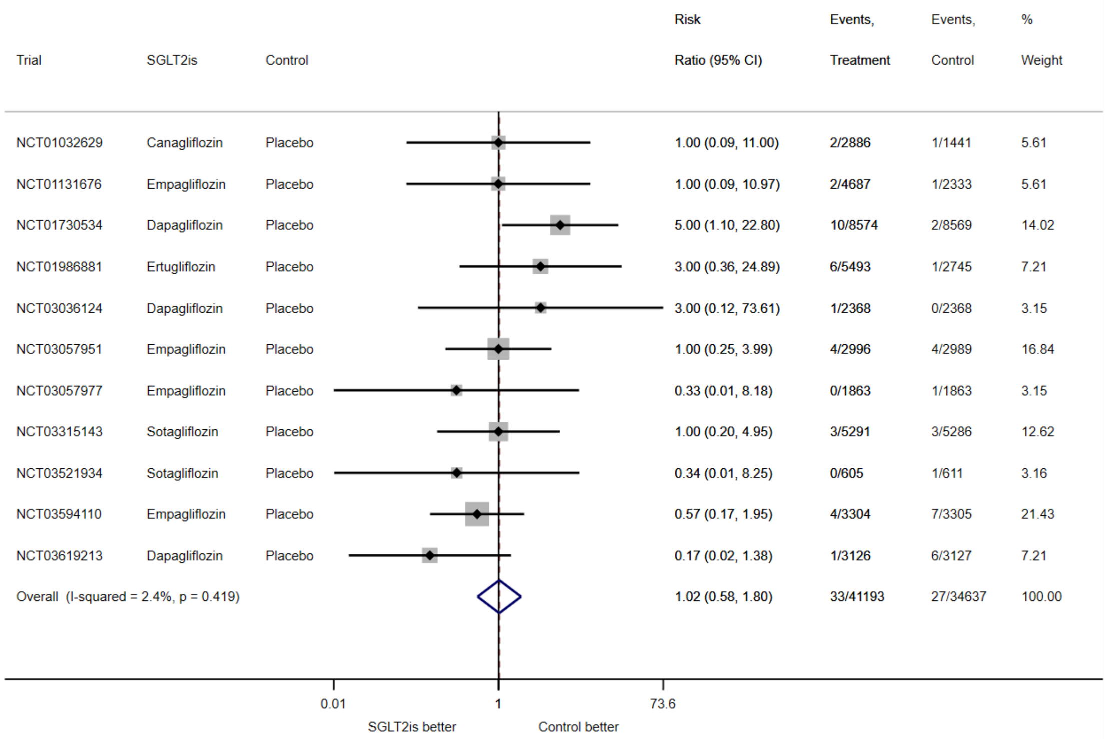

System 2-Cardiac Disorders-Figure S9 Meta-analysis of SGLT2is and Aortic valve stenosis

Trial	SGLT2is	Control	Risk Ratio (95% CI)	Events, Treatment	Events, Control	% Weight
NCT01032629	Canagliflozin	Placebo	0.67 (0.15, 2.97)	4/2886	3/1441	11.62
NCT01131676	Empagliflozin	Placebo	1.49 (0.30, 7.39)	6/4687	2/2333	10.16
NCT01164501	Empagliflozin	Placebo	0.25 (0.01, 6.21)	0/419	1/319	2.54
NCT01730534	Dapagliflozin	Placebo	0.83 (0.25, 2.73)	5/8574	6/8569	18.47
NCT01986881	Ertugliflozin	Placebo	1.00 (0.09, 11.02)	2/5493	1/2745	4.51
NCT01989754	Canagliflozin	Placebo	2.00 (0.18, 22.04)	2/2904	1/2903	4.51
NCT02065791	Canagliflozin	Placebo	2.00 (0.18, 22.01)	2/2200	1/2197	4.51
NCT03036124	Dapagliflozin	Placebo	0.25 (0.05, 1.18)	2/2368	8/2368	10.84
NCT03057951	Empagliflozin	Placebo	0.40 (0.08, 2.06)	2/2996	5/2989	9.68
NCT03057977	Empagliflozin	Placebo	1.00 (0.06, 15.98)	1/1863	1/1863	3.39
NCT03242252	Sotagliflozin	Placebo	0.16 (0.01, 4.03)	0/527	1/260	2.54
NCT03315143	Sotagliflozin	Placebo	1.00 (0.14, 7.09)	2/5291	2/5286	6.77
NCT03594110	Empagliflozin	Placebo	1.00 (0.06, 15.99)	1/3304	1/3305	3.38
NCT03619213	Dapagliflozin	Placebo	0.50 (0.05, 5.51)	1/3126	2/3127	4.51
NCT04350593	Dapagliflozin	Placebo	0.33 (0.01, 8.21)	0/613	1/616	2.54
Overall (I-squared = 0.0%, p = 0.957)			0.70 (0.42, 1.17)	30/47251	36/40321	100.00

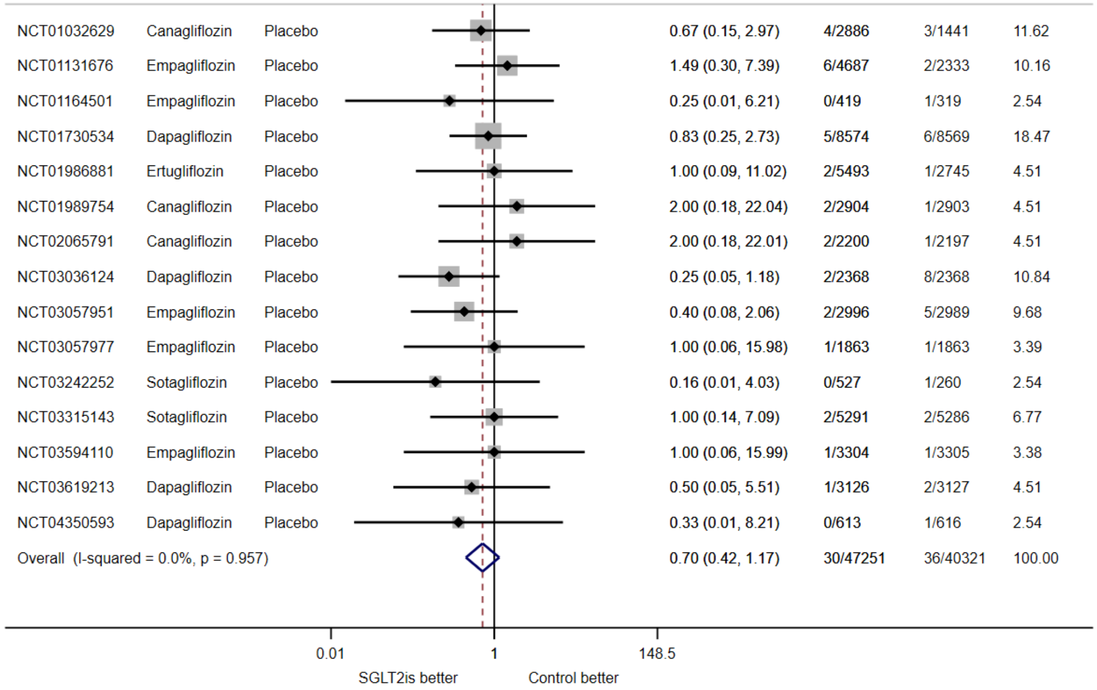

System 2-Cardiac Disorders-Figure S10 Meta-analysis of SGLT2is and Arrhythmia

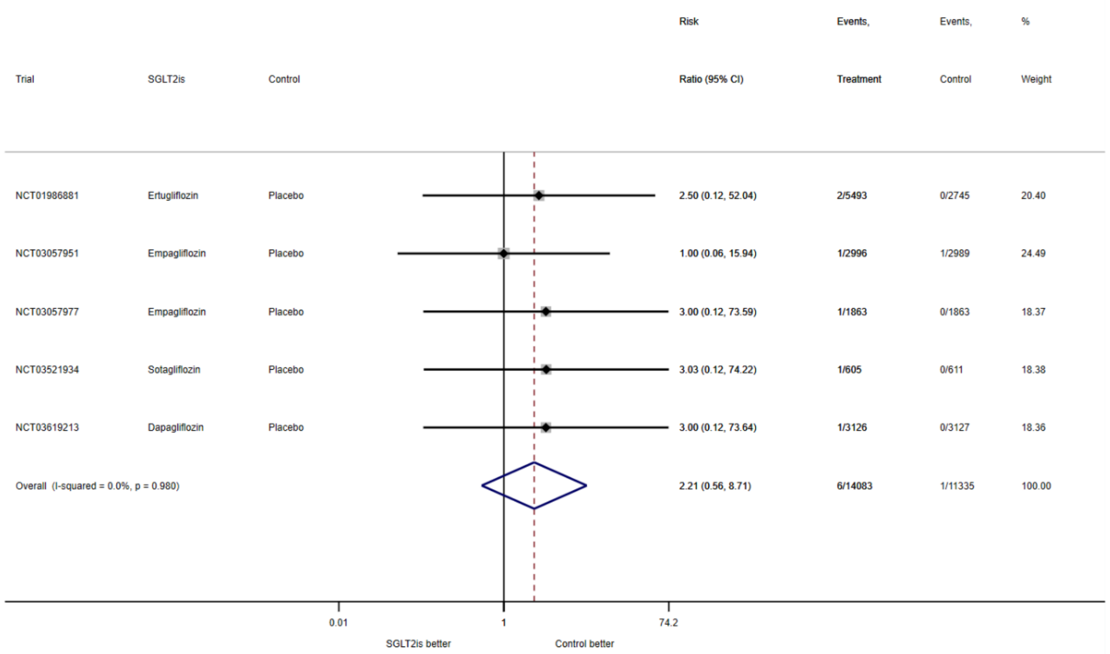

System 2-Cardiac Disorders-Figure S11 Meta-analysis of SGLT2is and Arrhythmia supraventricular

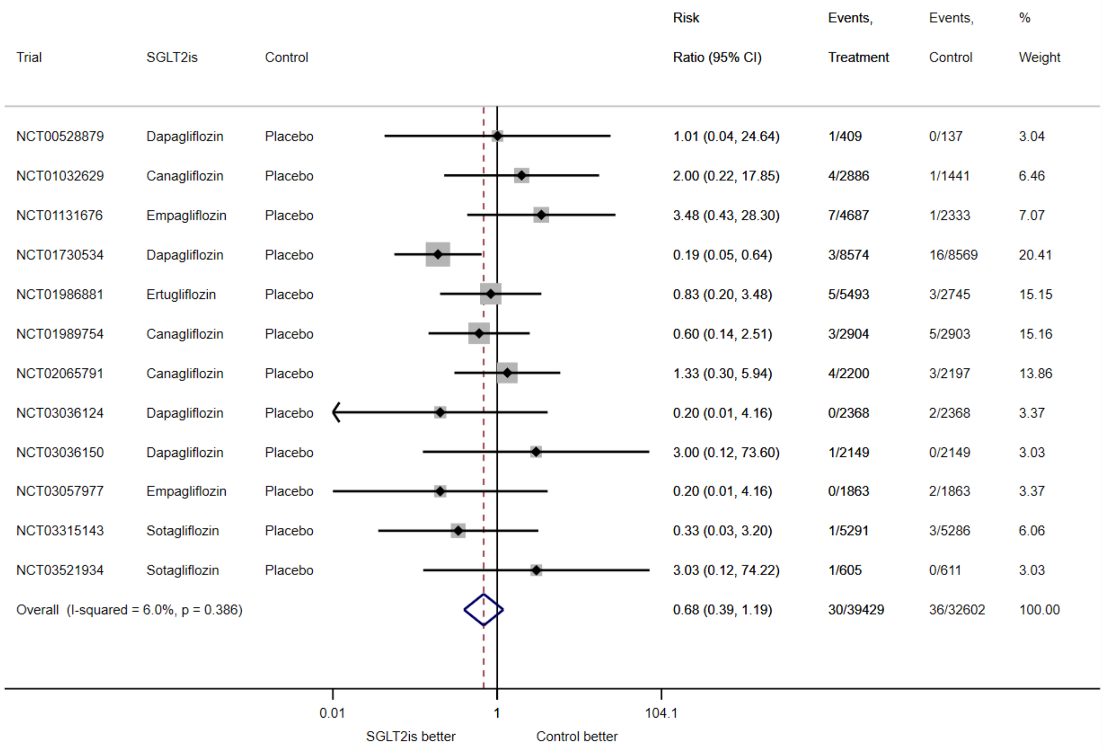

System 2-Cardiac Disorders-Figure S12 Meta-analysis of SGLT2is and Arteriosclerosis coronary artery

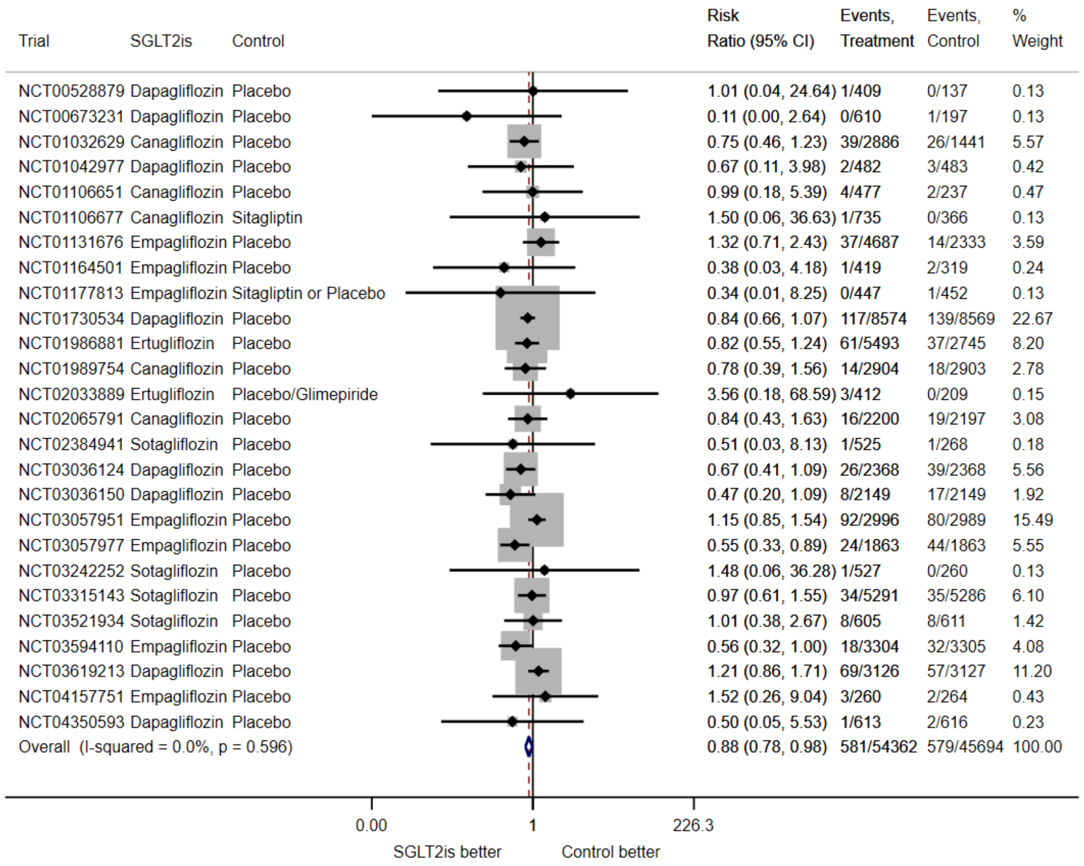

System 2-Cardiac Disorders-Figure S13 Meta-analysis of SGLT2is and Atrial fibrillation

Trial	SGLT2is	Control	Risk Ratio (95% CI)	Events, Treatment	Events, Control	% Weight
NCT01031680	Dapagliflozin	Placebo	0.33 (0.01, 8.20)	0/460	1/462	0.69
NCT01032629	Canagliflozin	Placebo	1.17 (0.45, 3.03)	14/2886	6/1441	7.79
NCT01131676	Empagliflozin	Placebo	1.10 (0.38, 3.15)	11/4687	5/2333	6.36
NCT01730534	Dapagliflozin	Placebo	0.62 (0.36, 1.06)	21/8574	34/8569	24.03
NCT01986881	Ertugliflozin	Placebo	1.11 (0.51, 2.44)	20/5493	9/2745	11.49
NCT01989754	Canagliflozin	Placebo	0.71 (0.23, 2.25)	5/2904	7/2903	5.39
NCT02065791	Canagliflozin	Placebo	1.00 (0.14, 7.08)	2/2200	2/2197	1.85
NCT03036124	Dapagliflozin	Placebo	2.67 (0.71, 10.04)	8/2368	3/2368	4.03
NCT03036150	Dapagliflozin	Placebo	0.33 (0.03, 3.20)	1/2149	3/2149	1.39
NCT03057951	Empagliflozin	Placebo	0.67 (0.34, 1.31)	14/2996	21/2989	15.59
NCT03057977	Empagliflozin	Placebo	0.27 (0.08, 0.98)	3/1863	11/1863	4.36
NCT03242252	Sotagliflozin	Placebo	0.16 (0.01, 4.03)	0/527	1/260	0.69
NCT03315143	Sotagliflozin	Placebo	0.50 (0.19, 1.33)	6/5291	12/5286	7.39
NCT03521934	Sotagliflozin	Placebo	1.01 (0.06, 16.11)	1/605	1/611	0.92
NCT03594110	Empagliflozin	Placebo	1.00 (0.25, 4.00)	4/3304	4/3305	3.70
NCT03619213	Dapagliflozin	Placebo	5.00 (1.10, 22.81)	10/3126	2/3127	3.08
NCT04157751	Empagliflozin	Placebo	0.51 (0.05, 5.56)	1/260	2/264	1.24
Overall (I-squared = 7.6%, p = 0.365)			0.79 (0.61, 1.04)	121/49693	124/42872	100.00

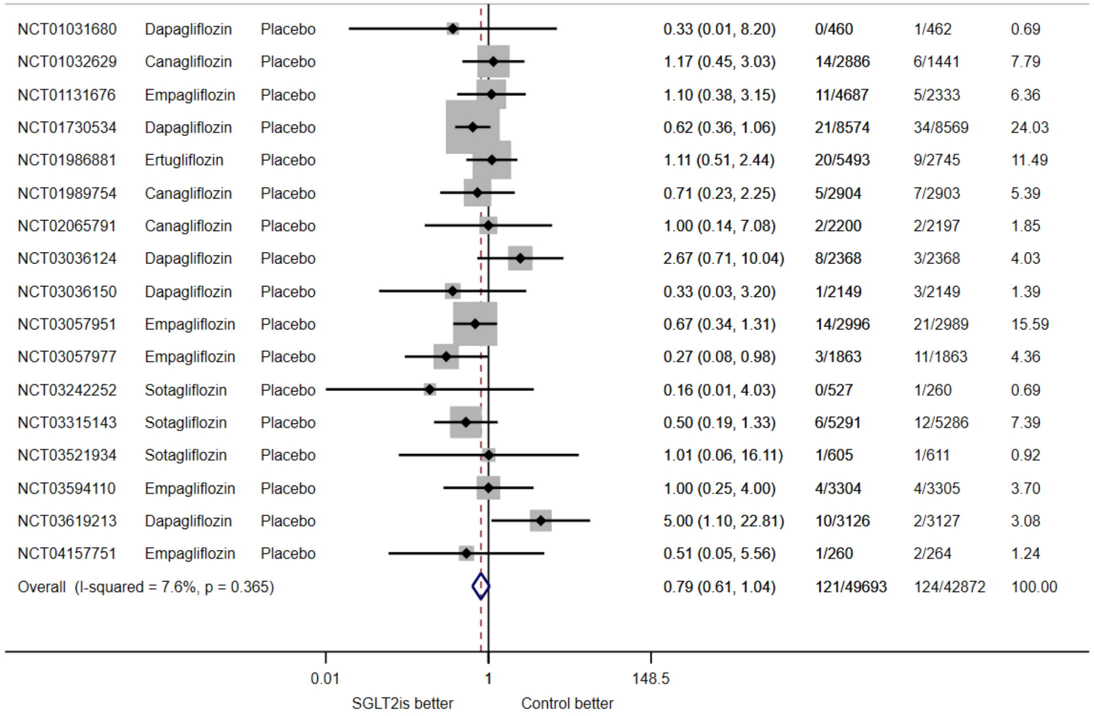

System 2-Cardiac Disorders-Figure S14 Meta-analysis of SGLT2is and Atrial flutter

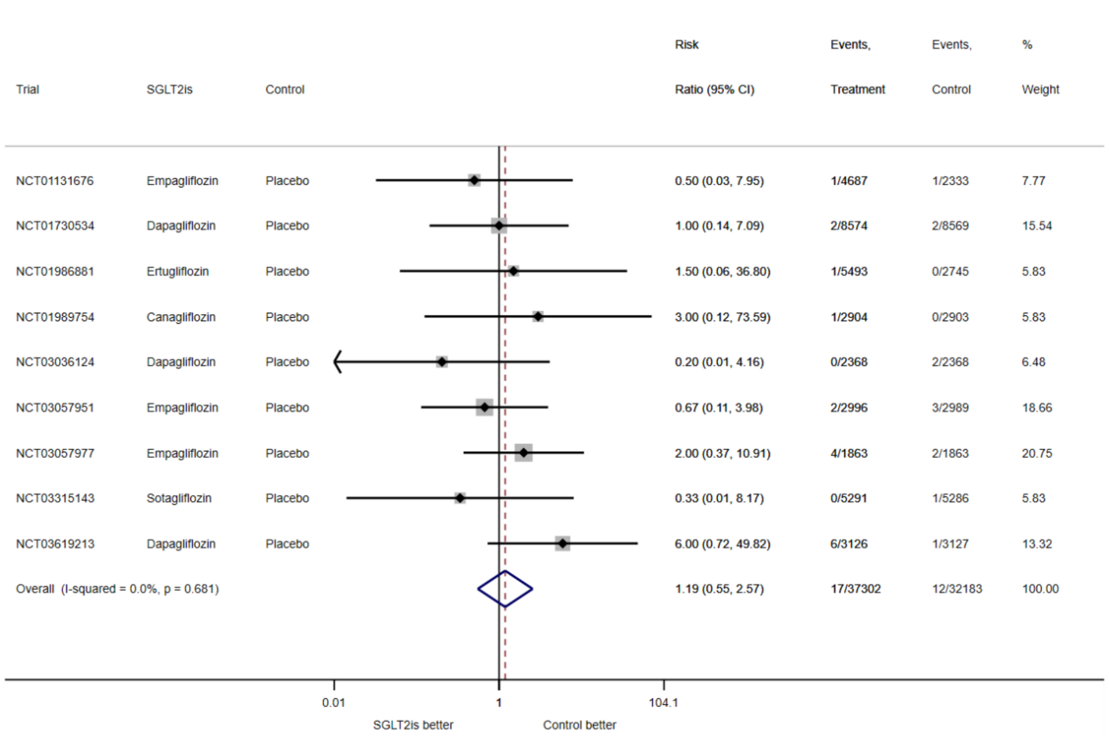

System 2-Cardiac Disorders-Figure S15 Meta-analysis of SGLT2is and Atrial tachycardia

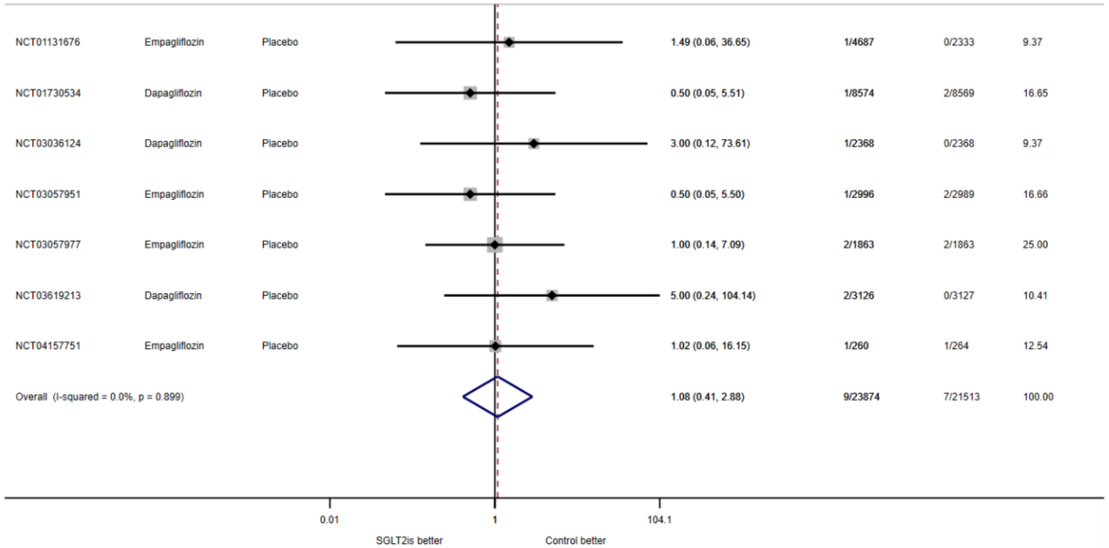

Trial	SGLT2is	Control	Risk Ratio (95% CI)	Events, Treatment	Events, Control	% Weight
NCT01131676	Empagliflozin	Placebo	1.49 (0.06, 36.65)	1/4687	0/2333	9.37
NCT01730534	Dapagliflozin	Placebo	0.50 (0.05, 5.51)	1/8574	2/8569	16.65
NCT03036124	Dapagliflozin	Placebo	3.00 (0.12, 73.61)	1/2368	0/2368	9.37
NCT03057951	Empagliflozin	Placebo	0.50 (0.05, 5.50)	1/2996	2/2989	16.66
NCT03057977	Empagliflozin	Placebo	1.00 (0.14, 7.09)	2/1863	2/1863	25.00
NCT03619213	Dapagliflozin	Placebo	5.00 (0.24, 104.14)	2/3126	0/3127	10.41
NCT04157751	Empagliflozin	Placebo	1.02 (0.06, 16.15)	1/260	1/264	12.54
Overall (I-squared = 0.0%, p = 0.899)			1.08 (0.41, 2.88)	9/23874	7/21513	100.00

System 2-Cardiac Disorders-Figure S16 Meta-analysis of SGLT2is and Atrial thrombosis

Trial	SGLT2is	Control	Risk Ratio (95% CI)	Events, Treatment	Events, Control	% Weight
NCT01032629	Canagliflozin	Placebo	0.25 (0.02, 2.75)	1/2886	2/1441	5.92
NCT01131676	Empagliflozin	Placebo	0.50 (0.10, 2.46)	3/4687	3/2333	13.33
NCT01164501	Empagliflozin	Placebo	2.29 (0.09, 55.92)	1/419	0/319	3.34
NCT01730534	Dapagliflozin	Placebo	0.29 (0.06, 1.37)	2/8574	7/8569	13.81
NCT01986881	Ertugliflozin	Placebo	1.00 (0.18, 5.45)	4/5493	2/2745	11.85
NCT02065791	Canagliflozin	Placebo	1.00 (0.14, 7.08)	2/2200	2/2197	8.89
NCT03036150	Dapagliflozin	Placebo	1.00 (0.14, 7.09)	2/2149	2/2149	8.89
NCT03057951	Empagliflozin	Placebo	1.00 (0.14, 7.08)	2/2996	2/2989	8.88
NCT03315143	Sotagliflozin	Placebo	0.33 (0.01, 8.17)	0/5291	1/5286	3.33
NCT03521934	Sotagliflozin	Placebo	1.01 (0.06, 16.11)	1/605	1/611	4.45
NCT03594110	Empagliflozin	Placebo	1.50 (0.25, 8.97)	3/3304	2/3305	10.66
NCT03619213	Dapagliflozin	Placebo	0.33 (0.03, 3.20)	1/3126	3/3127	6.66
Overall (I-squared = 0.0%, p = 0.950)			0.68 (0.38, 1.22)	22/41730	27/35071	100.00

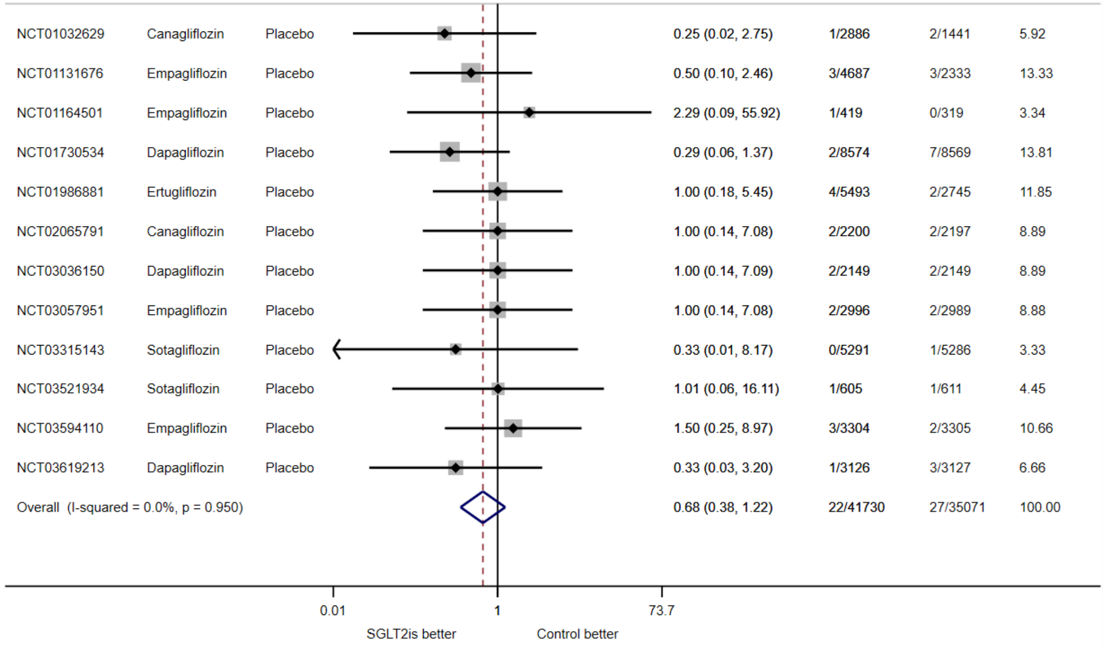

System 2-Cardiac Disorders-Figure S17 Meta-analysis of SGLT2is and Atrioventricular block

Trial	SGLT2is	Control	Risk Ratio (95% CI)	Events, Treatment	Events, Control	% Weight
NCT01032629	Canagliflozin	Placebo	1.25 (0.24, 6.43)	5/2886	2/1441	4.99
NCT01131676	Empagliflozin	Placebo	1.24 (0.24, 6.41)	5/4687	2/2333	4.98
NCT01730534	Dapagliflozin	Placebo	1.36 (0.68, 2.70)	19/8574	14/8569	28.15
NCT01986881	Ertugliflozin	Placebo	0.83 (0.20, 3.48)	5/5493	3/2745	6.54
NCT01989754	Canagliflozin	Placebo	3.00 (0.31, 28.81)	3/2904	1/2903	2.62
NCT02065791	Canagliflozin	Placebo	0.50 (0.05, 5.50)	1/2200	2/2197	2.33
NCT03036124	Dapagliflozin	Placebo	0.75 (0.17, 3.35)	3/2368	4/2368	5.98
NCT03036150	Dapagliflozin	Placebo	0.60 (0.14, 2.51)	3/2149	5/2149	6.55
NCT03057951	Empagliflozin	Placebo	2.19 (0.76, 6.31)	11/2996	5/2989	12.01
NCT03057977	Empagliflozin	Placebo	4.00 (0.45, 35.75)	4/1863	1/1863	2.79
NCT03315143	Sotagliflozin	Placebo	0.36 (0.13, 0.99)	5/5291	14/5286	12.86
NCT03594110	Empagliflozin	Placebo	3.00 (0.12, 73.64)	1/3304	0/3305	1.31
NCT03619213	Dapagliflozin	Placebo	1.75 (0.51, 5.97)	7/3126	4/3127	8.89
Overall (I-squared = 0.0%, p = 0.510)			1.13 (0.78, 1.63)	72/47841	57/41275	100.00

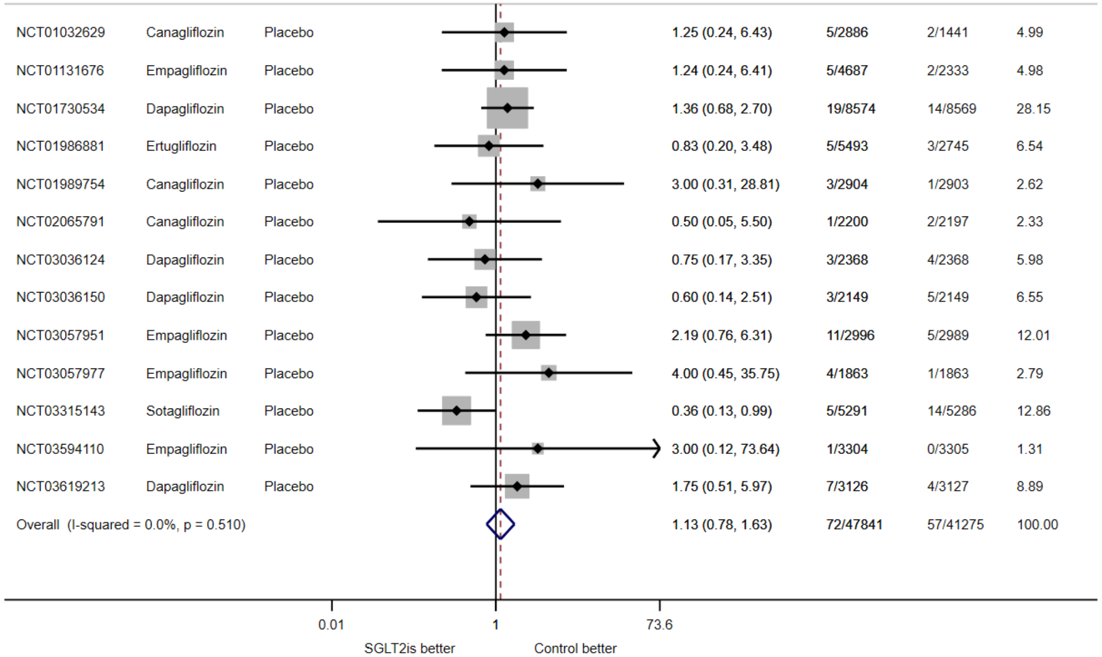

System 2-Cardiac Disorders-Figure S18 Meta-analysis of SGLT2is and Atrioventricular block complete

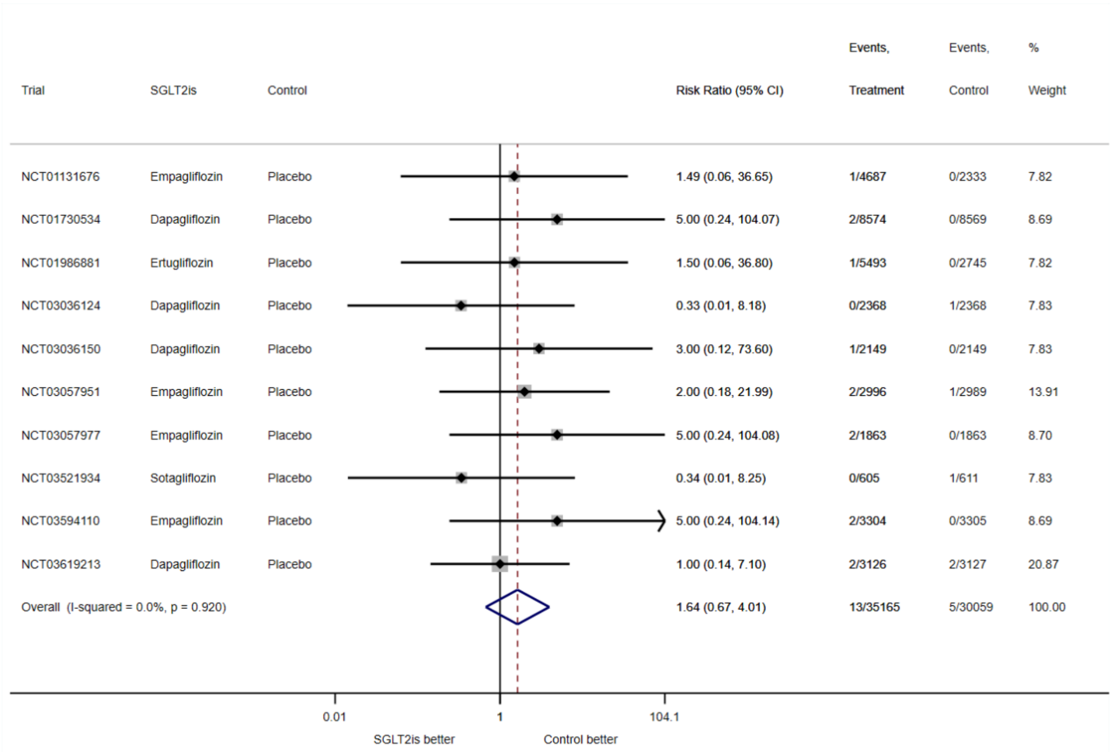

System 2-Cardiac Disorders-Figure S19 Meta-analysis of SGLT2is and Bradyarrhythmia

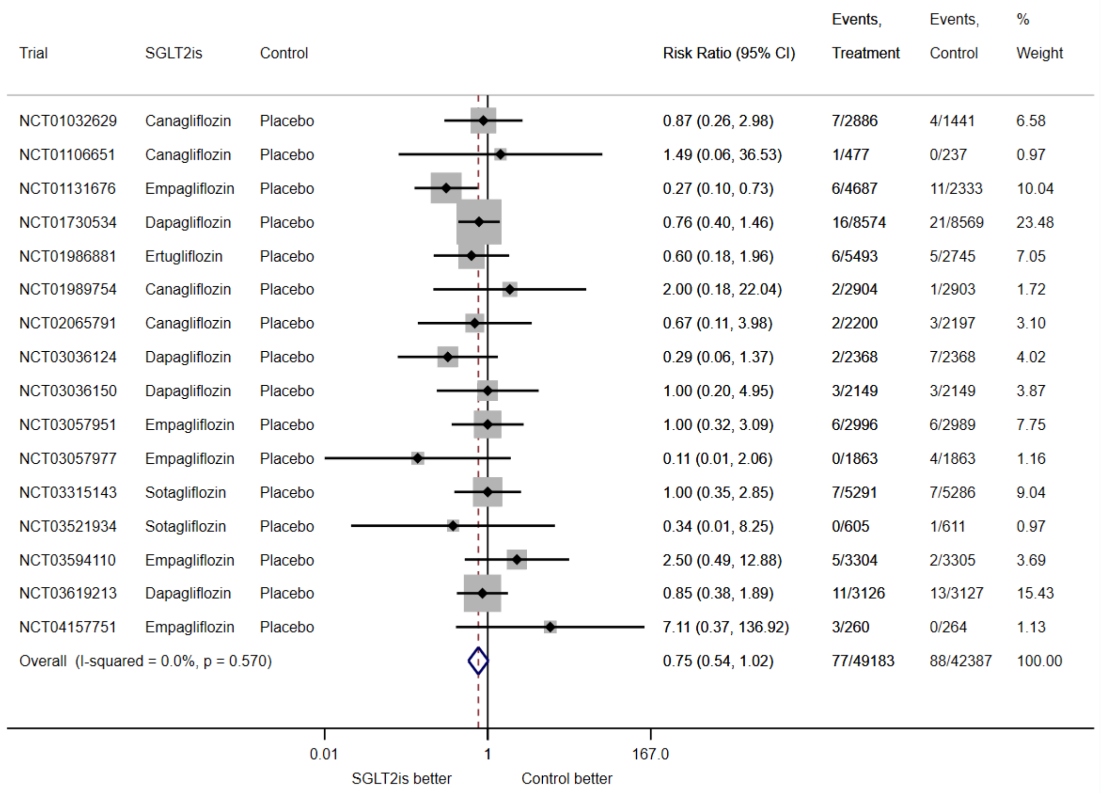

System 2-Cardiac Disorders-Figure S20 Meta-analysis of SGLT2is and Bradycardia

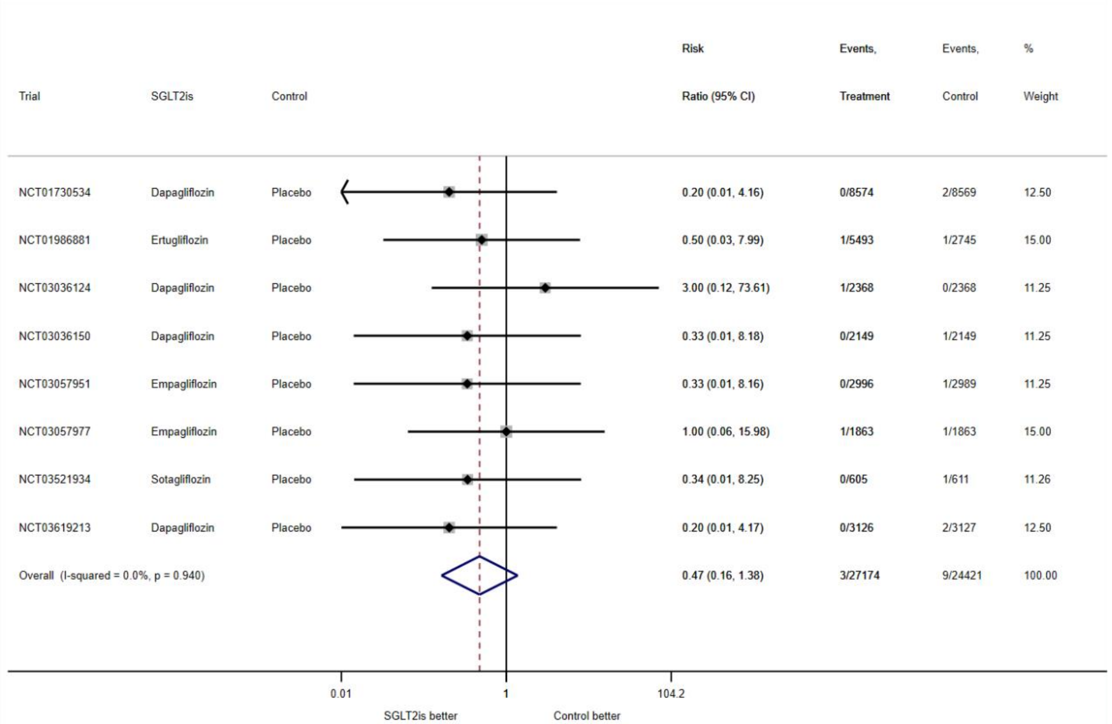

System 2-Cardiac Disorders-Figure S21 Meta-analysis of SGLT2is and Bundle branch block left

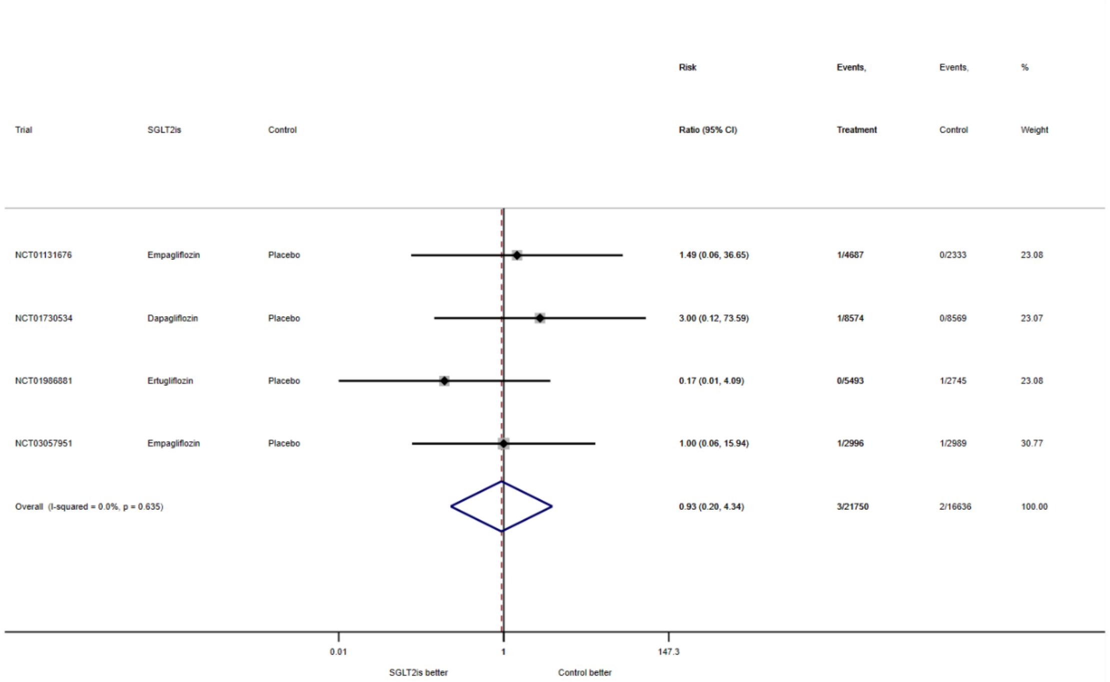

System 2-Cardiac Disorders-Figure S22 Meta-analysis of SGLT2is and Bundle branch block right

Trial	SGLT2is	Control	Risk Ratio (95% CI)	Events, Treatment	Events, Control	% Weight
NCT00528879	Dapagliflozin	Placebo	0.07 (0.00, 1.39)	0/409	2/137	0.56
NCT01032629	Canagliflozin	Placebo	1.08 (0.41, 2.84)	13/2886	6/1441	5.57
NCT01106677	Canagliflozin	Sitagliptin	0.17 (0.01, 4.07)	0/735	1/366	0.51
NCT01131676	Empagliflozin	Placebo	0.46 (0.20, 1.03)	11/4687	12/2333	7.78
NCT01164501	Empagliflozin	Placebo	0.76 (0.05, 12.13)	1/419	1/319	0.68
NCT01730534	Dapagliflozin	Placebo	1.06 (0.65, 1.74)	33/8574	31/8569	21.66
NCT01986881	Ertugliflozin	Placebo	0.85 (0.43, 1.68)	22/5493	13/2745	11.08
NCT01989754	Canagliflozin	Placebo	0.56 (0.19, 1.66)	5/2904	9/2903	4.35
NCT02065791	Canagliflozin	Placebo	0.71 (0.23, 2.24)	5/2200	7/2197	3.95
NCT03036124	Dapagliflozin	Placebo	0.90 (0.37, 2.21)	9/2368	10/2368	6.42
NCT03036150	Dapagliflozin	Placebo	0.40 (0.13, 1.27)	4/2149	10/2149	3.87
NCT03057951	Empagliflozin	Placebo	1.50 (0.61, 3.66)	12/2996	8/2989	6.50
NCT03057977	Empagliflozin	Placebo	0.90 (0.37, 2.21)	9/1863	10/1863	6.43
NCT03242252	Sotagliflozin	Placebo	1.48 (0.06, 36.28)	1/527	0/260	0.51
NCT03315143	Sotagliflozin	Placebo	0.45 (0.16, 1.31)	5/5291	11/5286	4.65
NCT03521934	Sotagliflozin	Placebo	3.03 (0.12, 74.22)	1/605	0/611	0.51
NCT03594110	Empagliflozin	Placebo	0.33 (0.01, 8.18)	0/3304	1/3305	0.51
NCT03619213	Dapagliflozin	Placebo	1.06 (0.55, 2.05)	18/3126	17/3127	11.87
NCT04157751	Empagliflozin	Placebo	0.41 (0.08, 2.07)	2/260	5/264	1.95
NCT04350593	Dapagliflozin	Placebo	1.00 (0.06, 16.03)	1/613	1/616	0.68
Overall (I-squared = 0.0%, p = 0.760)			0.82 (0.66, 1.03)	152/51409	155/43843	100.00

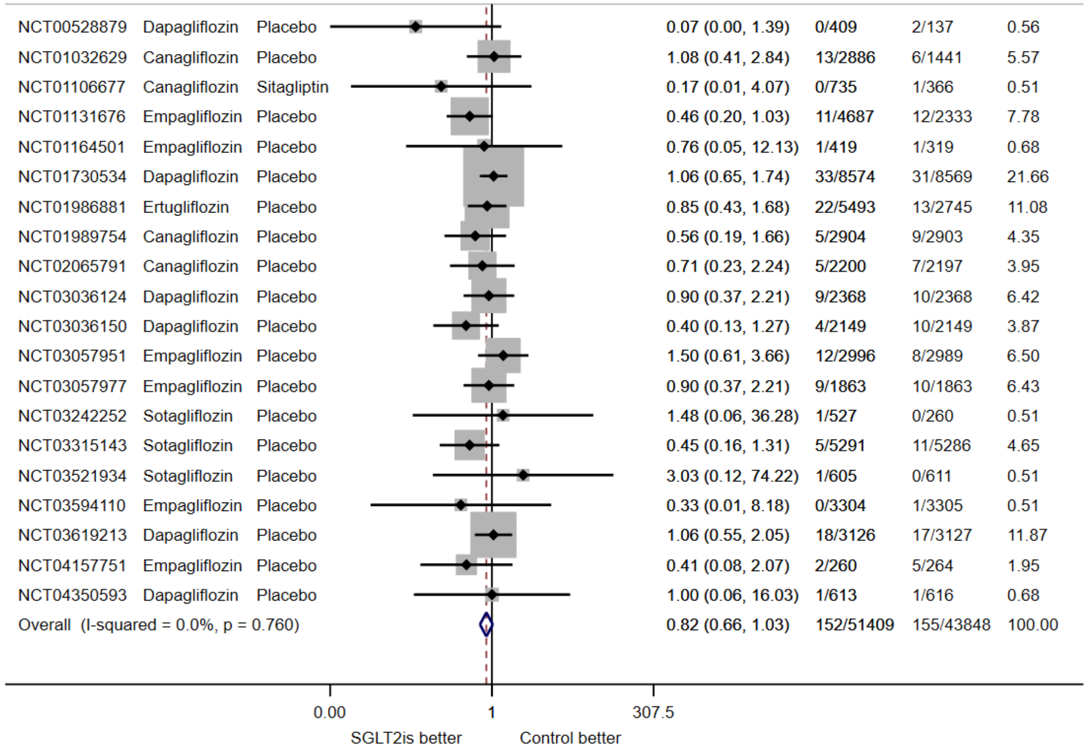

System 2-Cardiac Disorders-Figure S23 Meta-analysis of SGLT2is and Cardiac arrest

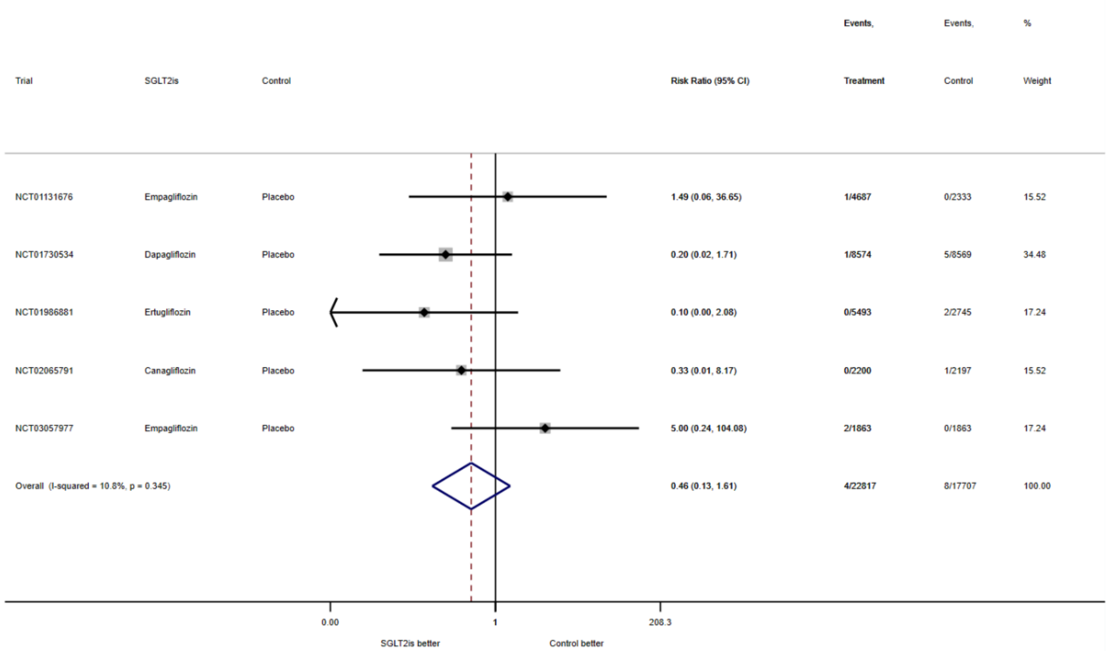

System 2-Cardiac Disorders-Figure S24 Meta-analysis of SGLT2is and Cardiac asthma

Trial	SGLT2is	Control	Risk Ratio (95% CI)	Events, Treatment	Events, Control	% Weight
NCT01032629	Canagliflozin	Placebo	2.50 (0.12, 51.99)	2/2886	0/1441	10.00
NCT01131676	Empagliflozin	Placebo	1.49 (0.06, 36.65)	1/4687	0/2333	9.00
NCT01730534	Dapagliflozin	Placebo	3.00 (0.12, 73.59)	1/8574	0/8569	9.00
NCT01986881	Ertugliflozin	Placebo	1.50 (0.06, 36.80)	1/5493	0/2745	9.00
NCT01989754	Canagliflozin	Placebo	3.00 (0.12, 73.59)	1/2904	0/2903	9.00
NCT02065791	Canagliflozin	Placebo	0.33 (0.01, 8.17)	0/2200	1/2197	9.00
NCT03036124	Dapagliflozin	Placebo	3.00 (0.12, 73.61)	1/2368	0/2368	9.00
NCT03057977	Empagliflozin	Placebo	3.00 (0.12, 73.59)	1/1863	0/1863	9.00
NCT03315143	Sotagliflozin	Placebo	3.00 (0.12, 73.56)	1/5291	0/5286	9.00
NCT03619213	Dapagliflozin	Placebo	0.33 (0.01, 8.18)	0/3126	1/3127	9.00
NCT04350593	Dapagliflozin	Placebo	3.01 (0.12, 73.86)	1/613	0/616	9.01
Overall (I-squared = 0.0%, p = 0.986)			1.75 (0.67, 4.57)	10/40005	2/33448	100.00

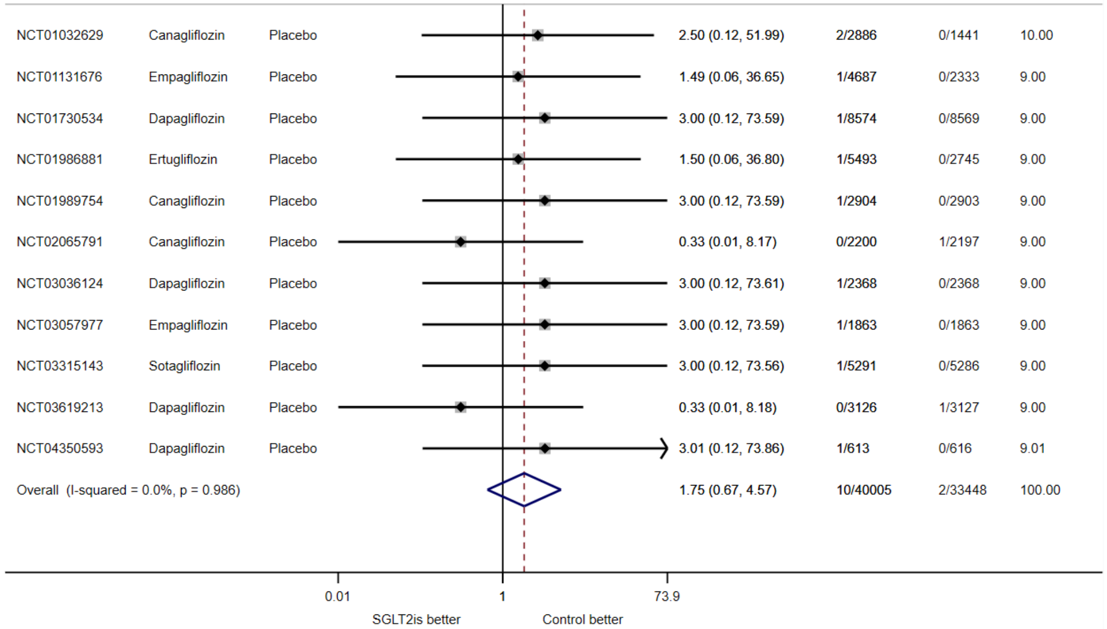

System 2-Cardiac Disorders-Figure S25 Meta-analysis of SGLT2is and Cardiac disorder

Trial	SGLT2is	Control	Risk Ratio (95% CI)	Events, Treatment	Events, Control	% Weight
NCT01032629	Canagliflozin	Placebo	1.10 (0.64, 1.89)	42/2886	19/1441	1.04
NCT01042977	Dapagliflozin	Placebo	1.00 (0.06, 15.97)	1/482	1/483	0.04
NCT01131676	Empagliflozin	Placebo	0.60 (0.42, 0.85)	66/4687	55/2333	2.39
NCT01164501	Empagliflozin	Placebo	0.11 (0.01, 2.10)	0/419	3/319	0.03
NCT01730534	Dapagliflozin	Placebo	0.78 (0.63, 0.97)	147/8574	188/8569	6.58
NCT01986881	Ertugliflozin	Placebo	0.80 (0.55, 1.17)	69/5493	43/2745	2.10
NCT01989754	Canagliflozin	Placebo	0.46 (0.27, 0.80)	19/2904	41/2903	1.03
NCT02033889	Ertugliflozin	Placebo/Glimepiride	1.53 (0.06, 37.28)	1/412	0/209	0.03
NCT02065791	Canagliflozin	Placebo	0.68 (0.44, 1.05)	34/2200	50/2197	1.61
NCT03036124	Dapagliflozin	Placebo	0.75 (0.64, 0.87)	262/2368	351/2368	13.45
NCT03036150	Dapagliflozin	Placebo	0.66 (0.44, 0.99)	39/2149	59/2149	1.88
NCT03057951	Empagliflozin	Placebo	0.75 (0.67, 0.84)	448/2996	594/2989	24.11
NCT03057977	Empagliflozin	Placebo	0.75 (0.66, 0.85)	332/1863	444/1863	18.68
NCT03242252	Sotagliflozin	Placebo	0.16 (0.01, 4.03)	0/527	1/260	0.03
NCT03315143	Sotagliflozin	Placebo	0.64 (0.49, 0.83)	94/5291	147/5286	4.59
NCT03521934	Sotagliflozin	Placebo	0.67 (0.52, 0.87)	80/605	121/611	4.48
NCT03594110	Empagliflozin	Placebo	0.93 (0.61, 1.42)	41/3304	44/3305	1.68
NCT03619213	Dapagliflozin	Placebo	0.82 (0.71, 0.94)	308/3126	377/3127	14.88
NCT04157751	Empagliflozin	Placebo	0.69 (0.43, 1.11)	25/260	37/264	1.32
NCT04350593	Dapagliflozin	Placebo	1.00 (0.06, 16.03)	1/613	1/616	0.04
Overall (I-squared = 0.0%, p = 0.720)			0.75 (0.71, 0.79)	2009/51159	2576/44037	100.00

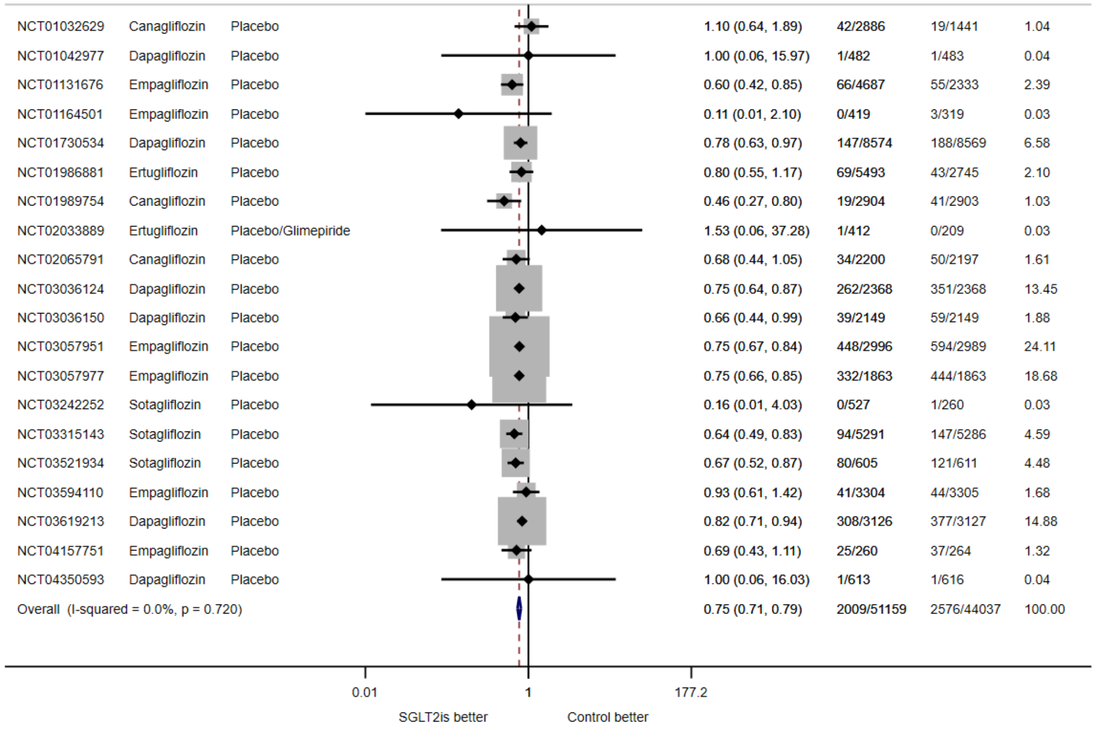

System 2-Cardiac Disorders-Figure S26 Meta-analysis of SGLT2is and Cardiac failure

Trial	SGLT2is	Control	Risk Ratio (95% CI)	Events, Treatment	Events, Control	% Weight
NCT01032629	Canagliflozin	Placebo	0.25 (0.02, 2.75)	1/2886	2/1441	0.54
NCT01131676	Empagliflozin	Placebo	0.33 (0.12, 0.93)	6/4687	9/2333	2.90
NCT01730534	Dapagliflozin	Placebo	0.71 (0.41, 1.22)	22/8574	31/8569	10.39
NCT01986881	Ertugliflozin	Placebo	0.87 (0.37, 2.08)	14/5493	8/2745	4.11
NCT01989754	Canagliflozin	Placebo	0.67 (0.11, 3.99)	2/2904	3/2903	0.97
NCT02033889	Ertugliflozin	Placebo/Glimepiride	1.53 (0.06, 37.28)	1/412	0/209	0.30
NCT02065791	Canagliflozin	Placebo	1.00 (0.32, 3.09)	6/2200	6/2197	2.42
NCT03036124	Dapagliflozin	Placebo	0.71 (0.48, 1.05)	42/2368	59/2368	20.16
NCT03036150	Dapagliflozin	Placebo	0.57 (0.17, 1.95)	4/2149	7/2149	2.05
NCT03057951	Empagliflozin	Placebo	0.41 (0.21, 0.81)	12/2996	29/2989	6.87
NCT03057977	Empagliflozin	Placebo	0.66 (0.37, 1.16)	19/1863	29/1863	9.35
NCT03242252	Sotagliflozin	Placebo	0.16 (0.01, 4.03)	0/527	1/260	0.30
NCT03315143	Sotagliflozin	Placebo	0.64 (0.36, 1.16)	18/5291	28/5286	8.85
NCT03521934	Sotagliflozin	Placebo	1.10 (0.49, 2.48)	12/605	11/611	4.71
NCT03619213	Dapagliflozin	Placebo	0.91 (0.63, 1.29)	57/3126	63/3127	24.55
NCT04157751	Empagliflozin	Placebo	1.02 (0.21, 4.98)	3/260	3/264	1.22
NCT04350593	Dapagliflozin	Placebo	3.01 (0.12, 73.86)	1/613	0/616	0.30
Overall (I-squared = 0.0%, p = 0.803)			0.72 (0.61, 0.86)	220/46954	289/39930	100.00

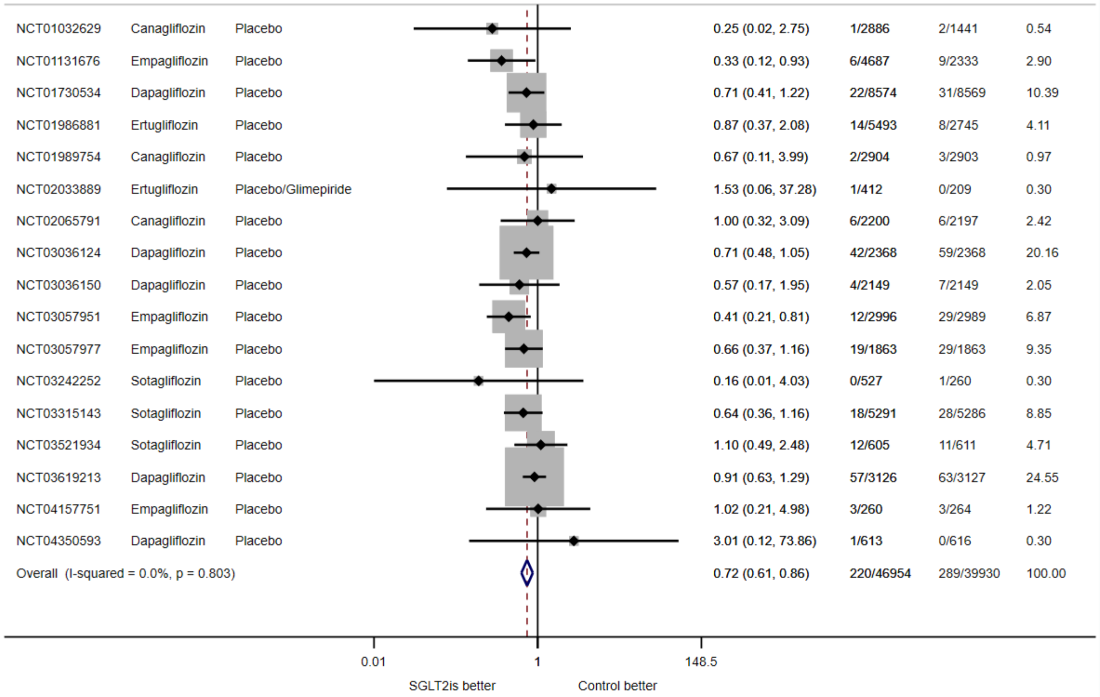

System 2-Cardiac Disorders-Figure S27 Meta-analysis of SGLT2is and Cardiac failure acute

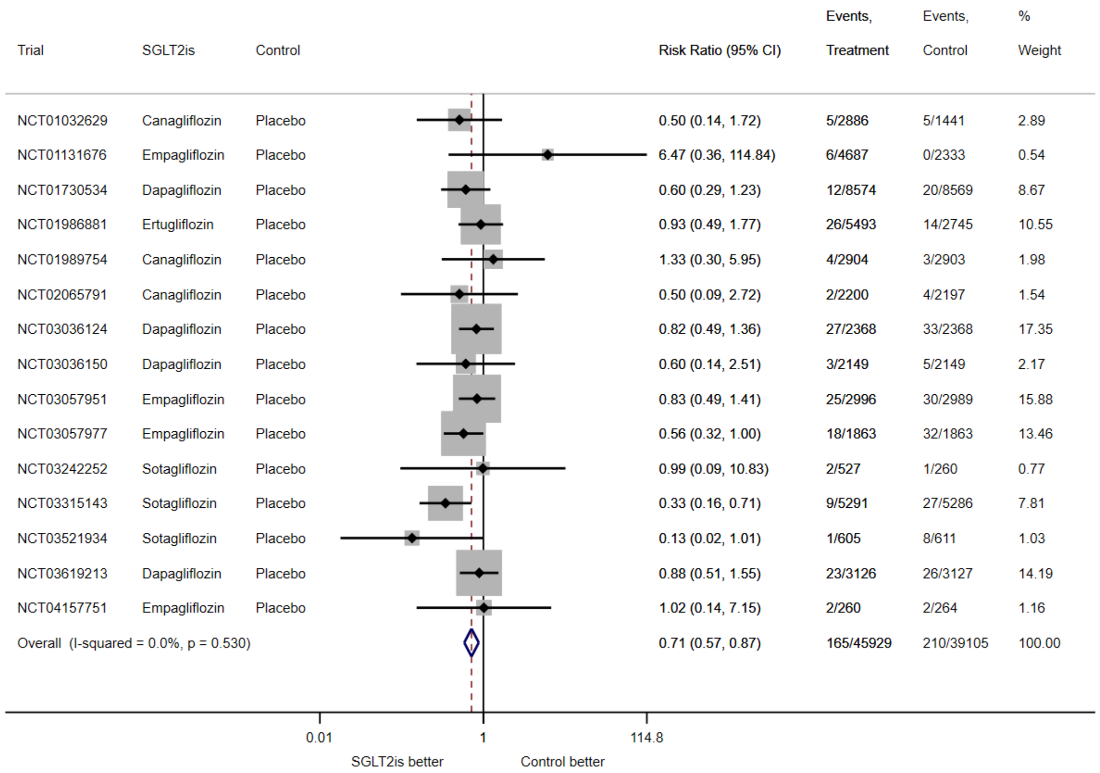

System 2-Cardiac Disorders-Figure S28 Meta-analysis of SGLT2is and Cardiac failure chronic

Trial	SGLT2is	Control	Risk Ratio (95% CI)	Events, Treatment	Events, Control	% Weight
NCT01031680	Dapagliflozin	Placebo	0.33 (0.01, 8.20)	0/460	1/462	0.12
NCT01032629	Canagliflozin	Placebo	0.72 (0.41, 1.28)	29/2886	20/1441	3.95
NCT01042977	Dapagliflozin	Placebo	0.33 (0.01, 8.18)	0/482	1/483	0.12
NCT01106651	Canagliflozin	Placebo	1.49 (0.06, 36.53)	1/477	0/237	0.12
NCT01131676	Empagliflozin	Placebo	0.72 (0.49, 1.05)	65/4687	45/2333	8.92
NCT01164501	Empagliflozin	Placebo	0.25 (0.01, 6.21)	0/419	1/319	0.12
NCT01730534	Dapagliflozin	Placebo	0.82 (0.65, 1.04)	121/8574	147/8569	22.22
NCT01986881	Ertugliflozin	Placebo	0.67 (0.43, 1.04)	47/5493	35/2745	6.68
NCT01989754	Canagliflozin	Placebo	0.61 (0.29, 1.29)	11/2904	18/2903	2.26
NCT02033889	Ertugliflozin	Placebo/Glimepiride	1.53 (0.06, 37.28)	1/412	0/209	0.12
NCT02065791	Canagliflozin	Placebo	0.50 (0.31, 0.81)	24/2200	48/2197	5.35
NCT02384941	Sotagliflozin	Placebo	1.53 (0.06, 37.53)	1/525	0/268	0.12
NCT03036124	Dapagliflozin	Placebo	0.93 (0.67, 1.30)	65/2368	70/2368	11.43
NCT03036150	Dapagliflozin	Placebo	0.59 (0.30, 1.17)	13/2149	22/2149	2.71
NCT03057951	Empagliflozin	Placebo	0.86 (0.61, 1.22)	57/2996	66/2989	10.29
NCT03057977	Empagliflozin	Placebo	0.63 (0.39, 1.03)	26/1863	41/1863	5.33
NCT03242252	Sotagliflozin	Placebo	1.48 (0.06, 36.28)	1/527	0/260	0.12
NCT03315143	Sotagliflozin	Placebo	0.50 (0.30, 0.82)	23/5291	46/5286	5.08
NCT03521934	Sotagliflozin	Placebo	0.69 (0.32, 1.48)	11/605	16/611	2.20
NCT03619213	Dapagliflozin	Placebo	0.77 (0.56, 1.06)	63/3126	82/3127	12.02
NCT04157751	Empagliflozin	Placebo	0.18 (0.04, 0.82)	2/260	11/264	0.57
NCT04350593	Dapagliflozin	Placebo	0.33 (0.01, 8.21)	0/613	1/616	0.12
Overall (I-squared = 0.0%, p = 0.838)			0.73 (0.65, 0.82)	561/49317	671/41699	100.00

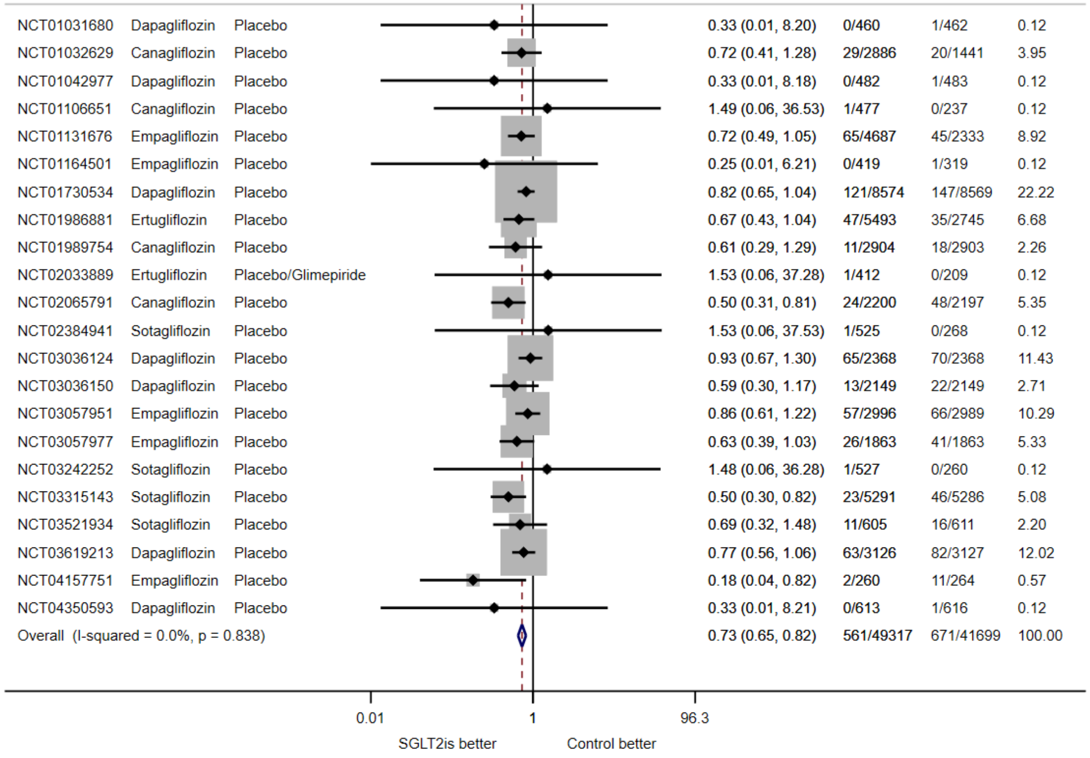

System 2-Cardiac Disorders-Figure S29 Meta-analysis of SGLT2is and Cardiac failure congestive

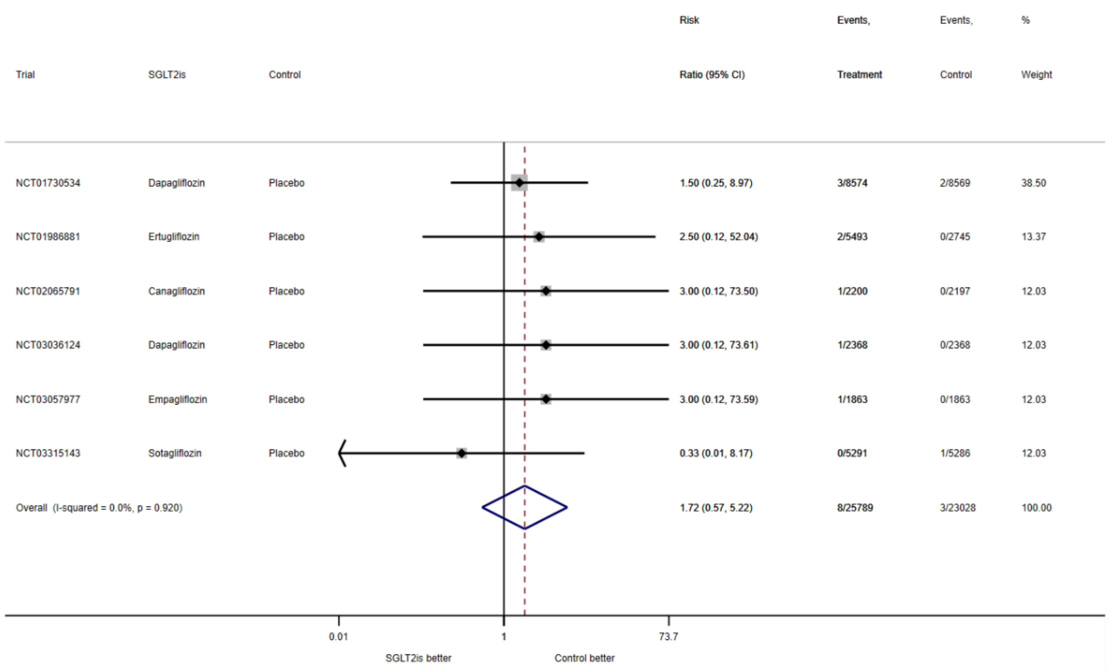

System 2-Cardiac Disorders-Figure S30 Meta-analysis of SGLT2is and Cardiac tamponade

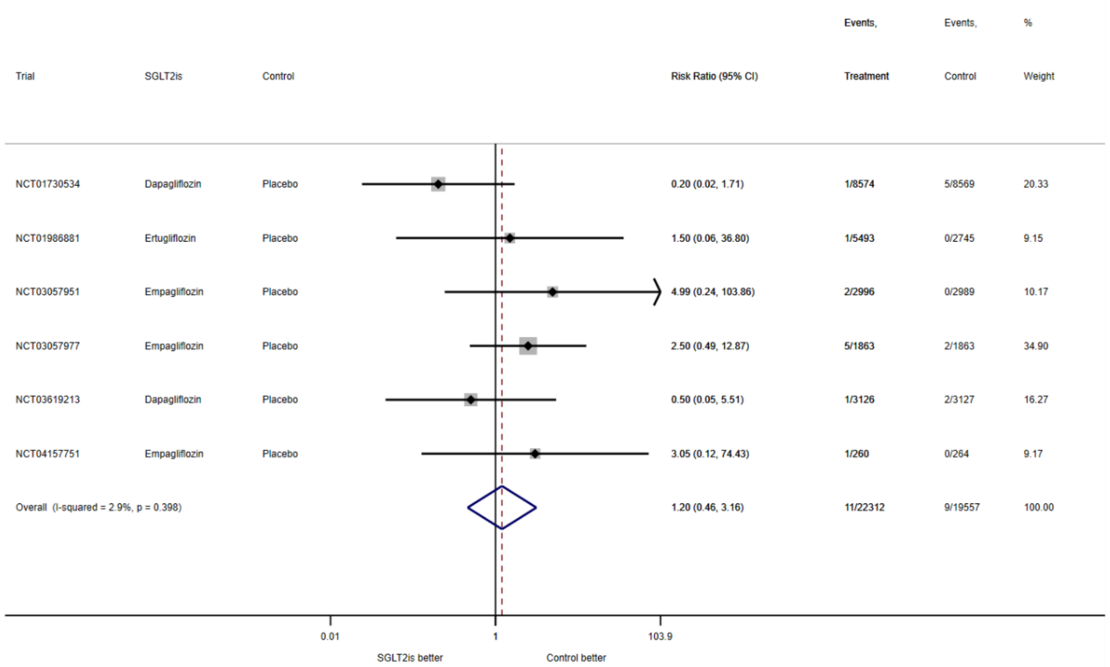

System 2-Cardiac Disorders-Figure S31 Meta-analysis of SGLT2is and Cardiac ventricular thrombosis

Trial	SGLT2is	Control	Risk Ratio (95% CI)	Events, Treatment	Events, Control	% Weight
NCT00528879	Dapagliflozin	Placebo	1.01 (0.04, 24.64)	1/409	0/137	1.41
NCT01032629	Canagliflozin	Placebo	1.00 (0.18, 5.45)	4/2886	2/1441	5.00
NCT01131676	Empagliflozin	Placebo	0.58 (0.20, 1.73)	7/4687	6/2333	12.11
NCT01730534	Dapagliflozin	Placebo	1.09 (0.48, 2.47)	12/8574	11/8569	21.50
NCT01986881	Ertugliflozin	Placebo	2.25 (0.49, 10.40)	9/5493	2/2745	6.13
NCT01989754	Canagliflozin	Placebo	1.00 (0.06, 15.97)	1/2904	1/2903	1.87
NCT02065791	Canagliflozin	Placebo	1.33 (0.30, 5.94)	4/2200	3/2197	6.42
NCT03036124	Dapagliflozin	Placebo	1.00 (0.14, 7.09)	2/2368	2/2368	3.74
NCT03036150	Dapagliflozin	Placebo	1.67 (0.40, 6.97)	5/2149	3/2149	7.03
NCT03057951	Empagliflozin	Placebo	2.49 (0.48, 12.85)	5/2996	2/2989	5.35
NCT03057977	Empagliflozin	Placebo	0.25 (0.03, 2.23)	1/1863	4/1863	3.00
NCT03242252	Sotagliflozin	Placebo	0.16 (0.01, 4.03)	0/527	1/260	1.41
NCT03315143	Sotagliflozin	Placebo	0.86 (0.29, 2.55)	6/5291	7/5286	12.10
NCT03521934	Sotagliflozin	Placebo	3.03 (0.12, 74.22)	1/605	0/611	1.40
NCT03619213	Dapagliflozin	Placebo	0.60 (0.14, 2.51)	3/3126	5/3127	7.02
NCT04350593	Dapagliflozin	Placebo	1.51 (0.25, 8.99)	3/613	2/616	4.51
Overall (I-squared = 0.0%, p = 0.928)			1.02 (0.70, 1.49)	64/46691	51/39594	100.00

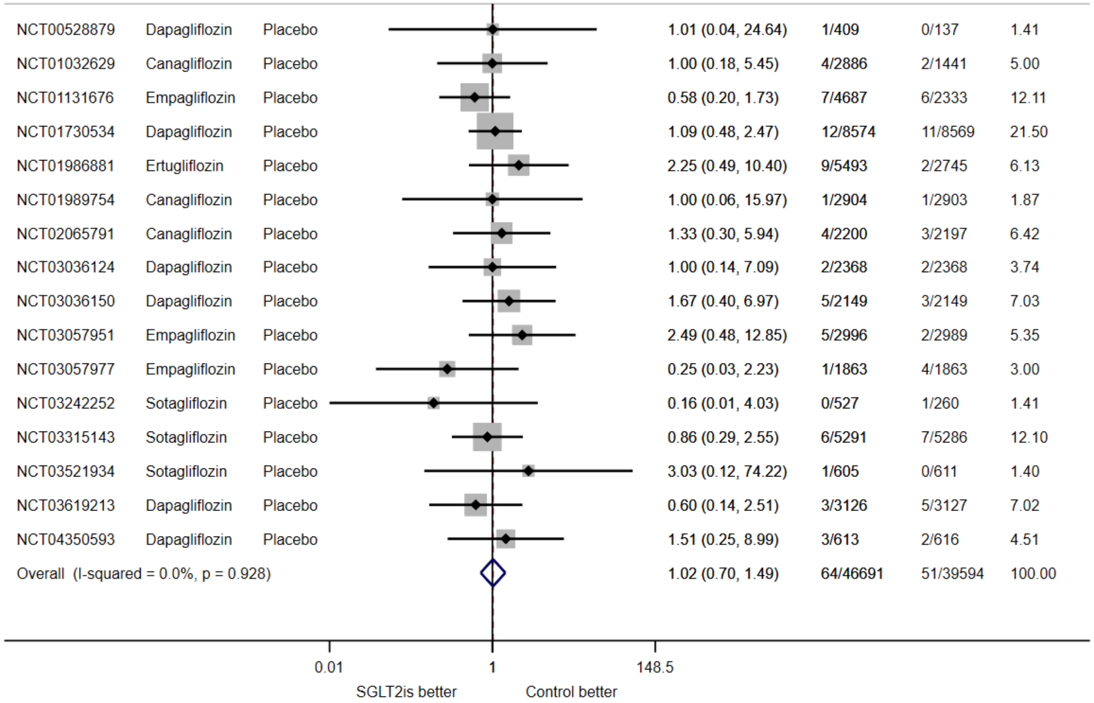

System 2-Cardiac Disorders-Figure S32 Meta-analysis of SGLT2is and Cardio-respiratory arrest

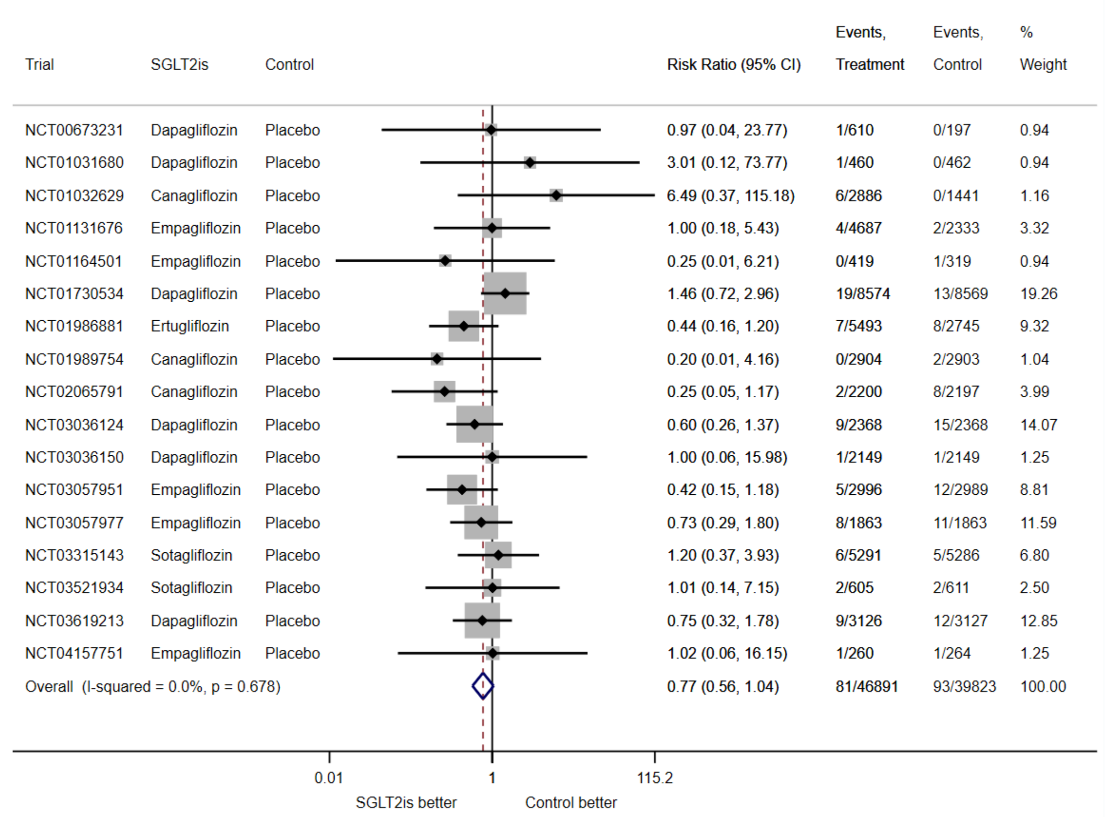

System 2-Cardiac Disorders-Figure S33 Meta-analysis of SGLT2is and Cardiogenic shock

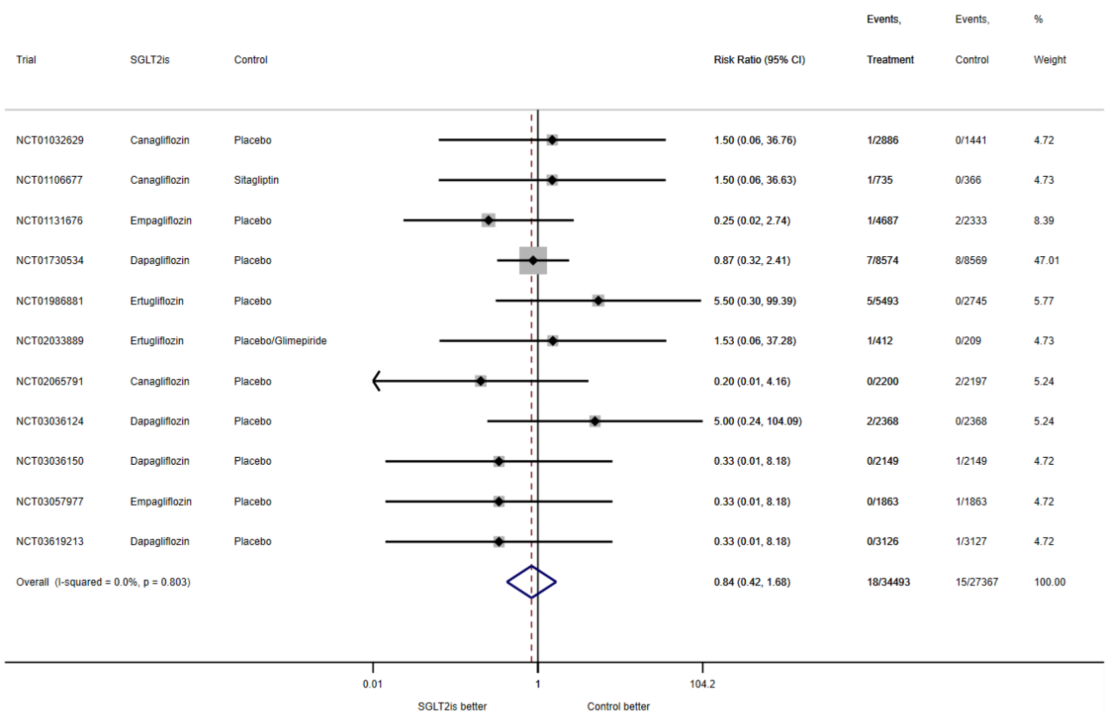

System 2-Cardiac Disorders-Figure S34 Meta-analysis of SGLT2is and Cardiomyopathy

Trial	SGLT2is	Control	Risk Ratio (95% CI)	Events, Treatment	Events, Control	% Weight
NCT01032629	Canagliflozin	Placebo	0.17 (0.01, 4.08)	0/2886	1/1441	4.26
NCT01131676	Empagliflozin	Placebo	0.25 (0.02, 2.74)	1/4687	2/2333	7.57
NCT01730534	Dapagliflozin	Placebo	0.43 (0.11, 1.66)	3/8574	7/8569	23.84
NCT01986881	Ertugliflozin	Placebo	0.17 (0.02, 1.60)	1/5493	3/2745	8.51
NCT01989754	Canagliflozin	Placebo	3.00 (0.12, 73.59)	1/2904	0/2903	4.26
NCT03036124	Dapagliflozin	Placebo	2.00 (0.18, 22.04)	2/2368	1/2368	7.57
NCT03036150	Dapagliflozin	Placebo	1.00 (0.06, 15.98)	1/2149	1/2149	5.68
NCT03057951	Empagliflozin	Placebo	1.00 (0.14, 7.08)	2/2996	2/2989	11.35
NCT03057977	Empagliflozin	Placebo	1.00 (0.06, 15.98)	1/1863	1/1863	5.68
NCT03315143	Sotagliflozin	Placebo	3.00 (0.12, 73.56)	1/5291	0/5286	4.26
NCT03619213	Dapagliflozin	Placebo	1.00 (0.20, 4.95)	3/3126	3/3127	17.04
Overall (I-squared = 0.0%, p = 0.790)			0.68 (0.35, 1.31)	16/42337	21/35773	100.00

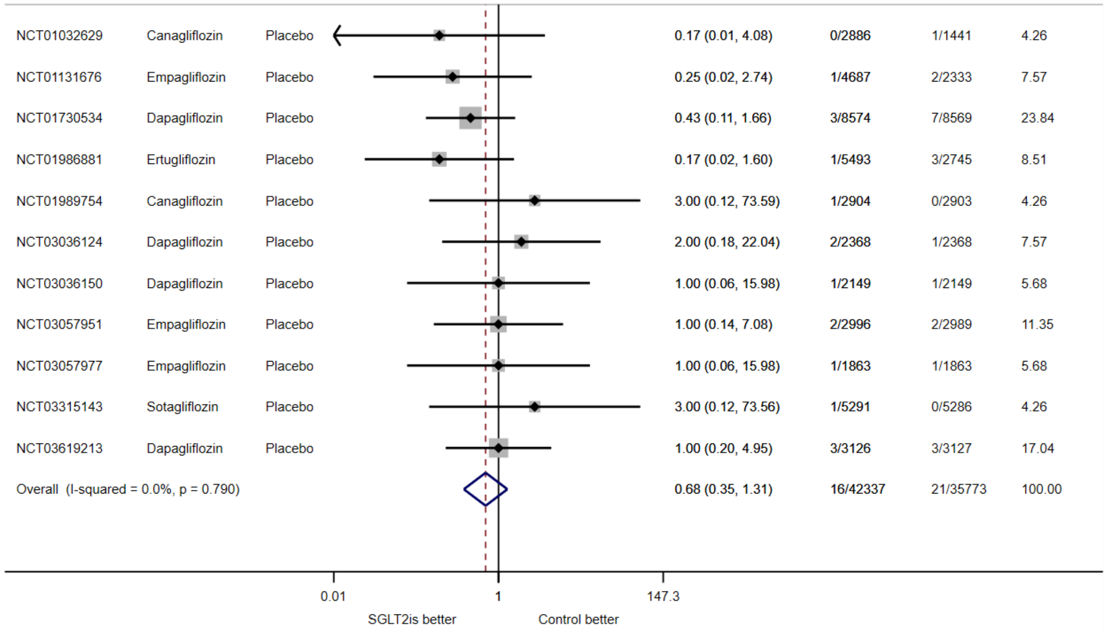

System 2-Cardiac Disorders-Figure S35 Meta-analysis of SGLT2is and Cardiopulmonary failure

Trial	SGLT2is	Control	Risk Ratio (95% CI)	Events, Treatment	Events, Control	% Weight
NCT01131676	Empagliflozin	Placebo	1.49 (0.06, 36.65)	1/4687	0/2333	10.59
NCT02065791	Canagliflozin	Placebo	1.00 (0.06, 15.96)	1/2200	1/2197	14.12
NCT03036124	Dapagliflozin	Placebo	0.33 (0.03, 3.20)	1/2368	3/2368	21.18
NCT03036150	Dapagliflozin	Placebo	0.33 (0.01, 8.18)	0/2149	1/2149	10.59
NCT03057951	Empagliflozin	Placebo	2.00 (0.18, 21.99)	2/2996	1/2989	18.82
NCT03057977	Empagliflozin	Placebo	0.33 (0.01, 8.18)	0/1863	1/1863	10.59
NCT03619213	Dapagliflozin	Placebo	1.00 (0.06, 15.99)	1/3126	1/3127	14.12
Overall (I-squared = 0.0%, p = 0.930)			0.75 (0.26, 2.11)	6/19389	8/17026	100.00

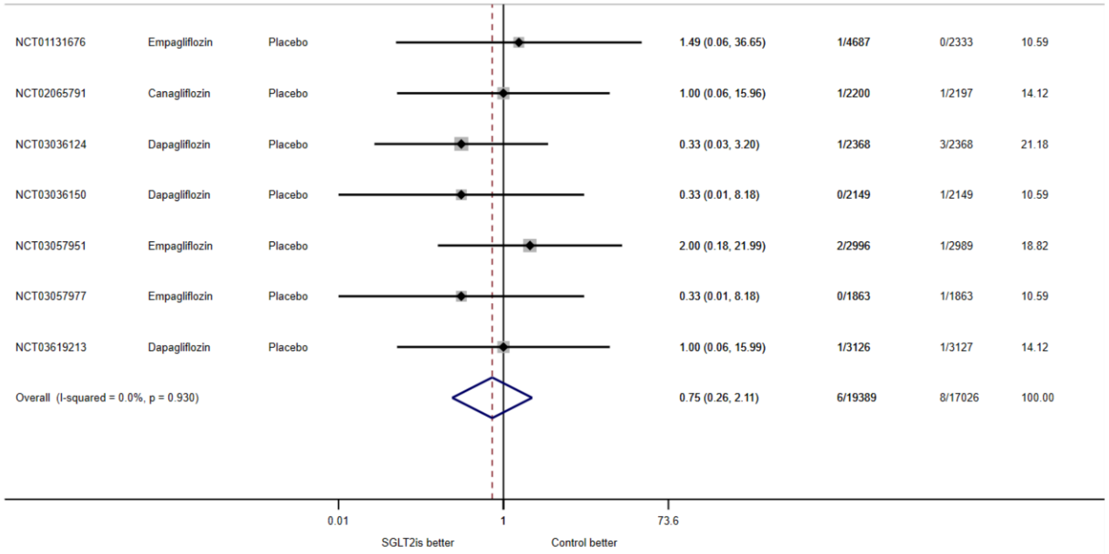

System 2-Cardiac Disorders-Figure S36 Meta-analysis of SGLT2is and Cardiorenal syndrome

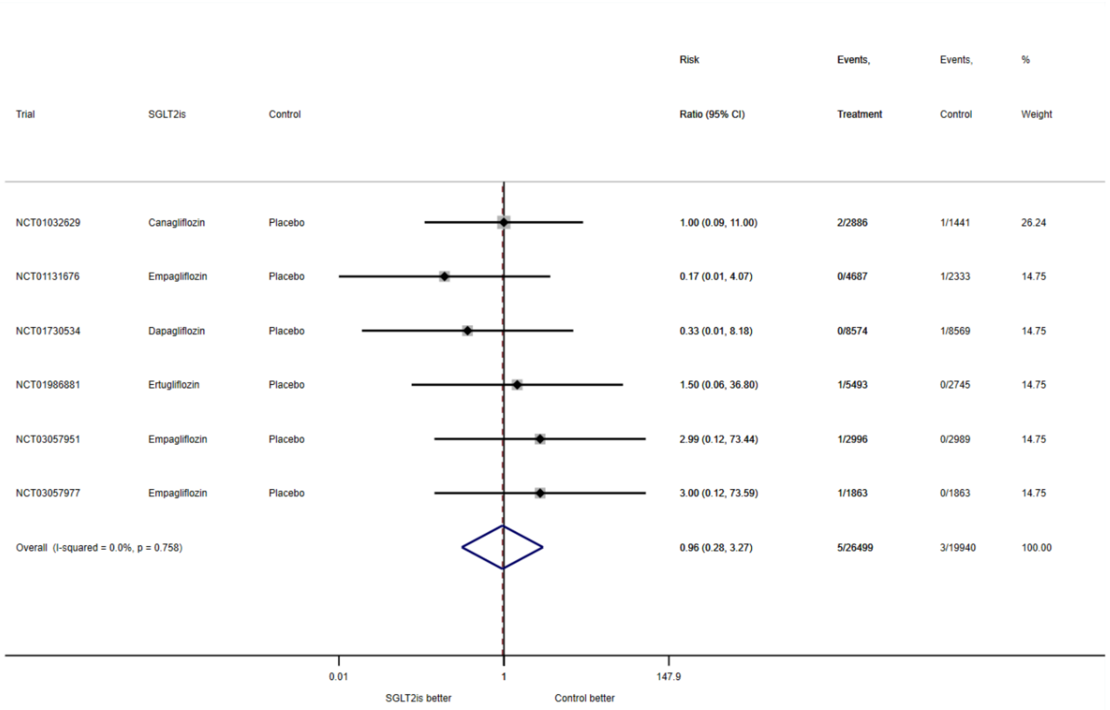

System 2-Cardiac Disorders-Figure S37 Meta-analysis of SGLT2is and Cardiovascular disorder

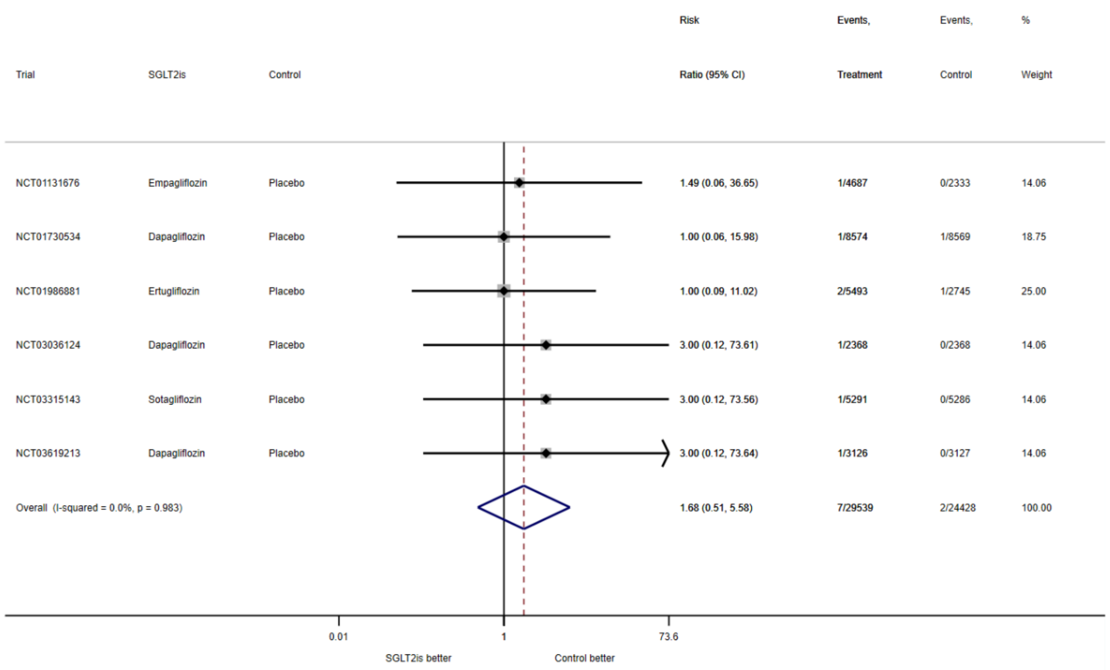

System 2-Cardiac Disorders-Figure S38 Meta-analysis of SGLT2is and Cardiovascular insufficiency

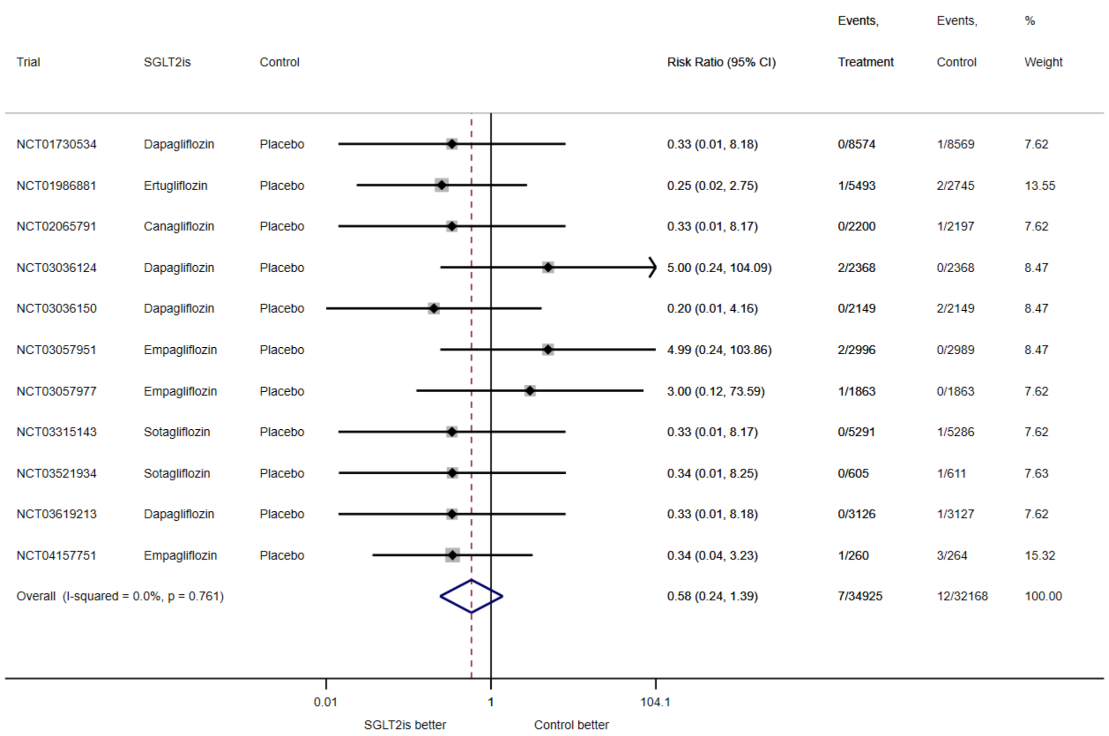

System 2-Cardiac Disorders-Figure S39 Meta-analysis of SGLT2is and Chronic left ventricular failure

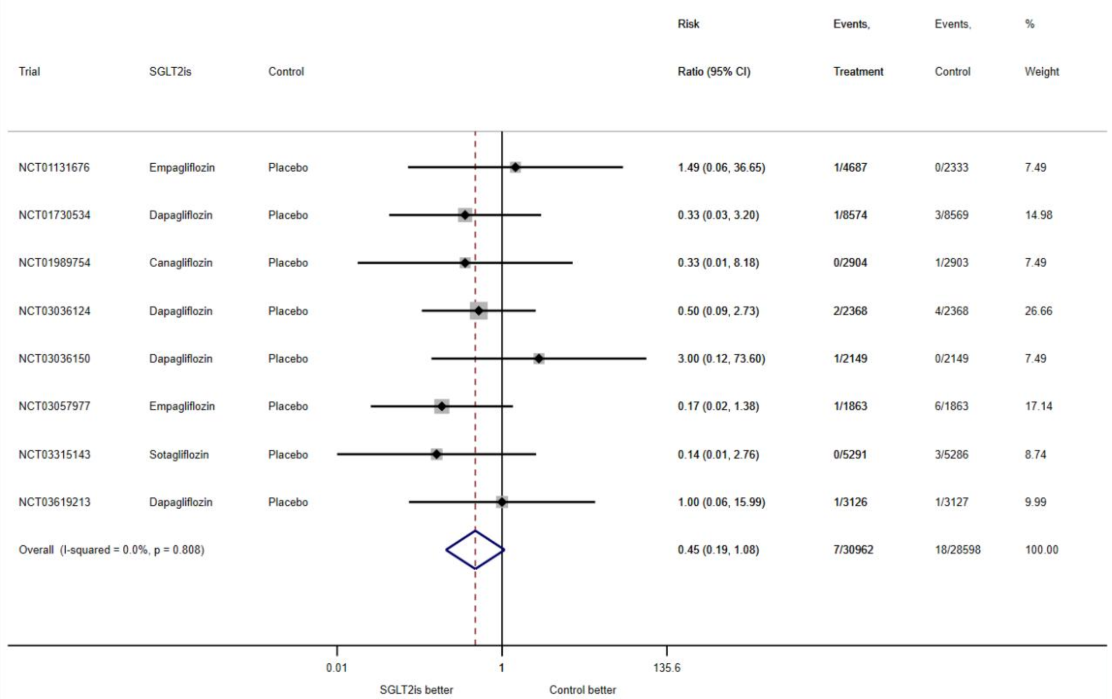

System 2-Cardiac Disorders-Figure S40 Meta-analysis of SGLT2is and Congestive cardiomyopathy

Trial	SGLT2is	Control	Risk Ratio (95% CI)	Events, Treatment	Events, Control	% Weight
NCT01031680	Dapagliflozin	Placebo	1.00 (0.06, 16.01)	1/460	1/462	0.28
NCT01032629	Canagliflozin	Placebo	0.98 (0.60, 1.59)	47/2886	24/1441	8.87
NCT01042977	Dapagliflozin	Placebo	1.00 (0.06, 15.97)	1/482	1/483	0.28
NCT01106651	Canagliflozin	Placebo	0.99 (0.25, 3.94)	6/477	3/237	1.11
NCT01106677	Canagliflozin	Sitagliptin	0.50 (0.03, 7.94)	1/735	1/366	0.28
NCT01131676	Empagliflozin	Placebo	0.54 (0.36, 0.80)	50/4687	46/2333	13.36
NCT01730534	Dapagliflozin	Placebo	1.26 (0.95, 1.68)	106/8574	84/8569	26.02
NCT01986881	Ertugliflozin	Placebo	0.91 (0.61, 1.34)	69/5493	38/2745	13.64
NCT01989754	Canagliflozin	Placebo	1.17 (0.72, 1.92)	34/2904	29/2903	8.69
NCT02033889	Ertugliflozin	Placebo/Glimepiride	0.17 (0.01, 4.14)	0/412	1/209	0.21
NCT02065791	Canagliflozin	Placebo	0.39 (0.18, 0.84)	9/2200	23/2197	3.57
NCT02384941	Sotagliflozin	Placebo	0.51 (0.03, 8.13)	1/525	1/268	0.28
NCT03036124	Dapagliflozin	Placebo	1.13 (0.43, 2.91)	9/2368	8/2368	2.33
NCT03036150	Dapagliflozin	Placebo	0.40 (0.13, 1.27)	4/2149	10/2149	1.57
NCT03057951	Empagliflozin	Placebo	0.69 (0.40, 1.18)	22/2996	32/2989	7.22
NCT03057977	Empagliflozin	Placebo	0.13 (0.03, 0.54)	2/1863	16/1863	0.98
NCT03242252	Sotagliflozin	Placebo	0.25 (0.02, 2.71)	1/527	2/260	0.37
NCT03315143	Sotagliflozin	Placebo	0.68 (0.35, 1.31)	15/5291	22/5286	4.91
NCT03521934	Sotagliflozin	Placebo	5.05 (0.24, 104.96)	2/605	0/611	0.23
NCT03594110	Empagliflozin	Placebo	0.50 (0.20, 1.24)	7/3304	14/3305	2.57
NCT03619213	Dapagliflozin	Placebo	0.64 (0.28, 1.48)	9/3126	14/3127	3.02
NCT04157751	Empagliflozin	Placebo	5.08 (0.24, 105.24)	2/260	0/264	0.23
Overall (I-squared = 39.3%, p = 0.031)			0.84 (0.73, 0.97)	398/52324	370/44435	100.00

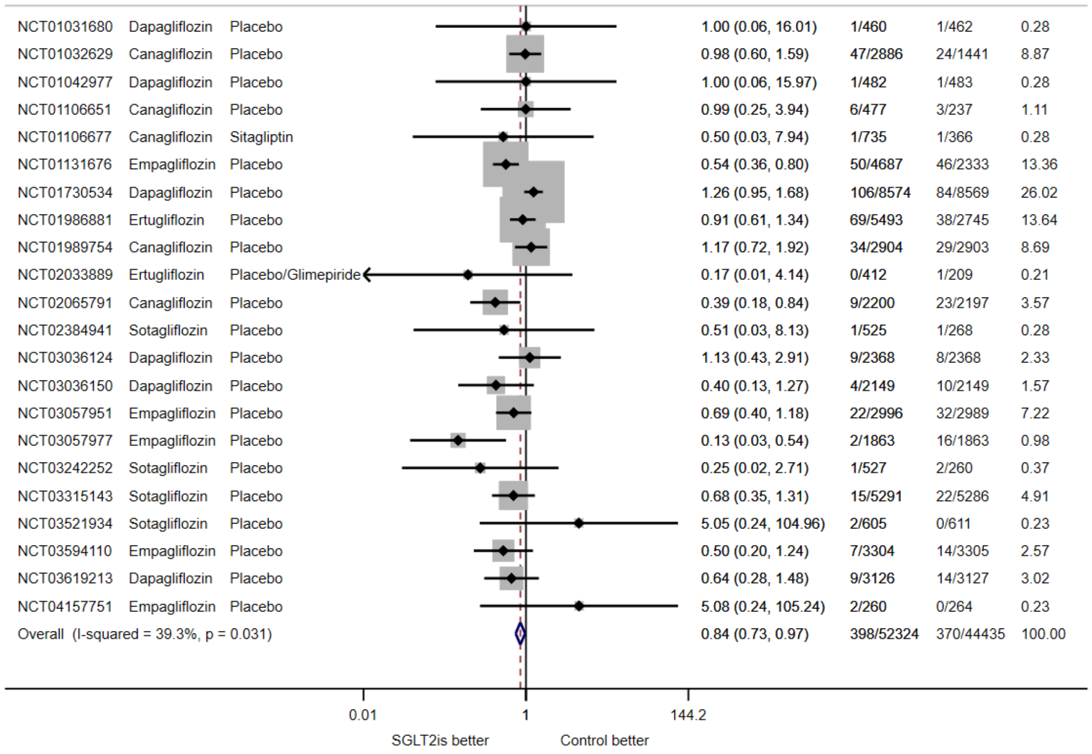

System 2-Cardiac Disorders-Figure S41 Meta-analysis of SGLT2is and Coronary artery disease

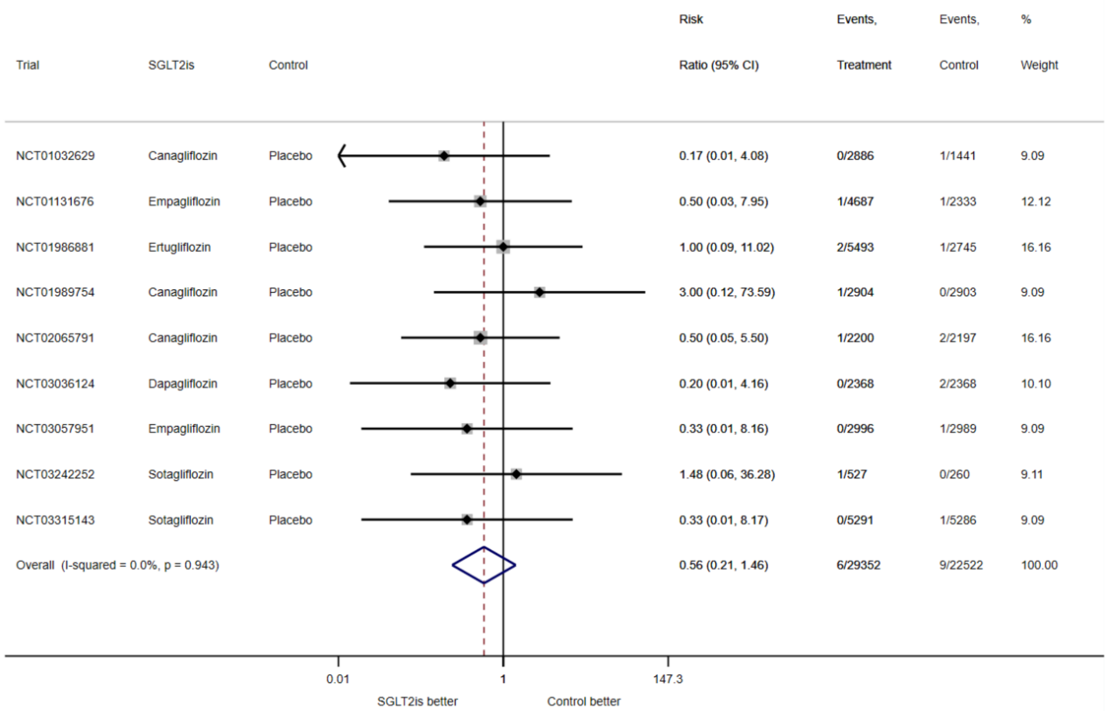

System 2-Cardiac Disorders-Figure S42 Meta-analysis of SGLT2is and Coronary artery insufficiency

Trial	SGLT2is	Control	Risk Ratio (95% CI)	Events, Treatment	Events, Control	% Weight
NCT01032629	Canagliflozin	Placebo	0.50 (0.07, 3.54)	2/2886	2/1441	5.57
NCT01131676	Empagliflozin	Placebo	0.83 (0.30, 2.28)	10/4687	6/2333	20.91
NCT01164501	Empagliflozin	Placebo	2.29 (0.09, 55.92)	1/419	0/319	2.09
NCT01730534	Dapagliflozin	Placebo	1.80 (0.60, 5.37)	9/8574	5/8569	17.90
NCT01986881	Ertugliflozin	Placebo	0.70 (0.22, 2.20)	7/5493	5/2745	16.25
NCT01989754	Canagliflozin	Placebo	1.33 (0.30, 5.95)	4/2904	3/2903	9.55
NCT02065791	Canagliflozin	Placebo	6.99 (0.36, 135.25)	3/2200	0/2197	2.43
NCT03057951	Empagliflozin	Placebo	1.00 (0.20, 4.94)	3/2996	3/2989	8.35
NCT03057977	Empagliflozin	Placebo	1.00 (0.20, 4.95)	3/1863	3/1863	8.36
NCT03315143	Sotagliflozin	Placebo	3.00 (0.12, 73.56)	1/5291	0/5286	2.09
NCT03521934	Sotagliflozin	Placebo	3.03 (0.12, 74.22)	1/605	0/611	2.09
NCT03619213	Dapagliflozin	Placebo	3.00 (0.12, 73.64)	1/3126	0/3127	2.09
NCT04157751	Empagliflozin	Placebo	0.20 (0.01, 4.21)	0/260	2/264	2.33
Overall (I-squared = 0.0%, p = 0.893)			1.10 (0.69, 1.74)	45/41304	29/34647	100.00

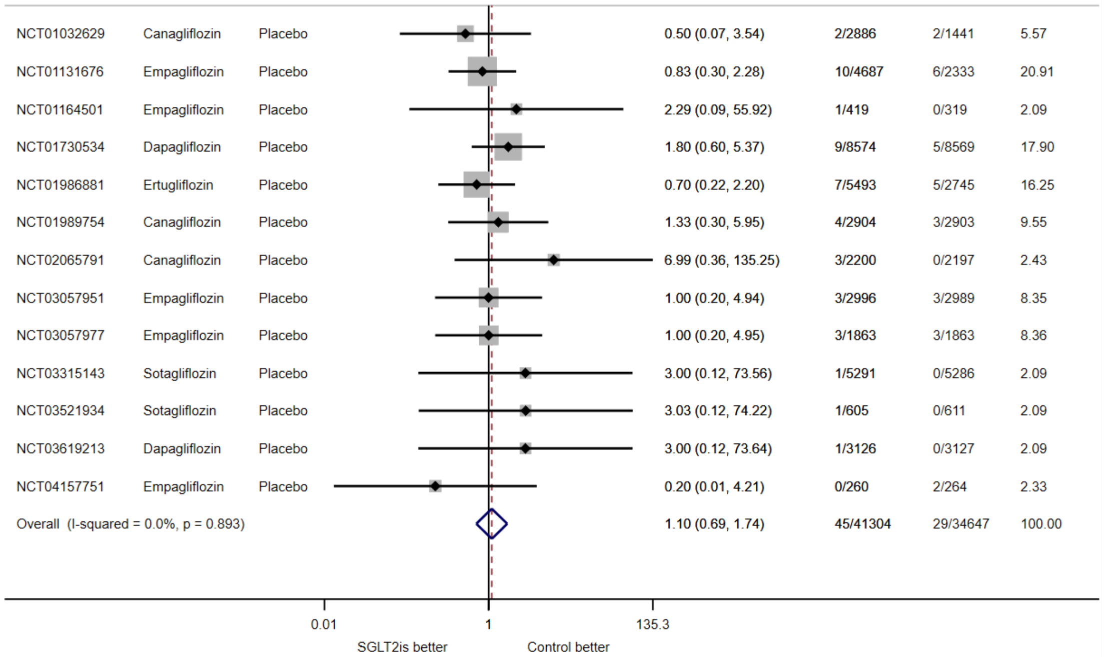

System 2-Cardiac Disorders-Figure S43 Meta-analysis of SGLT2is and Coronary artery occlusion

Trial	SGLT2is	Control	Risk Ratio (95% CI)	Events, Treatment	Events, Control	% Weight
NCT00528879	Dapagliflozin	Placebo	0.11 (0.00, 2.74)	0/409	1/137	0.89
NCT01032629	Canagliflozin	Placebo	1.20 (0.42, 3.39)	12/2886	5/1441	8.38
NCT01131676	Empagliflozin	Placebo	1.00 (0.34, 2.91)	10/4687	5/2333	7.91
NCT01730534	Dapagliflozin	Placebo	0.55 (0.27, 1.10)	12/8574	22/8569	18.41
NCT01986881	Ertugliflozin	Placebo	1.06 (0.59, 1.92)	34/5493	16/2745	25.90
NCT01989754	Canagliflozin	Placebo	0.62 (0.20, 1.91)	5/2904	8/2903	7.30
NCT02065791	Canagliflozin	Placebo	0.60 (0.14, 2.50)	3/2200	5/2197	4.45
NCT03036124	Dapagliflozin	Placebo	0.33 (0.03, 3.20)	1/2368	3/2368	1.78
NCT03036150	Dapagliflozin	Placebo	0.33 (0.03, 3.20)	1/2149	3/2149	1.78
NCT03057951	Empagliflozin	Placebo	0.50 (0.15, 1.65)	4/2996	8/2989	6.32
NCT03057977	Empagliflozin	Placebo	1.50 (0.25, 8.97)	3/1863	2/1863	2.84
NCT03242252	Sotagliflozin	Placebo	0.16 (0.01, 4.03)	0/527	1/260	0.89
NCT03315143	Sotagliflozin	Placebo	1.25 (0.34, 4.65)	5/5291	4/5286	5.26
NCT03594110	Empagliflozin	Placebo	0.75 (0.17, 3.35)	3/3304	4/3305	4.06
NCT03619213	Dapagliflozin	Placebo	1.50 (0.25, 8.97)	3/3126	2/3127	2.84
NCT04157751	Empagliflozin	Placebo	5.08 (0.24, 105.24)	2/260	0/264	0.99
Overall (I-squared = 0.0%, p = 0.816)			0.80 (0.59, 1.09)	98/49037	89/41936	100.00

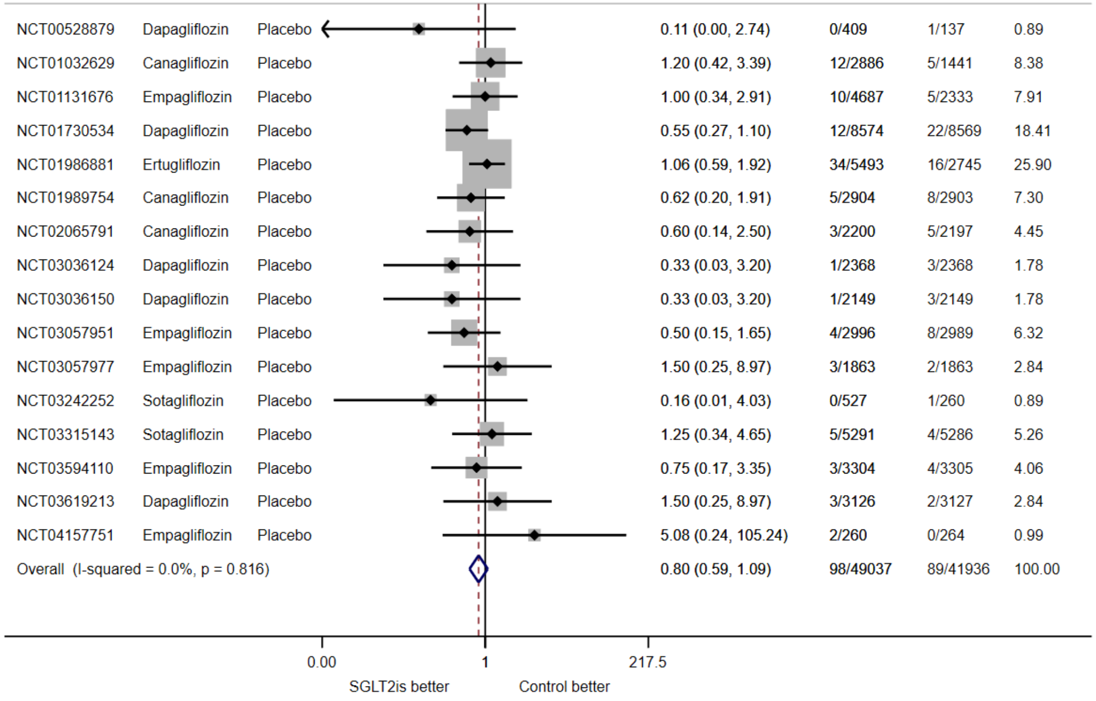

System 2-Cardiac Disorders-Figure S44 Meta-analysis of SGLT2is and Coronary artery stenosis

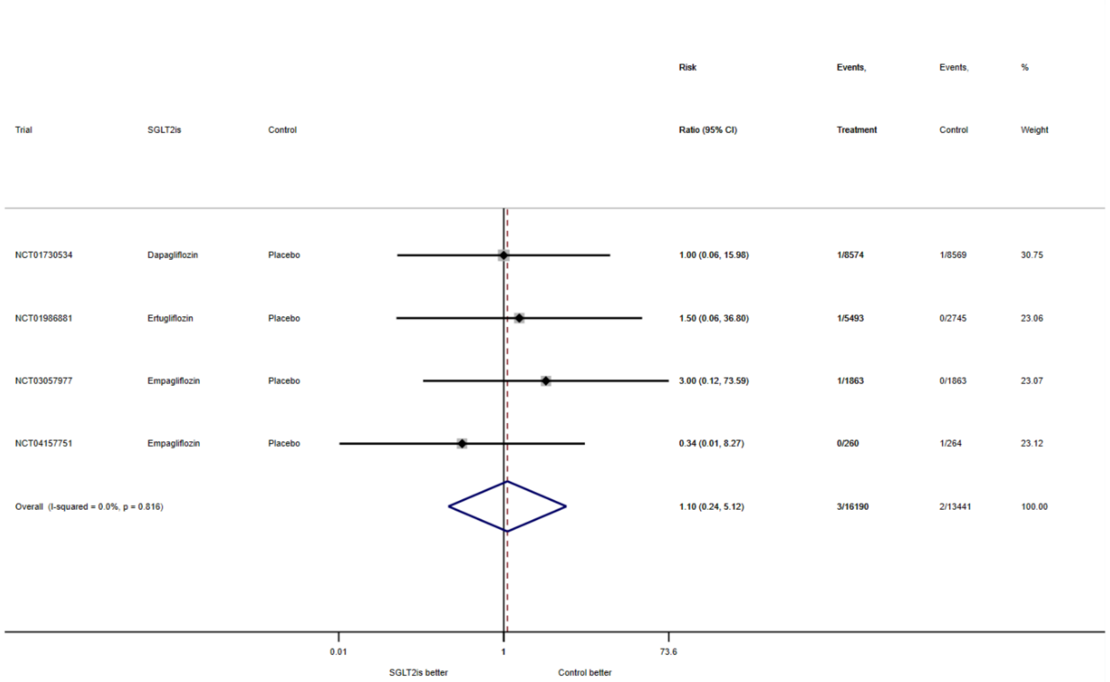

System 2-Cardiac Disorders-Figure S45 Meta-analysis of SGLT2is and Coronary ostial stenosis

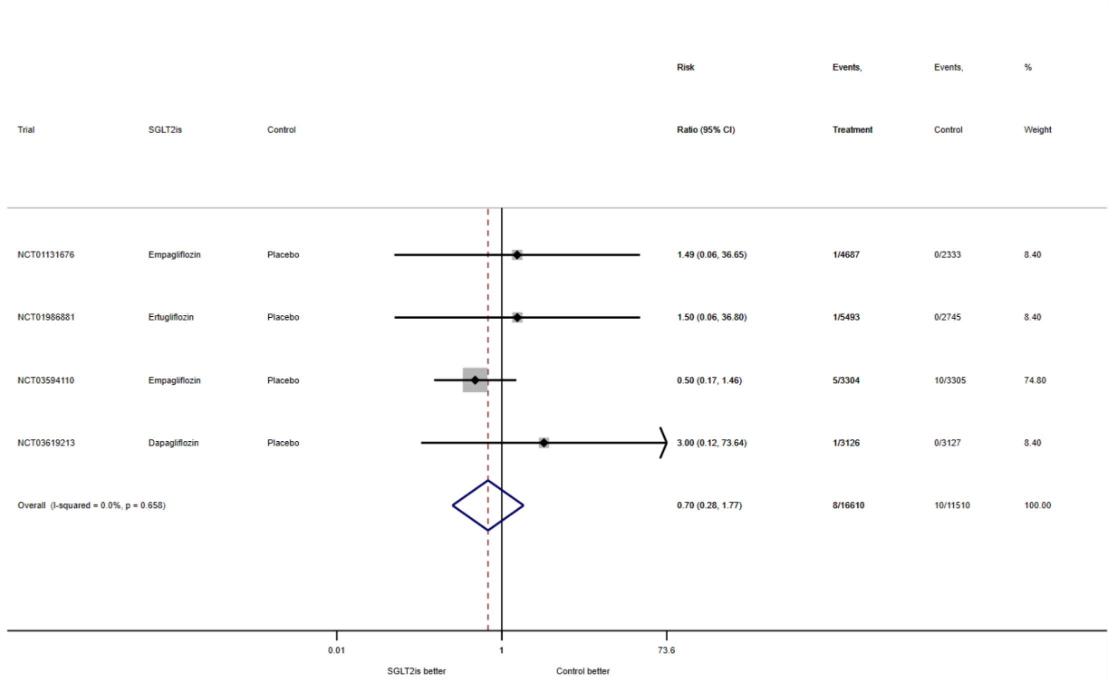

System 2-Cardiac Disorders-Figure S46 Meta-analysis of SGLT2is and Hypertensive cardiomyopathy

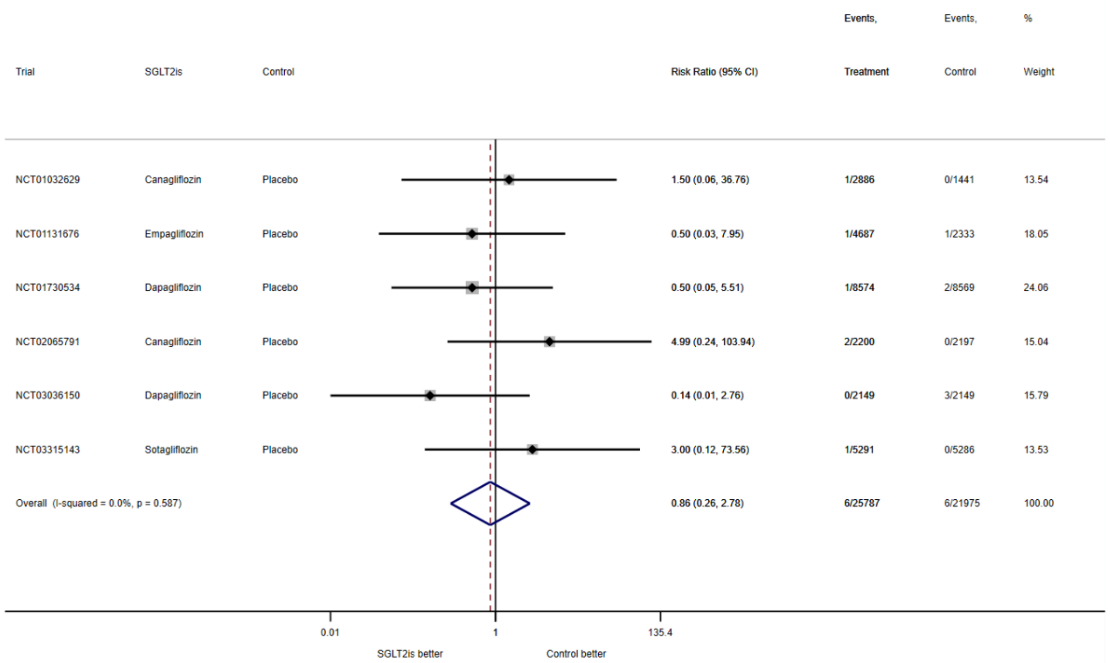

System 2-Cardiac Disorders-Figure S47 Meta-analysis of SGLT2is and Hypertensive heart disease

Trial	SGLT2is	Control	Risk Ratio (95% CI)	Events, Treatment	Events, Control	% Weight
NCT01032629	Canagliflozin	Placebo	0.50 (0.03, 7.98)	1/2886	1/1441	1.38
NCT01131676	Empagliflozin	Placebo	1.99 (0.22, 17.80)	4/4687	1/2333	2.21
NCT01730534	Dapagliflozin	Placebo	0.56 (0.26, 1.20)	10/8574	18/8569	17.78
NCT01986881	Ertugliflozin	Placebo	2.50 (0.29, 21.38)	5/5493	1/2745	2.30
NCT01989754	Canagliflozin	Placebo	0.25 (0.03, 2.23)	1/2904	4/2903	2.21
NCT02065791	Canagliflozin	Placebo	0.67 (0.19, 2.36)	4/2200	6/2197	6.64
NCT03036124	Dapagliflozin	Placebo	2.00 (0.18, 22.04)	2/2368	1/2368	1.84
NCT03036150	Dapagliflozin	Placebo	1.00 (0.25, 3.99)	4/2149	4/2149	5.53
NCT03057951	Empagliflozin	Placebo	0.33 (0.03, 3.20)	1/2996	3/2989	2.07
NCT03057977	Empagliflozin	Placebo	1.00 (0.06, 15.98)	1/1863	1/1863	1.38
NCT03315143	Sotagliflozin	Placebo	1.00 (0.06, 15.97)	1/5291	1/5286	1.38
NCT03594110	Empagliflozin	Placebo	0.73 (0.47, 1.15)	33/3304	45/3305	53.19
NCT03619213	Dapagliflozin	Placebo	0.33 (0.03, 3.20)	1/3126	3/3127	2.07
Overall (I-squared = 0.0%, p = 0.941)			0.72 (0.52, 0.99)	68/47841	89/41275	100.00

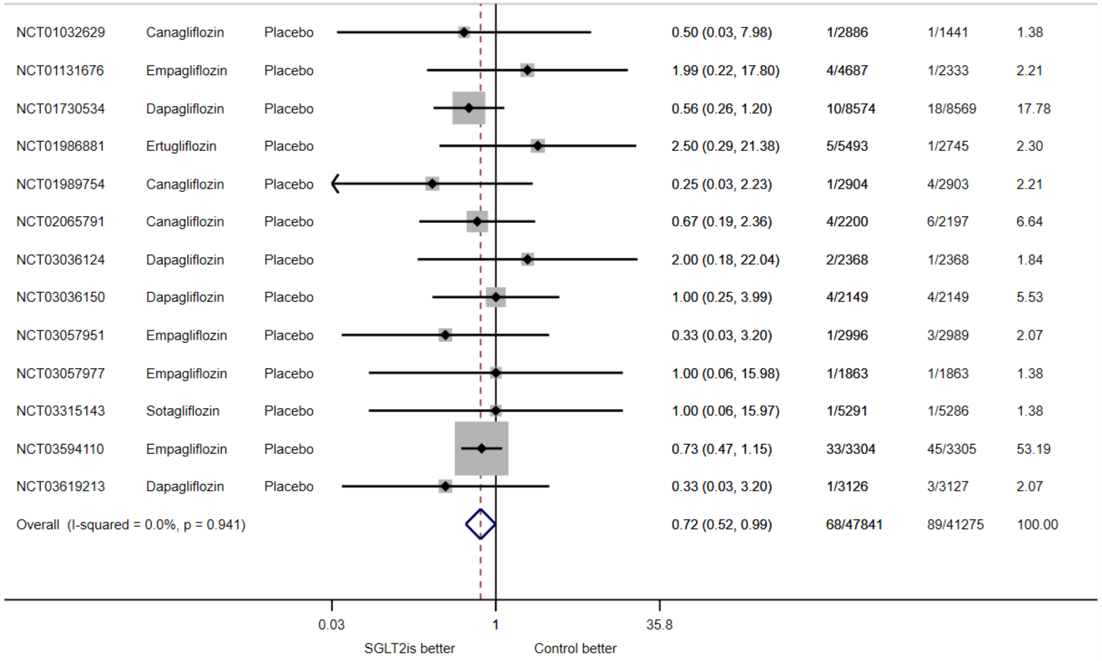

System 2-Cardiac Disorders-Figure S48 Meta-analysis of SGLT2is and Ischaemic cardiomyopathy

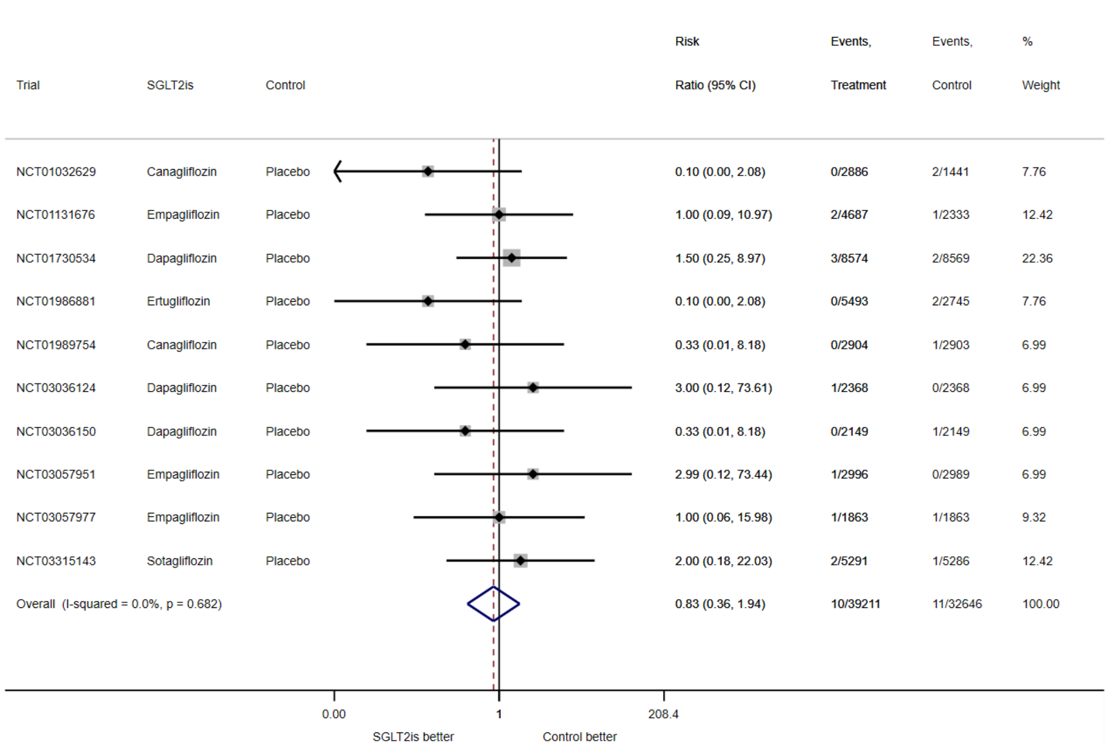

System 2-Cardiac Disorders-Figure S49 Meta-analysis of SGLT2is and Left ventricular dysfunction

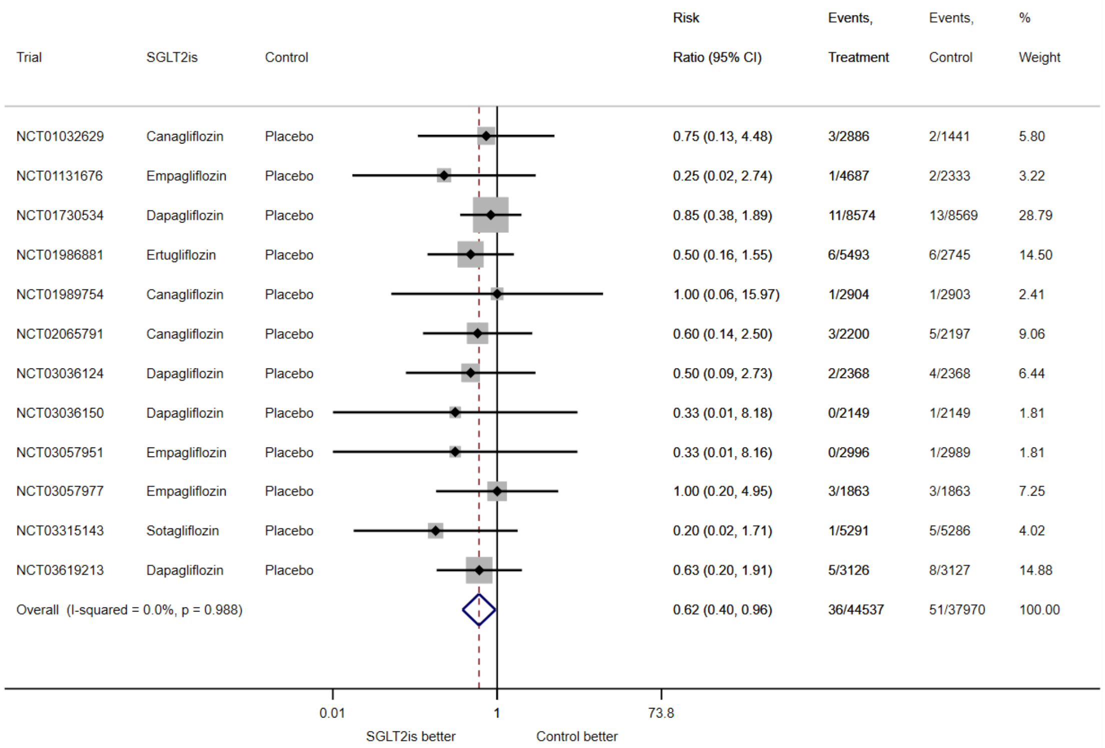

System 2-Cardiac Disorders-Figure S50 Meta-analysis of SGLT2is and Left ventricular failure

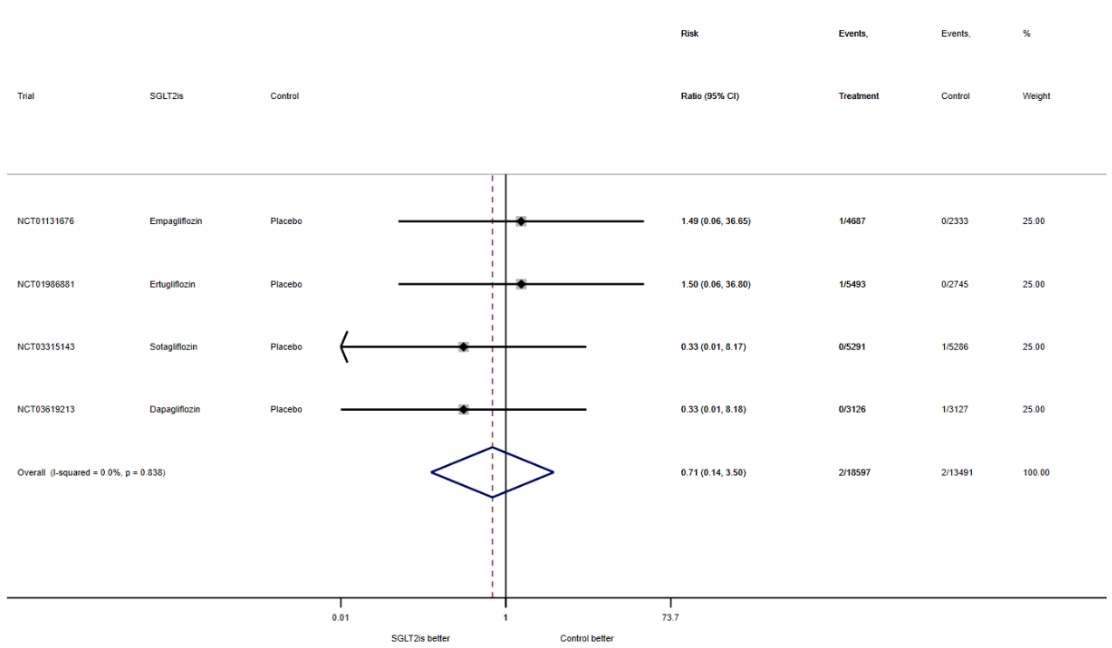

System 2-Cardiac Disorders-Figure S51 Meta-analysis of SGLT2is and Microvascular coronary artery disease

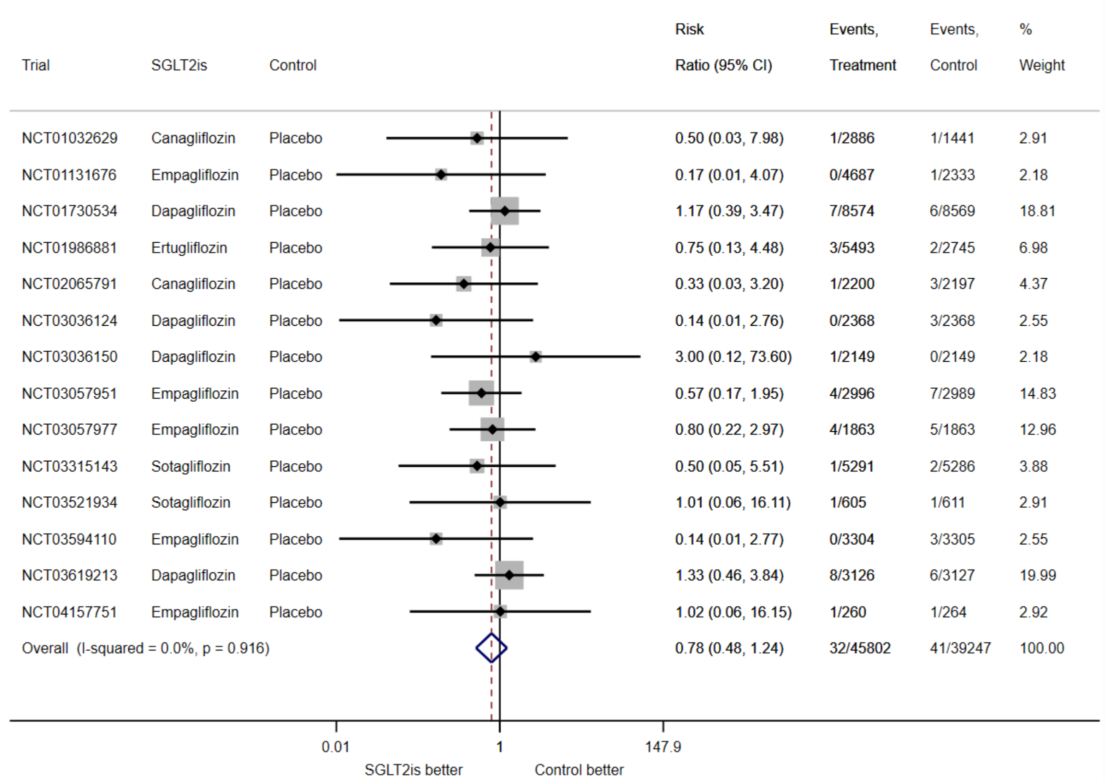

System 2-Cardiac Disorders-Figure S52 Meta-analysis of SGLT2is and Mitral valve incompetence

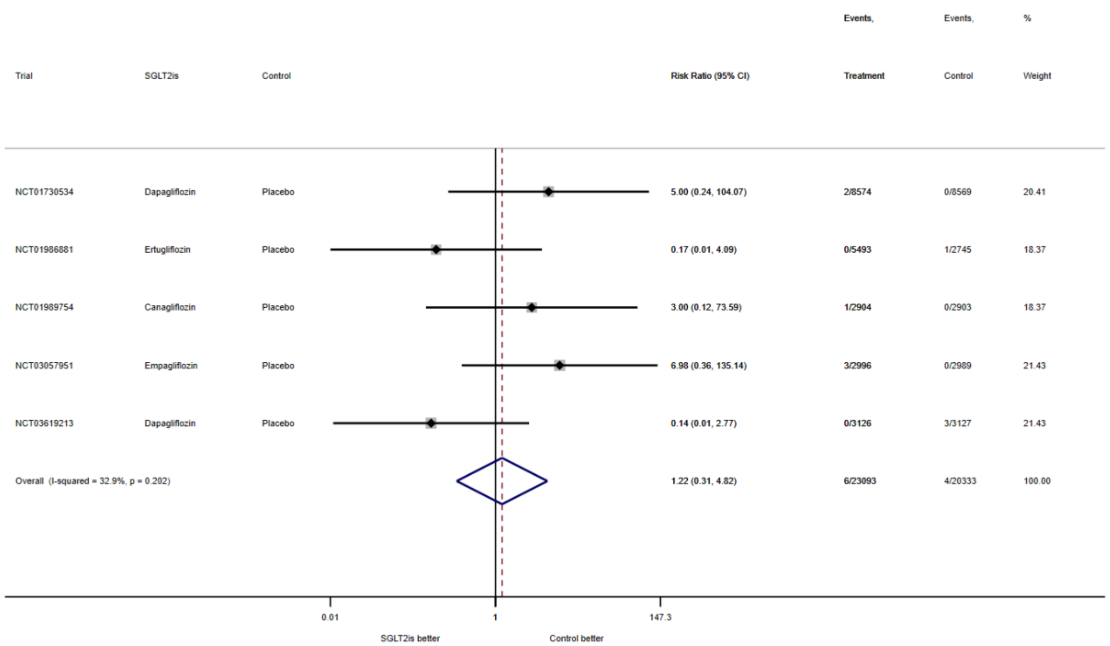

System 2-Cardiac Disorders-Figure S53 Meta-analysis of SGLT2is and Mitral valve stenosis

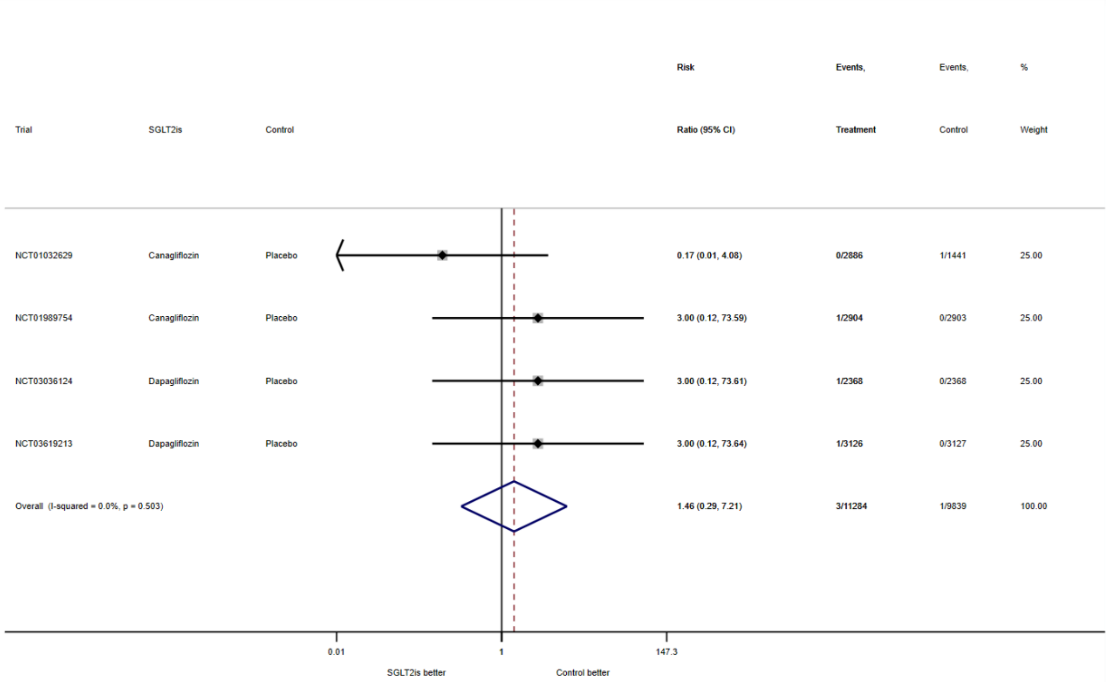

System 2-Cardiac Disorders-Figure S54 Meta-analysis of SGLT2is and Myocardial fibrosis

Trial	SGLT2is	Control	Risk Ratio (95% CI)	Events, Treatment	Events, Control	% Weight
NCT00528879	Dapagliflozin	Placebo	1.68 (0.08, 34.84)	2/409	0/137	0.18
NCT01032629	Canagliflozin	Placebo	0.90 (0.49, 1.66)	29/2886	16/1441	4.45
NCT01042977	Dapagliflozin	Placebo	3.01 (0.31, 28.80)	3/482	1/483	0.32
NCT01106651	Canagliflozin	Placebo	0.75 (0.13, 4.43)	3/477	2/237	0.52
NCT01106677	Canagliflozin	Sitagliptin	1.50 (0.06, 36.63)	1/735	0/366	0.16
NCT01131676	Empagliflozin	Placebo	1.00 (0.70, 1.41)	94/4687	47/2333	13.65
NCT01177813	Empagliflozin	Sitagliptin or Placebo	0.34 (0.01, 8.25)	0/447	1/452	0.16
NCT01730534	Dapagliflozin	Placebo	0.89 (0.68, 1.17)	96/8574	108/8569	21.96
NCT01986881	Ertugliflozin	Placebo	1.19 (0.81, 1.74)	88/5493	37/2745	11.28
NCT01989754	Canagliflozin	Placebo	1.29 (0.76, 2.19)	31/2904	24/2903	5.83
NCT02033889	Ertugliflozin	Placebo/Glimepiride	1.52 (0.16, 14.54)	3/412	1/209	0.32
NCT02065791	Canagliflozin	Placebo	0.81 (0.46, 1.42)	22/2200	27/2197	5.23
NCT02384941	Sotagliflozin	Placebo	0.17 (0.01, 4.17)	0/525	1/268	0.16
NCT03036124	Dapagliflozin	Placebo	0.94 (0.48, 1.86)	16/2368	17/2368	3.54
NCT03036150	Dapagliflozin	Placebo	1.83 (0.68, 4.95)	11/2149	6/2149	1.66
NCT03057951	Empagliflozin	Placebo	1.37 (0.83, 2.24)	37/2996	27/2989	6.73
NCT03057977	Empagliflozin	Placebo	1.00 (0.50, 1.99)	16/1863	16/1863	3.45
NCT03242252	Sotagliflozin	Placebo	1.97 (0.22, 17.57)	4/527	1/260	0.34
NCT03315143	Sotagliflozin	Placebo	0.44 (0.27, 0.70)	24/5291	55/5286	7.18
NCT03521934	Sotagliflozin	Placebo	0.67 (0.24, 1.88)	6/605	9/611	1.56
NCT03594110	Empagliflozin	Placebo	1.26 (0.79, 2.01)	39/3304	31/3305	7.45
NCT03619213	Dapagliflozin	Placebo	0.83 (0.42, 1.65)	15/3126	18/3127	3.51
NCT04157751	Empagliflozin	Placebo	0.15 (0.01, 2.79)	0/260	3/264	0.19
NCT04350593	Dapagliflozin	Placebo	0.33 (0.01, 8.21)	0/613	1/616	0.16
Overall (I-squared = 6.0%, p = 0.378)			0.96 (0.85, 1.10)	540/53333	449/45178	100.00

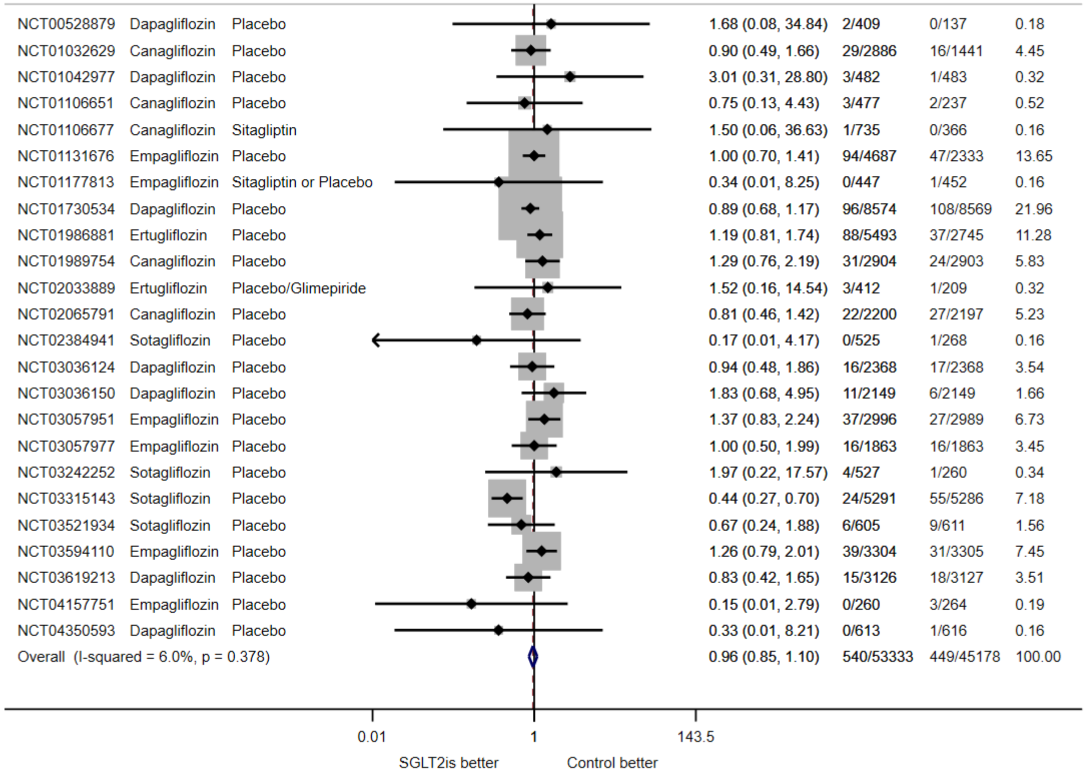

System 2-Cardiac Disorders-Figure S55 Meta-analysis of SGLT2is and Myocardial infarction

Trial	SGLT2is	Control	Risk Ratio (95% CI)	Events, Treatment	Events, Control	% Weight
NCT00528879	Dapagliflozin	Placebo	0.33 (0.02, 5.32)	1/409	1/137	0.68
NCT01032629	Canagliflozin	Placebo	0.94 (0.42, 2.11)	17/2886	9/1441	7.99
NCT01131676	Empagliflozin	Placebo	0.40 (0.19, 0.85)	12/4687	15/2333	9.03
NCT01164501	Empagliflozin	Placebo	0.25 (0.01, 6.21)	0/419	1/319	0.51
NCT01730534	Dapagliflozin	Placebo	0.87 (0.59, 1.28)	47/8574	54/8569	34.09
NCT01986881	Ertugliflozin	Placebo	0.83 (0.47, 1.49)	30/5493	18/2745	15.27
NCT01989754	Canagliflozin	Placebo	0.78 (0.29, 2.08)	7/2904	9/2903	5.33
NCT02033889	Ertugliflozin	Placebo/Glimepiride	1.53 (0.06, 37.28)	1/412	0/209	0.51
NCT02065791	Canagliflozin	Placebo	1.00 (0.38, 2.66)	8/2200	8/2197	5.41
NCT03036124	Dapagliflozin	Placebo	0.75 (0.17, 3.35)	3/2368	4/2368	2.32
NCT03036150	Dapagliflozin	Placebo	1.00 (0.32, 3.10)	6/2149	6/2149	4.06
NCT03057951	Empagliflozin	Placebo	0.89 (0.34, 2.30)	8/2996	9/2989	5.73
NCT03057977	Empagliflozin	Placebo	3.00 (0.31, 28.81)	3/1863	1/1863	1.01
NCT03242252	Sotagliflozin	Placebo	1.48 (0.06, 36.28)	1/527	0/260	0.51
NCT03315143	Sotagliflozin	Placebo	0.86 (0.29, 2.55)	6/5291	7/5286	4.36
NCT03521934	Sotagliflozin	Placebo	2.02 (0.18, 22.22)	2/605	1/611	0.90
NCT03619213	Dapagliflozin	Placebo	0.50 (0.09, 2.73)	2/3126	4/3127	1.80
NCT04157751	Empagliflozin	Placebo	3.05 (0.12, 74.43)	1/260	0/264	0.51
Overall (I-squared = 0.0%, p = 0.966)			0.82 (0.65, 1.03)	155/47169	147/39770	100.00

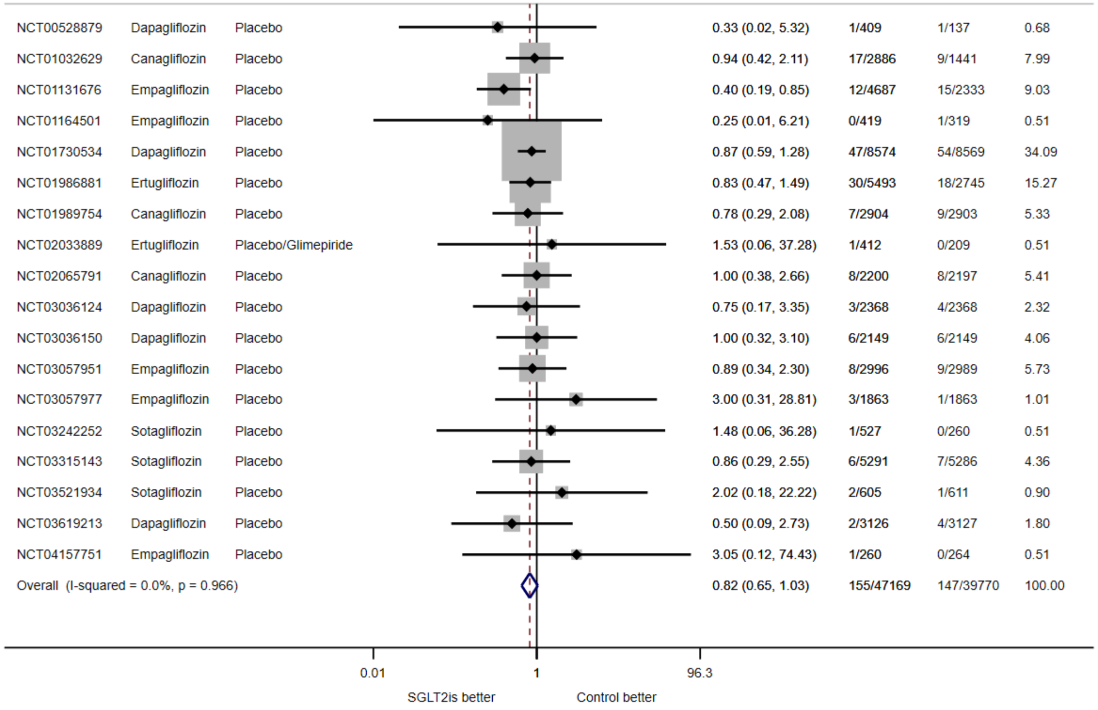

System 2-Cardiac Disorders-Figure S56 Meta-analysis of SGLT2is and Myocardial ischaemia

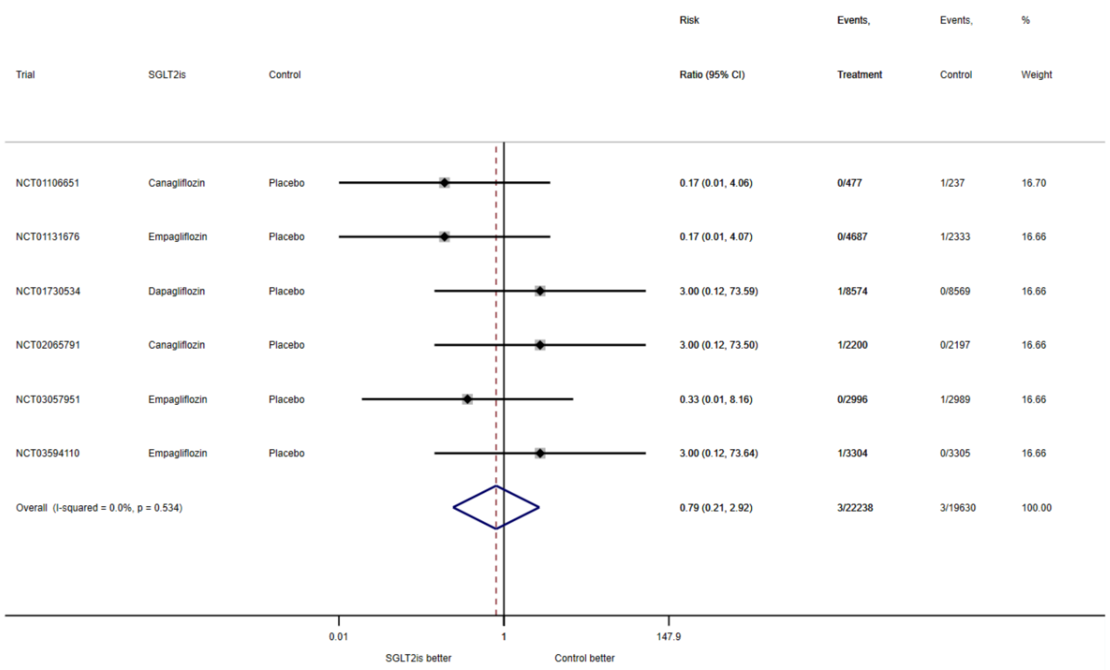

System 2-Cardiac Disorders-Figure S57 Meta-analysis of SGLT2is and Myocarditis

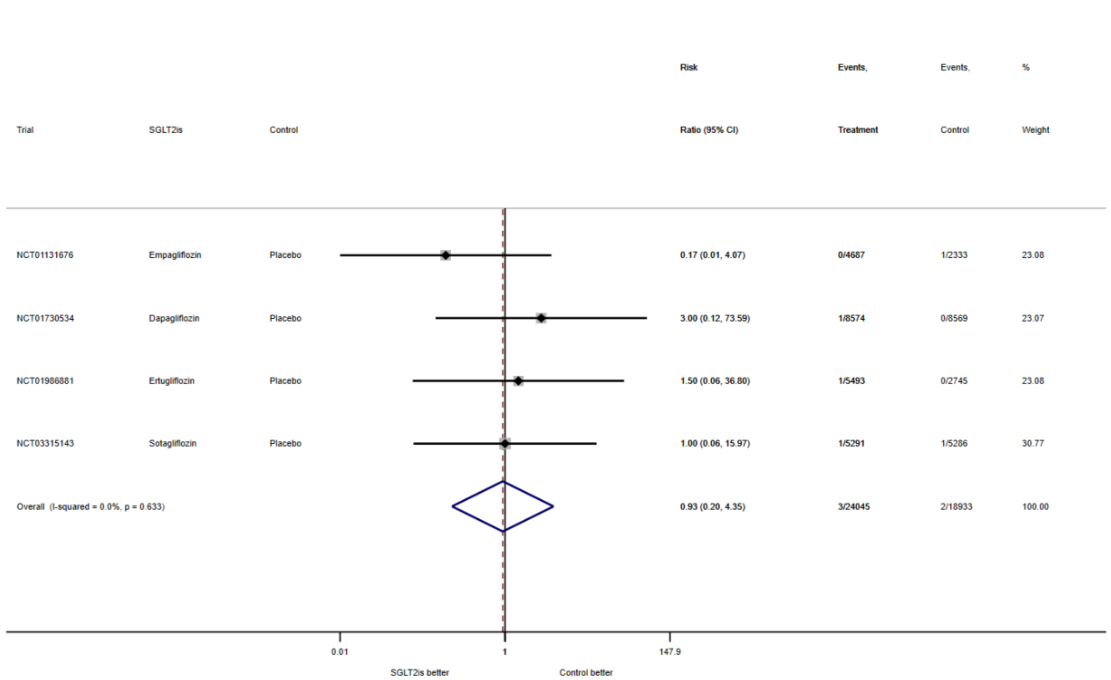

System 2-Cardiac Disorders-Figure S58 Meta-analysis of SGLT2is and Nodal arrhythmia

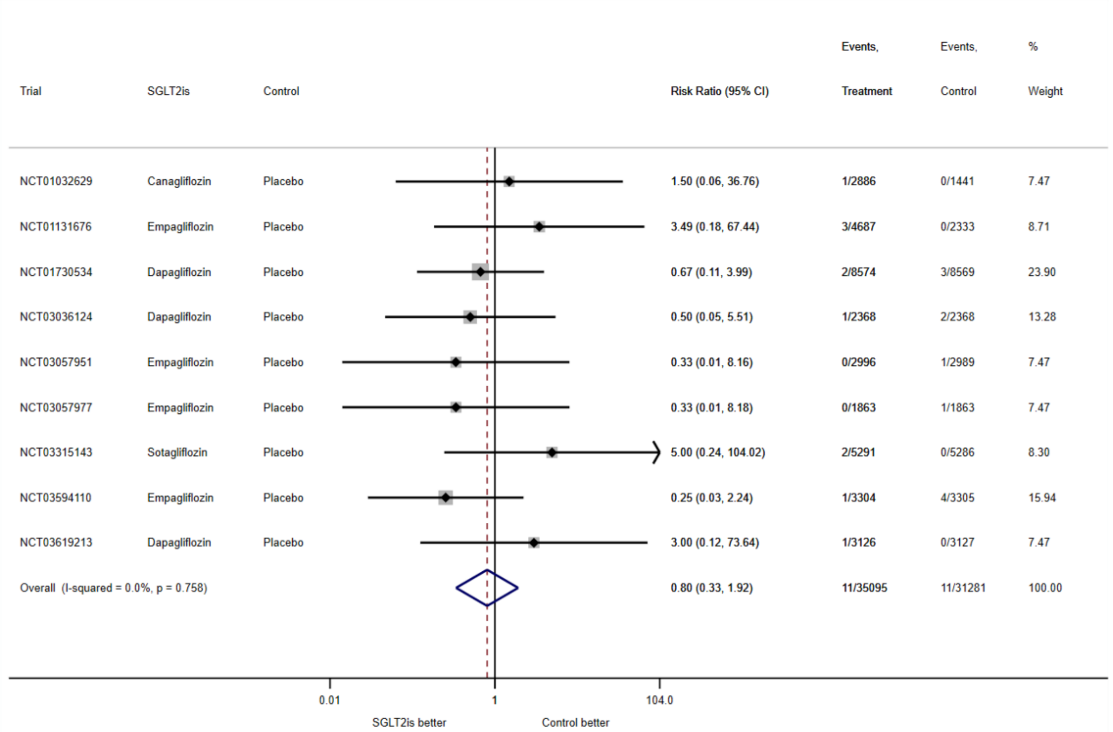

System 2-Cardiac Disorders-Figure S59 Meta-analysis of SGLT2is and Palpitations

Trial	SGLT2is	Control	Risk Ratio (95% CI)	Events, Treatment	Events, Control	% Weight
NCT01032629	Canagliflozin	Placebo	1.50 (0.06, 36.76)	1/2886	0/1441	5.08
NCT01131676	Empagliflozin	Placebo	0.50 (0.07, 3.53)	2/4687	2/2333	13.56
NCT01730534	Dapagliflozin	Placebo	0.33 (0.03, 3.20)	1/8574	3/8569	10.17
NCT01986881	Ertugliflozin	Placebo	1.50 (0.06, 36.80)	1/5493	0/2745	5.08
NCT01989754	Canagliflozin	Placebo	5.00 (0.24, 104.07)	2/2904	0/2903	5.65
NCT02065791	Canagliflozin	Placebo	1.00 (0.14, 7.08)	2/2200	2/2197	13.56
NCT03036124	Dapagliflozin	Placebo	1.00 (0.06, 15.98)	1/2368	1/2368	6.78
NCT03036150	Dapagliflozin	Placebo	0.20 (0.01, 4.16)	0/2149	2/2149	5.65
NCT03057951	Empagliflozin	Placebo	0.50 (0.05, 5.50)	1/2996	2/2989	9.04
NCT03057977	Empagliflozin	Placebo	3.00 (0.12, 73.59)	1/1863	0/1863	5.08
NCT03521934	Sotagliflozin	Placebo	0.20 (0.01, 4.20)	0/605	2/611	5.65
NCT03594110	Empagliflozin	Placebo	2.00 (0.18, 22.05)	2/3304	1/3305	9.04
NCT03619213	Dapagliflozin	Placebo	0.20 (0.01, 4.17)	0/3126	2/3127	5.65
Overall (I-squared = 0.0%, p = 0.901)			0.75 (0.36, 1.54)	14/43155	17/36600	100.00

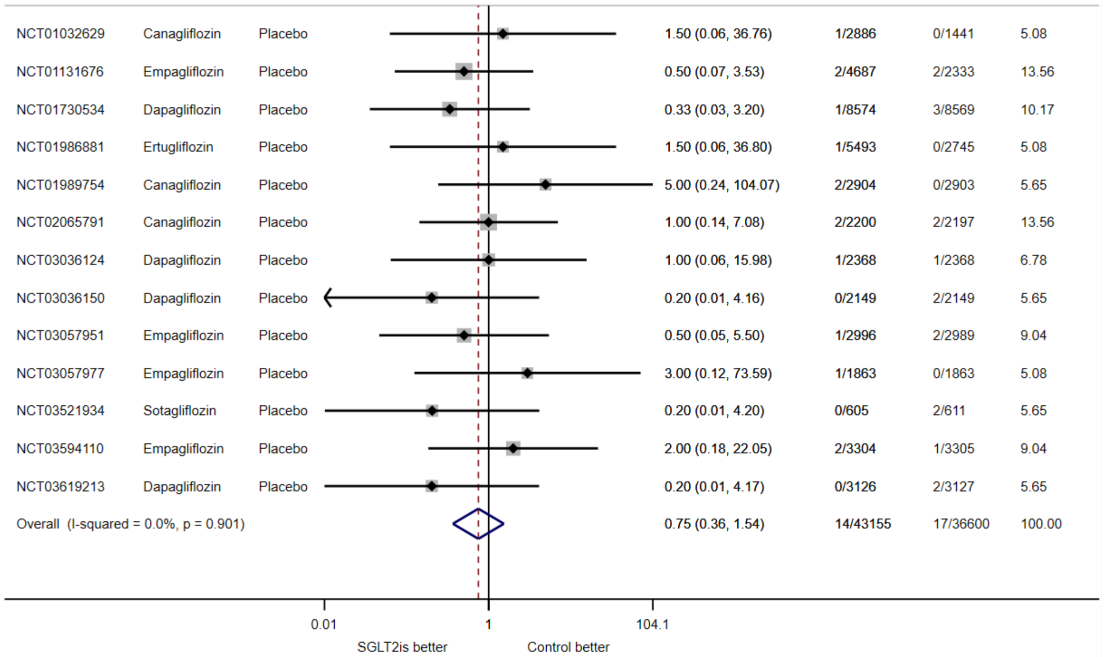

System 2-Cardiac Disorders-Figure S60 Meta-analysis of SGLT2is and Pericardial effusion

Trial	SGLT2is	Control	Risk Ratio (95% CI)	Events, Treatment	Events, Control	% Weight
NCT01131676	Empagliflozin	Placebo	1.49 (0.06, 36.65)	1/4687	0/2333	5.13
NCT01730534	Dapagliflozin	Placebo	2.00 (0.60, 6.64)	8/8574	4/8569	36.51
NCT01986881	Ertugliflozin	Placebo	2.50 (0.12, 52.04)	2/5493	0/2745	5.70
NCT01989754	Canagliflozin	Placebo	0.14 (0.01, 2.76)	0/2904	3/2903	5.99
NCT02065791	Canagliflozin	Placebo	1.00 (0.06, 15.96)	1/2200	1/2197	6.84
NCT03036124	Dapagliflozin	Placebo	1.00 (0.06, 15.98)	1/2368	1/2368	6.84
NCT03036150	Dapagliflozin	Placebo	3.00 (0.12, 73.60)	1/2149	0/2149	5.13
NCT03242252	Sotagliflozin	Placebo	1.48 (0.06, 36.28)	1/527	0/260	5.14
NCT03315143	Sotagliflozin	Placebo	8.99 (0.48, 166.96)	4/5291	0/5286	6.16
NCT03521934	Sotagliflozin	Placebo	5.05 (0.24, 104.96)	2/605	0/611	5.71
NCT03594110	Empagliflozin	Placebo	0.20 (0.01, 4.17)	0/3304	2/3305	5.70
NCT03619213	Dapagliflozin	Placebo	3.00 (0.12, 73.64)	1/3126	0/3127	5.13
Overall (I-squared = 0.0%, p = 0.800)			1.61 (0.78, 3.33)	22/41228	11/35853	100.00

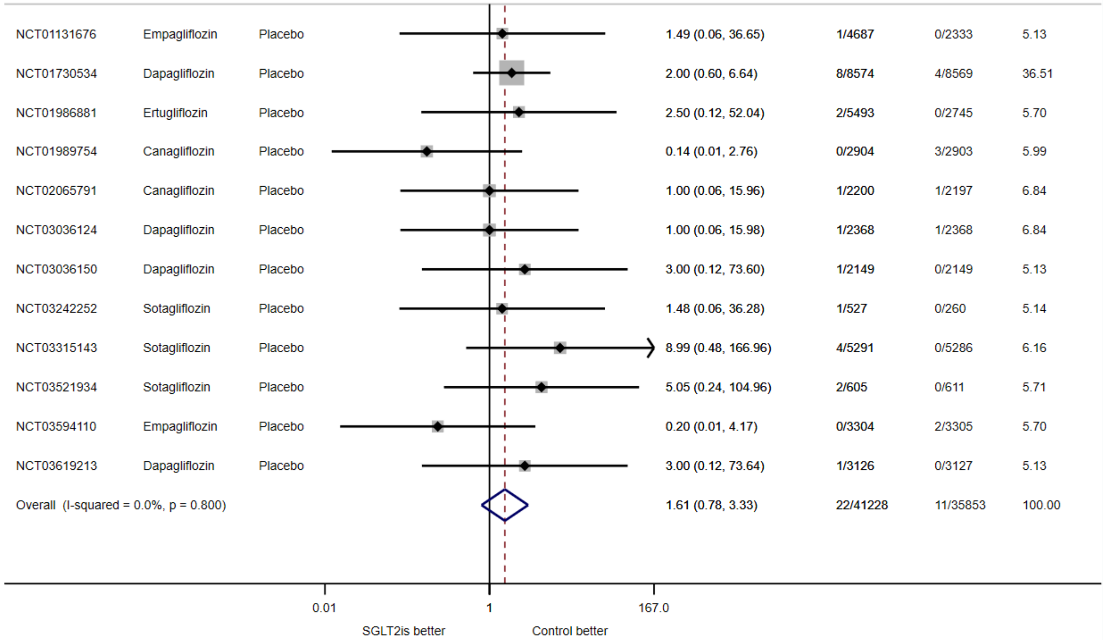

System 2-Cardiac Disorders-Figure S61 Meta-analysis of SGLT2is and Pericarditis

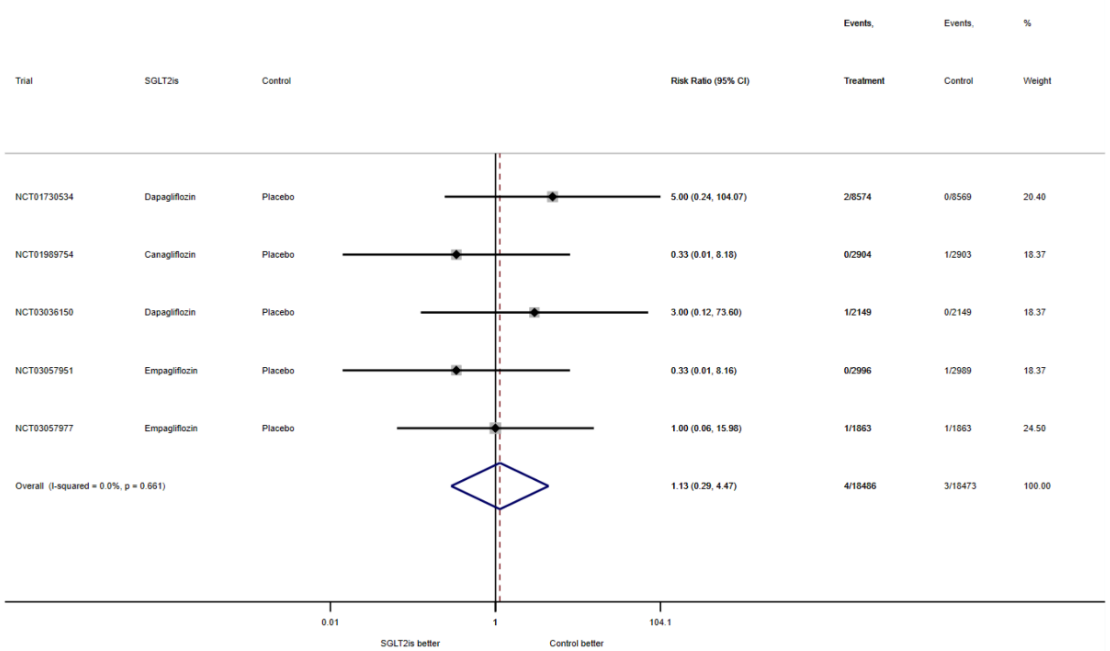

System 2-Cardiac Disorders-Figure S62 Meta-analysis of SGLT2is and Prinzmetal angina

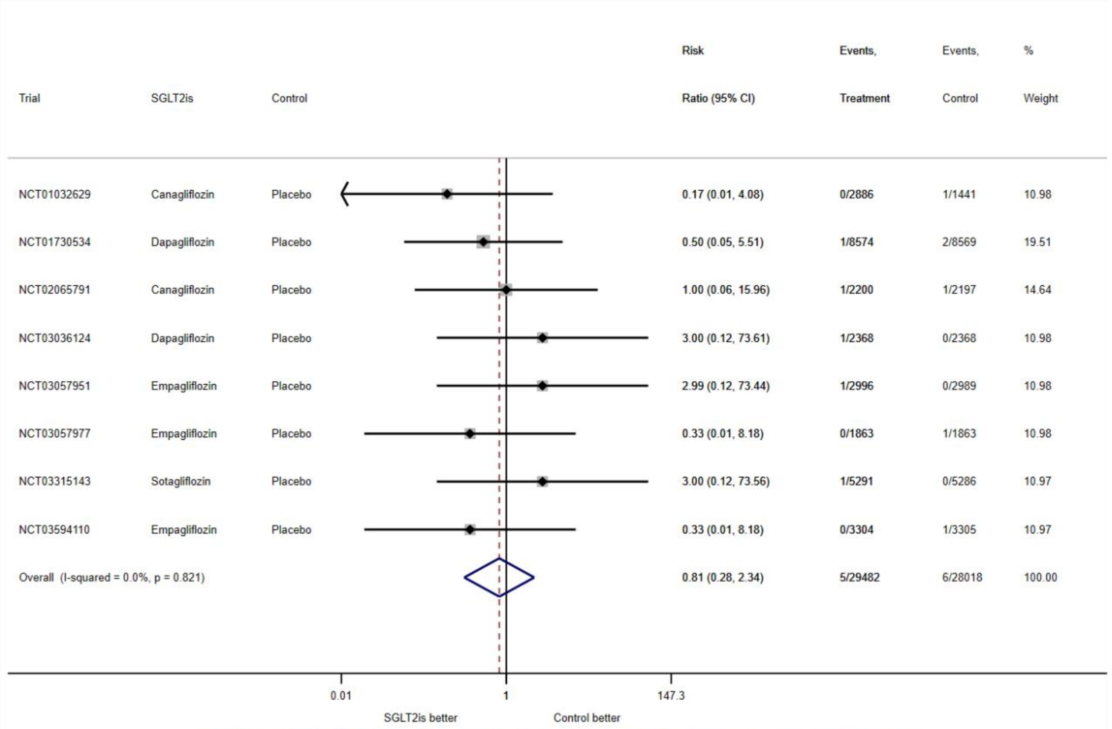

System 2-Cardiac Disorders-Figure S63 Meta-analysis of SGLT2is and Pulseless electrical activity

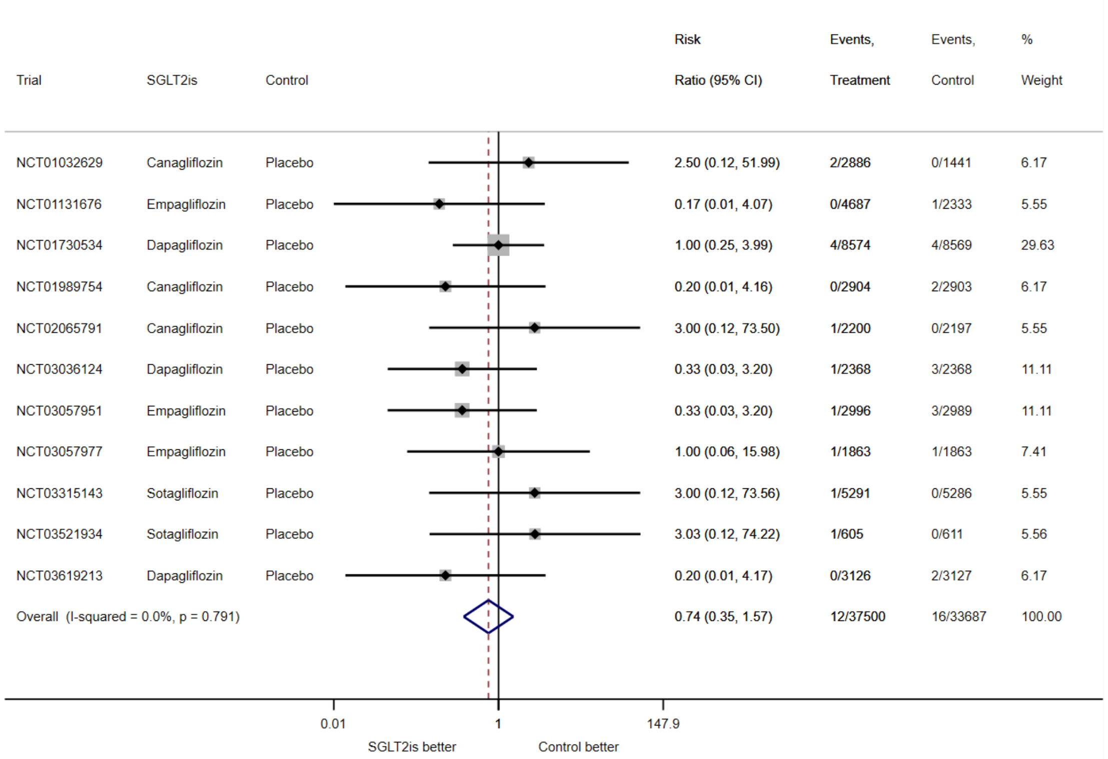

System 2-Cardiac Disorders-Figure S64 Meta-analysis of SGLT2is and Right ventricular failure

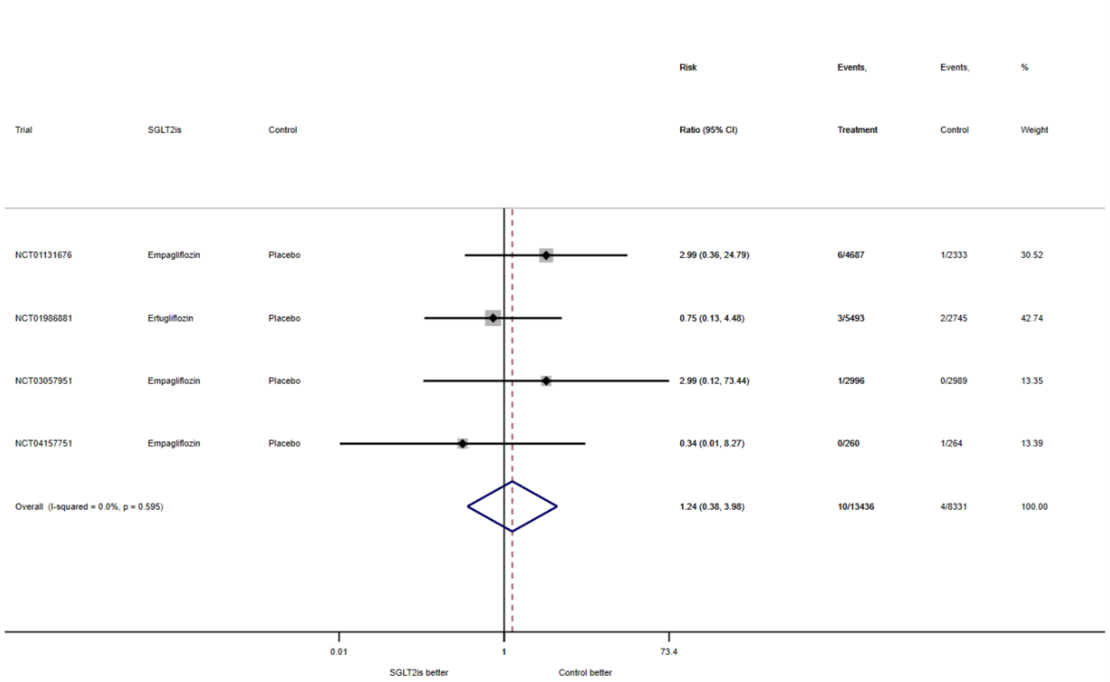

System 2-Cardiac Disorders-Figure S65 Meta-analysis of SGLT2is and Silent myocardial infarction

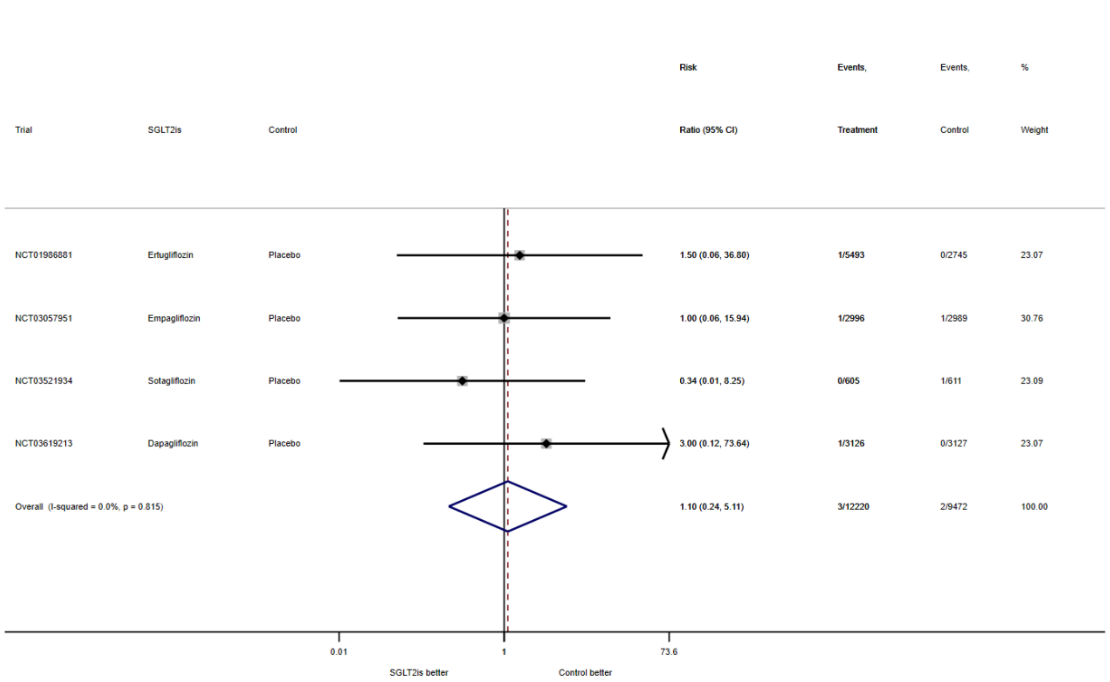

System 2-Cardiac Disorders-Figure S66 Meta-analysis of SGLT2is and Sinoatrial block

Trial	SGLT2is	Control	Risk Ratio (95% CI)	Events, Treatment	Events, Control	% Weight
NCT01032629	Canagliflozin	Placebo	1.50 (0.06, 36.76)	1/2886	0/1441	5.76
NCT01131676	Empagliflozin	Placebo	1.49 (0.16, 14.35)	3/4687	1/2333	11.52
NCT01730534	Dapagliflozin	Placebo	0.40 (0.08, 2.06)	2/8574	5/8569	21.94
NCT01986881	Ertugliflozin	Placebo	2.50 (0.12, 52.04)	2/5493	0/2745	6.40
NCT01989754	Canagliflozin	Placebo	0.33 (0.01, 8.18)	0/2904	1/2903	5.76
NCT03036150	Dapagliflozin	Placebo	0.33 (0.01, 8.18)	0/2149	1/2149	5.76
NCT03057951	Empagliflozin	Placebo	6.98 (0.86, 56.73)	7/2996	1/2989	13.44
NCT03315143	Sotagliflozin	Placebo	2.00 (0.18, 22.03)	2/5291	1/5286	10.24
NCT03594110	Empagliflozin	Placebo	0.33 (0.03, 3.20)	1/3304	3/3305	11.52
NCT03619213	Dapagliflozin	Placebo	1.00 (0.06, 15.99)	1/3126	1/3127	7.68
Overall (I-squared = 0.0%, p = 0.619)			1.01 (0.47, 2.17)	19/41410	14/34847	100.00

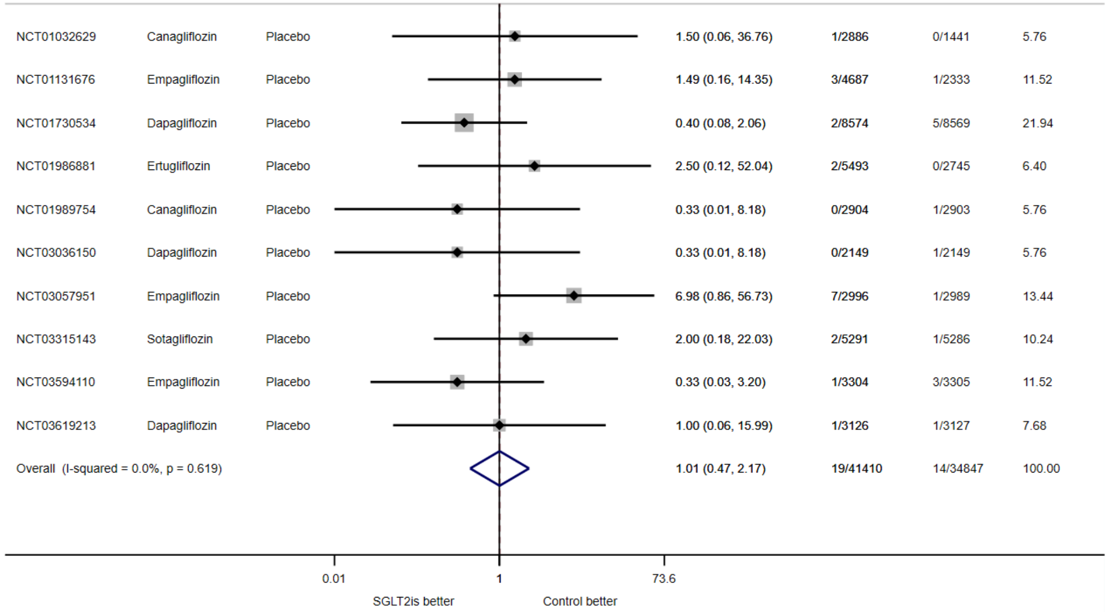

System 2-Cardiac Disorders-Figure S67 Meta-analysis of SGLT2is and Sinus bradycardia

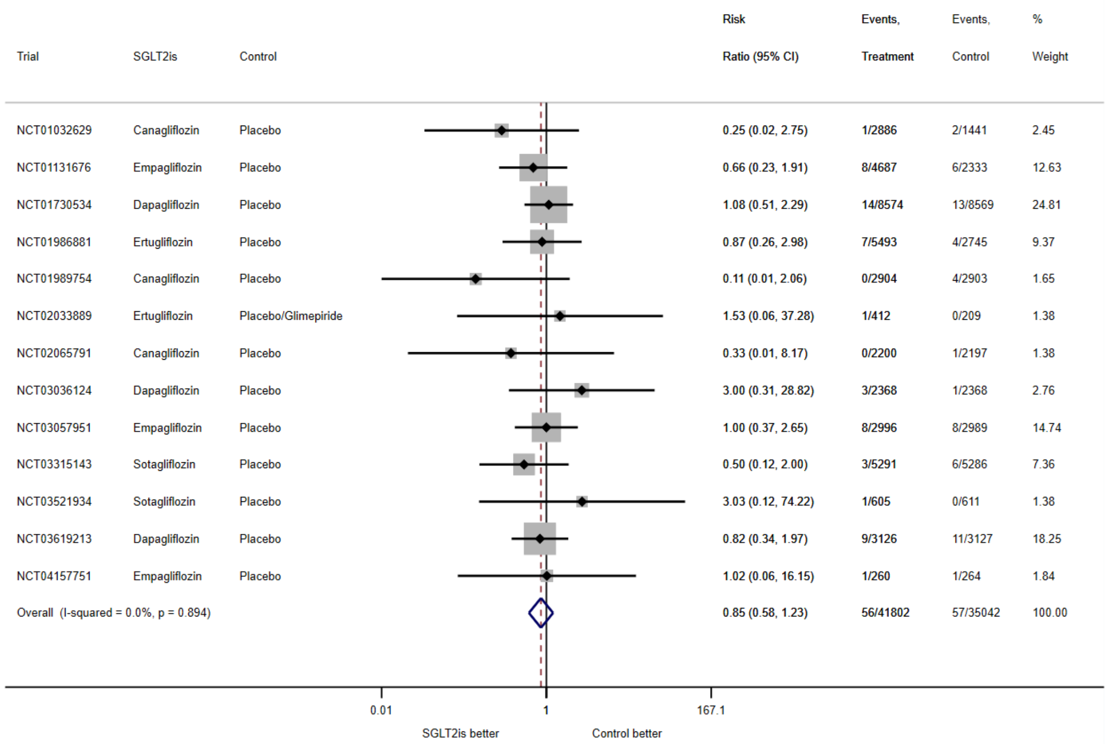

System 2-Cardiac Disorders-Figure S68 Meta-analysis of SGLT2is and Sinus node dysfunction

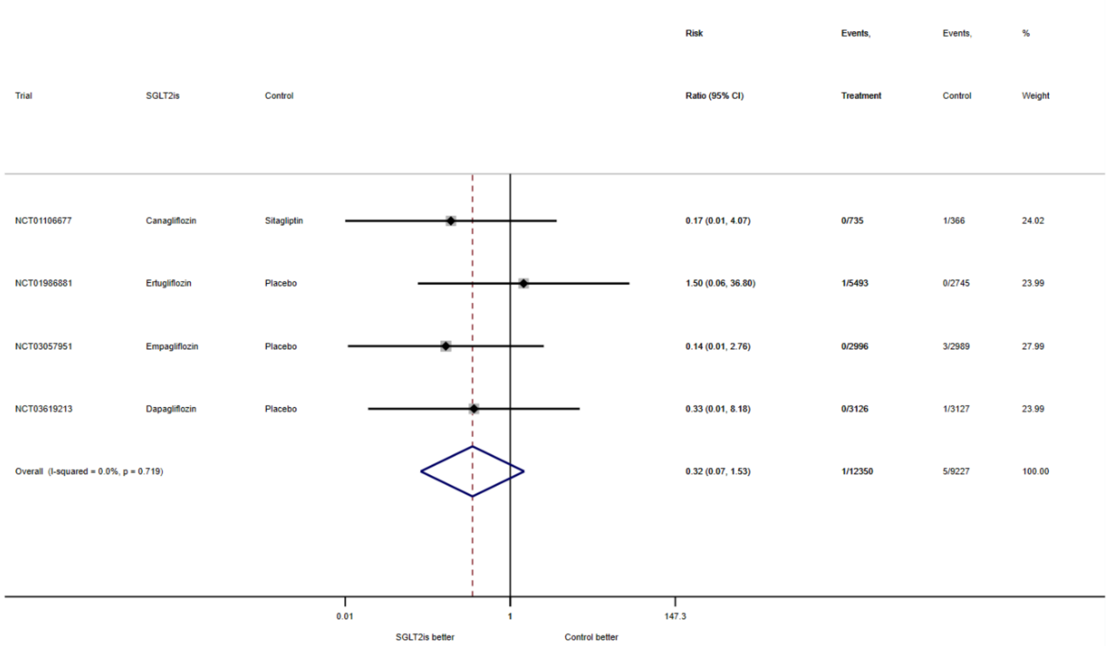

System 2-Cardiac Disorders-Figure S69 Meta-analysis of SGLT2is and Stress cardiomyopathy

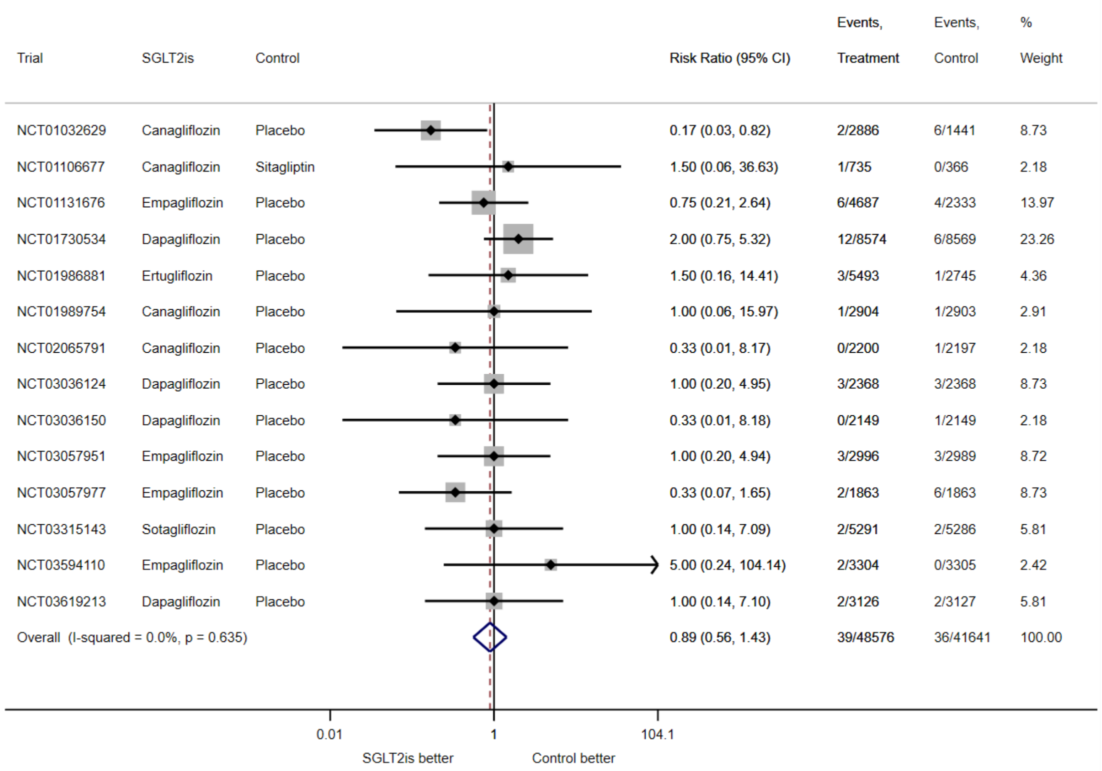

System 2-Cardiac Disorders-Figure S70 Meta-analysis of SGLT2is and Supraventricular tachycardia

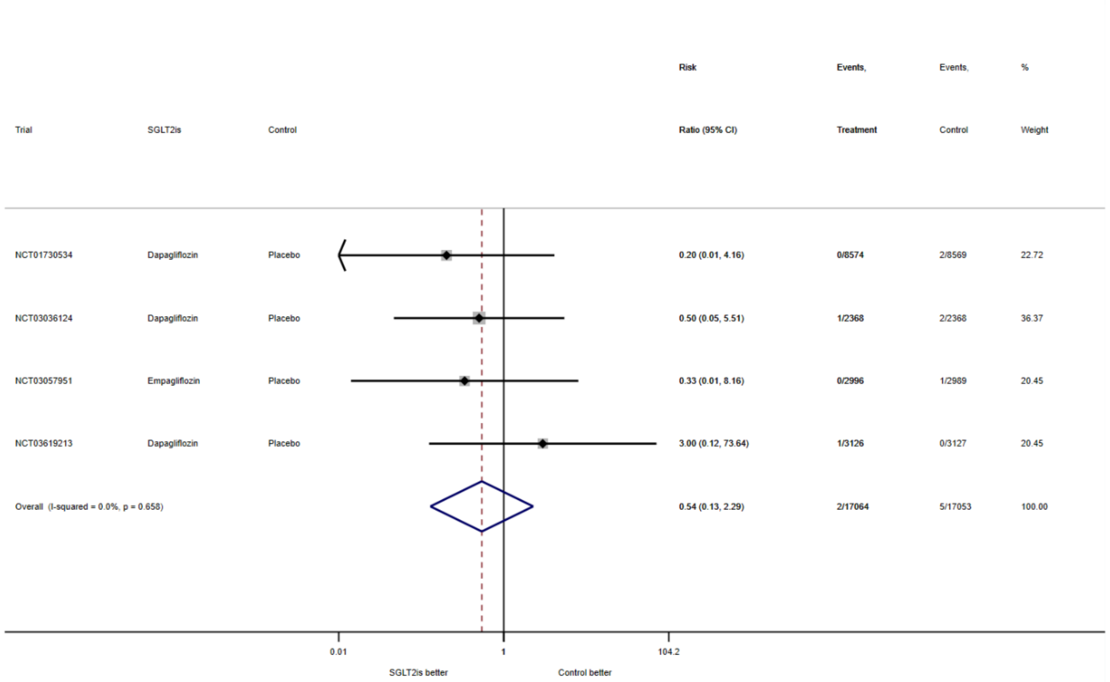

System 2-Cardiac Disorders-Figure S71 Meta-analysis of SGLT2is and Tachyarrhythmia

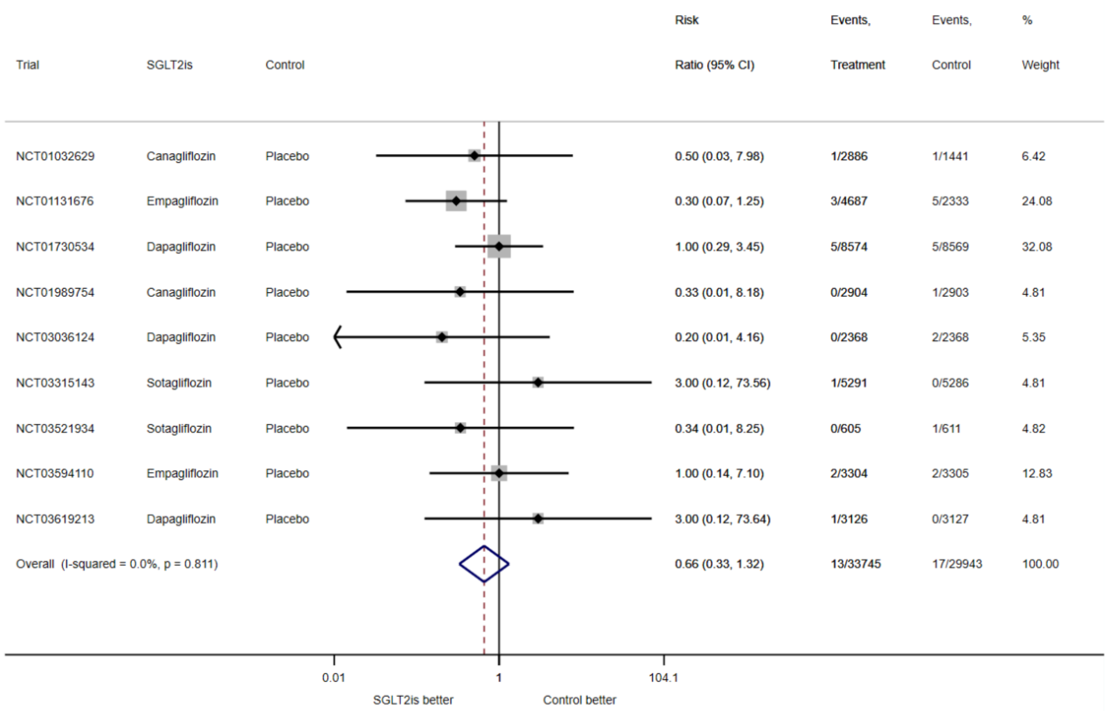

System 2-Cardiac Disorders-Figure S72 Meta-analysis of SGLT2is and Tachycardia

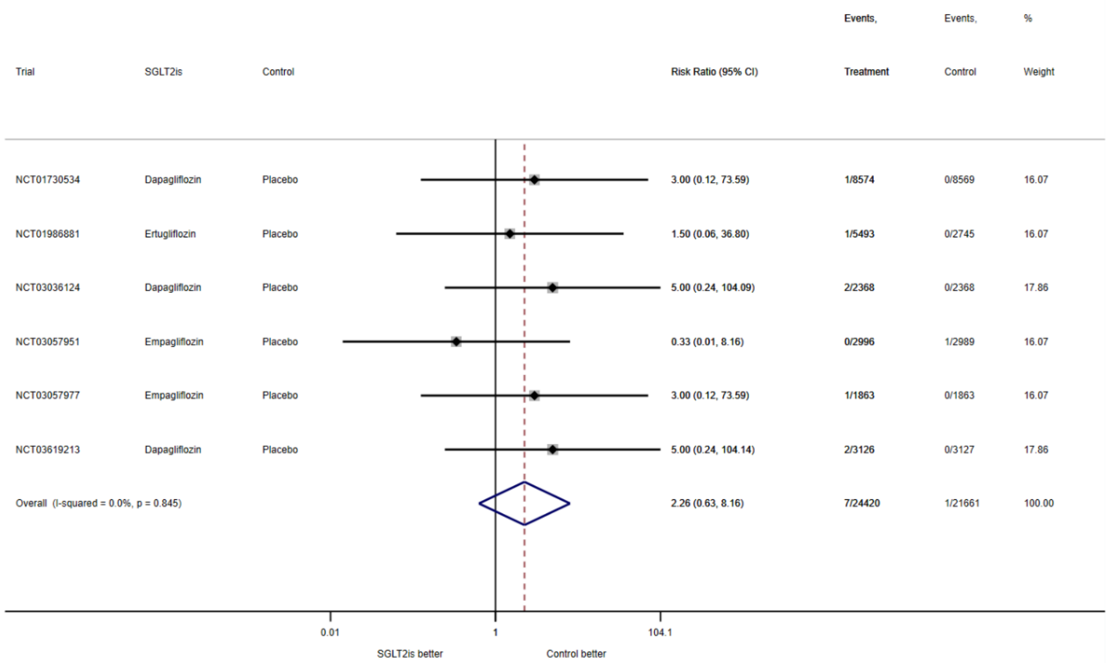

System 2-Cardiac Disorders-Figure S73 Meta-analysis of SGLT2is and Torsade de pointes

Trial	SGLT2is	Control	Risk Ratio (95% CI)	Events, Treatment	Events, Control	% Weight
NCT01730534	Dapagliflozin	Placebo	3.00 (0.31, 28.82)	3/8574	1/8569	18.78
NCT01986881	Ertugliflozin	Placebo	0.50 (0.03, 7.99)	1/5493	1/2745	12.52
NCT01989754	Canagliflozin	Placebo	3.00 (0.12, 73.59)	1/2904	0/2903	9.39
NCT02065791	Canagliflozin	Placebo	0.20 (0.01, 4.16)	0/2200	2/2197	10.44
NCT03036124	Dapagliflozin	Placebo	3.00 (0.12, 73.61)	1/2368	0/2368	9.39
NCT03057951	Empagliflozin	Placebo	0.67 (0.11, 3.98)	2/2996	3/2989	30.07
NCT03315143	Sotagliflozin	Placebo	0.33 (0.01, 8.17)	0/5291	1/5286	9.39
Overall (I-squared = 0.0%, p = 0.708)			0.93 (0.35, 2.49)	8/29826	8/27057	100.00

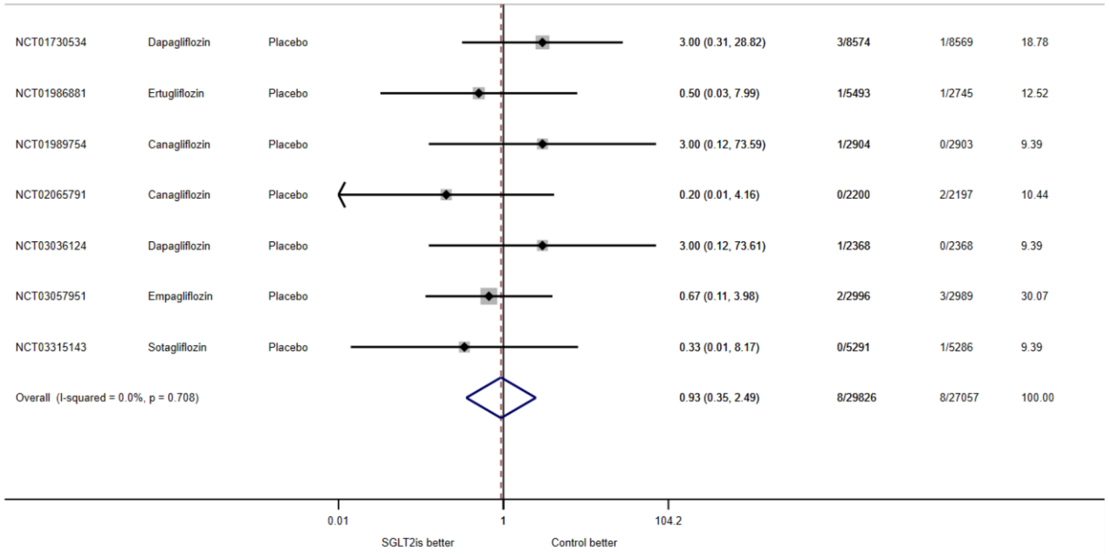

System 2-Cardiac Disorders-Figure S74 Meta-analysis of SGLT2is and Tricuspid valve incompetence

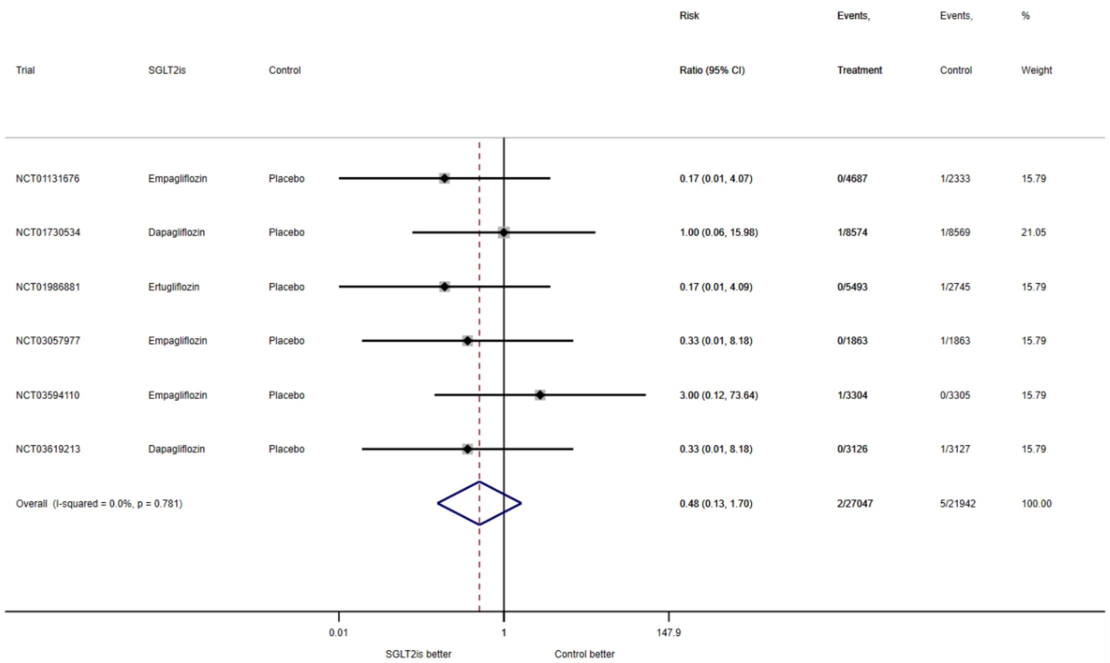

System 2-Cardiac Disorders-Figure S75 Meta-analysis of SGLT2is and Trifascicular block

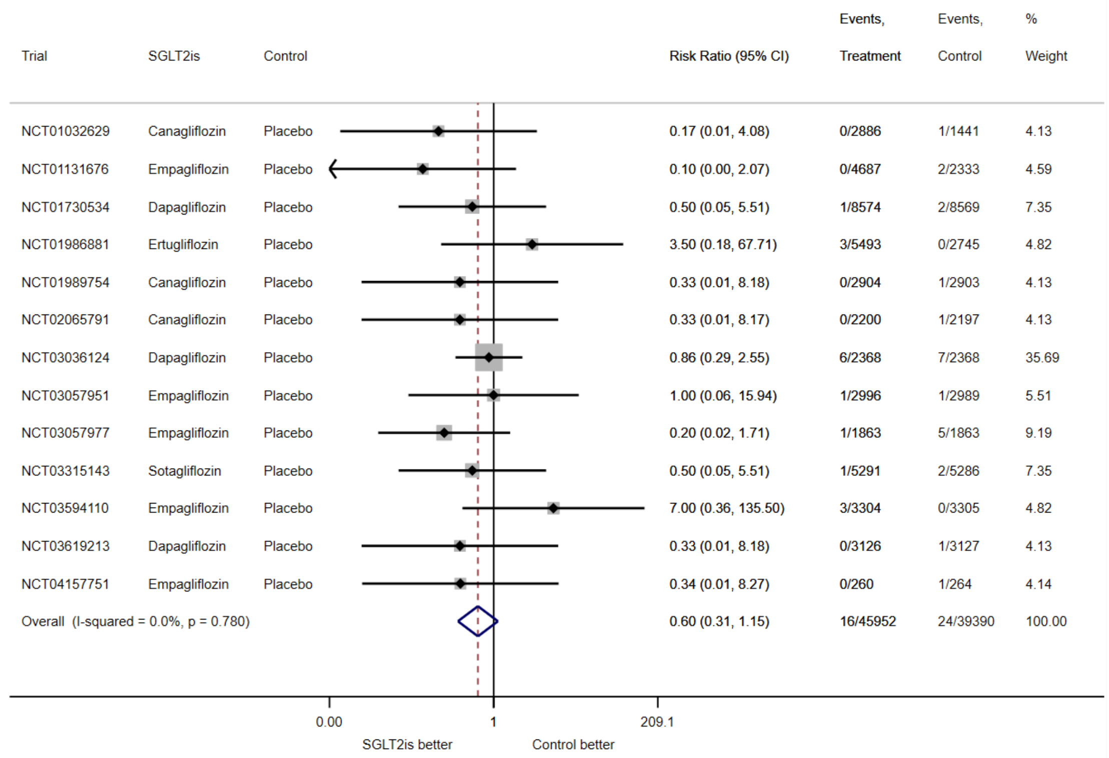

System 2-Cardiac Disorders-Figure S76 Meta-analysis of SGLT2is and Ventricular arrhythmia

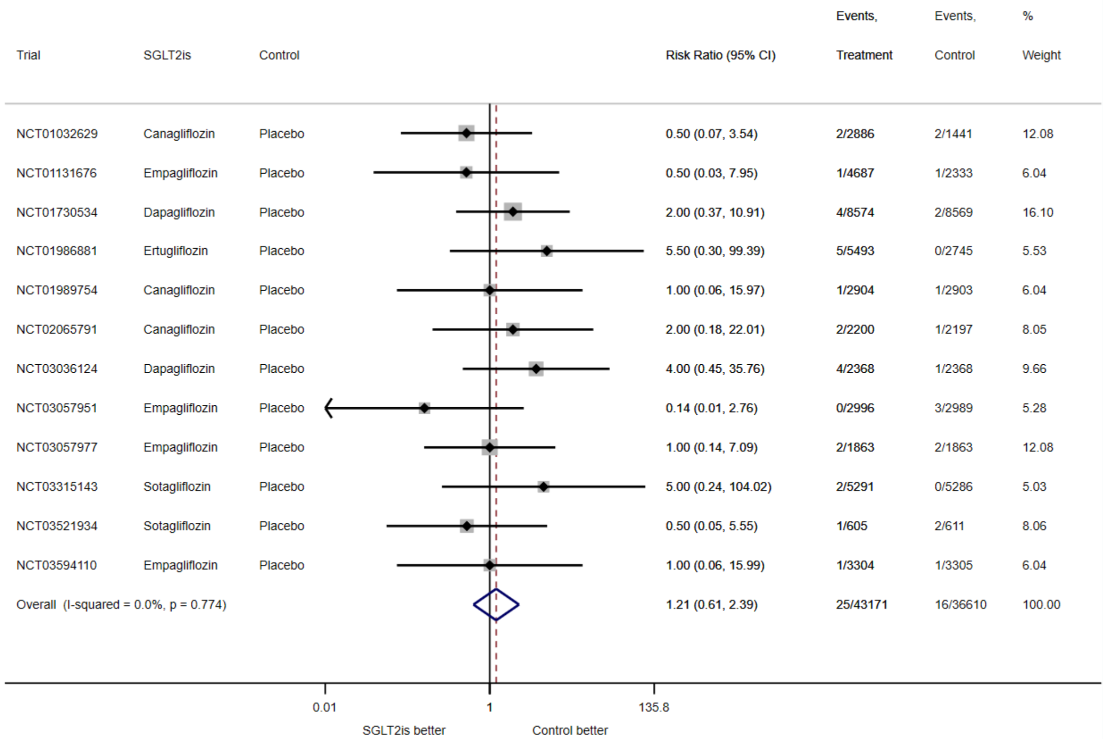

System 2-Cardiac Disorders-Figure S77 Meta-analysis of SGLT2is and Ventricular extrasystoles

Trial	SGLT2is	Control	Risk Ratio (95% CI)	Events, Treatment	Events, Control	% Weight
NCT01032629	Canagliflozin	Placebo	0.50 (0.10, 2.47)	3/2886	3/1441	5.80
NCT01131676	Empagliflozin	Placebo	0.75 (0.12, 4.47)	3/4687	2/2333	4.64
NCT01730534	Dapagliflozin	Placebo	0.89 (0.34, 2.30)	8/8574	9/8569	16.37
NCT01986881	Ertugliflozin	Placebo	2.00 (0.42, 9.41)	8/5493	2/2745	6.18
NCT01989754	Canagliflozin	Placebo	5.00 (0.24, 104.07)	2/2904	0/2903	1.61
NCT02065791	Canagliflozin	Placebo	0.50 (0.05, 5.50)	1/2200	2/2197	2.58
NCT03036124	Dapagliflozin	Placebo	1.83 (0.68, 4.95)	11/2368	6/2368	15.04
NCT03057951	Empagliflozin	Placebo	1.25 (0.34, 4.64)	5/2996	4/2989	8.59
NCT03057977	Empagliflozin	Placebo	1.25 (0.49, 3.16)	10/1863	8/1863	17.24
NCT03315143	Sotagliflozin	Placebo	0.25 (0.03, 2.23)	1/5291	4/5286	3.09
NCT03521934	Sotagliflozin	Placebo	1.01 (0.06, 16.11)	1/605	1/611	1.93
NCT03594110	Empagliflozin	Placebo	1.00 (0.14, 7.10)	2/3304	2/3305	3.86
NCT03619213	Dapagliflozin	Placebo	1.00 (0.32, 3.10)	6/3126	6/3127	11.61
NCT04157751	Empagliflozin	Placebo	0.34 (0.01, 8.27)	0/260	1/264	1.45
Overall (I-squared = 0.0%, p = 0.918)			1.07 (0.73, 1.57)	61/46557	50/40001	100.00

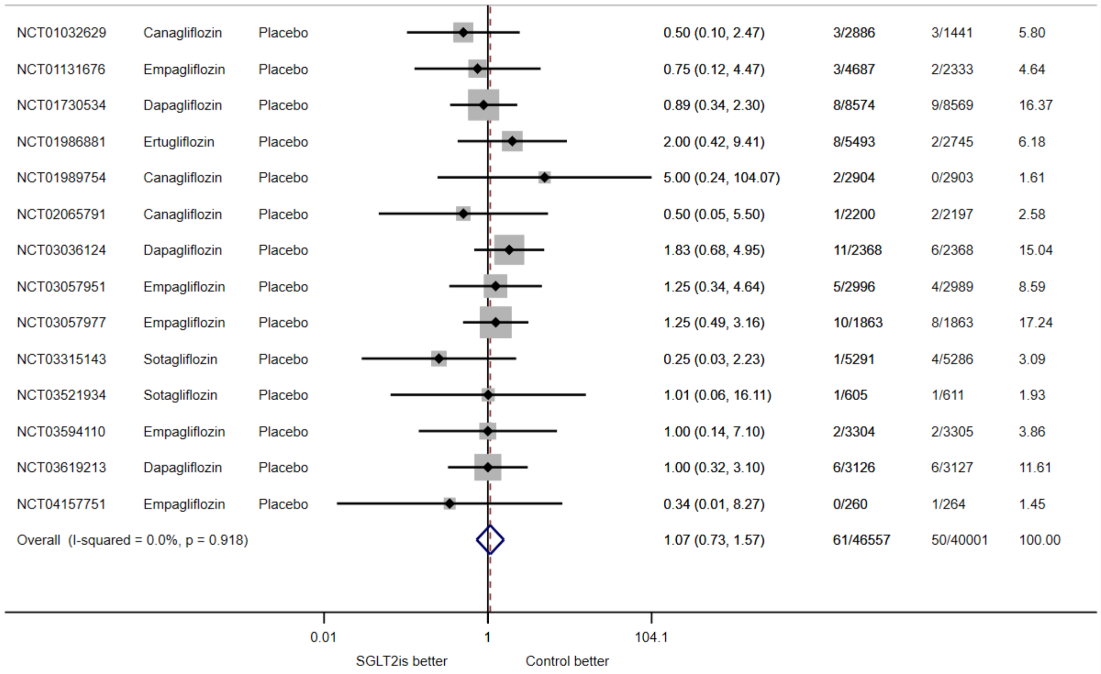

System 2-Cardiac Disorders-Figure S78 Meta-analysis of SGLT2is and Ventricular fibrillation

Trial	SGLT2is	Control	Risk Ratio (95% CI)	Events, Treatment	Events, Control	% Weight
NCT01032629	Canagliflozin	Placebo	0.83 (0.20, 3.48)	5/2886	3/1441	2.07
NCT01042977	Dapagliflozin	Placebo	3.01 (0.12, 73.61)	1/482	0/483	0.41
NCT01131676	Empagliflozin	Placebo	0.44 (0.16, 1.20)	7/4687	8/2333	4.13
NCT01730534	Dapagliflozin	Placebo	1.27 (0.64, 2.49)	19/8574	15/8569	9.26
NCT01986881	Ertugliflozin	Placebo	1.07 (0.44, 2.62)	15/5493	7/2745	5.28
NCT01989754	Canagliflozin	Placebo	0.50 (0.05, 5.51)	1/2904	2/2903	0.74
NCT02065791	Canagliflozin	Placebo	0.50 (0.05, 5.50)	1/2200	2/2197	0.74
NCT03036124	Dapagliflozin	Placebo	0.63 (0.41, 0.96)	34/2368	54/2368	23.42
NCT03036150	Dapagliflozin	Placebo	3.00 (0.12, 73.60)	1/2149	0/2149	0.41
NCT03057951	Empagliflozin	Placebo	1.00 (0.55, 1.80)	22/2996	22/2989	12.22
NCT03057977	Empagliflozin	Placebo	1.49 (0.98, 2.24)	55/1863	37/1863	24.99
NCT03315143	Sotagliflozin	Placebo	1.00 (0.25, 3.99)	4/5291	4/5286	2.21
NCT03521934	Sotagliflozin	Placebo	1.01 (0.25, 4.02)	4/605	4/611	2.22
NCT03594110	Empagliflozin	Placebo	5.00 (0.24, 104.14)	2/3304	0/3305	0.46
NCT03619213	Dapagliflozin	Placebo	1.00 (0.46, 2.15)	13/3126	13/3127	7.20
NCT04157751	Empagliflozin	Placebo	1.16 (0.43, 3.15)	8/260	7/264	4.24
Overall (I-squared = 0.0%, p = 0.529)			0.99 (0.81, 1.22)	192/49188	178/42633	100.00

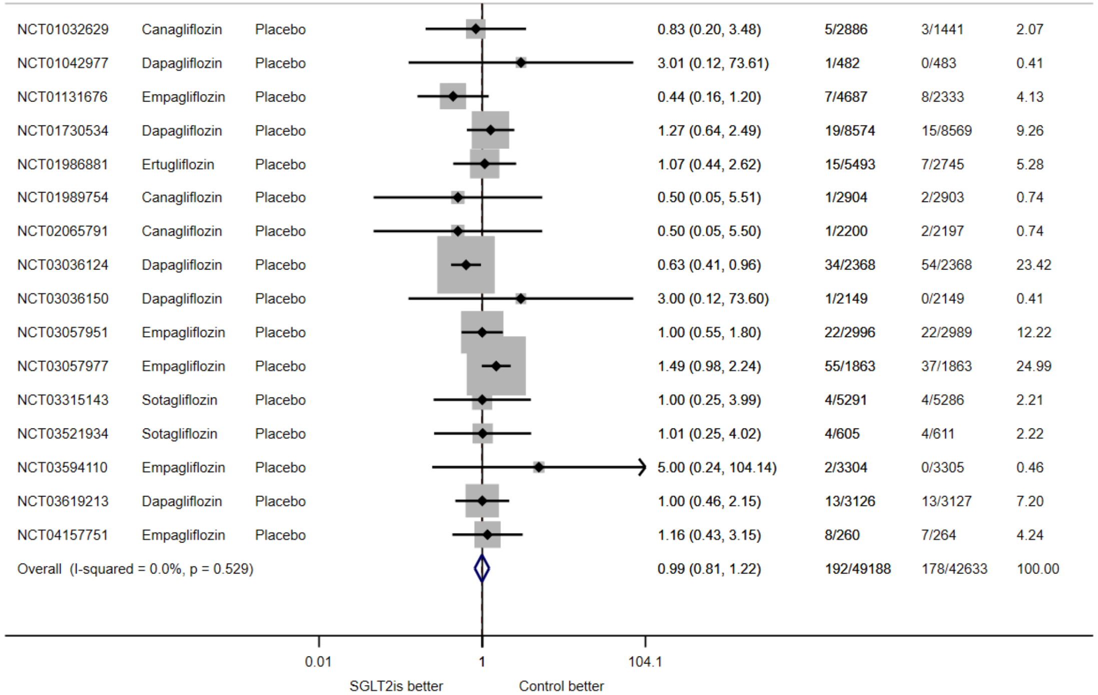

System 2-Cardiac Disorders-Figure S79 Meta-analysis of SGLT2is and Ventricular tachycardia

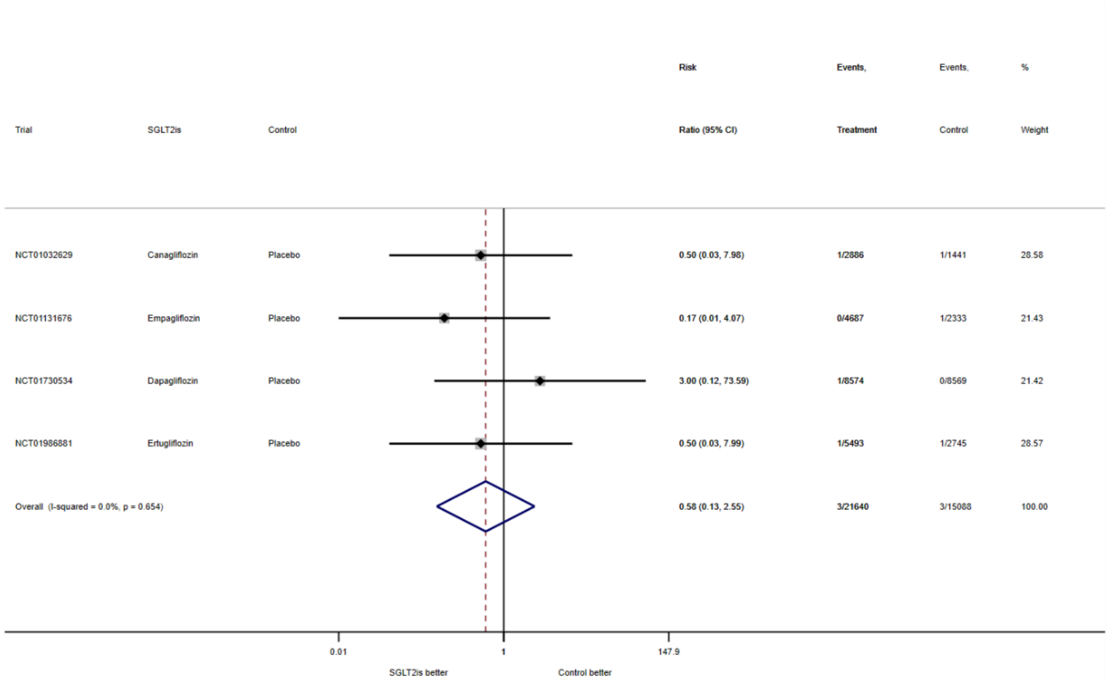

System 3-Congenital, Familial And Genetic Disorders-Figure S1 Meta-analysis of SGLT2is and Hydrocele

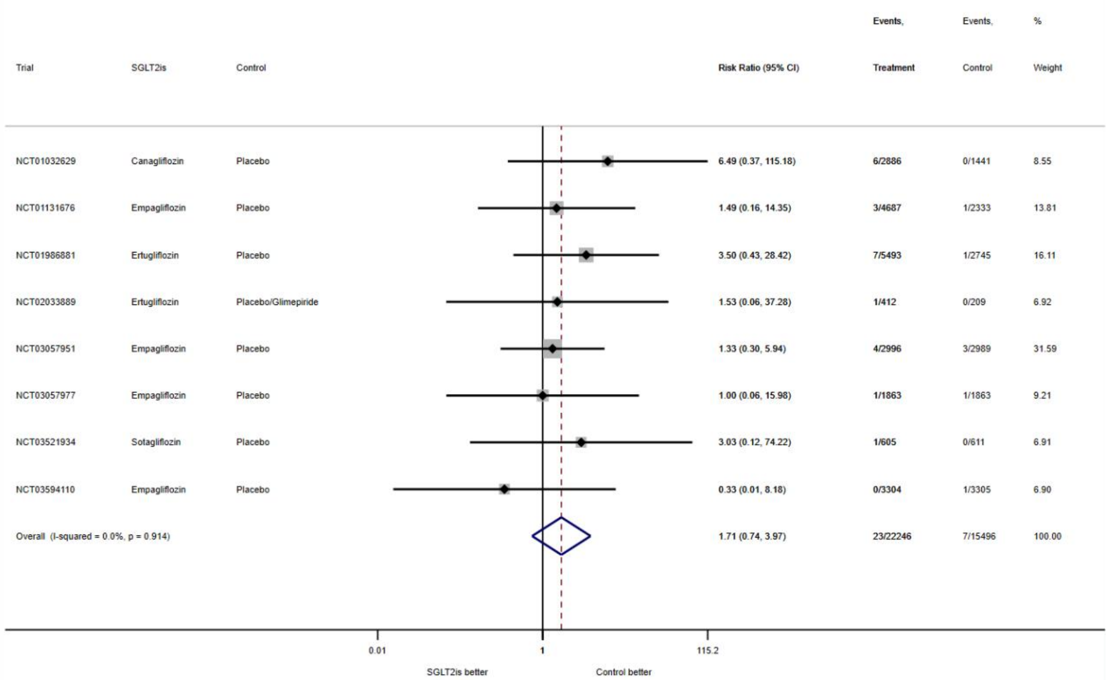

System 3-Congenital, Familial And Genetic Disorders-Figure S2 Meta-analysis of SGLT2is and Phimosis

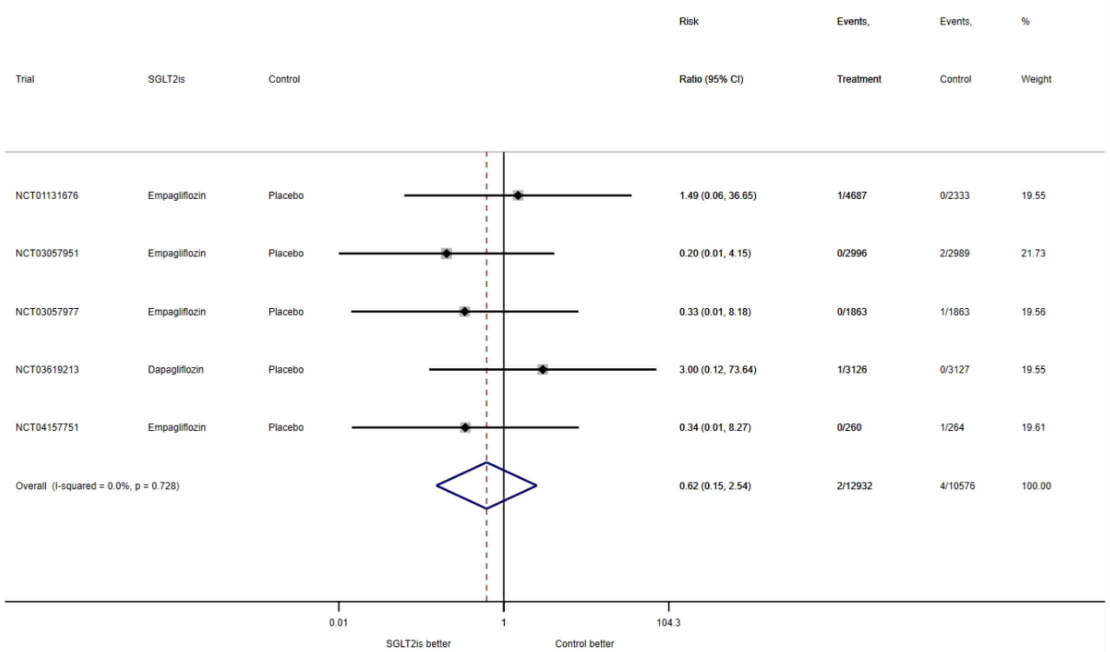

System 4-Ear And Labyrinth Disorders-Figure S1 Meta-analysis of SGLT2is and Deafness

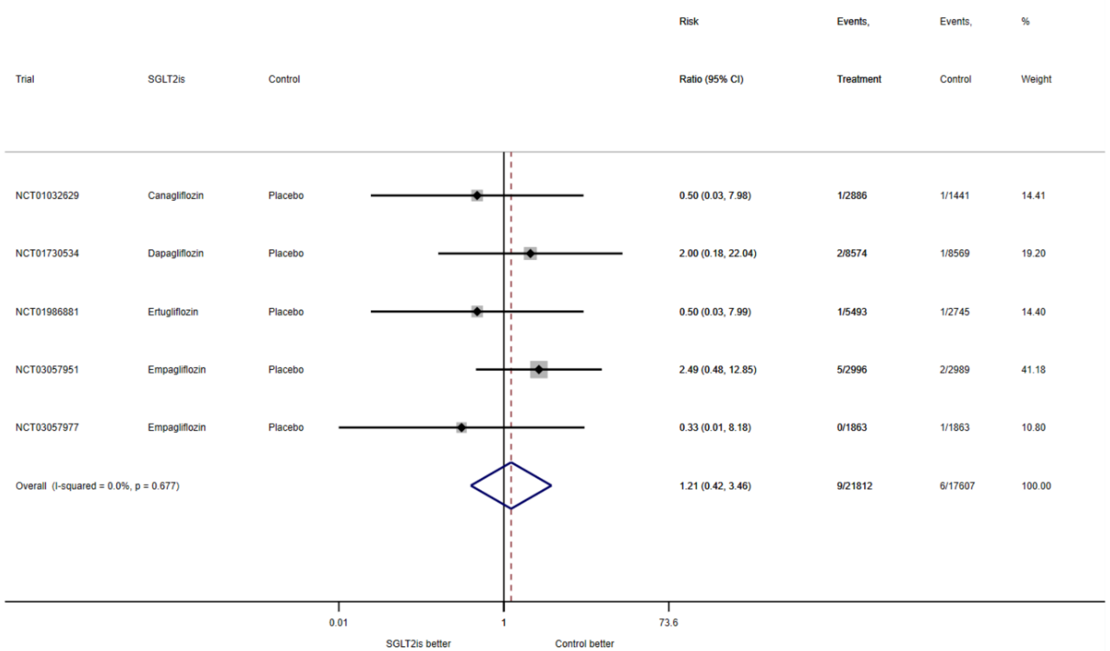

System 4-Ear And Labyrinth Disorders-Figure S2 Meta-analysis of SGLT2is and Deafness neurosensory

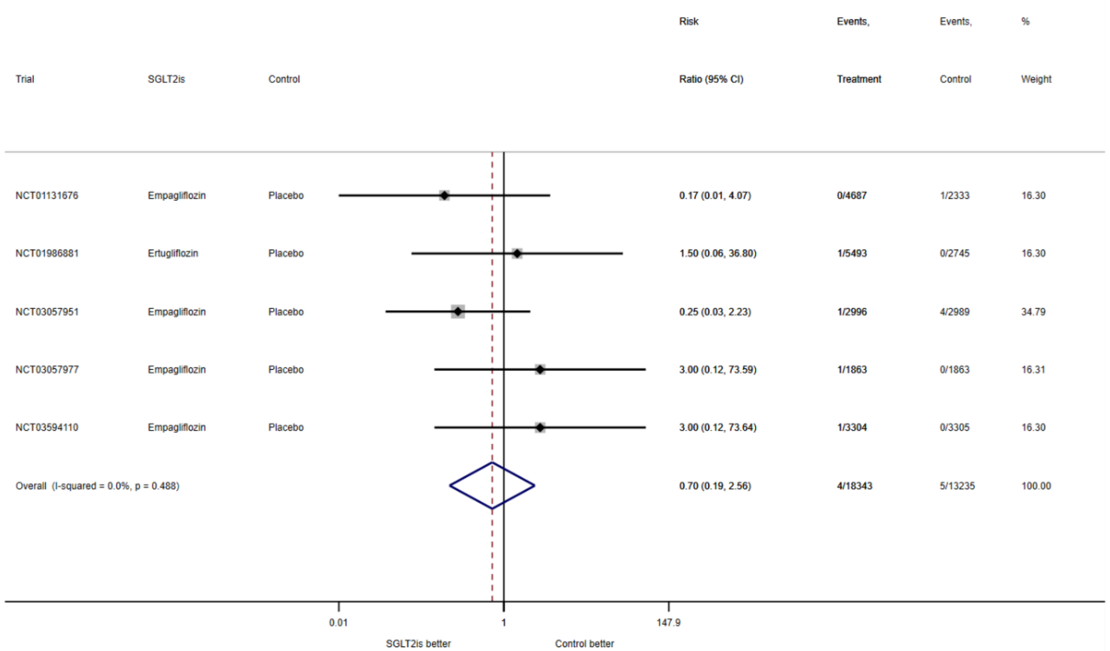

System 4-Ear And Labyrinth Disorders-Figure S3 Meta-analysis of SGLT2is and Deafness unilateral

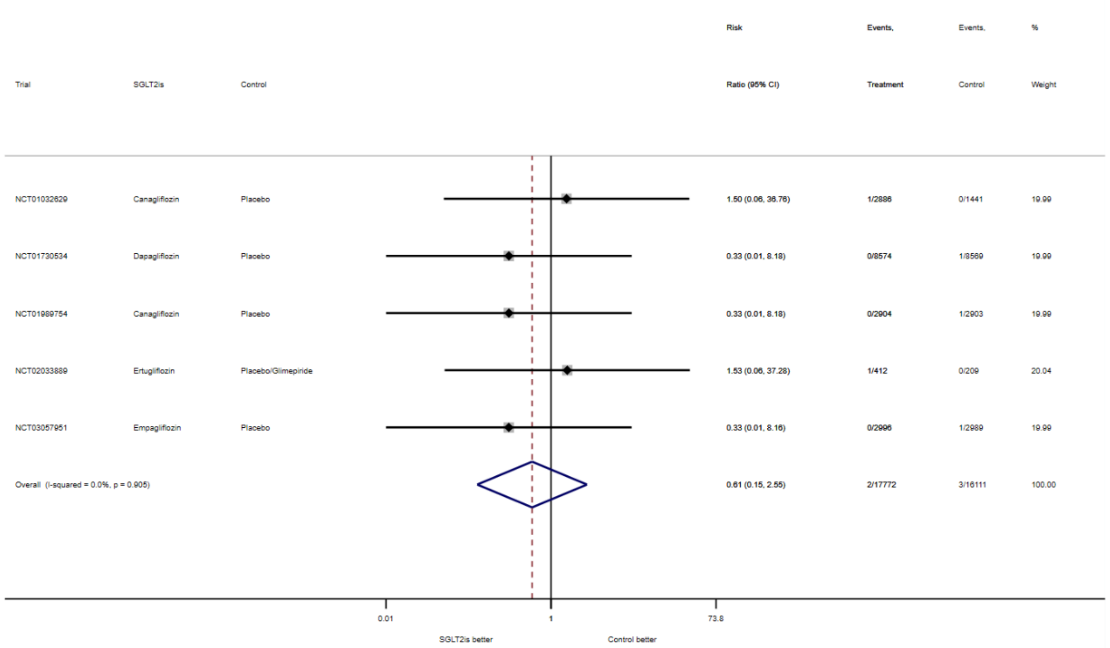

System 4-Ear And Labyrinth Disorders-Figure S4 Meta-analysis of SGLT2is and Hypoacusis

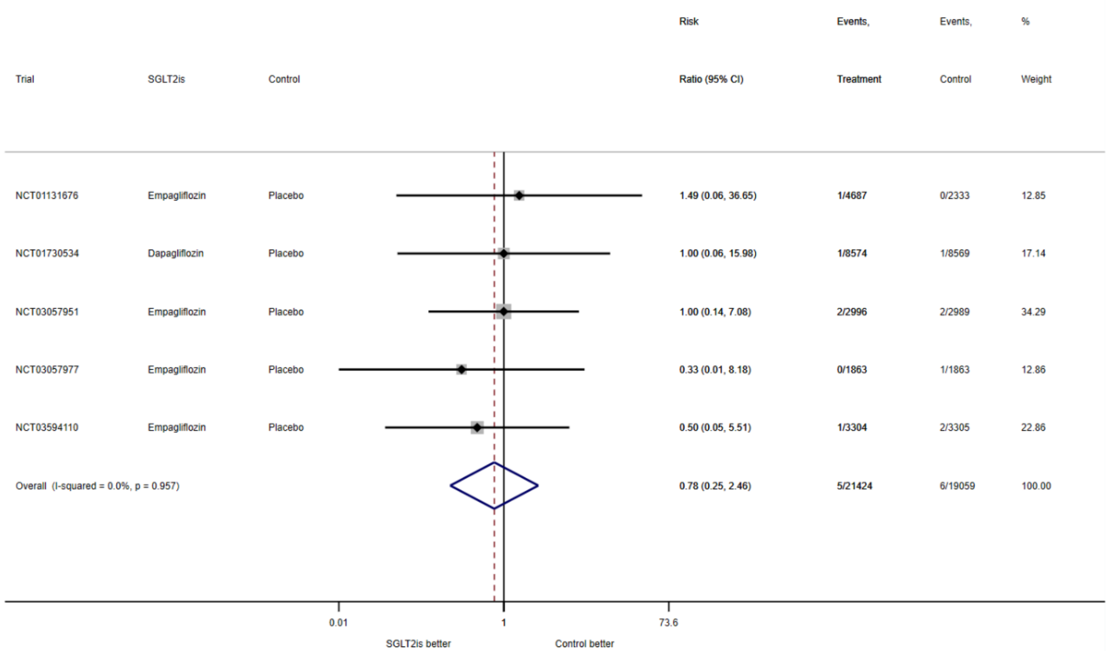

System 4-Ear And Labyrinth Disorders-Figure S5 Meta-analysis of SGLT2is and Sudden hearing loss

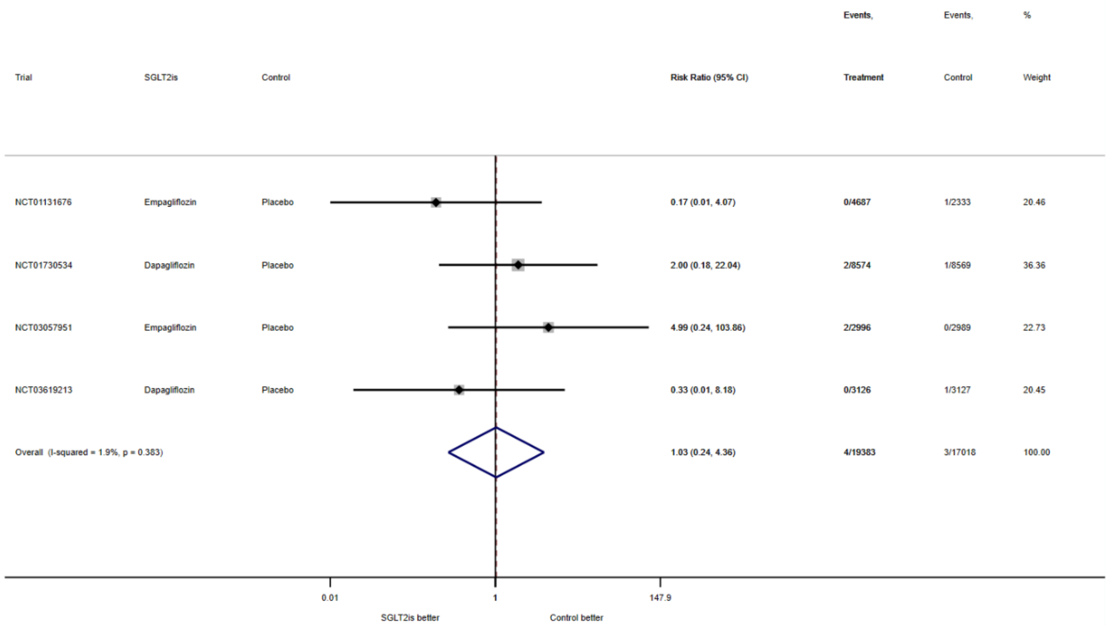

System 4-Ear And Labyrinth Disorders-Figure S6 Meta-analysis of SGLT2is and Tinnitus

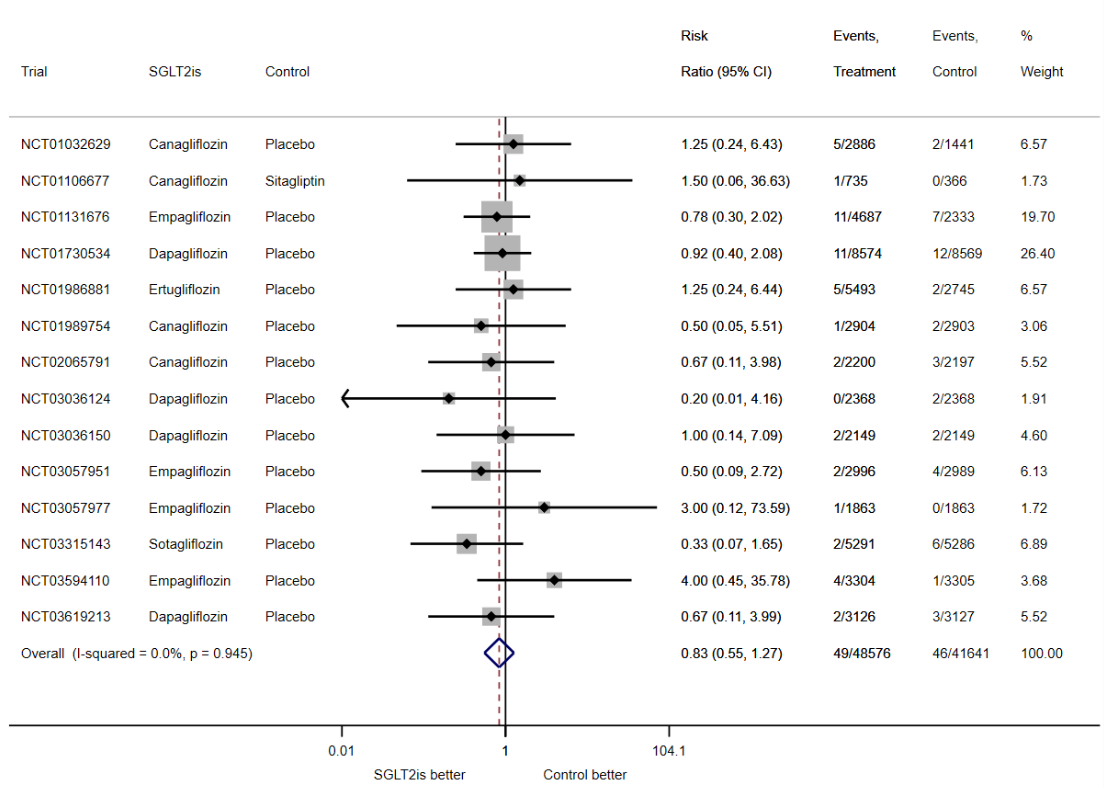

System 4-Ear And Labyrinth Disorders-Figure S7 Meta-analysis of SGLT2is and Vertigo

Trial	SGLT2is	Control	Risk Ratio (95% CI)	Events, Treatment	Events, Control	% Weight
NCT00673231	Dapagliflozin	Placebo	0.16 (0.01, 1.77)	1/610	2/197	5.92
NCT01032629	Canagliflozin	Placebo	0.50 (0.07, 3.54)	2/2886	2/1441	8.84
NCT01131676	Empagliflozin	Placebo	0.17 (0.03, 0.82)	2/4687	6/2333	13.26
NCT01730534	Dapagliflozin	Placebo	0.62 (0.20, 1.91)	5/8574	8/8569	27.20
NCT01986881	Ertugliflozin	Placebo	0.75 (0.13, 4.48)	3/5493	2/2745	10.61
NCT02065791	Canagliflozin	Placebo	3.00 (0.12, 73.50)	1/2200	0/2197	3.31
NCT03036124	Dapagliflozin	Placebo	0.33 (0.03, 3.20)	1/2368	3/2368	6.63
NCT03057951	Empagliflozin	Placebo	8.98 (0.48, 166.70)	4/2996	0/2989	3.98
NCT03057977	Empagliflozin	Placebo	3.00 (0.12, 73.59)	1/1863	0/1863	3.31
NCT03315143	Sotagliflozin	Placebo	5.00 (0.24, 104.02)	2/5291	0/5286	3.68
NCT03521934	Sotagliflozin	Placebo	3.03 (0.12, 74.22)	1/605	0/611	3.32
NCT03594110	Empagliflozin	Placebo	0.33 (0.01, 8.18)	0/3304	1/3305	3.31
NCT03619213	Dapagliflozin	Placebo	3.00 (0.31, 28.83)	3/3126	1/3127	6.63
Overall (I-squared = 13.4%, p = 0.310)			0.71 (0.39, 1.27)	26/44003	25/37031	100.00

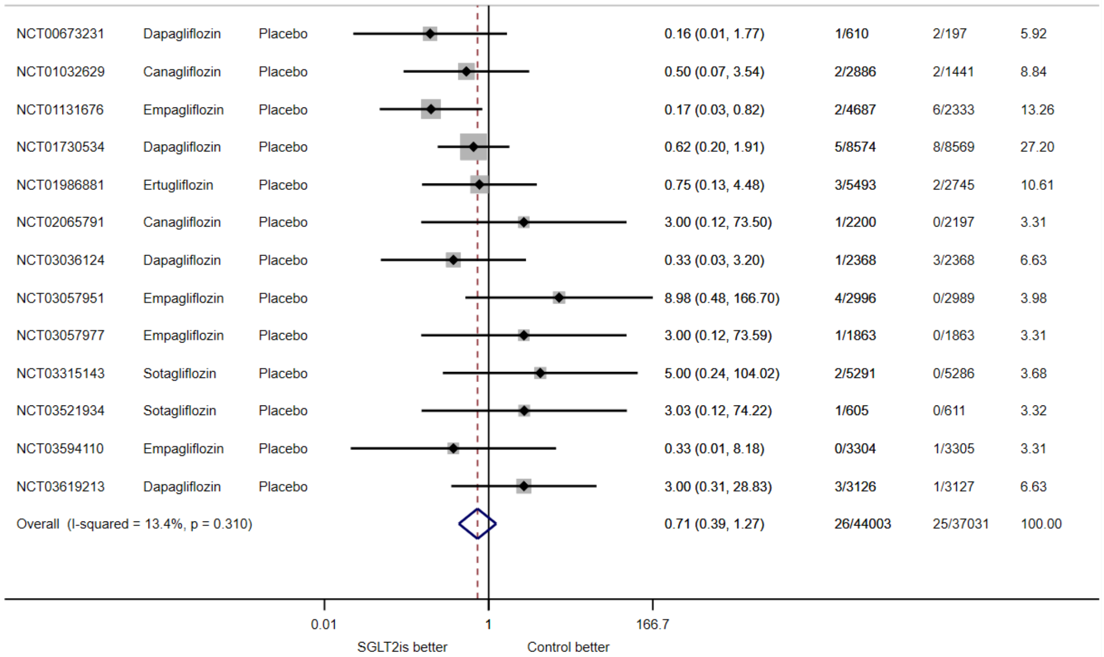

System 4-Ear And Labyrinth Disorders-Figure S8 Meta-analysis of SGLT2is and Vertigo positional

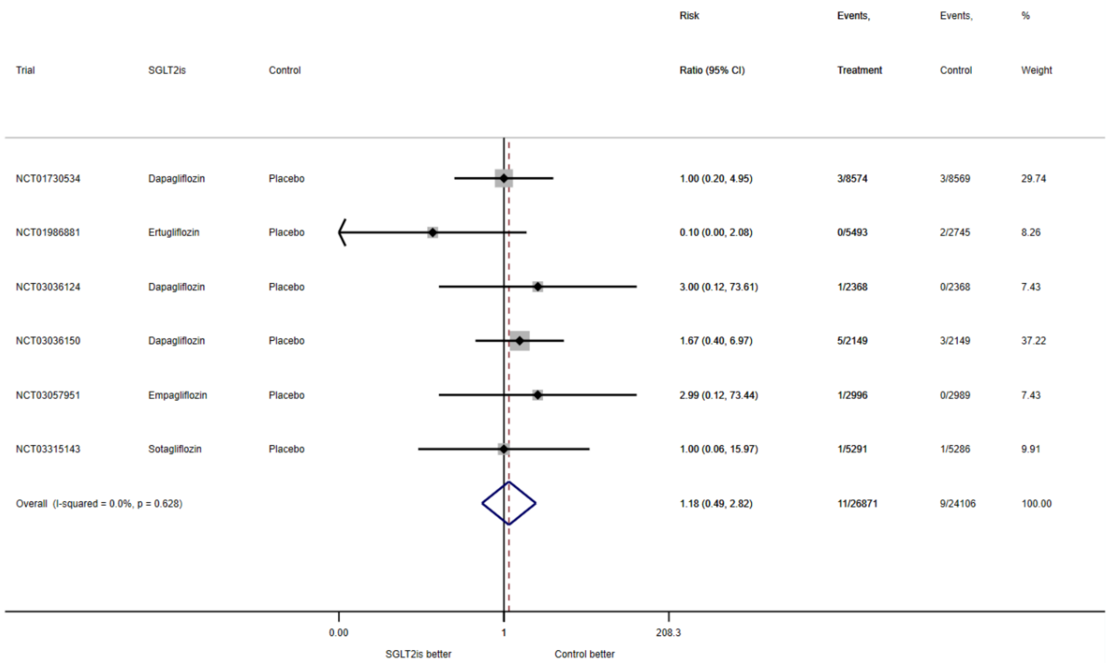

System 4-Ear And Labyrinth Disorders-Figure S9 Meta-analysis of SGLT2is and Vestibular disorder

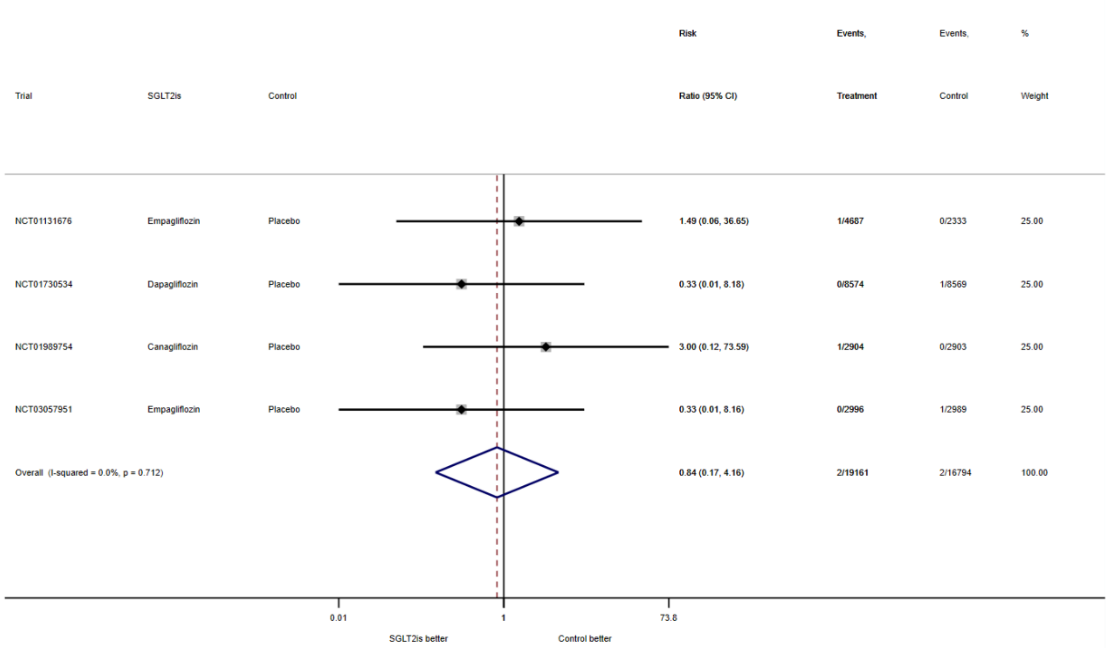

System 5-Endocrine Disorders-Figure S1 Meta-analysis of SGLT2is and Basedow's disease

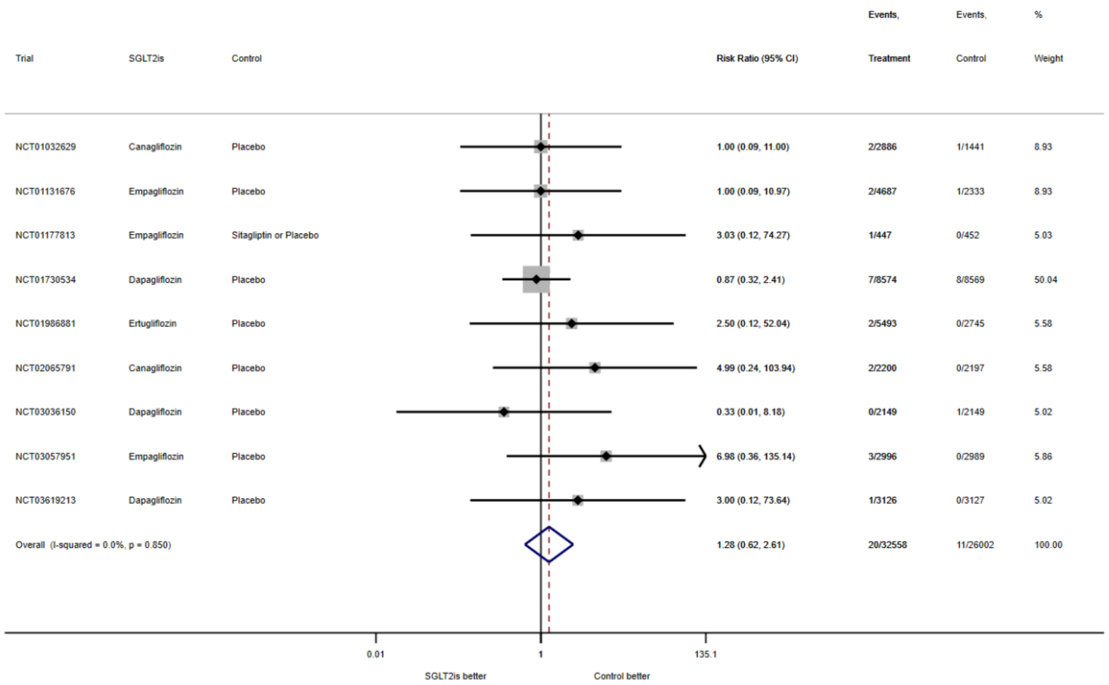

System 5-Endocrine Disorders-Figure S2 Meta-analysis of SGLT2is and Goitre

Trial	SGLT2is	Control	Risk Ratio (95% CI)	Events, Treatment	Events, Control	% Weight
NCT01032629	Canagliflozin	Placebo	1.50 (0.06, 36.76)	1/2886	0/1441	14.18
NCT01131676	Empagliflozin	Placebo	3.49 (0.18, 67.44)	3/4687	0/2333	16.54
NCT01730534	Dapagliflozin	Placebo	2.00 (0.18, 22.04)	2/8574	1/8569	25.19
NCT01989754	Canagliflozin	Placebo	0.20 (0.01, 4.16)	0/2904	2/2903	15.75
NCT02065791	Canagliflozin	Placebo	0.33 (0.01, 8.17)	0/2200	1/2197	14.17
NCT03315143	Sotagliflozin	Placebo	0.33 (0.01, 8.17)	0/5291	1/5286	14.17
Overall (I-squared = 0.0%, p = 0.699)			0.88 (0.26, 2.94)	6/26542	5/22729	100.00

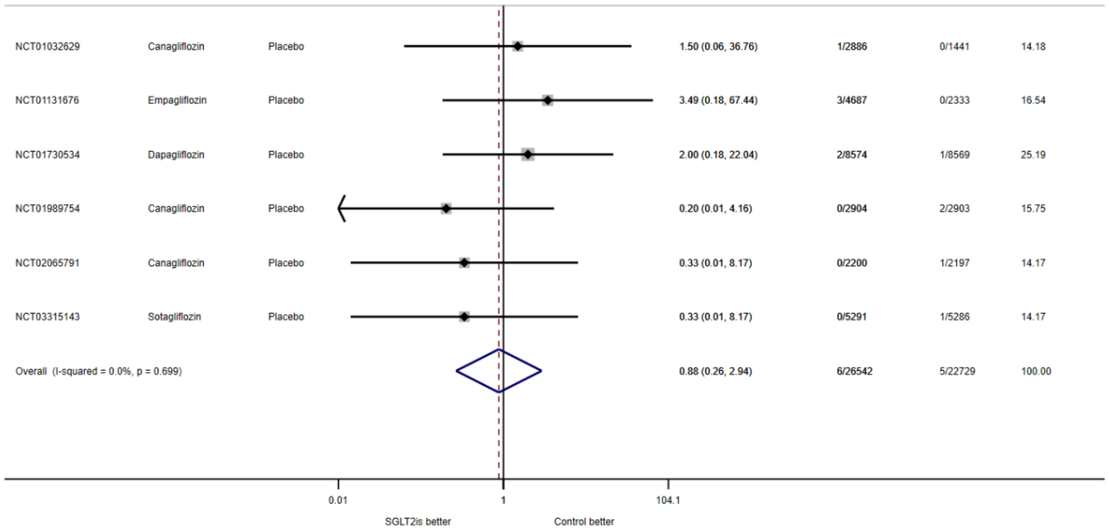

System 5-Endocrine Disorders-Figure S3 Meta-analysis of SGLT2is and Hyperparathyroidism

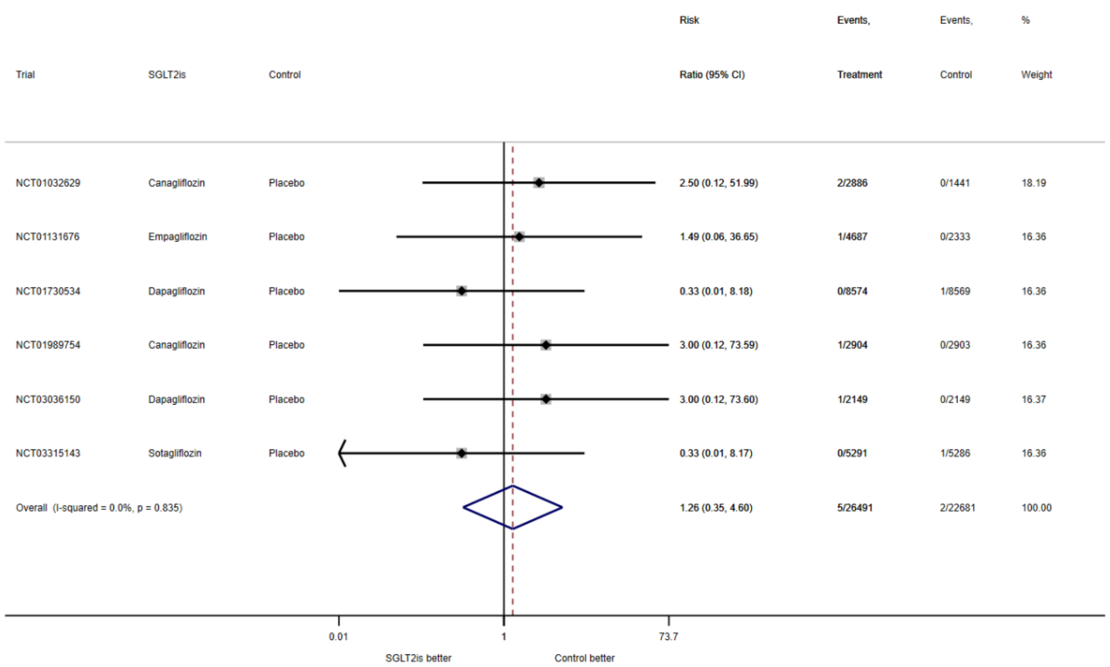

System 5-Endocrine Disorders-Figure S4 Meta-analysis of SGLT2is and Hyperparathyroidism primary

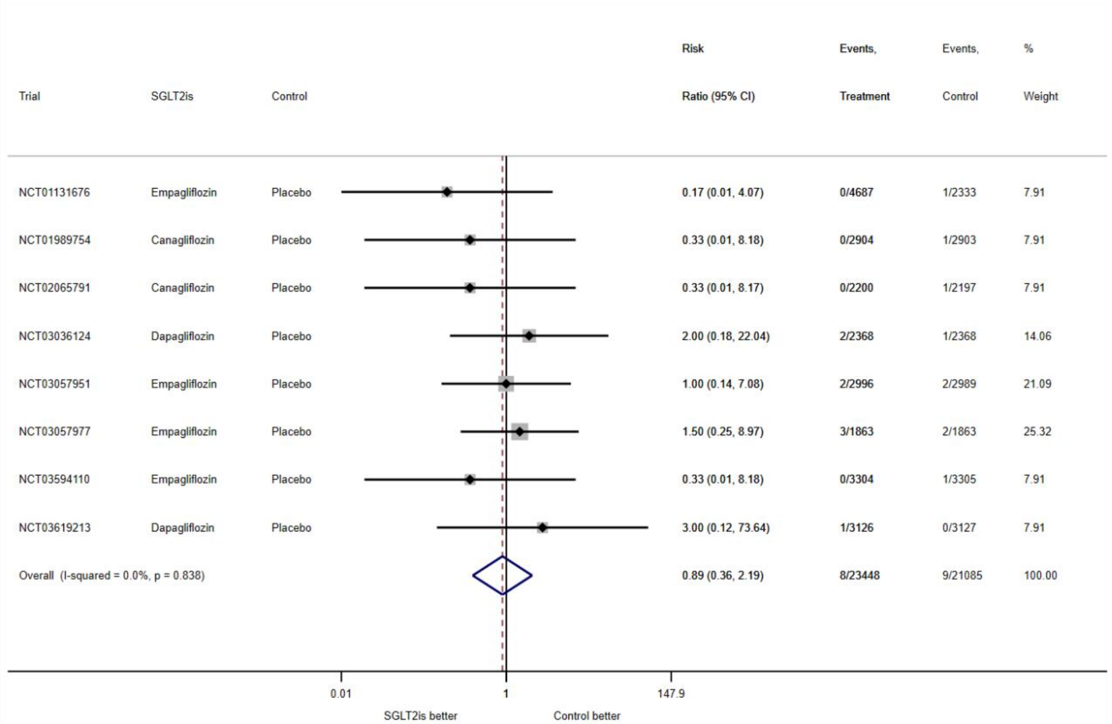

System 5-Endocrine Disorders-Figure S5 Meta-analysis of SGLT2is and Hyperthyroidism

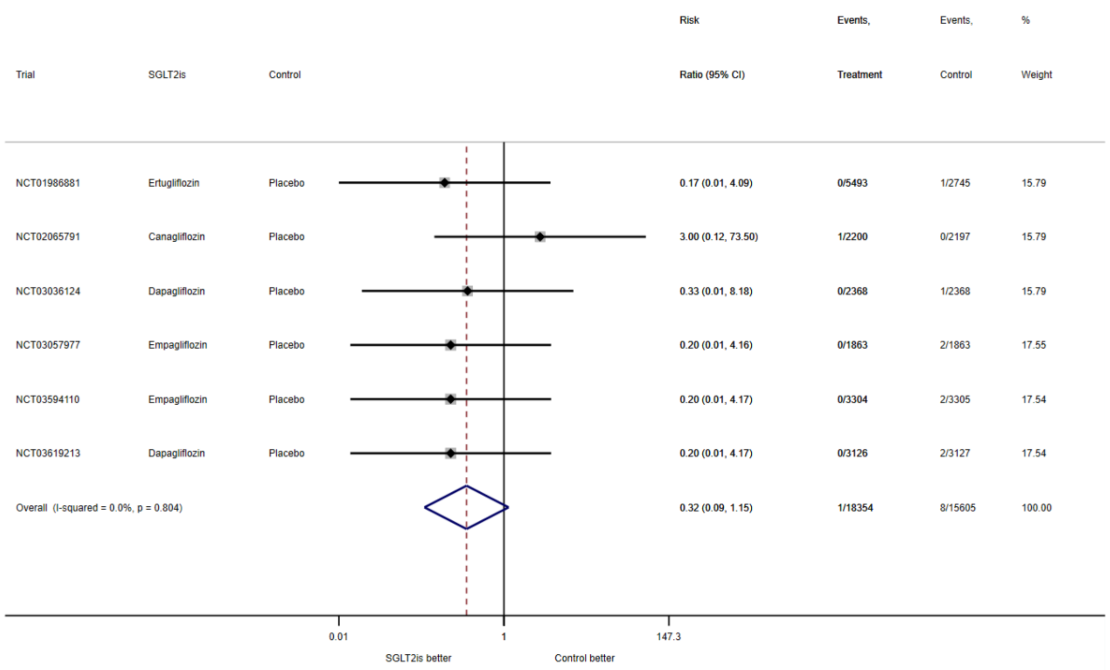

System 5-Endocrine Disorders-Figure S6 Meta-analysis of SGLT2is and Hypothyroidism

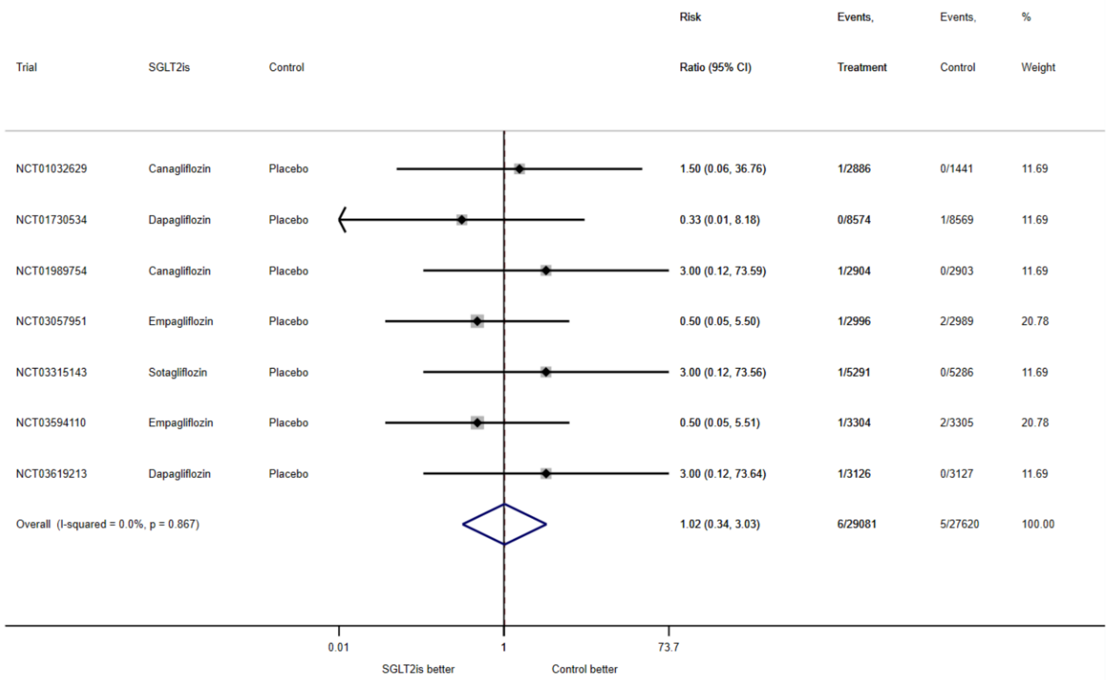

System 6-Eye Disorders-Figure S1 Meta-analysis of SGLT2is and Amaurosis fugax

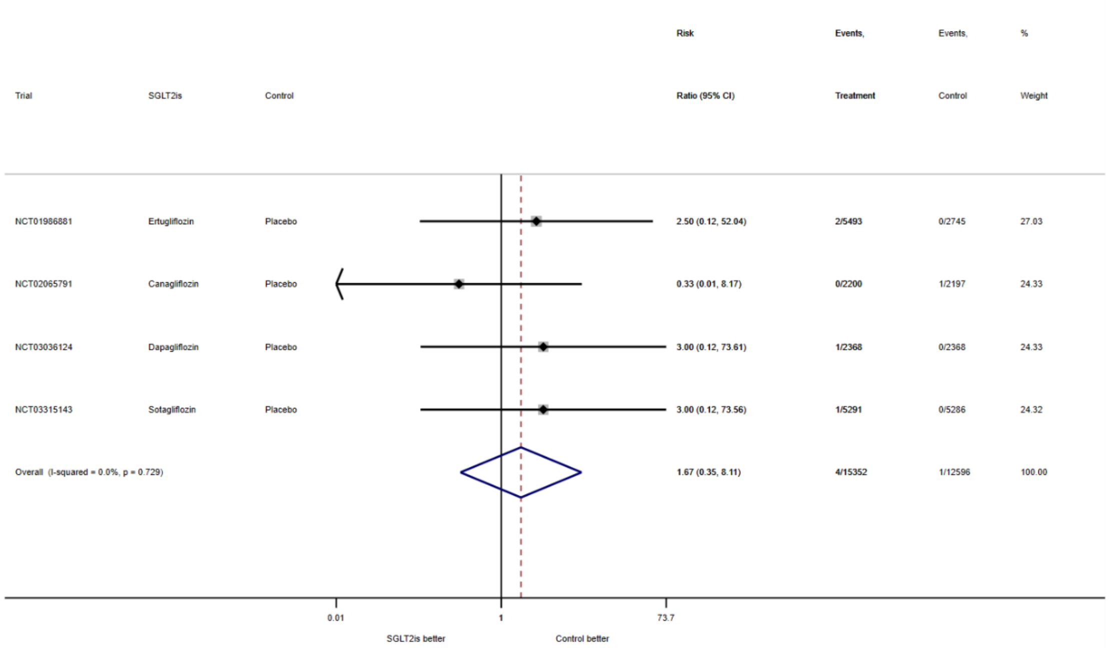

System 6-Eye Disorders-Figure S2 Meta-analysis of SGLT2is and Angle closure glaucoma

Trial	SGLT2is	Control	Risk Ratio (95% CI)	Events, Treatment	Events, Control	% Weight
NCT01032629	Canagliflozin	Placebo	1.12 (0.49, 2.58)	18/2886	8/1441	8.82
NCT01131676	Empagliflozin	Placebo	1.33 (0.62, 2.85)	24/4687	9/2333	10.41
NCT01164501	Empagliflozin	Placebo	0.25 (0.01, 6.21)	0/419	1/319	0.60
NCT01177813	Empagliflozin	Sitagliptin or Placebo	3.03 (0.12, 74.27)	1/447	0/452	0.59
NCT01730534	Dapagliflozin	Placebo	1.08 (0.63, 1.83)	28/8574	26/8569	21.41
NCT01986881	Ertugliflozin	Placebo	0.62 (0.33, 1.17)	21/5493	17/2745	14.95
NCT01989754	Canagliflozin	Placebo	3.33 (0.92, 12.10)	10/2904	3/2903	3.66
NCT02033889	Ertugliflozin	Placebo/Glimepiride	1.53 (0.06, 37.28)	1/412	0/209	0.60
NCT02065791	Canagliflozin	Placebo	0.89 (0.34, 2.30)	8/2200	9/2197	6.73
NCT03036124	Dapagliflozin	Placebo	0.50 (0.15, 1.66)	4/2368	8/2368	4.23
NCT03036150	Dapagliflozin	Placebo	1.33 (0.46, 3.84)	8/2149	6/2149	5.45
NCT03057951	Empagliflozin	Placebo	1.00 (0.42, 2.39)	10/2996	10/2989	7.94
NCT03057977	Empagliflozin	Placebo	1.67 (0.40, 6.96)	5/1863	3/1863	2.97
NCT03315143	Sotagliflozin	Placebo	1.00 (0.29, 3.45)	5/5291	5/5286	3.96
NCT03594110	Empagliflozin	Placebo	1.00 (0.20, 4.95)	3/3304	3/3305	2.38
NCT03619213	Dapagliflozin	Placebo	2.00 (0.68, 5.85)	10/3126	5/3127	5.29
Overall (I-squared = 0.0%, p = 0.755)			1.06 (0.83, 1.36)	156/49119	113/42255	100.00

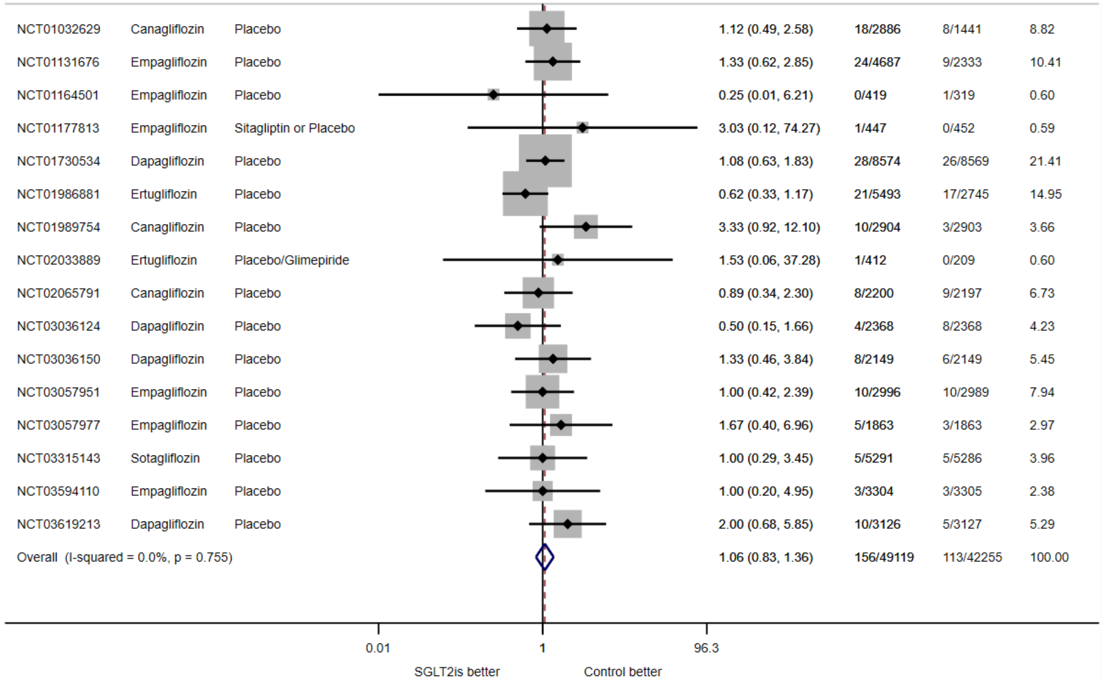

System 6-Eye Disorders-Figure S3 Meta-analysis of SGLT2is and Cataract

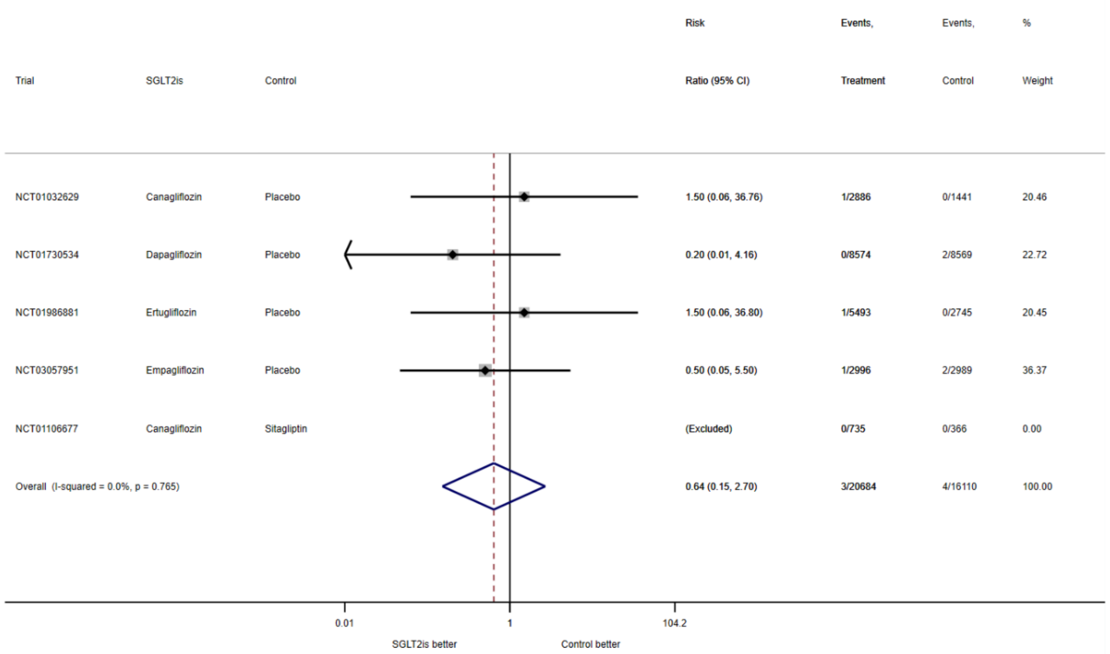

System 6-Eye Disorders-Figure S4 Meta-analysis of SGLT2is and Cataract nuclear

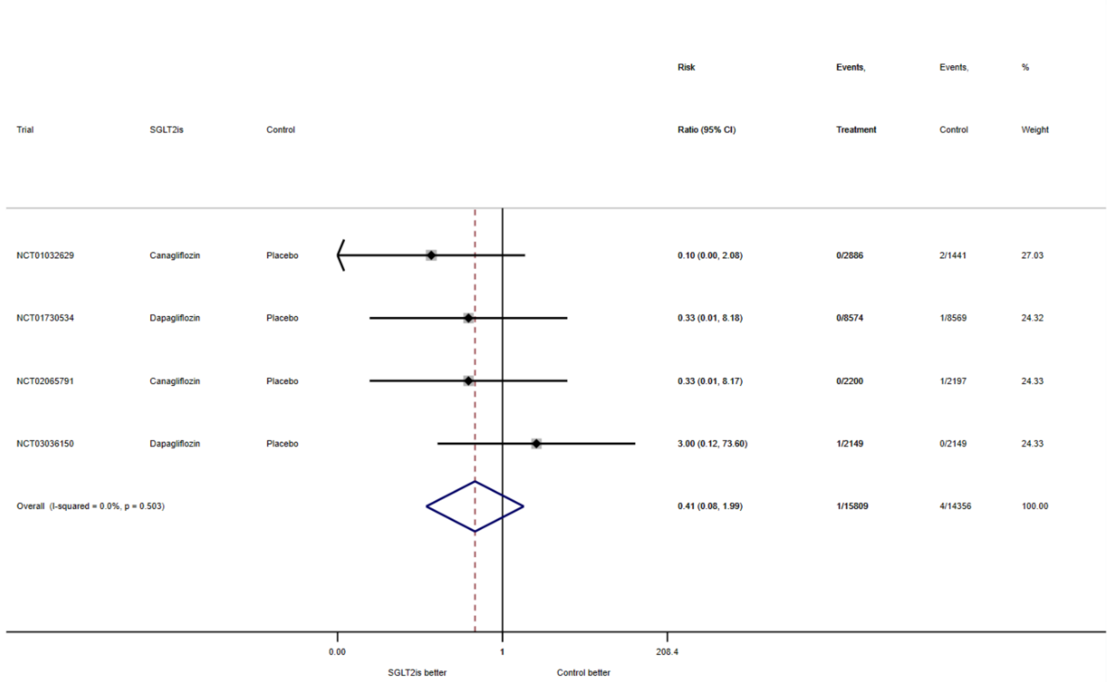

System 6-Eye Disorders-Figure S5 Meta-analysis of SGLT2is and Cataract subcapsular

Trial	SGLT2is	Control	Risk Ratio (95% CI)	Events, Treatment	Events, Control	% Weight
NCT01032629	Canagliflozin	Placebo	2.50 (0.29, 21.35)	5/2886	1/1441	8.11
NCT01131676	Empagliflozin	Placebo	0.37 (0.08, 1.67)	3/4687	4/2333	16.70
NCT01730534	Dapagliflozin	Placebo	1.00 (0.14, 7.09)	2/8574	2/8569	9.73
NCT01986881	Ertugliflozin	Placebo	0.17 (0.01, 4.09)	0/5493	1/2745	3.65
NCT01989754	Canagliflozin	Placebo	3.00 (0.12, 73.59)	1/2904	0/2903	3.65
NCT02065791	Canagliflozin	Placebo	1.75 (0.51, 5.96)	7/2200	4/2197	24.82
NCT03036124	Dapagliflozin	Placebo	0.33 (0.01, 8.18)	0/2368	1/2368	3.65
NCT03036150	Dapagliflozin	Placebo	4.00 (0.45, 35.76)	4/2149	1/2149	7.79
NCT03057951	Empagliflozin	Placebo	0.33 (0.01, 8.16)	0/2996	1/2989	3.65
NCT03057977	Empagliflozin	Placebo	0.33 (0.01, 8.18)	0/1863	1/1863	3.65
NCT03315143	Sotagliflozin	Placebo	2.00 (0.18, 22.03)	2/5291	1/5286	6.49
NCT03594110	Empagliflozin	Placebo	5.00 (0.24, 104.14)	2/3304	0/3305	4.05
NCT03619213	Dapagliflozin	Placebo	5.00 (0.24, 104.14)	2/3126	0/3127	4.05
Overall (I-squared = 0.0%, p = 0.630)			1.20 (0.65, 2.22)	28/47841	17/41275	100.00

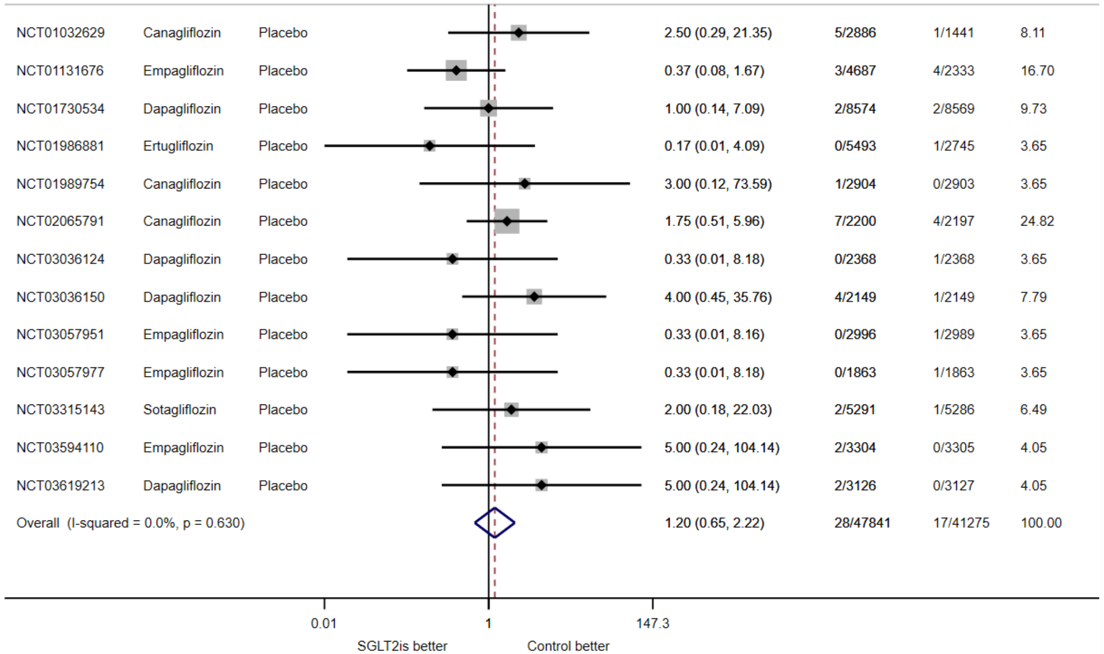

System 6-Eye Disorders-Figure S6 Meta-analysis of SGLT2is and Diabetic retinopathy

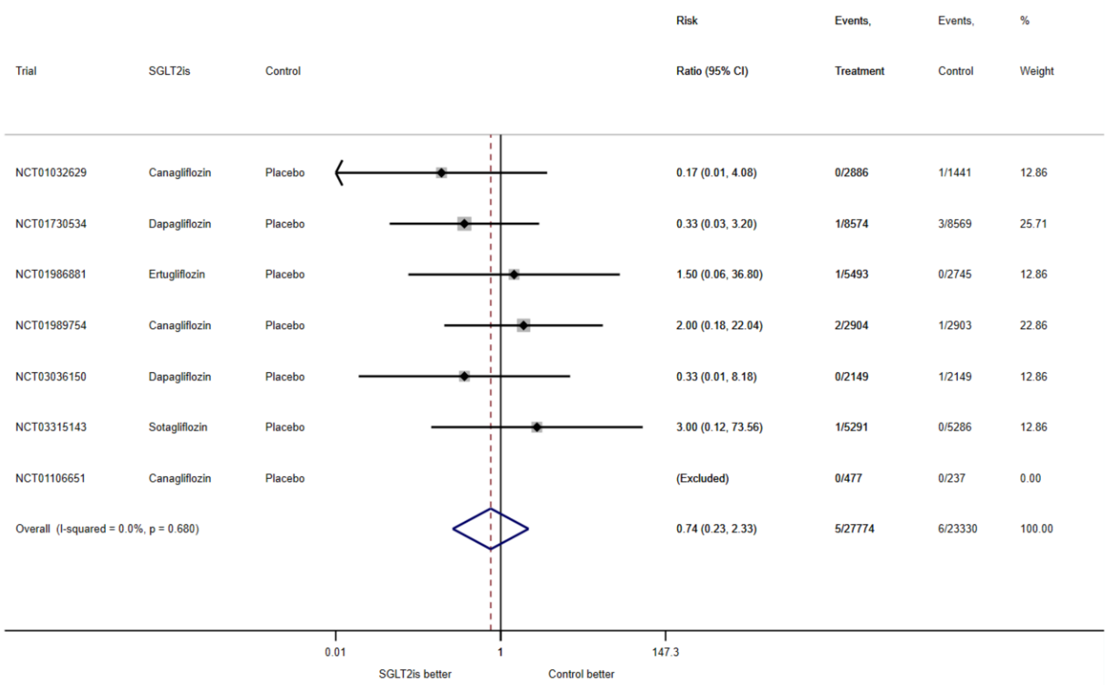

System 6-Eye Disorders-Figure S7 Meta-analysis of SGLT2is and Diplopia

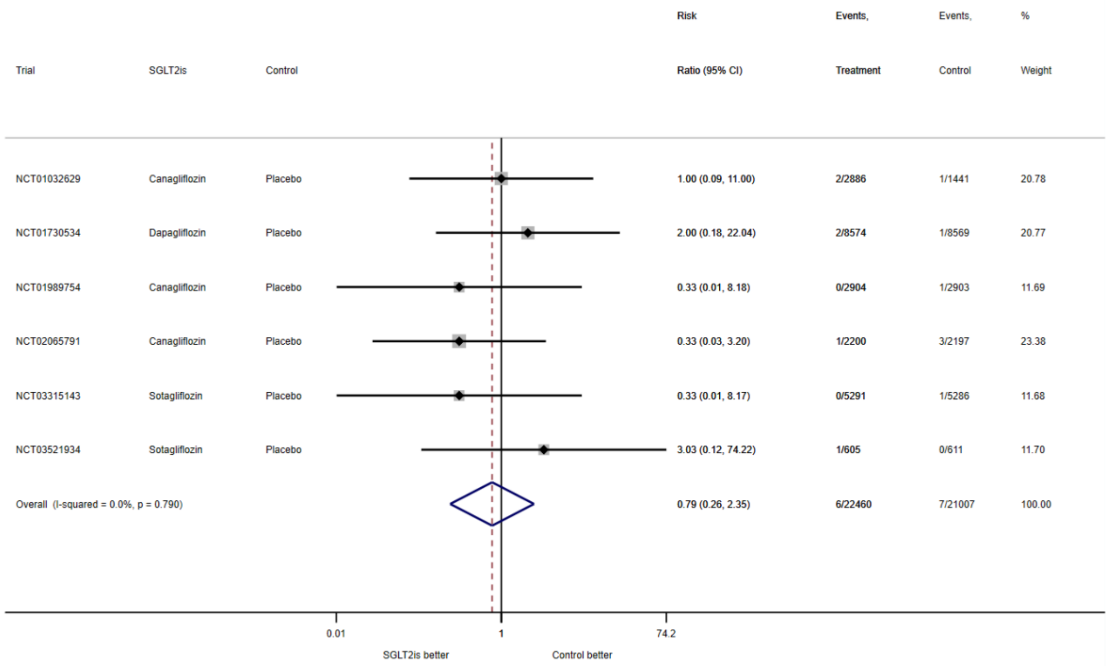

System 6-Eye Disorders-Figure S8 Meta-analysis of SGLT2is and Eye haemorrhage

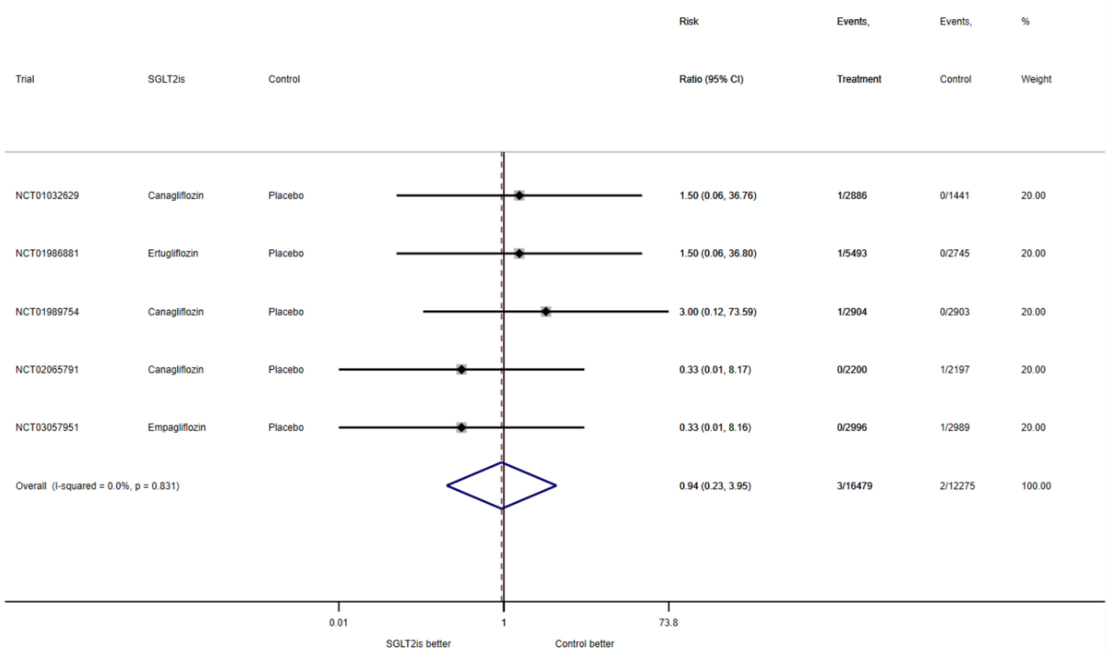

System 6-Eye Disorders-Figure S9 Meta-analysis of SGLT2is and Eyelid ptosis

Trial	SGLT2is	Control	Risk Ratio (95% CI)	Events, Treatment	Events, Control	% Weight
NCT00673231	Dapagliflozin	Placebo	0.97 (0.04, 23.77)	1/610	0/197	2.62
NCT01032629	Canagliflozin	Placebo	0.75 (0.13, 4.48)	3/2886	2/1441	8.38
NCT01131676	Empagliflozin	Placebo	2.49 (0.29, 21.29)	5/4687	1/2333	5.82
NCT01164501	Empagliflozin	Placebo	2.29 (0.09, 55.92)	1/419	0/319	2.62
NCT01730534	Dapagliflozin	Placebo	0.20 (0.01, 4.16)	0/8574	2/8569	2.91
NCT01986881	Ertugliflozin	Placebo	0.17 (0.02, 1.60)	1/5493	3/2745	5.23
NCT01989754	Canagliflozin	Placebo	0.33 (0.01, 8.18)	0/2904	1/2903	2.62
NCT02065791	Canagliflozin	Placebo	0.50 (0.05, 5.50)	1/2200	2/2197	4.65
NCT03036124	Dapagliflozin	Placebo	0.33 (0.03, 3.20)	1/2368	3/2368	5.24
NCT03036150	Dapagliflozin	Placebo	1.00 (0.06, 15.98)	1/2149	1/2149	3.49
NCT03057951	Empagliflozin	Placebo	0.80 (0.32, 2.02)	8/2996	10/2989	31.10
NCT03057977	Empagliflozin	Placebo	0.60 (0.14, 2.51)	3/1863	5/1863	13.11
NCT03242252	Sotagliflozin	Placebo	0.16 (0.01, 4.03)	0/527	1/260	2.62
NCT03315143	Sotagliflozin	Placebo	1.00 (0.06, 15.97)	1/5291	1/5286	3.49
NCT03594110	Empagliflozin	Placebo	0.33 (0.01, 8.18)	0/3304	1/3305	2.62
NCT03619213	Dapagliflozin	Placebo	1.00 (0.06, 15.99)	1/3126	1/3127	3.49
Overall (I-squared = 0.0%, p = 0.979)			0.66 (0.39, 1.10)	27/49397	34/42051	100.00

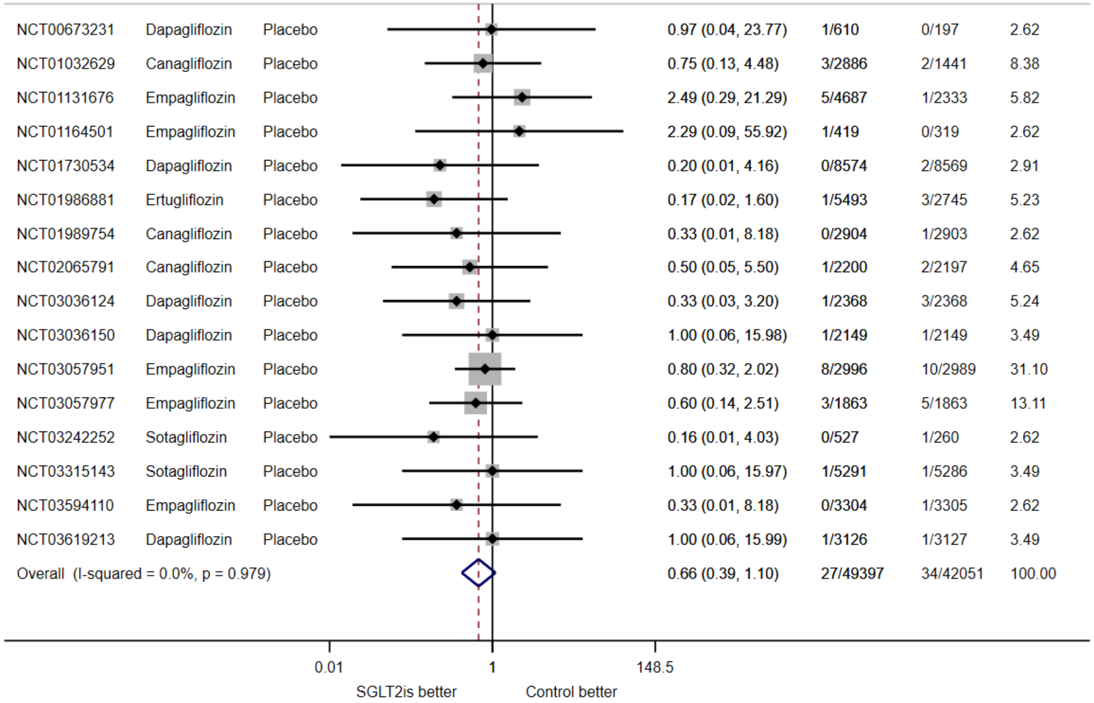

System 6-Eye Disorders-Figure S10 Meta-analysis of SGLT2is and Glaucoma

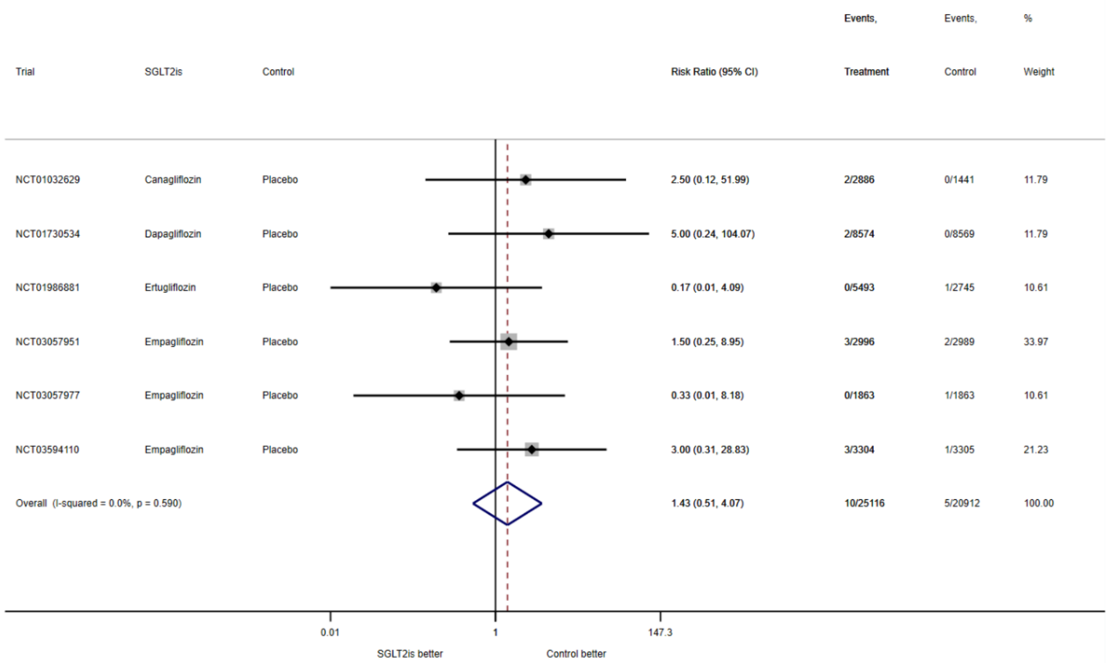

System 6-Eye Disorders-Figure S11 Meta-analysis of SGLT2is and Macular degeneration

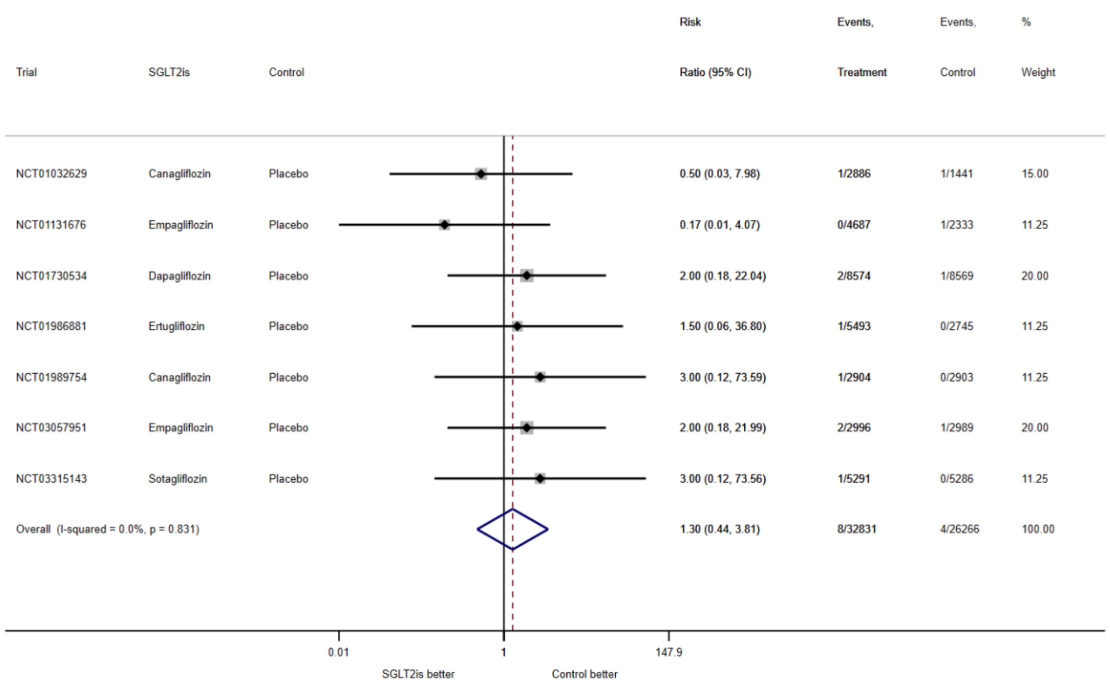

System 6-Eye Disorders-Figure S12 Meta-analysis of SGLT2is and Macular fibrosis

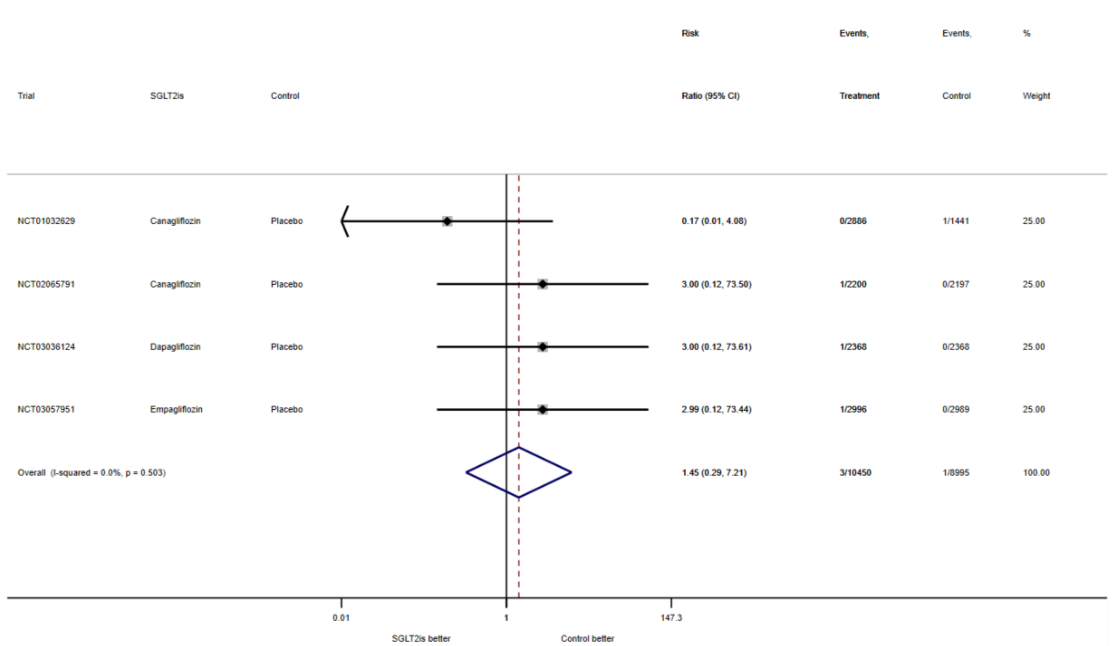

System 6-Eye Disorders-Figure S13 Meta-analysis of SGLT2is and Optic ischaemic neuropathy

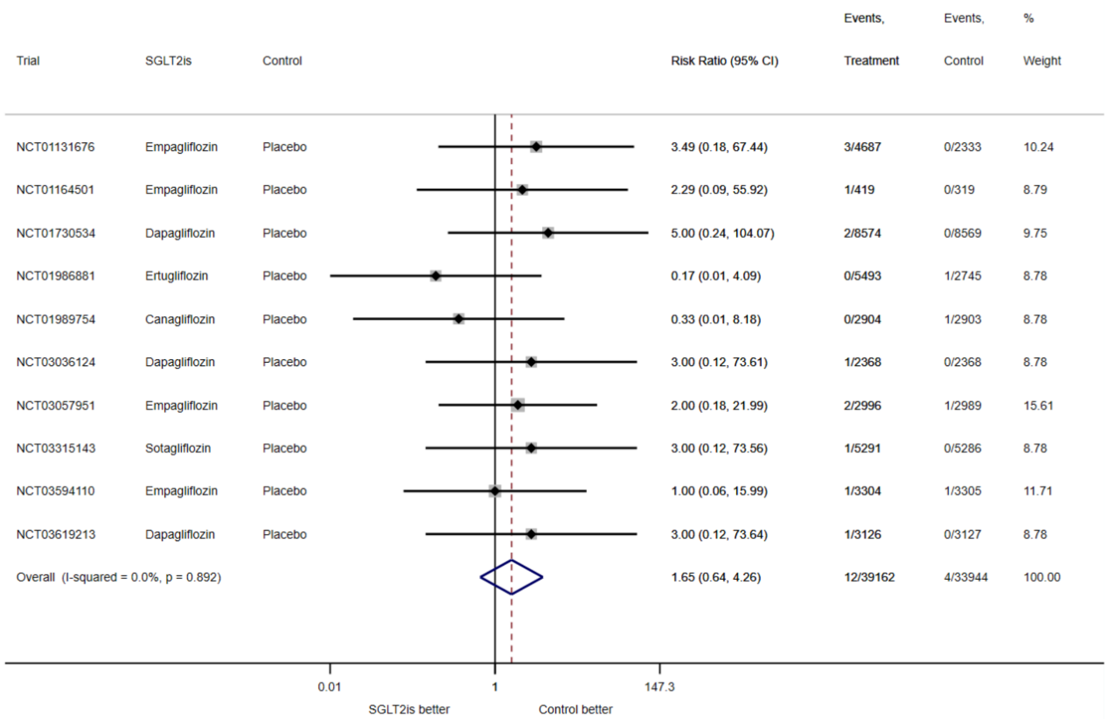

System 6-Eye Disorders-Figure S14 Meta-analysis of SGLT2is and Retinal artery occlusion

Trial	SGLT2is	Control	Risk Ratio (95% CI)	Events, Treatment	Events, Control	% Weight
NCT01032629	Canagliflozin	Placebo	0.10 (0.00, 2.08)	0/2886	2/1441	5.06
NCT01131676	Empagliflozin	Placebo	6.47 (0.36, 114.84)	6/4687	0/2333	5.64
NCT01730534	Dapagliflozin	Placebo	0.50 (0.09, 2.73)	2/8574	4/8569	16.20
NCT01986881	Ertugliflozin	Placebo	0.07 (0.00, 1.38)	0/5493	3/2745	5.32
NCT01989754	Canagliflozin	Placebo	3.00 (0.12, 73.59)	1/2904	0/2903	4.56
NCT02065791	Canagliflozin	Placebo	1.50 (0.25, 8.96)	3/2200	2/2197	14.60
NCT03036124	Dapagliflozin	Placebo	0.33 (0.01, 8.18)	0/2368	1/2368	4.56
NCT03036150	Dapagliflozin	Placebo	0.14 (0.01, 2.76)	0/2149	3/2149	5.32
NCT03057951	Empagliflozin	Placebo	0.50 (0.05, 5.50)	1/2996	2/2989	8.10
NCT03057977	Empagliflozin	Placebo	7.00 (0.36, 135.42)	3/1863	0/1863	5.32
NCT03315143	Sotagliflozin	Placebo	5.00 (0.24, 104.02)	2/5291	0/5286	5.06
NCT03594110	Empagliflozin	Placebo	1.00 (0.14, 7.10)	2/3304	2/3305	12.16
NCT03619213	Dapagliflozin	Placebo	2.00 (0.18, 22.05)	2/3126	1/3127	8.10
Overall (I-squared = 11.3%, p = 0.331)			0.88 (0.45, 1.75)	22/47841	20/41275	100.00

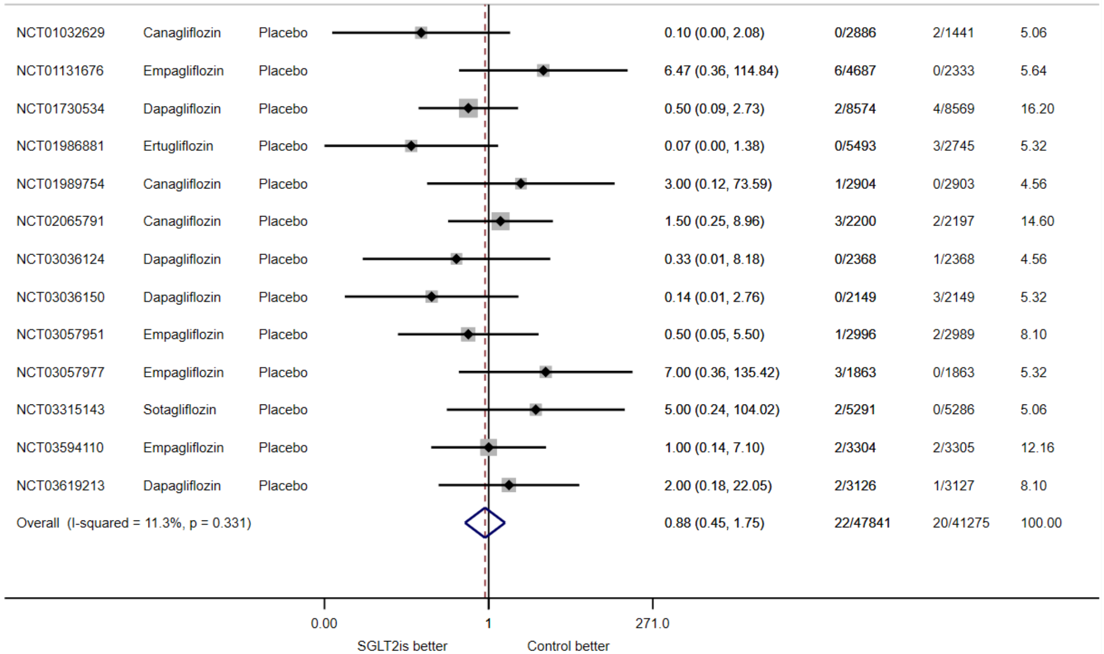

System 6-Eye Disorders-Figure S15 Meta-analysis of SGLT2is and Retinal detachment

Trial	SGLT2is	Control	Risk Ratio (95% CI)	Events, Treatment	Events, Control	% Weight
NCT01131676	Empagliflozin	Placebo	0.17 (0.01, 4.07)	0/4687	1/2333	14.40
NCT01730534	Dapagliflozin	Placebo	2.00 (0.18, 22.04)	2/8574	1/8569	25.60
NCT01986881	Ertugliflozin	Placebo	3.50 (0.18, 67.71)	3/5493	0/2745	16.80
NCT03036150	Dapagliflozin	Placebo	0.33 (0.01, 8.18)	0/2149	1/2149	14.40
NCT03315143	Sotagliflozin	Placebo	3.00 (0.12, 73.56)	1/5291	0/5286	14.40
NCT03594110	Empagliflozin	Placebo	0.33 (0.01, 8.18)	0/3304	1/3305	14.40
Overall (I-squared = 0.0%, p = 0.613)			0.97 (0.29, 3.27)	6/29498	4/24387	100.00

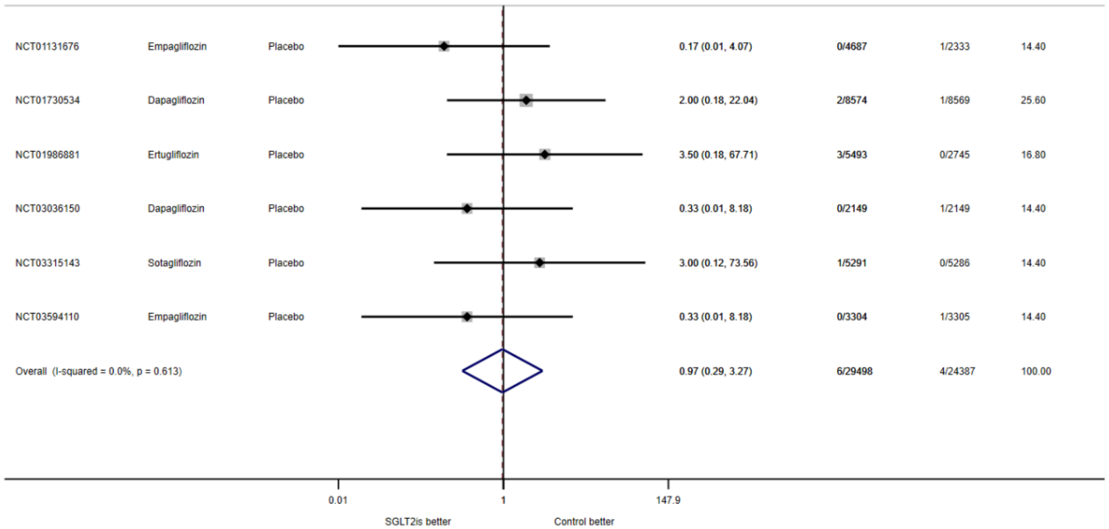

System 6-Eye Disorders-Figure S16 Meta-analysis of SGLT2is and Retinal haemorrhage

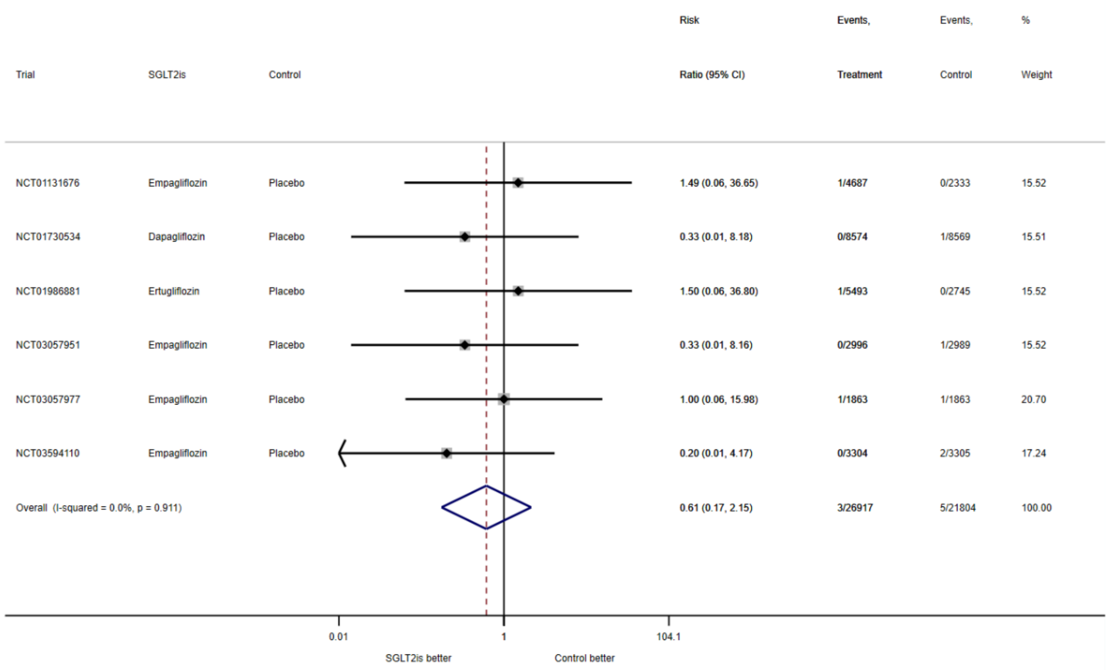

System 6-Eye Disorders-Figure S17 Meta-analysis of SGLT2is and Retinal tear

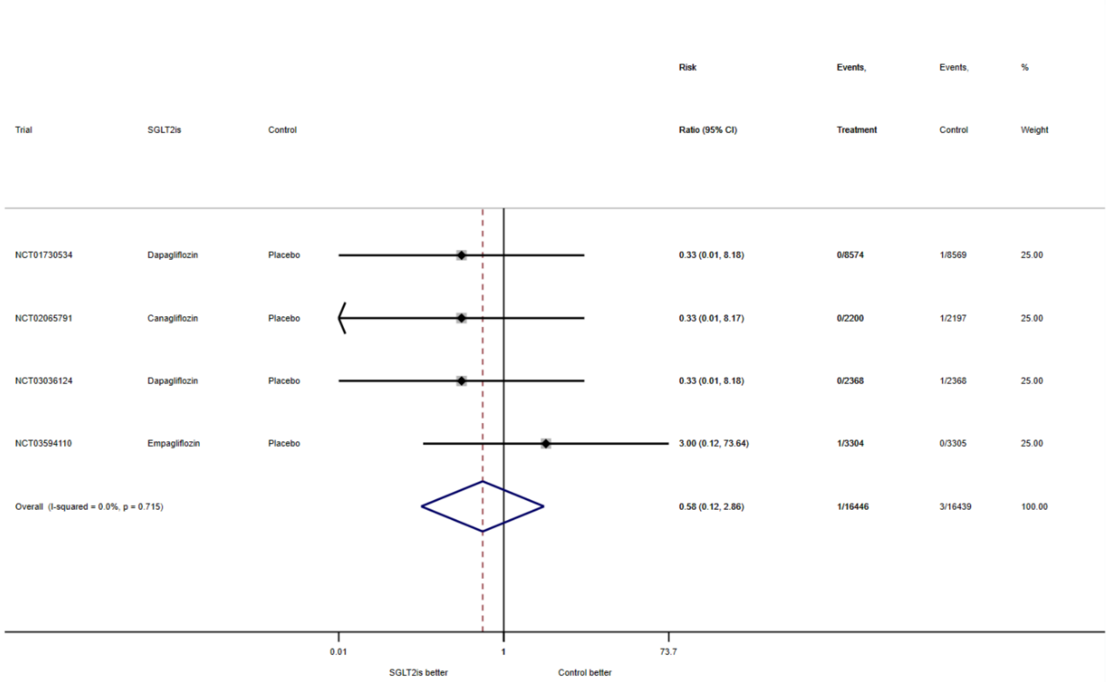

System 6-Eye Disorders-Figure S18 Meta-analysis of SGLT2is and Vision blurred

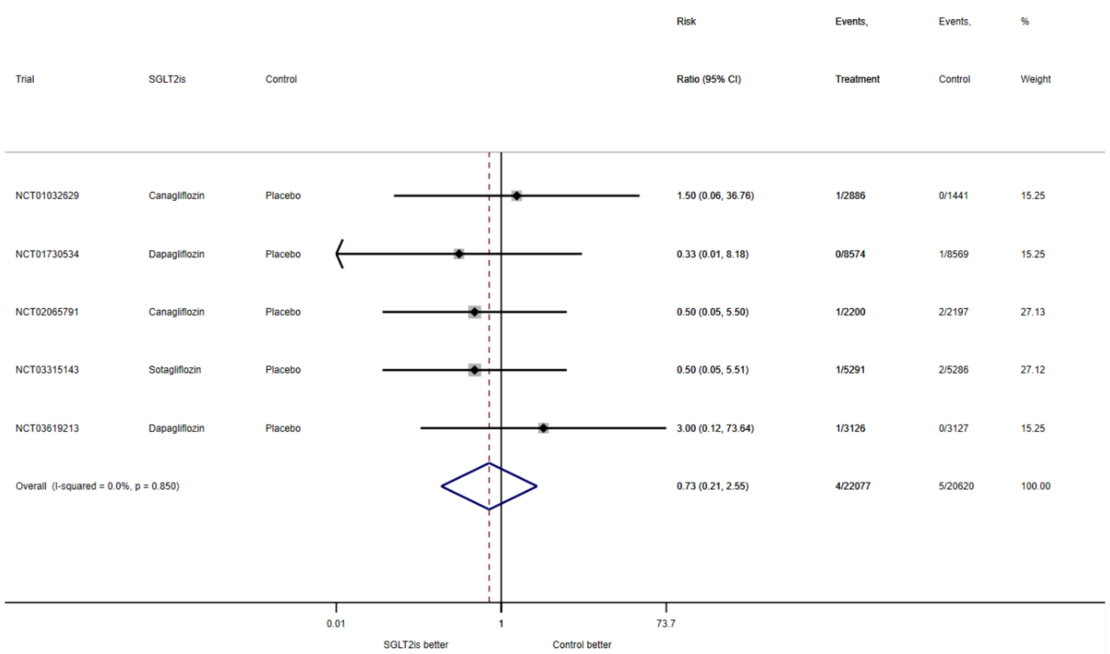

System 6-Eye Disorders-Figure S19 Meta-analysis of SGLT2is and Visual impairment

Trial	SGLT2is	Control	Risk Ratio (95% CI)	Events, Treatment	Events, Control	% Weight
NCT01032629	Canagliflozin	Placebo	3.50 (0.18, 67.64)	3/2886	0/1441	4.30
NCT01131676	Empagliflozin	Placebo	0.50 (0.12, 1.99)	4/4687	4/2333	19.66
NCT01730534	Dapagliflozin	Placebo	0.67 (0.19, 2.36)	4/8574	6/8569	23.58
NCT01986881	Ertugliflozin	Placebo	0.50 (0.03, 7.99)	1/5493	1/2745	4.91
NCT02065791	Canagliflozin	Placebo	3.99 (0.45, 35.71)	4/2200	1/2197	7.86
NCT03036124	Dapagliflozin	Placebo	0.20 (0.01, 4.16)	0/2368	2/2368	4.09
NCT03036150	Dapagliflozin	Placebo	0.50 (0.05, 5.51)	1/2149	2/2149	6.55
NCT03057951	Empagliflozin	Placebo	2.00 (0.18, 21.99)	2/2996	1/2989	6.55
NCT03057977	Empagliflozin	Placebo	0.33 (0.01, 8.18)	0/1863	1/1863	3.68
NCT03315143	Sotagliflozin	Placebo	1.00 (0.14, 7.09)	2/5291	2/5286	9.82
NCT03594110	Empagliflozin	Placebo	1.00 (0.06, 15.99)	1/3304	1/3305	4.91
NCT03619213	Dapagliflozin	Placebo	0.20 (0.01, 4.17)	0/3126	2/3127	4.09
Overall (I-squared = 0.0%, p = 0.852)			0.76 (0.41, 1.40)	22/44937	23/38372	100.00

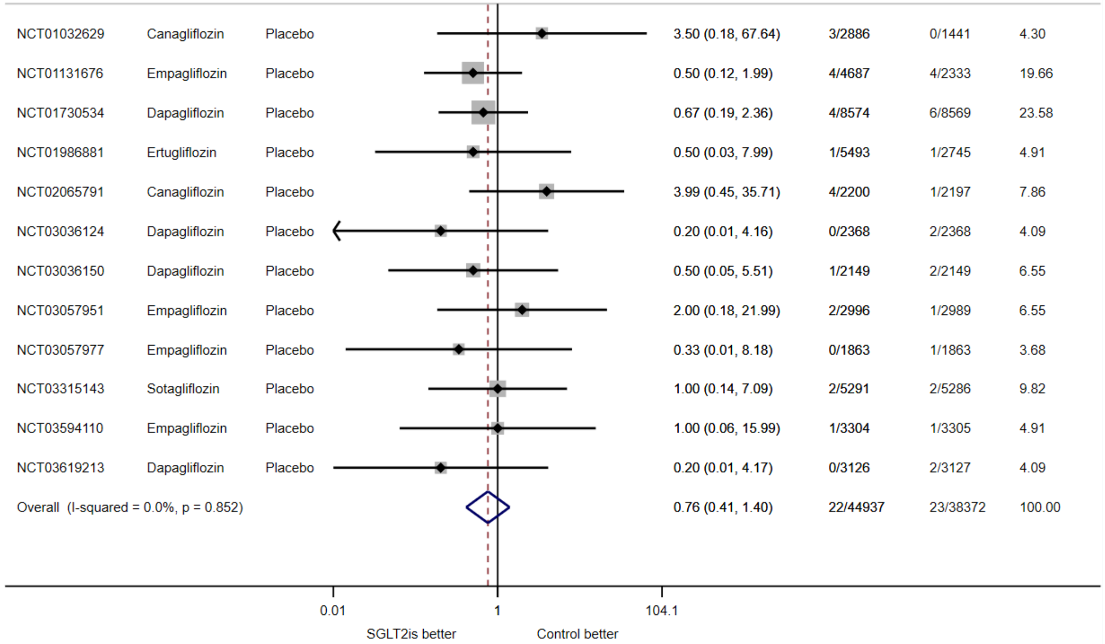

System 6-Eye Disorders-Figure S20 Meta-analysis of SGLT2is and Vitreous haemorrhage

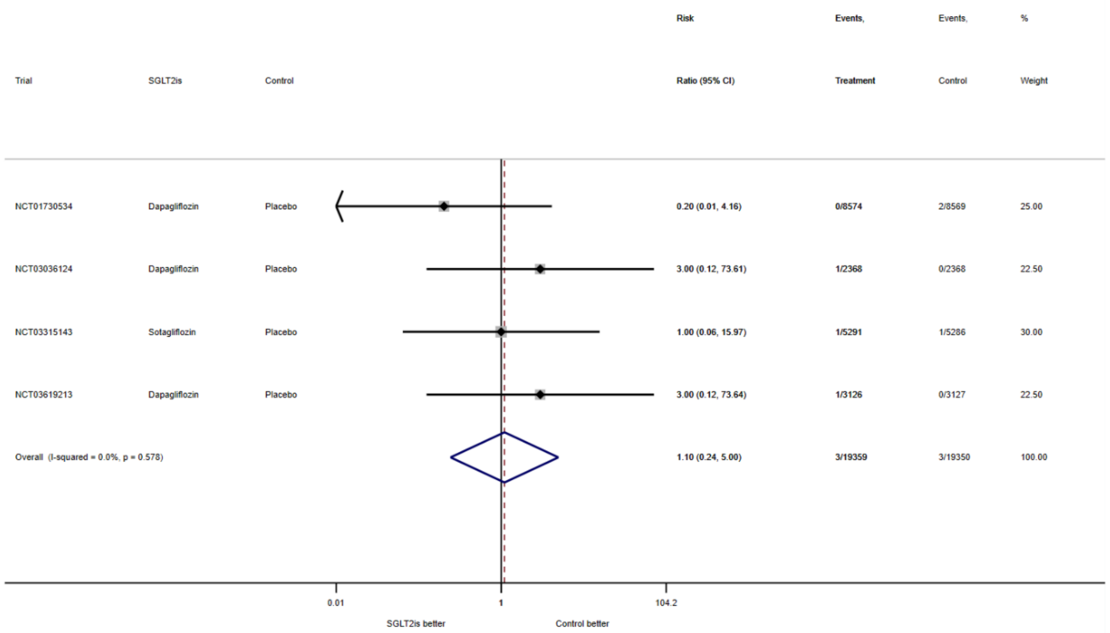

System 7-Gastrointestinal Disorders-Figure S1 Meta-analysis of SGLT2is and Abdominal adhesions

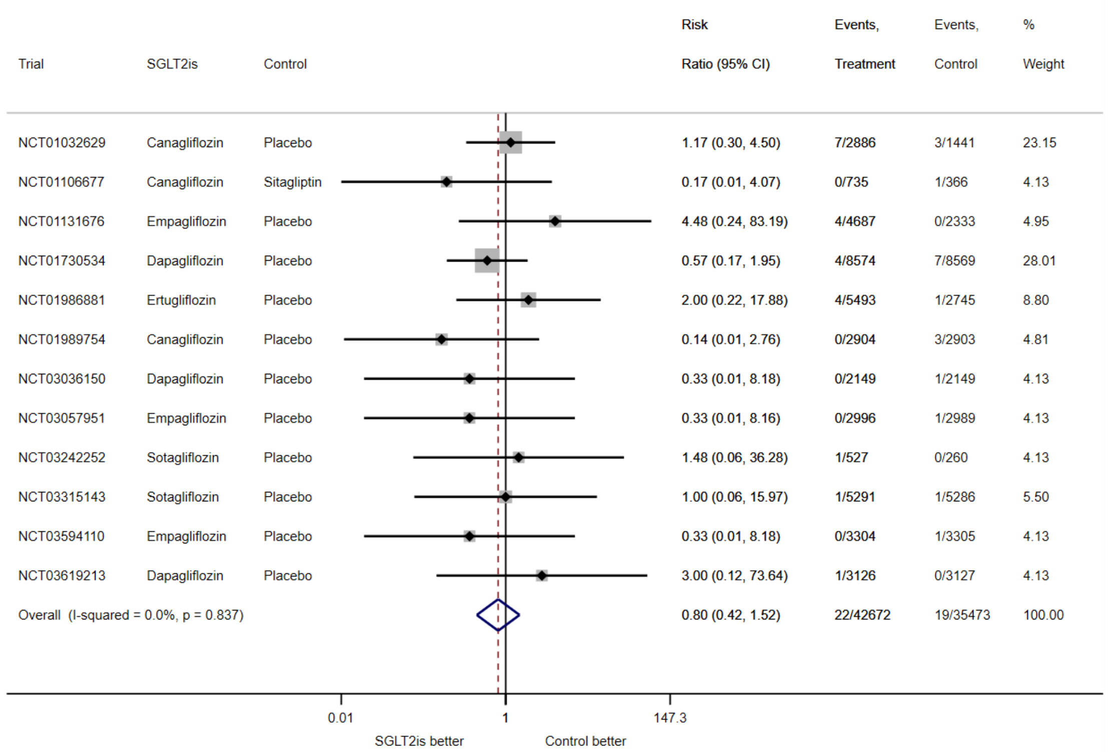

System 7-Gastrointestinal Disorders-Figure S2 Meta-analysis of SGLT2is and Abdominal hernia

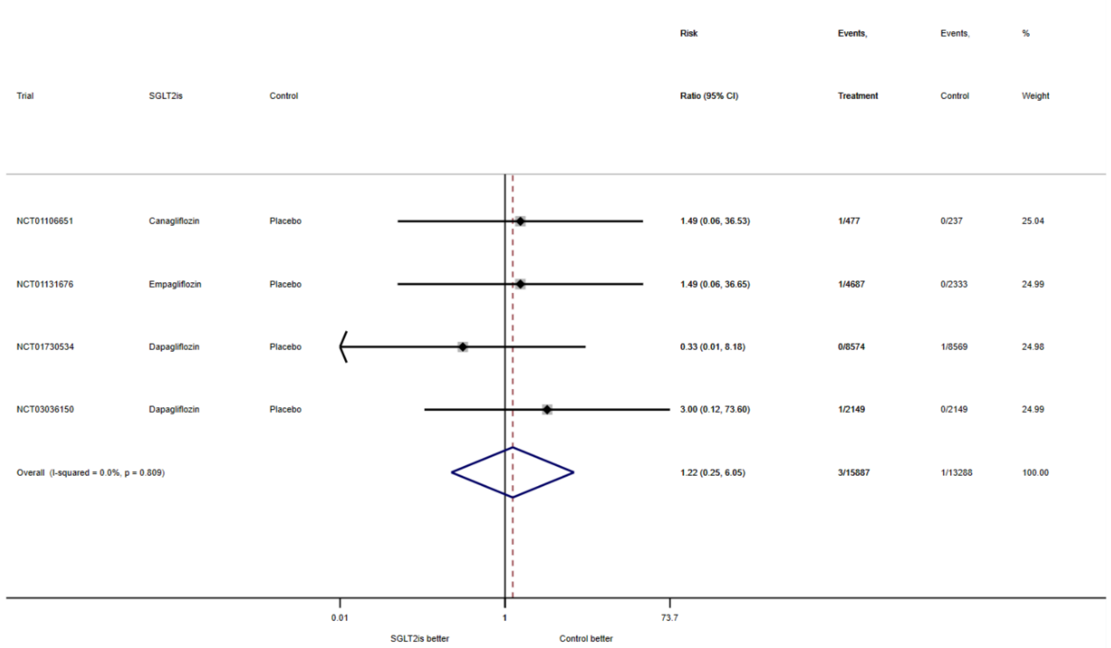

System 7-Gastrointestinal Disorders-Figure S3 Meta-analysis of SGLT2is and Abdominal hernia obstructive

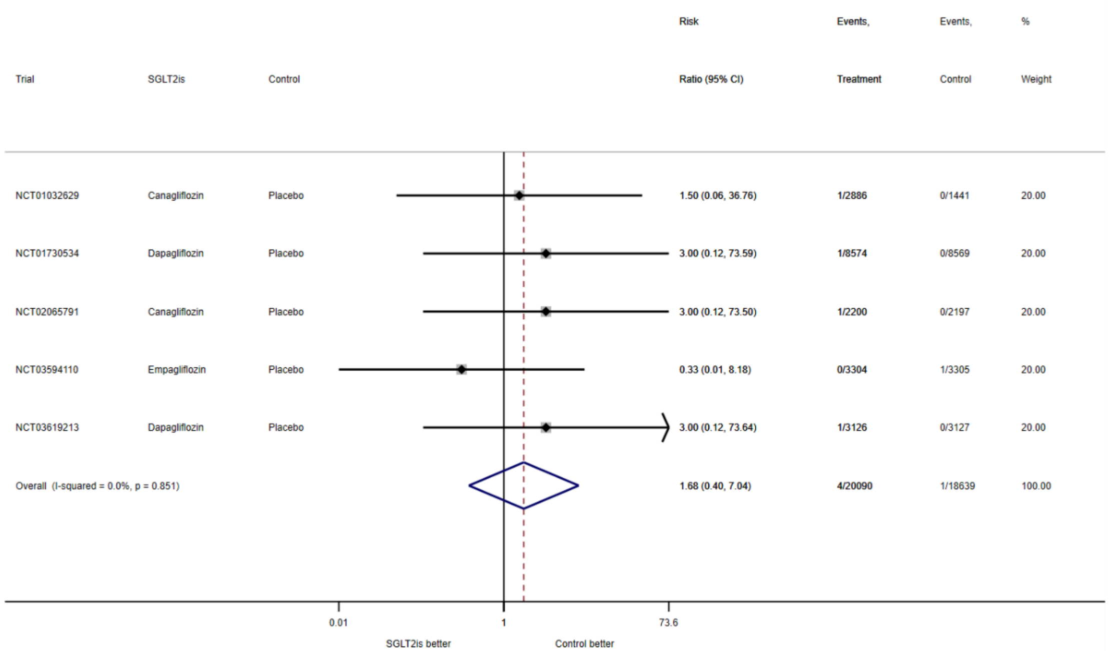

System 7-Gastrointestinal Disorders-Figure S4 Meta-analysis of SGLT2is and Abdominal incarcerated hernia

Trial	SGLT2is	Control	Risk Ratio (95% CI)	Events, Treatment	Events, Control	% Weight
NCT00528879	Dapagliflozin	Placebo	1.68 (0.08, 34.84)	2/409	0/137	1.57
NCT01032629	Canagliflozin	Placebo	1.00 (0.30, 3.31)	8/2886	4/1441	10.02
NCT01042977	Dapagliflozin	Placebo	3.01 (0.12, 73.61)	1/482	0/483	1.41
NCT01131676	Empagliflozin	Placebo	0.70 (0.22, 2.19)	7/4687	5/2333	10.95
NCT01164501	Empagliflozin	Placebo	2.29 (0.09, 55.92)	1/419	0/319	1.41
NCT01730534	Dapagliflozin	Placebo	1.44 (0.62, 3.38)	13/8574	9/8569	19.95
NCT01986881	Ertugliflozin	Placebo	2.00 (0.42, 9.41)	8/5493	2/2745	6.00
NCT01989754	Canagliflozin	Placebo	0.40 (0.08, 2.06)	2/2904	5/2903	5.36
NCT02065791	Canagliflozin	Placebo	0.20 (0.01, 4.16)	0/2200	2/2197	1.56
NCT03036124	Dapagliflozin	Placebo	1.33 (0.30, 5.95)	4/2368	3/2368	6.43
NCT03036150	Dapagliflozin	Placebo	0.67 (0.11, 3.99)	2/2149	3/2149	4.50
NCT03057951	Empagliflozin	Placebo	0.33 (0.03, 3.20)	1/2996	3/2989	2.81
NCT03057977	Empagliflozin	Placebo	1.00 (0.20, 4.95)	3/1863	3/1863	5.63
NCT03242252	Sotagliflozin	Placebo	0.16 (0.01, 4.03)	0/527	1/260	1.41
NCT03315143	Sotagliflozin	Placebo	0.67 (0.19, 2.36)	4/5291	6/5286	9.00
NCT03521934	Sotagliflozin	Placebo	0.14 (0.01, 2.79)	0/605	3/611	1.64
NCT03594110	Empagliflozin	Placebo	3.50 (0.73, 16.84)	7/3304	2/3305	5.83
NCT03619213	Dapagliflozin	Placebo	0.11 (0.01, 2.06)	0/3126	4/3127	1.69
NCT04157751	Empagliflozin	Placebo	3.05 (0.12, 74.43)	1/260	0/264	1.41
NCT04350593	Dapagliflozin	Placebo	3.01 (0.12, 73.86)	1/613	0/616	1.41
NCT01106677	Canagliflozin	Sitagliptin	(Excluded)	0/735	0/366	0.00
Overall (I-squared = 0.0%, p = 0.722)			0.98 (0.67, 1.43)	65/51891	55/44331	100.00

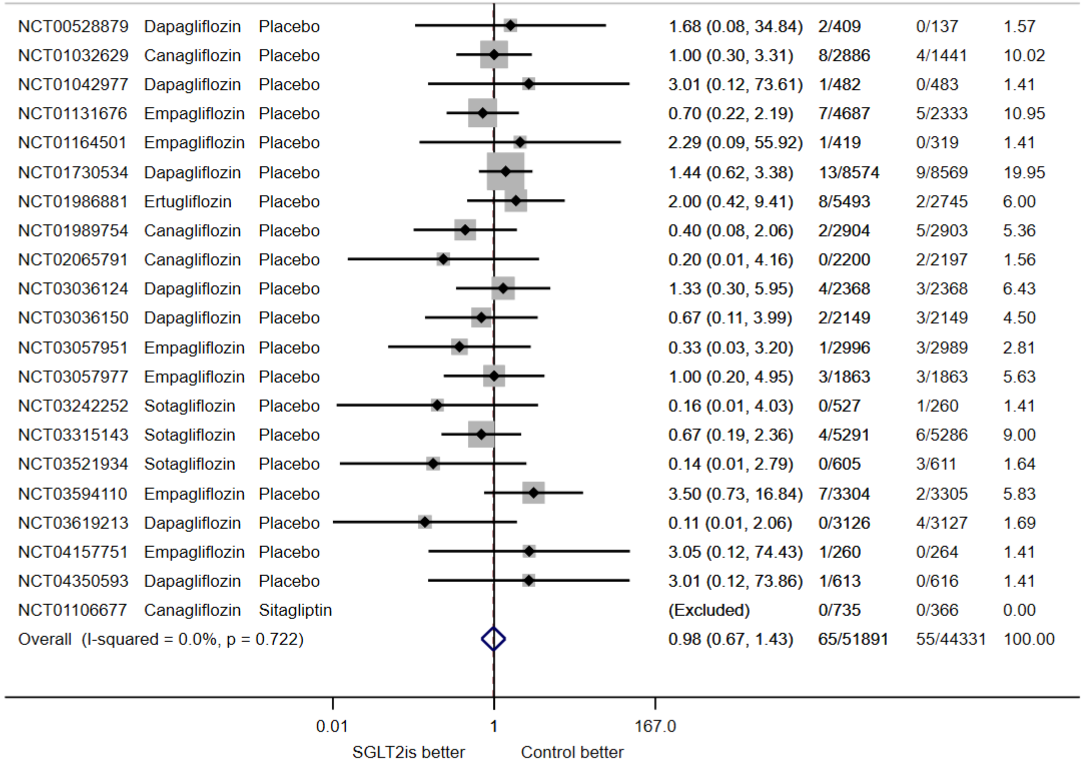

System 7-Gastrointestinal Disorders-Figure S5 Meta-analysis of SGLT2is and Abdominal pain

Trial	SGLT2is	Control	Risk Ratio (95% CI)	Events, Treatment	Events, Control	% Weight
NCT01131676	Empagliflozin	Placebo	1.49 (0.06, 36.65)	1/4687	0/2333	14.06
NCT01730534	Dapagliflozin	Placebo	0.33 (0.01, 8.18)	0/8574	1/8569	14.06
NCT01989754	Canagliflozin	Placebo	3.00 (0.12, 73.59)	1/2904	0/2903	14.06
NCT02065791	Canagliflozin	Placebo	3.00 (0.12, 73.50)	1/2200	0/2197	14.06
NCT03057951	Empagliflozin	Placebo	0.20 (0.01, 4.15)	0/2996	2/2989	15.63
NCT03315143	Sotagliflozin	Placebo	3.00 (0.12, 73.56)	1/5291	0/5286	14.06
NCT03619213	Dapagliflozin	Placebo	0.33 (0.01, 8.18)	0/3126	1/3127	14.06
Overall (I-squared = 0.0%, p = 0.757)			0.96 (0.29, 3.19)	4/29778	4/27404	100.00

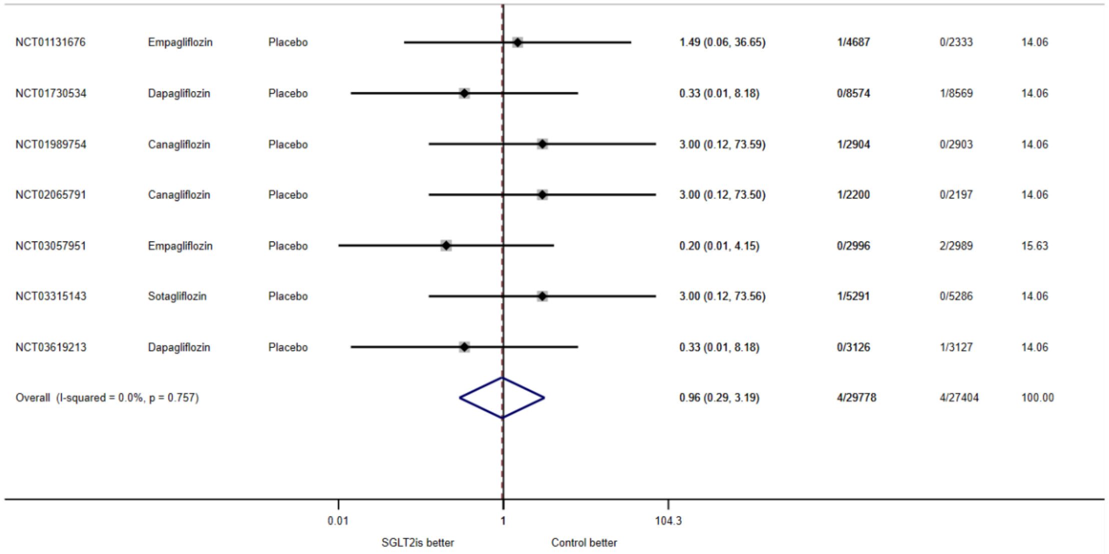

System 7-Gastrointestinal Disorders-Figure S6 Meta-analysis of SGLT2is and Abdominal pain lower

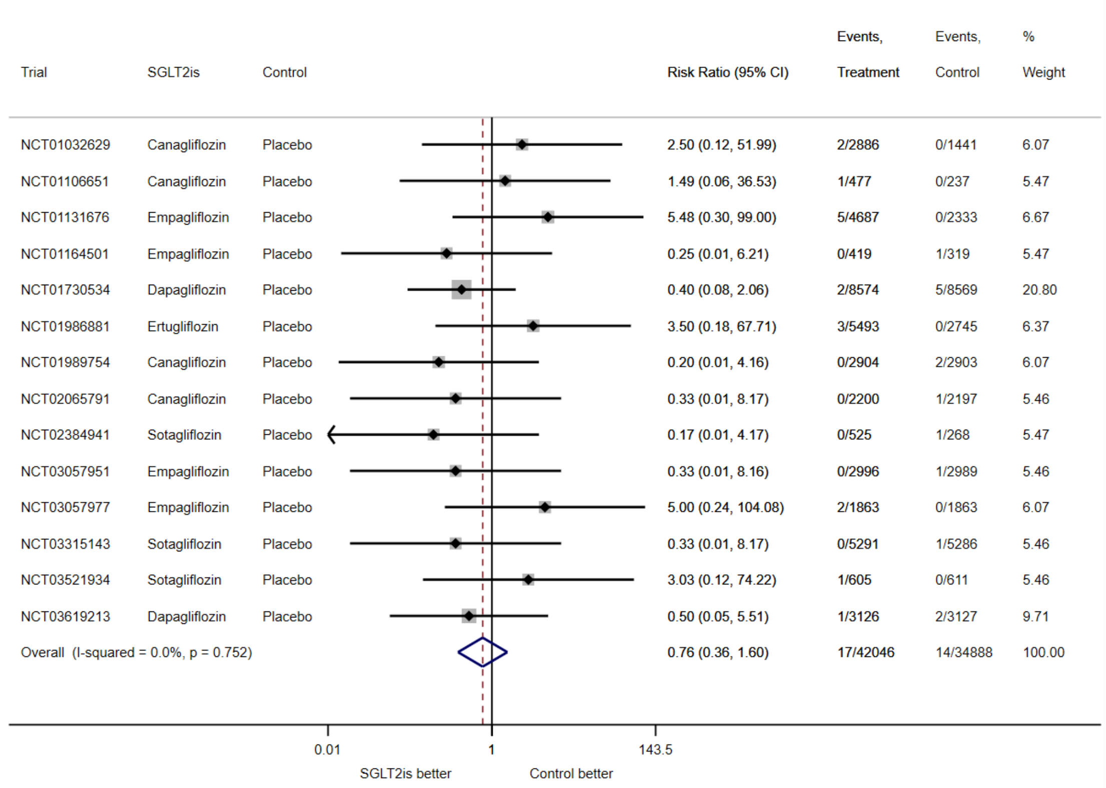

System 7-Gastrointestinal Disorders-Figure S7 Meta-analysis of SGLT2is and Abdominal pain upper

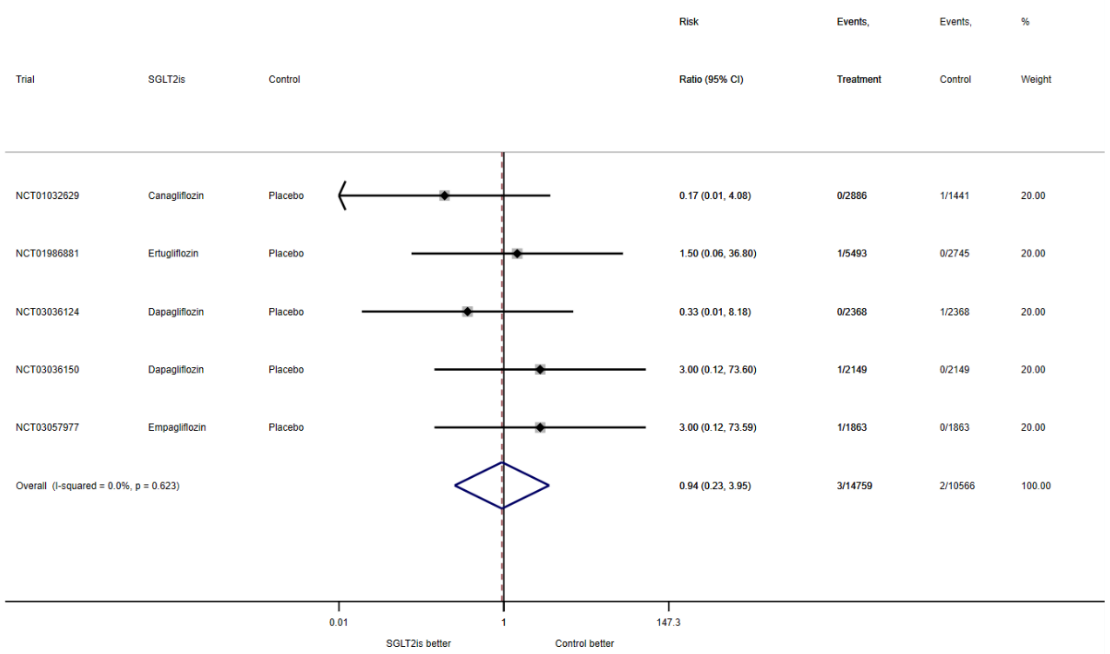

System 7-Gastrointestinal Disorders-Figure S8 Meta-analysis of SGLT2is and Abdominal wall haematoma

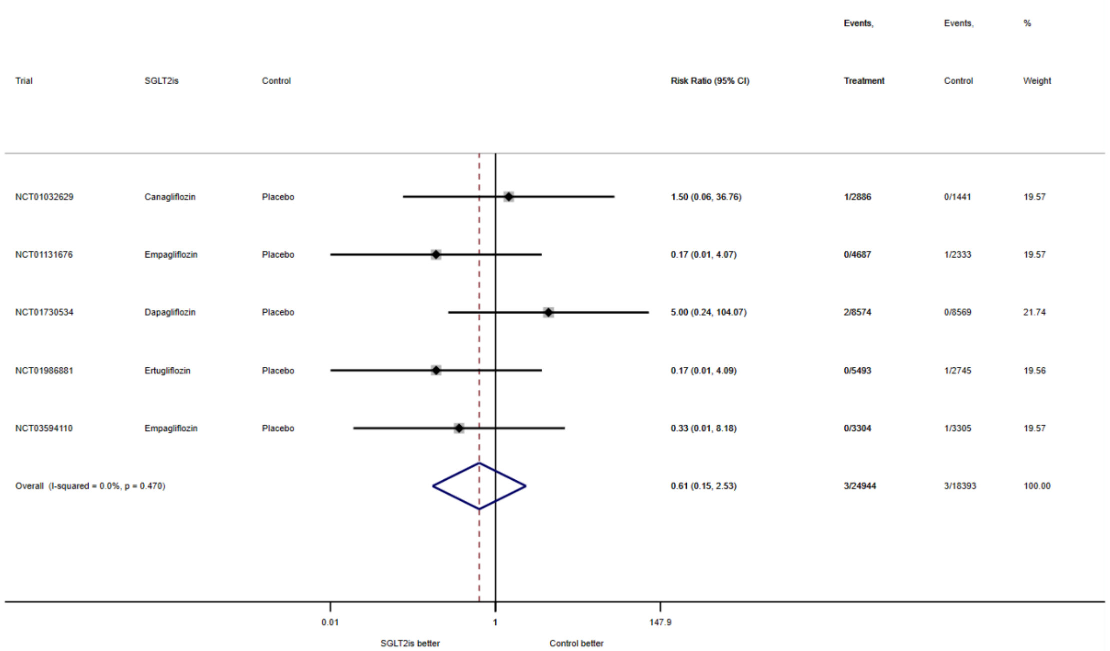

System 7-Gastrointestinal Disorders-Figure S9 Meta-analysis of SGLT2is and Anal fissure

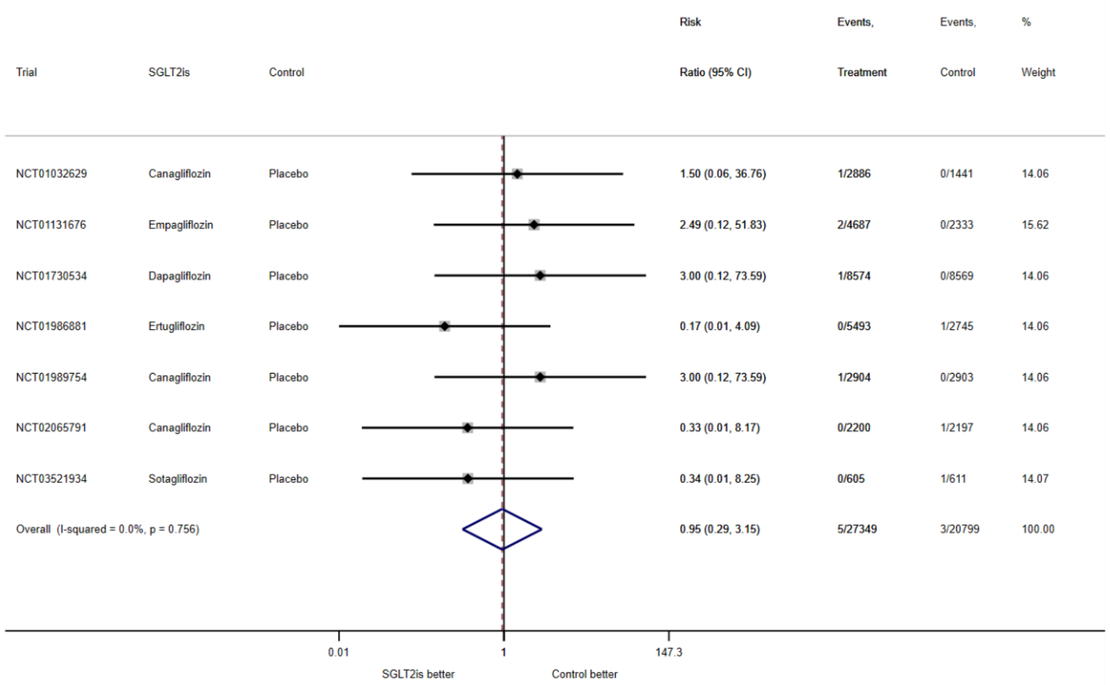

System 7-Gastrointestinal Disorders-Figure S10 Meta-analysis of SGLT2is and Anal fistula

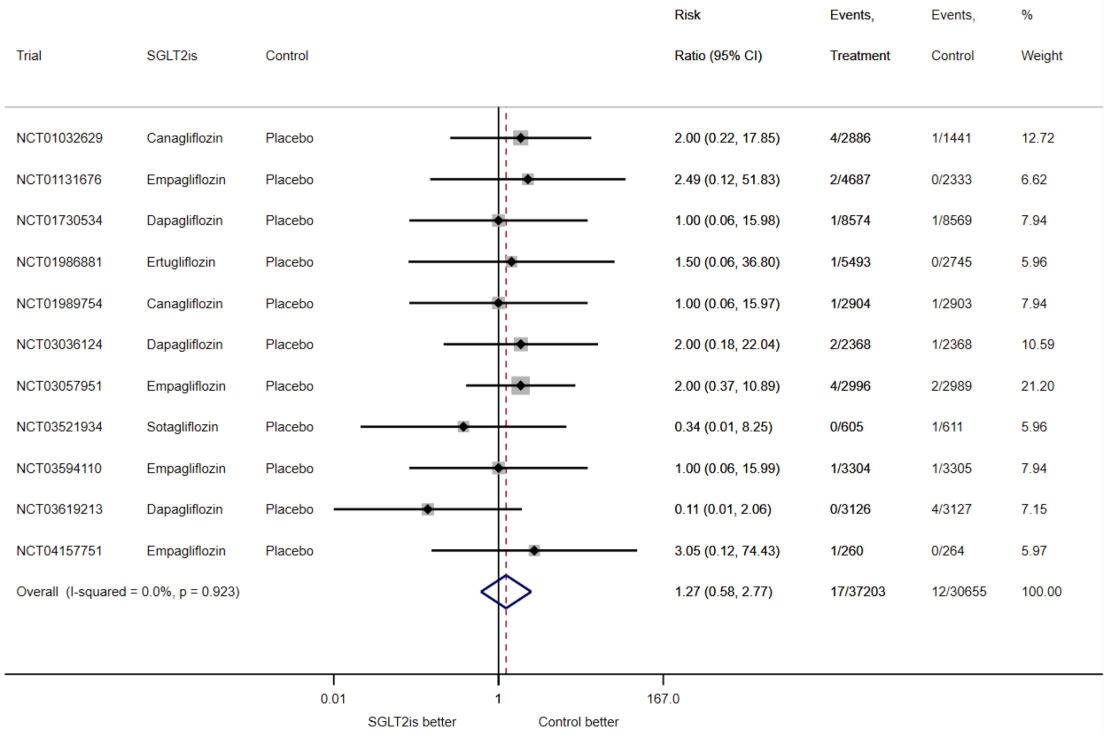

System 7-Gastrointestinal Disorders-Figure S11 Meta-analysis of SGLT2is and Ascites

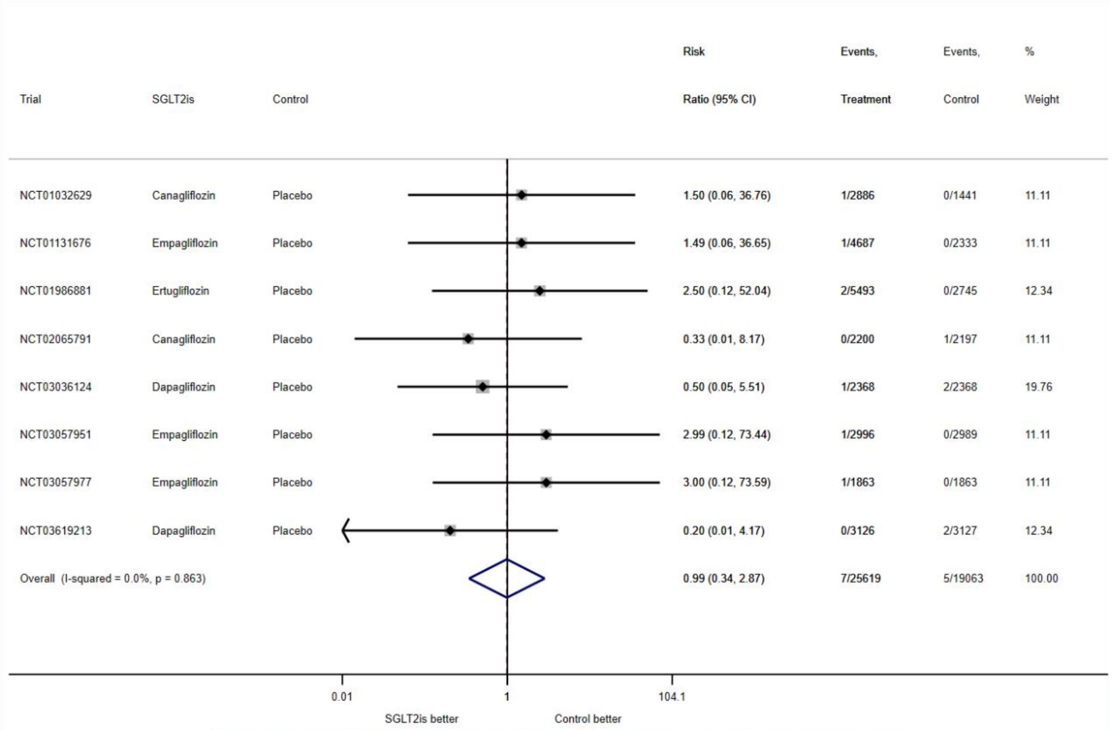

System 7-Gastrointestinal Disorders-Figure S12 Meta-analysis of SGLT2is and Chronic gastritis

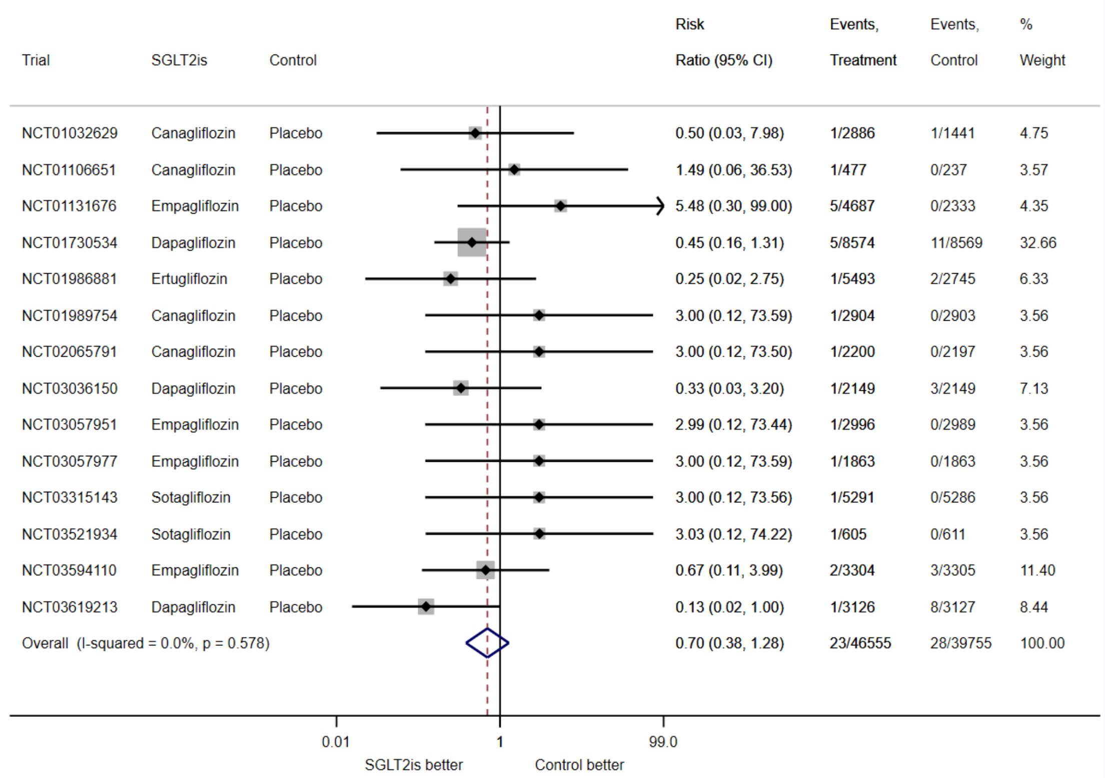

System 7-Gastrointestinal Disorders-Figure S13 Meta-analysis of SGLT2is and Colitis

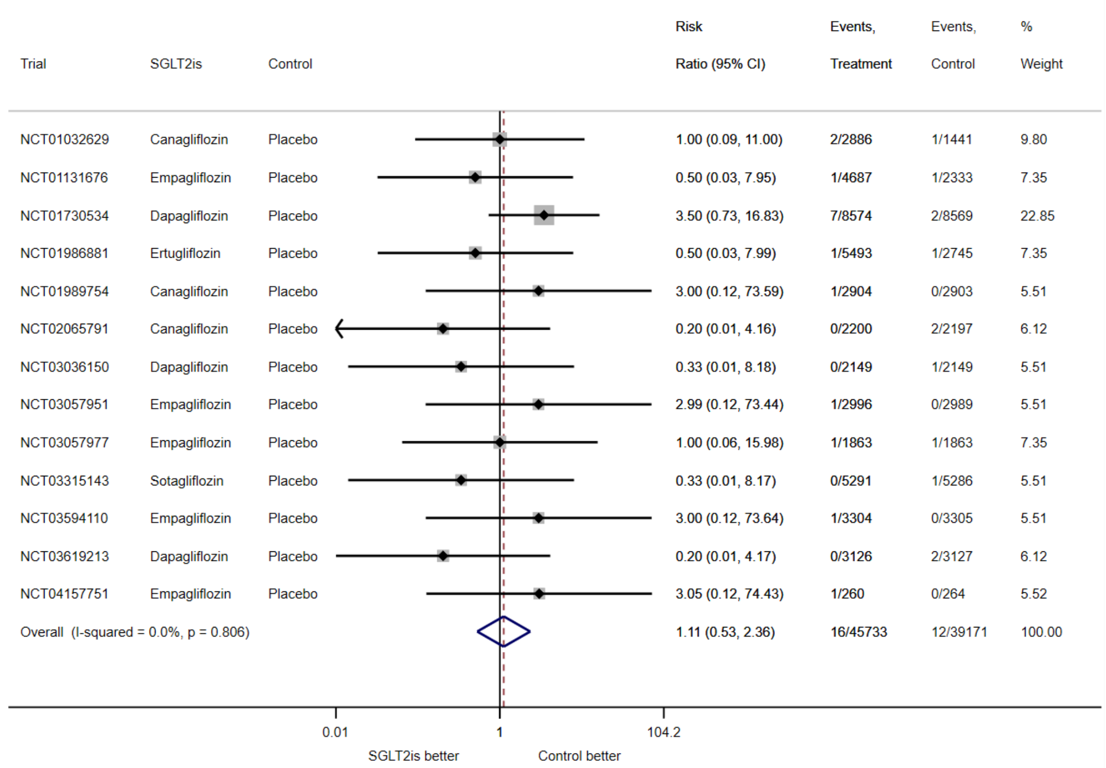

System 7-Gastrointestinal Disorders-Figure S14 Meta-analysis of SGLT2is and Colitis ischaemic

Trial	SGLT2is	Control	Risk Ratio (95% CI)	Events, Treatment	Events, Control	% Weight
NCT01032629	Canagliflozin	Placebo	2.50 (0.12, 51.99)	2/2886	0/1441	14.08
NCT01730534	Dapagliflozin	Placebo	0.50 (0.05, 5.51)	1/8574	2/8569	22.53
NCT01989754	Canagliflozin	Placebo	3.00 (0.12, 73.59)	1/2904	0/2903	12.67
NCT03057951	Empagliflozin	Placebo	0.33 (0.01, 8.16)	0/2996	1/2989	12.67
NCT03521934	Sotagliflozin	Placebo	3.03 (0.12, 74.22)	1/605	0/611	12.69
NCT03619213	Dapagliflozin	Placebo	0.33 (0.03, 3.20)	1/3126	3/3127	25.35
Overall (I-squared = 0.0%, p = 0.721)			0.85 (0.27, 2.65)	6/21091	6/19640	100.00

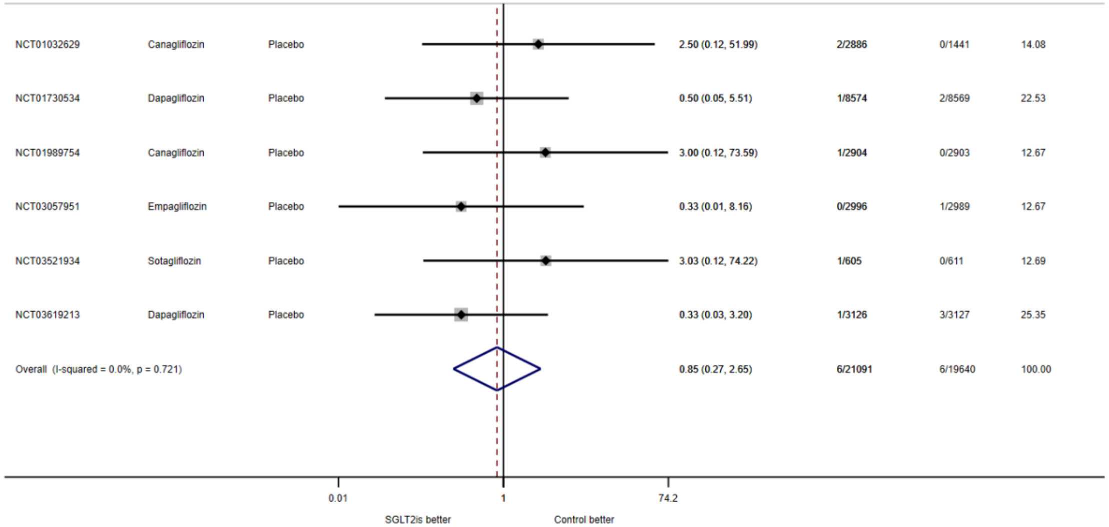

System 7-Gastrointestinal Disorders-Figure S15 Meta-analysis of SGLT2is and Colitis ulcerative

Trial	SGLT2is	Control	Risk Ratio (95% CI)	Events, Treatment	Events, Control	% Weight
NCT00673231	Dapagliflozin	Placebo	0.97 (0.04, 23.77)	1/610	0/197	2.73
NCT01032629	Canagliflozin	Placebo	0.50 (0.07, 3.54)	2/2886	2/1441	7.26
NCT01106677	Canagliflozin	Sitagliptin	1.50 (0.06, 36.63)	1/735	0/366	2.72
NCT01131676	Empagliflozin	Placebo	0.50 (0.12, 1.99)	4/4687	4/2333	14.53
NCT01164501	Empagliflozin	Placebo	2.29 (0.09, 55.92)	1/419	0/319	2.73
NCT01730534	Dapagliflozin	Placebo	4.33 (1.23, 15.19)	13/8574	3/8569	17.69
NCT01986881	Ertugliflozin	Placebo	5.50 (0.30, 99.39)	5/5493	0/2745	3.33
NCT01989754	Canagliflozin	Placebo	1.00 (0.06, 15.97)	1/2904	1/2903	3.63
NCT02065791	Canagliflozin	Placebo	0.20 (0.01, 4.16)	0/2200	2/2197	3.02
NCT02384941	Sotagliflozin	Placebo	0.17 (0.01, 4.17)	0/525	1/268	2.73
NCT03036124	Dapagliflozin	Placebo	1.00 (0.06, 15.98)	1/2368	1/2368	3.63
NCT03036150	Dapagliflozin	Placebo	0.50 (0.05, 5.51)	1/2149	2/2149	4.84
NCT03057951	Empagliflozin	Placebo	1.00 (0.06, 15.94)	1/2996	1/2989	3.63
NCT03057977	Empagliflozin	Placebo	3.00 (0.31, 28.81)	3/1863	1/1863	5.45
NCT03315143	Sotagliflozin	Placebo	3.00 (0.31, 28.80)	3/5291	1/5286	5.44
NCT03521934	Sotagliflozin	Placebo	1.01 (0.06, 16.11)	1/605	1/611	3.63
NCT03594110	Empagliflozin	Placebo	0.50 (0.05, 5.51)	1/3304	2/3305	4.84
NCT03619213	Dapagliflozin	Placebo	0.33 (0.03, 3.20)	1/3126	3/3127	5.44
NCT04157751	Empagliflozin	Placebo	3.05 (0.12, 74.43)	1/260	0/264	2.73
Overall (I-squared = 0.0%, p = 0.715)			1.13 (0.67, 1.92)	41/50995	25/43300	100.00

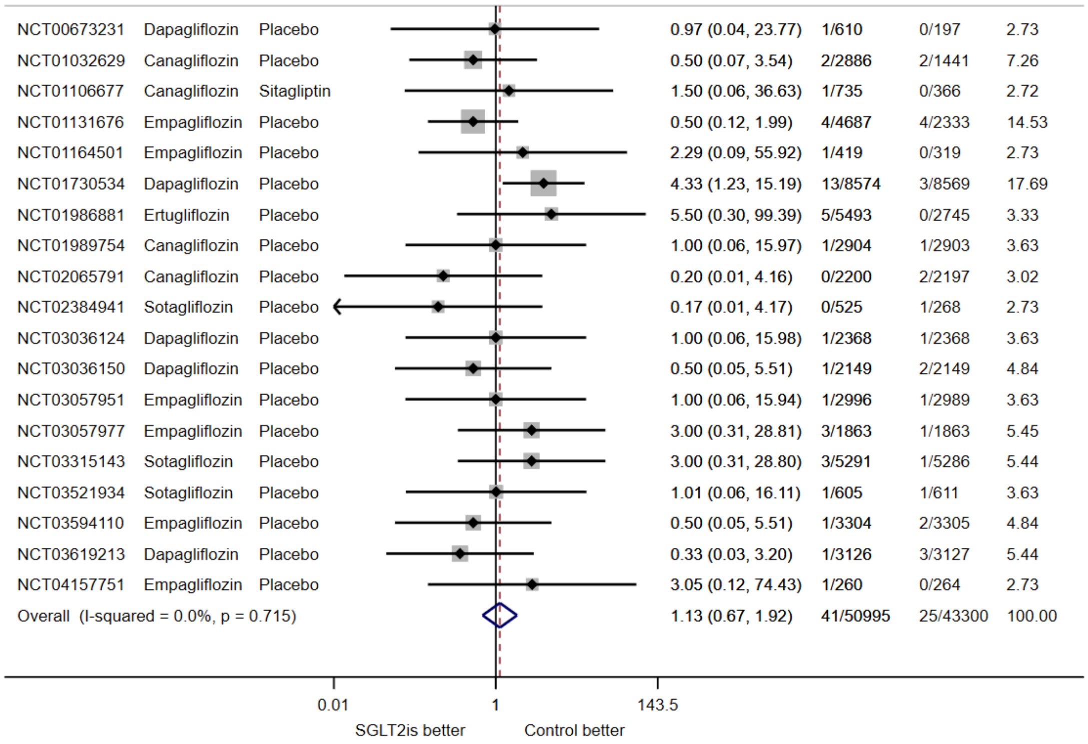

System 7-Gastrointestinal Disorders-Figure S16 Meta-analysis of SGLT2is and Constipation

Trial	SGLT2is	Control	Risk Ratio (95% CI)	Events, Treatment	Events, Control	% Weight
NCT00528879	Dapagliflozin	Placebo	1.01 (0.04, 24.64)	1/409	0/137	14.10
NCT01131676	Empagliflozin	Placebo	1.49 (0.06, 36.65)	1/4687	0/2333	14.06
NCT01730534	Dapagliflozin	Placebo	0.20 (0.01, 4.16)	0/8574	2/8569	15.62
NCT01986881	Ertugliflozin	Placebo	1.50 (0.06, 36.80)	1/5493	0/2745	14.06
NCT02065791	Canagliflozin	Placebo	0.33 (0.01, 8.17)	0/2200	1/2197	14.06
NCT03036150	Dapagliflozin	Placebo	3.00 (0.12, 73.60)	1/2149	0/2149	14.06
NCT03315143	Sotagliflozin	Placebo	3.00 (0.12, 73.56)	1/5291	0/5286	14.05
Overall (I-squared = 0.0%, p = 0.862)			1.02 (0.31, 3.38)	5/28803	3/23416	100.00

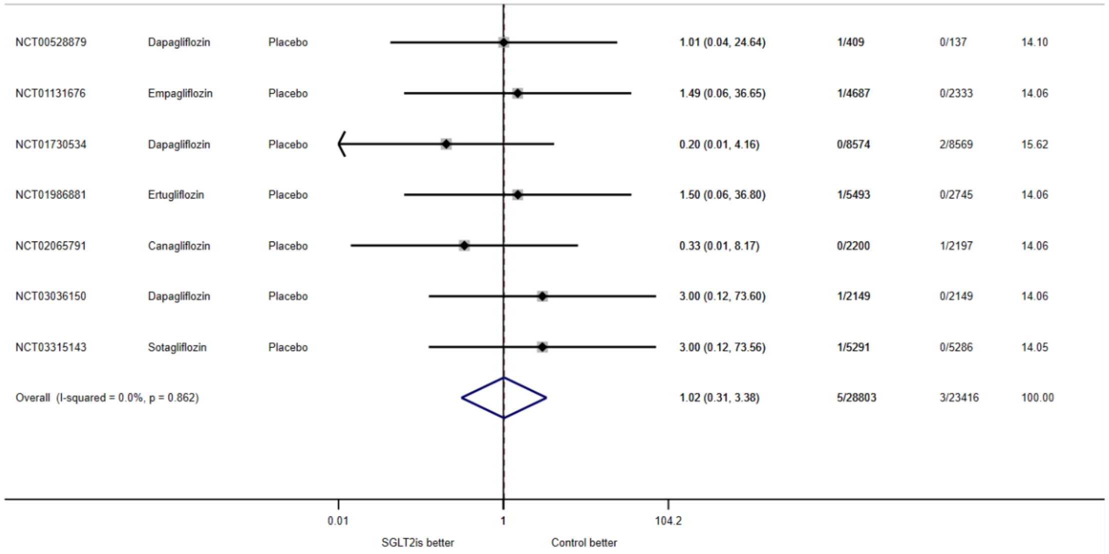

System 7-Gastrointestinal Disorders-Figure S17 Meta-analysis of SGLT2is and Diabetic gastroparesis

Trial	SGLT2is	Control	Risk Ratio (95% CI)	Events, Treatment	Events, Control	% Weight
NCT01031680	Dapagliflozin	Placebo	3.01 (0.12, 73.77)	1/460	0/462	1.42
NCT01032629	Canagliflozin	Placebo	0.67 (0.15, 2.97)	4/2886	3/1441	6.48
NCT01131676	Empagliflozin	Placebo	0.83 (0.30, 2.28)	10/4687	6/2333	14.19
NCT01164501	Empagliflozin	Placebo	2.29 (0.09, 55.92)	1/419	0/319	1.42
NCT01730534	Dapagliflozin	Placebo	0.91 (0.39, 2.14)	10/8574	11/8569	19.79
NCT01986881	Ertugliflozin	Placebo	3.50 (0.18, 67.71)	3/5493	0/2745	1.65
NCT01989754	Canagliflozin	Placebo	0.25 (0.03, 2.23)	1/2904	4/2903	3.02
NCT02065791	Canagliflozin	Placebo	1.50 (0.25, 8.96)	3/2200	2/2197	4.53
NCT03036124	Dapagliflozin	Placebo	1.00 (0.25, 3.99)	4/2368	4/2368	7.56
NCT03036150	Dapagliflozin	Placebo	2.00 (0.37, 10.91)	4/2149	2/2149	5.04
NCT03057951	Empagliflozin	Placebo	1.00 (0.25, 3.99)	4/2996	4/2989	7.56
NCT03057977	Empagliflozin	Placebo	1.00 (0.14, 7.09)	2/1863	2/1863	3.78
NCT03315143	Sotagliflozin	Placebo	0.80 (0.21, 2.97)	4/5291	5/5286	8.39
NCT03521934	Sotagliflozin	Placebo	0.25 (0.03, 2.25)	1/605	4/611	3.03
NCT03594110	Empagliflozin	Placebo	1.00 (0.20, 4.95)	3/3304	3/3305	5.67
NCT03619213	Dapagliflozin	Placebo	0.75 (0.17, 3.35)	3/3126	4/3127	6.48
Overall (I-squared = 0.0%, p = 0.983)			0.91 (0.62, 1.34)	58/49325	54/42667	100.00

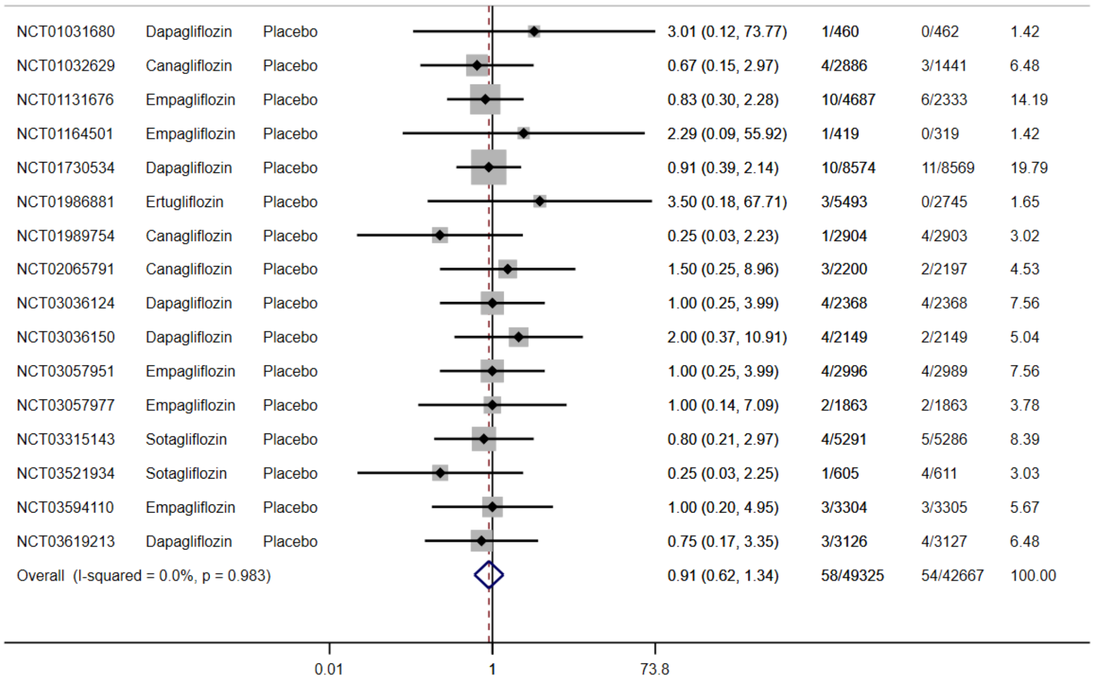

System 7-Gastrointestinal Disorders-Figure S18 Meta-analysis of SGLT2is and Diarrhoea

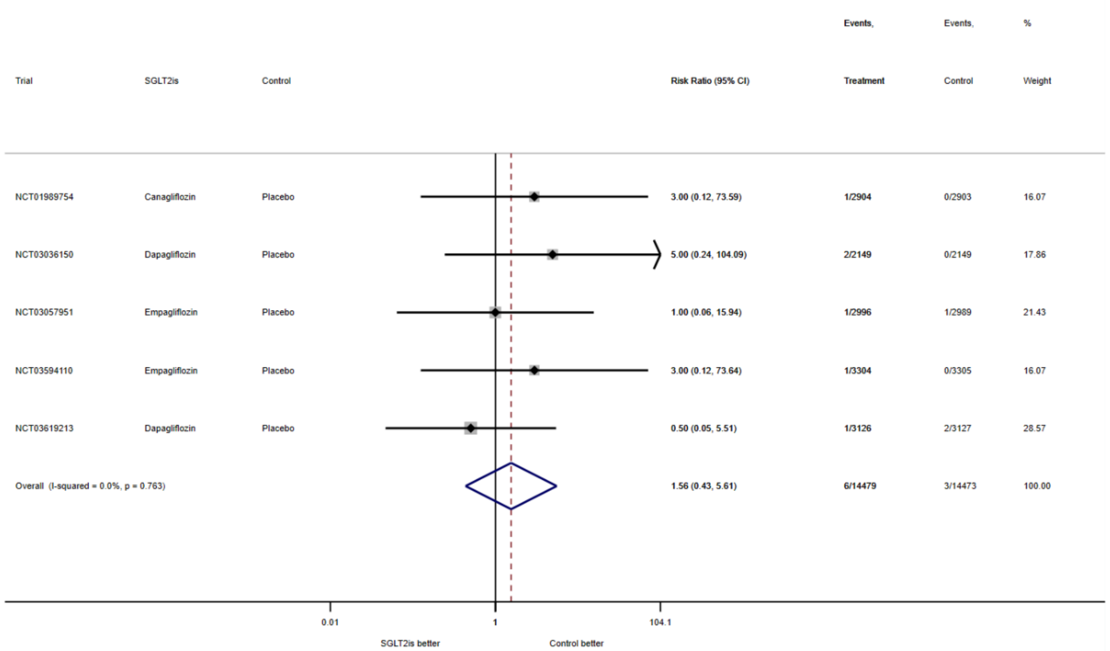

System 7-Gastrointestinal Disorders-Figure S19 Meta-analysis of SGLT2is and Diverticular perforation

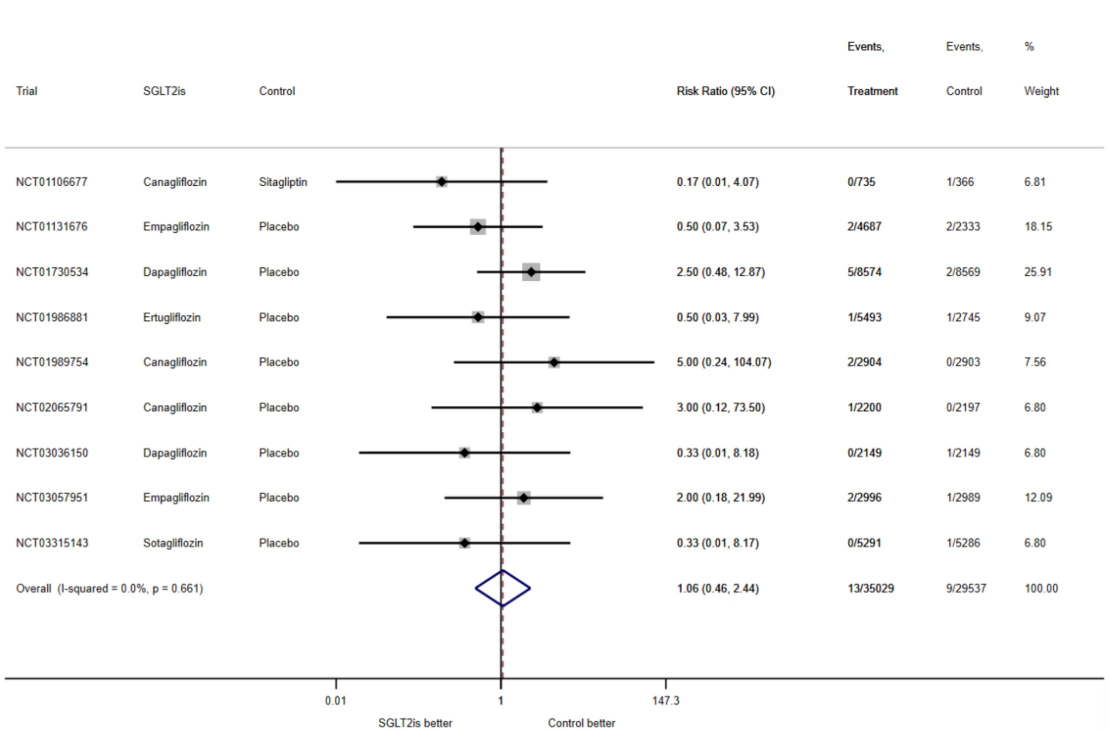

System 7-Gastrointestinal Disorders-Figure S20 Meta-analysis of SGLT2is and Diverticulum

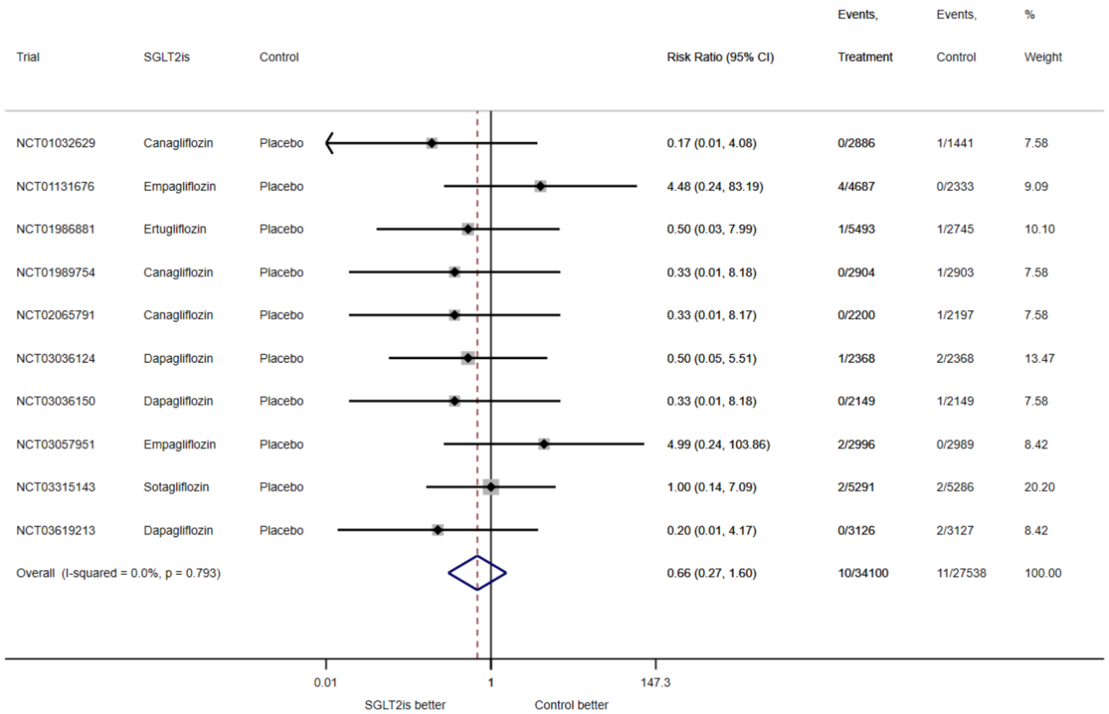

System 7-Gastrointestinal Disorders-Figure S21 Meta-analysis of SGLT2is and Diverticulum intestinal

Trial	SGLT2is	Control	Risk Ratio (95% CI)	Events, Treatment	Events, Control	% Weight
NCT01032629	Canagliflozin	Placebo	2.50 (0.12, 51.99)	2/2886	0/1441	9.17
NCT01730534	Dapagliflozin	Placebo	2.00 (0.18, 22.04)	2/8574	1/8569	14.68
NCT01986881	Ertugliflozin	Placebo	0.17 (0.01, 4.09)	0/5493	1/2745	8.26
NCT02065791	Canagliflozin	Placebo	3.00 (0.12, 73.50)	1/2200	0/2197	8.26
NCT03036124	Dapagliflozin	Placebo	3.00 (0.12, 73.61)	1/2368	0/2368	8.26
NCT03036150	Dapagliflozin	Placebo	0.33 (0.03, 3.20)	1/2149	3/2149	16.52
NCT03057951	Empagliflozin	Placebo	2.00 (0.18, 21.99)	2/2996	1/2989	14.68
NCT03315143	Sotagliflozin	Placebo	1.00 (0.06, 15.97)	1/5291	1/5286	11.01
NCT03619213	Dapagliflozin	Placebo	5.00 (0.24, 104.14)	2/3126	0/3127	9.17
Overall (I-squared = 0.0%, p = 0.788)			1.33 (0.53, 3.34)	12/35083	7/30871	100.00

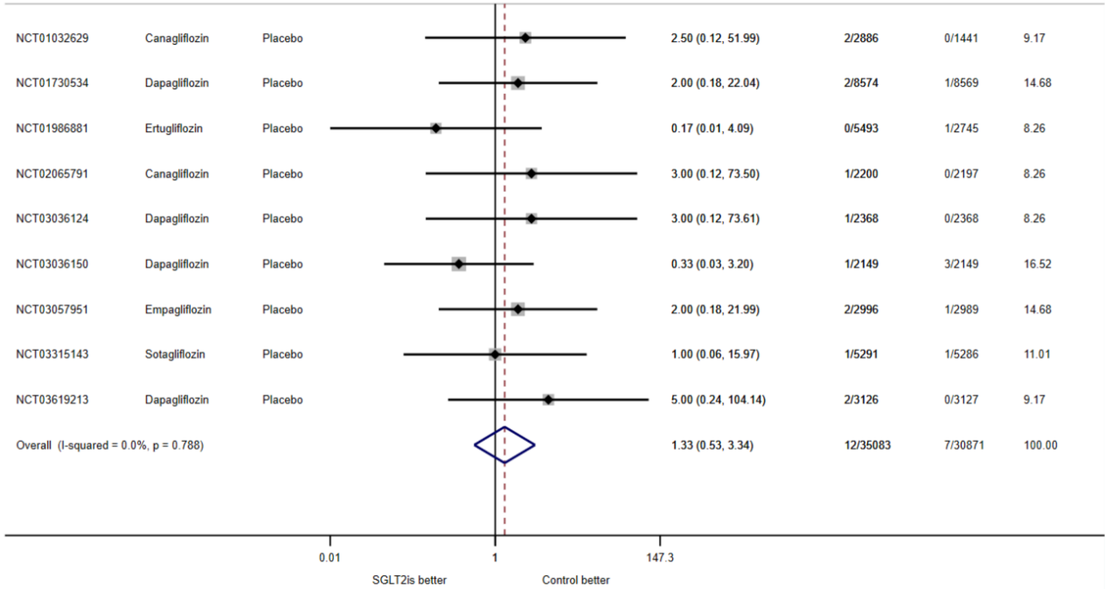

System 7-Gastrointestinal Disorders-Figure S22 Meta-analysis of SGLT2is and Diverticulum intestinal haemorrhagic

Trial	SGLT2is	Control	Risk Ratio (95% CI)	Events, Treatment	Events, Control	% Weight
NCT01032629	Canagliflozin	Placebo	0.25 (0.05, 1.36)	2/2886	4/1441	14.21
NCT01131676	Empagliflozin	Placebo	0.50 (0.07, 3.53)	2/4687	2/2333	10.65
NCT01730534	Dapagliflozin	Placebo	1.00 (0.20, 4.95)	3/8574	3/8569	15.97
NCT01986881	Ertugliflozin	Placebo	1.25 (0.24, 6.44)	5/5493	2/2745	15.22
NCT01989754	Canagliflozin	Placebo	1.00 (0.06, 15.97)	1/2904	1/2903	5.32
NCT02065791	Canagliflozin	Placebo	2.00 (0.18, 22.01)	2/2200	1/2197	7.10
NCT03036150	Dapagliflozin	Placebo	1.00 (0.14, 7.09)	2/2149	2/2149	10.66
NCT03057977	Empagliflozin	Placebo	5.00 (0.24, 104.08)	2/1863	0/1863	4.44
NCT03315143	Sotagliflozin	Placebo	0.33 (0.01, 8.17)	0/5291	1/5286	3.99
NCT03521934	Sotagliflozin	Placebo	0.34 (0.01, 8.25)	0/605	1/611	4.00
NCT03594110	Empagliflozin	Placebo	0.33 (0.01, 8.18)	0/3304	1/3305	3.99
NCT03619213	Dapagliflozin	Placebo	0.20 (0.01, 4.17)	0/3126	2/3127	4.44
Overall (I-squared = 0.0%, p = 0.874)			0.73 (0.38, 1.38)	19/43082	20/36529	100.00

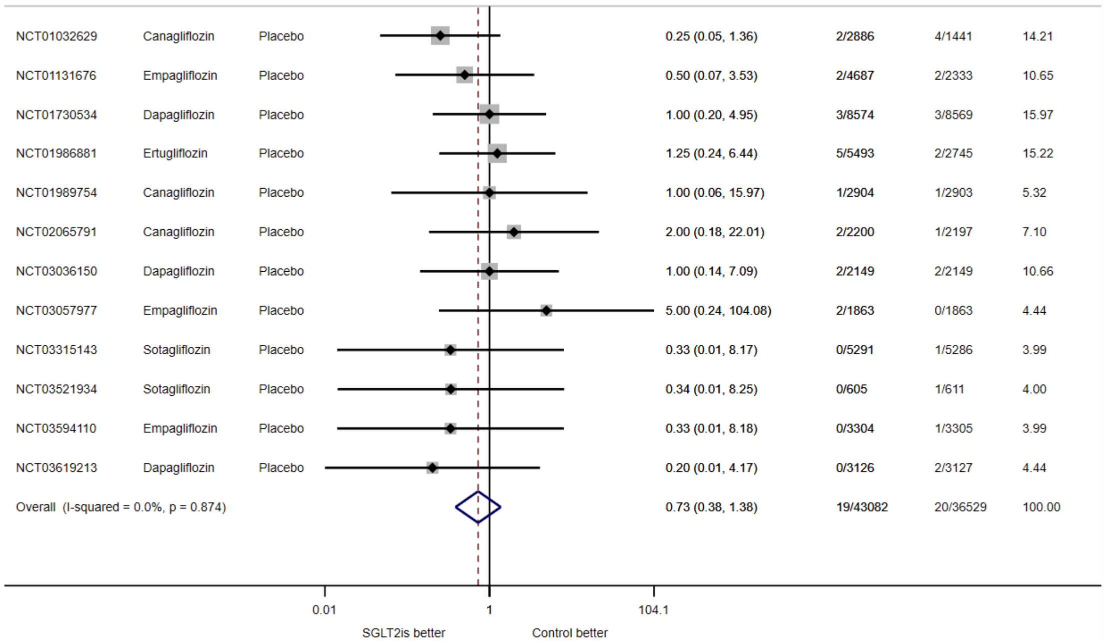

System 7-Gastrointestinal Disorders-Figure S23 Meta-analysis of SGLT2is and Duodenal ulcer

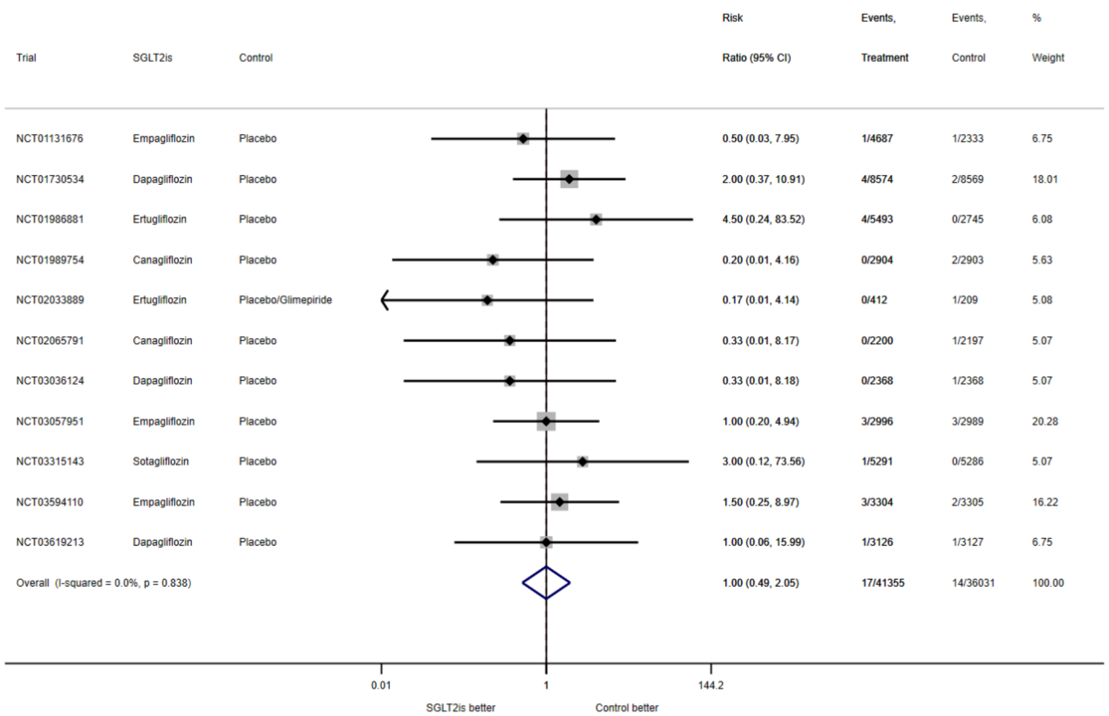

System 7-Gastrointestinal Disorders-Figure S24 Meta-analysis of SGLT2is and Duodenal ulcer haemorrhage

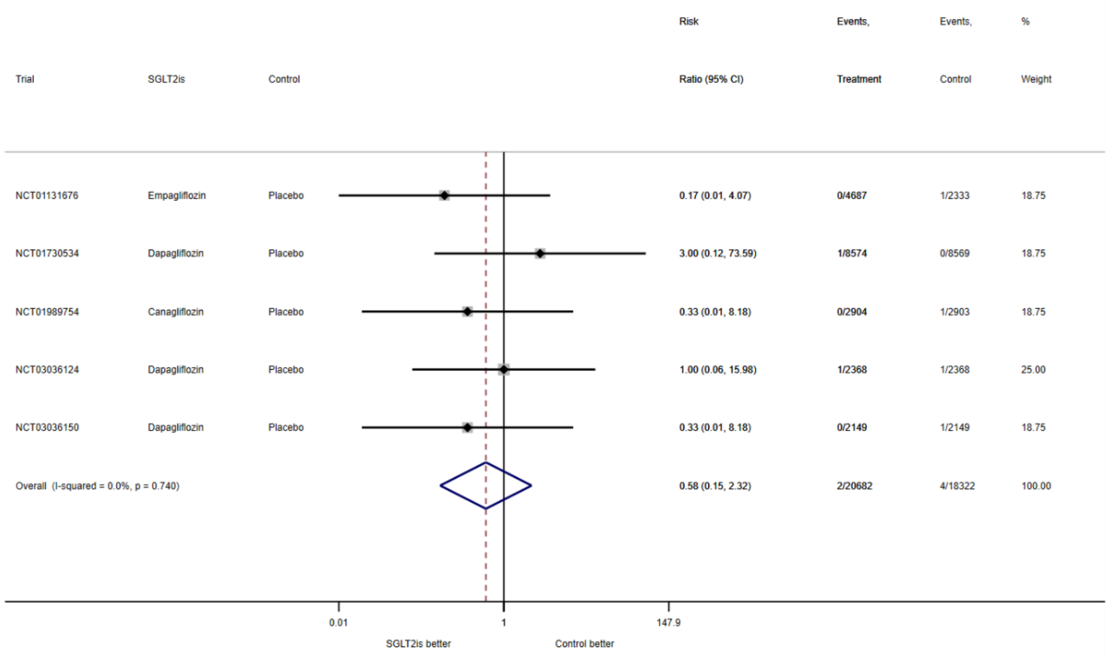

System 7-Gastrointestinal Disorders-Figure S25 Meta-analysis of SGLT2is and Duodenal ulcer perforation

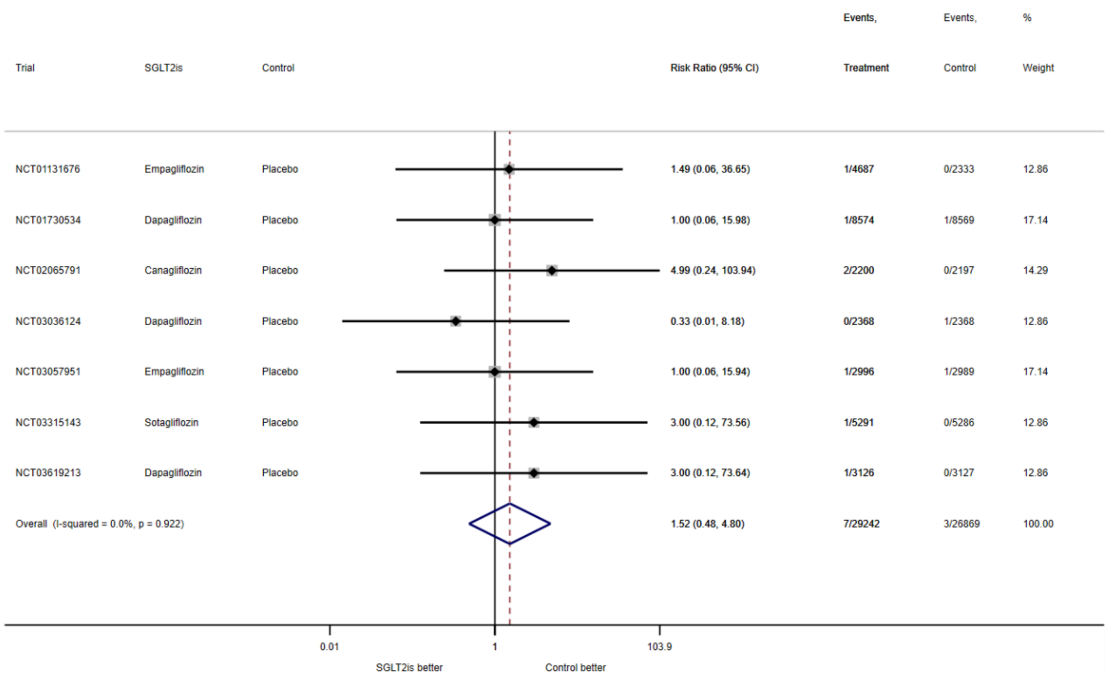

System 7-Gastrointestinal Disorders-Figure S26 Meta-analysis of SGLT2is and Duodenitis

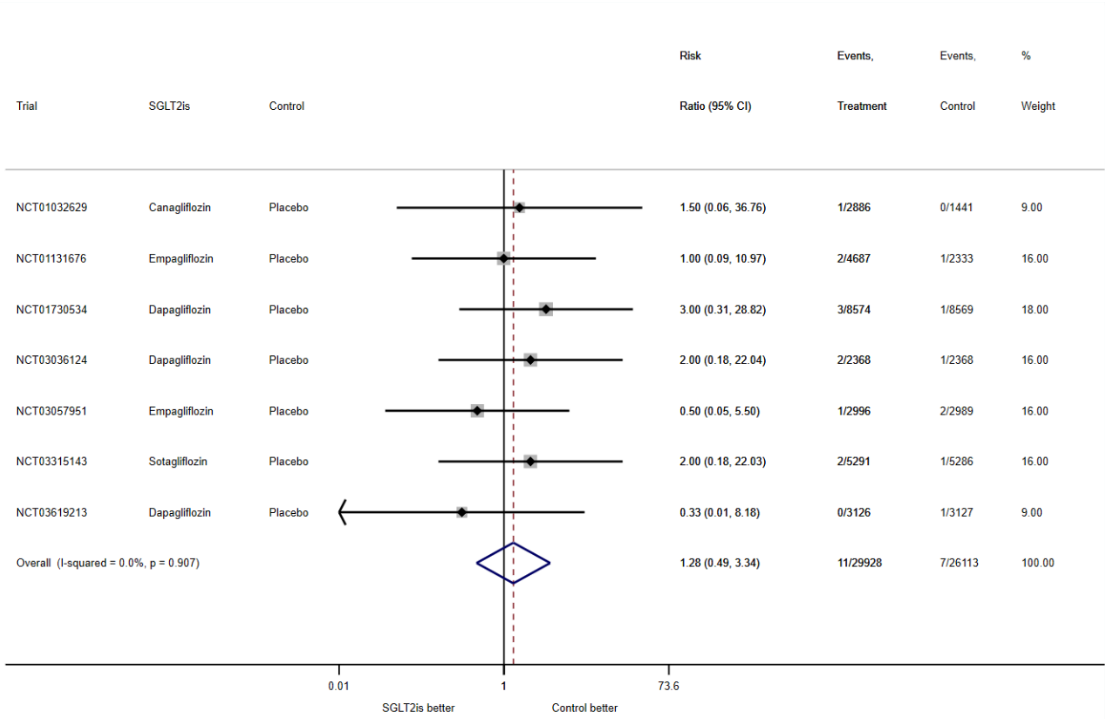

System 7-Gastrointestinal Disorders-Figure S27 Meta-analysis of SGLT2is and Dyspepsia

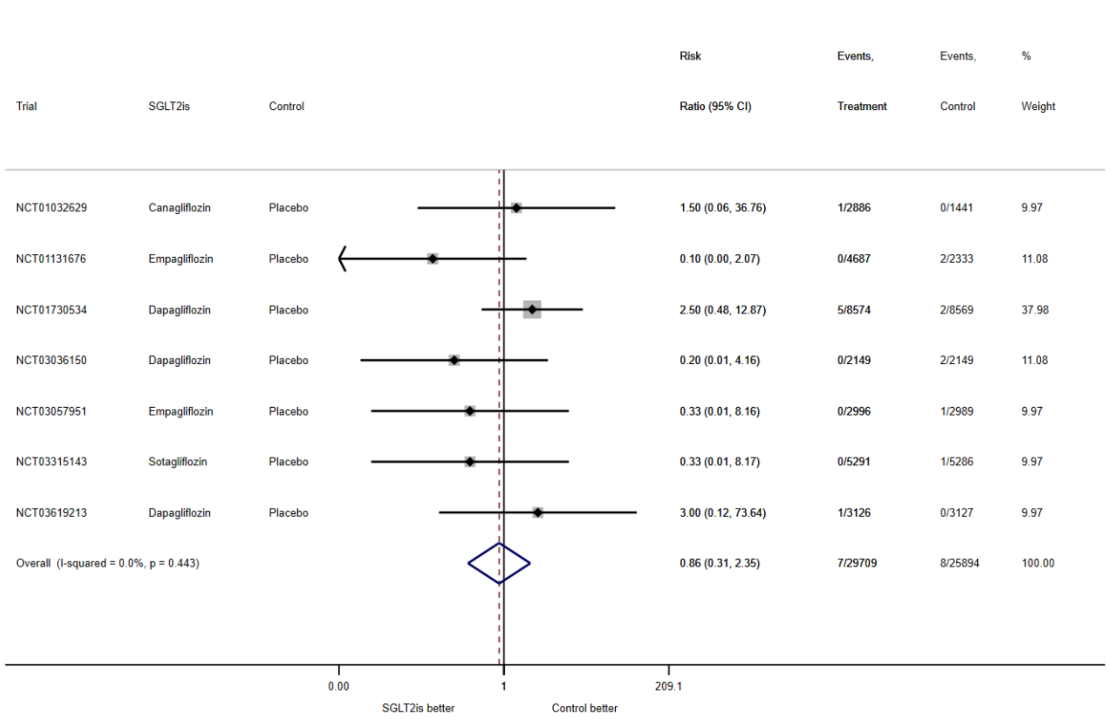

System 7-Gastrointestinal Disorders-Figure S28 Meta-analysis of SGLT2is and Dysphagia

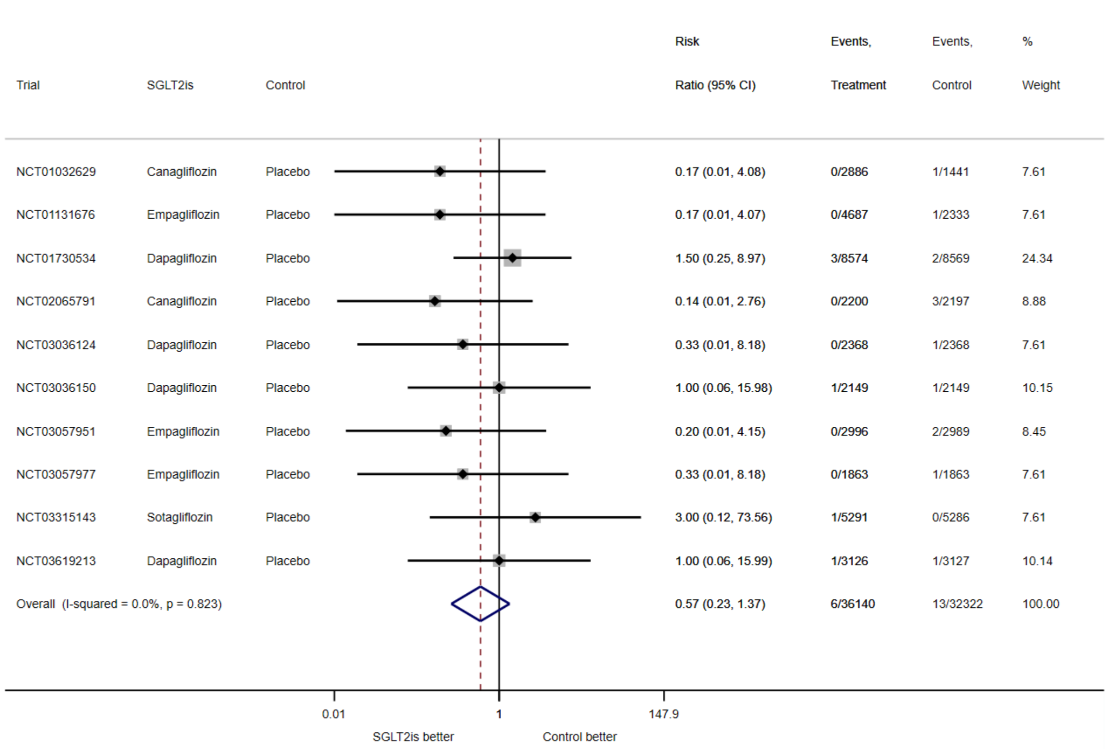

System 7-Gastrointestinal Disorders-Figure S29 Meta-analysis of SGLT2is and Enteritis

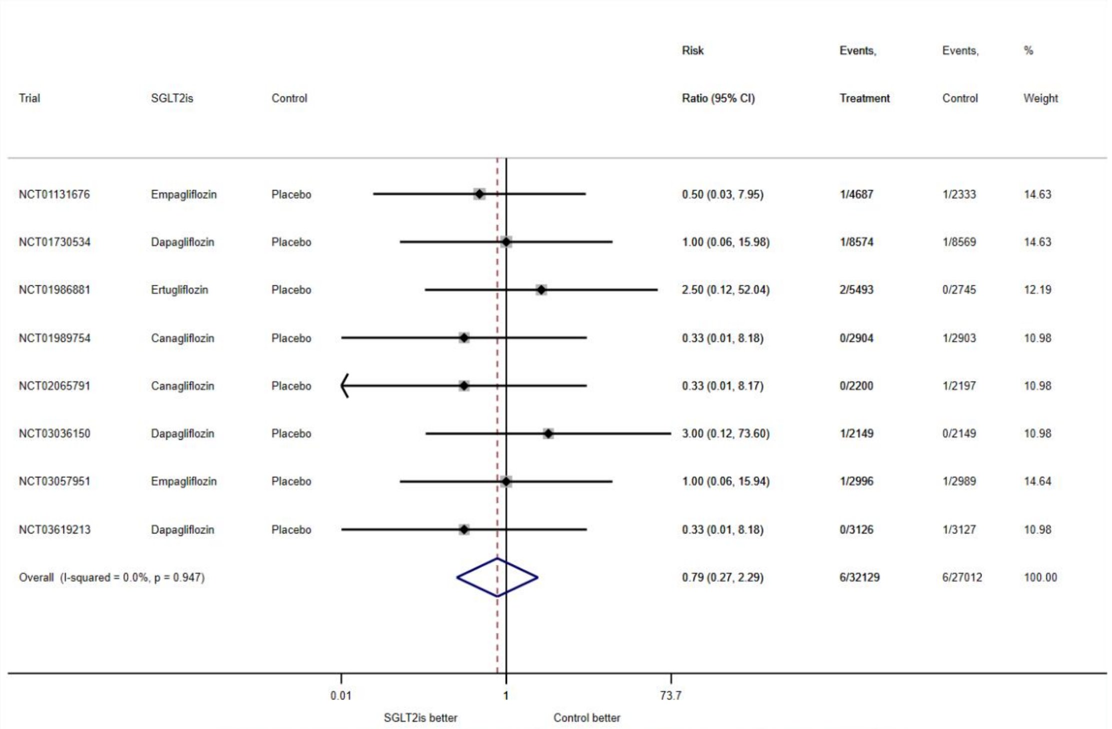

System 7-Gastrointestinal Disorders-Figure S30 Meta-analysis of SGLT2is and Enterocolitis

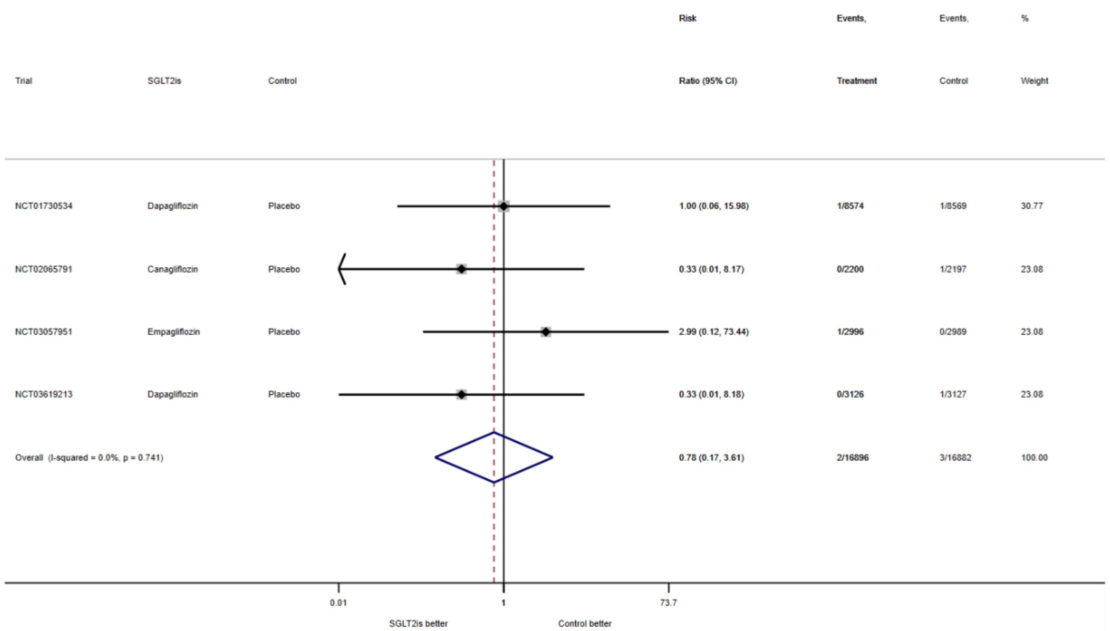

System 7-Gastrointestinal Disorders-Figure S31 Meta-analysis of SGLT2is and Enterovesical fistula

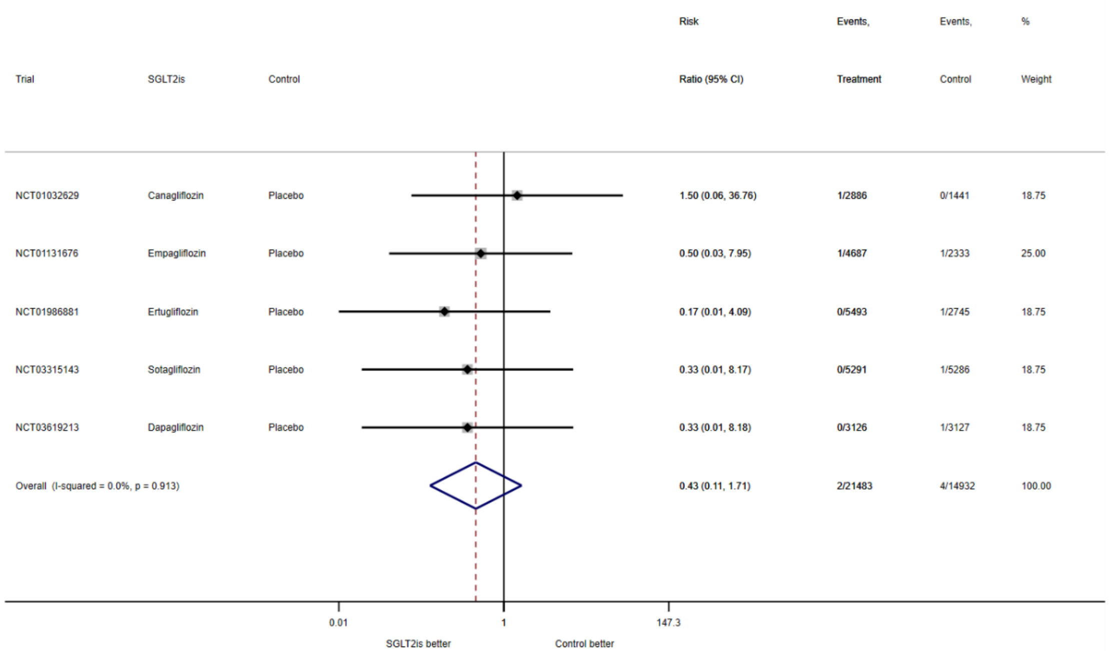

System 7-Gastrointestinal Disorders-Figure S32 Meta-analysis of SGLT2is and Erosive duodenitis

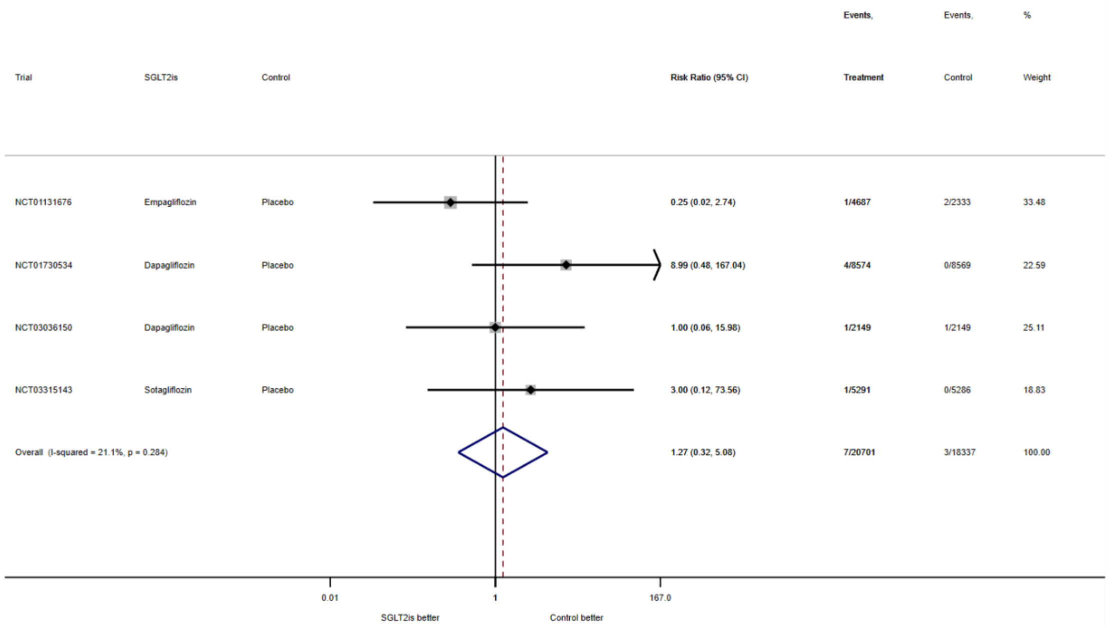

System 7-Gastrointestinal Disorders-Figure S33 Meta-analysis of SGLT2is and Faecaloma

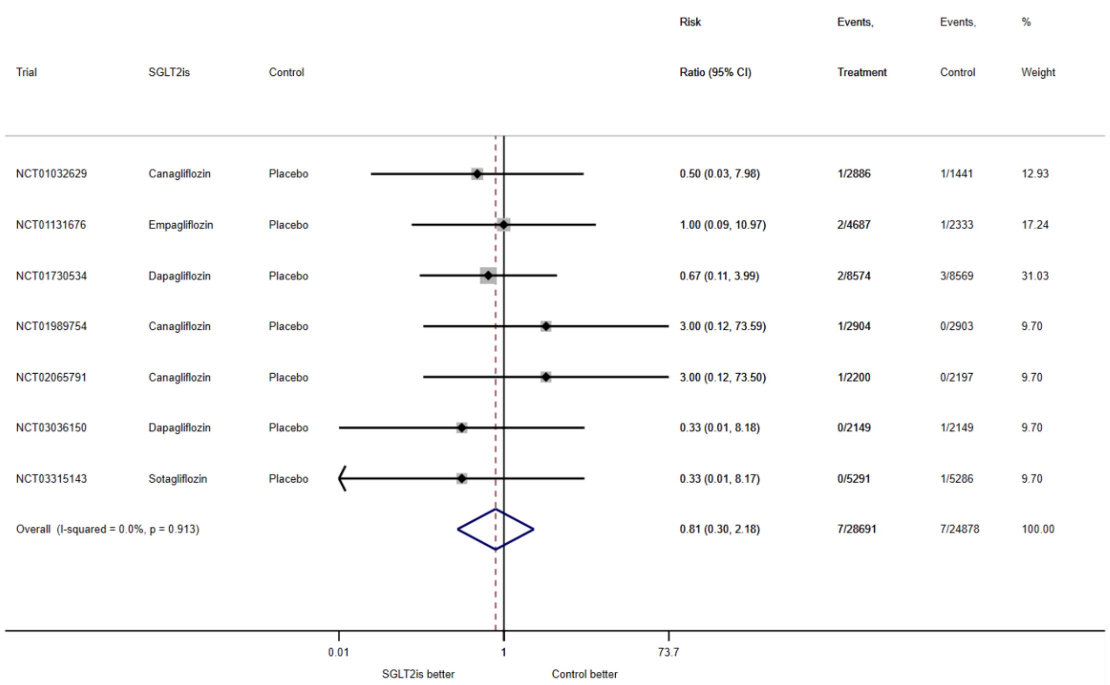

System 7-Gastrointestinal Disorders-Figure S34 Meta-analysis of SGLT2is and Food poisoning

Trial	SGLT2is	Control	Risk Ratio (95% CI)	Events, Treatment	Events, Control	% Weight
NCT01042977	Dapagliflozin	Placebo	3.01 (0.12, 73.61)	1/482	0/483	7.83
NCT01131676	Empagliflozin	Placebo	0.17 (0.01, 4.07)	0/4687	1/2333	7.82
NCT01730534	Dapagliflozin	Placebo	1.00 (0.14, 7.09)	2/8574	2/8569	20.86
NCT01986881	Ertugliflozin	Placebo	2.50 (0.12, 52.04)	2/5493	0/2745	8.69
NCT03036124	Dapagliflozin	Placebo	3.00 (0.12, 73.61)	1/2368	0/2368	7.82
NCT03057951	Empagliflozin	Placebo	0.33 (0.01, 8.16)	0/2996	1/2989	7.82
NCT03057977	Empagliflozin	Placebo	0.33 (0.01, 8.18)	0/1863	1/1863	7.83
NCT03242252	Sotagliflozin	Placebo	0.16 (0.01, 4.03)	0/527	1/260	7.84
NCT03315143	Sotagliflozin	Placebo	0.33 (0.01, 8.17)	0/5291	1/5286	7.82
NCT03594110	Empagliflozin	Placebo	0.33 (0.01, 8.18)	0/3304	1/3305	7.82
NCT03619213	Dapagliflozin	Placebo	3.00 (0.12, 73.64)	1/3126	0/3127	7.82
Overall (I-squared = 0.0%, p = 0.851)			0.75 (0.31, 1.83)	7/38711	8/33328	100.00

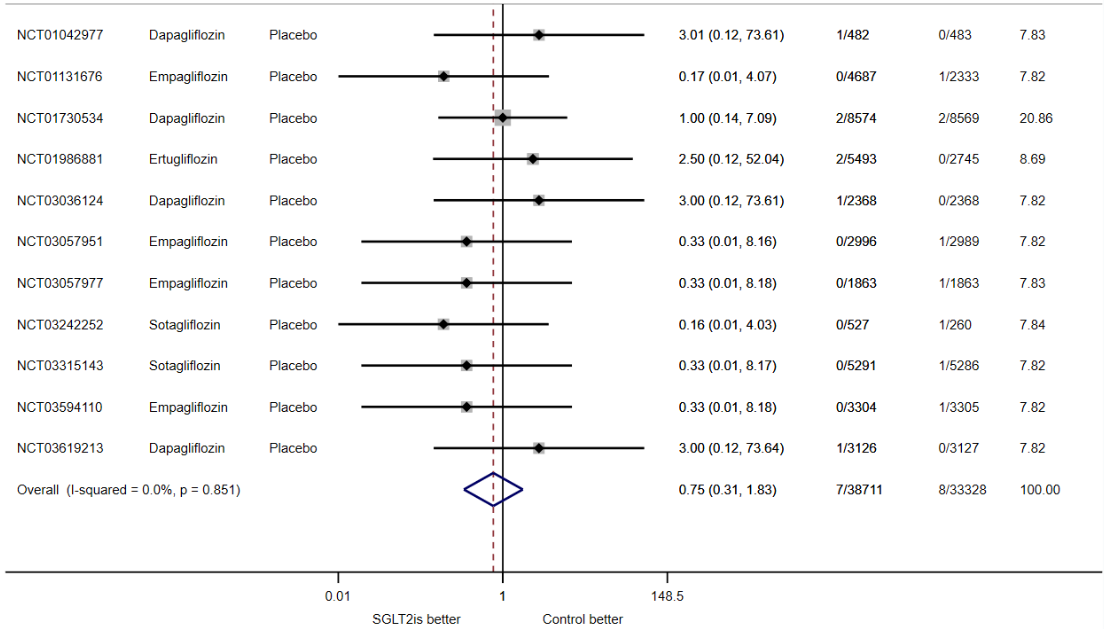

System 7-Gastrointestinal Disorders-Figure S35 Meta-analysis of SGLT2is and Gastric haemorrhage

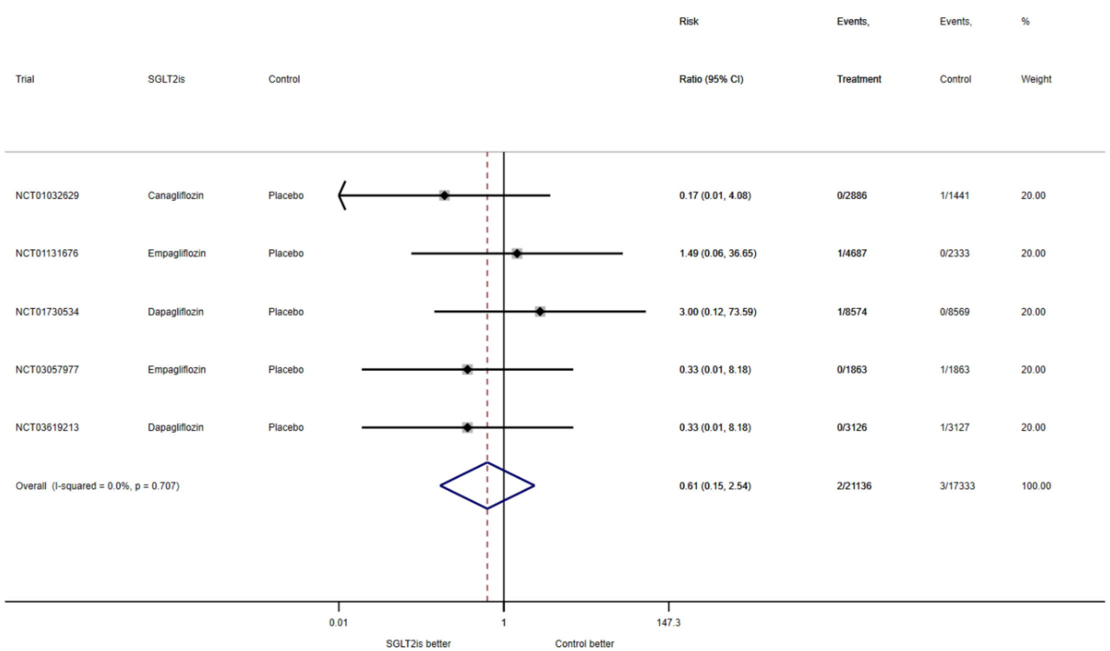

System 7-Gastrointestinal Disorders-Figure S36 Meta-analysis of SGLT2is and Gastric perforation

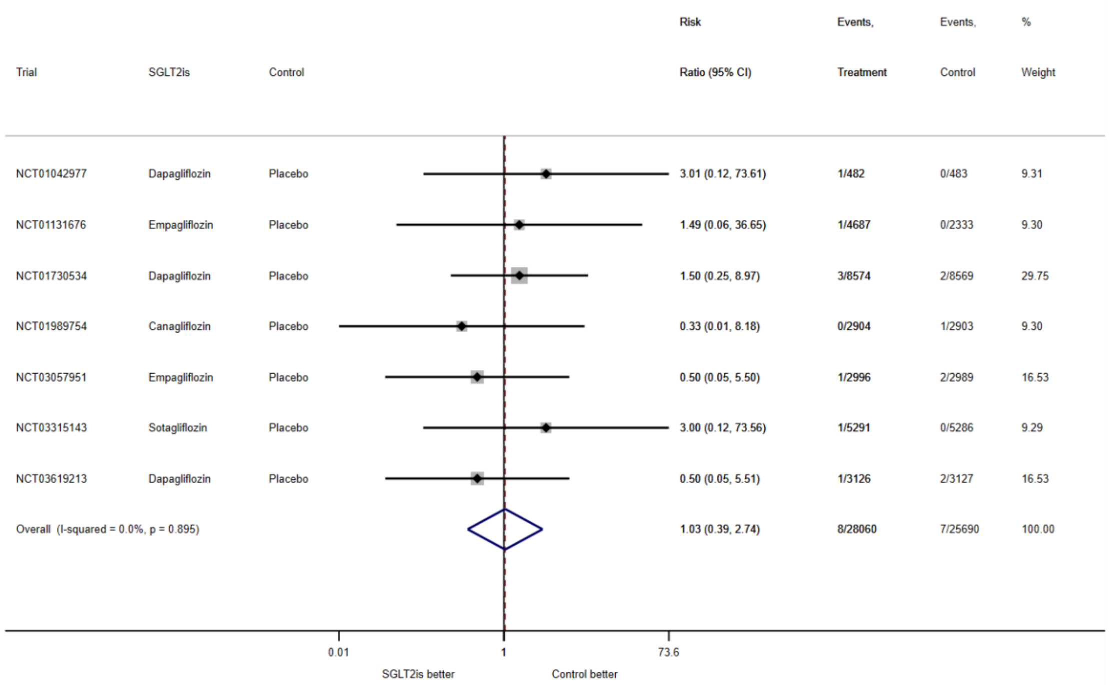

System 7-Gastrointestinal Disorders-Figure S37 Meta-analysis of SGLT2is and Gastric polyps

Trial	SGLT2is	Control	Risk Ratio (95% CI)	Events, Treatment	Events, Control	% Weight
NCT01031680	Dapagliflozin	Placebo	3.01 (0.12, 73.77)	1/460	0/462	2.67
NCT01032629	Canagliflozin	Placebo	1.00 (0.09, 11.00)	2/2886	1/1441	4.74
NCT01131676	Empagliflozin	Placebo	0.50 (0.10, 2.46)	3/4687	3/2333	10.66
NCT01730534	Dapagliflozin	Placebo	0.82 (0.34, 1.97)	9/8574	11/8569	35.18
NCT01986881	Ertugliflozin	Placebo	2.50 (0.12, 52.04)	2/5493	0/2745	2.96
NCT02065791	Canagliflozin	Placebo	4.99 (0.24, 103.94)	2/2200	0/2197	2.96
NCT03036124	Dapagliflozin	Placebo	1.00 (0.06, 15.98)	1/2368	1/2368	3.55
NCT03036150	Dapagliflozin	Placebo	0.50 (0.05, 5.51)	1/2149	2/2149	4.74
NCT03057951	Empagliflozin	Placebo	1.00 (0.14, 7.08)	2/2996	2/2989	7.10
NCT03057977	Empagliflozin	Placebo	3.00 (0.12, 73.59)	1/1863	0/1863	2.66
NCT03315143	Sotagliflozin	Placebo	2.00 (0.18, 22.03)	2/5291	1/5286	4.73
NCT03594110	Empagliflozin	Placebo	2.00 (0.18, 22.05)	2/3304	1/3305	4.73
NCT03619213	Dapagliflozin	Placebo	0.60 (0.14, 2.51)	3/3126	5/3127	13.33
Overall (I-squared = 0.0%, p = 0.968)			0.95 (0.57, 1.61)	31/45397	27/38834	100.00

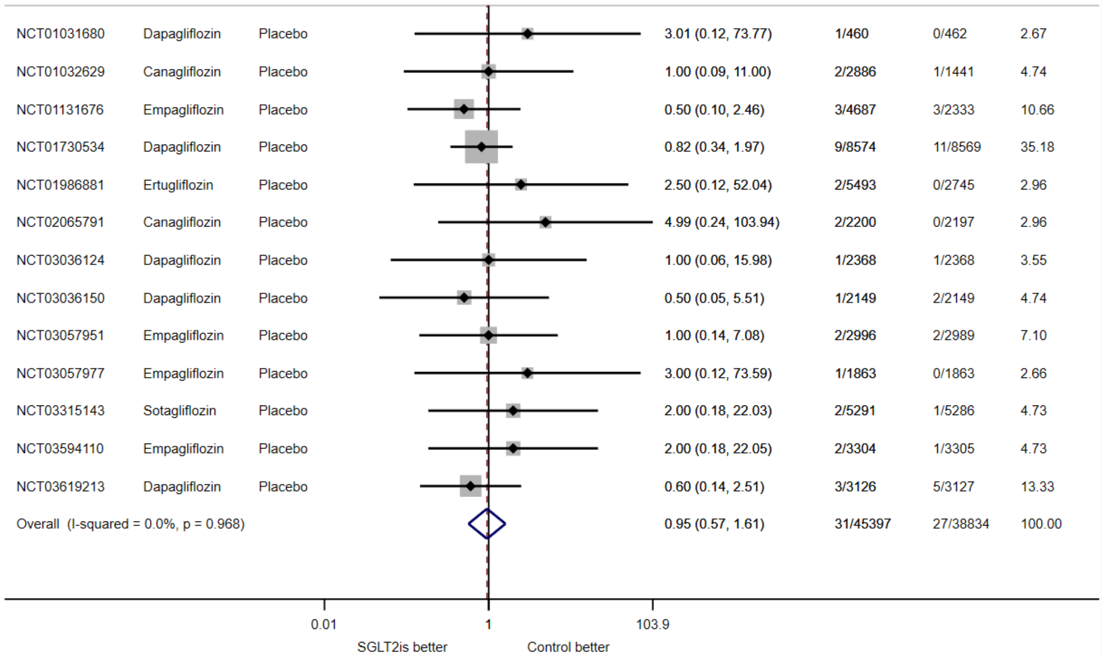

System 7-Gastrointestinal Disorders-Figure S38 Meta-analysis of SGLT2is and Gastric ulcer

Trial	SGLT2is	Control	Risk Ratio (95% CI)	Events, Treatment	Events, Control	% Weight
NCT01131676	Empagliflozin	Placebo	1.49 (0.06, 36.65)	1/4687	0/2333	5.24
NCT01730534	Dapagliflozin	Placebo	2.00 (0.37, 10.91)	4/8574	2/8569	18.64
NCT01986881	Ertugliflozin	Placebo	0.50 (0.03, 7.99)	1/5493	1/2745	6.99
NCT03036124	Dapagliflozin	Placebo	1.00 (0.14, 7.09)	2/2368	2/2368	13.99
NCT03036150	Dapagliflozin	Placebo	2.00 (0.18, 22.04)	2/2149	1/2149	9.33
NCT03057951	Empagliflozin	Placebo	0.33 (0.01, 8.16)	0/2996	1/2989	5.24
NCT03315143	Sotagliflozin	Placebo	1.50 (0.25, 8.96)	3/5291	2/5286	16.78
NCT03594110	Empagliflozin	Placebo	1.00 (0.06, 15.99)	1/3304	1/3305	6.99
NCT03619213	Dapagliflozin	Placebo	0.67 (0.11, 3.99)	2/3126	3/3127	16.79
Overall (I-squared = 0.0%, p = 0.980)			1.11 (0.54, 2.32)	16/37988	13/32871	100.00

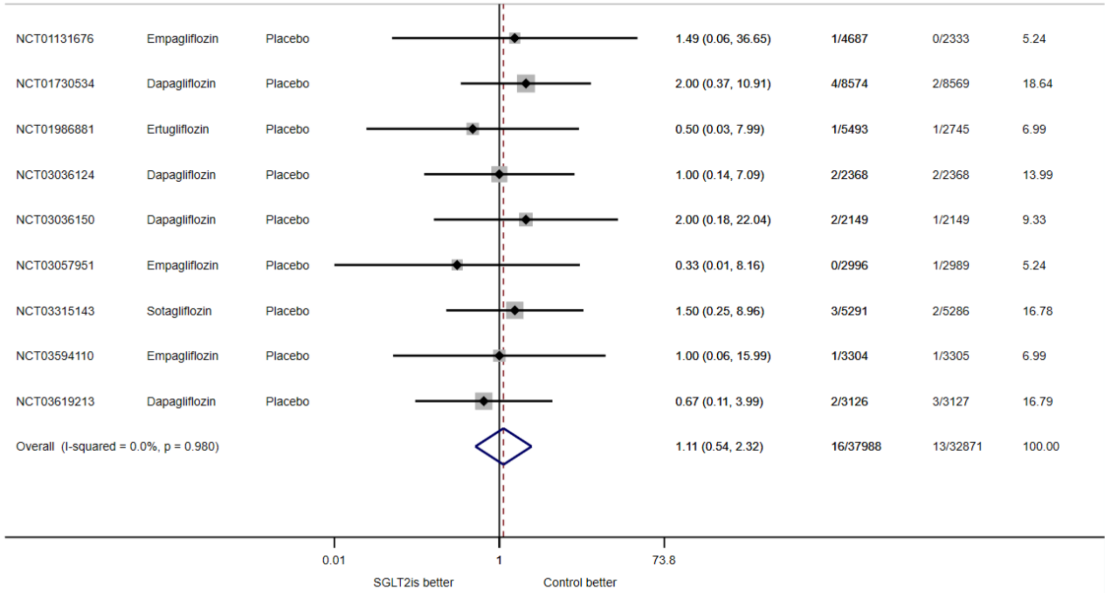

System 7-Gastrointestinal Disorders-Figure S39 Meta-analysis of SGLT2is and Gastric ulcer haemorrhage

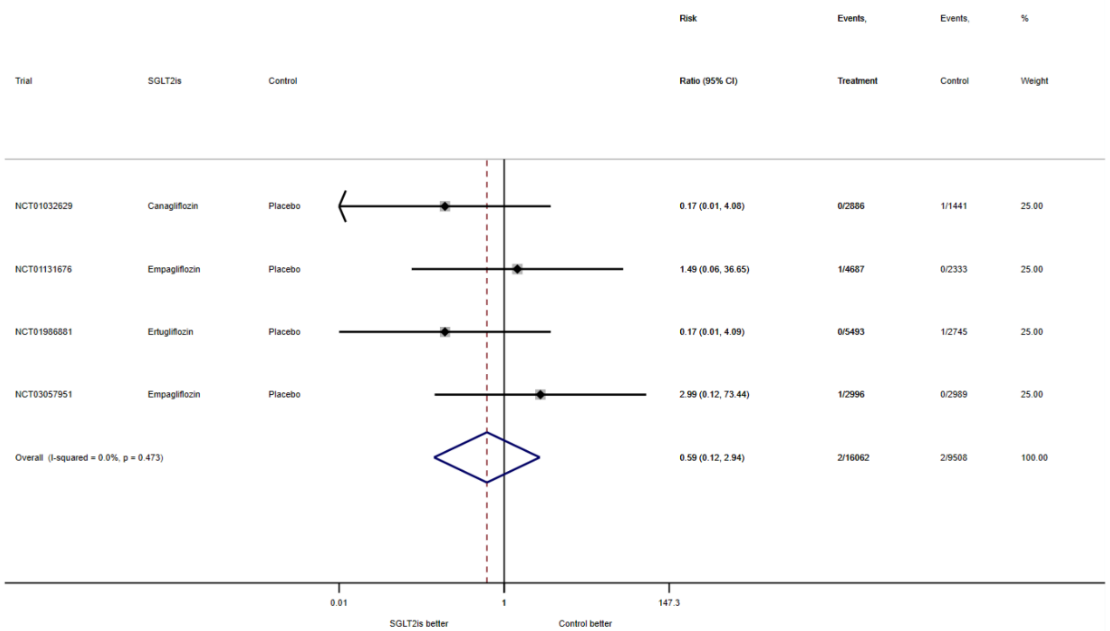

System 7-Gastrointestinal Disorders-Figure S40 Meta-analysis of SGLT2is and Gastric ulcer perforation

Trial	SGLT2is	Control	Risk Ratio (95% CI)	Events, Treatment	Events, Control	% Weight
NCT01031680	Dapagliflozin	Placebo	0.33 (0.01, 8.20)	0/460	1/462	1.75
NCT01032629	Canagliflozin	Placebo	2.00 (0.22, 17.85)	4/2886	1/1441	3.72
NCT01042977	Dapagliflozin	Placebo	3.01 (0.12, 73.61)	1/482	0/483	1.75
NCT01131676	Empagliflozin	Placebo	2.24 (0.48, 10.36)	9/4687	2/2333	7.61
NCT01730534	Dapagliflozin	Placebo	0.65 (0.32, 1.31)	13/8574	20/8569	36.69
NCT01986881	Ertugliflozin	Placebo	0.12 (0.01, 1.12)	1/5493	4/2745	3.72
NCT02065791	Canagliflozin	Placebo	3.00 (0.31, 28.78)	3/2200	1/2197	3.49
NCT03036124	Dapagliflozin	Placebo	0.40 (0.08, 2.06)	2/2368	5/2368	6.65
NCT03036150	Dapagliflozin	Placebo	0.60 (0.14, 2.51)	3/2149	5/2149	8.73
NCT03057951	Empagliflozin	Placebo	0.67 (0.19, 2.35)	4/2996	6/2989	11.17
NCT03057977	Empagliflozin	Placebo	2.00 (0.18, 22.04)	2/1863	1/1863	3.10
NCT03315143	Sotagliflozin	Placebo	1.00 (0.06, 15.97)	1/5291	1/5286	2.32
NCT03594110	Empagliflozin	Placebo	0.25 (0.03, 2.24)	1/3304	4/3305	3.72
NCT03619213	Dapagliflozin	Placebo	1.50 (0.25, 8.97)	3/3126	2/3127	5.58
Overall (I-squared = 0.0%, p = 0.629)			0.76 (0.50, 1.17)	47/45879	53/39317	100.00

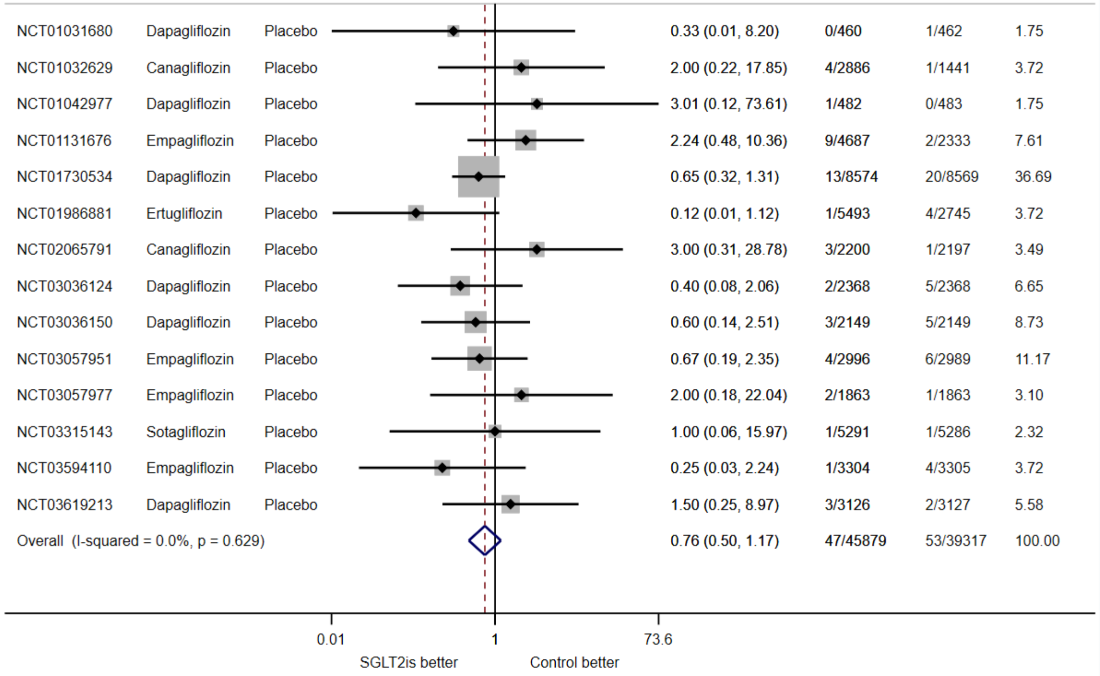

System 7-Gastrointestinal Disorders-Figure S41 Meta-analysis of SGLT2is and Gastritis

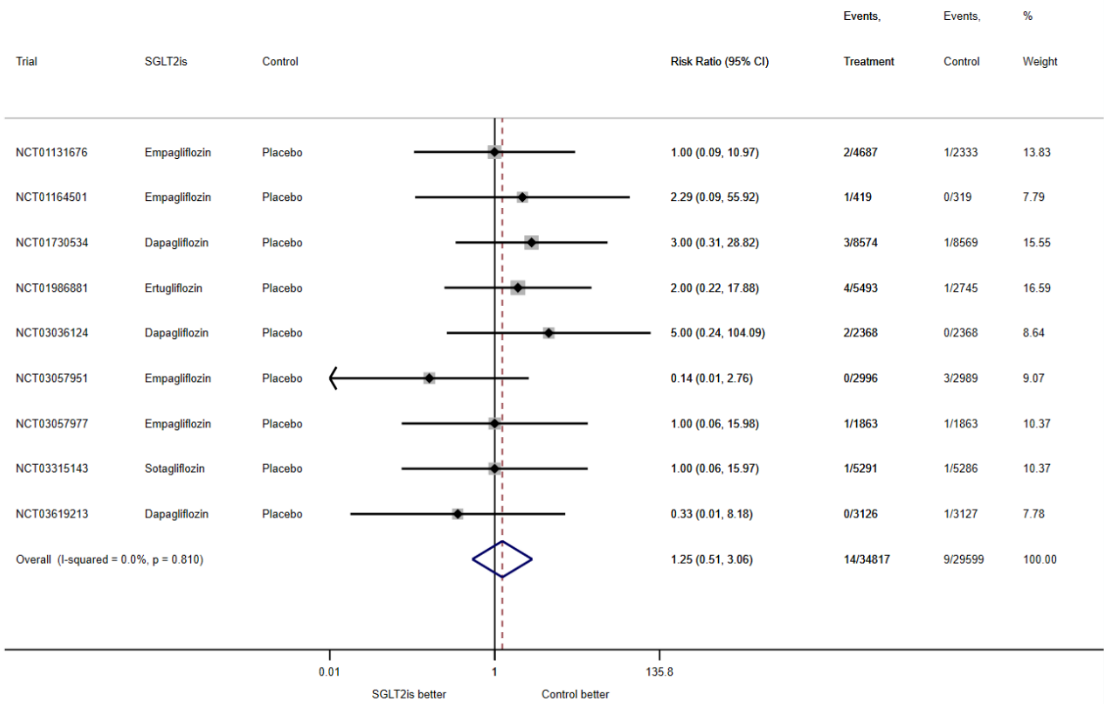

System 7-Gastrointestinal Disorders-Figure S42 Meta-analysis of SGLT2is and Gastritis erosive

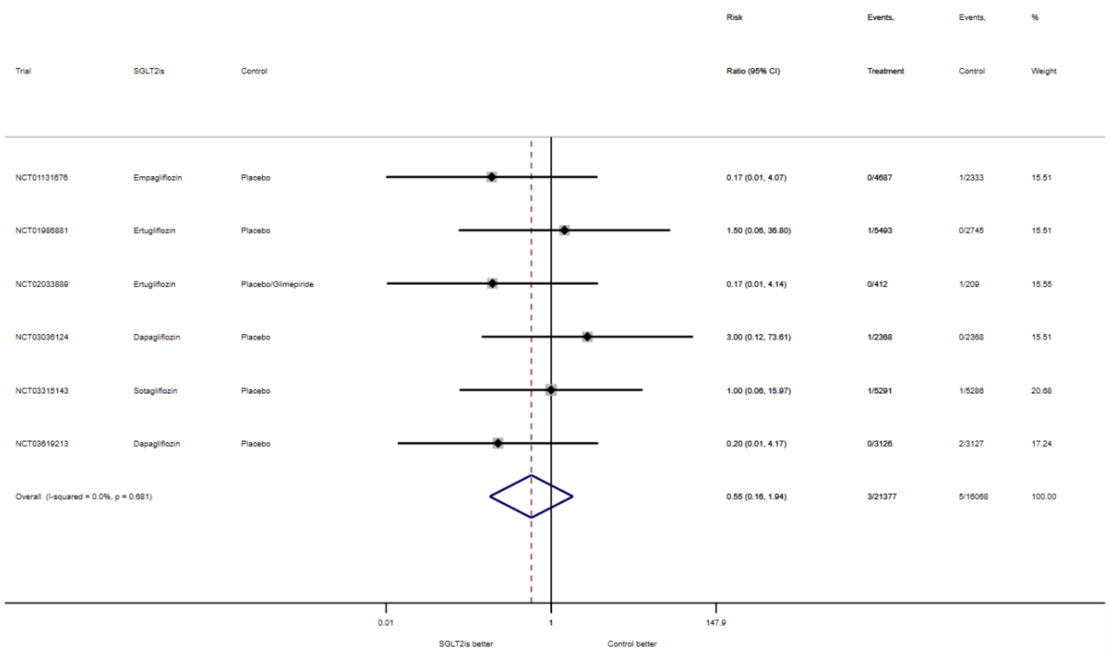

System 7-Gastrointestinal Disorders-Figure S43 Meta-analysis of SGLT2is and Gastritis haemorrhagic

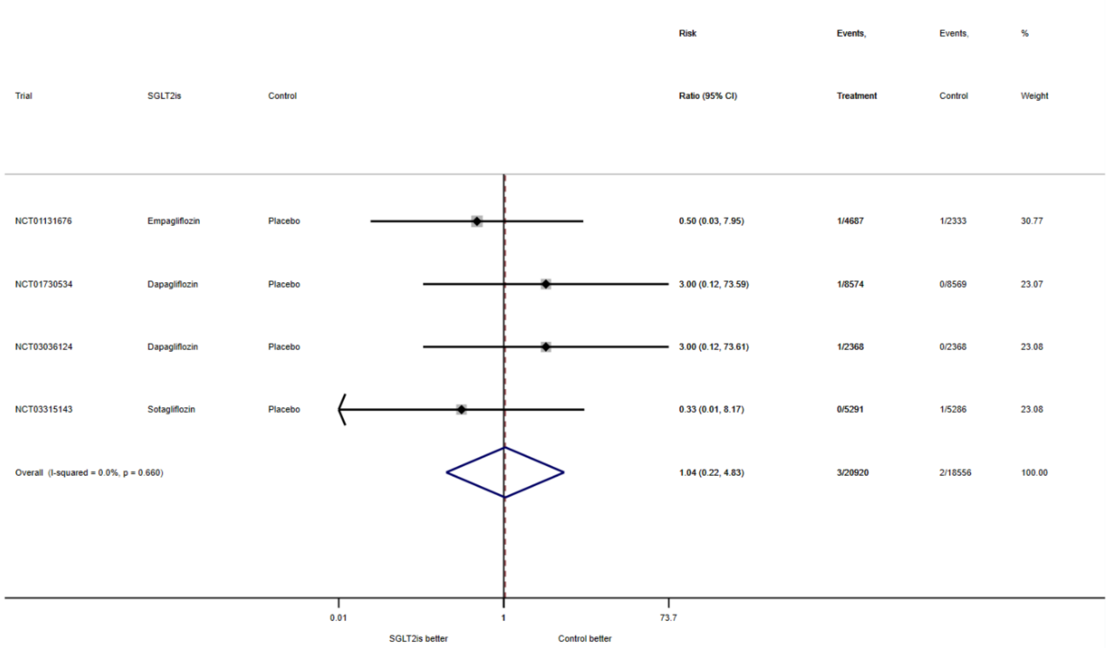

System 7-Gastrointestinal Disorders-Figure S44 Meta-analysis of SGLT2is and Gastrointestinal angiodysplasia

Trial	SGLT2is	Control	Risk Ratio (95% CI)	Events, Treatment	Events, Control	% Weight
NCT01032629	Canagliflozin	Placebo	1.00 (0.18, 5.45)	4/2886	2/1441	2.63
NCT01106651	Canagliflozin	Placebo	0.17 (0.01, 4.06)	0/477	1/237	0.74
NCT01131676	Empagliflozin	Placebo	0.44 (0.17, 1.15)	8/4687	9/2333	8.35
NCT01730534	Dapagliflozin	Placebo	0.94 (0.49, 1.83)	17/8574	18/8569	17.23
NCT01986881	Ertugliflozin	Placebo	1.00 (0.38, 2.66)	12/5493	6/2745	7.88
NCT01989754	Canagliflozin	Placebo	1.00 (0.20, 4.95)	3/2904	3/2903	2.95
NCT02065791	Canagliflozin	Placebo	0.67 (0.19, 2.36)	4/2200	6/2197	4.73
NCT03036124	Dapagliflozin	Placebo	0.57 (0.17, 1.95)	4/2368	7/2368	5.02
NCT03036150	Dapagliflozin	Placebo	0.83 (0.25, 2.73)	5/2149	6/2149	5.38
NCT03057951	Empagliflozin	Placebo	0.82 (0.41, 1.66)	14/2996	17/2989	15.18
NCT03057977	Empagliflozin	Placebo	2.00 (0.60, 6.63)	8/1863	4/1863	5.26
NCT03315143	Sotagliflozin	Placebo	1.00 (0.29, 3.45)	5/5291	5/5286	4.92
NCT03521934	Sotagliflozin	Placebo	0.34 (0.01, 8.25)	0/605	1/611	0.74
NCT03594110	Empagliflozin	Placebo	2.00 (0.50, 7.99)	6/3304	3/3305	3.94
NCT03619213	Dapagliflozin	Placebo	0.93 (0.45, 1.93)	14/3126	15/3127	14.31
NCT04157751	Empagliflozin	Placebo	3.05 (0.12, 74.43)	1/260	0/264	0.74
Overall (I-squared = 0.0%, p = 0.921)			0.88 (0.67, 1.16)	105/49183	103/42387	100.00

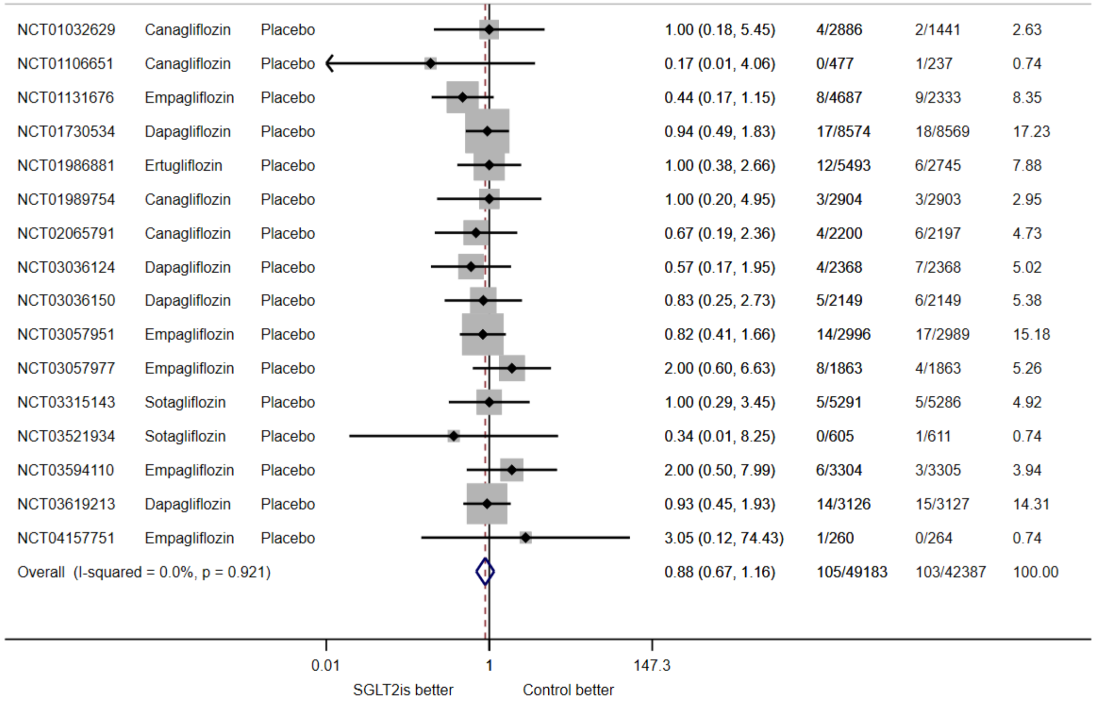

System 7-Gastrointestinal Disorders-Figure S45 Meta-analysis of SGLT2is and Gastrointestinal haemorrhage

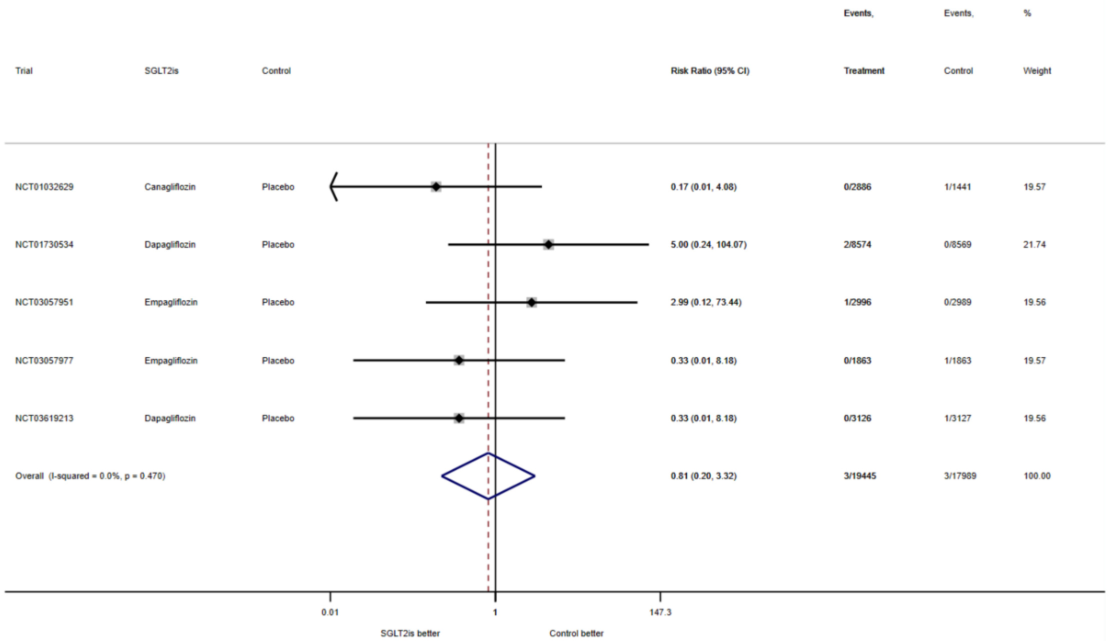

System 7-Gastrointestinal Disorders-Figure S46 Meta-analysis of SGLT2is and Gastrointestinal necrosis

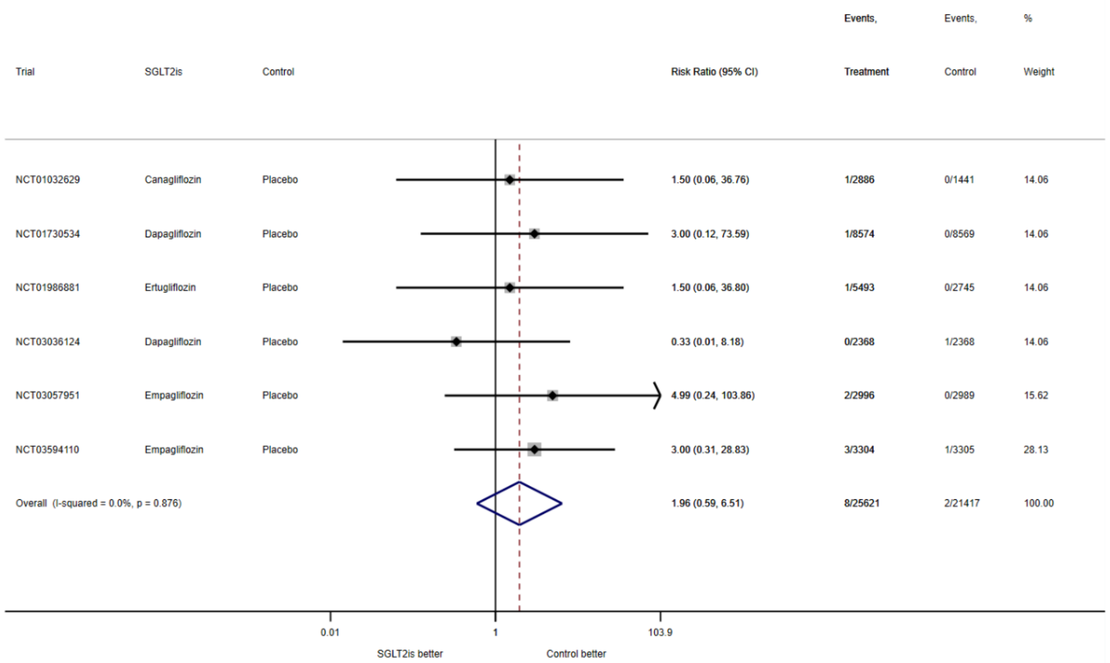

System 7-Gastrointestinal Disorders-Figure S47 Meta-analysis of SGLT2is and Gastrointestinal obstruction

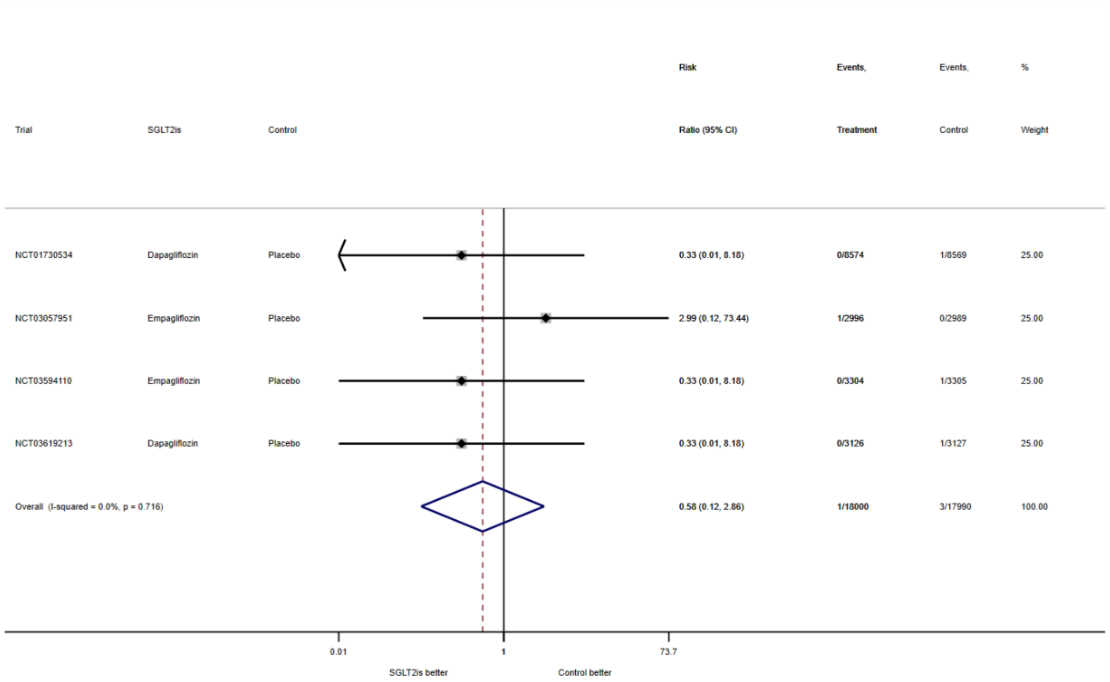

System 7-Gastrointestinal Disorders-Figure S48 Meta-analysis of SGLT2is and Gastrointestinal perforation

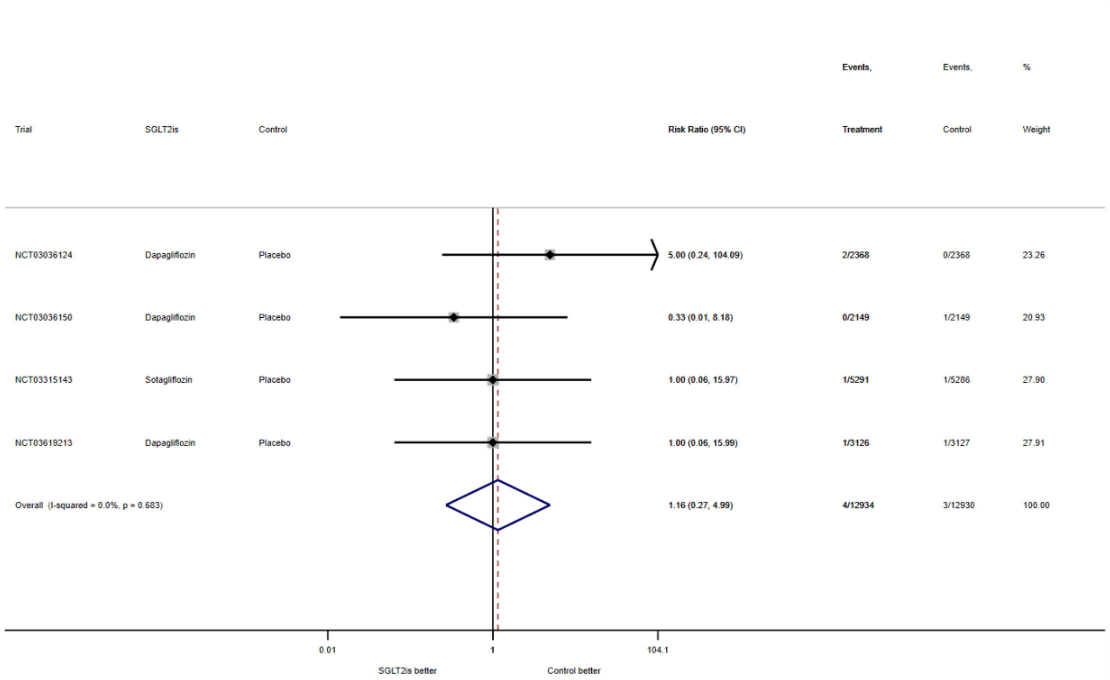

System 7-Gastrointestinal Disorders-Figure S49 Meta-analysis of SGLT2is and Gastrointestinal polyp haemorrhage

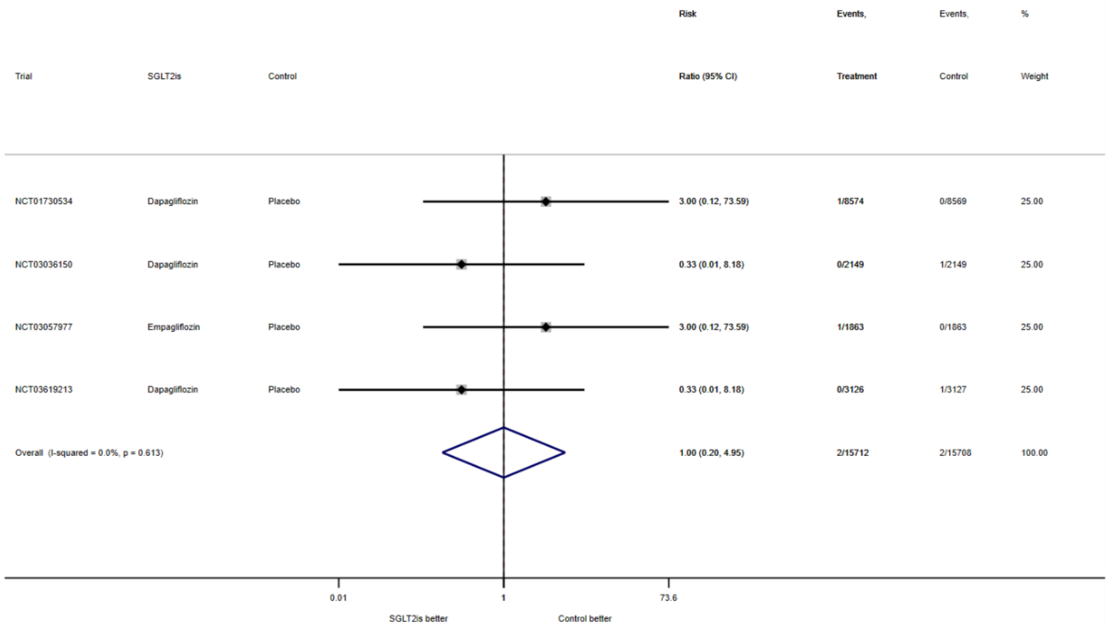

System 7-Gastrointestinal Disorders-Figure S50 Meta-analysis of SGLT2is and Gastrointestinal ulcer haemorrhage

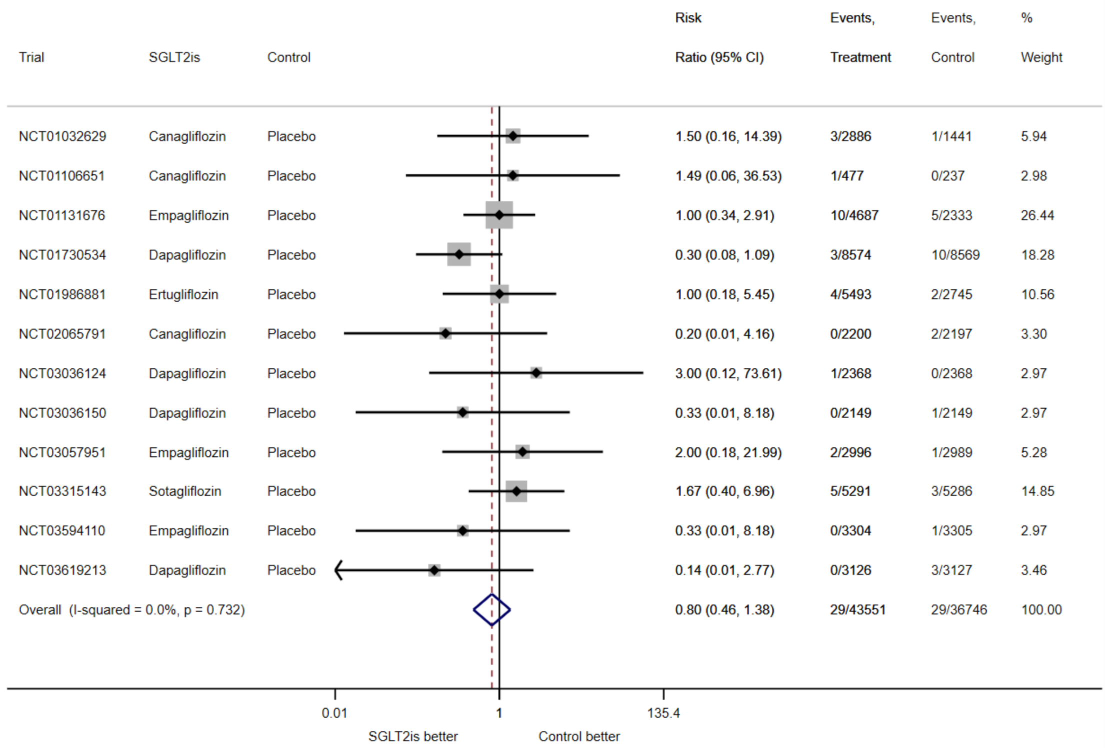

System 7-Gastrointestinal Disorders-Figure S51 Meta-analysis of SGLT2is and Gastrooesophageal reflux disease

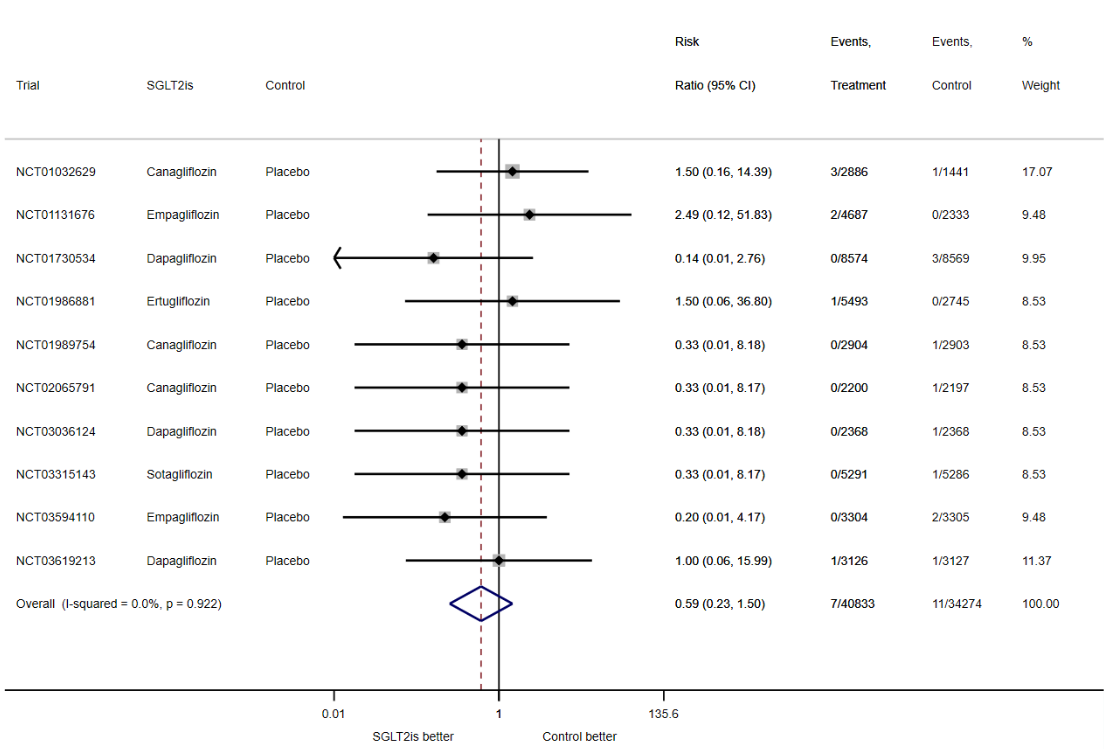

System 7-Gastrointestinal Disorders-Figure S52 Meta-analysis of SGLT2is and Haematemesis

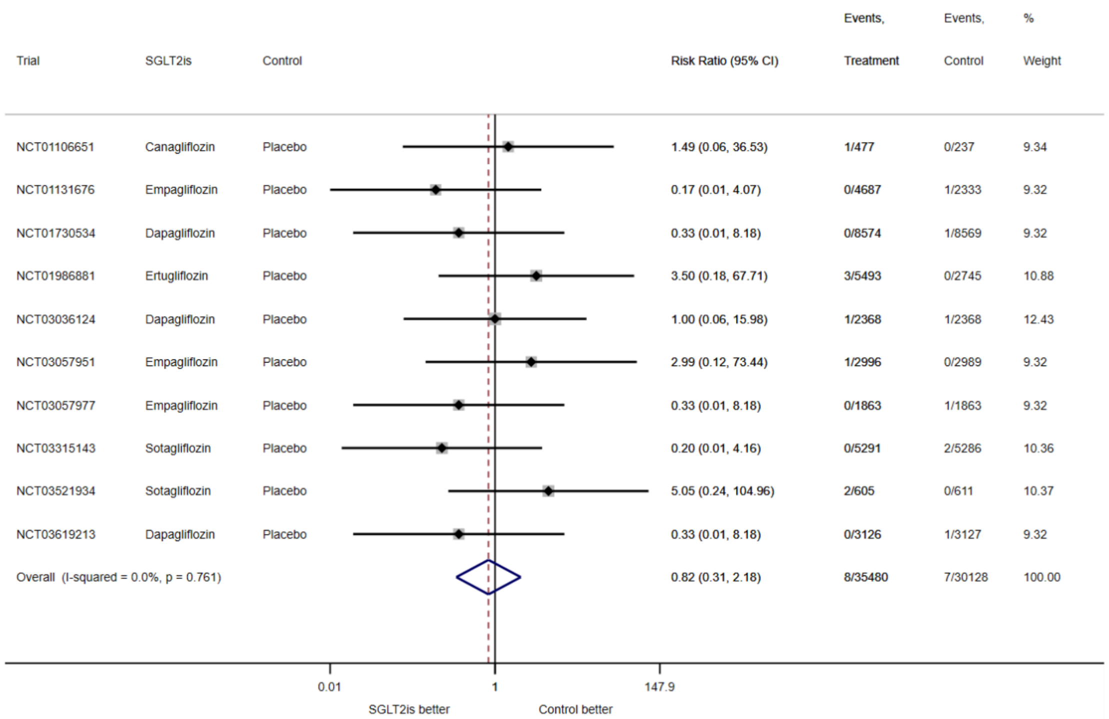

System 7-Gastrointestinal Disorders-Figure S53 Meta-analysis of SGLT2is and Haematochezia

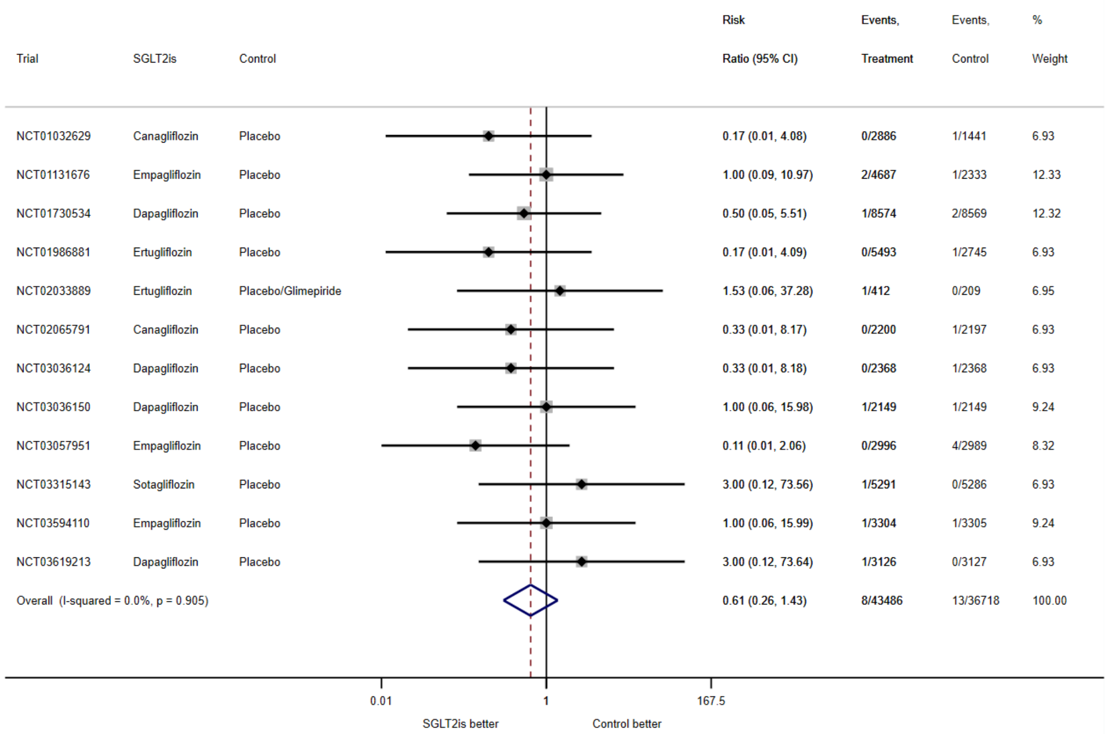

System 7-Gastrointestinal Disorders-Figure S54 Meta-analysis of SGLT2is and Haemorrhoidal haemorrhage

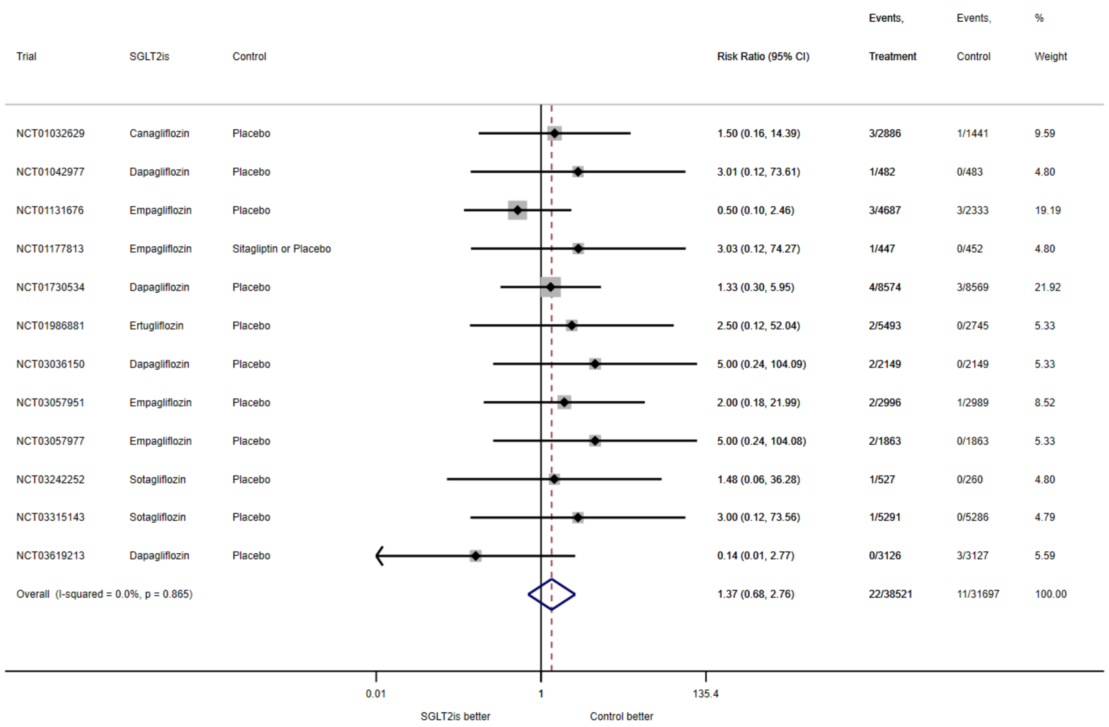

System 7-Gastrointestinal Disorders-Figure S55 Meta-analysis of SGLT2is and Haemorrhoids

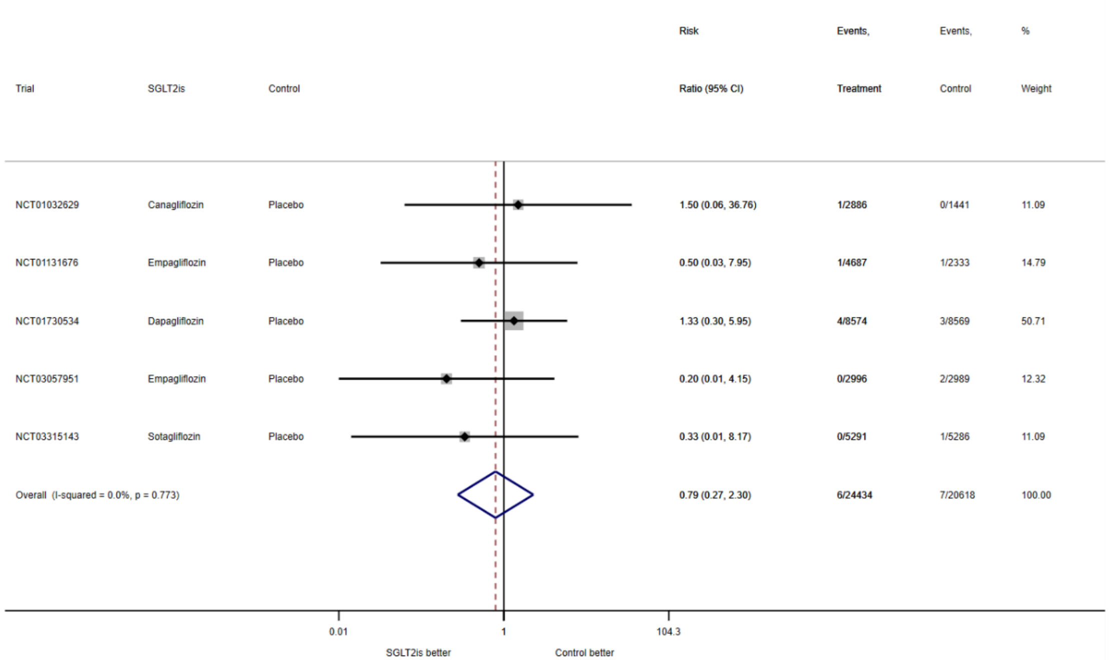

System 7-Gastrointestinal Disorders-Figure S56 Meta-analysis of SGLT2is and Hiatus hernia

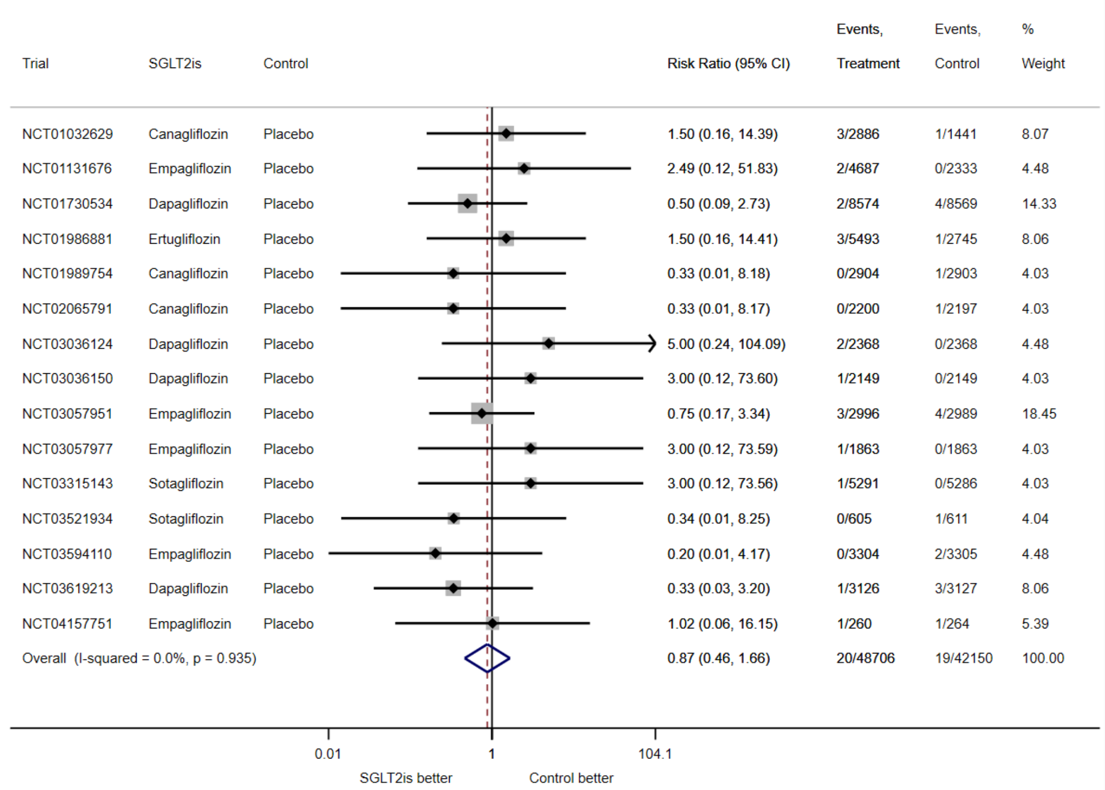

System 7-Gastrointestinal Disorders-Figure S57 Meta-analysis of SGLT2is and Ileus

Trial	SGLT2is	Control	Risk Ratio (95% CI)	Events, Treatment	Events, Control	% Weight
NCT01032629	Canagliflozin	Placebo	1.50 (0.06, 36.76)	1/2886	0/1441	7.85
NCT01131676	Empagliflozin	Placebo	0.17 (0.02, 1.59)	1/4687	3/2333	15.69
NCT01730534	Dapagliflozin	Placebo	1.00 (0.06, 15.98)	1/8574	1/8569	10.46
NCT01986881	Ertugliflozin	Placebo	1.50 (0.06, 36.80)	1/5493	0/2745	7.84
NCT03036124	Dapagliflozin	Placebo	0.33 (0.01, 8.18)	0/2368	1/2368	7.85
NCT03057951	Empagliflozin	Placebo	3.99 (0.45, 35.68)	4/2996	1/2989	16.74
NCT03057977	Empagliflozin	Placebo	0.33 (0.01, 8.18)	0/1863	1/1863	7.85
NCT03315143	Sotagliflozin	Placebo	0.20 (0.01, 4.16)	0/5291	2/5286	8.72
NCT03521934	Sotagliflozin	Placebo	3.03 (0.12, 74.22)	1/605	0/611	7.85
NCT03594110	Empagliflozin	Placebo	0.14 (0.01, 2.77)	0/3304	3/3305	9.15
Overall (I-squared = 0.0%, p = 0.590)			0.68 (0.28, 1.66)	9/38067	12/31510	100.00

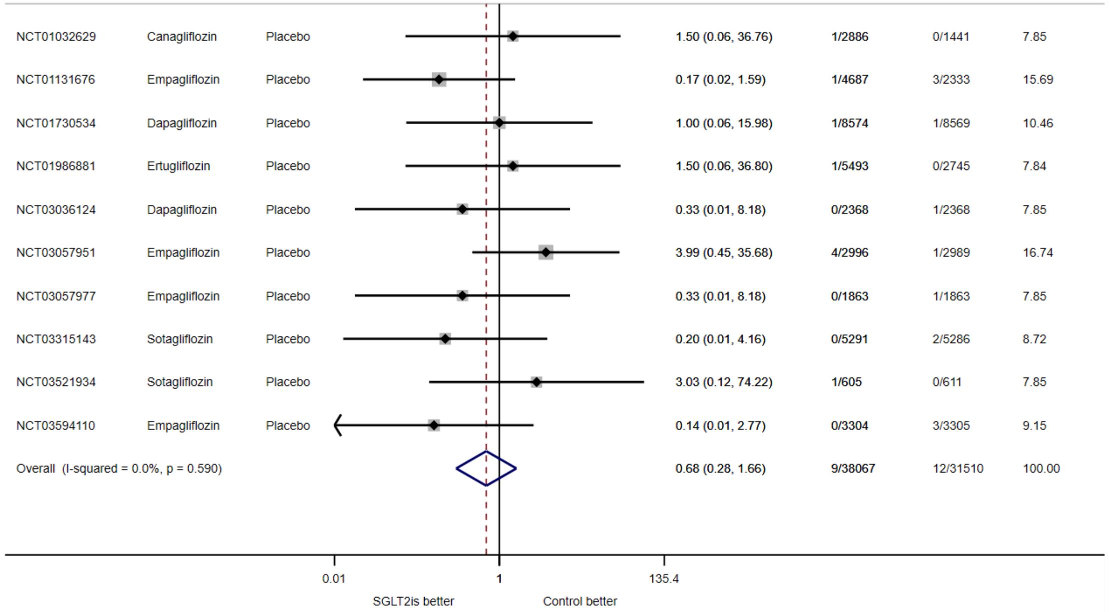

System 7-Gastrointestinal Disorders-Figure S58 Meta-analysis of SGLT2is and Ileus paralytic

Trial	SGLT2is	Control	Risk Ratio (95% CI)	Events, Treatment	Events, Control	% Weight
NCT01131676	Empagliflozin	Placebo	3.49 (0.18, 67.44)	3/4687	0/2333	14.89
NCT01730534	Dapagliflozin	Placebo	1.00 (0.14, 7.09)	2/8574	2/8569	34.03
NCT01989754	Canagliflozin	Placebo	0.33 (0.01, 8.18)	0/2904	1/2903	12.76
NCT02384941	Sotagliflozin	Placebo	0.17 (0.01, 4.17)	0/525	1/268	12.79
NCT03036150	Dapagliflozin	Placebo	0.33 (0.01, 8.18)	0/2149	1/2149	12.76
NCT03619213	Dapagliflozin	Placebo	3.00 (0.12, 73.64)	1/3126	0/3127	12.76
Overall (I-squared = 0.0%, p = 0.681)			0.83 (0.27, 2.62)	6/21965	5/19349	100.00

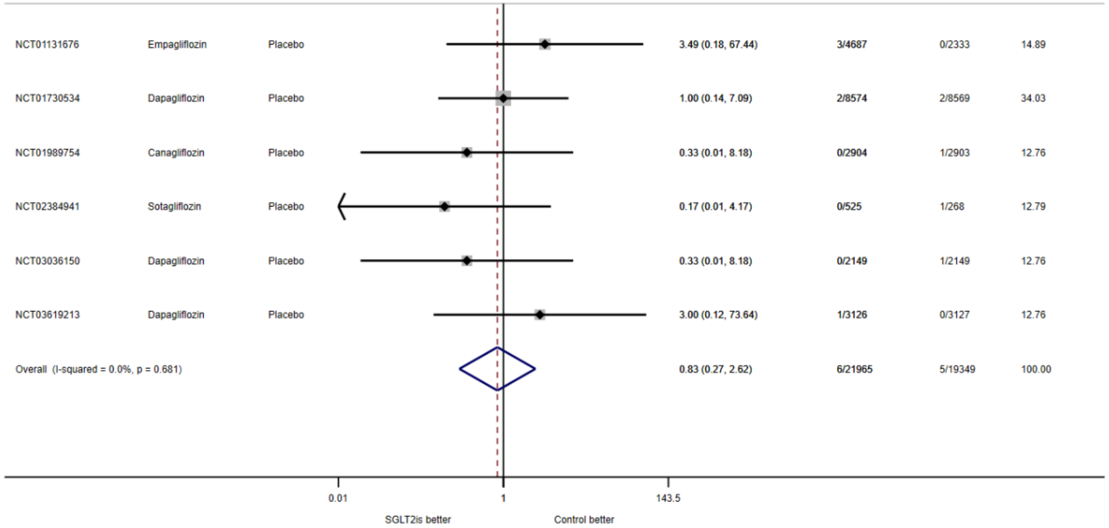

System 7-Gastrointestinal Disorders-Figure S59 Meta-analysis of SGLT2is and Impaired gastric emptying

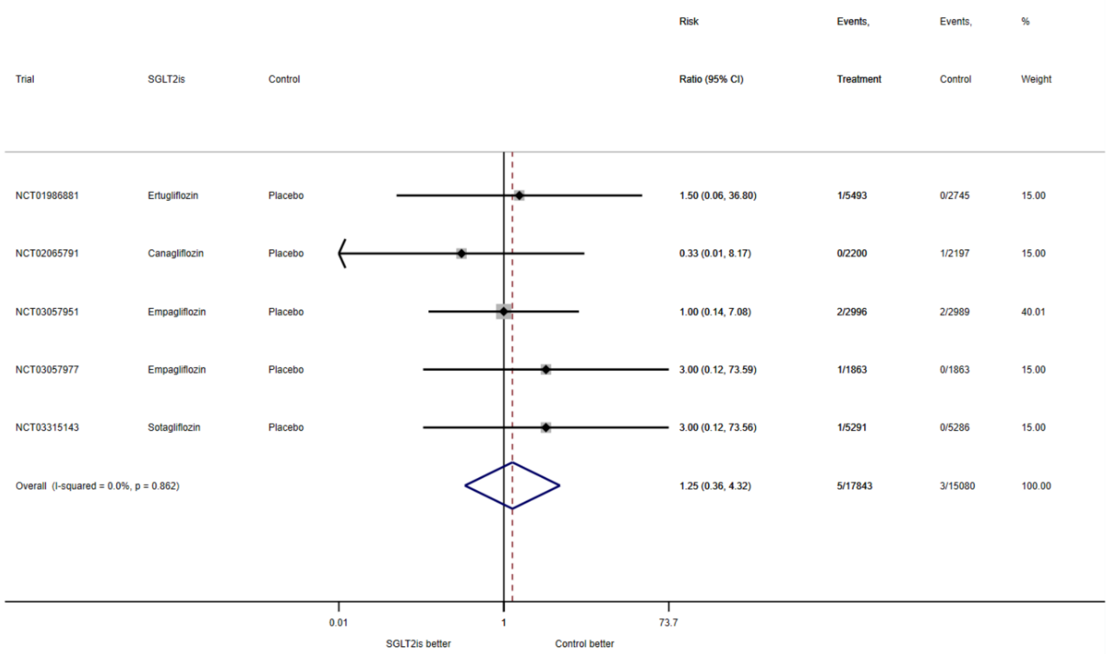

System 7-Gastrointestinal Disorders-Figure S60 Meta-analysis of SGLT2is and Incarcerated inguinal hernia

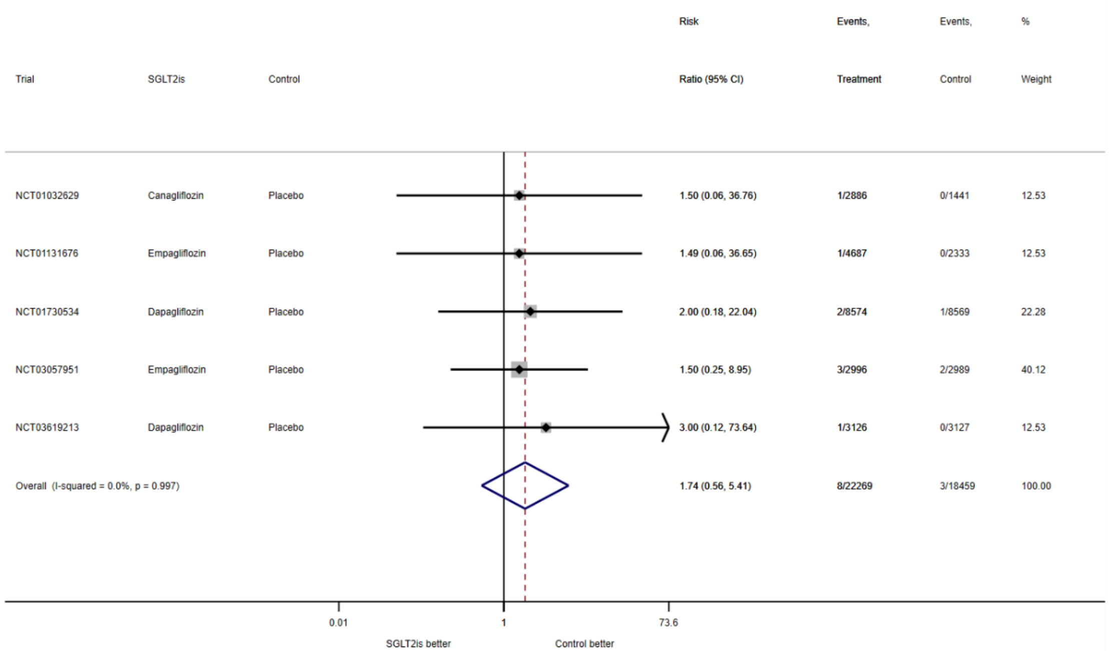

System 7-Gastrointestinal Disorders-Figure S61 Meta-analysis of SGLT2is and Incarcerated umbilical hernia

Trial	SGLT2is	Control	Risk Ratio (95% CI)	Events, Treatment	Events, Control	% Weight
NCT01032629	Canagliflozin	Placebo	3.50 (0.18, 67.64)	3/2886	0/1441	1.66
NCT01106677	Canagliflozin	Sitagliptin	2.49 (0.12, 51.80)	2/735	0/366	1.58
NCT01131676	Empagliflozin	Placebo	1.49 (0.40, 5.51)	9/4687	3/2333	8.52
NCT01730534	Dapagliflozin	Placebo	0.77 (0.34, 1.75)	10/8574	13/8569	21.40
NCT01986881	Ertugliflozin	Placebo	4.50 (0.57, 35.48)	9/5493	1/2745	3.41
NCT01989754	Canagliflozin	Placebo	0.50 (0.09, 2.73)	2/2904	4/2903	5.05
NCT02065791	Canagliflozin	Placebo	3.99 (0.45, 35.71)	4/2200	1/2197	3.03
NCT03036124	Dapagliflozin	Placebo	1.80 (0.60, 5.36)	9/2368	5/2368	12.19
NCT03036150	Dapagliflozin	Placebo	1.00 (0.14, 7.09)	2/2149	2/2149	3.79
NCT03057951	Empagliflozin	Placebo	0.89 (0.34, 2.30)	8/2996	9/2989	16.06
NCT03057977	Empagliflozin	Placebo	15.00 (0.86, 262.44)	7/1863	0/1863	1.77
NCT03315143	Sotagliflozin	Placebo	0.33 (0.03, 3.20)	1/5291	3/5286	2.84
NCT03521934	Sotagliflozin	Placebo	1.01 (0.06, 16.11)	1/605	1/611	1.89
NCT03594110	Empagliflozin	Placebo	0.75 (0.17, 3.35)	3/3304	4/3305	6.49
NCT03619213	Dapagliflozin	Placebo	1.20 (0.37, 3.93)	6/3126	5/3127	10.33
Overall (I-squared = 0.0%, p = 0.668)			1.15 (0.79, 1.69)	76/49181	51/42252	100.00

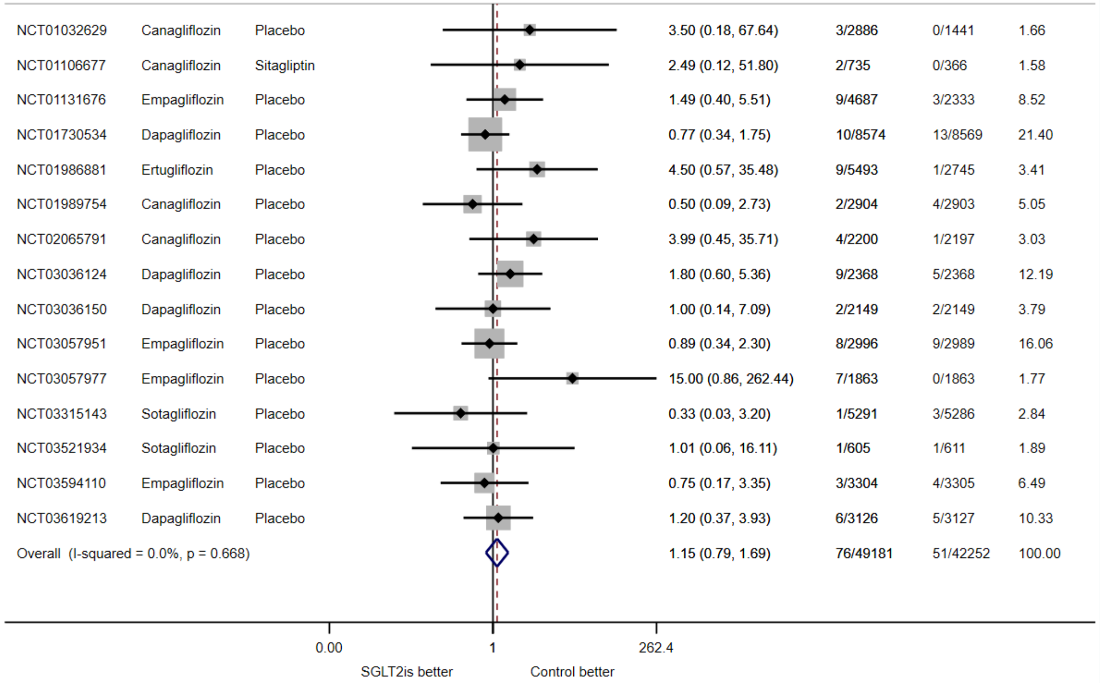

System 7-Gastrointestinal Disorders-Figure S62 Meta-analysis of SGLT2is and Inguinal hernia

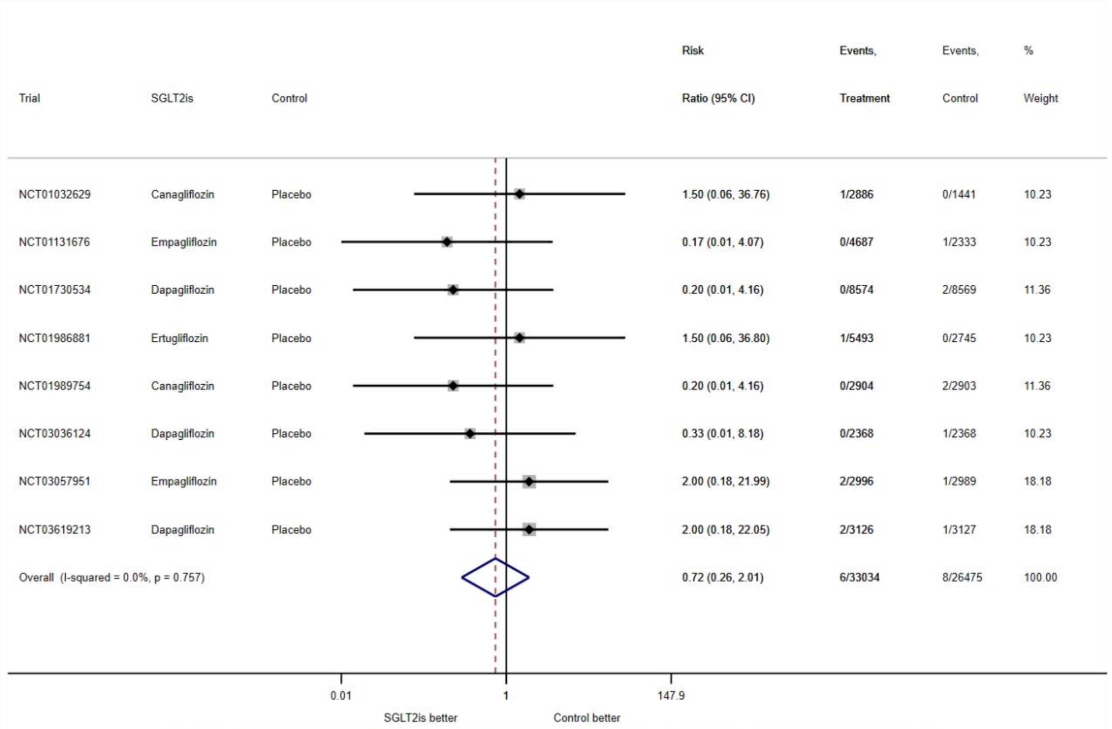

System 7-Gastrointestinal Disorders-Figure S63 Meta-analysis of SGLT2is and Intestinal haemorrhage

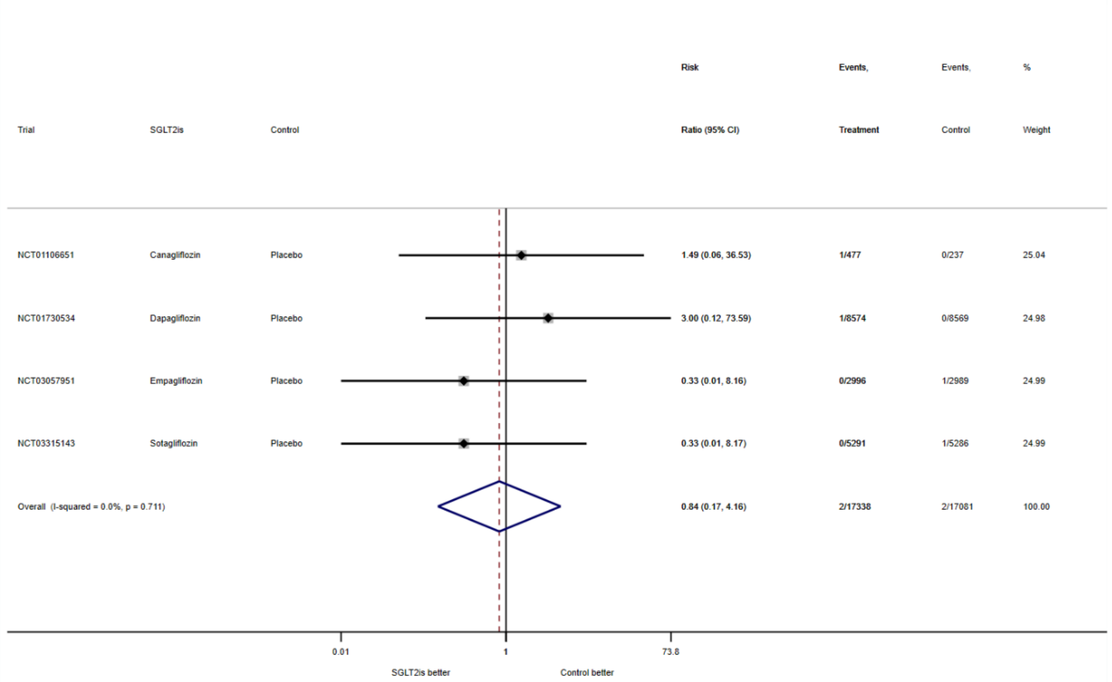

System 7-Gastrointestinal Disorders-Figure S64 Meta-analysis of SGLT2is and Intestinal infarction

Trial	SGLT2is	Control	Risk Ratio (95% CI)	Events, Treatment	Events, Control	% Weight
NCT01032629	Canagliflozin	Placebo	1.50 (0.06, 36.76)	1/2886	0/1441	6.20
NCT01131676	Empagliflozin	Placebo	0.50 (0.07, 3.53)	2/4687	2/2333	16.53
NCT01730534	Dapagliflozin	Placebo	4.00 (0.45, 35.76)	4/8574	1/8569	13.22
NCT01986881	Ertugliflozin	Placebo	1.50 (0.06, 36.80)	1/5493	0/2745	6.20
NCT01989754	Canagliflozin	Placebo	0.14 (0.01, 2.76)	0/2904	3/2903	7.23
NCT03036124	Dapagliflozin	Placebo	3.00 (0.12, 73.61)	1/2368	0/2368	6.20
NCT03057951	Empagliflozin	Placebo	4.99 (0.24, 103.86)	2/2996	0/2989	6.88
NCT03057977	Empagliflozin	Placebo	0.20 (0.01, 4.16)	0/1863	2/1863	6.89
NCT03315143	Sotagliflozin	Placebo	1.00 (0.06, 15.97)	1/5291	1/5286	8.26
NCT03521934	Sotagliflozin	Placebo	5.05 (0.24, 104.96)	2/605	0/611	6.89
NCT03619213	Dapagliflozin	Placebo	0.14 (0.01, 2.77)	0/3126	3/3127	7.23
NCT04157751	Empagliflozin	Placebo	1.02 (0.06, 16.15)	1/260	1/264	8.29
Overall (I-squared = 0.0%, p = 0.607)			1.02 (0.46, 2.26)	15/41053	13/34499	100.00

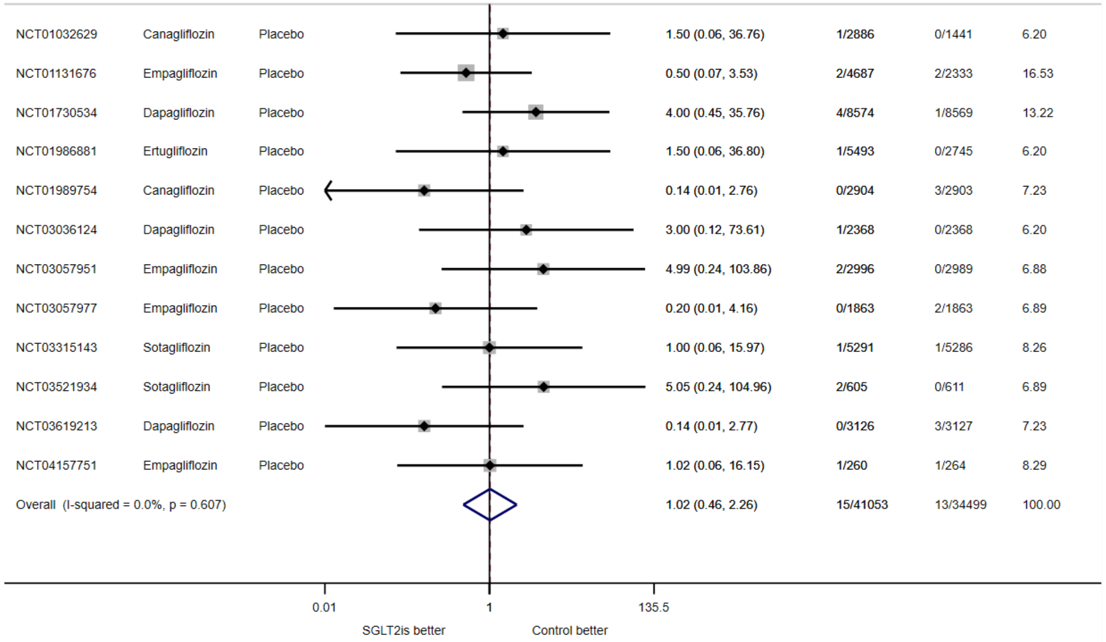

System 7-Gastrointestinal Disorders-Figure S65 Meta-analysis of SGLT2is and Intestinal ischaemia

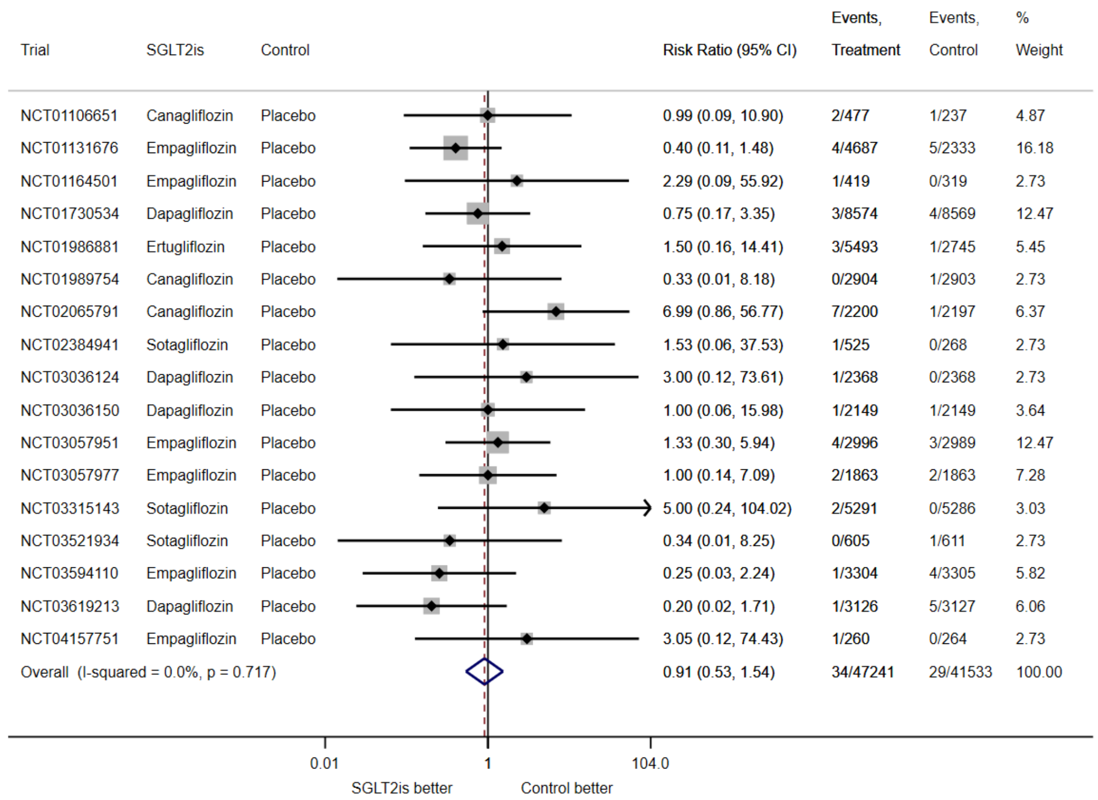

System 7-Gastrointestinal Disorders-Figure S66 Meta-analysis of SGLT2is and Intestinal obstruction

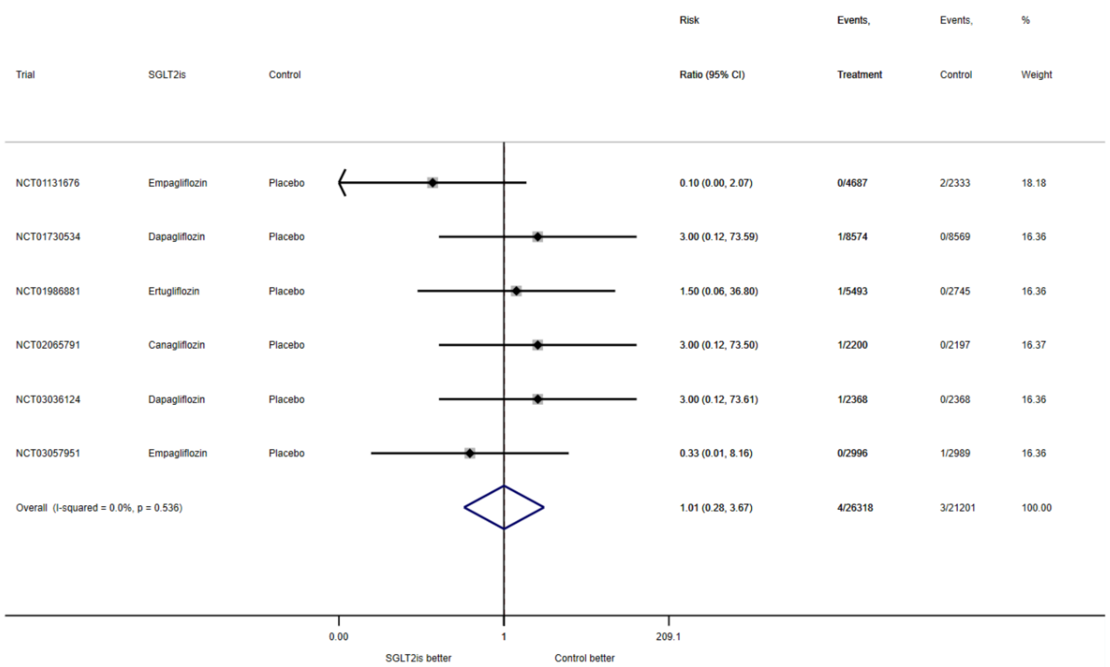

System 7-Gastrointestinal Disorders-Figure S67 Meta-analysis of SGLT2is and Intestinal perforation

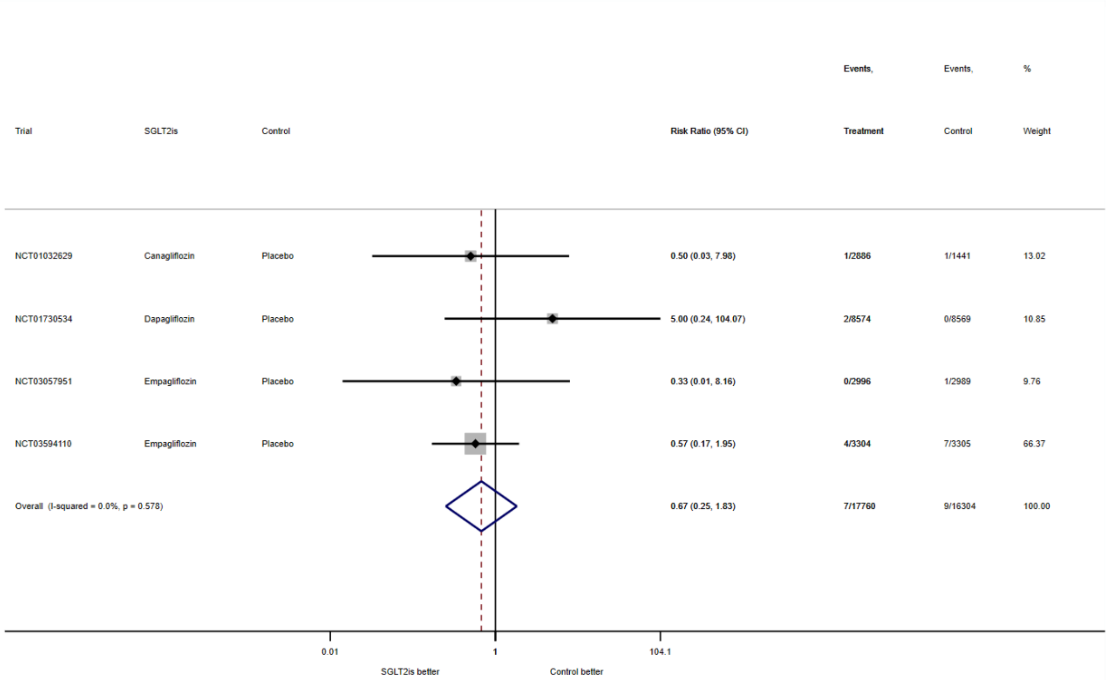

System 7-Gastrointestinal Disorders-Figure S68 Meta-analysis of SGLT2is and Intestinal polyp

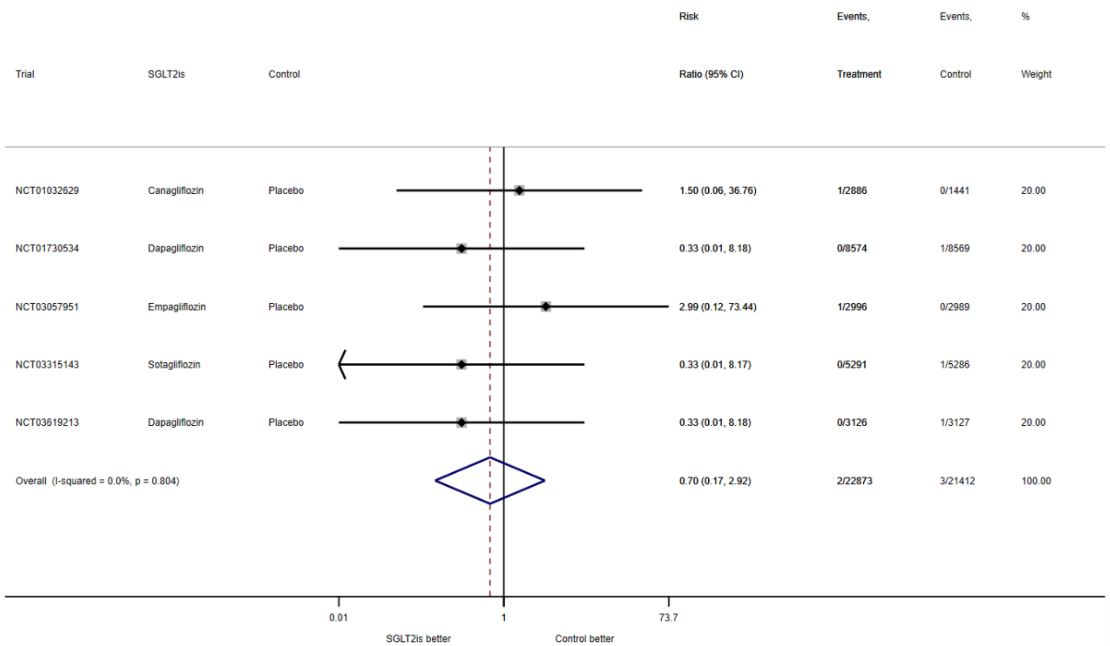

System 7-Gastrointestinal Disorders-Figure S69 Meta-analysis of SGLT2is and Intra-abdominal haemorrhage

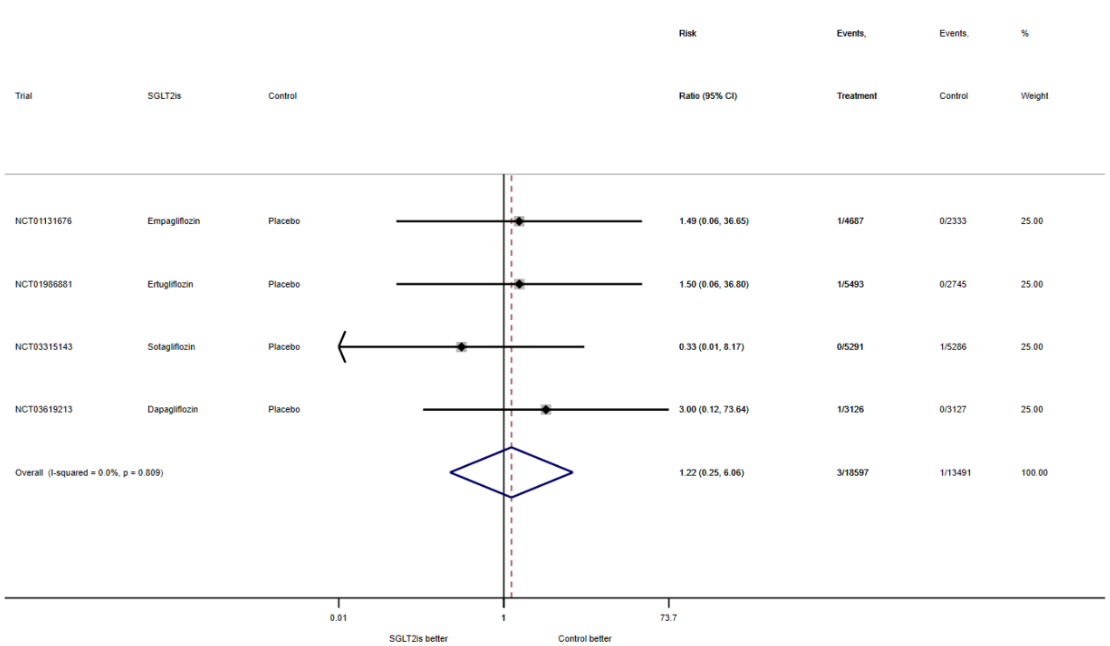

System 7-Gastrointestinal Disorders-Figure S70 Meta-analysis of SGLT2is and Irritable bowel syndrome

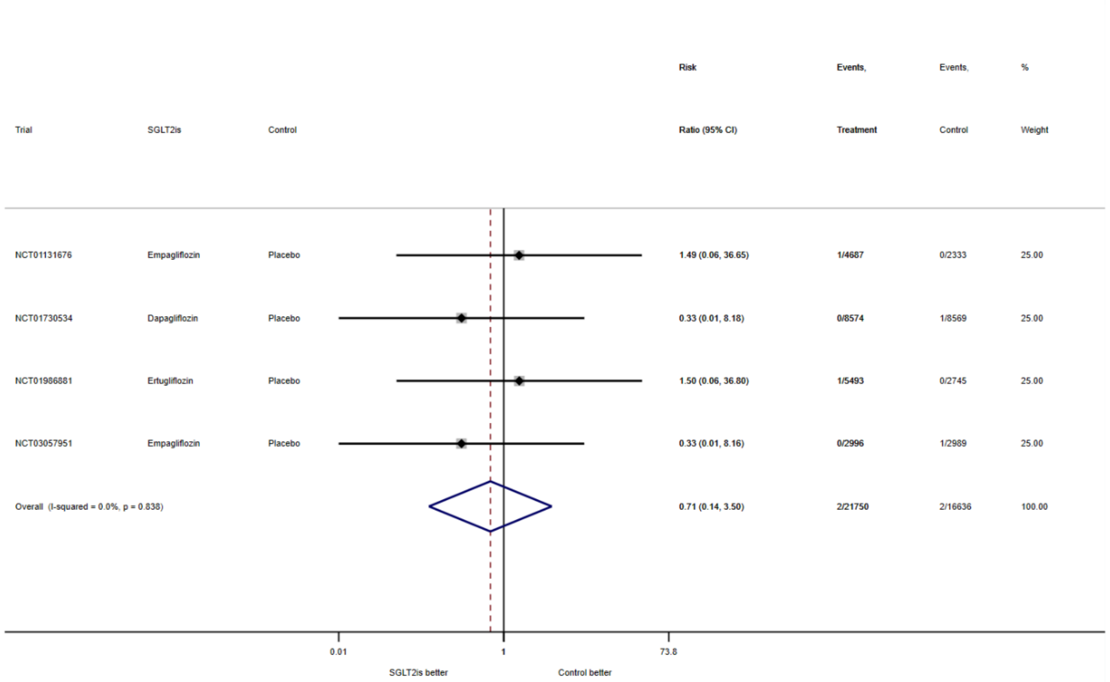

System 7-Gastrointestinal Disorders-Figure S71 Meta-analysis of SGLT2is and Large intestinal ulcer

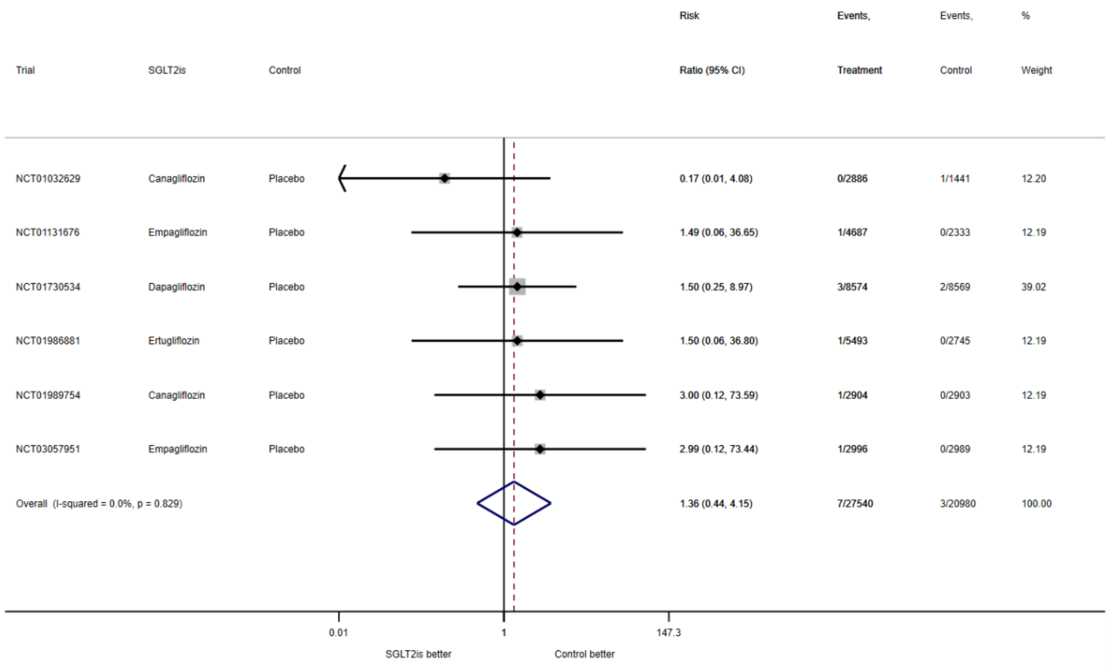

System 7-Gastrointestinal Disorders-Figure S72 Meta-analysis of SGLT2is and Large intestine perforation

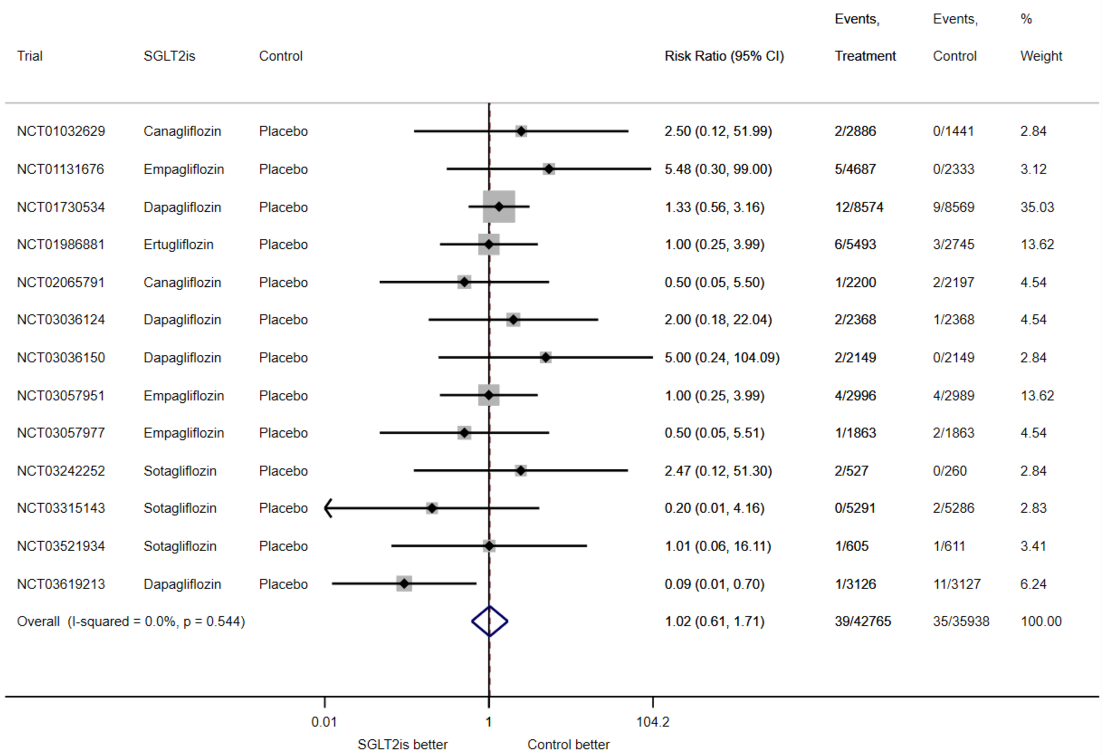

System 7-Gastrointestinal Disorders-Figure S73 Meta-analysis of SGLT2is and Large intestine polyp

Trial	SGLT2is	Control	Risk Ratio (95% CI)	Events, Treatment	Events, Control	% Weight
NCT01032629	Canagliflozin	Placebo	1.50 (0.06, 36.76)	1/2886	0/1441	3.70
NCT01106651	Canagliflozin	Placebo	2.49 (0.12, 51.65)	2/477	0/237	4.12
NCT01730534	Dapagliflozin	Placebo	1.00 (0.14, 7.09)	2/8574	2/8569	9.85
NCT01986881	Ertugliflozin	Placebo	3.50 (0.18, 67.71)	3/5493	0/2745	4.31
NCT01989754	Canagliflozin	Placebo	1.00 (0.06, 15.97)	1/2904	1/2903	4.93
NCT02065791	Canagliflozin	Placebo	3.00 (0.12, 73.50)	1/2200	0/2197	3.70
NCT03036124	Dapagliflozin	Placebo	2.00 (0.18, 22.04)	2/2368	1/2368	6.57
NCT03036150	Dapagliflozin	Placebo	3.00 (0.12, 73.60)	1/2149	0/2149	3.70
NCT03057951	Empagliflozin	Placebo	0.25 (0.03, 2.23)	1/2996	4/2989	7.89
NCT03242252	Sotagliflozin	Placebo	1.48 (0.06, 36.28)	1/527	0/260	3.70
NCT03315143	Sotagliflozin	Placebo	3.00 (0.12, 73.56)	1/5291	0/5286	3.70
NCT03594110	Empagliflozin	Placebo	1.25 (0.34, 4.65)	5/3304	4/3305	21.92
NCT03619213	Dapagliflozin	Placebo	1.25 (0.34, 4.65)	5/3126	4/3127	21.92
Overall (I-squared = 0.0%, p = 0.986)			1.32 (0.71, 2.44)	26/42295	16/37576	100.00

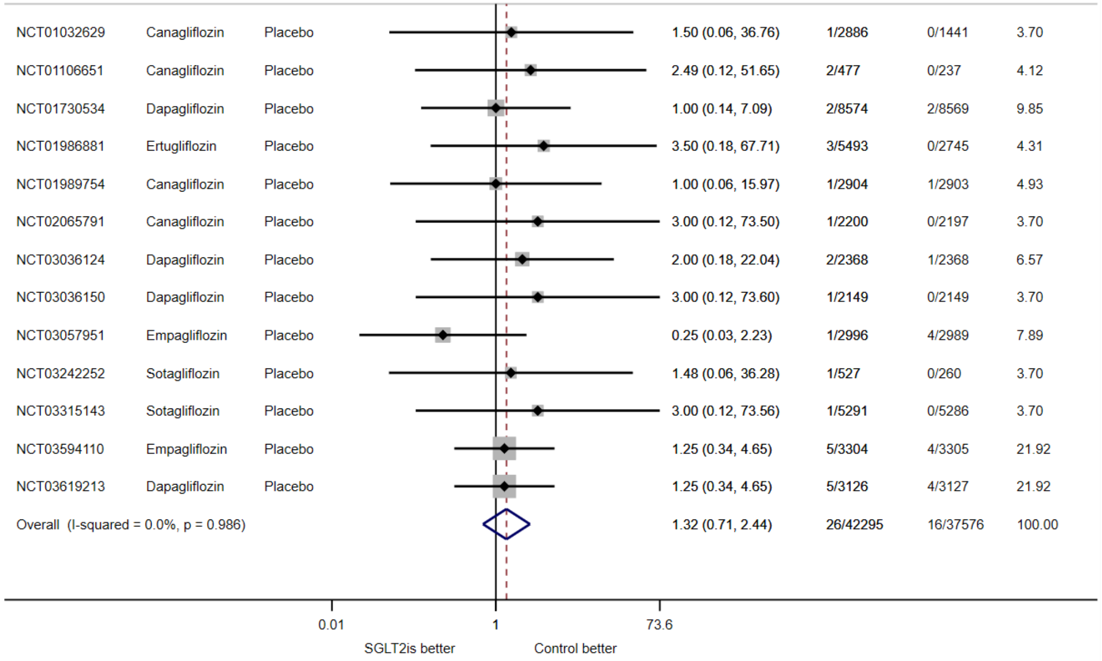

System 7-Gastrointestinal Disorders-Figure S74 Meta-analysis of SGLT2is and Lower gastrointestinal haemorrhage

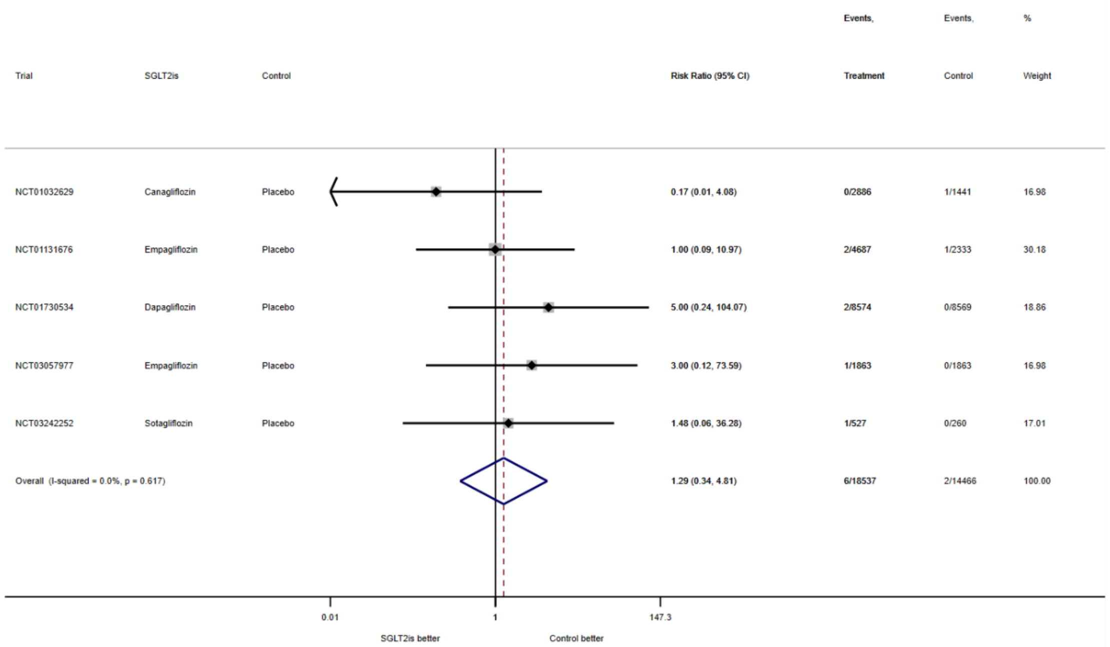

System 7-Gastrointestinal Disorders-Figure S75 Meta-analysis of SGLT2is and Mallory-weiss syndrome

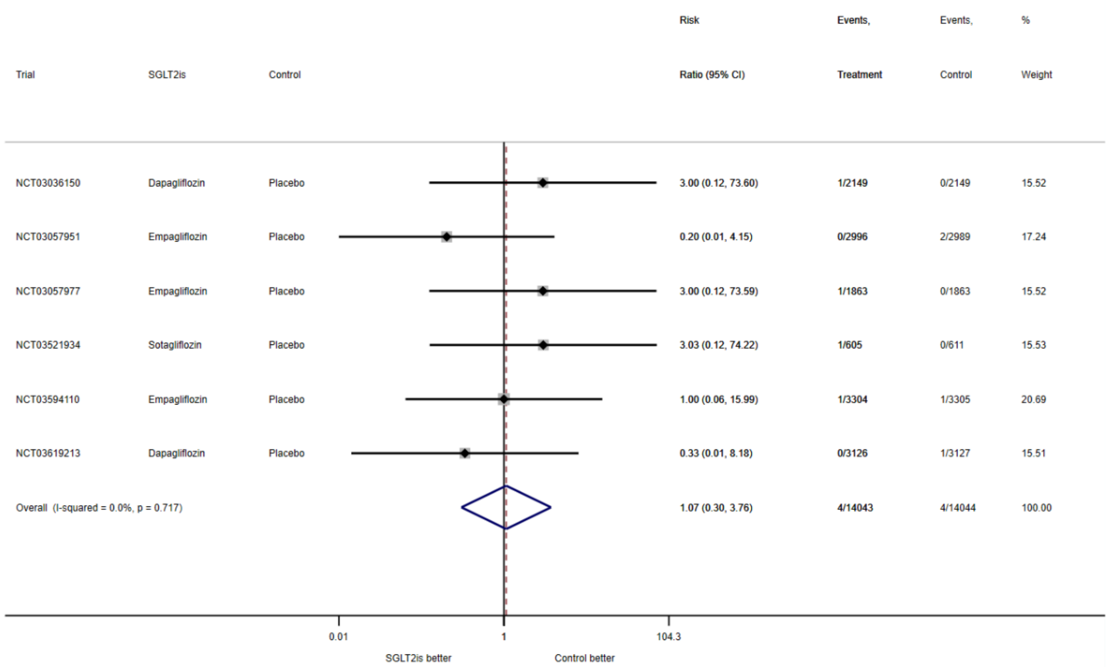

System 7-Gastrointestinal Disorders-Figure S76 Meta-analysis of SGLT2is and Mechanical ileus

Trial	SGLT2is	Control	Risk Ratio (95% CI)	Events, Treatment	Events, Control	% Weight
NCT01131676	Empagliflozin	Placebo	2.49 (0.12, 51.83)	2/4687	0/2333	9.17
NCT01730534	Dapagliflozin	Placebo	3.00 (0.12, 73.59)	1/8574	0/8569	8.26
NCT01986881	Ertugliflozin	Placebo	1.50 (0.06, 36.80)	1/5493	0/2745	8.26
NCT01989754	Canagliflozin	Placebo	5.00 (0.24, 104.07)	2/2904	0/2903	9.17
NCT02065791	Canagliflozin	Placebo	3.00 (0.12, 73.50)	1/2200	0/2197	8.26
NCT03036124	Dapagliflozin	Placebo	0.33 (0.01, 8.18)	0/2368	1/2368	8.26
NCT03057951	Empagliflozin	Placebo	1.00 (0.06, 15.94)	1/2996	1/2989	11.01
NCT03315143	Sotagliflozin	Placebo	3.00 (0.12, 73.56)	1/5291	0/5286	8.26
NCT03594110	Empagliflozin	Placebo	2.00 (0.18, 22.05)	2/3304	1/3305	14.68
NCT03619213	Dapagliflozin	Placebo	0.50 (0.05, 5.51)	1/3126	2/3127	14.68
Overall (I-squared = 0.0%, p = 0.963)			1.56 (0.62, 3.92)	12/40943	5/35822	100.00

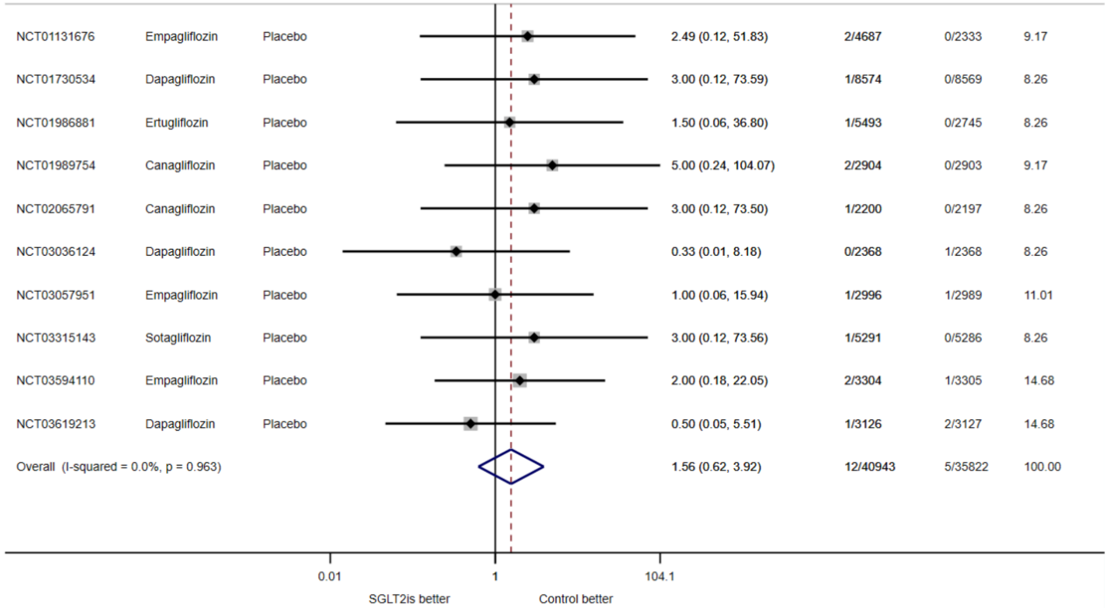

System 7-Gastrointestinal Disorders-Figure S77 Meta-analysis of SGLT2is and Melaena

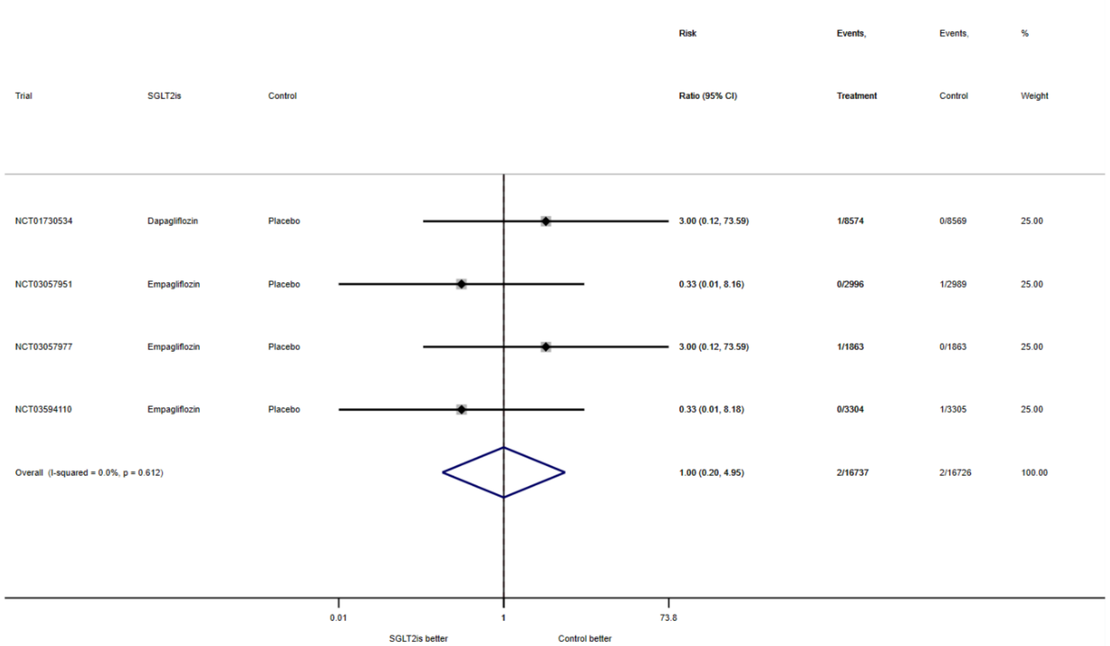

System 7-Gastrointestinal Disorders-Figure S78 Meta-analysis of SGLT2is and Mesenteric artery stenosis

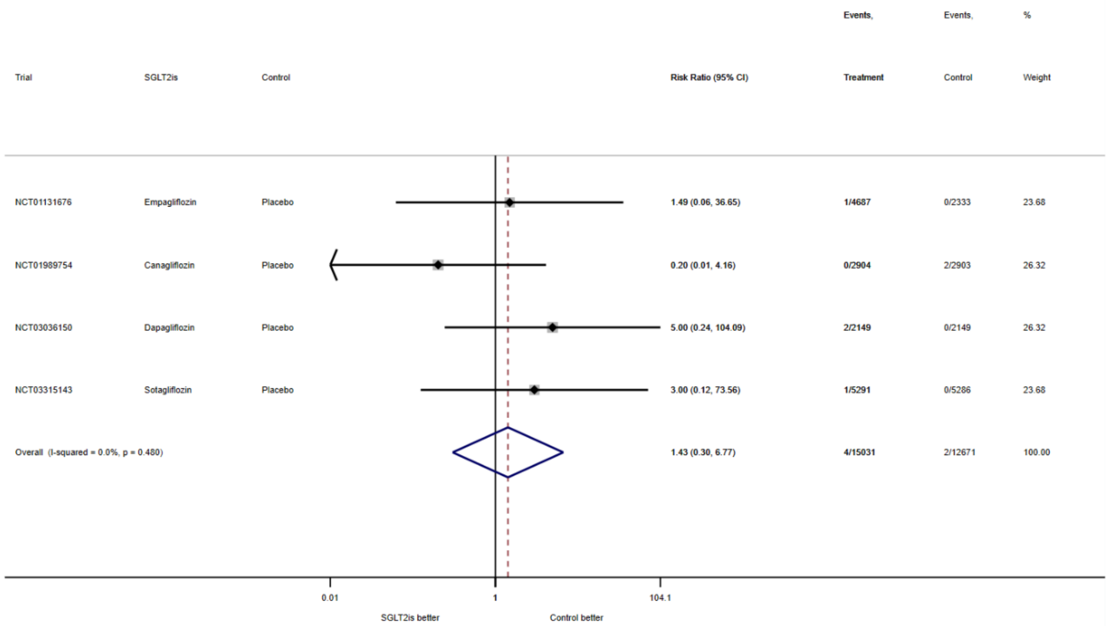

System 7-Gastrointestinal Disorders-Figure S79 Meta-analysis of SGLT2is and Mesenteric vein thrombosis

Trial	SGLT2is	Control	Risk Ratio (95% CI)	Events, Treatment	Events, Control	% Weight
NCT01031680	Dapagliflozin	Placebo	0.33 (0.01, 8.20)	0/460	1/462	5.38
NCT01131676	Empagliflozin	Placebo	1.49 (0.30, 7.39)	6/4687	2/2333	21.50
NCT01730534	Dapagliflozin	Placebo	1.00 (0.14, 7.09)	2/8574	2/8569	14.32
NCT01986881	Ertugliflozin	Placebo	0.25 (0.02, 2.75)	1/5493	2/2745	9.55
NCT03036124	Dapagliflozin	Placebo	1.00 (0.06, 15.98)	1/2368	1/2368	7.16
NCT03036150	Dapagliflozin	Placebo	1.00 (0.06, 15.98)	1/2149	1/2149	7.16
NCT03057951	Empagliflozin	Placebo	0.20 (0.01, 4.15)	0/2996	2/2989	5.97
NCT03057977	Empagliflozin	Placebo	1.00 (0.06, 15.98)	1/1863	1/1863	7.16
NCT03315143	Sotagliflozin	Placebo	0.14 (0.01, 2.76)	0/5291	3/5286	6.27
NCT03594110	Empagliflozin	Placebo	2.00 (0.18, 22.05)	2/3304	1/3305	9.55
NCT03619213	Dapagliflozin	Placebo	0.20 (0.01, 4.17)	0/3126	2/3127	5.97
Overall (I-squared = 0.0%, p = 0.874)			0.70 (0.33, 1.47)	14/40311	18/35196	100.00

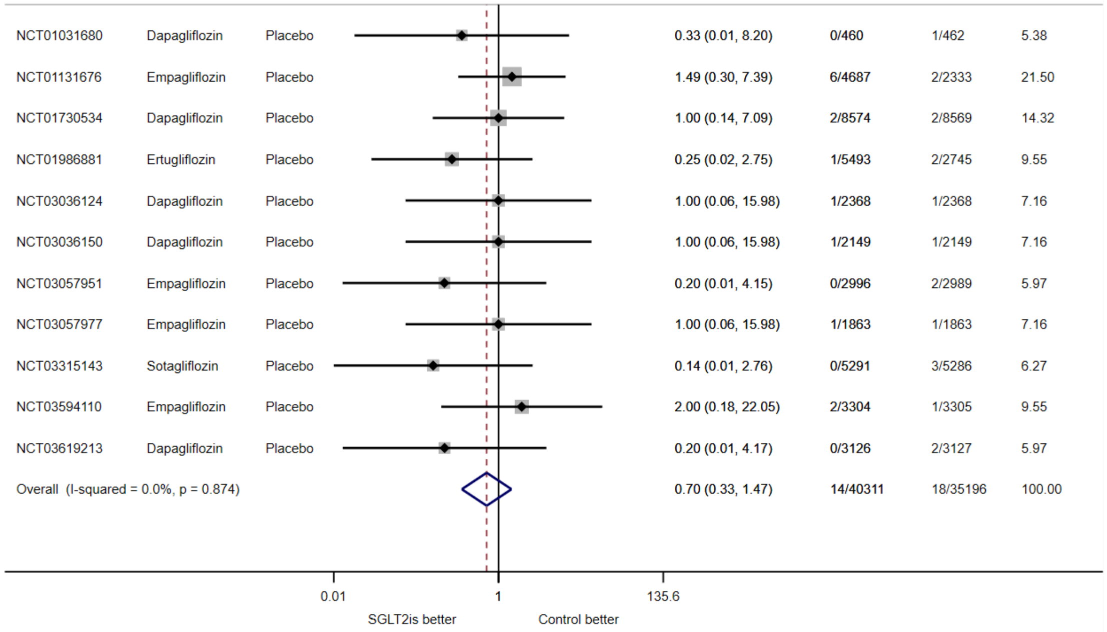

System 7-Gastrointestinal Disorders-Figure S80 Meta-analysis of SGLT2is and Nausea

Trial	SGLT2is	Control	Risk Ratio (95% CI)	Events, Treatment	Events, Control	% Weight
NCT01730534	Dapagliflozin	Placebo	0.50 (0.05, 5.51)	1/8574	2/8569	11.05
NCT01986881	Ertugliflozin	Placebo	0.50 (0.03, 7.99)	1/5493	1/2745	8.29
NCT02065791	Canagliflozin	Placebo	3.00 (0.12, 73.50)	1/2200	0/2197	6.21
NCT03036124	Dapagliflozin	Placebo	3.00 (0.12, 73.61)	1/2368	0/2368	6.21
NCT03036150	Dapagliflozin	Placebo	5.00 (0.24, 104.09)	2/2149	0/2149	6.91
NCT03057951	Empagliflozin	Placebo	0.50 (0.05, 5.50)	1/2996	2/2989	11.05
NCT03057977	Empagliflozin	Placebo	0.20 (0.01, 4.16)	0/1863	2/1863	6.91
NCT03315143	Sotagliflozin	Placebo	1.50 (0.25, 8.96)	3/5291	2/5286	19.89
NCT03594110	Empagliflozin	Placebo	0.33 (0.01, 8.18)	0/3304	1/3305	6.21
NCT03619213	Dapagliflozin	Placebo	0.50 (0.05, 5.51)	1/3126	2/3127	11.05
NCT04157751	Empagliflozin	Placebo	0.34 (0.01, 8.27)	0/260	1/264	6.23
Overall (I-squared = 0.0%, p = 0.884)			0.81 (0.37, 1.81)	11/37624	13/34862	100.00

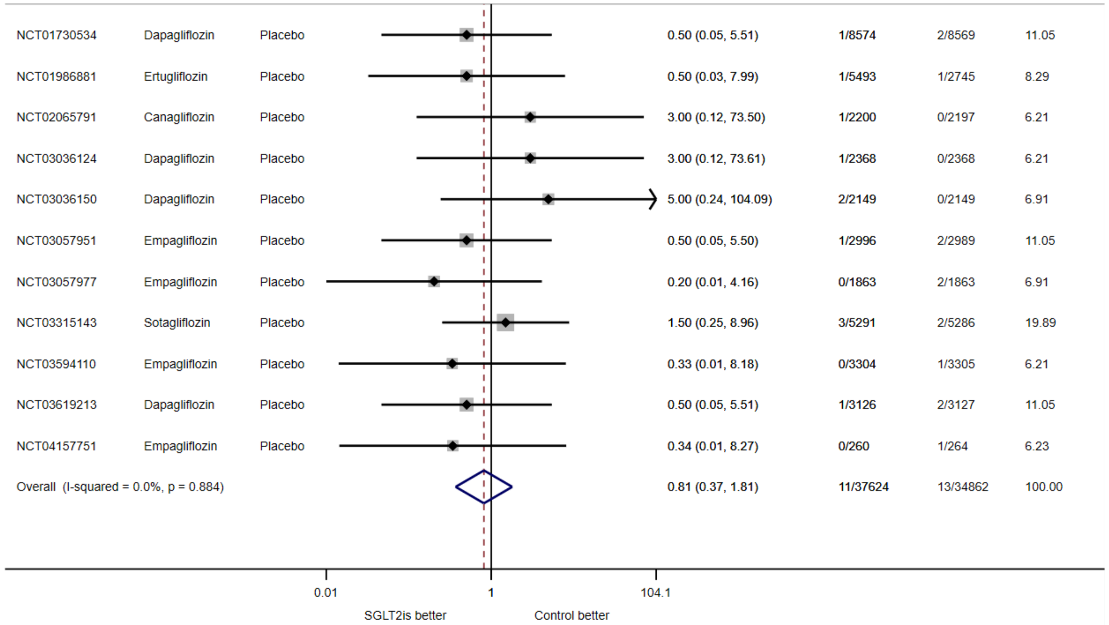

System 7-Gastrointestinal Disorders-Figure S81 Meta-analysis of SGLT2is and Obstructive pancreatitis

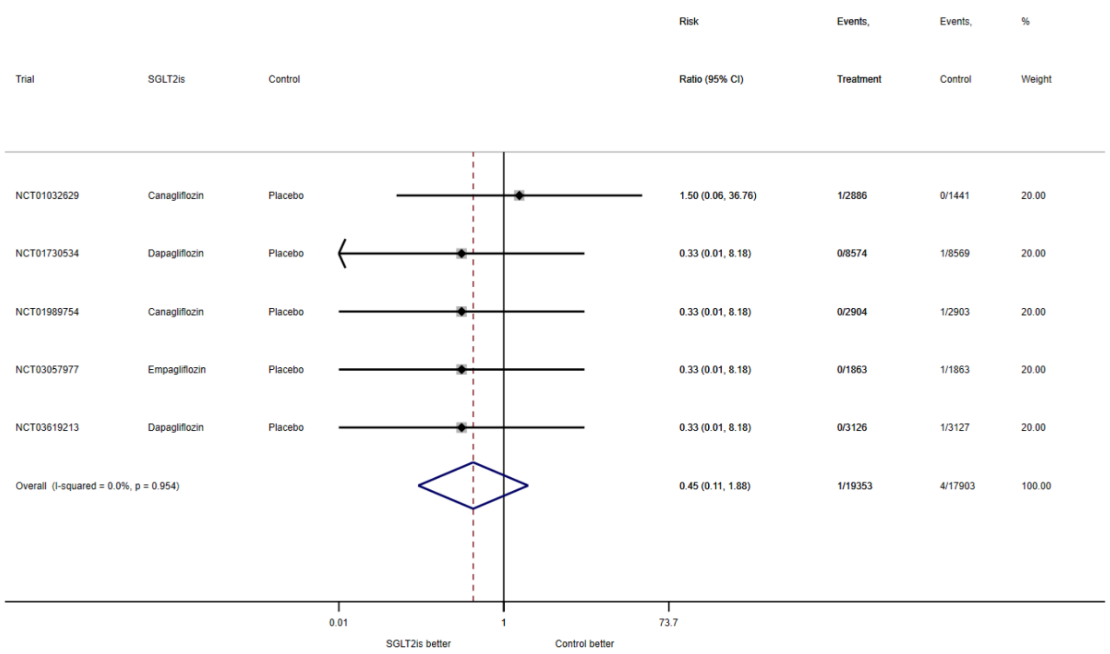

System 7-Gastrointestinal Disorders-Figure S82 Meta-analysis of SGLT2is and Oesophageal obstruction

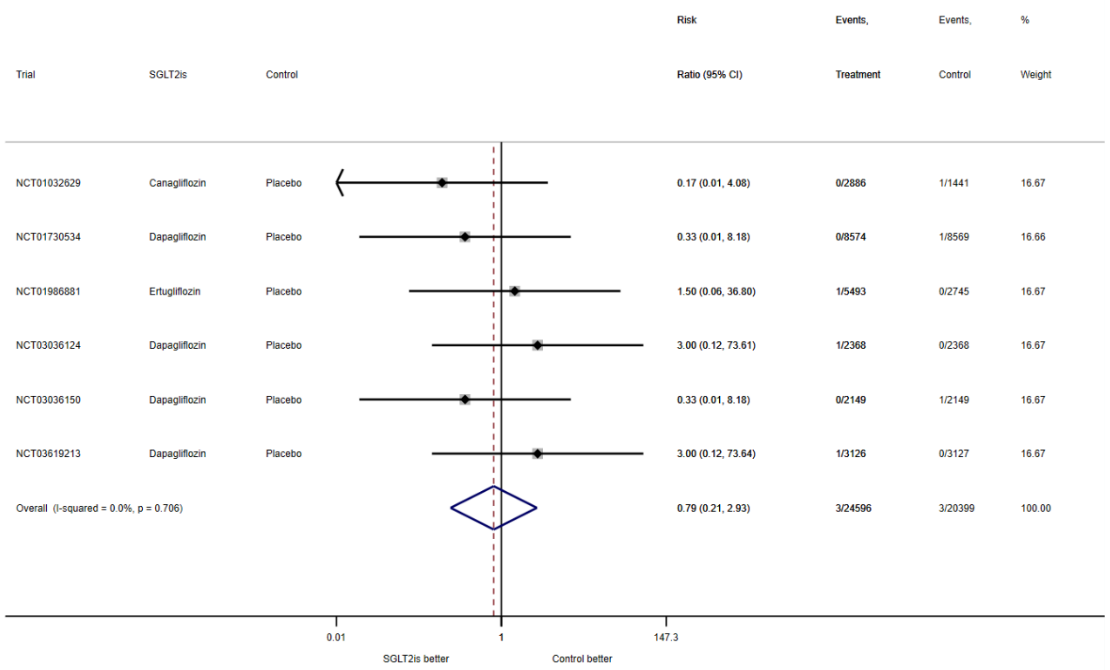

System 7-Gastrointestinal Disorders-Figure S83 Meta-analysis of SGLT2is and Oesophageal ulcer

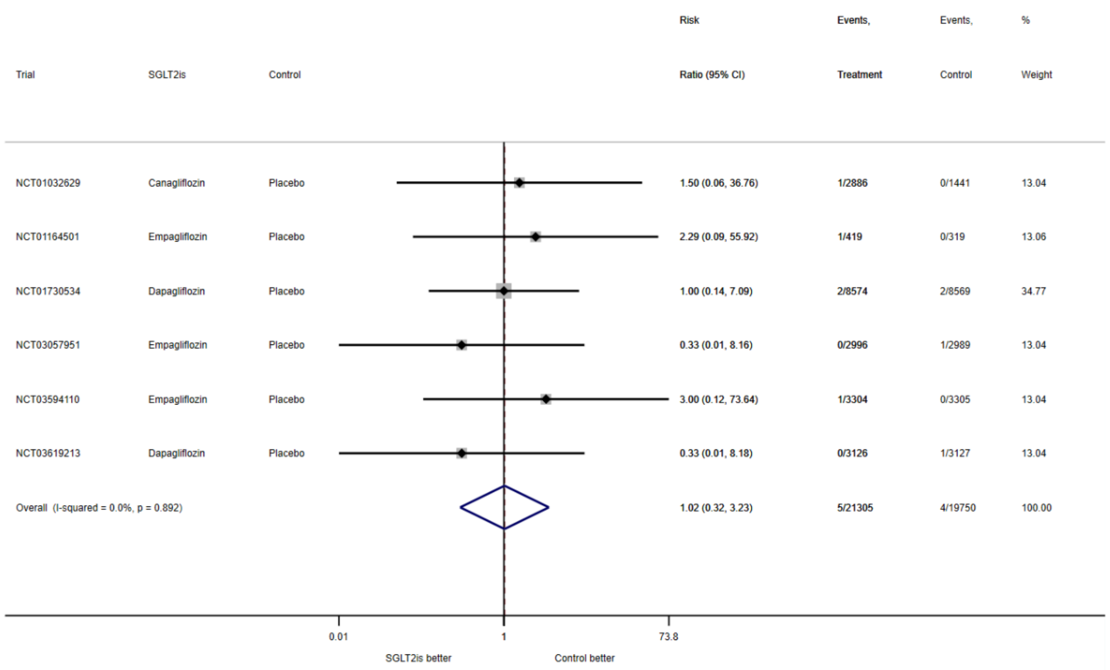

System 7-Gastrointestinal Disorders-Figure S84 Meta-analysis of SGLT2is and Oesophageal varices haemorrhage

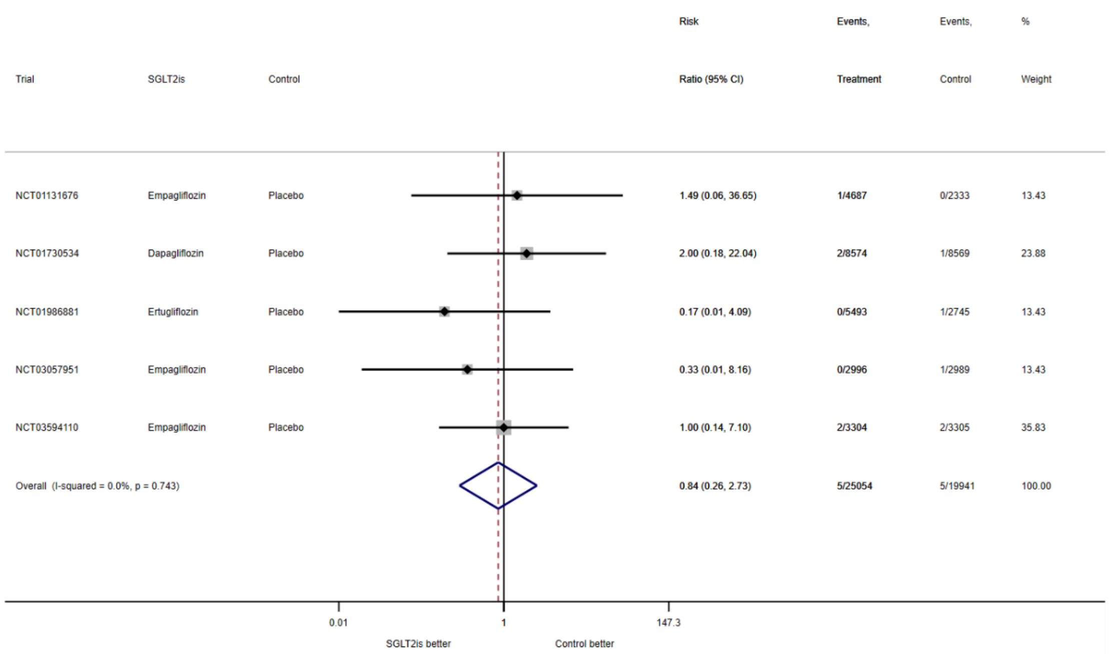

System 7-Gastrointestinal Disorders-Figure S85 Meta-analysis of SGLT2is and Oesophagitis

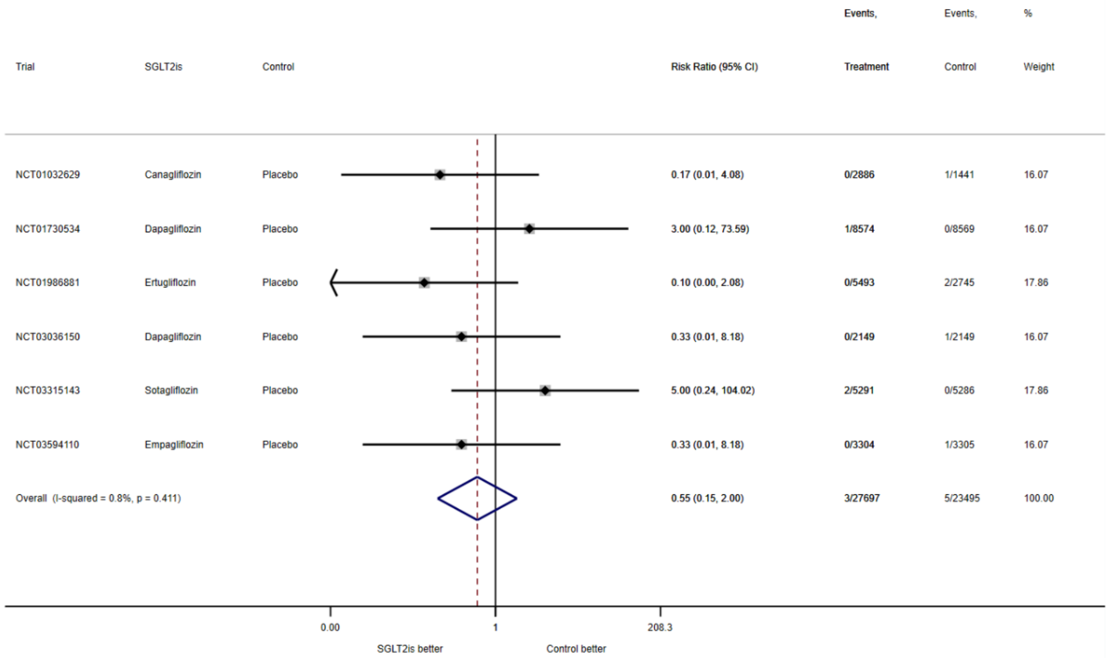

System 7-Gastrointestinal Disorders-Figure S86 Meta-analysis of SGLT2is and Pancreatic cyst

Trial	SGLT2is	Control	Risk Ratio (95% CI)	Events, Treatment	Events, Control	% Weight
NCT01032629	Canagliflozin	Placebo	2.50 (0.29, 21.35)	5/2886	1/1441	4.47
NCT01106651	Canagliflozin	Placebo	1.49 (0.06, 36.53)	1/477	0/237	2.02
NCT01131676	Empagliflozin	Placebo	0.75 (0.21, 2.64)	6/4687	4/2333	12.89
NCT01164501	Empagliflozin	Placebo	0.15 (0.01, 3.16)	0/419	2/319	2.24
NCT01730534	Dapagliflozin	Placebo	1.00 (0.45, 2.22)	12/8574	12/8569	32.23
NCT01986881	Ertugliflozin	Placebo	3.00 (0.36, 24.89)	6/5493	1/2745	4.60
NCT01989754	Canagliflozin	Placebo	0.33 (0.01, 8.18)	0/2904	1/2903	2.01
NCT02033889	Ertugliflozin	Placebo/Glimepiride	0.10 (0.00, 2.11)	0/412	2/209	2.24
NCT02065791	Canagliflozin	Placebo	8.99 (0.48, 166.83)	4/2200	0/2197	2.41
NCT03036124	Dapagliflozin	Placebo	3.00 (0.12, 73.61)	1/2368	0/2368	2.01
NCT03036150	Dapagliflozin	Placebo	1.50 (0.25, 8.97)	3/2149	2/2149	6.44
NCT03057951	Empagliflozin	Placebo	0.33 (0.03, 3.20)	1/2996	3/2989	4.03
NCT03057977	Empagliflozin	Placebo	1.00 (0.06, 15.98)	1/1863	1/1863	2.68
NCT03315143	Sotagliflozin	Placebo	0.75 (0.17, 3.35)	3/5291	4/5286	9.20
NCT03521934	Sotagliflozin	Placebo	0.34 (0.01, 8.25)	0/605	1/611	2.01
NCT03594110	Empagliflozin	Placebo	0.20 (0.02, 1.71)	1/3304	5/3305	4.47
NCT03619213	Dapagliflozin	Placebo	0.33 (0.03, 3.20)	1/3126	3/3127	4.02
Overall (I-squared = 0.0%, p = 0.672)			0.85 (0.54, 1.34)	45/49754	42/42651	100.00

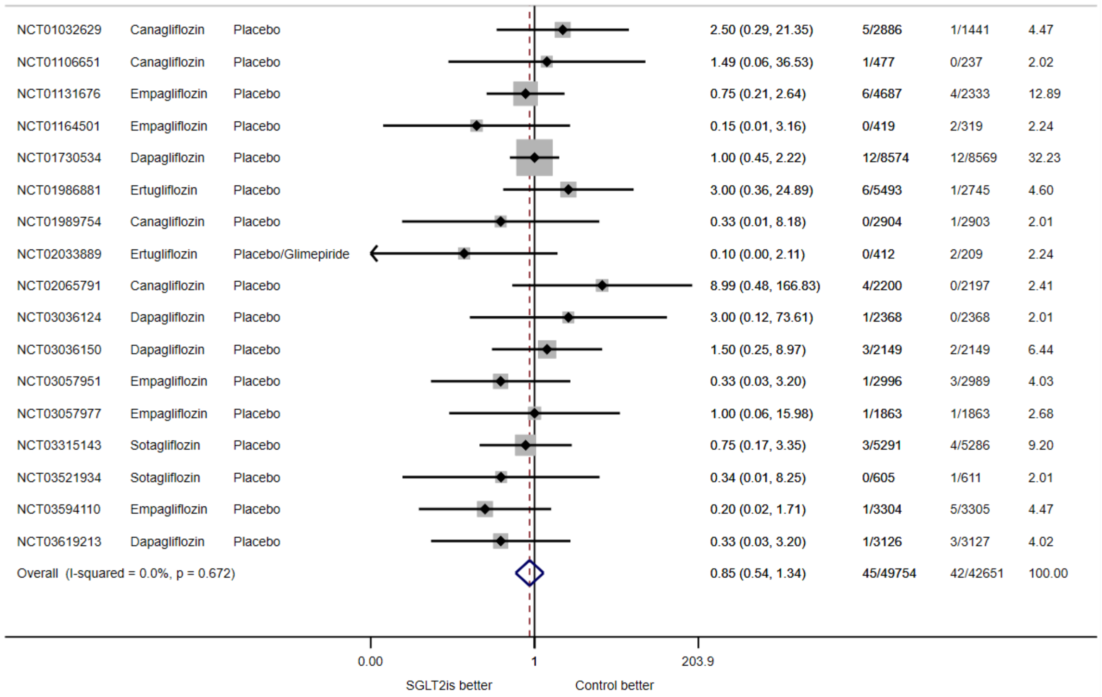

System 7-Gastrointestinal Disorders-Figure S87 Meta-analysis of SGLT2is and Pancreatitis

Trial	SGLT2is	Control	Risk Ratio (95% CI)	Events, Treatment	Events, Control	% Weight
NCT01032629	Canagliflozin	Placebo	2.50 (0.29, 21.35)	5/2886	1/1441	3.05
NCT01131676	Empagliflozin	Placebo	0.66 (0.15, 2.96)	4/4687	3/2333	6.27
NCT01164501	Empagliflozin	Placebo	2.29 (0.09, 55.92)	1/419	0/319	1.37
NCT01730534	Dapagliflozin	Placebo	0.87 (0.41, 1.82)	13/8574	15/8569	25.49
NCT01986881	Ertugliflozin	Placebo	1.62 (0.53, 4.98)	13/5493	4/2745	11.19
NCT01989754	Canagliflozin	Placebo	1.50 (0.25, 8.97)	3/2904	2/2903	4.39
NCT02065791	Canagliflozin	Placebo	1.00 (0.20, 4.94)	3/2200	3/2197	5.49
NCT03036124	Dapagliflozin	Placebo	1.50 (0.42, 5.31)	6/2368	4/2368	8.79
NCT03036150	Dapagliflozin	Placebo	5.00 (0.58, 42.76)	5/2149	1/2149	3.05
NCT03057951	Empagliflozin	Placebo	1.50 (0.25, 8.95)	3/2996	2/2989	4.39
NCT03057977	Empagliflozin	Placebo	0.50 (0.05, 5.51)	1/1863	2/1863	2.44
NCT03315143	Sotagliflozin	Placebo	0.42 (0.15, 1.18)	5/5291	12/5286	12.91
NCT03521934	Sotagliflozin	Placebo	0.34 (0.01, 8.25)	0/605	1/611	1.37
NCT03594110	Empagliflozin	Placebo	3.00 (0.12, 73.64)	1/3304	0/3305	1.37
NCT03619213	Dapagliflozin	Placebo	0.29 (0.06, 1.37)	2/3126	7/3127	5.69
NCT04157751	Empagliflozin	Placebo	0.34 (0.01, 8.27)	0/260	1/264	1.37
NCT04350593	Dapagliflozin	Placebo	0.33 (0.01, 8.21)	0/613	1/616	1.37
Overall (I-squared = 0.0%, p = 0.734)			0.92 (0.63, 1.34)	65/49738	59/43085	100.00

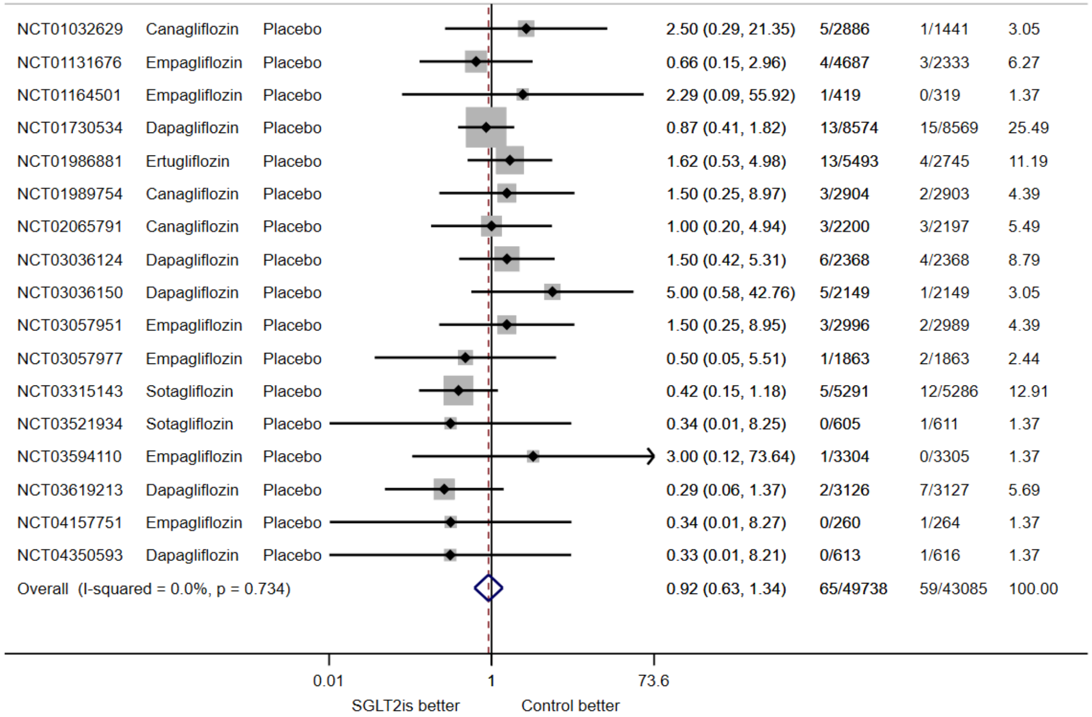

System 7-Gastrointestinal Disorders-Figure S88 Meta-analysis of SGLT2is and Pancreatitis acute

Trial	SGLT2is	Control	Risk Ratio (95% CI)	Events, Treatment	Events, Control	% Weight
NCT01131676	Empagliflozin	Placebo	0.17 (0.01, 4.07)	0/4687	1/2333	5.88
NCT01730534	Dapagliflozin	Placebo	2.00 (0.18, 22.04)	2/8574	1/8569	10.44
NCT01986881	Ertugliflozin	Placebo	1.00 (0.09, 11.02)	2/5493	1/2745	10.45
NCT03036124	Dapagliflozin	Placebo	0.50 (0.05, 5.51)	1/2368	2/2368	10.45
NCT03036150	Dapagliflozin	Placebo	3.00 (0.12, 73.60)	1/2149	0/2149	5.88
NCT03057951	Empagliflozin	Placebo	0.33 (0.01, 8.16)	0/2996	1/2989	5.88
NCT03057977	Empagliflozin	Placebo	0.33 (0.01, 8.18)	0/1863	1/1863	5.88
NCT03315143	Sotagliflozin	Placebo	1.33 (0.30, 5.95)	4/5291	3/5286	26.87
NCT03521934	Sotagliflozin	Placebo	0.34 (0.01, 8.25)	0/605	1/611	5.88
NCT03594110	Empagliflozin	Placebo	3.00 (0.12, 73.64)	1/3304	0/3305	5.88
NCT03619213	Dapagliflozin	Placebo	5.00 (0.24, 104.14)	2/3126	0/3127	6.53
Overall (I-squared = 0.0%, p = 0.868)			1.01 (0.47, 2.20)	13/40456	11/35345	100.00

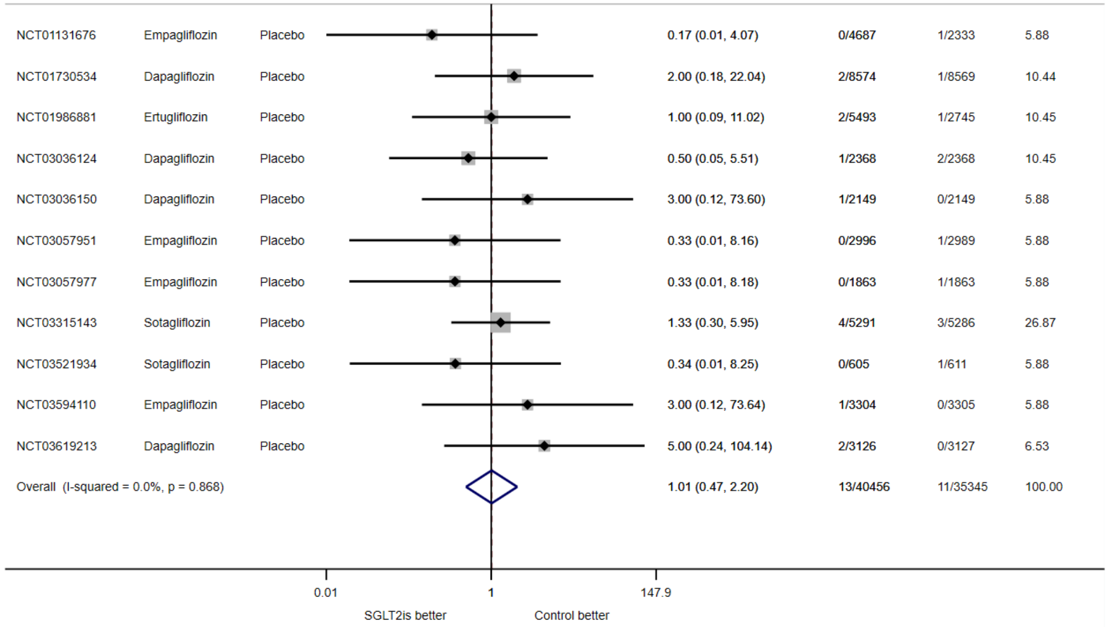

System 7-Gastrointestinal Disorders-Figure S89 Meta-analysis of SGLT2is and Pancreatitis chronic

Trial	SGLT2is	Control	Risk Ratio (95% CI)	Events, Treatment	Events, Control	% Weight
NCT01031680	Dapagliflozin	Placebo	3.01 (0.12, 73.77)	1/460	0/462	7.10
NCT01032629	Canagliflozin	Placebo	0.17 (0.01, 4.08)	0/2886	1/1441	7.09
NCT01131676	Empagliflozin	Placebo	2.49 (0.12, 51.83)	2/4687	0/2333	7.87
NCT01730534	Dapagliflozin	Placebo	1.00 (0.14, 7.09)	2/8574	2/8569	18.89
NCT01986881	Ertugliflozin	Placebo	2.50 (0.12, 52.04)	2/5493	0/2745	7.87
NCT01989754	Canagliflozin	Placebo	2.00 (0.18, 22.04)	2/2904	1/2903	12.60
NCT02065791	Canagliflozin	Placebo	0.33 (0.01, 8.17)	0/2200	1/2197	7.09
NCT03036150	Dapagliflozin	Placebo	1.00 (0.06, 15.98)	1/2149	1/2149	9.45
NCT03057951	Empagliflozin	Placebo	0.33 (0.01, 8.16)	0/2996	1/2989	7.09
NCT03315143	Sotagliflozin	Placebo	0.20 (0.01, 4.16)	0/5291	2/5286	7.87
NCT03619213	Dapagliflozin	Placebo	0.33 (0.01, 8.18)	0/3126	1/3127	7.09
Overall (I-squared = 0.0%, p = 0.894)			0.84 (0.36, 1.96)	10/40766	10/34201	100.00

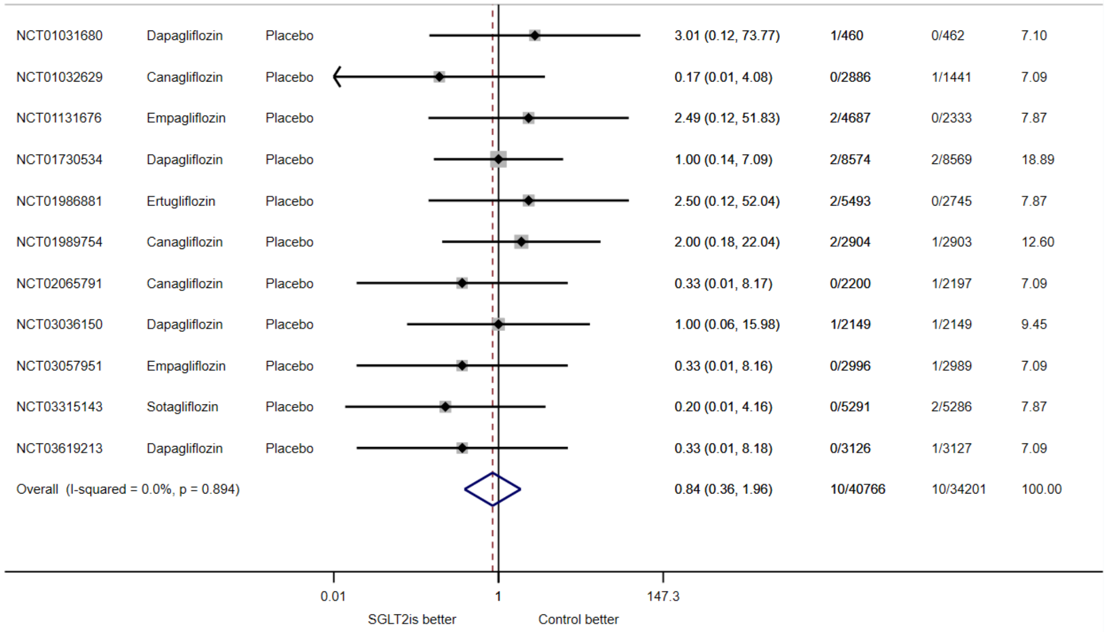

System 7-Gastrointestinal Disorders-Figure S90 Meta-analysis of SGLT2is and Peptic ulcer

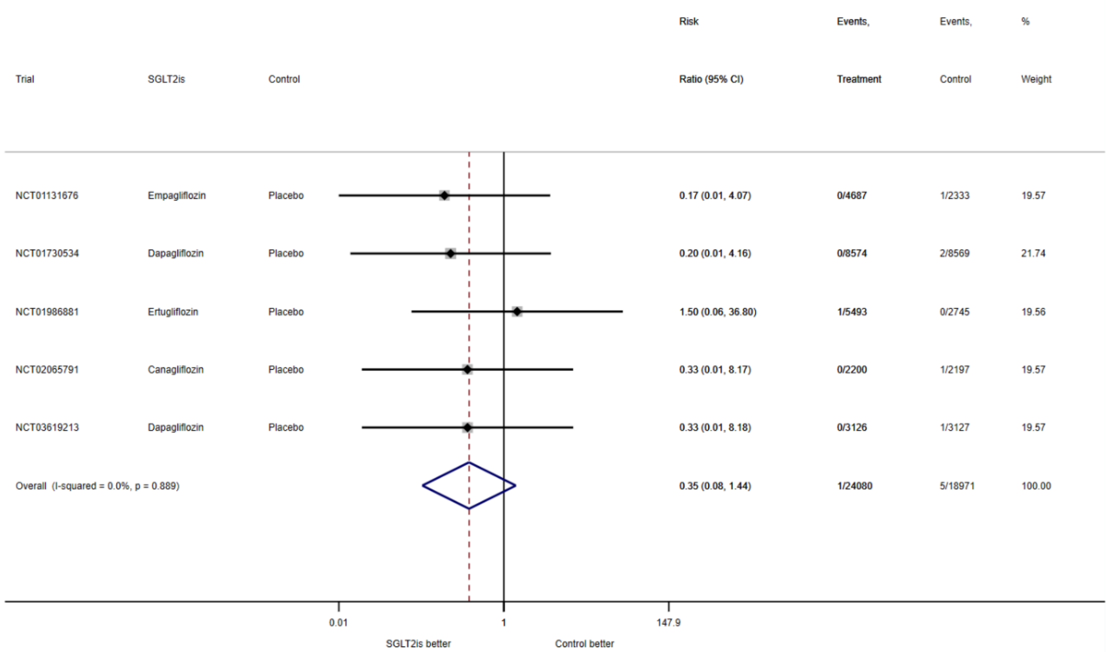

System 7-Gastrointestinal Disorders-Figure S91 Meta-analysis of SGLT2is and Peptic ulcer haemorrhage

Trial	SGLT2is	Control	Risk Ratio (95% CI)	Events, Treatment	Events, Control	% Weight
NCT01032629	Canagliflozin	Placebo	1.12 (0.35, 3.64)	9/2886	4/1441	29.52
NCT01042977	Dapagliflozin	Placebo	0.33 (0.01, 8.18)	0/482	1/483	3.99
NCT01131676	Empagliflozin	Placebo	1.99 (0.22, 17.80)	4/4687	1/2333	8.51
NCT01730534	Dapagliflozin	Placebo	2.50 (0.48, 12.87)	5/8574	2/8569	15.19
NCT01986881	Ertugliflozin	Placebo	3.50 (0.18, 67.71)	3/5493	0/2745	4.65
NCT02065791	Canagliflozin	Placebo	1.00 (0.06, 15.96)	1/2200	1/2197	5.32
NCT03057951	Empagliflozin	Placebo	0.33 (0.03, 3.20)	1/2996	3/2989	7.98
NCT03057977	Empagliflozin	Placebo	1.00 (0.20, 4.95)	3/1863	3/1863	15.97
NCT03315143	Sotagliflozin	Placebo	0.20 (0.01, 4.16)	0/5291	2/5286	4.43
NCT03619213	Dapagliflozin	Placebo	0.20 (0.01, 4.17)	0/3126	2/3127	4.43
Overall (I-squared = 0.0%, p = 0.758)			1.02 (0.54, 1.93)	26/37598	19/31033	100.00

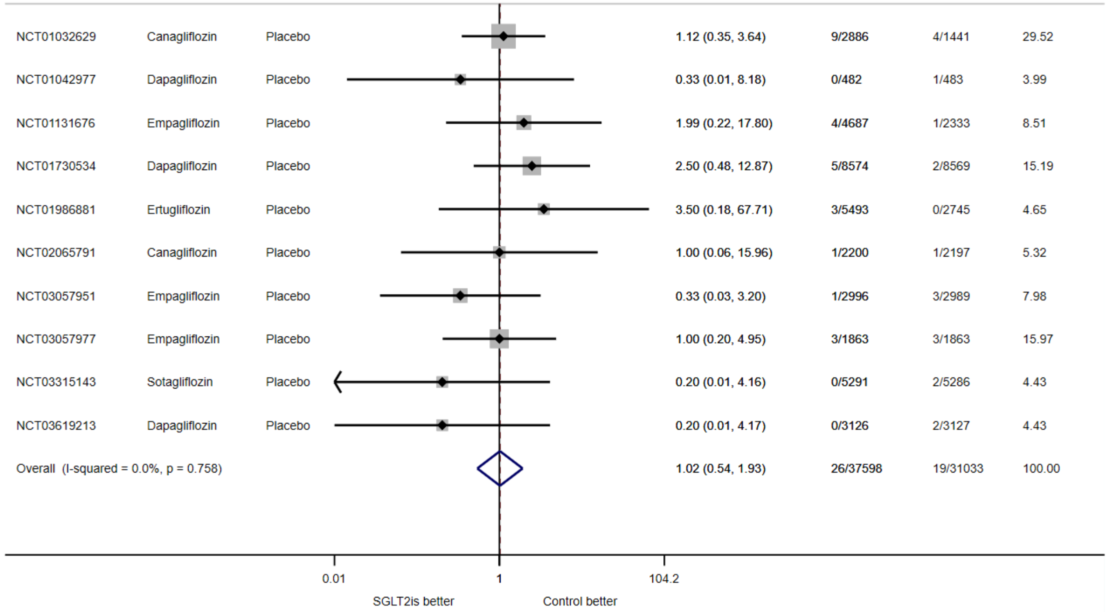

System 7-Gastrointestinal Disorders-Figure S92 Meta-analysis of SGLT2is and Rectal haemorrhage

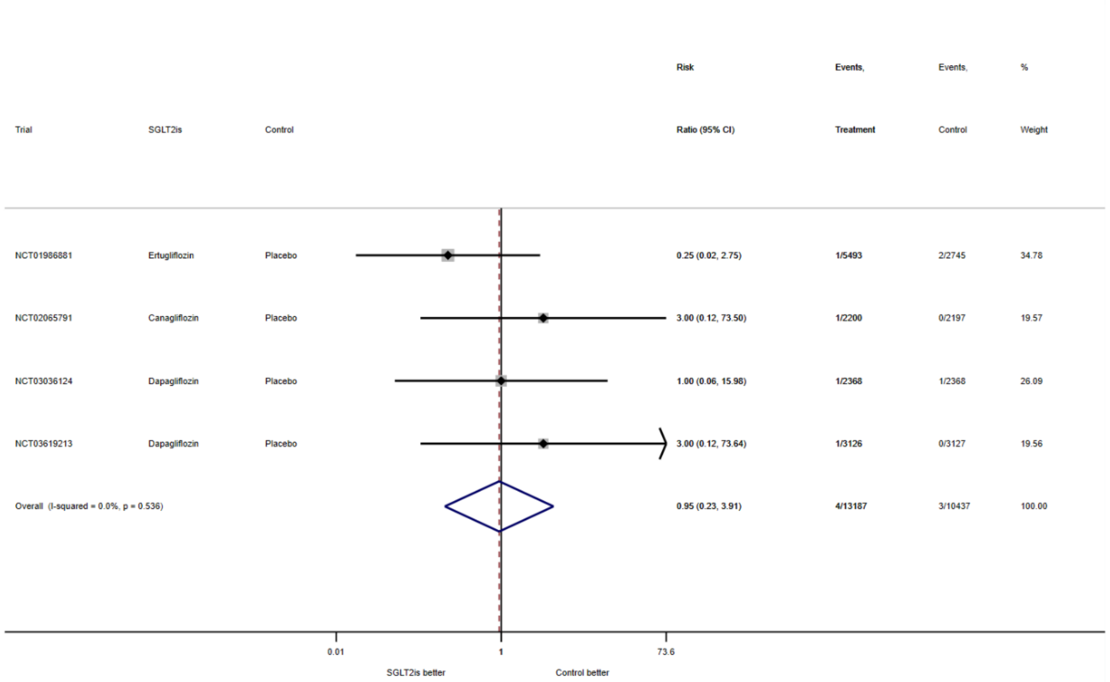

System 7-Gastrointestinal Disorders-Figure S93 Meta-analysis of SGLT2is and Rectal polyp

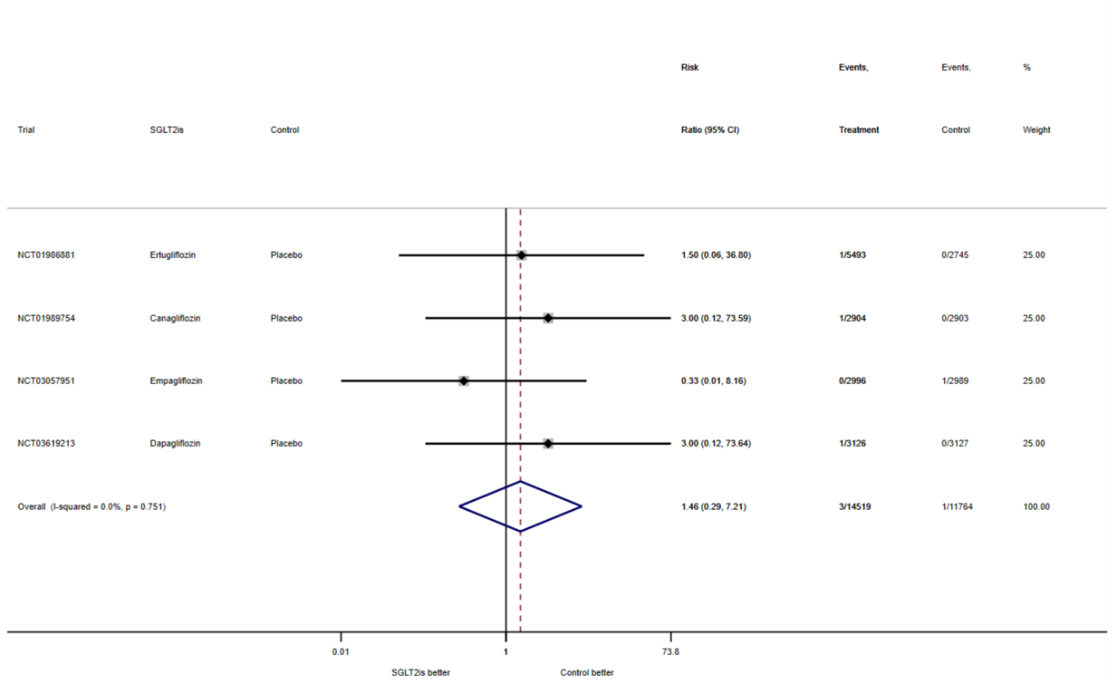

System 7-Gastrointestinal Disorders-Figure S94 Meta-analysis of SGLT2is and Salivary gland calculus

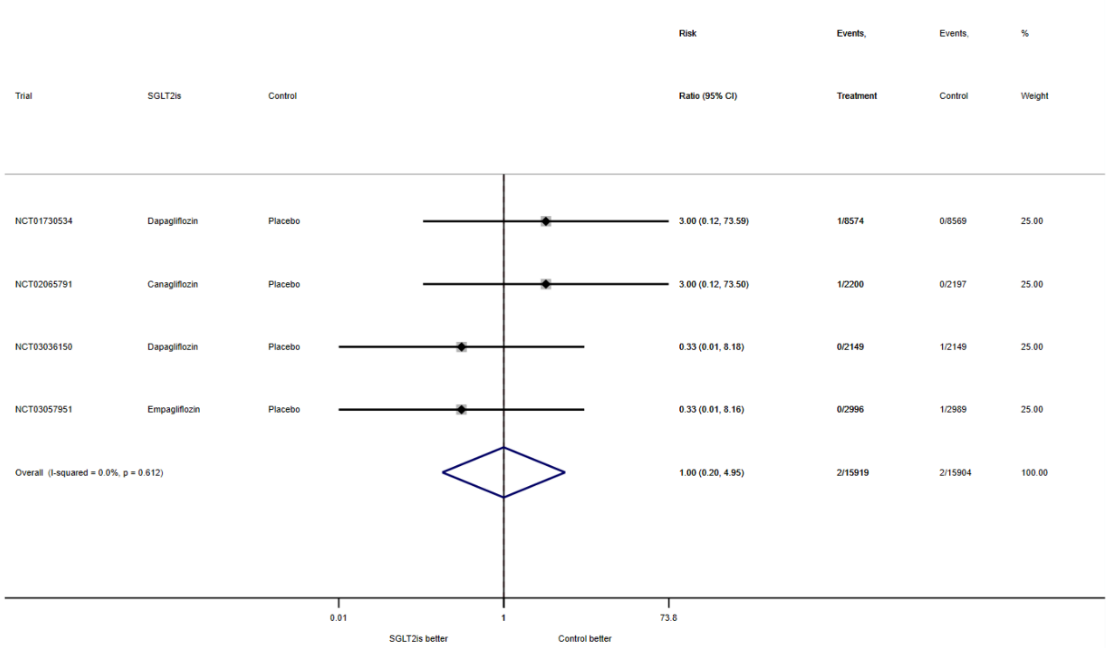

System 7-Gastrointestinal Disorders-Figure S95 Meta-analysis of SGLT2is and Small intestinal haemorrhage

Trial	SGLT2is	Control	Risk Ratio (95% CI)	Events, Treatment	Events, Control	% Weight
NCT00673231	Dapagliflozin	Placebo	0.97 (0.04, 23.77)	1/610	0/197	2.43
NCT01032629	Canagliflozin	Placebo	5.49 (0.30, 99.29)	5/2886	0/1441	2.96
NCT01106651	Canagliflozin	Placebo	1.49 (0.06, 36.53)	1/477	0/237	2.43
NCT01131676	Empagliflozin	Placebo	2.49 (0.29, 21.29)	5/4687	1/2333	5.39
NCT01730534	Dapagliflozin	Placebo	1.11 (0.45, 2.73)	10/8574	9/8569	30.63
NCT01986881	Ertugliflozin	Placebo	0.50 (0.07, 3.55)	2/5493	2/2745	6.46
NCT01989754	Canagliflozin	Placebo	5.00 (0.24, 104.07)	2/2904	0/2903	2.69
NCT02065791	Canagliflozin	Placebo	0.50 (0.05, 5.50)	1/2200	2/2197	4.31
NCT02384941	Sotagliflozin	Placebo	1.53 (0.06, 37.53)	1/525	0/268	2.43
NCT03036124	Dapagliflozin	Placebo	5.00 (0.24, 104.09)	2/2368	0/2368	2.69
NCT03036150	Dapagliflozin	Placebo	3.00 (0.31, 28.82)	3/2149	1/2149	4.85
NCT03057951	Empagliflozin	Placebo	2.49 (0.48, 12.85)	5/2996	2/2989	9.24
NCT03057977	Empagliflozin	Placebo	0.33 (0.01, 8.18)	0/1863	1/1863	2.42
NCT03242252	Sotagliflozin	Placebo	2.47 (0.12, 51.30)	2/527	0/260	2.70
NCT03315143	Sotagliflozin	Placebo	0.25 (0.03, 2.23)	1/5291	4/5286	5.17
NCT03594110	Empagliflozin	Placebo	1.00 (0.14, 7.10)	2/3304	2/3305	6.46
NCT03619213	Dapagliflozin	Placebo	2.00 (0.18, 22.05)	2/3126	1/3127	4.31
NCT04157751	Empagliflozin	Placebo	0.34 (0.01, 8.27)	0/260	1/264	2.43
Overall (I-squared = 0.0%, p = 0.922)			1.26 (0.77, 2.07)	45/50240	26/42501	100.00

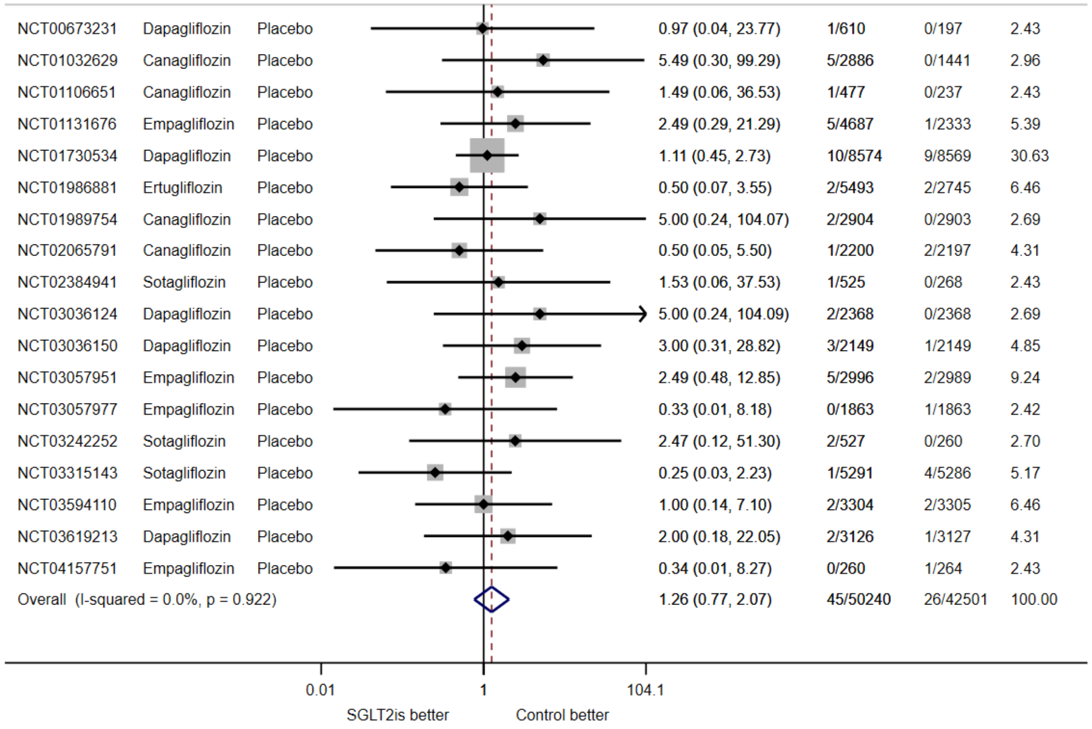

System 7-Gastrointestinal Disorders-Figure S96 Meta-analysis of SGLT2is and Small intestinal obstruction

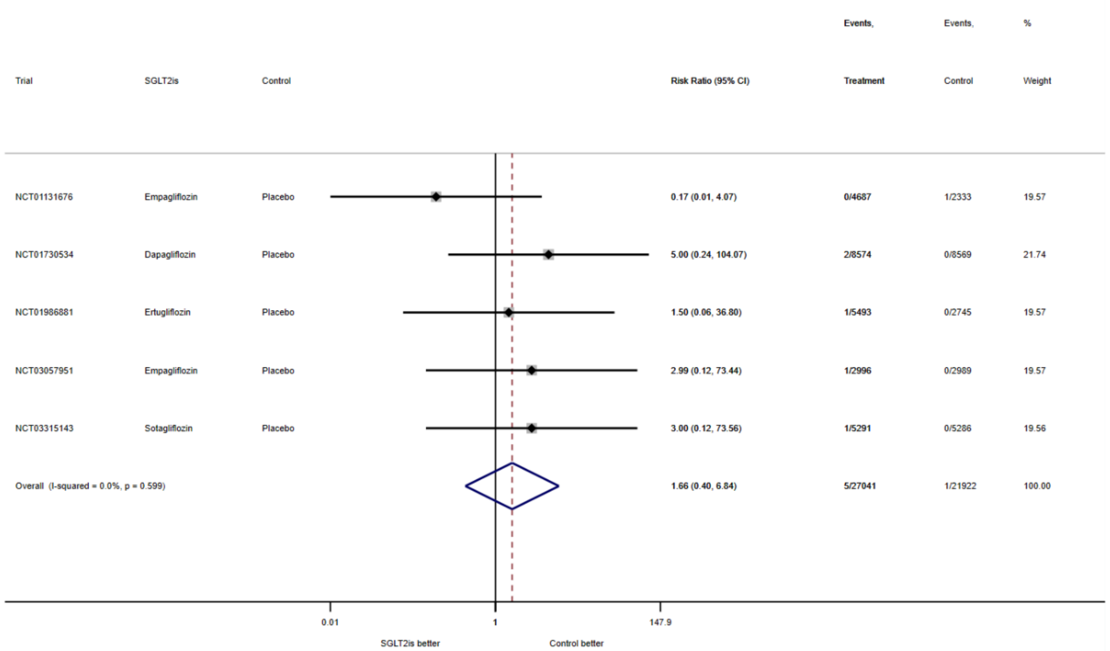

System 7-Gastrointestinal Disorders-Figure S97 Meta-analysis of SGLT2is and Small intestinal perforation

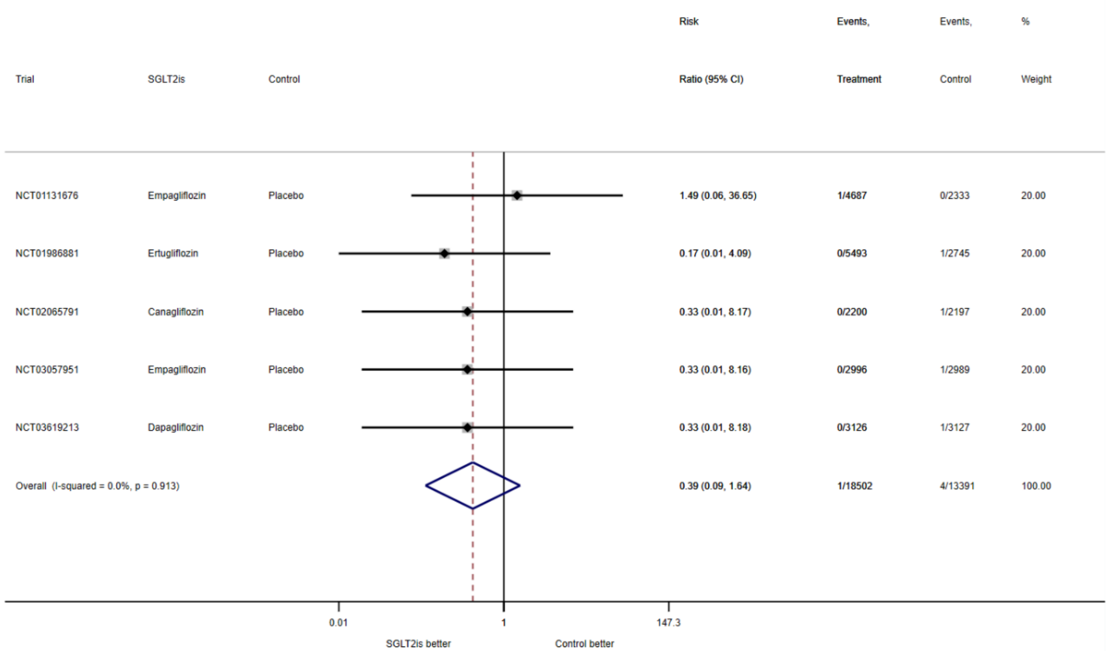

System 7-Gastrointestinal Disorders-Figure S98 Meta-analysis of SGLT2is and Subileus

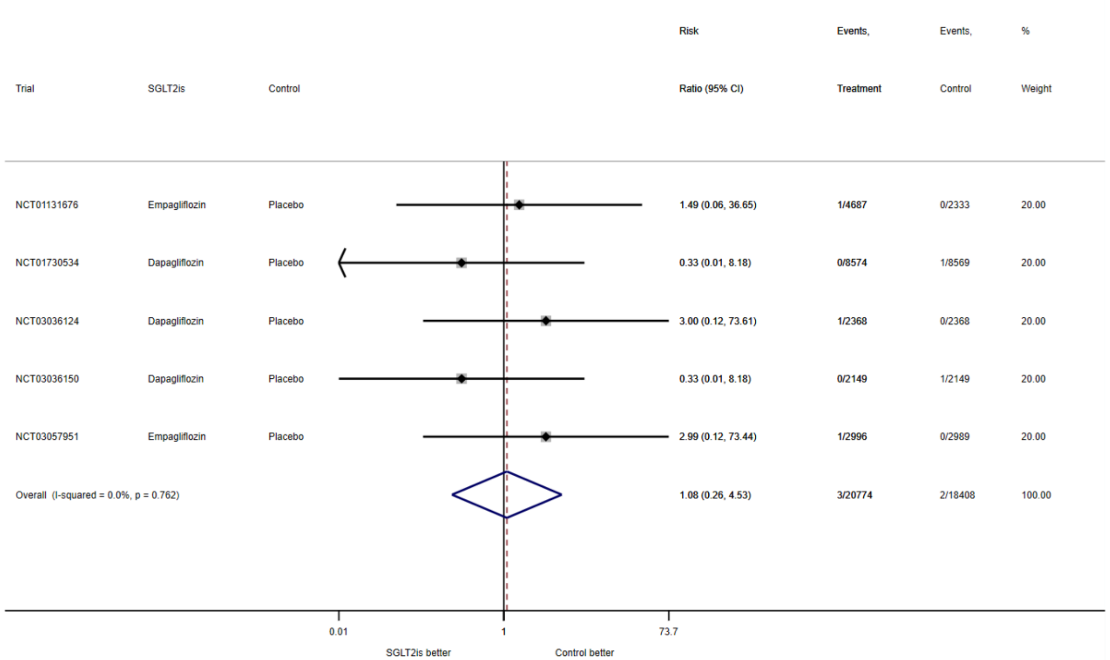

System 7-Gastrointestinal Disorders-Figure S99 Meta-analysis of SGLT2is and Thrombosis mesenteric vessel

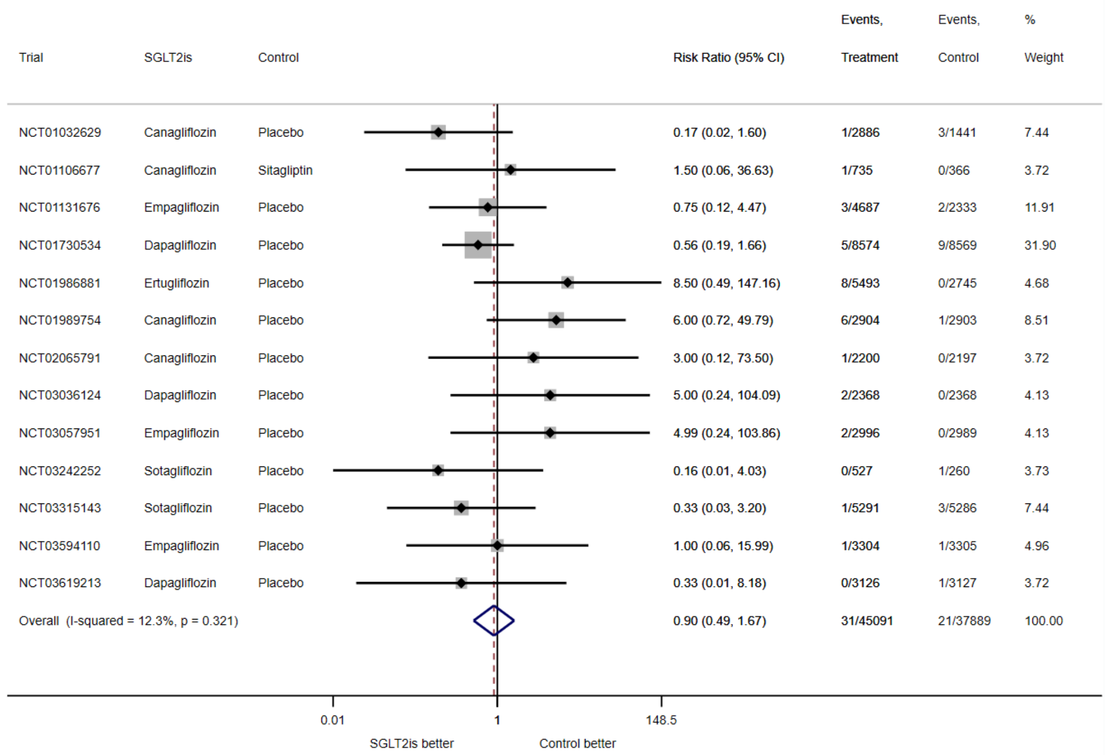

System 7-Gastrointestinal Disorders-Figure S100 Meta-analysis of SGLT2is and Umbilical hernia

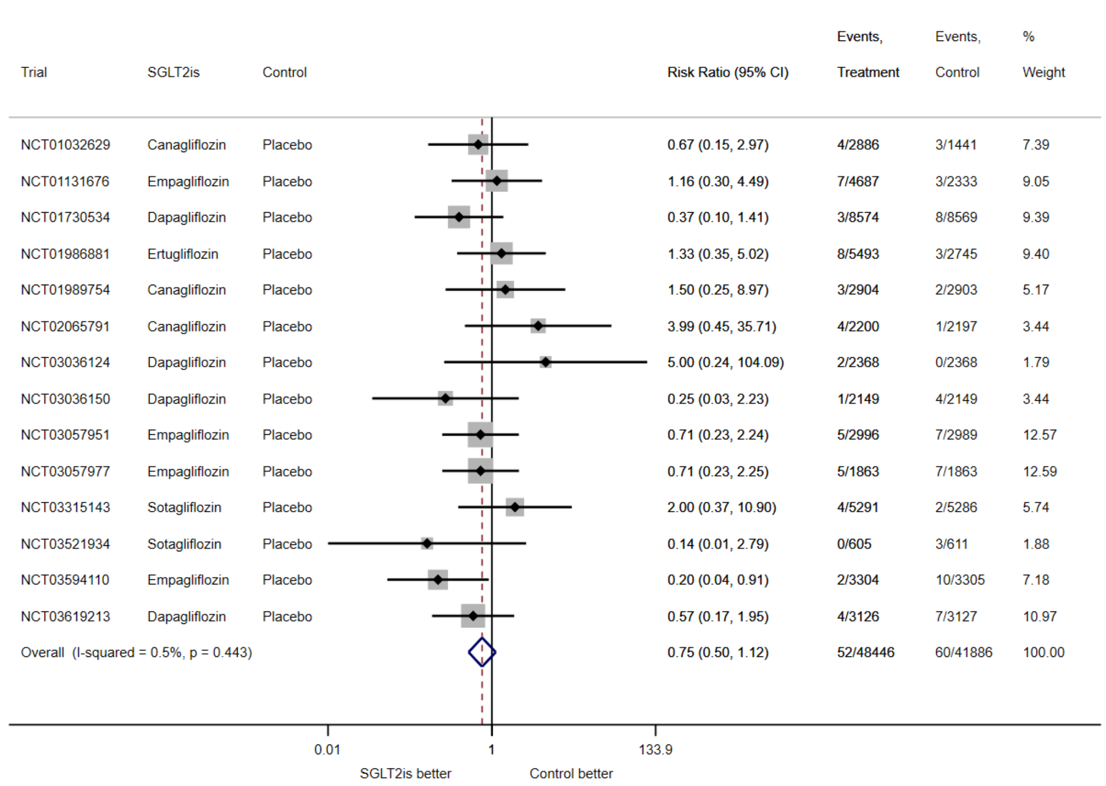

System 7-Gastrointestinal Disorders-Figure S101 Meta-analysis of SGLT2is and Upper gastrointestinal haemorrhage

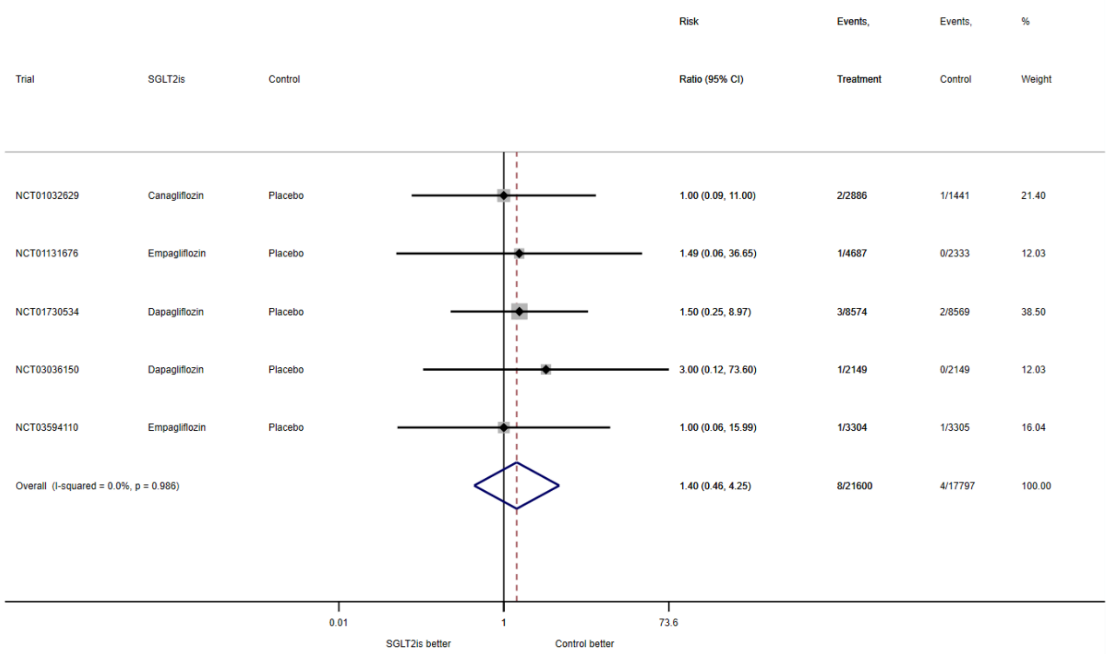

System 7-Gastrointestinal Disorders-Figure S102 Meta-analysis of SGLT2is and Varices oesophageal

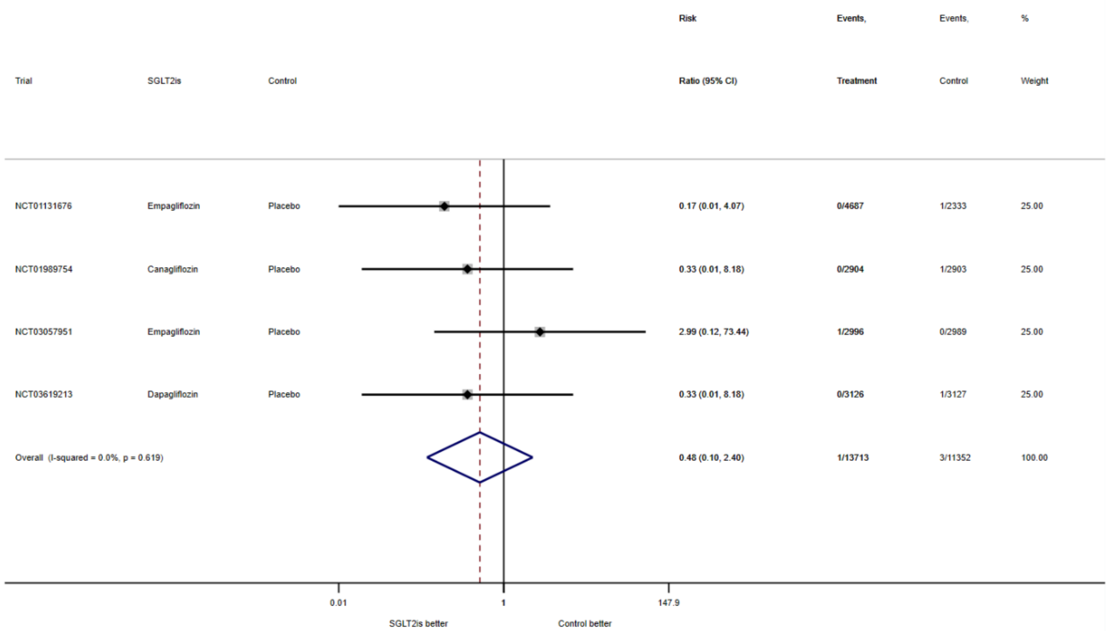

System 7-Gastrointestinal Disorders-Figure S103 Meta-analysis of SGLT2is and Volvulus

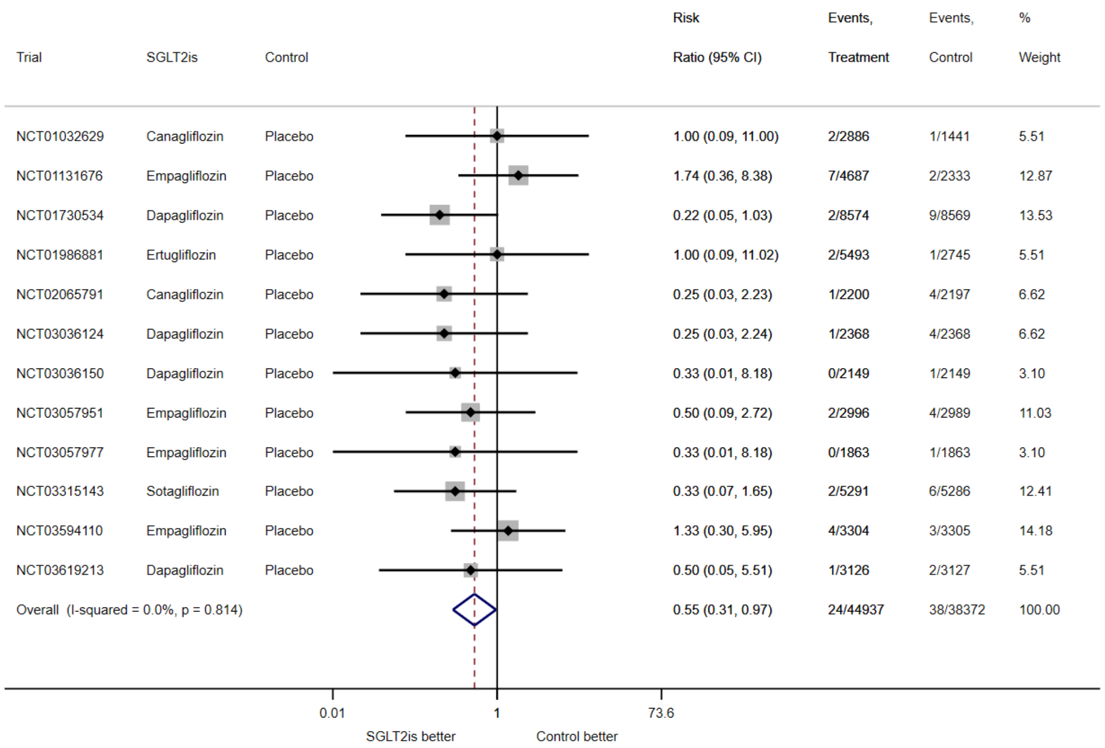

System 7-Gastrointestinal Disorders-Figure S104 Meta-analysis of SGLT2is and Vomiting

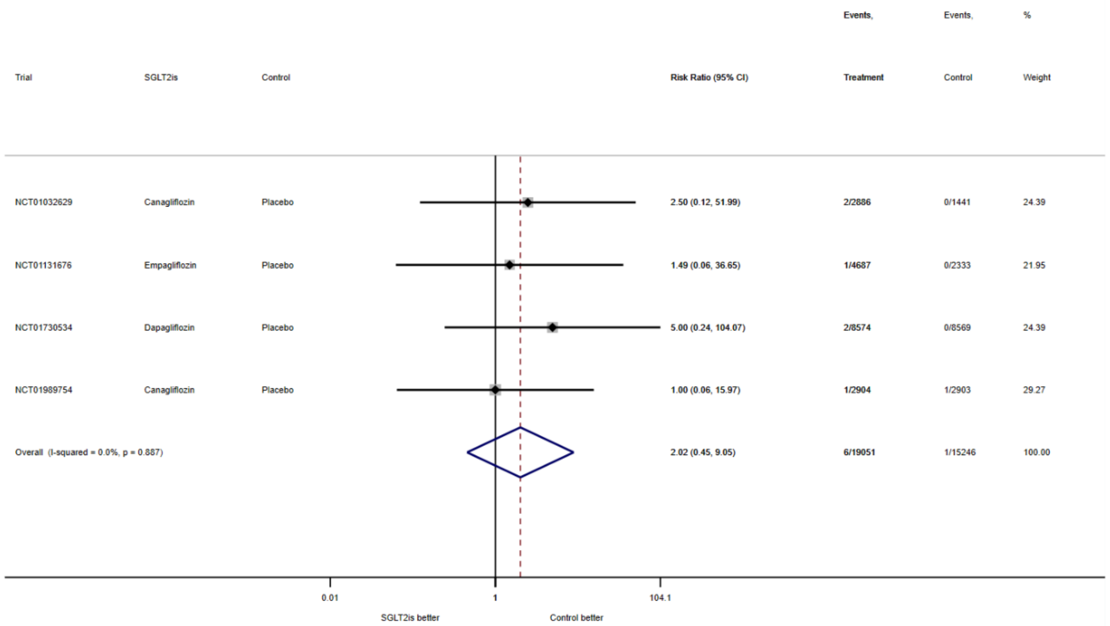

System 8-General Disorders-Figure S1 Meta-analysis of SGLT2is and Adverse drug reaction

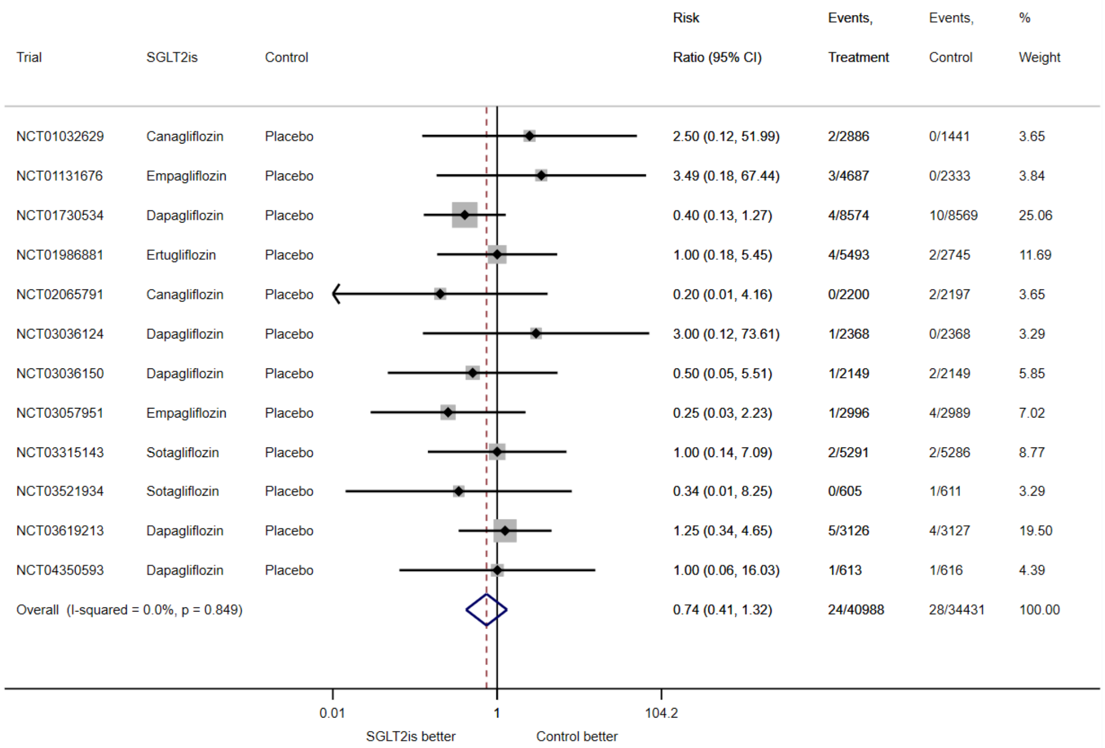

System 8-General Disorders-Figure S2 Meta-analysis of SGLT2is and Asthenia

Trial	SGLT2is	Control	Risk Ratio (95% CI)	Events, Treatment	Events, Control	% Weight
NCT01131676	Empagliflozin	Placebo	1.00 (0.25, 3.98)	6/4687	3/2333	16.63
NCT01730534	Dapagliflozin	Placebo	0.50 (0.05, 5.51)	1/8574	2/8569	5.54
NCT01989754	Canagliflozin	Placebo	0.33 (0.01, 8.18)	0/2904	1/2903	3.11
NCT02033889	Ertugliflozin	Placebo/Glimepiride	0.17 (0.01, 4.14)	0/412	1/209	3.12
NCT02065791	Canagliflozin	Placebo	3.00 (0.31, 28.78)	3/2200	1/2197	6.23
NCT03036124	Dapagliflozin	Placebo	0.20 (0.01, 4.16)	0/2368	2/2368	3.46
NCT03036150	Dapagliflozin	Placebo	0.33 (0.01, 8.18)	0/2149	1/2149	3.11
NCT03057951	Empagliflozin	Placebo	1.33 (0.30, 5.94)	4/2996	3/2989	14.25
NCT03057977	Empagliflozin	Placebo	0.50 (0.15, 1.66)	4/1863	8/1863	22.21
NCT03242252	Sotagliflozin	Placebo	1.48 (0.06, 36.28)	1/527	0/260	3.12
NCT03315143	Sotagliflozin	Placebo	6.99 (0.36, 135.36)	3/5291	0/5286	3.63
NCT03521934	Sotagliflozin	Placebo	0.34 (0.01, 8.25)	0/605	1/611	3.12
NCT03619213	Dapagliflozin	Placebo	1.00 (0.20, 4.95)	3/3126	3/3127	12.47
Overall (I-squared = 0.0%, p = 0.828)			0.81 (0.46, 1.42)	25/37702	26/34864	100.00

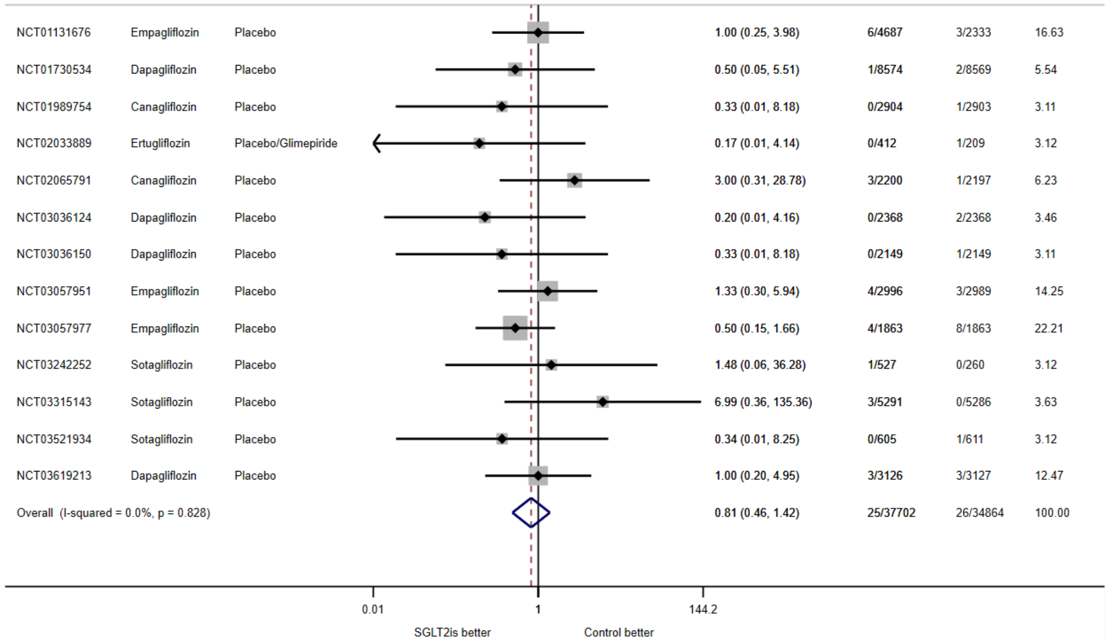

System 8-General Disorders-Figure S3 Meta-analysis of SGLT2is and Cardiac death

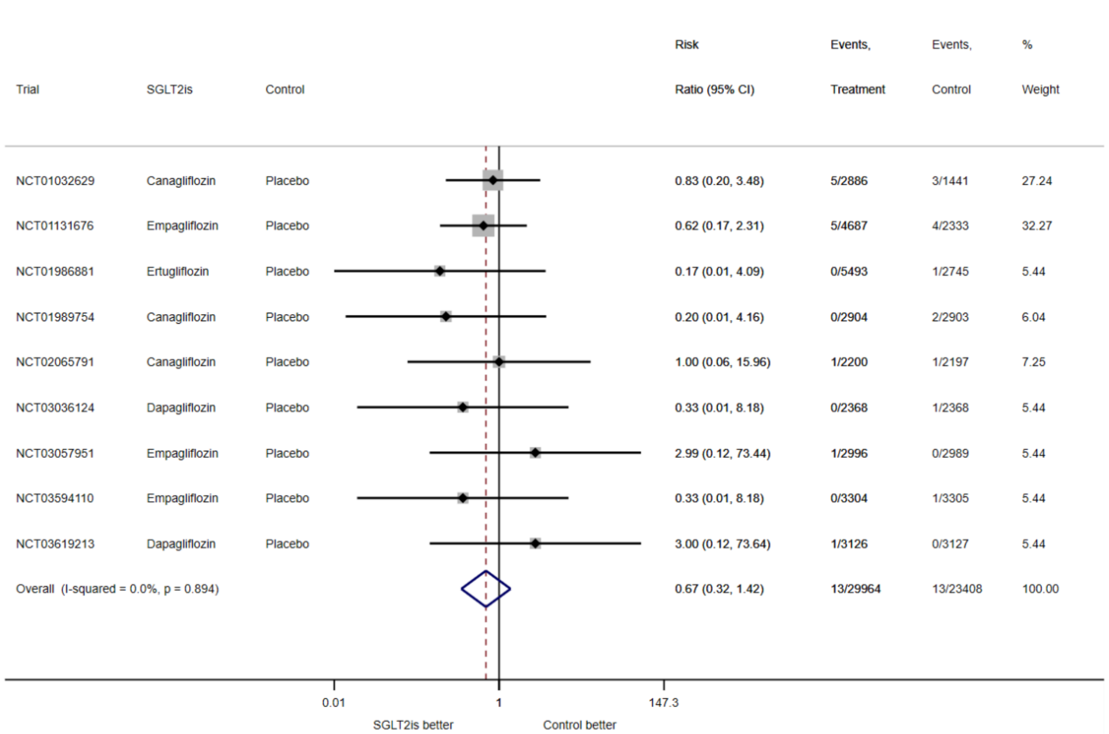

System 8-General Disorders-Figure S4 Meta-analysis of SGLT2is and Chest discomfort

Trial	SGLT2is	Control	Risk Ratio (95% CI)	Events, Treatment	Events, Control	% Weight
NCT00528879	Dapagliflozin	Placebo	1.68 (0.08, 34.84)	2/409	0/137	0.50
NCT01031680	Dapagliflozin	Placebo	0.50 (0.05, 5.52)	1/460	2/462	0.80
NCT01032629	Canagliflozin	Placebo	0.85 (0.51, 1.41)	39/2886	23/1441	17.55
NCT01042977	Dapagliflozin	Placebo	0.14 (0.01, 2.76)	0/482	3/483	0.52
NCT01131676	Empagliflozin	Placebo	1.16 (0.74, 1.79)	65/4687	28/2333	23.68
NCT01164501	Empagliflozin	Placebo	0.25 (0.03, 2.43)	1/419	3/319	0.90
NCT01730534	Dapagliflozin	Placebo	1.45 (0.82, 2.56)	29/8574	20/8569	14.18
NCT01986881	Ertugliflozin	Placebo	1.56 (0.71, 3.46)	25/5493	8/2745	7.27
NCT01989754	Canagliflozin	Placebo	1.29 (0.69, 2.43)	22/2904	17/2903	11.53
NCT02033889	Ertugliflozin	Placebo/Glimepiride	1.53 (0.06, 37.28)	1/412	0/209	0.45
NCT02065791	Canagliflozin	Placebo	0.33 (0.03, 3.20)	1/2200	3/2197	0.90
NCT02384941	Sotagliflozin	Placebo	1.53 (0.06, 37.53)	1/525	0/268	0.45
NCT03036124	Dapagliflozin	Placebo	0.80 (0.22, 2.98)	4/2368	5/2368	2.66
NCT03036150	Dapagliflozin	Placebo	0.67 (0.11, 3.99)	2/2149	3/2149	1.44
NCT03057951	Empagliflozin	Placebo	0.50 (0.12, 1.99)	3/2996	6/2989	2.39
NCT03057977	Empagliflozin	Placebo	1.00 (0.14, 7.09)	2/1863	2/1863	1.20
NCT03315143	Sotagliflozin	Placebo	1.75 (0.51, 5.97)	7/5291	4/5286	3.04
NCT03521934	Sotagliflozin	Placebo	7.07 (0.37, 136.57)	3/605	0/611	0.52
NCT03594110	Empagliflozin	Placebo	0.58 (0.23, 1.48)	7/3304	12/3305	5.30
NCT03619213	Dapagliflozin	Placebo	0.78 (0.29, 2.09)	7/3126	9/3127	4.72
Overall (I-squared = 0.0%, p = 0.787)			1.05 (0.85, 1.30)	222/51153	148/43764	100.00

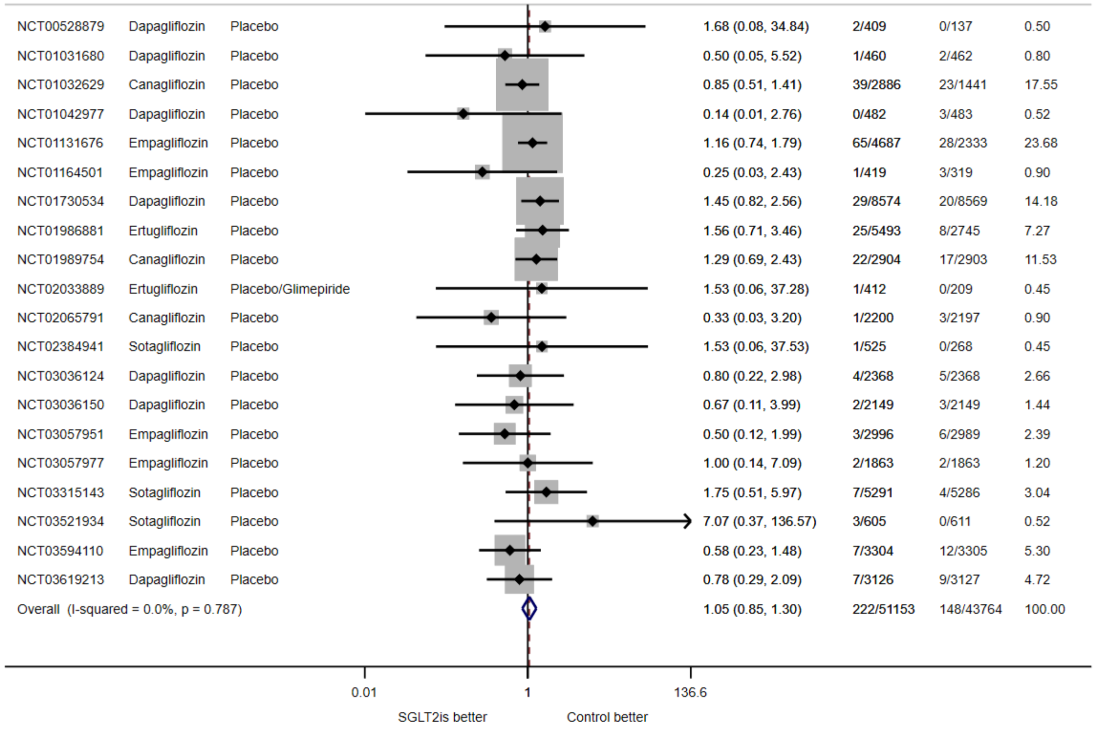

System 8-General Disorders-Figure S5 Meta-analysis of SGLT2is and Chest pain

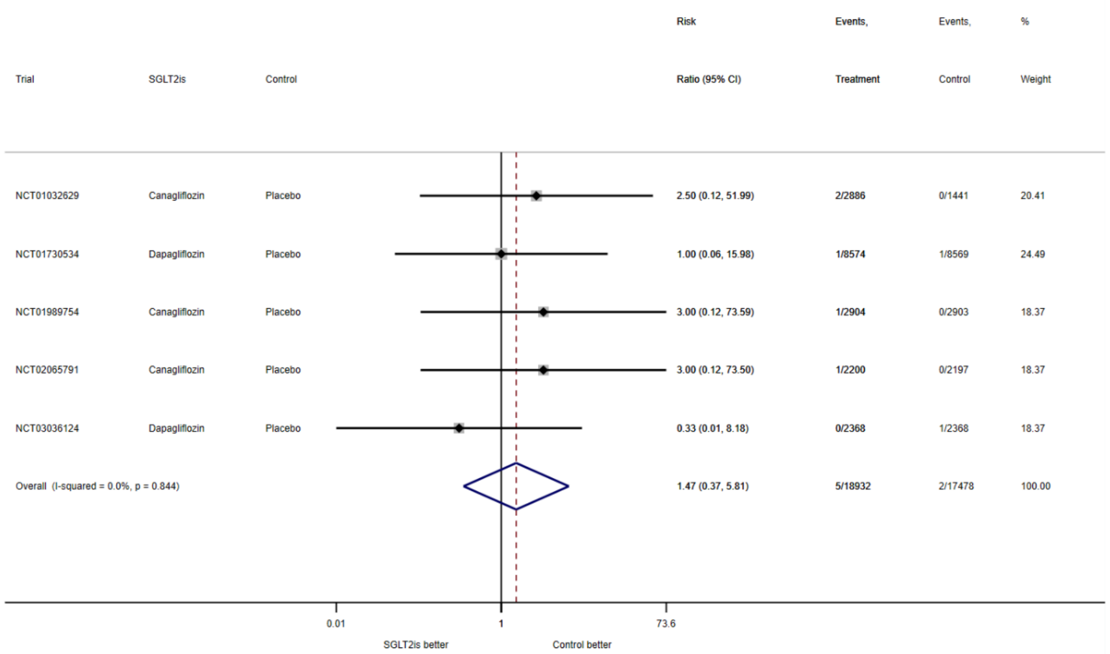

System 8-General Disorders-Figure S6 Meta-analysis of SGLT2is and Complication associated with device

Trial	SGLT2is	Control	Risk Ratio (95% CI)	Events, Treatment	Events, Control	% Weight
NCT01032629	Canagliflozin	Placebo	1.00 (0.43, 2.33)	16/2886	8/1441	2.87
NCT01131676	Empagliflozin	Placebo	0.62 (0.29, 1.33)	15/4687	12/2333	3.58
NCT01730534	Dapagliflozin	Placebo	0.91 (0.67, 1.23)	81/8574	89/8569	22.90
NCT01986881	Ertugliflozin	Placebo	1.14 (0.71, 1.82)	57/5493	25/2745	9.38
NCT01989754	Canagliflozin	Placebo	0.71 (0.23, 2.25)	5/2904	7/2903	1.56
NCT02033889	Ertugliflozin	Placebo/Glimepiride	1.53 (0.06, 37.28)	1/412	0/209	0.20
NCT02065791	Canagliflozin	Placebo	1.17 (0.39, 3.46)	7/2200	6/2197	1.73
NCT03036124	Dapagliflozin	Placebo	1.00 (0.67, 1.49)	48/2368	48/2368	13.10
NCT03036150	Dapagliflozin	Placebo	0.56 (0.30, 1.04)	15/2149	27/2149	5.20
NCT03057951	Empagliflozin	Placebo	1.47 (0.98, 2.21)	56/2996	38/2989	12.29
NCT03057977	Empagliflozin	Placebo	0.74 (0.42, 1.32)	20/1863	27/1863	6.22
NCT03315143	Sotagliflozin	Placebo	0.75 (0.32, 1.78)	9/5291	12/5286	2.76
NCT03521934	Sotagliflozin	Placebo	0.50 (0.09, 2.75)	2/605	4/611	0.72
NCT03594110	Empagliflozin	Placebo	1.11 (0.45, 2.73)	10/3304	9/3305	2.54
NCT03619213	Dapagliflozin	Placebo	1.04 (0.71, 1.52)	54/3126	52/3127	14.41
NCT04157751	Empagliflozin	Placebo	1.02 (0.14, 7.15)	2/260	2/264	0.54
Overall (I-squared = 0.0%, p = 0.723)			0.97 (0.84, 1.11)	398/49118	366/42359	100.00

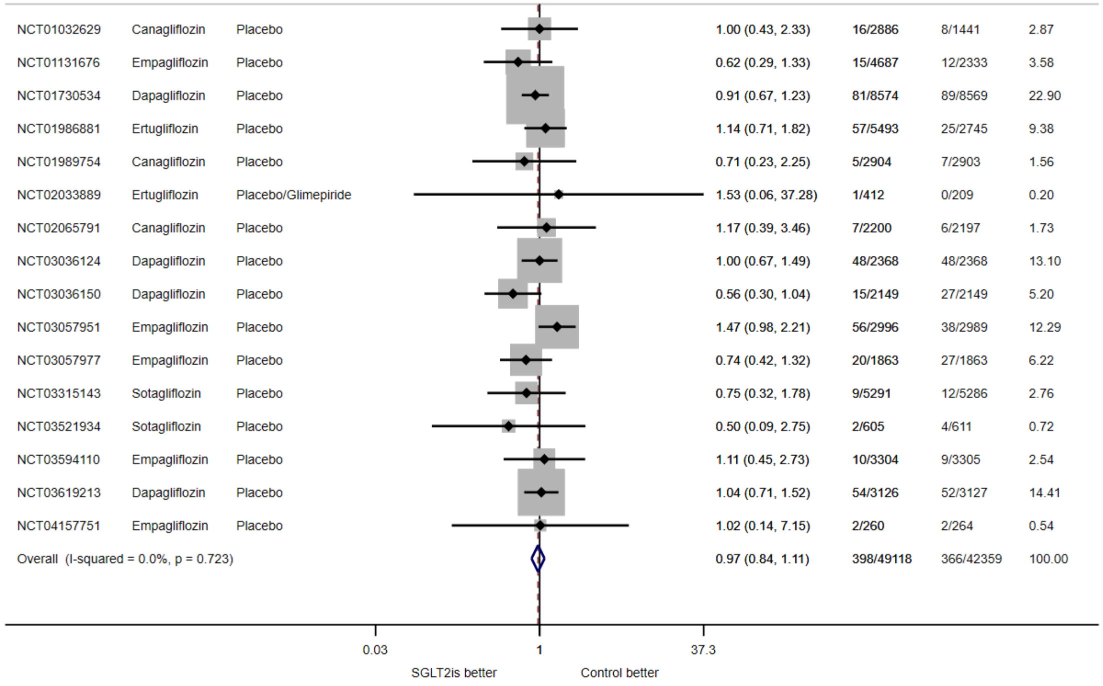

System 8-General Disorders-Figure S7 Meta-analysis of SGLT2is and Death

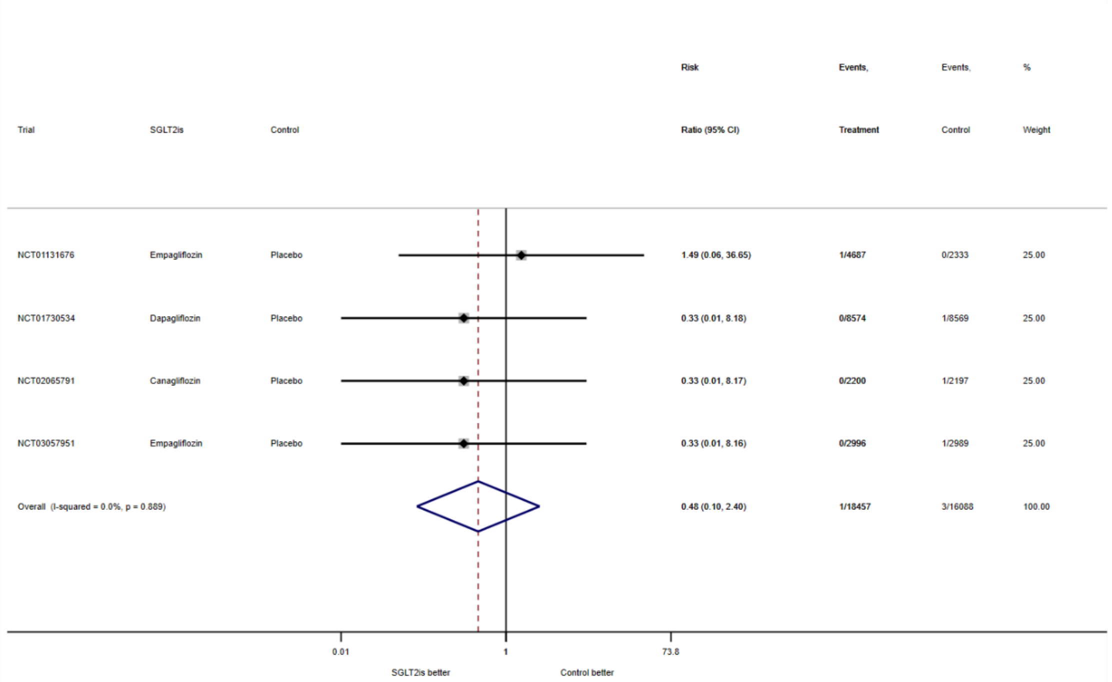

System 8-General Disorders-Figure S8 Meta-analysis of SGLT2is and Drowning

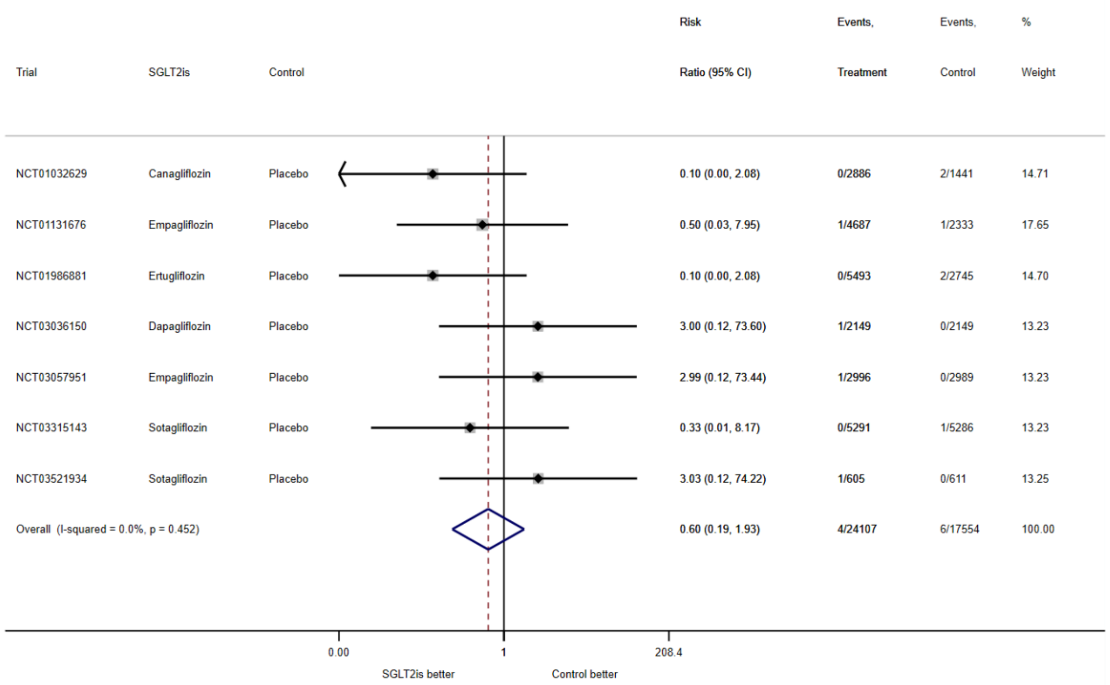

System 8-General Disorders-Figure S9 Meta-analysis of SGLT2is and Fatigue

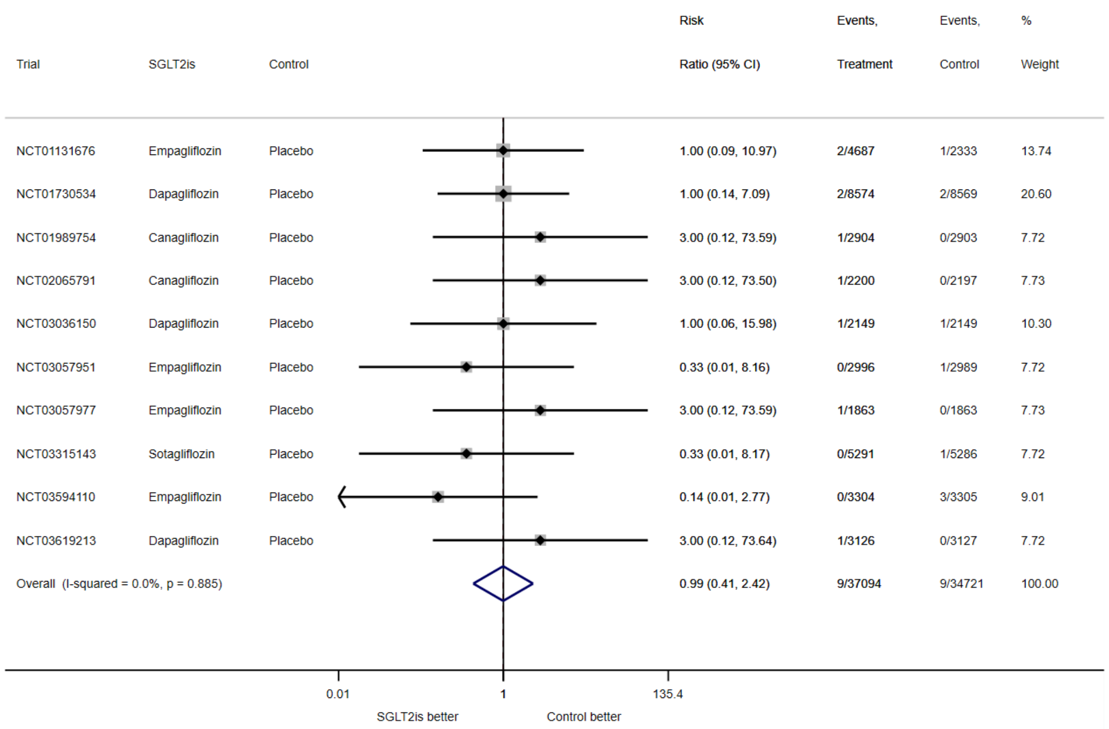

System 8-General Disorders-Figure S10 Meta-analysis of SGLT2is and Gait disturbance

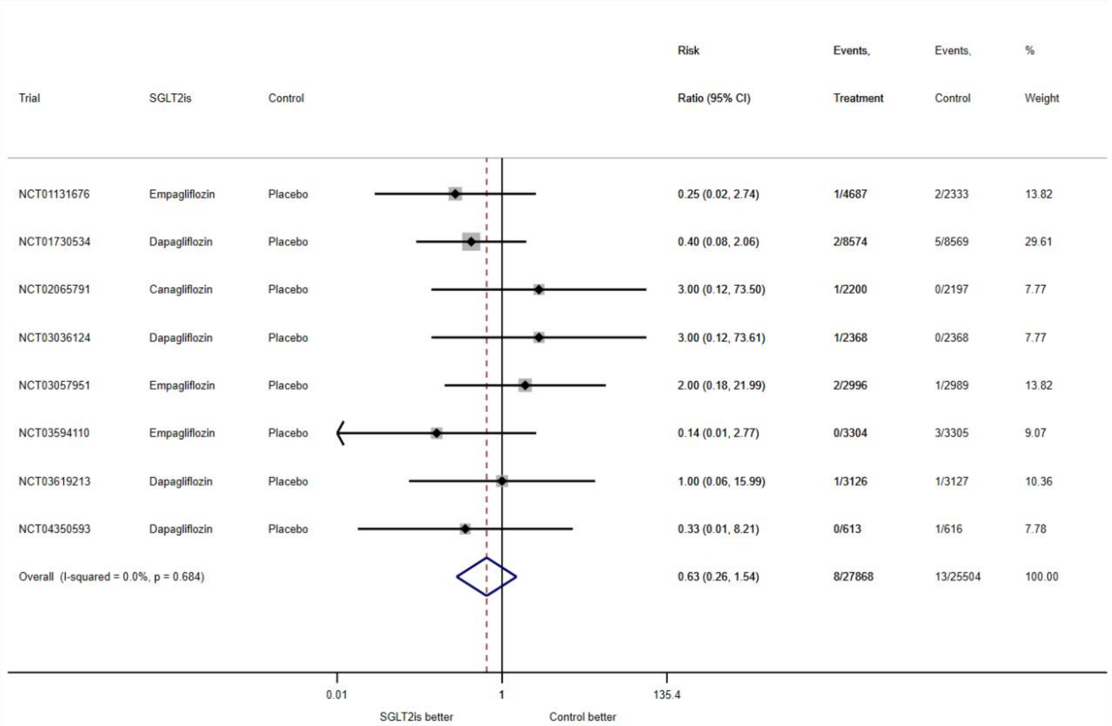

System 8-General Disorders-Figure S11 Meta-analysis of SGLT2is and General physical health deterioration

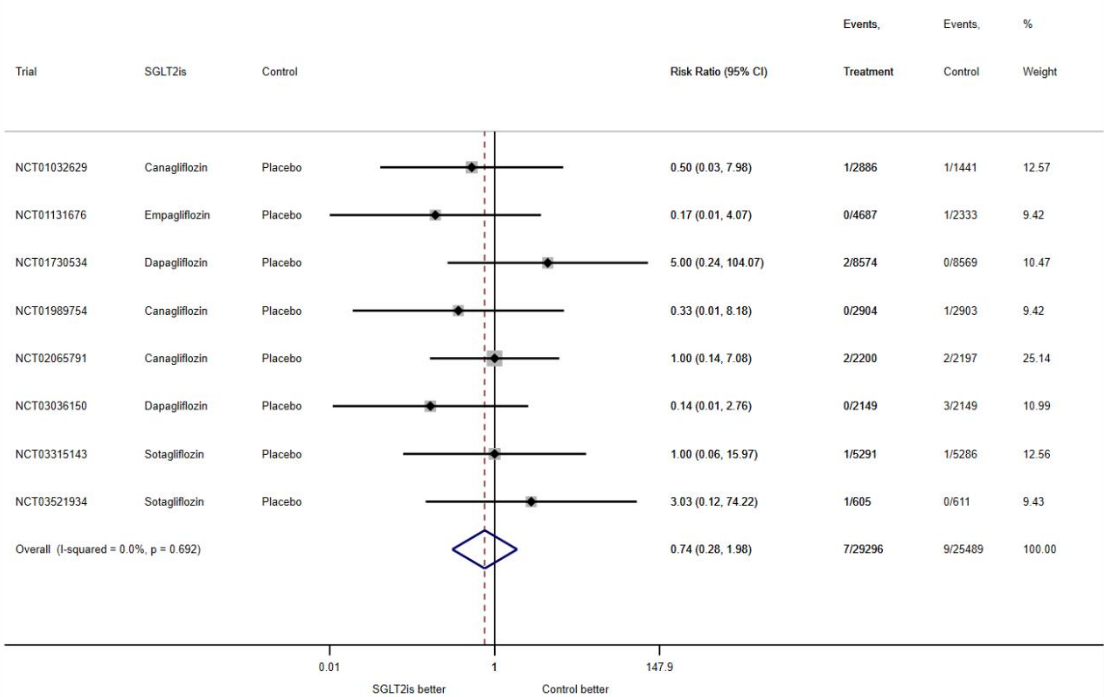

System 8-General Disorders-Figure S12 Meta-analysis of SGLT2is and Generalised oedema

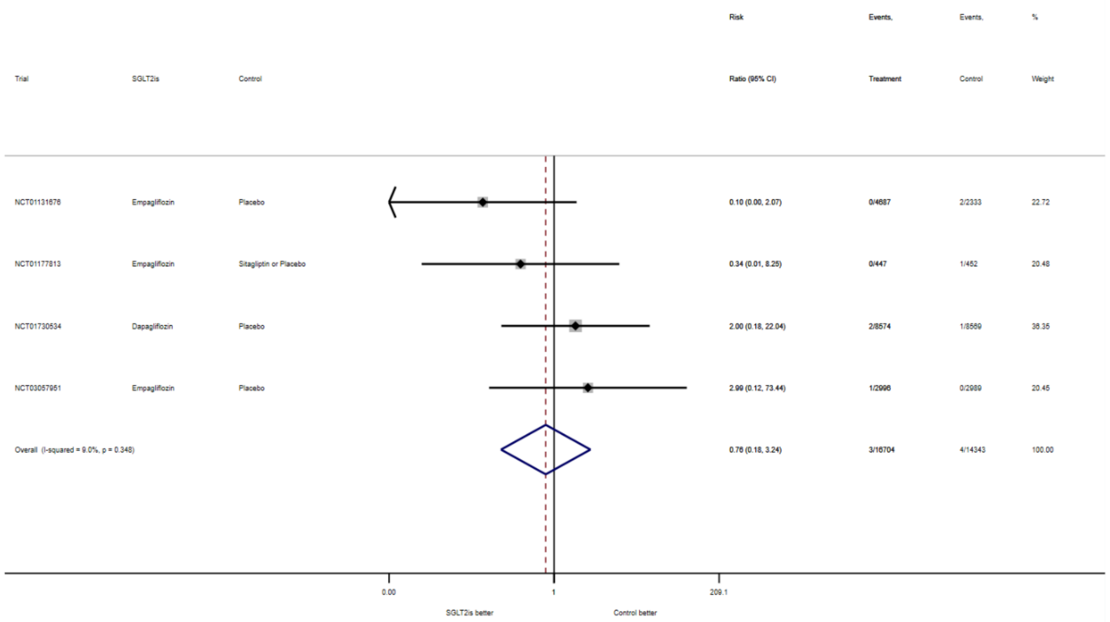

System 8-General Disorders-Figure S13 Meta-analysis of SGLT2is and Hernia

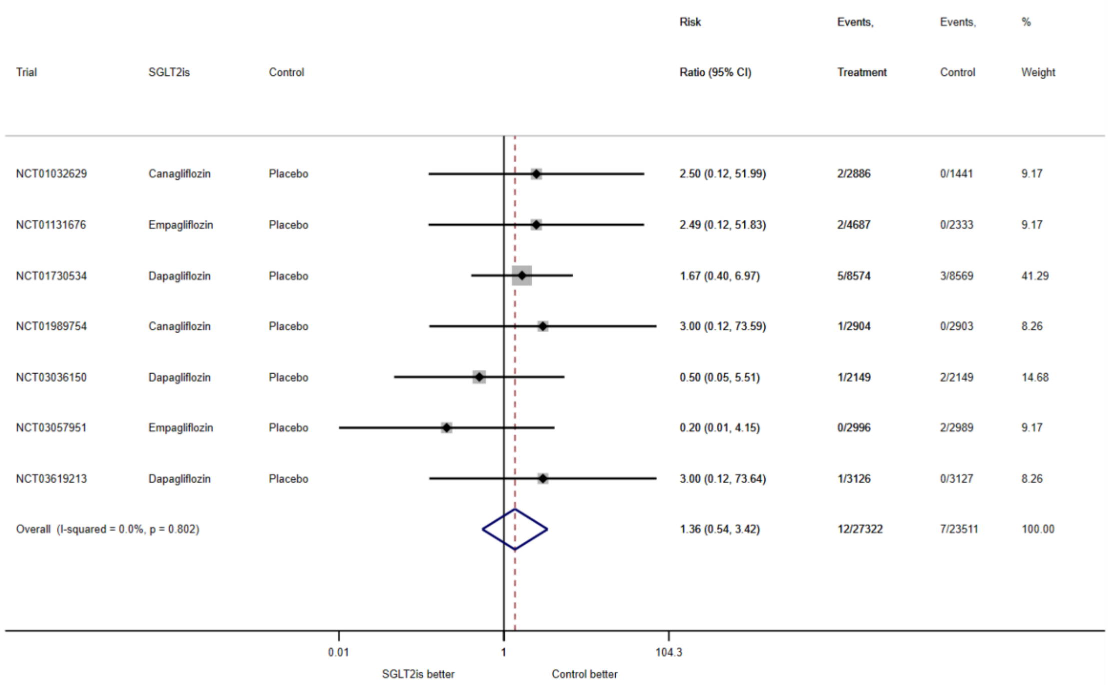

System 8-General Disorders-Figure S14 Meta-analysis of SGLT2is and Impaired healing

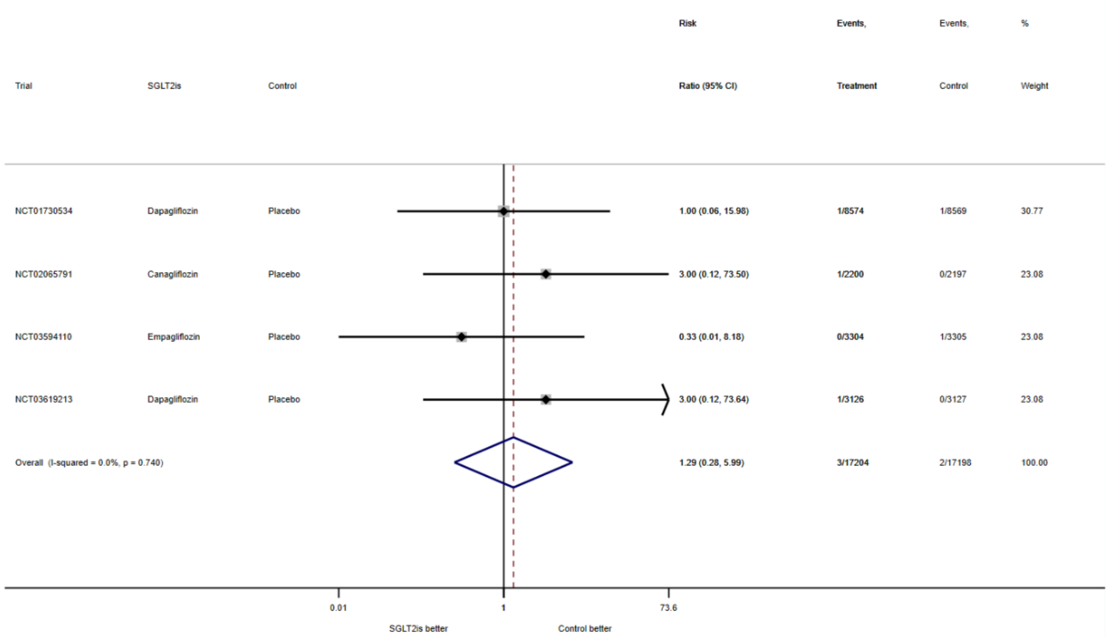

System 8-General Disorders-Figure S15 Meta-analysis of SGLT2is and Incarcerated hernia

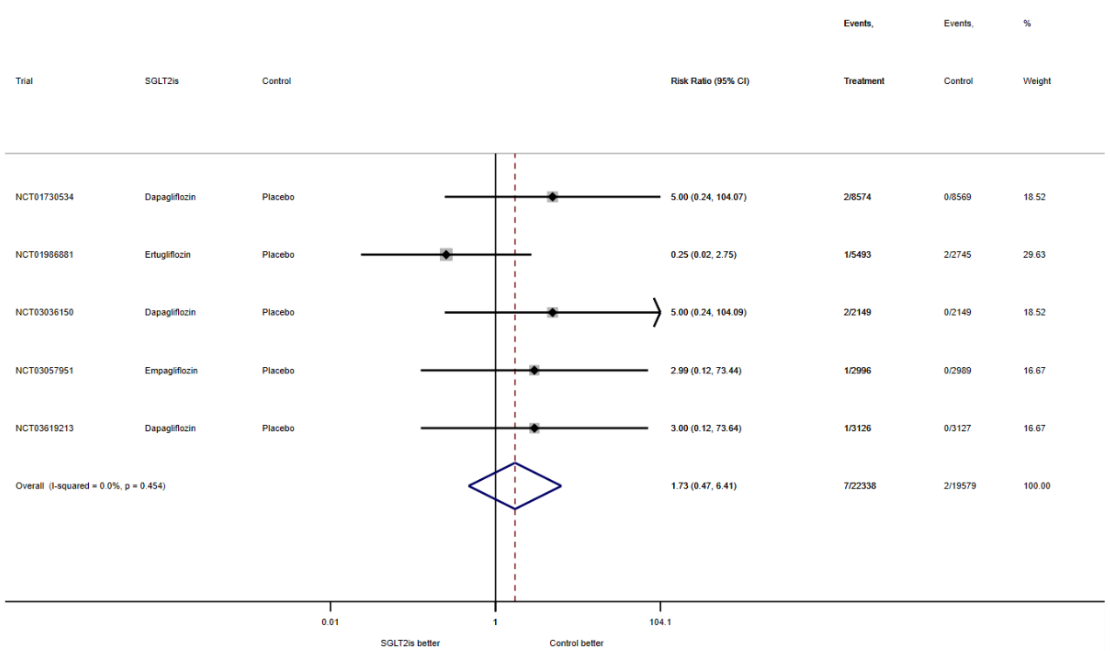

System 8-General Disorders-Figure S16 Meta-analysis of SGLT2is and Inflammation

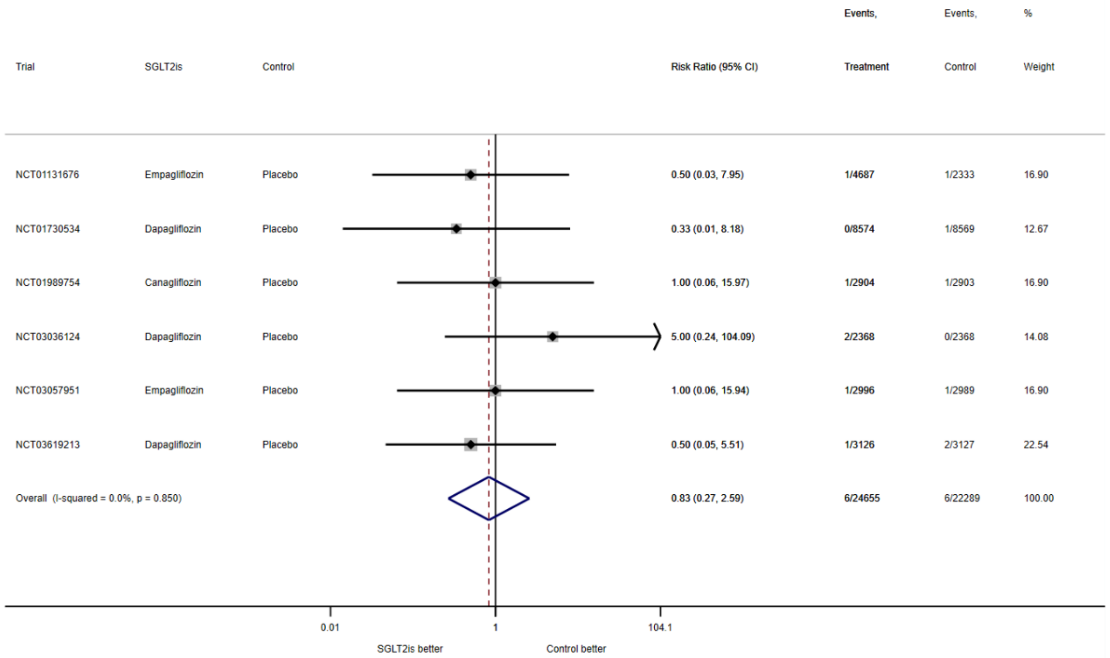

System 8-General Disorders-Figure S17 Meta-analysis of SGLT2is and Malaise

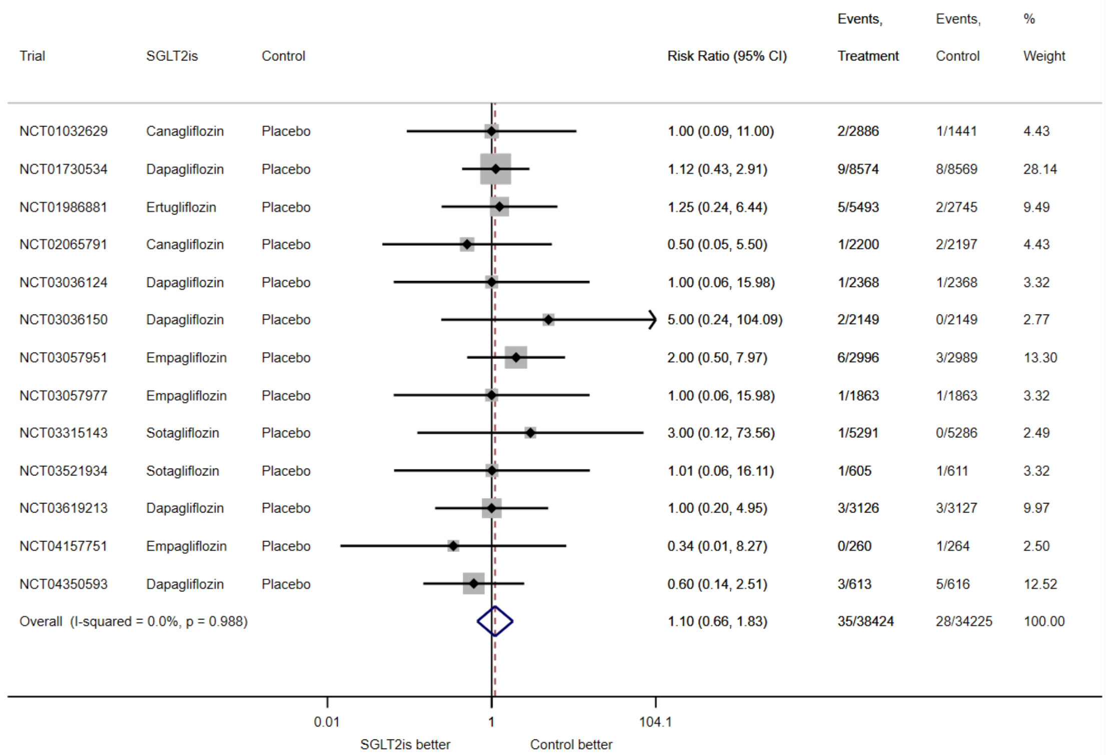

System 8-General Disorders-Figure S18 Meta-analysis of SGLT2is and Multiple organ dysfunction syndrome

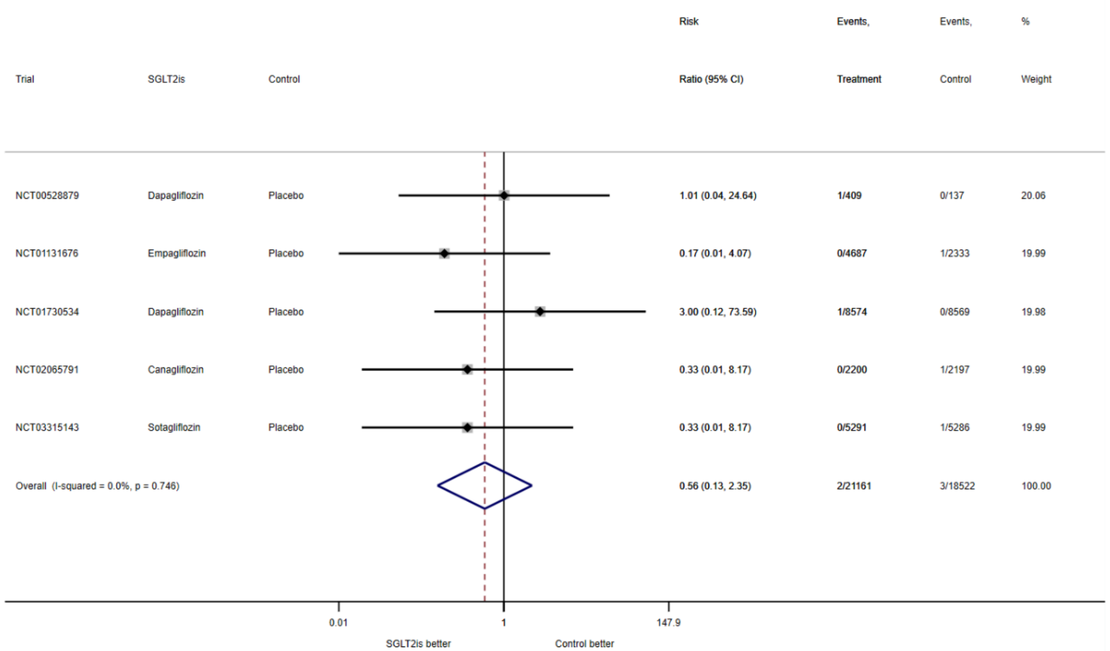

System 8-General Disorders-Figure S19 Meta-analysis of SGLT2is and Necrobiosis

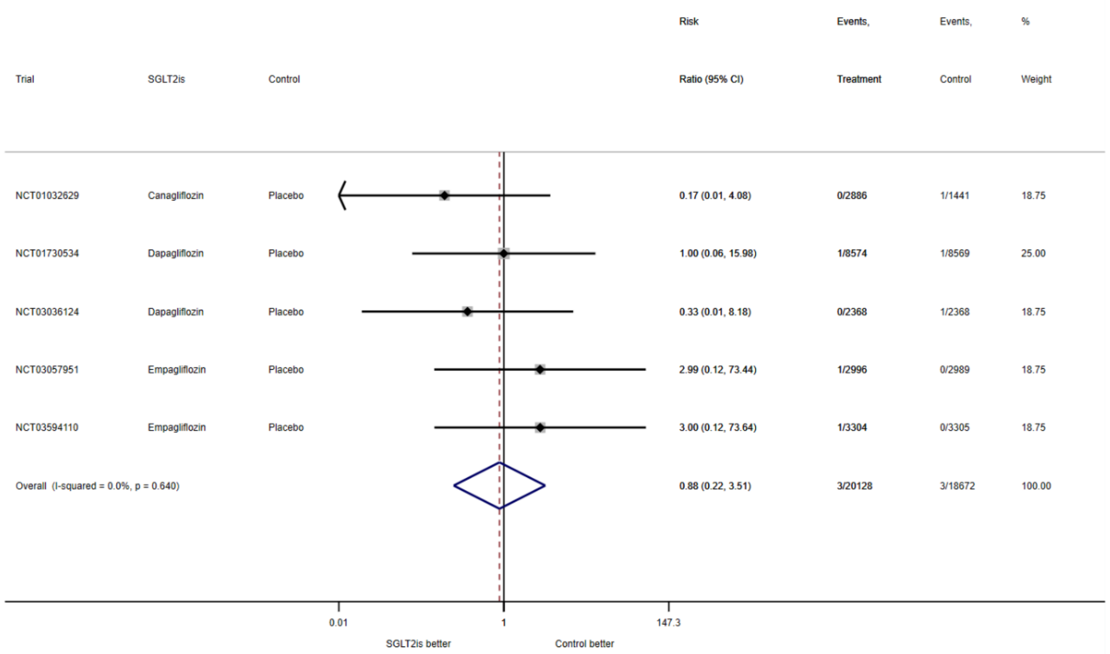

System 8-General Disorders-Figure S20 Meta-analysis of SGLT2is and Necrosis

Trial	SGLT2is	Control	Risk Ratio (95% CI)	Events, Treatment	Events, Control	% Weight
NCT00673231	Dapagliflozin	Placebo	0.97 (0.04, 23.77)	1/610	0/197	0.35
NCT01032629	Canagliflozin	Placebo	3.25 (0.73, 14.36)	13/2886	2/1441	1.63
NCT01106651	Canagliflozin	Placebo	0.25 (0.02, 2.73)	1/477	2/237	0.63
NCT01131676	Empagliflozin	Placebo	1.66 (0.67, 4.13)	20/4687	6/2333	4.34
NCT01730534	Dapagliflozin	Placebo	0.87 (0.66, 1.15)	93/8574	107/8569	47.16
NCT01986881	Ertugliflozin	Placebo	0.95 (0.57, 1.59)	42/5493	22/2745	13.63
NCT01989754	Canagliflozin	Placebo	1.29 (0.48, 3.45)	9/2904	7/2903	3.70
NCT02065791	Canagliflozin	Placebo	3.99 (0.85, 18.79)	8/2200	2/2197	1.50
NCT02384941	Sotagliflozin	Placebo	1.53 (0.06, 37.53)	1/525	0/268	0.35
NCT03036124	Dapagliflozin	Placebo	0.38 (0.15, 0.96)	6/2368	16/2368	4.10
NCT03036150	Dapagliflozin	Placebo	0.60 (0.14, 2.51)	3/2149	5/2149	1.76
NCT03057951	Empagliflozin	Placebo	0.87 (0.43, 1.79)	14/2996	16/2989	7.03
NCT03057977	Empagliflozin	Placebo	0.71 (0.23, 2.25)	5/1863	7/1863	2.74
NCT03242252	Sotagliflozin	Placebo	1.48 (0.06, 36.28)	1/527	0/260	0.35
NCT03315143	Sotagliflozin	Placebo	0.83 (0.36, 1.93)	10/5291	12/5286	5.12
NCT03521934	Sotagliflozin	Placebo	0.34 (0.04, 3.23)	1/605	3/611	0.70
NCT03594110	Empagliflozin	Placebo	1.33 (0.30, 5.95)	4/3304	3/3305	1.61
NCT03619213	Dapagliflozin	Placebo	1.00 (0.35, 2.85)	7/3126	7/3127	3.29
Overall (I-squared = 0.0%, p = 0.600)			0.92 (0.76, 1.11)	239/50585	217/42848	100.00

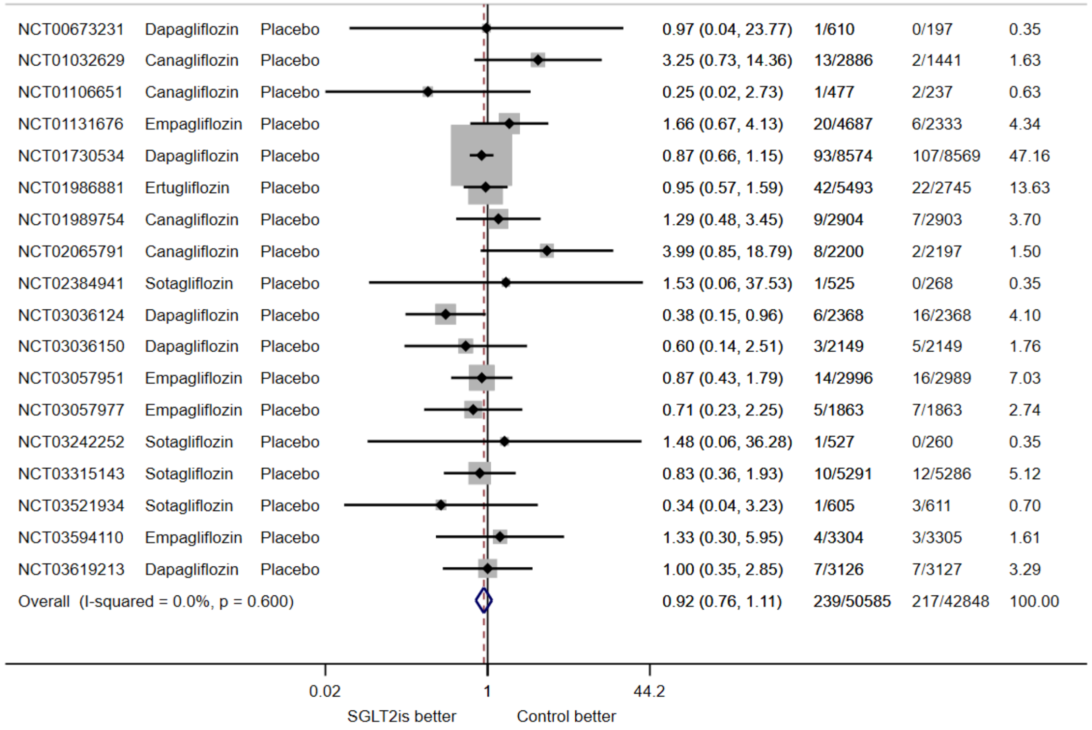

System 8-General Disorders-Figure S21 Meta-analysis of SGLT2is and Non-cardiac chest pain

Trial	SGLT2is	Control	Risk Ratio (95% CI)	Events, Treatment	Events, Control	% Weight
NCT01032629	Canagliflozin	Placebo	0.67 (0.15, 2.97)	4/2886	3/1441	16.19
NCT01131676	Empagliflozin	Placebo	0.20 (0.04, 1.03)	2/4687	5/2333	13.48
NCT01164501	Empagliflozin	Placebo	0.25 (0.01, 6.21)	0/419	1/319	3.54
NCT01730534	Dapagliflozin	Placebo	0.20 (0.02, 1.71)	1/8574	5/8569	7.86
NCT01989754	Canagliflozin	Placebo	0.20 (0.01, 4.16)	0/2904	2/2903	3.93
NCT02065791	Canagliflozin	Placebo	0.75 (0.17, 3.34)	3/2200	4/2197	16.18
NCT03036150	Dapagliflozin	Placebo	2.00 (0.18, 22.04)	2/2149	1/2149	6.29
NCT03057951	Empagliflozin	Placebo	0.20 (0.01, 4.15)	0/2996	2/2989	3.93
NCT03057977	Empagliflozin	Placebo	1.50 (0.25, 8.97)	3/1863	2/1863	11.32
NCT03315143	Sotagliflozin	Placebo	0.50 (0.05, 5.51)	1/5291	2/5286	6.29
NCT03521934	Sotagliflozin	Placebo	0.20 (0.01, 4.20)	0/605	2/611	3.93
NCT03594110	Empagliflozin	Placebo	3.00 (0.12, 73.64)	1/3304	0/3305	3.54
NCT03619213	Dapagliflozin	Placebo	0.33 (0.01, 8.18)	0/3126	1/3127	3.54
Overall (I-squared = 0.0%, p = 0.821)			0.52 (0.29, 0.95)	17/41004	30/37092	100.00

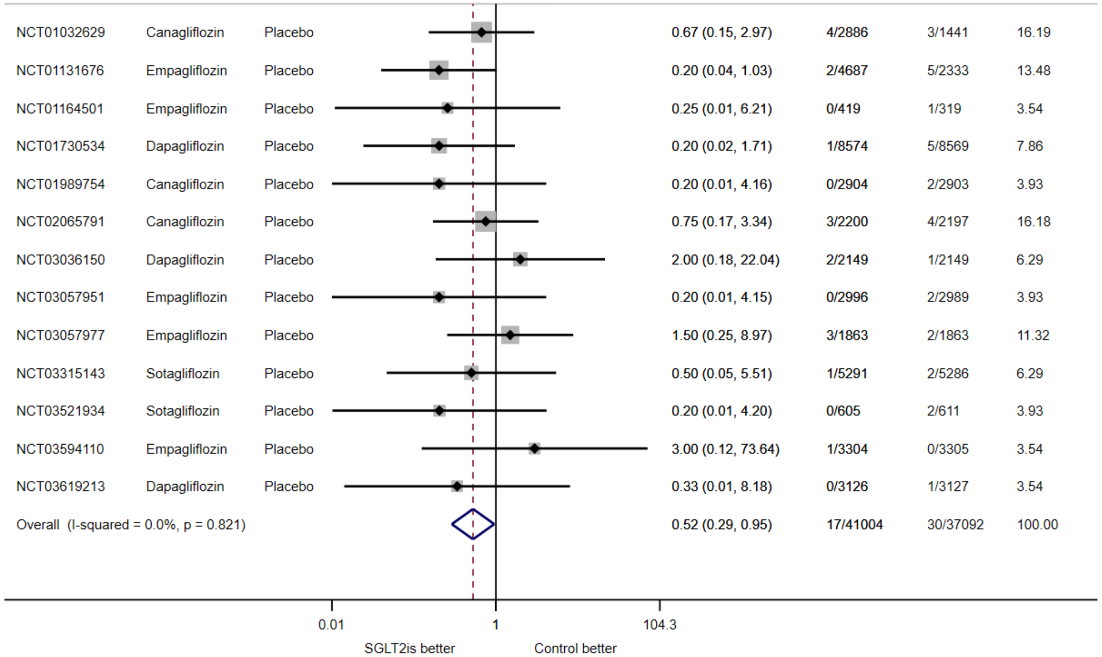

System 8-General Disorders-Figure S22 Meta-analysis of SGLT2is and Oedema peripheral

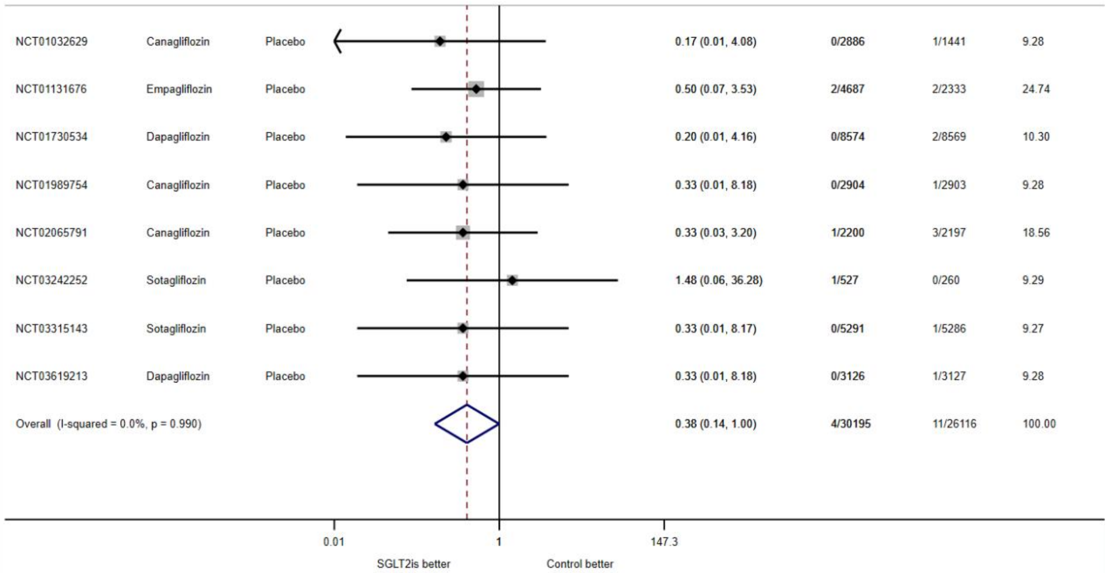

Trial	SGLT2is	Control	Risk Ratio (95% CI)	Events, Treatment	Events, Control	% Weight
NCT01032629	Canagliflozin	Placebo	0.17 (0.01, 4.08)	0/2886	1/1441	9.28
NCT01131676	Empagliflozin	Placebo	0.50 (0.07, 3.53)	2/4687	2/2333	24.74
NCT01730534	Dapagliflozin	Placebo	0.20 (0.01, 4.16)	0/8574	2/8569	10.30
NCT01989754	Canagliflozin	Placebo	0.33 (0.01, 8.18)	0/2904	1/2903	9.28
NCT02065791	Canagliflozin	Placebo	0.33 (0.03, 3.20)	1/2200	3/2197	18.56
NCT03242252	Sotagliflozin	Placebo	1.48 (0.06, 36.28)	1/527	0/260	9.29
NCT03315143	Sotagliflozin	Placebo	0.33 (0.01, 8.17)	0/5291	1/5286	9.27
NCT03619213	Dapagliflozin	Placebo	0.33 (0.01, 8.18)	0/3126	1/3127	9.28
Overall (I-squared = 0.0%, p = 0.990)			0.38 (0.14, 1.00)	4/30195	11/26116	100.00

System 8-General Disorders-Figure S23 Meta-analysis of SGLT2is and Peripheral swelling

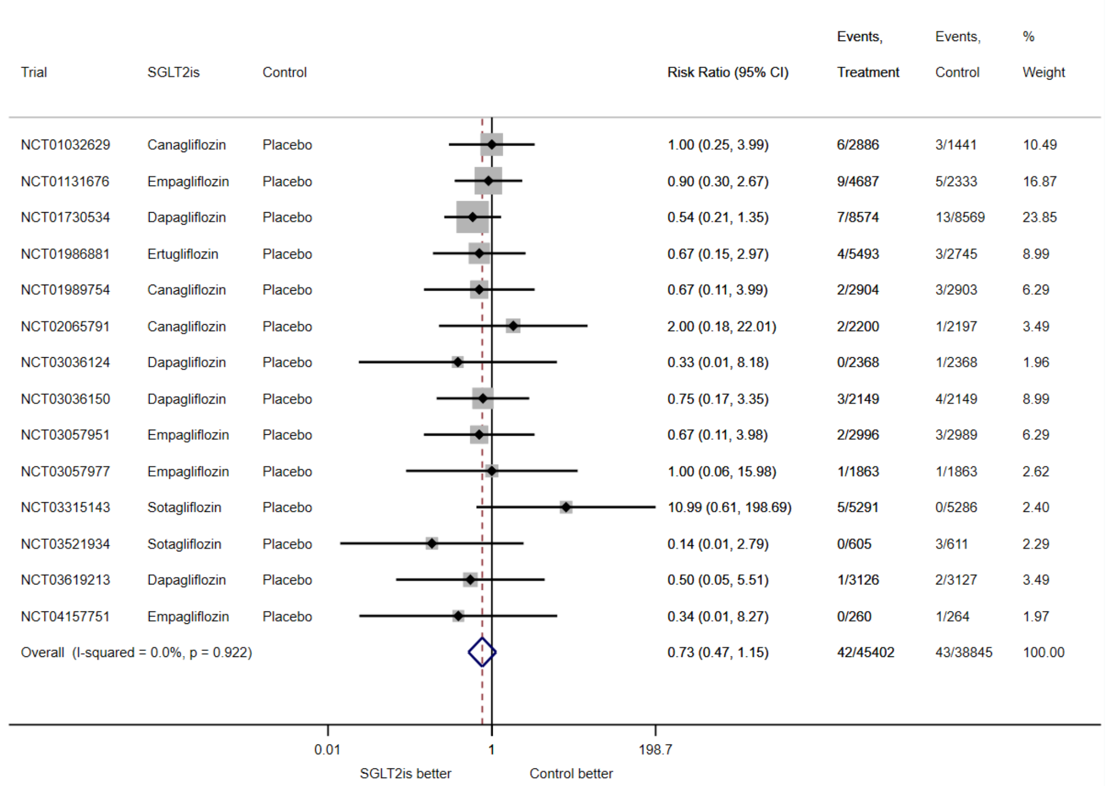

System 8-General Disorders-Figure S24 Meta-analysis of SGLT2is and Pyrexia

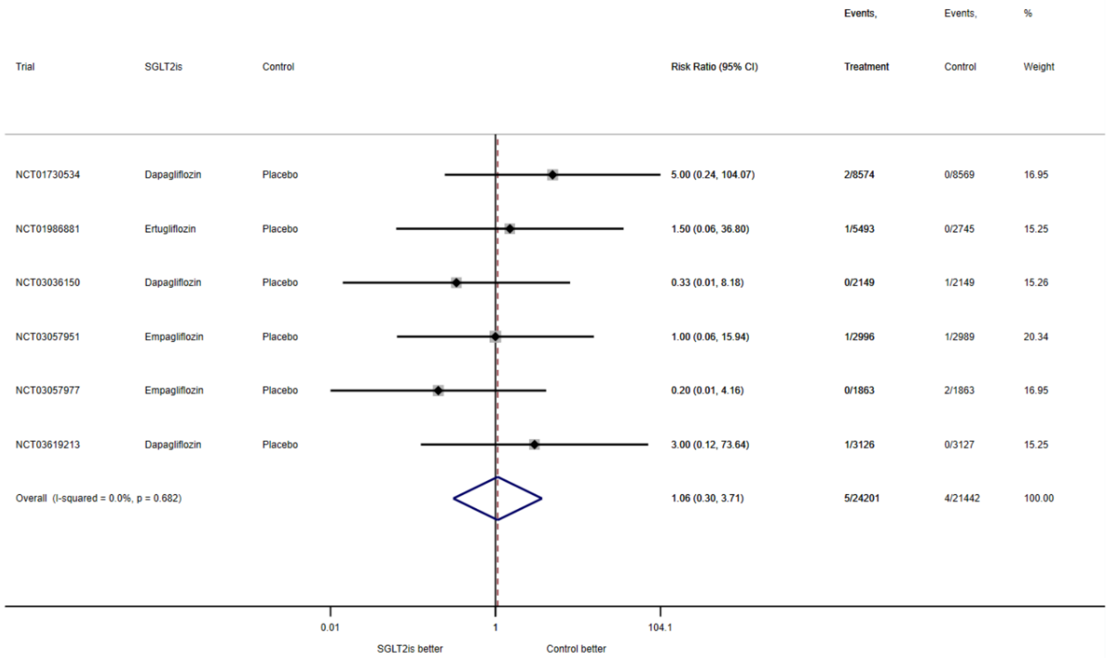

System 8-General Disorders-Figure S25 Meta-analysis of SGLT2is and Stent-graft endoleak

Trial	SGLT2is	Control	Risk Ratio (95% CI)	Events, Treatment	Events, Control	% Weight
NCT01032629	Canagliflozin	Placebo	0.90 (0.30, 2.68)	9/2886	5/1441	4.84
NCT01131676	Empagliflozin	Placebo	1.00 (0.18, 5.43)	4/4687	2/2333	2.00
NCT01164501	Empagliflozin	Placebo	0.25 (0.01, 6.21)	0/419	1/319	0.56
NCT01730534	Dapagliflozin	Placebo	0.87 (0.43, 1.79)	14/8574	16/8569	11.22
NCT01986881	Ertugliflozin	Placebo	1.17 (0.30, 4.51)	7/5493	3/2745	3.15
NCT01989754	Canagliflozin	Placebo	0.33 (0.01, 8.18)	0/2904	1/2903	0.56
NCT02065791	Canagliflozin	Placebo	1.00 (0.14, 7.08)	2/2200	2/2197	1.50
NCT03036124	Dapagliflozin	Placebo	0.67 (0.37, 1.21)	18/2368	27/2368	16.35
NCT03036150	Dapagliflozin	Placebo	0.50 (0.05, 5.51)	1/2149	2/2149	1.00
NCT03057951	Empagliflozin	Placebo	0.44 (0.14, 1.44)	4/2996	9/2989	4.16
NCT03057977	Empagliflozin	Placebo	0.80 (0.32, 2.02)	8/1863	10/1863	6.70
NCT03315143	Sotagliflozin	Placebo	1.75 (0.73, 4.16)	14/5291	8/5286	7.65
NCT03521934	Sotagliflozin	Placebo	0.79 (0.29, 2.10)	7/605	9/611	5.99
NCT03594110	Empagliflozin	Placebo	0.71 (0.34, 1.48)	12/3304	17/3305	10.60
NCT03619213	Dapagliflozin	Placebo	0.70 (0.43, 1.16)	26/3126	37/3127	23.14
NCT04157751	Empagliflozin	Placebo	0.34 (0.01, 8.27)	0/260	1/264	0.56
Overall (I-squared = 0.0%, p = 0.970)			0.78 (0.61, 0.99)	126/49125	150/42469	100.00

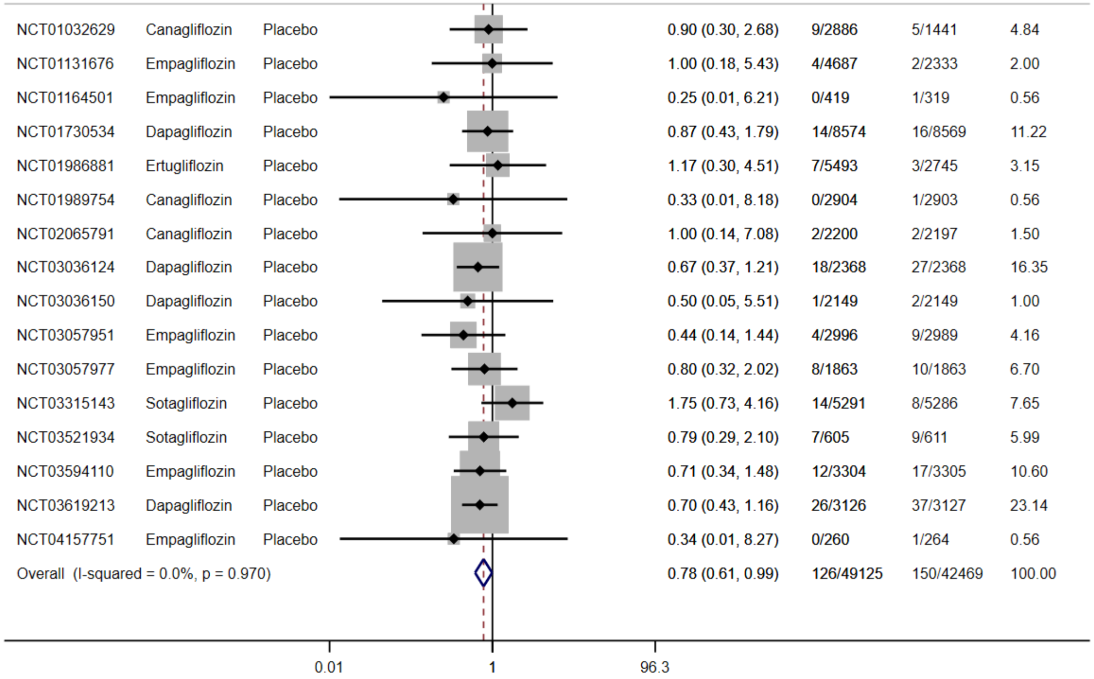

System 8-General Disorders-Figure S26 Meta-analysis of SGLT2is and Sudden cardiac death

Trial	SGLT2is	Control	Risk Ratio (95% CI)	Events, Treatment	Events, Control	% Weight
NCT01031680	Dapagliflozin	Placebo	3.01 (0.12, 73.77)	1/460	0/462	0.66
NCT01032629	Canagliflozin	Placebo	0.67 (0.23, 1.92)	8/2886	6/1441	6.07
NCT01131676	Empagliflozin	Placebo	1.08 (0.41, 2.83)	13/4687	6/2333	7.26
NCT01730534	Dapagliflozin	Placebo	0.91 (0.51, 1.65)	21/8574	23/8569	19.41
NCT01986881	Ertugliflozin	Placebo	0.25 (0.05, 1.36)	2/5493	4/2745	2.35
NCT01989754	Canagliflozin	Placebo	1.00 (0.32, 3.10)	6/2904	6/2903	5.30
NCT02065791	Canagliflozin	Placebo	0.37 (0.10, 1.41)	3/2200	8/2197	3.86
NCT03036124	Dapagliflozin	Placebo	1.90 (0.89, 4.08)	19/2368	10/2368	11.62
NCT03036150	Dapagliflozin	Placebo	4.00 (0.45, 35.76)	4/2149	1/2149	1.41
NCT03057951	Empagliflozin	Placebo	1.25 (0.49, 3.16)	10/2996	8/2989	7.86
NCT03057977	Empagliflozin	Placebo	1.00 (0.29, 3.45)	5/1863	5/1863	4.42
NCT03315143	Sotagliflozin	Placebo	1.40 (0.44, 4.40)	7/5291	5/5286	5.15
NCT03521934	Sotagliflozin	Placebo	1.01 (0.14, 7.15)	2/605	2/611	1.77
NCT03594110	Empagliflozin	Placebo	0.33 (0.01, 8.18)	0/3304	1/3305	0.66
NCT03619213	Dapagliflozin	Placebo	0.92 (0.53, 1.60)	24/3126	26/3127	22.19
Overall (I-squared = 0.0%, p = 0.674)			1.00 (0.77, 1.30)	125/48906	111/42348	100.00

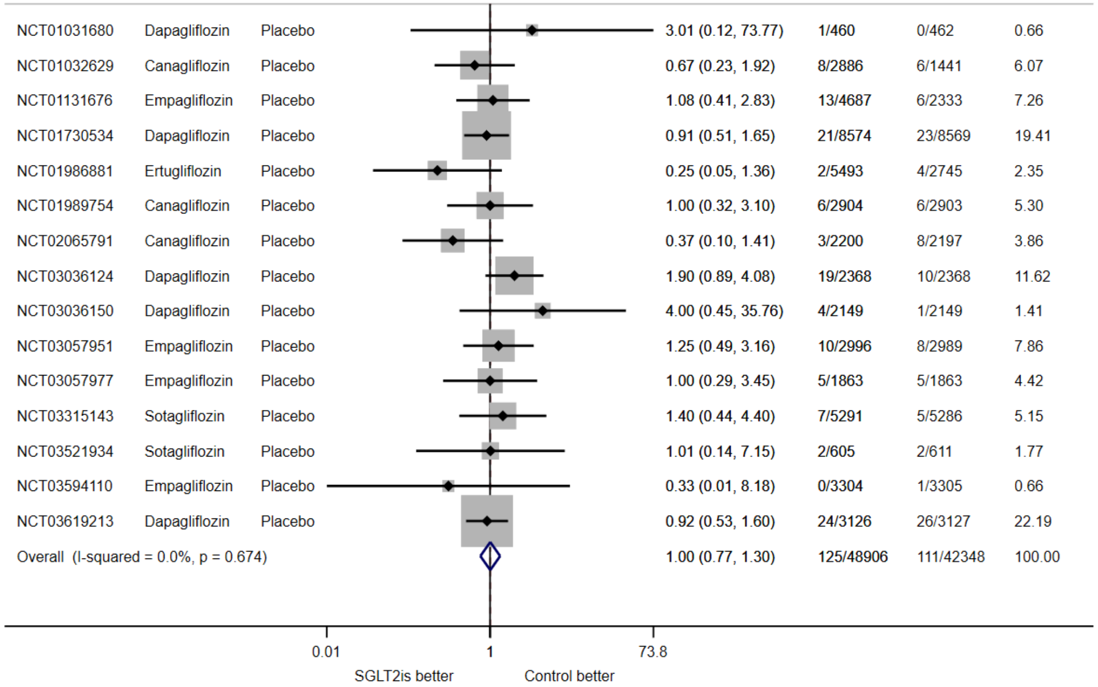

System 8-General Disorders-Figure S27 Meta-analysis of SGLT2is and Sudden death

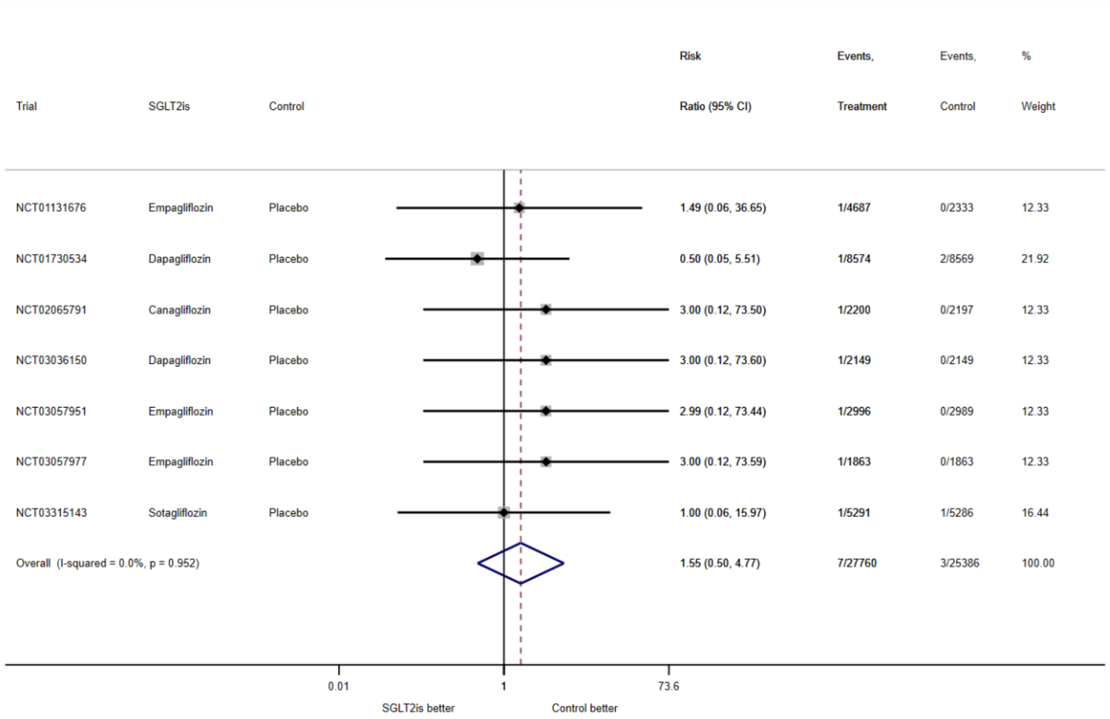

System 8-General Disorders-Figure S28 Meta-analysis of SGLT2is and Systemic inflammatory response syndrome

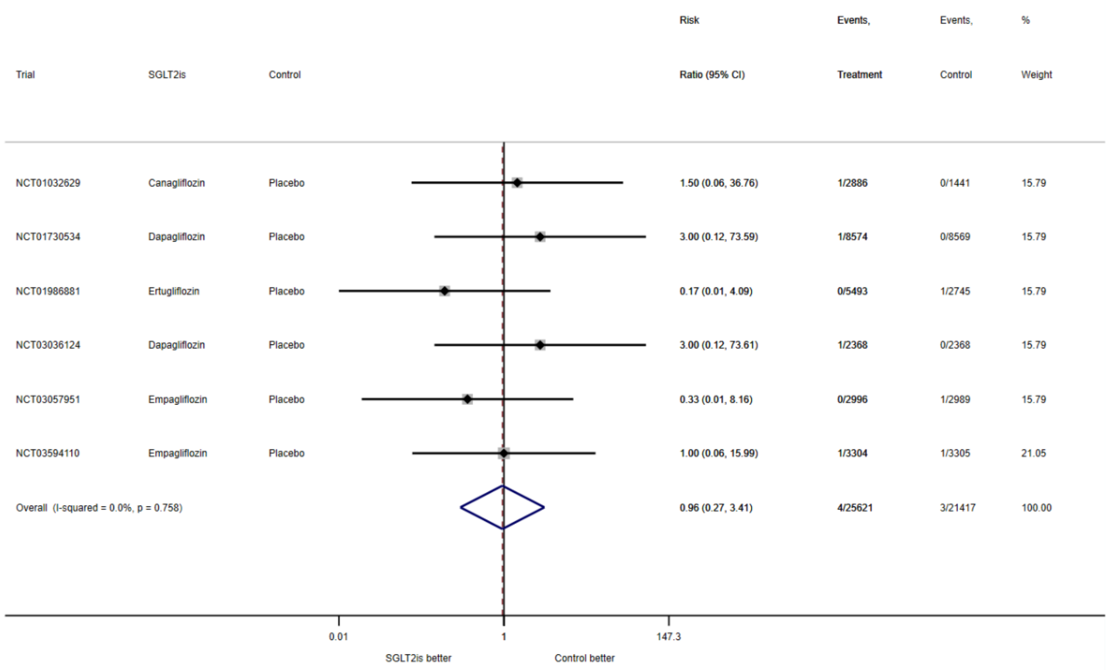

System 8-General Disorders-Figure S29 Meta-analysis of SGLT2is and Vascular stent occlusion

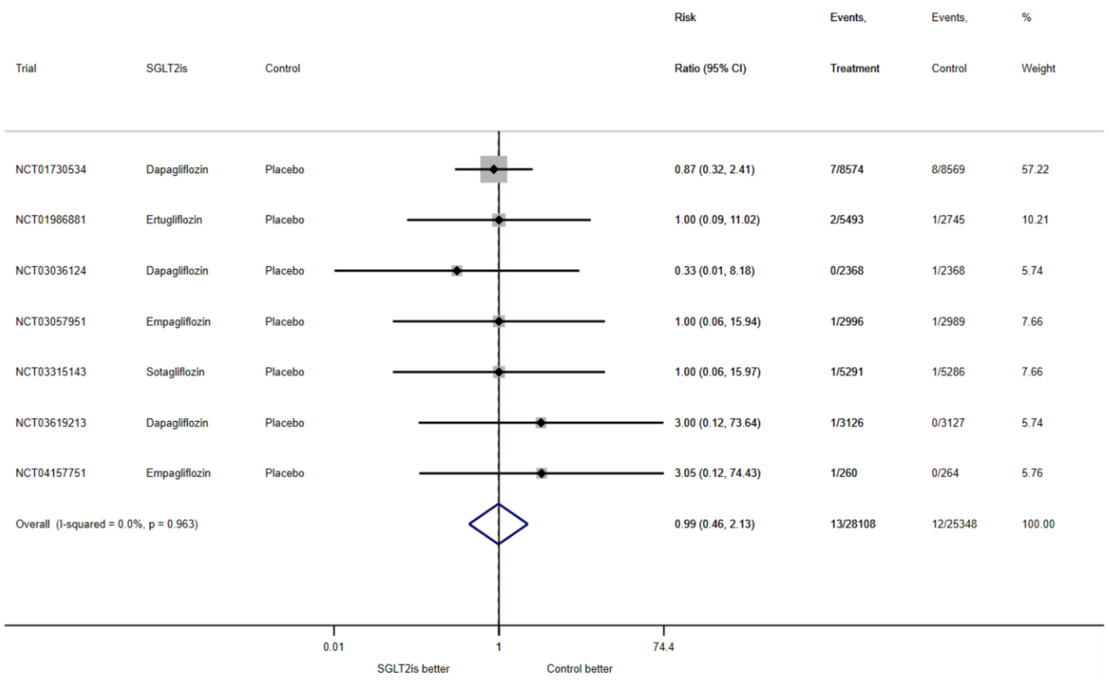

System 8-General Disorders-Figure S30 Meta-analysis of SGLT2is and Vascular stent stenosis

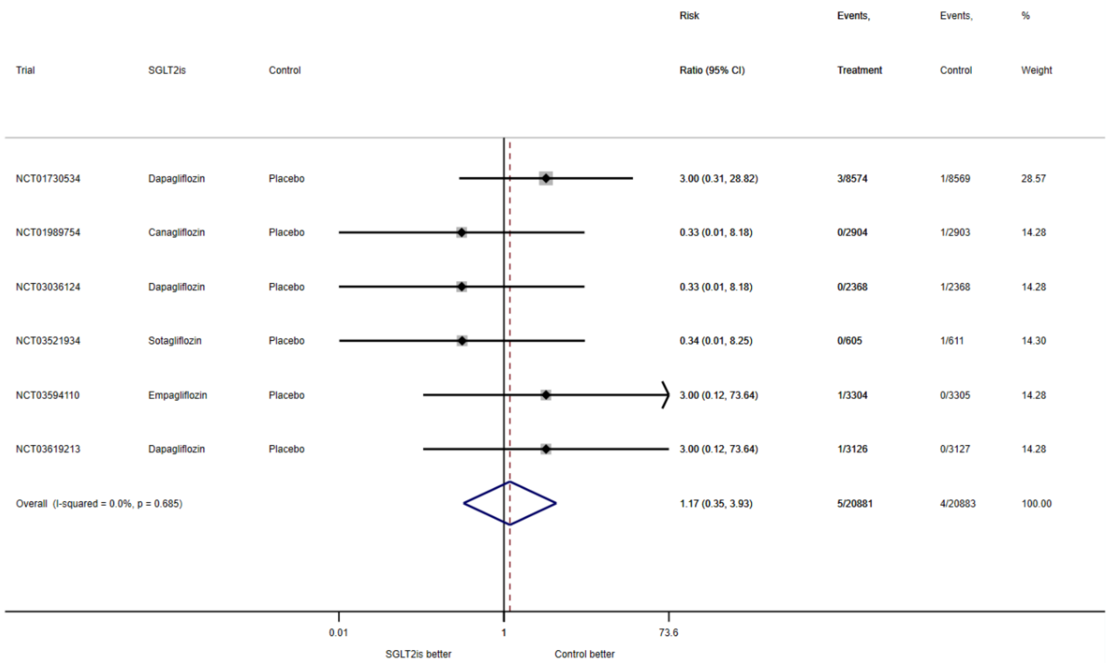

System 8-General Disorders-Figure S31 Meta-analysis of SGLT2is and Vascular stent thrombosis

Trial	SGLT2is	Control	Risk Ratio (95% CI)	Events, Treatment	Events, Control	% Weight
NCT01032629	Canagliflozin	Placebo	1.50 (0.06, 36.76)	1/2886	0/1441	7.20
NCT01106651	Canagliflozin	Placebo	1.49 (0.06, 36.53)	1/477	0/237	7.21
NCT01131676	Empagliflozin	Placebo	1.49 (0.06, 36.65)	1/4687	0/2333	7.20
NCT01730534	Dapagliflozin	Placebo	1.00 (0.14, 7.09)	2/8574	2/8569	19.19
NCT01986881	Ertugliflozin	Placebo	0.25 (0.02, 2.75)	1/5493	2/2745	12.80
NCT02065791	Canagliflozin	Placebo	0.33 (0.01, 8.17)	0/2200	1/2197	7.20
NCT03036124	Dapagliflozin	Placebo	0.33 (0.01, 8.18)	0/2368	1/2368	7.20
NCT03036150	Dapagliflozin	Placebo	5.00 (0.24, 104.09)	2/2149	0/2149	8.00
NCT03057977	Empagliflozin	Placebo	0.33 (0.01, 8.18)	0/1863	1/1863	7.20
NCT03315143	Sotagliflozin	Placebo	1.00 (0.06, 15.97)	1/5291	1/5286	9.60
NCT03521934	Sotagliflozin	Placebo	3.03 (0.12, 74.22)	1/605	0/611	7.21
Overall (I-squared = 0.0%, p = 0.933)			0.89 (0.38, 2.09)	10/36593	8/29799	100.00

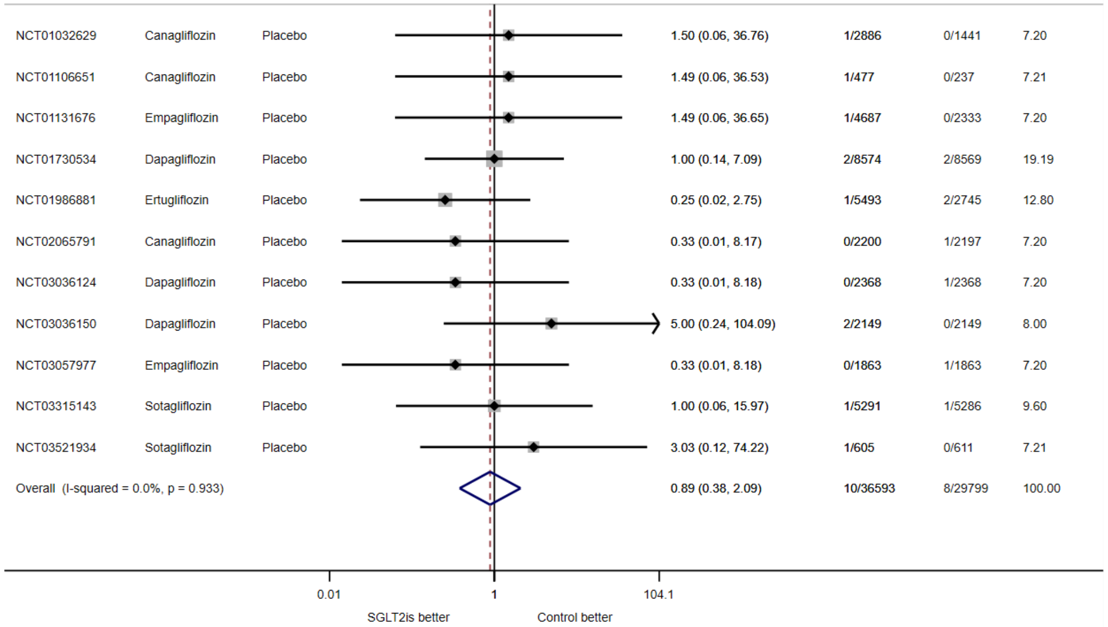

System 9-Hepatobiliary Disorders-Figure S1 Meta-analysis of SGLT2is and Bile duct obstruction

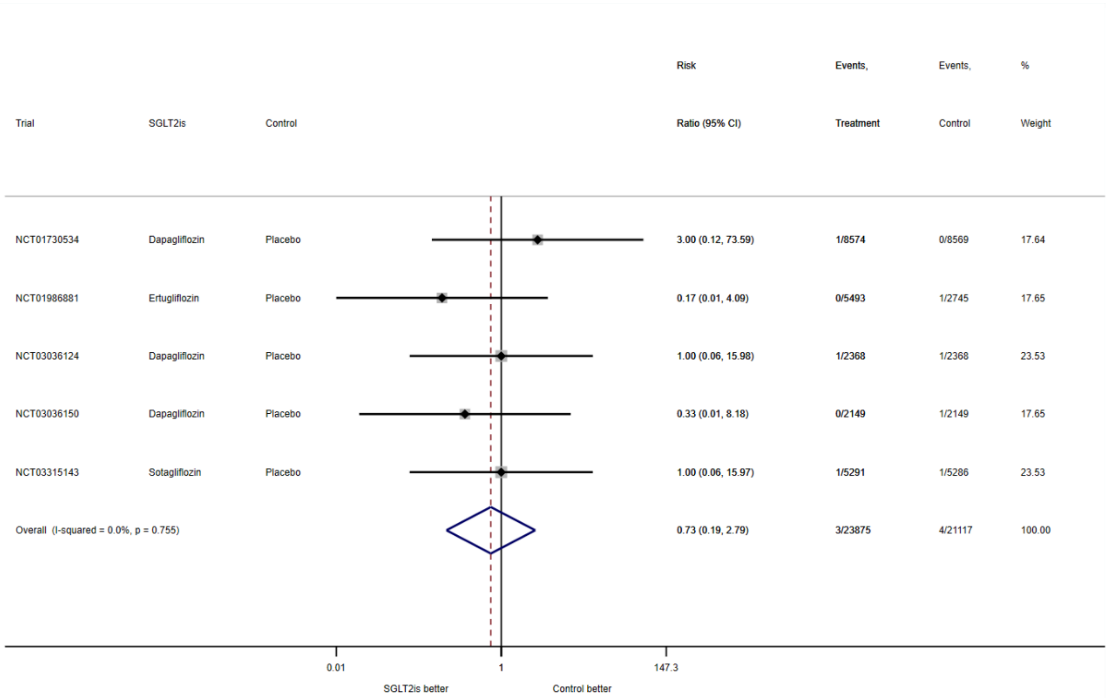

System 9-Hepatobiliary Disorders-Figure S2 Meta-analysis of SGLT2is and Bile duct stenosis

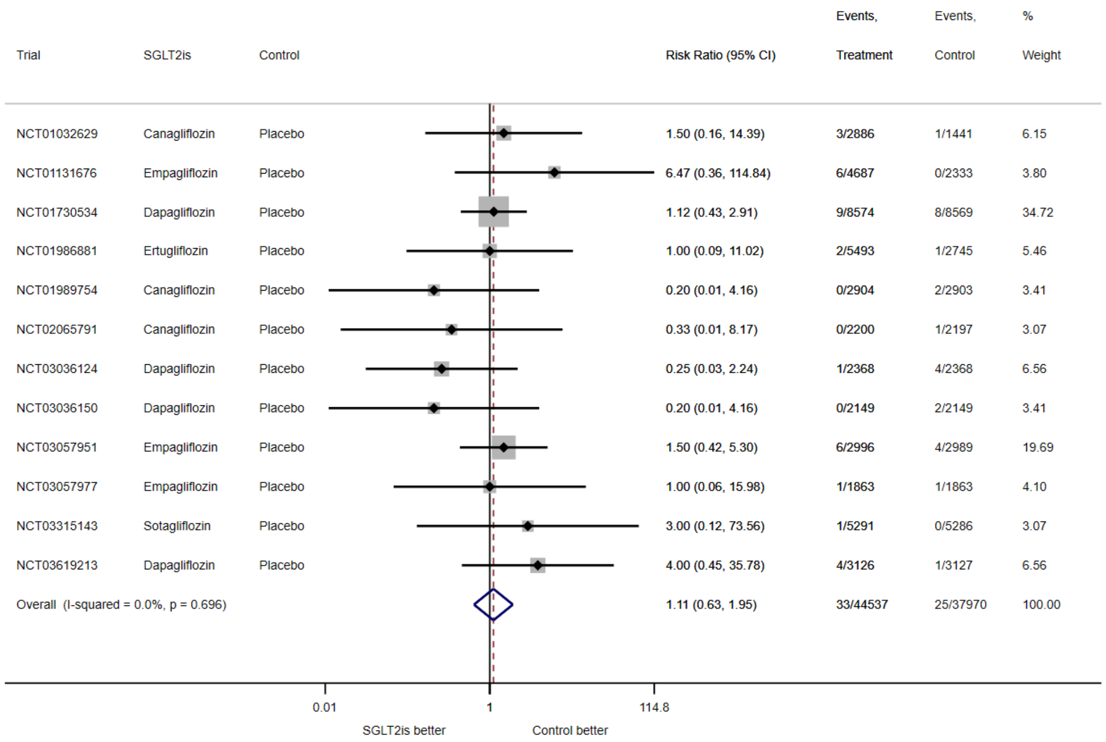

System 9-Hepatobiliary Disorders-Figure S3 Meta-analysis of SGLT2is and Bile duct stone

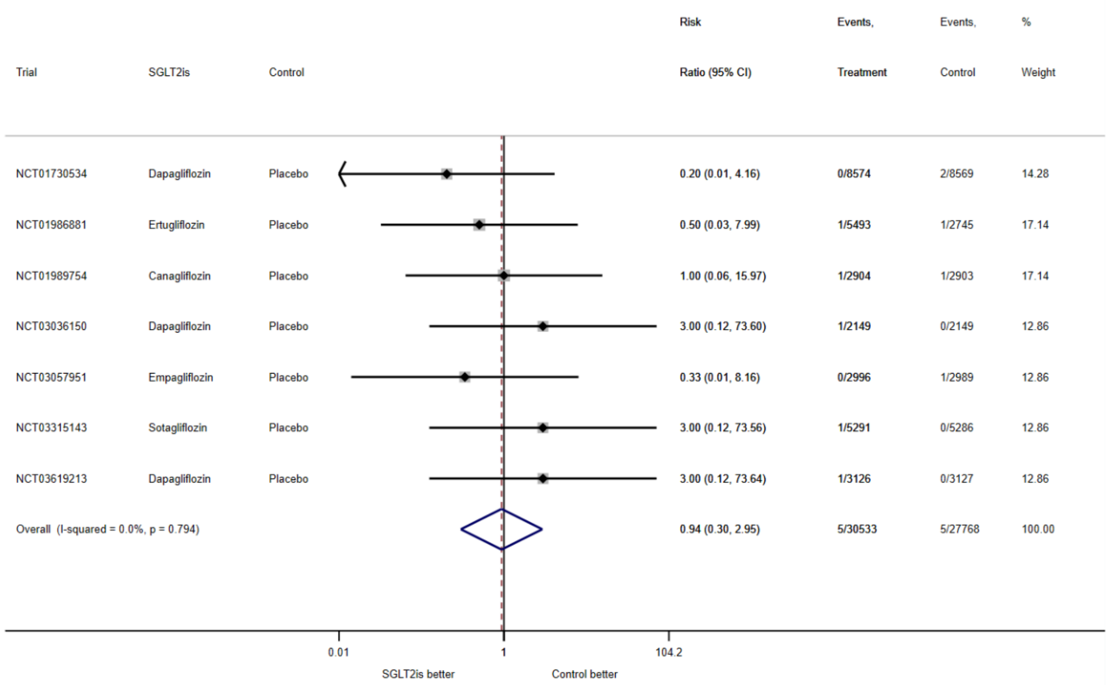

System 9-Hepatobiliary Disorders-Figure S4 Meta-analysis of SGLT2is and Biliary colic

Trial	SGLT2is	Control	Risk Ratio (95% CI)	Events, Treatment	Events, Control	% Weight
NCT01032629	Canagliflozin	Placebo	1.50 (0.06, 36.76)	1/2886	0/1441	3.08
NCT01106677	Canagliflozin	Sitagliptin	1.50 (0.06, 36.63)	1/735	0/366	3.08
NCT01131676	Empagliflozin	Placebo	0.50 (0.03, 7.95)	1/4687	1/2333	4.10
NCT01730534	Dapagliflozin	Placebo	2.20 (0.76, 6.33)	11/8574	5/8569	28.22
NCT01986881	Ertugliflozin	Placebo	0.17 (0.01, 4.09)	0/5493	1/2745	3.08
NCT01989754	Canagliflozin	Placebo	5.00 (0.24, 104.07)	2/2904	0/2903	3.42
NCT02065791	Canagliflozin	Placebo	0.33 (0.01, 8.17)	0/2200	1/2197	3.08
NCT03036124	Dapagliflozin	Placebo	0.33 (0.01, 8.18)	0/2368	1/2368	3.08
NCT03036150	Dapagliflozin	Placebo	1.00 (0.25, 3.99)	4/2149	4/2149	16.44
NCT03057951	Empagliflozin	Placebo	1.50 (0.25, 8.95)	3/2996	2/2989	9.85
NCT03057977	Empagliflozin	Placebo	0.33 (0.01, 8.18)	0/1863	1/1863	3.08
NCT03315143	Sotagliflozin	Placebo	3.00 (0.12, 73.56)	1/5291	0/5286	3.08
NCT03594110	Empagliflozin	Placebo	4.00 (0.45, 35.78)	4/3304	1/3305	6.57
NCT03619213	Dapagliflozin	Placebo	0.67 (0.11, 3.99)	2/3126	3/3127	9.85
Overall (I-squared = 0.0%, p = 0.855)			1.27 (0.73, 2.23)	30/48576	20/41641	100.00

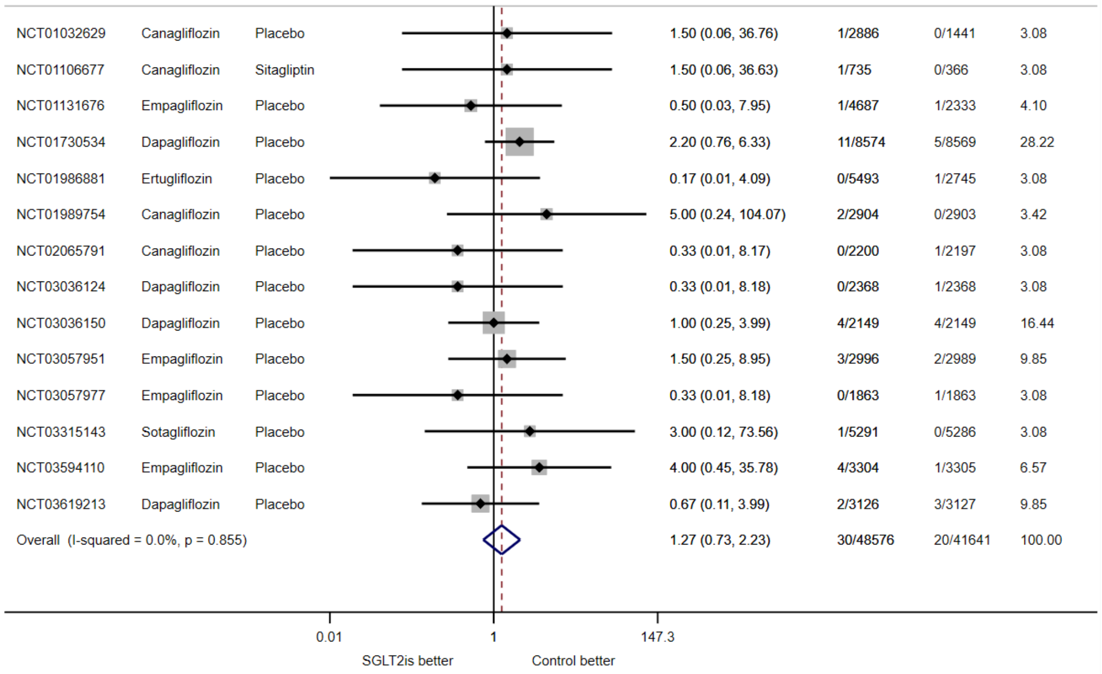

System 9-Hepatobiliary Disorders-Figure S5 Meta-analysis of SGLT2is and Cholangitis

Trial	SGLT2is	Control	Risk Ratio (95% CI)	Events, Treatment	Events, Control	% Weight
NCT01032629	Canagliflozin	Placebo	1.50 (0.06, 36.76)	1/2886	0/1441	5.74
NCT01730534	Dapagliflozin	Placebo	2.00 (0.37, 10.91)	4/8574	2/8569	20.40
NCT01989754	Canagliflozin	Placebo	0.33 (0.01, 8.18)	0/2904	1/2903	5.74
NCT02065791	Canagliflozin	Placebo	3.00 (0.12, 73.50)	1/2200	0/2197	5.74
NCT03036124	Dapagliflozin	Placebo	1.00 (0.06, 15.98)	1/2368	1/2368	7.65
NCT03036150	Dapagliflozin	Placebo	0.33 (0.01, 8.18)	0/2149	1/2149	5.74
NCT03057951	Empagliflozin	Placebo	2.99 (0.31, 28.76)	3/2996	1/2989	11.48
NCT03057977	Empagliflozin	Placebo	1.00 (0.06, 15.98)	1/1863	1/1863	7.65
NCT03315143	Sotagliflozin	Placebo	3.00 (0.12, 73.56)	1/5291	0/5286	5.74
NCT03521934	Sotagliflozin	Placebo	3.03 (0.12, 74.22)	1/605	0/611	5.74
NCT03619213	Dapagliflozin	Placebo	0.67 (0.11, 3.99)	2/3126	3/3127	18.37
Overall (I-squared = 0.0%, p = 0.965)			1.32 (0.61, 2.85)	15/34962	10/33503	100.00

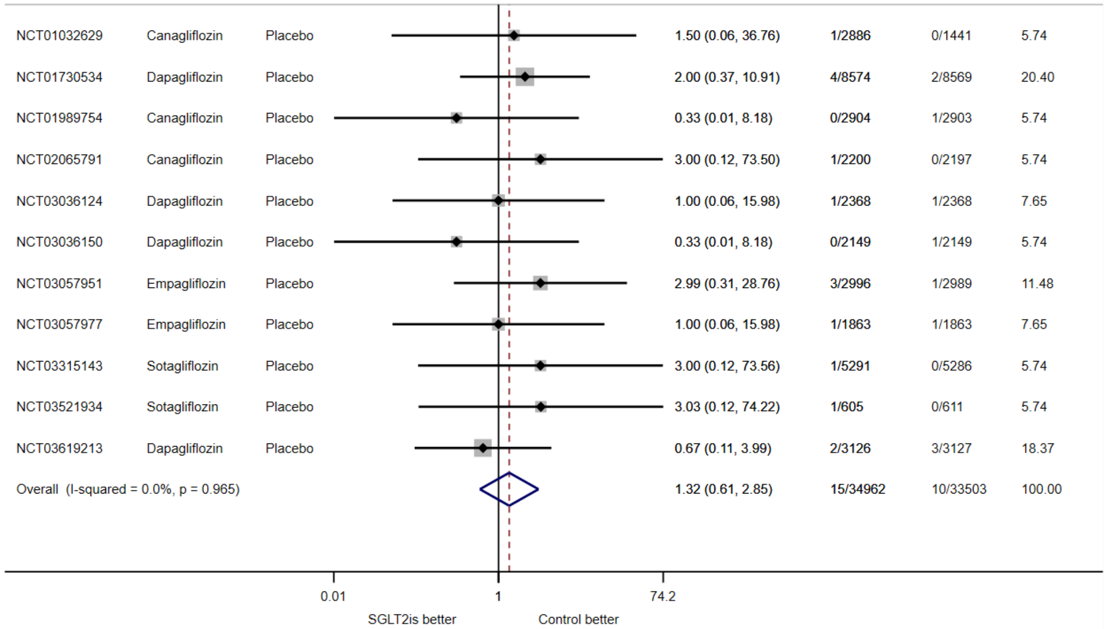

System 9-Hepatobiliary Disorders-Figure S6 Meta-analysis of SGLT2is and Cholangitis acute

Trial	SGLT2is	Control	Risk Ratio (95% CI)	Events, Treatment	Events, Control	% Weight
NCT00528879	Dapagliflozin	Placebo	1.01 (0.04, 24.64)	1/409	0/137	0.92
NCT01032629	Canagliflozin	Placebo	2.50 (0.55, 11.38)	10/2886	2/1441	4.09
NCT01106651	Canagliflozin	Placebo	0.50 (0.03, 7.91)	1/477	1/237	1.23
NCT01106677	Canagliflozin	Sitagliptin	1.50 (0.06, 36.63)	1/735	0/366	0.92
NCT01131676	Empagliflozin	Placebo	1.12 (0.35, 3.63)	9/4687	4/2333	6.80
NCT01164501	Empagliflozin	Placebo	0.15 (0.01, 3.16)	0/419	2/319	1.02
NCT01177813	Empagliflozin	Sitagliptin or Placebo	0.34 (0.01, 8.25)	0/447	1/452	0.92
NCT01730534	Dapagliflozin	Placebo	0.82 (0.44, 1.52)	18/8574	22/8569	24.33
NCT01986881	Ertugliflozin	Placebo	0.75 (0.27, 2.10)	9/5493	6/2745	8.84
NCT01989754	Canagliflozin	Placebo	1.00 (0.20, 4.95)	3/2904	3/2903	3.68
NCT02033889	Ertugliflozin	Placebo/Glimepiride	0.17 (0.01, 4.14)	0/412	1/209	0.92
NCT02065791	Canagliflozin	Placebo	0.40 (0.08, 2.06)	2/2200	5/2197	3.51
NCT02384941	Sotagliflozin	Placebo	1.53 (0.06, 37.53)	1/525	0/268	0.92
NCT03036124	Dapagliflozin	Placebo	0.60 (0.14, 2.51)	3/2368	5/2368	4.60
NCT03036150	Dapagliflozin	Placebo	2.00 (0.18, 22.04)	2/2149	1/2149	1.64
NCT03057951	Empagliflozin	Placebo	1.00 (0.32, 3.09)	6/2996	6/2989	7.37
NCT03057977	Empagliflozin	Placebo	0.67 (0.11, 3.99)	2/1863	3/1863	2.95
NCT03315143	Sotagliflozin	Placebo	0.80 (0.21, 2.97)	4/5291	5/5286	5.45
NCT03521934	Sotagliflozin	Placebo	3.03 (0.12, 74.22)	1/605	0/611	0.92
NCT03594110	Empagliflozin	Placebo	1.50 (0.53, 4.21)	9/3304	6/3305	8.85
NCT03619213	Dapagliflozin	Placebo	1.14 (0.42, 3.15)	8/3126	7/3127	9.18
NCT04157751	Empagliflozin	Placebo	0.34 (0.01, 8.27)	0/260	1/264	0.92
Overall (I-squared = 0.0%, p = 0.988)			0.91 (0.67, 1.23)	90/52130	81/44138	100.00

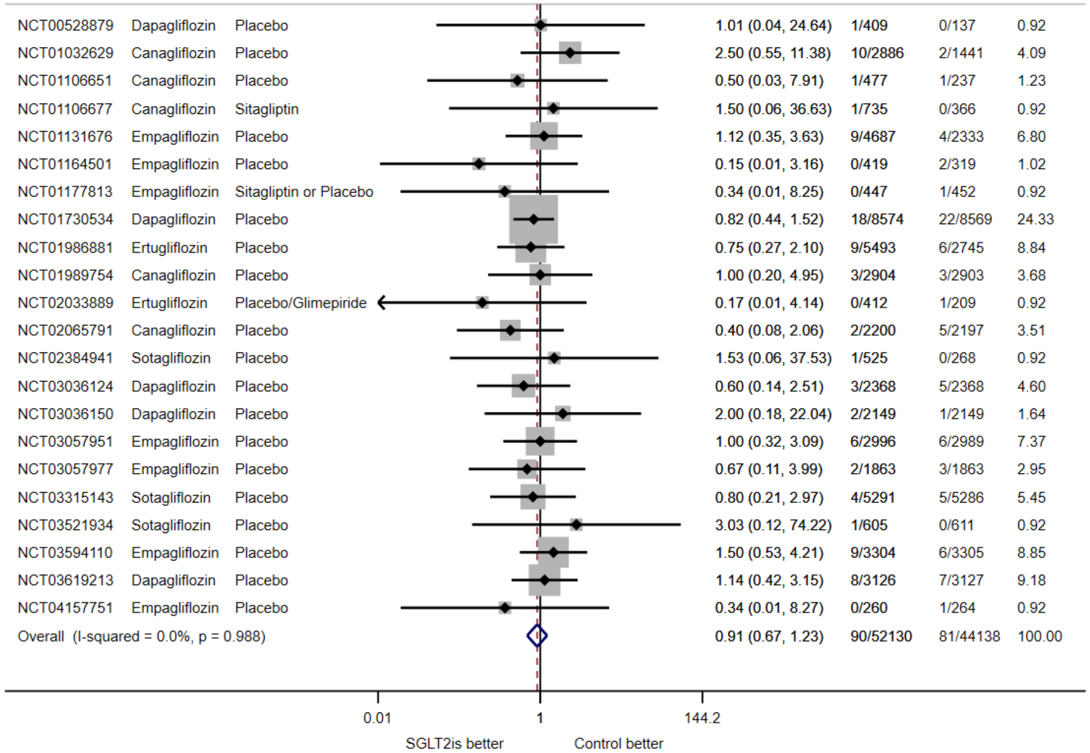

System 9-Hepatobiliary Disorders-Figure S7 Meta-analysis of SGLT2is and Cholecystitis

Trial	SGLT2is	Control	Risk Ratio (95% CI)	Events, Treatment	Events, Control	% Weight
NCT01032629	Canagliflozin	Placebo	1.12 (0.35, 3.64)	9/2886	4/1441	6.28
NCT01106651	Canagliflozin	Placebo	1.49 (0.06, 36.53)	1/477	0/237	0.85
NCT01106677	Canagliflozin	Sitagliptin	1.50 (0.06, 36.63)	1/735	0/366	0.85
NCT01131676	Empagliflozin	Placebo	1.62 (0.53, 4.96)	13/4687	4/2333	6.93
NCT01164501	Empagliflozin	Placebo	2.29 (0.09, 55.92)	1/419	0/319	0.85
NCT01177813	Empagliflozin	Sitagliptin or Placebo	0.20 (0.01, 4.20)	0/447	2/452	0.94
NCT01730534	Dapagliflozin	Placebo	1.14 (0.64, 2.01)	25/8574	22/8569	26.53
NCT01986881	Ertugliflozin	Placebo	0.68 (0.31, 1.48)	15/5493	11/2745	14.40
NCT01989754	Canagliflozin	Placebo	2.00 (0.18, 22.04)	2/2904	1/2903	1.51
NCT02065791	Canagliflozin	Placebo	1.25 (0.34, 4.64)	5/2200	4/2197	5.03
NCT03036124	Dapagliflozin	Placebo	0.75 (0.26, 2.16)	6/2368	8/2368	7.77
NCT03036150	Dapagliflozin	Placebo	0.60 (0.14, 2.51)	3/2149	5/2149	4.25
NCT03057951	Empagliflozin	Placebo	1.33 (0.30, 5.94)	4/2996	3/2989	3.88
NCT03057977	Empagliflozin	Placebo	0.40 (0.08, 2.06)	2/1863	5/1863	3.23
NCT03315143	Sotagliflozin	Placebo	1.00 (0.42, 2.40)	10/5291	10/5286	11.32
NCT03521934	Sotagliflozin	Placebo	3.03 (0.12, 74.22)	1/605	0/611	0.85
NCT03619213	Dapagliflozin	Placebo	0.50 (0.13, 2.00)	3/3126	6/3127	4.53
Overall (I-squared = 0.0%, p = 0.969)			0.96 (0.71, 1.28)	101/47220	85/39955	100.00

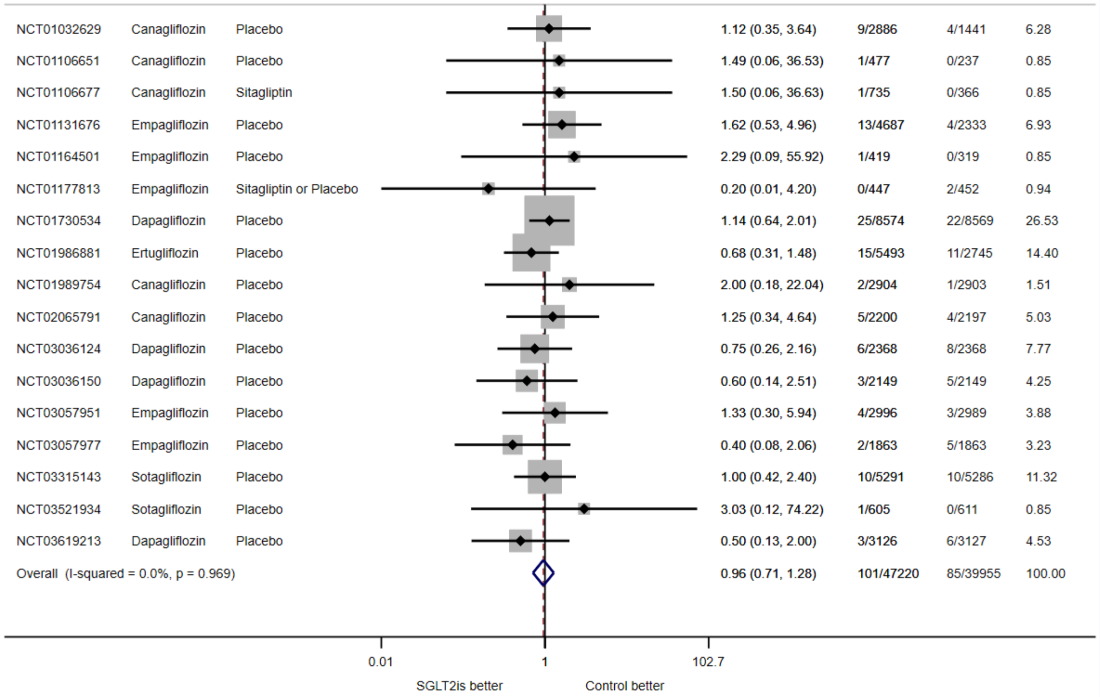

System 9-Hepatobiliary Disorders-Figure S8 Meta-analysis of SGLT2is and Cholecystitis acute

Trial	SGLT2is	Control	Risk Ratio (95% CI)	Events, Treatment	Events, Control	% Weight
NCT01032629	Canagliflozin	Placebo	1.50 (0.06, 36.76)	1/2886	0/1441	6.28
NCT01131676	Empagliflozin	Placebo	0.17 (0.01, 4.07)	0/4687	1/2333	6.28
NCT01730534	Dapagliflozin	Placebo	0.17 (0.02, 1.38)	1/8574	6/8569	14.34
NCT01986881	Ertugliflozin	Placebo	4.50 (0.24, 83.52)	4/5493	0/2745	7.53
NCT02065791	Canagliflozin	Placebo	3.00 (0.31, 28.78)	3/2200	1/2197	12.56
NCT03036124	Dapagliflozin	Placebo	1.00 (0.06, 15.98)	1/2368	1/2368	8.37
NCT03036150	Dapagliflozin	Placebo	1.00 (0.14, 7.09)	2/2149	2/2149	16.75
NCT03057951	Empagliflozin	Placebo	2.99 (0.12, 73.44)	1/2996	0/2989	6.28
NCT03057977	Empagliflozin	Placebo	0.33 (0.01, 8.18)	0/1863	1/1863	6.28
NCT03315143	Sotagliflozin	Placebo	1.00 (0.06, 15.97)	1/5291	1/5286	8.37
NCT03619213	Dapagliflozin	Placebo	5.00 (0.24, 104.14)	2/3126	0/3127	6.97
Overall (I-squared = 0.0%, p = 0.636)			1.02 (0.46, 2.27)	16/41633	13/35067	100.00

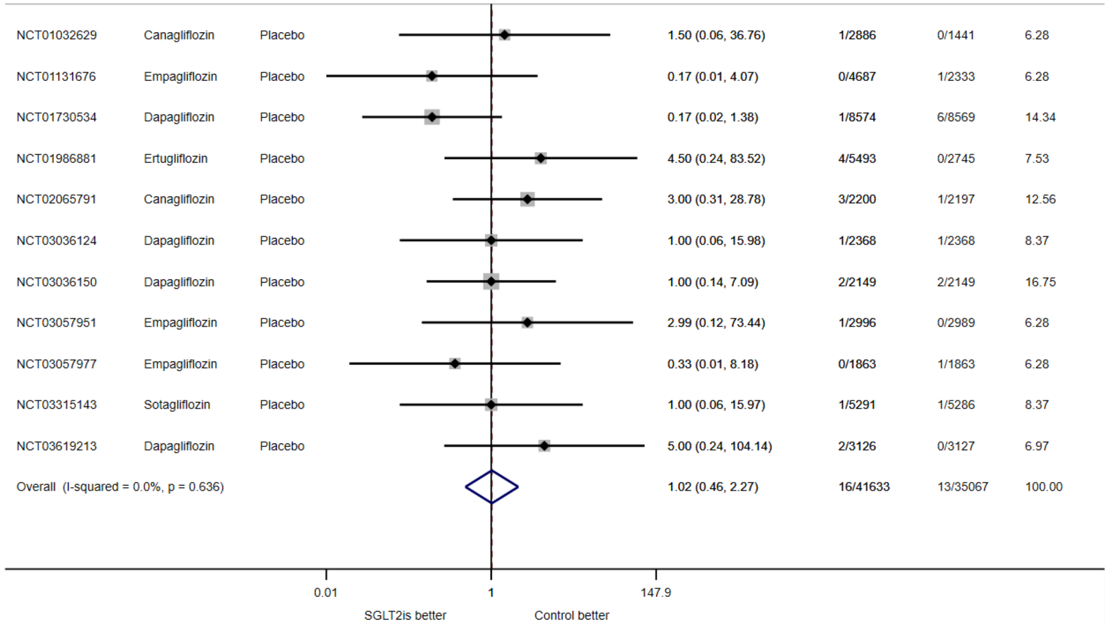

System 9-Hepatobiliary Disorders-Figure S9 Meta-analysis of SGLT2is and Cholecystitis chronic

Trial	SGLT2is	Control	Risk Ratio (95% CI)	Events, Treatment	Events, Control	% Weight
NCT00673231	Dapagliflozin	Placebo	0.97 (0.04, 23.77)	1/610	0/197	0.77
NCT01031680	Dapagliflozin	Placebo	3.01 (0.12, 73.77)	1/460	0/462	0.77
NCT01032629	Canagliflozin	Placebo	0.73 (0.34, 1.56)	16/2886	11/1441	13.43
NCT01106651	Canagliflozin	Placebo	1.49 (0.06, 36.53)	1/477	0/237	0.77
NCT01131676	Empagliflozin	Placebo	1.49 (0.64, 3.51)	21/4687	7/2333	10.78
NCT01164501	Empagliflozin	Placebo	0.76 (0.05, 12.13)	1/419	1/319	1.03
NCT01730534	Dapagliflozin	Placebo	0.68 (0.42, 1.13)	26/8574	38/8569	31.70
NCT01986881	Ertugliflozin	Placebo	1.17 (0.45, 3.03)	14/5493	6/2745	8.61
NCT01989754	Canagliflozin	Placebo	0.71 (0.23, 2.25)	5/2904	7/2903	5.98
NCT02033889	Ertugliflozin	Placebo/Glimepiride	1.53 (0.06, 37.28)	1/412	0/209	0.77
NCT02065791	Canagliflozin	Placebo	2.50 (0.48, 12.85)	5/2200	2/2197	2.93
NCT03036124	Dapagliflozin	Placebo	2.00 (0.18, 22.04)	2/2368	1/2368	1.36
NCT03036150	Dapagliflozin	Placebo	7.00 (0.36, 135.44)	3/2149	0/2149	0.90
NCT03057951	Empagliflozin	Placebo	0.86 (0.29, 2.54)	6/2996	7/2989	6.62
NCT03057977	Empagliflozin	Placebo	0.50 (0.05, 5.51)	1/1863	2/1863	1.36
NCT03242252	Sotagliflozin	Placebo	1.48 (0.06, 36.28)	1/527	0/260	0.77
NCT03315143	Sotagliflozin	Placebo	1.25 (0.34, 4.65)	5/5291	4/5286	4.55
NCT03594110	Empagliflozin	Placebo	3.00 (0.61, 14.86)	6/3304	2/3305	3.07
NCT03619213	Dapagliflozin	Placebo	1.67 (0.40, 6.97)	5/3126	3/3127	3.84
Overall (I-squared = 0.0%, p = 0.901)			0.99 (0.74, 1.30)	121/50746	91/42959	100.00

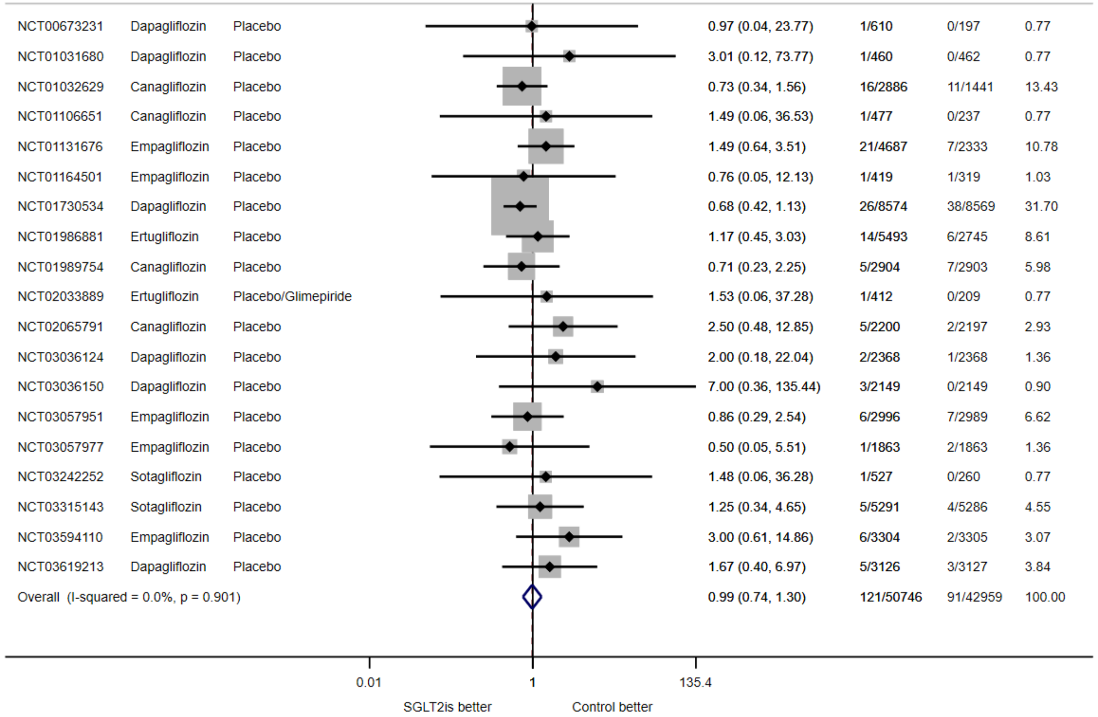

System 9-Hepatobiliary Disorders-Figure S10 Meta-analysis of SGLT2is and Cholelithiasis

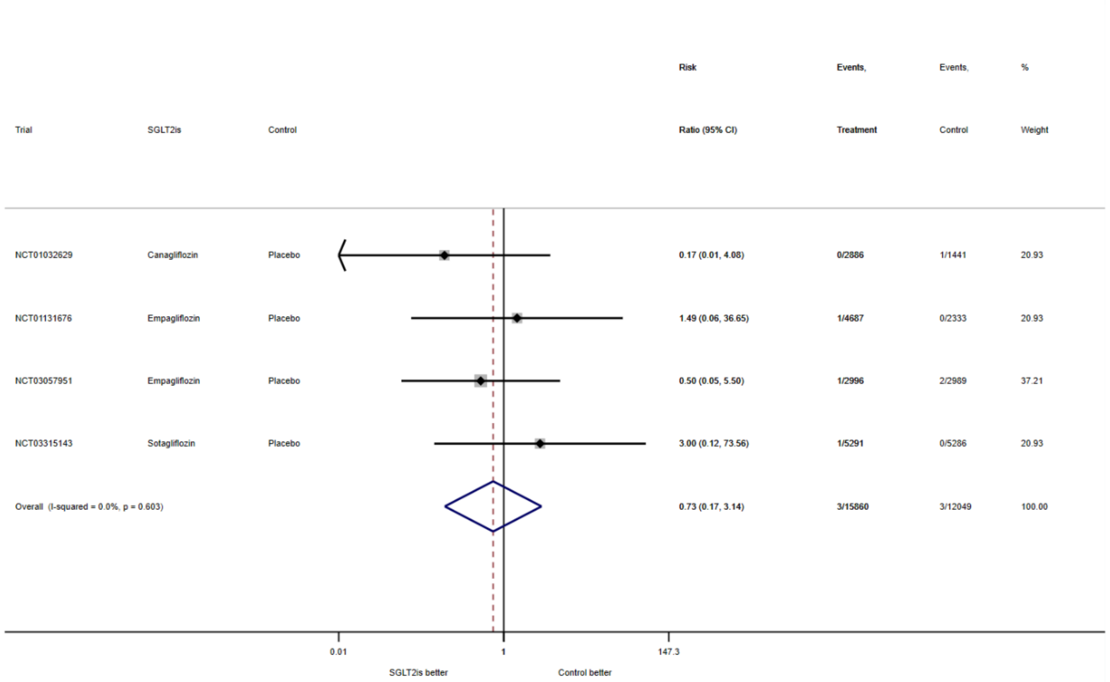

System 9-Hepatobiliary Disorders-Figure S11 Meta-analysis of SGLT2is and Cholestasis

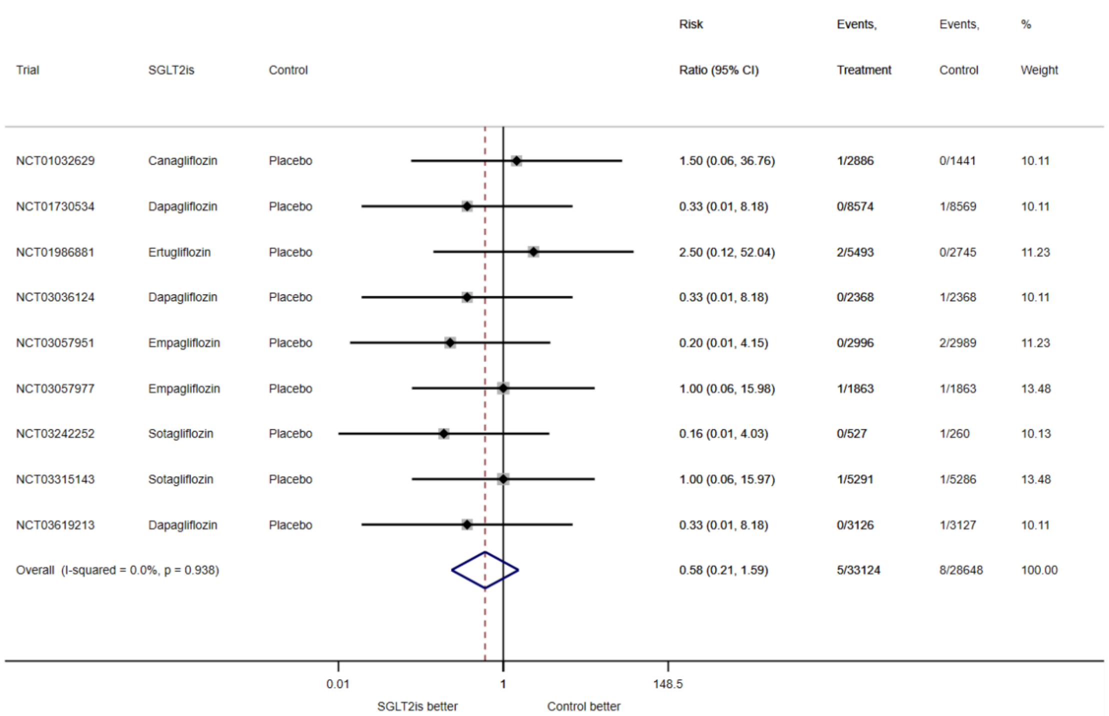

System 9-Hepatobiliary Disorders-Figure S12 Meta-analysis of SGLT2is and Drug-induced liver injury

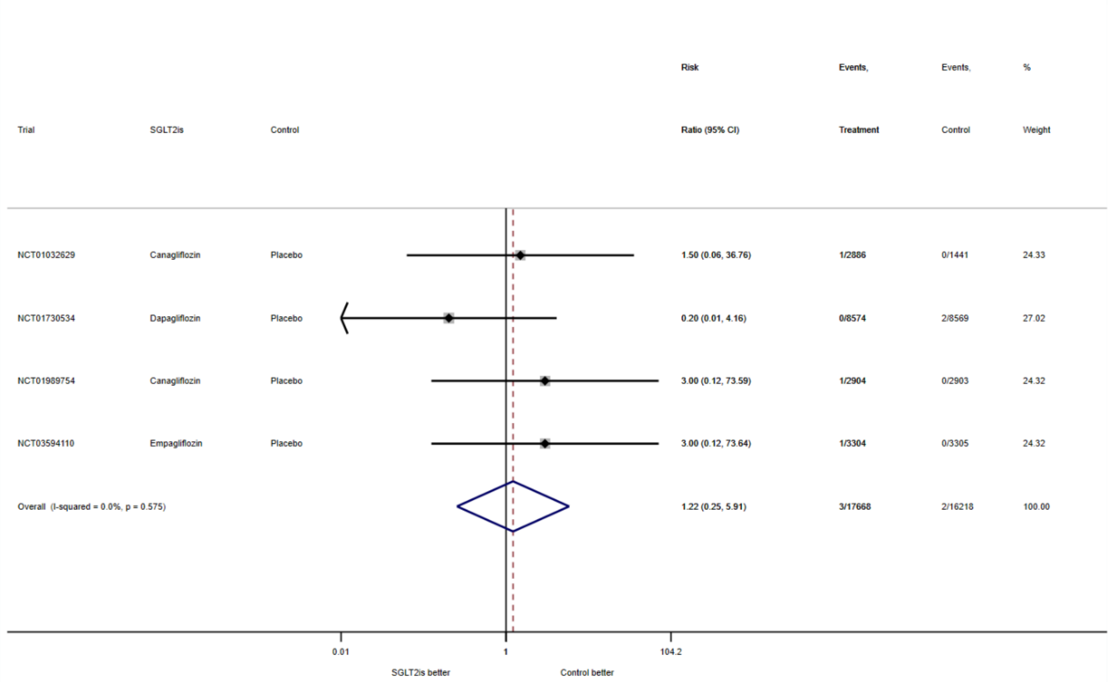

System 9-Hepatobiliary Disorders-Figure S13 Meta-analysis of SGLT2is and Gallbladder polyp

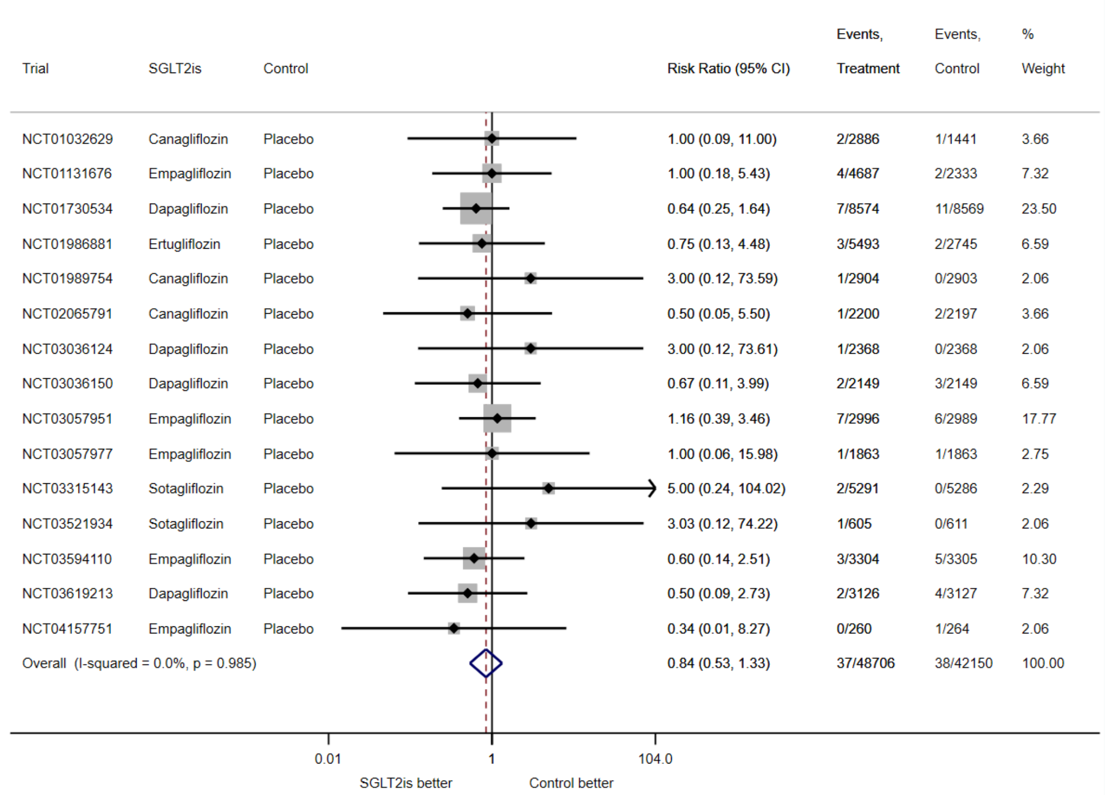

System 9-Hepatobiliary Disorders-Figure S14 Meta-analysis of SGLT2is and Hepatic cirrhosis

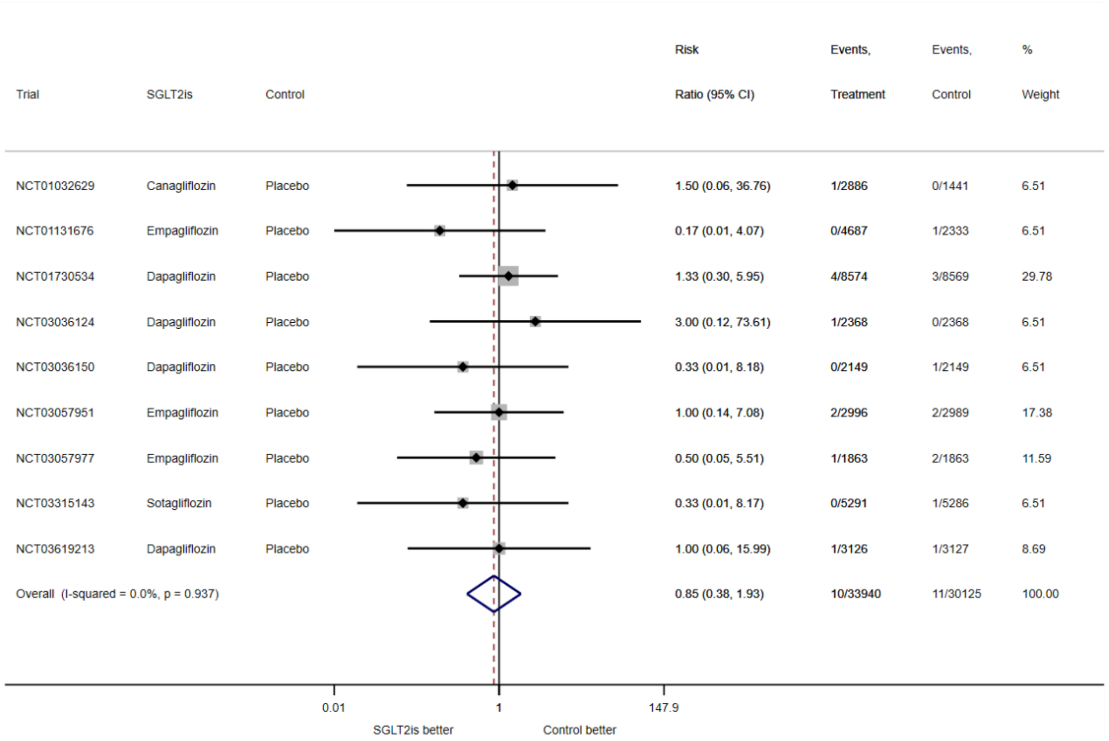

System 9-Hepatobiliary Disorders-Figure S15 Meta-analysis of SGLT2is and Hepatic failure

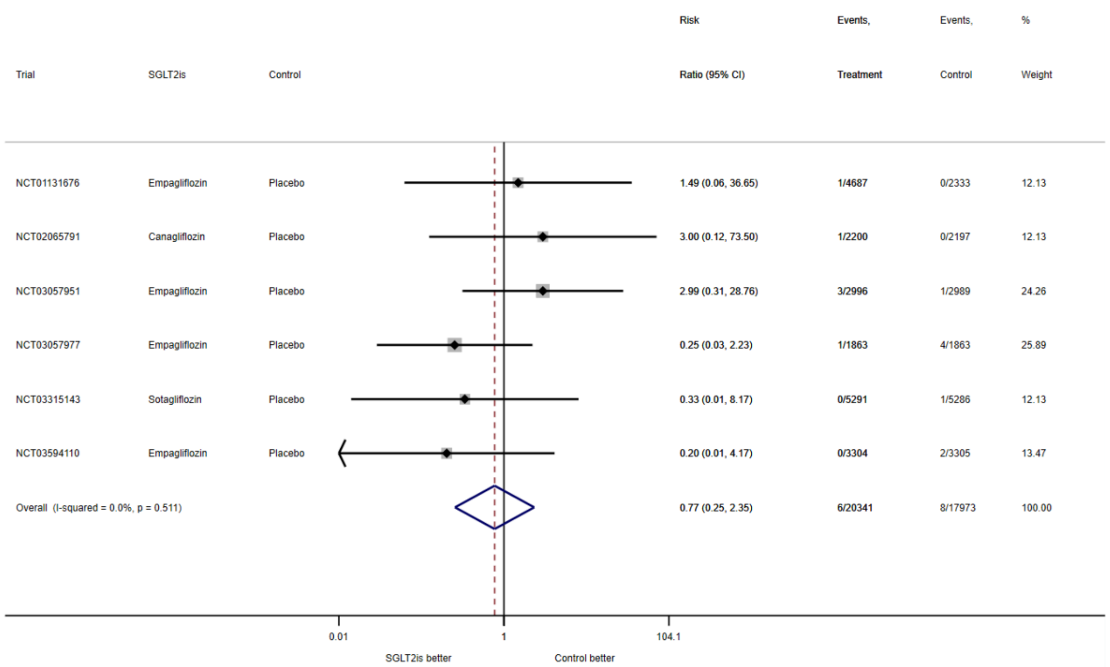

System 9-Hepatobiliary Disorders-Figure S16 Meta-analysis of SGLT2is and Hepatic function abnormal

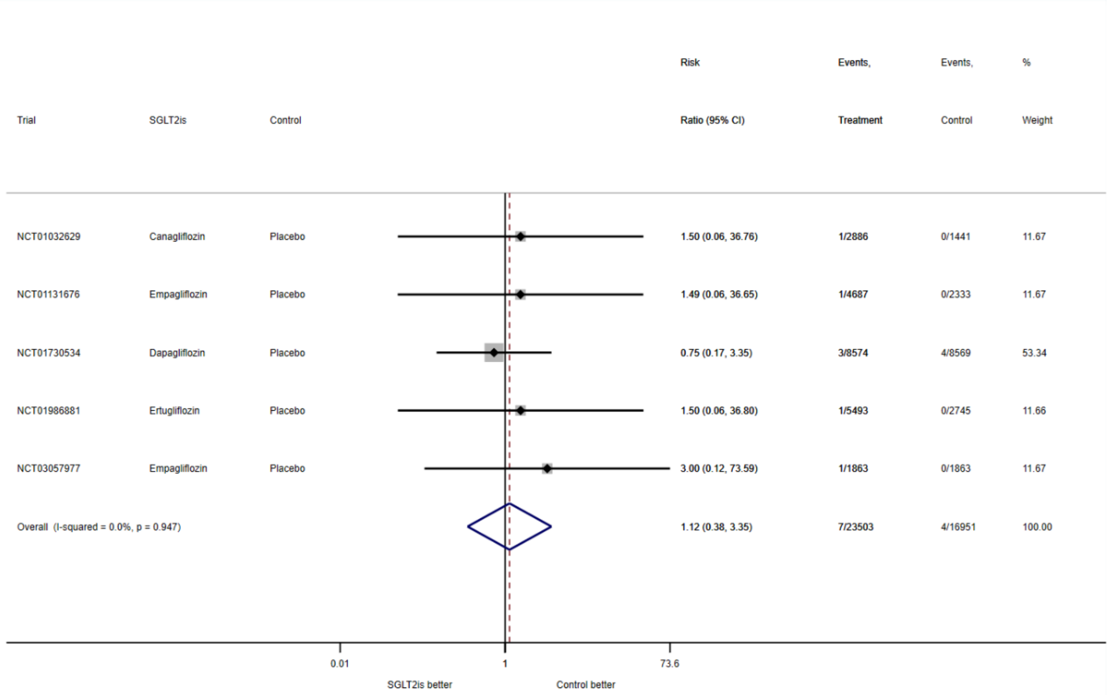

System 9-Hepatobiliary Disorders-Figure S17 Meta-analysis of SGLT2is and Hepatic steatosis

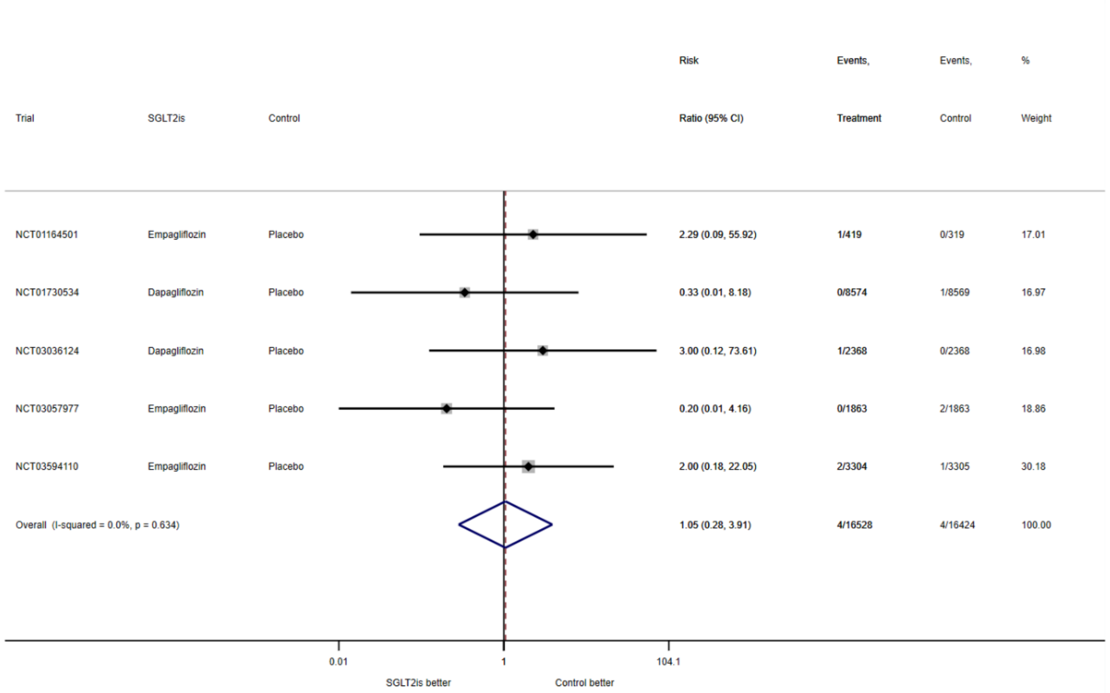

System 9-Hepatobiliary Disorders-Figure S18 Meta-analysis of SGLT2is and Hepatitis

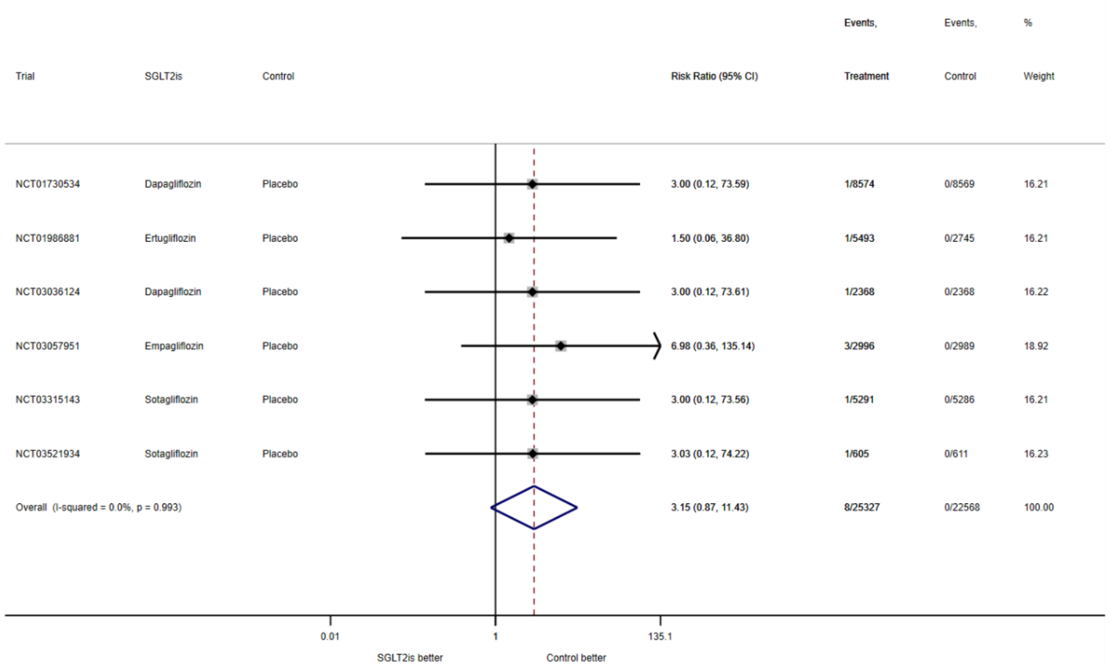

System 9-Hepatobiliary Disorders-Figure S19 Meta-analysis of SGLT2is and Hepatitis acute

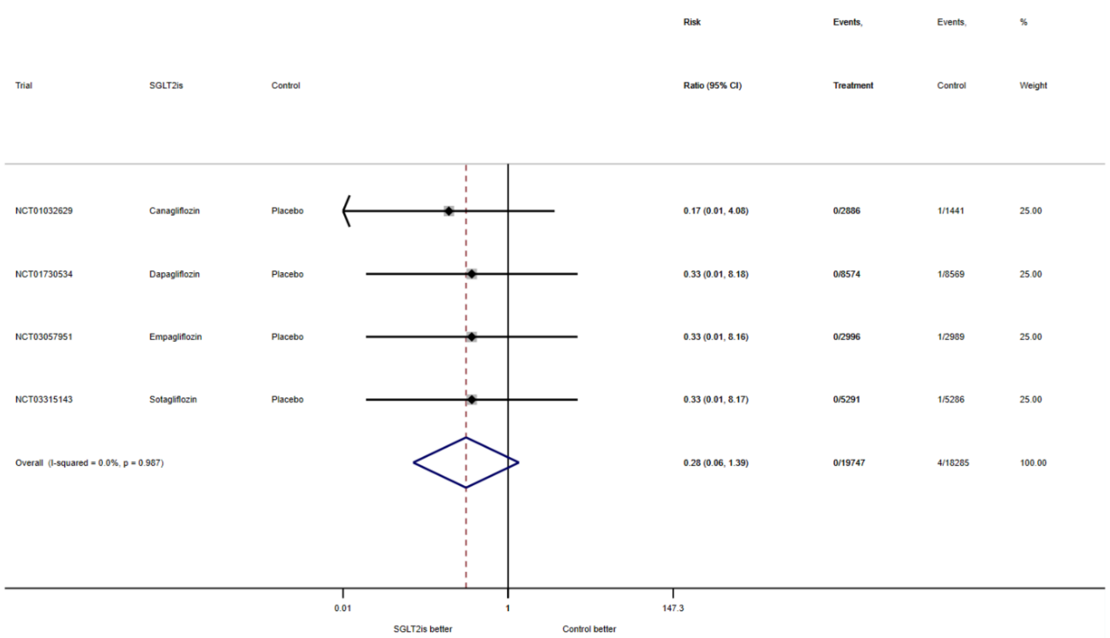

System 9-Hepatobiliary Disorders-Figure S20 Meta-analysis of SGLT2is and Hepatorenal syndrome

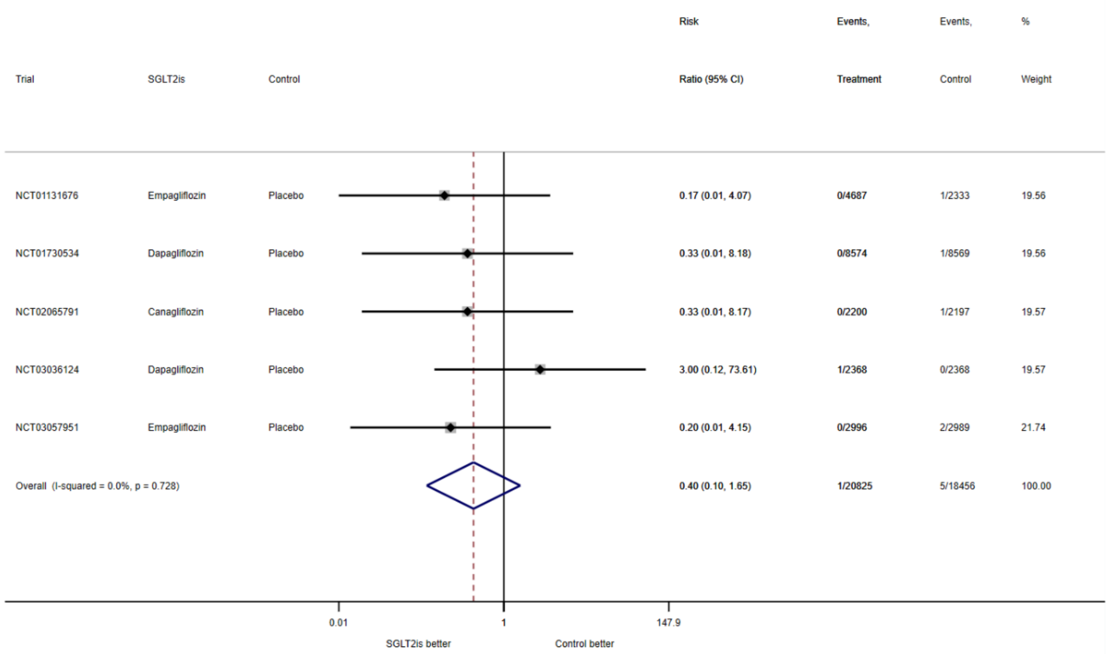

System 9-Hepatobiliary Disorders-Figure S21 Meta-analysis of SGLT2is and Ischaemic hepatitis

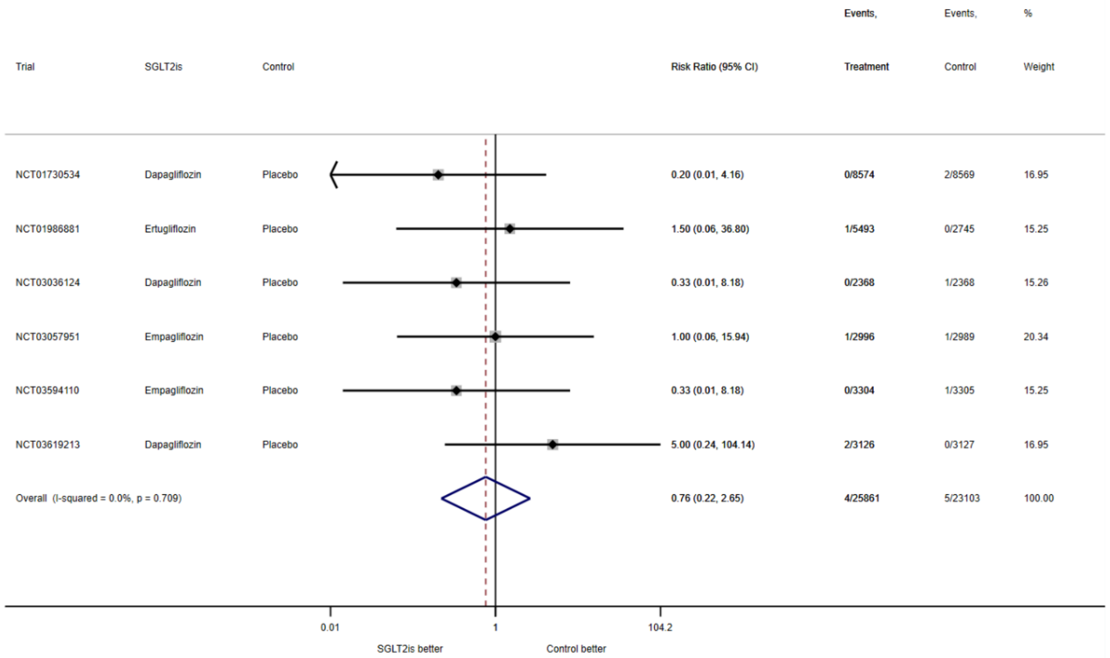

System 9-Hepatobiliary Disorders-Figure S22 Meta-analysis of SGLT2is and Jaundice

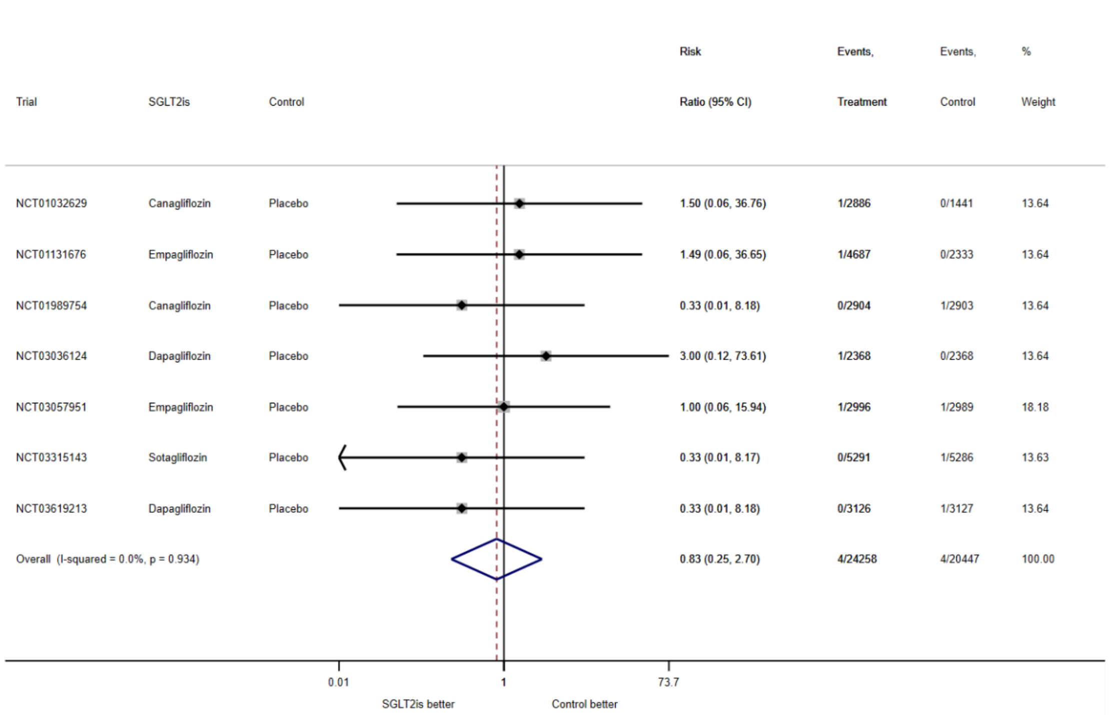

System 9-Hepatobiliary Disorders-Figure S23 Meta-analysis of SGLT2is and Jaundice cholestatic

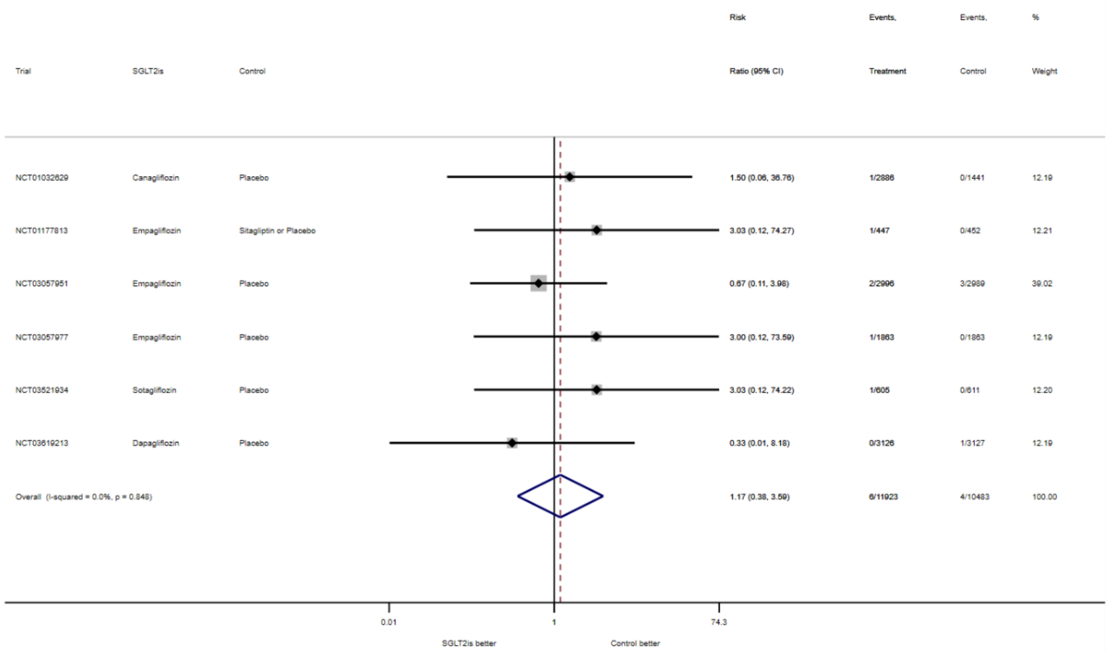

System 9-Hepatobiliary Disorders-Figure S24 Meta-analysis of SGLT2is and Liver disorder

Trial	SGLT2is	Control	Risk Ratio (95% CI)	Events, Treatment	Events, Control	% Weight
NCT01730534	Dapagliflozin	Placebo	5.00 (0.24, 104.07)	2/8574	0/8569	3.58
NCT01986881	Ertugliflozin	Placebo	0.25 (0.02, 2.75)	1/5493	2/2745	5.74
NCT01989754	Canagliflozin	Placebo	3.00 (0.12, 73.59)	1/2904	0/2903	3.23
NCT03036150	Dapagliflozin	Placebo	0.33 (0.01, 8.18)	0/2149	1/2149	3.23
NCT03057951	Empagliflozin	Placebo	0.78 (0.29, 2.08)	7/2996	9/2989	33.96
NCT03057977	Empagliflozin	Placebo	0.60 (0.14, 2.51)	3/1863	5/1863	16.16
NCT03242252	Sotagliflozin	Placebo	1.48 (0.06, 36.28)	1/527	0/260	3.23
NCT03521934	Sotagliflozin	Placebo	0.50 (0.05, 5.55)	1/605	2/611	5.75
NCT03594110	Empagliflozin	Placebo	0.71 (0.23, 2.25)	5/3304	7/3305	25.13
Overall (I-squared = 0.0%, p = 0.887)			0.74 (0.42, 1.31)	21/28415	26/25394	100.00

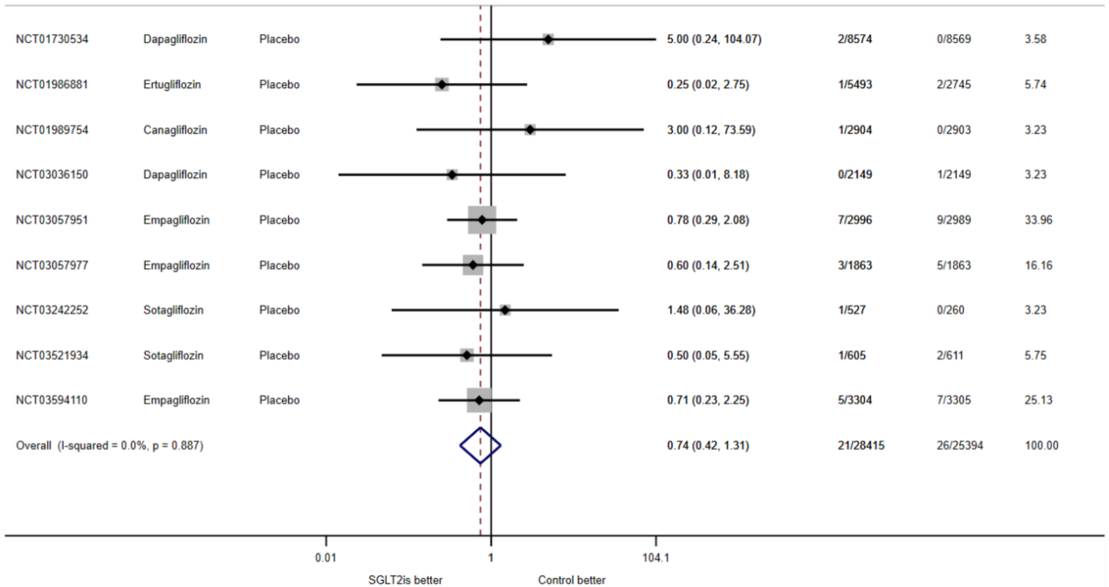

System 9-Hepatobiliary Disorders-Figure S25 Meta-analysis of SGLT2is and Liver injury

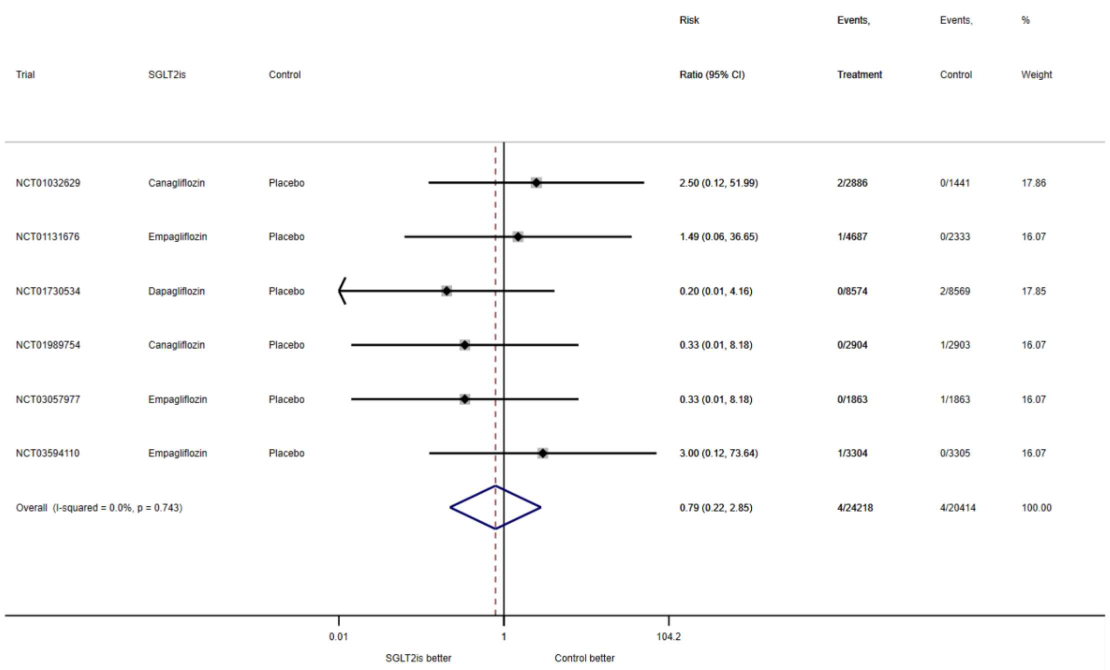

System 9-Hepatobiliary Disorders-Figure S26 Meta-analysis of SGLT2is and Non-alcoholic steatohepatitis

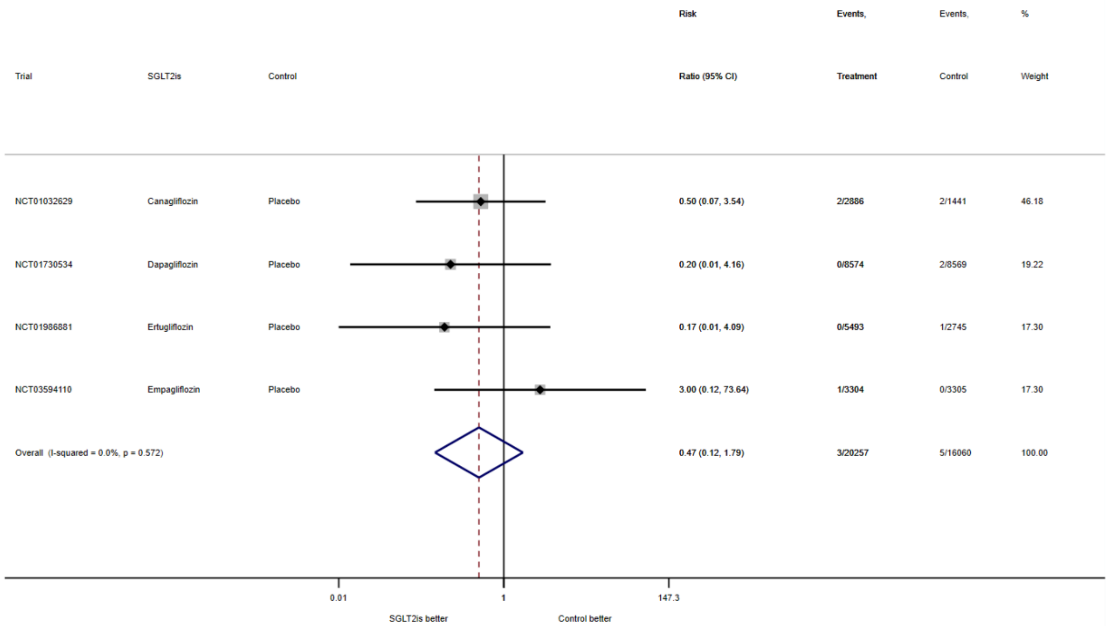

System 9-Hepatobiliary Disorders-Figure S27 Meta-analysis of SGLT2is and Portal vein thrombosis

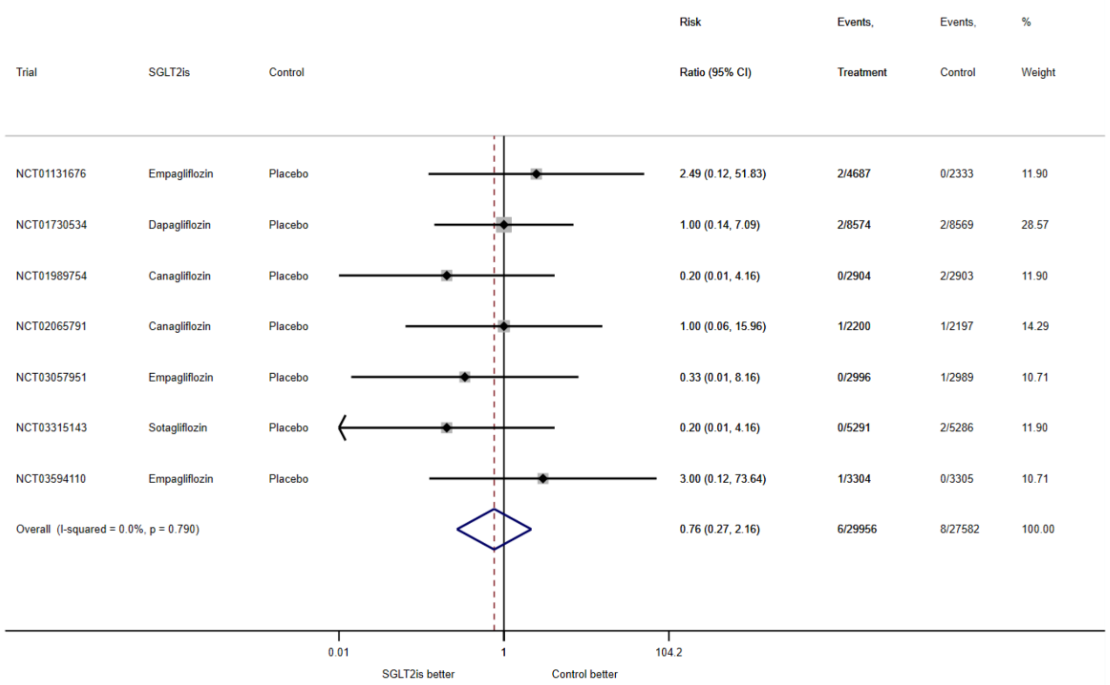

System 10-Immune System Disorders-Figure S1 Meta-analysis of SGLT2is and Anaphylactic reaction

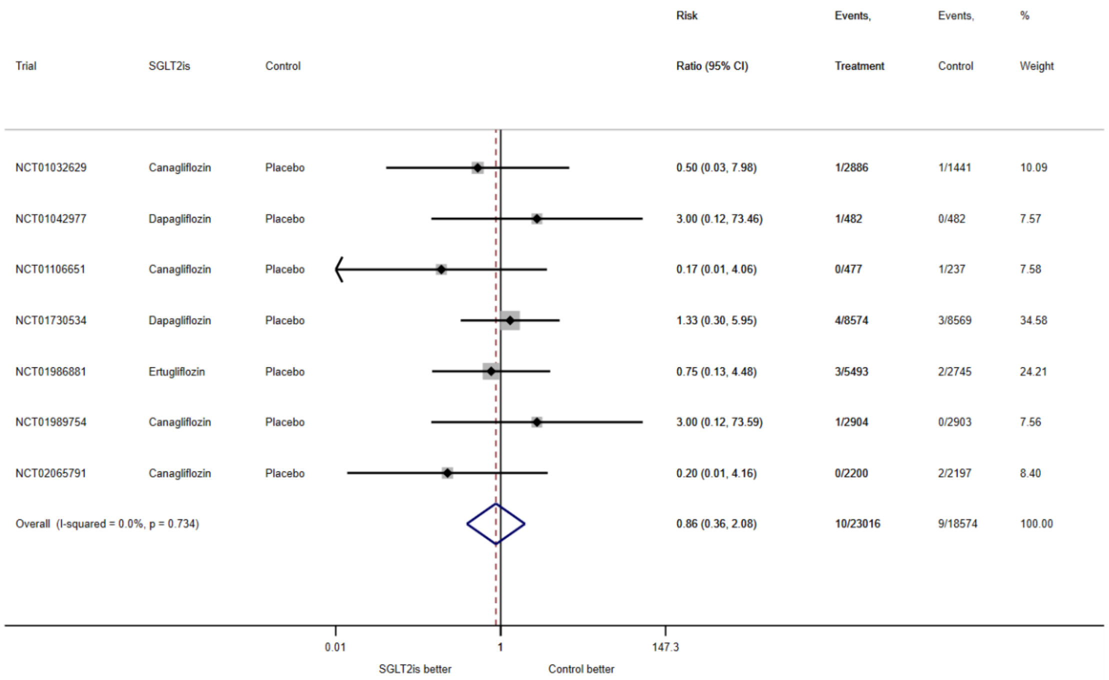

System 10-Immune System Disorders-Figure S2 Meta-analysis of SGLT2is and Drug hypersensitivity

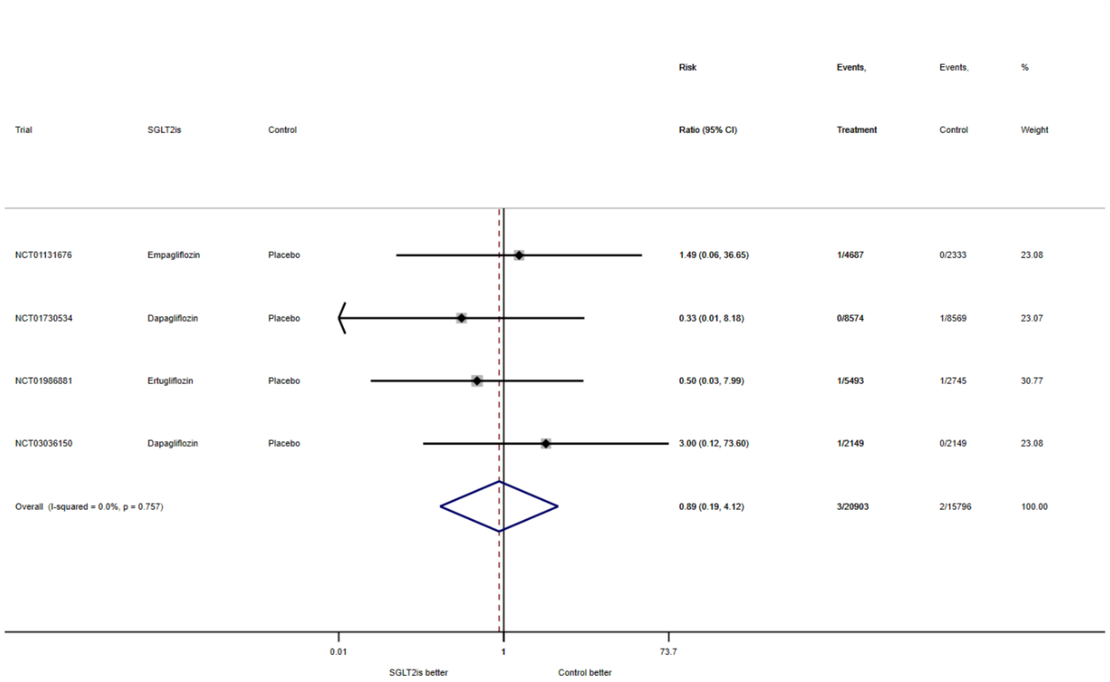

System 10-Immune System Disorders-Figure S3 Meta-analysis of SGLT2is and Hypersensitivity

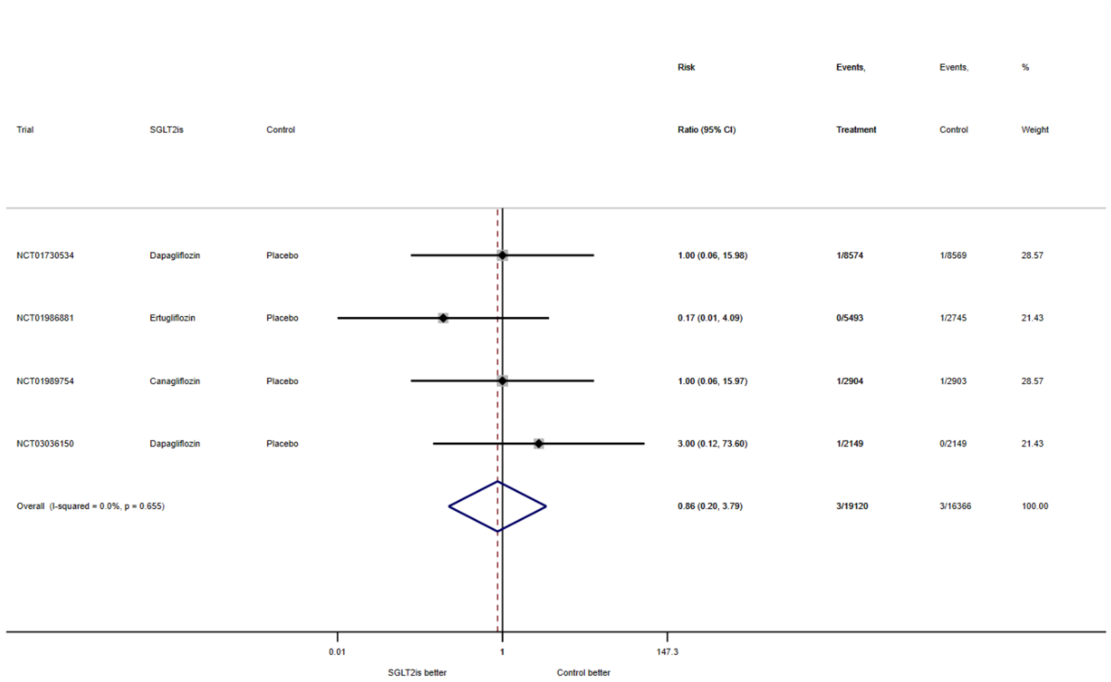

System 10-Immune System Disorders-Figure S4 Meta-analysis of SGLT2is and Sarcoidosis

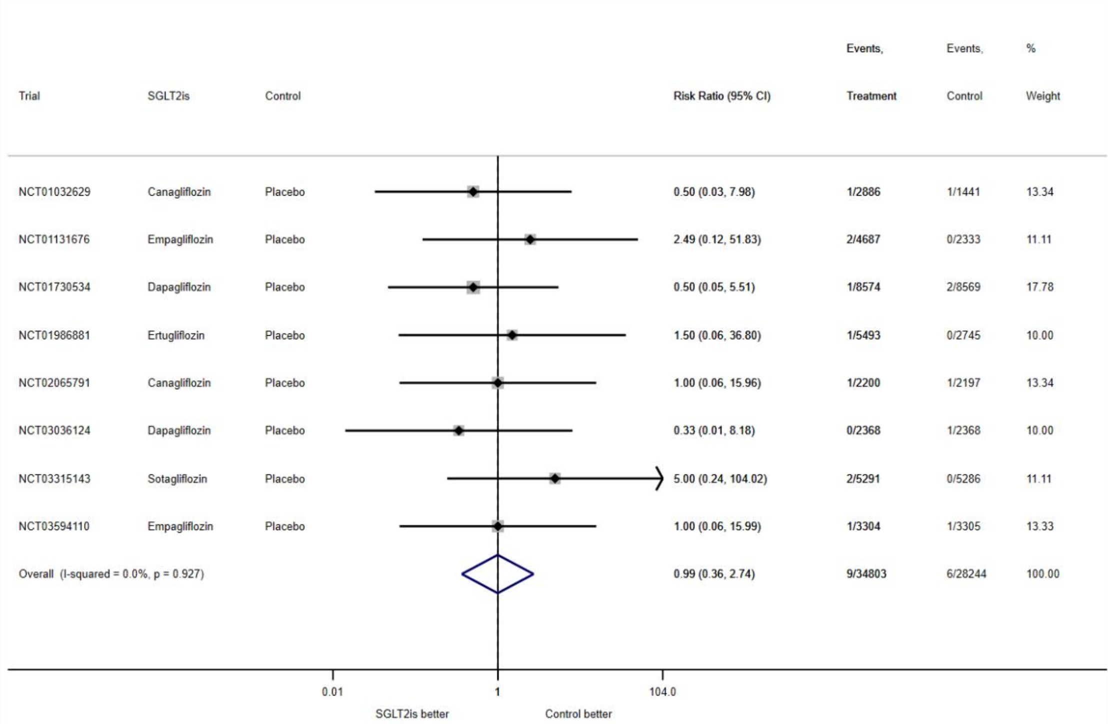

System 11-Infections And Infestations-Figure S1 Meta-analysis of SGLT2is and Abdominal abscess

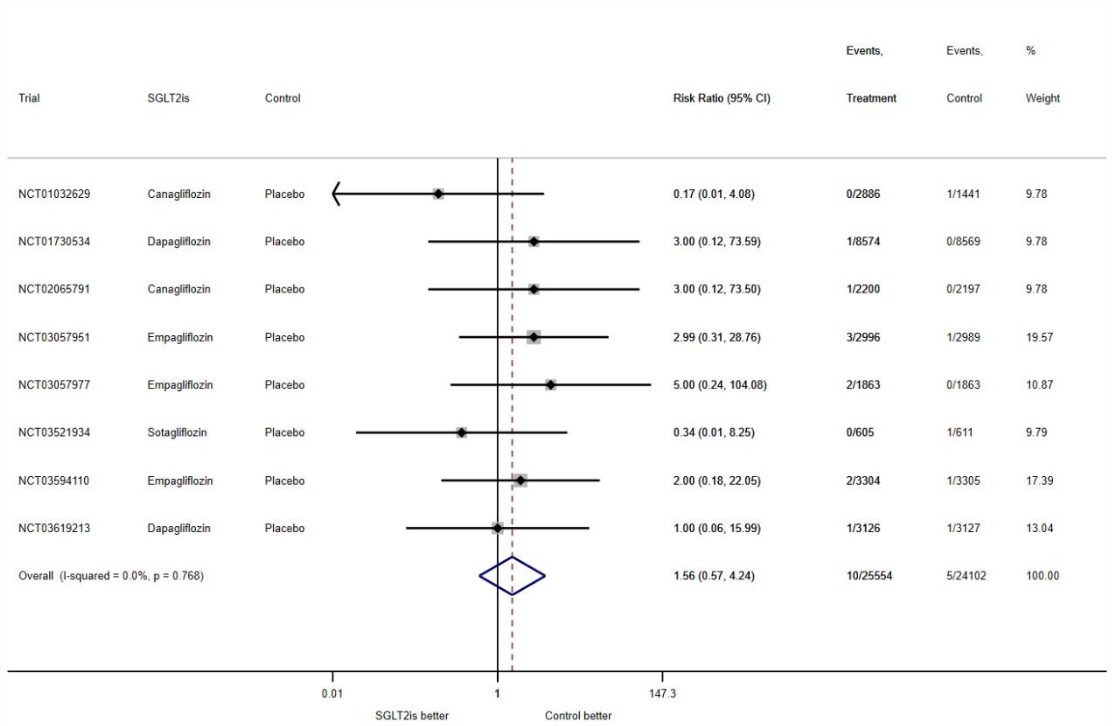

System 11-Infections And Infestations-Figure S2 Meta-analysis of SGLT2is and Abdominal sepsis

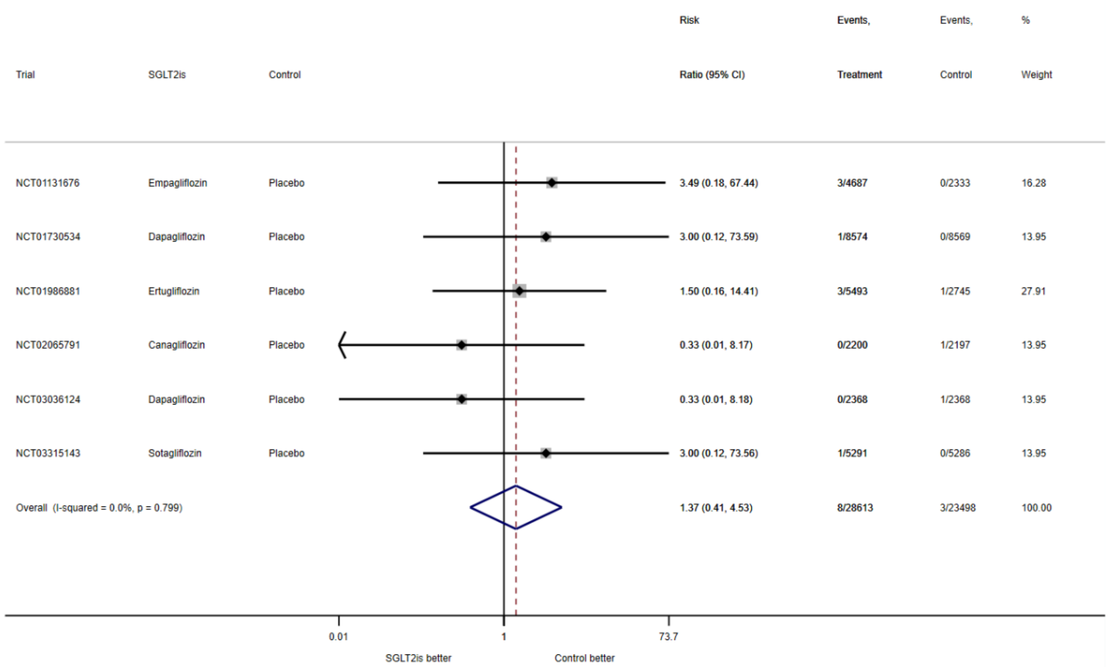

System 11-Infections And Infestations-Figure S3 Meta-analysis of SGLT2is and Abdominal wall abscess

Trial	SGLT2is	Control	Risk Ratio (95% CI)	Events, Treatment	Events, Control	% Weight
NCT01032629	Canagliflozin	Placebo	1.50 (0.06, 36.76)	1/2886	0/1441	8.33
NCT01131676	Empagliflozin	Placebo	1.49 (0.06, 36.65)	1/4687	0/2333	8.33
NCT01730534	Dapagliflozin	Placebo	1.00 (0.06, 15.98)	1/8574	1/8569	11.11
NCT01986881	Ertugliflozin	Placebo	1.50 (0.06, 36.80)	1/5493	0/2745	8.33
NCT01989754	Canagliflozin	Placebo	0.33 (0.01, 8.18)	0/2904	1/2903	8.33
NCT03036124	Dapagliflozin	Placebo	0.33 (0.01, 8.18)	0/2368	1/2368	8.33
NCT03036150	Dapagliflozin	Placebo	3.00 (0.12, 73.60)	1/2149	0/2149	8.33
NCT03057951	Empagliflozin	Placebo	2.99 (0.12, 73.44)	1/2996	0/2989	8.33
NCT03594110	Empagliflozin	Placebo	1.00 (0.14, 7.10)	2/3304	2/3305	22.23
NCT03619213	Dapagliflozin	Placebo	3.00 (0.12, 73.64)	1/3126	0/3127	8.33
Overall (I-squared = 0.0%, p = 0.986)			1.21 (0.48, 3.05)	9/38487	5/31929	100.00

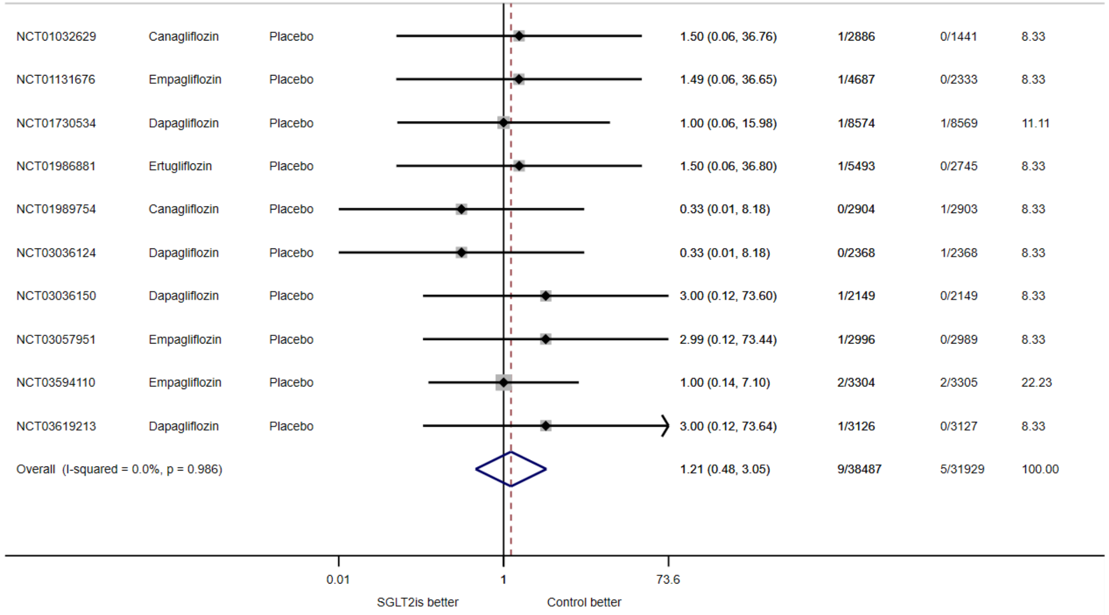

System 11-Infections And Infestations-Figure S4 Meta-analysis of SGLT2is and Abscess

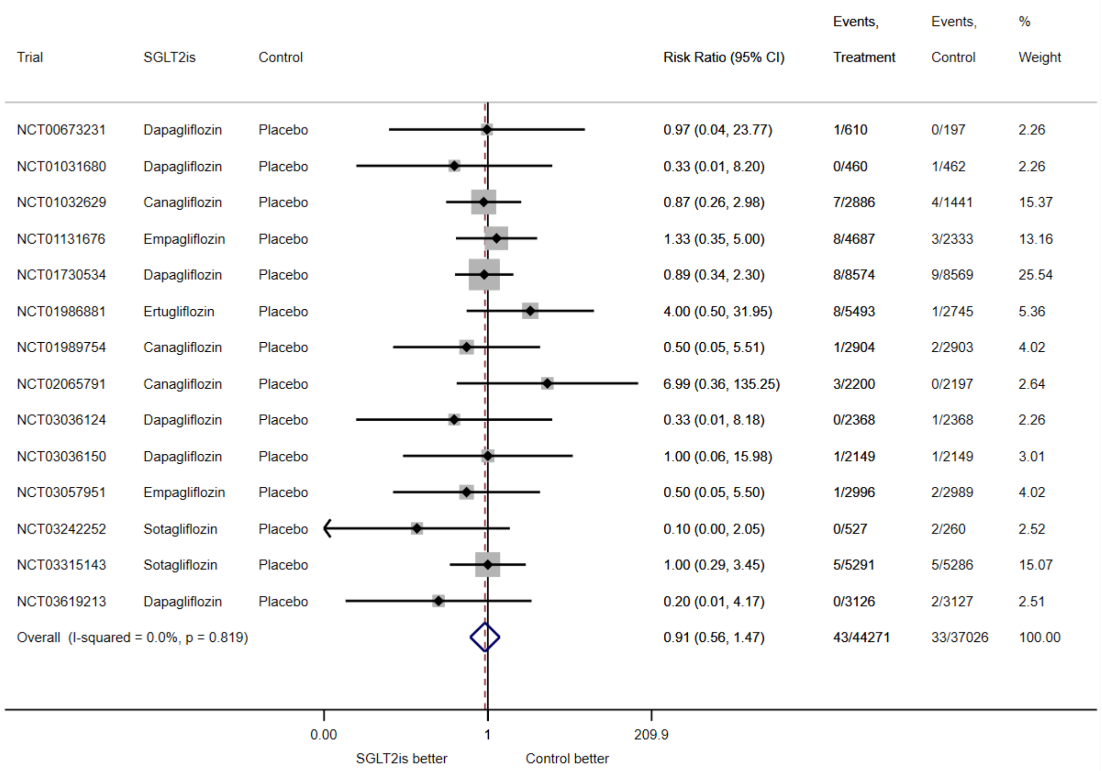

System 11-Infections And Infestations-Figure S5 Meta-analysis of SGLT2is and Abscess limb

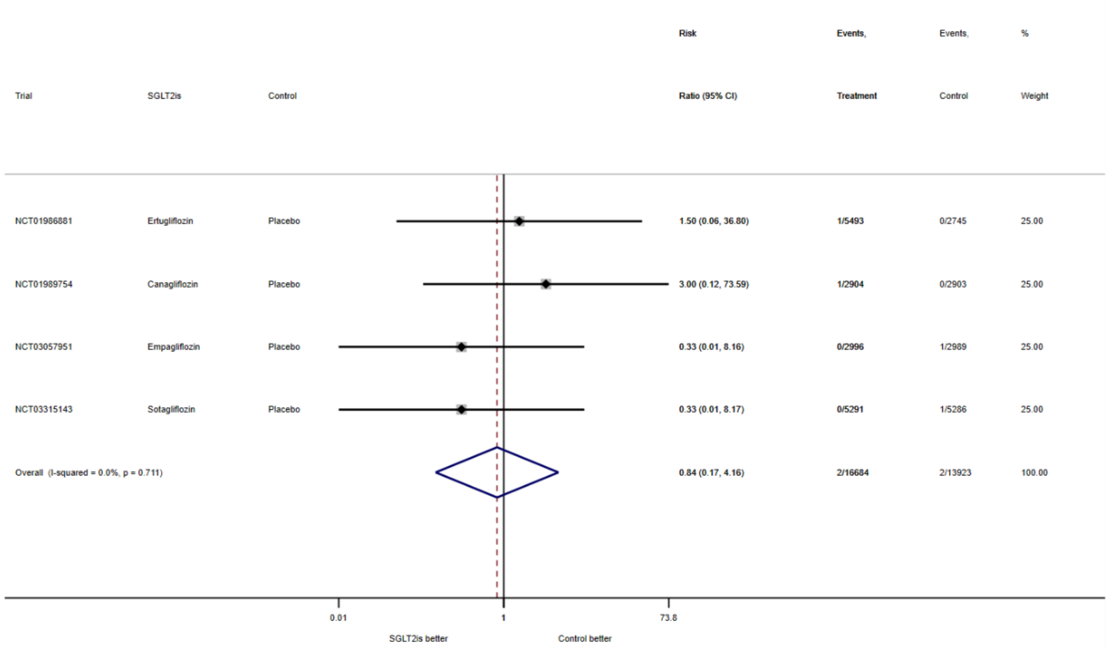

System 11-Infections And Infestations-Figure S6 Meta-analysis of SGLT2is and Abscess neck

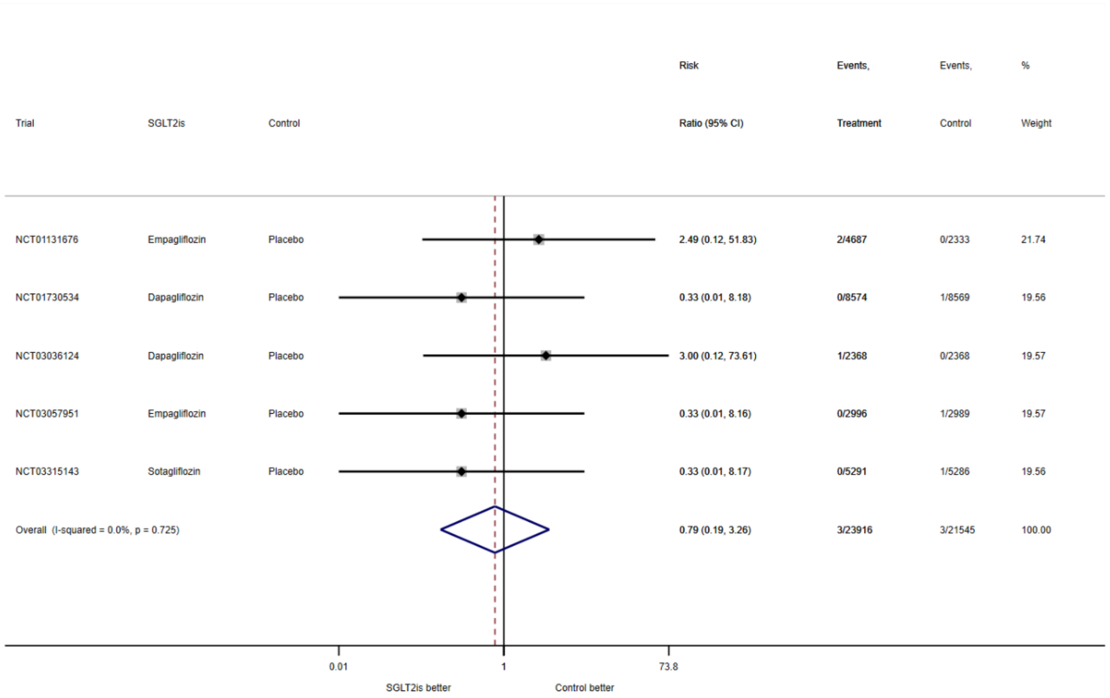

System 11-Infections And Infestations-Figure S7 Meta-analysis of SGLT2is and Acute sinusitis

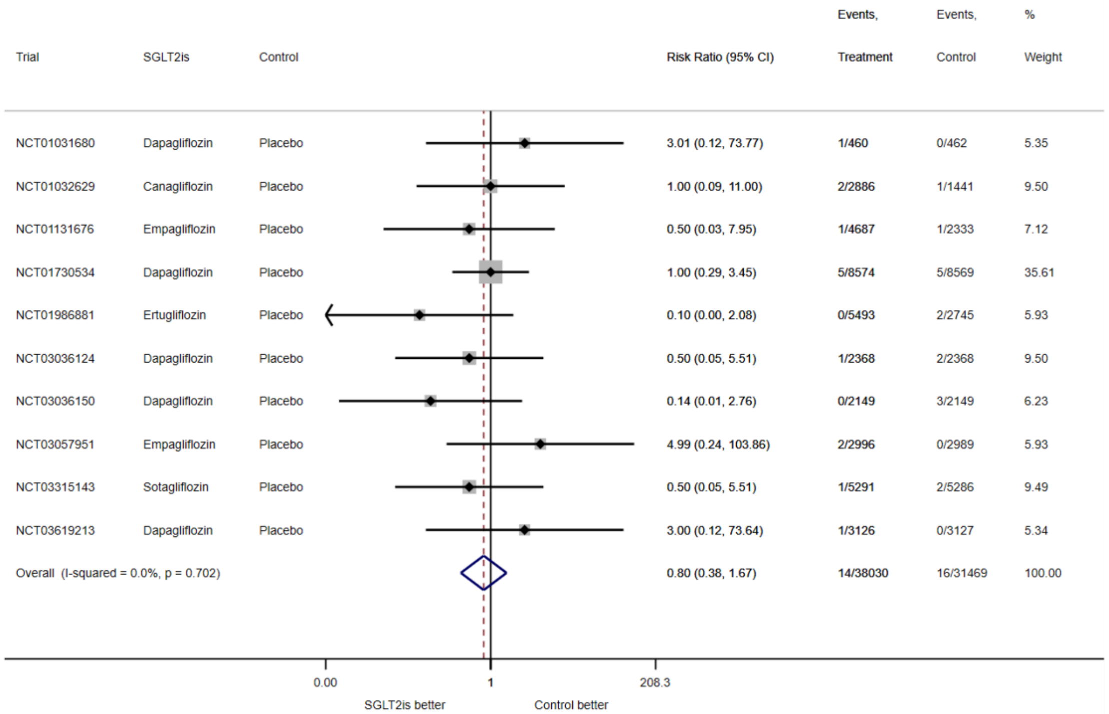

System 11-Infections And Infestations-Figure S8 Meta-analysis of SGLT2is and Anal abscess

Trial	SGLT2is	Control	Risk Ratio (95% CI)	Events, Treatment	Events, Control	% Weight
NCT00528879	Dapagliflozin	Placebo	1.01 (0.04, 24.64)	1/409	0/137	1.48
NCT01032629	Canagliflozin	Placebo	2.25 (0.49, 10.39)	9/2886	2/1441	6.45
NCT01131676	Empagliflozin	Placebo	1.33 (0.35, 5.00)	8/4687	3/2333	8.60
NCT01730534	Dapagliflozin	Placebo	1.00 (0.45, 2.22)	12/8574	12/8569	23.65
NCT01986881	Ertugliflozin	Placebo	0.67 (0.15, 2.97)	4/5493	3/2745	6.75
NCT01989754	Canagliflozin	Placebo	3.00 (0.61, 14.85)	6/2904	2/2903	5.91
NCT02065791	Canagliflozin	Placebo	0.67 (0.11, 3.98)	2/2200	3/2197	4.73
NCT02384941	Sotagliflozin	Placebo	2.56 (0.12, 53.07)	2/525	0/268	1.64
NCT03036124	Dapagliflozin	Placebo	0.50 (0.05, 5.51)	1/2368	2/2368	2.63
NCT03036150	Dapagliflozin	Placebo	1.00 (0.20, 4.95)	3/2149	3/2149	5.91
NCT03057951	Empagliflozin	Placebo	0.80 (0.21, 2.97)	4/2996	5/2989	8.76
NCT03057977	Empagliflozin	Placebo	1.00 (0.20, 4.95)	3/1863	3/1863	5.91
NCT03242252	Sotagliflozin	Placebo	2.47 (0.12, 51.30)	2/527	0/260	1.64
NCT03315143	Sotagliflozin	Placebo	1.33 (0.30, 5.95)	4/5291	3/5286	6.75
NCT03594110	Empagliflozin	Placebo	5.00 (0.58, 42.79)	5/3304	1/3305	3.28
NCT03619213	Dapagliflozin	Placebo	0.33 (0.07, 1.65)	2/3126	6/3127	5.91
Overall (I-squared = 0.0%, p = 0.899)			1.10 (0.75, 1.62)	68/49302	48/41940	100.00

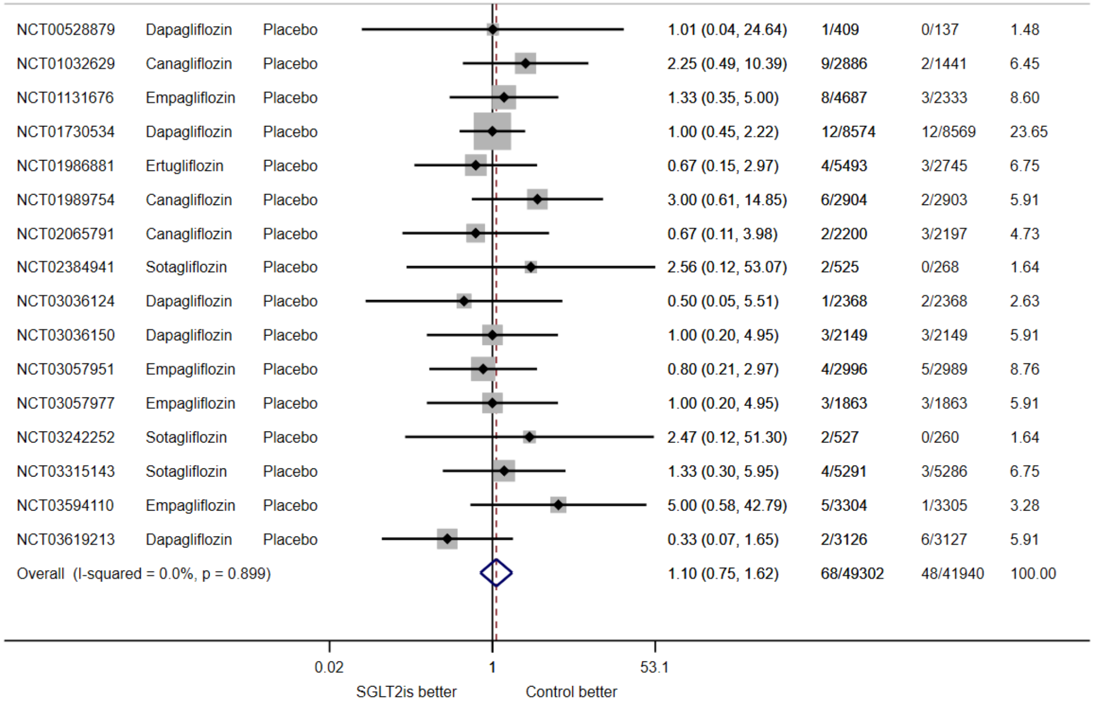

System 11-Infections And Infestations-Figure S9 Meta-analysis of SGLT2is and Appendicitis

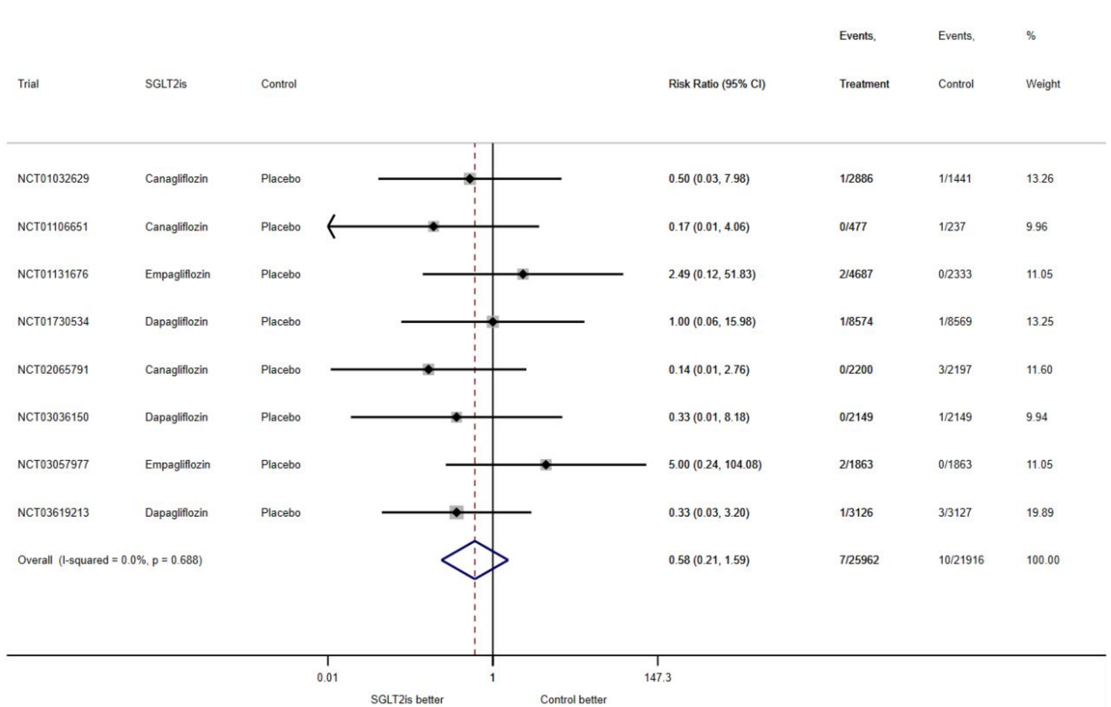

System 11-Infections And Infestations-Figure S10 Meta-analysis of SGLT2is and Appendicitis perforated

Trial	SGLT2is	Control	Risk Ratio (95% CI)	Events, Treatment	Events, Control	% Weight
NCT01032629	Canagliflozin	Placebo	0.17 (0.01, 4.08)	0/2886	1/1441	4.56
NCT01131676	Empagliflozin	Placebo	1.49 (0.16, 14.35)	3/4687	1/2333	9.12
NCT01730534	Dapagliflozin	Placebo	0.55 (0.20, 1.47)	6/8574	11/8569	47.22
NCT01989754	Canagliflozin	Placebo	1.00 (0.06, 15.97)	1/2904	1/2903	6.08
NCT02065791	Canagliflozin	Placebo	2.00 (0.18, 22.01)	2/2200	1/2197	8.11
NCT03036150	Dapagliflozin	Placebo	0.33 (0.01, 8.18)	0/2149	1/2149	4.56
NCT03057951	Empagliflozin	Placebo	2.99 (0.12, 73.44)	1/2996	0/2989	4.56
NCT03315143	Sotagliflozin	Placebo	0.25 (0.03, 2.23)	1/5291	4/5286	9.72
NCT03619213	Dapagliflozin	Placebo	1.00 (0.06, 15.99)	1/3126	1/3127	6.08
Overall (I-squared = 0.0%, p = 0.845)			0.66 (0.33, 1.31)	15/34813	21/30994	100.00

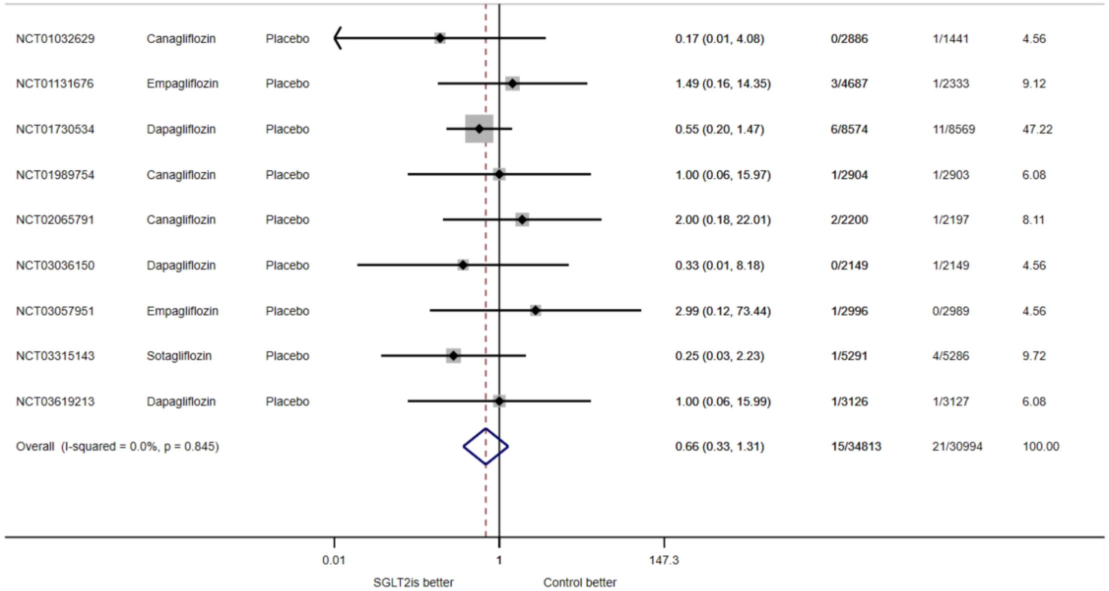

System 11-Infections And Infestations-Figure S11 Meta-analysis of SGLT2is and Arthritis bacterial

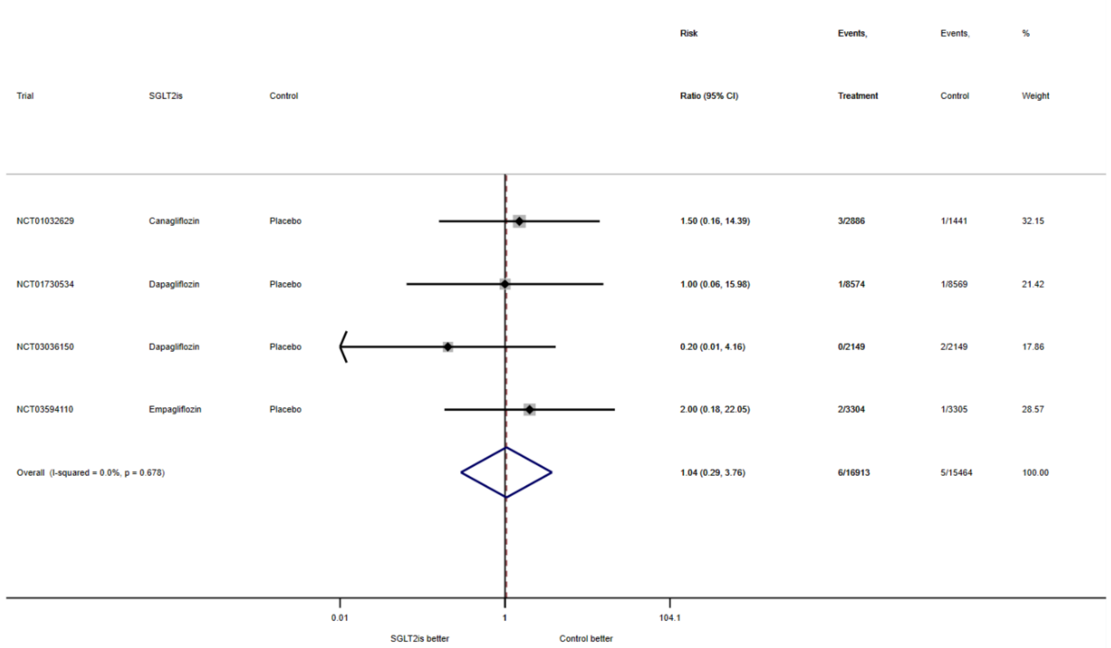

System 11-Infections And Infestations-Figure S12 Meta-analysis of SGLT2is and Arthritis infective

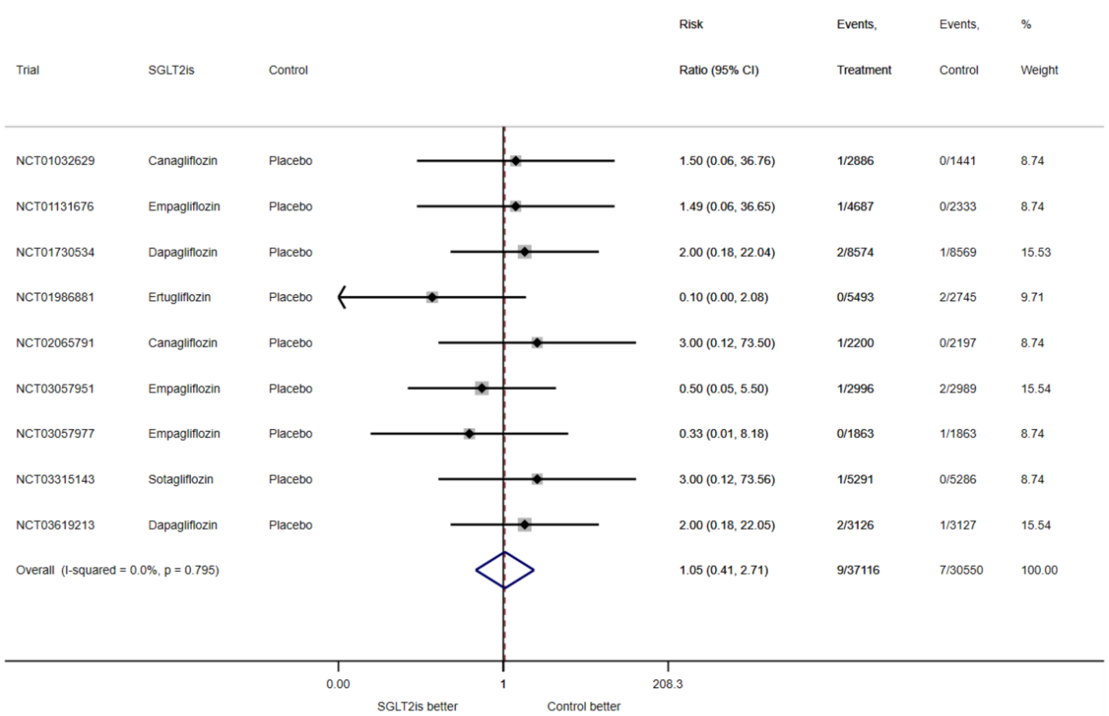

System 11-Infections And Infestations-Figure S13 Meta-analysis of SGLT2is and Atypical pneumonia

Trial	SGLT2is	Control	Risk Ratio (95% CI)	Events, Treatment	Events, Control	% Weight
NCT00528879	Dapagliflozin	Placebo	1.01 (0.04, 24.64)	1/409	0/137	4.71
NCT01032629	Canagliflozin	Placebo	0.50 (0.03, 7.98)	1/2886	1/1441	6.26
NCT01131676	Empagliflozin	Placebo	0.75 (0.12, 4.47)	3/4687	2/2333	15.02
NCT01730534	Dapagliflozin	Placebo	2.00 (0.50, 7.99)	6/8574	3/8569	25.02
NCT01986881	Ertugliflozin	Placebo	0.07 (0.00, 1.38)	0/5493	3/2745	5.47
NCT02065791	Canagliflozin	Placebo	1.00 (0.06, 15.96)	1/2200	1/2197	6.26
NCT03036124	Dapagliflozin	Placebo	3.00 (0.12, 73.61)	1/2368	0/2368	4.69
NCT03036150	Dapagliflozin	Placebo	7.00 (0.36, 135.44)	3/2149	0/2149	5.47
NCT03057951	Empagliflozin	Placebo	0.33 (0.01, 8.16)	0/2996	1/2989	4.69
NCT03057977	Empagliflozin	Placebo	3.00 (0.12, 73.59)	1/1863	0/1863	4.69
NCT03315143	Sotagliflozin	Placebo	0.33 (0.03, 3.20)	1/5291	3/5286	9.38
NCT03619213	Dapagliflozin	Placebo	2.00 (0.18, 22.05)	2/3126	1/3127	8.34
Overall (I-squared = 0.0%, p = 0.661)			1.06 (0.53, 2.11)	20/42042	15/35204	100.00

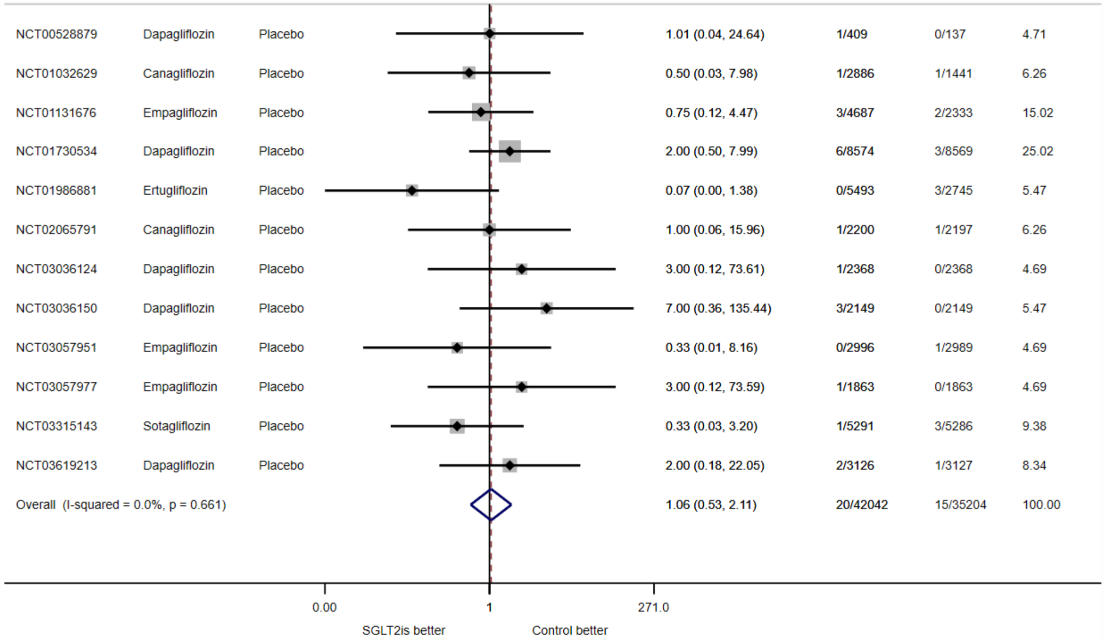

System 11-Infections And Infestations-Figure S14 Meta-analysis of SGLT2is and Bacteraemia

Trial	SGLT2is	Control	Risk Ratio (95% CI)	Events, Treatment	Events, Control	% Weight
NCT01032629	Canagliflozin	Placebo	0.17 (0.01, 4.08)	0/2886	1/1441	6.43
NCT01131676	Empagliflozin	Placebo	1.49 (0.06, 36.65)	1/4687	0/2333	6.43
NCT01730534	Dapagliflozin	Placebo	1.00 (0.06, 15.98)	1/8574	1/8569	8.57
NCT01986881	Ertugliflozin	Placebo	0.50 (0.03, 7.99)	1/5493	1/2745	8.57
NCT02065791	Canagliflozin	Placebo	0.33 (0.01, 8.17)	0/2200	1/2197	6.43
NCT03036124	Dapagliflozin	Placebo	0.20 (0.01, 4.16)	0/2368	2/2368	7.14
NCT03036150	Dapagliflozin	Placebo	0.20 (0.01, 4.16)	0/2149	2/2149	7.14
NCT03057951	Empagliflozin	Placebo	0.33 (0.03, 3.20)	1/2996	3/2989	12.86
NCT03057977	Empagliflozin	Placebo	5.00 (0.24, 104.08)	2/1863	0/1863	7.14
NCT03315143	Sotagliflozin	Placebo	2.00 (0.37, 10.90)	4/5291	2/5286	22.86
NCT03594110	Empagliflozin	Placebo	3.00 (0.12, 73.64)	1/3304	0/3305	6.43
Overall (I-squared = 0.0%, p = 0.740)			0.78 (0.35, 1.76)	11/41811	13/35245	100.00

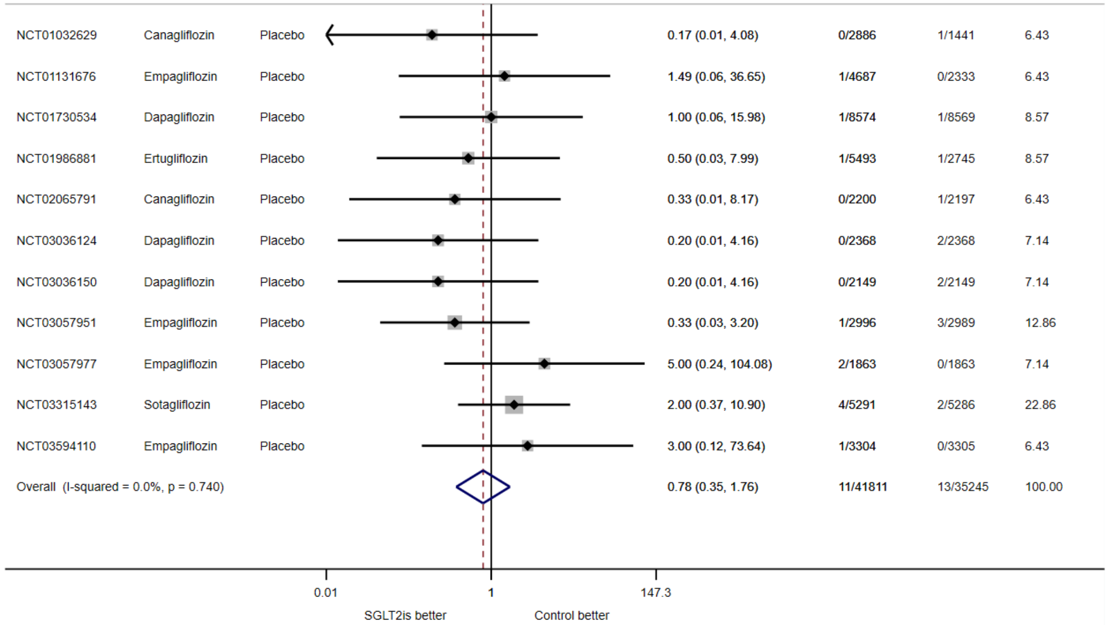

System 11-Infections And Infestations-Figure S15 Meta-analysis of SGLT2is and Bacterial sepsis

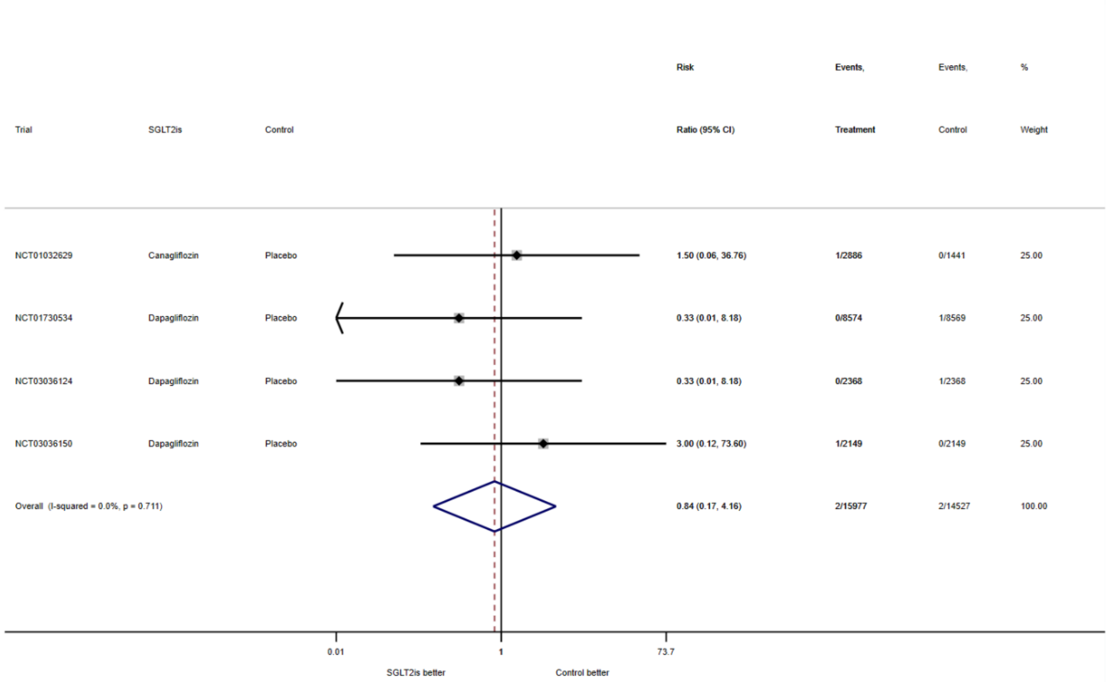

System 11-Infections And Infestations-Figure S16 Meta-analysis of SGLT2is and Biliary sepsis

Trial	SGLT2is	Control	Risk Ratio (95% CI)	Events, Treatment	Events, Control	% Weight
NCT01032629	Canagliflozin	Placebo	1.00 (0.30, 3.31)	8/2886	4/1441	5.60
NCT01042977	Dapagliflozin	Placebo	3.01 (0.12, 73.61)	1/482	0/483	0.79
NCT01131676	Empagliflozin	Placebo	0.50 (0.21, 1.19)	10/4687	10/2333	10.51
NCT01730534	Dapagliflozin	Placebo	0.43 (0.23, 0.78)	15/8574	35/8569	22.05
NCT01986881	Ertugliflozin	Placebo	0.50 (0.22, 1.15)	11/5493	11/2745	11.56
NCT01989754	Canagliflozin	Placebo	0.14 (0.01, 2.76)	0/2904	3/2903	0.92
NCT02065791	Canagliflozin	Placebo	0.71 (0.23, 2.24)	5/2200	7/2197	6.13
NCT03036124	Dapagliflozin	Placebo	2.00 (0.75, 5.32)	12/2368	6/2368	8.41
NCT03036150	Dapagliflozin	Placebo	0.83 (0.25, 2.73)	5/2149	6/2149	5.73
NCT03057951	Empagliflozin	Placebo	0.33 (0.11, 1.03)	4/2996	12/2989	6.30
NCT03057977	Empagliflozin	Placebo	1.00 (0.29, 3.45)	5/1863	5/1863	5.25
NCT03242252	Sotagliflozin	Placebo	0.16 (0.01, 4.03)	0/527	1/260	0.79
NCT03315143	Sotagliflozin	Placebo	0.07 (0.00, 1.17)	0/5291	7/5286	0.98
NCT03521934	Sotagliflozin	Placebo	1.01 (0.14, 7.15)	2/605	2/611	2.10
NCT03594110	Empagliflozin	Placebo	0.50 (0.09, 2.73)	2/3304	4/3305	2.80
NCT03619213	Dapagliflozin	Placebo	0.67 (0.27, 1.63)	8/3126	12/3127	10.09
Overall (I-squared = 1.8%, p = 0.432)			0.61 (0.46, 0.81)	88/49455	125/42629	100.00

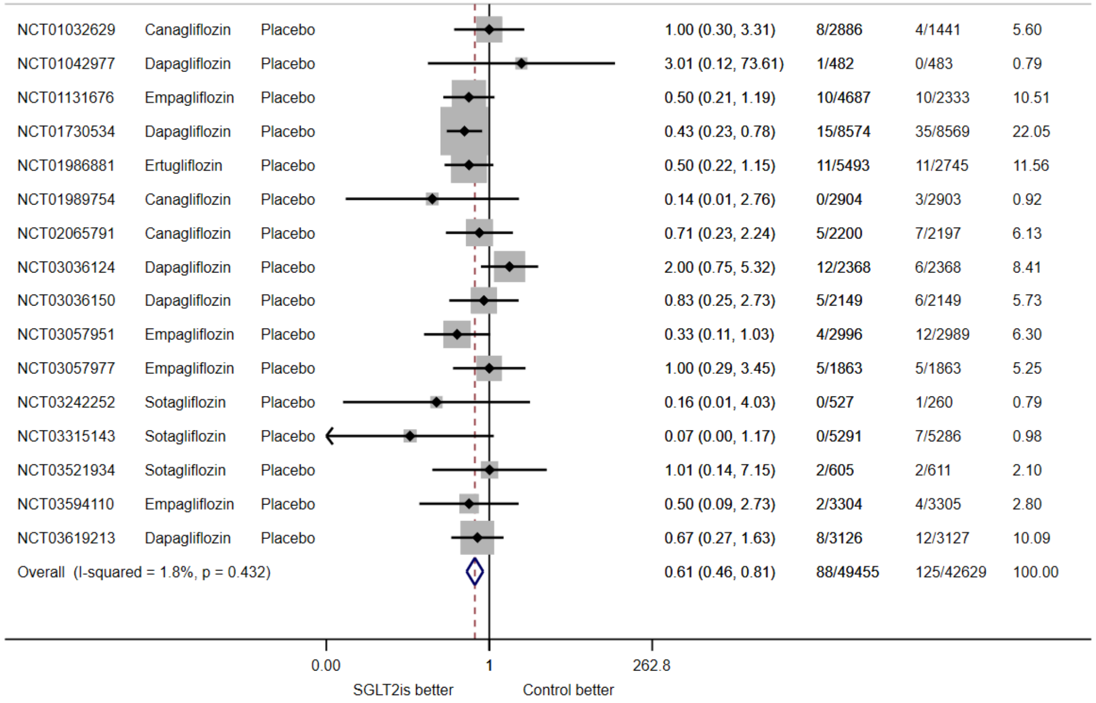

System 11-Infections And Infestations-Figure S17 Meta-analysis of SGLT2is and Bronchitis

Trial	SGLT2is	Control	Risk Ratio (95% CI)	Events, Treatment	Events, Control	% Weight
NCT01032629	Canagliflozin	Placebo	0.17 (0.01, 4.08)	0/2886	1/1441	10.08
NCT01730534	Dapagliflozin	Placebo	5.00 (0.24, 104.07)	2/8574	0/8569	11.20
NCT01986881	Ertugliflozin	Placebo	1.50 (0.06, 36.80)	1/5493	0/2745	10.08
NCT03036150	Dapagliflozin	Placebo	0.33 (0.01, 8.18)	0/2149	1/2149	10.08
NCT03057951	Empagliflozin	Placebo	0.33 (0.01, 8.16)	0/2996	1/2989	10.08
NCT03315143	Sotagliflozin	Placebo	0.40 (0.08, 2.06)	2/5291	5/5286	38.41
NCT03594110	Empagliflozin	Placebo	3.00 (0.12, 73.64)	1/3304	0/3305	10.08
Overall (I-squared = 0.0%, p = 0.644)			0.66 (0.24, 1.81)	6/30693	8/26484	100.00

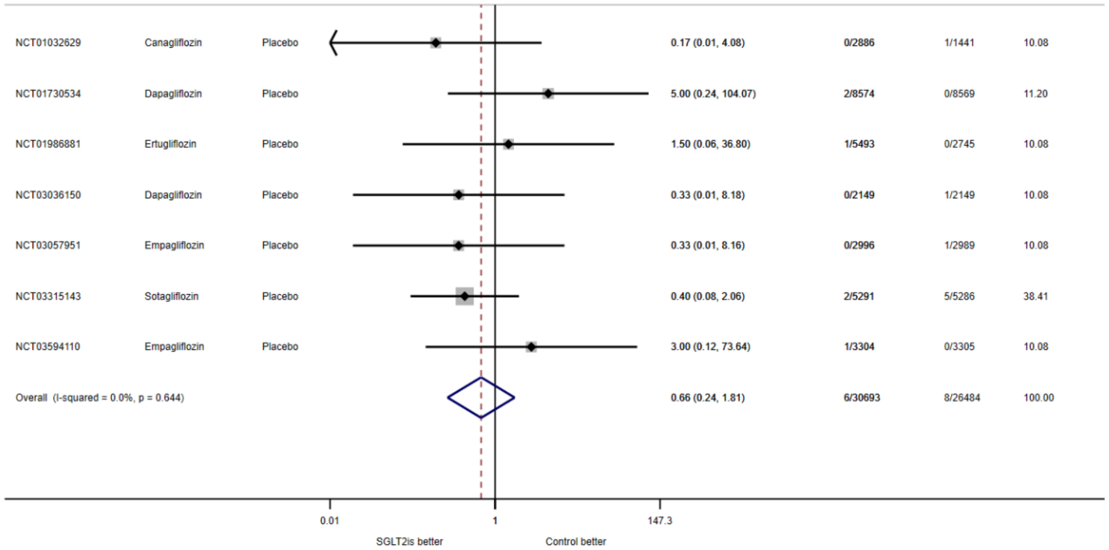

System 11-Infections And Infestations-Figure S18 Meta-analysis of SGLT2is and Bronchitis bacterial

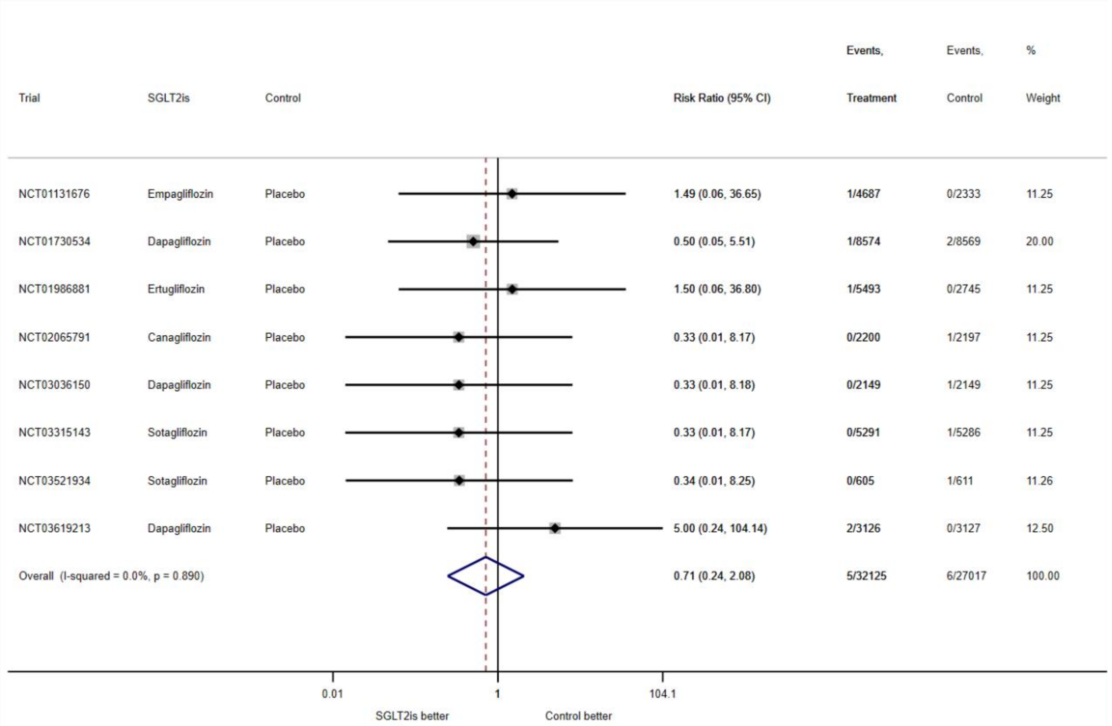

System 11-Infections And Infestations-Figure S19 Meta-analysis of SGLT2is and Bronchitis viral

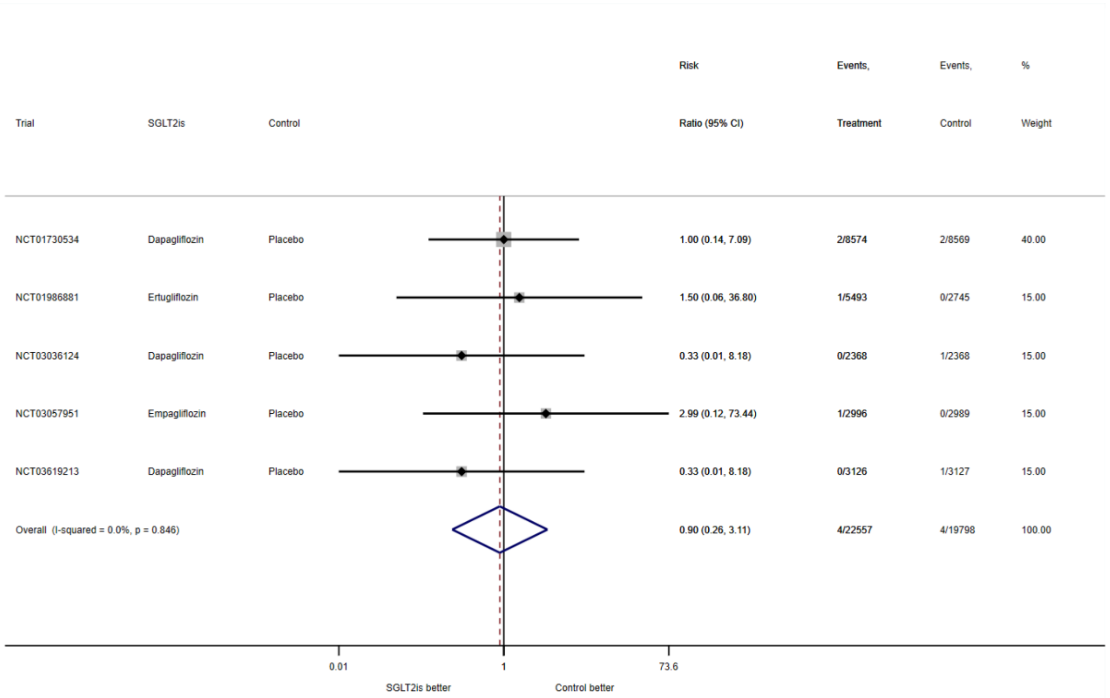

System 11-Infections And Infestations-Figure S20 Meta-analysis of SGLT2is and Bursitis infective

Trial	SGLT2is	Control	Risk Ratio (95% CI)	Events, Treatment	Events, Control	% Weight
NCT01032629	Canagliflozin	Placebo	2.50 (0.12, 51.99)	2/2886	0/1441	18.18
NCT01164501	Empagliflozin	Placebo	0.25 (0.01, 6.21)	0/419	1/319	16.39
NCT01730534	Dapagliflozin	Placebo	3.00 (0.12, 73.59)	1/8574	0/8569	16.36
NCT01986881	Ertugliflozin	Placebo	0.17 (0.01, 4.09)	0/5493	1/2745	16.36
NCT01989754	Canagliflozin	Placebo	0.33 (0.01, 8.18)	0/2904	1/2903	16.36
NCT03594110	Empagliflozin	Placebo	3.00 (0.12, 73.64)	1/3304	0/3305	16.36
Overall (I-squared = 0.0%, p = 0.616)			0.84 (0.23, 3.07)	4/23580	3/19282	100.00

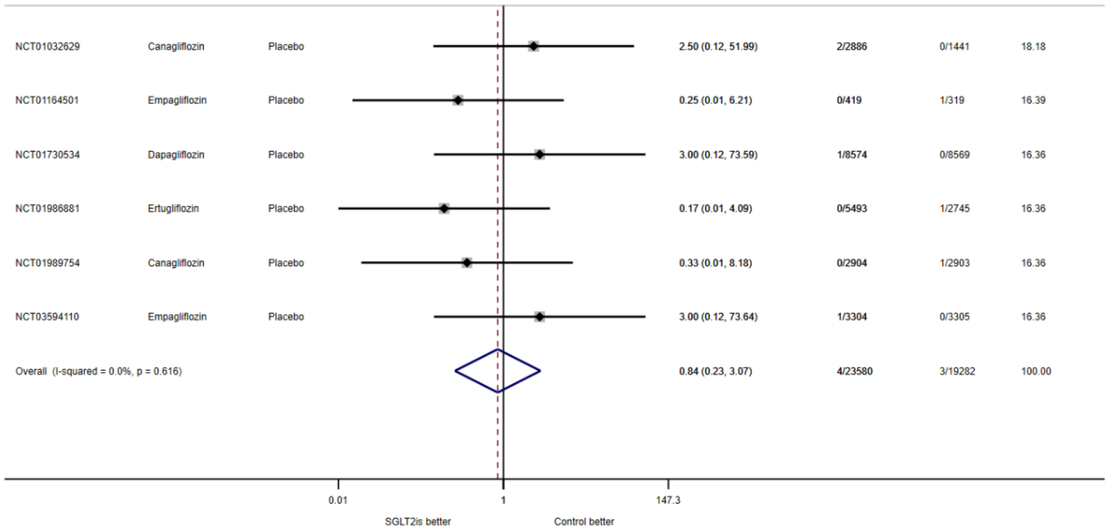

System 11-Infections And Infestations-Figure S21 Meta-analysis of SGLT2is and Campylobacter gastroenteritis

Trial	SGLT2is	Control	Risk Ratio (95% CI)	Events, Treatment	Events, Control	% Weight
NCT01032629	Canagliflozin	Placebo	0.79 (0.45, 1.40)	30/2886	19/1441	7.83
NCT01106677	Canagliflozin	Sitagliptin	0.17 (0.01, 4.07)	0/735	1/366	0.25
NCT01131676	Empagliflozin	Placebo	0.70 (0.39, 1.23)	28/4687	20/2333	7.81
NCT01164501	Empagliflozin	Placebo	3.81 (0.18, 79.07)	2/419	0/319	0.28
NCT01730534	Dapagliflozin	Placebo	0.90 (0.67, 1.21)	84/8574	93/8569	29.64
NCT01986881	Ertugliflozin	Placebo	1.24 (0.75, 2.05)	52/5493	21/2745	10.02
NCT01989754	Canagliflozin	Placebo	0.82 (0.34, 1.97)	9/2904	11/2903	3.30
NCT02033889	Ertugliflozin	Placebo/Glimepiride	1.53 (0.06, 37.28)	1/412	0/209	0.25
NCT02065791	Canagliflozin	Placebo	0.69 (0.38, 1.26)	18/2200	26/2197	7.14
NCT02384941	Sotagliflozin	Placebo	1.53 (0.06, 37.53)	1/525	0/268	0.25
NCT03036124	Dapagliflozin	Placebo	0.90 (0.37, 2.21)	9/2368	10/2368	3.16
NCT03036150	Dapagliflozin	Placebo	0.93 (0.45, 1.93)	14/2149	15/2149	4.85
NCT03057951	Empagliflozin	Placebo	2.74 (1.22, 6.15)	22/2996	8/2989	3.91
NCT03057977	Empagliflozin	Placebo	2.67 (0.71, 10.04)	8/1863	3/1863	1.45
NCT03242252	Sotagliflozin	Placebo	0.99 (0.09, 10.83)	2/527	1/260	0.44
NCT03315143	Sotagliflozin	Placebo	1.29 (0.69, 2.43)	22/5291	17/5286	6.40
NCT03521934	Sotagliflozin	Placebo	4.04 (0.45, 36.04)	4/605	1/611	0.53
NCT03594110	Empagliflozin	Placebo	0.75 (0.32, 1.78)	9/3304	12/3305	3.43
NCT03619213	Dapagliflozin	Placebo	1.59 (0.94, 2.71)	35/3126	22/3127	9.06
Overall (I-squared = 13.6%, p = 0.288)			1.02 (0.87, 1.19)	350/51064	280/43308	100.00

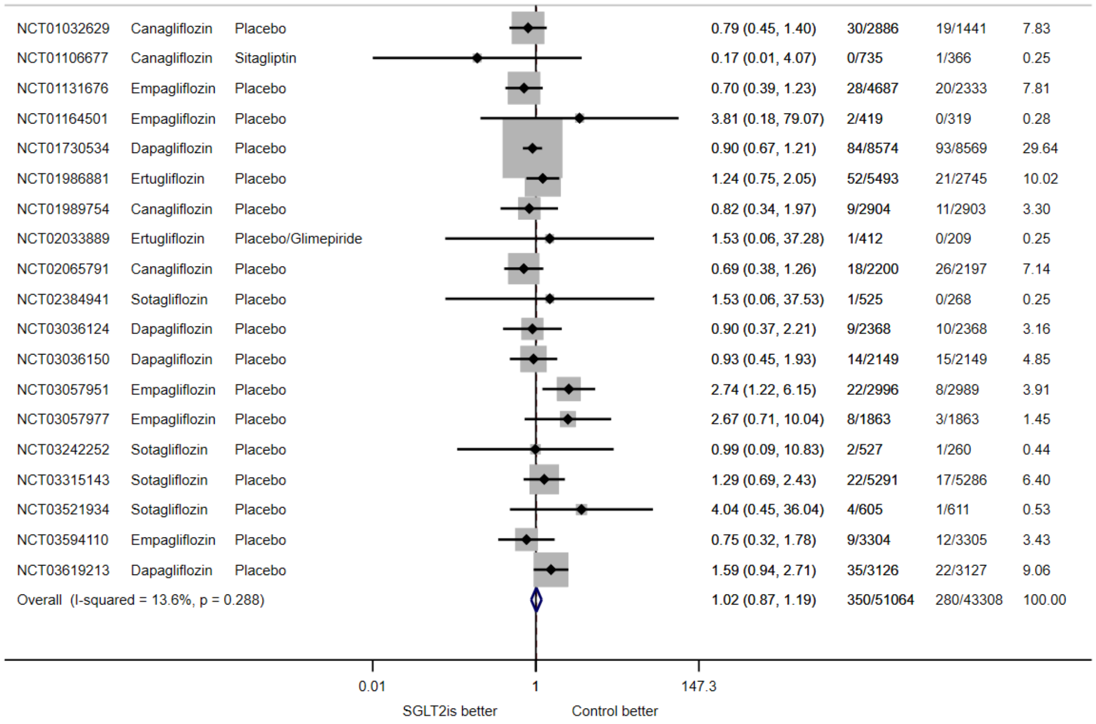

System 11-Infections And Infestations-Figure S22 Meta-analysis of SGLT2is and Cellulitis

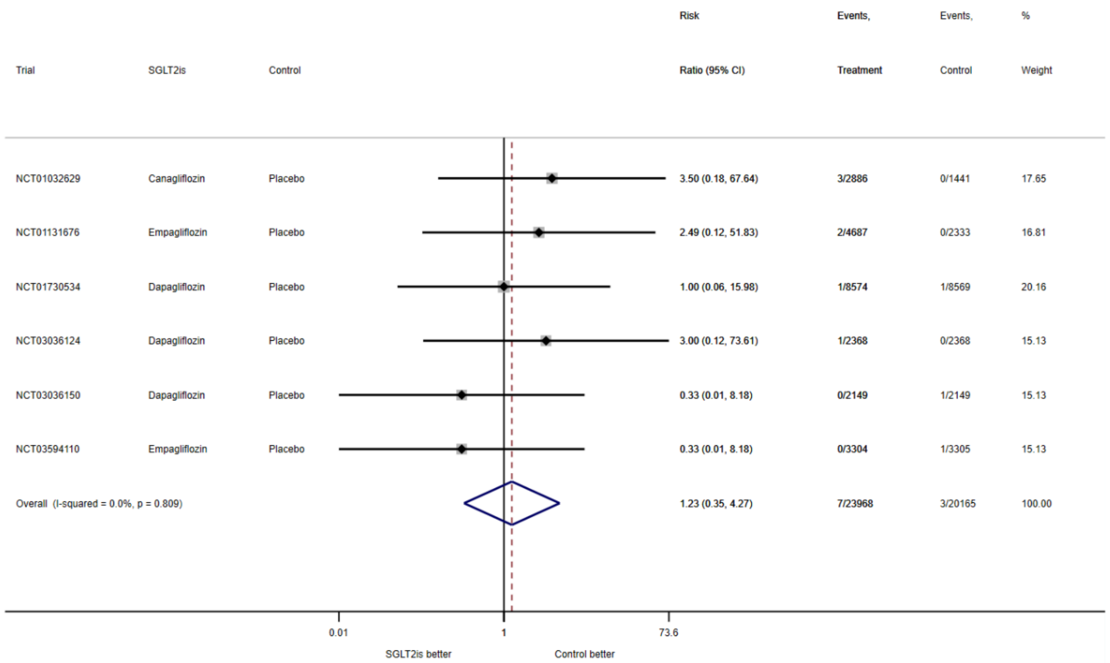

System 11-Infections And Infestations-Figure S23 Meta-analysis of SGLT2is and Cellulitis gangrenous

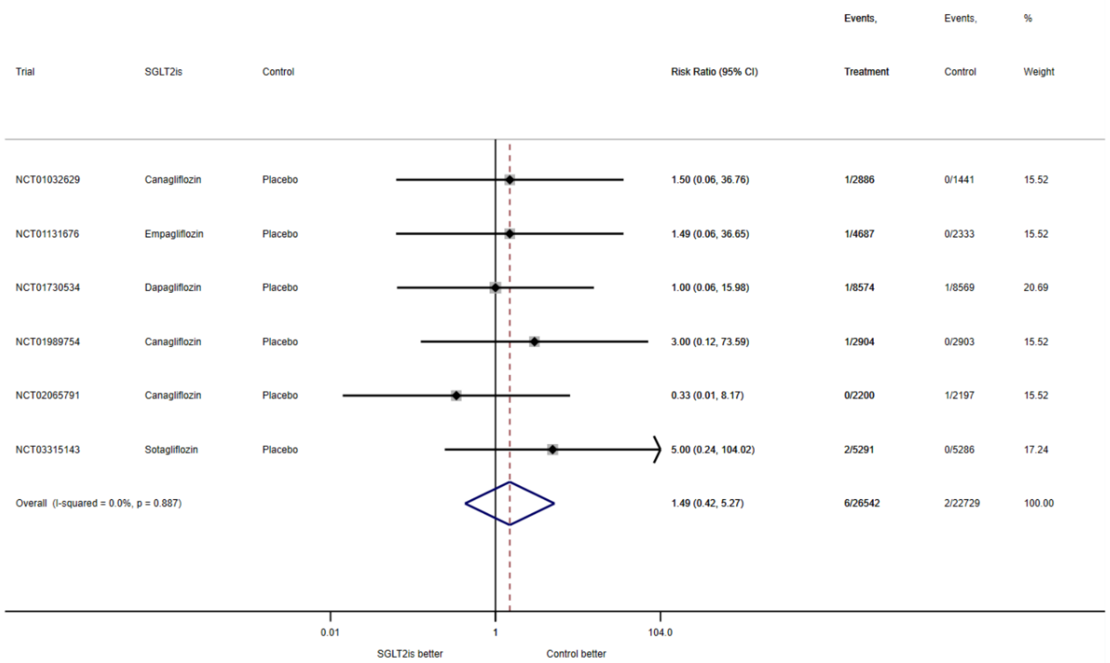

System 11-Infections And Infestations-Figure S24 Meta-analysis of SGLT2is and Cellulitis staphylococcal

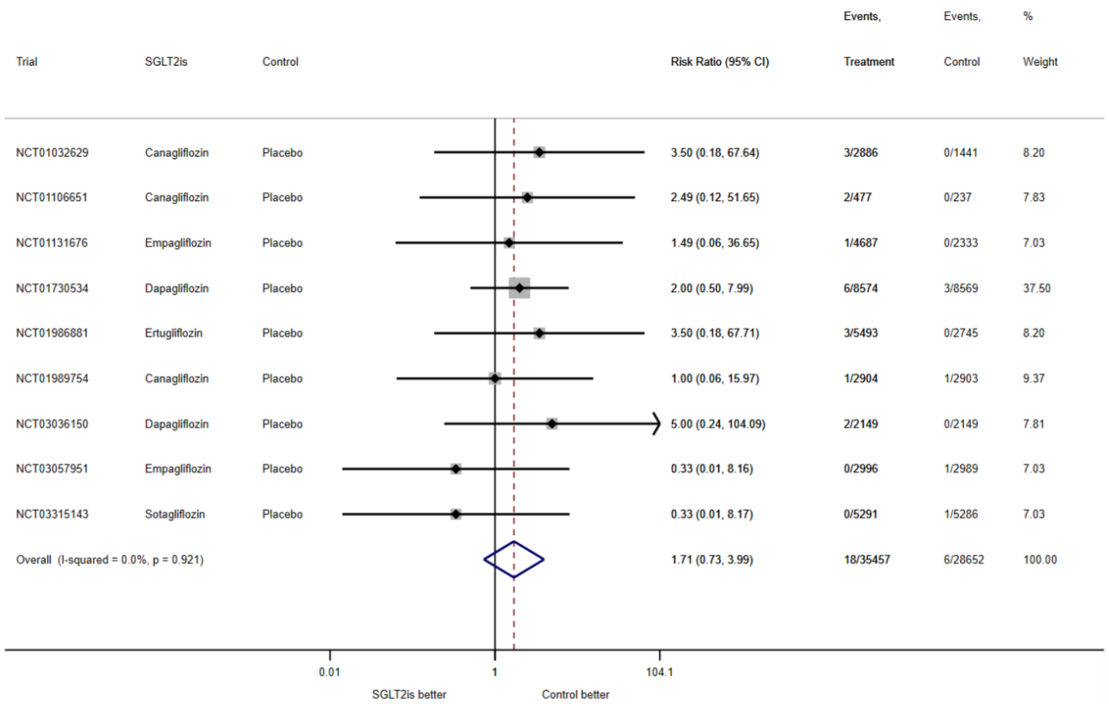

System 11-Infections And Infestations-Figure S25 Meta-analysis of SGLT2is and Cholecystitis infective

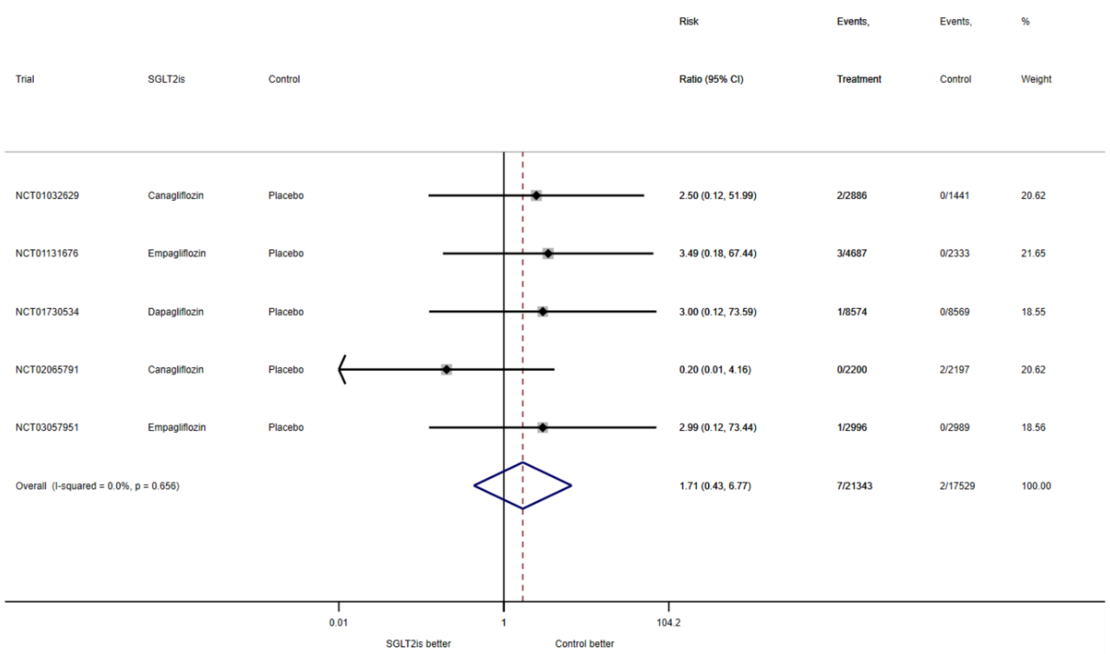

System 11-Infections And Infestations-Figure S26 Meta-analysis of SGLT2is and Chronic sinusitis

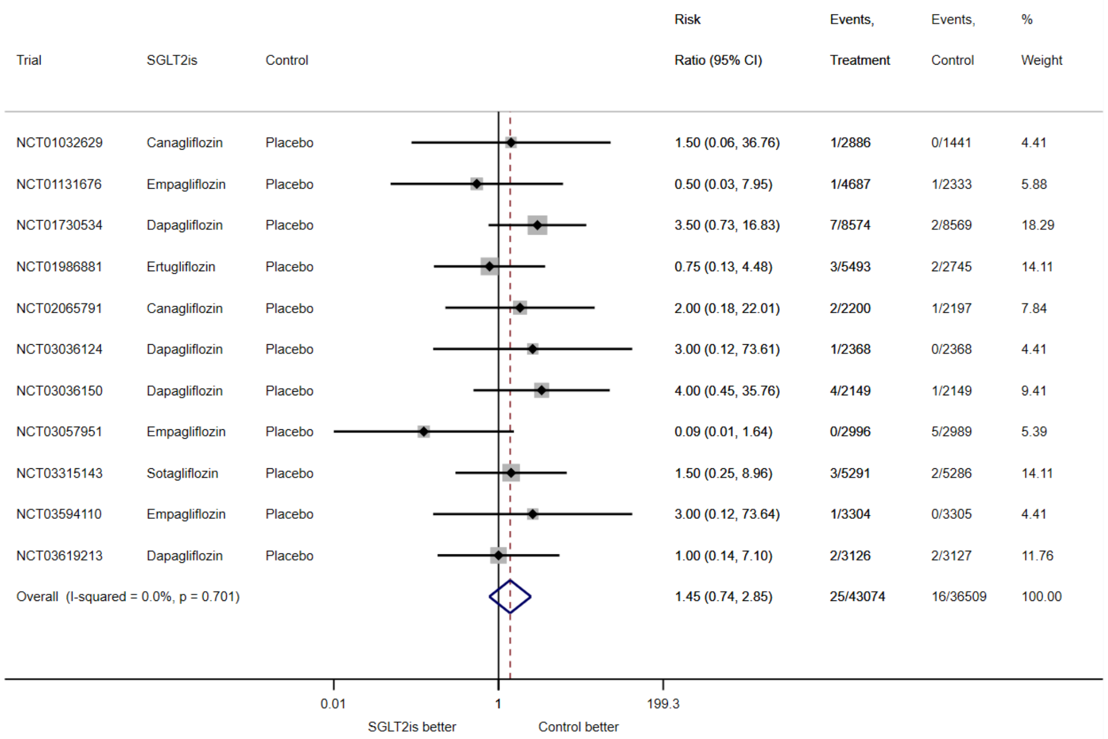

System 11-Infections And Infestations-Figure S27 Meta-analysis of SGLT2is and Clostridium difficile colitis

Trial	SGLT2is	Control	Risk Ratio (95% CI)	Events, Treatment	Events, Control	% Weight
NCT01032629	Canagliflozin	Placebo	0.17 (0.01, 4.08)	0/2886	1/1441	8.82
NCT01730534	Dapagliflozin	Placebo	4.00 (0.45, 35.76)	4/8574	1/8569	18.82
NCT02065791	Canagliflozin	Placebo	3.00 (0.12, 73.50)	1/2200	0/2197	8.82
NCT03036124	Dapagliflozin	Placebo	0.33 (0.01, 8.18)	0/2368	1/2368	8.82
NCT03036150	Dapagliflozin	Placebo	3.00 (0.12, 73.60)	1/2149	0/2149	8.82
NCT03057951	Empagliflozin	Placebo	0.33 (0.03, 3.20)	1/2996	3/2989	17.65
NCT03057977	Empagliflozin	Placebo	0.33 (0.01, 8.18)	0/1863	1/1863	8.82
NCT03521934	Sotagliflozin	Placebo	3.03 (0.12, 74.22)	1/605	0/611	8.83
NCT03619213	Dapagliflozin	Placebo	0.11 (0.01, 2.06)	0/3126	4/3127	10.59
Overall (I-squared = 0.0%, p = 0.446)			0.80 (0.31, 2.06)	8/26767	11/25314	100.00

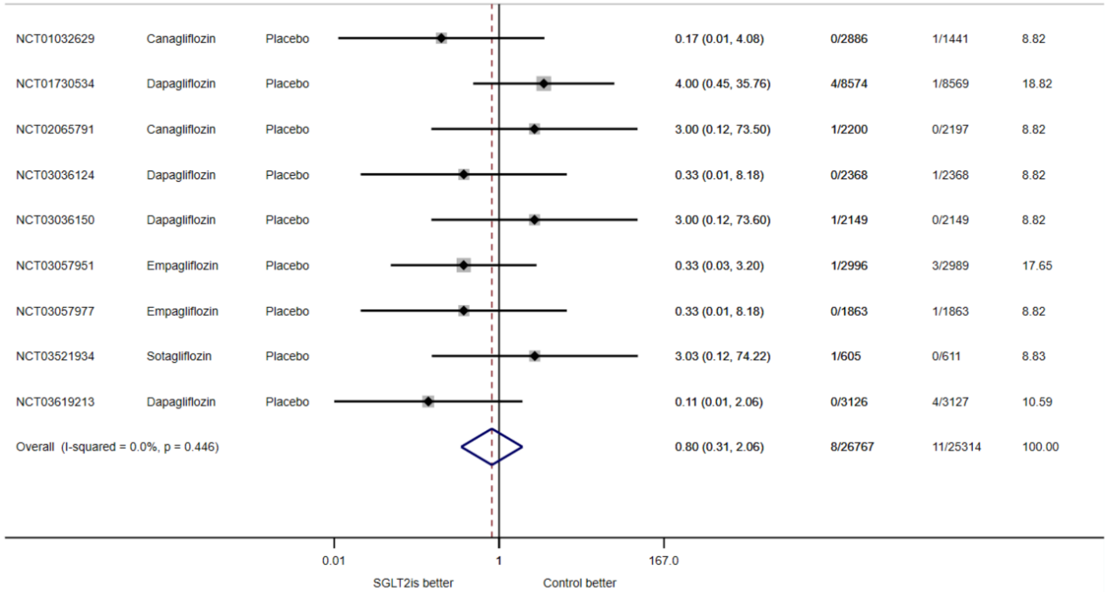

System 11-Infections And Infestations-Figure S28 Meta-analysis of SGLT2is and Clostridium difficile infection

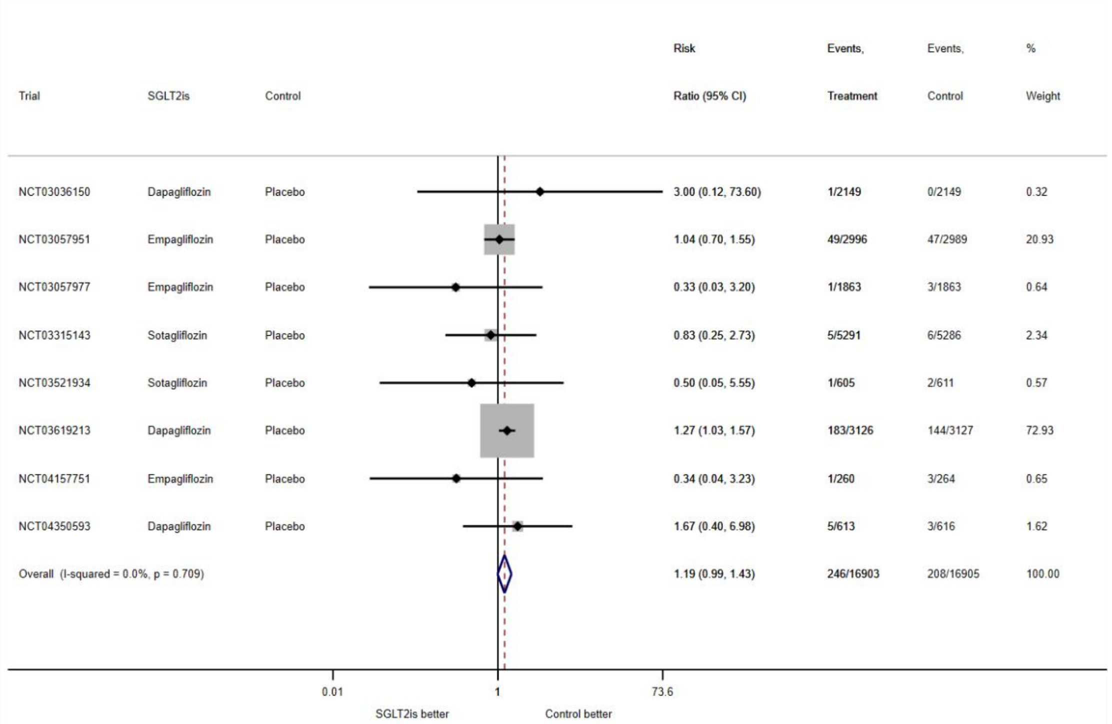

System 11-Infections And Infestations-Figure S29 Meta-analysis of SGLT2is and Covid-19

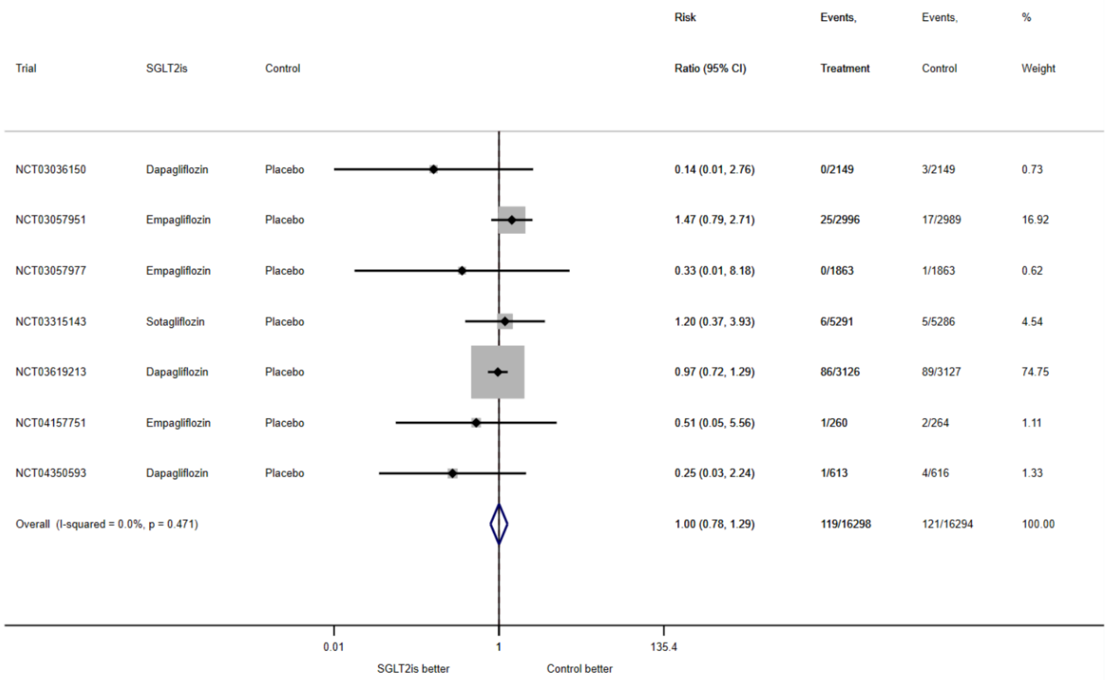

System 11-Infections And Infestations-Figure S30 Meta-analysis of SGLT2is and Covid-19 pneumonia

Trial	SGLT2is	Control	Risk Ratio (95% CI)	Events, Treatment	Events, Control	% Weight
NCT01131676	Empagliflozin	Placebo	0.10 (0.00, 2.07)	0/4687	2/2333	4.22
NCT01730534	Dapagliflozin	Placebo	3.00 (0.81, 11.07)	9/8574	3/8569	22.80
NCT01986881	Ertugliflozin	Placebo	1.00 (0.09, 11.02)	2/5493	1/2745	6.76
NCT02065791	Canagliflozin	Placebo	4.99 (0.24, 103.94)	2/2200	0/2197	4.22
NCT03036124	Dapagliflozin	Placebo	0.33 (0.01, 8.18)	0/2368	1/2368	3.80
NCT03036150	Dapagliflozin	Placebo	0.50 (0.05, 5.51)	1/2149	2/2149	6.76
NCT03057951	Empagliflozin	Placebo	1.00 (0.14, 7.08)	2/2996	2/2989	10.14
NCT03057977	Empagliflozin	Placebo	0.33 (0.01, 8.18)	0/1863	1/1863	3.80
NCT03315143	Sotagliflozin	Placebo	2.00 (0.50, 7.99)	6/5291	3/5286	20.27
NCT03594110	Empagliflozin	Placebo	1.00 (0.06, 15.99)	1/3304	1/3305	5.07
NCT03619213	Dapagliflozin	Placebo	1.50 (0.25, 8.97)	3/3126	2/3127	12.16
Overall (I-squared = 0.0%, p = 0.666)			1.32 (0.71, 2.47)	26/42051	18/36931	100.00

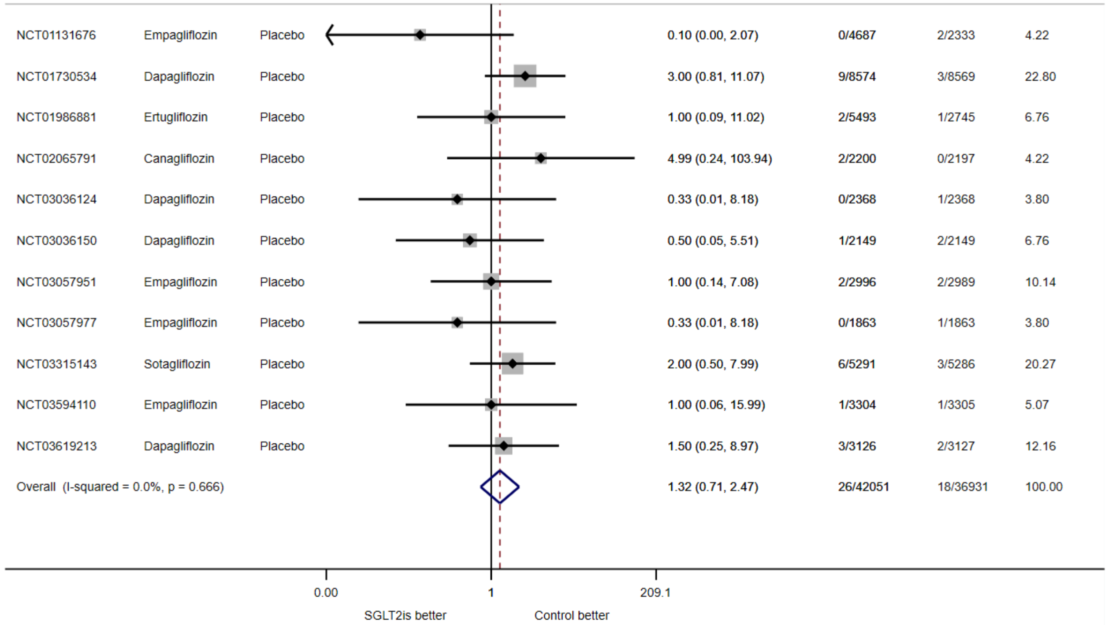

System 11-Infections And Infestations-Figure S31 Meta-analysis of SGLT2is and Cystitis

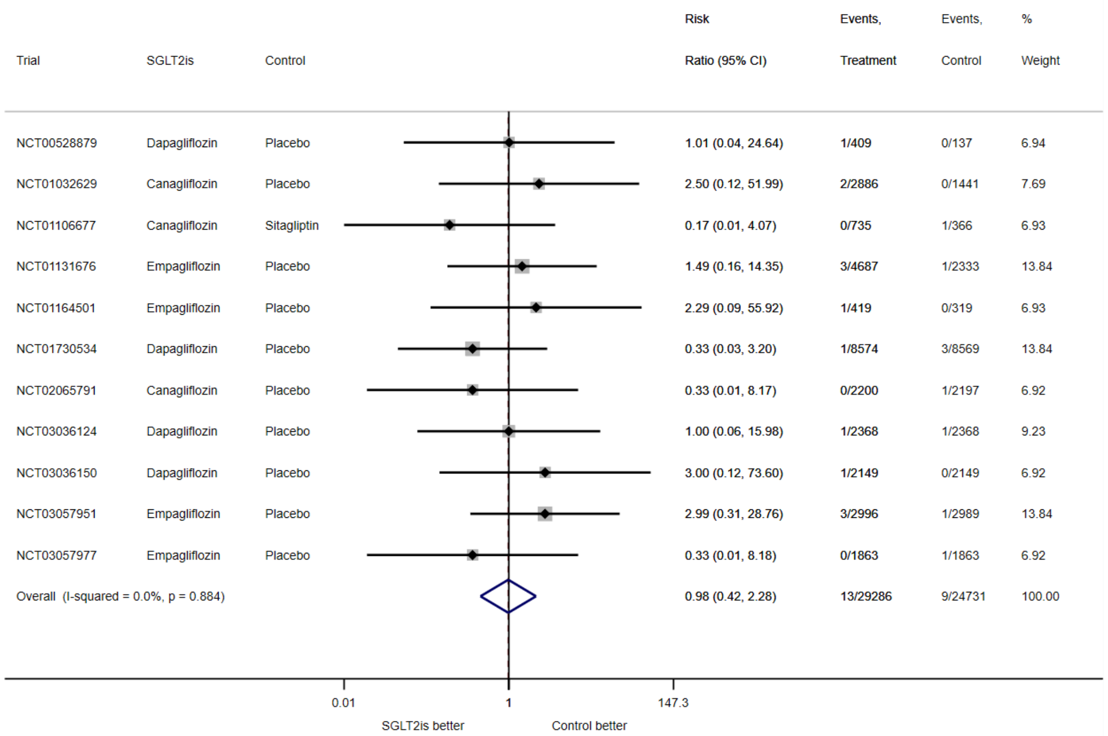

System 11-Infections And Infestations-Figure S32 Meta-analysis of SGLT2is and Dengue fever

Trial	SGLT2is	Control	Risk Ratio (95% CI)	Events, Treatment	Events, Control	% Weight
NCT01032629	Canagliflozin	Placebo	1.50 (0.16, 14.39)	3/2886	1/1441	10.41
NCT01131676	Empagliflozin	Placebo	0.17 (0.01, 4.07)	0/4687	1/2333	5.20
NCT01730534	Dapagliflozin	Placebo	2.00 (0.37, 10.91)	4/8574	2/8569	18.49
NCT01989754	Canagliflozin	Placebo	2.00 (0.18, 22.04)	2/2904	1/2903	9.25
NCT02065791	Canagliflozin	Placebo	3.00 (0.12, 73.50)	1/2200	0/2197	5.20
NCT03036124	Dapagliflozin	Placebo	1.00 (0.20, 4.95)	3/2368	3/2368	20.82
NCT03036150	Dapagliflozin	Placebo	1.00 (0.06, 15.98)	1/2149	1/2149	6.94
NCT03057951	Empagliflozin	Placebo	0.33 (0.01, 8.16)	0/2996	1/2989	5.20
NCT03057977	Empagliflozin	Placebo	5.00 (0.24, 104.08)	2/1863	0/1863	5.78
NCT03594110	Empagliflozin	Placebo	1.00 (0.06, 15.99)	1/3304	1/3305	6.93
NCT03619213	Dapagliflozin	Placebo	5.00 (0.24, 104.14)	2/3126	0/3127	5.78
Overall (I-squared = 0.0%, p = 0.917)			1.39 (0.67, 2.88)	19/37057	11/33244	100.00

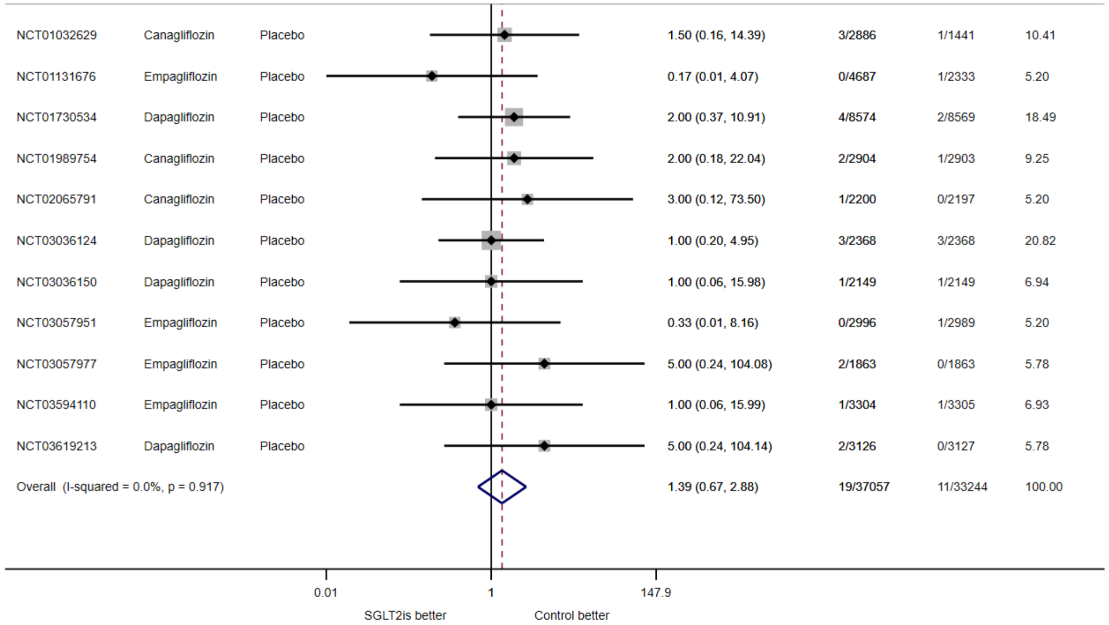

System 11-Infections And Infestations-Figure S33 Meta-analysis of SGLT2is and Device related infection

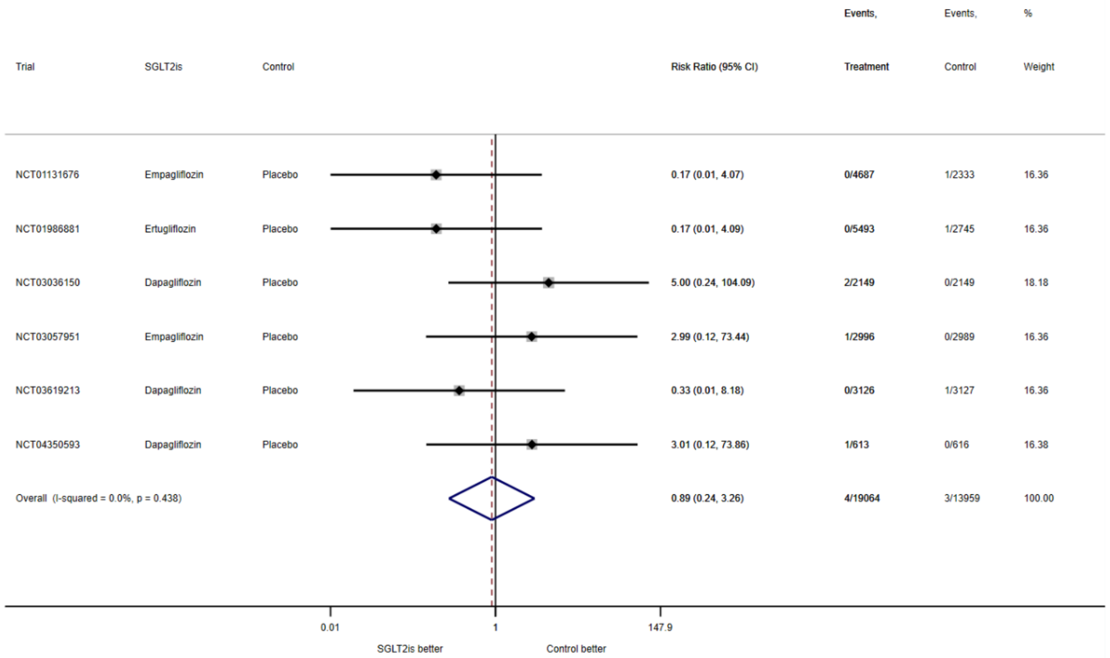

System 11-Infections And Infestations-Figure S34 Meta-analysis of SGLT2is and Device related sepsis

Trial	SGLT2is	Control	Risk Ratio (95% CI)	Events, Treatment	Events, Control	% Weight
NCT01032629	Canagliflozin	Placebo	3.99 (0.50, 31.91)	8/2886	1/1441	3.24
NCT01131676	Empagliflozin	Placebo	1.00 (0.37, 2.65)	12/4687	6/2333	14.61
NCT01730534	Dapagliflozin	Placebo	0.77 (0.34, 1.75)	10/8574	13/8569	20.62
NCT01986881	Ertugliflozin	Placebo	0.50 (0.10, 2.47)	3/5493	3/2745	5.47
NCT01989754	Canagliflozin	Placebo	0.75 (0.17, 3.35)	3/2904	4/2903	6.25
NCT02065791	Canagliflozin	Placebo	0.75 (0.17, 3.34)	3/2200	4/2197	6.26
NCT03036124	Dapagliflozin	Placebo	3.00 (0.12, 73.61)	1/2368	0/2368	1.37
NCT03036150	Dapagliflozin	Placebo	1.00 (0.29, 3.45)	5/2149	5/2149	9.13
NCT03057951	Empagliflozin	Placebo	0.20 (0.01, 4.15)	0/2996	2/2989	1.52
NCT03057977	Empagliflozin	Placebo	7.00 (0.36, 135.42)	3/1863	0/1863	1.59
NCT03315143	Sotagliflozin	Placebo	2.25 (0.69, 7.29)	9/5291	4/5286	10.10
NCT03594110	Empagliflozin	Placebo	2.83 (1.12, 7.18)	17/3304	6/3305	16.20
NCT03619213	Dapagliflozin	Placebo	1.00 (0.14, 7.10)	2/3126	2/3127	3.65
Overall (I-squared = 0.0%, p = 0.448)			1.21 (0.83, 1.75)	76/47841	50/41275	100.00

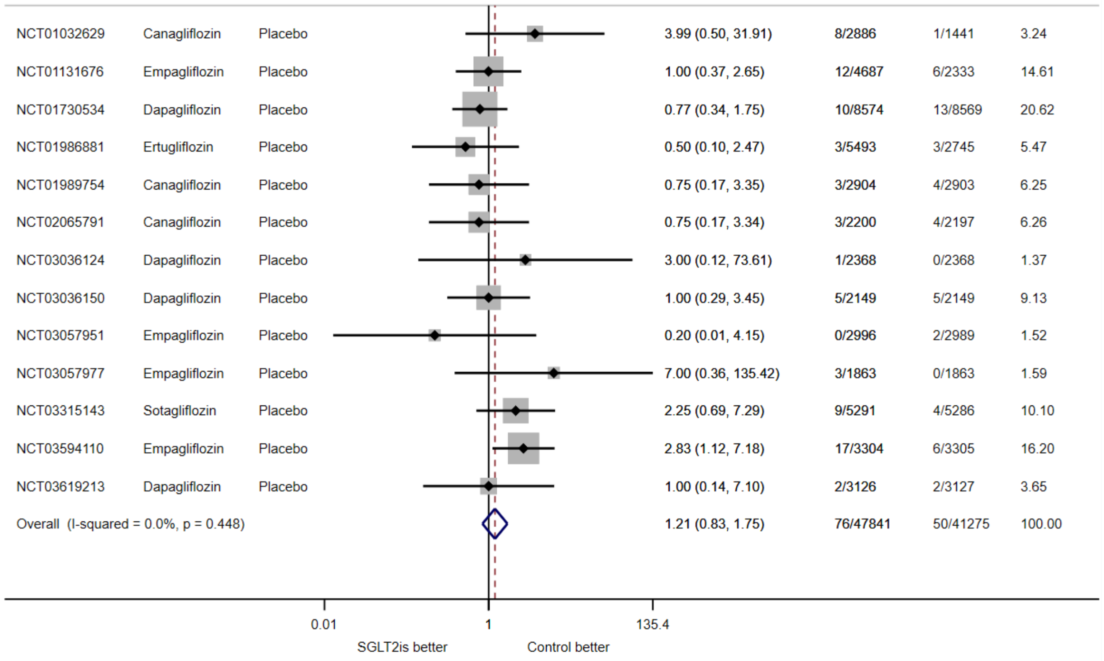

System 11-Infections And Infestations-Figure S35 Meta-analysis of SGLT2is and Diabetic foot infection

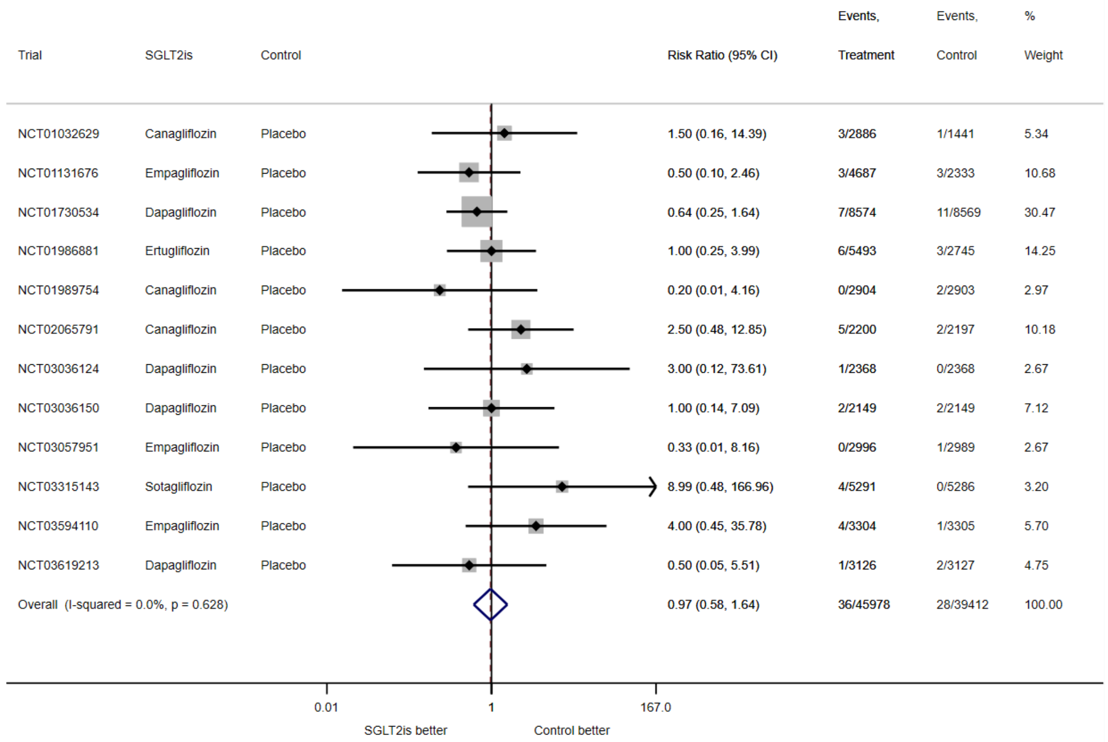

System 11-Infections And Infestations-Figure S36 Meta-analysis of SGLT2is and Diabetic gangrene

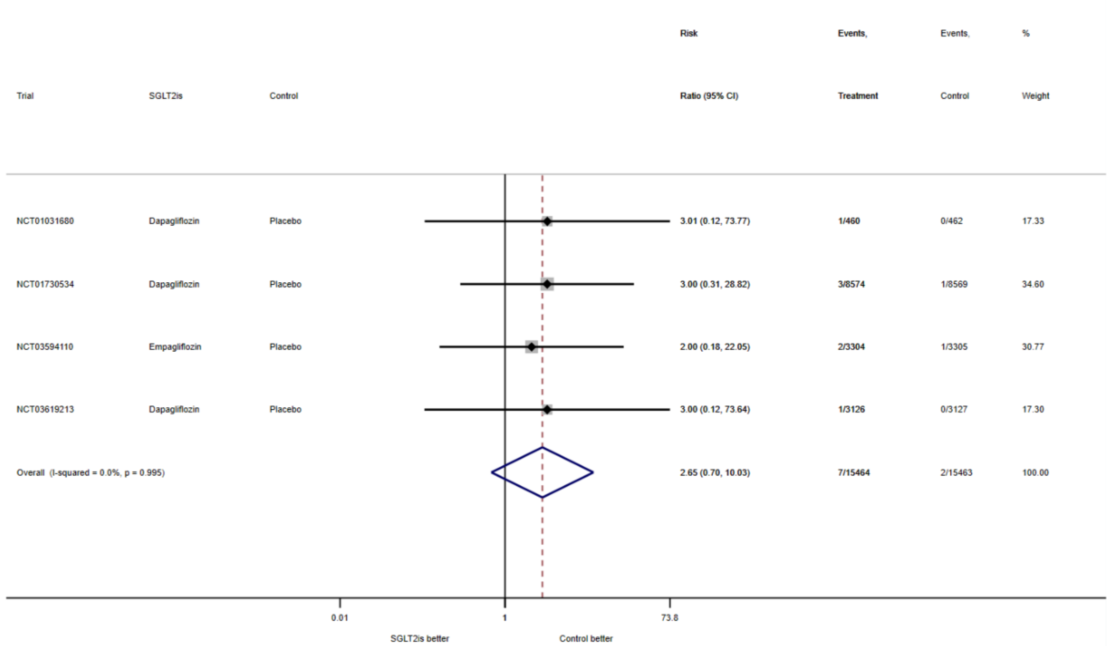

System 11-Infections And Infestations-Figure S37 Meta-analysis of SGLT2is and Diarrhoea infectious

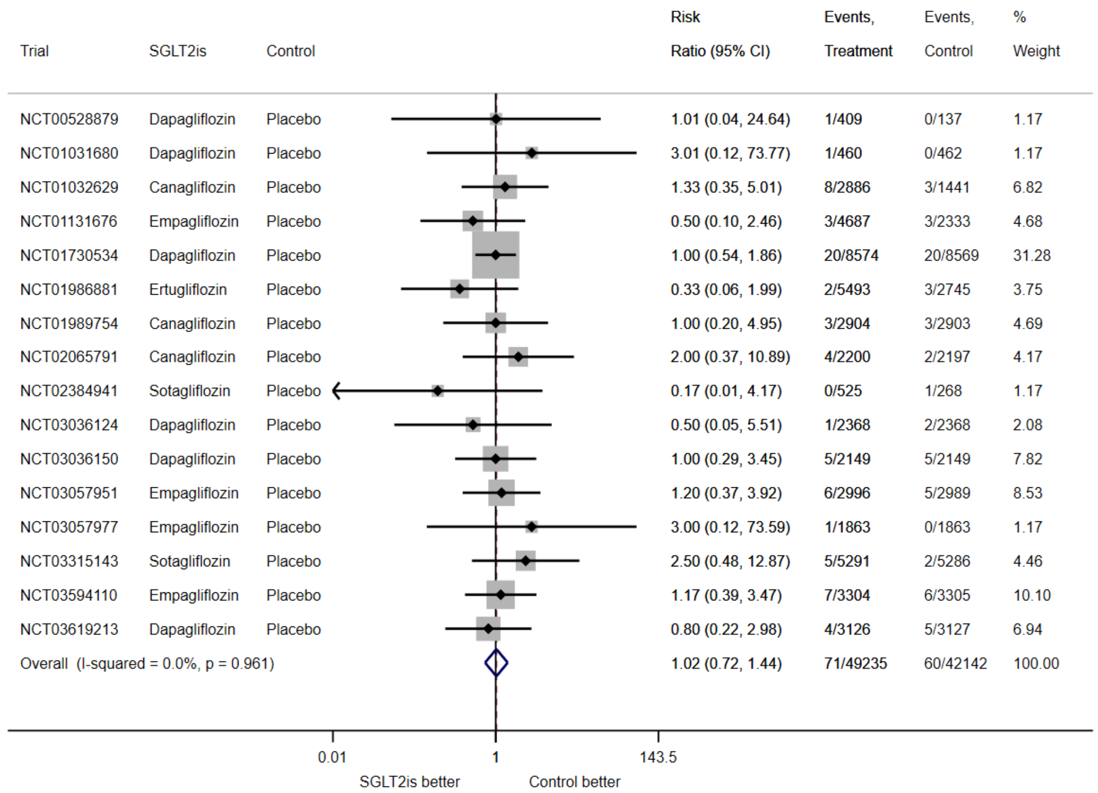

System 11-Infections And Infestations-Figure S38 Meta-analysis of SGLT2is and Diverticulitis

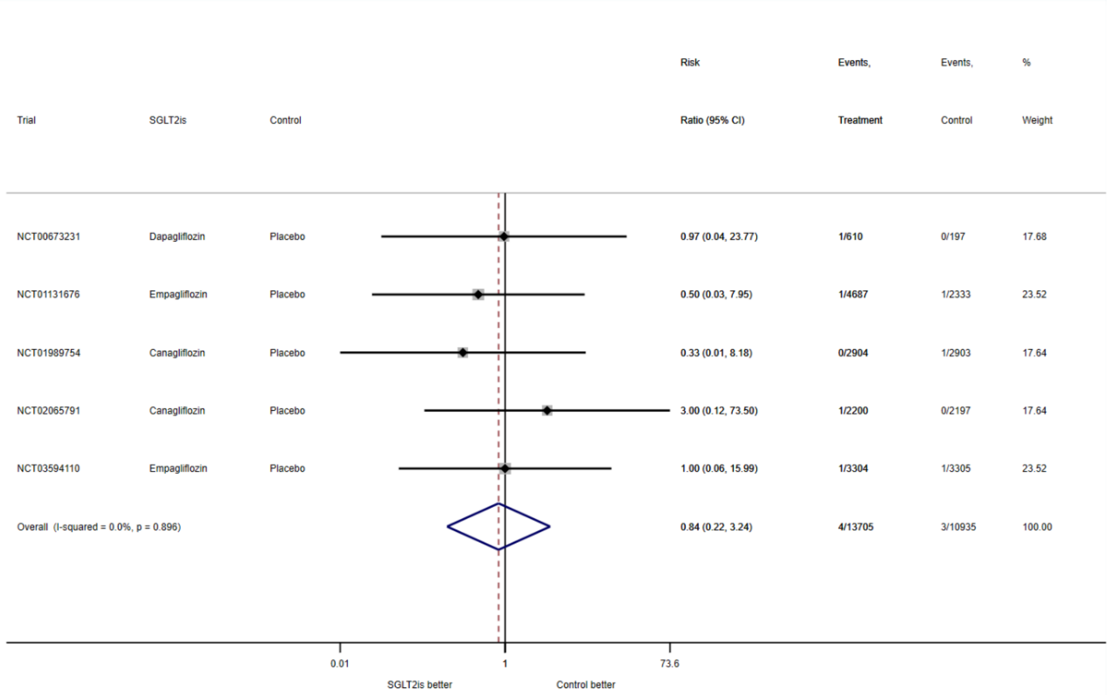

System 11-Infections And Infestations-Figure S39 Meta-analysis of SGLT2is and Ear infection

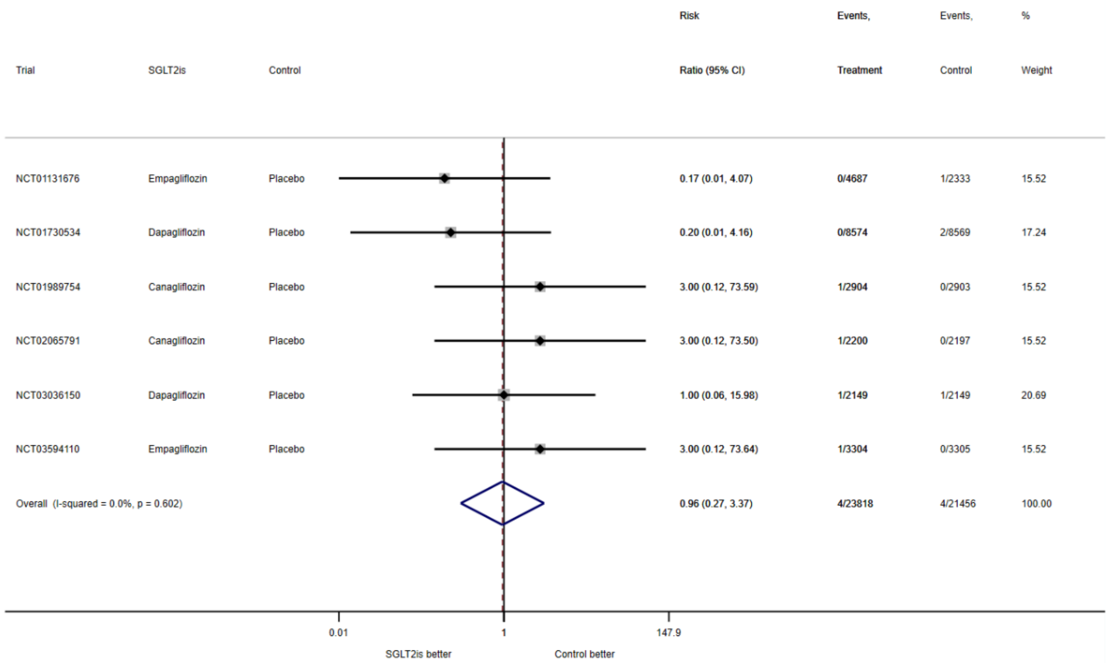

System 11-Infections And Infestations-Figure S40 Meta-analysis of SGLT2is and Empyema

Trial	SGLT2is	Control	Risk Ratio (95% CI)	Events, Treatment	Events, Control	% Weight
NCT01032629	Canagliflozin	Placebo	1.50 (0.06, 36.76)	1/2886	0/1441	3.47
NCT01131676	Empagliflozin	Placebo	0.50 (0.03, 7.95)	1/4687	1/2333	4.63
NCT01730534	Dapagliflozin	Placebo	2.00 (0.37, 10.91)	4/8574	2/8569	12.34
NCT01986881	Ertugliflozin	Placebo	1.50 (0.06, 36.80)	1/5493	0/2745	3.47
NCT01989754	Canagliflozin	Placebo	5.00 (0.24, 104.07)	2/2904	0/2903	3.85
NCT02065791	Canagliflozin	Placebo	0.33 (0.01, 8.17)	0/2200	1/2197	3.47
NCT02384941	Sotagliflozin	Placebo	0.17 (0.01, 4.17)	0/525	1/268	3.48
NCT03036124	Dapagliflozin	Placebo	0.25 (0.03, 2.24)	1/2368	4/2368	7.40
NCT03036150	Dapagliflozin	Placebo	3.00 (0.12, 73.60)	1/2149	0/2149	3.47
NCT03057951	Empagliflozin	Placebo	1.66 (0.40, 6.95)	5/2996	3/2989	17.36
NCT03057977	Empagliflozin	Placebo	0.67 (0.11, 3.99)	2/1863	3/1863	11.11
NCT03315143	Sotagliflozin	Placebo	0.50 (0.05, 5.51)	1/5291	2/5286	6.17
NCT03521934	Sotagliflozin	Placebo	1.01 (0.06, 16.11)	1/605	1/611	4.63
NCT03594110	Empagliflozin	Placebo	0.14 (0.01, 2.77)	0/3304	3/3305	4.05
NCT03619213	Dapagliflozin	Placebo	0.67 (0.11, 3.99)	2/3126	3/3127	11.11
Overall (I-squared = 0.0%, p = 0.871)			0.87 (0.48, 1.57)	22/48971	24/42154	100.00

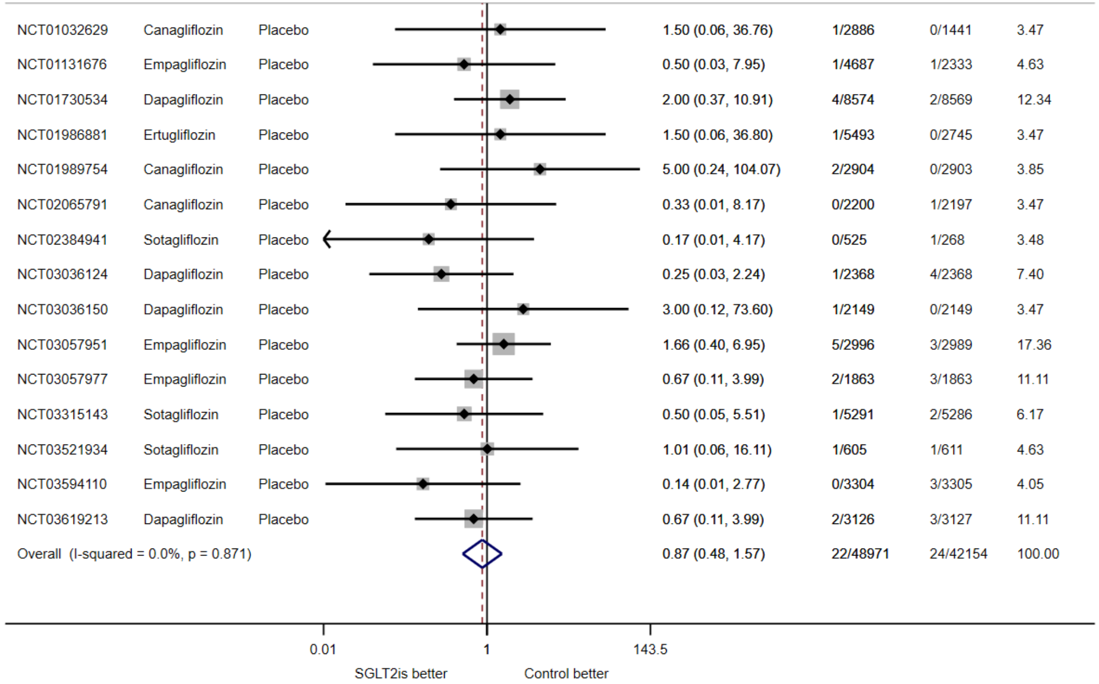

System 11-Infections And Infestations-Figure S41 Meta-analysis of SGLT2is and Endocarditis

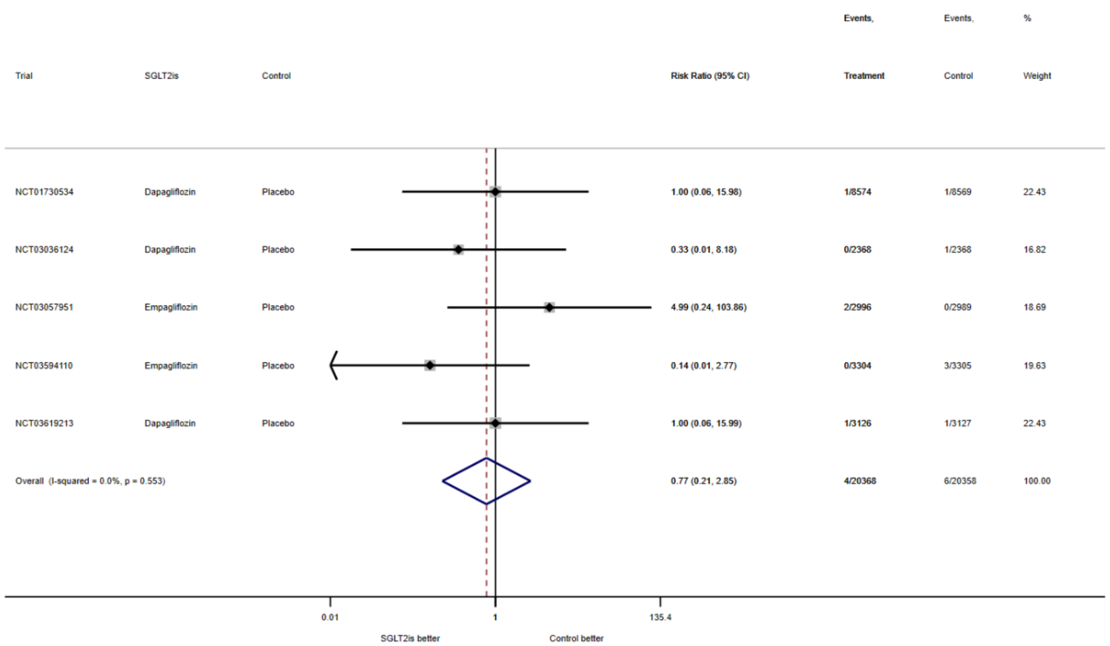

System 11-Infections And Infestations-Figure S42 Meta-analysis of SGLT2is and Endocarditis bacterial

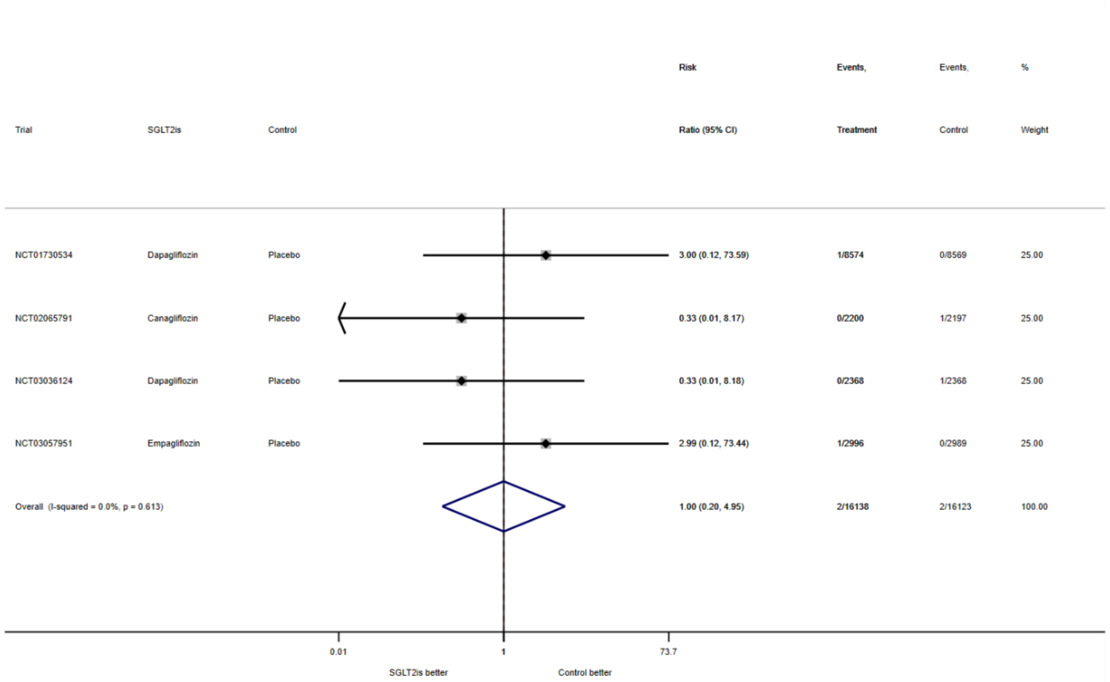

System 11-Infections And Infestations-Figure S43 Meta-analysis of SGLT2is and Enteritis infectious

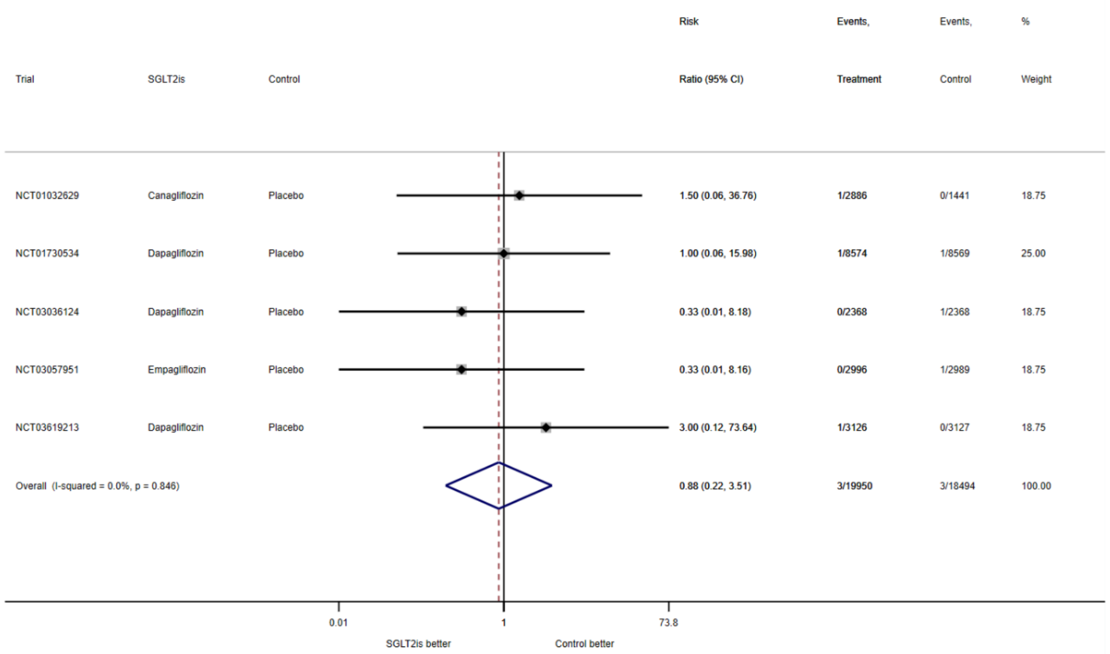

System 11-Infections And Infestations-Figure S44 Meta-analysis of SGLT2is and Enterococcal bacteraemia

Trial	SGLT2is	Control	Risk Ratio (95% CI)	Events, Treatment	Events, Control	% Weight
NCT01131676	Empagliflozin	Placebo	0.17 (0.01, 4.07)	0/4687	1/2333	10.91
NCT01730534	Dapagliflozin	Placebo	1.00 (0.14, 7.09)	2/8574	2/8569	29.09
NCT01986881	Ertugliflozin	Placebo	0.50 (0.03, 7.99)	1/5493	1/2745	14.55
NCT01989754	Canagliflozin	Placebo	0.33 (0.01, 8.18)	0/2904	1/2903	10.91
NCT02065791	Canagliflozin	Placebo	0.33 (0.01, 8.17)	0/2200	1/2197	10.91
NCT03036124	Dapagliflozin	Placebo	0.14 (0.01, 2.76)	0/2368	3/2368	12.73
NCT03057951	Empagliflozin	Placebo	2.99 (0.12, 73.44)	1/2996	0/2989	10.91
Overall (I-squared = 0.0%, p = 0.816)			0.51 (0.18, 1.48)	4/29222	9/24104	100.00

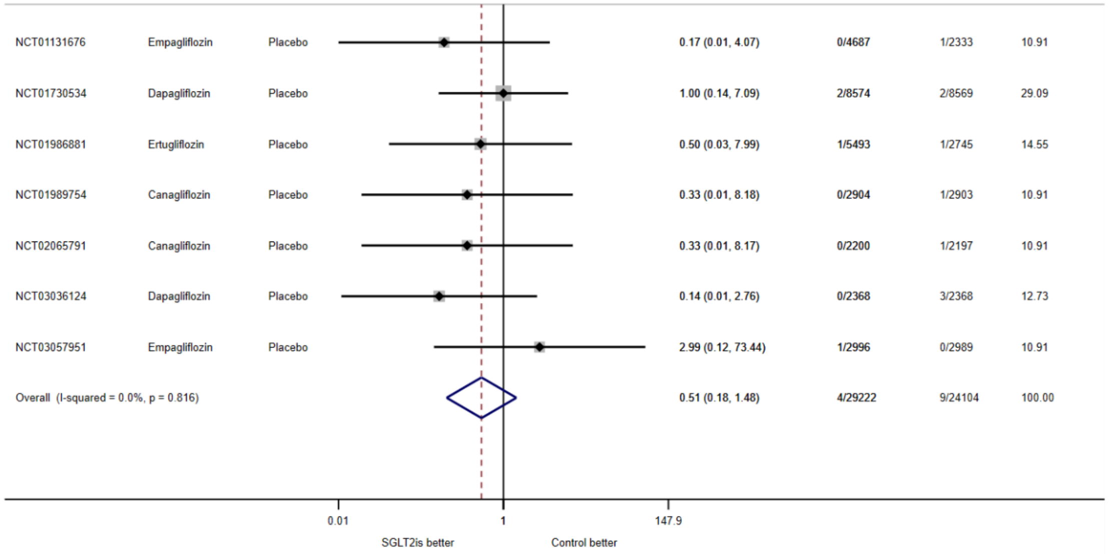

System 11-Infections And Infestations-Figure S45 Meta-analysis of SGLT2is and Epididymitis

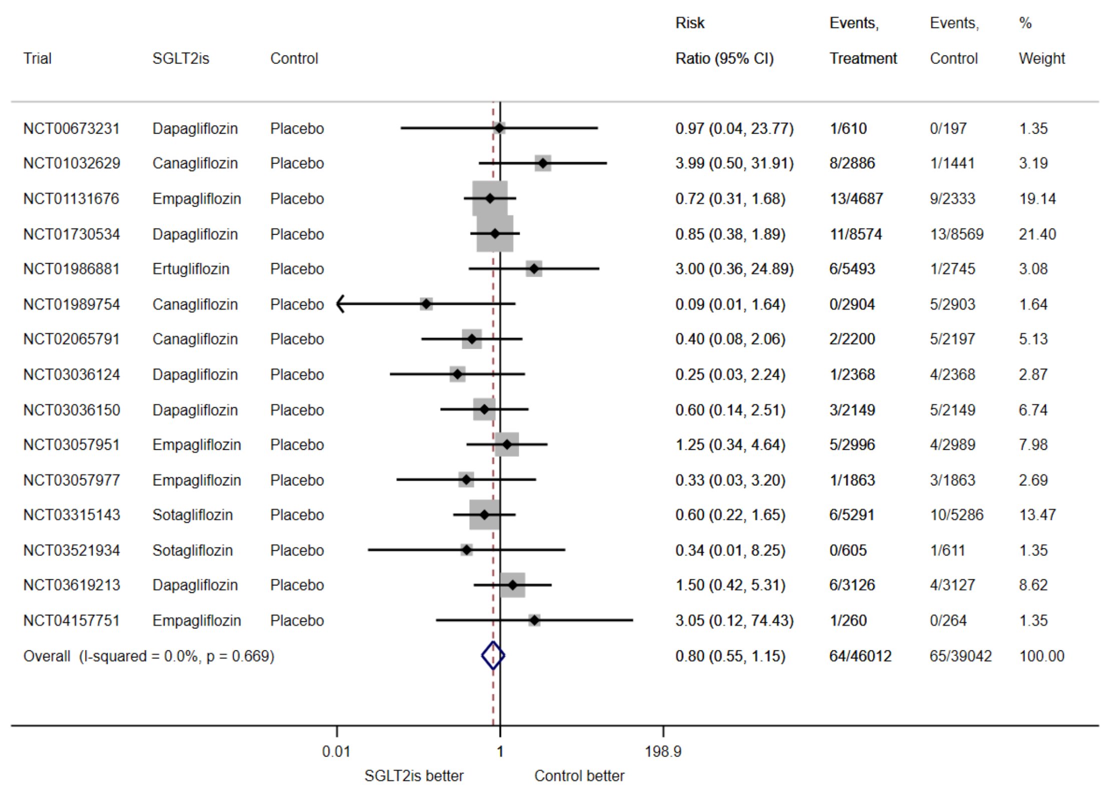

System 11-Infections And Infestations-Figure S46 Meta-analysis of SGLT2is and Erysipelas

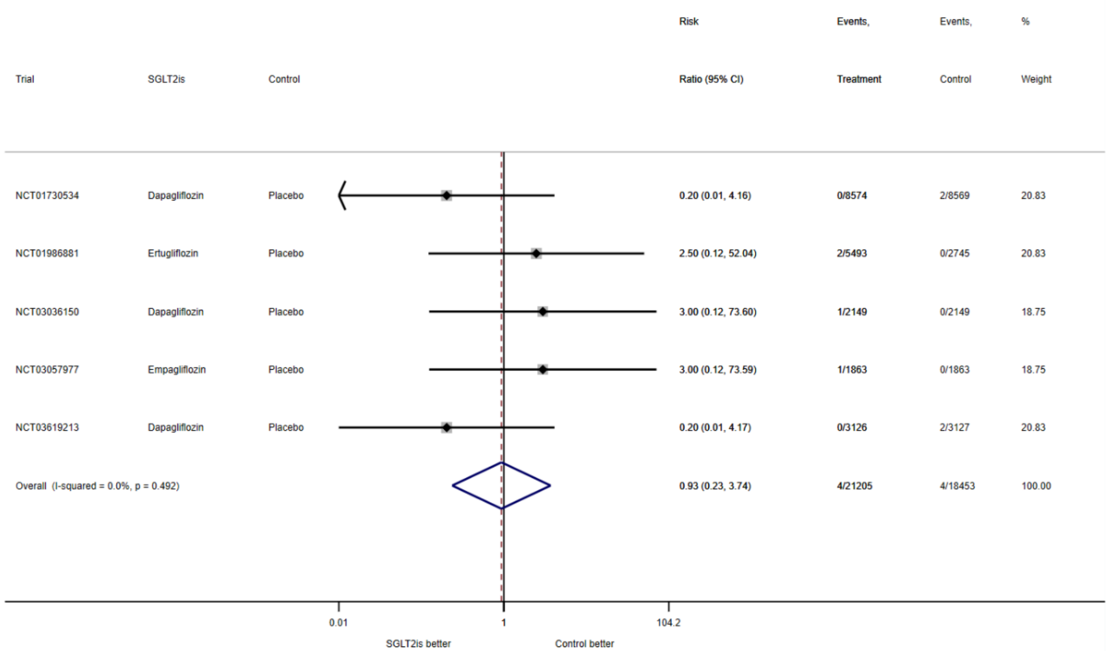

System 11-Infections And Infestations-Figure S47 Meta-analysis of SGLT2is and Escherichia bacteraemia

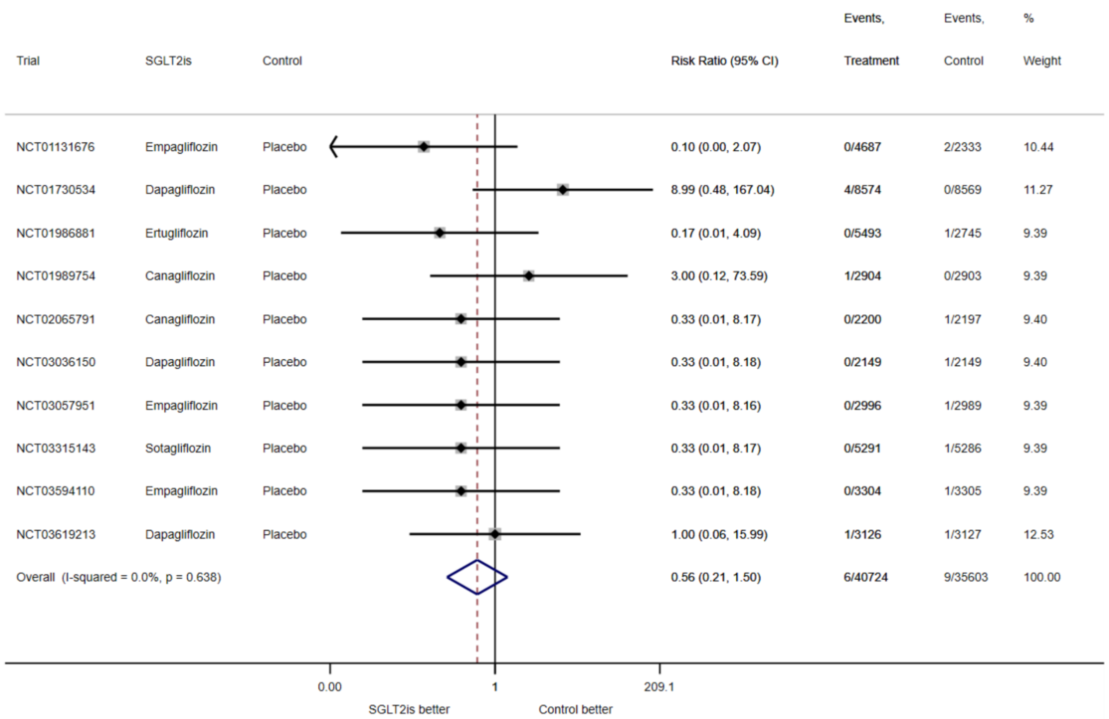

System 11-Infections And Infestations-Figure S48 Meta-analysis of SGLT2is and Escherichia sepsis

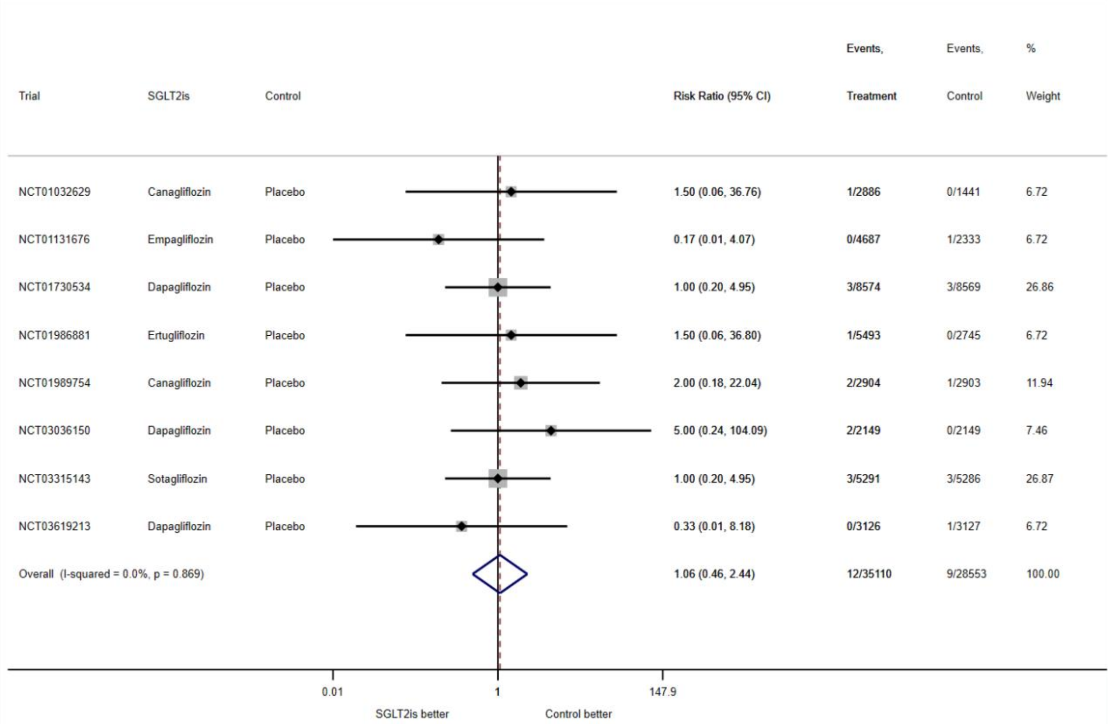

System 11-Infections And Infestations-Figure S49 Meta-analysis of SGLT2is and Escherichia urinary tract infection

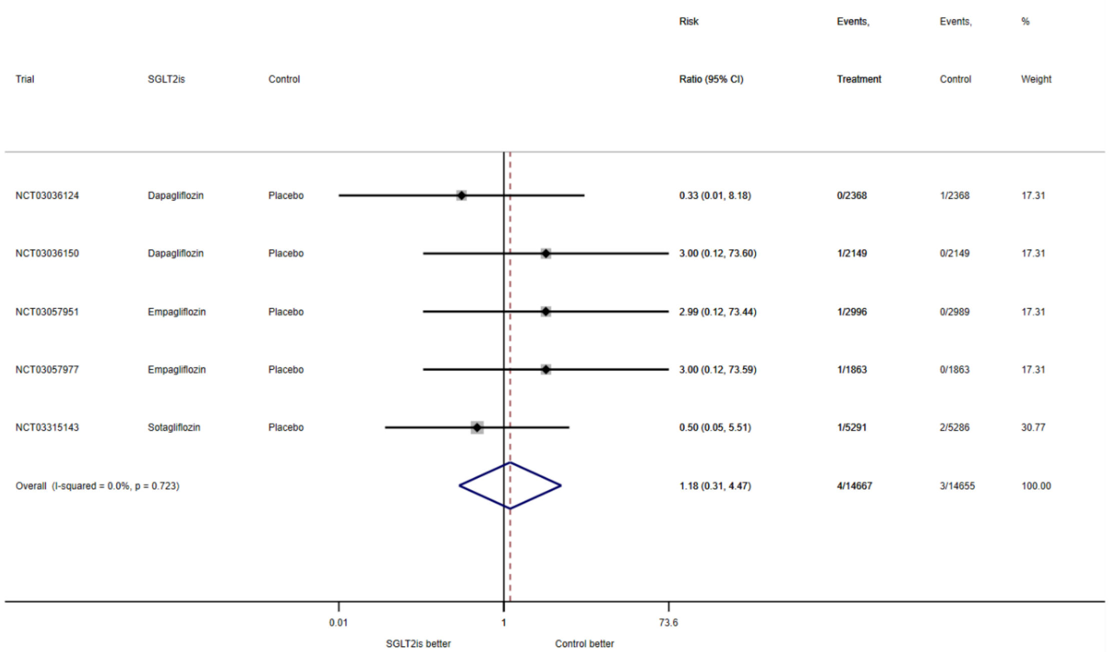

System 11-Infections And Infestations-Figure S50 Meta-analysis of SGLT2is and Fournier's gangrene

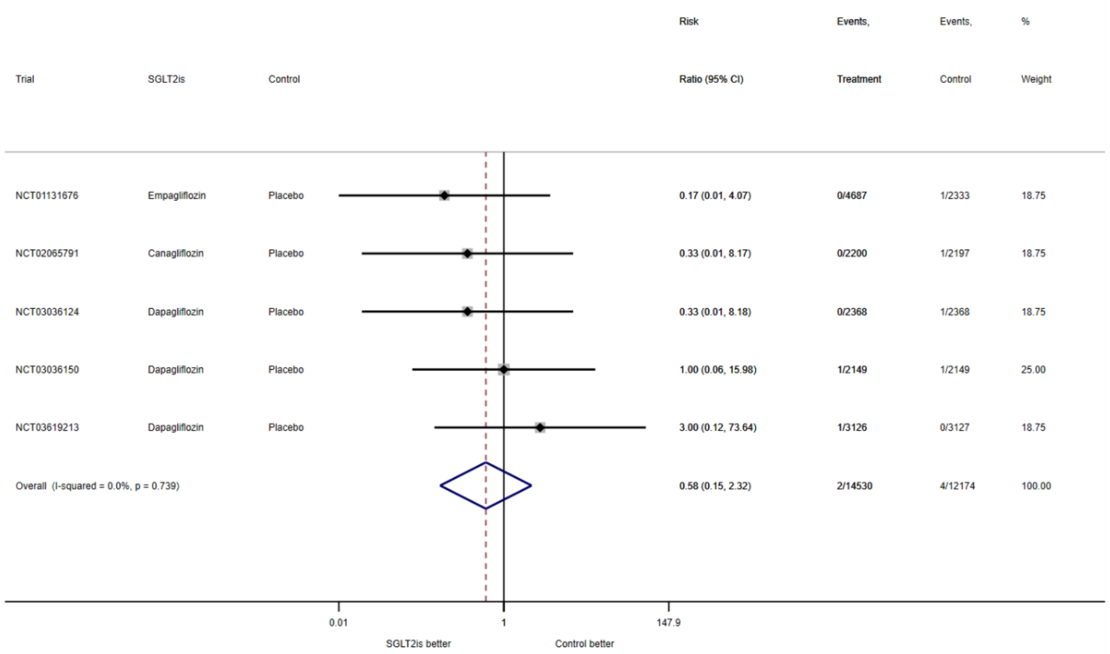

System 11-Infections And Infestations-Figure S51 Meta-analysis of SGLT2is and Furuncle

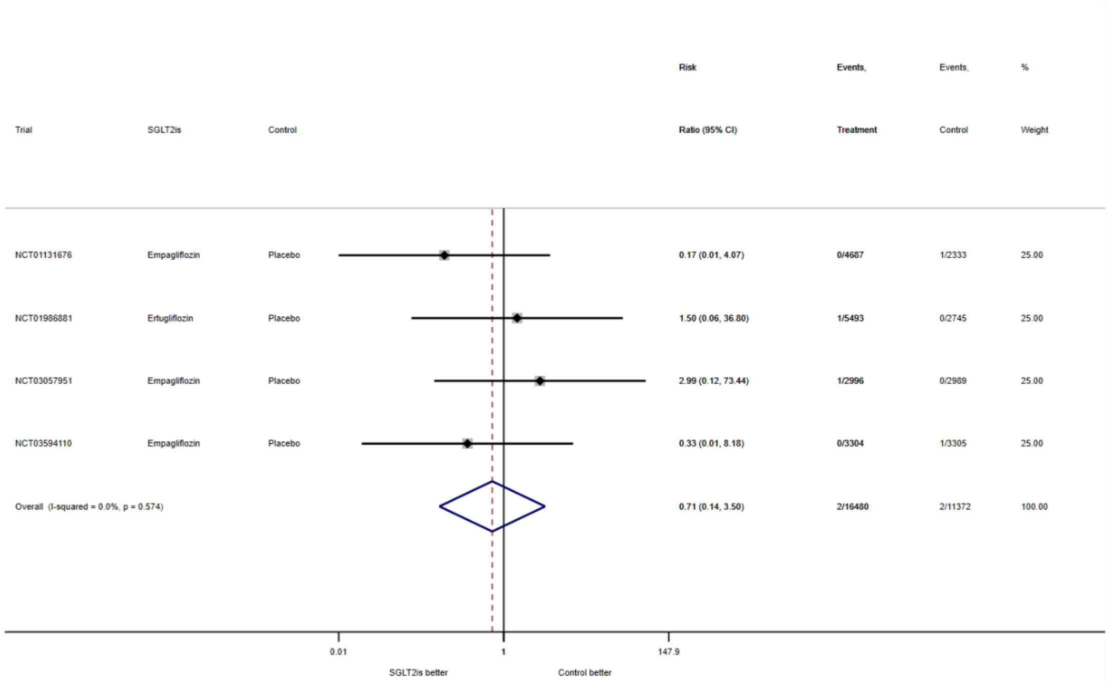

System 11-Infections And Infestations-Figure S52 Meta-analysis of SGLT2is and Gallbladder empyema

Trial	SGLT2is	Control	Risk Ratio (95% CI)	Events, Treatment	Events, Control	% Weight
NCT01031680	Dapagliflozin	Placebo	1.00 (0.06, 16.01)	1/460	1/462	1.04
NCT01032629	Canagliflozin	Placebo	2.62 (0.90, 7.62)	21/2886	4/1441	7.02
NCT01131676	Empagliflozin	Placebo	1.00 (0.43, 2.32)	16/4687	8/2333	11.13
NCT01164501	Empagliflozin	Placebo	2.29 (0.09, 55.92)	1/419	0/319	0.78
NCT01730534	Dapagliflozin	Placebo	0.73 (0.42, 1.27)	22/8574	30/8569	26.49
NCT01986881	Ertugliflozin	Placebo	2.00 (0.92, 4.33)	32/5493	8/2745	13.36
NCT01989754	Canagliflozin	Placebo	2.50 (0.49, 12.87)	5/2904	2/2903	2.98
NCT02065791	Canagliflozin	Placebo	0.67 (0.30, 1.48)	10/2200	15/2197	12.55
NCT03036124	Dapagliflozin	Placebo	0.33 (0.07, 1.65)	2/2368	6/2368	3.12
NCT03036150	Dapagliflozin	Placebo	2.33 (0.60, 9.01)	7/2149	3/2149	4.38
NCT03057951	Empagliflozin	Placebo	1.00 (0.25, 3.99)	4/2996	4/2989	4.17
NCT03057977	Empagliflozin	Placebo	4.00 (0.45, 35.75)	4/1863	1/1863	1.67
NCT03242252	Sotagliflozin	Placebo	0.10 (0.00, 2.05)	0/527	2/260	0.87
NCT03315143	Sotagliflozin	Placebo	0.37 (0.10, 1.41)	3/5291	8/5286	4.54
NCT03521934	Sotagliflozin	Placebo	5.05 (0.24, 104.96)	2/605	0/611	0.87
NCT03594110	Empagliflozin	Placebo	0.20 (0.01, 4.17)	0/3304	2/3305	0.87
NCT03619213	Dapagliflozin	Placebo	1.00 (0.25, 4.00)	4/3126	4/3127	4.17
Overall (I-squared = 24.7%, p = 0.169)			1.02 (0.77, 1.36)	134/49852	98/42927	100.00

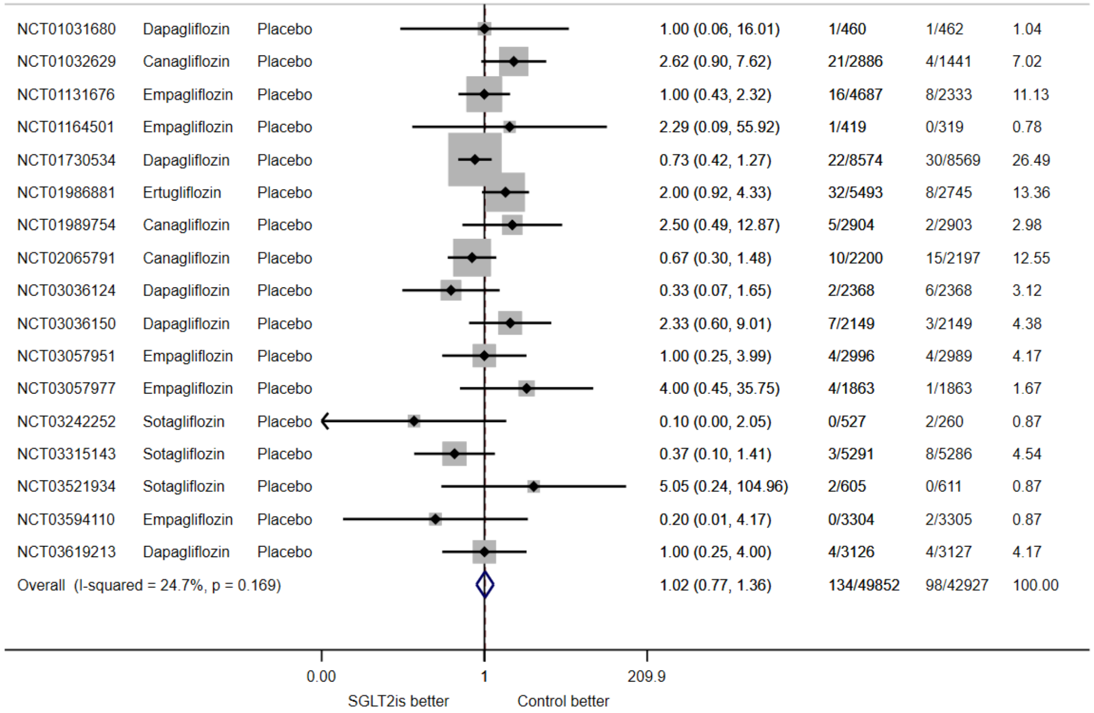

System 11-Infections And Infestations-Figure S53 Meta-analysis of SGLT2is and Gangrene

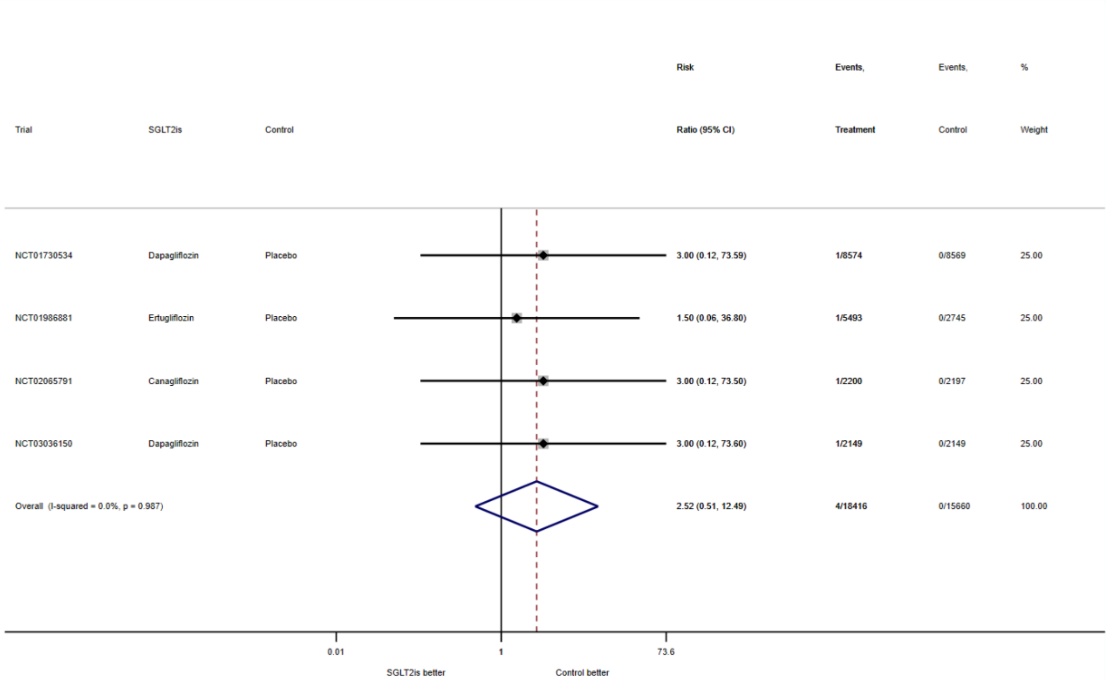

System 11-Infections And Infestations-Figure S54 Meta-analysis of SGLT2is and Gas gangrene

Trial	SGLT2is	Control	Risk Ratio (95% CI)	Events, Treatment	Events, Control	% Weight
NCT01031680	Dapagliflozin	Placebo	2.01 (0.18, 22.08)	2/460	1/462	0.95
NCT01032629	Canagliflozin	Placebo	1.08 (0.41, 2.84)	13/2886	6/1441	5.88
NCT01106651	Canagliflozin	Placebo	1.49 (0.06, 36.53)	1/477	0/237	0.54
NCT01106677	Canagliflozin	Sitagliptin	0.50 (0.03, 7.94)	1/735	1/366	0.71
NCT01131676	Empagliflozin	Placebo	1.10 (0.60, 2.01)	33/4687	15/2333	14.81
NCT01730534	Dapagliflozin	Placebo	0.96 (0.56, 1.65)	26/8574	27/8569	18.95
NCT01986881	Ertugliflozin	Placebo	0.90 (0.30, 2.68)	9/5493	5/2745	4.59
NCT01989754	Canagliflozin	Placebo	0.89 (0.34, 2.30)	8/2904	9/2903	6.06
NCT02033889	Ertugliflozin	Placebo/Glimepiride	0.17 (0.01, 4.14)	0/412	1/209	0.54
NCT02065791	Canagliflozin	Placebo	0.32 (0.13, 0.79)	6/2200	19/2197	6.53
NCT03036124	Dapagliflozin	Placebo	0.50 (0.13, 2.00)	3/2368	6/2368	2.86
NCT03036150	Dapagliflozin	Placebo	1.11 (0.45, 2.73)	10/2149	9/2149	6.79
NCT03057951	Empagliflozin	Placebo	1.14 (0.41, 3.14)	8/2996	7/2989	5.34
NCT03057977	Empagliflozin	Placebo	0.44 (0.14, 1.44)	4/1863	9/1863	3.96
NCT03315143	Sotagliflozin	Placebo	1.80 (0.83, 3.89)	18/5291	10/5286	9.19
NCT03521934	Sotagliflozin	Placebo	0.20 (0.01, 4.20)	0/605	2/611	0.60
NCT03594110	Empagliflozin	Placebo	0.80 (0.32, 2.03)	8/3304	10/3305	6.36
NCT03619213	Dapagliflozin	Placebo	0.88 (0.32, 2.41)	7/3126	8/3127	5.34
Overall (I-squared = 0.0%, p = 0.670)			0.91 (0.72, 1.15)	157/50530	145/43160	100.00

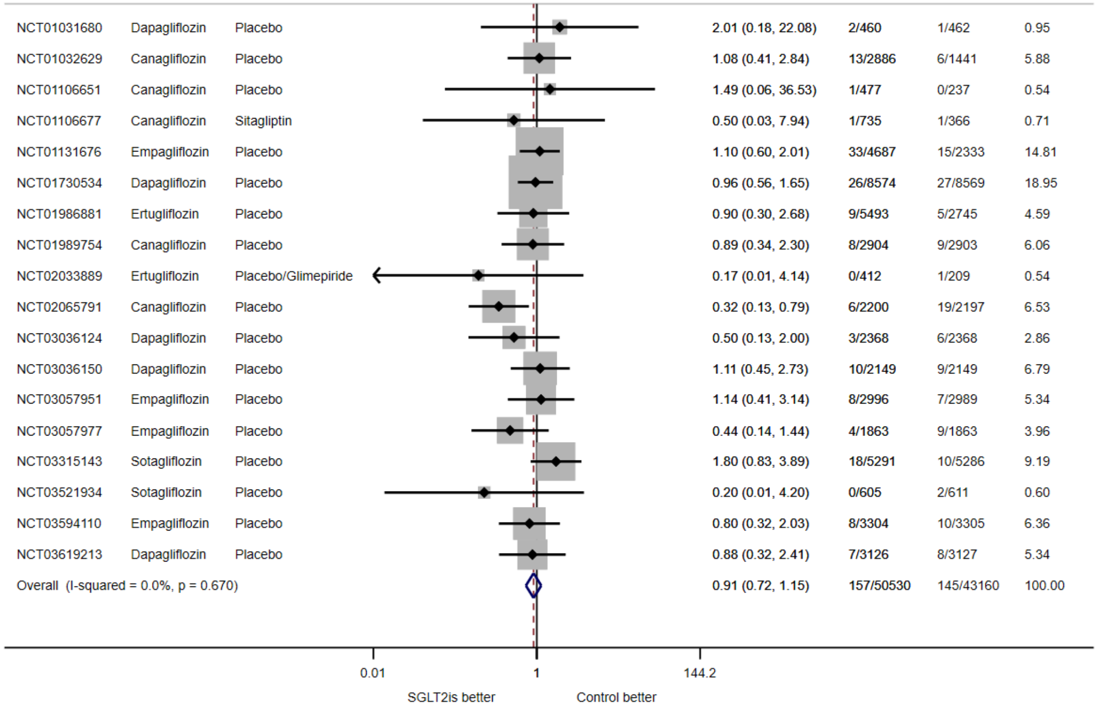

System 11-Infections And Infestations-Figure S55 Meta-analysis of SGLT2is and Gastroenteritis

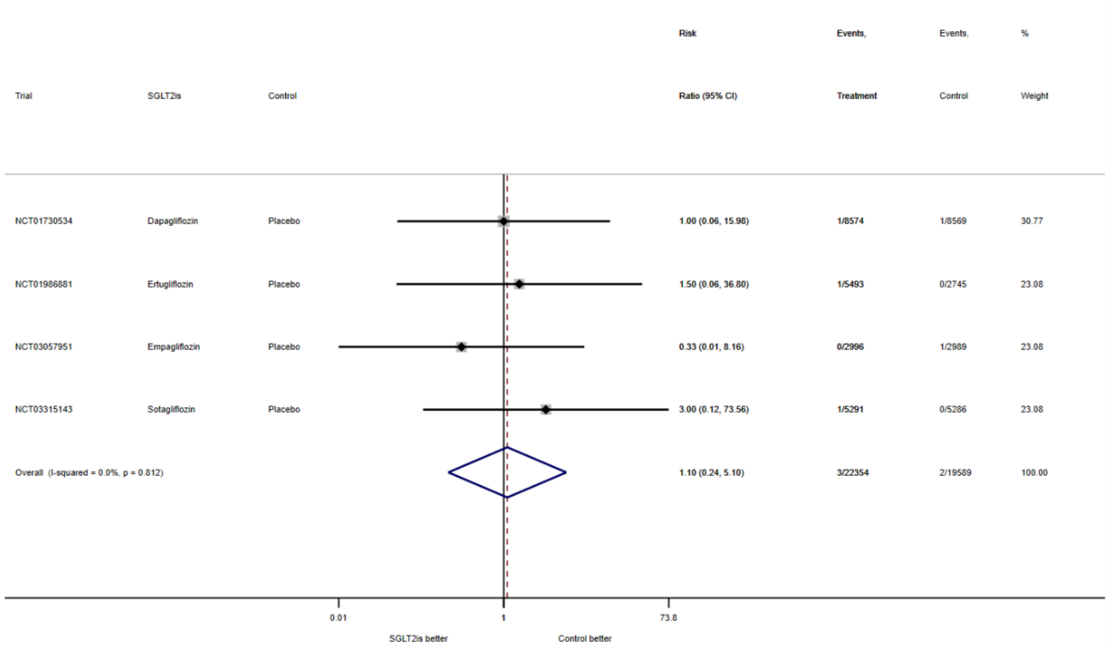

System 11-Infections And Infestations-Figure S56 Meta-analysis of SGLT2is and Gastroenteritis norovirus

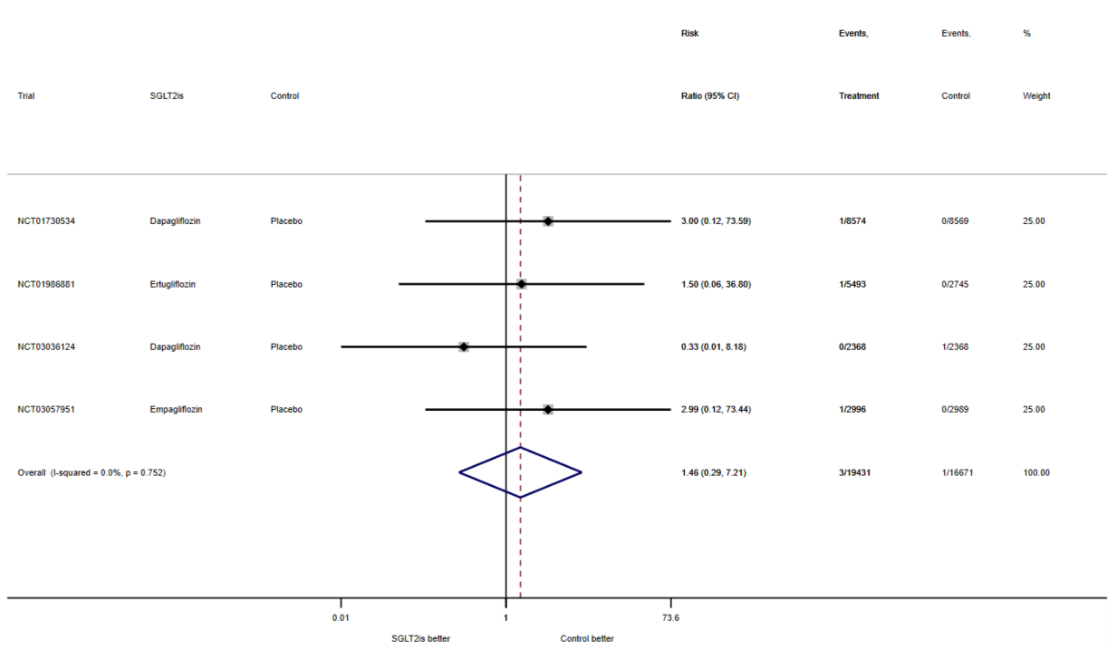

System 11-Infections And Infestations-Figure S57 Meta-analysis of SGLT2is and Gastroenteritis rotavirus

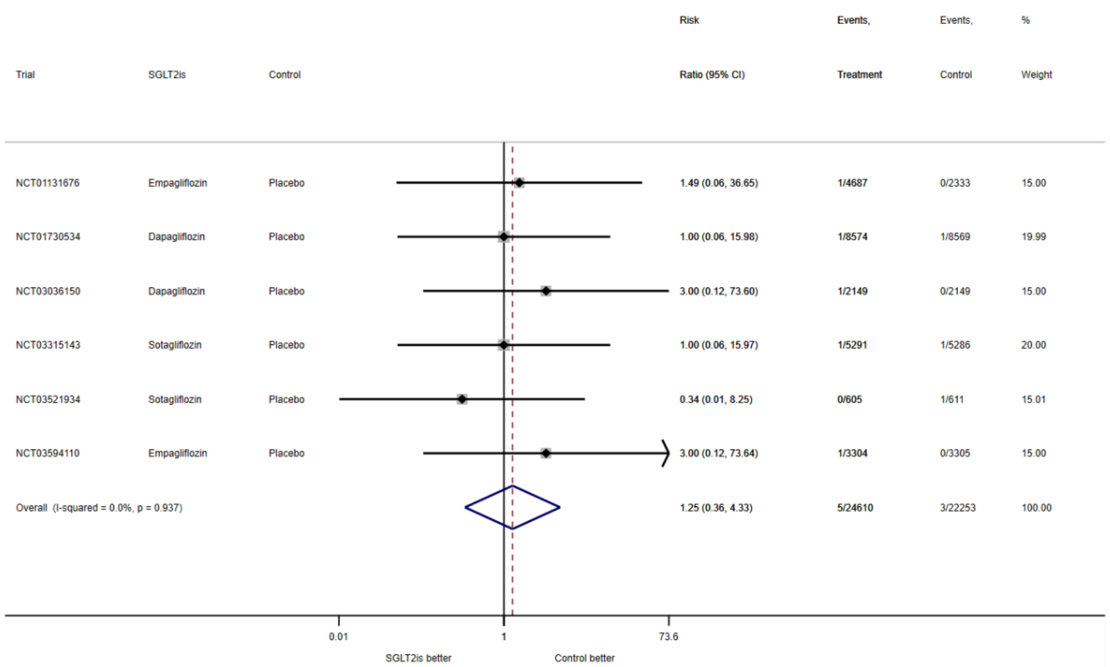

System 11-Infections And Infestations-Figure S58 Meta-analysis of SGLT2is and Gastroenteritis salmonella

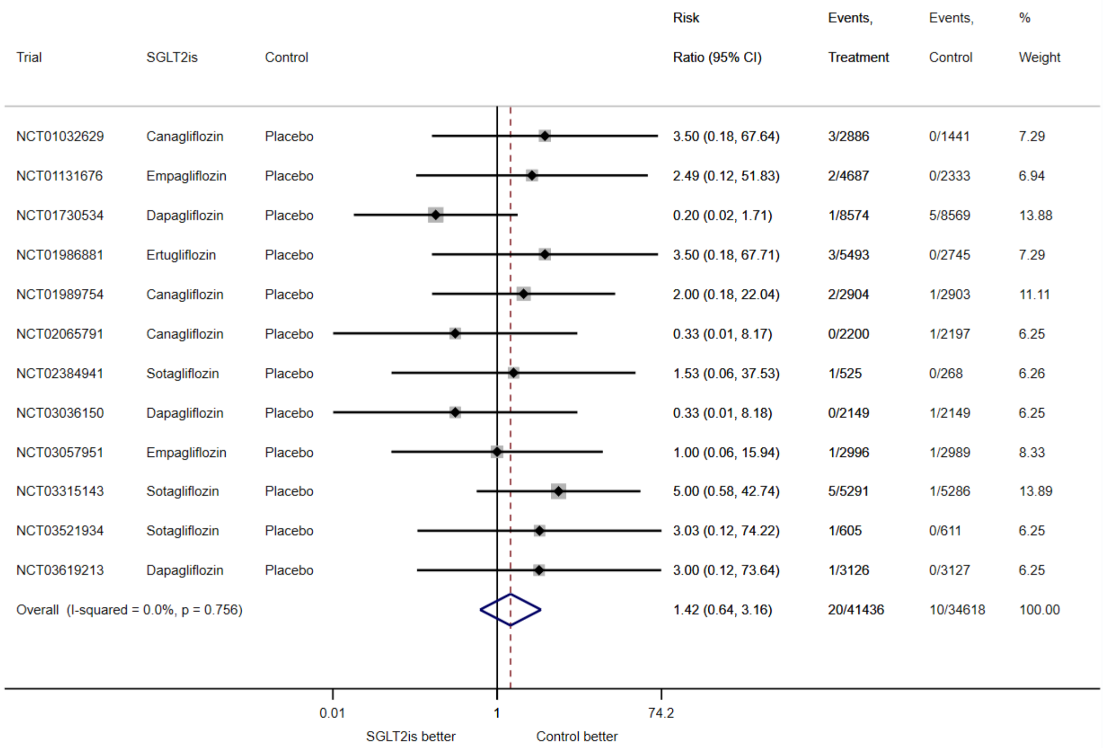

System 11-Infections And Infestations-Figure S59 Meta-analysis of SGLT2is and Gastroenteritis viral

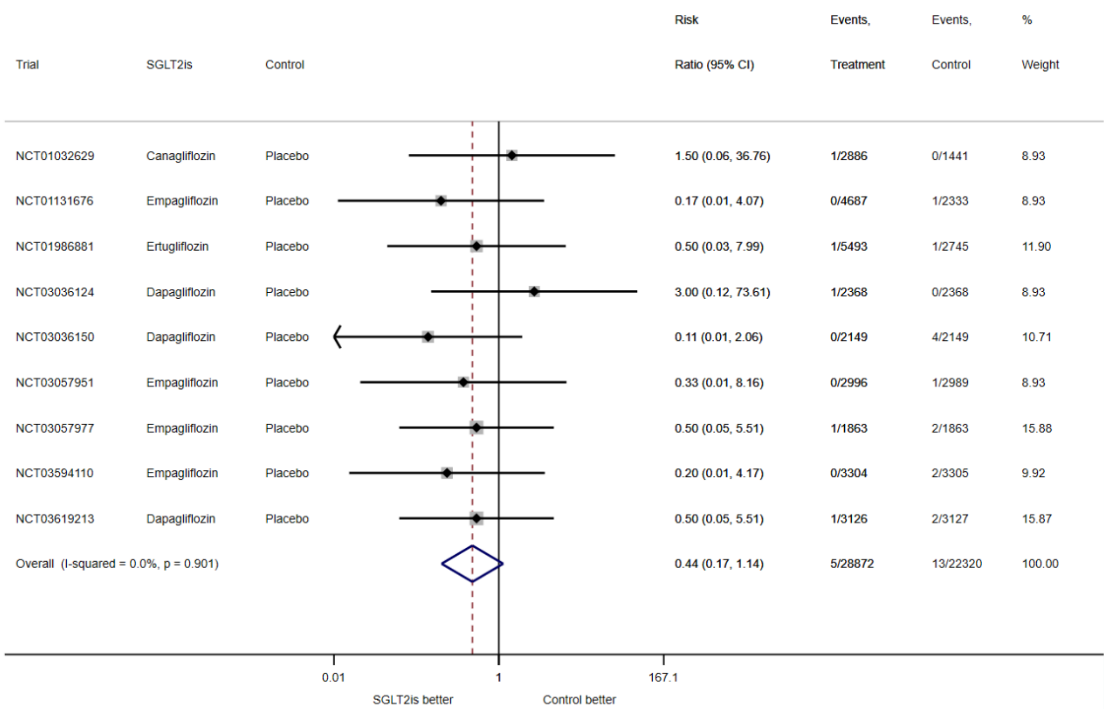

System 11-Infections And Infestations-Figure S60 Meta-analysis of SGLT2is and Gastrointestinal infection

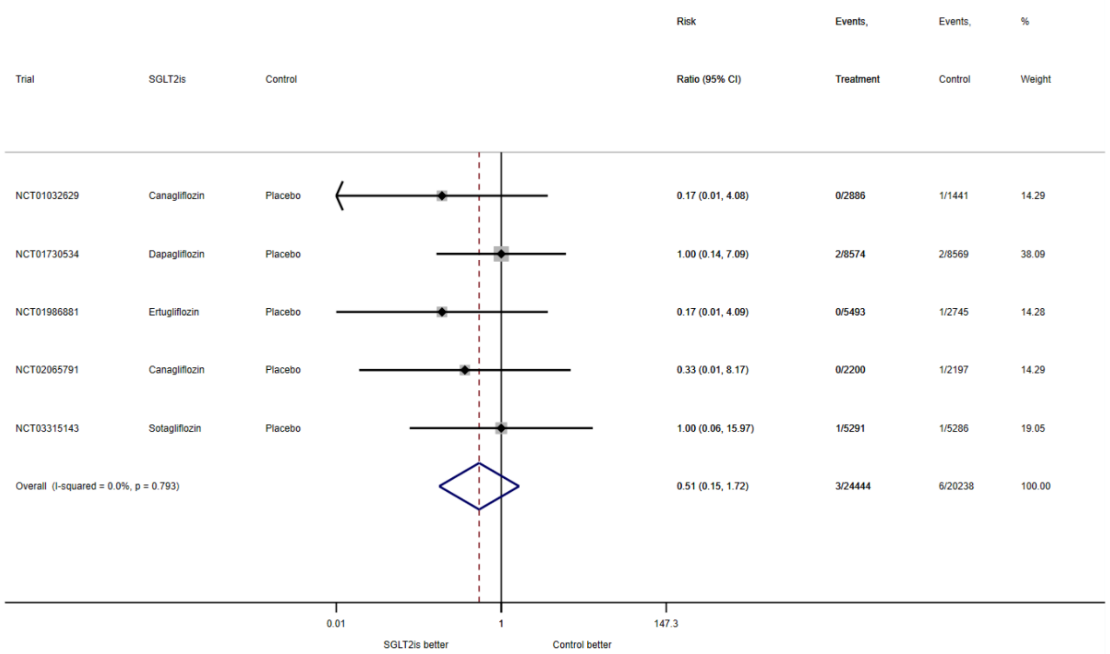

System 11-Infections And Infestations-Figure S61 Meta-analysis of SGLT2is and Groin abscess
