# Supplementary material for: A comprehensive meta-analysis on safety outcomes reveals the novel potentials of SGLT2is, especially preventing respiratory diseases
Source: Front Endocrinol (Lausanne). 2024 Apr 29;15:1376446. doi: 10.3389/fendo.2024.1376446 (PMC11089104; doi:10.3389/fendo.2024.1376446)
Supplement: Supplementary file 3 [file DataSheet_3.pdf]

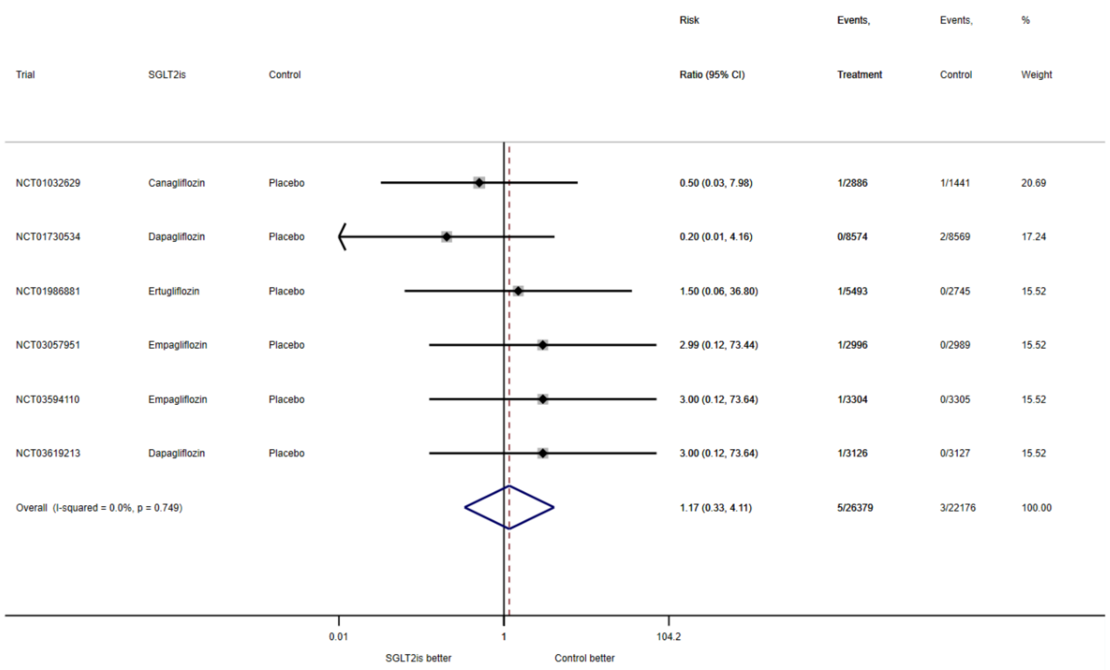

System 11-Infections And Infestations-Figure S62 Meta-analysis of SGLT2is and Haematoma infection

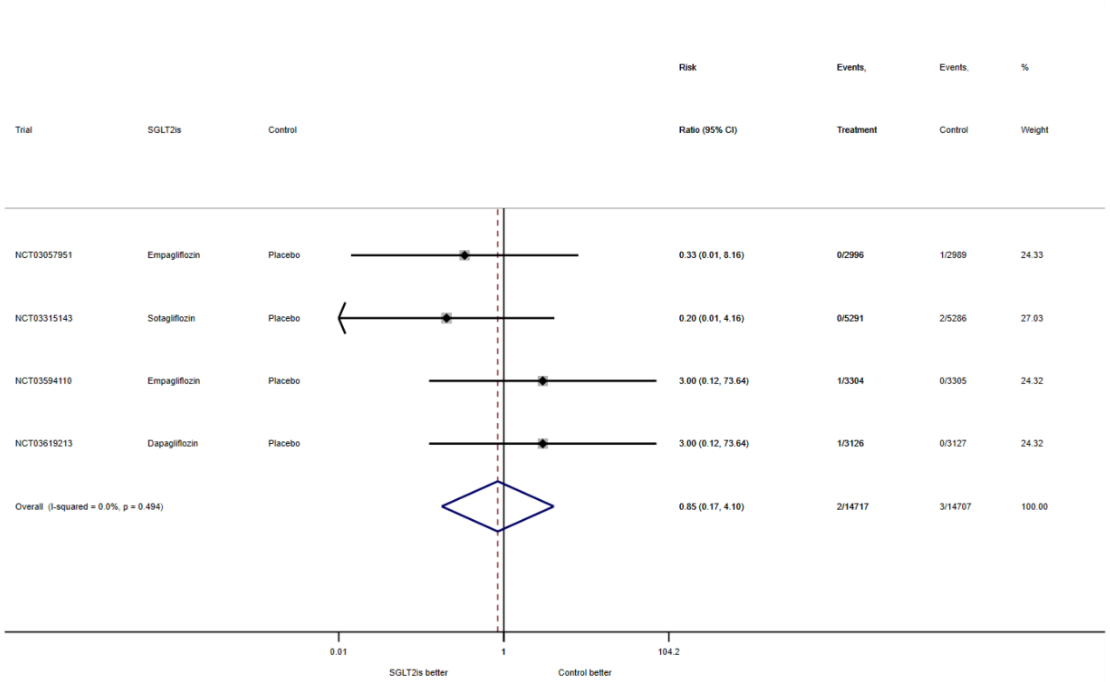

System 11-Infections And Infestations-Figure S63 Meta-analysis of SGLT2is and Helicobacter gastritis

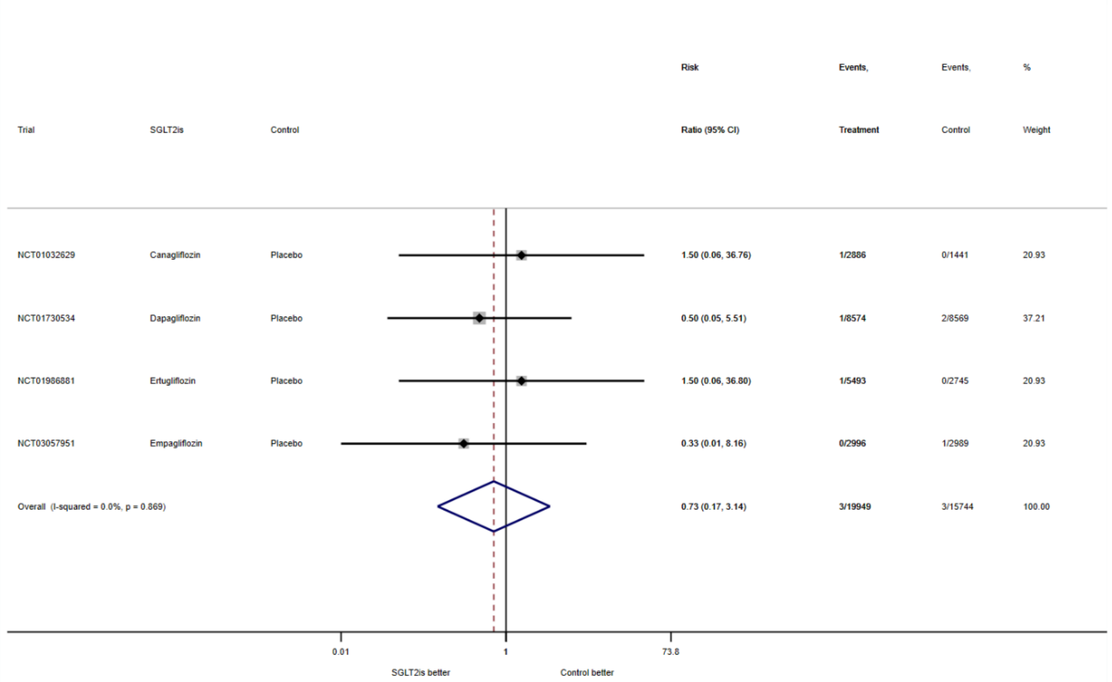

System 11-Infections And Infestations-Figure S64 Meta-analysis of SGLT2is and Helicobacter infection

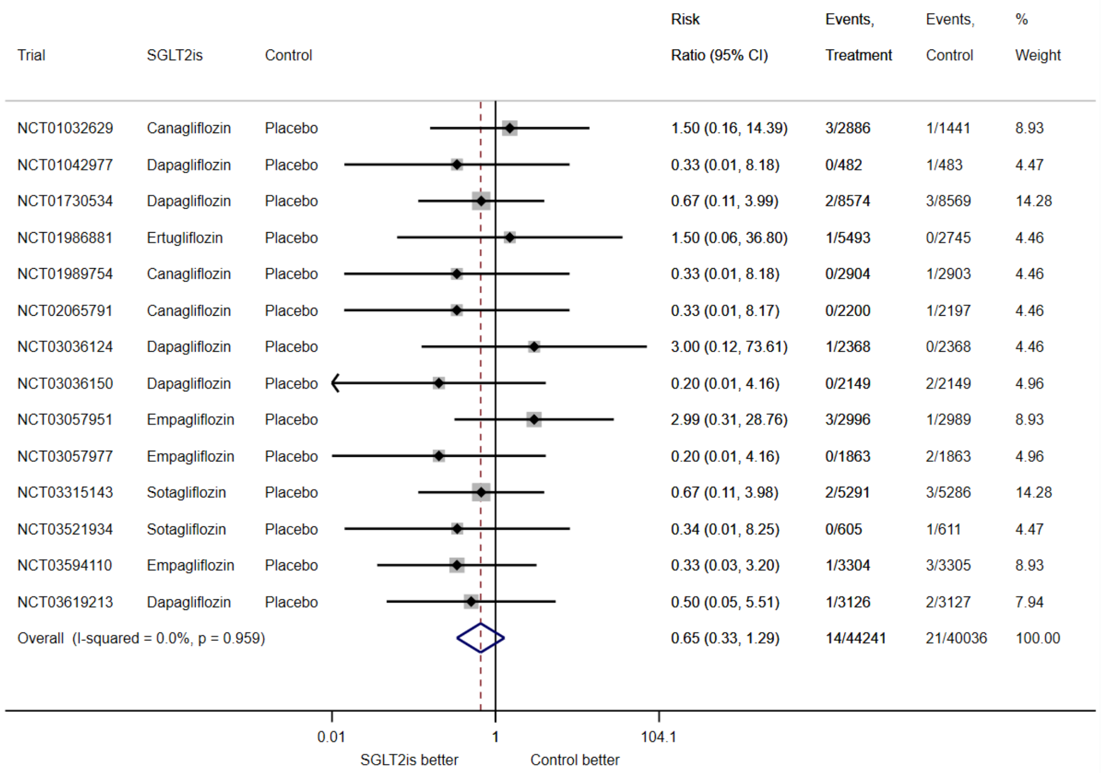

System 11-Infections And Infestations-Figure S65 Meta-analysis of SGLT2is and Herpes zoster

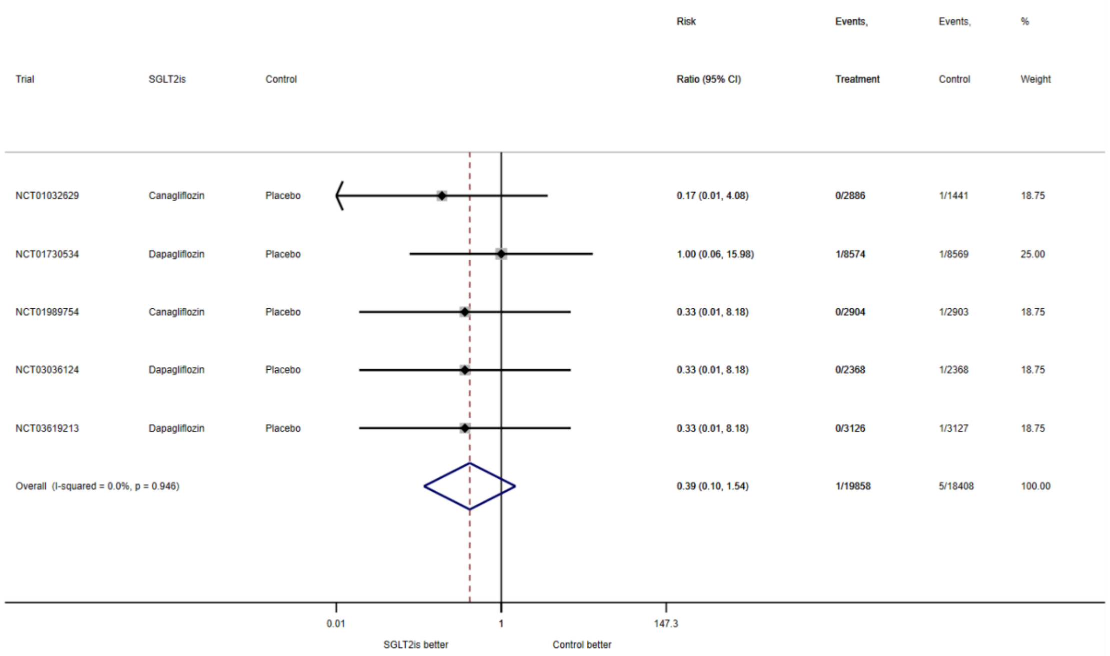

System 11-Infections And Infestations-Figure S66 Meta-analysis of SGLT2is and Hiv infection

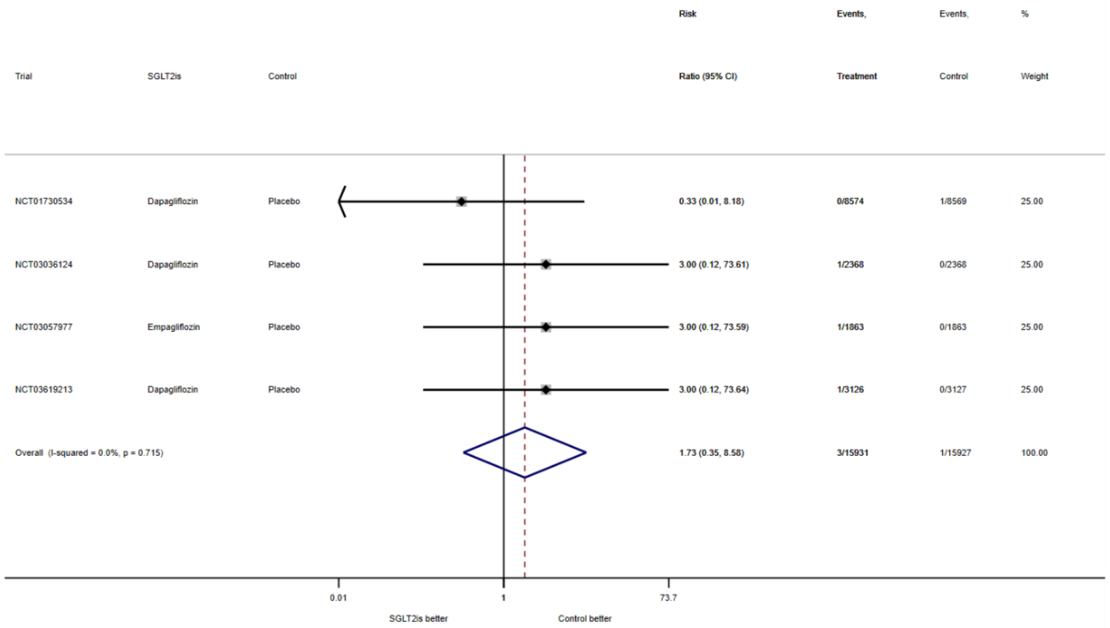

System 11-Infections And Infestations-Figure S67 Meta-analysis of SGLT2is and Implant site infection

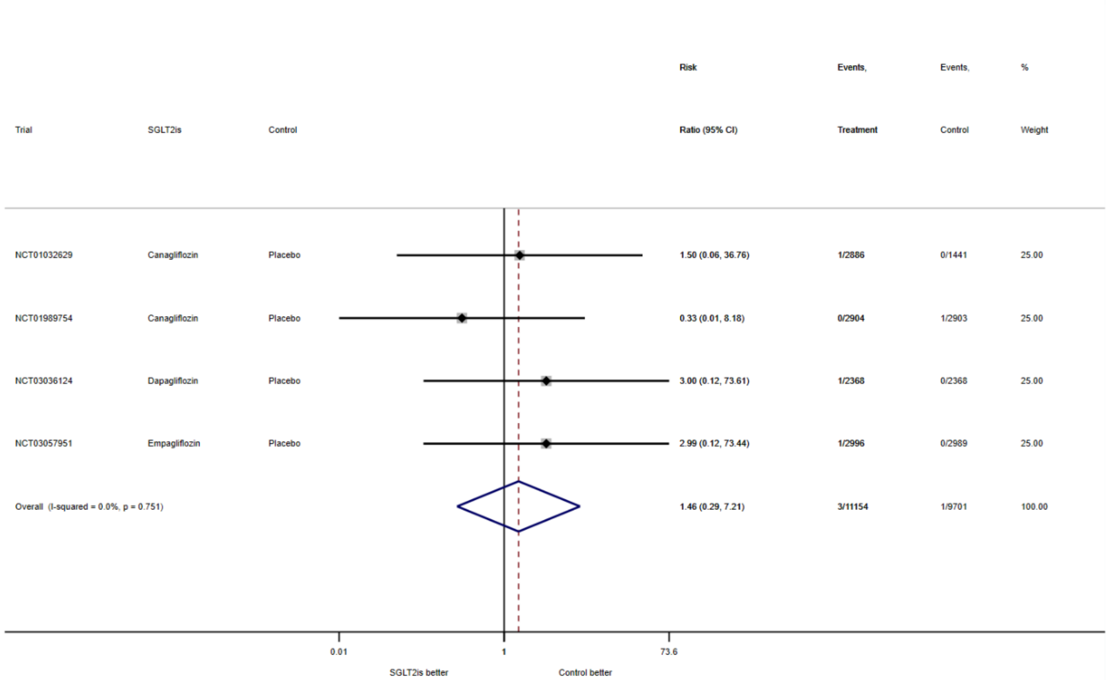

System 11-Infections And Infestations-Figure S68 Meta-analysis of SGLT2is and Infected dermal cyst

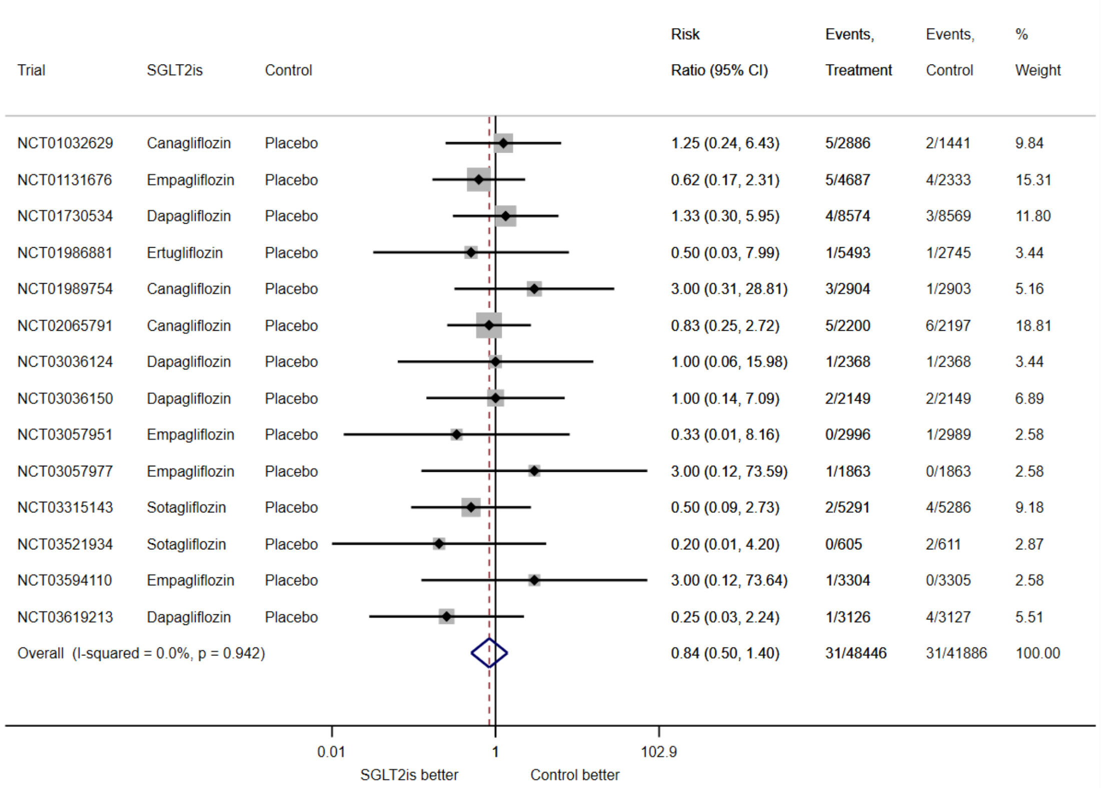

System 11-Infections And Infestations-Figure S69 Meta-analysis of SGLT2is and Infected skin ulcer

Trial	SGLT2is	Control	Risk Ratio (95% CI)	Events, Treatment	Events, Control	% Weight
NCT01730534	Dapagliflozin	Placebo	0.17 (0.02, 1.38)	1/8574	6/8569	14.12
NCT01986881	Ertugliflozin	Placebo	0.50 (0.03, 7.99)	1/5493	1/2745	8.24
NCT01989754	Canagliflozin	Placebo	1.00 (0.06, 15.97)	1/2904	1/2903	8.24
NCT03036124	Dapagliflozin	Placebo	1.00 (0.14, 7.09)	2/2368	2/2368	16.49
NCT03036150	Dapagliflozin	Placebo	3.00 (0.12, 73.60)	1/2149	0/2149	6.18
NCT03057951	Empagliflozin	Placebo	4.99 (0.24, 103.86)	2/2996	0/2989	6.87
NCT03057977	Empagliflozin	Placebo	0.20 (0.01, 4.16)	0/1863	2/1863	6.87
NCT03315143	Sotagliflozin	Placebo	3.00 (0.12, 73.56)	1/5291	0/5286	6.18
NCT03619213	Dapagliflozin	Placebo	0.33 (0.03, 3.20)	1/3126	3/3127	12.36
NCT04157751	Empagliflozin	Placebo	0.34 (0.01, 8.27)	0/260	1/264	6.20
NCT04350593	Dapagliflozin	Placebo	1.00 (0.06, 16.03)	1/613	1/616	8.25
Overall (I-squared = 0.0%, p = 0.768)			0.69 (0.31, 1.52)	11/35637	17/32879	100.00

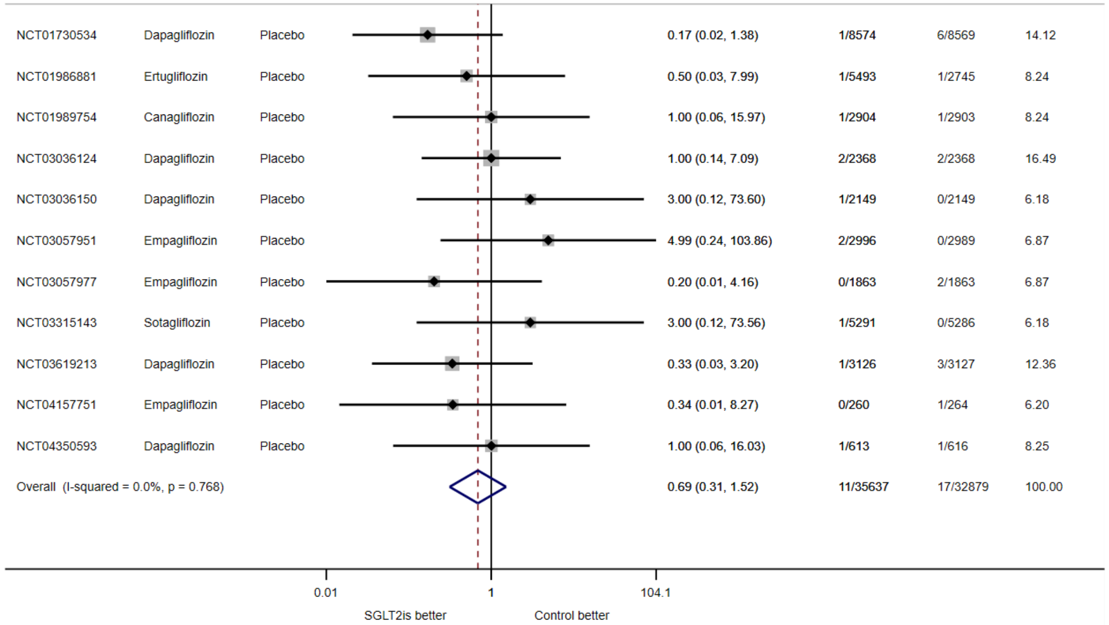

System 11-Infections And Infestations-Figure S70 Meta-analysis of SGLT2is and Infection

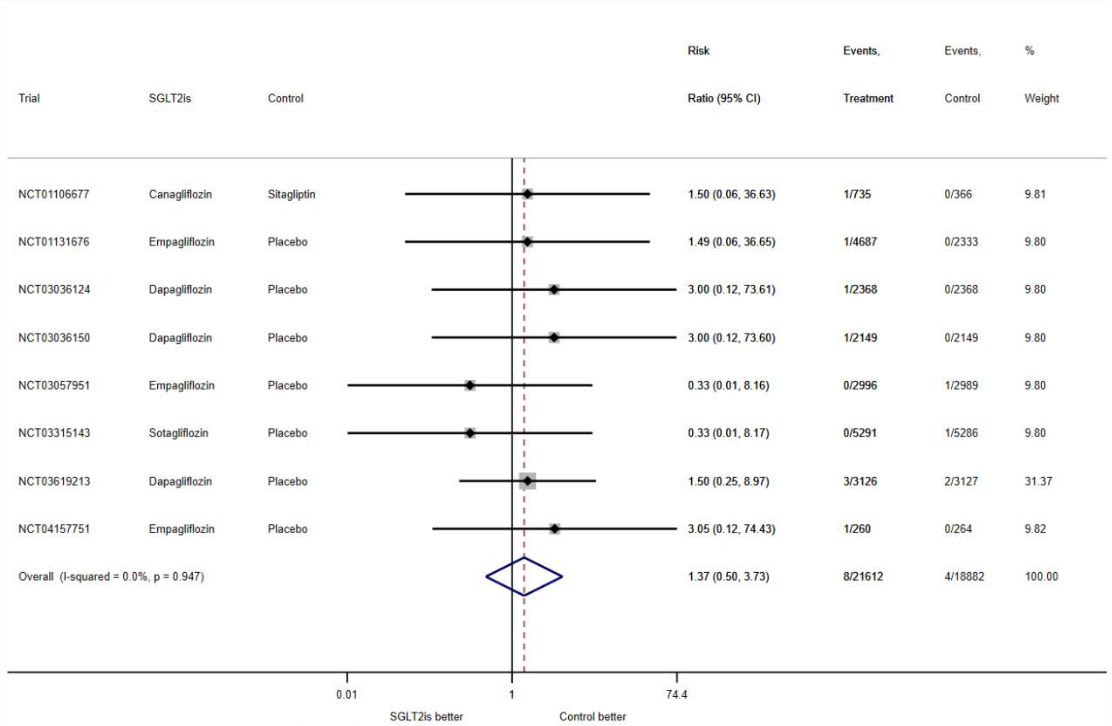

System 11-Infections And Infestations-Figure S71 Meta-analysis of SGLT2is and Infectious pleural effusion

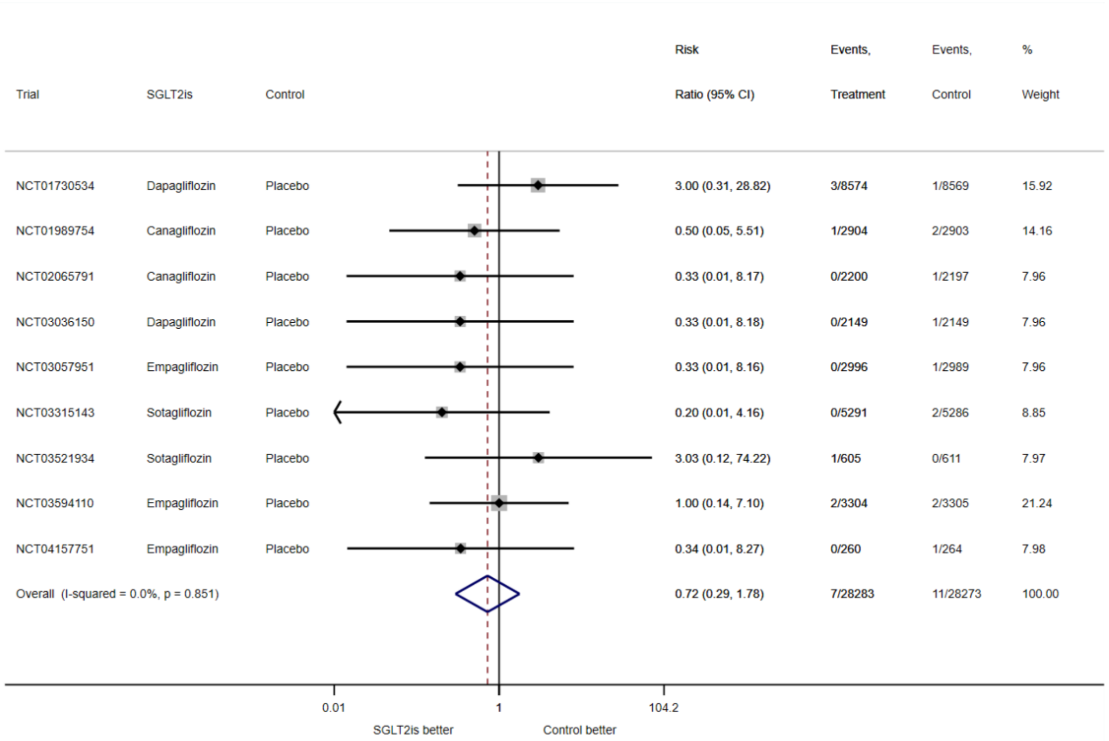

System 11-Infections And Infestations-Figure S72 Meta-analysis of SGLT2is and Infective exacerbation of chronic obstructive airways disease

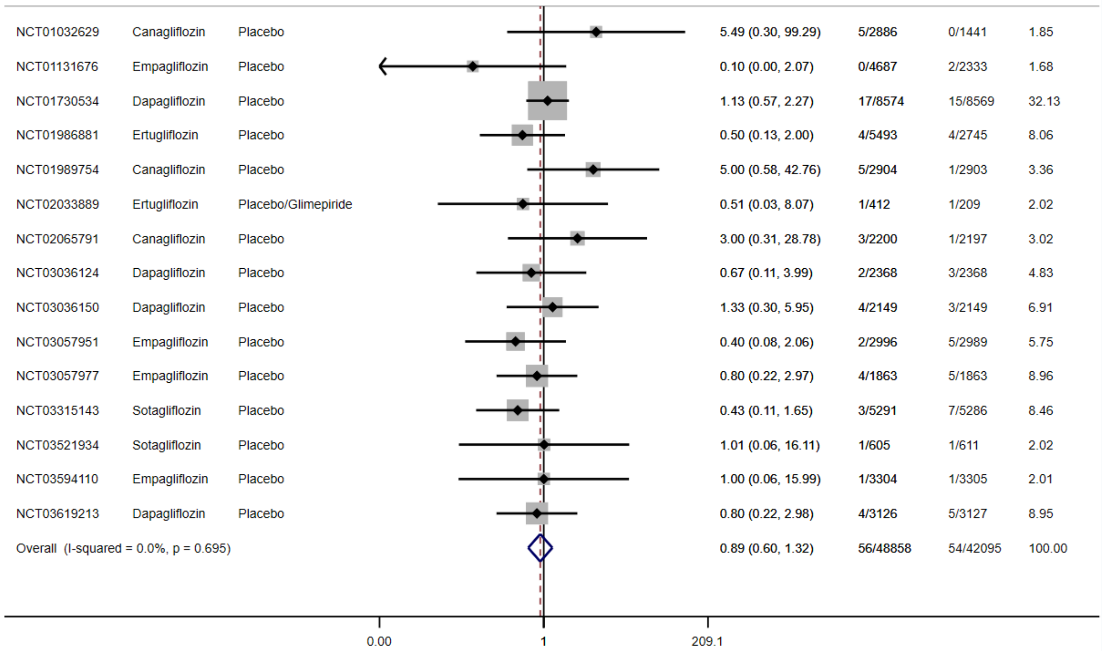

Trial	SGLT2is	Control	Risk Ratio (95% CI)	Events, Treatment	Events, Control	% Weight
NCT01032629	Canagliflozin	Placebo	5.49 (0.30, 99.29)	5/2886	0/1441	1.85
NCT01131676	Empagliflozin	Placebo	0.10 (0.00, 2.07)	0/4687	2/2333	1.68
NCT01730534	Dapagliflozin	Placebo	1.13 (0.57, 2.27)	17/8574	15/8569	32.13
NCT01986881	Ertugliflozin	Placebo	0.50 (0.13, 2.00)	4/5493	4/2745	8.06
NCT01989754	Canagliflozin	Placebo	5.00 (0.58, 42.76)	5/2904	1/2903	3.36
NCT02033889	Ertugliflozin	Placebo/Glimepiride	0.51 (0.03, 8.07)	1/412	1/209	2.02
NCT02065791	Canagliflozin	Placebo	3.00 (0.31, 28.78)	3/2200	1/2197	3.02
NCT03036124	Dapagliflozin	Placebo	0.67 (0.11, 3.99)	2/2368	3/2368	4.83
NCT03036150	Dapagliflozin	Placebo	1.33 (0.30, 5.95)	4/2149	3/2149	6.91
NCT03057951	Empagliflozin	Placebo	0.40 (0.08, 2.06)	2/2996	5/2989	5.75
NCT03057977	Empagliflozin	Placebo	0.80 (0.22, 2.97)	4/1863	5/1863	8.96
NCT03315143	Sotagliflozin	Placebo	0.43 (0.11, 1.65)	3/5291	7/5286	8.46
NCT03521934	Sotagliflozin	Placebo	1.01 (0.06, 16.11)	1/605	1/611	2.02
NCT03594110	Empagliflozin	Placebo	1.00 (0.06, 15.99)	1/3304	1/3305	2.01
NCT03619213	Dapagliflozin	Placebo	0.80 (0.22, 2.98)	4/3126	5/3127	8.95
Overall (I-squared = 0.0%, p = 0.695)			0.89 (0.60, 1.32)	56/48858	54/42095	100.00

System 11-Infections And Infestations-Figure S73 Meta-analysis of SGLT2is and Influenza

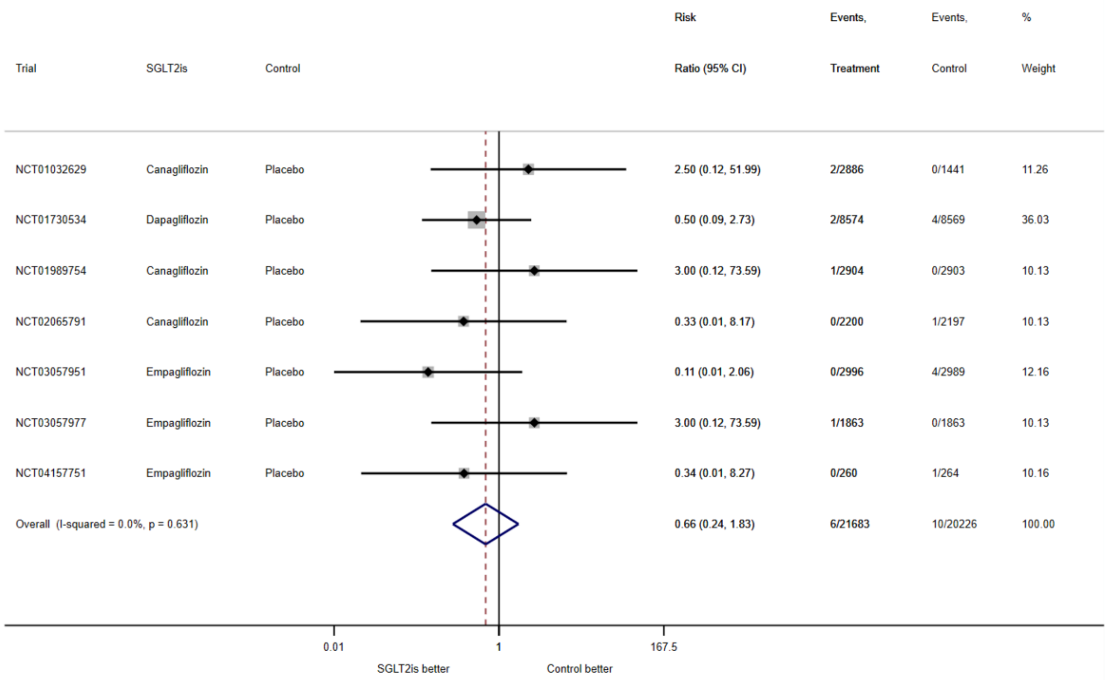

System 11-Infections And Infestations-Figure S74 Meta-analysis of SGLT2is and Intervertebral discitis

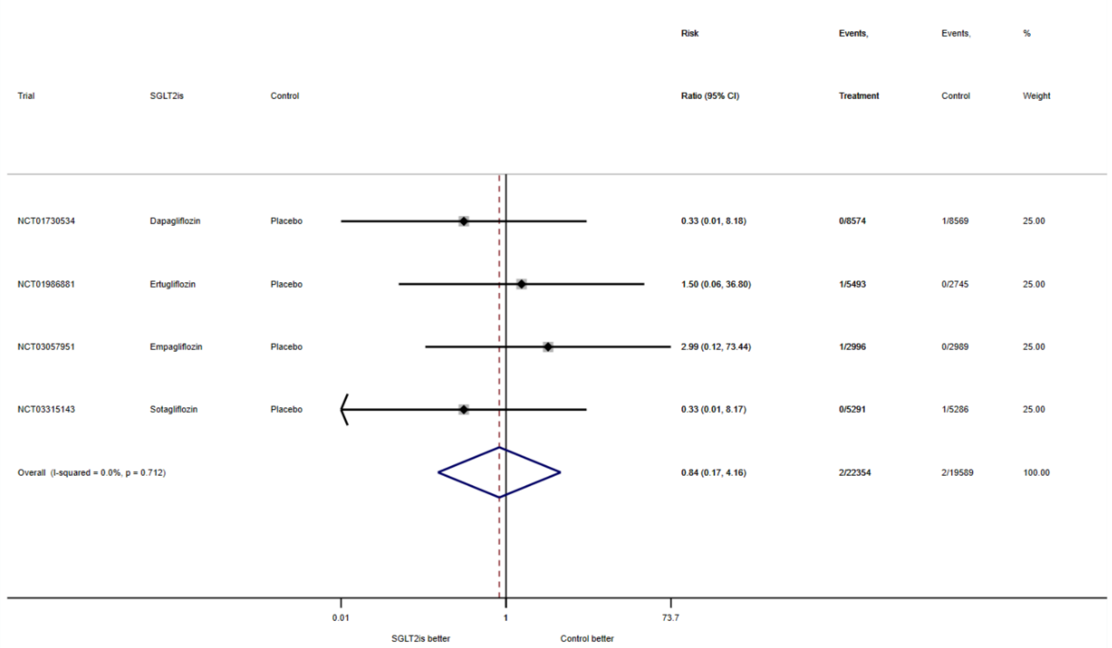

System 11-Infections And Infestations-Figure S75 Meta-analysis of SGLT2is and Klebsiella bacteraemia

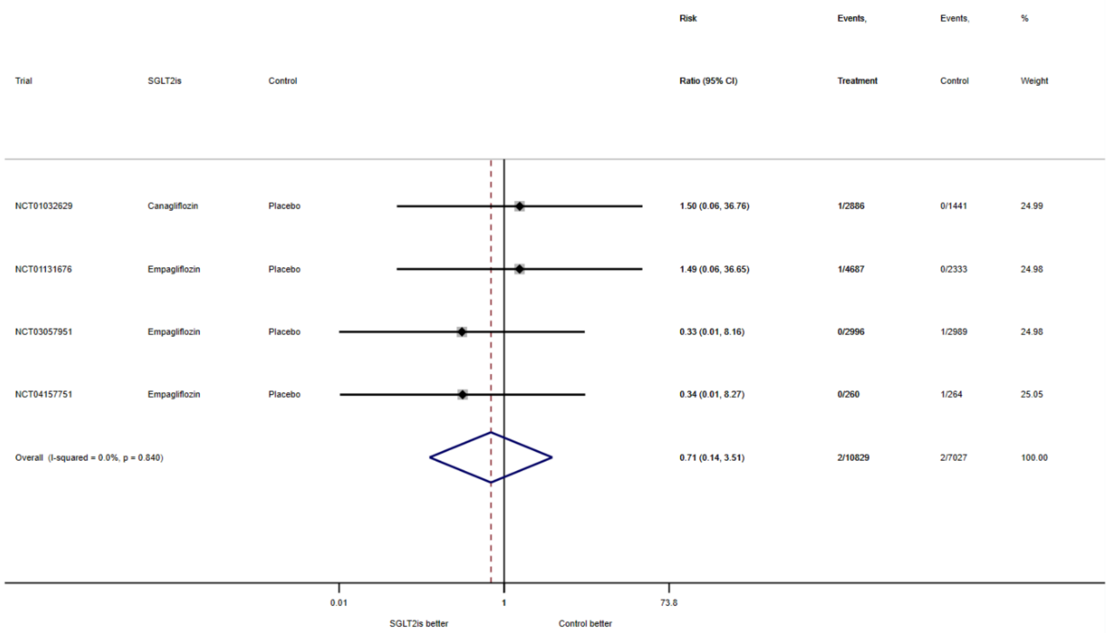

System 11-Infections And Infestations-Figure S76 Meta-analysis of SGLT2is and Klebsiella infection

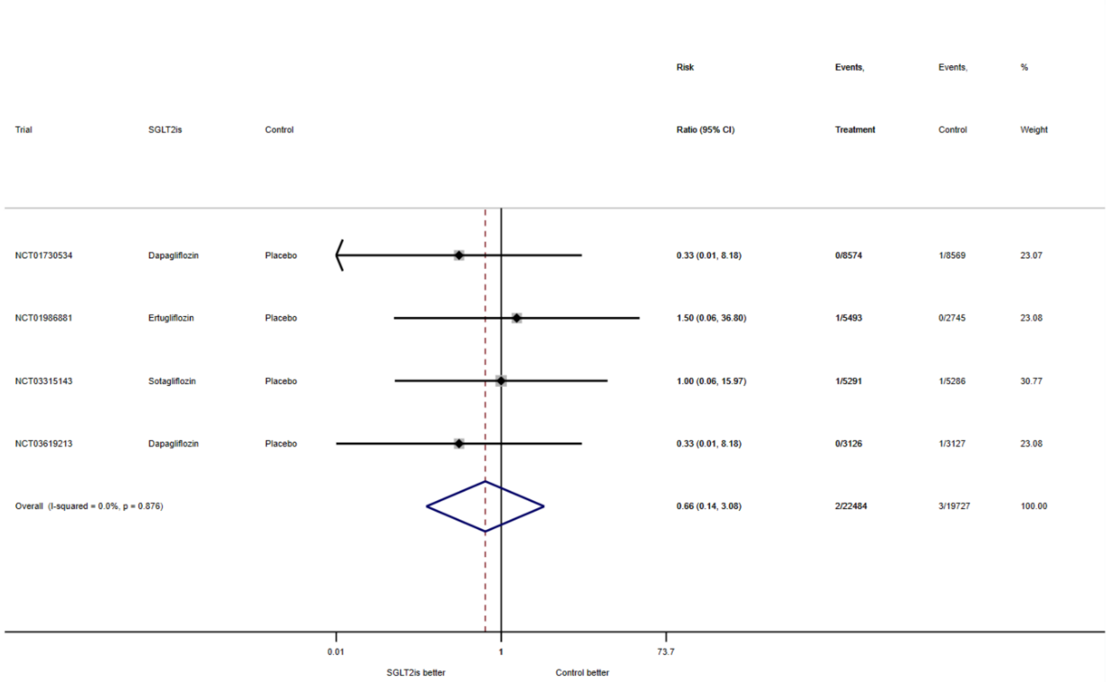

System 11-Infections And Infestations-Figure S77 Meta-analysis of SGLT2is and Klebsiella sepsis

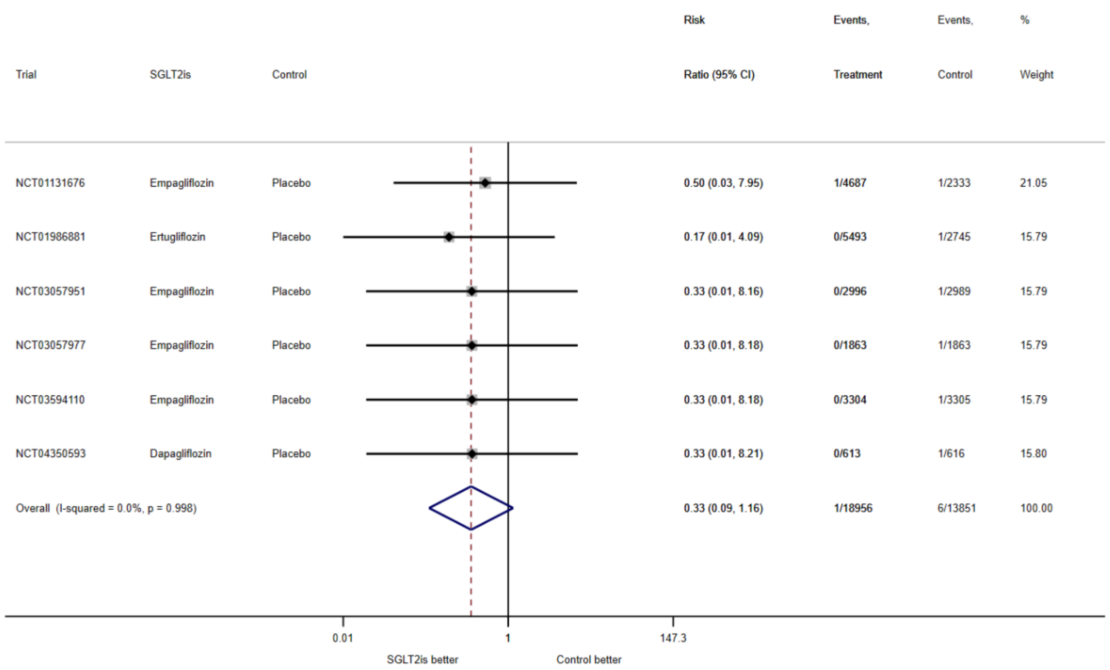

System 11-Infections And Infestations-Figure S78 Meta-analysis of SGLT2is and Labyrinthitis

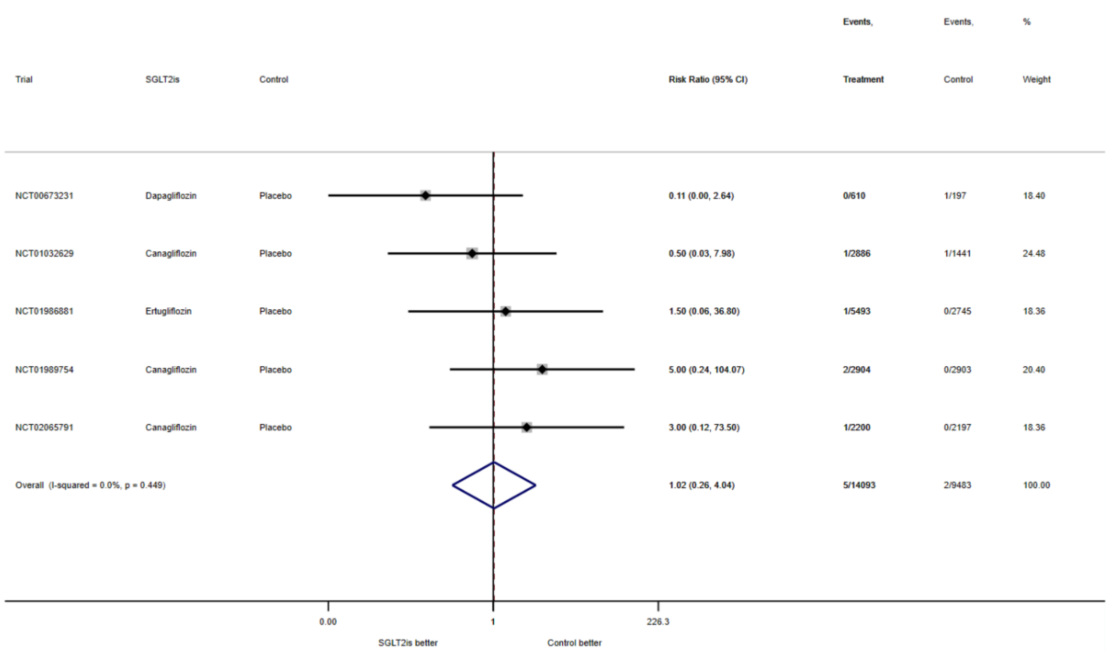

System 11-Infections And Infestations-Figure S79 Meta-analysis of SGLT2is and Laryngitis

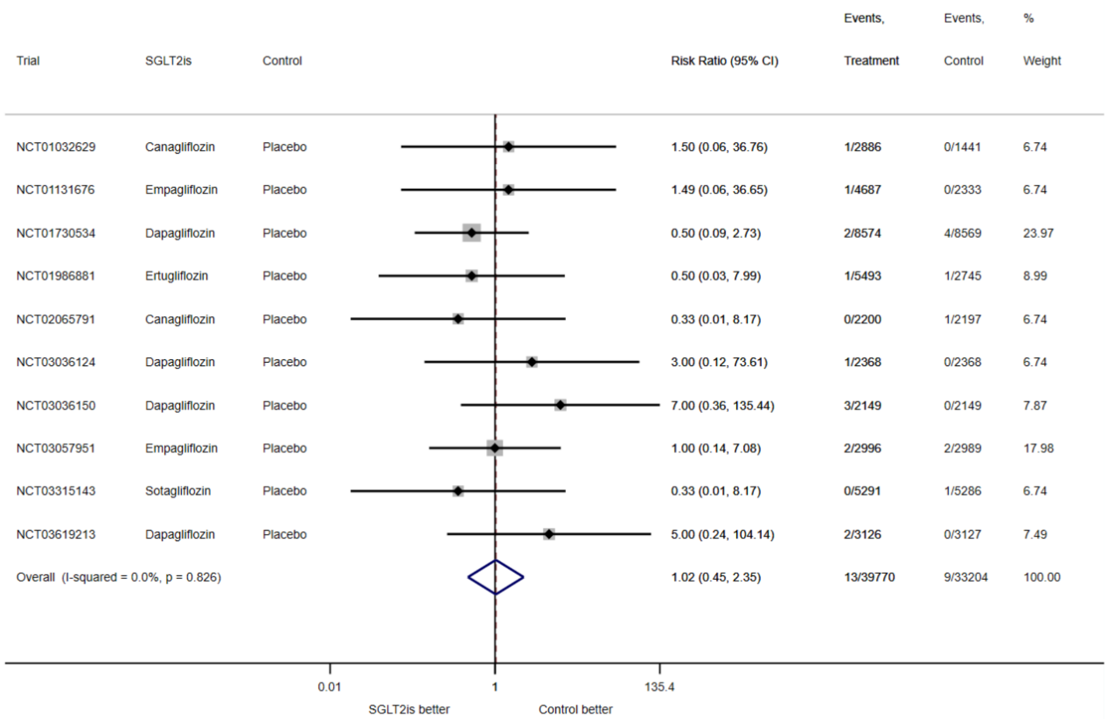

System 11-Infections And Infestations-Figure S80 Meta-analysis of SGLT2is and Liver abscess

Trial	SGLT2is	Control	Risk Ratio (95% CI)	Events, Treatment	Events, Control	% Weight
NCT01031680	Dapagliflozin	Placebo	3.01 (0.12, 73.77)	1/460	0/462	1.60
NCT01032629	Canagliflozin	Placebo	6.99 (0.92, 53.11)	14/2886	1/1441	3.98
NCT01131676	Empagliflozin	Placebo	0.60 (0.18, 1.96)	6/4687	5/2333	11.65
NCT01730534	Dapagliflozin	Placebo	1.60 (0.73, 3.52)	16/8574	10/8569	26.27
NCT01986881	Ertugliflozin	Placebo	1.50 (0.30, 7.42)	6/5493	2/2745	6.40
NCT01989754	Canagliflozin	Placebo	0.83 (0.25, 2.73)	5/2904	6/2903	11.65
NCT02065791	Canagliflozin	Placebo	1.00 (0.32, 3.09)	6/2200	6/2197	12.82
NCT03036124	Dapagliflozin	Placebo	3.00 (0.12, 73.61)	1/2368	0/2368	1.60
NCT03036150	Dapagliflozin	Placebo	0.38 (0.10, 1.41)	3/2149	8/2149	9.32
NCT03057951	Empagliflozin	Placebo	1.50 (0.25, 8.95)	3/2996	2/2989	5.12
NCT03057977	Empagliflozin	Placebo	0.20 (0.01, 4.16)	0/1863	2/1863	1.78
NCT03315143	Sotagliflozin	Placebo	5.00 (0.24, 104.02)	2/5291	0/5286	1.78
NCT03594110	Empagliflozin	Placebo	0.50 (0.05, 5.51)	1/3304	2/3305	2.84
NCT03619213	Dapagliflozin	Placebo	3.00 (0.31, 28.83)	3/3126	1/3127	3.20
Overall (I-squared = 0.0%, p = 0.513)			1.13 (0.76, 1.70)	67/48301	45/41737	100.00

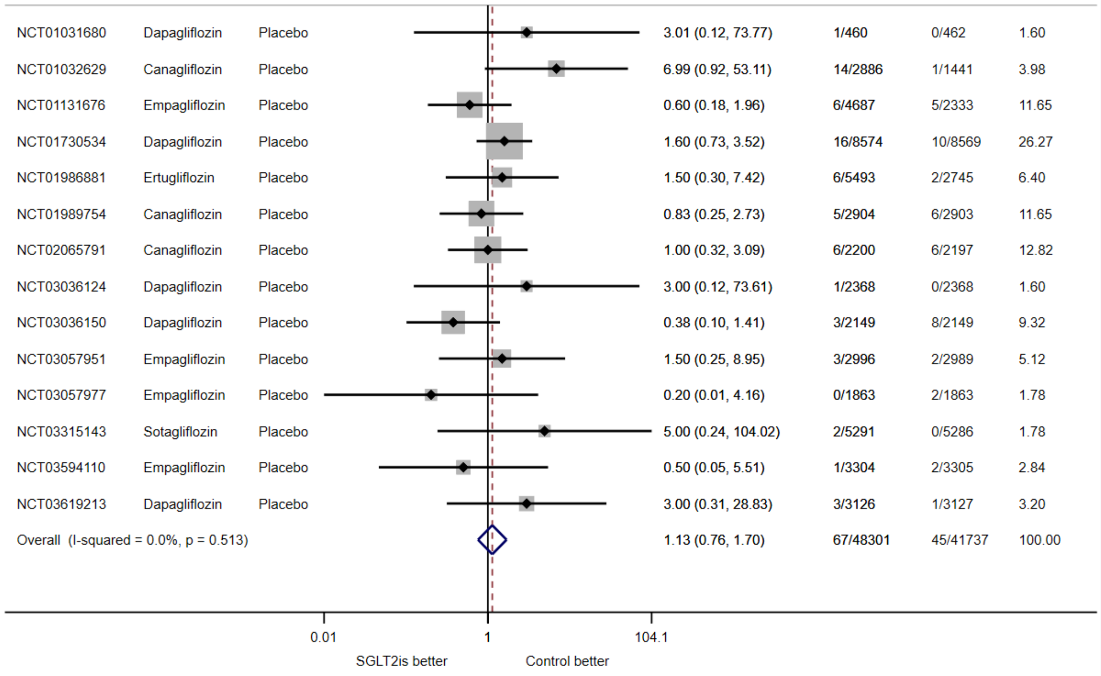

System 11-Infections And Infestations-Figure S81 Meta-analysis of SGLT2is and Localised infection

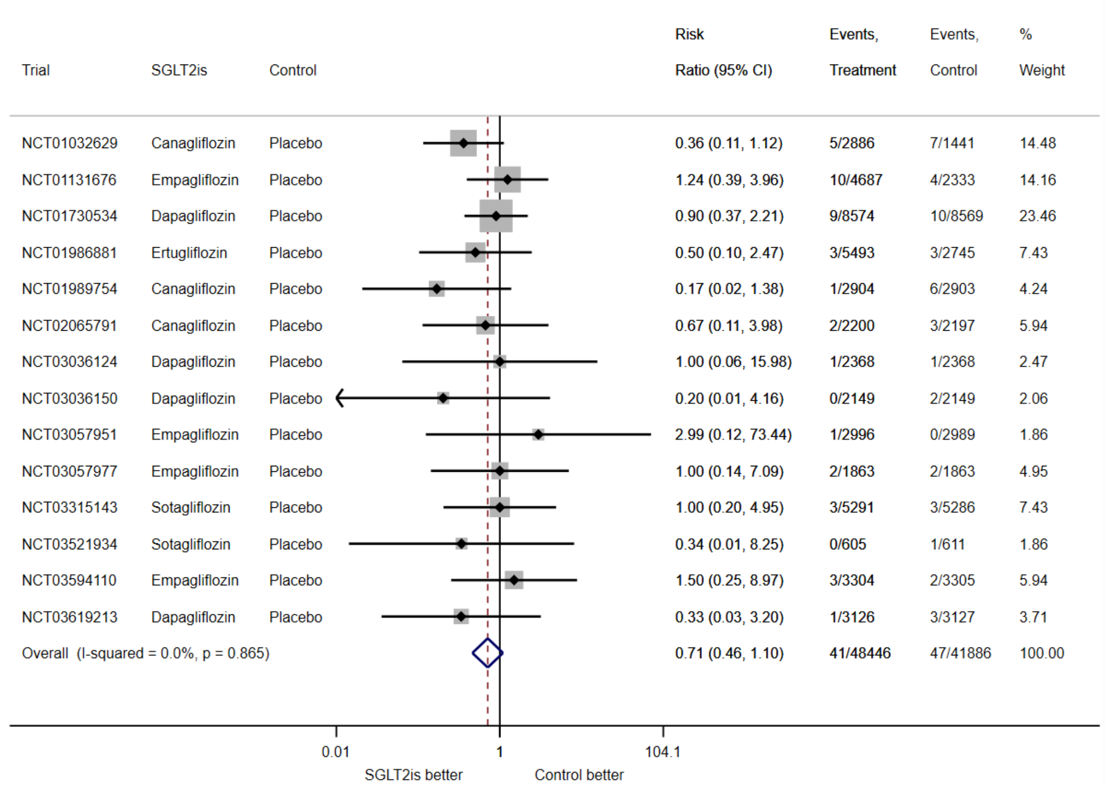

System 11-Infections And Infestations-Figure S82 Meta-analysis of SGLT2is and Lower respiratory tract infection

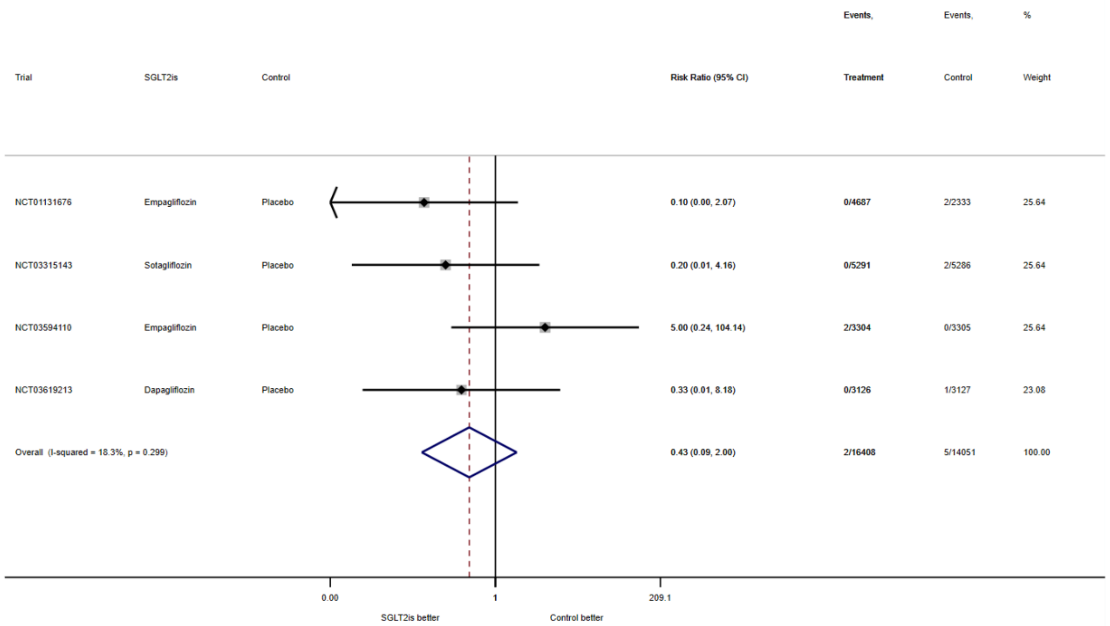

System 11-Infections And Infestations-Figure S83 Meta-analysis of SGLT2is and Lower respiratory tract infection bacterial

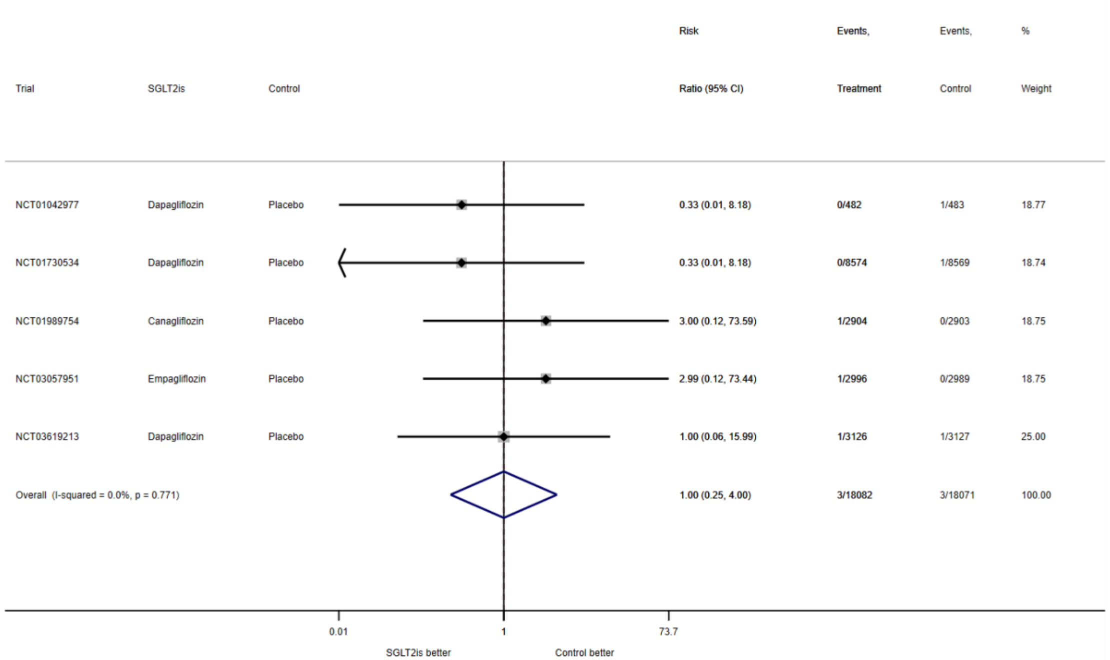

System 11-Infections And Infestations-Figure S84 Meta-analysis of SGLT2is and Lung abscess

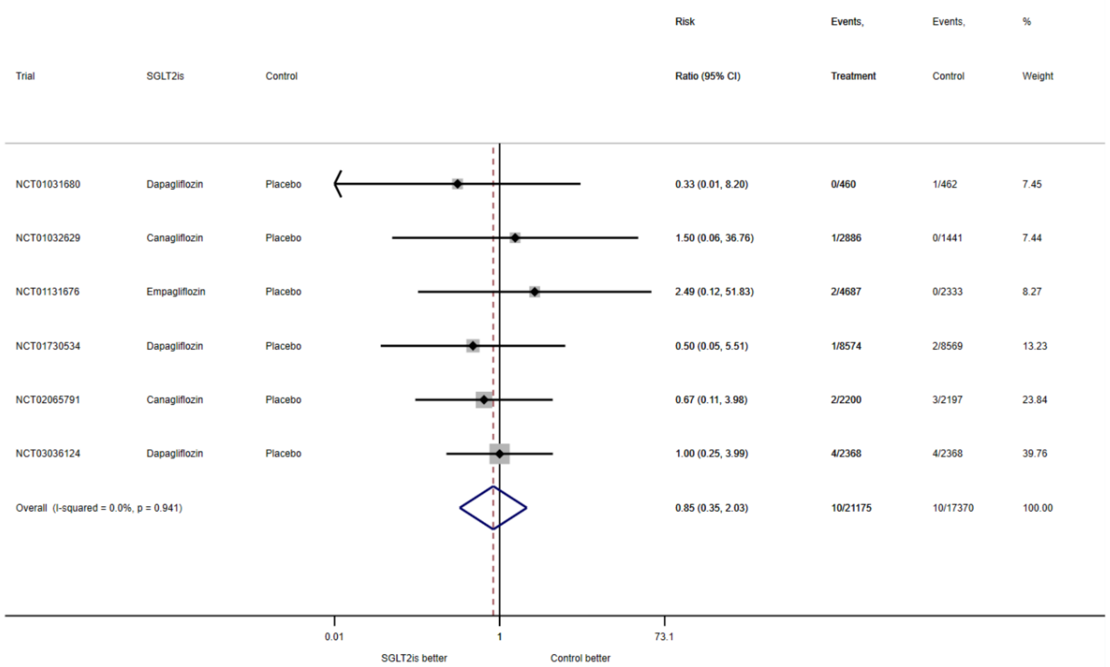

System 11-Infections And Infestations-Figure S85 Meta-analysis of SGLT2is and Lung infection

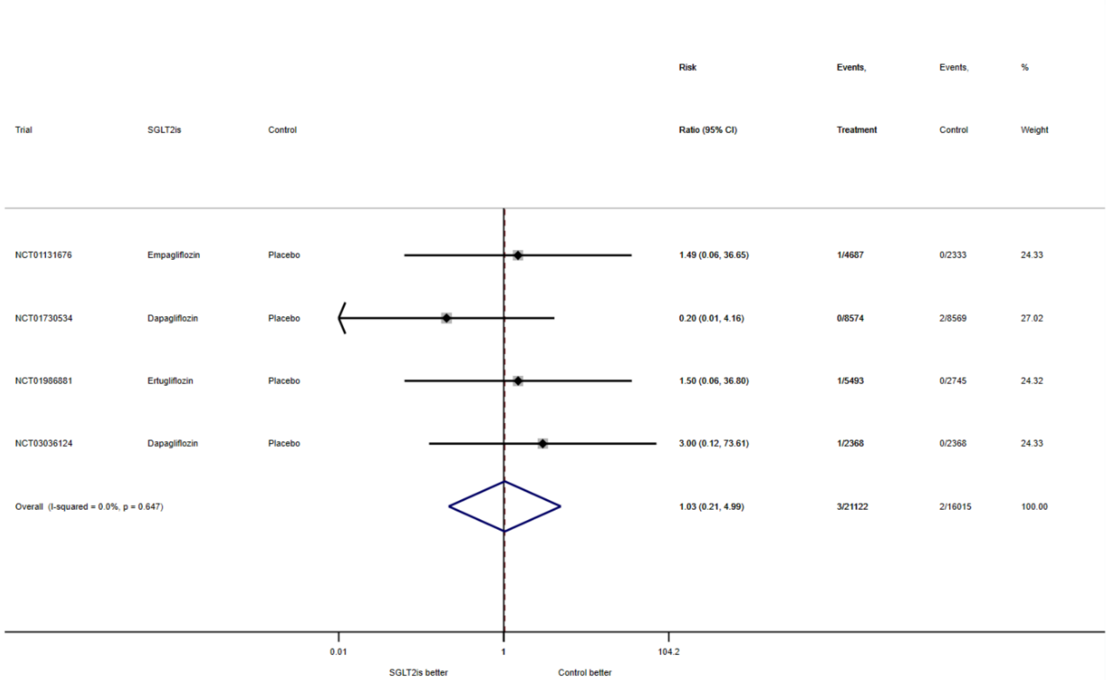

System 11-Infections And Infestations-Figure S86 Meta-analysis of SGLT2is and Lyme disease

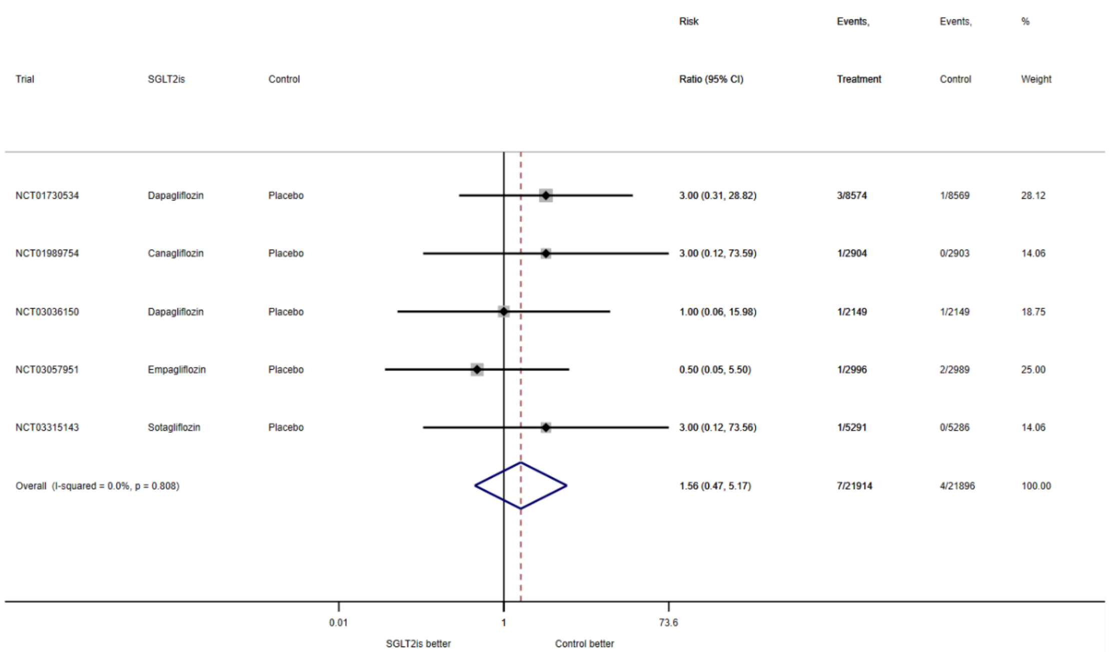

System 11-Infections And Infestations-Figure S87 Meta-analysis of SGLT2is and Mastoiditis

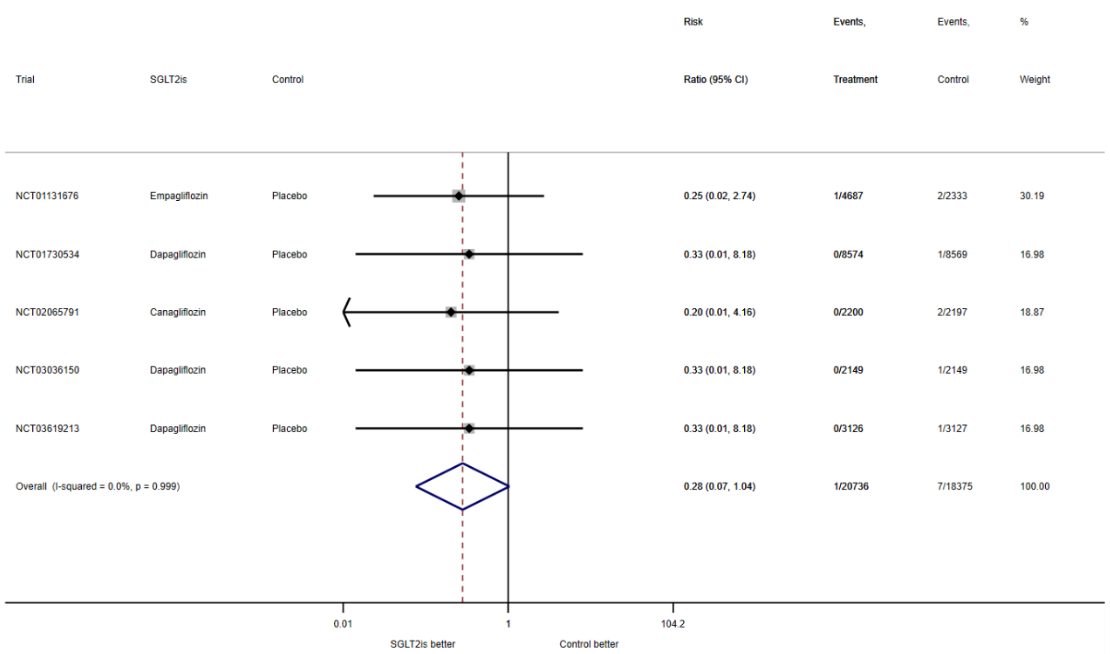

System 11-Infections And Infestations-Figure S88 Meta-analysis of SGLT2is and Mediastinitis

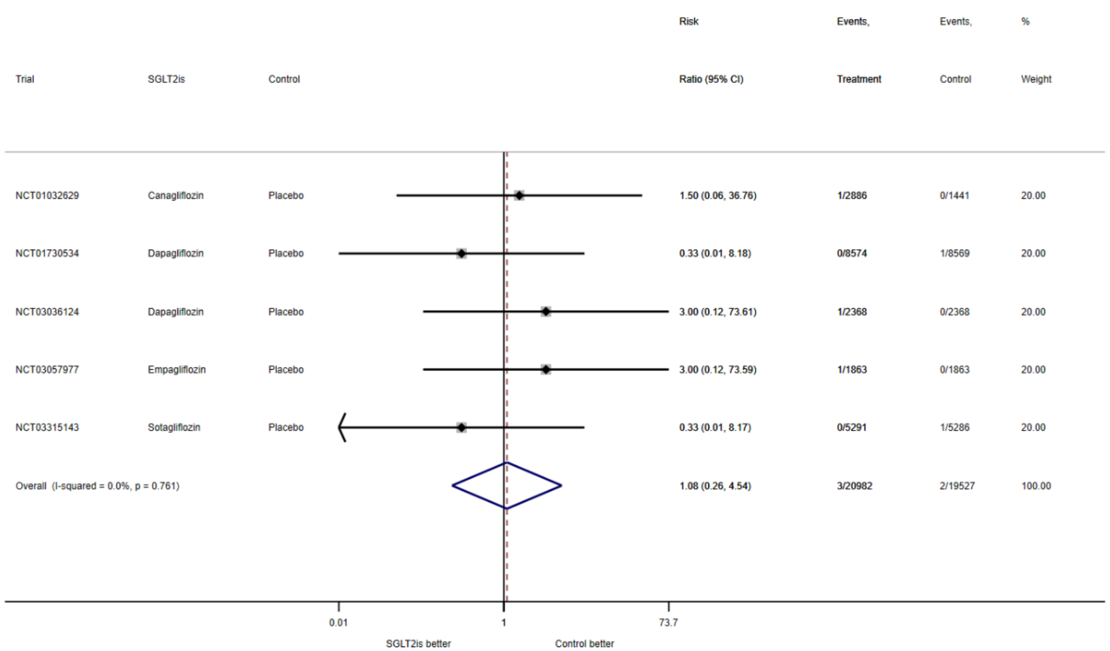

System 11-Infections And Infestations-Figure S89 Meta-analysis of SGLT2is and Medical device site infection

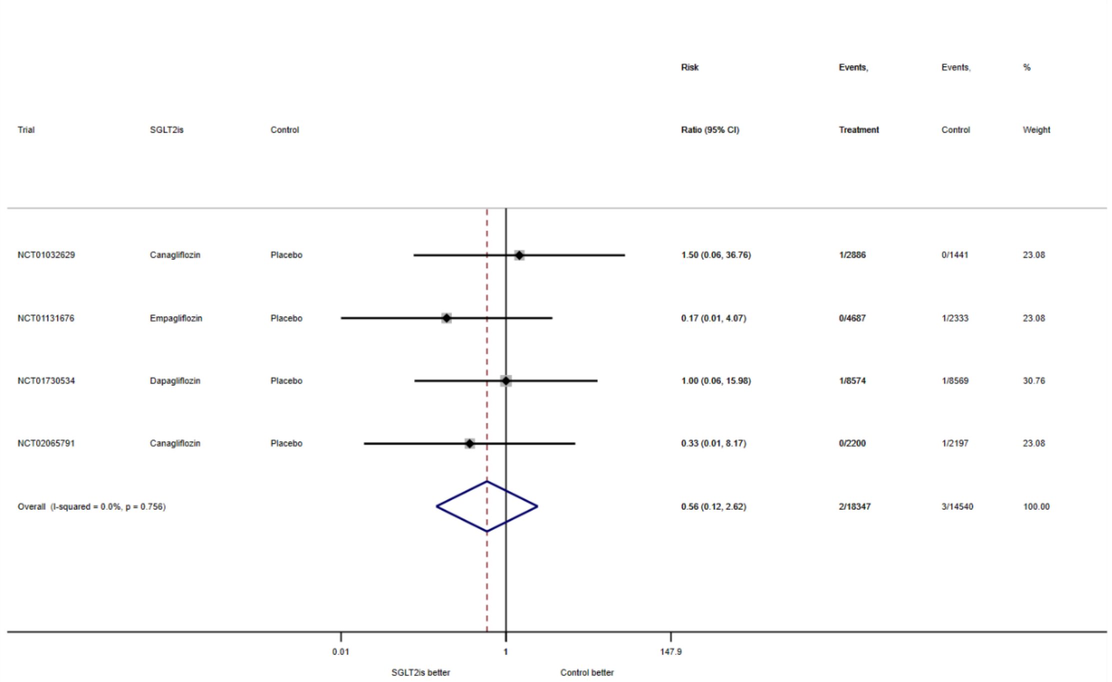

System 11-Infections And Infestations-Figure S90 Meta-analysis of SGLT2is and Meningitis

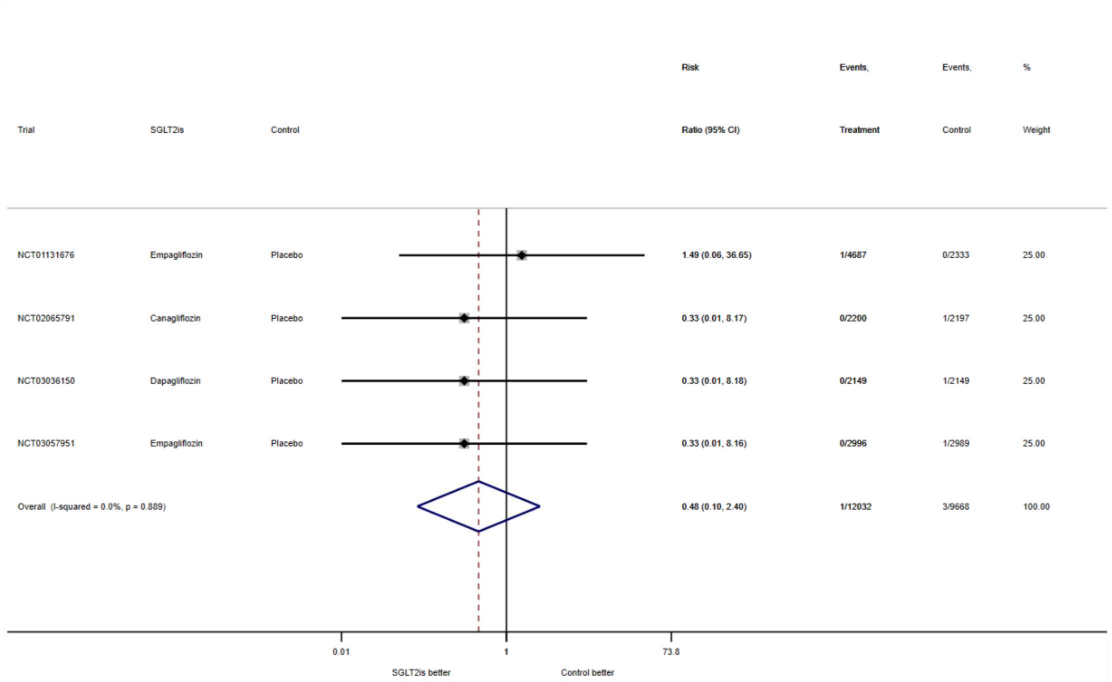

System 11-Infections And Infestations-Figure S91 Meta-analysis of SGLT2is and Meningitis bacterial

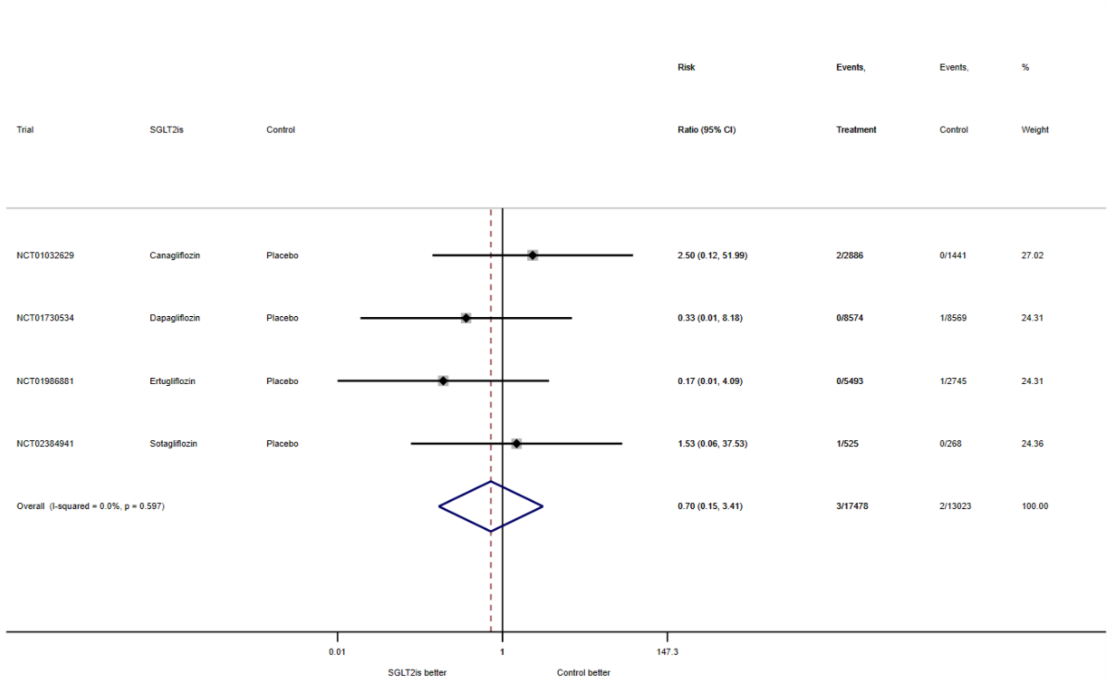

System 11-Infections And Infestations-Figure S92 Meta-analysis of SGLT2is and Meningitis viral

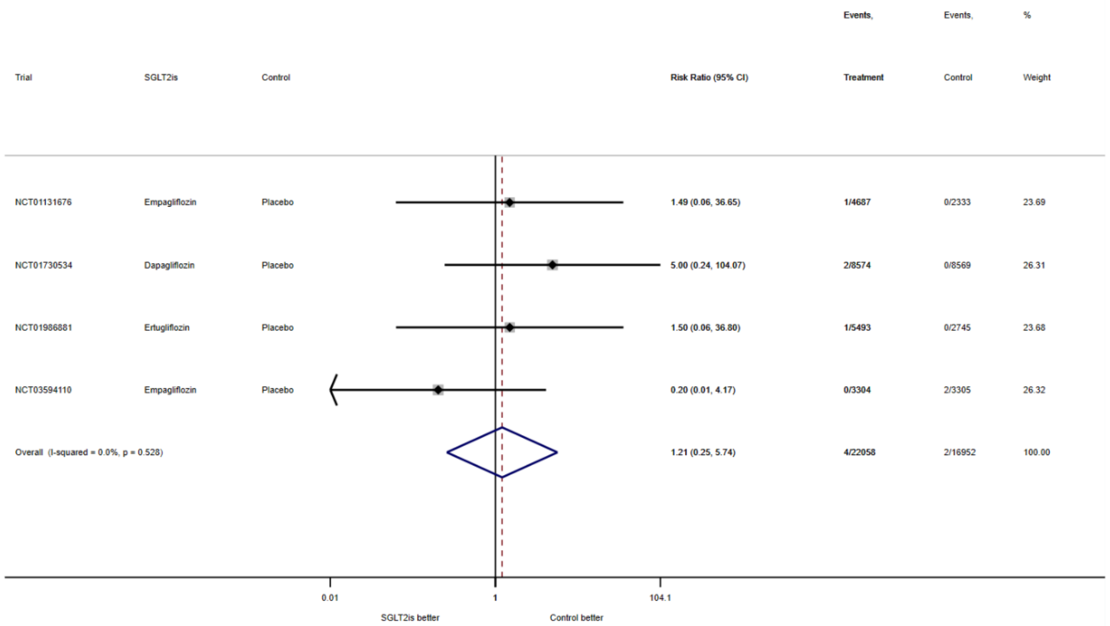

System 11-Infections And Infestations-Figure S93 Meta-analysis of SGLT2is and Muscle abscess

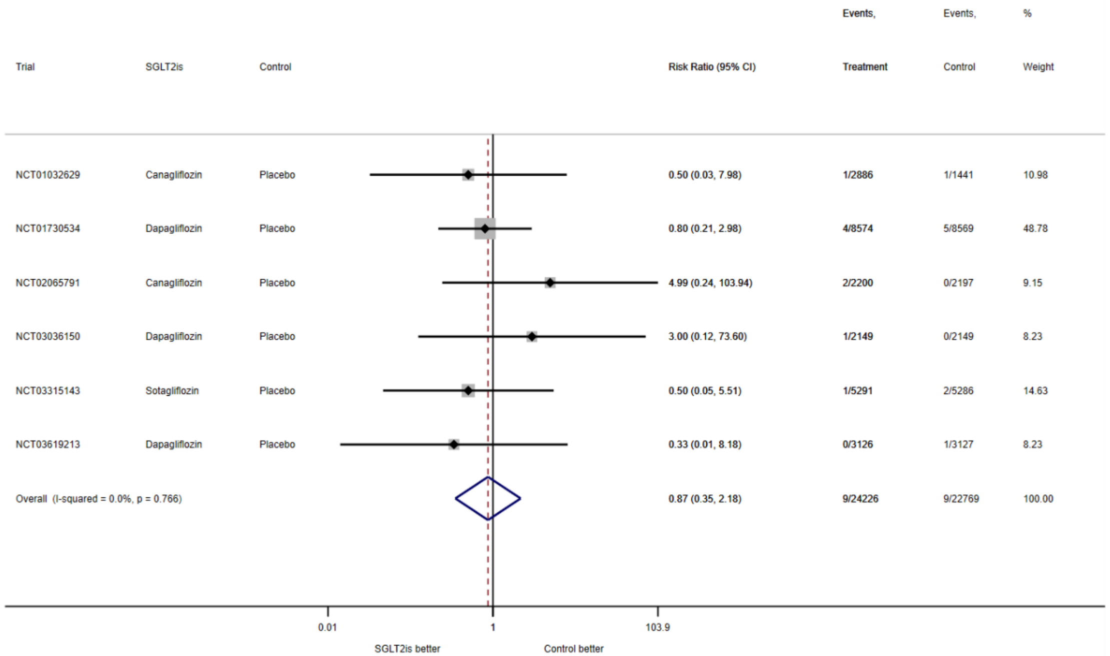

System 11-Infections And Infestations-Figure S94 Meta-analysis of SGLT2is and Necrotising fasciitis

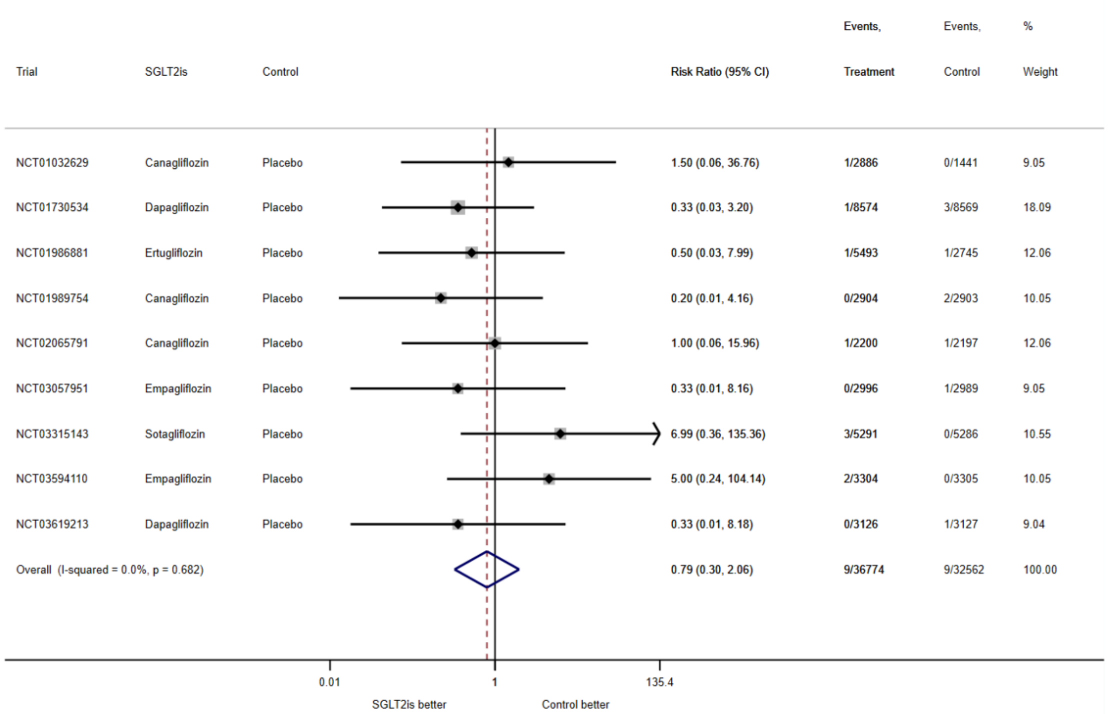

System 11-Infections And Infestations-Figure S95 Meta-analysis of SGLT2is and Orchitis

Trial	SGLT2is	Control	Risk Ratio (95% CI)	Events, Treatment	Events, Control	% Weight
NCT01032629	Canagliflozin	Placebo	1.64 (0.71, 3.81)	23/2886	7/1441	9.44
NCT01042977	Dapagliflozin	Placebo	3.01 (0.12, 73.61)	1/482	0/483	0.66
NCT01131676	Empagliflozin	Placebo	1.39 (0.50, 3.86)	14/4687	5/2333	6.46
NCT01730534	Dapagliflozin	Placebo	0.67 (0.41, 1.09)	26/8574	39/8569	27.39
NCT01986881	Ertugliflozin	Placebo	2.00 (0.82, 4.88)	24/5493	6/2745	8.42
NCT01989754	Canagliflozin	Placebo	1.20 (0.37, 3.93)	6/2904	5/2903	4.78
NCT02065791	Canagliflozin	Placebo	1.00 (0.43, 2.30)	11/2200	11/2197	9.67
NCT03036124	Dapagliflozin	Placebo	7.00 (0.86, 56.85)	7/2368	1/2368	1.53
NCT03036150	Dapagliflozin	Placebo	0.83 (0.36, 1.92)	10/2149	12/2149	9.59
NCT03057951	Empagliflozin	Placebo	1.20 (0.37, 3.92)	6/2996	5/2989	4.78
NCT03057977	Empagliflozin	Placebo	1.00 (0.14, 7.09)	2/1863	2/1863	1.75
NCT03315143	Sotagliflozin	Placebo	1.12 (0.43, 2.91)	9/5291	8/5286	7.42
NCT03521934	Sotagliflozin	Placebo	0.34 (0.01, 8.25)	0/605	1/611	0.66
NCT03594110	Empagliflozin	Placebo	3.00 (0.31, 28.83)	3/3304	1/3305	1.31
NCT03619213	Dapagliflozin	Placebo	1.00 (0.35, 2.85)	7/3126	7/3127	6.14
Overall (I-squared = 0.0%, p = 0.615)			1.07 (0.83, 1.39)	149/48928	110/42369	100.00

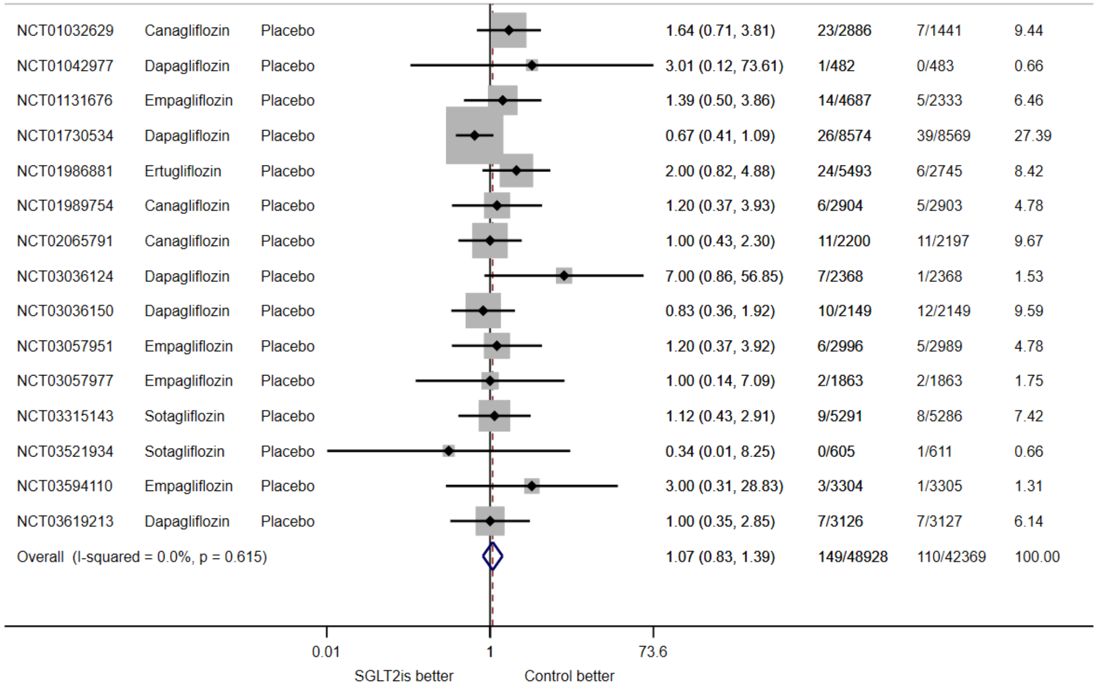

System 11-Infections And Infestations-Figure S96 Meta-analysis of SGLT2is and Osteomyelitis

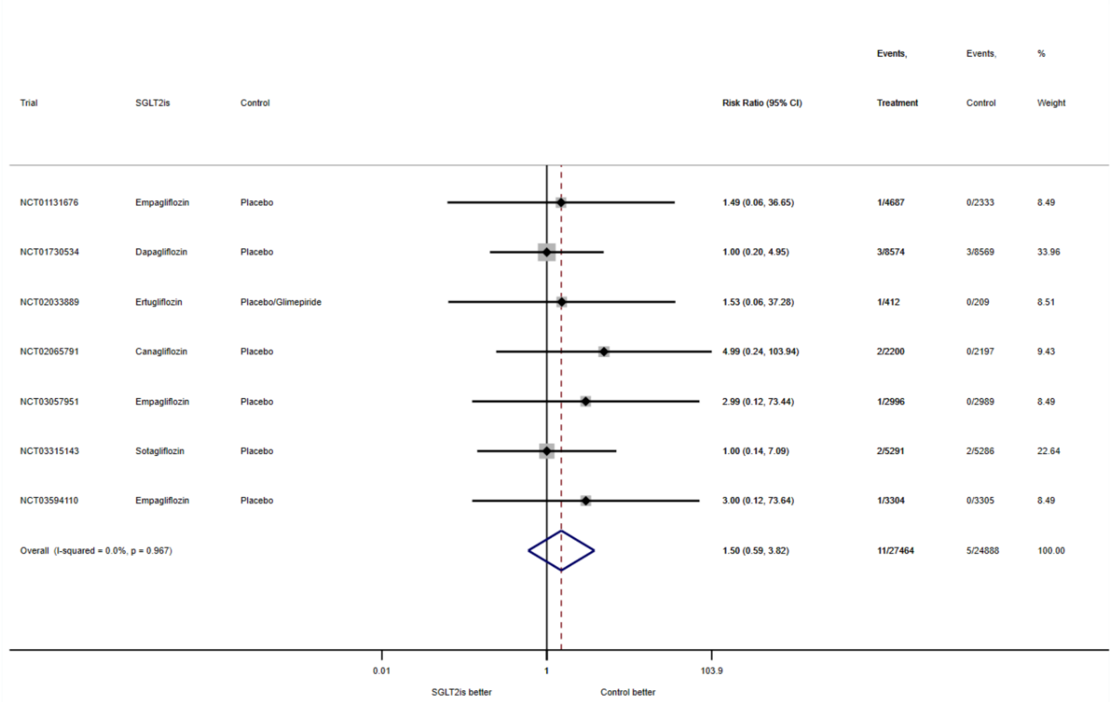

System 11-Infections And Infestations-Figure S97 Meta-analysis of SGLT2is and Osteomyelitis acute

Trial	SGLT2is	Control	Risk Ratio (95% CI)	Events, Treatment	Events, Control	% Weight
NCT01032629	Canagliflozin	Placebo	2.50 (0.12, 51.99)	2/2886	0/1441	7.68
NCT01131676	Empagliflozin	Placebo	1.49 (0.06, 36.65)	1/4687	0/2333	6.91
NCT01730534	Dapagliflozin	Placebo	1.33 (0.30, 5.95)	4/8574	3/8569	31.62
NCT01989754	Canagliflozin	Placebo	3.00 (0.12, 73.59)	1/2904	0/2903	6.91
NCT02065791	Canagliflozin	Placebo	3.00 (0.12, 73.50)	1/2200	0/2197	6.92
NCT03036150	Dapagliflozin	Placebo	3.00 (0.12, 73.60)	1/2149	0/2149	6.92
NCT03057977	Empagliflozin	Placebo	3.00 (0.12, 73.59)	1/1863	0/1863	6.92
NCT03315143	Sotagliflozin	Placebo	3.00 (0.12, 73.56)	1/5291	0/5286	6.91
NCT03594110	Empagliflozin	Placebo	2.00 (0.18, 22.05)	2/3304	1/3305	12.29
NCT03619213	Dapagliflozin	Placebo	3.00 (0.12, 73.64)	1/3126	0/3127	6.91
Overall (I-squared = 0.0%, p = 1.000)			2.07 (0.89, 4.81)	15/36984	4/33173	100.00

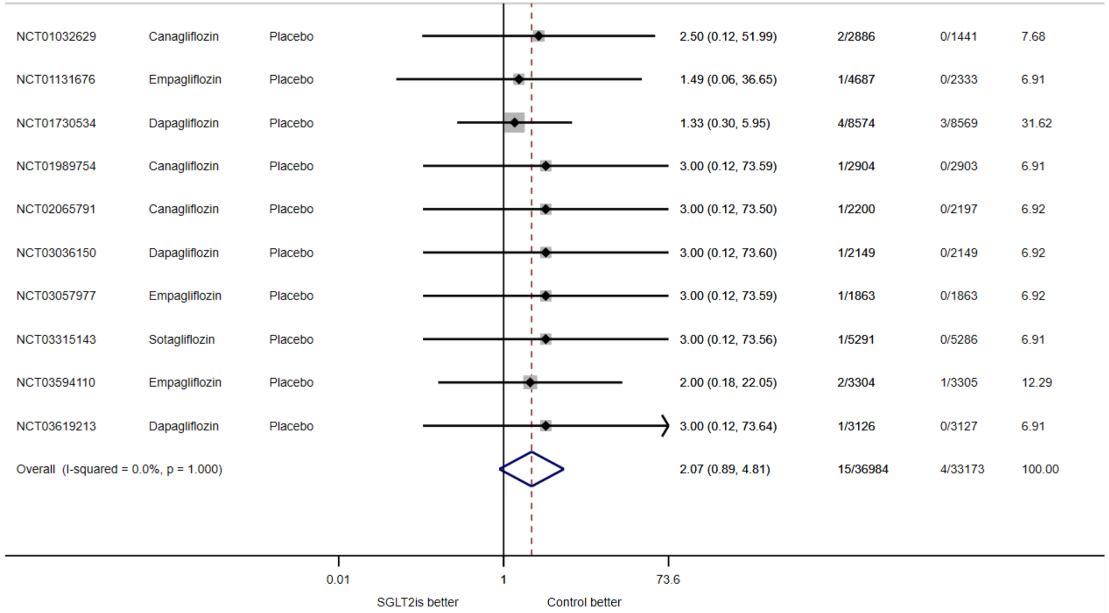

System 11-Infections And Infestations-Figure S98 Meta-analysis of SGLT2is and Osteomyelitis chronic

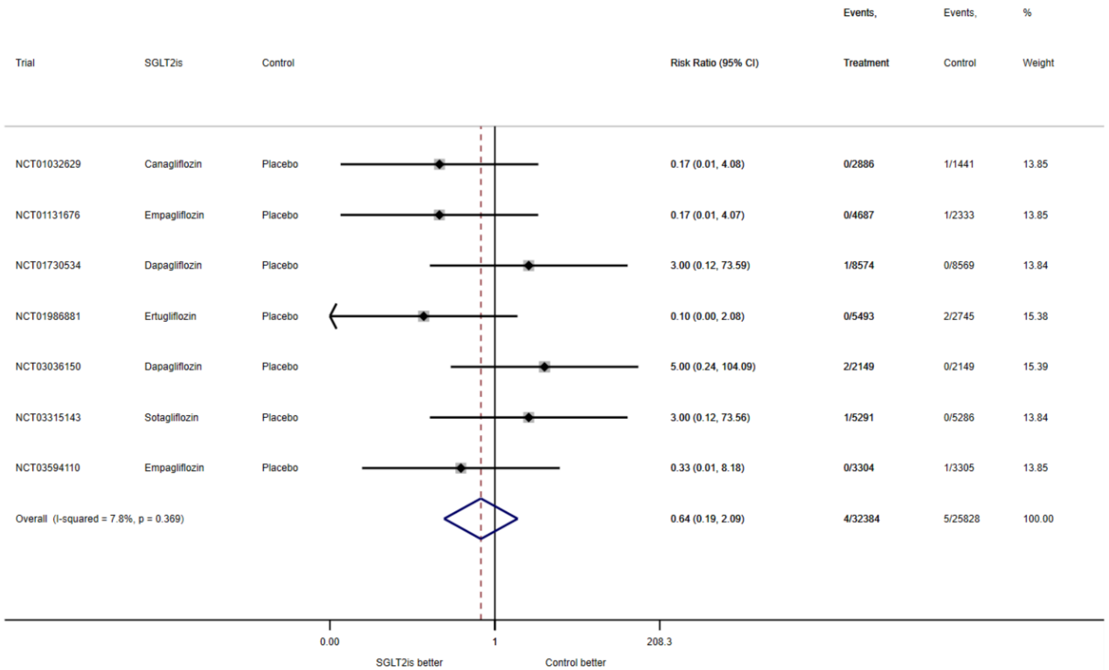

System 11-Infections And Infestations-Figure S99 Meta-analysis of SGLT2is and Otitis externa

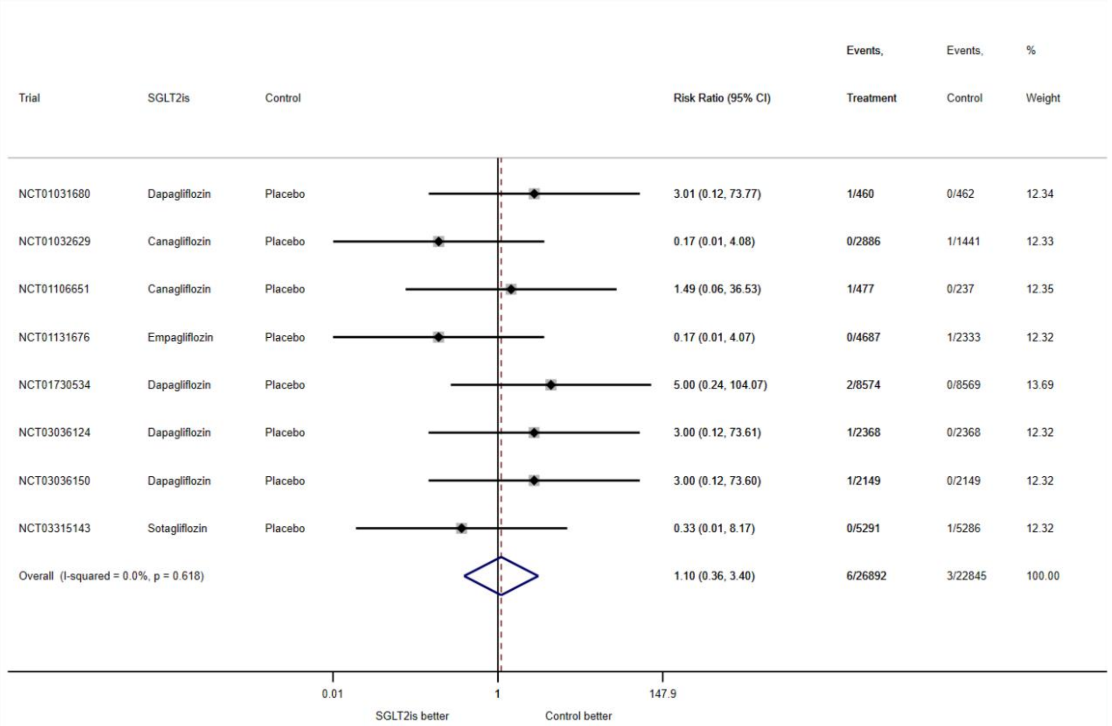

System 11-Infections And Infestations-Figure S100 Meta-analysis of SGLT2is and Otitis media

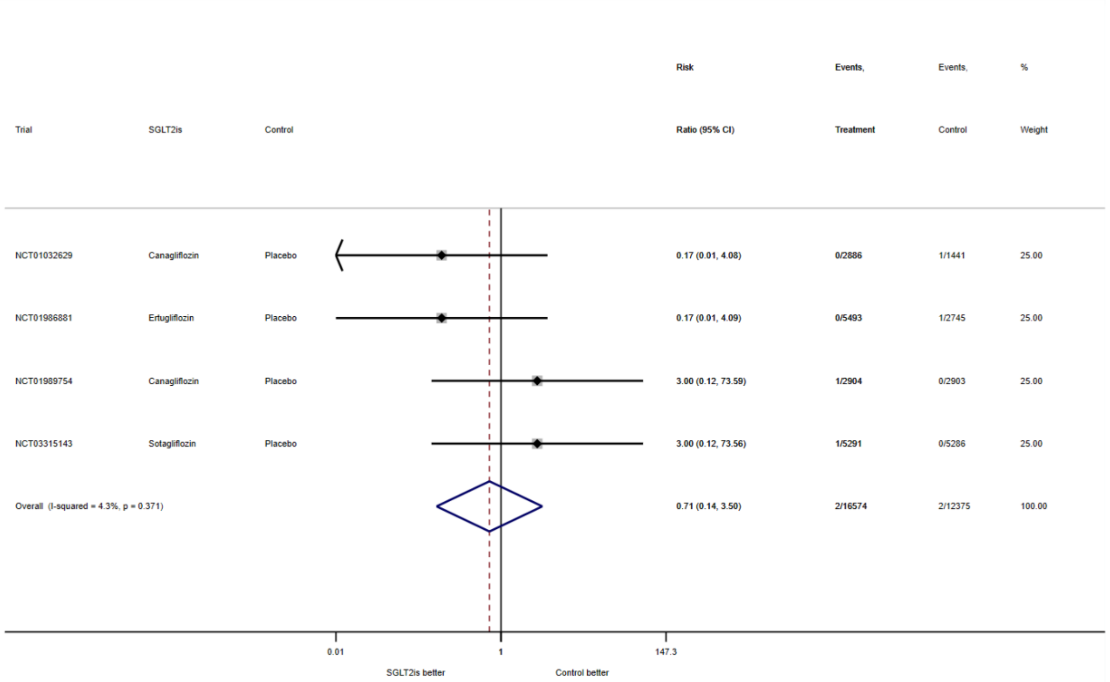

System 11-Infections And Infestations-Figure S101 Meta-analysis of SGLT2is and Paronychia

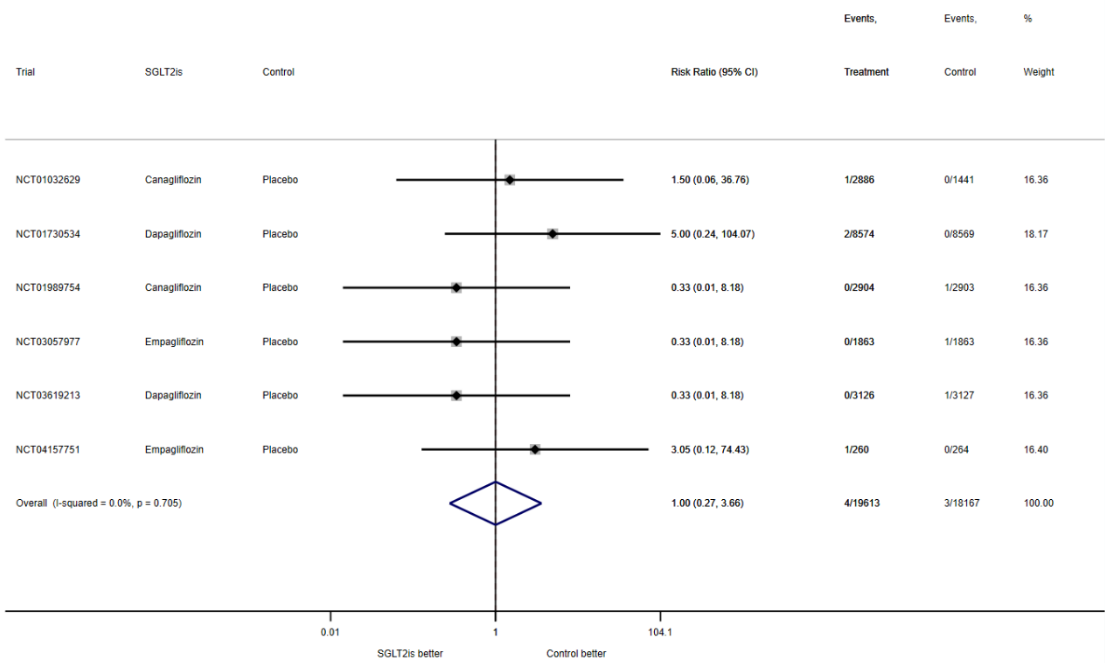

System 11-Infections And Infestations-Figure S102 Meta-analysis of SGLT2is and Pelvic abscess

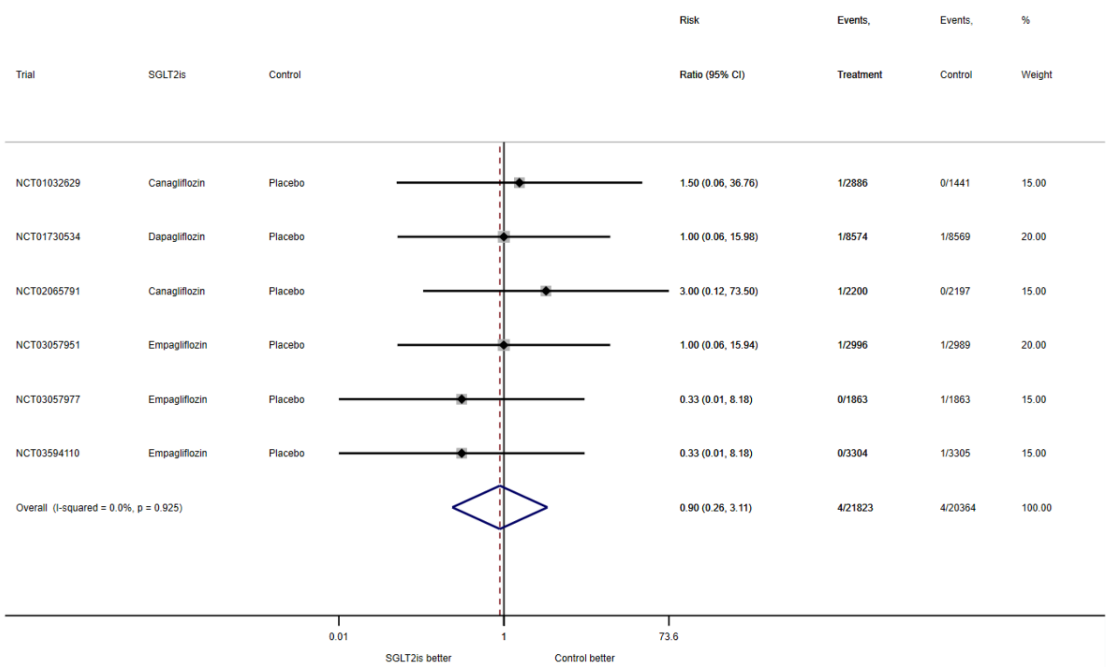

System 11-Infections And Infestations-Figure S103 Meta-analysis of SGLT2is and Perineal abscess

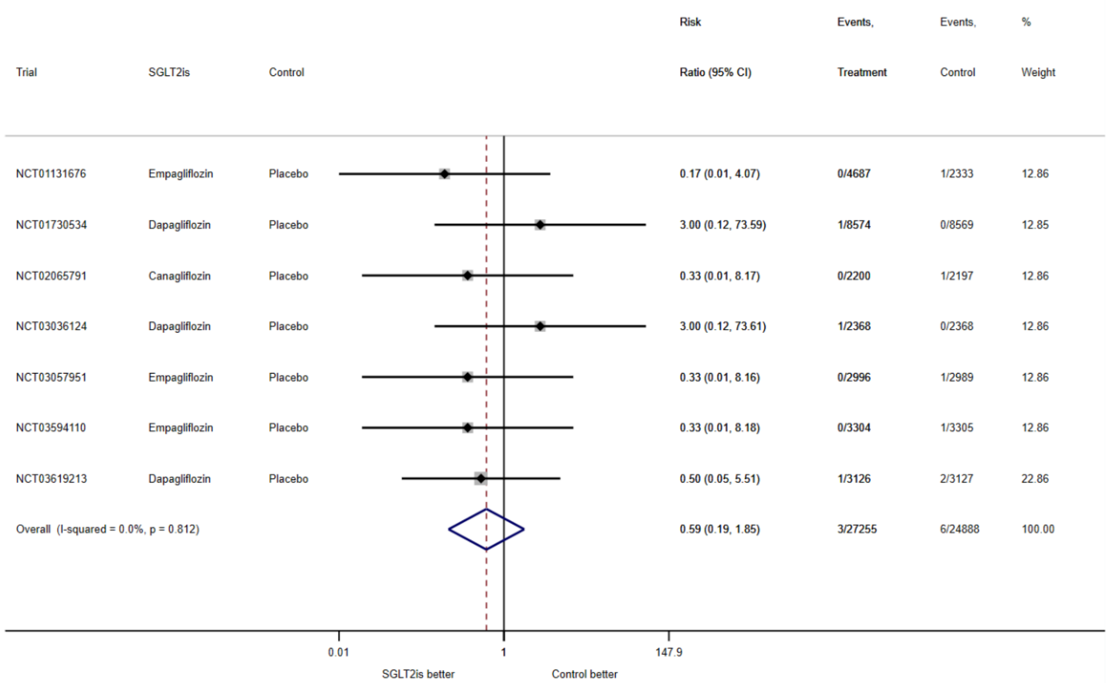

System 11-Infections And Infestations-Figure S104 Meta-analysis of SGLT2is and Periodontitis

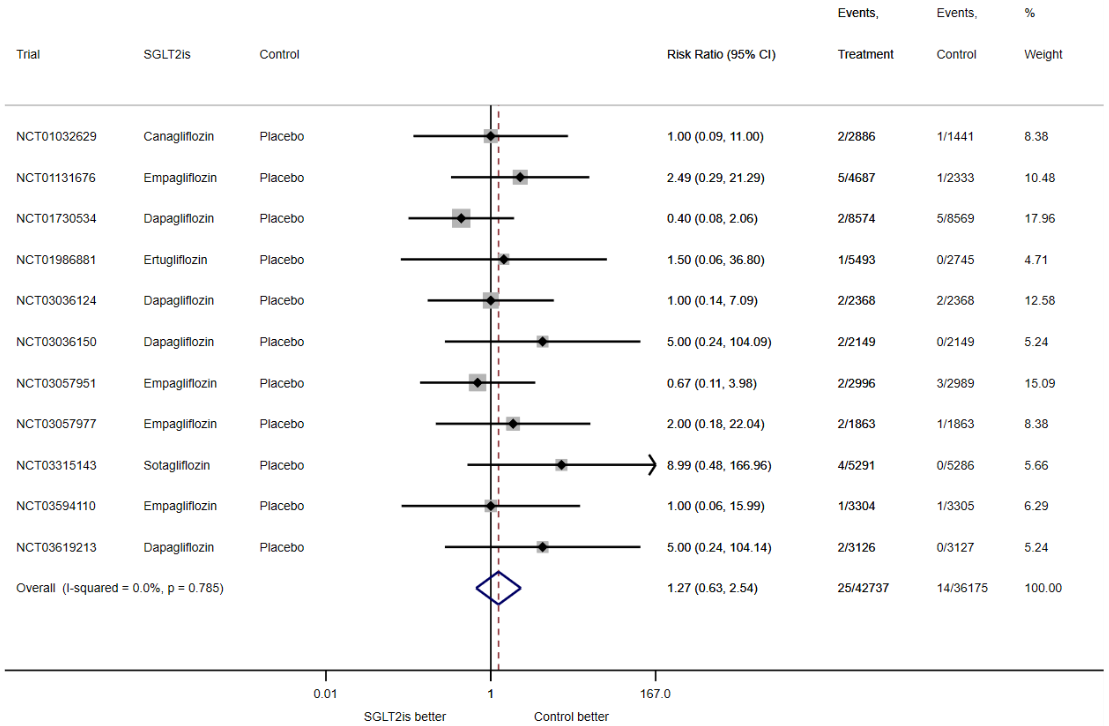

System 11-Infections And Infestations-Figure S105 Meta-analysis of SGLT2is and Peritonitis

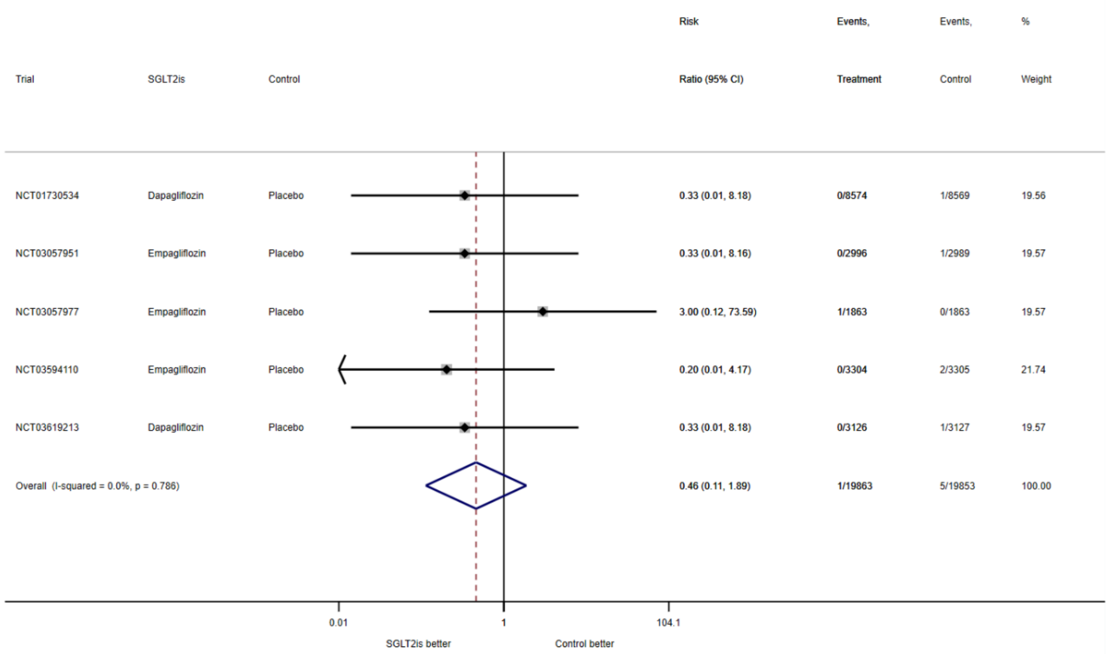

System 11-Infections And Infestations-Figure S106 Meta-analysis of SGLT2is and Peritonitis bacterial

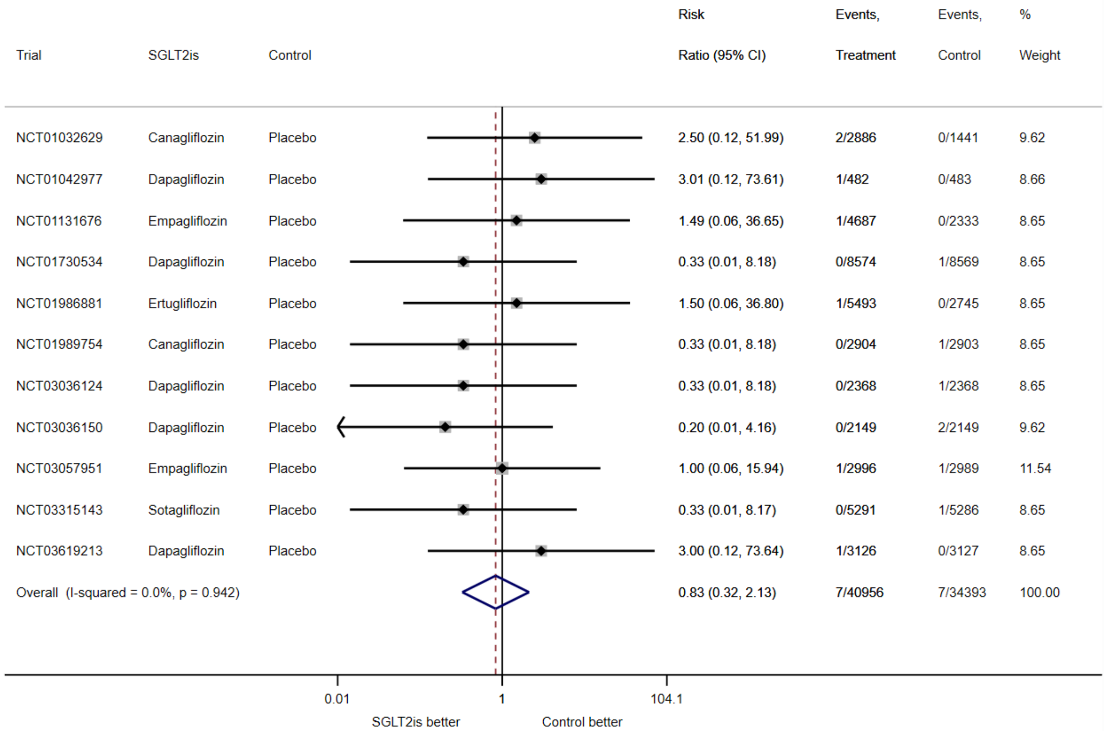

System 11-Infections And Infestations-Figure S107 Meta-analysis of SGLT2is and Peritonsillar abscess

Trial	SGLT2is	Control	Risk Ratio (95% CI)	Events, Treatment	Events, Control	% Weight
NCT01032629	Canagliflozin	Placebo	1.50 (0.06, 36.76)	1/2886	0/1441	14.06
NCT01164501	Empagliflozin	Placebo	2.29 (0.09, 55.92)	1/419	0/319	14.08
NCT01730534	Dapagliflozin	Placebo	0.20 (0.01, 4.16)	0/8574	2/8569	15.62
NCT01986881	Ertugliflozin	Placebo	1.50 (0.06, 36.80)	1/5493	0/2745	14.06
NCT03057977	Empagliflozin	Placebo	3.00 (0.12, 73.59)	1/1863	0/1863	14.06
NCT03315143	Sotagliflozin	Placebo	0.33 (0.01, 8.17)	0/5291	1/5286	14.06
NCT03619213	Dapagliflozin	Placebo	3.00 (0.12, 73.64)	1/3126	0/3127	14.06
Overall (I-squared = 0.0%, p = 0.837)			1.14 (0.34, 3.79)	5/27652	3/23350	100.00

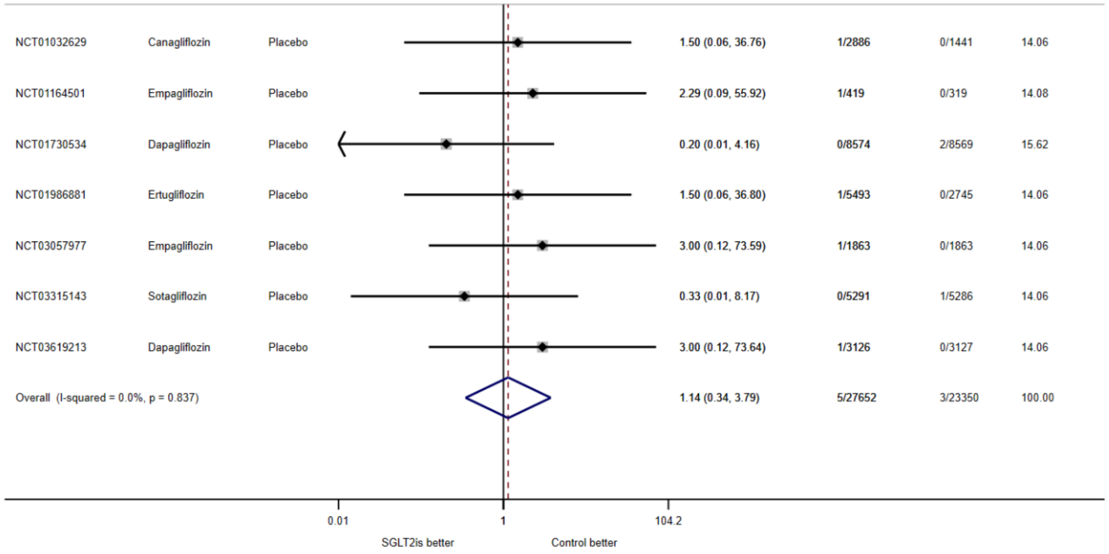

System 11-Infections And Infestations-Figure S108 Meta-analysis of SGLT2is and Pharyngitis

Trial	SGLT2is	Control	Risk Ratio (95% CI)	Events, Treatment	Events, Control	% Weight
NCT00673231	Dapagliflozin	Placebo	1.62 (0.08, 33.61)	2/610	0/197	0.08
NCT01031680	Dapagliflozin	Placebo	1.00 (0.06, 16.01)	1/460	1/462	0.10
NCT01032629	Canagliflozin	Placebo	0.79 (0.52, 1.19)	57/2886	36/1441	4.39
NCT01042977	Dapagliflozin	Placebo	0.20 (0.01, 4.16)	0/482	2/483	0.08
NCT01106651	Canagliflozin	Placebo	0.75 (0.13, 4.43)	3/477	2/237	0.23
NCT01106677	Canagliflozin	Sitagliptin	1.50 (0.06, 36.63)	1/735	0/366	0.07
NCT01131676	Empagliflozin	Placebo	0.74 (0.53, 1.05)	79/4687	53/2333	6.29
NCT01164501	Empagliflozin	Placebo	0.25 (0.01, 6.21)	0/419	1/319	0.07
NCT01730534	Dapagliflozin	Placebo	0.89 (0.73, 1.07)	194/8574	219/8569	20.47
NCT01986881	Ertugliflozin	Placebo	1.08 (0.76, 1.53)	97/5493	45/2745	6.07
NCT01989754	Canagliflozin	Placebo	0.84 (0.50, 1.41)	26/2904	31/2903	2.77
NCT02033889	Ertugliflozin	Placebo/Glimepiride	0.51 (0.03, 8.07)	1/412	1/209	0.10
NCT02065791	Canagliflozin	Placebo	0.73 (0.53, 1.01)	63/2200	86/2197	7.30
NCT02384941	Sotagliflozin	Placebo	2.56 (0.12, 53.07)	2/525	0/268	0.08
NCT03036124	Dapagliflozin	Placebo	0.93 (0.68, 1.26)	76/2368	82/2368	7.92
NCT03036150	Dapagliflozin	Placebo	0.63 (0.43, 0.91)	44/2149	70/2149	5.38
NCT03057951	Empagliflozin	Placebo	0.84 (0.65, 1.09)	100/2996	119/2989	10.95
NCT03057977	Empagliflozin	Placebo	0.85 (0.60, 1.23)	53/1863	62/1863	5.72
NCT03242252	Sotagliflozin	Placebo	1.48 (0.15, 14.16)	3/527	1/260	0.15
NCT03315143	Sotagliflozin	Placebo	0.65 (0.43, 0.98)	37/5291	57/5286	4.39
NCT03521934	Sotagliflozin	Placebo	0.74 (0.41, 1.32)	19/605	26/611	2.21
NCT03594110	Empagliflozin	Placebo	0.93 (0.60, 1.43)	39/3304	42/3305	3.98
NCT03619213	Dapagliflozin	Placebo	1.07 (0.82, 1.38)	113/3126	106/3127	11.01
NCT04157751	Empagliflozin	Placebo	0.11 (0.01, 2.08)	0/260	4/264	0.09
NCT04350593	Dapagliflozin	Placebo	0.06 (0.00, 1.02)	0/613	8/616	0.09
Overall (I-squared = 0.0%, p = 0.746)			0.85 (0.78, 0.93)	1010/53966	1054/45567	100.00

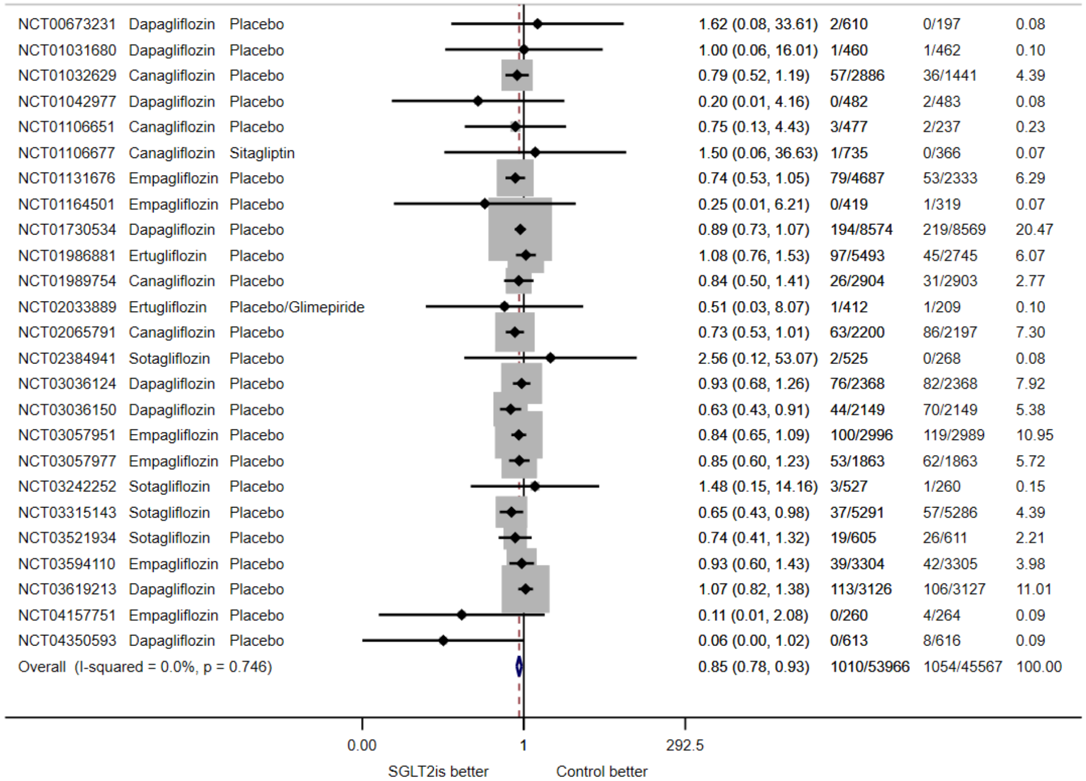

System 11-Infections And Infestations-Figure S109 Meta-analysis of SGLT2is and Pneumonia

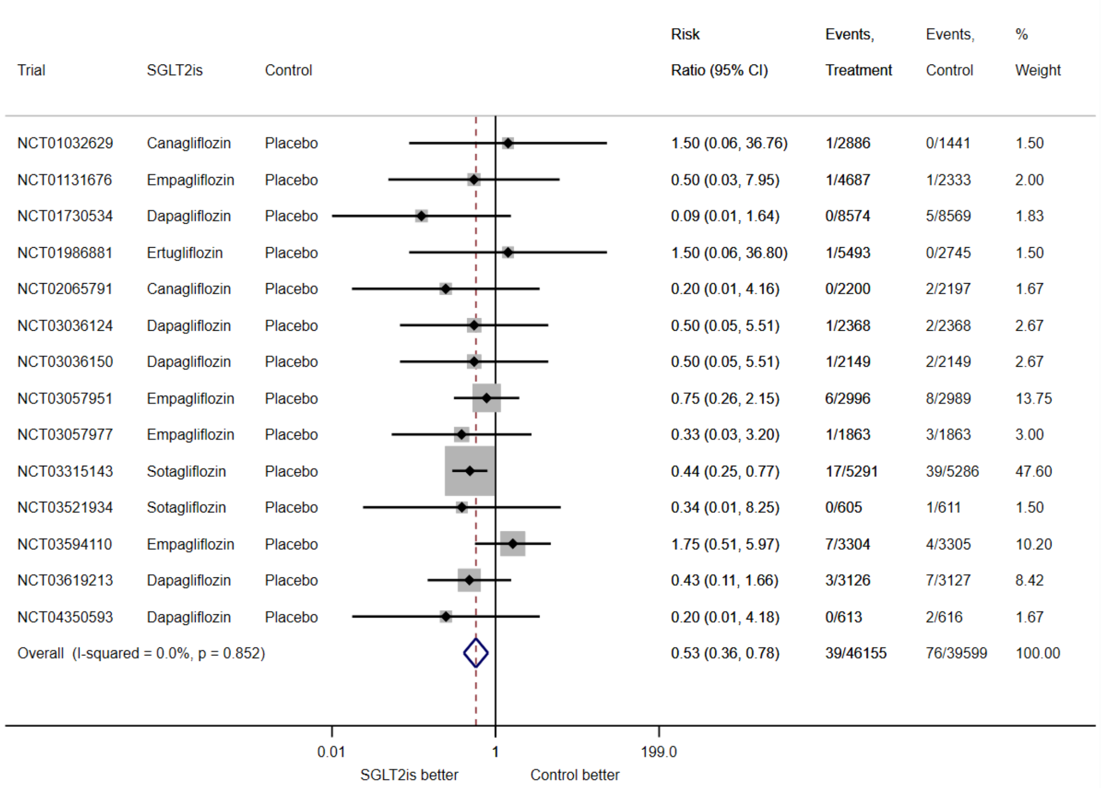

System 11-Infections And Infestations-Figure S110 Meta-analysis of SGLT2is and Pneumonia bacterial

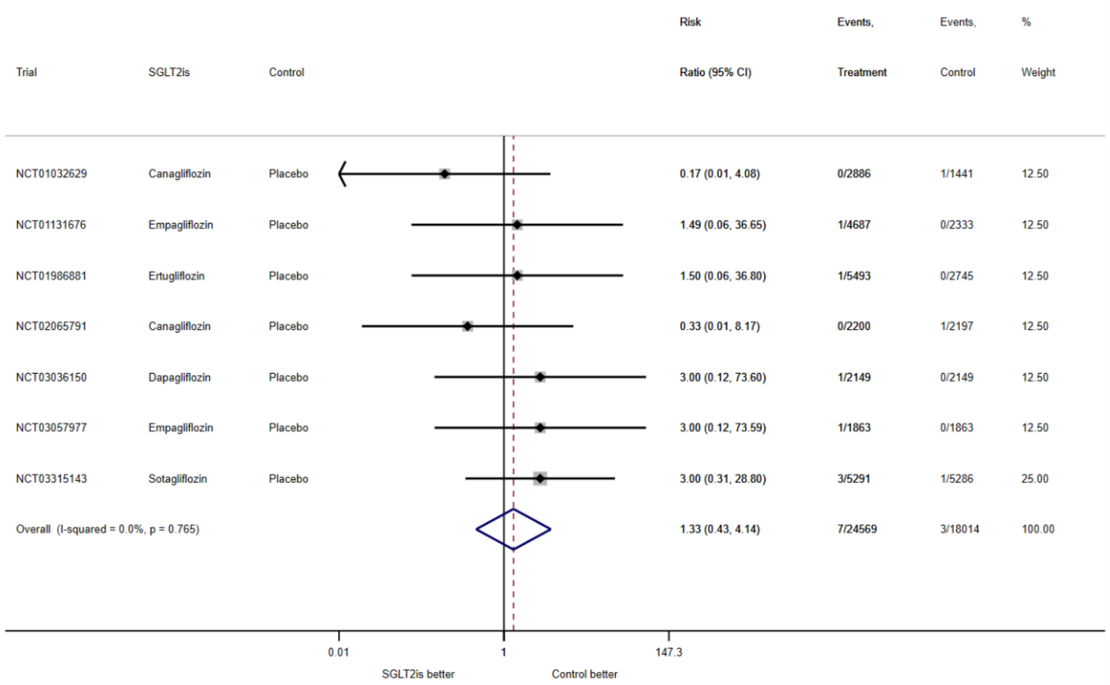

System 11-Infections And Infestations-Figure S111 Meta-analysis of SGLT2is and Pneumonia influenzal

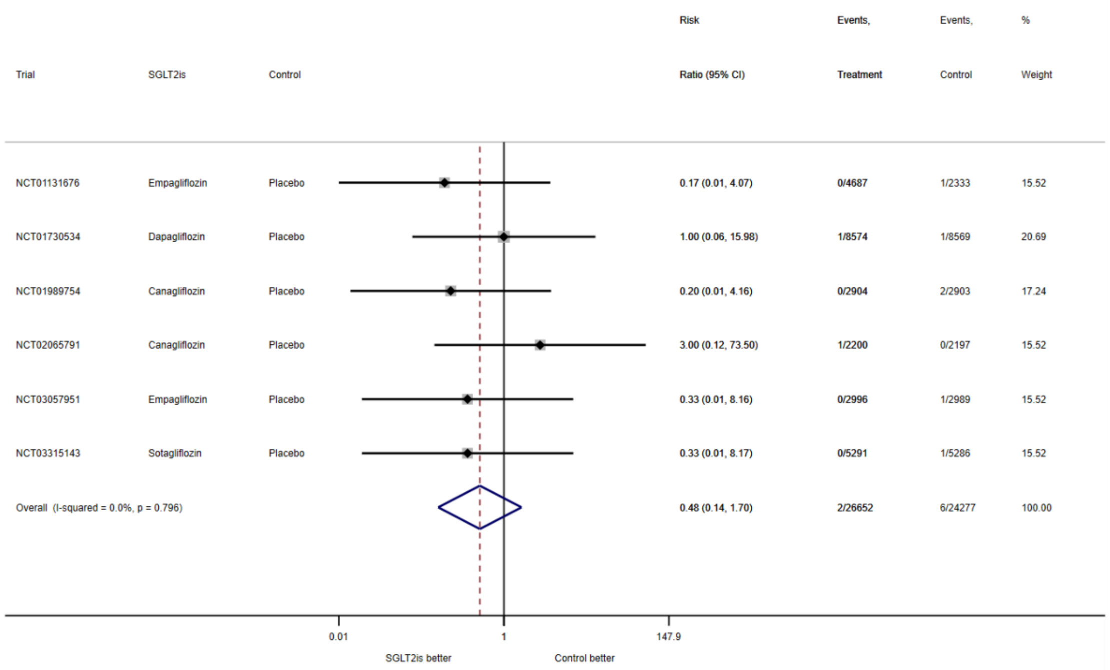

System 11-Infections And Infestations-Figure S112 Meta-analysis of SGLT2is and Pneumonia pneumococcal

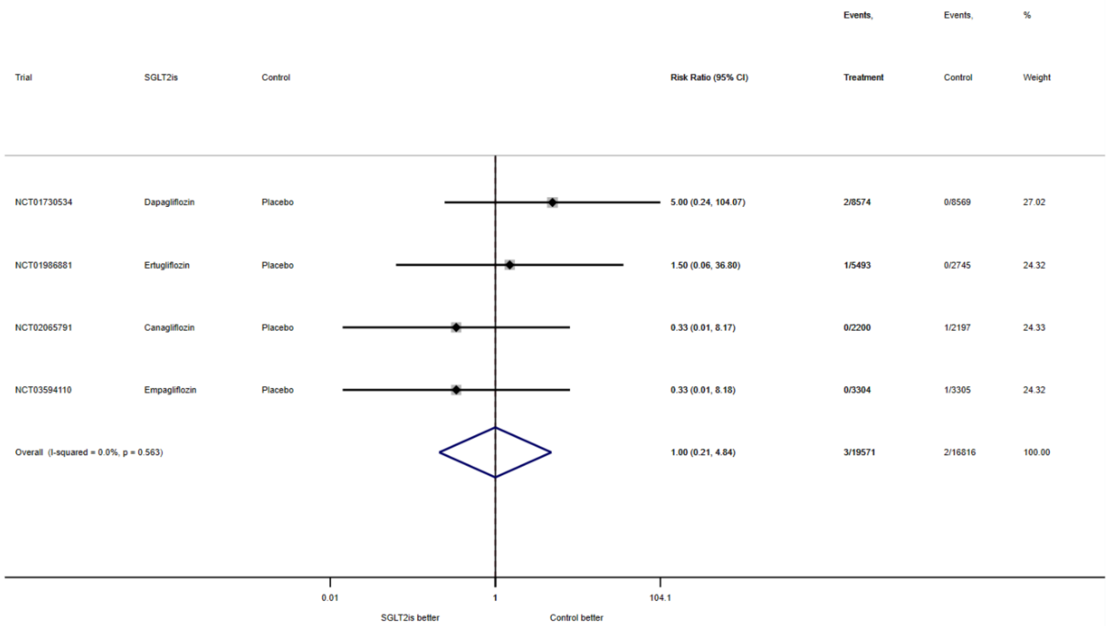

System 11-Infections And Infestations-Figure S113 Meta-analysis of SGLT2is and Pneumonia staphylococcal

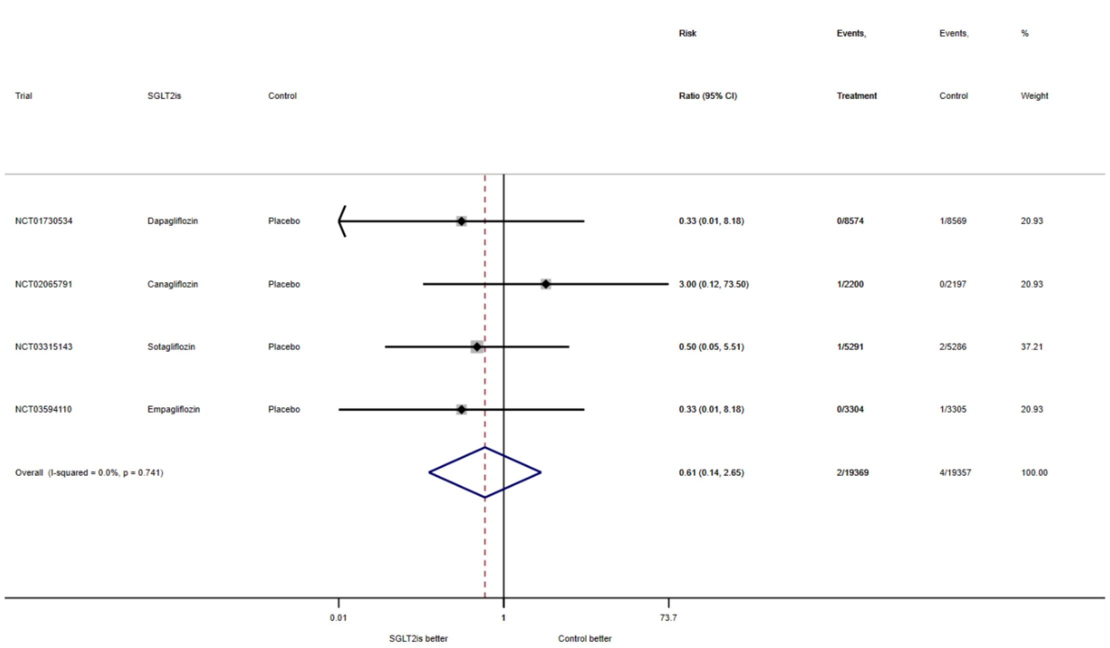

System 11-Infections And Infestations-Figure S114 Meta-analysis of SGLT2is and Pneumonia streptococcal

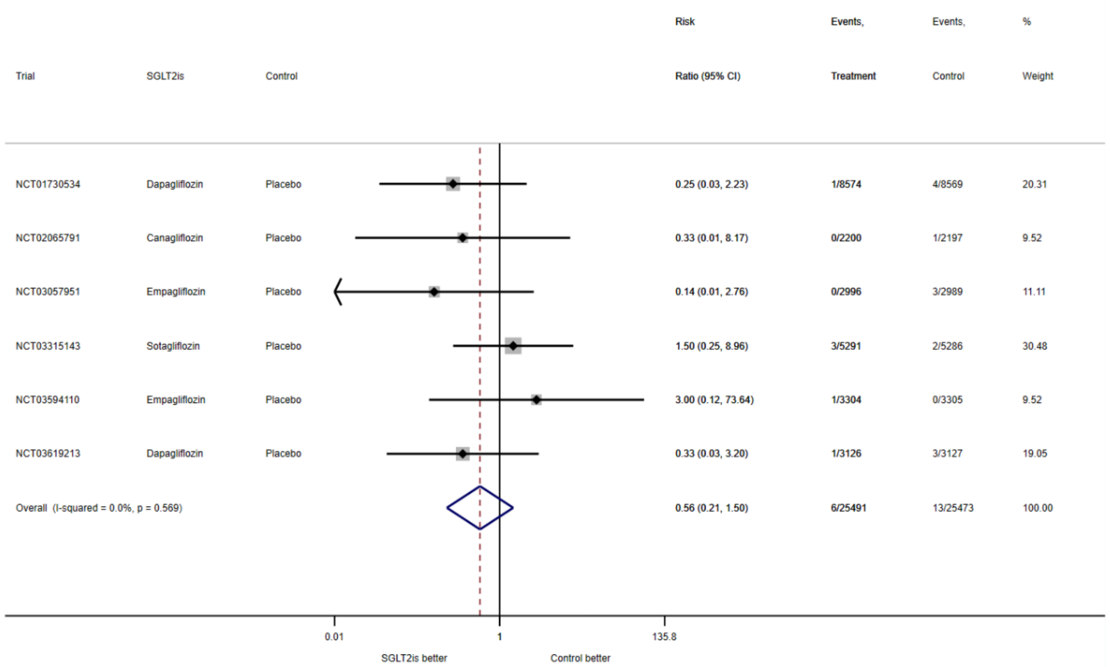

System 11-Infections And Infestations-Figure S115 Meta-analysis of SGLT2is and Pneumonia viral

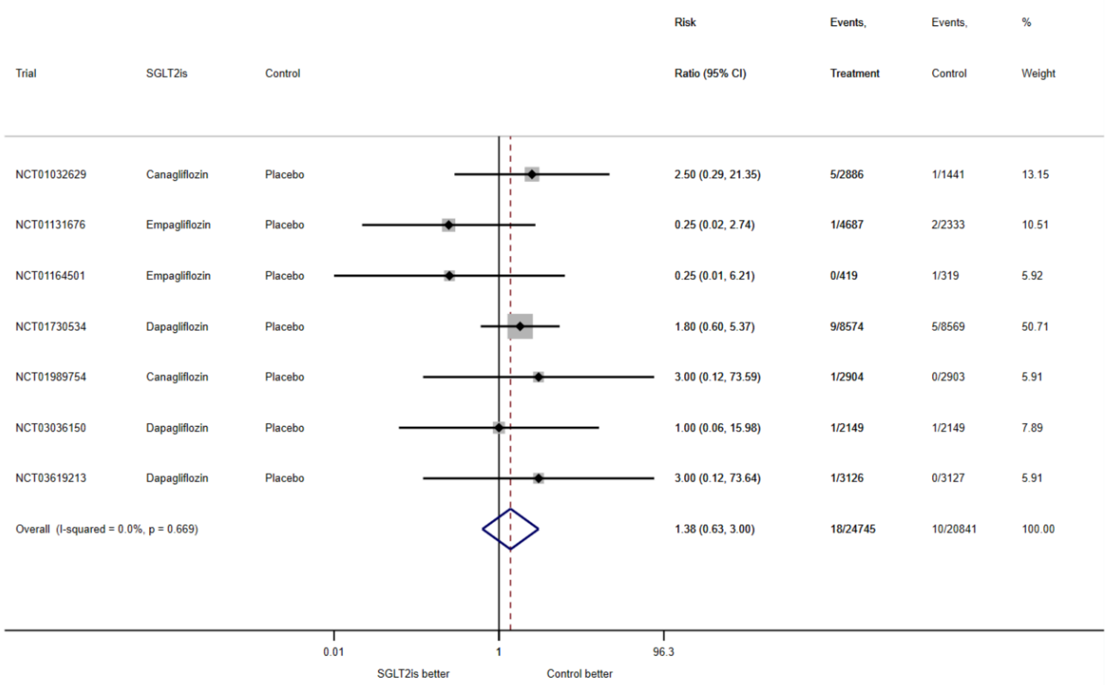

System 11-Infections And Infestations-Figure S116 Meta-analysis of SGLT2is and Post procedural infection

Trial	SGLT2is	Control	Risk Ratio (95% CI)	Events, Treatment	Events, Control	% Weight
NCT01032629	Canagliflozin	Placebo	1.50 (0.06, 36.76)	1/2886	0/1441	15.79
NCT01730534	Dapagliflozin	Placebo	3.00 (0.31, 28.82)	3/8574	1/8569	31.58
NCT01986881	Ertugliflozin	Placebo	0.17 (0.01, 4.09)	0/5493	1/2745	15.79
NCT03036124	Dapagliflozin	Placebo	0.33 (0.01, 8.18)	0/2368	1/2368	15.79
NCT03057951	Empagliflozin	Placebo	1.00 (0.06, 15.94)	1/2996	1/2989	21.05
Overall (I-squared = 0.0%, p = 0.624)			0.95 (0.27, 3.41)	5/22317	4/18112	100.00

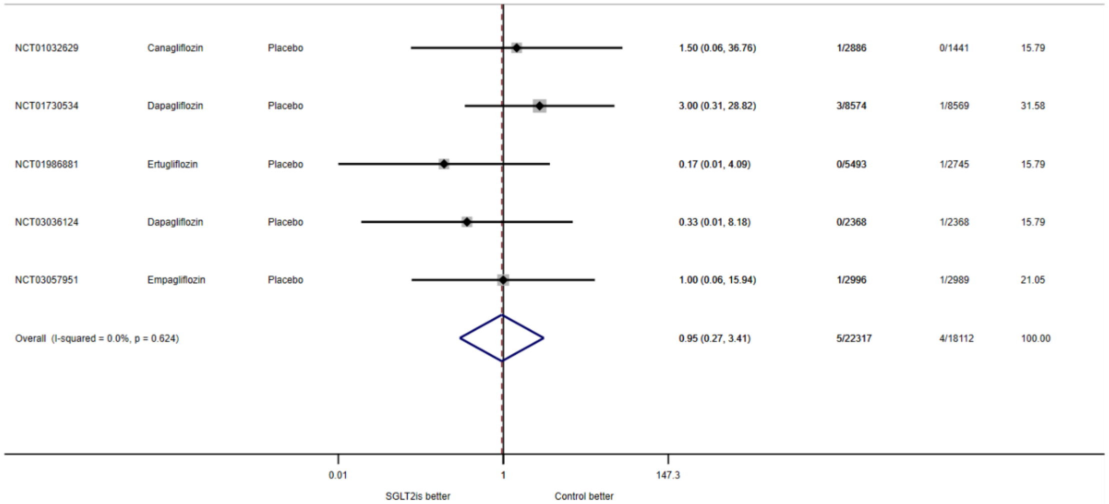

System 11-Infections And Infestations-Figure S117 Meta-analysis of SGLT2is and Postoperative abscess

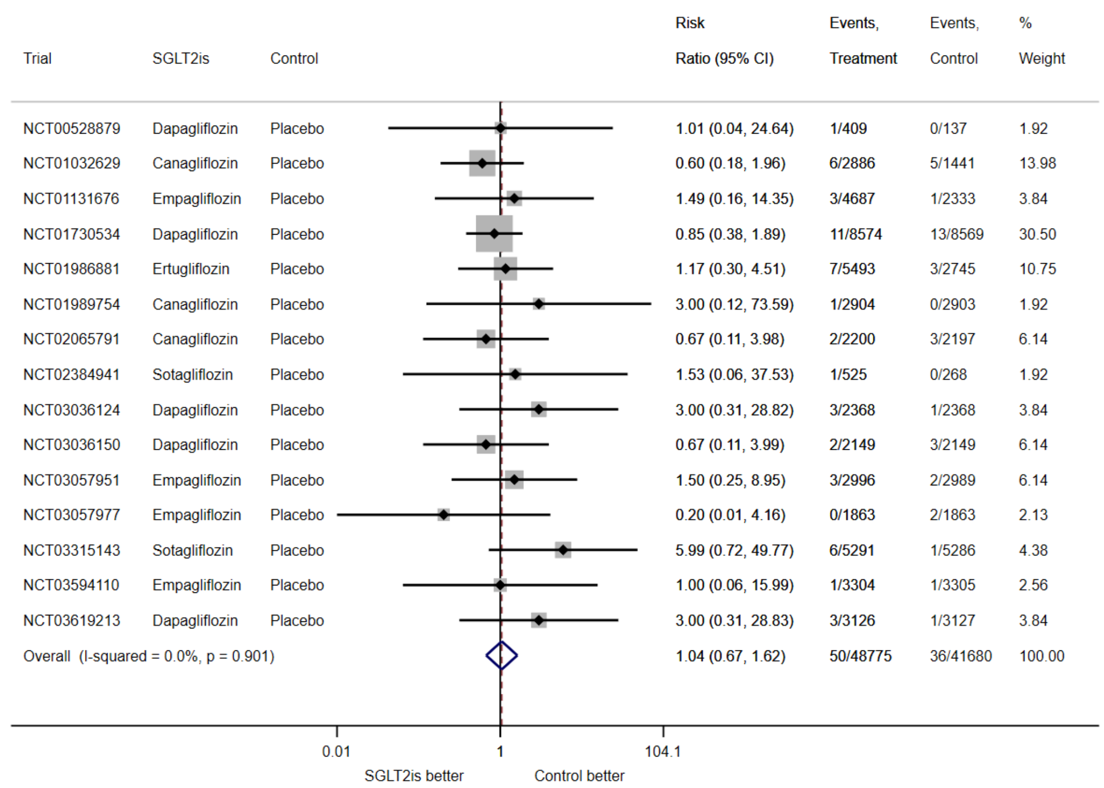

System 11-Infections And Infestations-Figure S118 Meta-analysis of SGLT2is and Postoperative wound infection

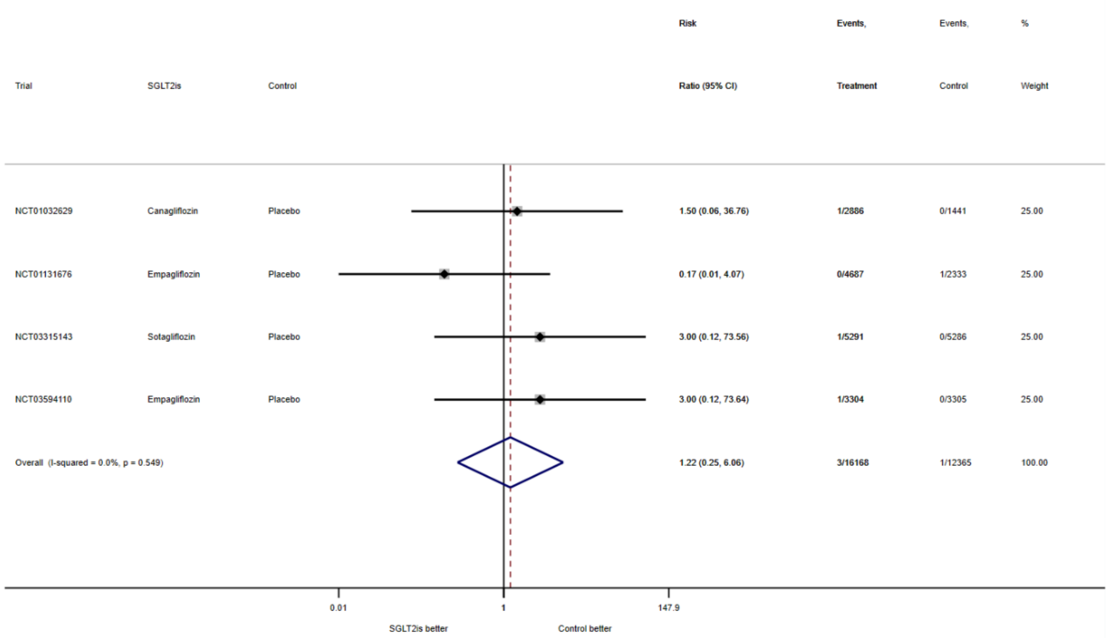

System 11-Infections And Infestations-Figure S119 Meta-analysis of SGLT2is and Psoas abscess

Trial	SGLT2is	Control	Risk Ratio (95% CI)	Events, Treatment	Events, Control	% Weight
NCT01032629	Canagliflozin	Placebo	0.17 (0.01, 4.08)	0/2886	1/1441	4.59
NCT01730534	Dapagliflozin	Placebo	1.00 (0.06, 15.98)	1/8574	1/8569	6.11
NCT01989754	Canagliflozin	Placebo	0.33 (0.01, 8.18)	0/2904	1/2903	4.59
NCT02065791	Canagliflozin	Placebo	3.00 (0.12, 73.50)	1/2200	0/2197	4.59
NCT03036124	Dapagliflozin	Placebo	0.50 (0.05, 5.51)	1/2368	2/2368	8.15
NCT03036150	Dapagliflozin	Placebo	3.00 (0.12, 73.60)	1/2149	0/2149	4.59
NCT03057951	Empagliflozin	Placebo	1.25 (0.34, 4.64)	5/2996	4/2989	27.21
NCT03057977	Empagliflozin	Placebo	5.00 (0.24, 104.08)	2/1863	0/1863	5.10
NCT03315143	Sotagliflozin	Placebo	1.50 (0.25, 8.96)	3/5291	2/5286	14.68
NCT03521934	Sotagliflozin	Placebo	0.34 (0.01, 8.25)	0/605	1/611	4.59
NCT03619213	Dapagliflozin	Placebo	5.00 (0.24, 104.14)	2/3126	0/3127	5.10
NCT04157751	Empagliflozin	Placebo	0.34 (0.01, 8.27)	0/260	1/264	4.60
NCT04350593	Dapagliflozin	Placebo	1.00 (0.06, 16.03)	1/613	1/616	6.12
Overall (I-squared = 0.0%, p = 0.908)			1.10 (0.55, 2.18)	17/35835	14/34383	100.00

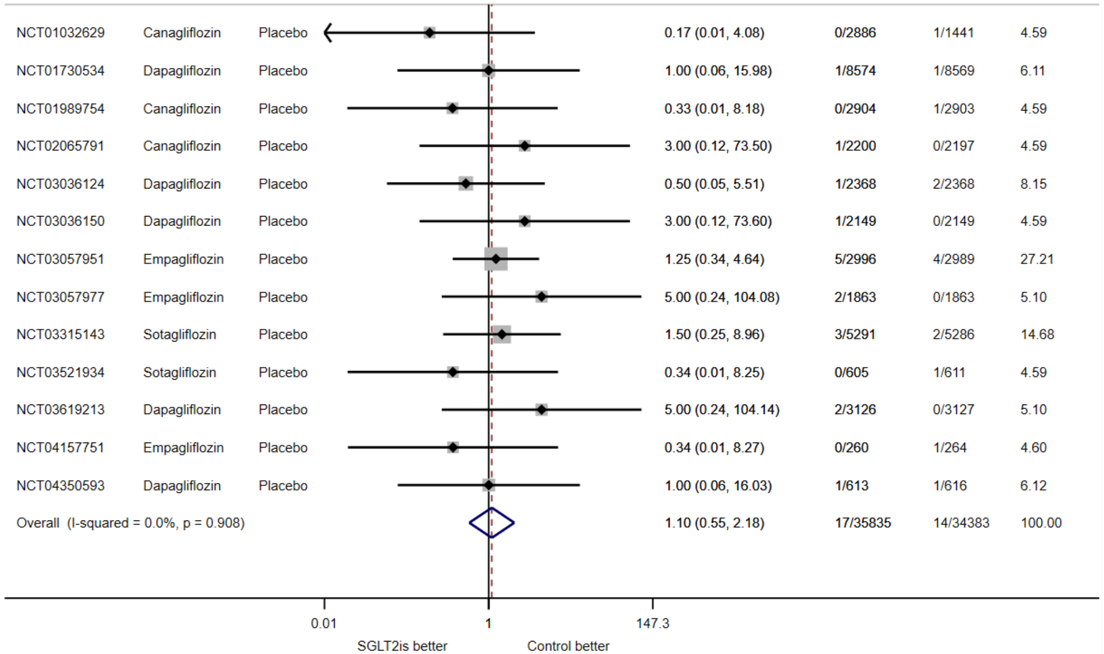

System 11-Infections And Infestations-Figure S120 Meta-analysis of SGLT2is and Pulmonary sepsis

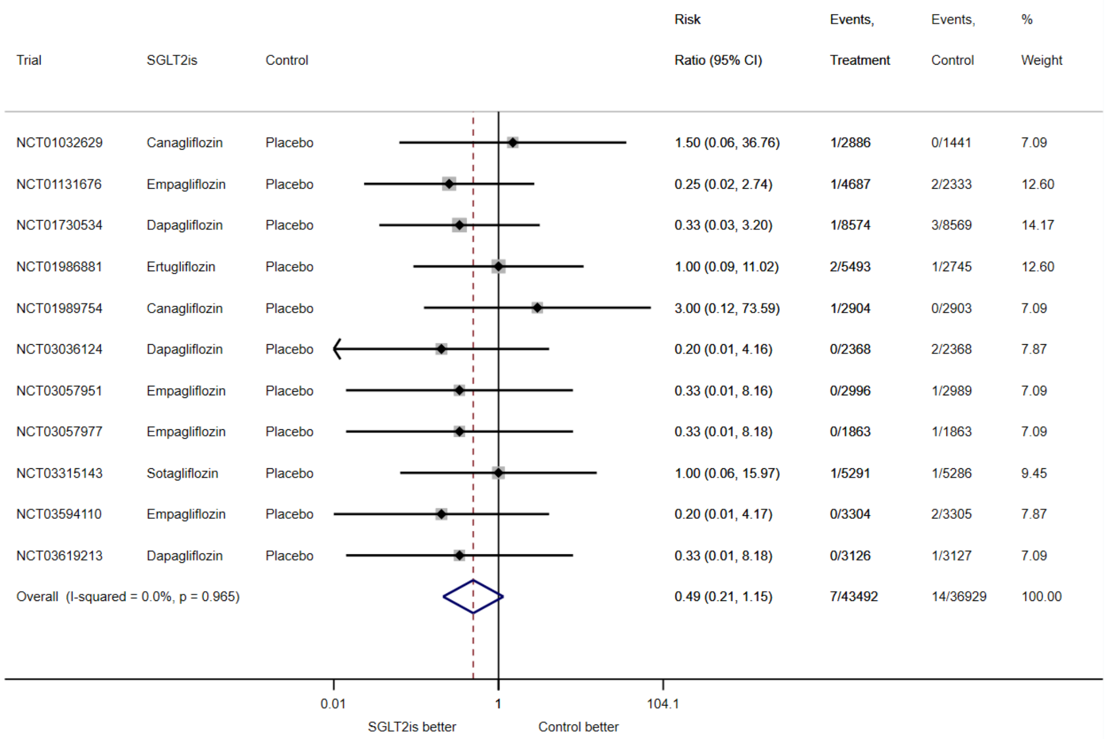

System 11-Infections And Infestations-Figure S121 Meta-analysis of SGLT2is and Pulmonary tuberculosis

Trial	SGLT2is	Control	Risk Ratio (95% CI)	Events, Treatment	Events, Control	% Weight
NCT01032629	Canagliflozin	Placebo	0.50 (0.10, 2.47)	3/2886	3/1441	5.82
NCT01042977	Dapagliflozin	Placebo	0.33 (0.01, 8.18)	0/482	1/483	1.45
NCT01131676	Empagliflozin	Placebo	1.24 (0.24, 6.41)	5/4687	2/2333	5.53
NCT01730534	Dapagliflozin	Placebo	1.00 (0.48, 2.10)	14/8574	14/8569	27.14
NCT01986881	Ertugliflozin	Placebo	1.50 (0.16, 14.41)	3/5493	1/2745	2.90
NCT01989754	Canagliflozin	Placebo	2.50 (0.49, 12.87)	5/2904	2/2903	5.54
NCT02065791	Canagliflozin	Placebo	0.60 (0.14, 2.50)	3/2200	5/2197	7.27
NCT03036124	Dapagliflozin	Placebo	1.00 (0.06, 15.98)	1/2368	1/2368	1.94
NCT03036150	Dapagliflozin	Placebo	1.00 (0.14, 7.09)	2/2149	2/2149	3.87
NCT03057951	Empagliflozin	Placebo	1.50 (0.25, 8.95)	3/2996	2/2989	4.65
NCT03057977	Empagliflozin	Placebo	3.00 (0.12, 73.59)	1/1863	0/1863	1.45
NCT03315143	Sotagliflozin	Placebo	1.00 (0.42, 2.40)	10/5291	10/5286	19.39
NCT03521934	Sotagliflozin	Placebo	2.02 (0.18, 22.22)	2/605	1/611	2.59
NCT03594110	Empagliflozin	Placebo	1.00 (0.20, 4.95)	3/3304	3/3305	5.81
NCT03619213	Dapagliflozin	Placebo	0.67 (0.11, 3.99)	2/3126	3/3127	4.65
Overall (I-squared = 0.0%, p = 0.994)			1.01 (0.69, 1.49)	57/48928	50/42369	100.00

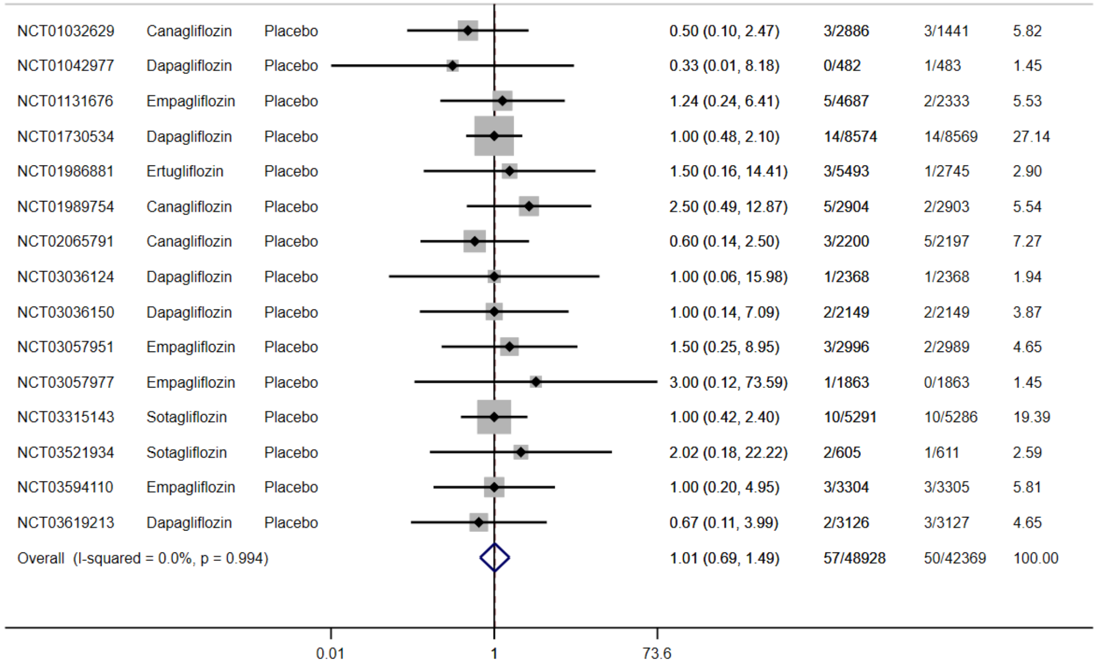

System 11-Infections And Infestations-Figure S122 Meta-analysis of SGLT2is and Pyelonephritis

Trial	SGLT2is	Control	Risk Ratio (95% CI)	Events, Treatment	Events, Control	% Weight
NCT01032629	Canagliflozin	Placebo	1.50 (0.06, 36.76)	1/2886	0/1441	1.89
NCT01131676	Empagliflozin	Placebo	0.41 (0.13, 1.36)	5/4687	6/2333	13.78
NCT01730534	Dapagliflozin	Placebo	0.67 (0.30, 1.48)	10/8574	15/8569	30.31
NCT01986881	Ertugliflozin	Placebo	2.50 (0.12, 52.04)	2/5493	0/2745	2.10
NCT01989754	Canagliflozin	Placebo	0.33 (0.03, 3.20)	1/2904	3/2903	3.79
NCT02065791	Canagliflozin	Placebo	2.00 (0.37, 10.89)	4/2200	2/2197	6.73
NCT03036124	Dapagliflozin	Placebo	5.00 (0.24, 104.09)	2/2368	0/2368	2.10
NCT03036150	Dapagliflozin	Placebo	2.50 (0.49, 12.87)	5/2149	2/2149	7.22
NCT03057951	Empagliflozin	Placebo	2.33 (0.60, 8.99)	7/2996	3/2989	10.61
NCT03057977	Empagliflozin	Placebo	0.20 (0.01, 4.16)	0/1863	2/1863	2.10
NCT03315143	Sotagliflozin	Placebo	1.75 (0.51, 5.97)	7/5291	4/5286	12.85
NCT03521934	Sotagliflozin	Placebo	3.03 (0.12, 74.22)	1/605	0/611	1.89
NCT03594110	Empagliflozin	Placebo	1.00 (0.06, 15.99)	1/3304	1/3305	2.52
NCT03619213	Dapagliflozin	Placebo	5.00 (0.24, 104.14)	2/3126	0/3127	2.10
Overall (I-squared = 0.0%, p = 0.514)			1.07 (0.69, 1.67)	48/48446	38/41886	100.00

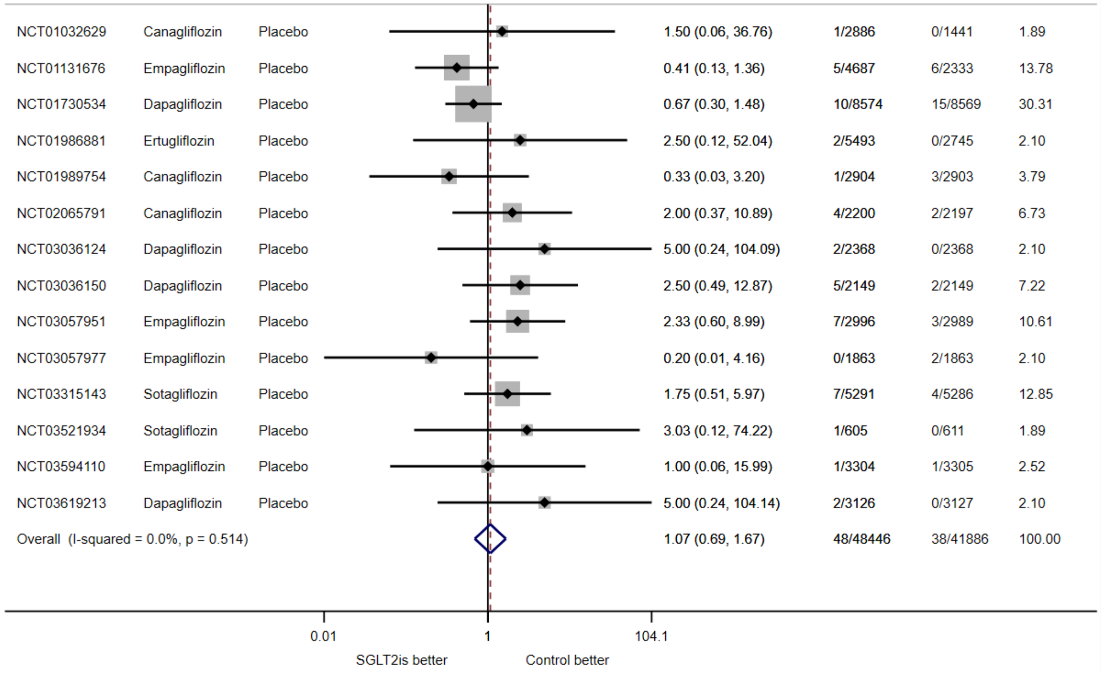

System 11-Infections And Infestations-Figure S123 Meta-analysis of SGLT2is and Pyelonephritis acute

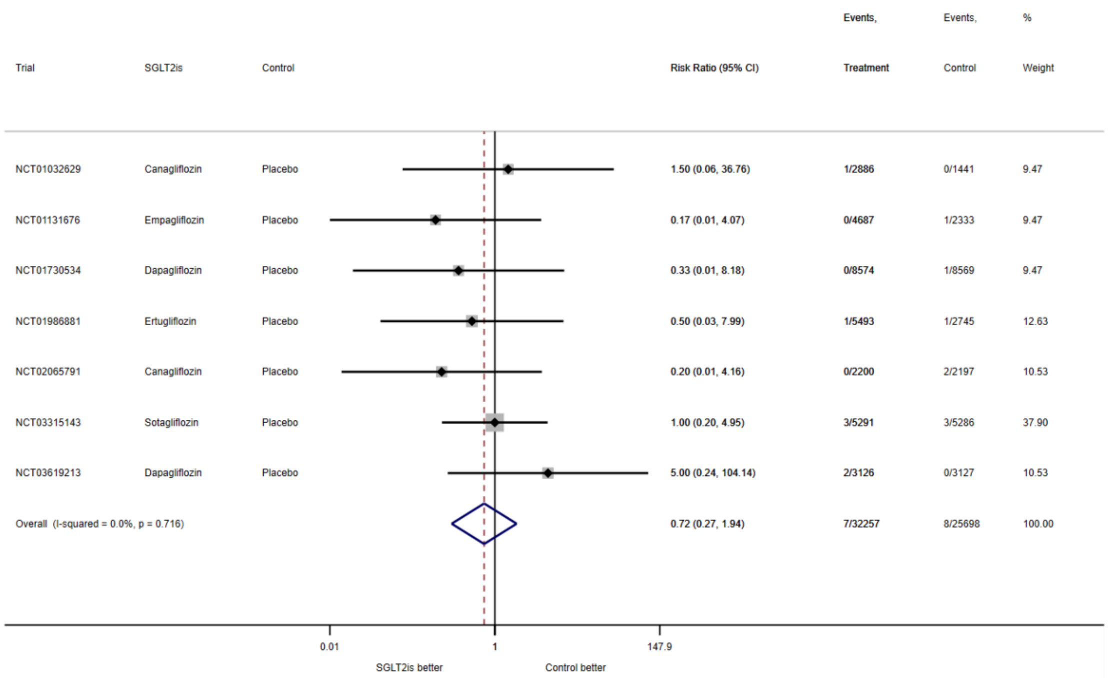

System 11-Infections And Infestations-Figure S124 Meta-analysis of SGLT2is and Pyelonephritis chronic

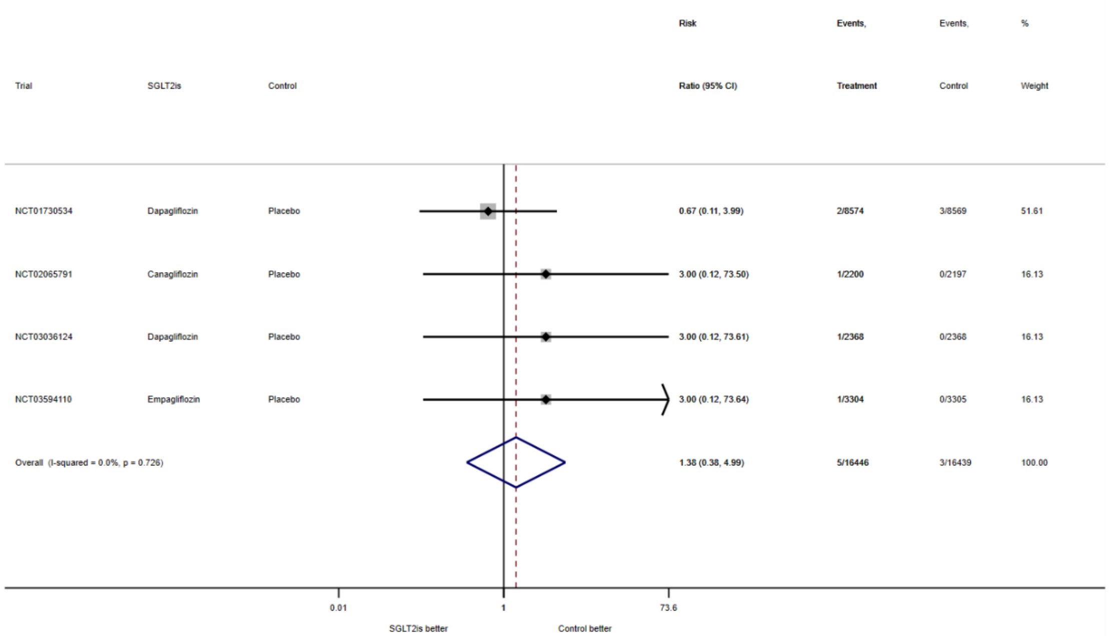

System 11-Infections And Infestations-Figure S125 Meta-analysis of SGLT2is and Rectal abscess

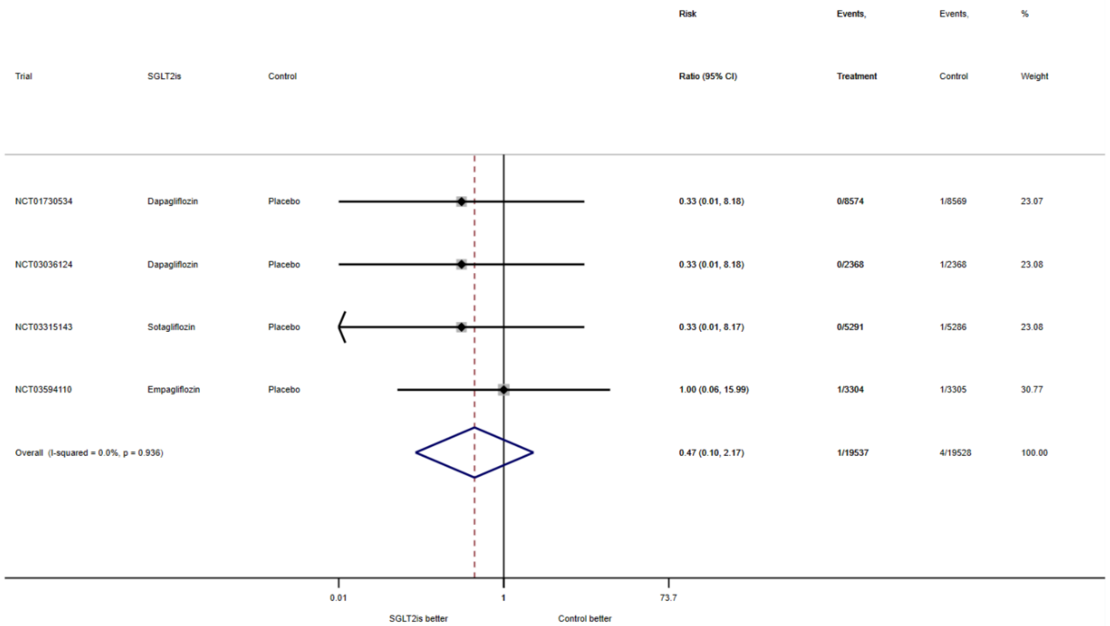

System 11-Infections And Infestations-Figure S126 Meta-analysis of SGLT2is and Renal abscess

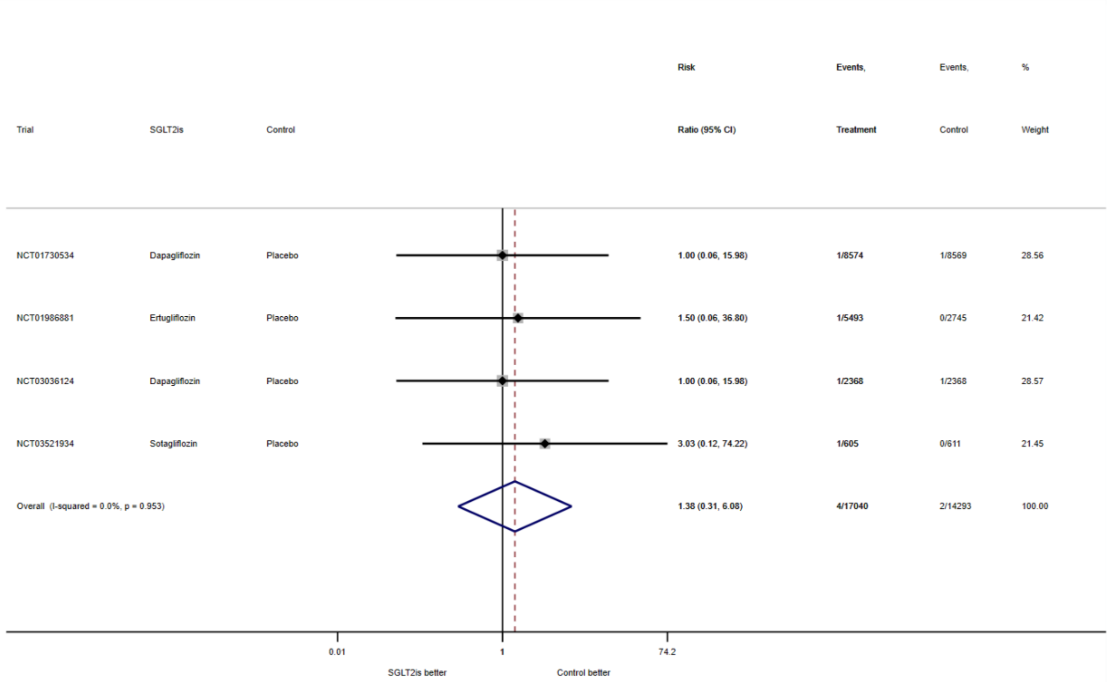

System 11-Infections And Infestations-Figure S127 Meta-analysis of SGLT2is and Respiratory syncytial virus infection

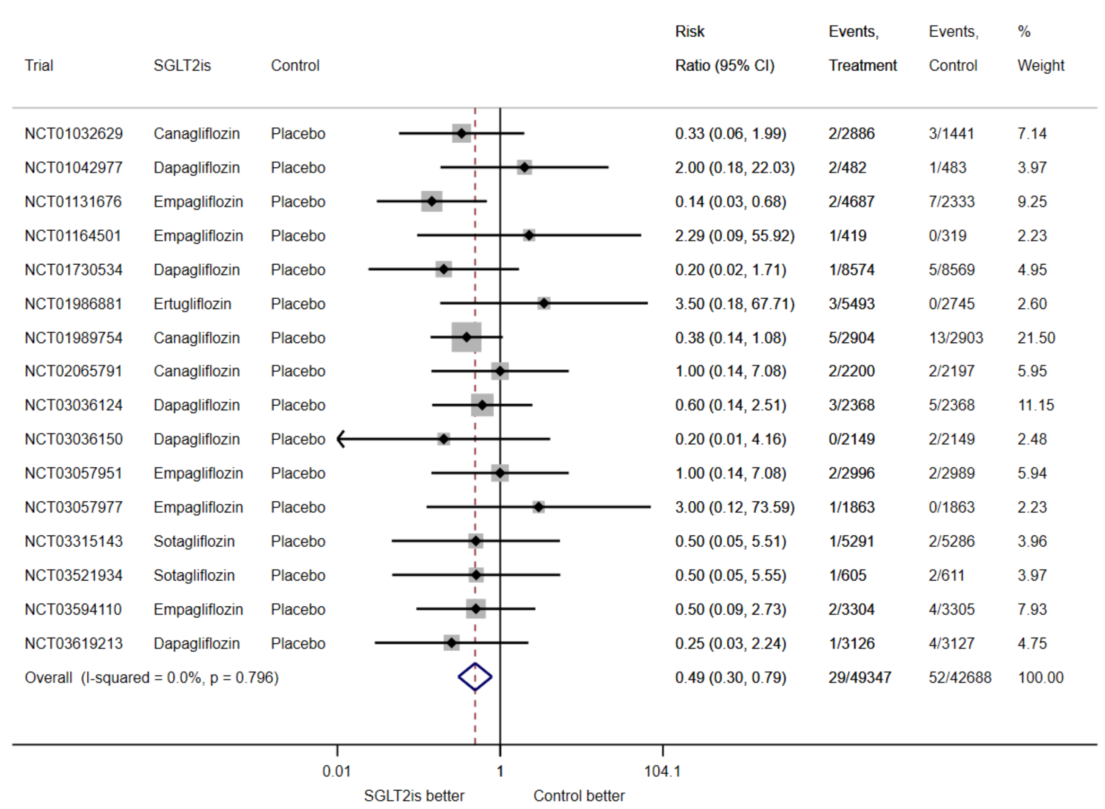

System 11-Infections And Infestations-Figure S128 Meta-analysis of SGLT2is and Respiratory tract infection

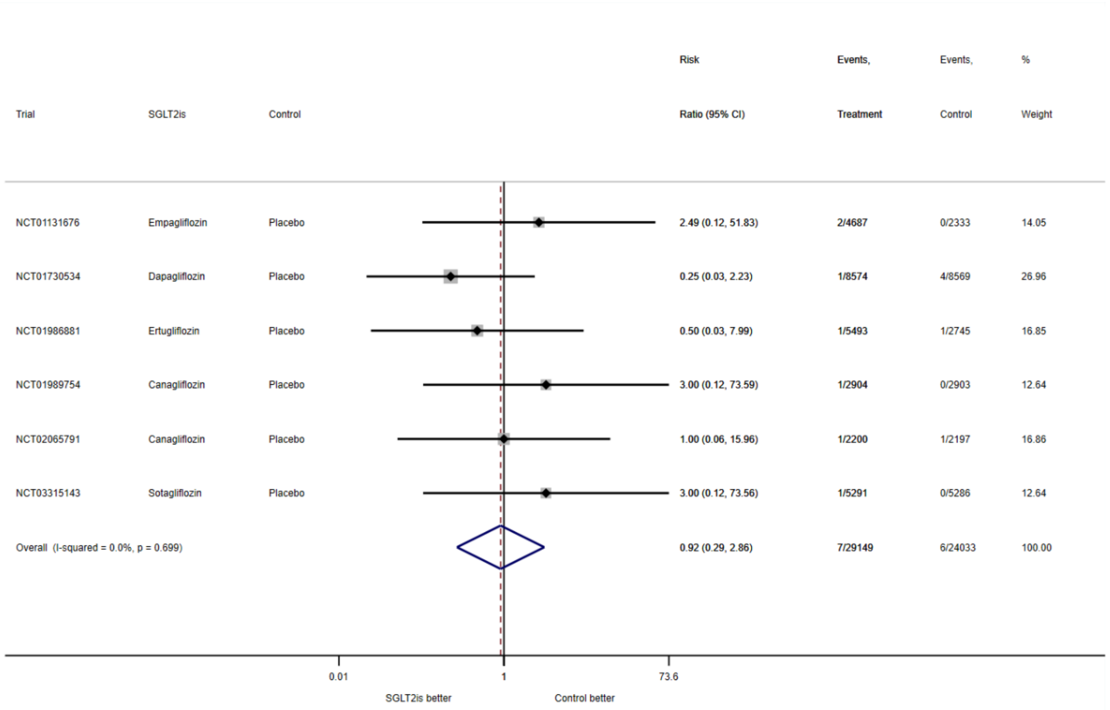

System 11-Infections And Infestations-Figure S129 Meta-analysis of SGLT2is and Scrotal abscess

Trial	SGLT2is	Control	Risk Ratio (95% CI)	Events, Treatment	Events, Control	% Weight
NCT01032629	Canagliflozin	Placebo	0.80 (0.36, 1.76)	16/2886	10/1441	5.47
NCT01106651	Canagliflozin	Placebo	0.50 (0.03, 7.91)	1/477	1/237	0.44
NCT01106677	Canagliflozin	Sitagliptin	1.50 (0.06, 36.63)	1/735	0/366	0.33
NCT01131676	Empagliflozin	Placebo	1.29 (0.46, 3.63)	13/4687	5/2333	3.20
NCT01164501	Empagliflozin	Placebo	2.29 (0.09, 55.92)	1/419	0/319	0.33
NCT01730534	Dapagliflozin	Placebo	1.10 (0.80, 1.52)	77/8574	70/8569	32.69
NCT01986881	Ertugliflozin	Placebo	2.43 (1.08, 5.47)	34/5493	7/2745	5.15
NCT01989754	Canagliflozin	Placebo	0.44 (0.14, 1.44)	4/2904	9/2903	2.45
NCT02065791	Canagliflozin	Placebo	0.92 (0.40, 2.07)	11/2200	12/2197	5.10
NCT02384941	Sotagliflozin	Placebo	0.17 (0.01, 4.17)	0/525	1/268	0.33
NCT03036124	Dapagliflozin	Placebo	0.93 (0.44, 1.97)	13/2368	14/2368	5.99
NCT03036150	Dapagliflozin	Placebo	0.72 (0.35, 1.47)	13/2149	18/2149	6.72
NCT03057951	Empagliflozin	Placebo	1.24 (0.70, 2.19)	26/2996	21/2989	10.35
NCT03057977	Empagliflozin	Placebo	1.00 (0.38, 2.66)	8/1863	8/1863	3.55
NCT03315143	Sotagliflozin	Placebo	1.08 (0.49, 2.37)	13/5291	12/5286	5.53
NCT03521934	Sotagliflozin	Placebo	0.11 (0.01, 2.08)	0/605	4/611	0.40
NCT03594110	Empagliflozin	Placebo	1.33 (0.46, 3.84)	8/3304	6/3305	3.04
NCT03619213	Dapagliflozin	Placebo	0.80 (0.42, 1.54)	16/3126	20/3127	7.90
NCT04157751	Empagliflozin	Placebo	0.20 (0.01, 4.21)	0/260	2/264	0.37
NCT04350593	Dapagliflozin	Placebo	0.33 (0.03, 3.21)	1/613	3/616	0.66
Overall (I-squared = 0.0%, p = 0.703)			1.01 (0.84, 1.22)	256/51475	223/43956	100.00

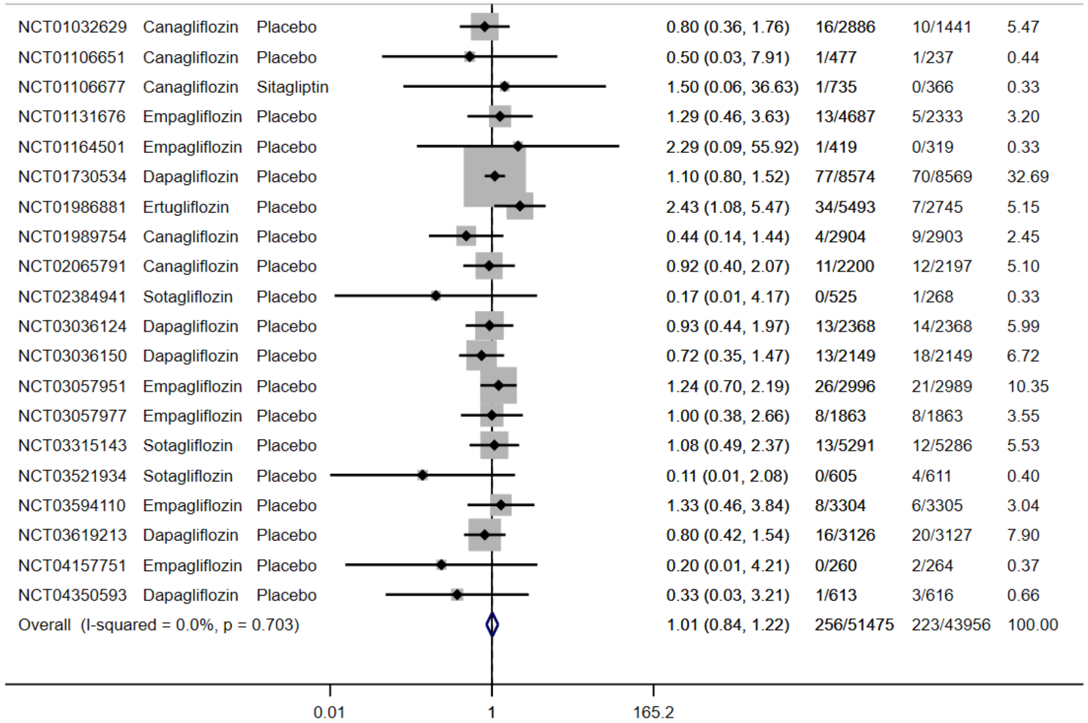

System 11-Infections And Infestations-Figure S130 Meta-analysis of SGLT2is and Sepsis

Trial	SGLT2is	Control	Risk Ratio (95% CI)	Events, Treatment	Events, Control	% Weight
NCT01031680	Dapagliflozin	Placebo	3.01 (0.12, 73.77)	1/460	0/462	0.62
NCT01032629	Canagliflozin	Placebo	0.33 (0.06, 1.99)	2/2886	3/1441	1.98
NCT01106677	Canagliflozin	Sitagliptin	0.17 (0.01, 4.07)	0/735	1/366	0.62
NCT01131676	Empagliflozin	Placebo	0.44 (0.16, 1.20)	7/4687	8/2333	6.15
NCT01730534	Dapagliflozin	Placebo	0.87 (0.49, 1.57)	21/8574	24/8569	18.47
NCT01986881	Ertugliflozin	Placebo	0.83 (0.30, 2.29)	10/5493	6/2745	6.18
NCT01989754	Canagliflozin	Placebo	0.67 (0.19, 2.36)	4/2904	6/2903	3.95
NCT02065791	Canagliflozin	Placebo	0.42 (0.15, 1.18)	5/2200	12/2197	5.82
NCT03036124	Dapagliflozin	Placebo	1.00 (0.38, 2.66)	8/2368	8/2368	6.60
NCT03036150	Dapagliflozin	Placebo	1.00 (0.43, 2.30)	11/2149	11/2149	9.09
NCT03057951	Empagliflozin	Placebo	1.00 (0.43, 2.30)	11/2996	11/2989	9.08
NCT03057977	Empagliflozin	Placebo	1.80 (0.60, 5.36)	9/1863	5/1863	5.30
NCT03242252	Sotagliflozin	Placebo	1.48 (0.06, 36.28)	1/527	0/260	0.62
NCT03315143	Sotagliflozin	Placebo	0.75 (0.17, 3.35)	3/5291	4/5286	2.82
NCT03521934	Sotagliflozin	Placebo	0.50 (0.09, 2.75)	2/605	4/611	2.20
NCT03594110	Empagliflozin	Placebo	2.00 (0.18, 22.05)	2/3304	1/3305	1.10
NCT03619213	Dapagliflozin	Placebo	1.00 (0.51, 1.96)	17/3126	17/3127	14.05
NCT04157751	Empagliflozin	Placebo	1.02 (0.06, 16.15)	1/260	1/264	0.83
NCT04350593	Dapagliflozin	Placebo	0.84 (0.26, 2.73)	5/613	6/616	4.52
Overall (I-squared = 0.0%, p = 0.940)			0.84 (0.66, 1.08)	120/51041	128/43854	100.00

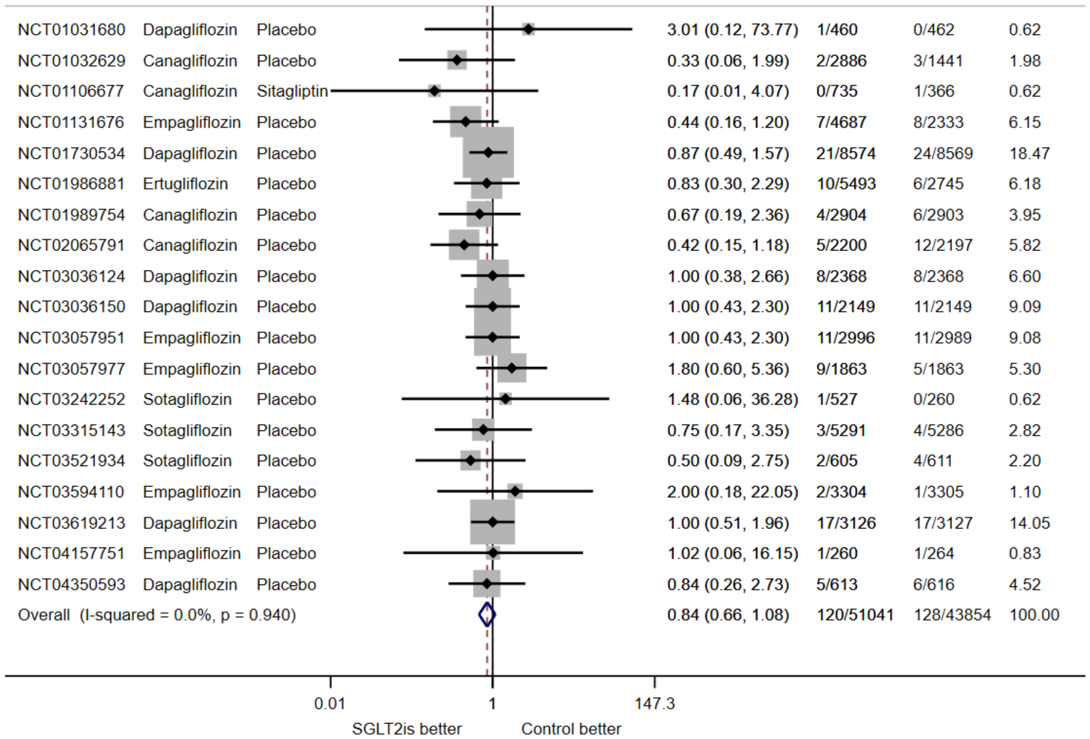

System 11-Infections And Infestations-Figure S131 Meta-analysis of SGLT2is and Septic shock

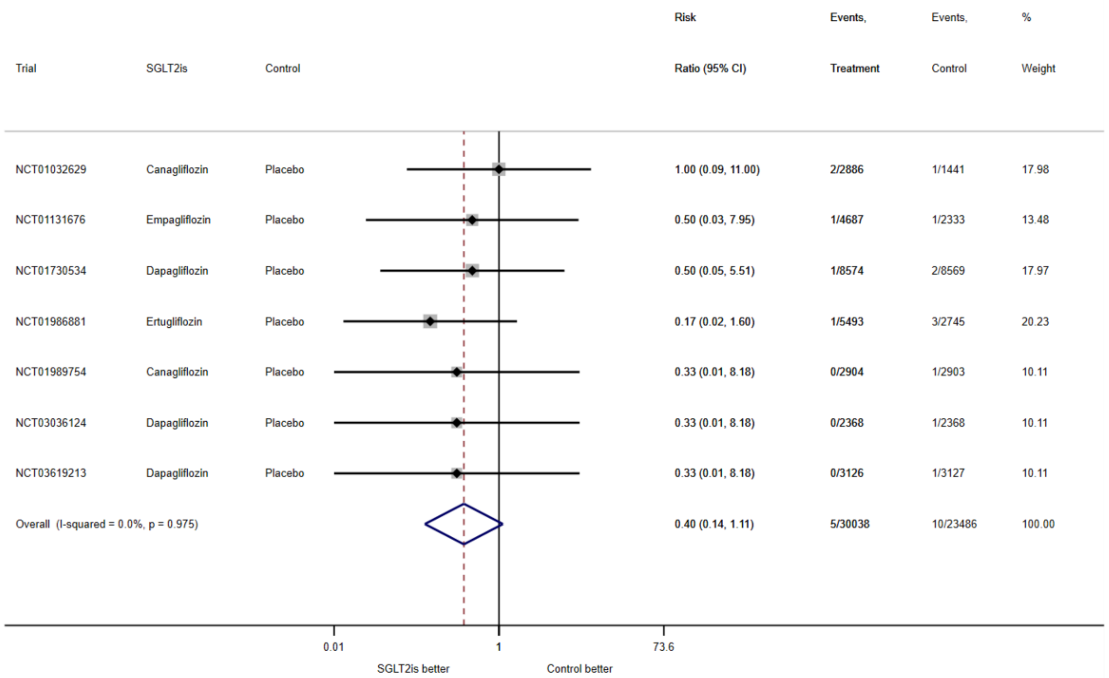

System 11-Infections And Infestations-Figure S132 Meta-analysis of SGLT2is and Sinusitis

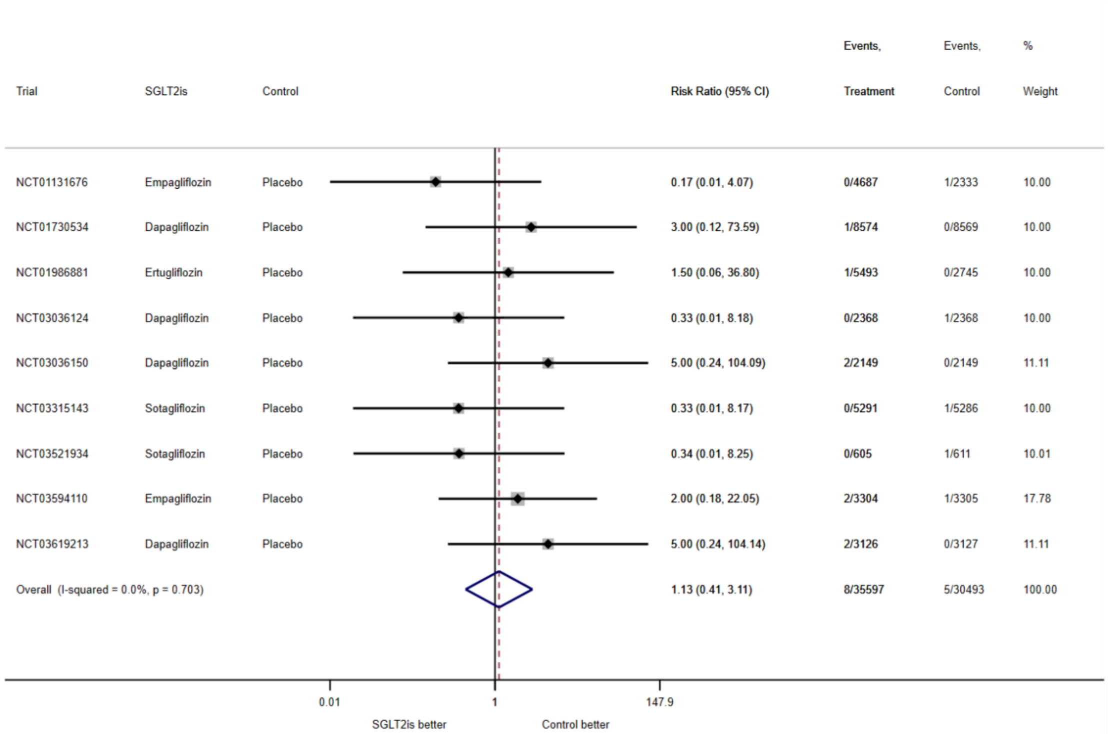

System 11-Infections And Infestations-Figure S133 Meta-analysis of SGLT2is and Skin infection

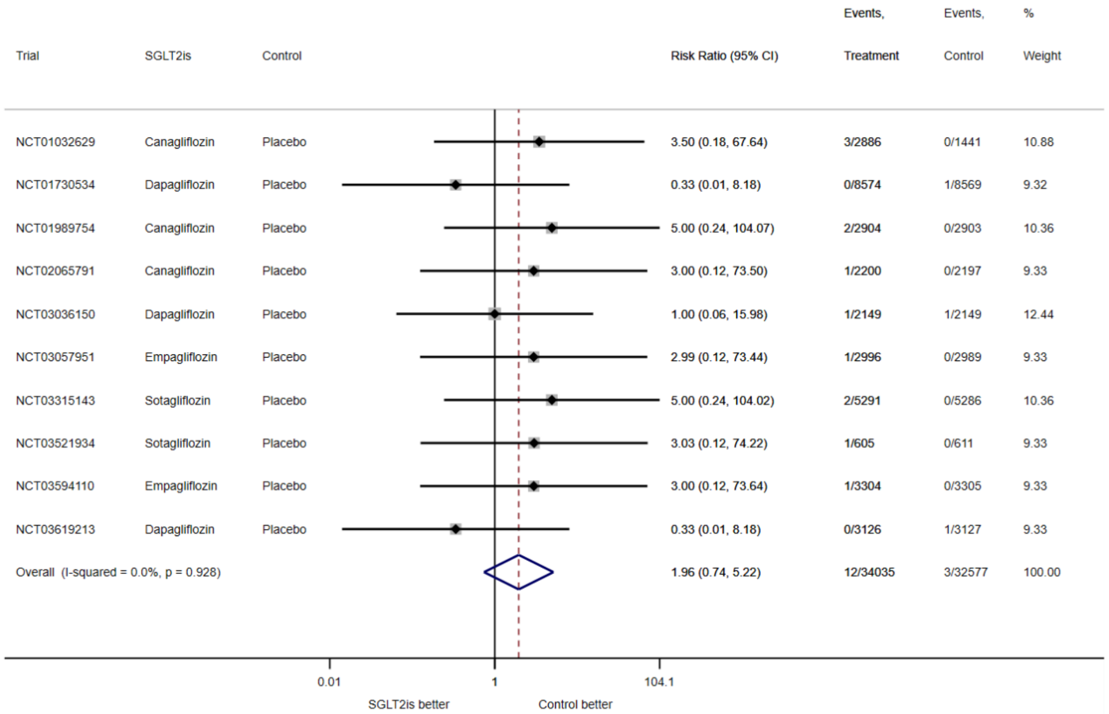

System 11-Infections And Infestations-Figure S134 Meta-analysis of SGLT2is and Soft tissue infection

Trial	SGLT2is	Control	Risk Ratio (95% CI)	Events, Treatment	Events, Control	% Weight
NCT01730534	Dapagliflozin	Placebo	0.67 (0.11, 3.99)	2/8574	3/8569	22.71
NCT01986881	Ertugliflozin	Placebo	0.25 (0.02, 2.75)	1/5493	2/2745	12.62
NCT02065791	Canagliflozin	Placebo	0.33 (0.01, 8.17)	0/2200	1/2197	7.10
NCT03036150	Dapagliflozin	Placebo	3.00 (0.12, 73.60)	1/2149	0/2149	7.10
NCT03057951	Empagliflozin	Placebo	0.50 (0.05, 5.50)	1/2996	2/2989	12.62
NCT03057977	Empagliflozin	Placebo	3.00 (0.12, 73.59)	1/1863	0/1863	7.10
NCT03315143	Sotagliflozin	Placebo	3.00 (0.12, 73.56)	1/5291	0/5286	7.09
NCT03521934	Sotagliflozin	Placebo	3.03 (0.12, 74.22)	1/605	0/611	7.10
NCT03619213	Dapagliflozin	Placebo	1.00 (0.06, 15.99)	1/3126	1/3127	9.46
NCT04157751	Empagliflozin	Placebo	0.34 (0.01, 8.27)	0/260	1/264	7.11
Overall (I-squared = 0.0%, p = 0.890)			0.82 (0.35, 1.93)	9/32557	10/29800	100.00

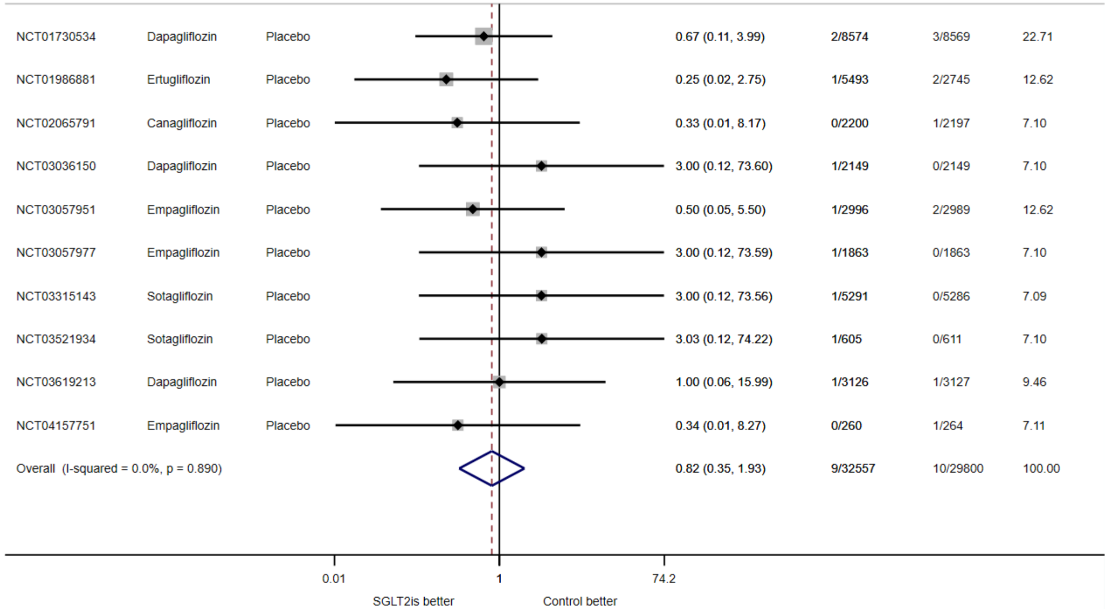

System 11-Infections And Infestations-Figure S135 Meta-analysis of SGLT2is and Staphylococcal bacteraemia

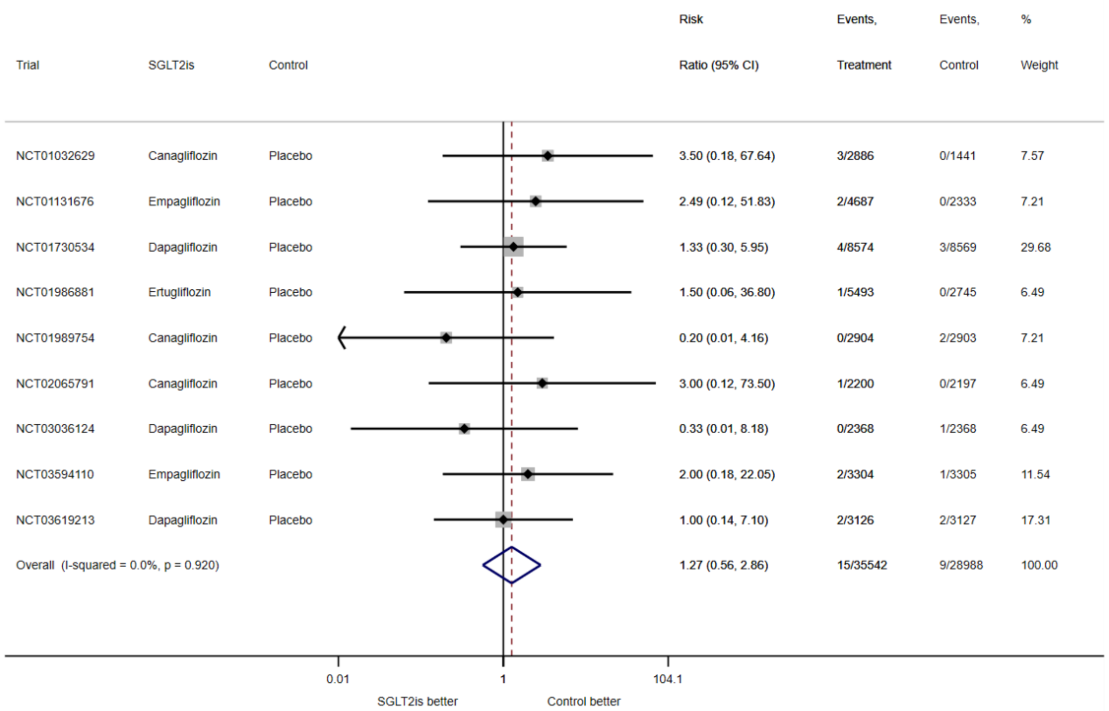

System 11-Infections And Infestations-Figure S136 Meta-analysis of SGLT2is and Staphylococcal infection

Trial	SGLT2is	Control	Risk Ratio (95% CI)	Events, Treatment	Events, Control	% Weight
NCT01032629	Canagliflozin	Placebo	4.50 (0.24, 83.44)	4/2886	0/1441	8.35
NCT01131676	Empagliflozin	Placebo	1.49 (0.06, 36.65)	1/4687	0/2333	6.96
NCT01730534	Dapagliflozin	Placebo	0.20 (0.02, 1.71)	1/8574	5/8569	15.46
NCT01986881	Ertugliflozin	Placebo	2.50 (0.12, 52.04)	2/5493	0/2745	7.73
NCT01989754	Canagliflozin	Placebo	3.00 (0.12, 73.59)	1/2904	0/2903	6.96
NCT02065791	Canagliflozin	Placebo	1.00 (0.14, 7.08)	2/2200	2/2197	18.57
NCT03036150	Dapagliflozin	Placebo	3.00 (0.12, 73.60)	1/2149	0/2149	6.96
NCT03057951	Empagliflozin	Placebo	0.14 (0.01, 2.76)	0/2996	3/2989	8.12
NCT03057977	Empagliflozin	Placebo	0.33 (0.01, 8.18)	0/1863	1/1863	6.96
NCT03315143	Sotagliflozin	Placebo	0.33 (0.01, 8.17)	0/5291	1/5286	6.96
NCT03594110	Empagliflozin	Placebo	0.33 (0.01, 8.18)	0/3304	1/3305	6.96
Overall (I-squared = 0.0%, p = 0.711)			0.77 (0.33, 1.79)	12/42347	13/35780	100.00

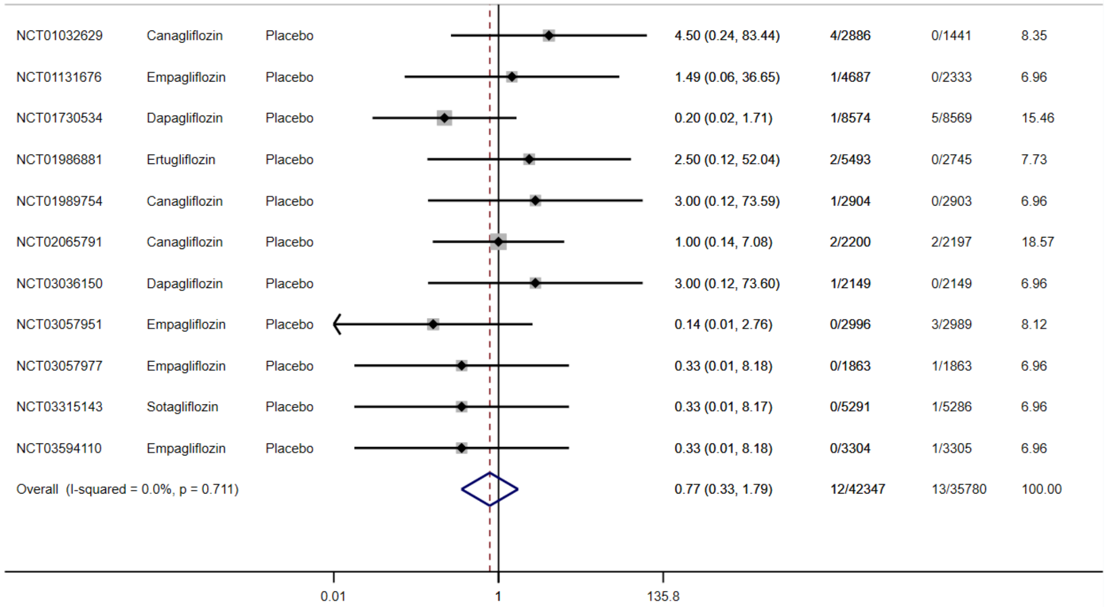

System 11-Infections And Infestations-Figure S137 Meta-analysis of SGLT2is and Staphylococcal sepsis

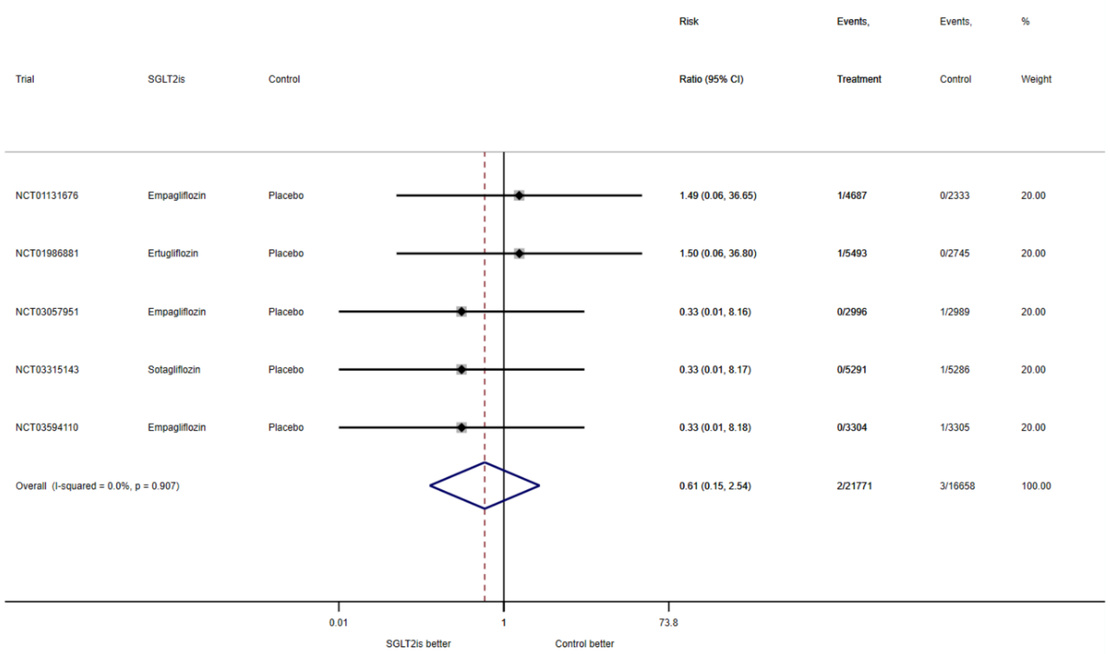

System 11-Infections And Infestations-Figure S138 Meta-analysis of SGLT2is and Staphylococcal skin infection

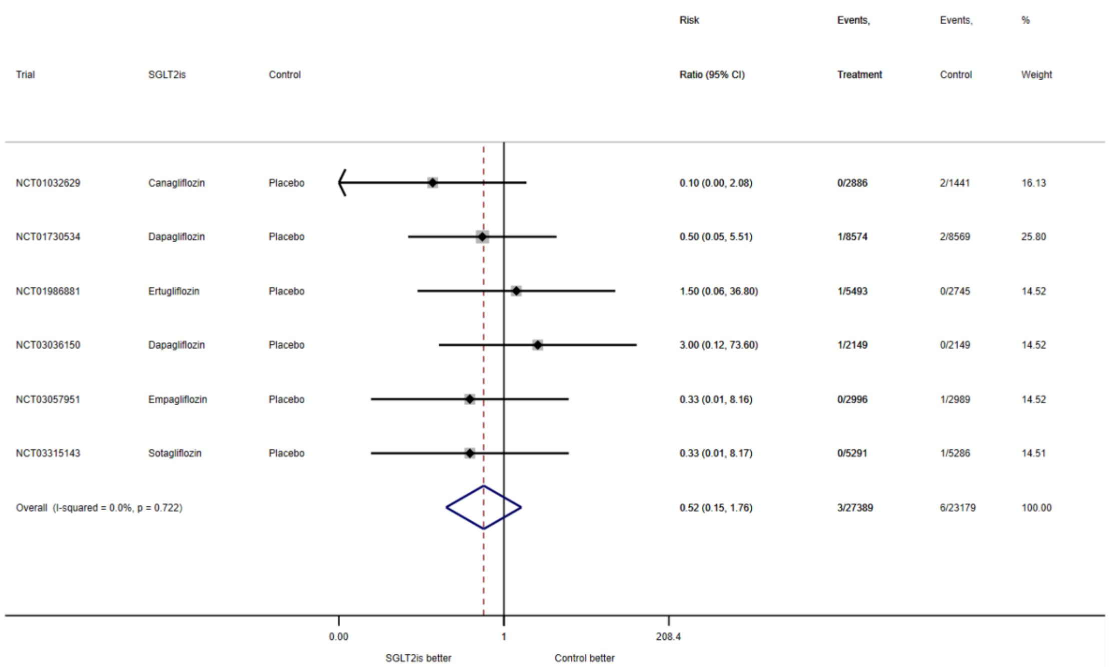

System 11-Infections And Infestations-Figure S139 Meta-analysis of SGLT2is and Streptococcal bacteraemia

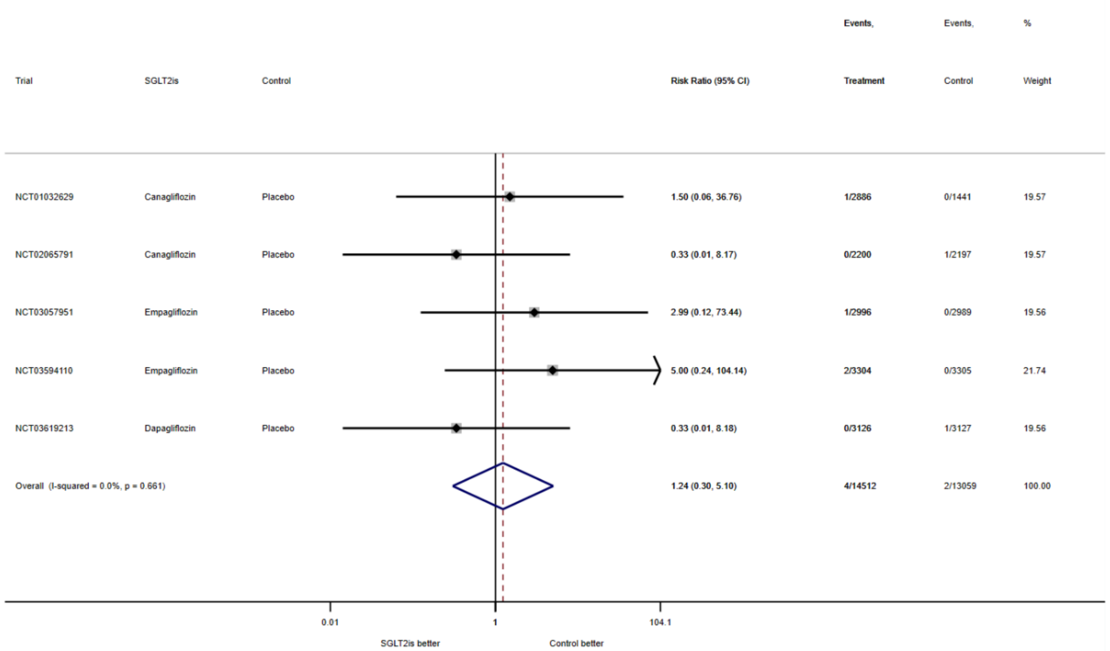

System 11-Infections And Infestations-Figure S140 Meta-analysis of SGLT2is and Streptococcal infection

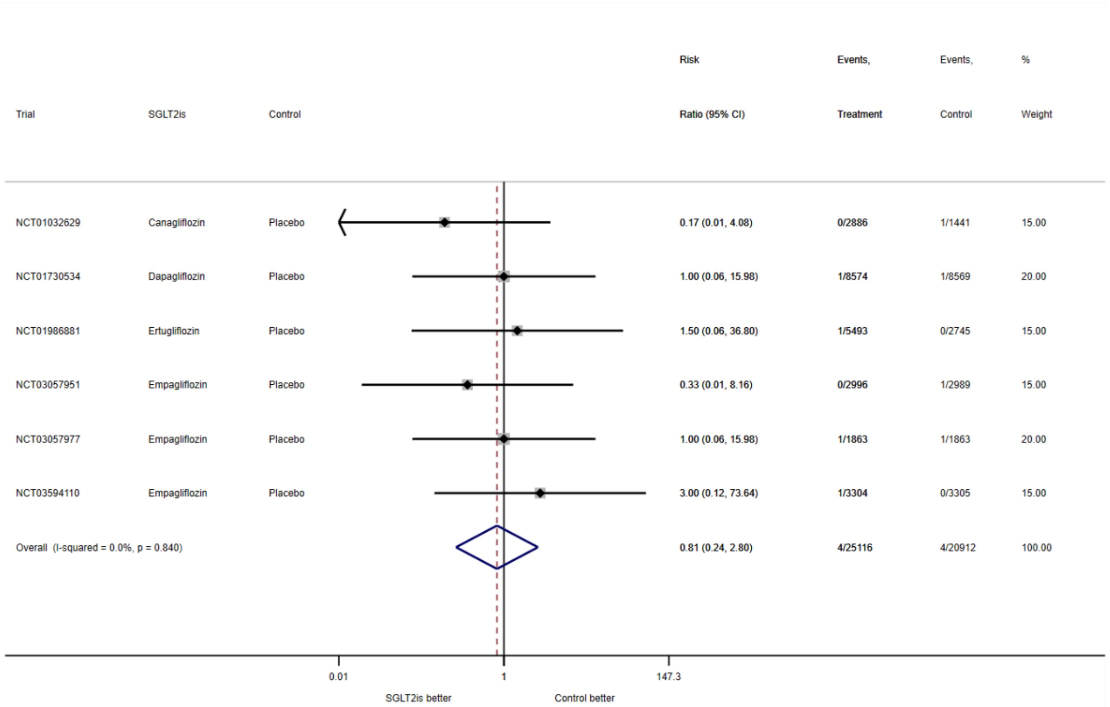

System 11-Infections And Infestations-Figure S141 Meta-analysis of SGLT2is and Streptococcal sepsis

Trial	SGLT2is	Control	Risk Ratio (95% CI)	Events, Treatment	Events, Control	% Weight
NCT01032629	Canagliflozin	Placebo	2.50 (0.12, 51.99)	2/2886	0/1441	7.98
NCT01106651	Canagliflozin	Placebo	1.49 (0.06, 36.53)	1/477	0/237	7.20
NCT01106677	Canagliflozin	Sitagliptin	1.50 (0.06, 36.63)	1/735	0/366	7.19
NCT01131676	Empagliflozin	Placebo	1.00 (0.09, 10.97)	2/4687	1/2333	12.78
NCT01989754	Canagliflozin	Placebo	0.20 (0.01, 4.16)	0/2904	2/2903	7.98
NCT02065791	Canagliflozin	Placebo	3.00 (0.12, 73.50)	1/2200	0/2197	7.19
NCT03036150	Dapagliflozin	Placebo	3.00 (0.12, 73.60)	1/2149	0/2149	7.19
NCT03315143	Sotagliflozin	Placebo	4.00 (0.45, 35.74)	4/5291	1/5286	15.33
NCT03594110	Empagliflozin	Placebo	0.50 (0.05, 5.51)	1/3304	2/3305	12.77
NCT03619213	Dapagliflozin	Placebo	3.00 (0.12, 73.64)	1/3126	0/3127	7.18
NCT04157751	Empagliflozin	Placebo	0.34 (0.01, 8.27)	0/260	1/264	7.20
Overall (I-squared = 0.0%, p = 0.905)			1.33 (0.56, 3.13)	14/28019	7/23608	100.00

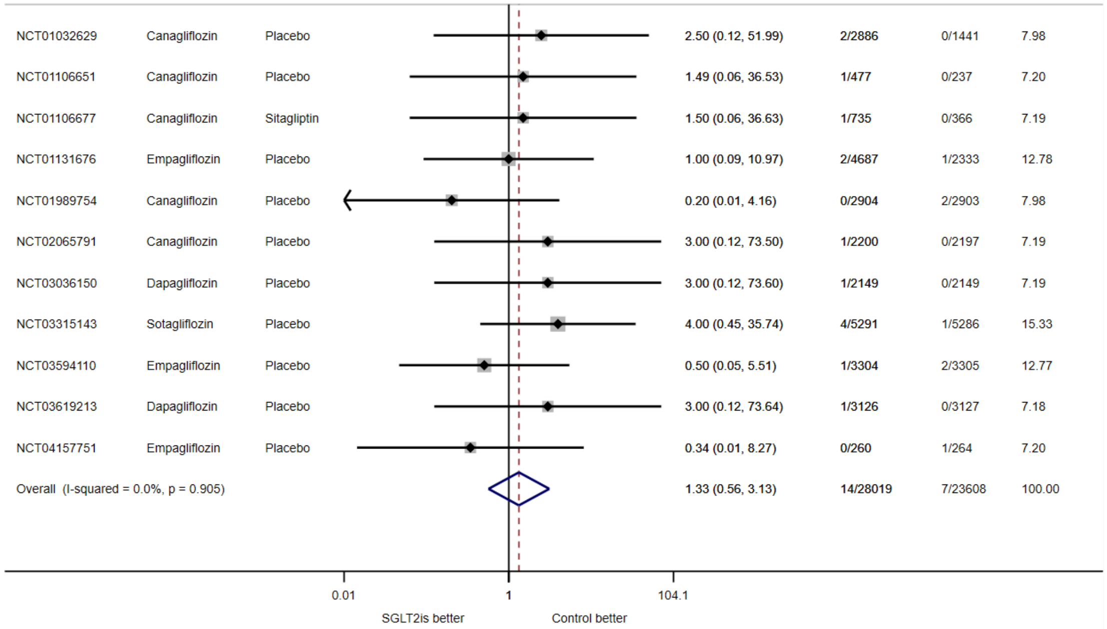

System 11-Infections And Infestations-Figure S142 Meta-analysis of SGLT2is and Subcutaneous abscess

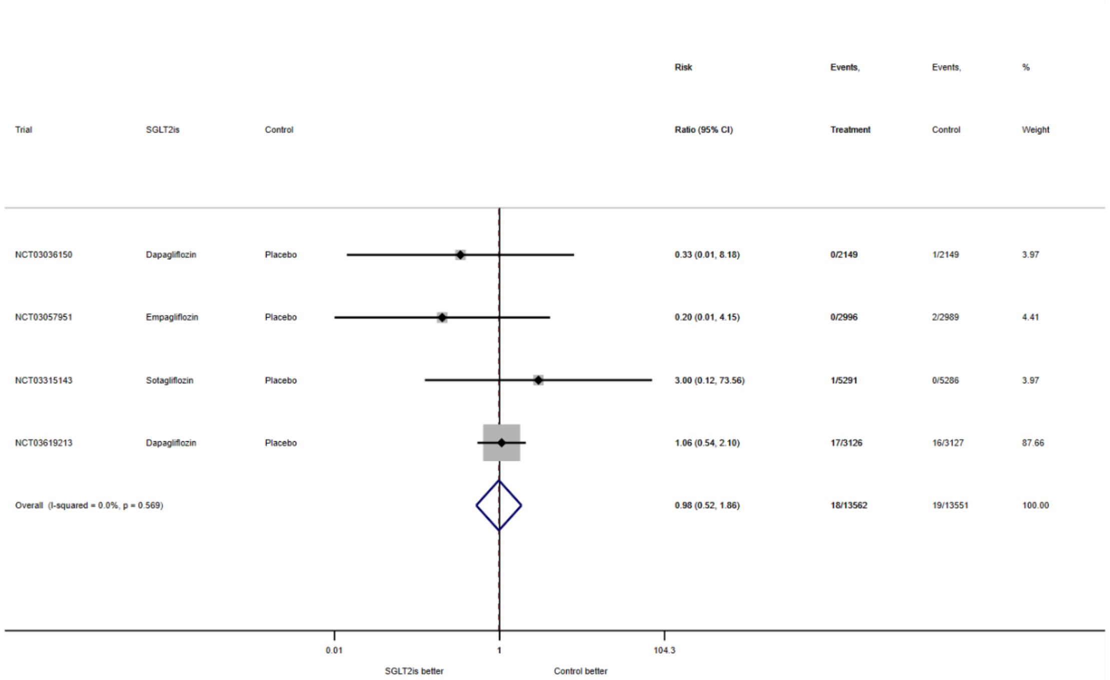

System 11-Infections And Infestations-Figure S143 Meta-analysis of SGLT2is and Suspected covid-19

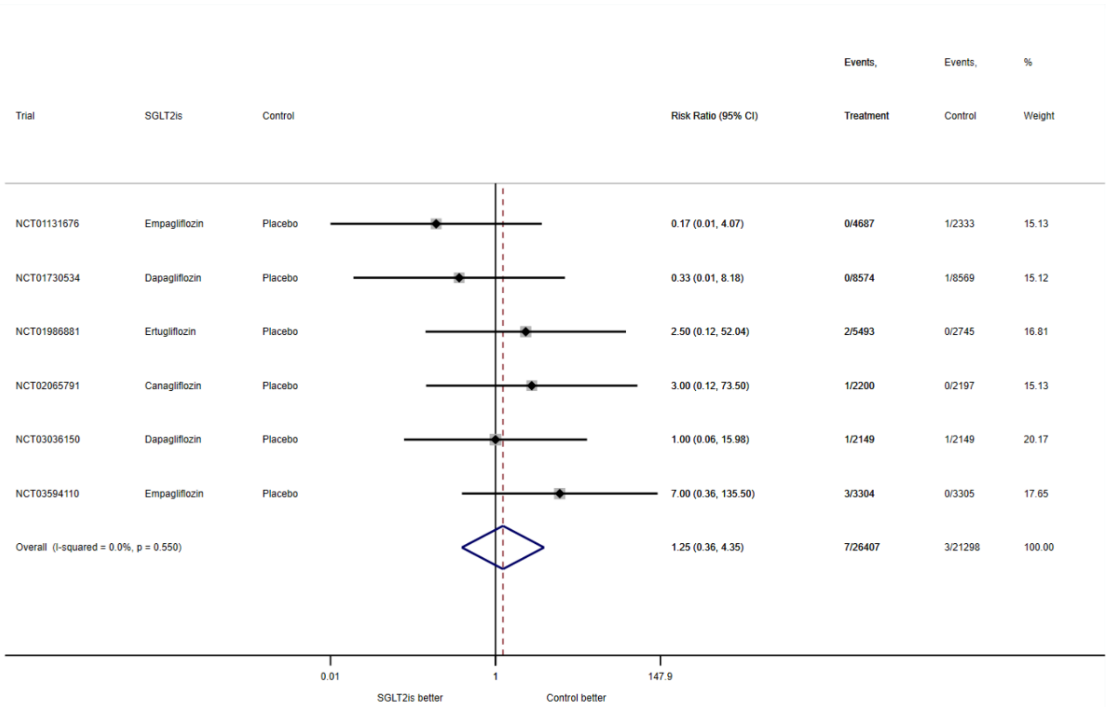

System 11-Infections And Infestations-Figure S144 Meta-analysis of SGLT2is and Tonsillitis

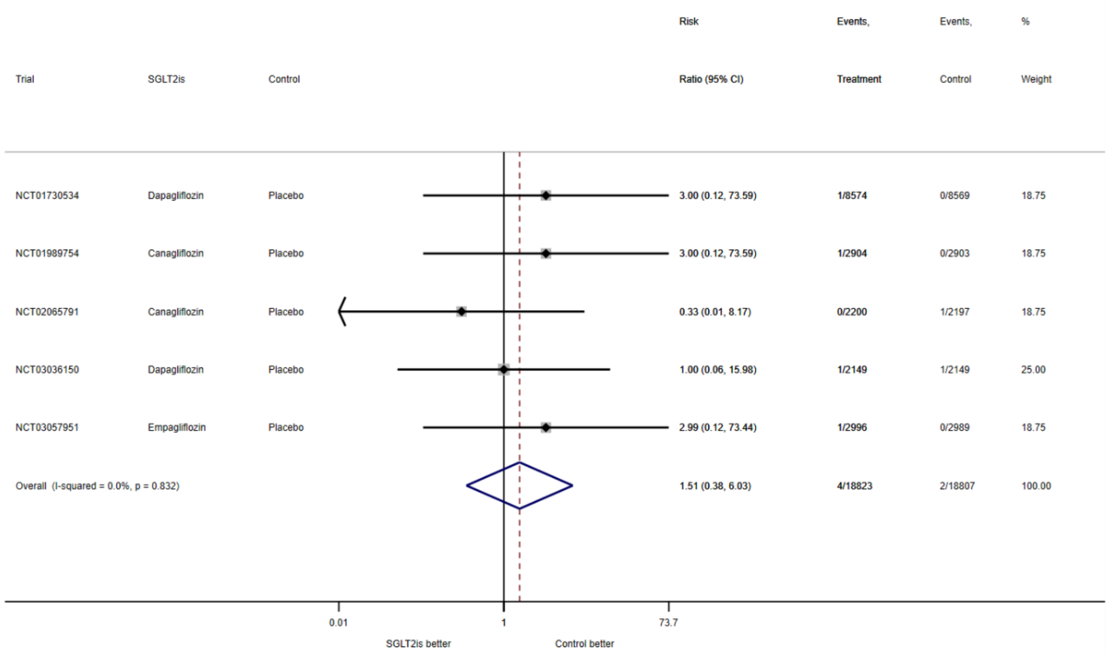

System 11-Infections And Infestations-Figure S145 Meta-analysis of SGLT2is and Tooth abscess

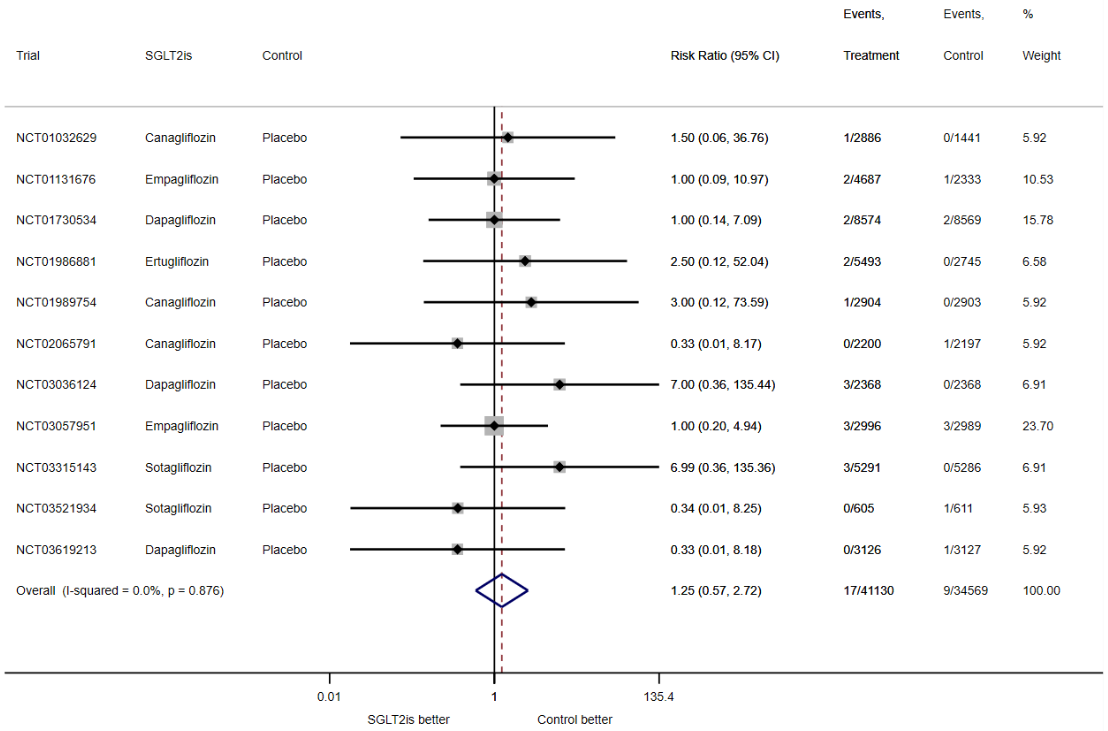

System 11-Infections And Infestations-Figure S146 Meta-analysis of SGLT2is and Tracheobronchitis

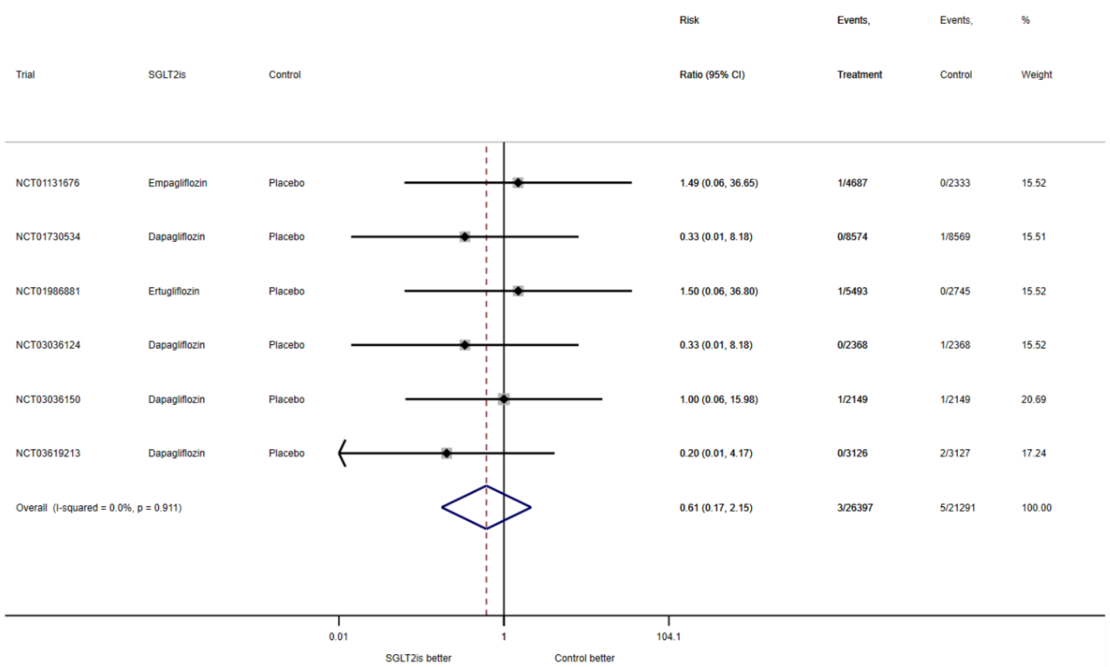

System 11-Infections And Infestations-Figure S147 Meta-analysis of SGLT2is and Tuberculosis

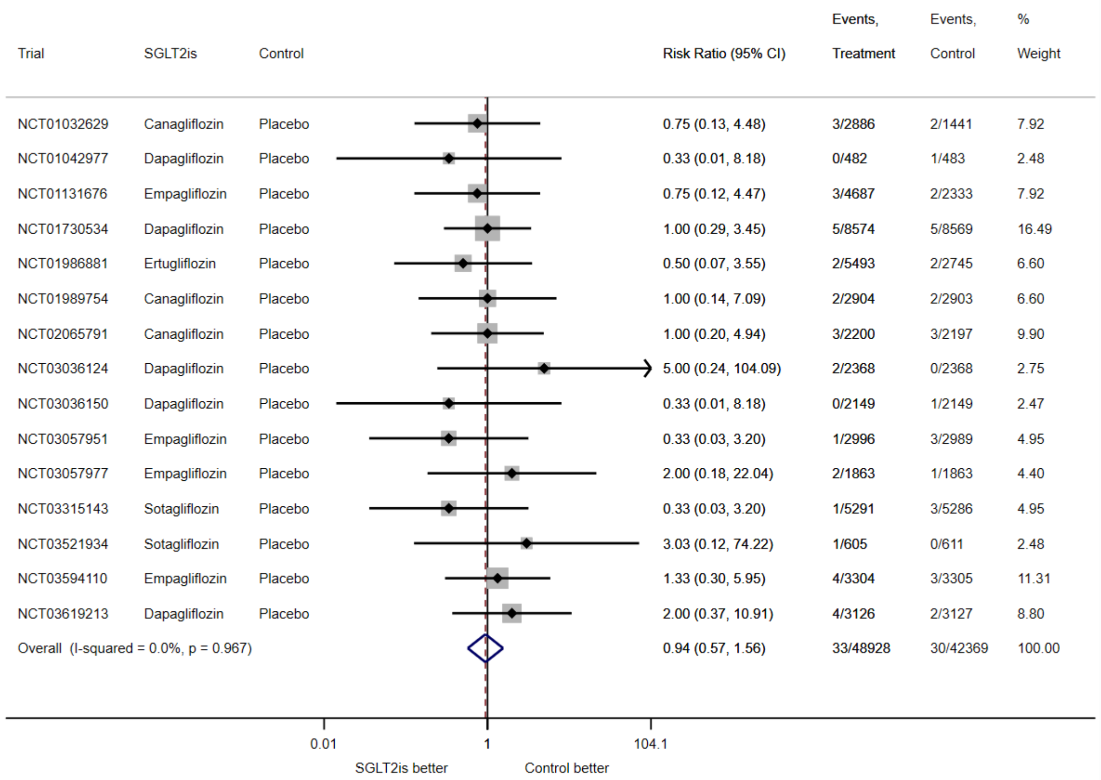

System 11-Infections And Infestations-Figure S148 Meta-analysis of SGLT2is and Upper respiratory tract infection

Trial	SGLT2is	Control	Risk Ratio (95% CI)	Events, Treatment	Events, Control	% Weight
NCT00528879	Dapagliflozin	Placebo	1.01 (0.04, 24.64)	1/409	0/137	0.24
NCT01031680	Dapagliflozin	Placebo	3.01 (0.12, 73.77)	1/460	0/462	0.24
NCT01032629	Canagliflozin	Placebo	0.66 (0.34, 1.25)	21/2886	16/1441	5.93
NCT01106651	Canagliflozin	Placebo	0.50 (0.03, 7.91)	1/477	1/237	0.32
NCT01106677	Canagliflozin	Sitagliptin	2.49 (0.12, 51.80)	2/735	0/366	0.27
NCT01131676	Empagliflozin	Placebo	0.90 (0.49, 1.66)	29/4687	16/2333	6.71
NCT01164501	Empagliflozin	Placebo	1.14 (0.19, 6.79)	3/419	2/319	0.78
NCT01730534	Dapagliflozin	Placebo	0.73 (0.51, 1.06)	49/8574	67/8569	18.41
NCT01986881	Ertugliflozin	Placebo	0.93 (0.49, 1.77)	26/5493	14/2745	5.91
NCT01989754	Canagliflozin	Placebo	0.85 (0.38, 1.88)	11/2904	13/2903	3.87
NCT02033889	Ertugliflozin	Placebo/Glimepiride	1.53 (0.06, 37.28)	1/412	0/209	0.24
NCT02065791	Canagliflozin	Placebo	1.19 (0.67, 2.12)	25/2200	21/2197	7.45
NCT03036124	Dapagliflozin	Placebo	0.65 (0.30, 1.38)	11/2368	17/2368	4.34
NCT03036150	Dapagliflozin	Placebo	1.33 (0.68, 2.60)	20/2149	15/2149	5.58
NCT03057951	Empagliflozin	Placebo	1.28 (0.78, 2.10)	36/2996	28/2989	10.29
NCT03057977	Empagliflozin	Placebo	1.40 (0.62, 3.14)	14/1863	10/1863	3.79
NCT03242252	Sotagliflozin	Placebo	3.46 (0.18, 66.74)	3/527	0/260	0.28
NCT03315143	Sotagliflozin	Placebo	0.80 (0.32, 2.02)	8/5291	10/5286	2.88
NCT03521934	Sotagliflozin	Placebo	0.34 (0.07, 1.66)	2/605	6/611	0.97
NCT03594110	Empagliflozin	Placebo	0.87 (0.52, 1.46)	27/3304	31/3305	9.41
NCT03619213	Dapagliflozin	Placebo	1.21 (0.77, 1.92)	40/3126	33/3127	11.82
NCT04157751	Empagliflozin	Placebo	3.05 (0.12, 74.43)	1/260	0/264	0.24
Overall (I-squared = 0.0%, p = 0.885)			0.95 (0.81, 1.12)	332/52145	300/44140	100.00

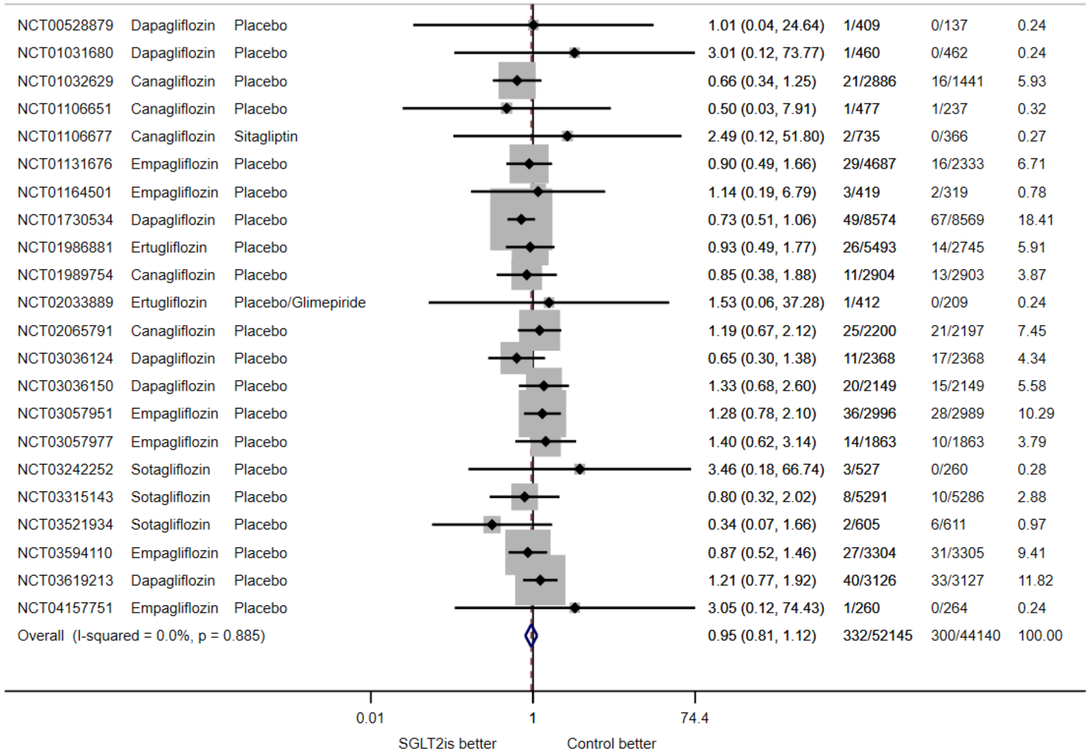

System 11-Infections And Infestations-Figure S149 Meta-analysis of SGLT2is and Urinary tract infection

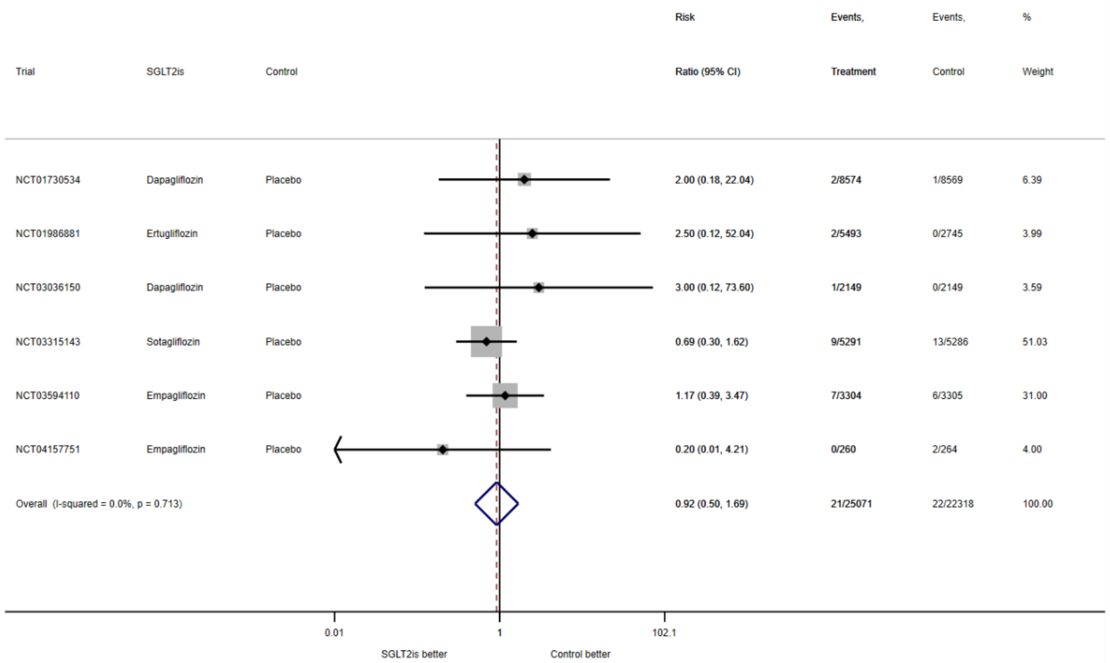

System 11-Infections And Infestations-Figure S150 Meta-analysis of SGLT2is and Urinary tract infection bacterial

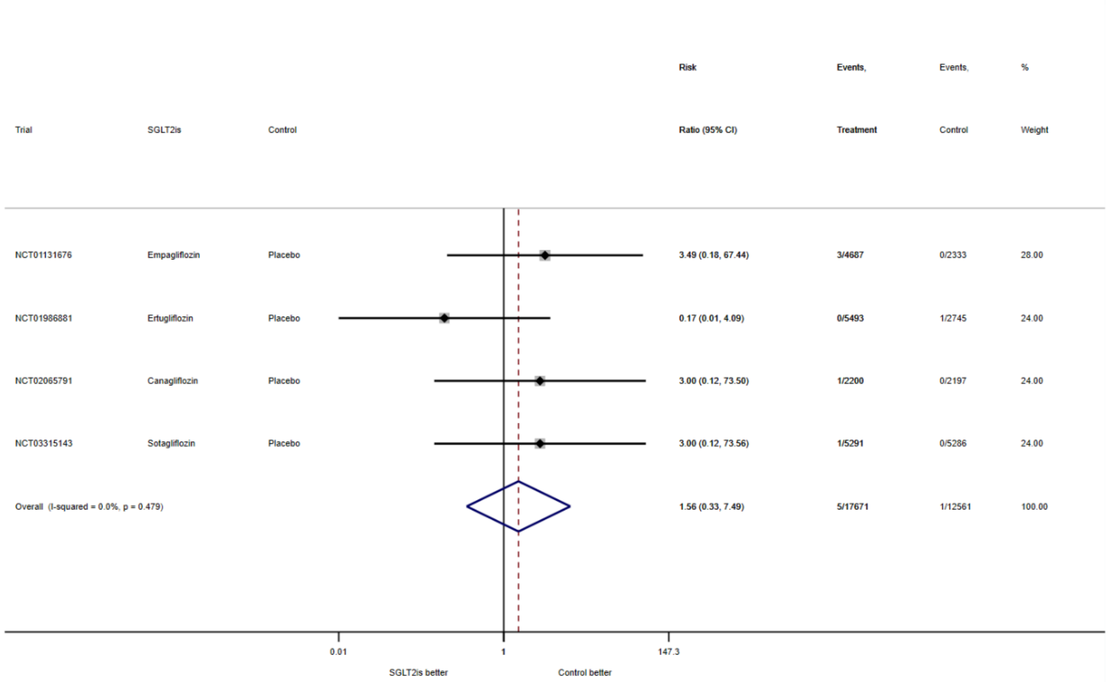

System 11-Infections And Infestations-Figure S151 Meta-analysis of SGLT2is and Urinary tract infection fungal

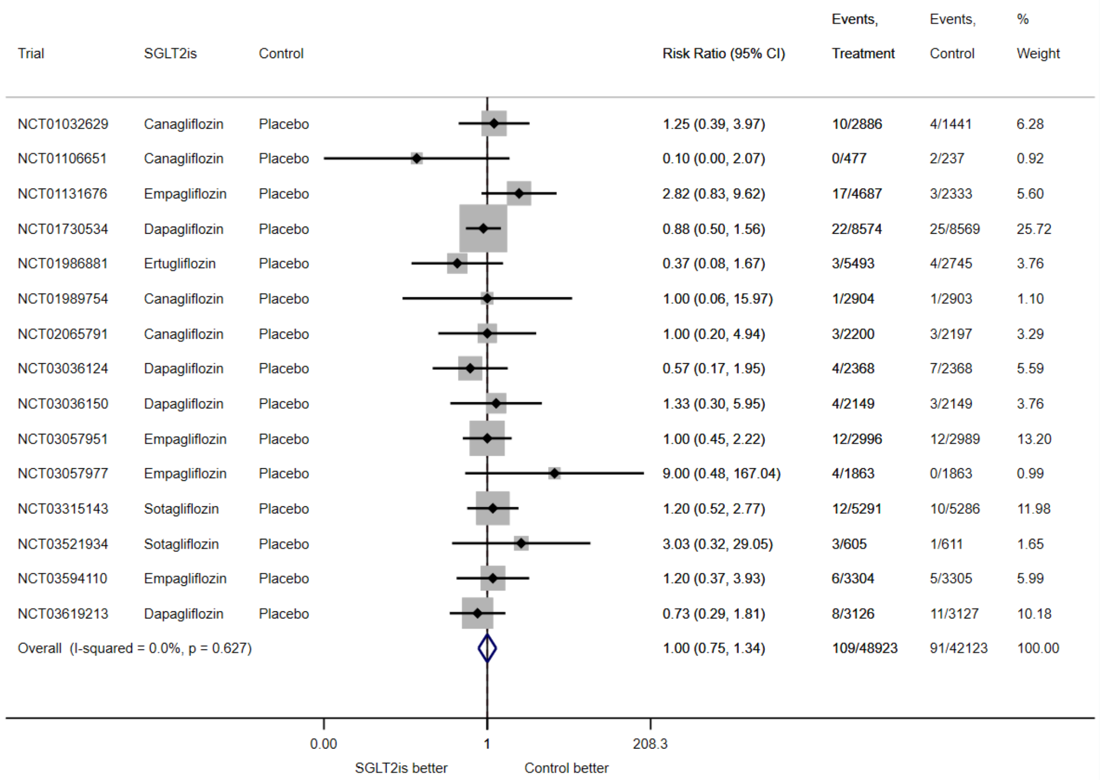

System 11-Infections And Infestations-Figure S152 Meta-analysis of SGLT2is and Urosepsis

Trial	SGLT2is	Control	Risk Ratio (95% CI)	Events, Treatment	Events, Control	% Weight
NCT01032629	Canagliflozin	Placebo	1.50 (0.06, 36.76)	1/2886	0/1441	14.06
NCT01131676	Empagliflozin	Placebo	0.25 (0.02, 2.74)	1/4687	2/2333	25.00
NCT01730534	Dapagliflozin	Placebo	0.33 (0.01, 8.18)	0/8574	1/8569	14.06
NCT01986881	Ertugliflozin	Placebo	0.17 (0.01, 4.09)	0/5493	1/2745	14.06
NCT03036150	Dapagliflozin	Placebo	0.33 (0.01, 8.18)	0/2149	1/2149	14.06
NCT03315143	Sotagliflozin	Placebo	1.00 (0.06, 15.97)	1/5291	1/5286	18.75
Overall (I-squared = 0.0%, p = 0.910)			0.43 (0.13, 1.42)	3/29080	6/22523	100.00

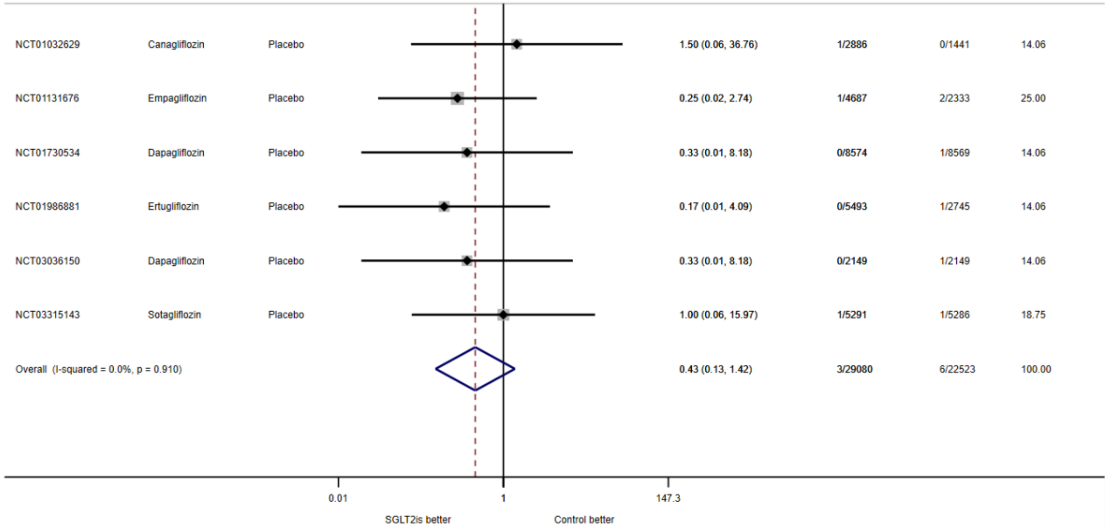

System 11-Infections And Infestations-Figure S153 Meta-analysis of SGLT2is and Vestibular neuronitis

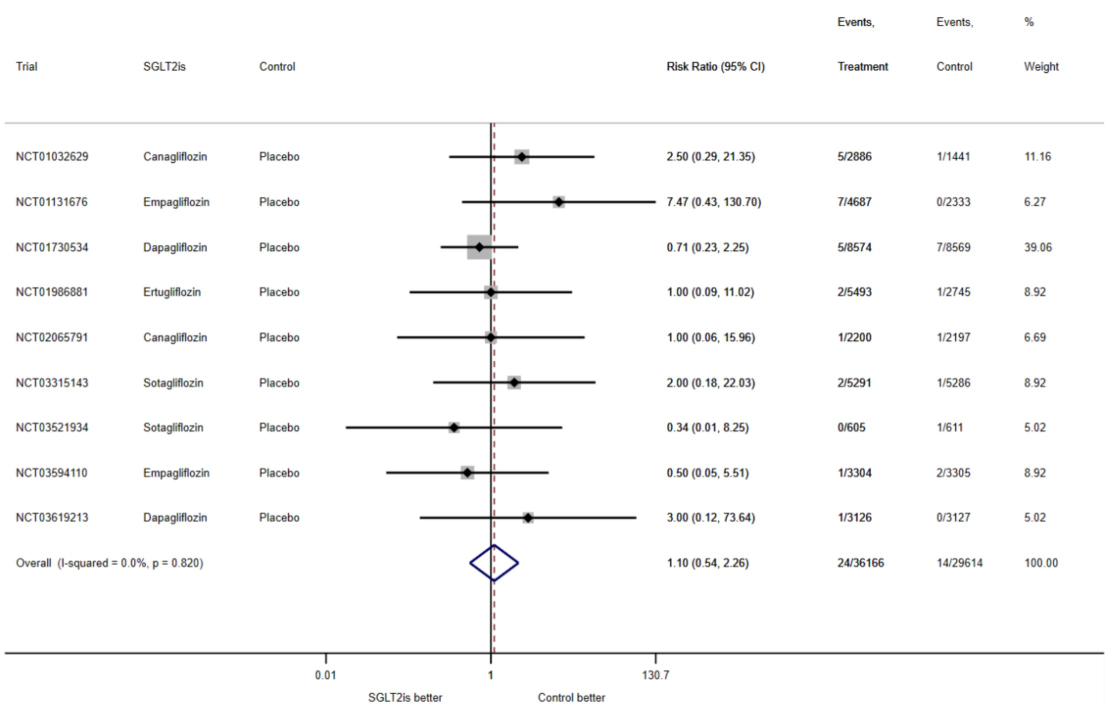

System 11-Infections And Infestations-Figure S154 Meta-analysis of SGLT2is and Viral infection

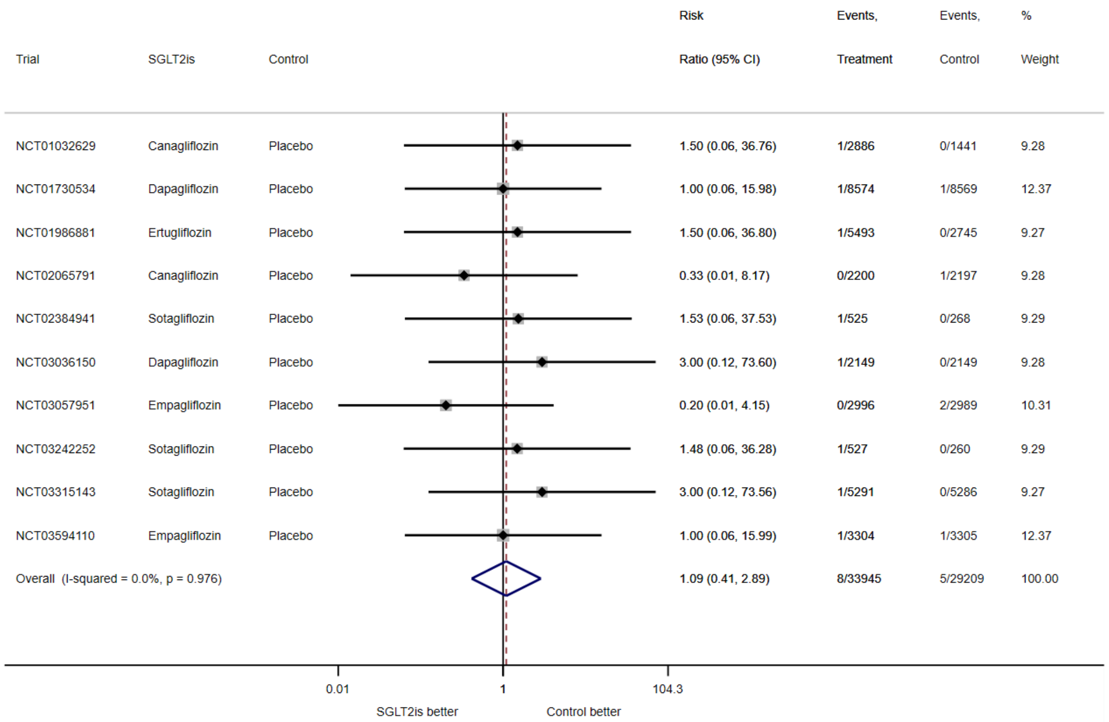

System 11-Infections And Infestations-Figure S155 Meta-analysis of SGLT2is and Viral upper respiratory tract infection

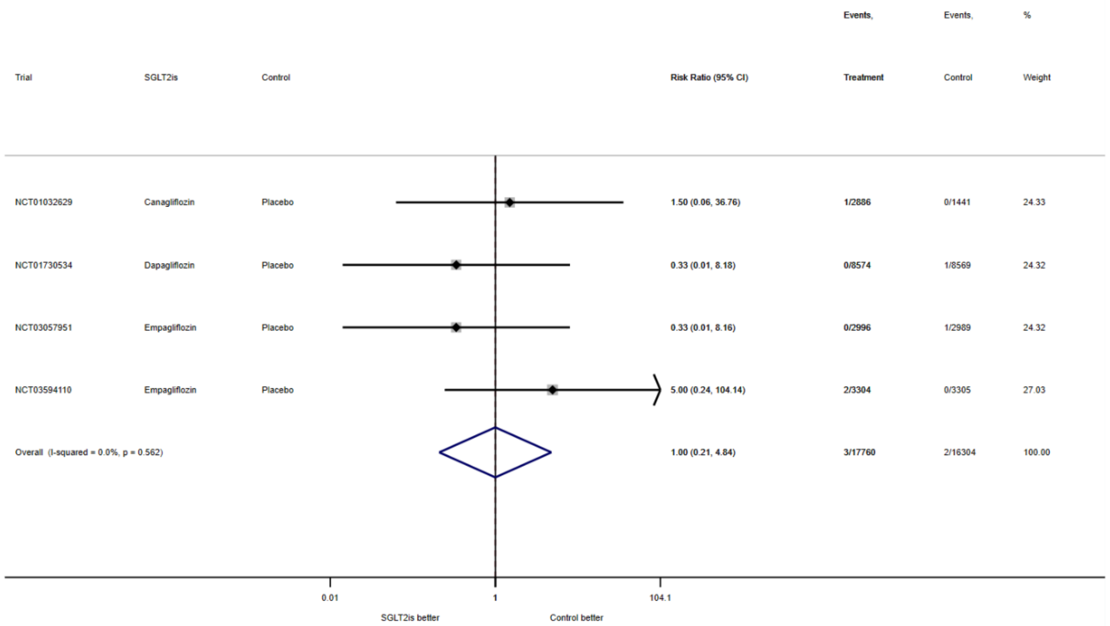

System 11-Infections And Infestations-Figure S156 Meta-analysis of SGLT2is and Wound abscess

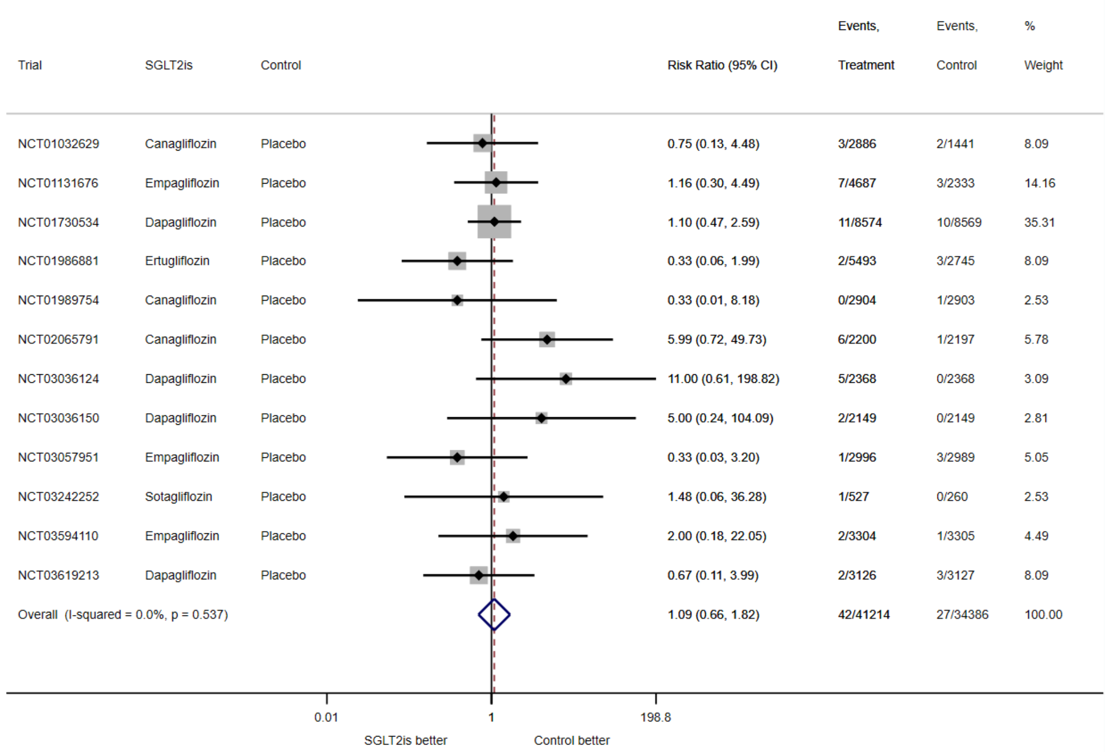

System 11-Infections And Infestations-Figure S157 Meta-analysis of SGLT2is and Wound infection

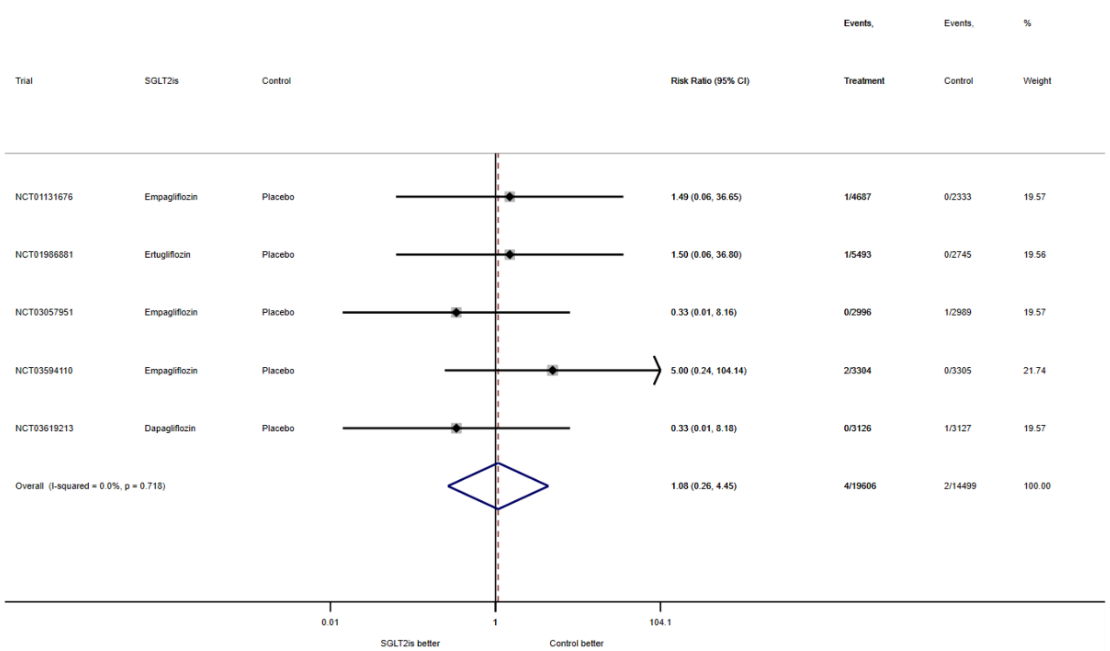

System 11-Infections And Infestations-Figure S158 Meta-analysis of SGLT2is and Wound sepsis

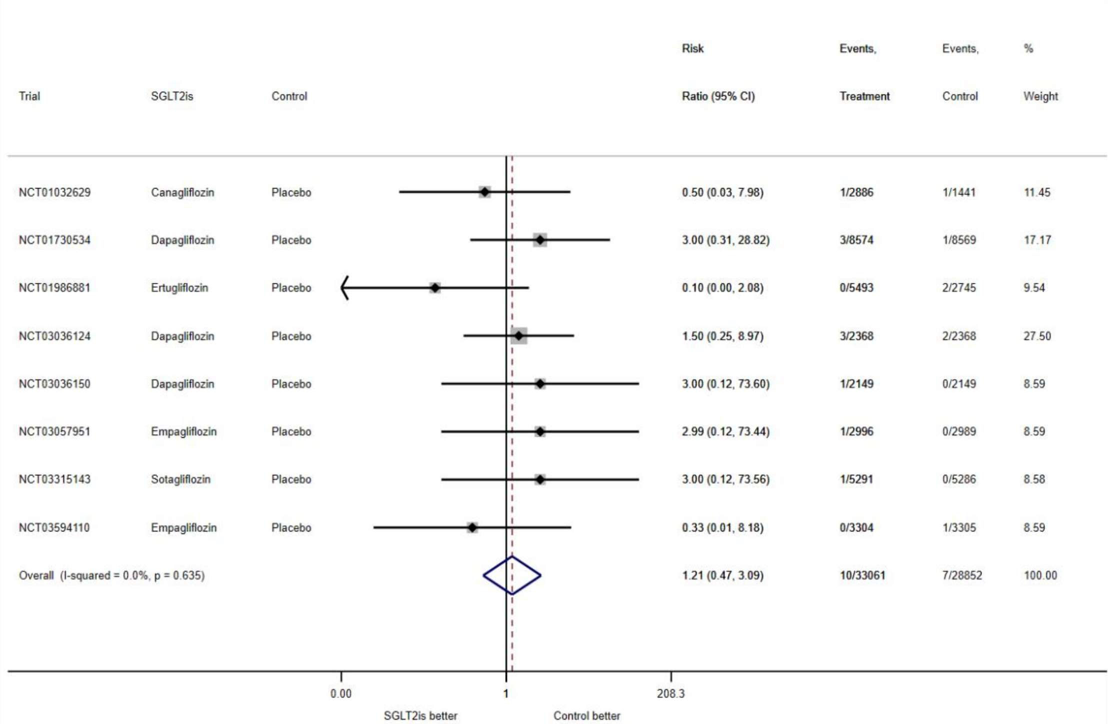

System 12-Injury, Poisoning And Procedural Complications-Figure S1 Meta-analysis of SGLT2is and Accidental overdose

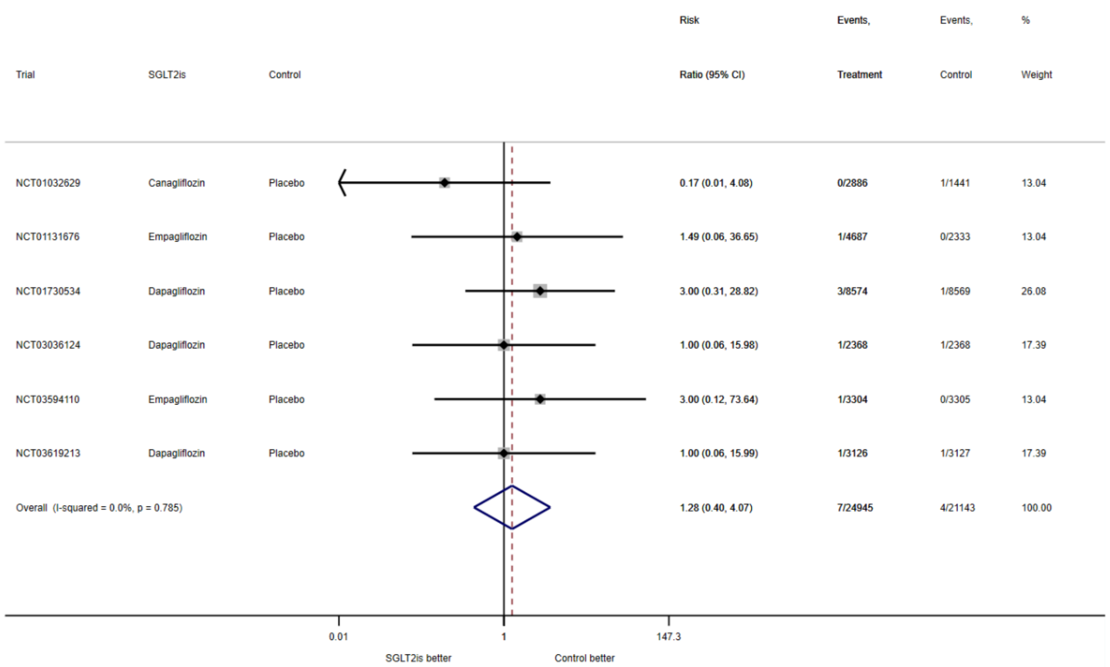

System 12-Injury, Poisoning And Procedural Complications-Figure S2 Meta-analysis of SGLT2is and Acetabulum fracture

Trial	SGLT2is	Control	Risk Ratio (95% CI)	Events, Treatment	Events, Control	% Weight
NCT01031680	Dapagliflozin	Placebo	0.33 (0.01, 8.20)	0/460	1/462	13.00
NCT01032629	Canagliflozin	Placebo	0.07 (0.00, 1.38)	0/2886	3/1441	15.15
NCT01730534	Dapagliflozin	Placebo	0.67 (0.11, 3.99)	2/8574	3/8569	41.55
NCT03036124	Dapagliflozin	Placebo	3.00 (0.12, 73.61)	1/2368	0/2368	12.98
NCT03057951	Empagliflozin	Placebo	1.00 (0.06, 15.94)	1/2996	1/2989	17.31
Overall (I-squared = 0.0%, p = 0.522)			0.57 (0.18, 1.79)	4/17284	8/15829	100.00

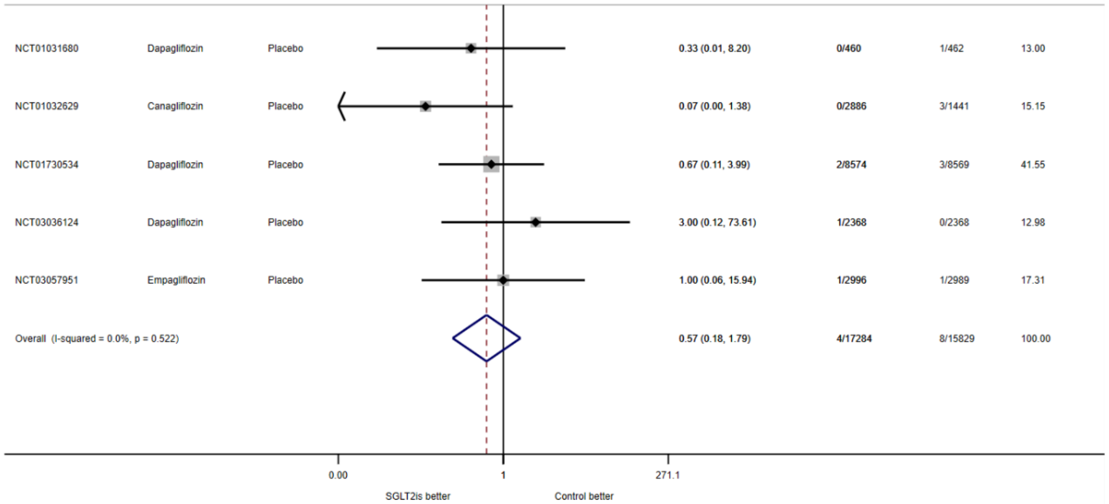

System 12-Injury, Poisoning And Procedural Complications-Figure S3 Meta-analysis of SGLT2is and Alcohol poisoning

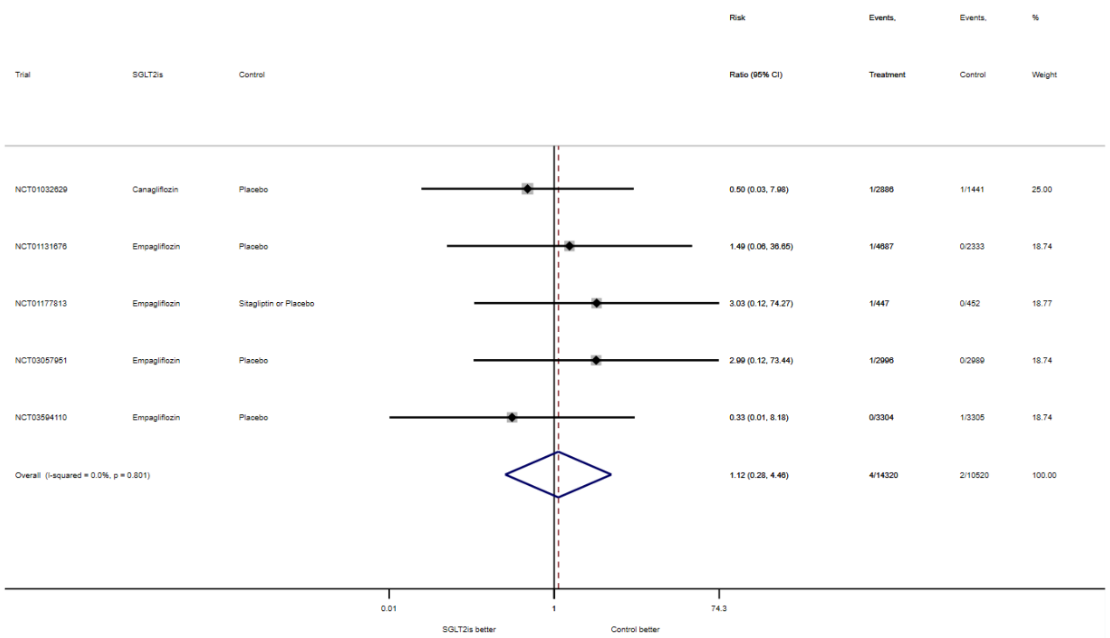

System 12-Injury, Poisoning And Procedural Complications-Figure S4 Meta-analysis of SGLT2is and Animal bite

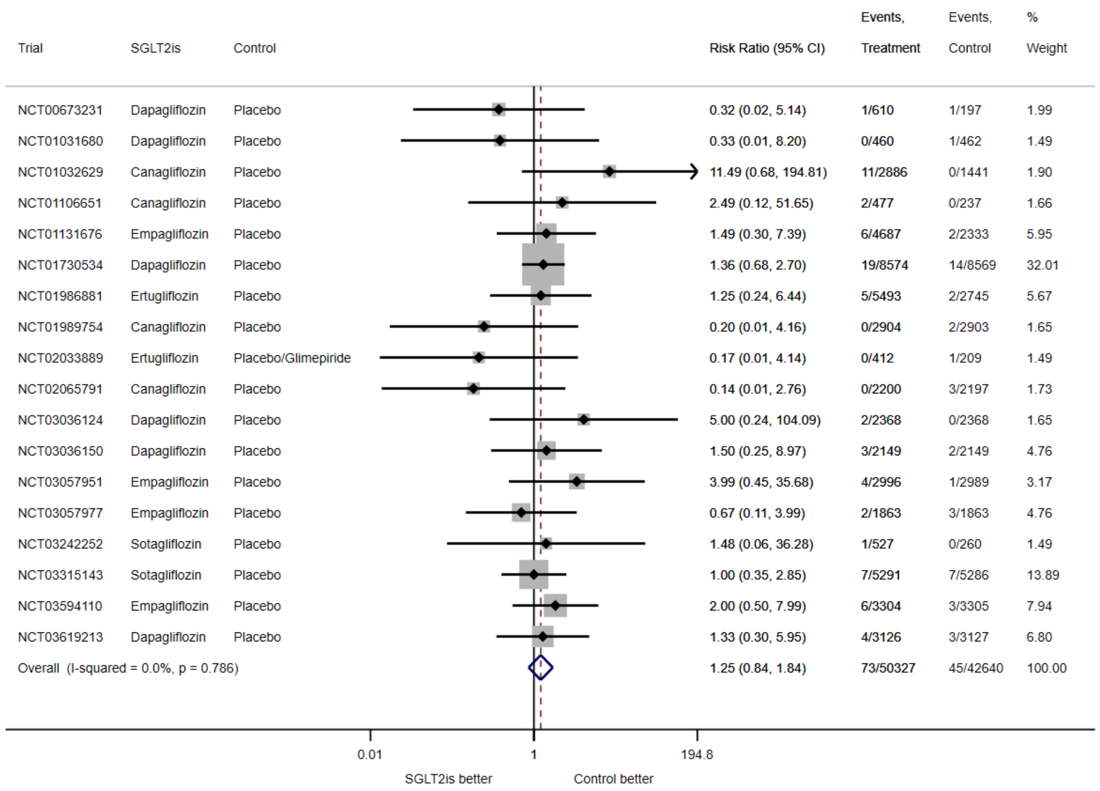

System 12-Injury, Poisoning And Procedural Complications-Figure S5 Meta-analysis of SGLT2is and Ankle fracture

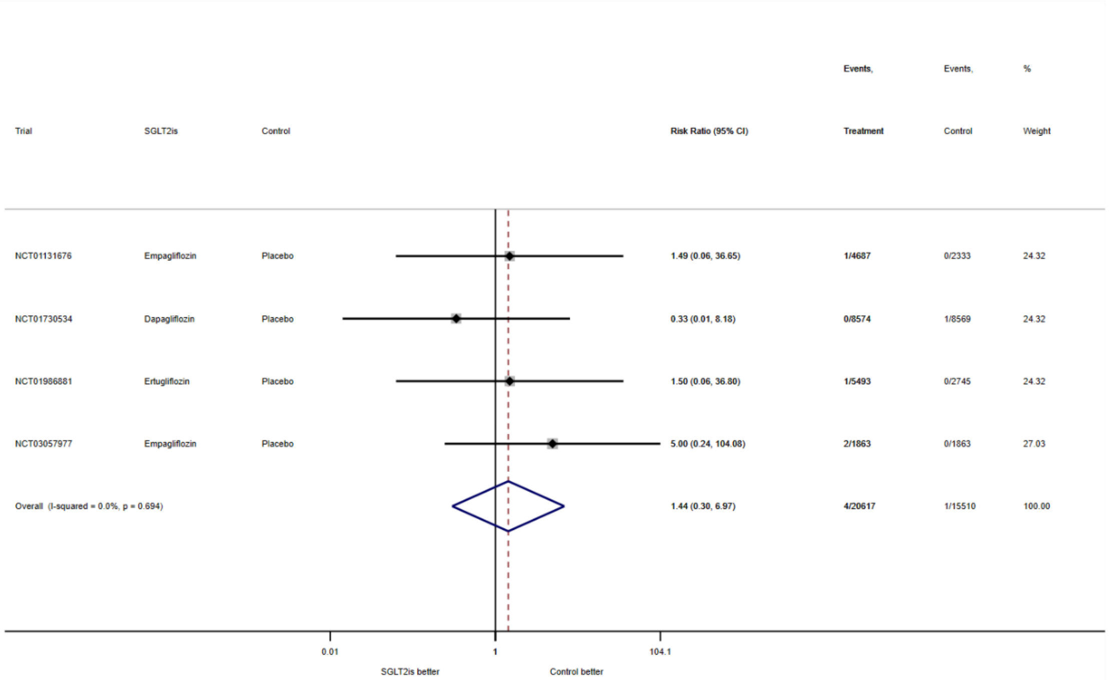

System 12-Injury, Poisoning And Procedural Complications-Figure S6 Meta-analysis of SGLT2is and Avulsion fracture

Trial	SGLT2is	Control	Risk Ratio (95% CI)	Events, Treatment	Events, Control	% Weight
NCT01131676	Empagliflozin	Placebo	0.17 (0.01, 4.07)	0/4687	1/2333	16.67
NCT01730534	Dapagliflozin	Placebo	0.33 (0.01, 8.18)	0/8574	1/8569	16.66
NCT01986881	Ertugliflozin	Placebo	1.50 (0.06, 36.80)	1/5493	0/2745	16.67
NCT03057951	Empagliflozin	Placebo	0.33 (0.01, 8.16)	0/2996	1/2989	16.67
NCT03594110	Empagliflozin	Placebo	3.00 (0.12, 73.64)	1/3304	0/3305	16.67
NCT03619213	Dapagliflozin	Placebo	3.00 (0.12, 73.64)	1/3126	0/3127	16.67
Overall (I-squared = 0.0%, p = 0.706)			0.79 (0.21, 2.93)	3/28180	3/23068	100.00

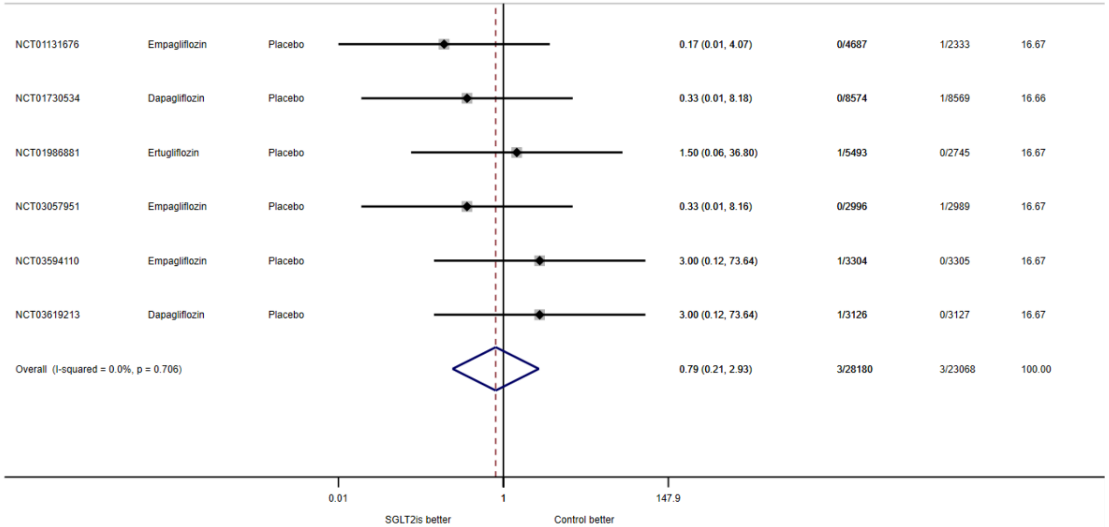

System 12-Injury, Poisoning And Procedural Complications-Figure S7 Meta-analysis of SGLT2is and Back injury

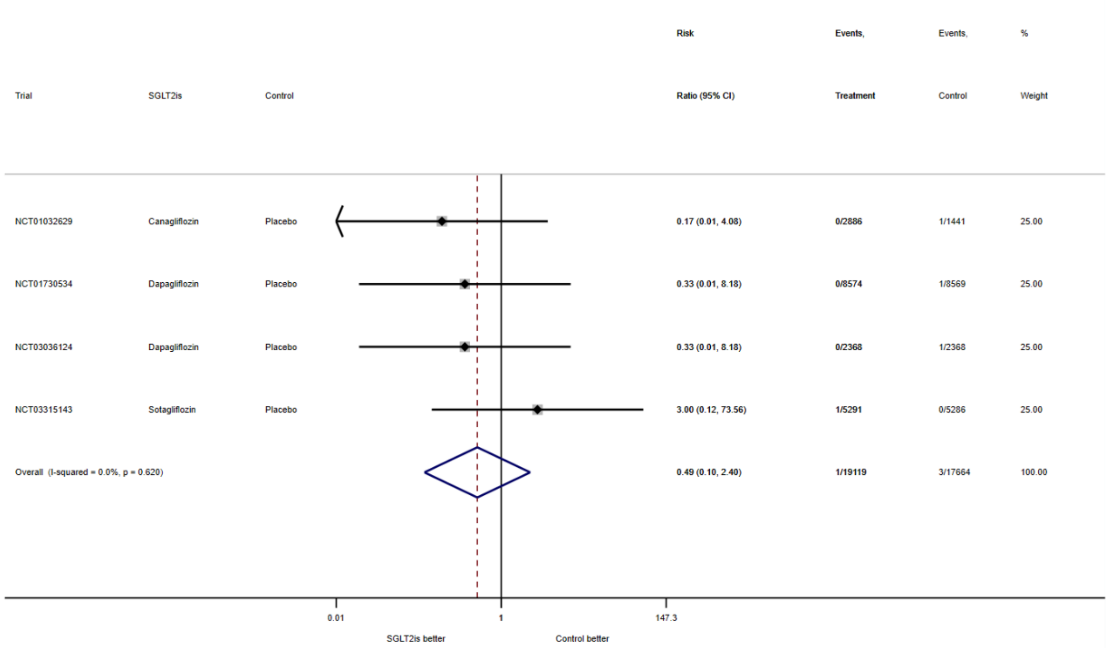

System 12-Injury, Poisoning And Procedural Complications-Figure S8 Meta-analysis of SGLT2is and Bone contusion

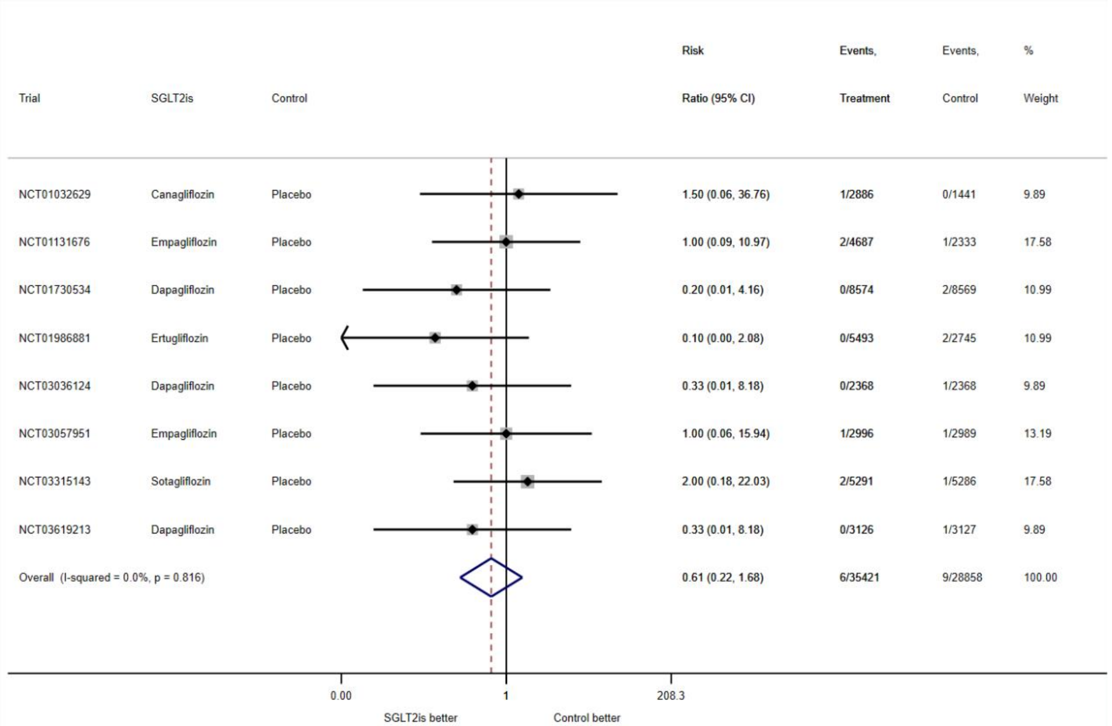

System 12-Injury, Poisoning And Procedural Complications-Figure S9 Meta-analysis of SGLT2is and Brain contusion

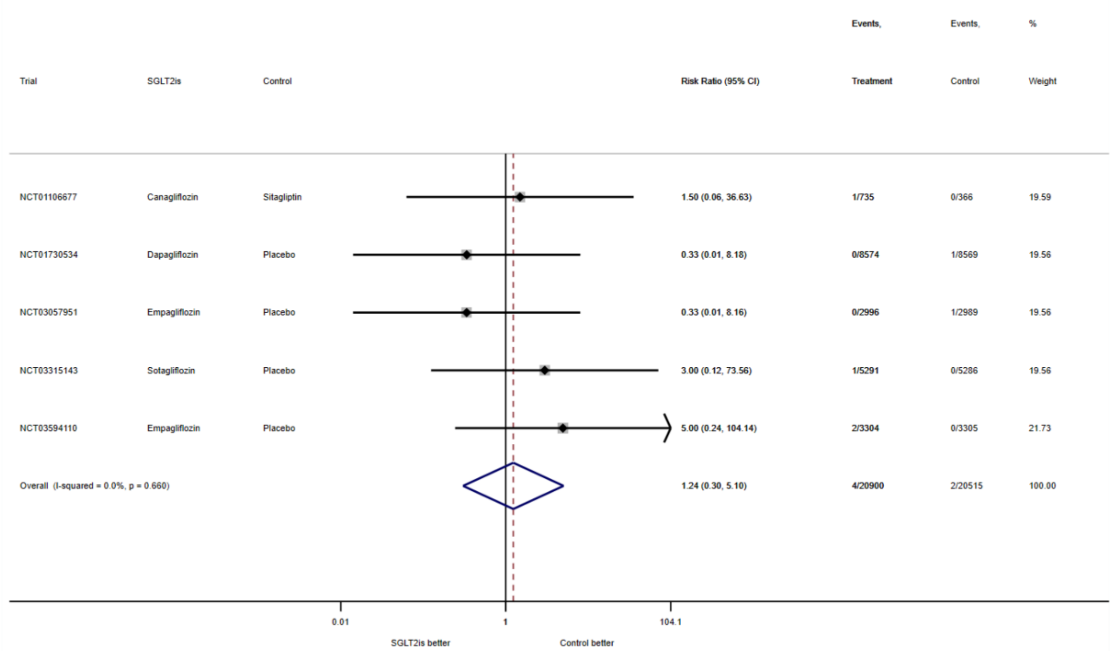

System 12-Injury, Poisoning And Procedural Complications-Figure S10 Meta-analysis of SGLT2is and Burns second degree

Trial	SGLT2is	Control	Risk Ratio (95% CI)	Events, Treatment	Events, Control	% Weight
NCT01032629	Canagliflozin	Placebo	0.50 (0.03, 7.98)	1/2886	1/1441	9.36
NCT01042977	Dapagliflozin	Placebo	0.33 (0.01, 8.16)	0/482	1/482	7.03
NCT01106651	Canagliflozin	Placebo	0.17 (0.01, 4.06)	0/477	1/237	7.03
NCT01106677	Canagliflozin	Sitagliptin	1.50 (0.06, 36.63)	1/735	0/366	7.02
NCT01730534	Dapagliflozin	Placebo	0.25 (0.03, 2.23)	1/8574	4/8569	14.97
NCT01986881	Ertugliflozin	Placebo	0.17 (0.01, 4.09)	0/5493	1/2745	7.02
NCT02065791	Canagliflozin	Placebo	0.33 (0.01, 8.17)	0/2200	1/2197	7.02
NCT03036150	Dapagliflozin	Placebo	2.00 (0.18, 22.04)	2/2149	1/2149	12.48
NCT03057951	Empagliflozin	Placebo	0.33 (0.03, 3.20)	1/2996	3/2989	14.04
NCT03619213	Dapagliflozin	Placebo	0.33 (0.01, 8.18)	0/3126	1/3127	7.02
NCT04157751	Empagliflozin	Placebo	3.05 (0.12, 74.43)	1/260	0/264	7.03
Overall (I-squared = 0.0%, p = 0.918)			0.49 (0.21, 1.14)	7/29378	14/24566	100.00

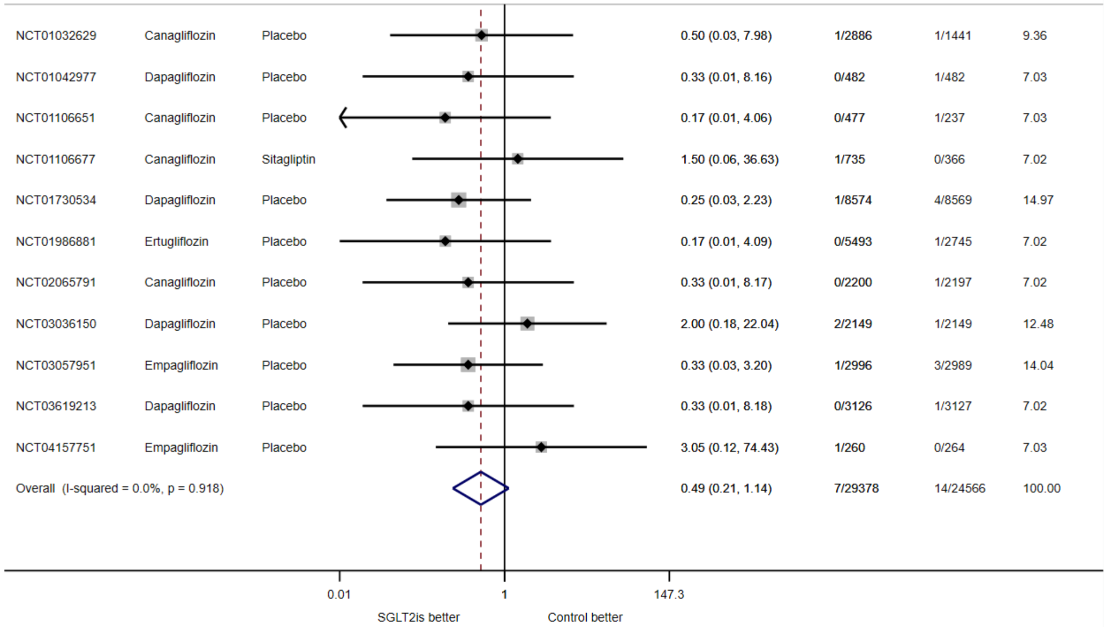

System 12-Injury, Poisoning And Procedural Complications-Figure S11 Meta-analysis of SGLT2is and Cervical vertebral fracture

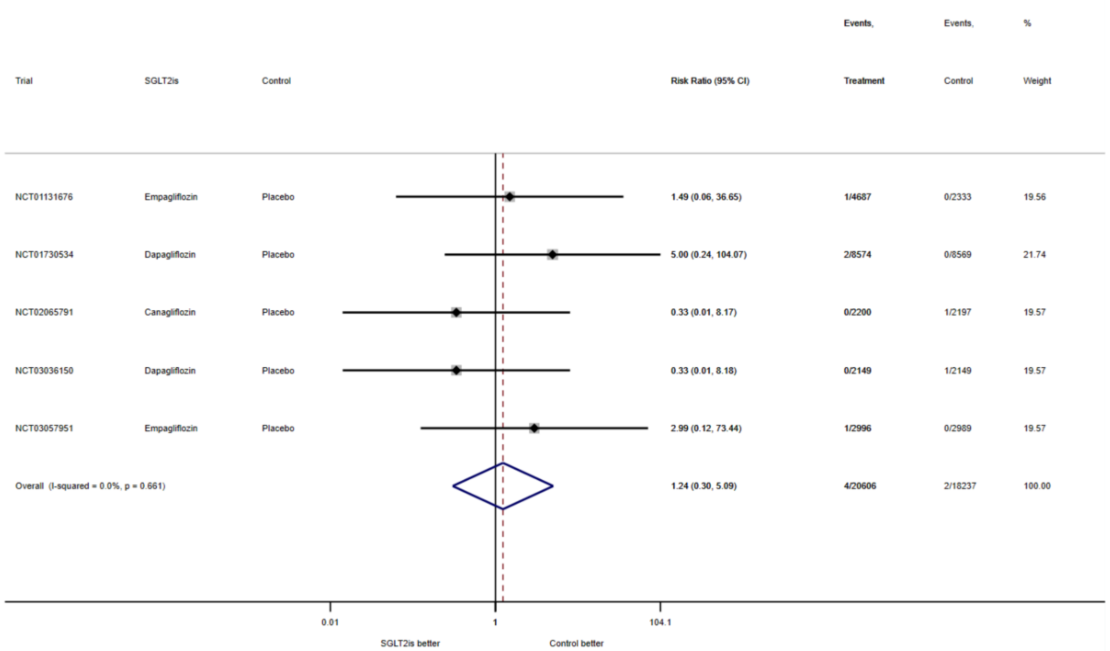

System 12-Injury, Poisoning And Procedural Complications-Figure S12 Meta-analysis of SGLT2is and Chest injury

Trial	SGLT2is	Control	Risk Ratio (95% CI)	Events, Treatment	Events, Control	% Weight
NCT01032629	Canagliflozin	Placebo	1.50 (0.06, 36.76)	1/2886	0/1441	8.74
NCT01042977	Dapagliflozin	Placebo	0.33 (0.01, 8.16)	0/482	1/482	8.75
NCT01131676	Empagliflozin	Placebo	0.50 (0.03, 7.95)	1/4687	1/2333	11.65
NCT01164501	Empagliflozin	Placebo	0.25 (0.01, 6.21)	0/419	1/319	8.75
NCT01730534	Dapagliflozin	Placebo	1.00 (0.06, 15.98)	1/8574	1/8569	11.65
NCT01989754	Canagliflozin	Placebo	3.00 (0.12, 73.59)	1/2904	0/2903	8.74
NCT02065791	Canagliflozin	Placebo	0.33 (0.01, 8.17)	0/2200	1/2197	8.74
NCT03057951	Empagliflozin	Placebo	0.33 (0.01, 8.16)	0/2996	1/2989	8.74
NCT03594110	Empagliflozin	Placebo	2.00 (0.18, 22.05)	2/3304	1/3305	15.53
NCT03619213	Dapagliflozin	Placebo	0.33 (0.01, 8.18)	0/3126	1/3127	8.74
Overall (I-squared = 0.0%, p = 0.961)			0.71 (0.27, 1.82)	6/31578	8/27665	100.00

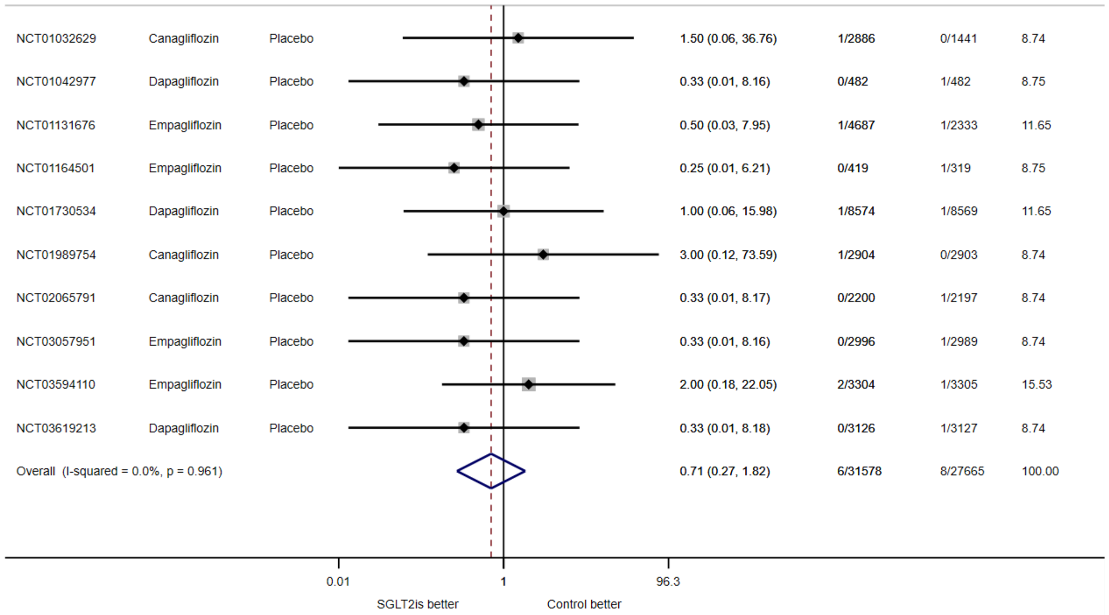

System 12-Injury, Poisoning And Procedural Complications-Figure S13 Meta-analysis of SGLT2is and Clavicle fracture

Trial	SGLT2is	Control	Risk Ratio (95% CI)	Events, Treatment	Events, Control	% Weight
NCT01032629	Canagliflozin	Placebo	0.75 (0.13, 4.48)	3/2886	2/1441	13.43
NCT01131676	Empagliflozin	Placebo	4.48 (0.24, 83.19)	4/4687	0/2333	5.03
NCT01730534	Dapagliflozin	Placebo	0.71 (0.23, 2.25)	5/8574	7/8569	32.62
NCT01986881	Ertugliflozin	Placebo	0.50 (0.07, 3.55)	2/5493	2/2745	11.18
NCT01989754	Canagliflozin	Placebo	3.00 (0.12, 73.59)	1/2904	0/2903	4.19
NCT02065791	Canagliflozin	Placebo	3.00 (0.12, 73.50)	1/2200	0/2197	4.19
NCT03036150	Dapagliflozin	Placebo	3.00 (0.12, 73.60)	1/2149	0/2149	4.19
NCT03057951	Empagliflozin	Placebo	2.00 (0.18, 21.99)	2/2996	1/2989	7.45
NCT03057977	Empagliflozin	Placebo	0.33 (0.01, 8.18)	0/1863	1/1863	4.19
NCT03521934	Sotagliflozin	Placebo	0.34 (0.01, 8.25)	0/605	1/611	4.20
NCT03594110	Empagliflozin	Placebo	5.00 (0.24, 104.14)	2/3304	0/3305	4.66
NCT03619213	Dapagliflozin	Placebo	5.00 (0.24, 104.14)	2/3126	0/3127	4.66
Overall (I-squared = 0.0%, p = 0.832)			1.10 (0.57, 2.12)	23/40787	14/34232	100.00

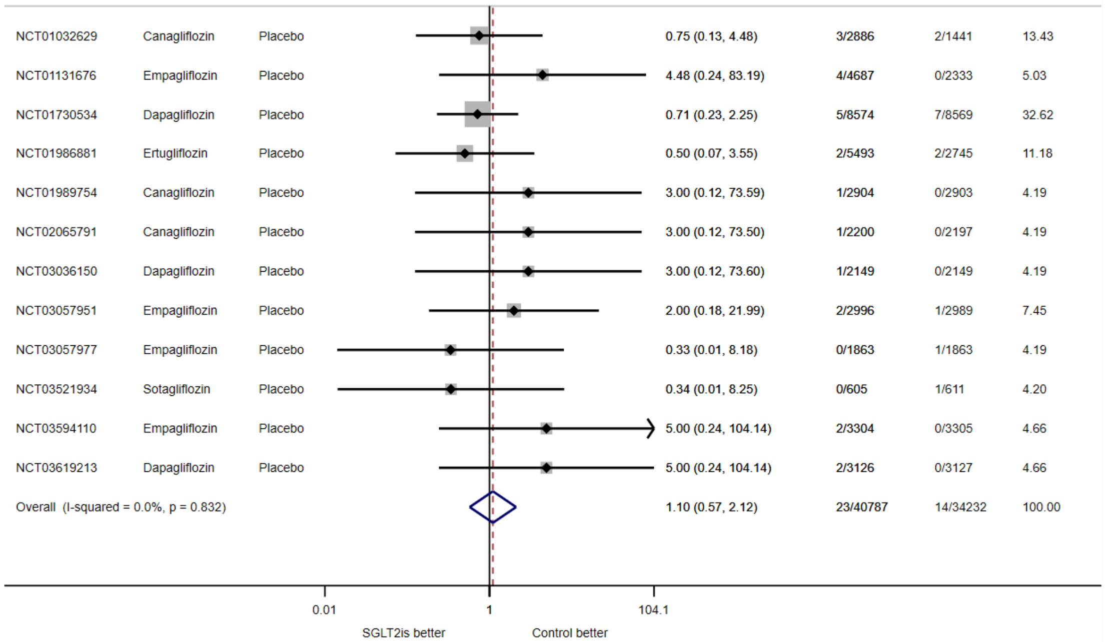

System 12-Injury, Poisoning And Procedural Complications-Figure S14 Meta-analysis of SGLT2is and Concussion

Trial	SGLT2is	Control	Risk Ratio (95% CI)	Events, Treatment	Events, Control	% Weight
NCT01032629	Canagliflozin	Placebo	0.75 (0.13, 4.48)	3/2886	2/1441	11.43
NCT01042977	Dapagliflozin	Placebo	0.33 (0.01, 8.16)	0/482	1/482	3.57
NCT01131676	Empagliflozin	Placebo	0.50 (0.12, 1.99)	4/4687	4/2333	19.06
NCT01730534	Dapagliflozin	Placebo	1.00 (0.25, 3.99)	4/8574	4/8569	19.04
NCT01986881	Ertugliflozin	Placebo	2.00 (0.22, 17.88)	4/5493	1/2745	7.62
NCT01989754	Canagliflozin	Placebo	0.33 (0.03, 3.20)	1/2904	3/2903	7.14
NCT02065791	Canagliflozin	Placebo	0.33 (0.01, 8.17)	0/2200	1/2197	3.57
NCT03057951	Empagliflozin	Placebo	2.99 (0.31, 28.76)	3/2996	1/2989	7.14
NCT03057977	Empagliflozin	Placebo	1.00 (0.06, 15.98)	1/1863	1/1863	4.76
NCT03315143	Sotagliflozin	Placebo	1.00 (0.14, 7.09)	2/5291	2/5286	9.52
NCT03619213	Dapagliflozin	Placebo	0.33 (0.03, 3.20)	1/3126	3/3127	7.14
Overall (I-squared = 0.0%, p = 0.932)			0.76 (0.42, 1.40)	23/40502	23/33935	100.00

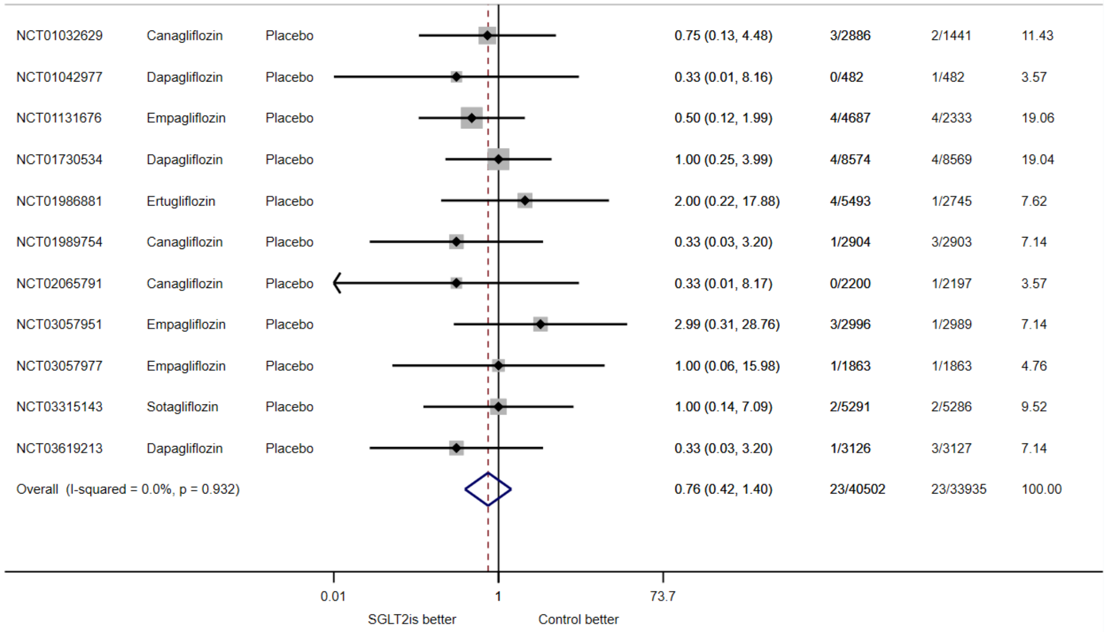

System 12-Injury, Poisoning And Procedural Complications-Figure S15 Meta-analysis of SGLT2is and Contusion

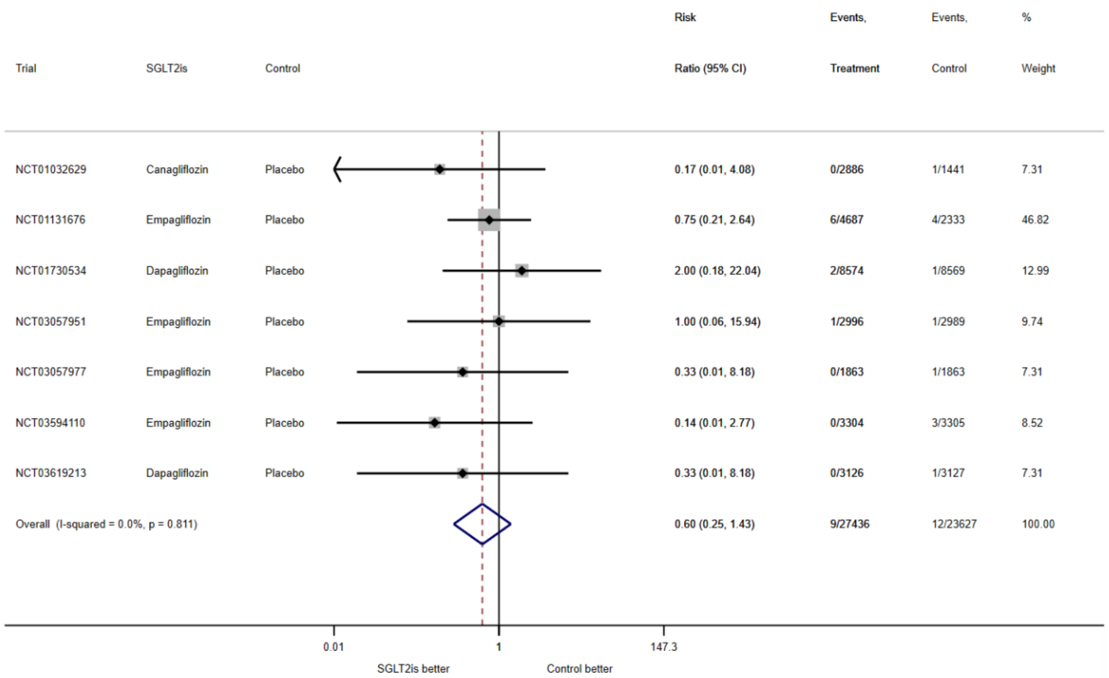

System 12-Injury, Poisoning And Procedural Complications-Figure S16 Meta-analysis of SGLT2is and Coronary artery restenosis

Trial	SGLT2is	Control	Risk Ratio (95% CI)	Events, Treatment	Events, Control	% Weight
NCT01032629	Canagliflozin	Placebo	4.50 (0.24, 83.44)	4/2886	0/1441	4.48
NCT01131676	Empagliflozin	Placebo	2.49 (0.12, 51.83)	2/4687	0/2333	4.15
NCT01730534	Dapagliflozin	Placebo	1.67 (0.40, 6.97)	5/8574	3/8569	18.67
NCT01986881	Ertugliflozin	Placebo	0.33 (0.06, 1.99)	2/5493	3/2745	11.95
NCT01989754	Canagliflozin	Placebo	5.00 (0.24, 104.07)	2/2904	0/2903	4.15
NCT02065791	Canagliflozin	Placebo	1.00 (0.06, 15.96)	1/2200	1/2197	4.98
NCT03036124	Dapagliflozin	Placebo	1.00 (0.06, 15.98)	1/2368	1/2368	4.98
NCT03036150	Dapagliflozin	Placebo	0.33 (0.01, 8.18)	0/2149	1/2149	3.73
NCT03057951	Empagliflozin	Placebo	3.99 (0.45, 35.68)	4/2996	1/2989	7.97
NCT03057977	Empagliflozin	Placebo	1.00 (0.06, 15.98)	1/1863	1/1863	4.98
NCT03242252	Sotagliflozin	Placebo	0.16 (0.01, 4.03)	0/527	1/260	3.74
NCT03315143	Sotagliflozin	Placebo	1.33 (0.30, 5.95)	4/5291	3/5286	17.08
NCT03521934	Sotagliflozin	Placebo	5.05 (0.24, 104.96)	2/605	0/611	4.15
NCT03619213	Dapagliflozin	Placebo	1.00 (0.06, 15.99)	1/3126	1/3127	4.98
Overall (I-squared = 0.0%, p = 0.832)			1.29 (0.69, 2.39)	29/45669	16/38841	100.00

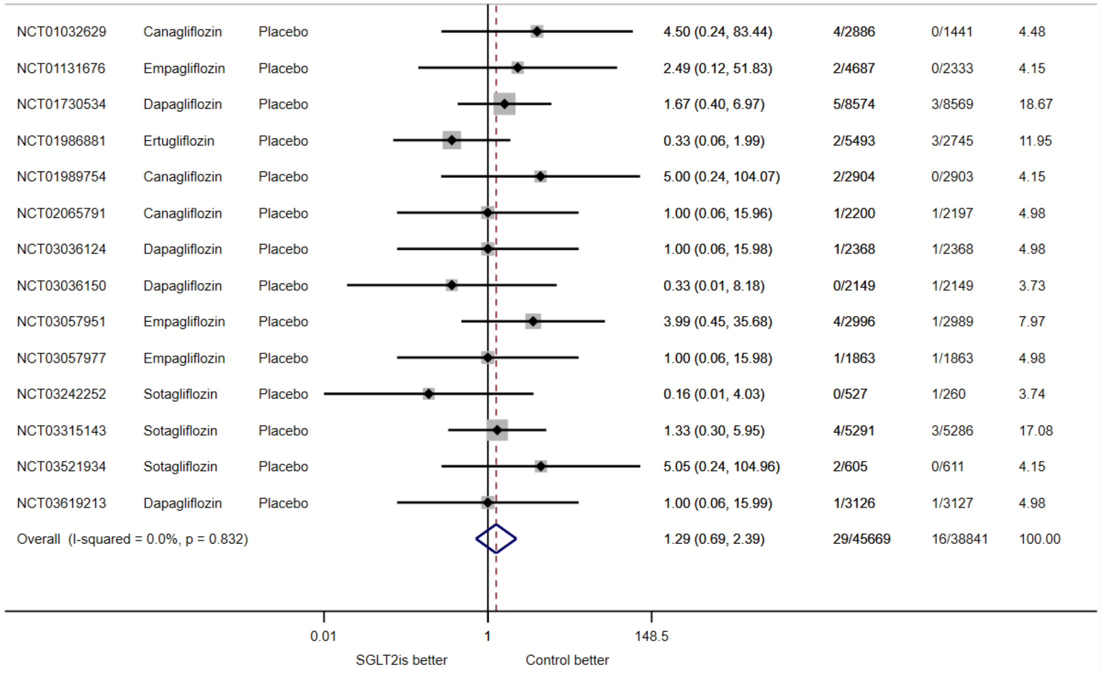

System 12-Injury, Poisoning And Procedural Complications-Figure S17 Meta-analysis of SGLT2is and Craniocerebral injury

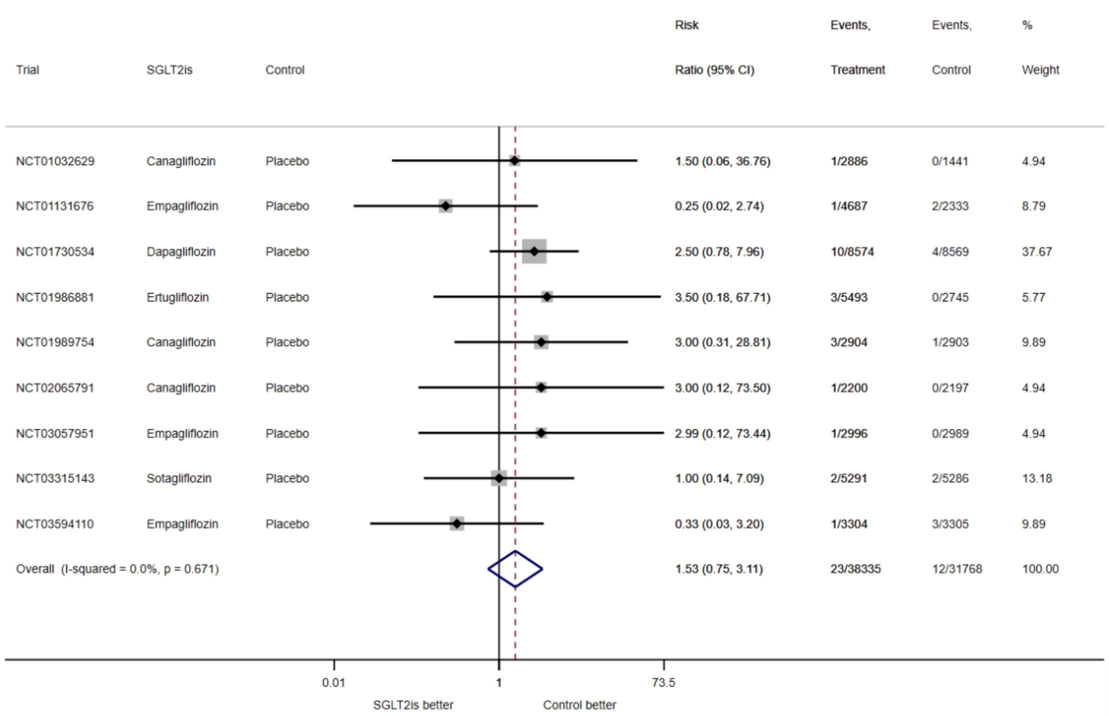

System 12-Injury, Poisoning And Procedural Complications-Figure S18 Meta-analysis of SGLT2is and Facial bones fracture

Trial	SGLT2is	Control	Risk Ratio (95% CI)	Events, Treatment	Events, Control	% Weight
NCT00528879	Dapagliflozin	Placebo	0.33 (0.02, 5.32)	1/409	1/137	0.78
NCT00673231	Dapagliflozin	Placebo	0.97 (0.04, 23.77)	1/610	0/197	0.58
NCT01032629	Canagliflozin	Placebo	2.50 (0.12, 51.99)	2/2886	0/1441	0.64
NCT01131676	Empagliflozin	Placebo	0.78 (0.42, 1.45)	25/4687	16/2333	15.19
NCT01164501	Empagliflozin	Placebo	0.15 (0.01, 3.16)	0/419	2/319	0.65
NCT01730534	Dapagliflozin	Placebo	0.70 (0.35, 1.38)	14/8574	20/8569	12.77
NCT01986881	Ertugliflozin	Placebo	1.50 (0.16, 14.41)	3/5493	1/2745	1.16
NCT01989754	Canagliflozin	Placebo	1.25 (0.34, 4.65)	5/2904	4/2903	3.44
NCT02065791	Canagliflozin	Placebo	0.33 (0.03, 3.20)	1/2200	3/2197	1.16
NCT03036124	Dapagliflozin	Placebo	2.00 (0.18, 22.04)	2/2368	1/2368	1.03
NCT03036150	Dapagliflozin	Placebo	1.00 (0.06, 15.98)	1/2149	1/2149	0.77
NCT03057951	Empagliflozin	Placebo	1.03 (0.63, 1.70)	31/2996	30/2989	23.83
NCT03057977	Empagliflozin	Placebo	3.75 (1.25, 11.28)	15/1863	4/1863	4.90
NCT03315143	Sotagliflozin	Placebo	1.69 (0.85, 3.35)	22/5291	13/5286	12.68
NCT03521934	Sotagliflozin	Placebo	4.04 (0.45, 36.04)	4/605	1/611	1.24
NCT03594110	Empagliflozin	Placebo	0.90 (0.47, 1.72)	17/3304	19/3305	13.96
NCT03619213	Dapagliflozin	Placebo	0.71 (0.23, 2.25)	5/3126	7/3127	4.52
NCT04157751	Empagliflozin	Placebo	7.11 (0.37, 136.92)	3/260	0/264	0.68
Overall (I-squared = 0.0%, p = 0.459)			1.05 (0.82, 1.34)	152/50144	123/42803	100.00

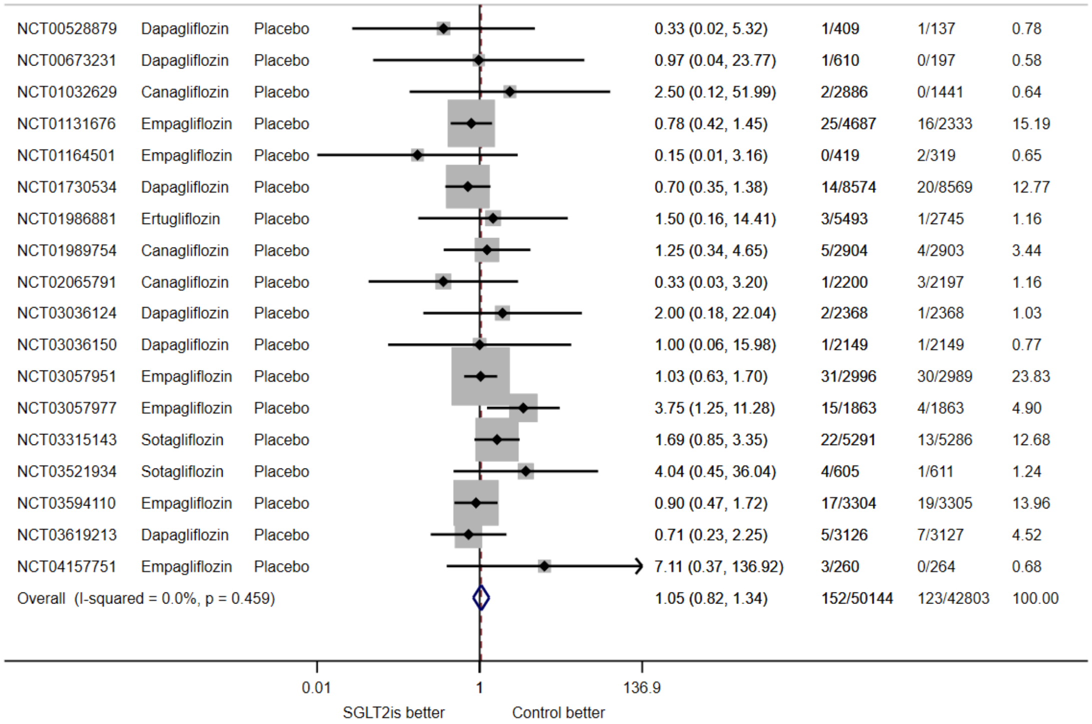

System 12-Injury, Poisoning And Procedural Complications-Figure S19 Meta-analysis of SGLT2is and Fall

Trial	SGLT2is	Control	Risk Ratio (95% CI)	Events, Treatment	Events, Control	% Weight
NCT01032629	Canagliflozin	Placebo	8.49 (0.49, 147.01)	8/2886	0/1441	2.46
NCT01042977	Dapagliflozin	Placebo	0.33 (0.01, 8.16)	0/482	1/482	1.96
NCT01131676	Empagliflozin	Placebo	1.49 (0.16, 14.35)	3/4687	1/2333	3.91
NCT01730534	Dapagliflozin	Placebo	0.56 (0.19, 1.66)	5/8574	9/8569	16.78
NCT01986881	Ertugliflozin	Placebo	1.50 (0.30, 7.42)	6/5493	2/2745	7.83
NCT01989754	Canagliflozin	Placebo	0.20 (0.01, 4.16)	0/2904	2/2903	2.17
NCT02065791	Canagliflozin	Placebo	2.00 (0.37, 10.89)	4/2200	2/2197	6.96
NCT02384941	Sotagliflozin	Placebo	1.53 (0.06, 37.53)	1/525	0/268	1.96
NCT03036124	Dapagliflozin	Placebo	1.50 (0.25, 8.97)	3/2368	2/2368	6.26
NCT03036150	Dapagliflozin	Placebo	0.67 (0.11, 3.99)	2/2149	3/2149	6.27
NCT03057951	Empagliflozin	Placebo	0.25 (0.03, 2.23)	1/2996	4/2989	4.17
NCT03057977	Empagliflozin	Placebo	0.50 (0.05, 5.51)	1/1863	2/1863	3.48
NCT03315143	Sotagliflozin	Placebo	1.67 (0.40, 6.96)	5/5291	3/5286	9.79
NCT03521934	Sotagliflozin	Placebo	3.03 (0.12, 74.22)	1/605	0/611	1.96
NCT03594110	Empagliflozin	Placebo	0.60 (0.14, 2.51)	3/3304	5/3305	9.79
NCT03619213	Dapagliflozin	Placebo	1.20 (0.37, 3.93)	6/3126	5/3127	14.25
Overall (I-squared = 0.0%, p = 0.841)			0.97 (0.62, 1.51)	49/49453	41/42636	100.00

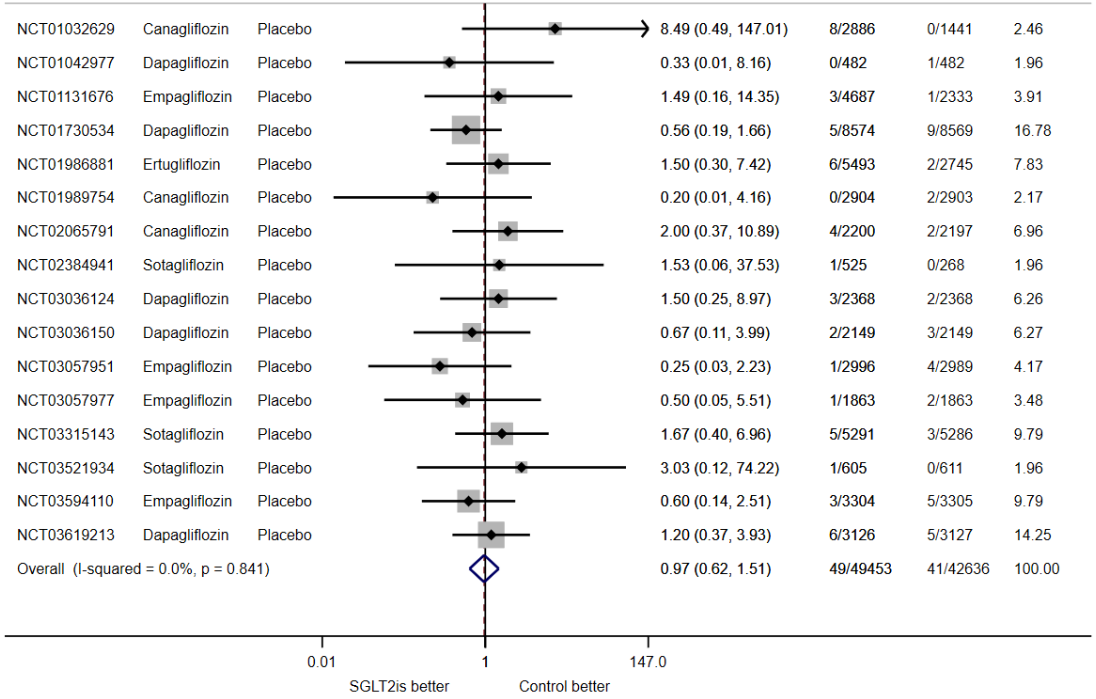

System 12-Injury, Poisoning And Procedural Complications-Figure S20 Meta-analysis of SGLT2is and Femoral neck fracture

Trial	SGLT2is	Control	Risk Ratio (95% CI)	Events, Treatment	Events, Control	% Weight
NCT01032629	Canagliflozin	Placebo	3.99 (0.50, 31.91)	8/2886	1/1441	2.16
NCT01131676	Empagliflozin	Placebo	0.33 (0.06, 1.98)	2/4687	3/2333	2.92
NCT01730534	Dapagliflozin	Placebo	0.89 (0.45, 1.74)	16/8574	18/8569	20.61
NCT01986881	Ertugliflozin	Placebo	0.80 (0.26, 2.44)	8/5493	5/2745	7.48
NCT01989754	Canagliflozin	Placebo	0.67 (0.19, 2.36)	4/2904	6/2903	5.84
NCT02065791	Canagliflozin	Placebo	4.99 (0.58, 42.70)	5/2200	1/2197	2.02
NCT03036124	Dapagliflozin	Placebo	1.33 (0.30, 5.95)	4/2368	3/2368	4.17
NCT03036150	Dapagliflozin	Placebo	1.75 (0.51, 5.97)	7/2149	4/2149	6.20
NCT03057951	Empagliflozin	Placebo	1.16 (0.54, 2.51)	14/2996	12/2989	15.76
NCT03057977	Empagliflozin	Placebo	1.67 (0.40, 6.96)	5/1863	3/1863	4.56
NCT03315143	Sotagliflozin	Placebo	1.00 (0.35, 2.85)	7/5291	7/5286	8.51
NCT03521934	Sotagliflozin	Placebo	1.01 (0.06, 16.11)	1/605	1/611	1.22
NCT03594110	Empagliflozin	Placebo	0.22 (0.05, 1.03)	2/3304	9/3305	3.98
NCT03619213	Dapagliflozin	Placebo	0.91 (0.39, 2.14)	10/3126	11/3127	12.76
NCT04157751	Empagliflozin	Placebo	0.34 (0.01, 8.27)	0/260	1/264	0.91
NCT04350593	Dapagliflozin	Placebo	0.33 (0.01, 8.21)	0/613	1/616	0.91
Overall (I-squared = 0.0%, p = 0.666)			0.97 (0.71, 1.31)	93/49319	86/42766	100.00

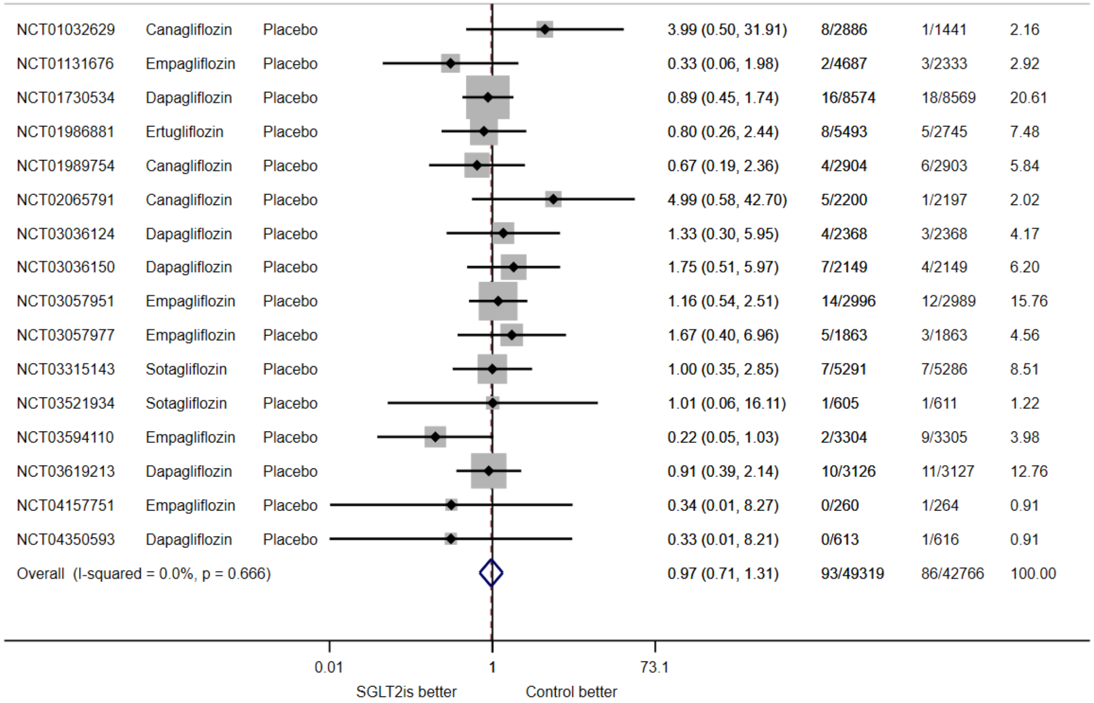

System 12-Injury, Poisoning And Procedural Complications-Figure S21 Meta-analysis of SGLT2is and Femur fracture

Trial	SGLT2is	Control	Risk Ratio (95% CI)	Events, Treatment	Events, Control	% Weight
NCT01032629	Canagliflozin	Placebo	2.50 (0.12, 51.99)	2/2886	0/1441	4.82
NCT01131676	Empagliflozin	Placebo	0.25 (0.02, 2.74)	1/4687	2/2333	7.72
NCT01730534	Dapagliflozin	Placebo	1.25 (0.34, 4.65)	5/8574	4/8569	25.72
NCT01986881	Ertugliflozin	Placebo	2.50 (0.12, 52.04)	2/5493	0/2745	4.82
NCT01989754	Canagliflozin	Placebo	2.00 (0.18, 22.04)	2/2904	1/2903	7.72
NCT02065791	Canagliflozin	Placebo	4.99 (0.24, 103.94)	2/2200	0/2197	4.82
NCT03036124	Dapagliflozin	Placebo	0.33 (0.01, 8.18)	0/2368	1/2368	4.34
NCT03057951	Empagliflozin	Placebo	2.99 (0.12, 73.44)	1/2996	0/2989	4.34
NCT03057977	Empagliflozin	Placebo	0.33 (0.01, 8.18)	0/1863	1/1863	4.34
NCT03315143	Sotagliflozin	Placebo	2.00 (0.37, 10.90)	4/5291	2/5286	15.43
NCT03594110	Empagliflozin	Placebo	1.00 (0.06, 15.99)	1/3304	1/3305	5.79
NCT03619213	Dapagliflozin	Placebo	1.00 (0.06, 15.99)	1/3126	1/3127	5.79
NCT04157751	Empagliflozin	Placebo	0.34 (0.01, 8.27)	0/260	1/264	4.35
Overall (I-squared = 0.0%, p = 0.933)			1.20 (0.62, 2.33)	21/45952	14/39390	100.00

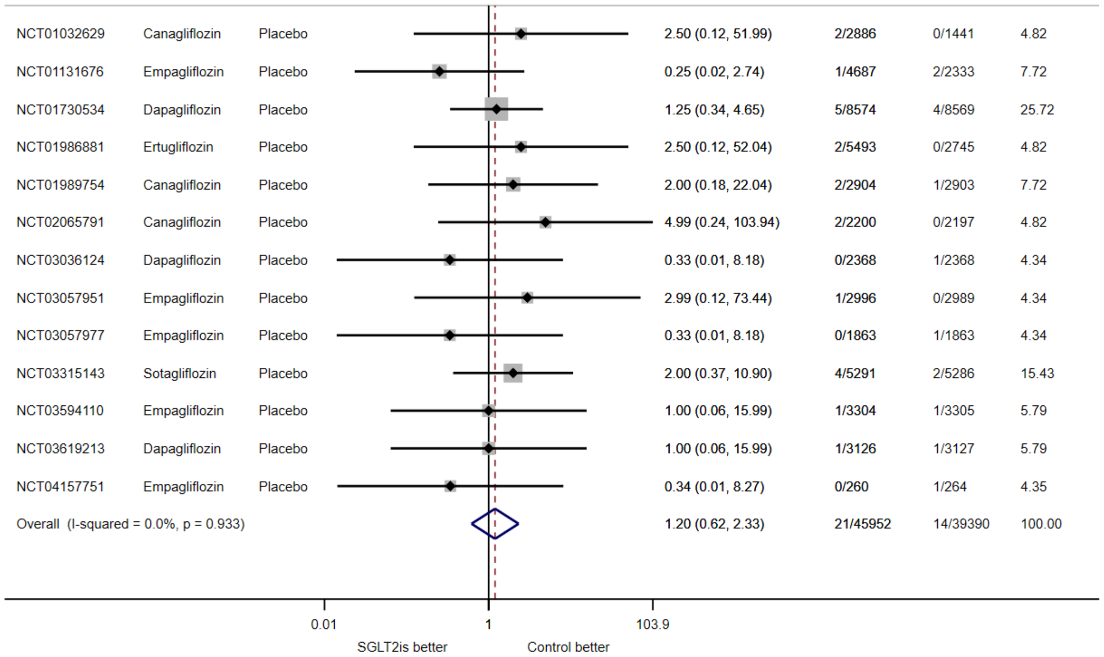

System 12-Injury, Poisoning And Procedural Complications-Figure S22 Meta-analysis of SGLT2is and Fibula fracture

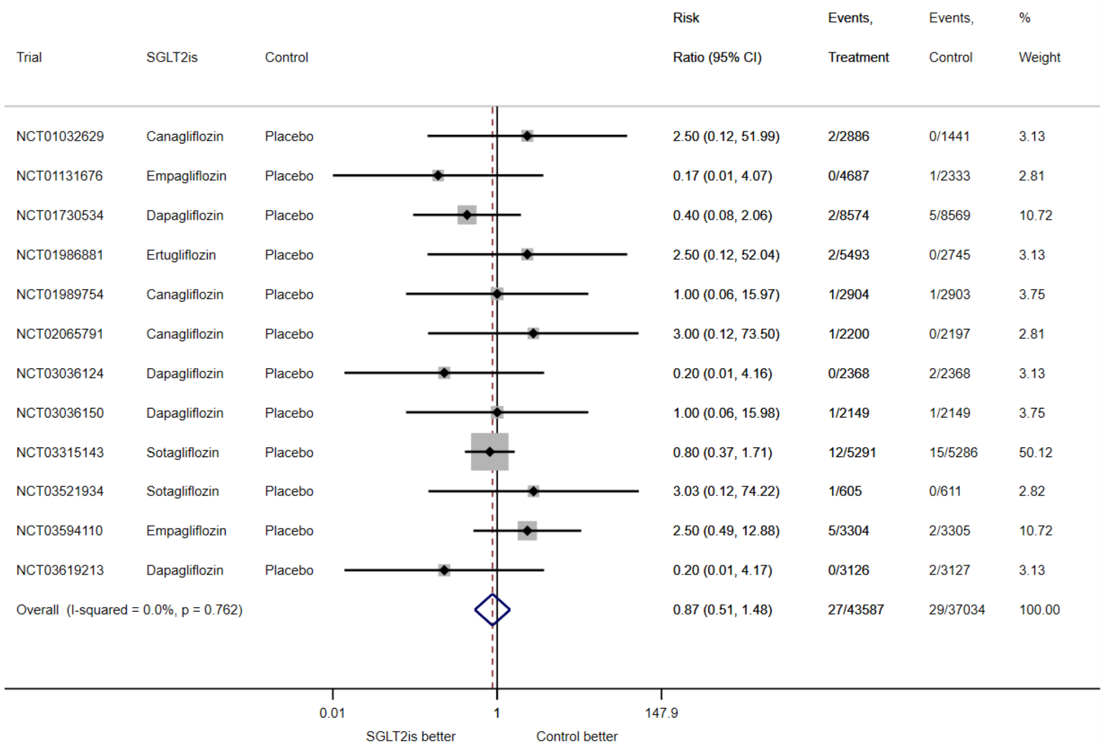

System 12-Injury, Poisoning And Procedural Complications-Figure S23 Meta-analysis of SGLT2is and Foot fracture

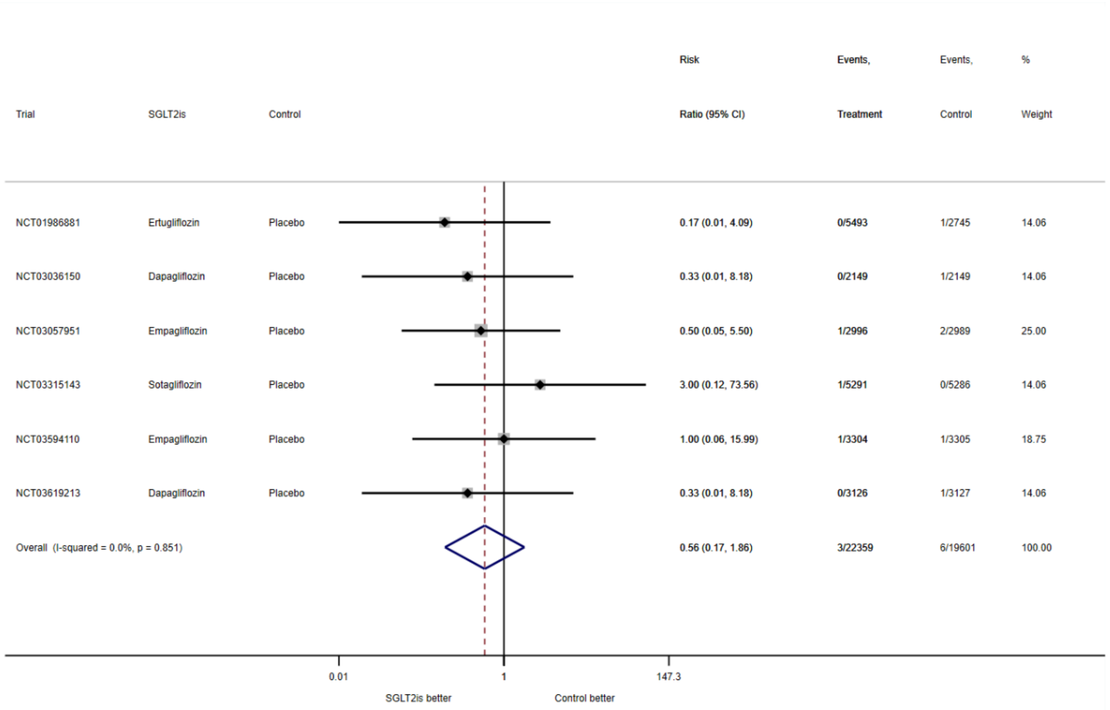

System 12-Injury, Poisoning And Procedural Complications-Figure S24 Meta-analysis of SGLT2is and Forearm fracture

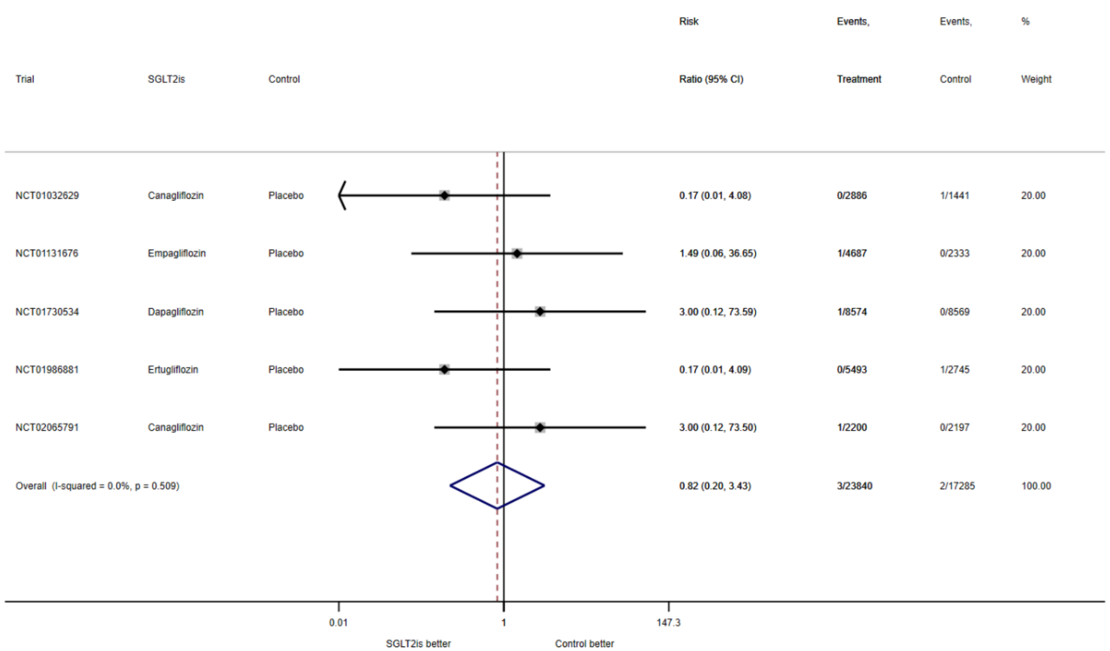

System 12-Injury, Poisoning And Procedural Complications-Figure S25 Meta-analysis of SGLT2is and Fracture displacement

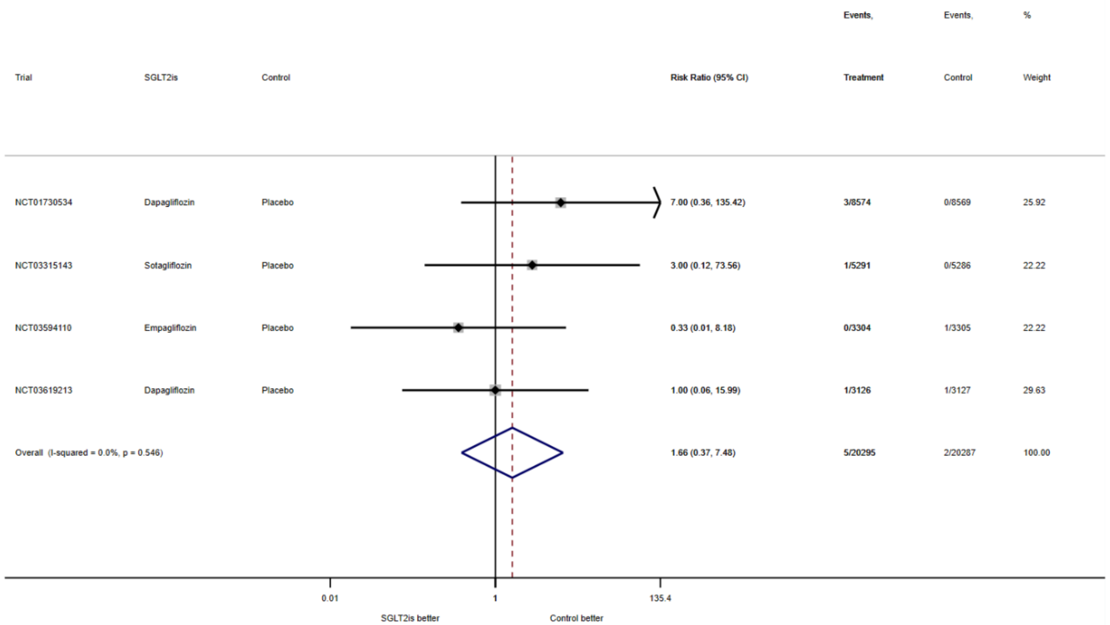

System 12-Injury, Poisoning And Procedural Complications-Figure S26 Meta-analysis of SGLT2is and Fractured sacrum

Trial	SGLT2is	Control	Risk Ratio (95% CI)	Events, Treatment	Events, Control	% Weight
NCT01106651	Canagliflozin	Placebo	0.17 (0.01, 4.06)	0/477	1/237	4.39
NCT01131676	Empagliflozin	Placebo	0.50 (0.03, 7.95)	1/4687	1/2333	5.84
NCT01730534	Dapagliflozin	Placebo	0.75 (0.17, 3.35)	3/8574	4/8569	20.04
NCT01986881	Ertugliflozin	Placebo	1.50 (0.06, 36.80)	1/5493	0/2745	4.38
NCT01989754	Canagliflozin	Placebo	2.00 (0.18, 22.04)	2/2904	1/2903	7.79
NCT03036150	Dapagliflozin	Placebo	0.50 (0.05, 5.51)	1/2149	2/2149	7.80
NCT03057951	Empagliflozin	Placebo	1.00 (0.06, 15.94)	1/2996	1/2989	5.84
NCT03315143	Sotagliflozin	Placebo	1.75 (0.51, 5.97)	7/5291	4/5286	29.77
NCT03521934	Sotagliflozin	Placebo	0.34 (0.01, 8.25)	0/605	1/611	4.39
NCT03594110	Empagliflozin	Placebo	5.00 (0.58, 42.79)	5/3304	1/3305	9.74
Overall (I-squared = 0.0%, p = 0.780)			1.12 (0.57, 2.19)	21/36480	16/31127	100.00

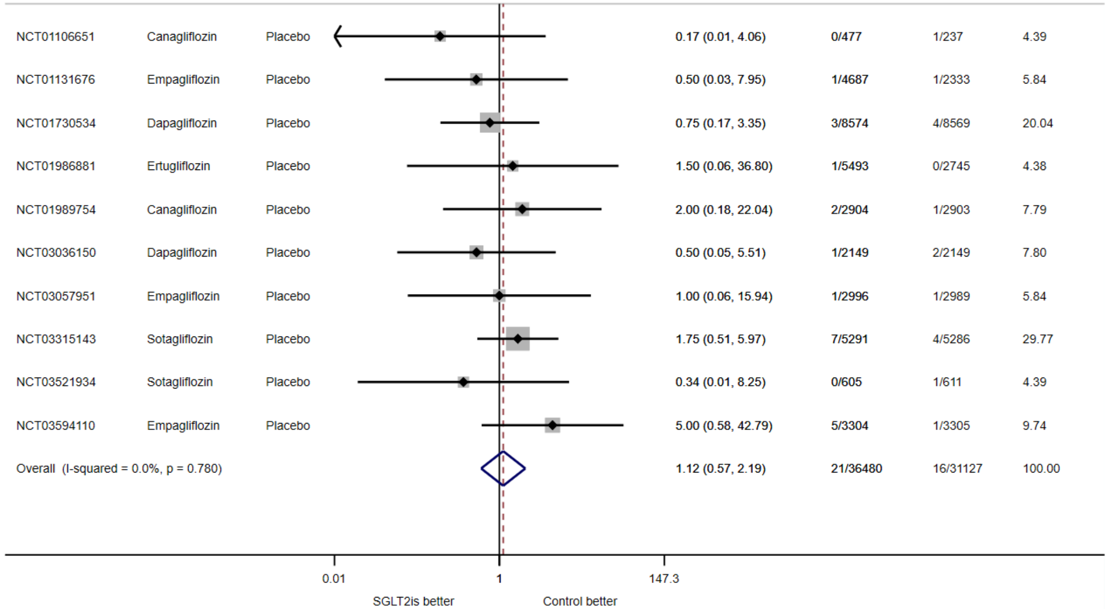

System 12-Injury, Poisoning And Procedural Complications-Figure S27 Meta-analysis of SGLT2is and Hand fracture

Trial	SGLT2is	Control	Risk Ratio (95% CI)	Events, Treatment	Events, Control	% Weight
NCT00528879	Dapagliflozin	Placebo	1.01 (0.04, 24.64)	1/409	0/137	2.83
NCT01032629	Canagliflozin	Placebo	1.00 (0.09, 11.00)	2/2886	1/1441	5.02
NCT01131676	Empagliflozin	Placebo	0.50 (0.07, 3.53)	2/4687	2/2333	7.53
NCT01730534	Dapagliflozin	Placebo	0.50 (0.15, 1.66)	4/8574	8/8569	20.08
NCT01986881	Ertugliflozin	Placebo	0.67 (0.15, 2.97)	4/5493	3/2745	12.91
NCT01989754	Canagliflozin	Placebo	0.20 (0.01, 4.16)	0/2904	2/2903	3.14
NCT02065791	Canagliflozin	Placebo	1.00 (0.06, 15.96)	1/2200	1/2197	3.76
NCT03036124	Dapagliflozin	Placebo	1.00 (0.06, 15.98)	1/2368	1/2368	3.76
NCT03036150	Dapagliflozin	Placebo	0.67 (0.11, 3.99)	2/2149	3/2149	9.04
NCT03057951	Empagliflozin	Placebo	0.75 (0.17, 3.34)	3/2996	4/2989	12.91
NCT03057977	Empagliflozin	Placebo	7.00 (0.36, 135.42)	3/1863	0/1863	3.29
NCT03315143	Sotagliflozin	Placebo	6.99 (0.86, 56.82)	7/5291	1/5286	6.59
NCT03594110	Empagliflozin	Placebo	3.00 (0.12, 73.64)	1/3304	0/3305	2.82
NCT03619213	Dapagliflozin	Placebo	0.08 (0.00, 1.37)	0/3126	6/3127	3.49
NCT04157751	Empagliflozin	Placebo	3.05 (0.12, 74.43)	1/260	0/264	2.83
Overall (I-squared = 0.0%, p = 0.617)			0.82 (0.48, 1.40)	32/48510	32/41676	100.00

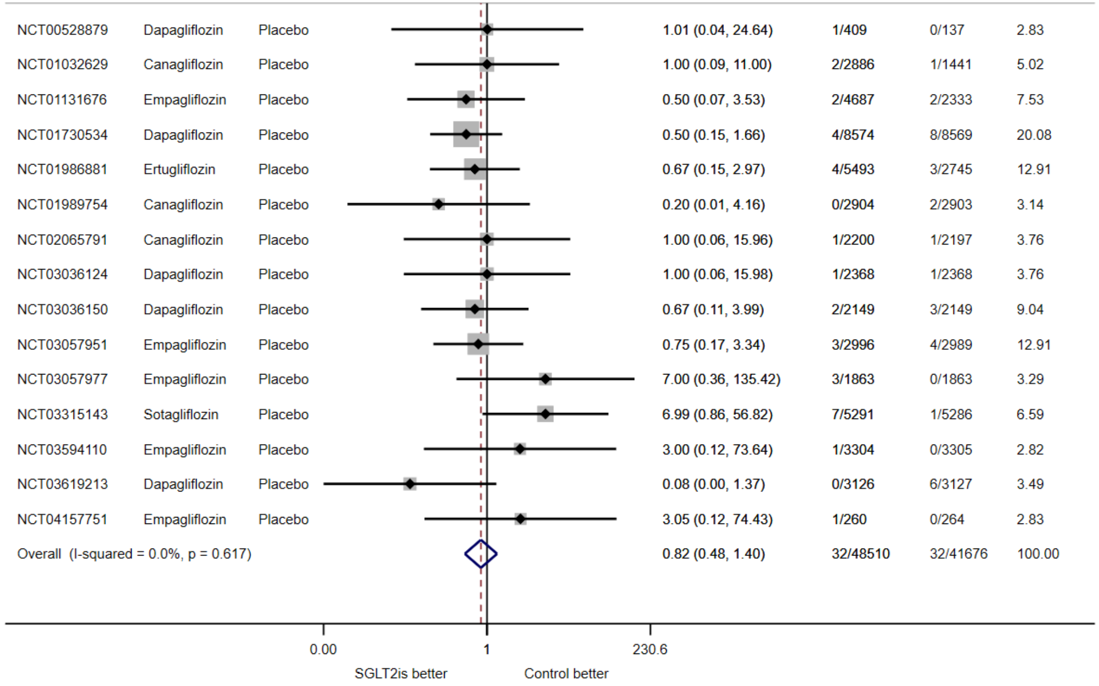

System 12-Injury, Poisoning And Procedural Complications-Figure S28 Meta-analysis of SGLT2is and Head injury

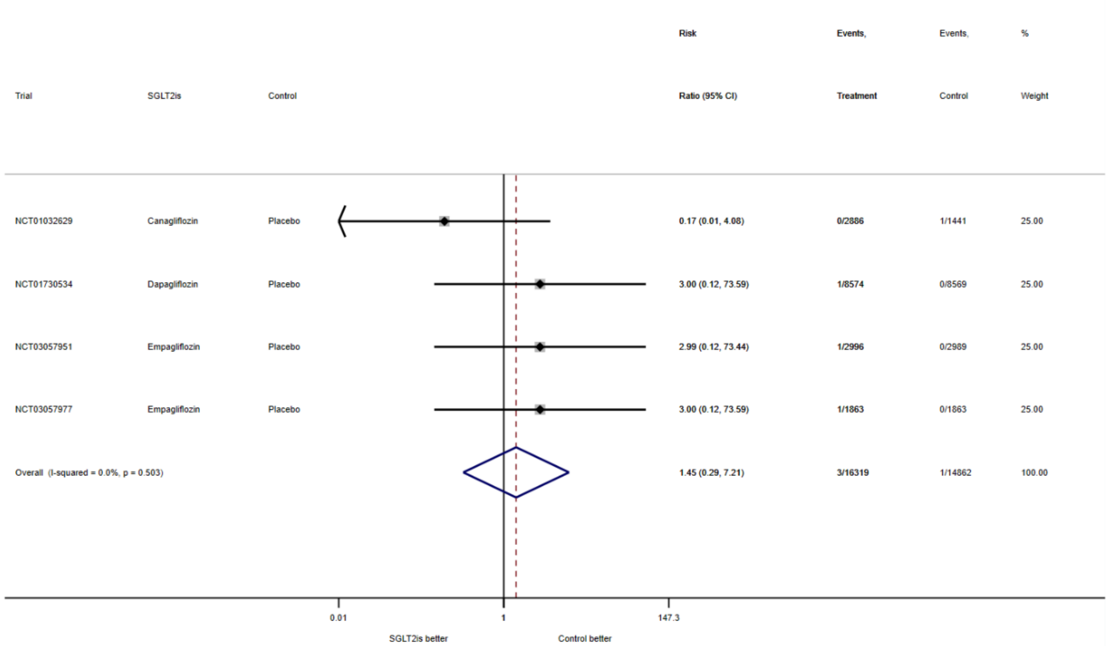

System 12-Injury, Poisoning And Procedural Complications-Figure S29 Meta-analysis of SGLT2is and Heat stroke

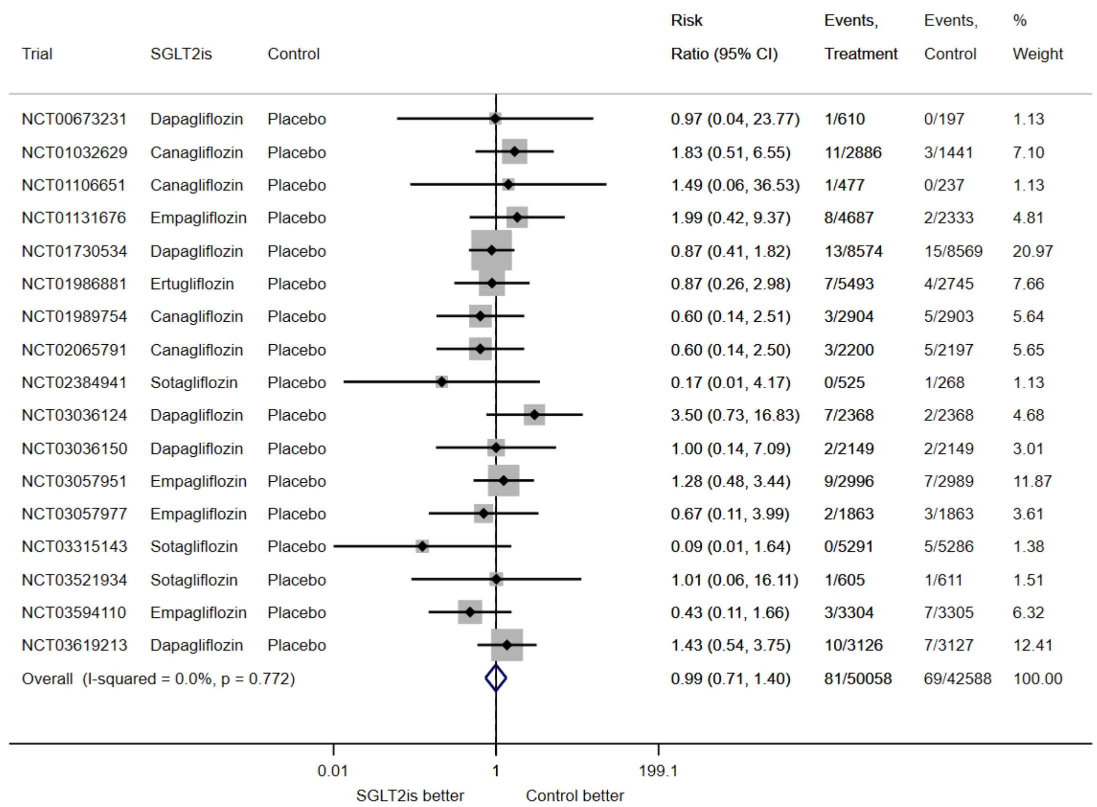

System 12-Injury, Poisoning And Procedural Complications-Figure S30 Meta-analysis of SGLT2is and Hip fracture

Trial	SGLT2is	Control	Risk Ratio (95% CI)	Events, Treatment	Events, Control	% Weight
NCT01032629	Canagliflozin	Placebo	3.00 (0.67, 13.37)	12/2886	2/1441	5.91
NCT01131676	Empagliflozin	Placebo	5.48 (0.30, 99.00)	5/4687	0/2333	1.58
NCT01164501	Empagliflozin	Placebo	0.25 (0.01, 6.21)	0/419	1/319	1.29
NCT01730534	Dapagliflozin	Placebo	0.55 (0.26, 1.15)	11/8574	20/8569	24.45
NCT01986881	Ertugliflozin	Placebo	0.50 (0.16, 1.55)	6/5493	6/2745	10.34
NCT01989754	Canagliflozin	Placebo	1.50 (0.25, 8.97)	3/2904	2/2903	4.13
NCT02065791	Canagliflozin	Placebo	2.00 (0.37, 10.89)	4/2200	2/2197	4.59
NCT03036124	Dapagliflozin	Placebo	2.00 (0.18, 22.04)	2/2368	1/2368	2.29
NCT03036150	Dapagliflozin	Placebo	1.00 (0.06, 15.98)	1/2149	1/2149	1.72
NCT03057951	Empagliflozin	Placebo	0.20 (0.02, 1.71)	1/2996	5/2989	2.87
NCT03057977	Empagliflozin	Placebo	2.00 (0.18, 22.04)	2/1863	1/1863	2.29
NCT03315143	Sotagliflozin	Placebo	0.87 (0.43, 1.79)	14/5291	16/5286	25.76
NCT03521934	Sotagliflozin	Placebo	1.01 (0.06, 16.11)	1/605	1/611	1.72
NCT03594110	Empagliflozin	Placebo	5.00 (0.58, 42.79)	5/3304	1/3305	2.87
NCT03619213	Dapagliflozin	Placebo	0.50 (0.13, 2.00)	3/3126	6/3127	6.89
NCT04157751	Empagliflozin	Placebo	3.05 (0.12, 74.43)	1/260	0/264	1.29
Overall (I-squared = 0.0%, p = 0.455)			0.88 (0.61, 1.26)	71/49125	65/42469	100.00

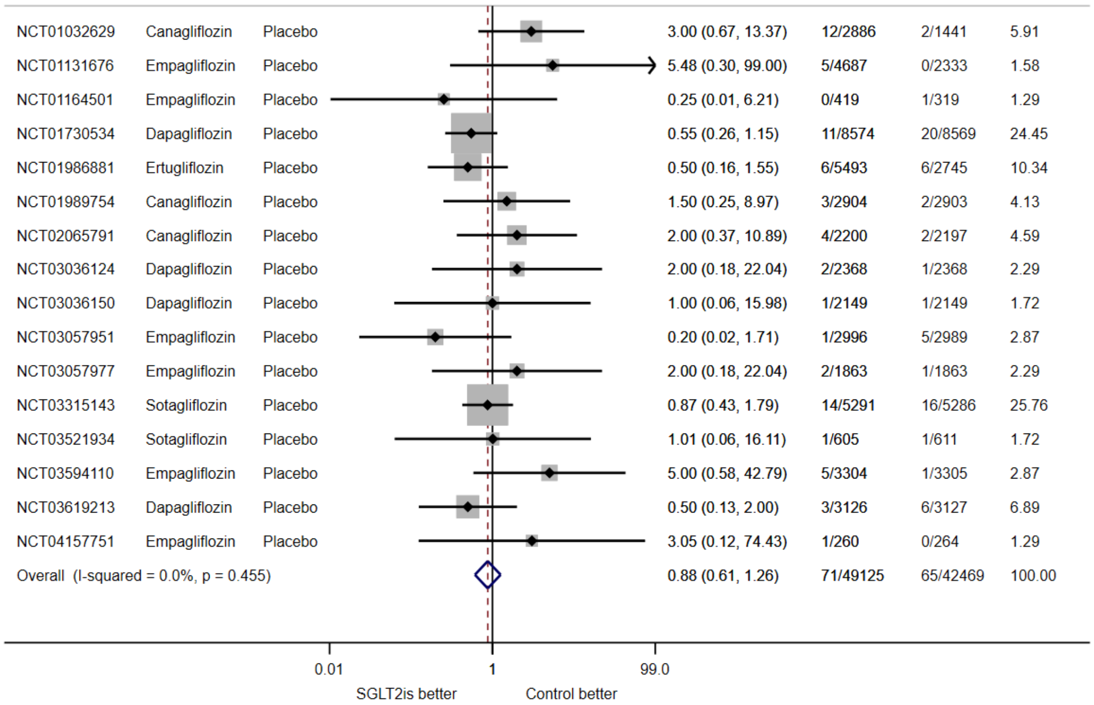

System 12-Injury, Poisoning And Procedural Complications-Figure S31 Meta-analysis of SGLT2is and Humerus fracture

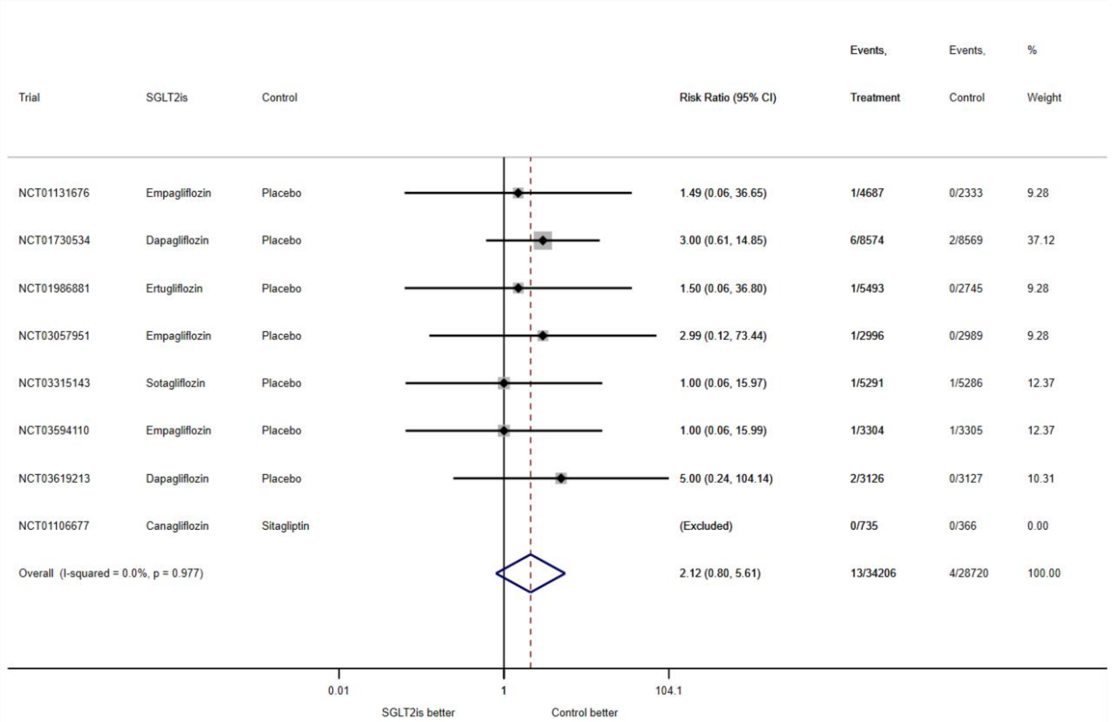

System 12-Injury, Poisoning And Procedural Complications-Figure S32 Meta-analysis of SGLT2is and Incisional hernia

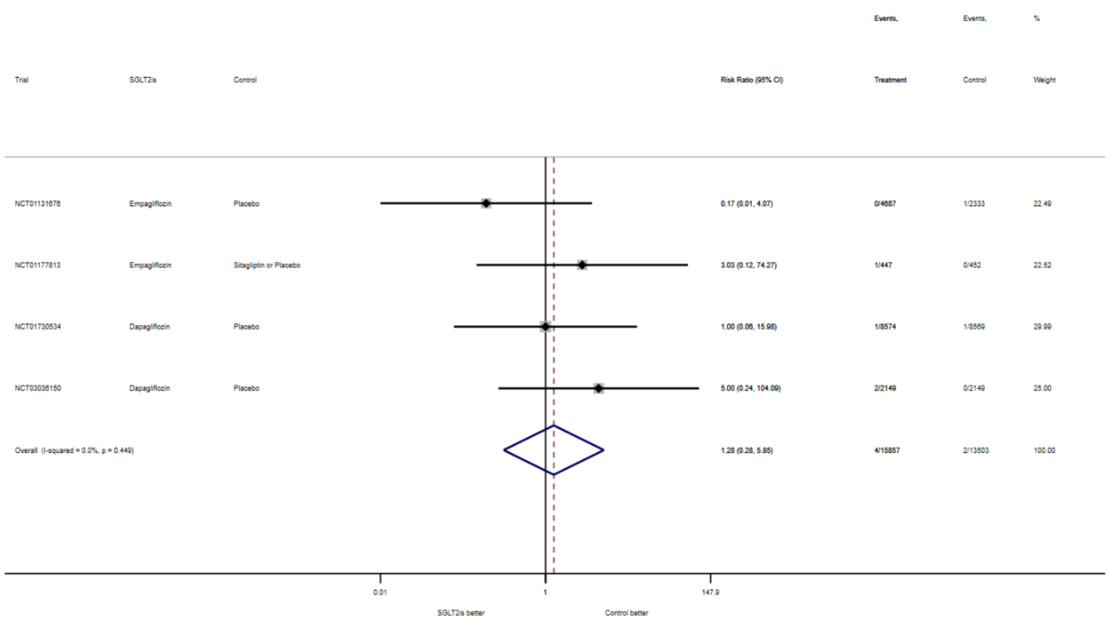

System 12-Injury, Poisoning And Procedural Complications-Figure S33 Meta-analysis of SGLT2is and Injury

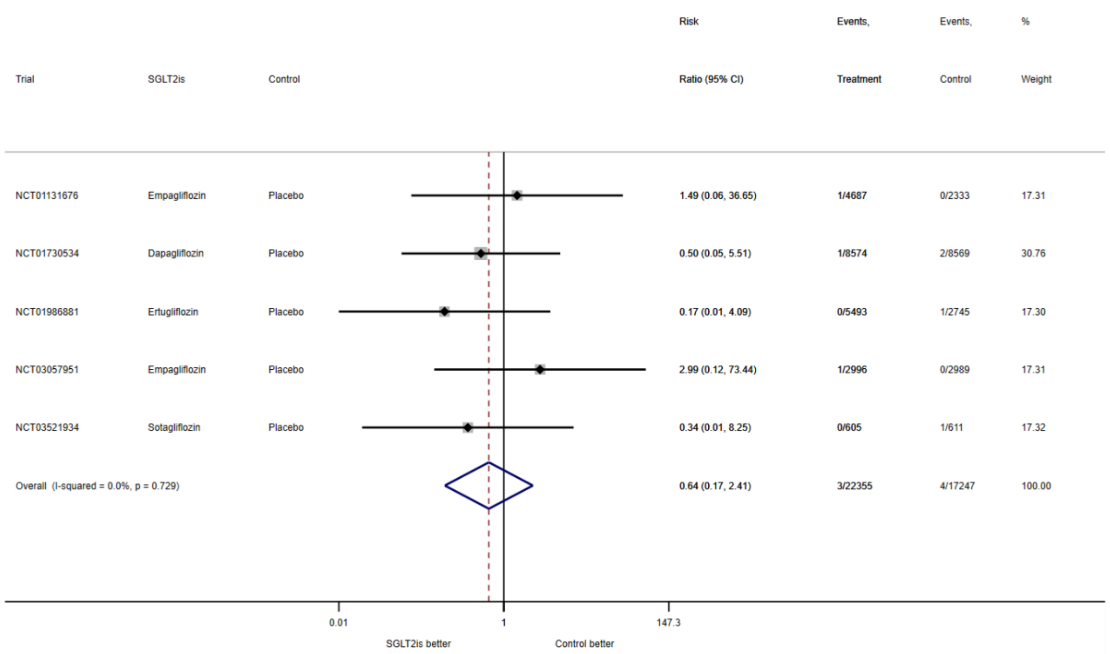

System 12-Injury, Poisoning And Procedural Complications-Figure S34 Meta-analysis of SGLT2is and Intentional overdose

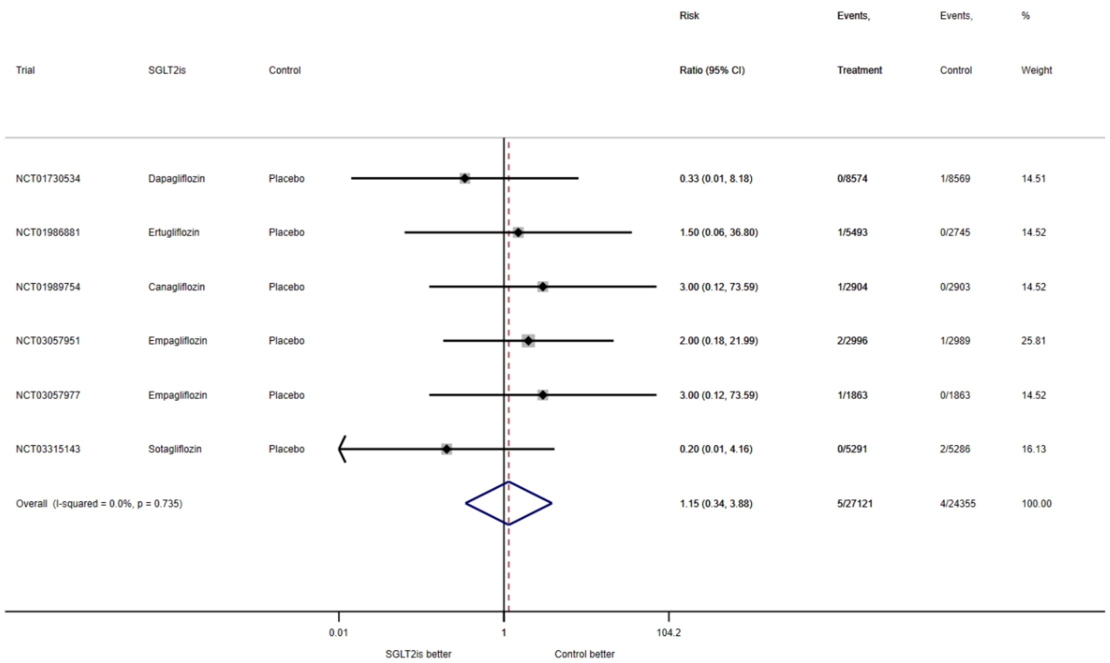

System 12-Injury, Poisoning And Procedural Complications-Figure S35 Meta-analysis of SGLT2is and Jaw fracture

Trial	SGLT2is	Control	Risk Ratio (95% CI)	Events, Treatment	Events, Control	% Weight
NCT01032629	Canagliflozin	Placebo	0.25 (0.02, 2.75)	1/2886	2/1441	11.19
NCT01106651	Canagliflozin	Placebo	0.17 (0.01, 4.06)	0/477	1/237	6.31
NCT01131676	Empagliflozin	Placebo	2.49 (0.12, 51.83)	2/4687	0/2333	6.99
NCT01730534	Dapagliflozin	Placebo	5.00 (0.58, 42.76)	5/8574	1/8569	13.98
NCT01986881	Ertugliflozin	Placebo	1.50 (0.16, 14.41)	3/5493	1/2745	12.59
NCT01989754	Canagliflozin	Placebo	3.00 (0.12, 73.59)	1/2904	0/2903	6.29
NCT02065791	Canagliflozin	Placebo	3.00 (0.12, 73.50)	1/2200	0/2197	6.29
NCT03036124	Dapagliflozin	Placebo	1.00 (0.06, 15.98)	1/2368	1/2368	8.39
NCT03036150	Dapagliflozin	Placebo	0.20 (0.01, 4.16)	0/2149	2/2149	6.99
NCT03057977	Empagliflozin	Placebo	0.33 (0.01, 8.18)	0/1863	1/1863	6.29
NCT03315143	Sotagliflozin	Placebo	0.33 (0.01, 8.17)	0/5291	1/5286	6.29
NCT03594110	Empagliflozin	Placebo	1.00 (0.06, 15.99)	1/3304	1/3305	8.39
Overall (I-squared = 0.0%, p = 0.713)			0.96 (0.43, 2.14)	15/42196	11/35396	100.00

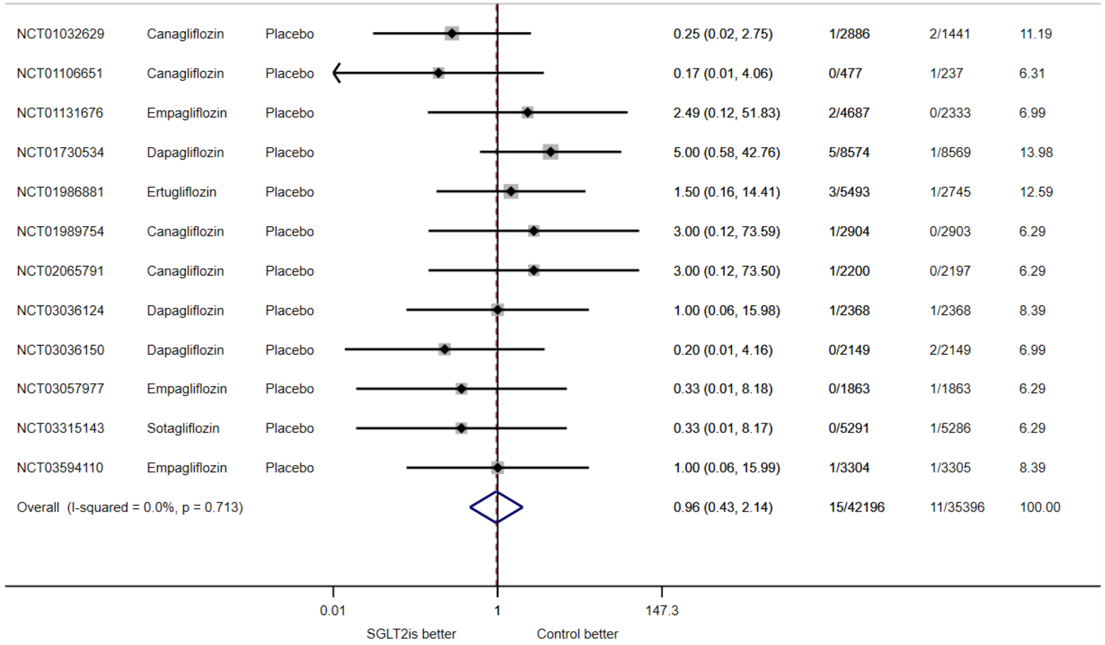

System 12-Injury, Poisoning And Procedural Complications-Figure S36 Meta-analysis of SGLT2is and Joint dislocation

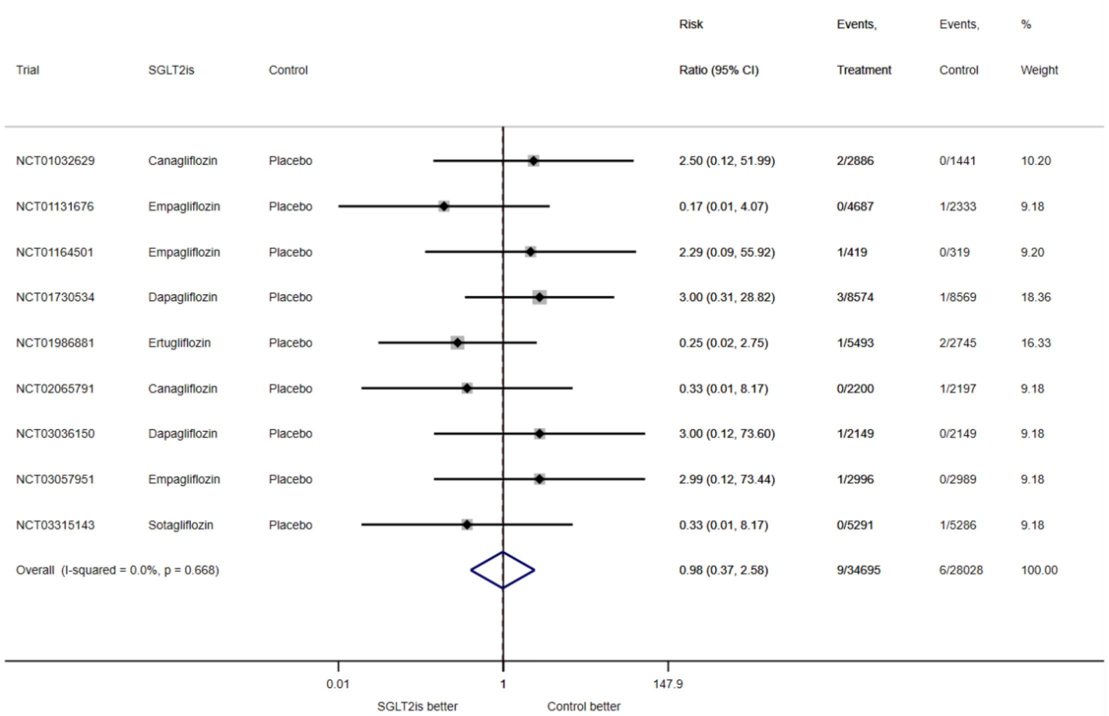

System 12-Injury, Poisoning And Procedural Complications-Figure S37 Meta-analysis of SGLT2is and Joint injury

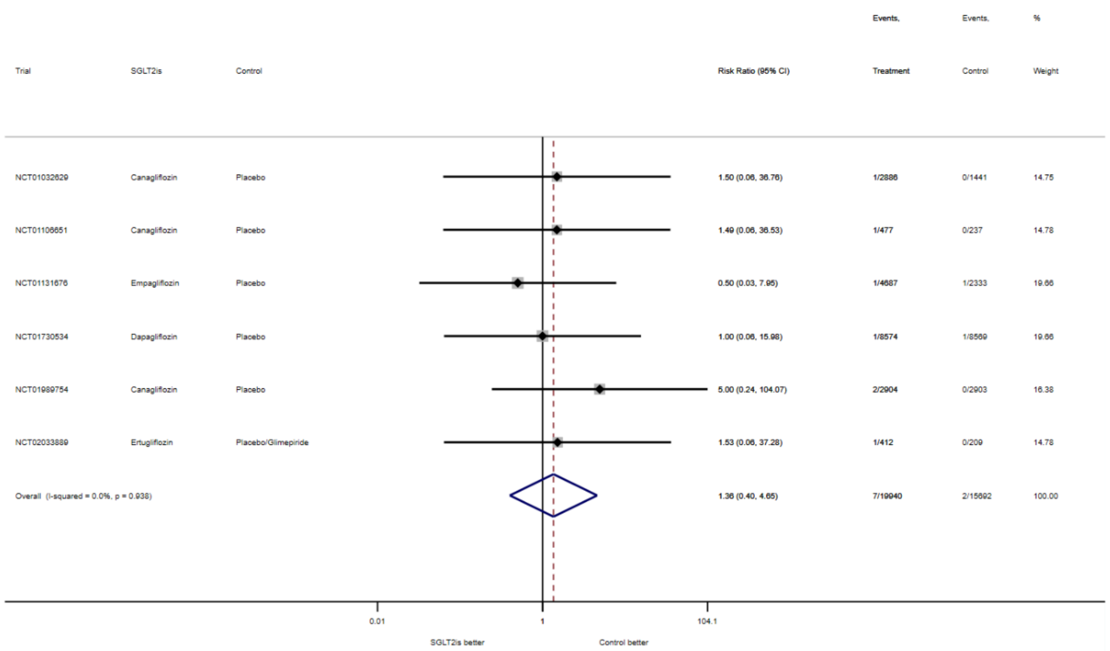

System 12-Injury, Poisoning And Procedural Complications-Figure S38 Meta-analysis of SGLT2is and Laceration

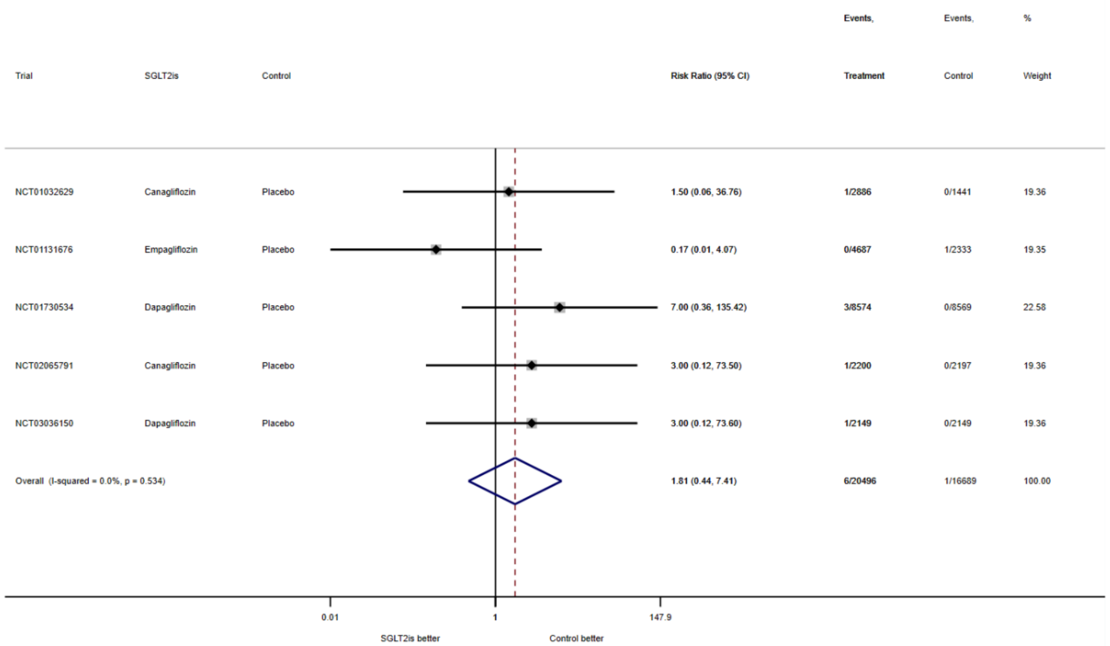

System 12-Injury, Poisoning And Procedural Complications-Figure S39 Meta-analysis of SGLT2is and Ligament rupture

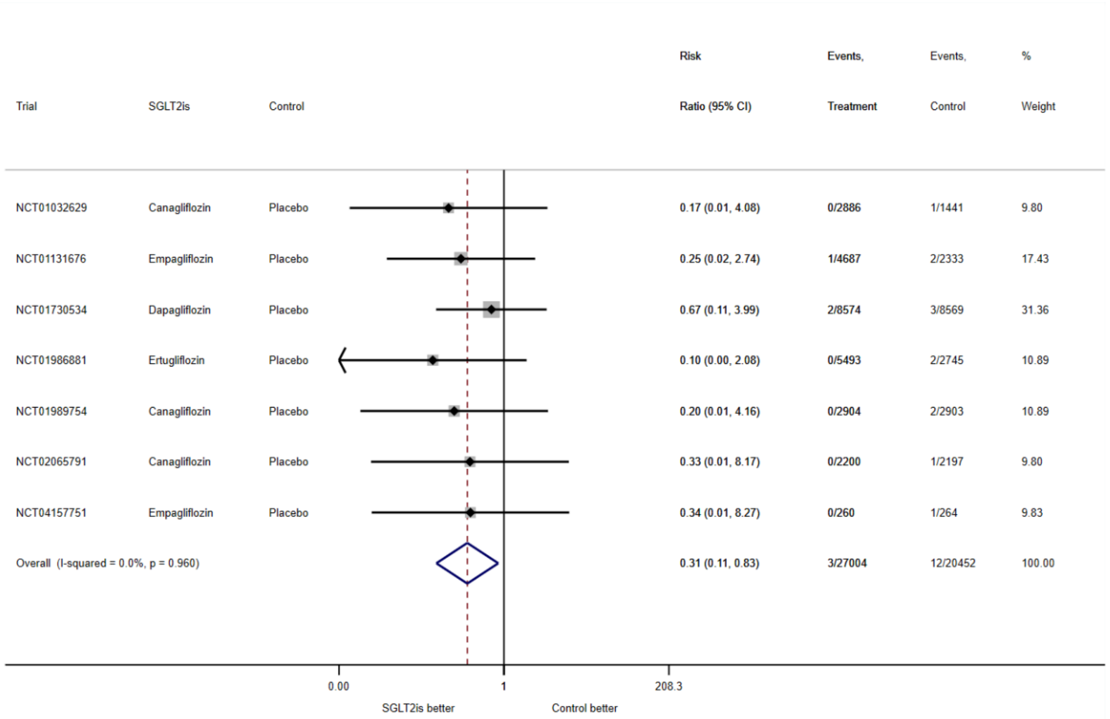

System 12-Injury, Poisoning And Procedural Complications-Figure S40 Meta-analysis of SGLT2is and Ligament sprain

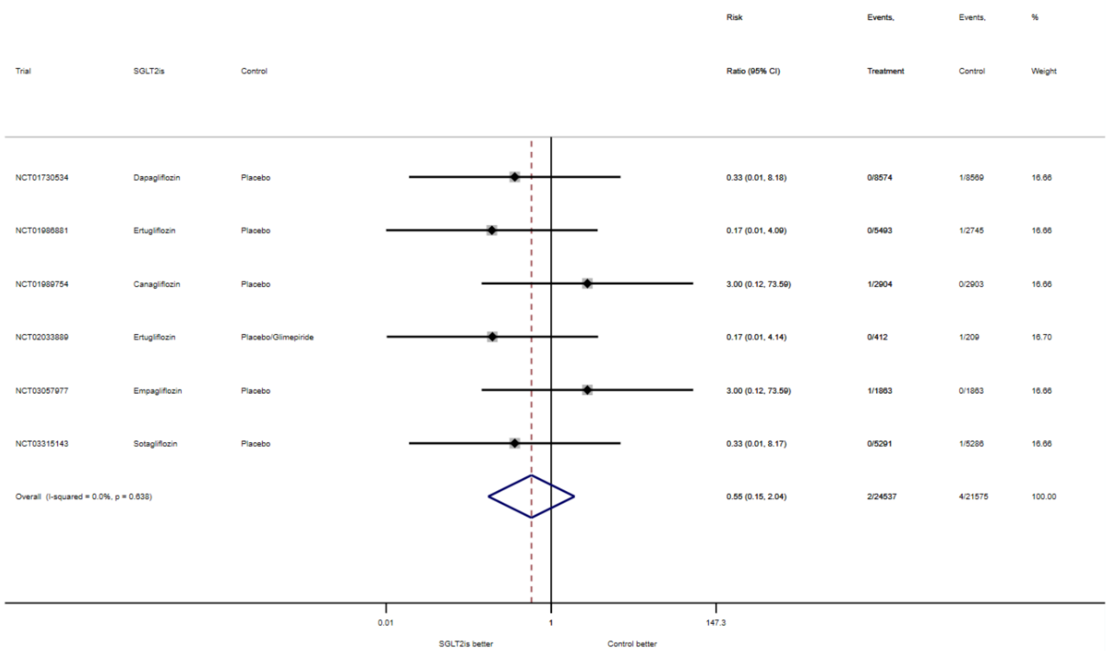

System 12-Injury, Poisoning And Procedural Complications-Figure S41 Meta-analysis of SGLT2is and Limb crushing injury

Trial	SGLT2is	Control	Risk Ratio (95% CI)	Events, Treatment	Events, Control	% Weight
NCT01032629	Canagliflozin	Placebo	3.50 (0.18, 67.64)	3/2886	0/1441	4.07
NCT01131676	Empagliflozin	Placebo	0.50 (0.03, 7.95)	1/4687	1/2333	4.66
NCT01164501	Empagliflozin	Placebo	0.25 (0.01, 6.21)	0/419	1/319	3.50
NCT01730534	Dapagliflozin	Placebo	1.75 (0.51, 5.97)	7/8574	4/8569	23.70
NCT01986881	Ertugliflozin	Placebo	3.50 (0.43, 28.42)	7/5493	1/2745	8.15
NCT01989754	Canagliflozin	Placebo	2.50 (0.49, 12.87)	5/2904	2/2903	13.31
NCT02065791	Canagliflozin	Placebo	3.00 (0.12, 73.50)	1/2200	0/2197	3.49
NCT02384941	Sotagliflozin	Placebo	1.53 (0.06, 37.53)	1/525	0/268	3.50
NCT03036124	Dapagliflozin	Placebo	3.00 (0.12, 73.61)	1/2368	0/2368	3.49
NCT03036150	Dapagliflozin	Placebo	1.00 (0.06, 15.98)	1/2149	1/2149	4.66
NCT03057951	Empagliflozin	Placebo	2.99 (0.12, 73.44)	1/2996	0/2989	3.49
NCT03057977	Empagliflozin	Placebo	1.00 (0.06, 15.98)	1/1863	1/1863	4.66
NCT03242252	Sotagliflozin	Placebo	1.48 (0.06, 36.28)	1/527	0/260	3.50
NCT03315143	Sotagliflozin	Placebo	1.00 (0.06, 15.97)	1/5291	1/5286	4.65
NCT03619213	Dapagliflozin	Placebo	0.67 (0.11, 3.99)	2/3126	3/3127	11.18
Overall (I-squared = 0.0%, p = 0.989)			1.53 (0.84, 2.78)	33/46008	15/38817	100.00

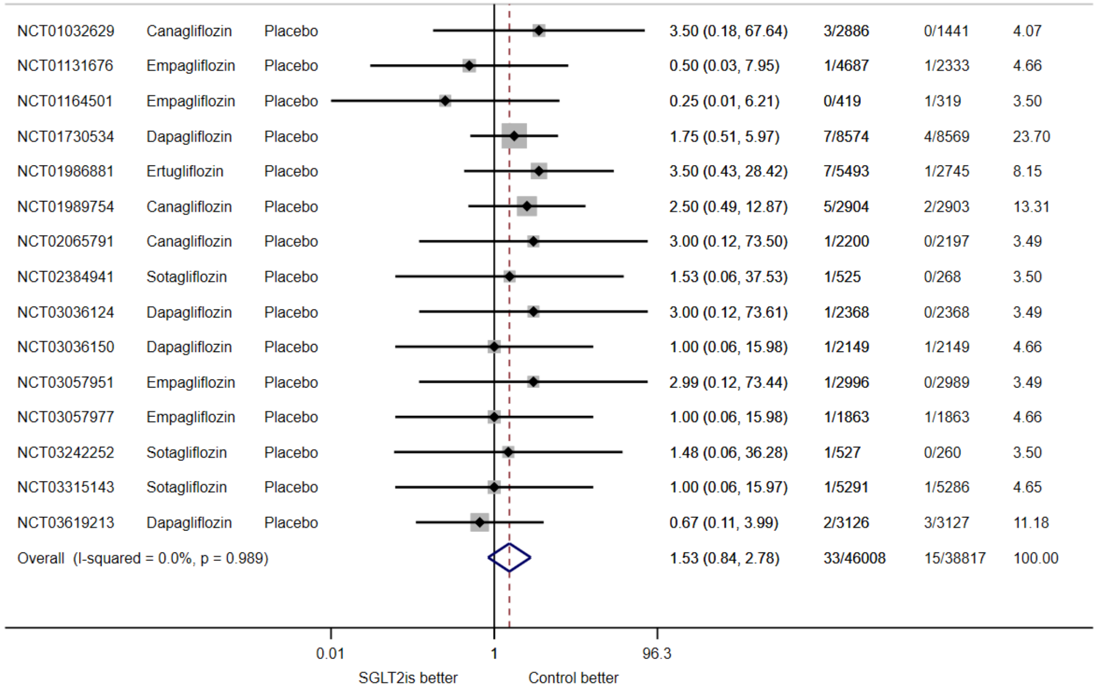

System 12-Injury, Poisoning And Procedural Complications-Figure S42 Meta-analysis of SGLT2is and Limb injury

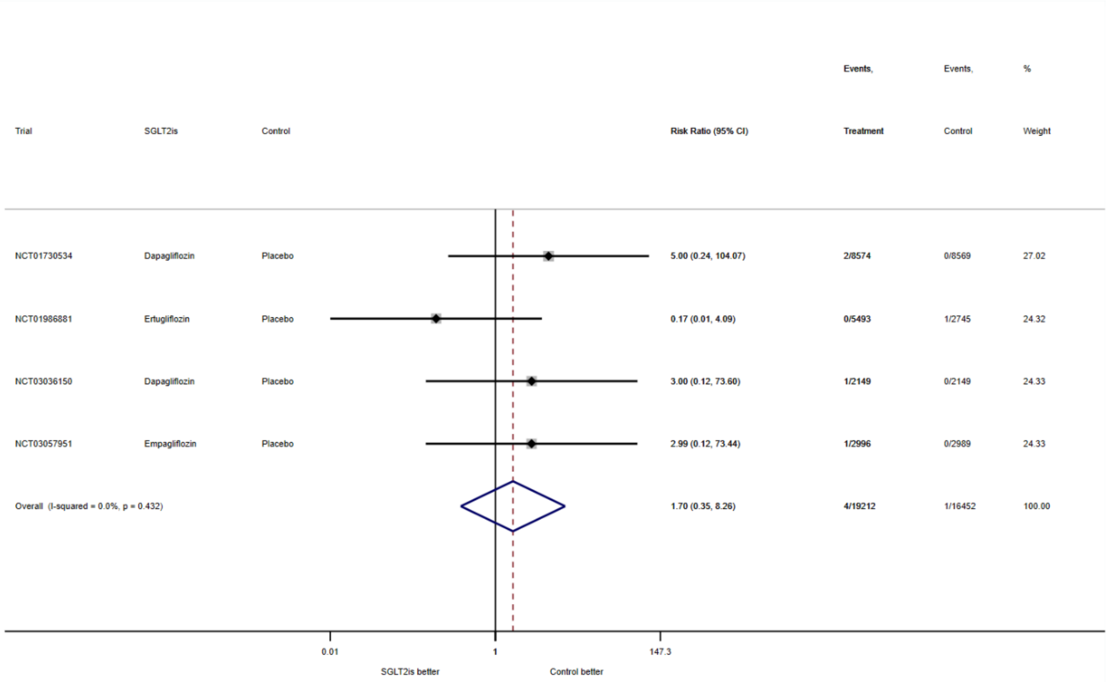

System 12-Injury, Poisoning And Procedural Complications-Figure S43 Meta-analysis of SGLT2is and Limb traumatic amputation

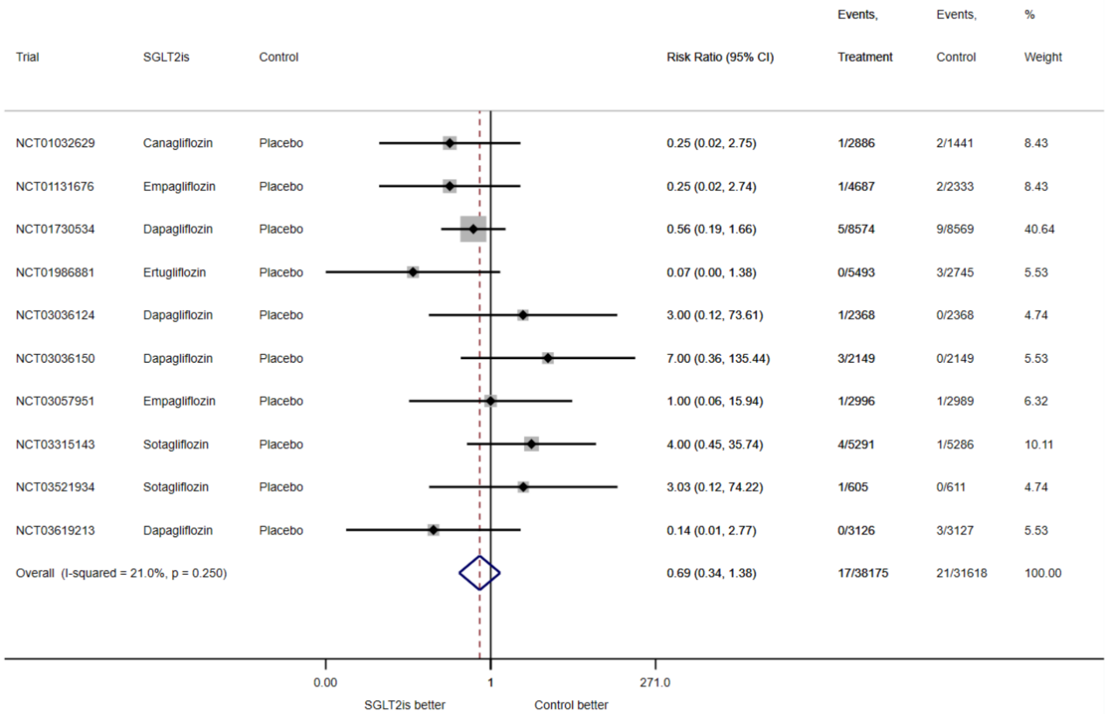

System 12-Injury, Poisoning And Procedural Complications-Figure S44 Meta-analysis of SGLT2is and Lower limb fracture

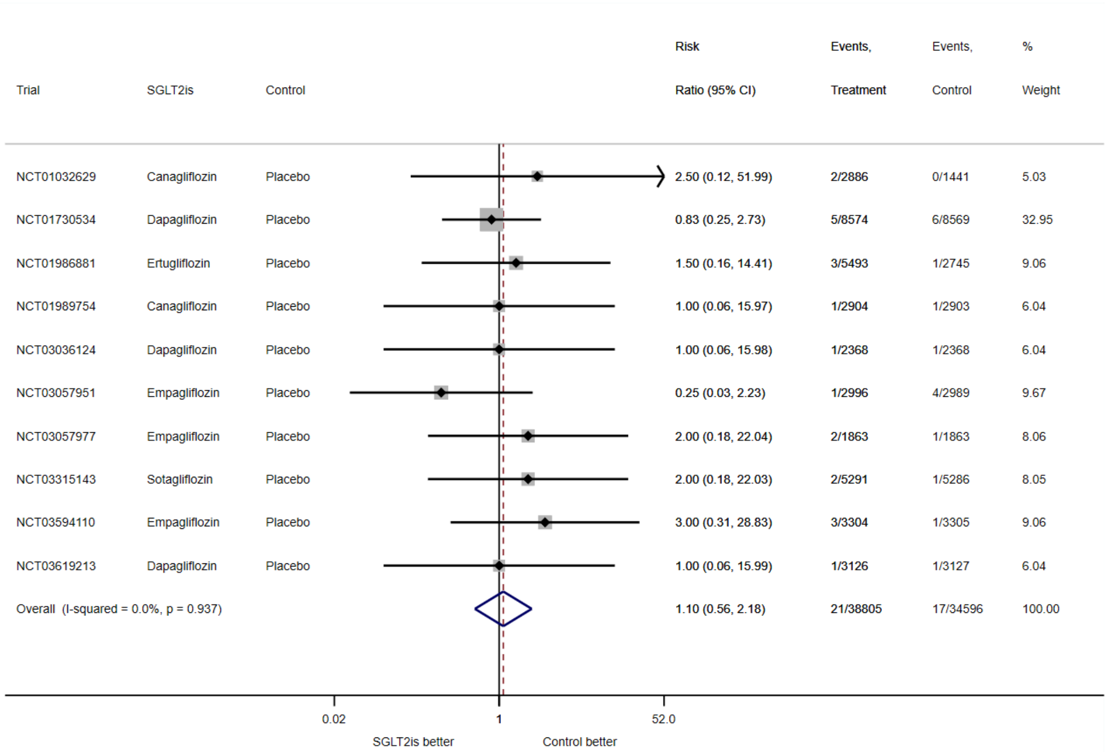

System 12-Injury, Poisoning And Procedural Complications-Figure S45 Meta-analysis of SGLT2is and Lumbar vertebral fracture

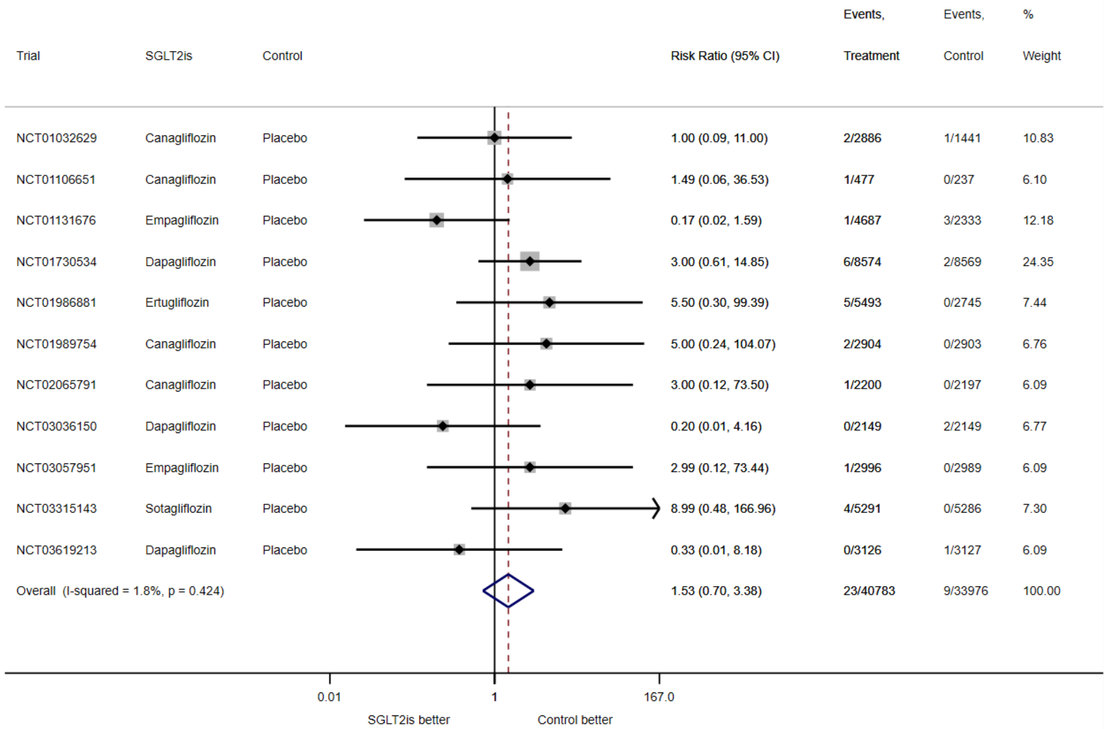

System 12-Injury, Poisoning And Procedural Complications-Figure S46 Meta-analysis of SGLT2is and Meniscus injury

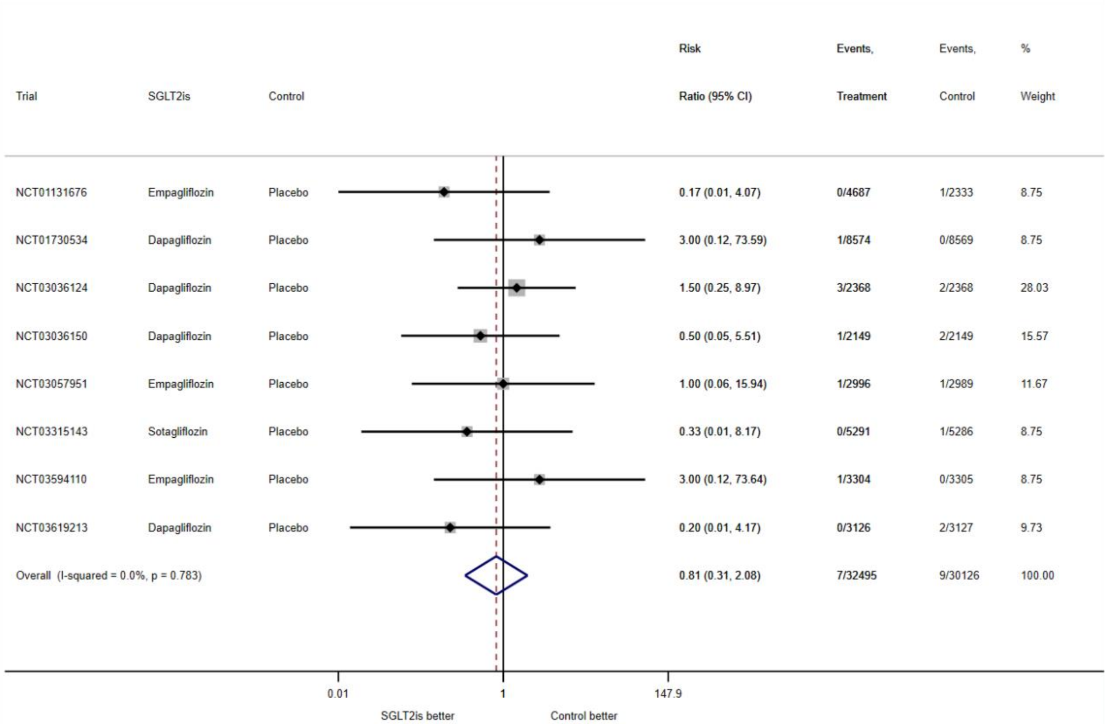

System 12-Injury, Poisoning And Procedural Complications-Figure S47 Meta-analysis of SGLT2is and Multiple fractures

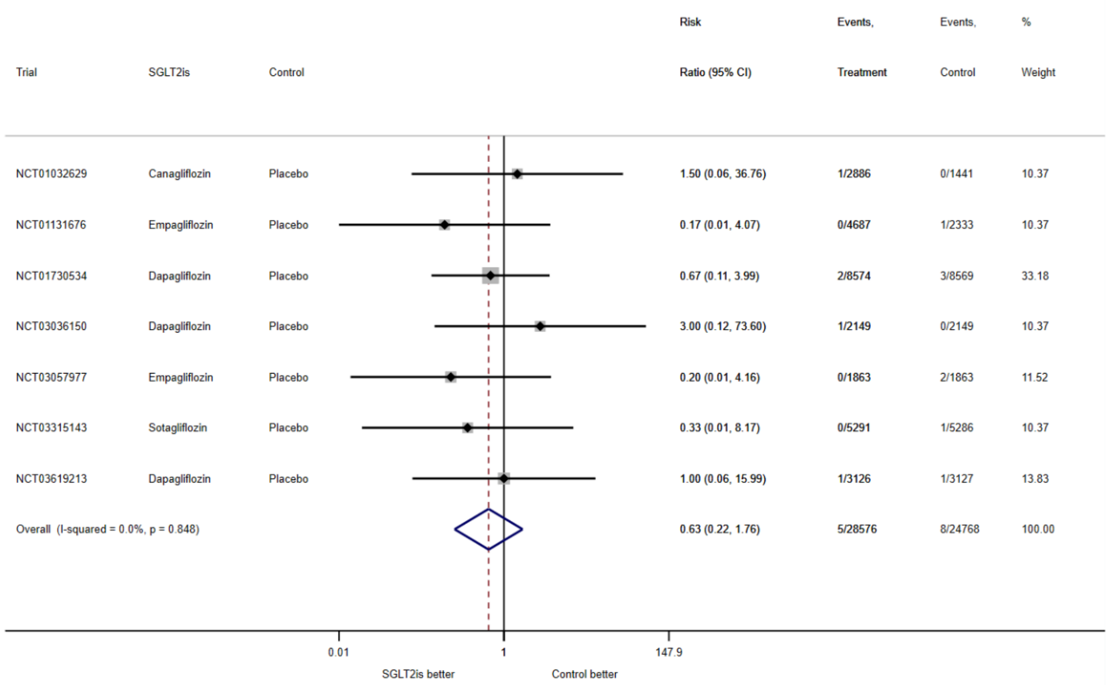

System 12-Injury, Poisoning And Procedural Complications-Figure S48 Meta-analysis of SGLT2is and Multiple injuries

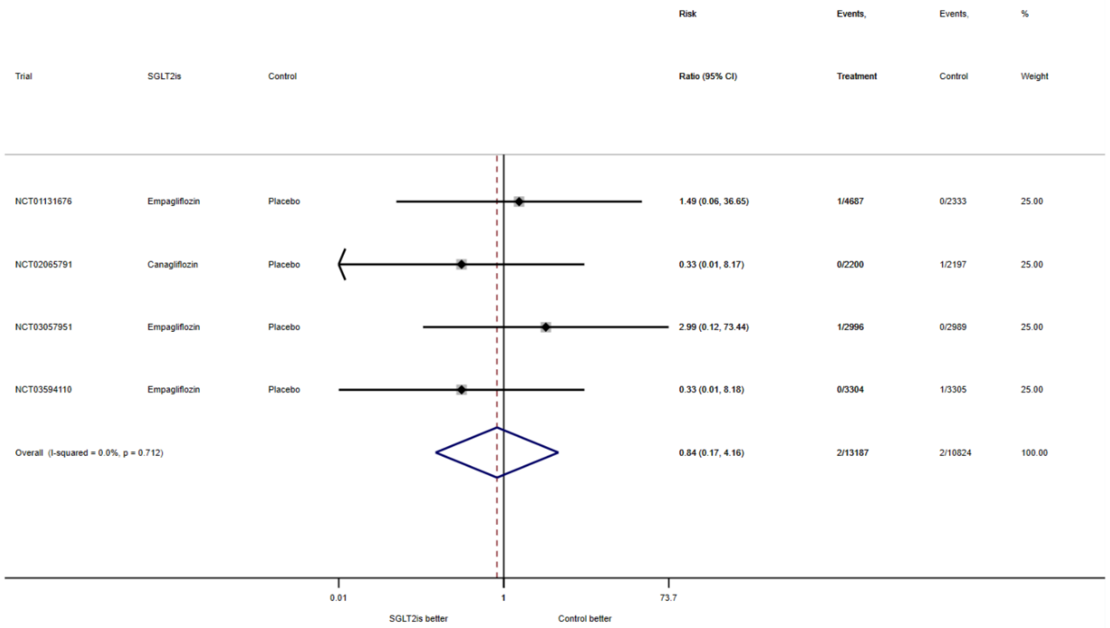

System 12-Injury, Poisoning And Procedural Complications-Figure S49 Meta-analysis of SGLT2is and Muscle injury

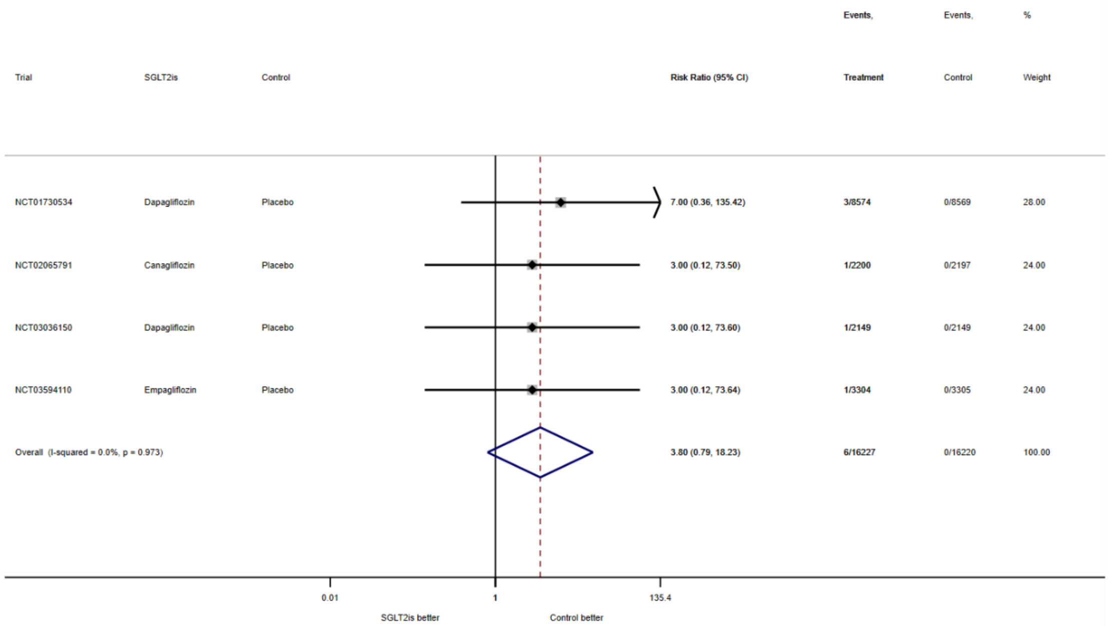

System 12-Injury, Poisoning And Procedural Complications-Figure S50 Meta-analysis of SGLT2is and Muscle strain

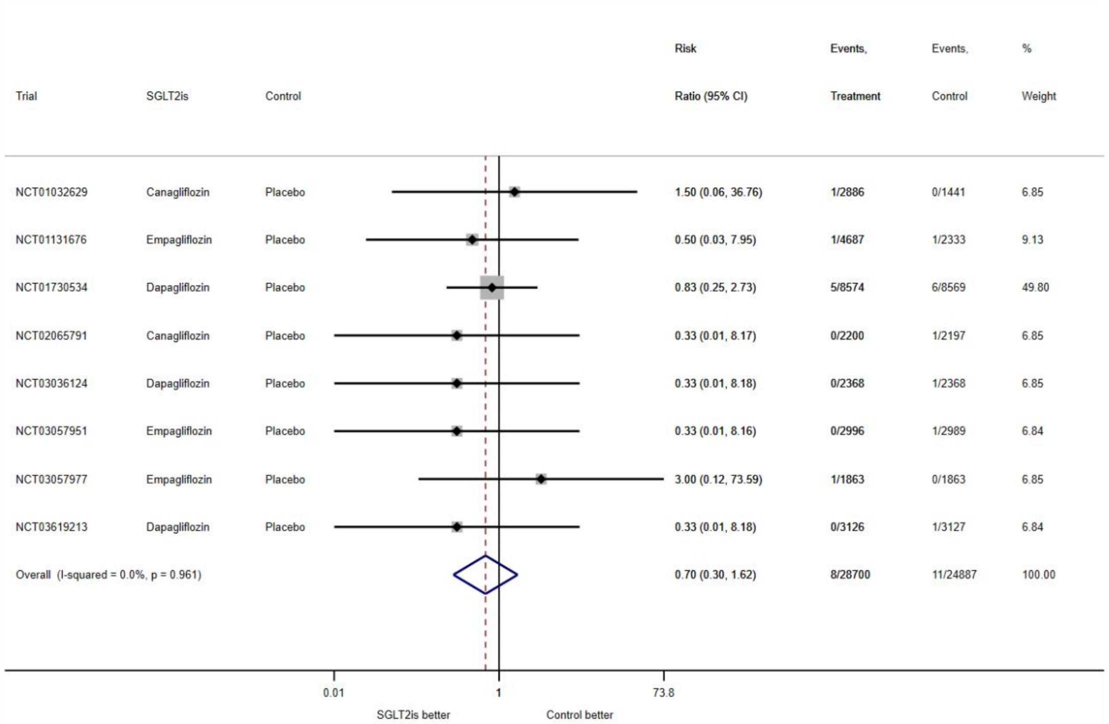

System 12-Injury, Poisoning And Procedural Complications-Figure S51 Meta-analysis of SGLT2is and Overdose

Trial	SGLT2is	Control	Risk Ratio (95% CI)	Events, Treatment	Events, Control	% Weight
NCT01031680	Dapagliflozin	Placebo	0.33 (0.01, 8.20)	0/460	1/462	5.72
NCT01032629	Canagliflozin	Placebo	3.50 (0.18, 67.64)	3/2886	0/1441	6.67
NCT01730534	Dapagliflozin	Placebo	3.00 (0.31, 28.82)	3/8574	1/8569	11.43
NCT01986881	Ertugliflozin	Placebo	1.50 (0.06, 36.80)	1/5493	0/2745	5.71
NCT01989754	Canagliflozin	Placebo	0.50 (0.05, 5.51)	1/2904	2/2903	10.16
NCT02065791	Canagliflozin	Placebo	0.33 (0.01, 8.17)	0/2200	1/2197	5.71
NCT03036124	Dapagliflozin	Placebo	3.00 (0.12, 73.61)	1/2368	0/2368	5.71
NCT03036150	Dapagliflozin	Placebo	1.00 (0.06, 15.98)	1/2149	1/2149	7.62
NCT03057951	Empagliflozin	Placebo	1.00 (0.06, 15.94)	1/2996	1/2989	7.62
NCT03315143	Sotagliflozin	Placebo	0.50 (0.09, 2.73)	2/5291	4/5286	20.32
NCT03594110	Empagliflozin	Placebo	0.33 (0.01, 8.18)	0/3304	1/3305	5.71
NCT03619213	Dapagliflozin	Placebo	1.00 (0.06, 15.99)	1/3126	1/3127	7.62
Overall (I-squared = 0.0%, p = 0.958)			0.90 (0.42, 1.93)	14/41751	13/37541	100.00

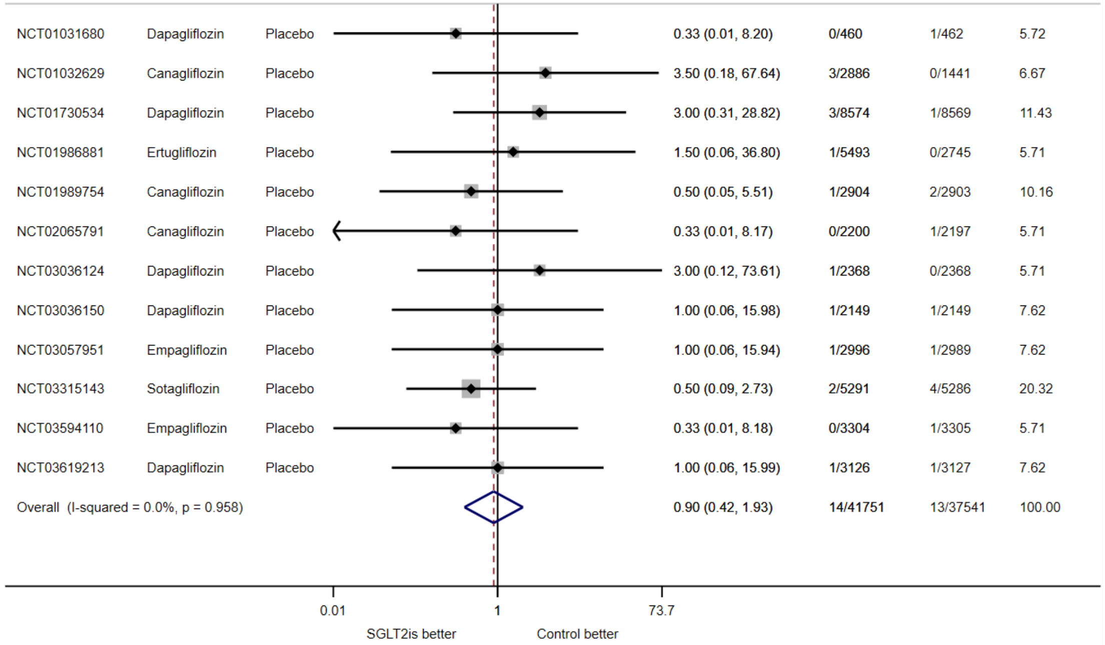

System 12-Injury, Poisoning And Procedural Complications-Figure S52 Meta-analysis of SGLT2is and Patella fracture

Trial	SGLT2is	Control	Risk Ratio (95% CI)	Events, Treatment	Events, Control	% Weight
NCT01032629	Canagliflozin	Placebo	0.50 (0.03, 7.98)	1/2886	1/1441	5.85
NCT01131676	Empagliflozin	Placebo	1.49 (0.06, 36.65)	1/4687	0/2333	4.39
NCT01164501	Empagliflozin	Placebo	2.29 (0.09, 55.92)	1/419	0/319	4.40
NCT01730534	Dapagliflozin	Placebo	0.75 (0.17, 3.35)	3/8574	4/8569	20.06
NCT01986881	Ertugliflozin	Placebo	1.50 (0.06, 36.80)	1/5493	0/2745	4.39
NCT03036124	Dapagliflozin	Placebo	0.33 (0.01, 8.18)	0/2368	1/2368	4.39
NCT03036150	Dapagliflozin	Placebo	3.00 (0.12, 73.60)	1/2149	0/2149	4.39
NCT03057951	Empagliflozin	Placebo	0.43 (0.11, 1.65)	3/2996	7/2989	24.60
NCT03057977	Empagliflozin	Placebo	0.33 (0.03, 3.20)	1/1863	3/1863	8.78
NCT03315143	Sotagliflozin	Placebo	6.99 (0.36, 135.36)	3/5291	0/5286	5.12
NCT03521934	Sotagliflozin	Placebo	3.03 (0.12, 74.22)	1/605	0/611	4.39
NCT03594110	Empagliflozin	Placebo	0.33 (0.01, 8.18)	0/3304	1/3305	4.39
NCT03619213	Dapagliflozin	Placebo	0.20 (0.01, 4.17)	0/3126	2/3127	4.87
Overall (I-squared = 0.0%, p = 0.863)			0.73 (0.37, 1.43)	16/43761	19/37105	100.00

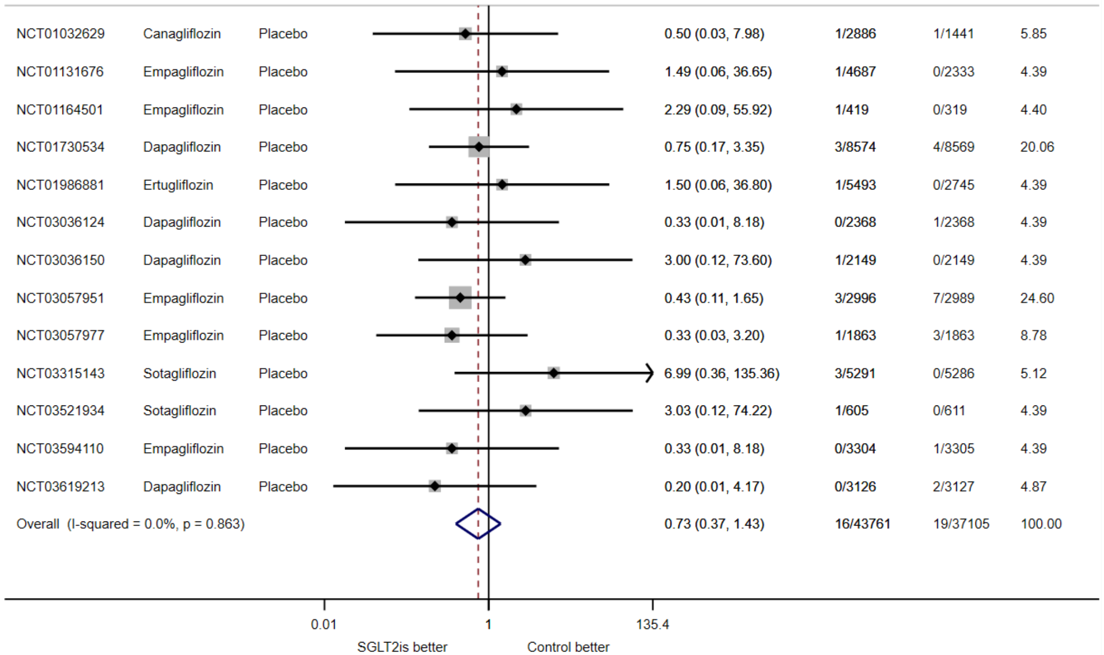

System 12-Injury, Poisoning And Procedural Complications-Figure S53 Meta-analysis of SGLT2is and Pelvic fracture

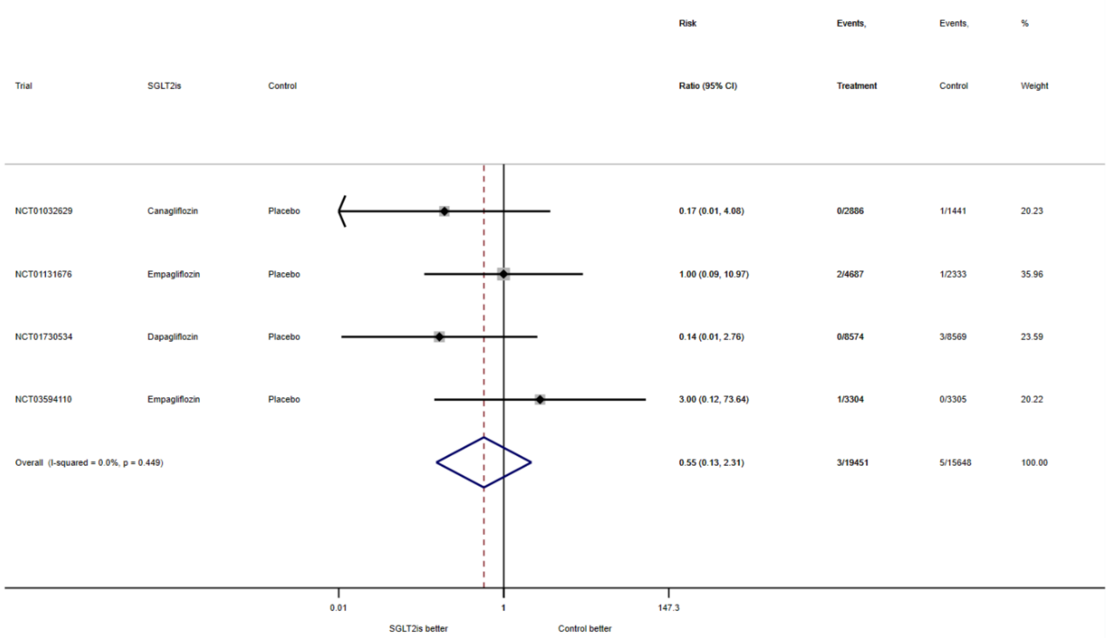

System 12-Injury, Poisoning And Procedural Complications-Figure S54 Meta-analysis of SGLT2is and Peripheral artery restenosis

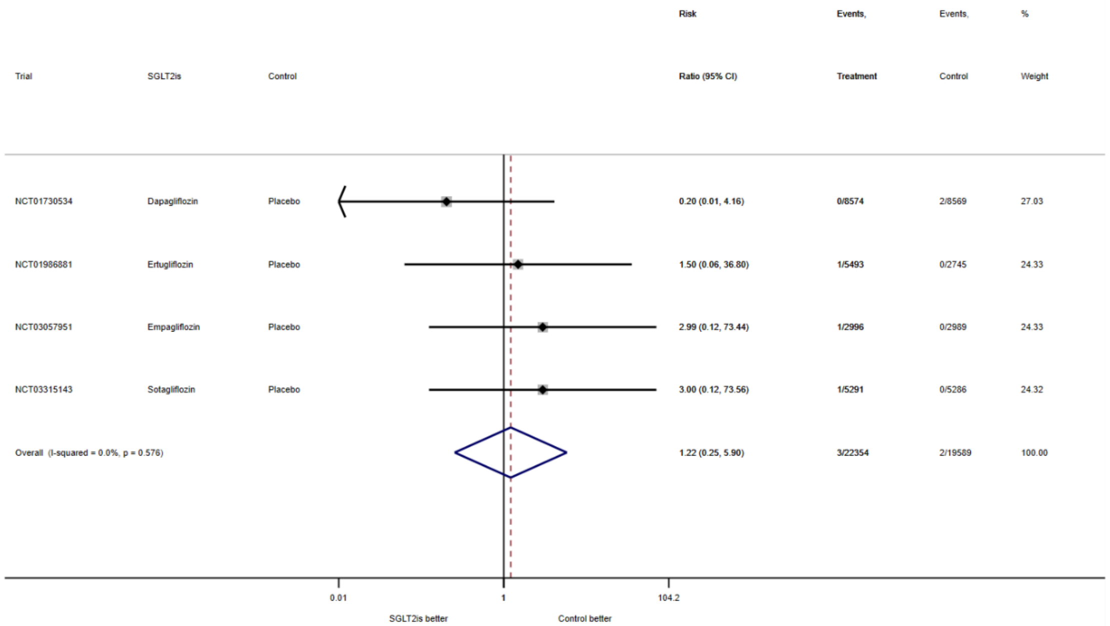

System 12-Injury, Poisoning And Procedural Complications-Figure S55 Meta-analysis of SGLT2is and Periprocedural myocardial infarction

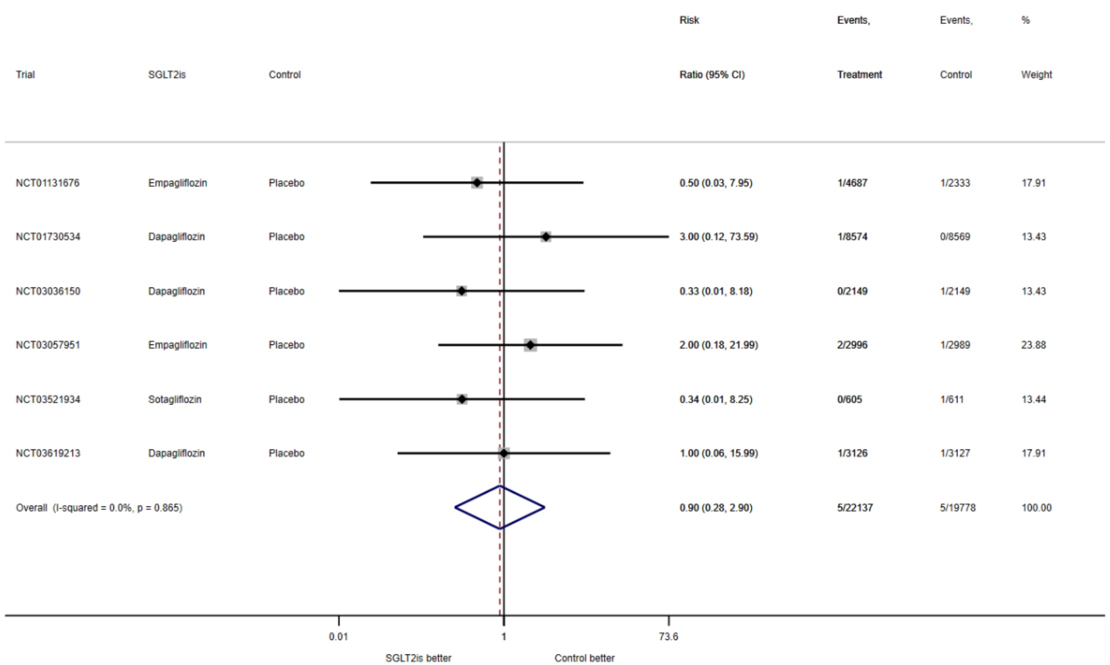

System 12-Injury, Poisoning And Procedural Complications-Figure S56 Meta-analysis of SGLT2is and Periprosthetic fracture

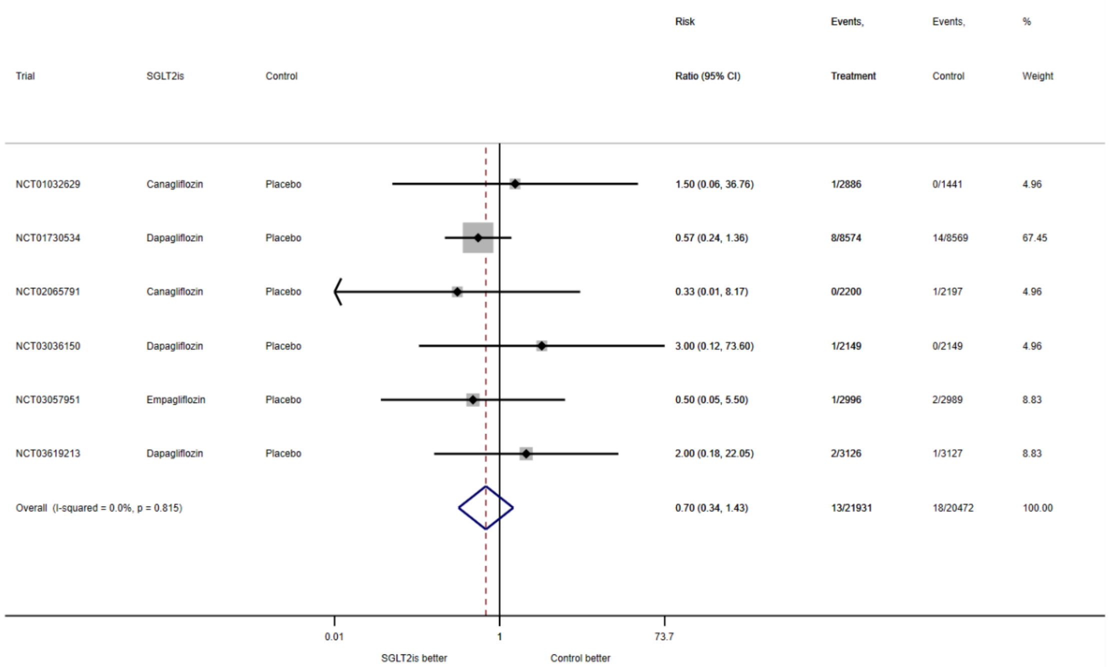

System 12-Injury, Poisoning And Procedural Complications-Figure S57 Meta-analysis of SGLT2is and Post procedural complication

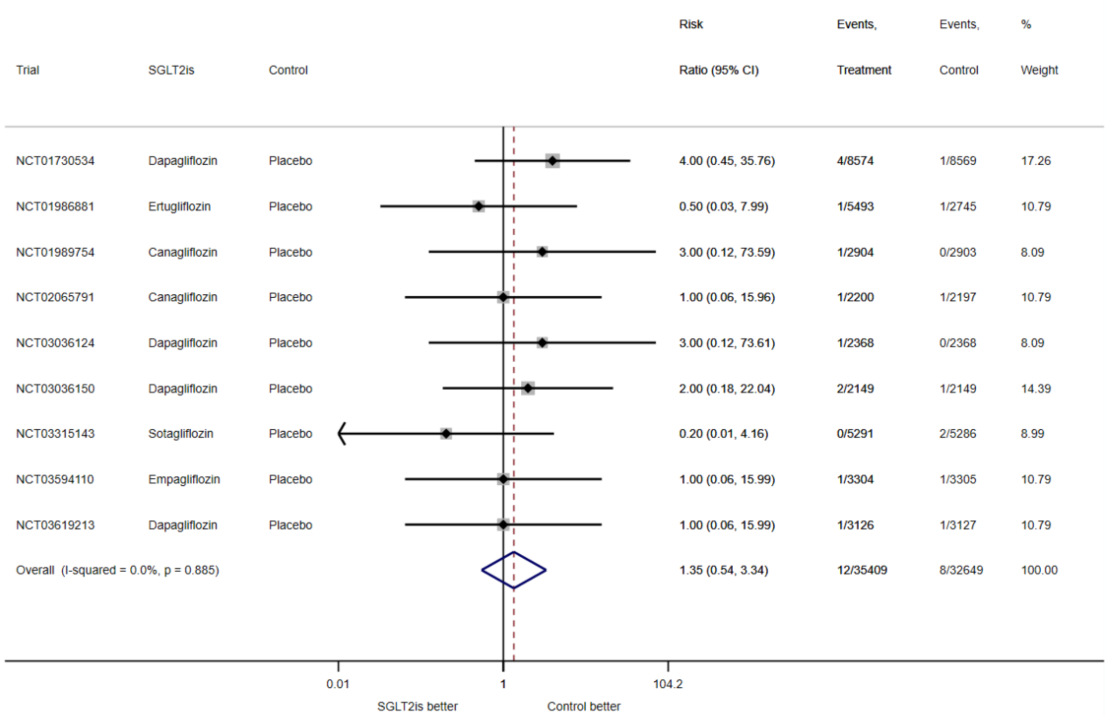

System 12-Injury, Poisoning And Procedural Complications-Figure S58 Meta-analysis of SGLT2is and Post procedural haematoma

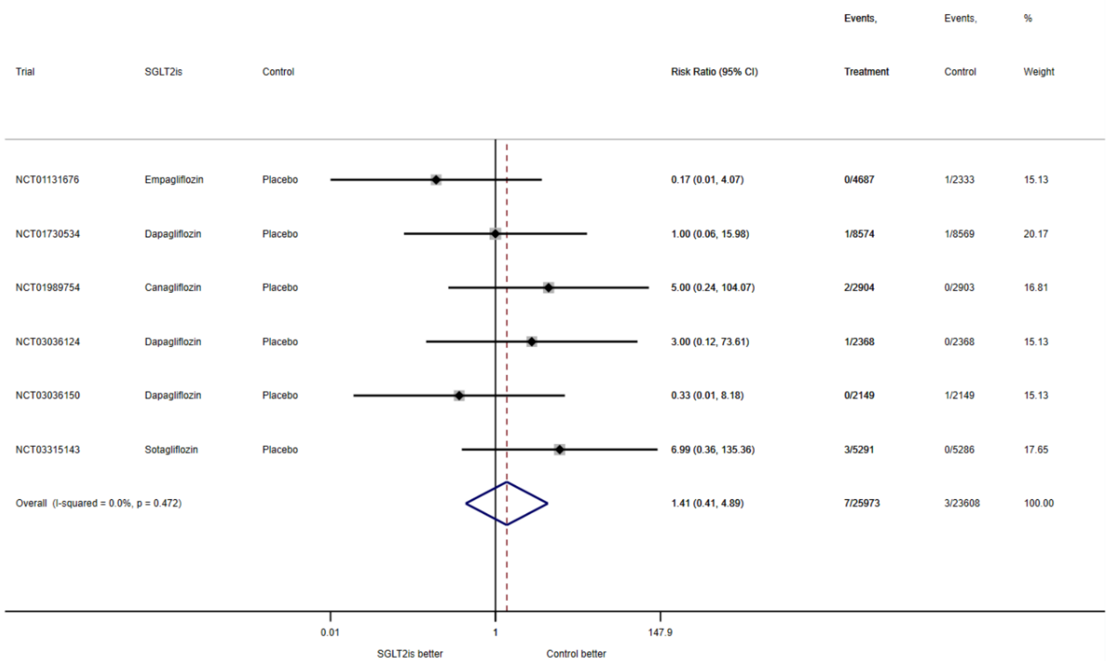

System 12-Injury, Poisoning And Procedural Complications-Figure S59 Meta-analysis of SGLT2is and Post procedural haematuria

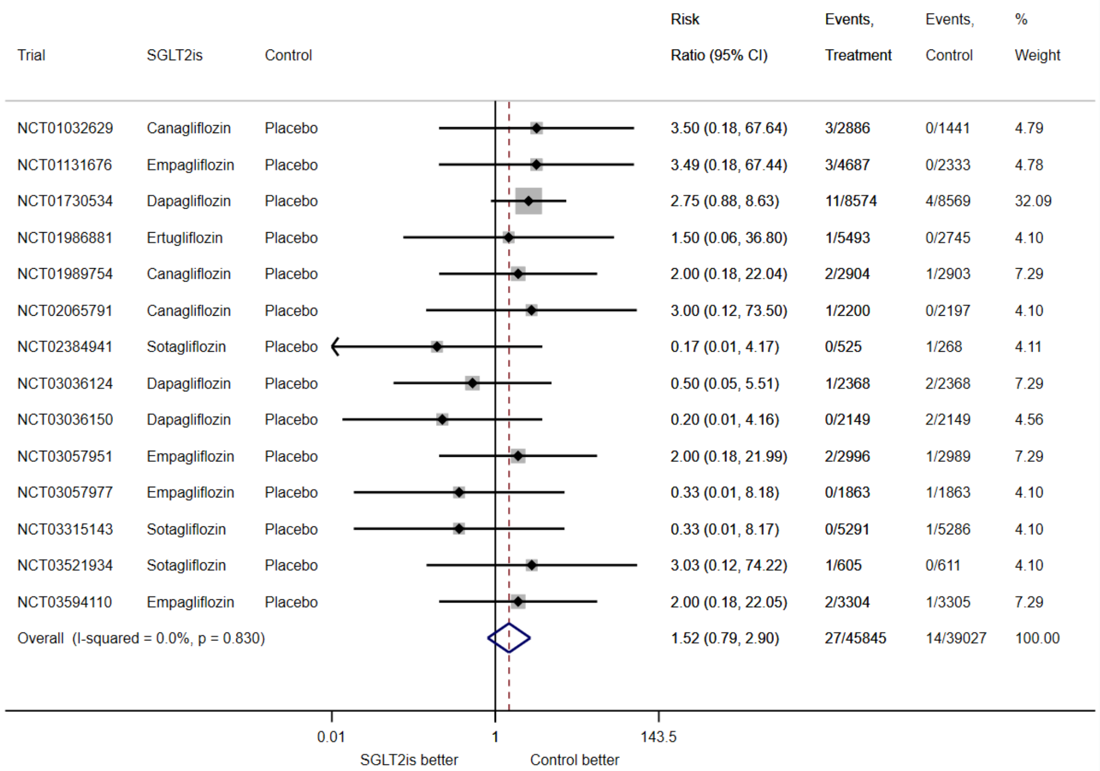

System 12-Injury, Poisoning And Procedural Complications-Figure S60 Meta-analysis of SGLT2is and Post procedural haemorrhage

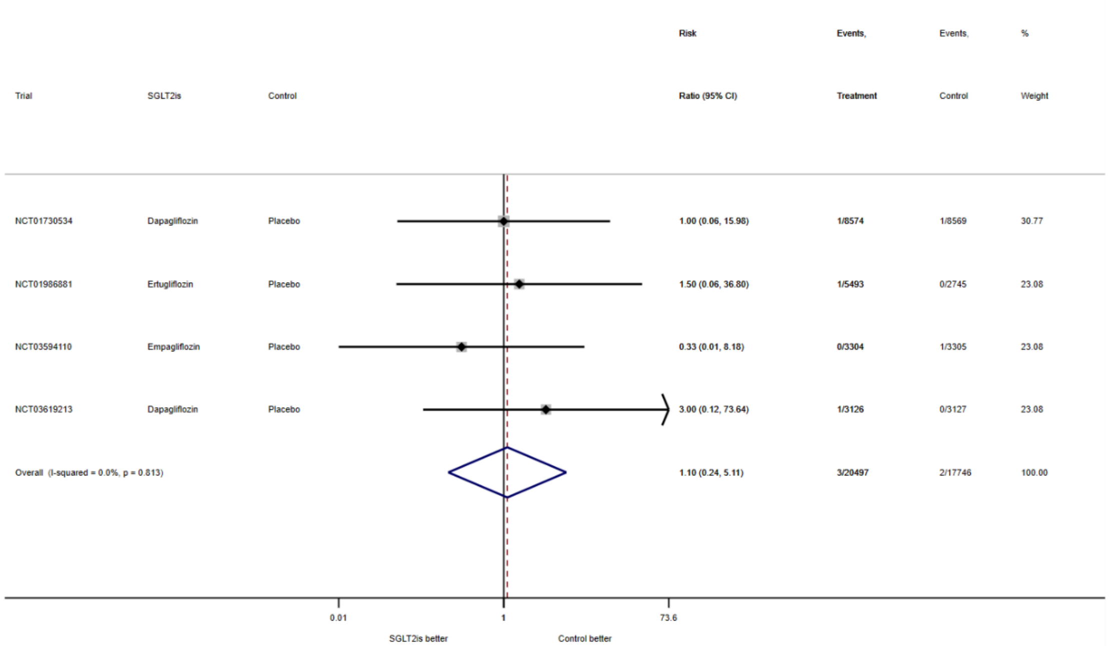

System 12-Injury, Poisoning And Procedural Complications-Figure S61 Meta-analysis of SGLT2is and Post procedural myocardial infarction

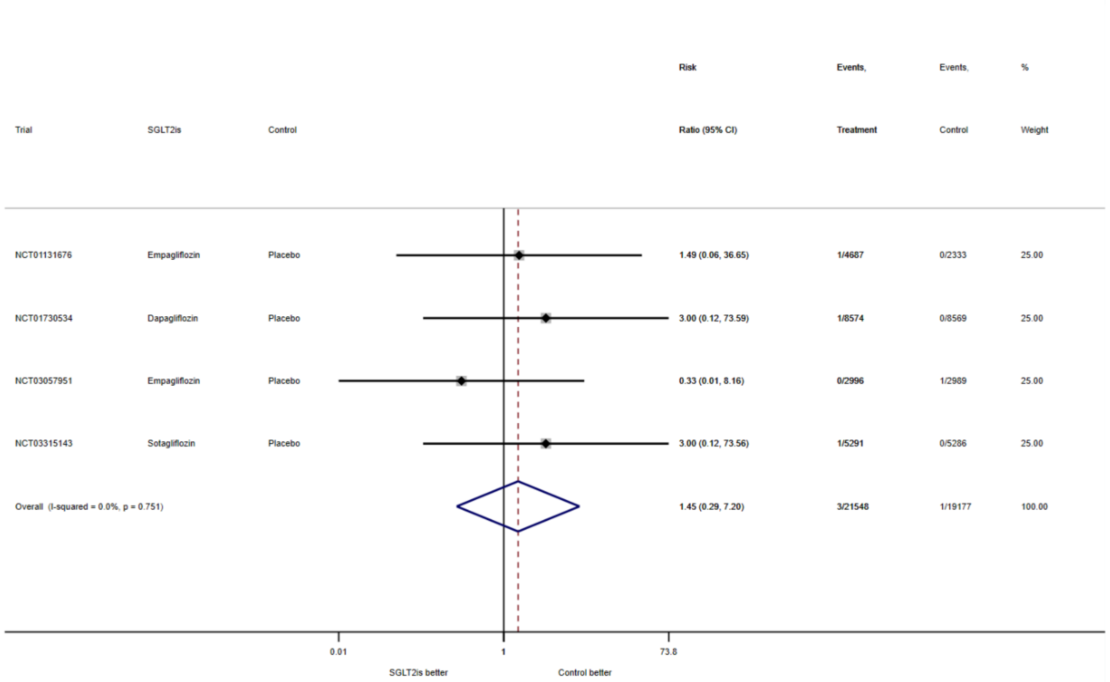

System 12-Injury, Poisoning And Procedural Complications-Figure S62 Meta-analysis of SGLT2is and Postoperative ileus

Trial	SGLT2is	Control	Risk Ratio (95% CI)	Events, Treatment	Events, Control	% Weight
NCT01032629	Canagliflozin	Placebo	0.17 (0.01, 4.08)	0/2886	1/1441	13.64
NCT01730534	Dapagliflozin	Placebo	1.00 (0.06, 15.98)	1/8574	1/8569	18.18
NCT01986881	Ertugliflozin	Placebo	1.50 (0.06, 36.80)	1/5493	0/2745	13.64
NCT01989754	Canagliflozin	Placebo	3.00 (0.12, 73.59)	1/2904	0/2903	13.64
NCT03057977	Empagliflozin	Placebo	3.00 (0.12, 73.59)	1/1863	0/1863	13.64
NCT03315143	Sotagliflozin	Placebo	3.00 (0.12, 73.56)	1/5291	0/5286	13.63
NCT03619213	Dapagliflozin	Placebo	0.33 (0.01, 8.18)	0/3126	1/3127	13.64
Overall (I-squared = 0.0%, p = 0.803)			1.12 (0.34, 3.64)	5/30137	3/25934	100.00

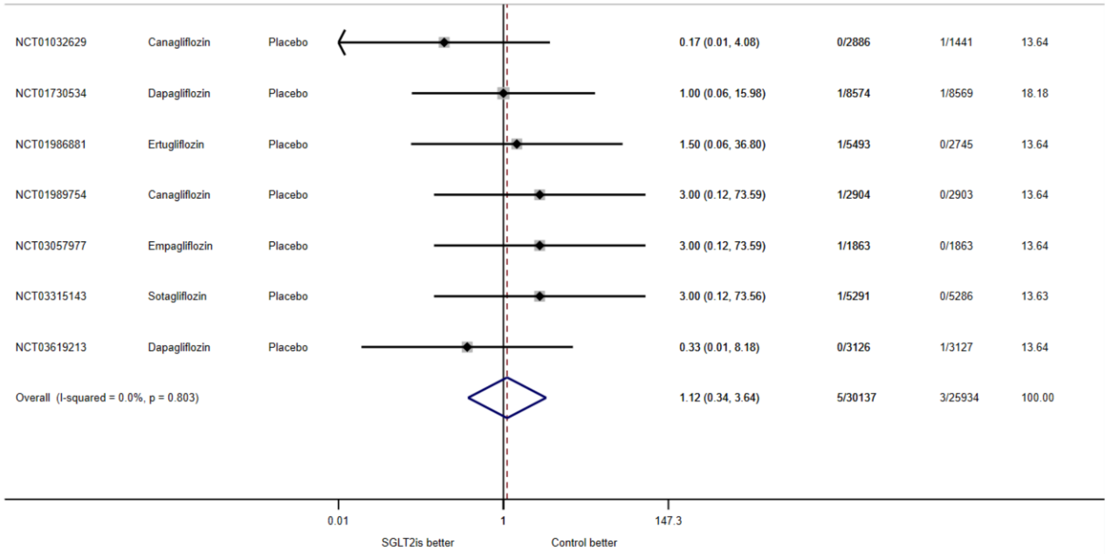

System 12-Injury, Poisoning And Procedural Complications-Figure S63 Meta-analysis of SGLT2is and Postoperative respiratory failure

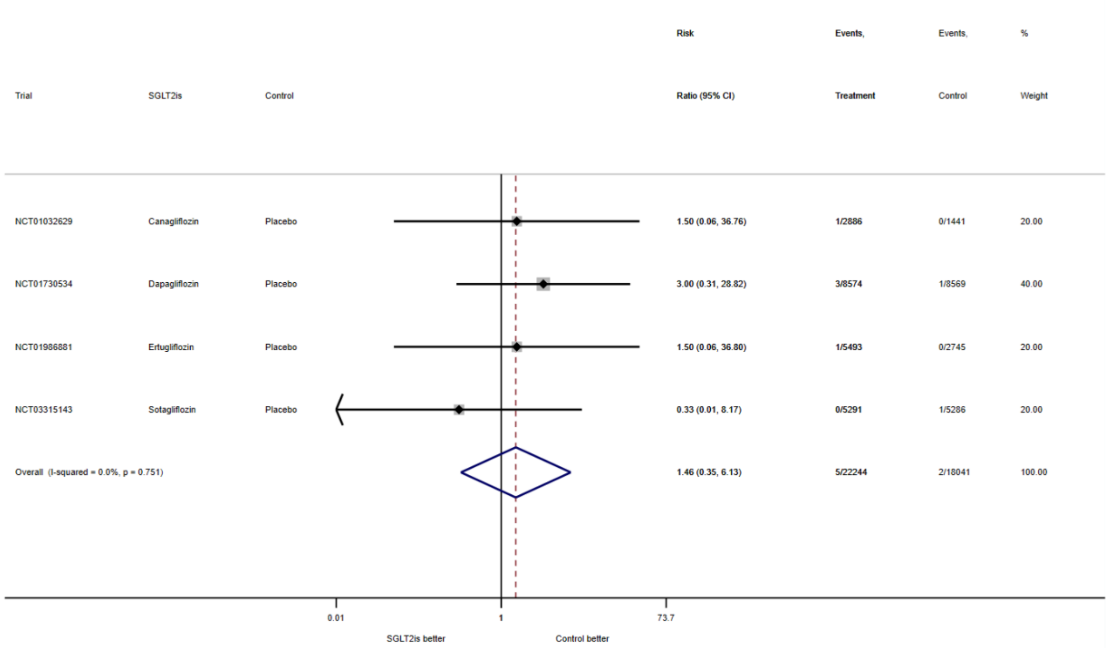

System 12-Injury, Poisoning And Procedural Complications-Figure S64 Meta-analysis of SGLT2is and Postoperative thoracic procedure complication

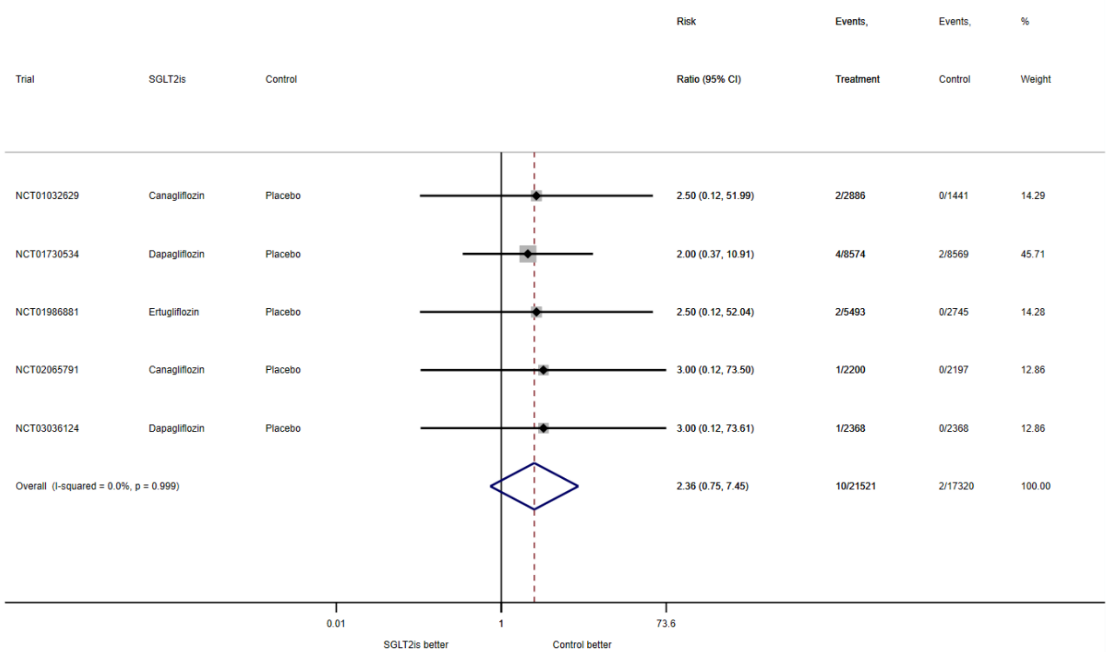

System 12-Injury, Poisoning And Procedural Complications-Figure S65 Meta-analysis of SGLT2is and Postoperative wound complication

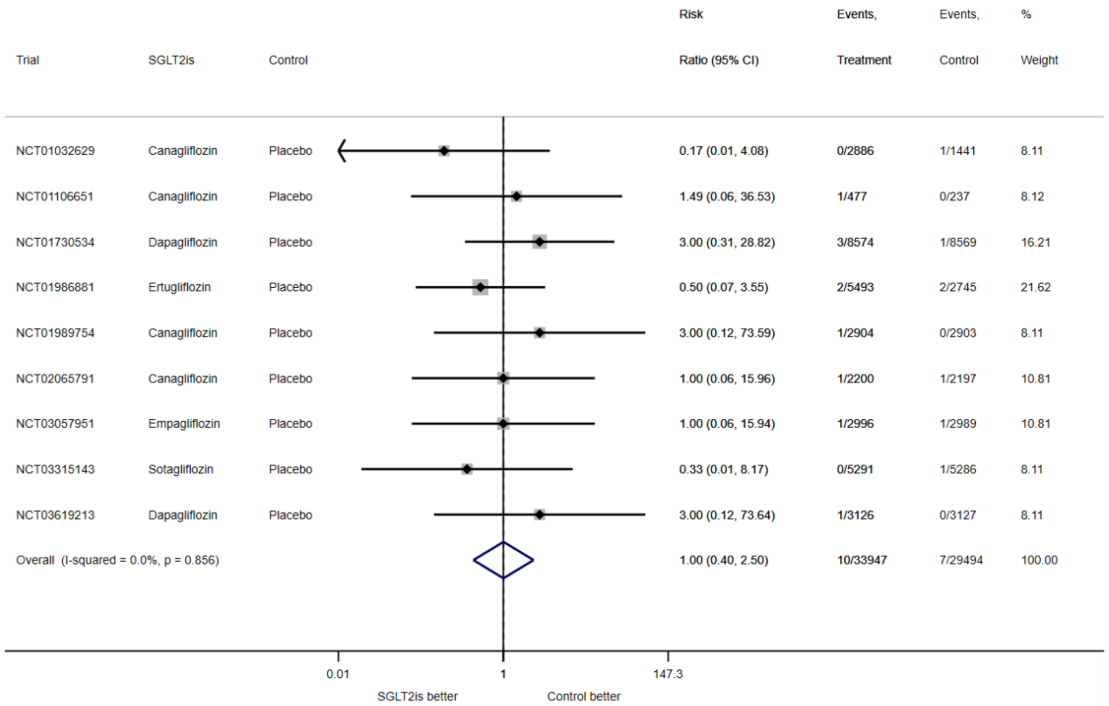

System 12-Injury, Poisoning And Procedural Complications-Figure S66 Meta-analysis of SGLT2is and Procedural pain

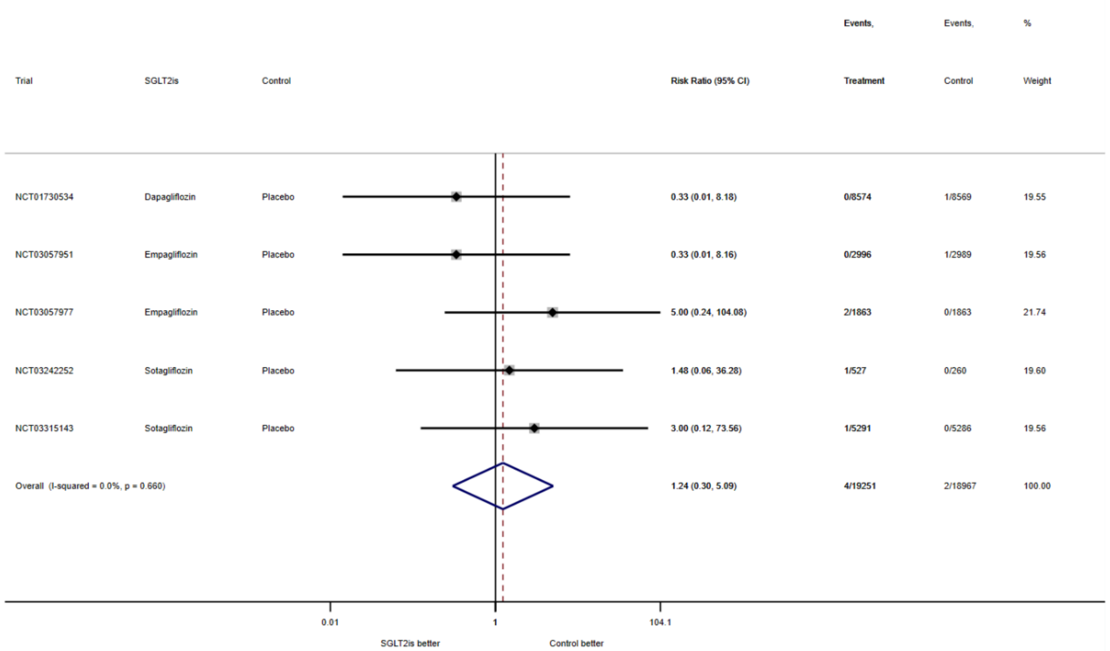

System 12-Injury, Poisoning And Procedural Complications-Figure S67 Meta-analysis of SGLT2is and Procedural shock

Trial	SGLT2is	Control	Risk Ratio (95% CI)	Events, Treatment	Events, Control	% Weight
NCT01032629	Canagliflozin	Placebo	2.50 (0.12, 51.99)	2/2886	0/1441	12.99
NCT01131676	Empagliflozin	Placebo	0.50 (0.03, 7.95)	1/4687	1/2333	15.58
NCT01730534	Dapagliflozin	Placebo	1.00 (0.14, 7.09)	2/8574	2/8569	31.17
NCT01989754	Canagliflozin	Placebo	3.00 (0.12, 73.59)	1/2904	0/2903	11.69
NCT02065791	Canagliflozin	Placebo	4.99 (0.24, 103.94)	2/2200	0/2197	12.99
NCT03594110	Empagliflozin	Placebo	1.00 (0.06, 15.99)	1/3304	1/3305	15.58
Overall (I-squared = 0.0%, p = 0.884)			1.42 (0.47, 4.23)	9/24555	4/20748	100.00

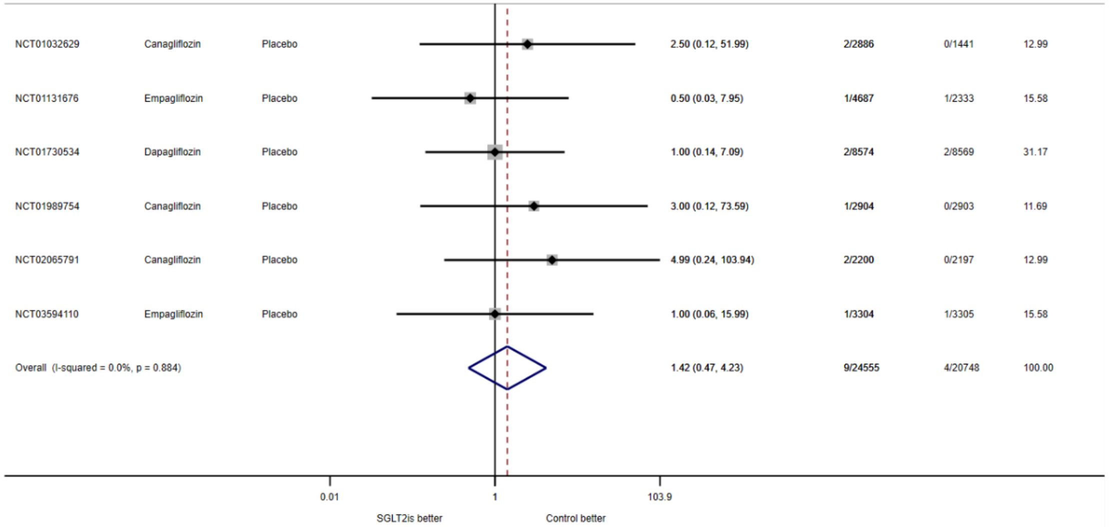

System 12-Injury, Poisoning And Procedural Complications-Figure S68 Meta-analysis of SGLT2is and Pubis fracture

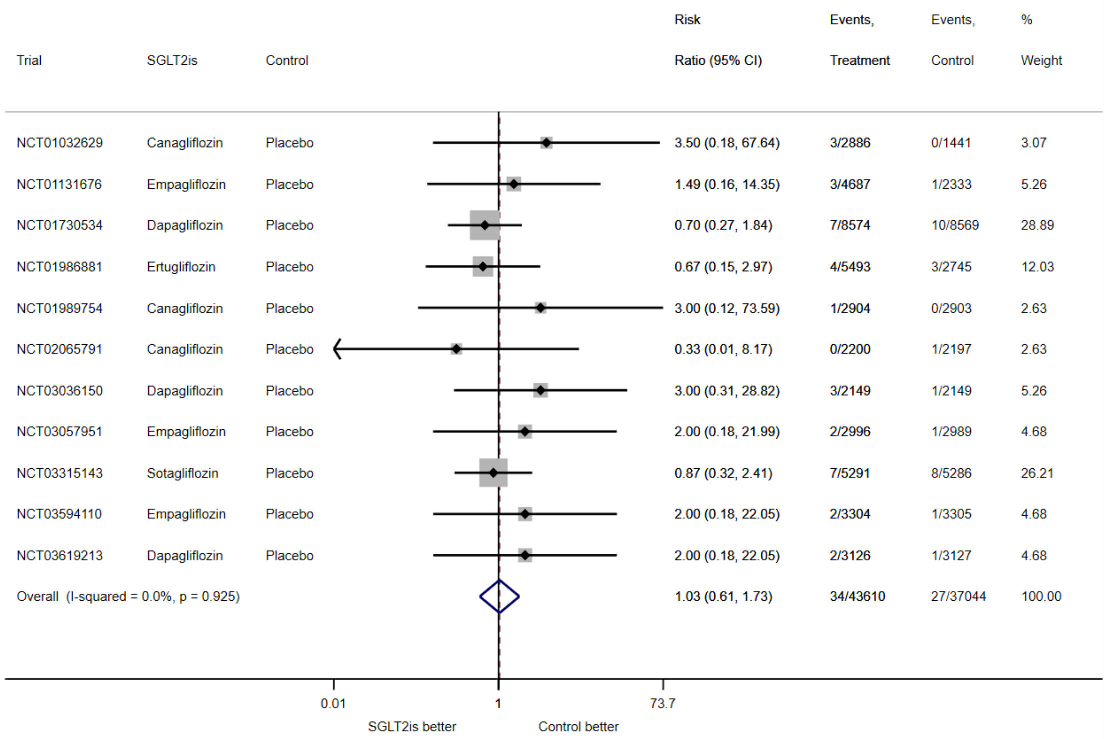

System 12-Injury, Poisoning And Procedural Complications-Figure S69 Meta-analysis of SGLT2is and Radius fracture

Trial	SGLT2is	Control	Risk Ratio (95% CI)	Events, Treatment	Events, Control	% Weight
NCT01032629	Canagliflozin	Placebo	5.49 (0.71, 42.50)	11/2886	1/1441	3.18
NCT01131676	Empagliflozin	Placebo	0.66 (0.23, 1.91)	8/4687	6/2333	11.91
NCT01164501	Empagliflozin	Placebo	0.25 (0.01, 6.21)	0/419	1/319	1.30
NCT01730534	Dapagliflozin	Placebo	0.80 (0.37, 1.71)	12/8574	15/8569	23.14
NCT01986881	Ertugliflozin	Placebo	3.50 (0.43, 28.42)	7/5493	1/2745	3.03
NCT01989754	Canagliflozin	Placebo	1.00 (0.25, 3.99)	4/2904	4/2903	6.94
NCT02065791	Canagliflozin	Placebo	0.50 (0.05, 5.50)	1/2200	2/2197	2.31
NCT03036124	Dapagliflozin	Placebo	1.00 (0.06, 15.98)	1/2368	1/2368	1.73
NCT03036150	Dapagliflozin	Placebo	2.50 (0.49, 12.87)	5/2149	2/2149	4.96
NCT03057951	Empagliflozin	Placebo	2.00 (0.50, 7.97)	6/2996	3/2989	6.94
NCT03057977	Empagliflozin	Placebo	0.20 (0.01, 4.16)	0/1863	2/1863	1.44
NCT03242252	Sotagliflozin	Placebo	0.16 (0.01, 4.03)	0/527	1/260	1.30
NCT03315143	Sotagliflozin	Placebo	1.44 (0.62, 3.37)	13/5291	9/5286	18.47
NCT03521934	Sotagliflozin	Placebo	1.01 (0.06, 16.11)	1/605	1/611	1.74
NCT03594110	Empagliflozin	Placebo	0.80 (0.22, 2.98)	4/3304	5/3305	7.71
NCT03619213	Dapagliflozin	Placebo	0.33 (0.03, 3.20)	1/3126	3/3127	2.60
NCT04350593	Dapagliflozin	Placebo	0.33 (0.01, 8.21)	0/613	1/616	1.30
Overall (I-squared = 0.0%, p = 0.701)			1.01 (0.70, 1.46)	74/50005	58/43081	100.00

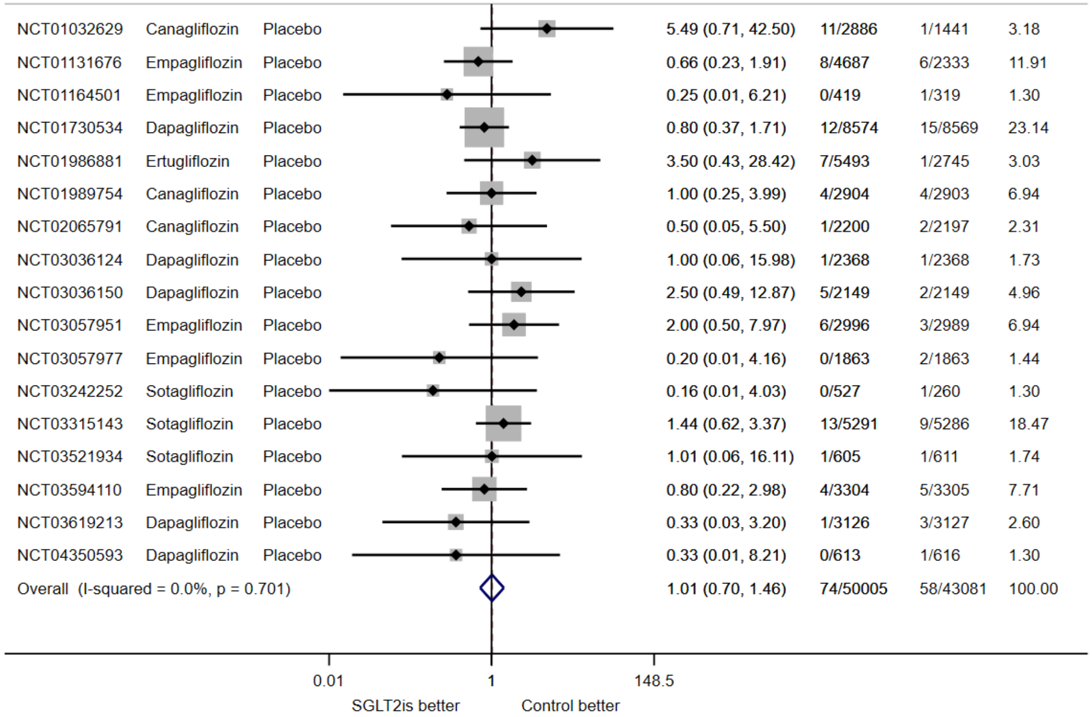

System 12-Injury, Poisoning And Procedural Complications-Figure S70 Meta-analysis of SGLT2is and Rib fracture

Trial	SGLT2is	Control	Risk Ratio (95% CI)	Events, Treatment	Events, Control	% Weight
NCT01032629	Canagliflozin	Placebo	0.75 (0.13, 4.48)	3/2886	2/1441	7.49
NCT01042977	Dapagliflozin	Placebo	0.33 (0.01, 8.16)	0/482	1/482	2.34
NCT01131676	Empagliflozin	Placebo	1.24 (0.48, 3.20)	15/4687	6/2333	26.81
NCT01730534	Dapagliflozin	Placebo	1.14 (0.41, 3.15)	8/8574	7/8569	23.31
NCT01986881	Ertugliflozin	Placebo	2.50 (0.12, 52.04)	2/5493	0/2745	2.60
NCT01989754	Canagliflozin	Placebo	5.00 (0.24, 104.07)	2/2904	0/2903	2.60
NCT02065791	Canagliflozin	Placebo	3.00 (0.12, 73.50)	1/2200	0/2197	2.34
NCT03036124	Dapagliflozin	Placebo	1.00 (0.06, 15.98)	1/2368	1/2368	3.12
NCT03036150	Dapagliflozin	Placebo	0.33 (0.01, 8.18)	0/2149	1/2149	2.34
NCT03057951	Empagliflozin	Placebo	2.99 (0.31, 28.76)	3/2996	1/2989	4.68
NCT03057977	Empagliflozin	Placebo	2.00 (0.37, 10.91)	4/1863	2/1863	8.33
NCT03315143	Sotagliflozin	Placebo	0.20 (0.02, 1.71)	1/5291	5/5286	5.20
NCT03594110	Empagliflozin	Placebo	2.00 (0.18, 22.05)	2/3304	1/3305	4.16
NCT03619213	Dapagliflozin	Placebo	3.00 (0.31, 28.83)	3/3126	1/3127	4.68
Overall (I-squared = 0.0%, p = 0.883)			1.24 (0.76, 2.02)	45/48323	28/41757	100.00

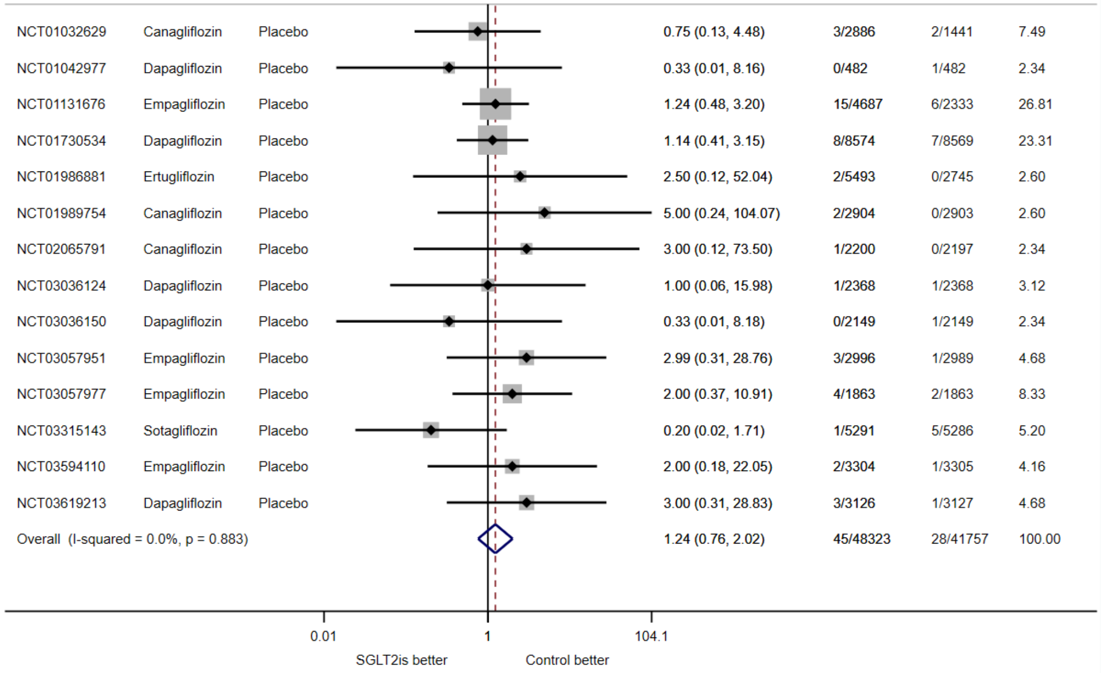

System 12-Injury, Poisoning And Procedural Complications-Figure S71 Meta-analysis of SGLT2is and Road traffic accident

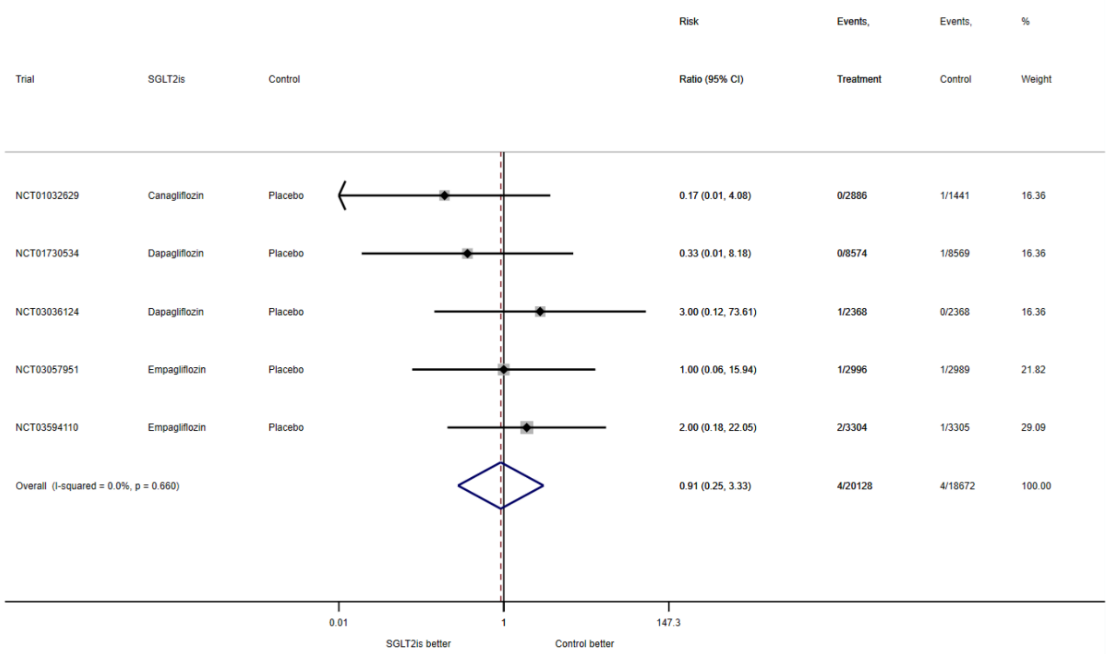

System 12-Injury, Poisoning And Procedural Complications-Figure S72 Meta-analysis of SGLT2is and Skin injury

Trial	SGLT2is	Control	Risk Ratio (95% CI)	Events, Treatment	Events, Control	% Weight
NCT01986881	Ertugliflozin	Placebo	0.50 (0.03, 7.99)	1/5493	1/2745	17.65
NCT03036150	Dapagliflozin	Placebo	1.00 (0.06, 15.98)	1/2149	1/2149	17.65
NCT03057951	Empagliflozin	Placebo	0.50 (0.05, 5.50)	1/2996	2/2989	23.53
NCT03057977	Empagliflozin	Placebo	5.00 (0.24, 104.08)	2/1863	0/1863	14.71
NCT03315143	Sotagliflozin	Placebo	0.33 (0.01, 8.17)	0/5291	1/5286	13.23
NCT03619213	Dapagliflozin	Placebo	3.00 (0.12, 73.64)	1/3126	0/3127	13.23
Overall (I-squared = 0.0%, p = 0.770)			0.95 (0.30, 3.05)	6/20918	5/18159	100.00

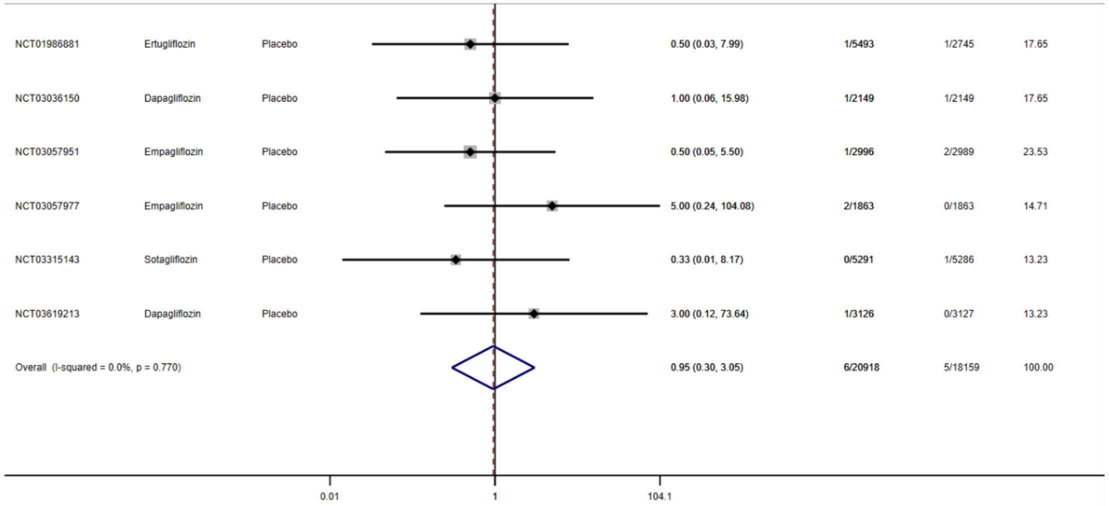

System 12-Injury, Poisoning And Procedural Complications-Figure S73 Meta-analysis of SGLT2is and Skin laceration

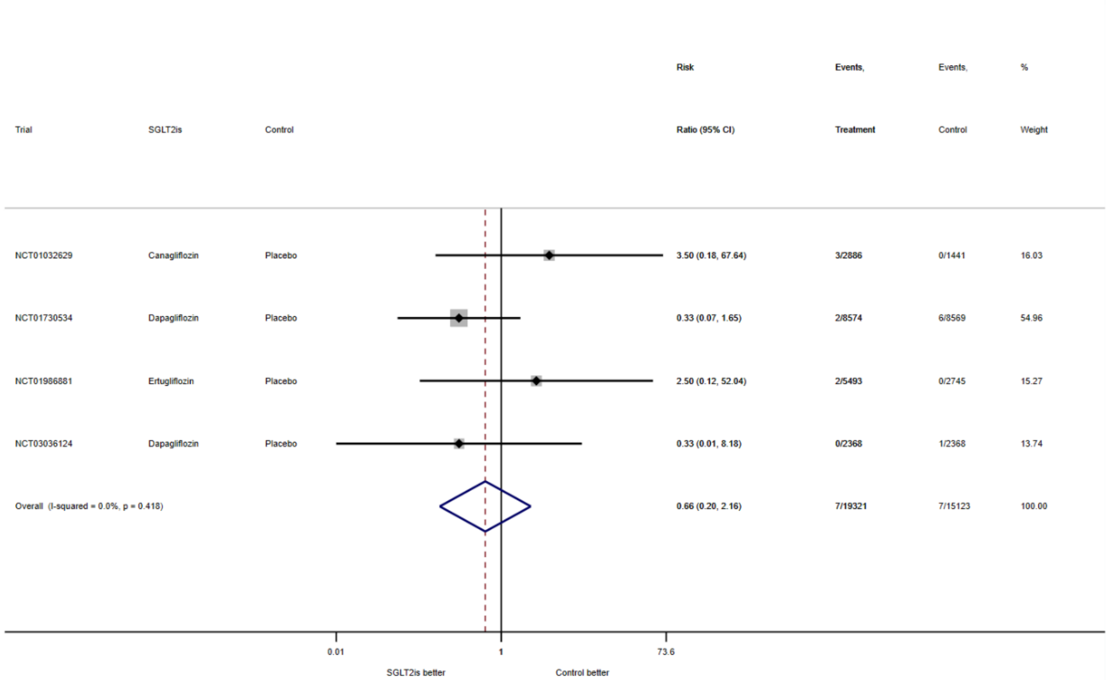

System 12-Injury, Poisoning And Procedural Complications-Figure S74 Meta-analysis of SGLT2is and Skull fracture

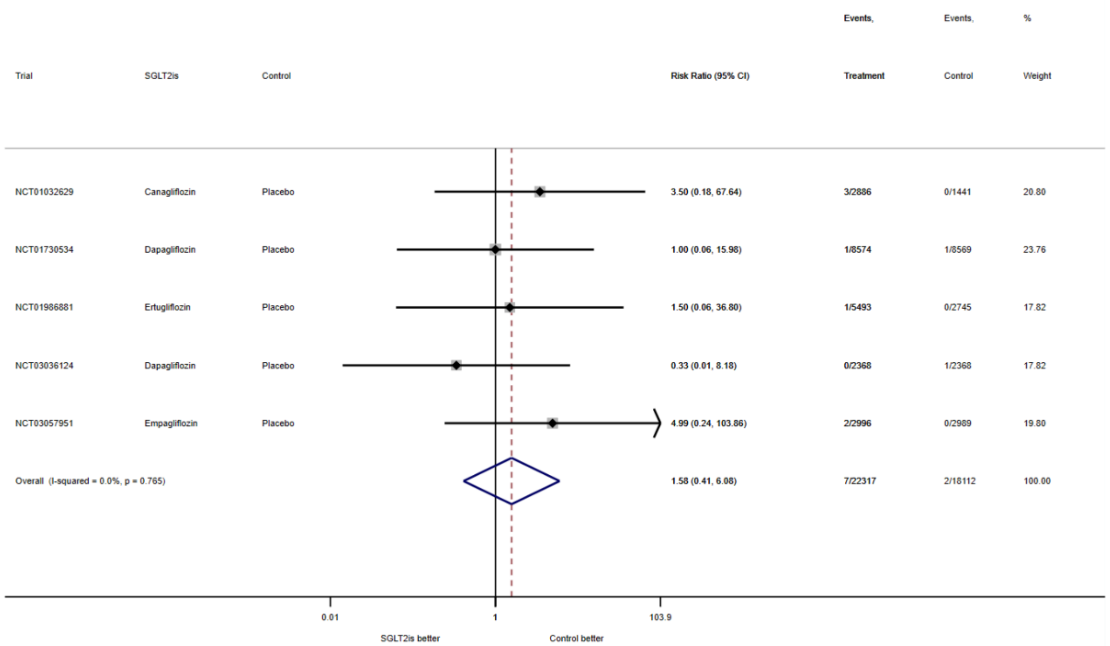

System 12-Injury, Poisoning And Procedural Complications-Figure S75 Meta-analysis of SGLT2is and Skull fractured base

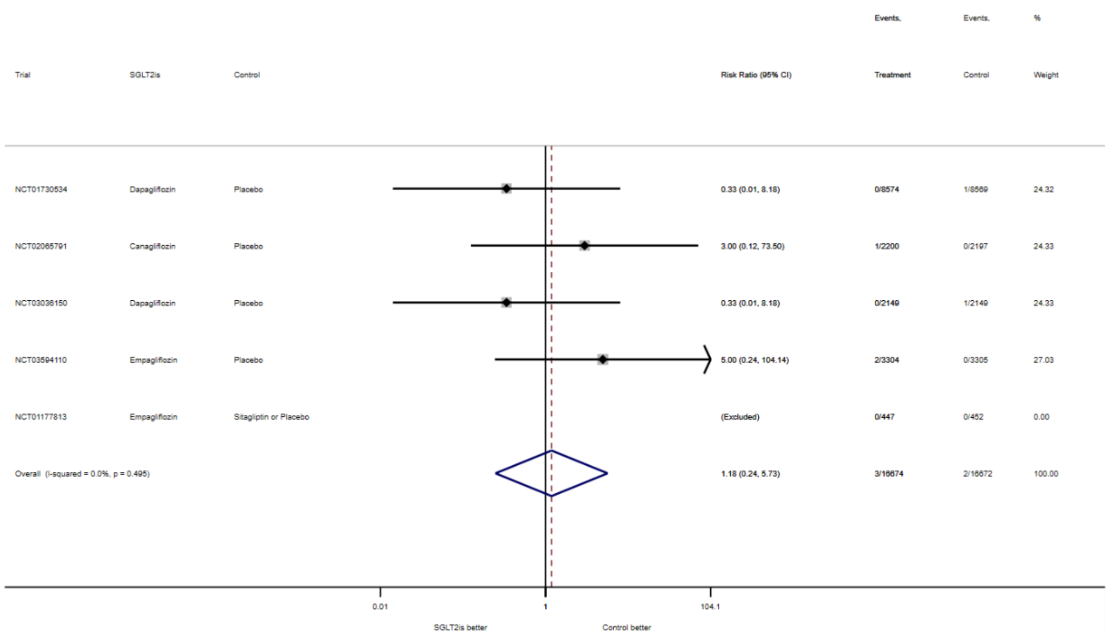

System 12-Injury, Poisoning And Procedural Complications-Figure S76 Meta-analysis of SGLT2is and Soft tissue injury

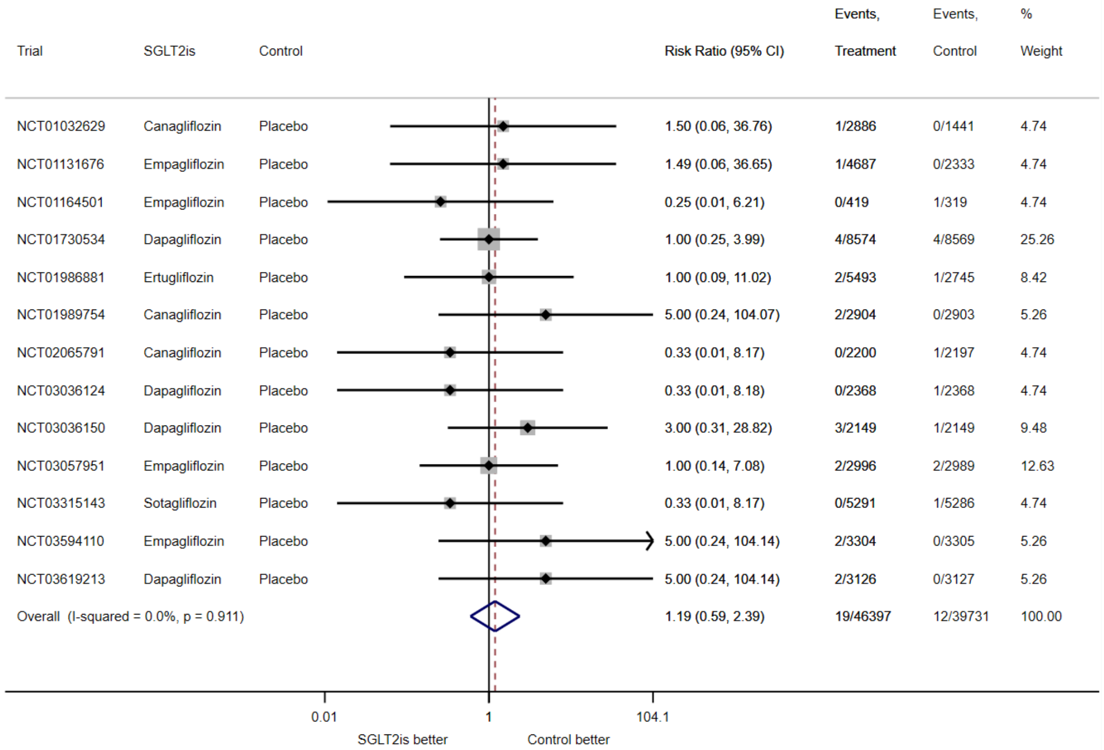

System 12-Injury, Poisoning And Procedural Complications-Figure S77 Meta-analysis of SGLT2is and Spinal fracture

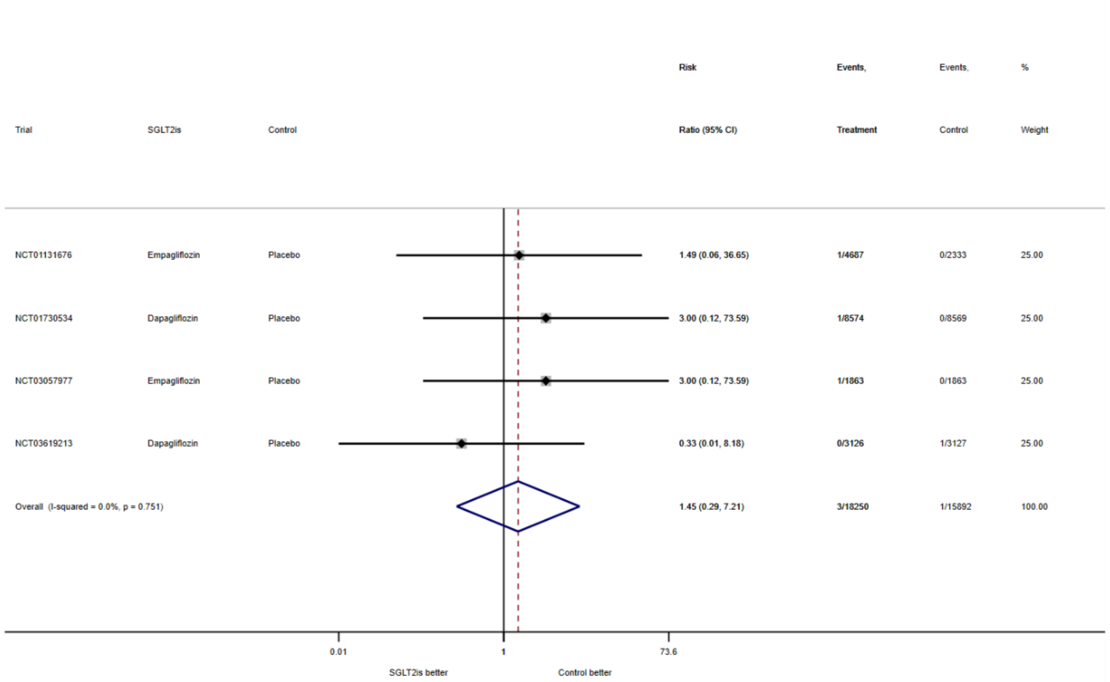

System 12-Injury, Poisoning And Procedural Complications-Figure S78 Meta-analysis of SGLT2is and Splenic rupture

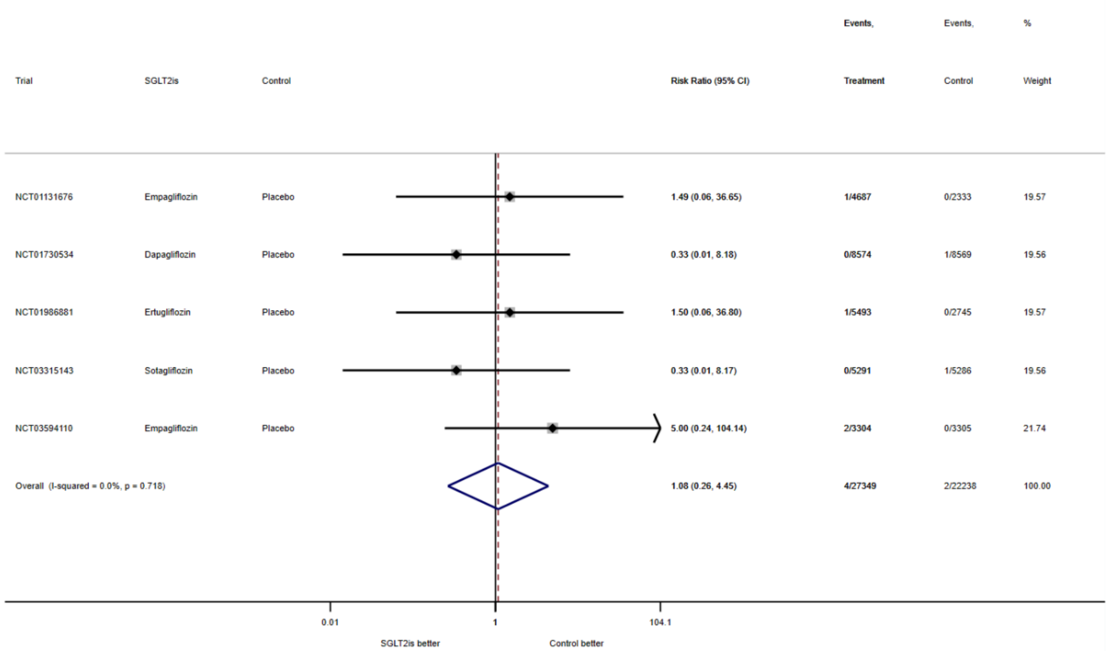

System 12-Injury, Poisoning And Procedural Complications-Figure S79 Meta-analysis of SGLT2is and Stab wound

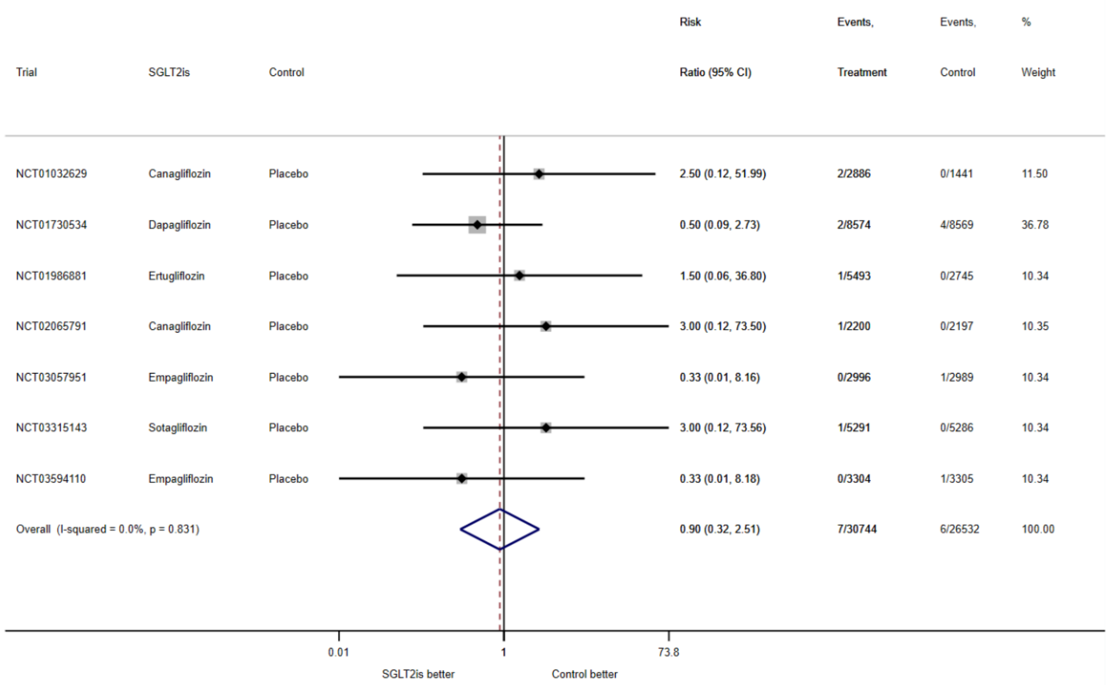

System 12-Injury, Poisoning And Procedural Complications-Figure S80 Meta-analysis of SGLT2is and Sternal fracture

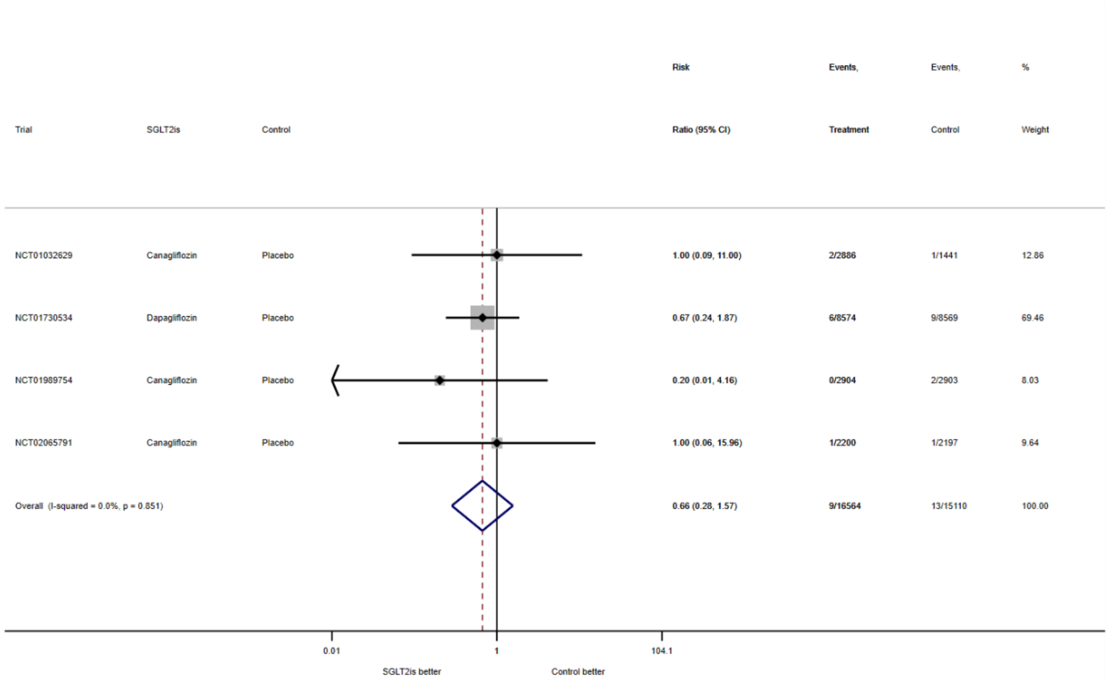

System 12-Injury, Poisoning And Procedural Complications-Figure S81 Meta-analysis of SGLT2is and Subarachnoid haemorrhage

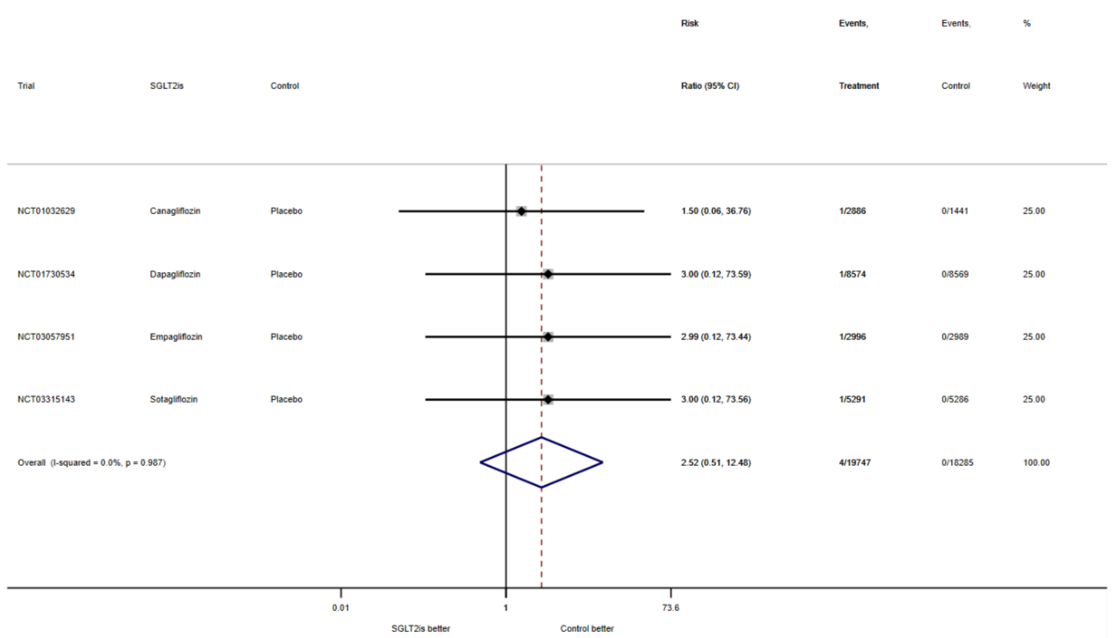

System 12-Injury, Poisoning And Procedural Complications-Figure S82 Meta-analysis of SGLT2is and Subcutaneous haematoma

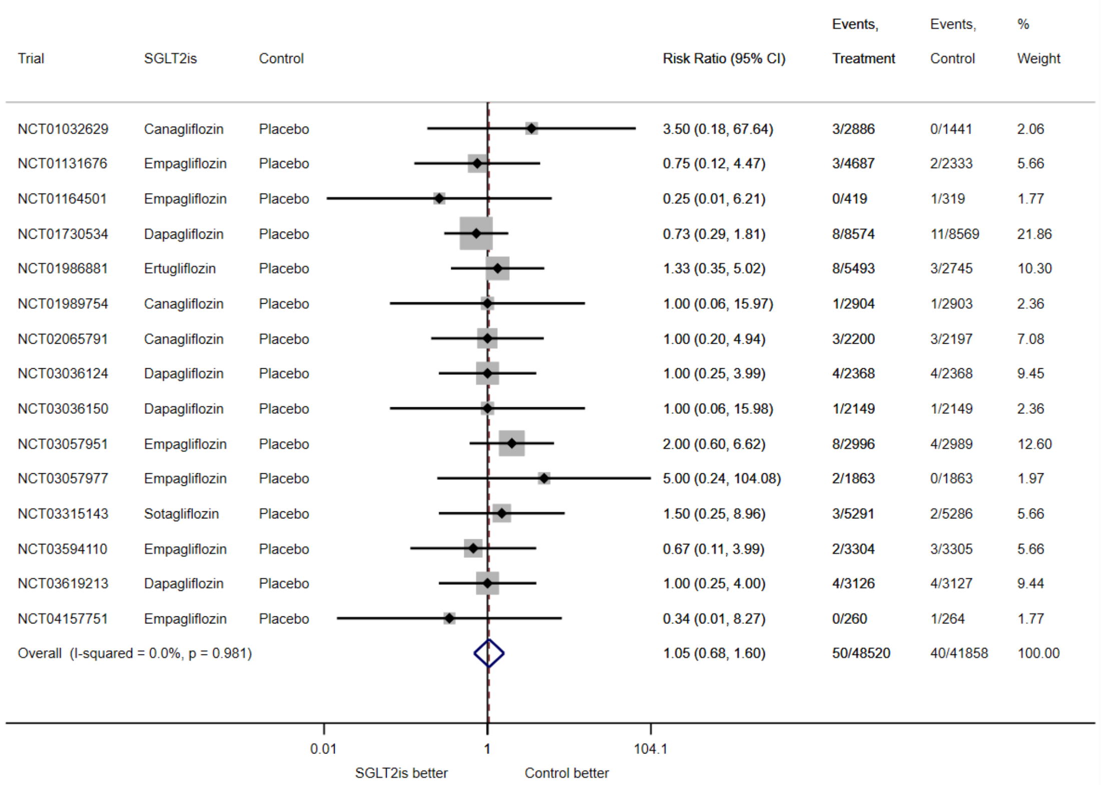

System 12-Injury, Poisoning And Procedural Complications-Figure S83 Meta-analysis of SGLT2is and Subdural haematoma

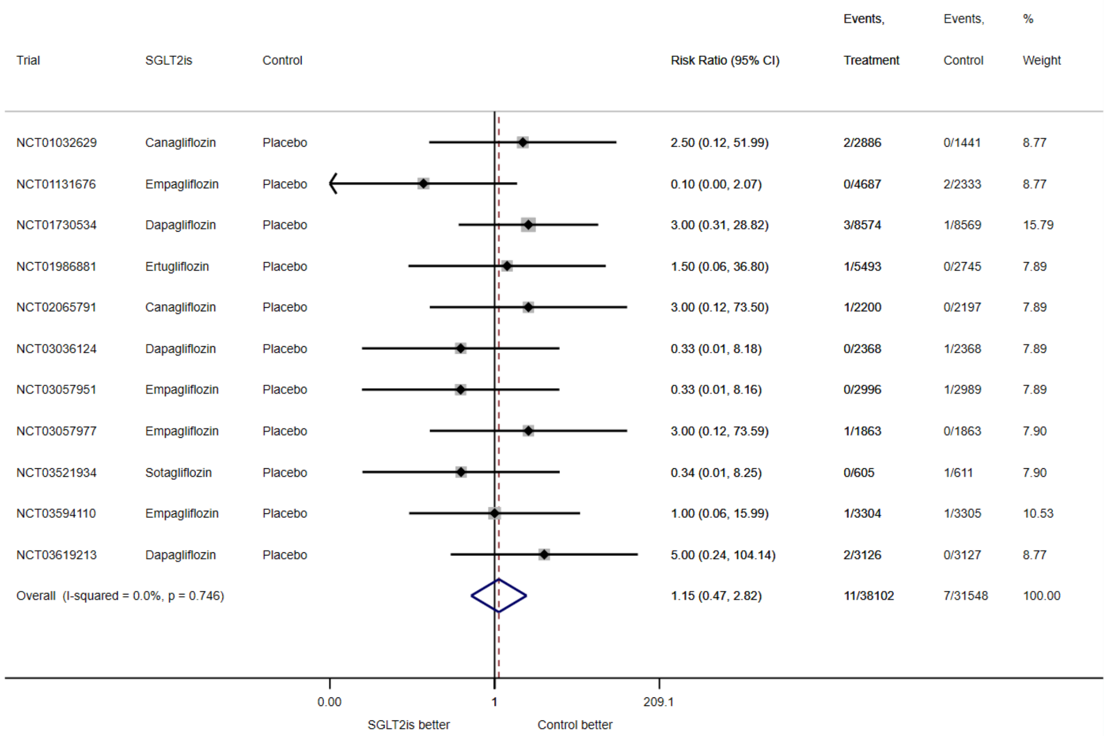

System 12-Injury, Poisoning And Procedural Complications-Figure S84 Meta-analysis of SGLT2is and Subdural haemorrhage

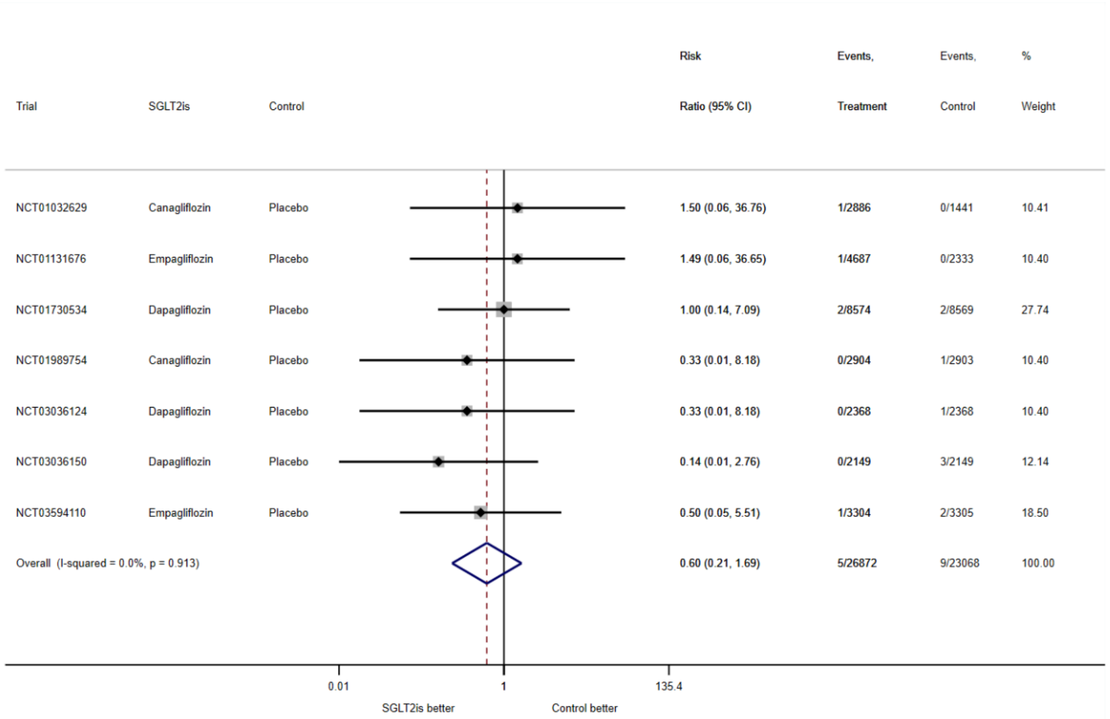

System 12-Injury, Poisoning And Procedural Complications-Figure S85 Meta-analysis of SGLT2is and Tendon injury

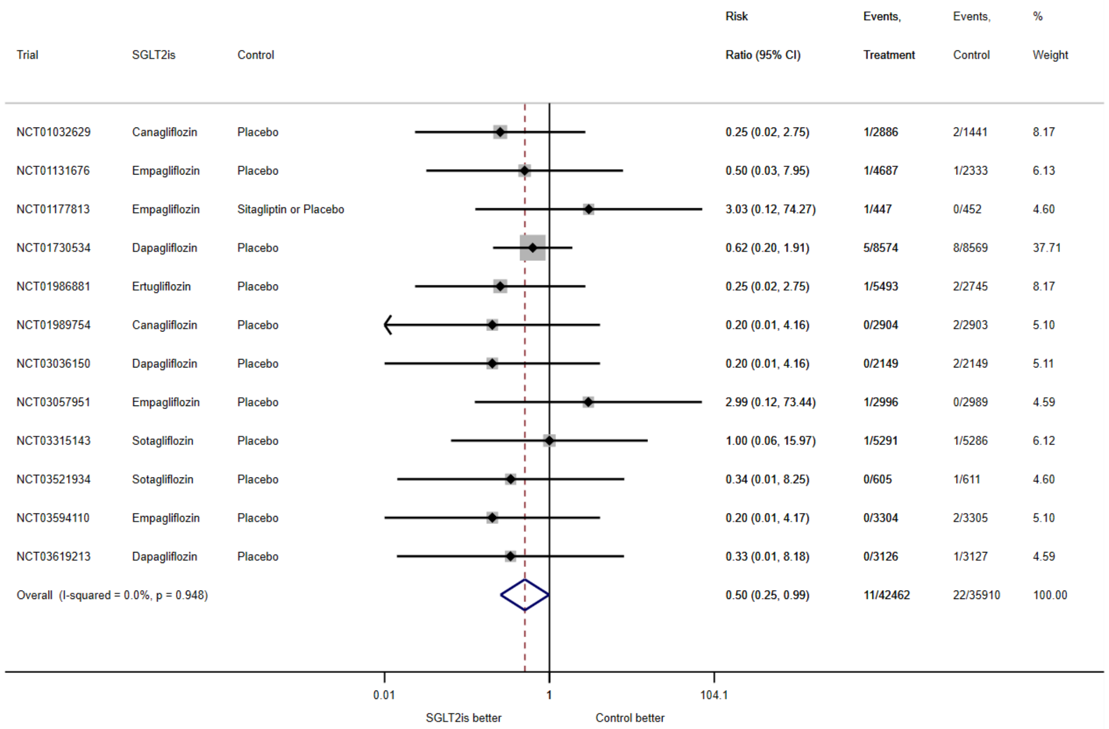

System 12-Injury, Poisoning And Procedural Complications-Figure S86 Meta-analysis of SGLT2is and Tendon rupture

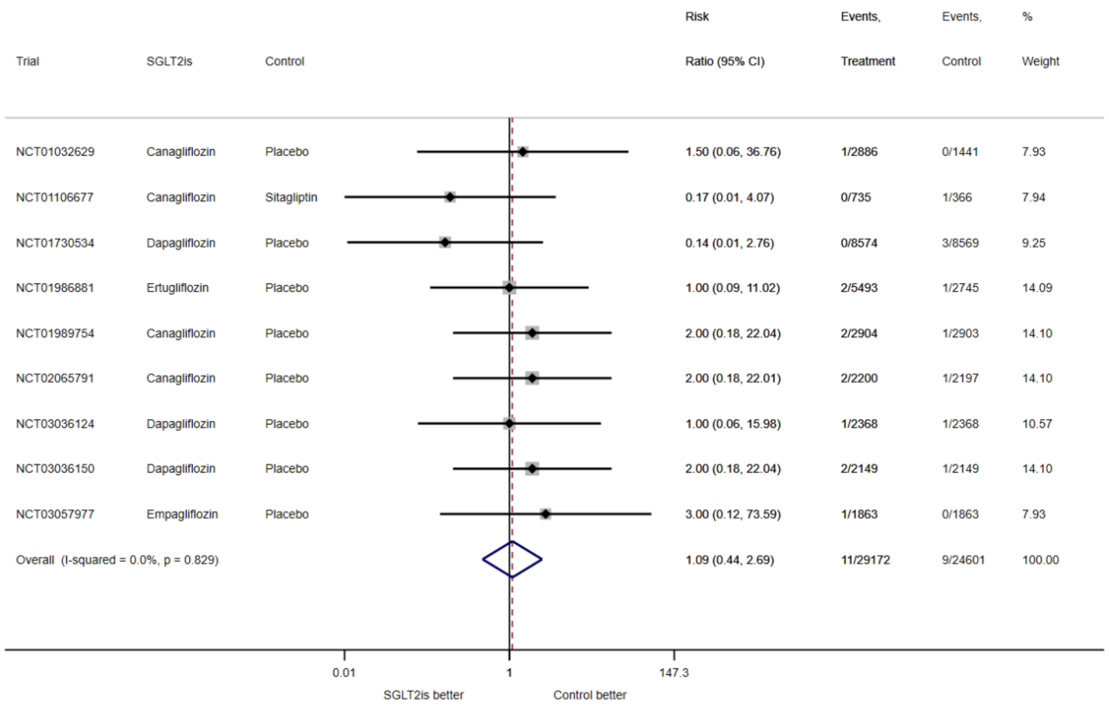

System 12-Injury, Poisoning And Procedural Complications-Figure S87 Meta-analysis of SGLT2is and Thermal burn

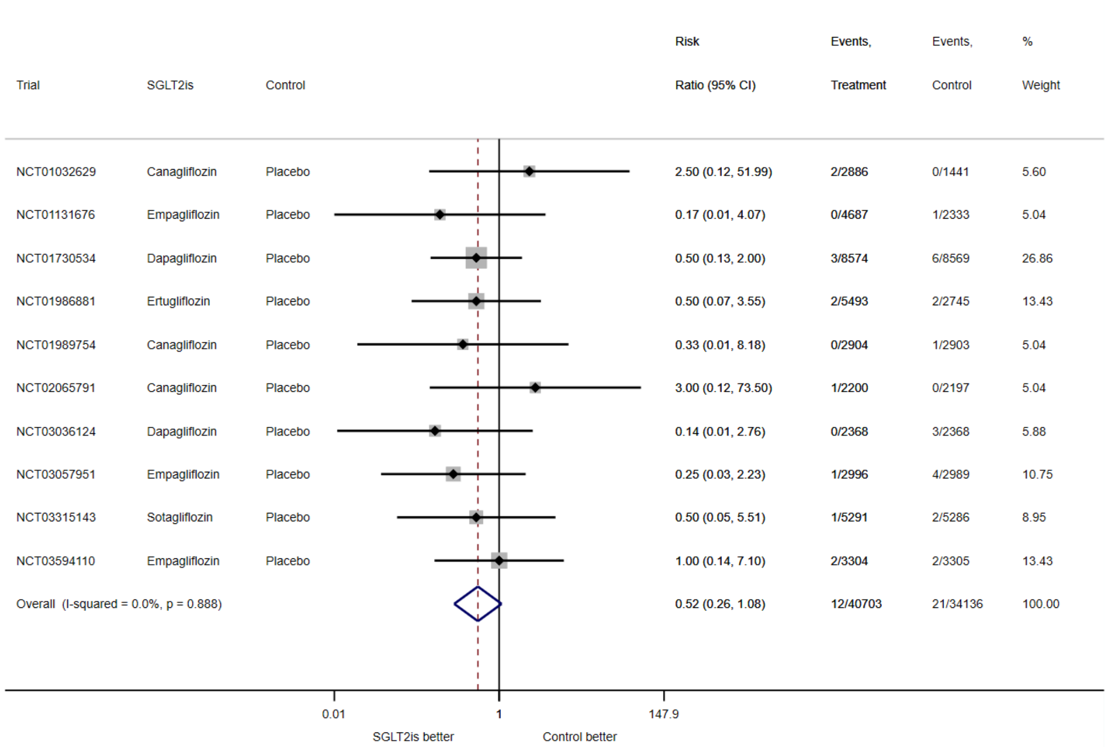

System 12-Injury, Poisoning And Procedural Complications-Figure S88 Meta-analysis of SGLT2is and Thoracic vertebral fracture

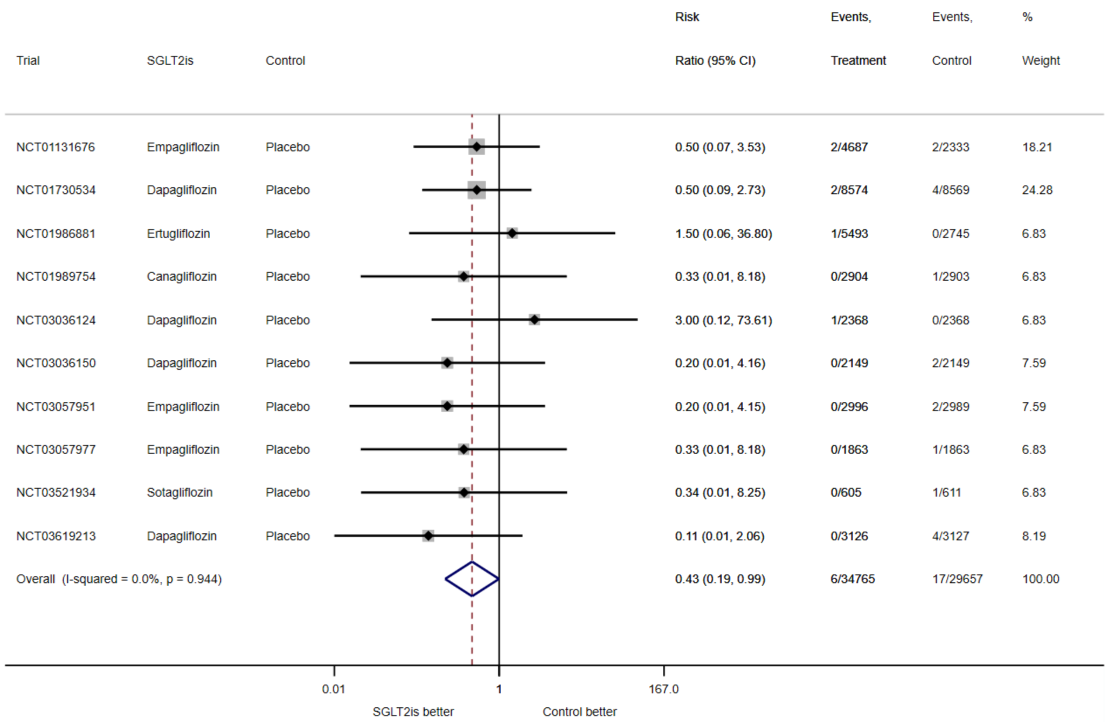

System 12-Injury, Poisoning And Procedural Complications-Figure S89 Meta-analysis of SGLT2is and Toxicity to various agents

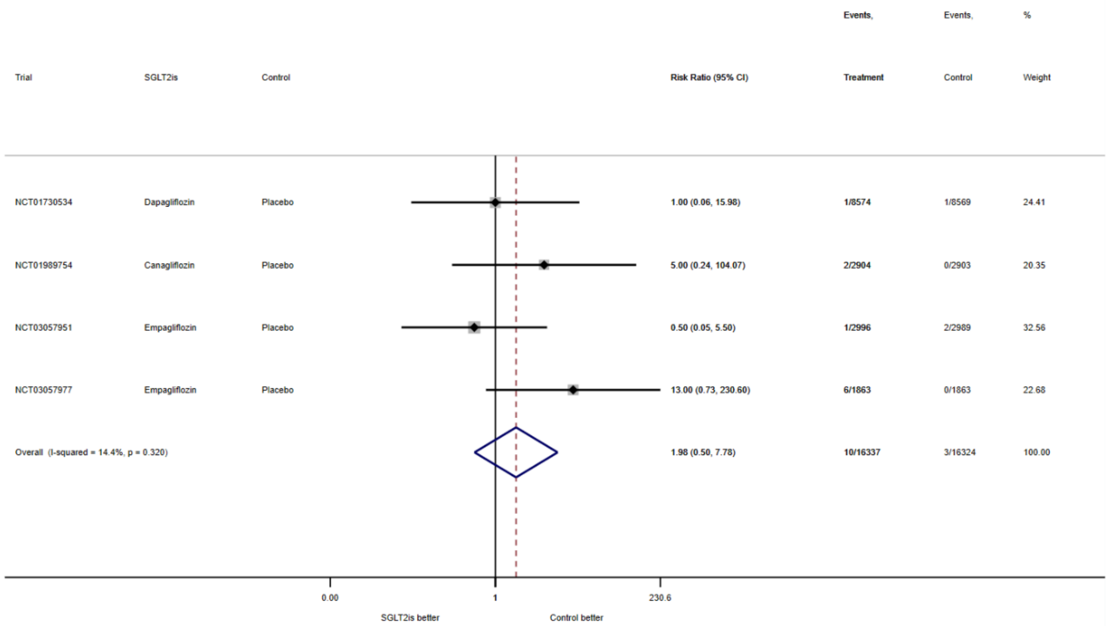

System 12-Injury, Poisoning And Procedural Complications-Figure S90 Meta-analysis of SGLT2is and Traumatic fracture

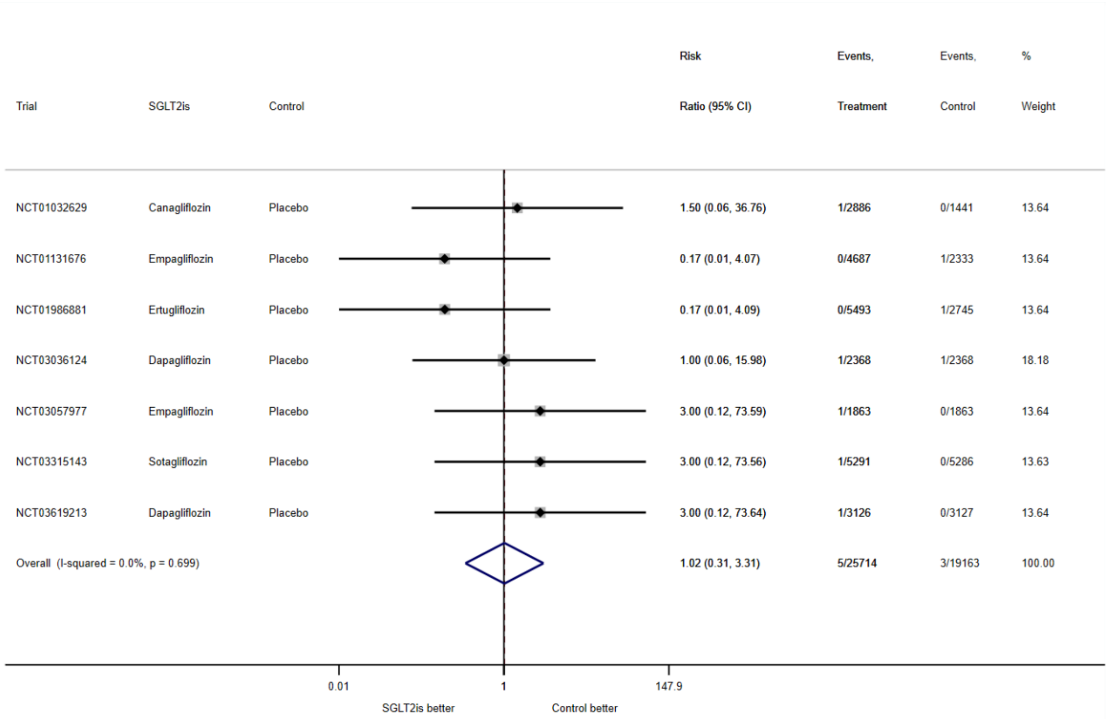

System 12-Injury, Poisoning And Procedural Complications-Figure S91 Meta-analysis of SGLT2is and Traumatic haematoma

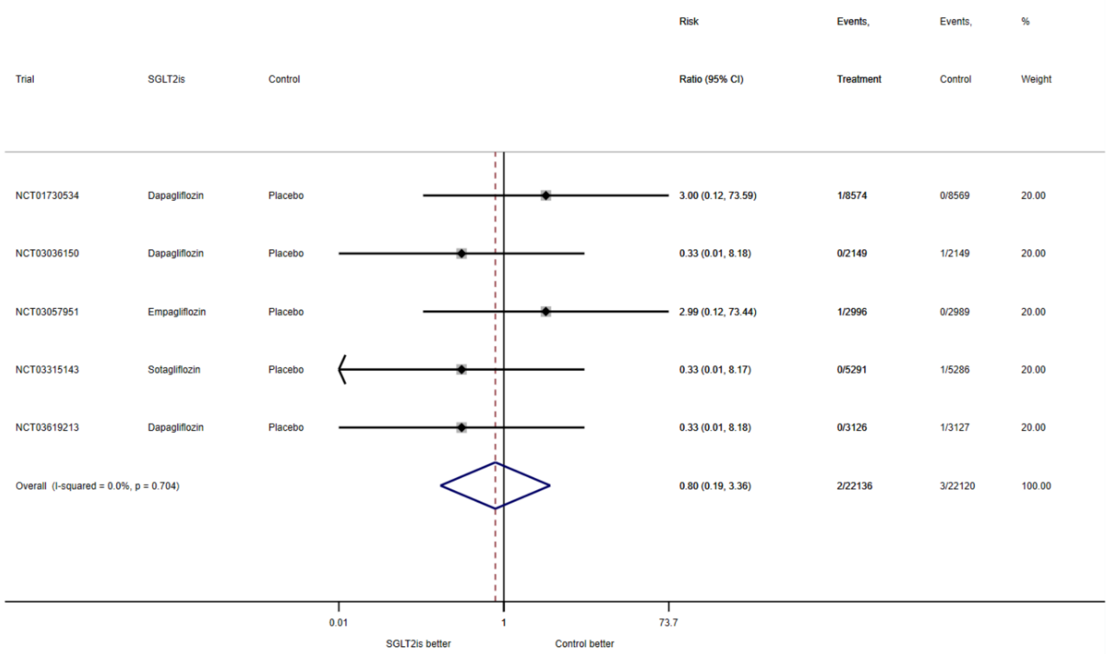

System 12-Injury, Poisoning And Procedural Complications-Figure S92 Meta-analysis of SGLT2is and Traumatic haemothorax

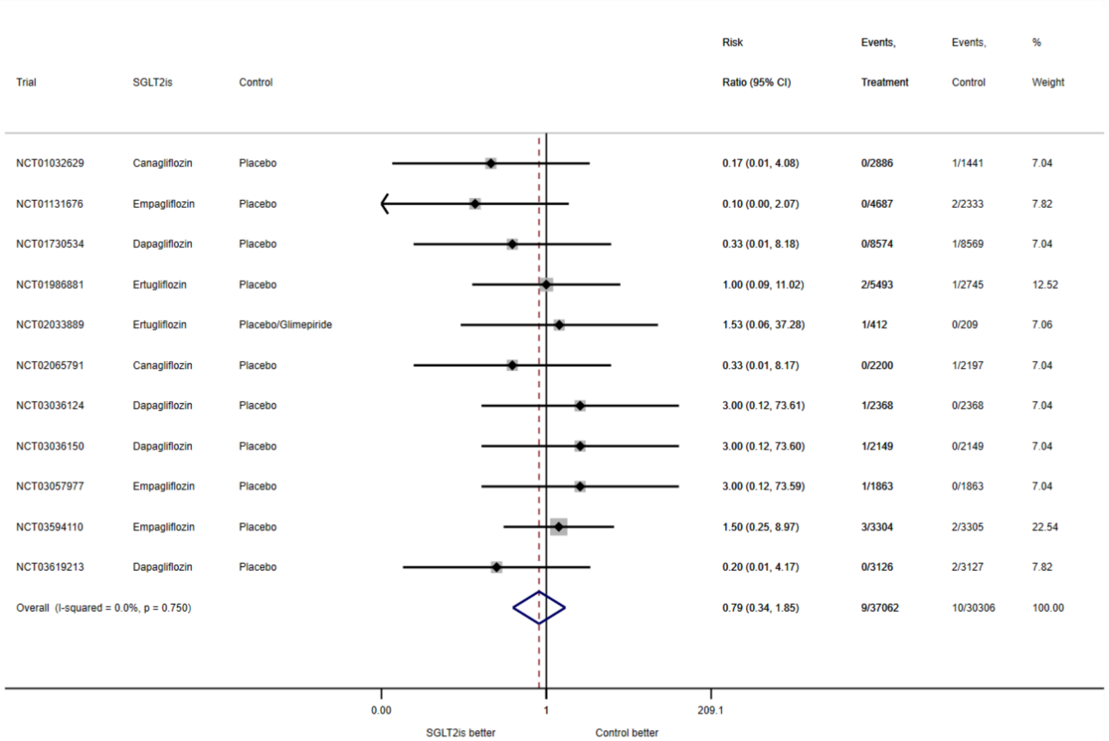

System 12-Injury, Poisoning And Procedural Complications-Figure S93 Meta-analysis of SGLT2is and Traumatic intracranial haemorrhage

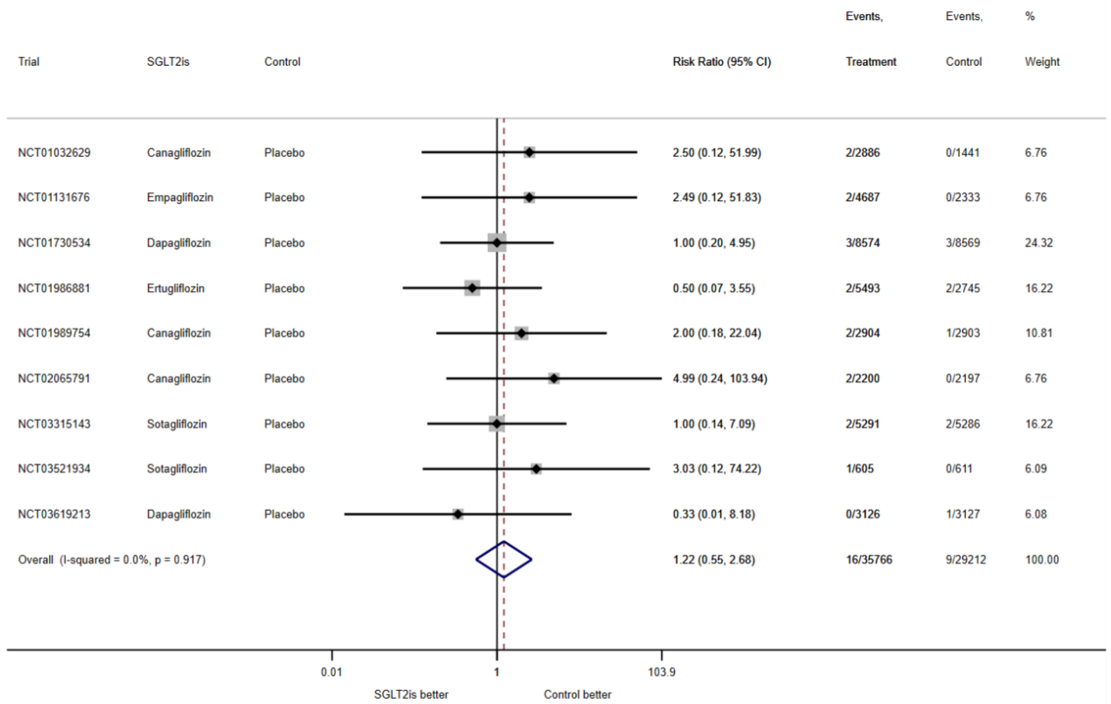

System 12-Injury, Poisoning And Procedural Complications-Figure S94 Meta-analysis of SGLT2is and Ulna fracture

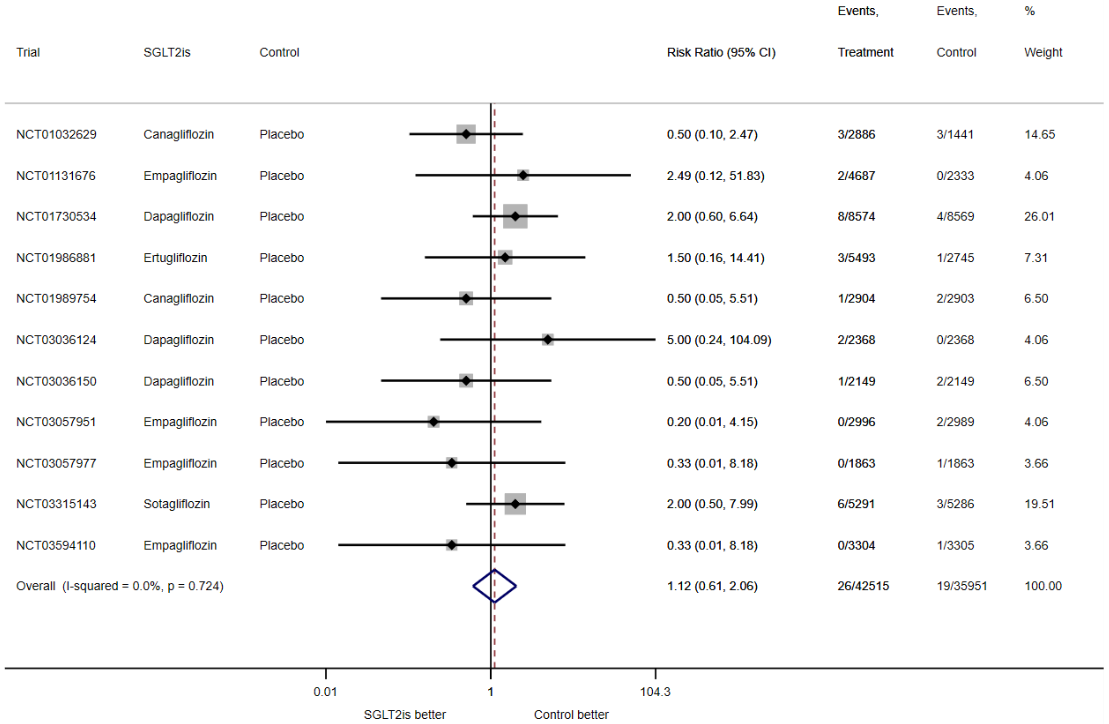

System 12-Injury, Poisoning And Procedural Complications-Figure S95 Meta-analysis of SGLT2is and Upper limb fracture

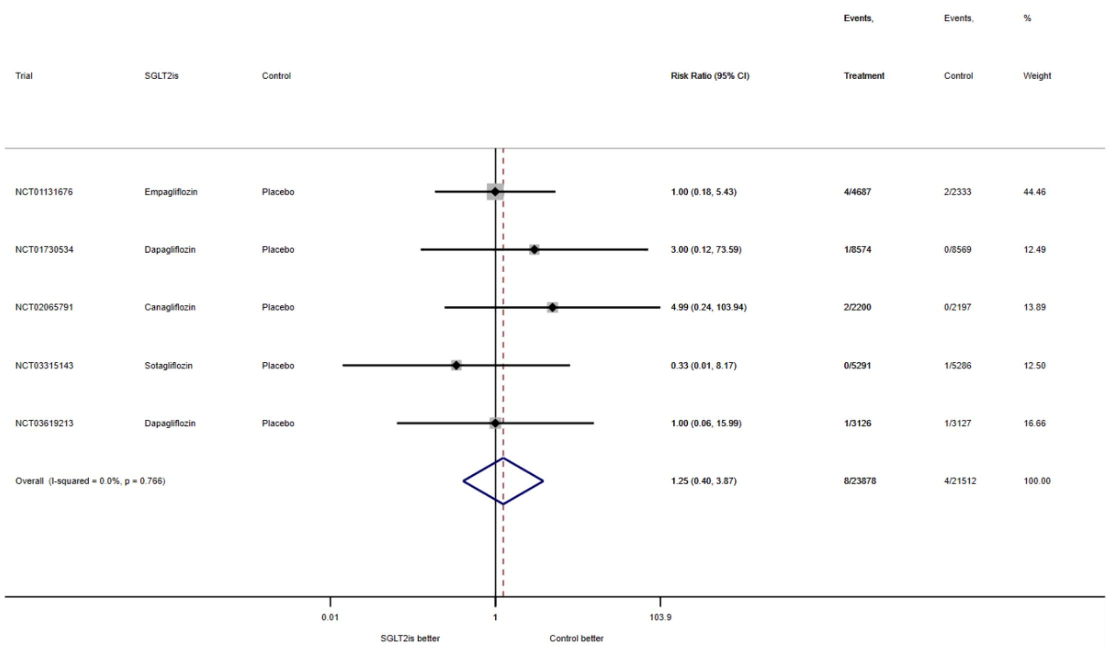

System 12-Injury, Poisoning And Procedural Complications-Figure S96 Meta-analysis of SGLT2is and Vascular graft occlusion

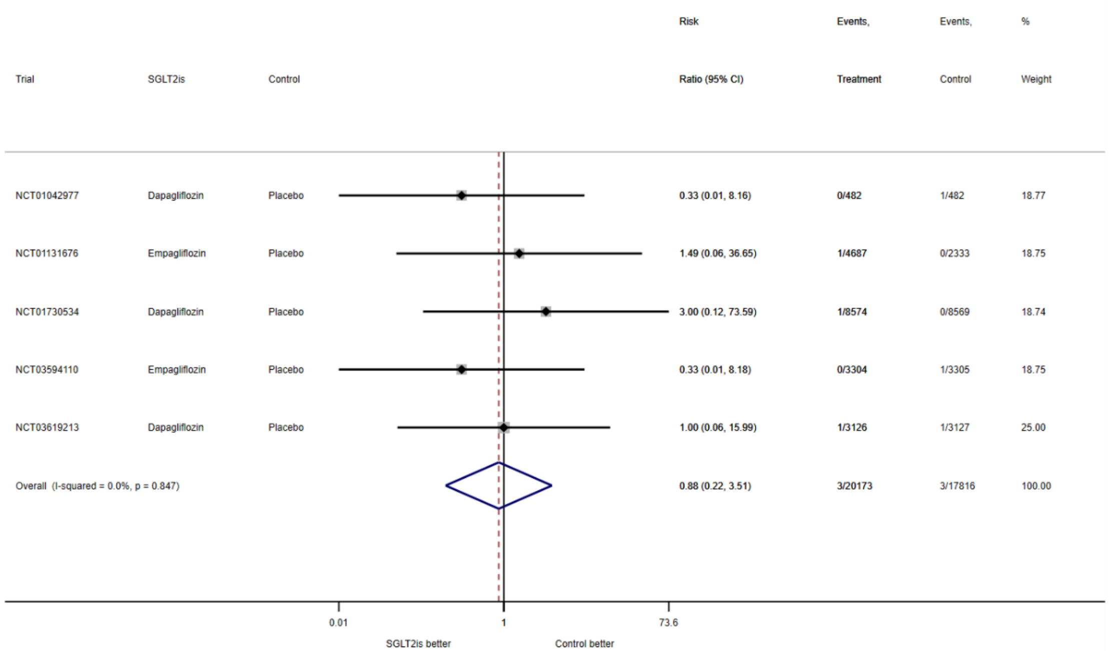

System 12-Injury, Poisoning And Procedural Complications-Figure S97 Meta-analysis of SGLT2is and Vascular graft thrombosis

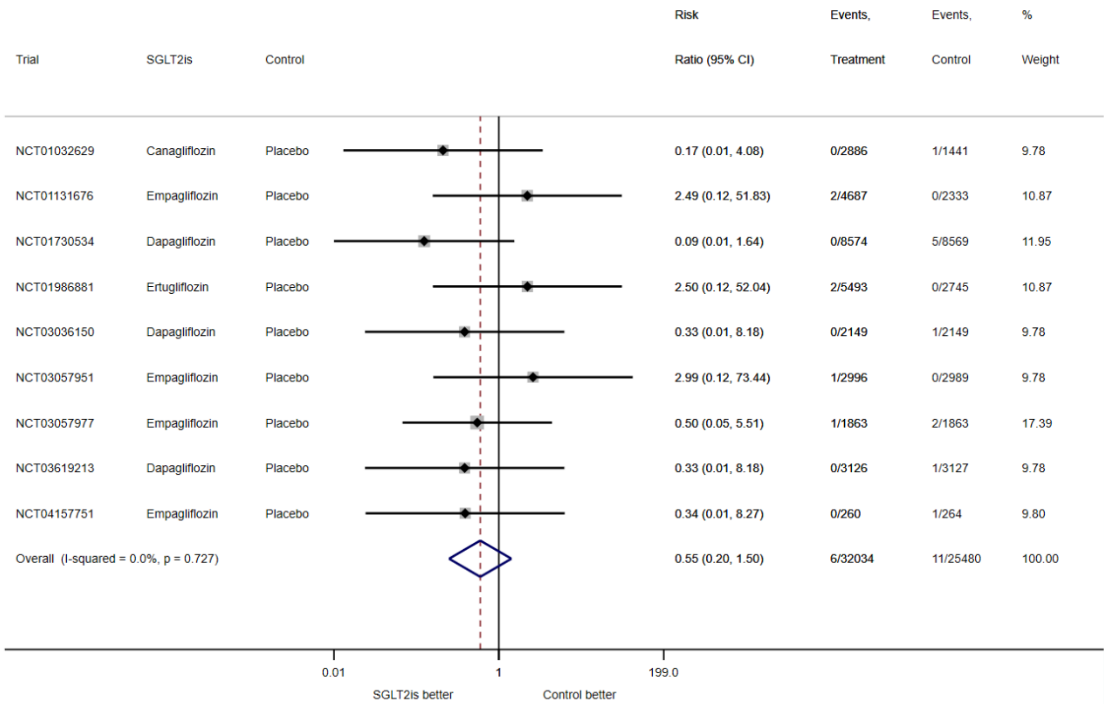

System 12-Injury, Poisoning And Procedural Complications-Figure S98 Meta-analysis of SGLT2is and Vascular pseudoaneurysm

Trial	SGLT2is	Control	Risk Ratio (95% CI)	Events, Treatment	Events, Control	% Weight
NCT01032629	Canagliflozin	Placebo	1.00 (0.18, 5.45)	4/2886	2/1441	23.54
NCT01131676	Empagliflozin	Placebo	1.00 (0.18, 5.43)	4/4687	2/2333	23.53
NCT01164501	Empagliflozin	Placebo	2.29 (0.09, 55.92)	1/419	0/319	6.63
NCT01730534	Dapagliflozin	Placebo	0.33 (0.01, 8.18)	0/8574	1/8569	6.61
NCT01986881	Ertugliflozin	Placebo	0.25 (0.02, 2.75)	1/5493	2/2745	11.76
NCT01989754	Canagliflozin	Placebo	0.33 (0.01, 8.18)	0/2904	1/2903	6.61
NCT02065791	Canagliflozin	Placebo	0.09 (0.01, 1.64)	0/2200	5/2197	8.09
NCT03315143	Sotagliflozin	Placebo	3.00 (0.12, 73.56)	1/5291	0/5286	6.61
NCT03594110	Empagliflozin	Placebo	3.00 (0.12, 73.64)	1/3304	0/3305	6.61
Overall (I-squared = 0.0%, p = 0.707)			0.74 (0.32, 1.68)	12/35758	13/29098	100.00

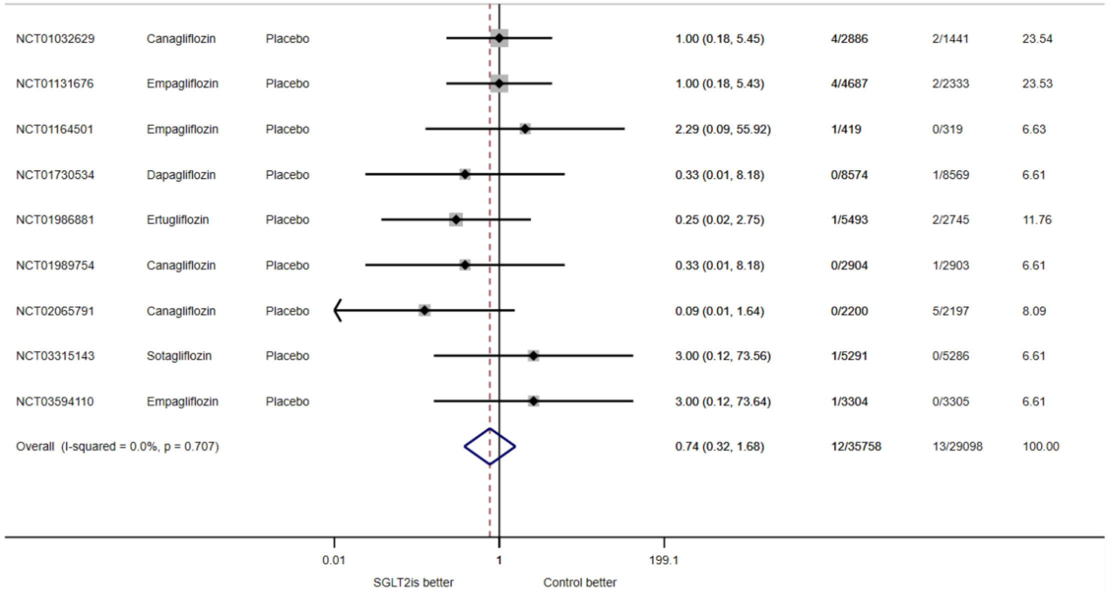

System 12-Injury, Poisoning And Procedural Complications-Figure S99 Meta-analysis of SGLT2is and Wound

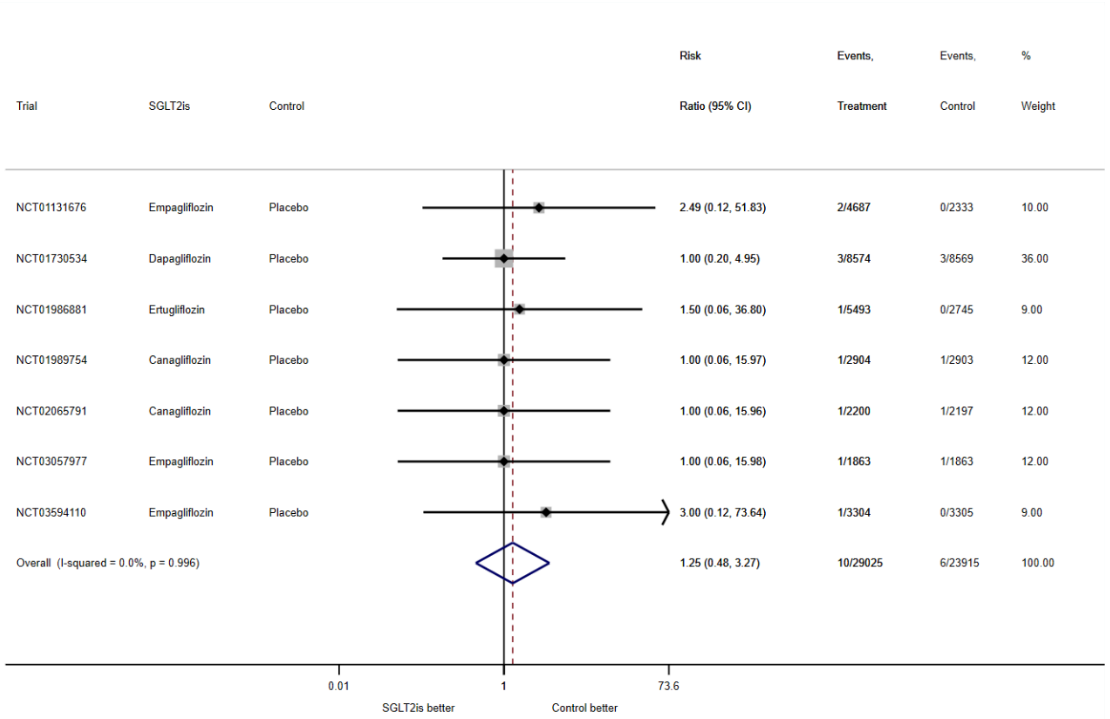

System 12-Injury, Poisoning And Procedural Complications-Figure S100 Meta-analysis of SGLT2is and Wound dehiscence

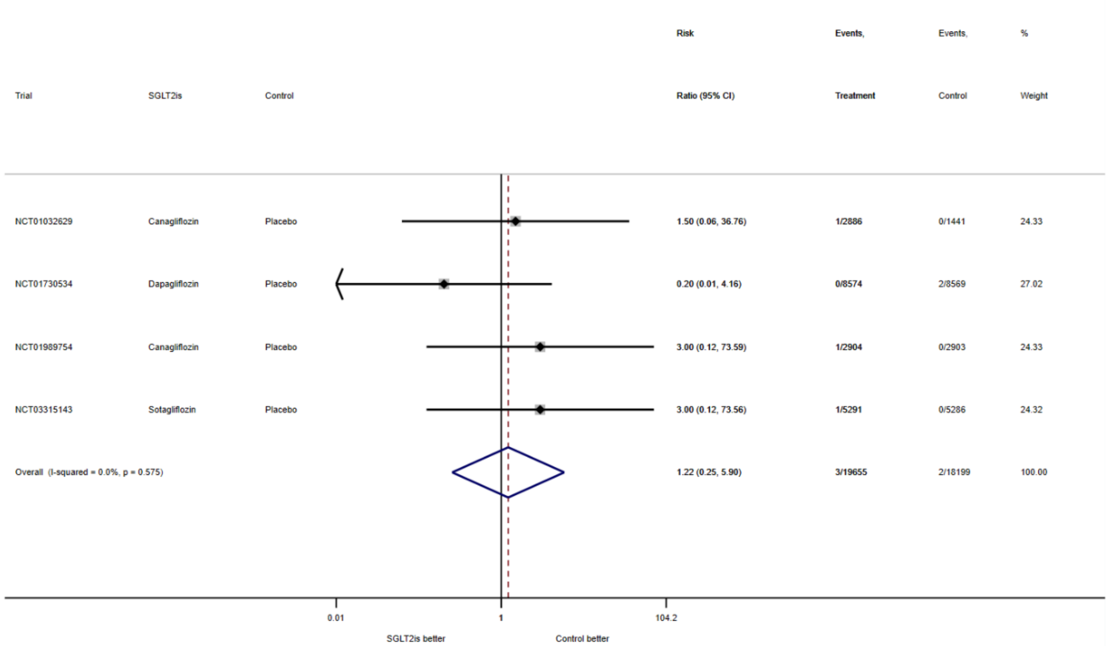

System 12-Injury, Poisoning And Procedural Complications-Figure S101 Meta-analysis of SGLT2is and Wound necrosis

Trial	SGLT2is	Control	Risk Ratio (95% CI)	Events, Treatment	Events, Control	% Weight
NCT01032629	Canagliflozin	Placebo	1.50 (0.06, 36.76)	1/2886	0/1441	3.90
NCT01131676	Empagliflozin	Placebo	2.49 (0.12, 51.83)	2/4687	0/2333	4.34
NCT01730534	Dapagliflozin	Placebo	1.20 (0.37, 3.93)	6/8574	5/8569	28.40
NCT01989754	Canagliflozin	Placebo	0.33 (0.01, 8.18)	0/2904	1/2903	3.90
NCT02065791	Canagliflozin	Placebo	0.33 (0.01, 8.17)	0/2200	1/2197	3.90
NCT03036124	Dapagliflozin	Placebo	1.00 (0.06, 15.98)	1/2368	1/2368	5.21
NCT03036150	Dapagliflozin	Placebo	0.33 (0.01, 8.18)	0/2149	1/2149	3.90
NCT03057951	Empagliflozin	Placebo	2.00 (0.18, 21.99)	2/2996	1/2989	6.94
NCT03057977	Empagliflozin	Placebo	0.33 (0.01, 8.18)	0/1863	1/1863	3.90
NCT03315143	Sotagliflozin	Placebo	1.40 (0.44, 4.40)	7/5291	5/5286	30.39
NCT03594110	Empagliflozin	Placebo	1.00 (0.06, 15.99)	1/3304	1/3305	5.21
Overall (I-squared = 0.0%, p = 0.984)			1.09 (0.58, 2.05)	20/39222	17/35403	100.00

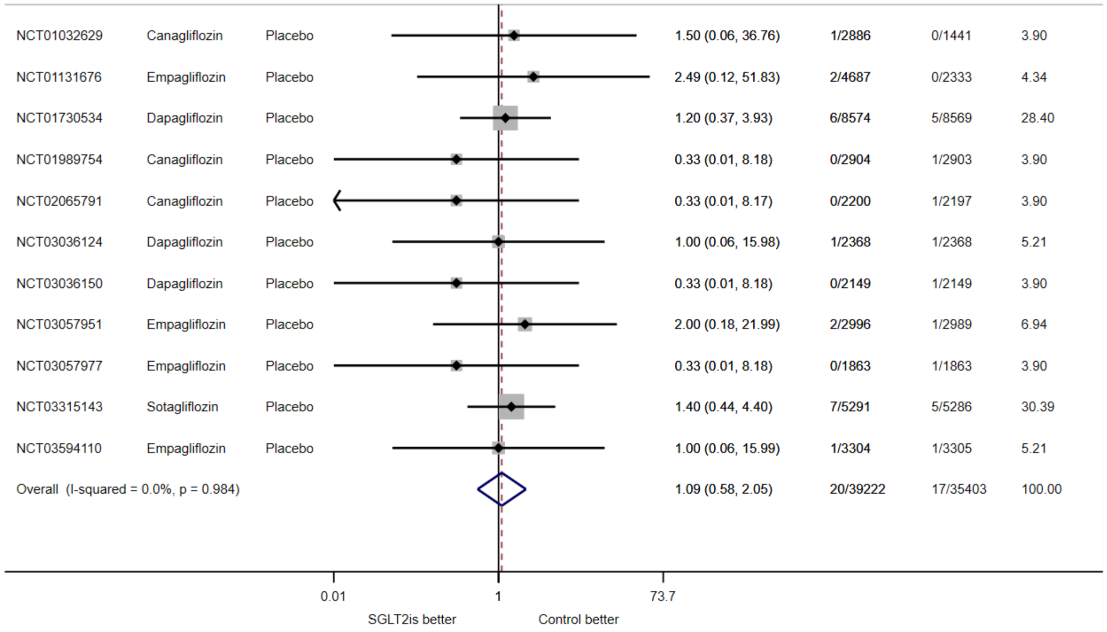

System 12-Injury, Poisoning And Procedural Complications-Figure S102 Meta-analysis of SGLT2is and Wrist fracture

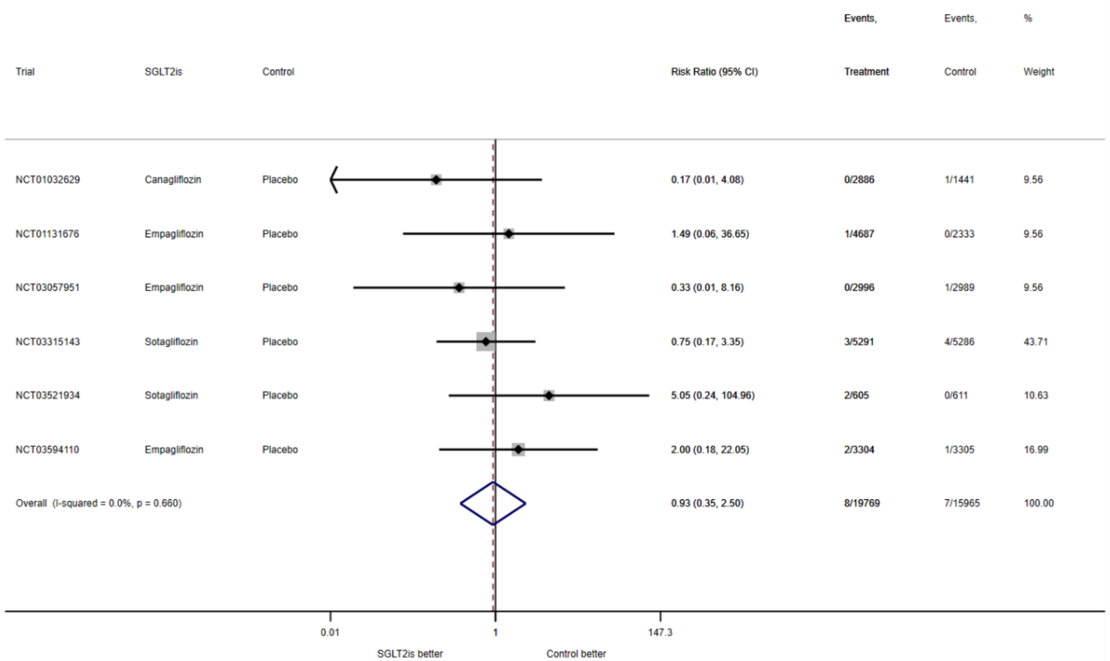

System 13-Investigations-Figure S1 Meta-analysis of SGLT2is and Alanine aminotransferase increased

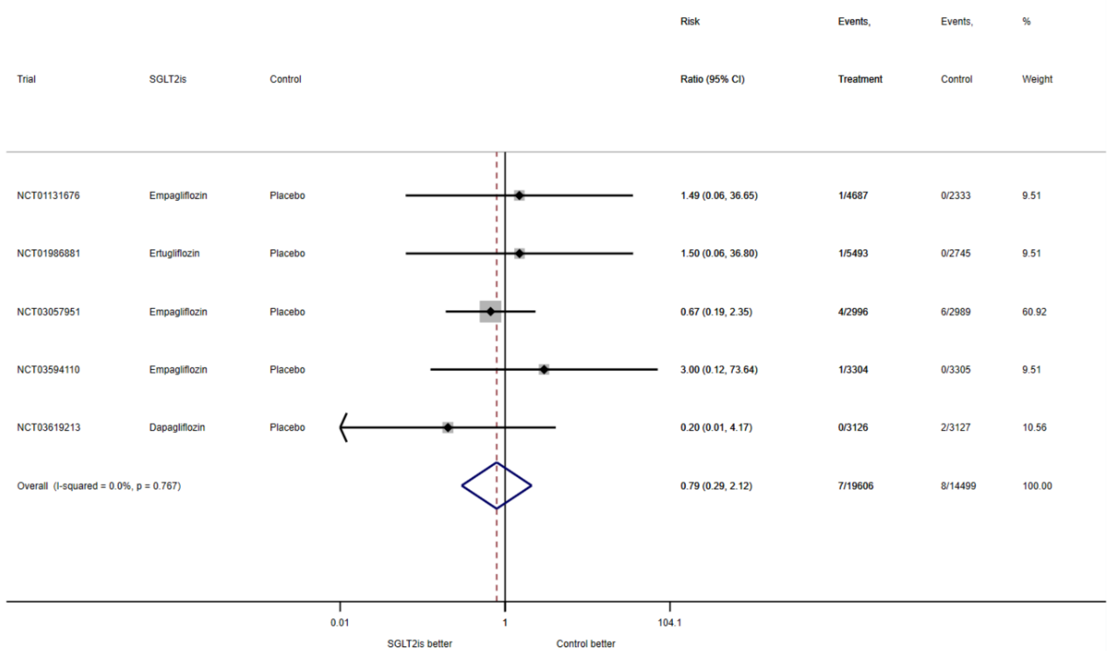

System 13-Investigations-Figure S2 Meta-analysis of SGLT2is and Anticoagulation drug level above therapeutic

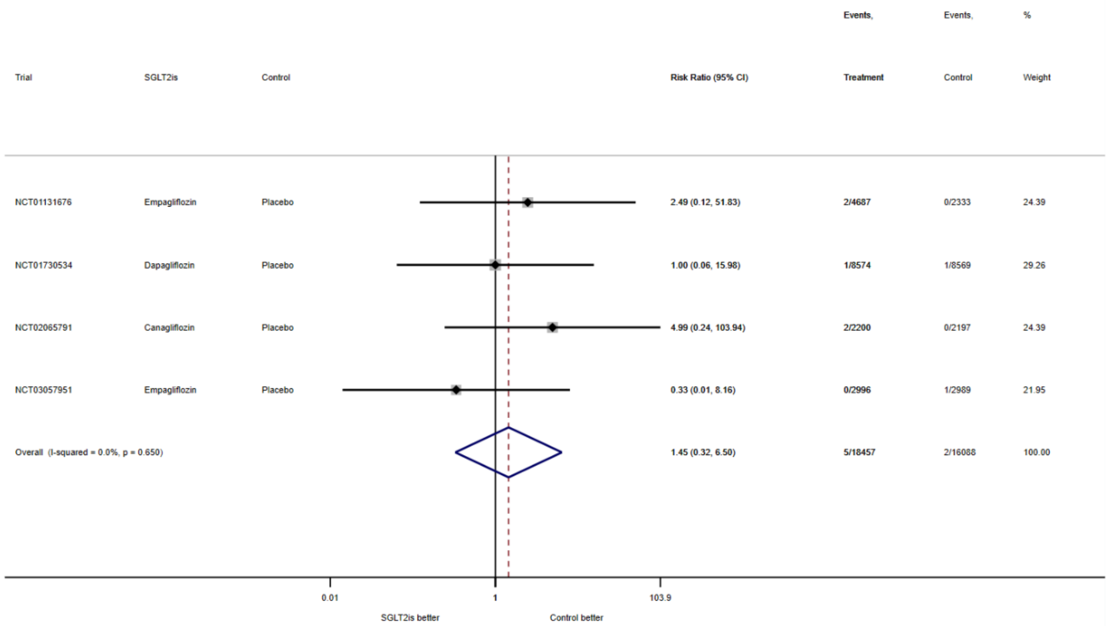

System 13-Investigations-Figure S3 Meta-analysis of SGLT2is and Blood creatine phosphokinase increased

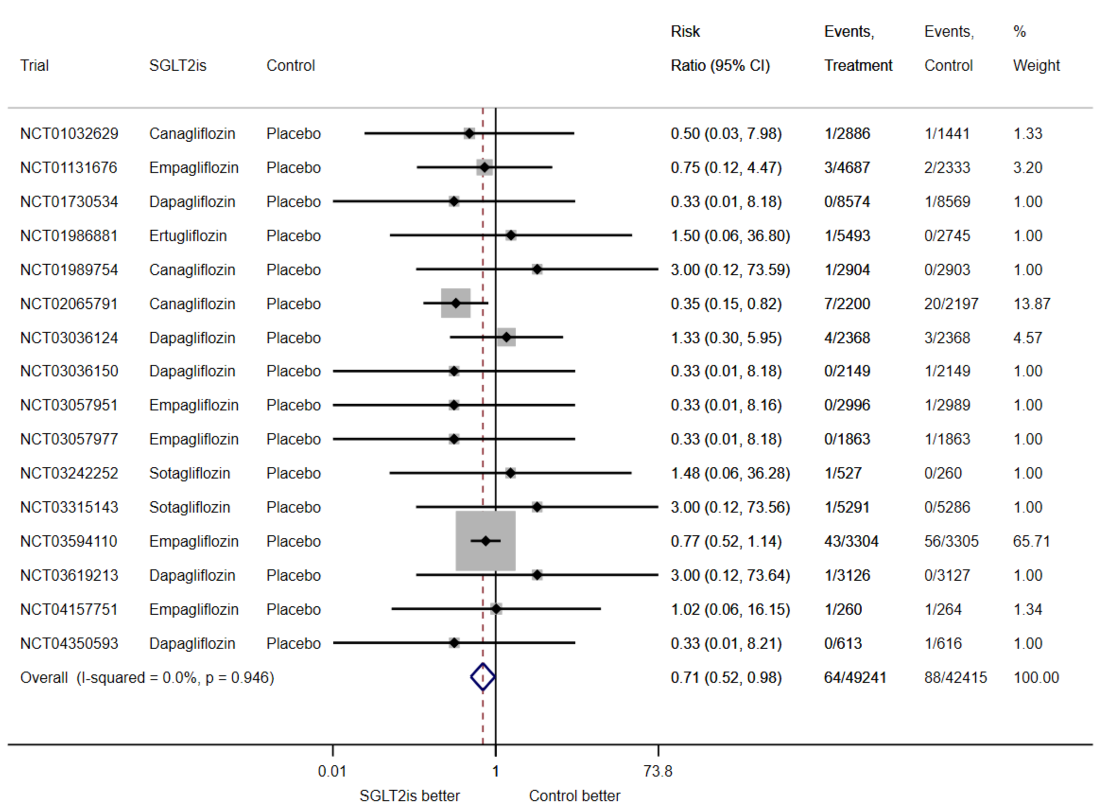

System 13-Investigations-Figure S4 Meta-analysis of SGLT2is and Blood creatinine increased

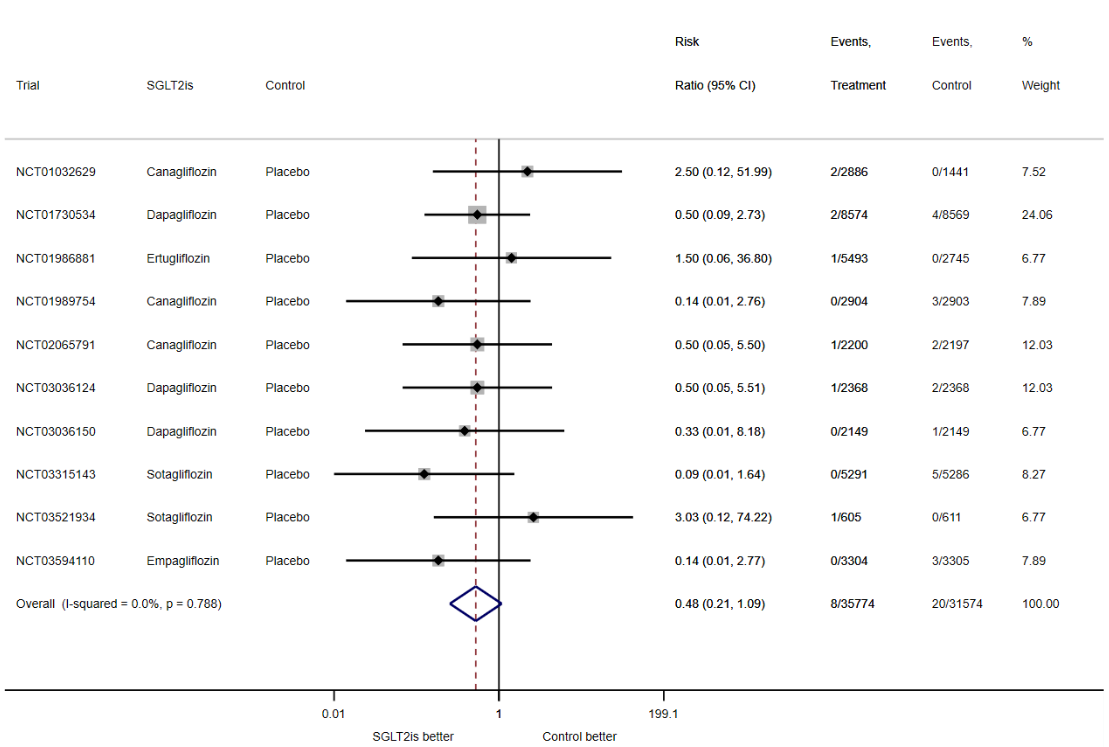

System 13-Investigations-Figure S5 Meta-analysis of SGLT2is and Blood glucose increased

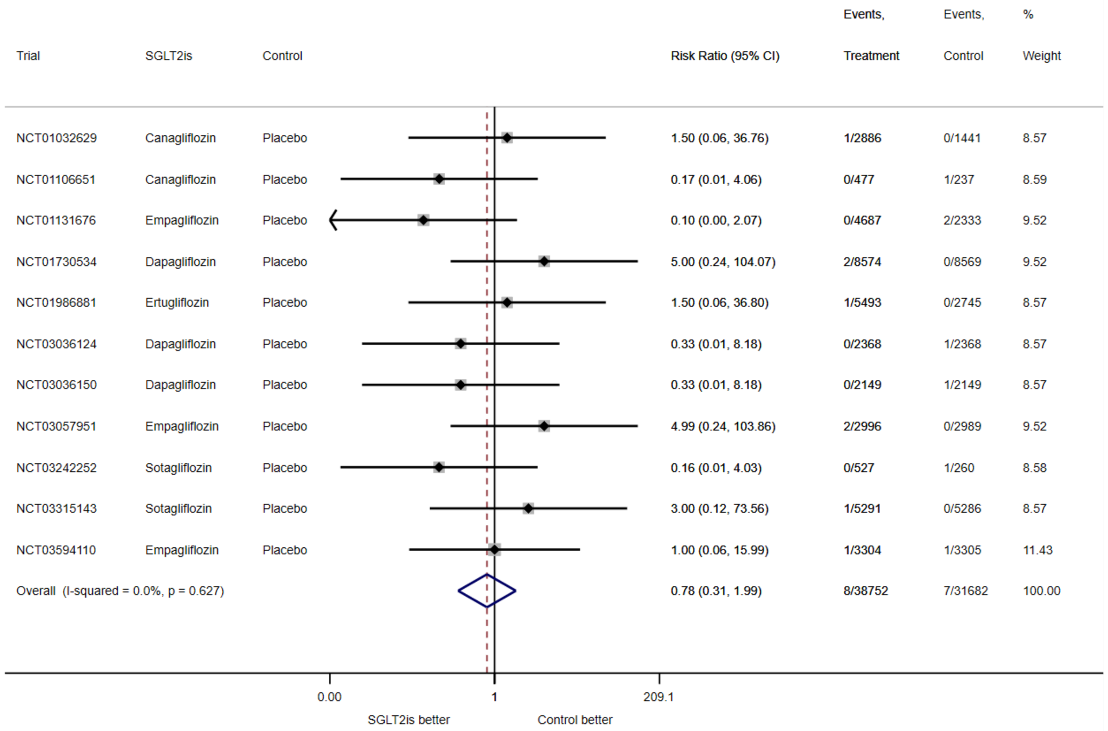

System 13-Investigations-Figure S6 Meta-analysis of SGLT2is and Blood pressure increased

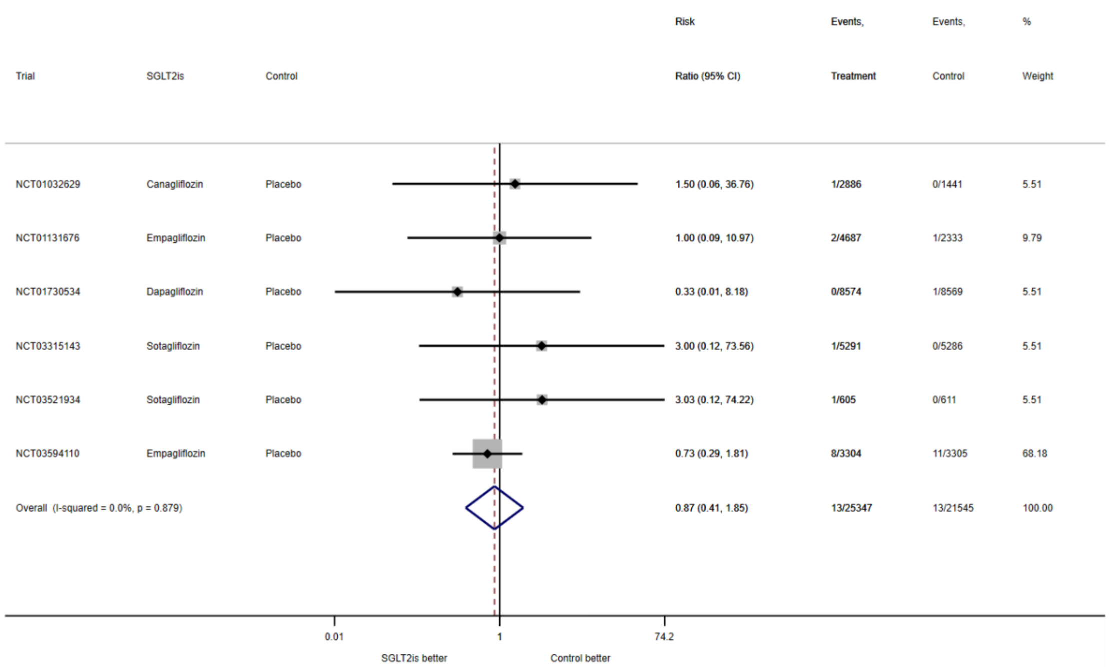

System 13-Investigations-Figure S7 Meta-analysis of SGLT2is and Catheterisation cardiac

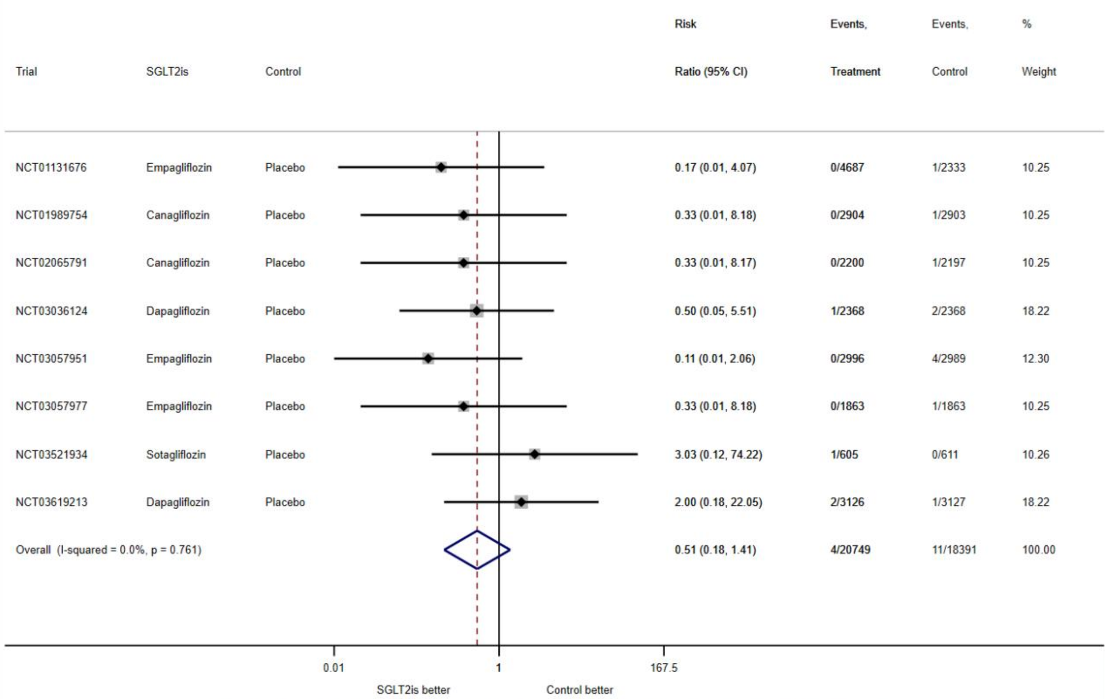

System 13-Investigations-Figure S8 Meta-analysis of SGLT2is and Ejection fraction decreased

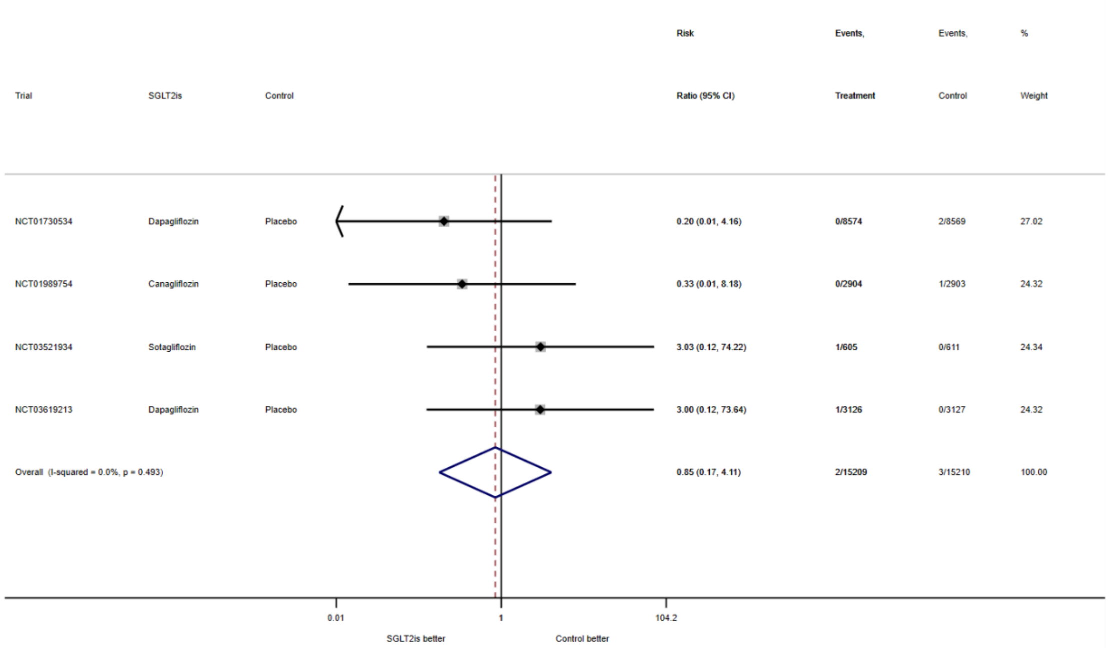

System 13-Investigations-Figure S9 Meta-analysis of SGLT2is and Electrocardiogram qt prolonged

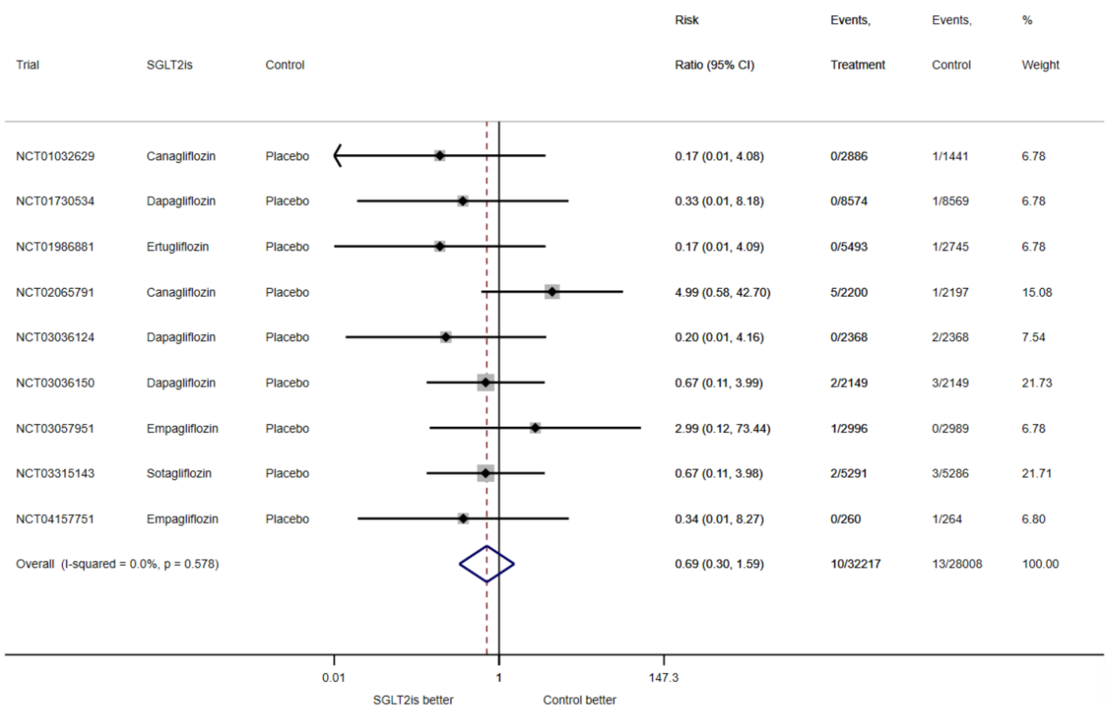

System 13-Investigations-Figure S10 Meta-analysis of SGLT2is and Glomerular filtration rate decreased

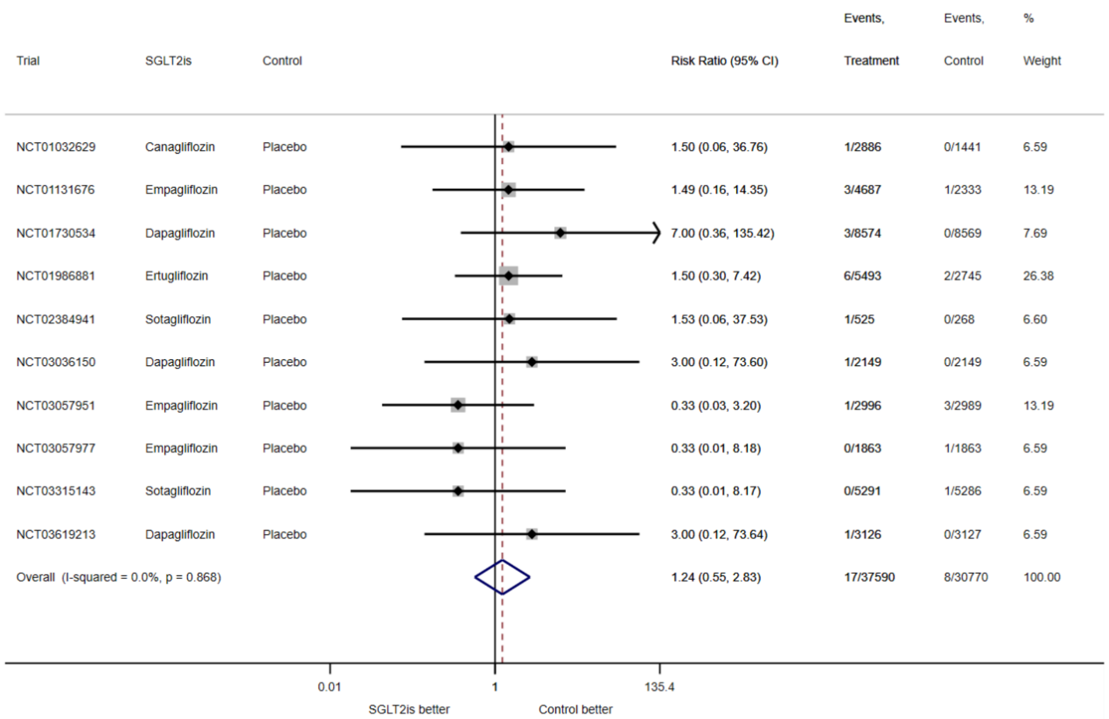

System 13-Investigations-Figure S11 Meta-analysis of SGLT2is and Hepatic enzyme increased

Trial	SGLT2is	Control	Risk Ratio (95% CI)	Events, Treatment	Events, Control	% Weight
NCT01730534	Dapagliflozin	Placebo	0.14 (0.01, 2.76)	0/8574	3/8569	16.27
NCT01989754	Canagliflozin	Placebo	0.33 (0.01, 8.18)	0/2904	1/2903	13.95
NCT02065791	Canagliflozin	Placebo	0.33 (0.01, 8.17)	0/2200	1/2197	13.95
NCT03036124	Dapagliflozin	Placebo	0.33 (0.01, 8.18)	0/2368	1/2368	13.95
NCT03315143	Sotagliflozin	Placebo	3.00 (0.12, 73.56)	1/5291	0/5286	13.95
NCT03619213	Dapagliflozin	Placebo	0.33 (0.01, 8.18)	0/3126	1/3127	13.95
NCT04157751	Empagliflozin	Placebo	0.34 (0.01, 8.27)	0/260	1/264	13.99
Overall (I-squared = 0.0%, p = 0.915)			0.40 (0.12, 1.31)	1/24723	8/24714	100.00

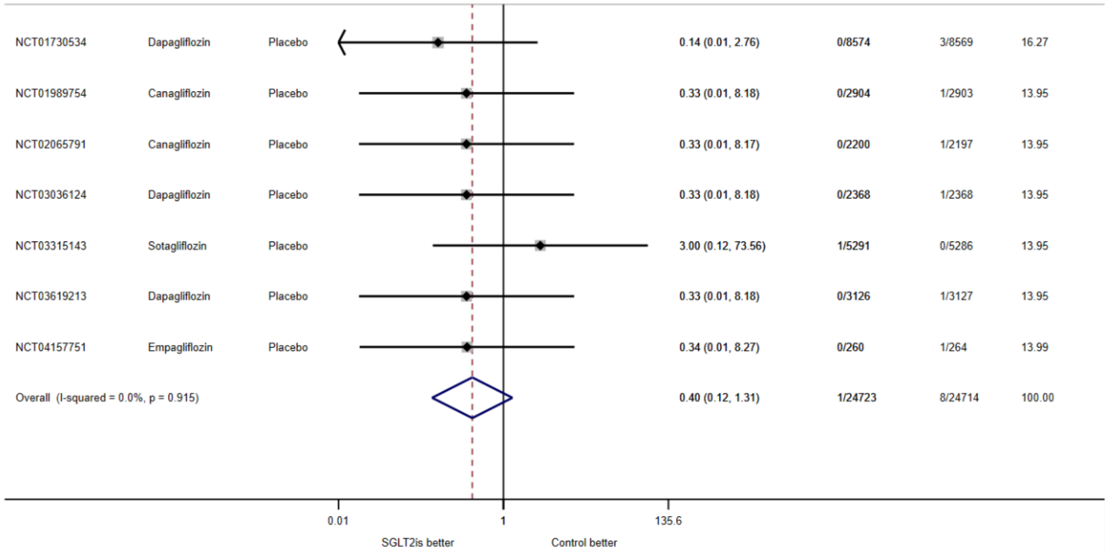

System 13-Investigations-Figure S12 Meta-analysis of SGLT2is and International normalised ratio increased

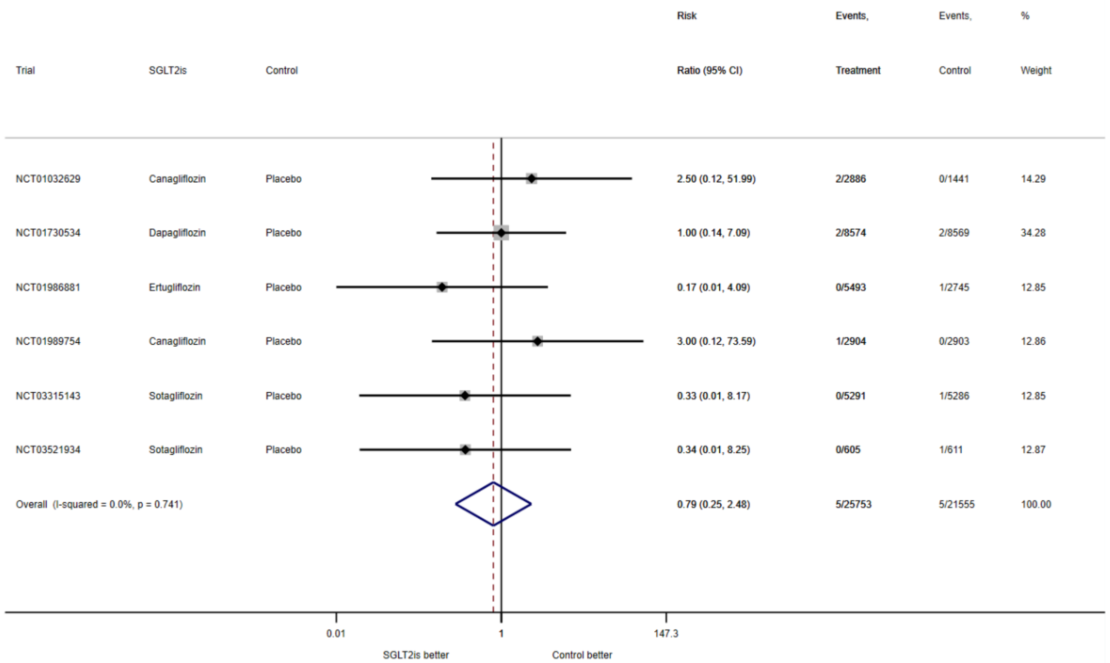

System 13-Investigations-Figure S13 Meta-analysis of SGLT2is and Liver function test increased

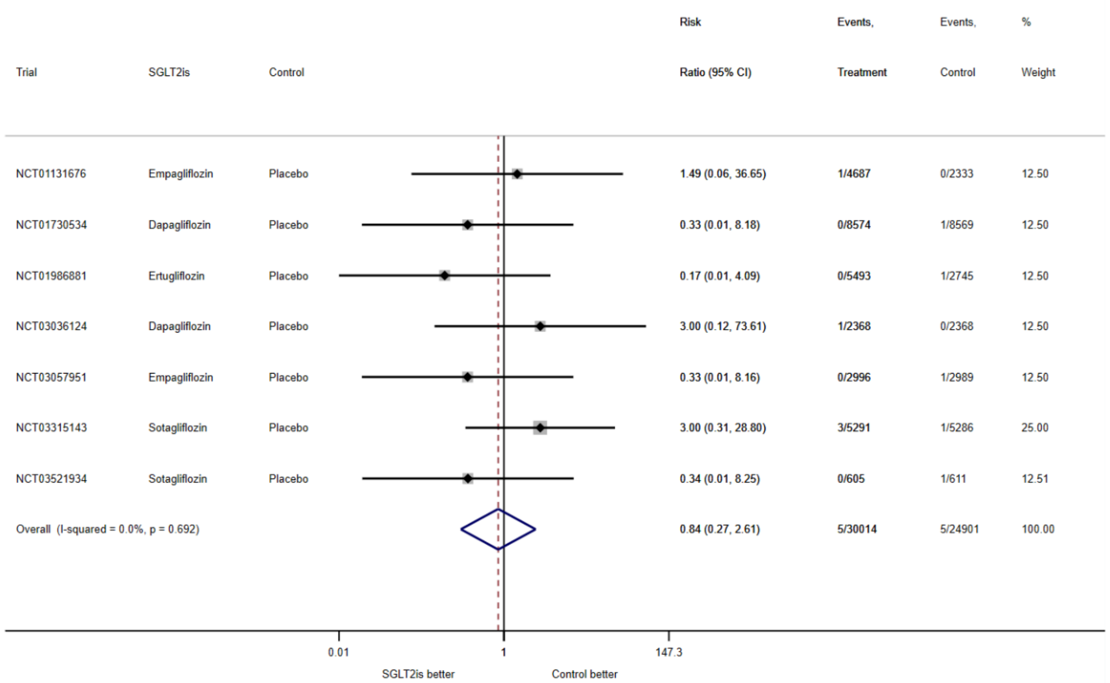

System 13-Investigations-Figure S14 Meta-analysis of SGLT2is and Transaminases increased

Trial	SGLT2is	Control	Risk Ratio (95% CI)	Events, Treatment	Events, Control	% Weight
NCT01032629	Canagliflozin	Placebo	0.17 (0.01, 4.08)	0/2886	1/1441	5.09
NCT01131676	Empagliflozin	Placebo	0.50 (0.03, 7.95)	1/4687	1/2333	6.79
NCT01730534	Dapagliflozin	Placebo	1.57 (0.61, 4.05)	11/8574	7/8569	58.12
NCT02065791	Canagliflozin	Placebo	0.33 (0.01, 8.17)	0/2200	1/2197	5.09
NCT03036124	Dapagliflozin	Placebo	5.00 (0.24, 104.09)	2/2368	0/2368	5.66
NCT03057951	Empagliflozin	Placebo	2.00 (0.18, 21.99)	2/2996	1/2989	9.05
NCT03315143	Sotagliflozin	Placebo	0.33 (0.01, 8.17)	0/5291	1/5286	5.09
NCT04157751	Empagliflozin	Placebo	0.34 (0.01, 8.27)	0/260	1/264	5.10
Overall (I-squared = 0.0%, p = 0.662)			1.12 (0.54, 2.30)	16/29262	13/25447	100.00

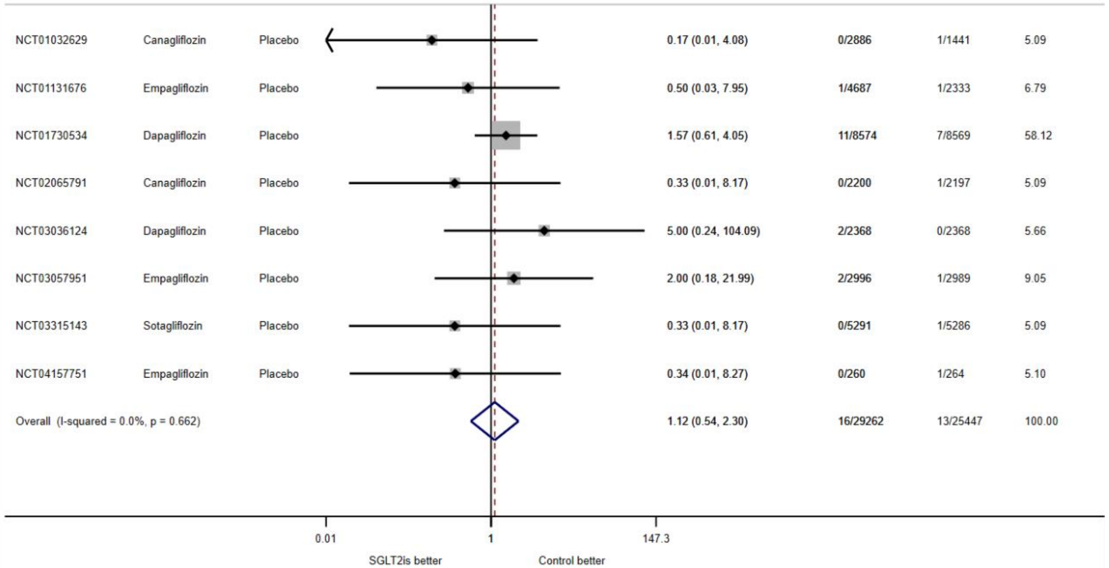

System 13-Investigations-Figure S15 Meta-analysis of SGLT2is and Troponin increased

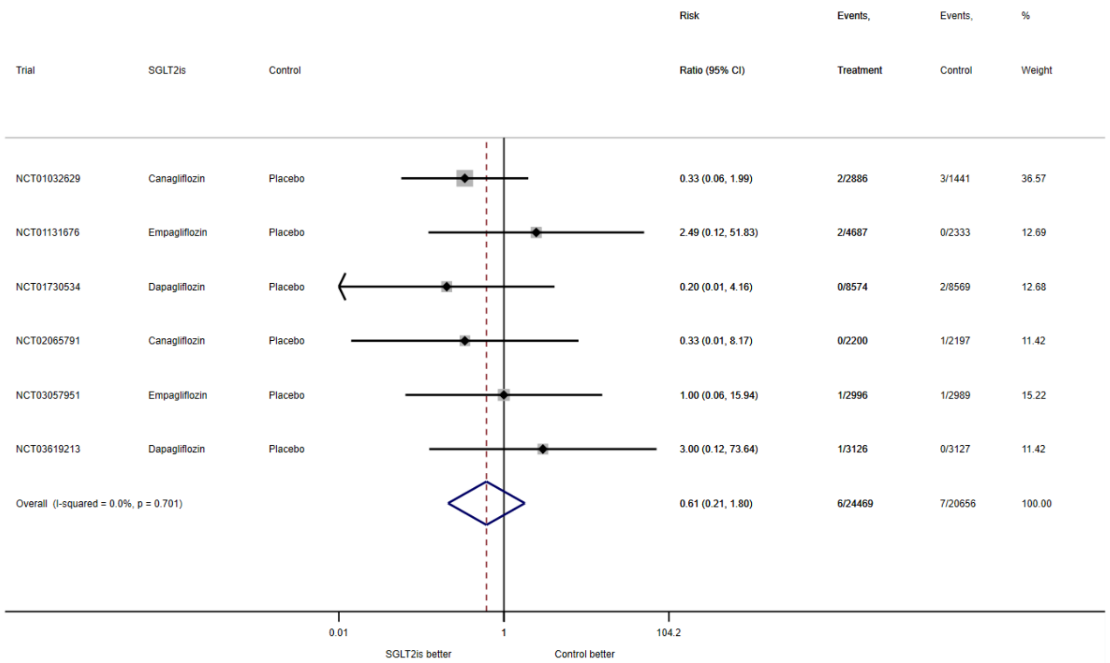

System 13-Investigations-Figure S16 Meta-analysis of SGLT2is and Weight decreased

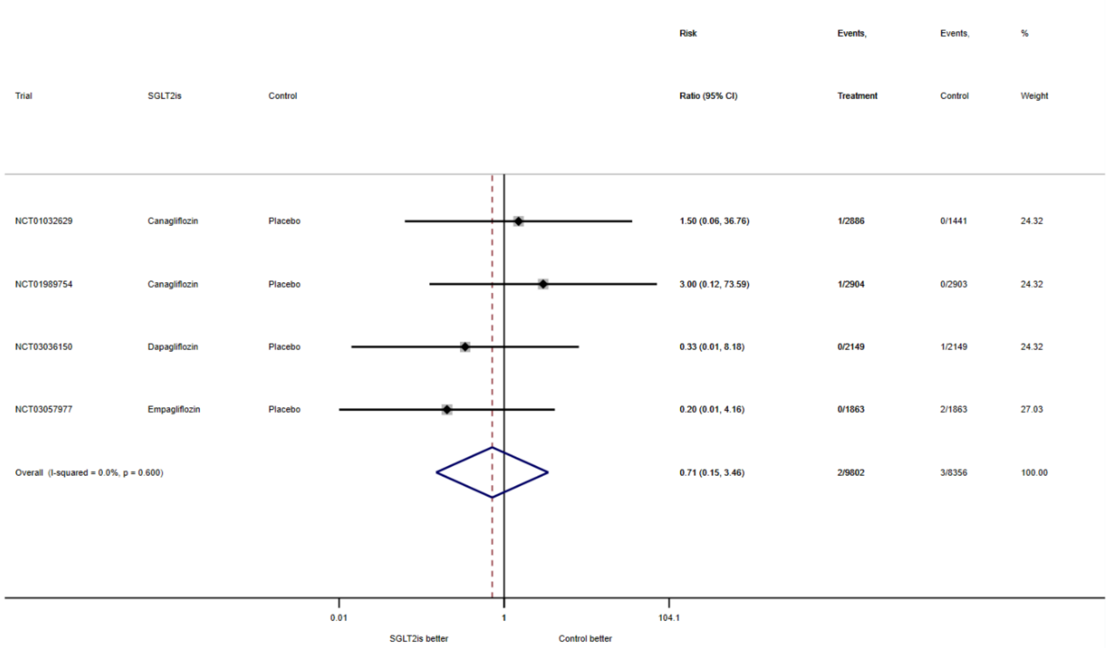

System 14-Metabolism And Nutrition Disorders-Figure S1 Meta-analysis of SGLT2is and Acidosis

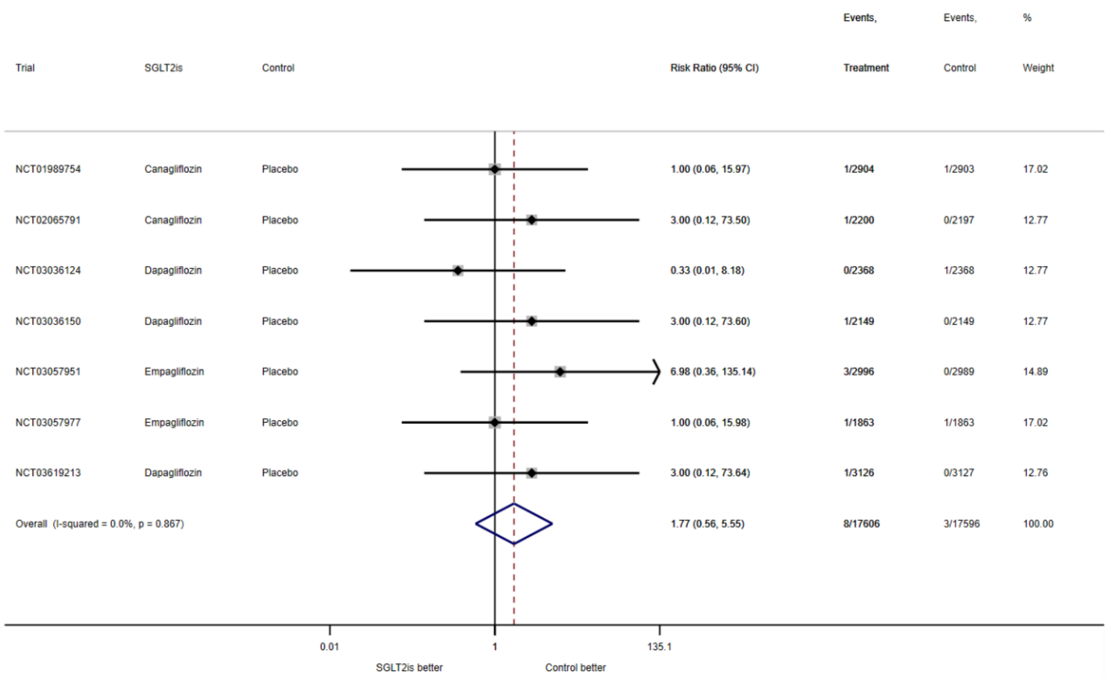

System 14-Metabolism And Nutrition Disorders-Figure S2 Meta-analysis of SGLT2is and Cachexia

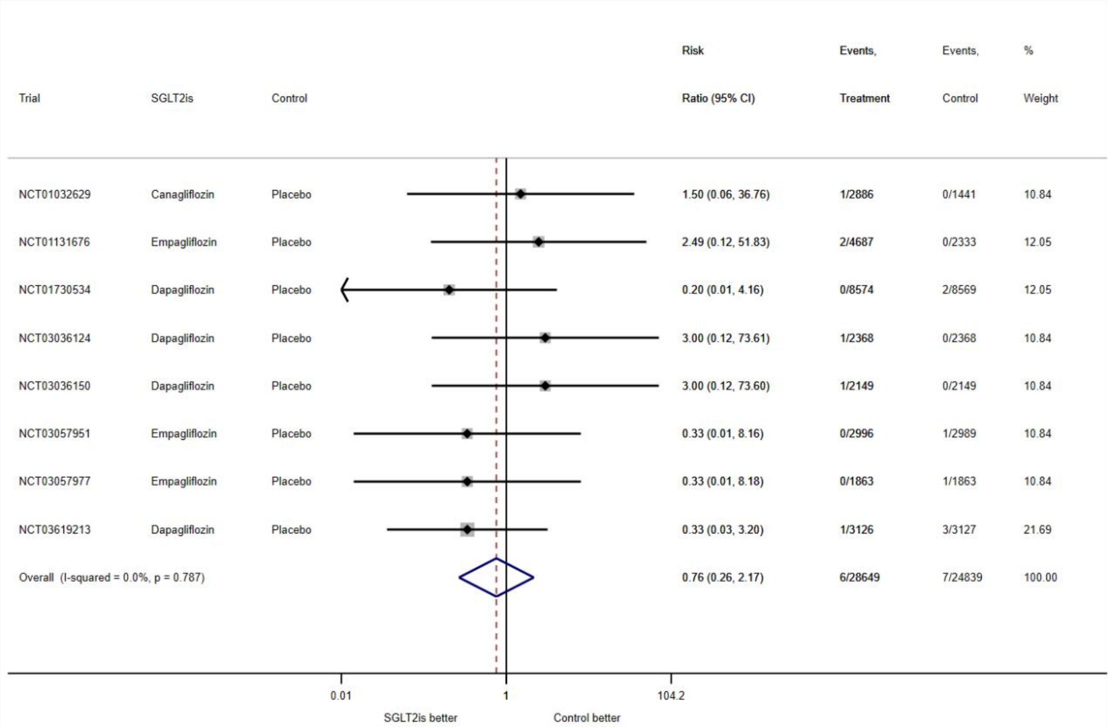

System 14-Metabolism And Nutrition Disorders-Figure S3 Meta-analysis of SGLT2is and Decreased appetite

Trial	SGLT2is	Control	Risk Ratio (95% CI)	Events, Treatment	Events, Control	% Weight
NCT01032629	Canagliflozin	Placebo	0.62 (0.17, 2.32)	5/2886	4/1441	4.10
NCT01131676	Empagliflozin	Placebo	1.00 (0.34, 2.91)	10/4687	5/2333	6.15
NCT01730534	Dapagliflozin	Placebo	1.05 (0.56, 1.97)	20/8574	19/8569	17.99
NCT01986881	Ertugliflozin	Placebo	0.50 (0.14, 1.72)	5/5493	5/2745	4.61
NCT01989754	Canagliflozin	Placebo	1.50 (0.25, 8.97)	3/2904	2/2903	2.21
NCT02065791	Canagliflozin	Placebo	0.50 (0.15, 1.66)	4/2200	8/2197	4.93
NCT03036124	Dapagliflozin	Placebo	1.25 (0.49, 3.16)	10/2368	8/2368	8.22
NCT03036150	Dapagliflozin	Placebo	0.43 (0.11, 1.66)	3/2149	7/2149	3.88
NCT03057951	Empagliflozin	Placebo	1.00 (0.37, 2.65)	8/2996	8/2989	7.39
NCT03057977	Empagliflozin	Placebo	2.00 (0.60, 6.63)	8/1863	4/1863	4.93
NCT03315143	Sotagliflozin	Placebo	0.86 (0.29, 2.55)	6/5291	7/5286	5.96
NCT03521934	Sotagliflozin	Placebo	0.76 (0.17, 3.37)	3/605	4/611	3.18
NCT03594110	Empagliflozin	Placebo	1.10 (0.60, 2.01)	22/3304	20/3305	19.43
NCT03619213	Dapagliflozin	Placebo	0.75 (0.26, 2.16)	6/3126	8/3127	6.33
NCT04157751	Empagliflozin	Placebo	3.05 (0.12, 74.43)	1/260	0/264	0.69
Overall (I-squared = 0.0%, p = 0.931)			0.94 (0.72, 1.23)	114/48706	109/42150	100.00

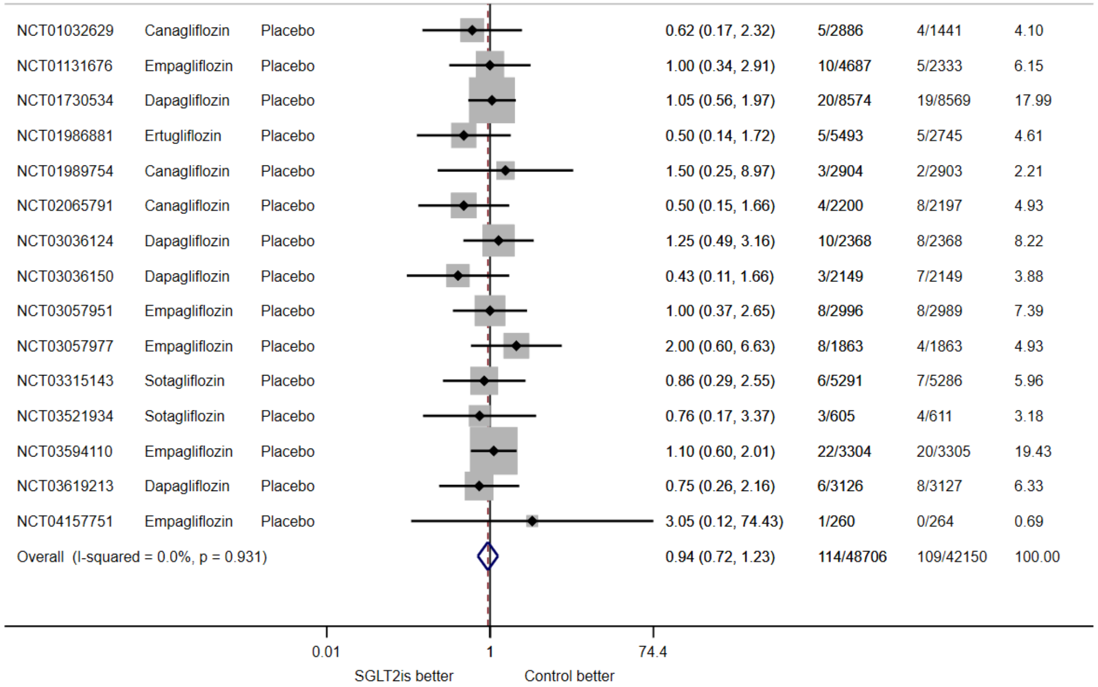

System 14-Metabolism And Nutrition Disorders-Figure S4 Meta-analysis of SGLT2is and Dehydration

Trial	SGLT2is	Control	Risk Ratio (95% CI)	Events, Treatment	Events, Control	% Weight
NCT01032629	Canagliflozin	Placebo	0.60 (0.26, 1.38)	12/2886	10/1441	11.52
NCT01131676	Empagliflozin	Placebo	0.41 (0.13, 1.36)	5/4687	6/2333	5.74
NCT01730534	Dapagliflozin	Placebo	0.38 (0.14, 1.08)	5/8574	13/8569	7.59
NCT01986881	Ertugliflozin	Placebo	0.50 (0.16, 1.55)	6/5493	6/2745	6.31
NCT01989754	Canagliflozin	Placebo	0.28 (0.10, 0.75)	5/2904	18/2903	8.24
NCT02033889	Ertugliflozin	Placebo/Glimepiride	0.17 (0.01, 4.14)	0/412	1/209	0.79
NCT02065791	Canagliflozin	Placebo	0.50 (0.17, 1.46)	5/2200	10/2197	7.02
NCT03036124	Dapagliflozin	Placebo	1.25 (0.34, 4.65)	5/2368	4/2368	4.68
NCT03036150	Dapagliflozin	Placebo	1.80 (0.60, 5.36)	9/2149	5/2149	6.77
NCT03057951	Empagliflozin	Placebo	0.33 (0.12, 0.91)	5/2996	15/2989	7.90
NCT03057977	Empagliflozin	Placebo	0.80 (0.22, 2.97)	4/1863	5/1863	4.68
NCT03315143	Sotagliflozin	Placebo	0.56 (0.19, 1.66)	5/5291	9/5286	6.76
NCT03594110	Empagliflozin	Placebo	0.83 (0.45, 1.51)	19/3304	23/3305	21.99
Overall (I-squared = 0.0%, p = 0.454)			0.59 (0.45, 0.79)	85/45127	125/38357	100.00

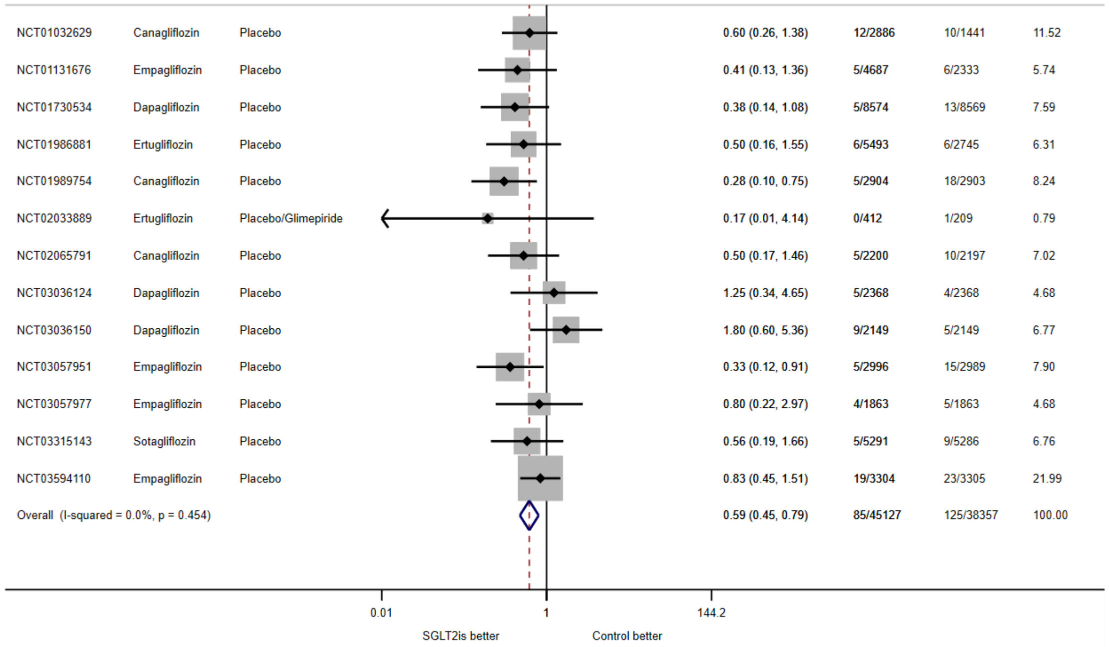

System 14-Metabolism And Nutrition Disorders-Figure S5 Meta-analysis of SGLT2is and Diabetes mellitus

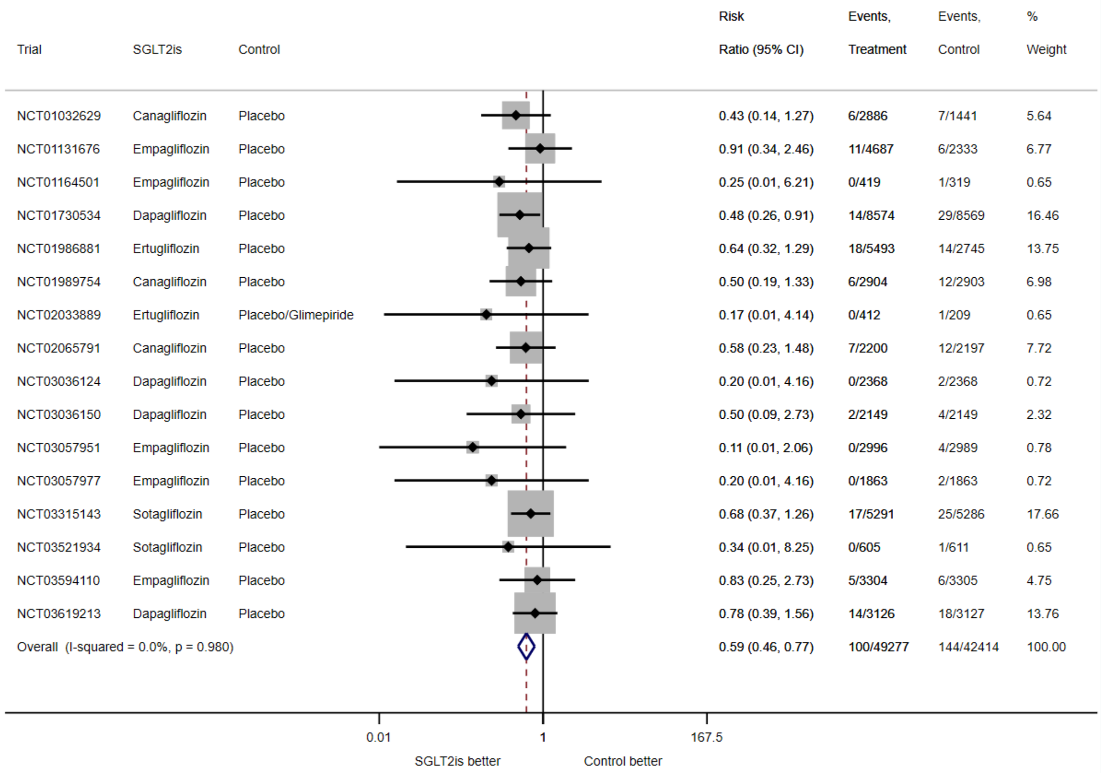

System 14-Metabolism And Nutrition Disorders-Figure S6 Meta-analysis of SGLT2is and Diabetes mellitus inadequate control

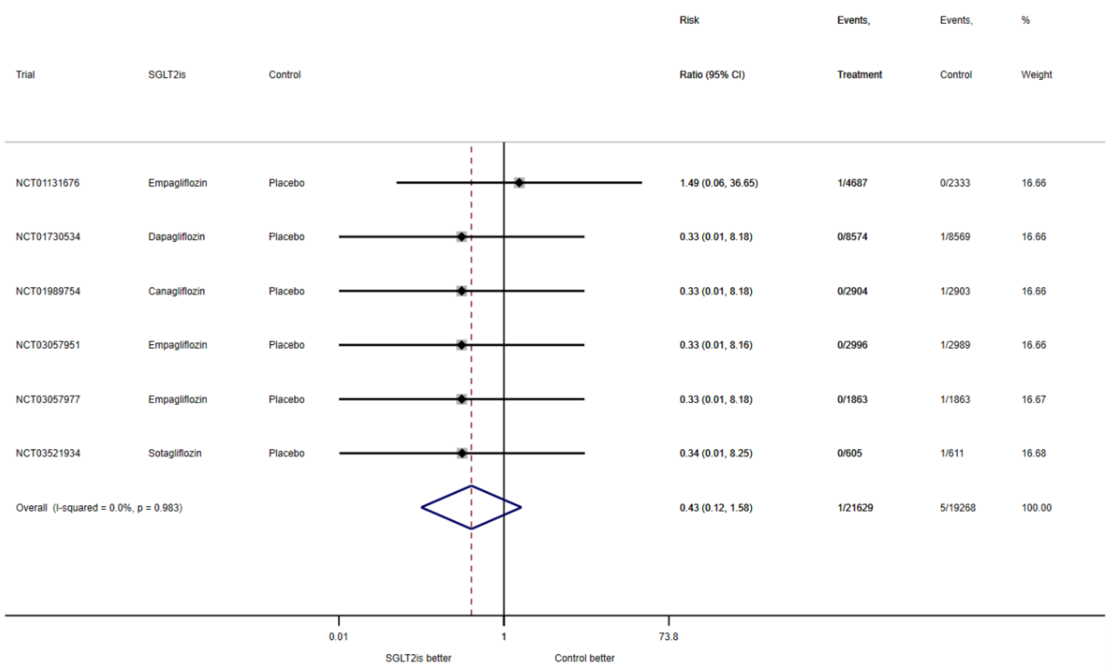

System 14-Metabolism And Nutrition Disorders-Figure S7 Meta-analysis of SGLT2is and Diabetic complication

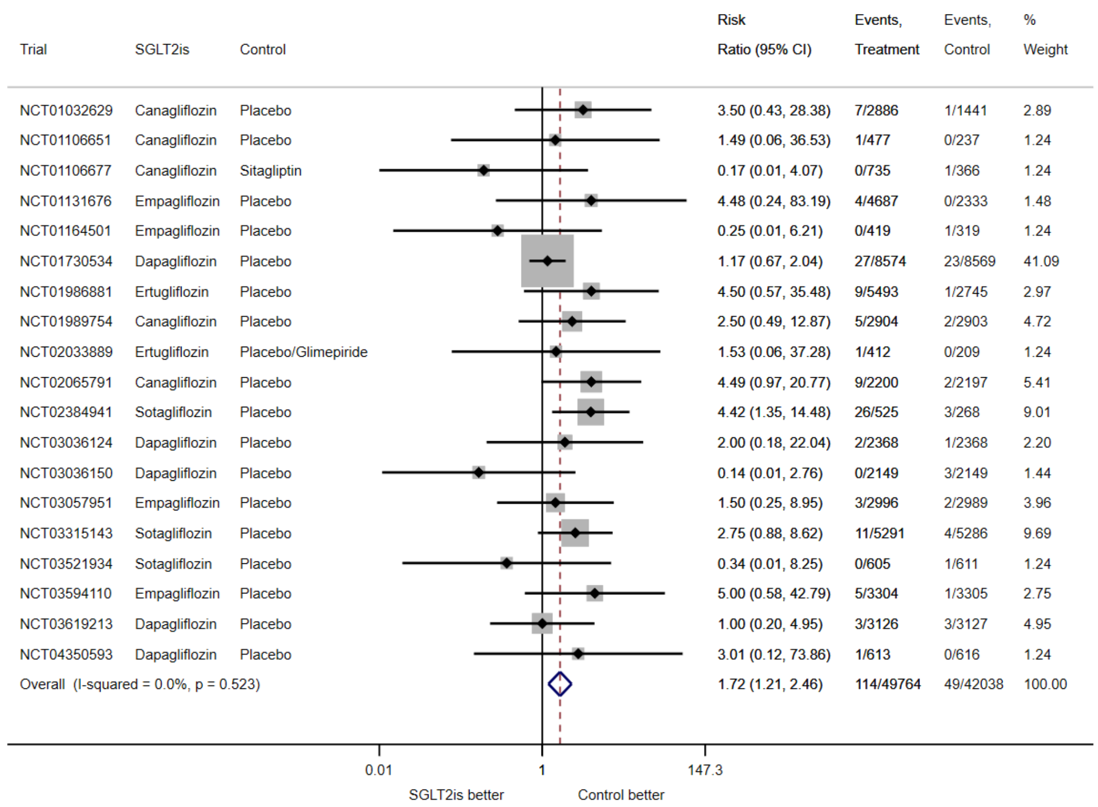

System 14-Metabolism And Nutrition Disorders-Figure S8 Meta-analysis of SGLT2is and Diabetic ketoacidosis

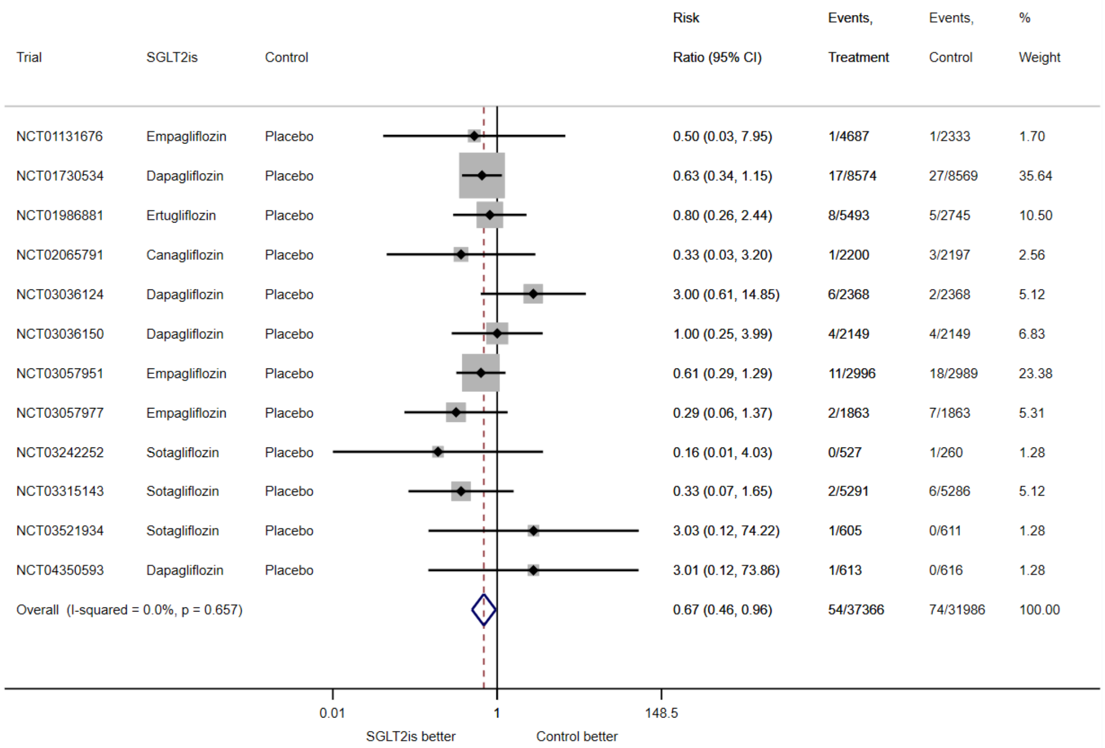

System 14-Metabolism And Nutrition Disorders-Figure S9 Meta-analysis of SGLT2is and Diabetic metabolic decompensation

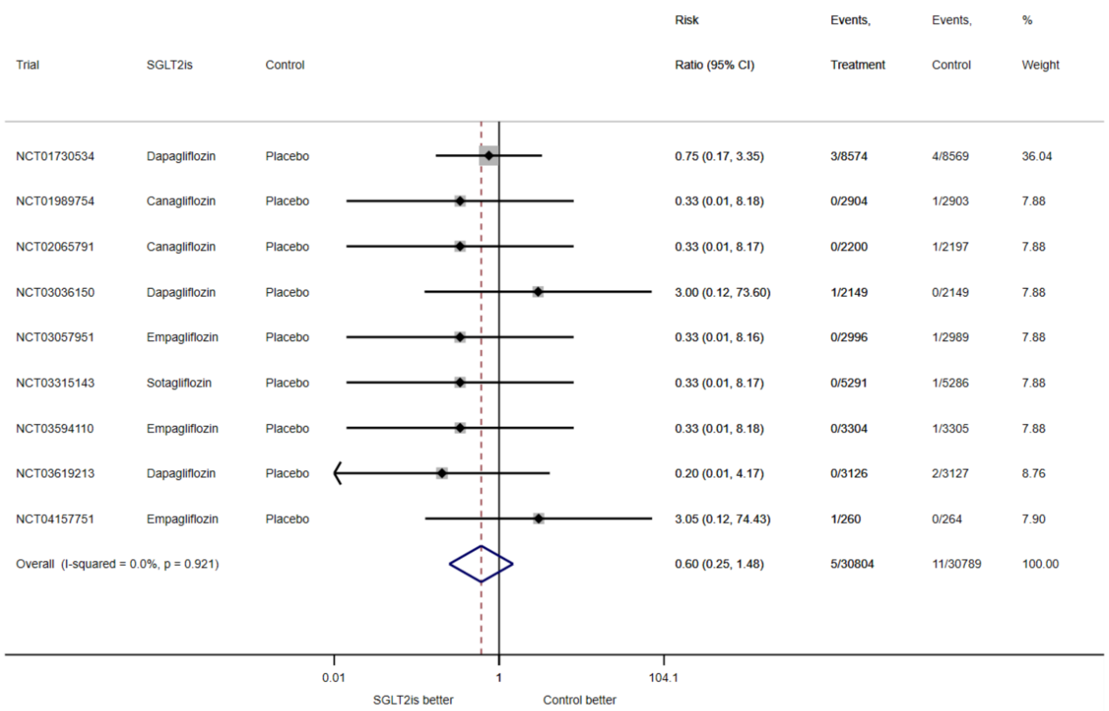

System 14-Metabolism And Nutrition Disorders-Figure S10 Meta-analysis of SGLT2is and Electrolyte imbalance

Trial	SGLT2is	Control	Risk Ratio (95% CI)	Events, Treatment	Events, Control	% Weight
NCT01032629	Canagliflozin	Placebo	1.50 (0.06, 36.76)	1/2886	0/1441	3.56
NCT01131676	Empagliflozin	Placebo	0.17 (0.01, 4.07)	0/4687	1/2333	3.55
NCT01164501	Empagliflozin	Placebo	0.25 (0.01, 6.21)	0/419	1/319	3.56
NCT01730534	Dapagliflozin	Placebo	1.00 (0.06, 15.98)	1/8574	1/8569	4.74
NCT01986881	Ertugliflozin	Placebo	0.12 (0.01, 1.12)	1/5493	4/2745	7.58
NCT01989754	Canagliflozin	Placebo	0.50 (0.05, 5.51)	1/2904	2/2903	6.32
NCT02065791	Canagliflozin	Placebo	0.75 (0.17, 3.34)	3/2200	4/2197	16.27
NCT03036124	Dapagliflozin	Placebo	0.33 (0.01, 8.18)	0/2368	1/2368	3.55
NCT03036150	Dapagliflozin	Placebo	0.75 (0.17, 3.35)	3/2149	4/2149	16.27
NCT03057951	Empagliflozin	Placebo	1.00 (0.06, 15.94)	1/2996	1/2989	4.74
NCT03521934	Sotagliflozin	Placebo	0.34 (0.01, 8.25)	0/605	1/611	3.56
NCT03594110	Empagliflozin	Placebo	0.44 (0.14, 1.44)	4/3304	9/3305	26.29
Overall (I-squared = 0.0%, p = 0.975)			0.50 (0.28, 0.92)	15/38585	29/31929	100.00

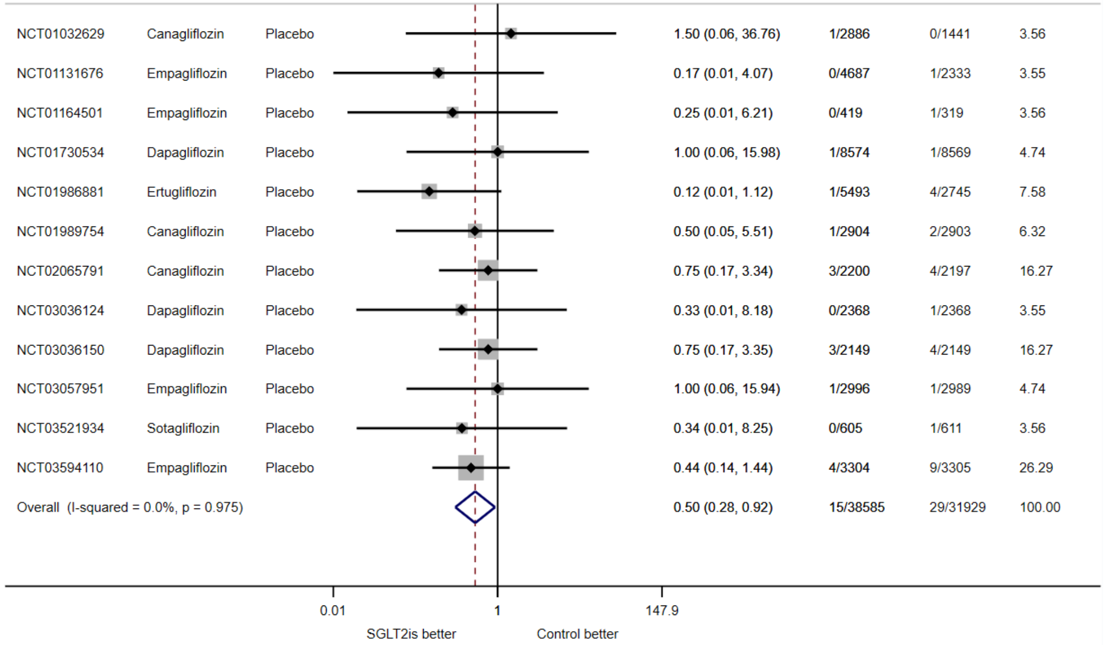

System 14-Metabolism And Nutrition Disorders-Figure S11 Meta-analysis of SGLT2is and Fluid overload

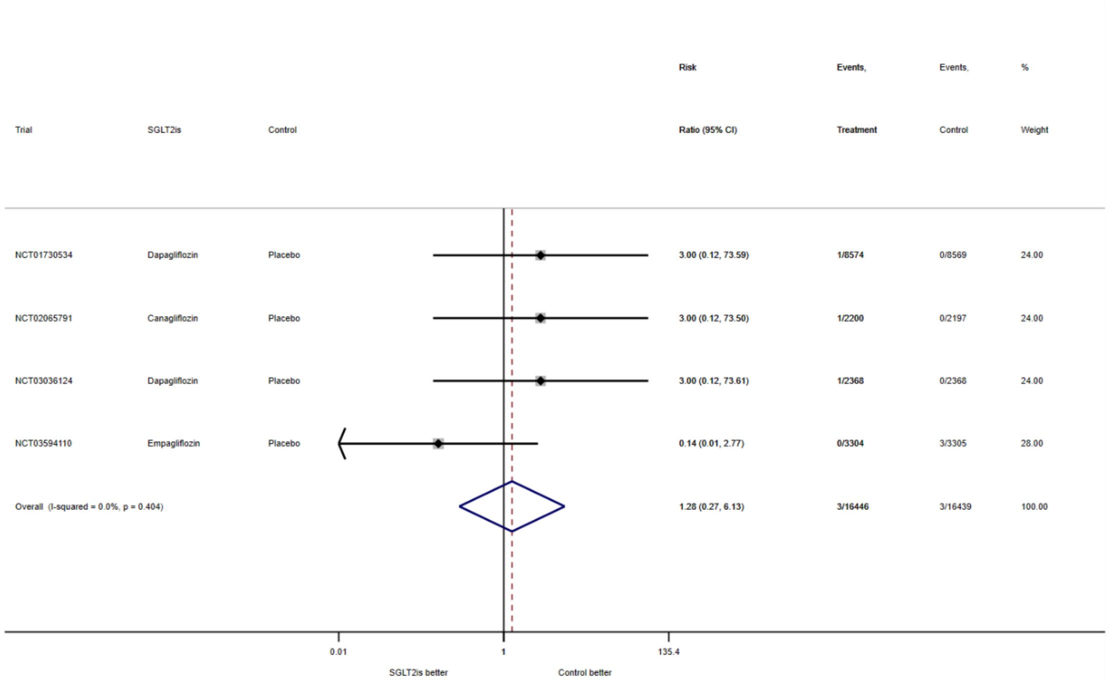

System 14-Metabolism And Nutrition Disorders-Figure S12 Meta-analysis of SGLT2is and Fluid retention

Trial	SGLT2is	Control	Risk Ratio (95% CI)	Events, Treatment	Events, Control	% Weight
NCT01032629	Canagliflozin	Placebo	2.00 (0.22, 17.85)	4/2886	1/1441	5.21
NCT01131676	Empagliflozin	Placebo	2.49 (0.12, 51.83)	2/4687	0/2333	2.71
NCT01730534	Dapagliflozin	Placebo	0.50 (0.13, 2.00)	3/8574	6/8569	13.02
NCT01986881	Ertugliflozin	Placebo	0.25 (0.02, 2.75)	1/5493	2/2745	4.34
NCT01989754	Canagliflozin	Placebo	1.00 (0.06, 15.97)	1/2904	1/2903	3.25
NCT03036124	Dapagliflozin	Placebo	0.50 (0.09, 2.73)	2/2368	4/2368	8.68
NCT03036150	Dapagliflozin	Placebo	0.50 (0.05, 5.51)	1/2149	2/2149	4.34
NCT03057951	Empagliflozin	Placebo	0.80 (0.21, 2.97)	4/2996	5/2989	14.48
NCT03057977	Empagliflozin	Placebo	0.33 (0.03, 3.20)	1/1863	3/1863	4.88
NCT03315143	Sotagliflozin	Placebo	0.33 (0.03, 3.20)	1/5291	3/5286	4.88
NCT03521934	Sotagliflozin	Placebo	3.03 (0.12, 74.22)	1/605	0/611	2.44
NCT03594110	Empagliflozin	Placebo	1.00 (0.29, 3.45)	5/3304	5/3305	16.29
NCT03619213	Dapagliflozin	Placebo	1.00 (0.25, 4.00)	4/3126	4/3127	13.03
NCT04157751	Empagliflozin	Placebo	0.34 (0.01, 8.27)	0/260	1/264	2.45
Overall (I-squared = 0.0%, p = 0.971)			0.73 (0.44, 1.20)	30/46506	37/39953	100.00

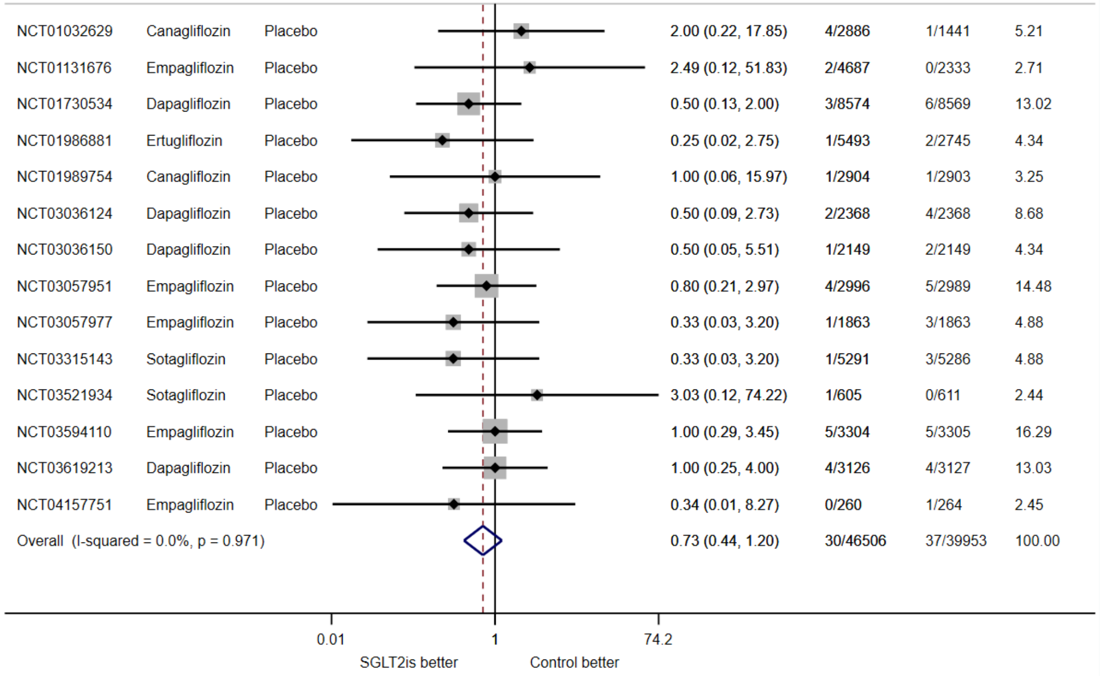

System 14-Metabolism And Nutrition Disorders-Figure S13 Meta-analysis of SGLT2is and Gout

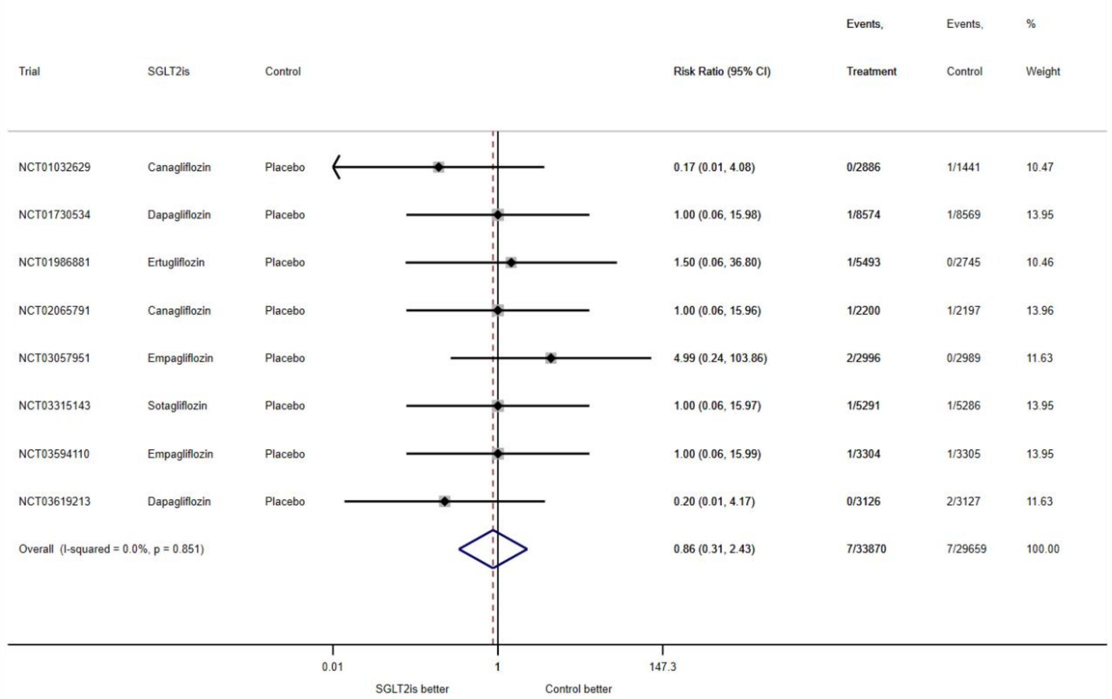

System 14-Metabolism And Nutrition Disorders-Figure S14 Meta-analysis of SGLT2is and Hypercalcaemia

Trial	SGLT2is	Control	Risk Ratio (95% CI)	Events, Treatment	Events, Control	% Weight
NCT00673231	Dapagliflozin	Placebo	0.97 (0.04, 23.77)	1/610	0/197	0.58
NCT01032629	Canagliflozin	Placebo	0.58 (0.20, 1.73)	7/2886	6/1441	5.00
NCT01042977	Dapagliflozin	Placebo	0.33 (0.01, 8.16)	0/482	1/482	0.58
NCT01131676	Empagliflozin	Placebo	0.28 (0.12, 0.68)	8/4687	14/2333	7.88
NCT01164501	Empagliflozin	Placebo	3.81 (0.18, 79.07)	2/419	0/319	0.64
NCT01177813	Empagliflozin	Sitagliptin or Placebo	0.34 (0.01, 8.25)	0/447	1/452	0.58
NCT01730534	Dapagliflozin	Placebo	0.67 (0.44, 1.02)	37/8574	55/8569	34.30
NCT01986881	Ertugliflozin	Placebo	0.44 (0.16, 1.20)	7/5493	8/2745	5.77
NCT01989754	Canagliflozin	Placebo	0.45 (0.16, 1.31)	5/2904	11/2903	5.32
NCT02065791	Canagliflozin	Placebo	0.09 (0.01, 0.70)	1/2200	11/2197	1.42
NCT03036124	Dapagliflozin	Placebo	0.60 (0.14, 2.51)	3/2368	5/2368	2.90
NCT03036150	Dapagliflozin	Placebo	0.35 (0.14, 0.89)	6/2149	17/2149	6.87
NCT03057951	Empagliflozin	Placebo	3.99 (0.45, 35.68)	4/2996	1/2989	1.23
NCT03057977	Empagliflozin	Placebo	0.20 (0.01, 4.16)	0/1863	2/1863	0.64
NCT03315143	Sotagliflozin	Placebo	0.43 (0.21, 0.91)	10/5291	23/5286	10.78
NCT03521934	Sotagliflozin	Placebo	0.50 (0.09, 2.75)	2/605	4/611	2.07
NCT03594110	Empagliflozin	Placebo	0.57 (0.24, 1.36)	8/3304	14/3305	7.88
NCT03619213	Dapagliflozin	Placebo	0.86 (0.29, 2.55)	6/3126	7/3127	4.99
NCT04157751	Empagliflozin	Placebo	0.34 (0.01, 8.27)	0/260	1/264	0.58
Overall (I-squared = 0.0%, p = 0.741)			0.53 (0.42, 0.68)	107/50664	181/43600	100.00

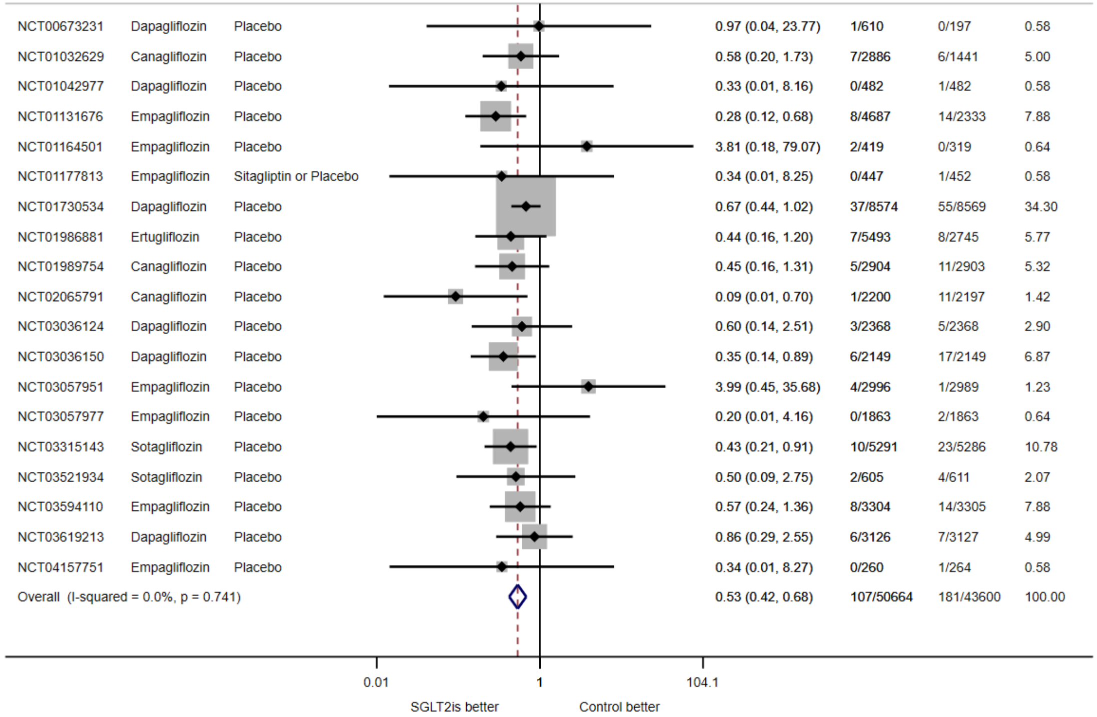

System 14-Metabolism And Nutrition Disorders-Figure S15 Meta-analysis of SGLT2is and Hyperglycaemia

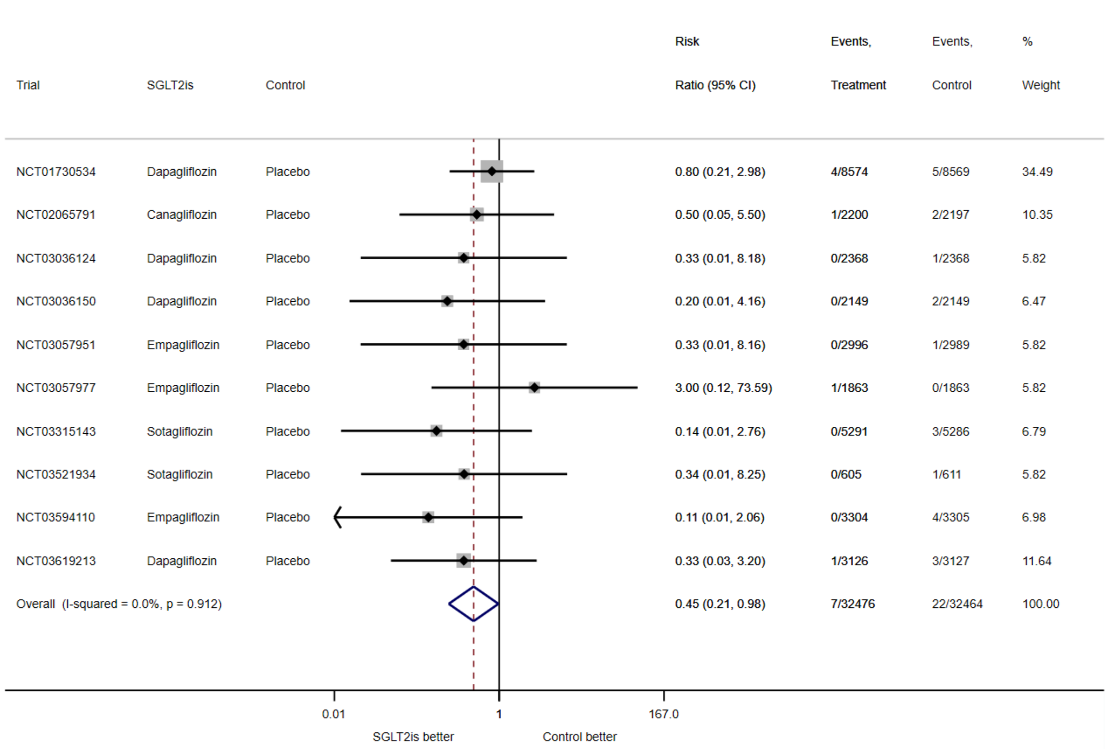

System 14-Metabolism And Nutrition Disorders-Figure S16 Meta-analysis of SGLT2is and Hyperglycaemic hyperosmolar nonketotic syndrome

Trial	SGLT2is	Control	Risk Ratio (95% CI)	Events, Treatment	Events, Control	% Weight
NCT01031680	Dapagliflozin	Placebo	3.01 (0.12, 73.77)	1/460	0/462	0.77
NCT01032629	Canagliflozin	Placebo	1.50 (0.30, 7.41)	6/2886	2/1441	3.08
NCT01131676	Empagliflozin	Placebo	0.50 (0.14, 1.72)	5/4687	5/2333	5.14
NCT01730534	Dapagliflozin	Placebo	1.00 (0.32, 3.10)	6/8574	6/8569	6.16
NCT01986881	Ertugliflozin	Placebo	1.00 (0.25, 3.99)	6/5493	3/2745	4.11
NCT01989754	Canagliflozin	Placebo	0.40 (0.08, 2.06)	2/2904	5/2903	2.93
NCT02065791	Canagliflozin	Placebo	1.00 (0.42, 2.39)	10/2200	10/2197	10.30
NCT03036124	Dapagliflozin	Placebo	0.40 (0.08, 2.06)	2/2368	5/2368	2.93
NCT03036150	Dapagliflozin	Placebo	0.50 (0.19, 1.33)	6/2149	12/2149	8.24
NCT03057951	Empagliflozin	Placebo	0.60 (0.29, 1.22)	12/2996	20/2989	15.46
NCT03057977	Empagliflozin	Placebo	0.22 (0.05, 1.03)	2/1863	9/1863	3.36
NCT03242252	Sotagliflozin	Placebo	0.99 (0.09, 10.83)	2/527	1/260	1.37
NCT03315143	Sotagliflozin	Placebo	1.27 (0.58, 2.80)	14/5291	11/5286	12.67
NCT03521934	Sotagliflozin	Placebo	0.25 (0.03, 2.25)	1/605	4/611	1.65
NCT03594110	Empagliflozin	Placebo	1.00 (0.48, 2.09)	14/3304	14/3305	14.42
NCT03619213	Dapagliflozin	Placebo	2.75 (0.88, 8.63)	11/3126	4/3127	6.03
NCT04157751	Empagliflozin	Placebo	2.03 (0.19, 22.26)	2/260	1/264	1.37
Overall (I-squared = 0.0%, p = 0.479)			0.84 (0.63, 1.11)	102/49693	112/42872	100.00

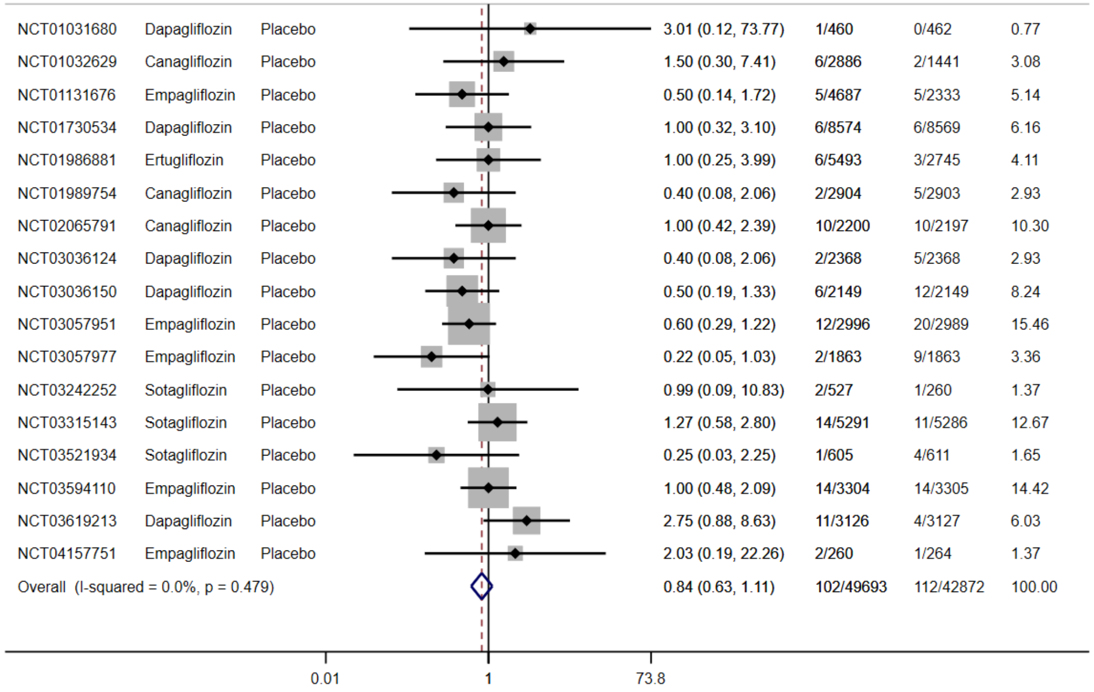

System 14-Metabolism And Nutrition Disorders-Figure S17 Meta-analysis of SGLT2is and Hyperkalaemia

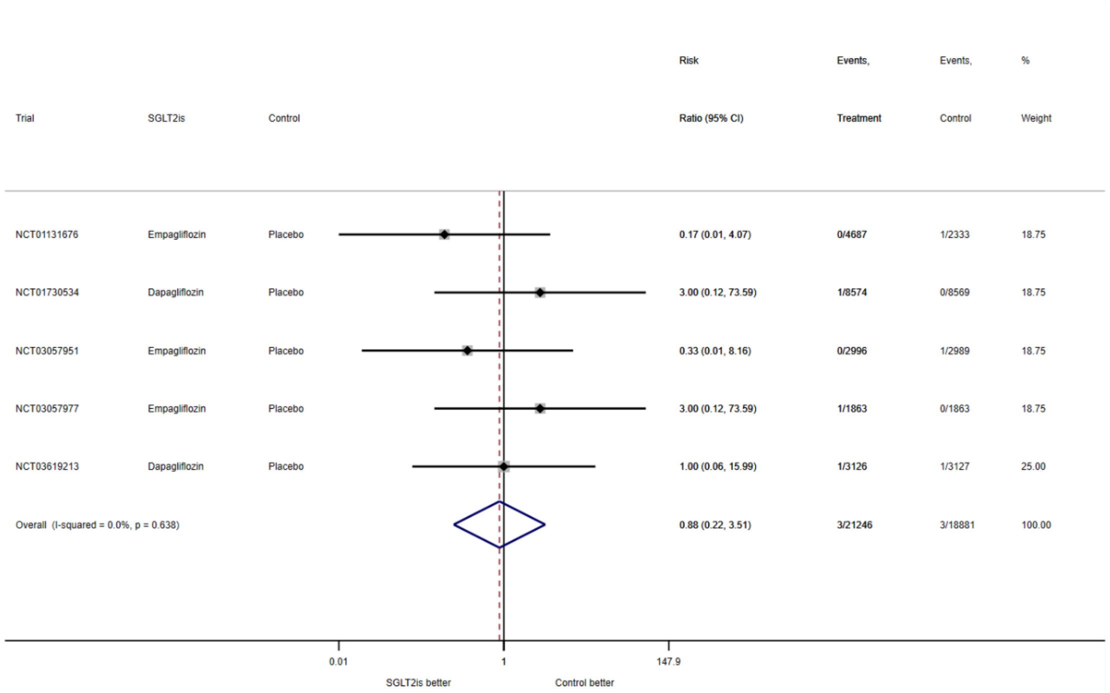

System 14-Metabolism And Nutrition Disorders-Figure S18 Meta-analysis of SGLT2is and Hypernatraemia

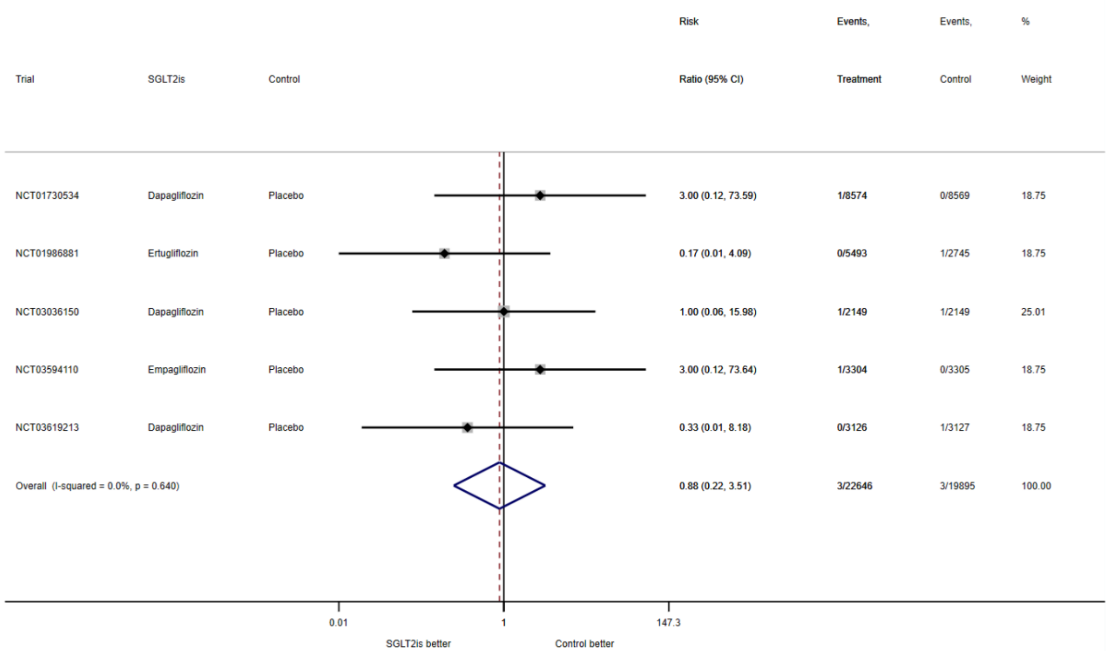

System 14-Metabolism And Nutrition Disorders-Figure S19 Meta-analysis of SGLT2is and Hypocalcaemia

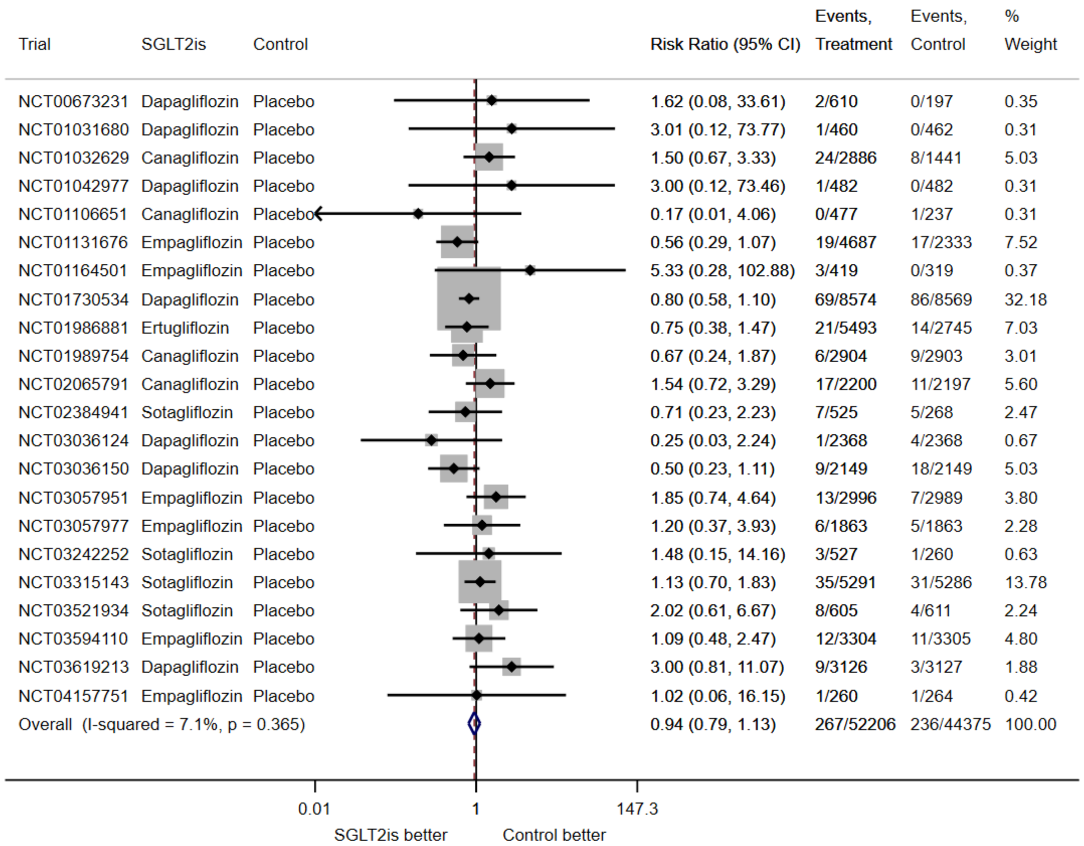

System 14-Metabolism And Nutrition Disorders-Figure S20 Meta-analysis of SGLT2is and Hypoglycaemia

Trial	SGLT2is	Control	Risk Ratio (95% CI)	Events, Treatment	Events, Control	% Weight
NCT01032629	Canagliflozin	Placebo	1.50 (0.06, 36.76)	1/2886	0/1441	3.37
NCT01131676	Empagliflozin	Placebo	0.17 (0.01, 4.07)	0/4687	1/2333	3.37
NCT01730534	Dapagliflozin	Placebo	1.25 (0.34, 4.65)	5/8574	4/8569	19.99
NCT01989754	Canagliflozin	Placebo	0.33 (0.01, 8.18)	0/2904	1/2903	3.37
NCT02065791	Canagliflozin	Placebo	0.14 (0.01, 2.76)	0/2200	3/2197	3.94
NCT03036124	Dapagliflozin	Placebo	5.00 (0.24, 104.09)	2/2368	0/2368	3.75
NCT03057951	Empagliflozin	Placebo	1.00 (0.29, 3.44)	5/2996	5/2989	22.52
NCT03057977	Empagliflozin	Placebo	1.50 (0.25, 8.97)	3/1863	2/1863	10.80
NCT03315143	Sotagliflozin	Placebo	5.00 (0.24, 104.02)	2/5291	0/5286	3.75
NCT03521934	Sotagliflozin	Placebo	0.34 (0.01, 8.25)	0/605	1/611	3.38
NCT03594110	Empagliflozin	Placebo	3.00 (0.12, 73.64)	1/3304	0/3305	3.37
NCT03619213	Dapagliflozin	Placebo	5.00 (1.10, 22.81)	10/3126	2/3127	15.00
NCT04157751	Empagliflozin	Placebo	0.34 (0.01, 8.27)	0/260	1/264	3.38
Overall (I-squared = 0.0%, p = 0.546)			1.29 (0.72, 2.32)	29/41064	20/37256	100.00

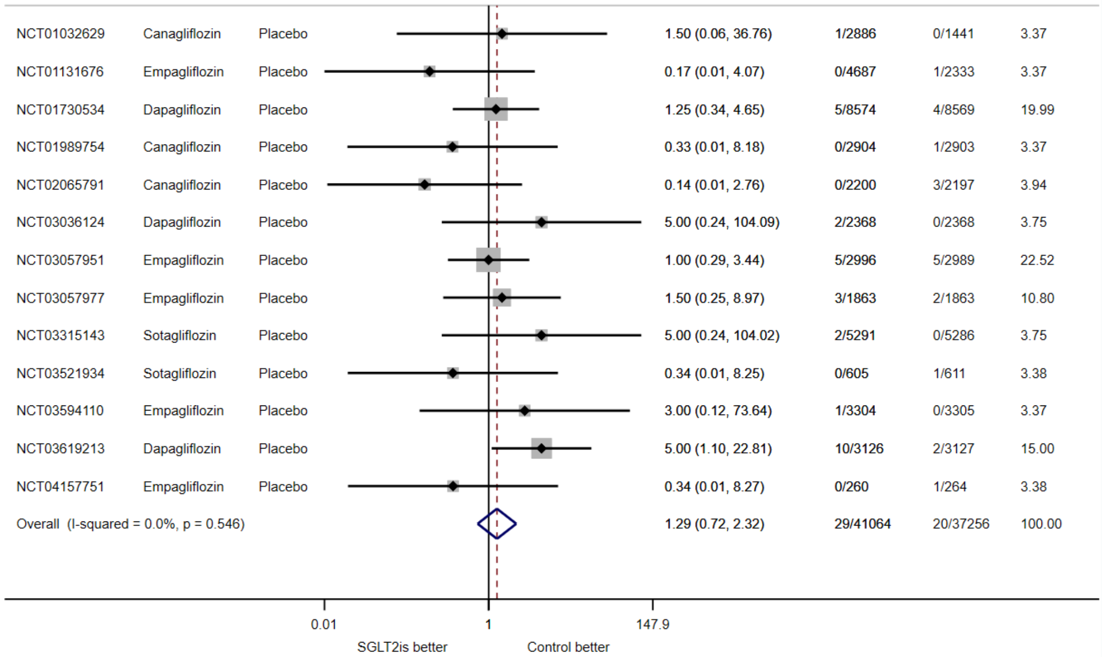

System 14-Metabolism And Nutrition Disorders-Figure S21 Meta-analysis of SGLT2is and Hypokalaemia

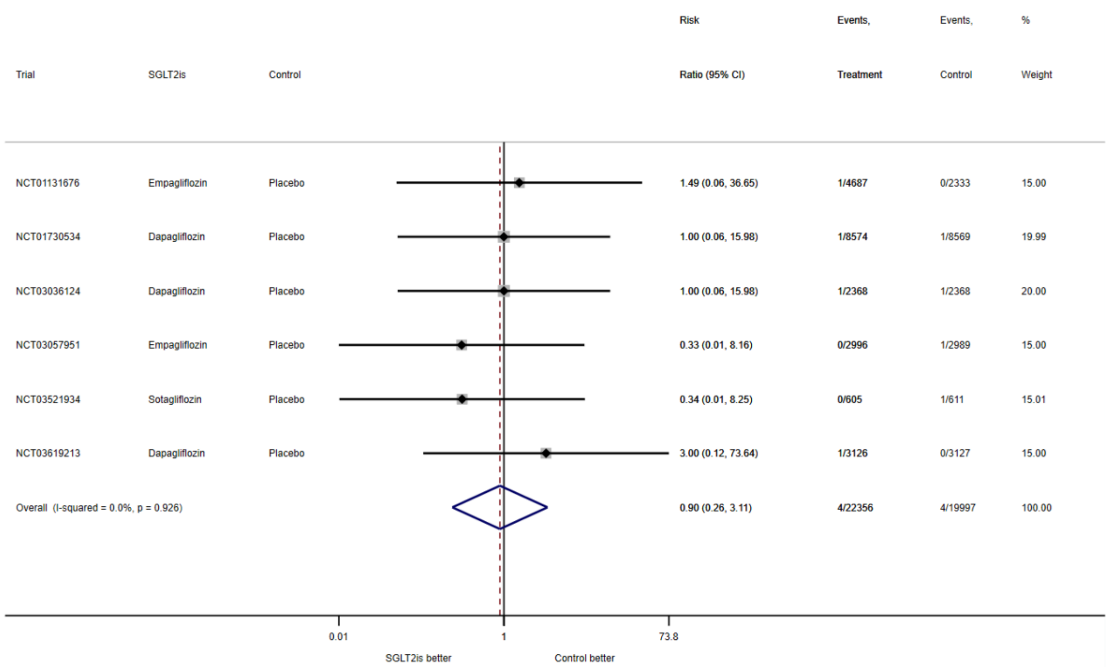

System 14-Metabolism And Nutrition Disorders-Figure S22 Meta-analysis of SGLT2is and Hypomagnesaemia

Trial	SGLT2is	Control	Risk Ratio (95% CI)	Events, Treatment	Events, Control	% Weight
NCT01032629	Canagliflozin	Placebo	0.50 (0.10, 2.47)	3/2886	3/1441	6.09
NCT01131676	Empagliflozin	Placebo	1.99 (0.22, 17.80)	4/4687	1/2333	3.24
NCT01730534	Dapagliflozin	Placebo	1.00 (0.40, 2.52)	9/8574	9/8569	18.25
NCT01986881	Ertugliflozin	Placebo	2.50 (0.12, 52.04)	2/5493	0/2745	1.69
NCT01989754	Canagliflozin	Placebo	0.75 (0.17, 3.35)	3/2904	4/2903	6.95
NCT02065791	Canagliflozin	Placebo	0.75 (0.17, 3.34)	3/2200	4/2197	6.95
NCT03036124	Dapagliflozin	Placebo	4.00 (0.45, 35.76)	4/2368	1/2368	3.24
NCT03036150	Dapagliflozin	Placebo	1.50 (0.25, 8.97)	3/2149	2/2149	4.87
NCT03057951	Empagliflozin	Placebo	0.30 (0.08, 1.09)	3/2996	10/2989	9.36
NCT03057977	Empagliflozin	Placebo	0.50 (0.05, 5.51)	1/1863	2/1863	2.70
NCT03242252	Sotagliflozin	Placebo	1.48 (0.06, 36.28)	1/527	0/260	1.52
NCT03315143	Sotagliflozin	Placebo	0.33 (0.09, 1.23)	3/5291	9/5286	9.12
NCT03521934	Sotagliflozin	Placebo	0.25 (0.03, 2.25)	1/605	4/611	3.25
NCT03594110	Empagliflozin	Placebo	0.60 (0.14, 2.51)	3/3304	5/3305	7.60
NCT03619213	Dapagliflozin	Placebo	0.88 (0.32, 2.41)	7/3126	8/3127	15.16
Overall (I-squared = 0.0%, p = 0.787)			0.73 (0.49, 1.09)	50/48973	62/42146	100.00

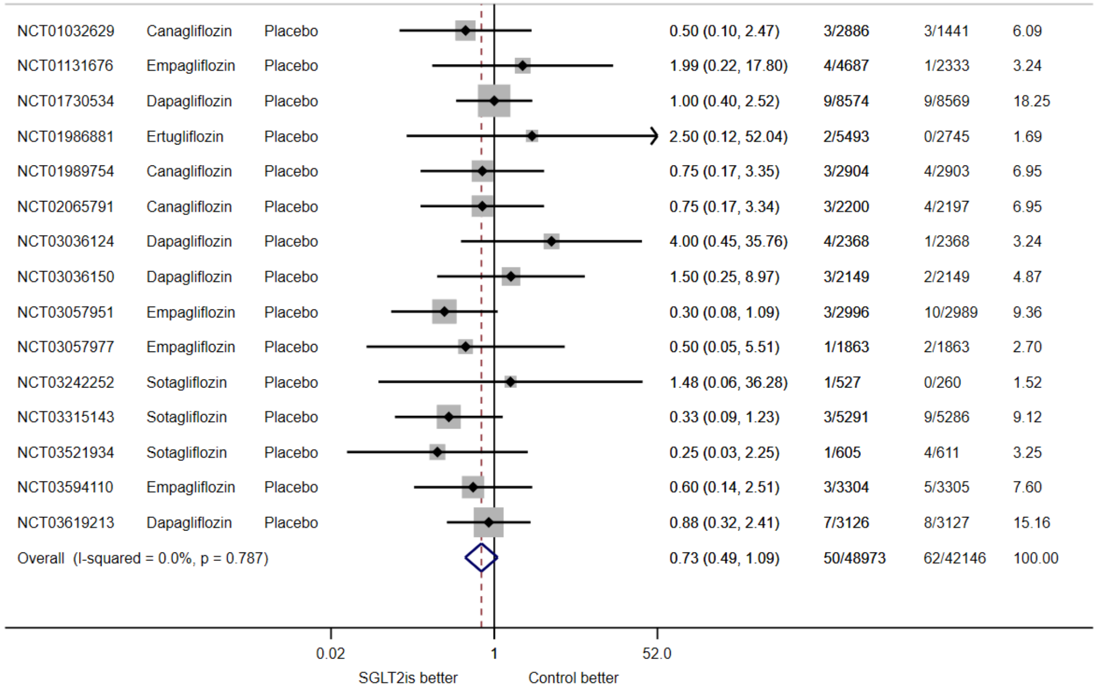

System 14-Metabolism And Nutrition Disorders-Figure S23 Meta-analysis of SGLT2is and Hyponatraemia

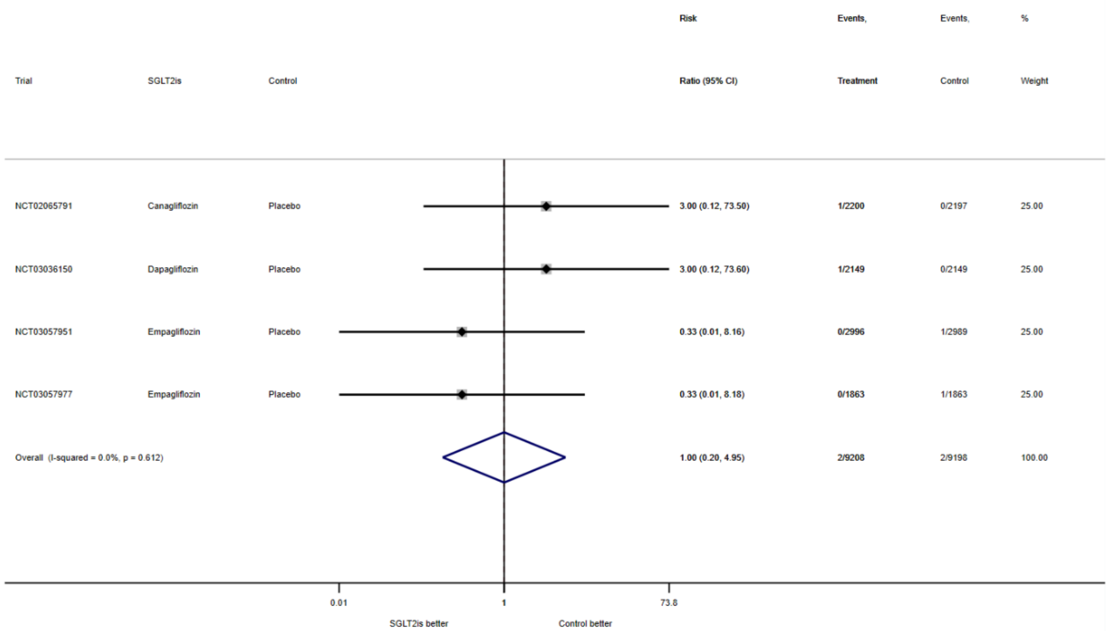

System 14-Metabolism And Nutrition Disorders-Figure S24 Meta-analysis of SGLT2is and Hypophagia

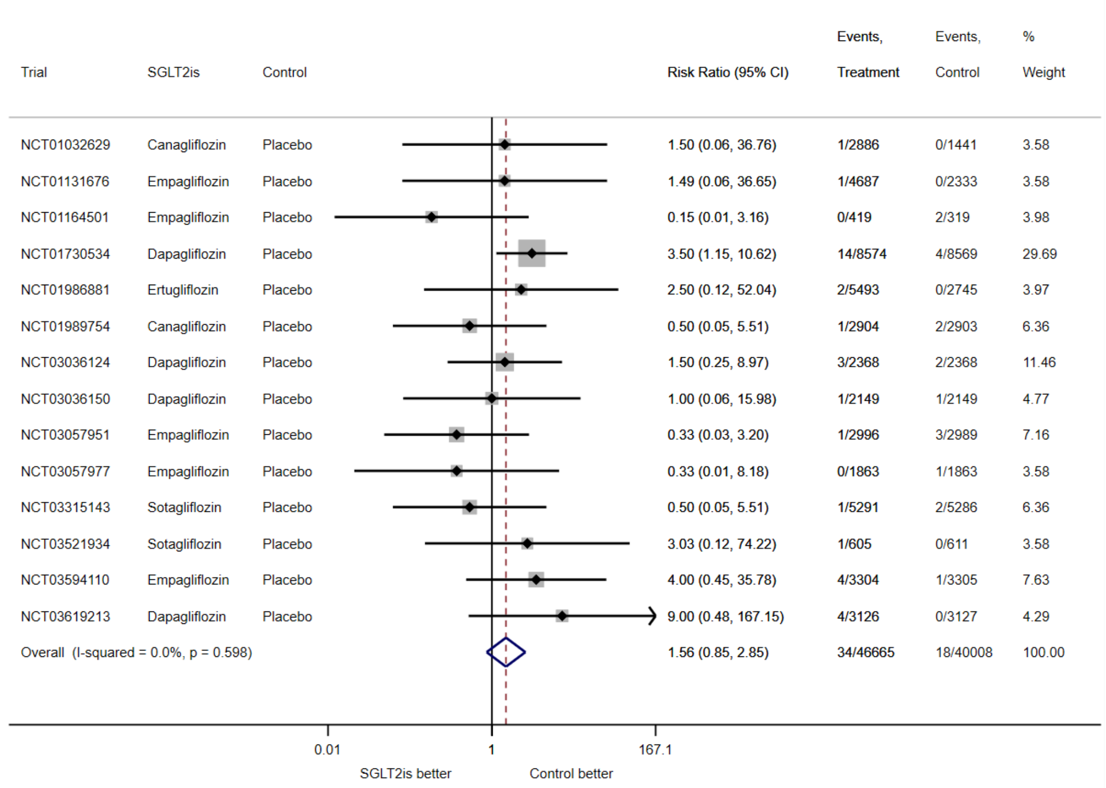

System 14-Metabolism And Nutrition Disorders-Figure S25 Meta-analysis of SGLT2is and Hypovolaemia
