# Supplementary material for: A comprehensive meta-analysis on safety outcomes reveals the novel potentials of SGLT2is, especially preventing respiratory diseases
Source: Front Endocrinol (Lausanne). 2024 Apr 29;15:1376446. doi: 10.3389/fendo.2024.1376446 (PMC11089104; doi:10.3389/fendo.2024.1376446)
Supplement: Supplementary file 4 [file DataSheet_4.pdf]

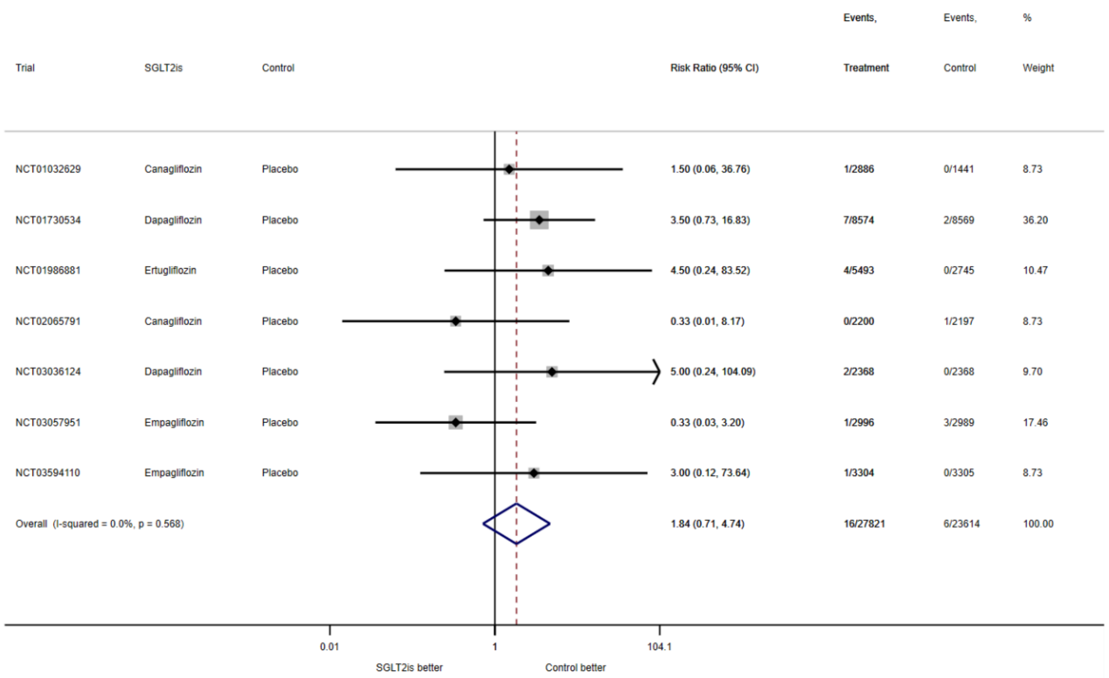

System 14-Metabolism And Nutrition Disorders-Figure S26 Meta-analysis of SGLT2is and Ketoacidosis

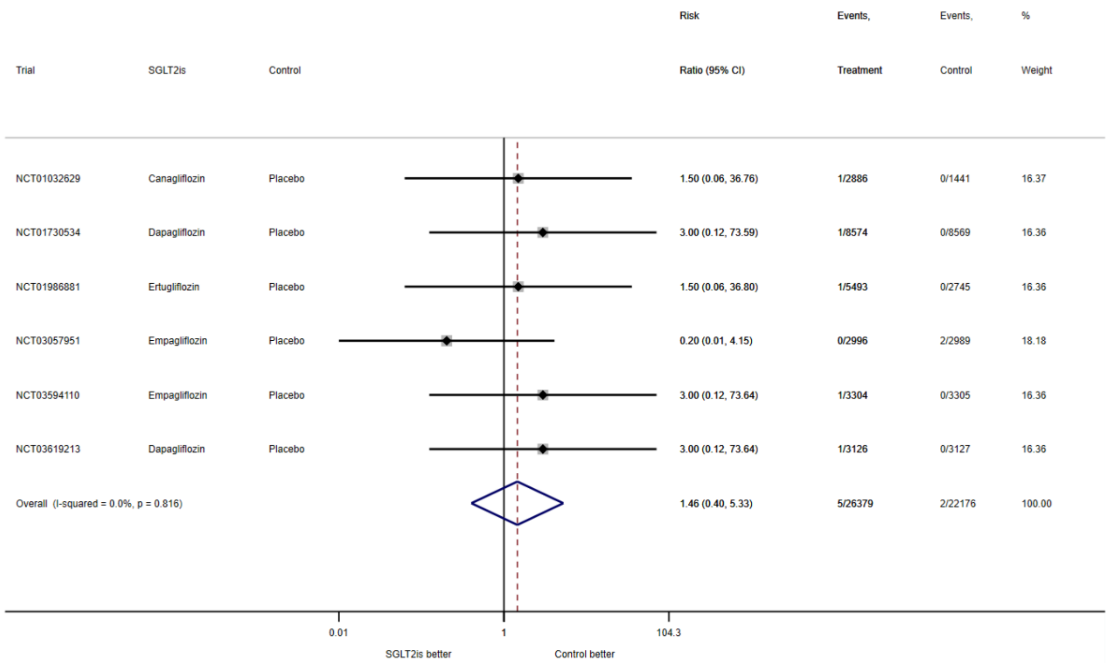

System 14-Metabolism And Nutrition Disorders-Figure S27 Meta-analysis of SGLT2is and Ketosis

Trial	SGLT2is	Control	Risk Ratio (95% CI)	Events, Treatment	Events, Control	% Weight
NCT01032629	Canagliflozin	Placebo	0.50 (0.03, 7.98)	1/2886	1/1441	6.48
NCT01131676	Empagliflozin	Placebo	1.49 (0.06, 36.65)	1/4687	0/2333	4.86
NCT01730534	Dapagliflozin	Placebo	1.33 (0.30, 5.95)	4/8574	3/8569	22.22
NCT01986881	Ertugliflozin	Placebo	0.50 (0.03, 7.99)	1/5493	1/2745	6.48
NCT01989754	Canagliflozin	Placebo	5.00 (0.24, 104.07)	2/2904	0/2903	5.40
NCT02065791	Canagliflozin	Placebo	3.00 (0.12, 73.50)	1/2200	0/2197	4.86
NCT03036124	Dapagliflozin	Placebo	0.33 (0.01, 8.18)	0/2368	1/2368	4.86
NCT03036150	Dapagliflozin	Placebo	1.00 (0.06, 15.98)	1/2149	1/2149	6.48
NCT03057951	Empagliflozin	Placebo	0.33 (0.03, 3.20)	1/2996	3/2989	9.72
NCT03057977	Empagliflozin	Placebo	5.00 (0.24, 104.08)	2/1863	0/1863	5.40
NCT03315143	Sotagliflozin	Placebo	1.00 (0.06, 15.97)	1/5291	1/5286	6.48
NCT03521934	Sotagliflozin	Placebo	3.03 (0.12, 74.22)	1/605	0/611	4.86
NCT03619213	Dapagliflozin	Placebo	1.00 (0.06, 15.99)	1/3126	1/3127	6.48
NCT04350593	Dapagliflozin	Placebo	5.02 (0.24, 104.44)	2/613	0/616	5.41
Overall (I-squared = 0.0%, p = 0.950)			1.22 (0.60, 2.48)	19/45755	12/39197	100.00

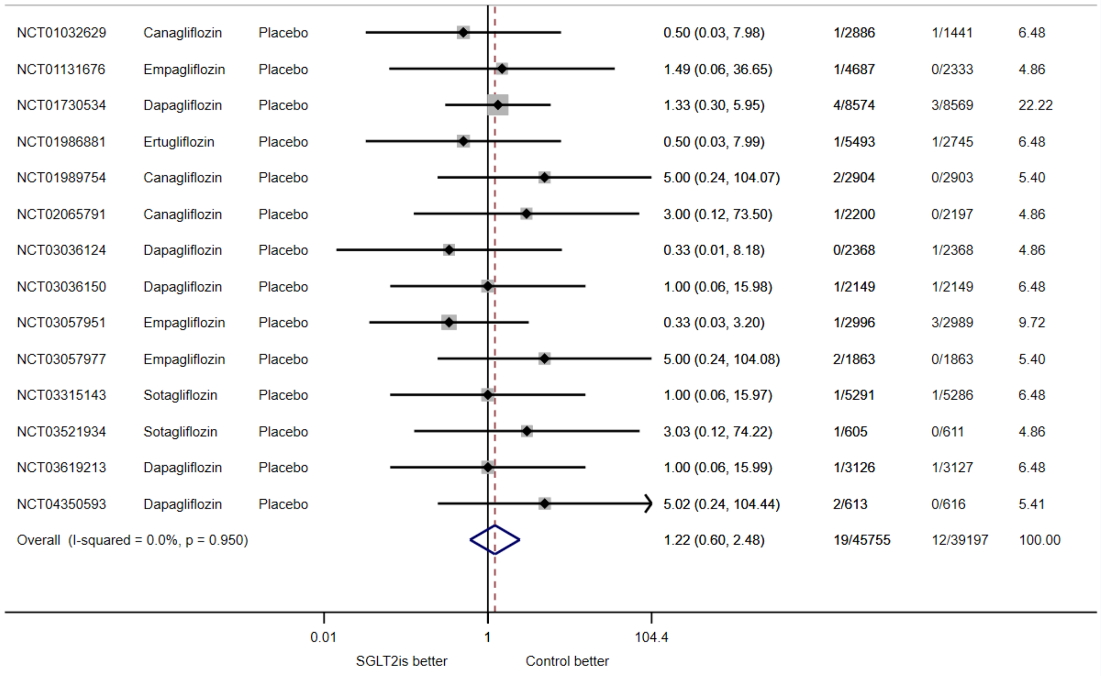

System 14-Metabolism And Nutrition Disorders-Figure S28 Meta-analysis of SGLT2is and Lactic acidosis

Trial	SGLT2is	Control	Risk Ratio (95% CI)	Events, Treatment	Events, Control	% Weight
NCT01032629	Canagliflozin	Placebo	0.75 (0.13, 4.48)	3/2886	2/1441	7.58
NCT01131676	Empagliflozin	Placebo	1.00 (0.09, 10.97)	2/4687	1/2333	4.21
NCT01730534	Dapagliflozin	Placebo	1.00 (0.20, 4.95)	3/8574	3/8569	9.46
NCT01986881	Ertugliflozin	Placebo	0.50 (0.03, 7.99)	1/5493	1/2745	3.15
NCT01989754	Canagliflozin	Placebo	1.00 (0.06, 15.97)	1/2904	1/2903	3.15
NCT02065791	Canagliflozin	Placebo	0.33 (0.07, 1.65)	2/2200	6/2197	9.47
NCT03036150	Dapagliflozin	Placebo	1.00 (0.06, 15.98)	1/2149	1/2149	3.15
NCT03057951	Empagliflozin	Placebo	0.54 (0.20, 1.47)	6/2996	11/2989	24.54
NCT03057977	Empagliflozin	Placebo	1.33 (0.30, 5.95)	4/1863	3/1863	10.83
NCT03315143	Sotagliflozin	Placebo	13.99 (1.84, 106.33)	14/5291	1/5286	5.89
NCT03521934	Sotagliflozin	Placebo	3.03 (0.12, 74.22)	1/605	0/611	2.37
NCT03594110	Empagliflozin	Placebo	6.00 (0.72, 49.82)	6/3304	1/3305	5.41
NCT03619213	Dapagliflozin	Placebo	0.20 (0.02, 1.71)	1/3126	5/3127	5.26
NCT04157751	Empagliflozin	Placebo	1.02 (0.06, 16.15)	1/260	1/264	3.16
NCT04350593	Dapagliflozin	Placebo	3.01 (0.12, 73.86)	1/613	0/616	2.37
Overall (I-squared = 12.7%, p = 0.311)			0.96 (0.58, 1.56)	47/46951	37/40398	100.00

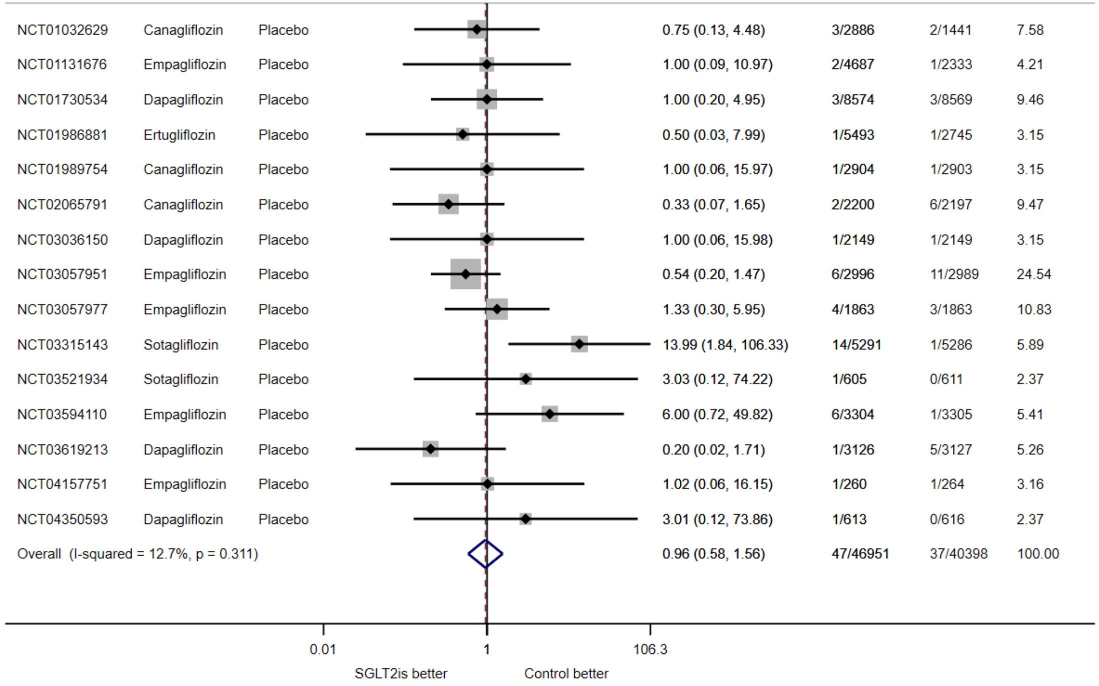

System 14-Metabolism And Nutrition Disorders-Figure S29 Meta-analysis of SGLT2is and Metabolic acidosis

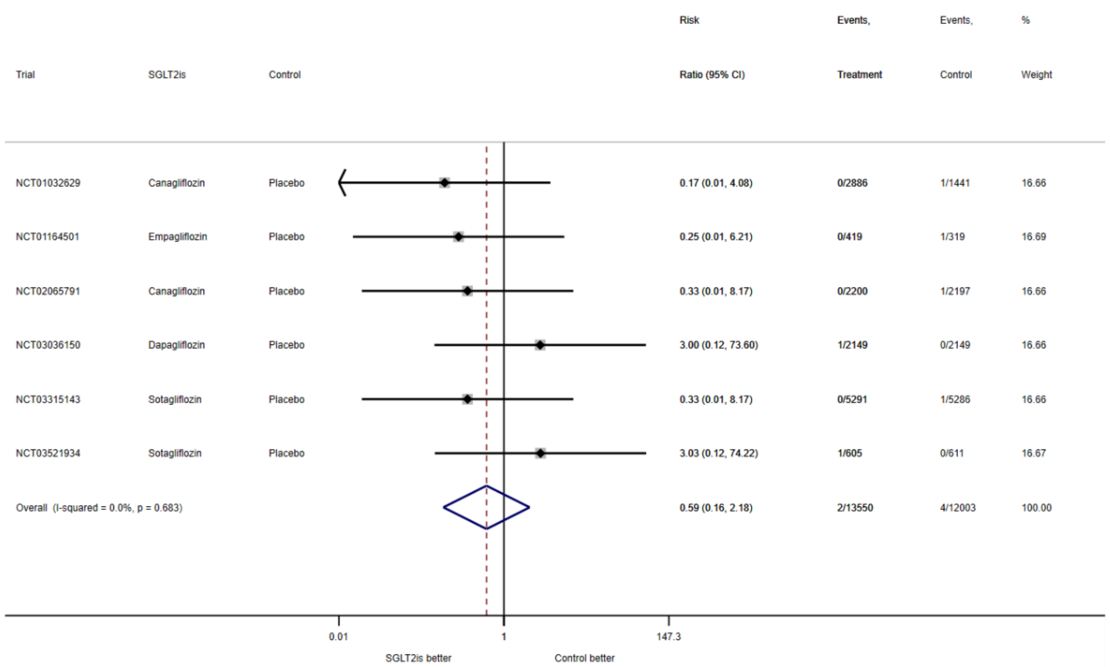

System 14-Metabolism And Nutrition Disorders-Figure S30 Meta-analysis of SGLT2is and Metabolic disorder

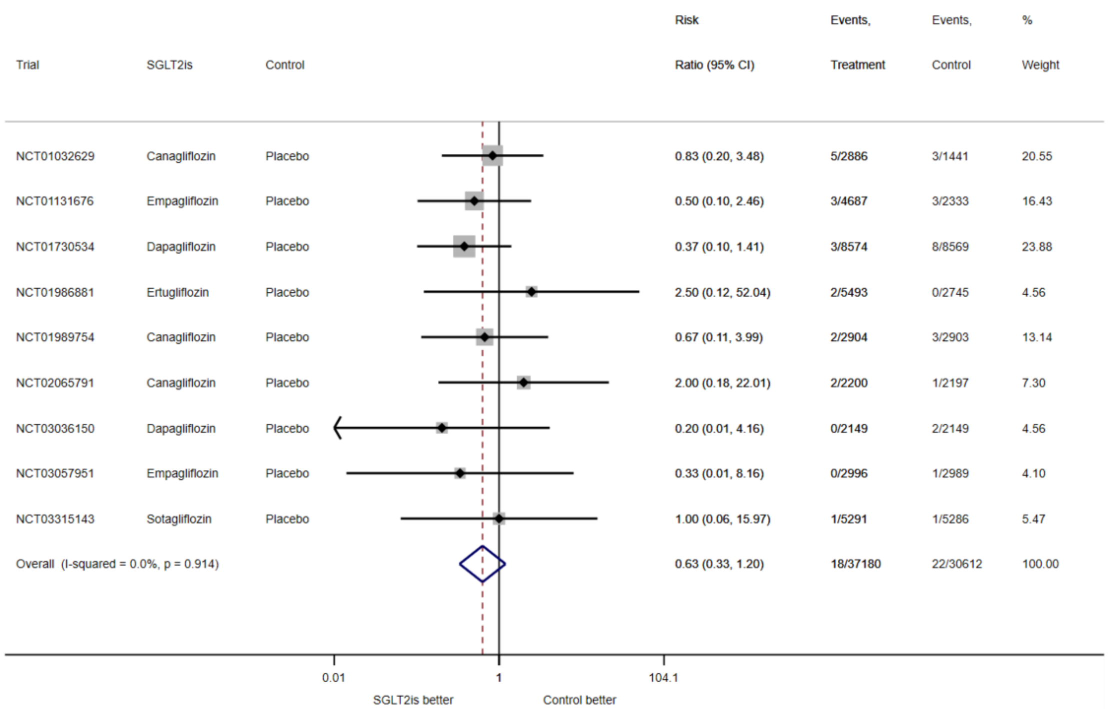

System 14-Metabolism And Nutrition Disorders-Figure S31 Meta-analysis of SGLT2is and Obesity

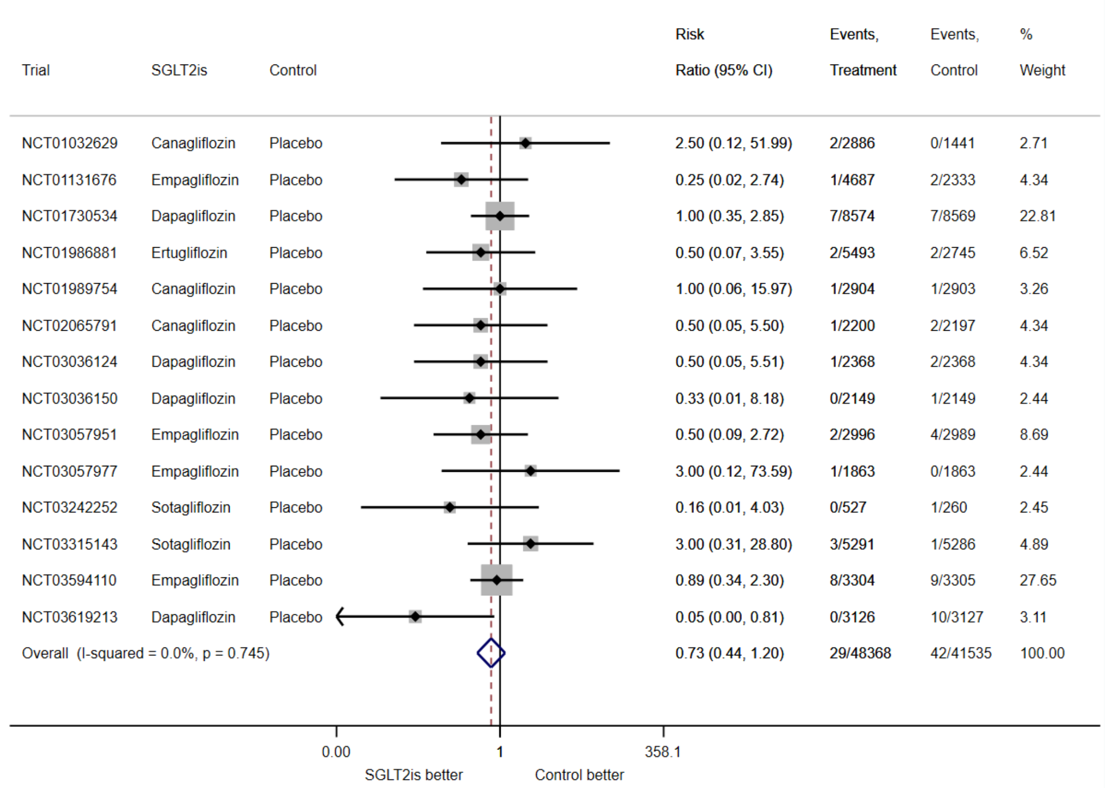

System 14-Metabolism And Nutrition Disorders-Figure S32 Meta-analysis of SGLT2is and Type 2 diabetes mellitus

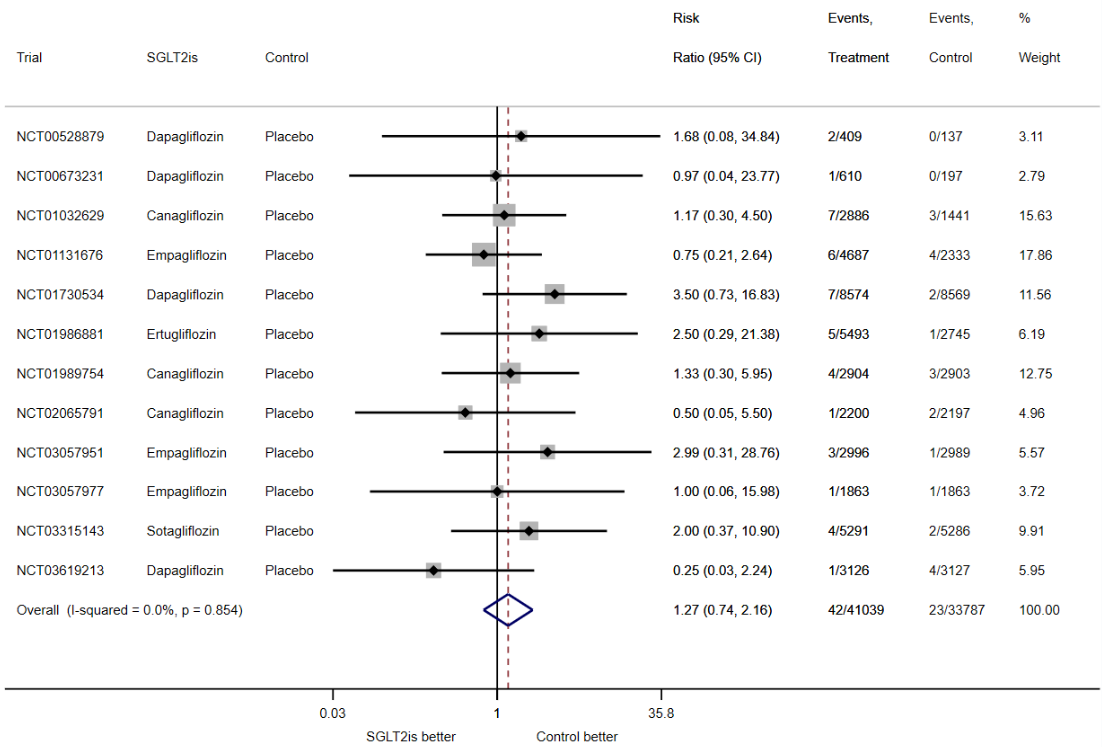

System 15-Musculoskeletal And Connective Tissue Disorders-Figure S1 Meta-analysis of SGLT2is and Arthralgia

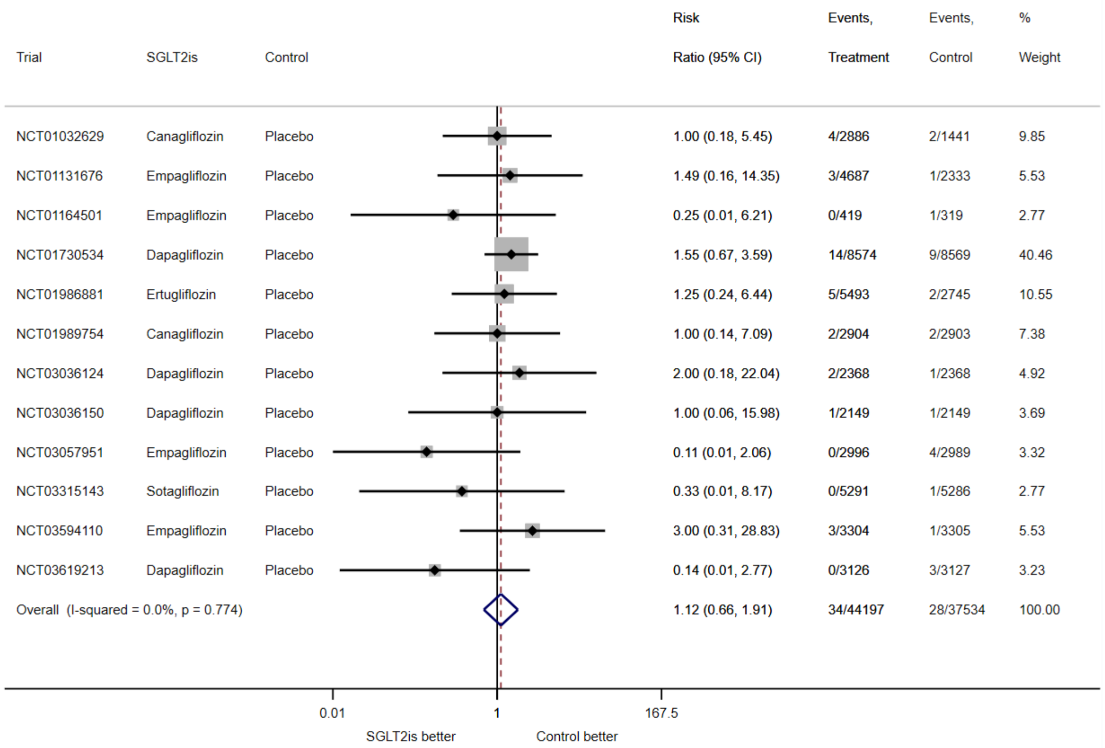

System 15-Musculoskeletal And Connective Tissue Disorders-Figure S2 Meta-analysis of SGLT2is and Arthritis

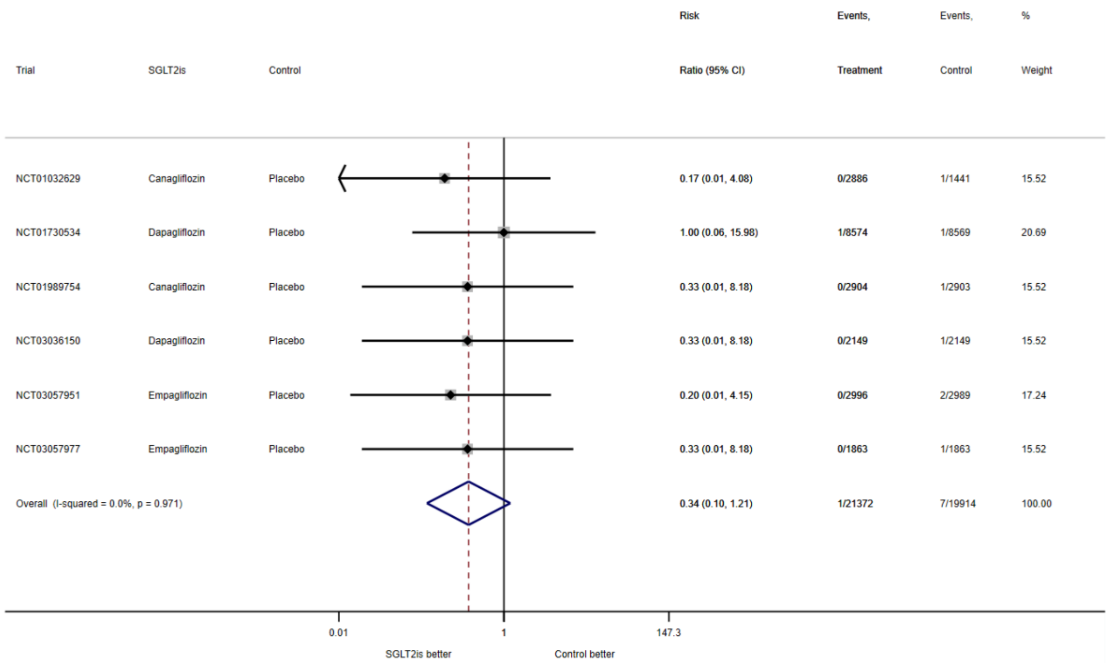

System 15-Musculoskeletal And Connective Tissue Disorders-Figure S3 Meta-analysis of SGLT2is and Arthropathy

Trial	SGLT2is	Control	Risk Ratio (95% CI)	Events, Treatment	Events, Control	% Weight
NCT00673231	Dapagliflozin	Placebo	0.32 (0.02, 5.14)	1/610	1/197	1.92
NCT01032629	Canagliflozin	Placebo	1.50 (0.41, 5.52)	9/2886	3/1441	8.65
NCT01042977	Dapagliflozin	Placebo	0.20 (0.01, 4.16)	0/482	2/483	1.60
NCT01131676	Empagliflozin	Placebo	0.61 (0.25, 1.47)	11/4687	9/2333	19.04
NCT01164501	Empagliflozin	Placebo	0.25 (0.01, 6.21)	0/419	1/319	1.44
NCT01730534	Dapagliflozin	Placebo	0.56 (0.19, 1.66)	5/8574	9/8569	12.33
NCT01986881	Ertugliflozin	Placebo	2.50 (0.29, 21.38)	5/5493	1/2745	3.20
NCT01989754	Canagliflozin	Placebo	0.18 (0.04, 0.82)	2/2904	11/2903	6.50
NCT02065791	Canagliflozin	Placebo	2.00 (0.18, 22.01)	2/2200	1/2197	2.56
NCT02384941	Sotagliflozin	Placebo	1.53 (0.06, 37.53)	1/525	0/268	1.44
NCT03036124	Dapagliflozin	Placebo	0.40 (0.08, 2.06)	2/2368	5/2368	5.48
NCT03036150	Dapagliflozin	Placebo	0.33 (0.03, 3.20)	1/2149	3/2149	2.88
NCT03057951	Empagliflozin	Placebo	0.37 (0.10, 1.41)	3/2996	8/2989	8.38
NCT03057977	Empagliflozin	Placebo	2.00 (0.18, 22.04)	2/1863	1/1863	2.56
NCT03315143	Sotagliflozin	Placebo	1.25 (0.34, 4.65)	5/5291	4/5286	8.53
NCT03521934	Sotagliflozin	Placebo	3.03 (0.12, 74.22)	1/605	0/611	1.44
NCT03594110	Empagliflozin	Placebo	0.40 (0.08, 2.06)	2/3304	5/3305	5.48
NCT03619213	Dapagliflozin	Placebo	0.75 (0.17, 3.35)	3/3126	4/3127	6.58
Overall (I-squared = 0.0%, p = 0.763)			0.65 (0.44, 0.95)	55/50482	68/43153	100.00

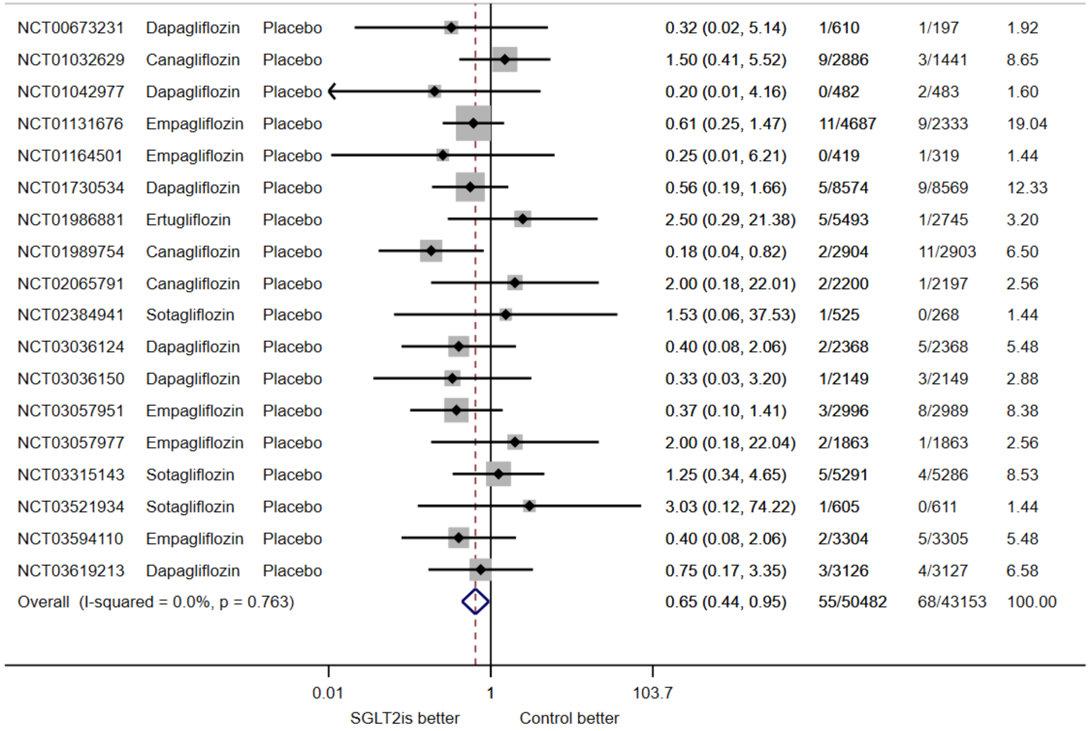

System 15-Musculoskeletal And Connective Tissue Disorders-Figure S4 Meta-analysis of SGLT2is and Back pain

Trial	SGLT2is	Control	Risk Ratio (95% CI)	Events, Treatment	Events, Control	% Weight
NCT01032629	Canagliflozin	Placebo	3.50 (0.18, 67.64)	3/2886	0/1441	7.42
NCT01131676	Empagliflozin	Placebo	1.49 (0.06, 36.65)	1/4687	0/2333	6.36
NCT01730534	Dapagliflozin	Placebo	1.67 (0.40, 6.97)	5/8574	3/8569	31.80
NCT01986881	Ertugliflozin	Placebo	0.17 (0.02, 1.60)	1/5493	3/2745	12.72
NCT01989754	Canagliflozin	Placebo	0.33 (0.01, 8.18)	0/2904	1/2903	6.36
NCT03036150	Dapagliflozin	Placebo	3.00 (0.12, 73.60)	1/2149	0/2149	6.36
NCT03057951	Empagliflozin	Placebo	4.99 (0.24, 103.86)	2/2996	0/2989	7.07
NCT03315143	Sotagliflozin	Placebo	5.00 (0.24, 104.02)	2/5291	0/5286	7.07
NCT03594110	Empagliflozin	Placebo	1.00 (0.06, 15.99)	1/3304	1/3305	8.48
NCT03619213	Dapagliflozin	Placebo	0.33 (0.01, 8.18)	0/3126	1/3127	6.36
Overall (I-squared = 0.0%, p = 0.648)			1.23 (0.55, 2.77)	16/41410	9/34847	100.00

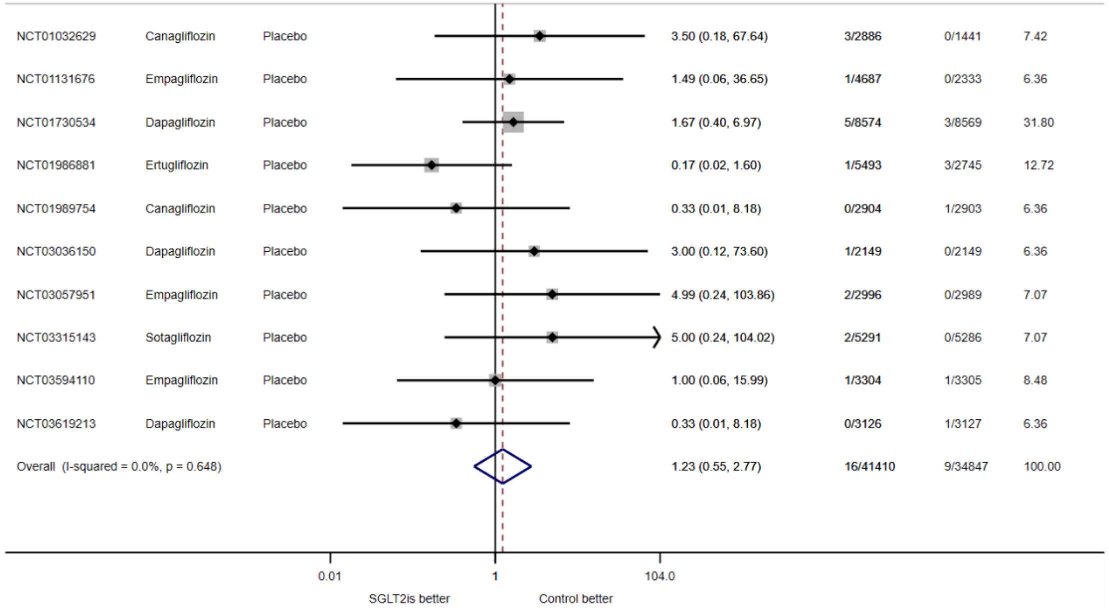

System 15-Musculoskeletal And Connective Tissue Disorders-Figure S5 Meta-analysis of SGLT2is and Bursitis

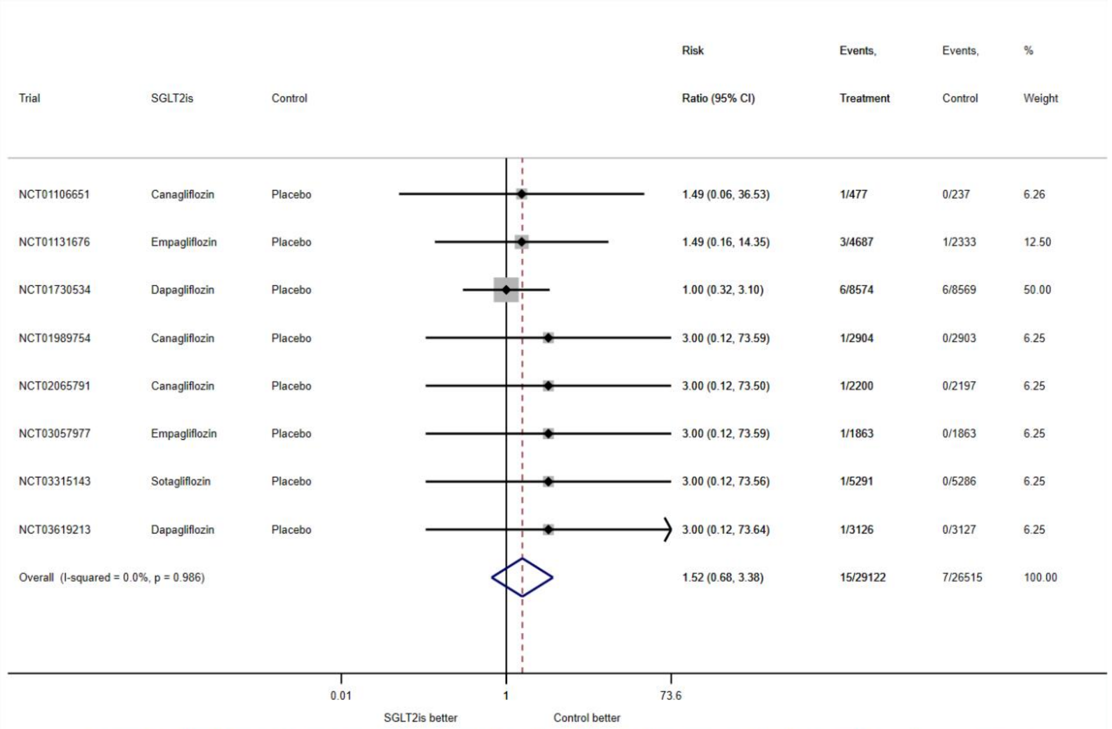

System 15-Musculoskeletal And Connective Tissue Disorders-Figure S6 Meta-analysis of SGLT2is and Cervical spinal stenosis

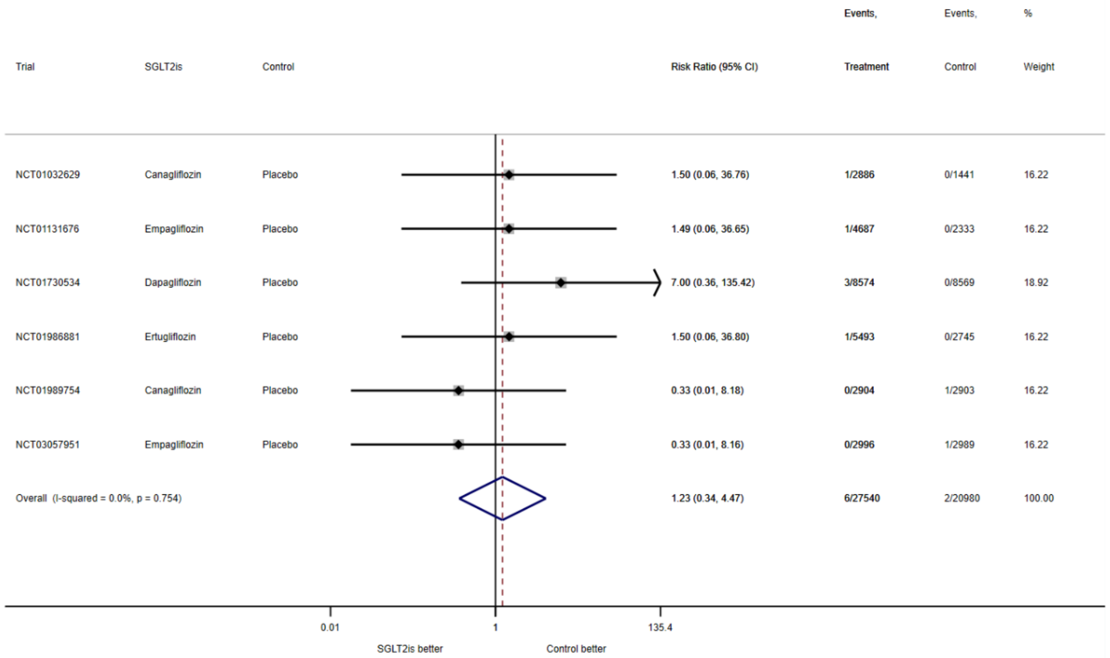

System 15-Musculoskeletal And Connective Tissue Disorders-Figure S7 Meta-analysis of SGLT2is and Costochondritis

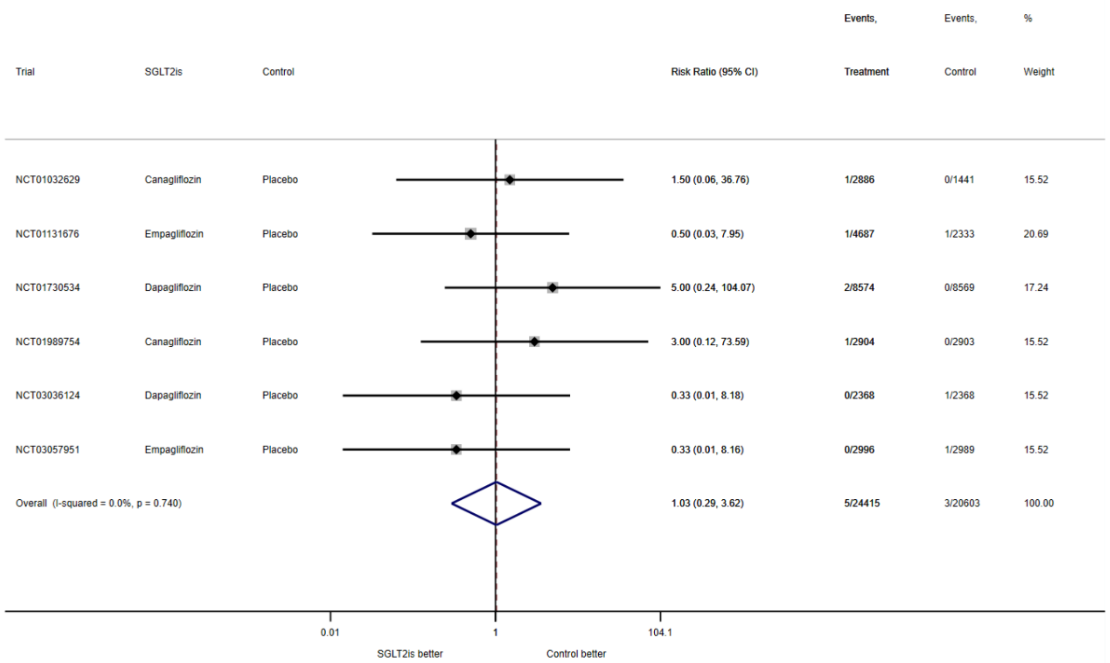

System 15-Musculoskeletal And Connective Tissue Disorders-Figure S8 Meta-analysis of SGLT2is and Dupuytren's contracture

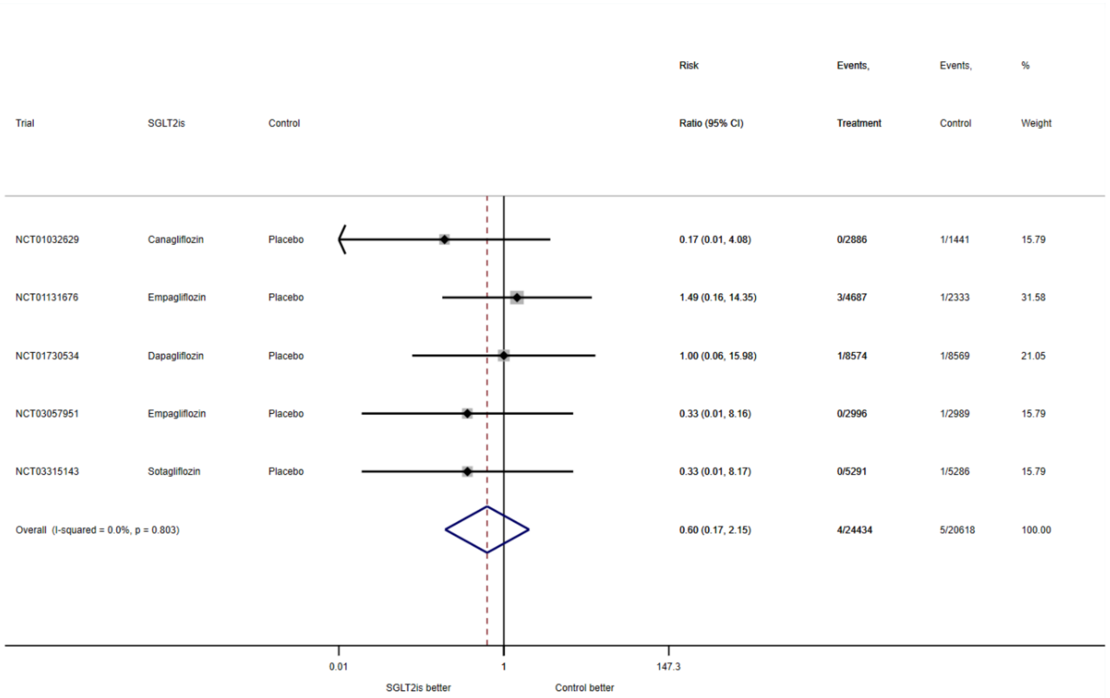

System 15-Musculoskeletal And Connective Tissue Disorders-Figure S9 Meta-analysis of SGLT2is and Flank pain

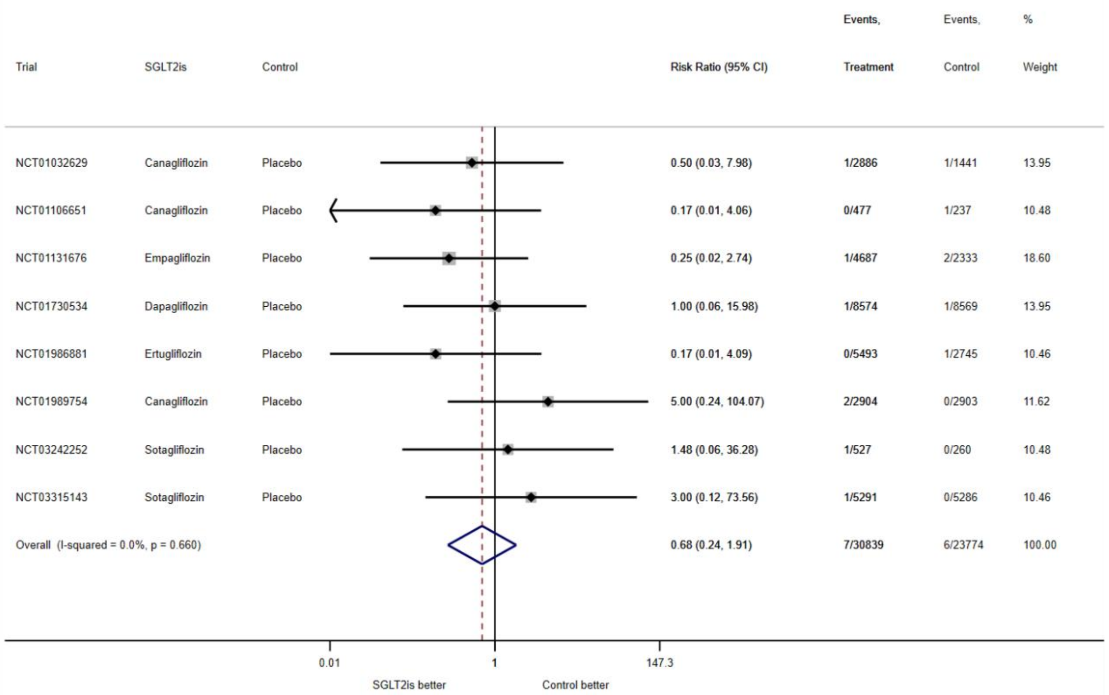

System 15-Musculoskeletal And Connective Tissue Disorders-Figure S10 Meta-analysis of SGLT2is and Foot deformity

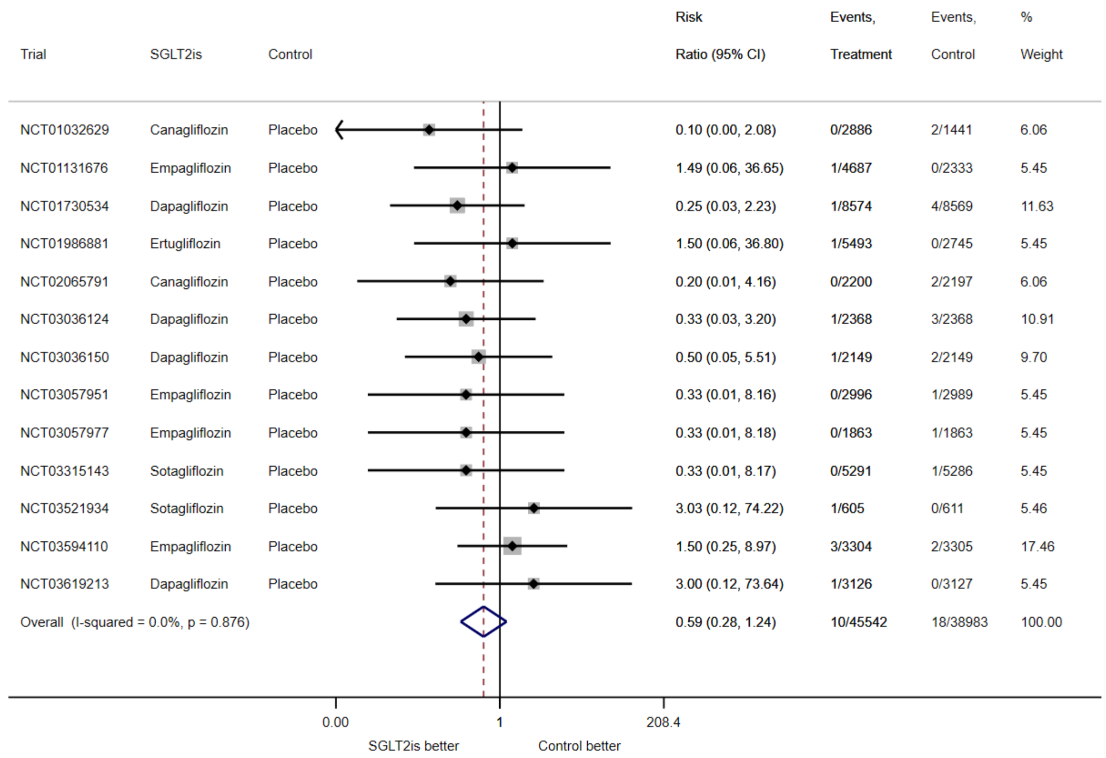

System 15-Musculoskeletal And Connective Tissue Disorders-Figure S11 Meta-analysis of SGLT2is and Gouty arthritis

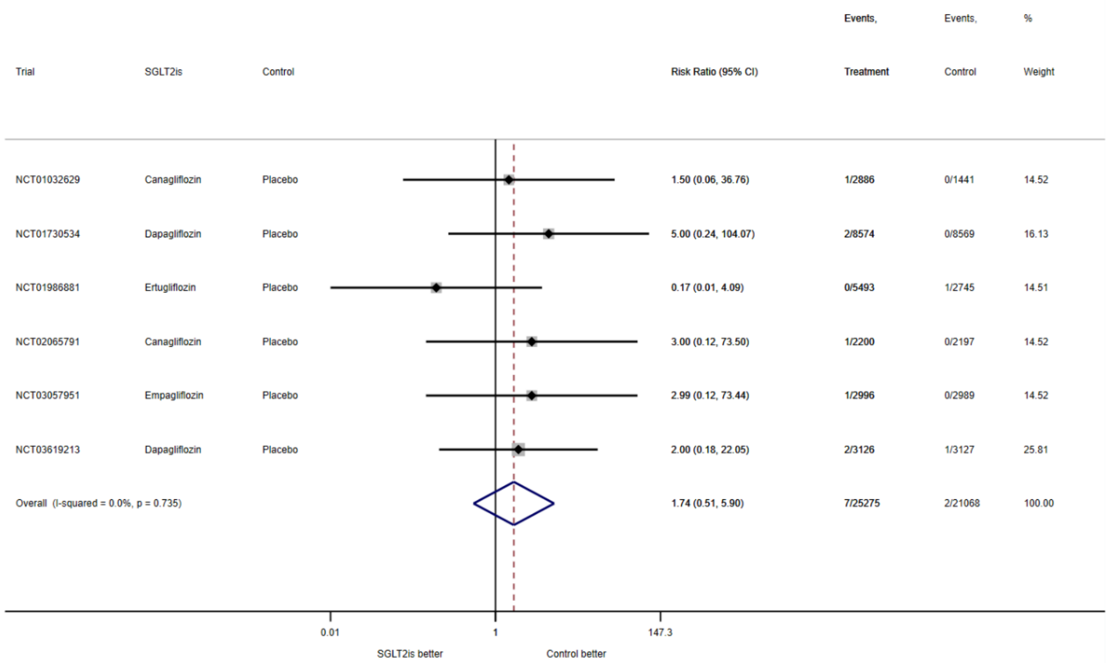

System 15-Musculoskeletal And Connective Tissue Disorders-Figure S12 Meta-analysis of SGLT2is and Haemarthrosis

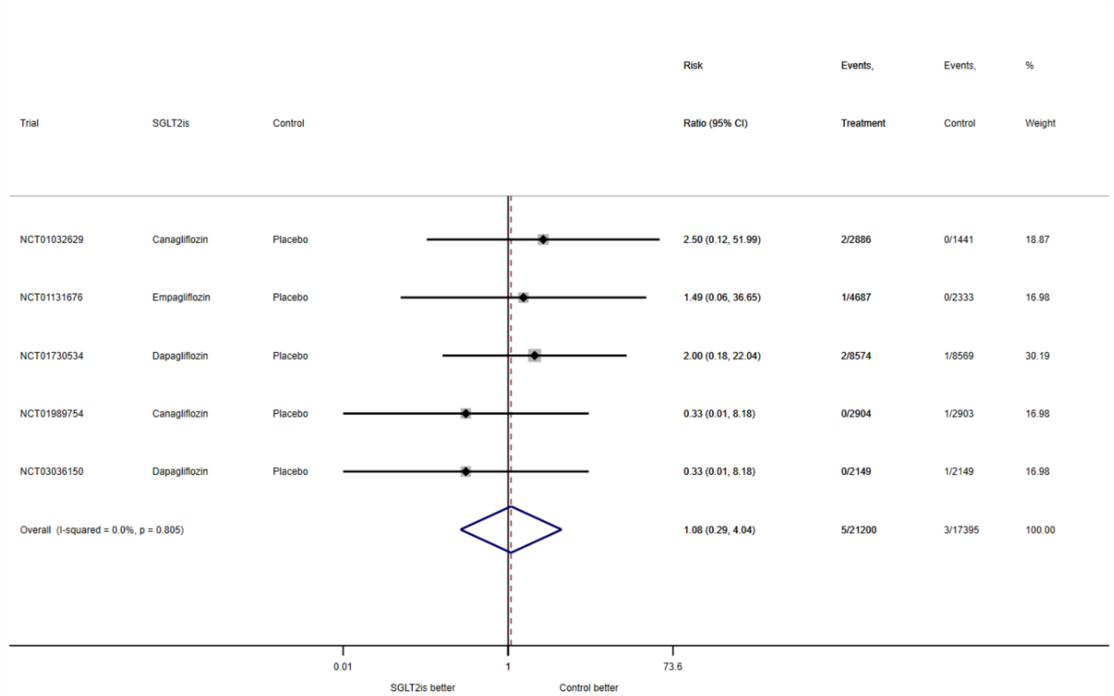

System 15-Musculoskeletal And Connective Tissue Disorders-Figure S13 Meta-analysis of SGLT2is and Intervertebral disc compression

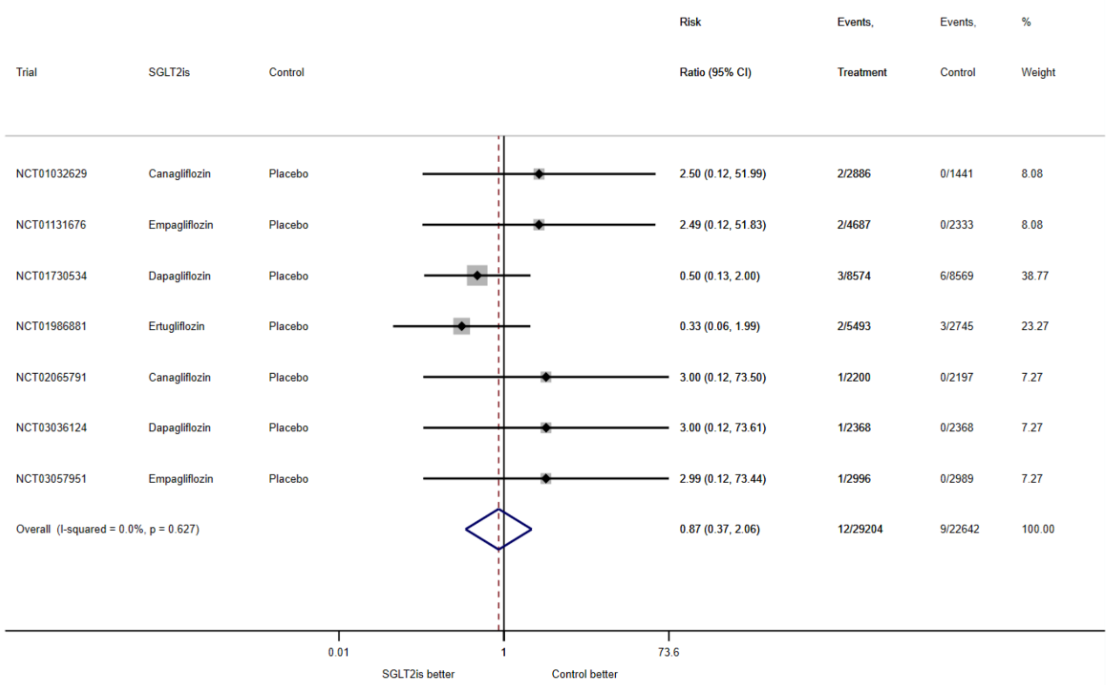

System 15-Musculoskeletal And Connective Tissue Disorders-Figure S14 Meta-analysis of SGLT2is and Intervertebral disc degeneration

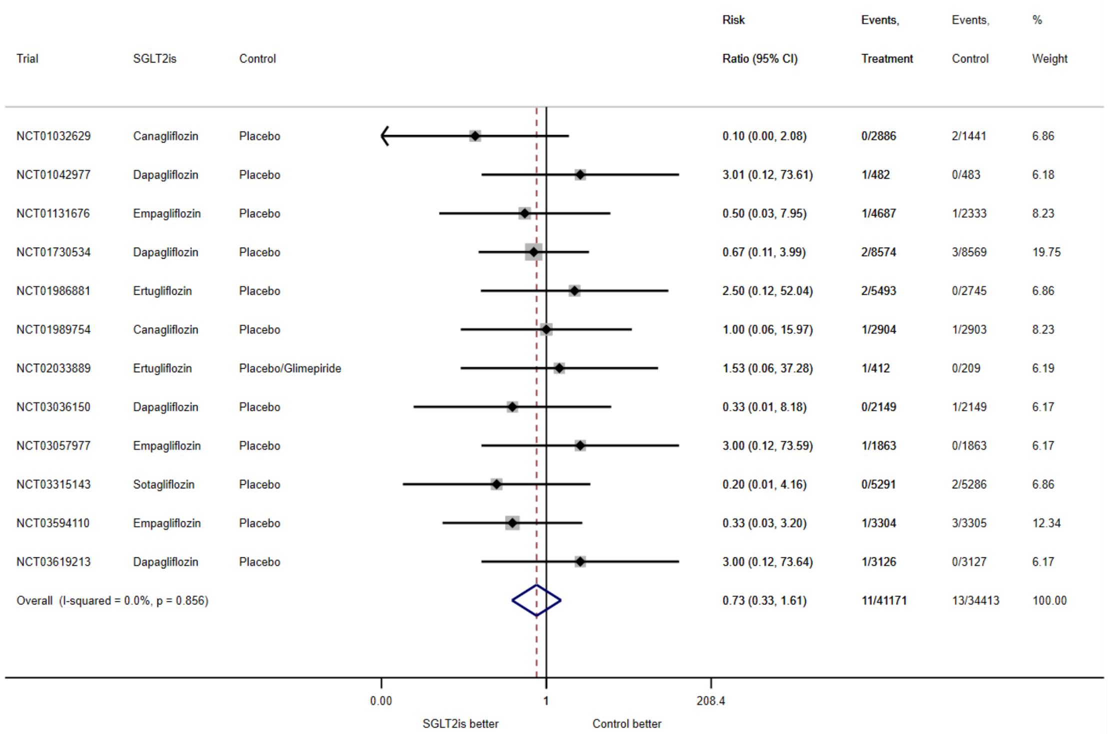

System 15-Musculoskeletal And Connective Tissue Disorders-Figure S15 Meta-analysis of SGLT2is and Intervertebral disc disorder

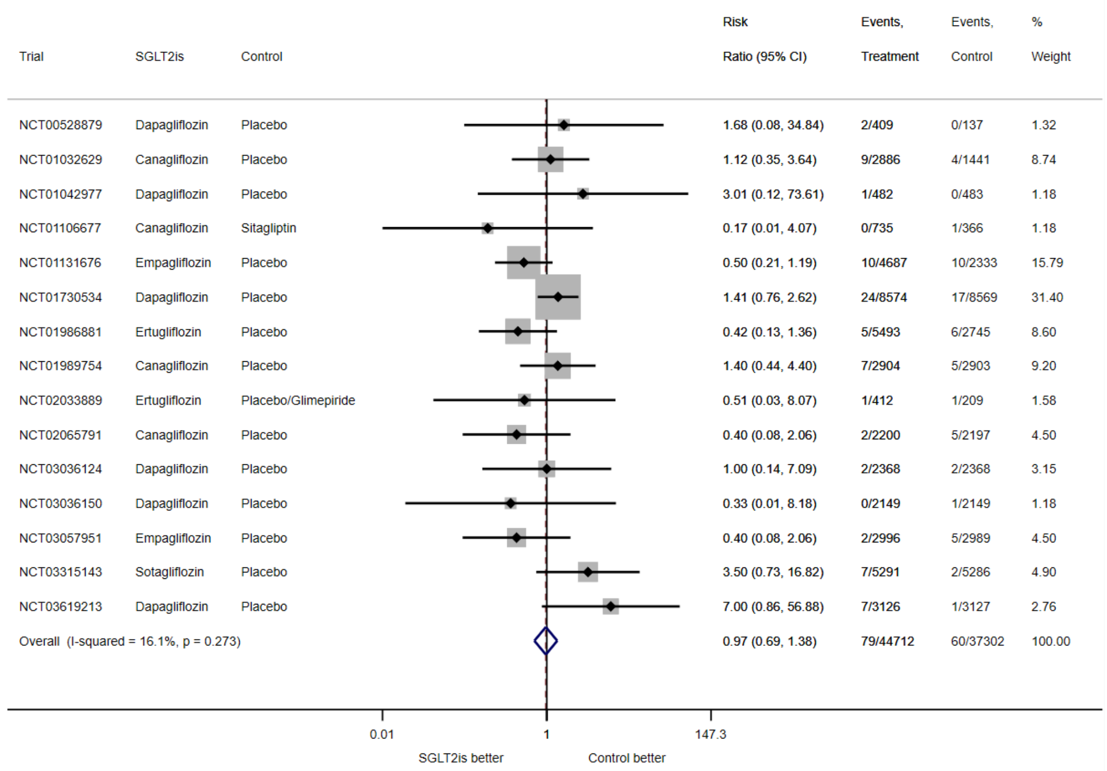

System 15-Musculoskeletal And Connective Tissue Disorders-Figure S16 Meta-analysis of SGLT2is and Intervertebral disc protrusion

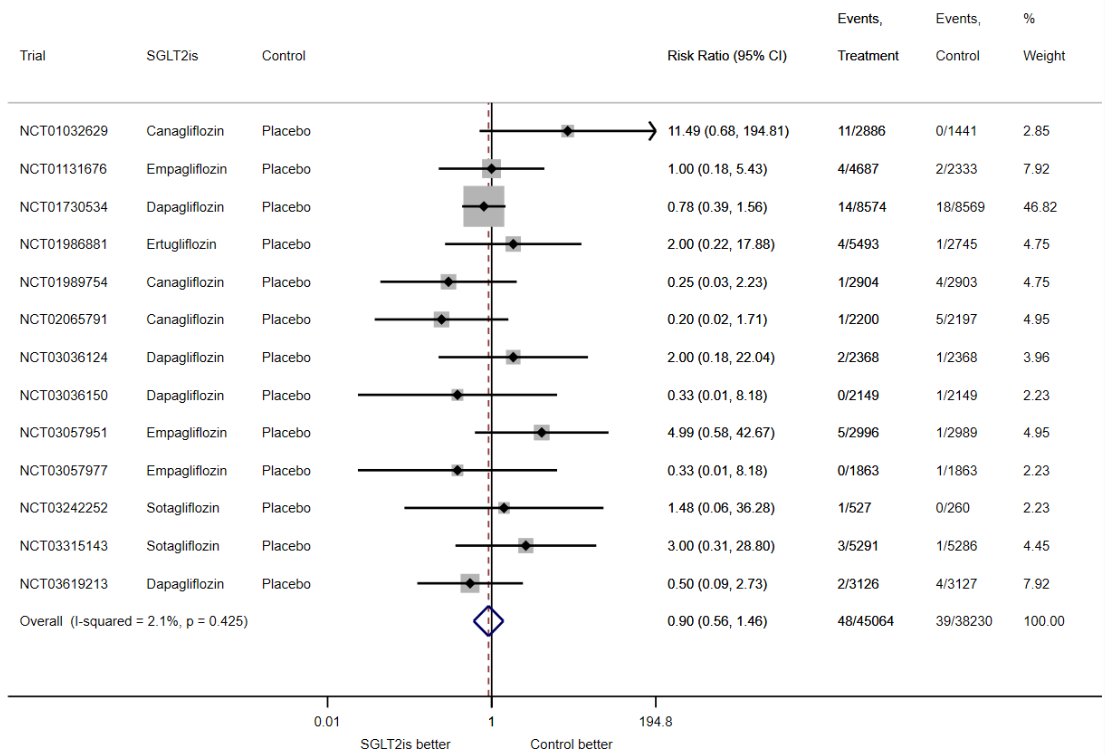

System 15-Musculoskeletal And Connective Tissue Disorders-Figure S17 Meta-analysis of SGLT2is and Lumbar spinal stenosis

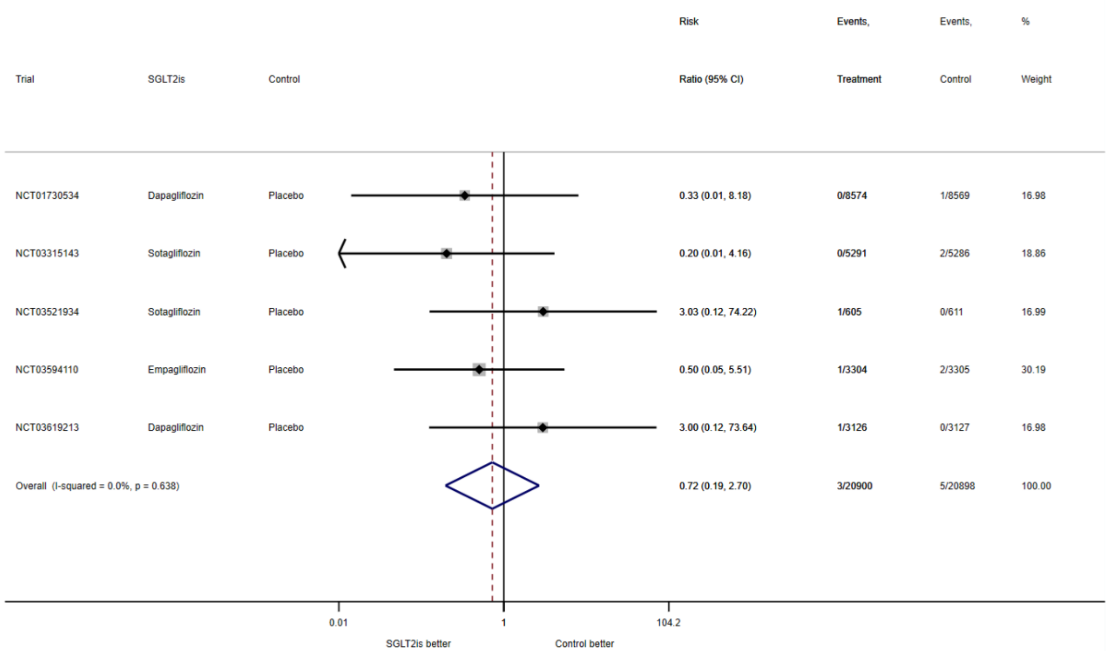

System 15-Musculoskeletal And Connective Tissue Disorders-Figure S18 Meta-analysis of SGLT2is and Mobility decreased

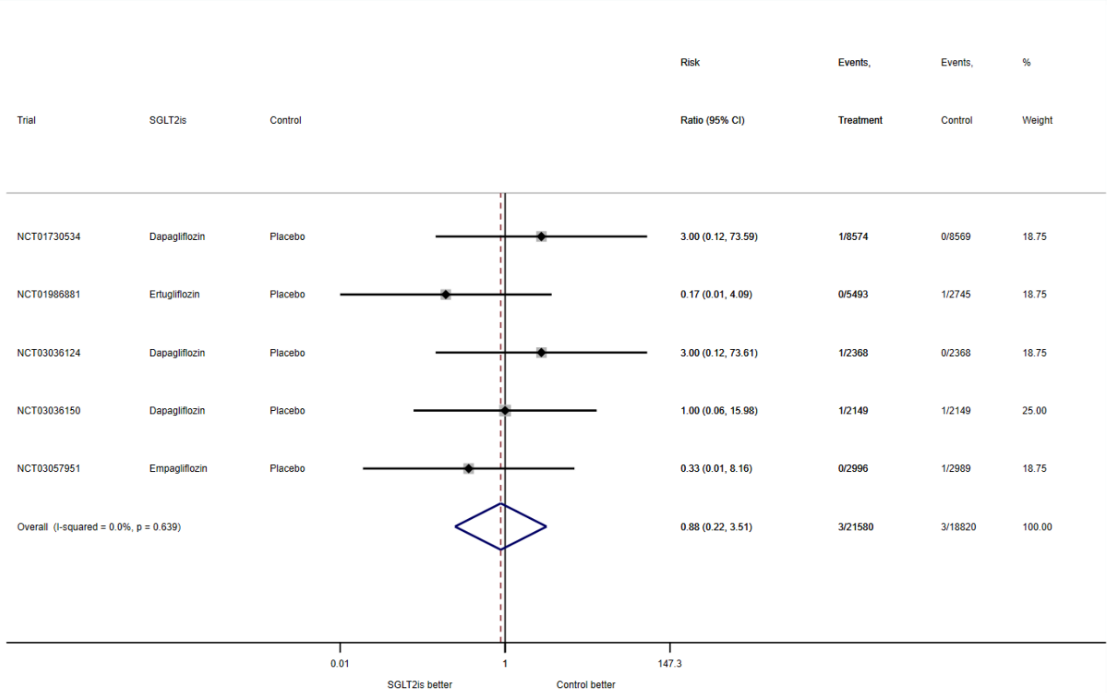

System 15-Musculoskeletal And Connective Tissue Disorders-Figure S19 Meta-analysis of SGLT2is and Muscle spasms

Trial	SGLT2is	Control	Risk Ratio (95% CI)	Events, Treatment	Events, Control	% Weight
NCT01106651	Canagliflozin	Placebo	1.49 (0.06, 36.53)	1/477	0/237	3.96
NCT01131676	Empagliflozin	Placebo	0.25 (0.02, 2.74)	1/4687	2/2333	7.02
NCT01730534	Dapagliflozin	Placebo	0.83 (0.25, 2.73)	5/8574	6/8569	28.74
NCT01986881	Ertugliflozin	Placebo	3.50 (0.18, 67.71)	3/5493	0/2745	4.61
NCT01989754	Canagliflozin	Placebo	1.00 (0.14, 7.09)	2/2904	2/2903	10.54
NCT02065791	Canagliflozin	Placebo	2.00 (0.18, 22.01)	2/2200	1/2197	7.02
NCT03036124	Dapagliflozin	Placebo	3.00 (0.12, 73.61)	1/2368	0/2368	3.95
NCT03057951	Empagliflozin	Placebo	1.50 (0.25, 8.95)	3/2996	2/2989	12.65
NCT03057977	Empagliflozin	Placebo	1.00 (0.06, 15.98)	1/1863	1/1863	5.27
NCT03315143	Sotagliflozin	Placebo	0.33 (0.01, 8.17)	0/5291	1/5286	3.95
NCT03594110	Empagliflozin	Placebo	3.00 (0.31, 28.83)	3/3304	1/3305	7.90
NCT03619213	Dapagliflozin	Placebo	0.20 (0.01, 4.17)	0/3126	2/3127	4.39
Overall (I-squared = 0.0%, p = 0.903)			1.04 (0.55, 1.96)	22/43283	18/37922	100.00

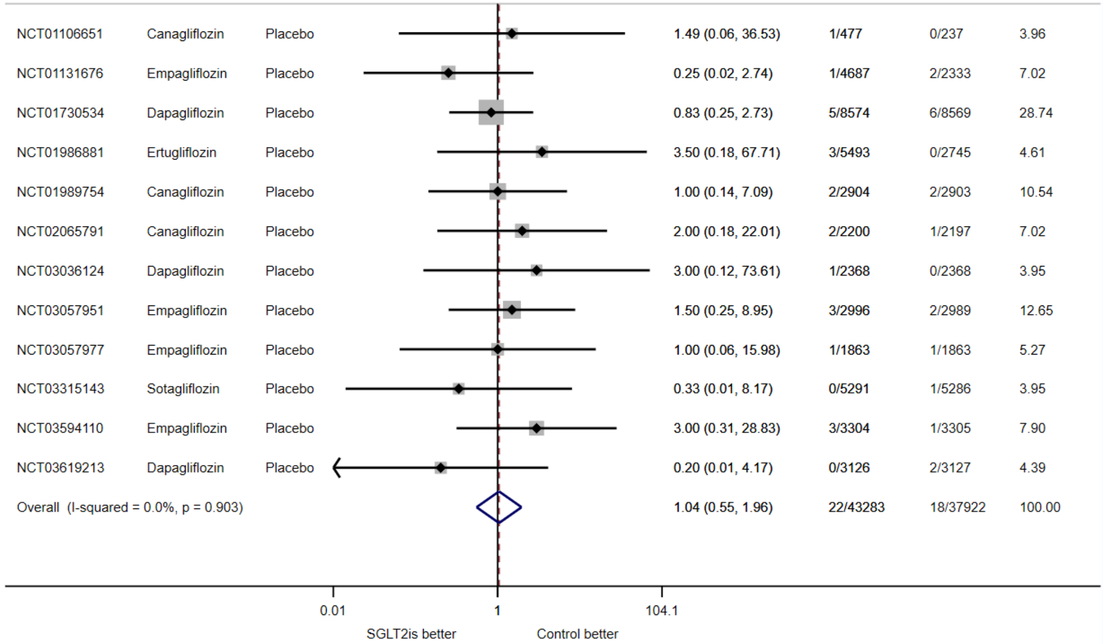

System 15-Musculoskeletal And Connective Tissue Disorders-Figure S20 Meta-analysis of SGLT2is and Muscular weakness

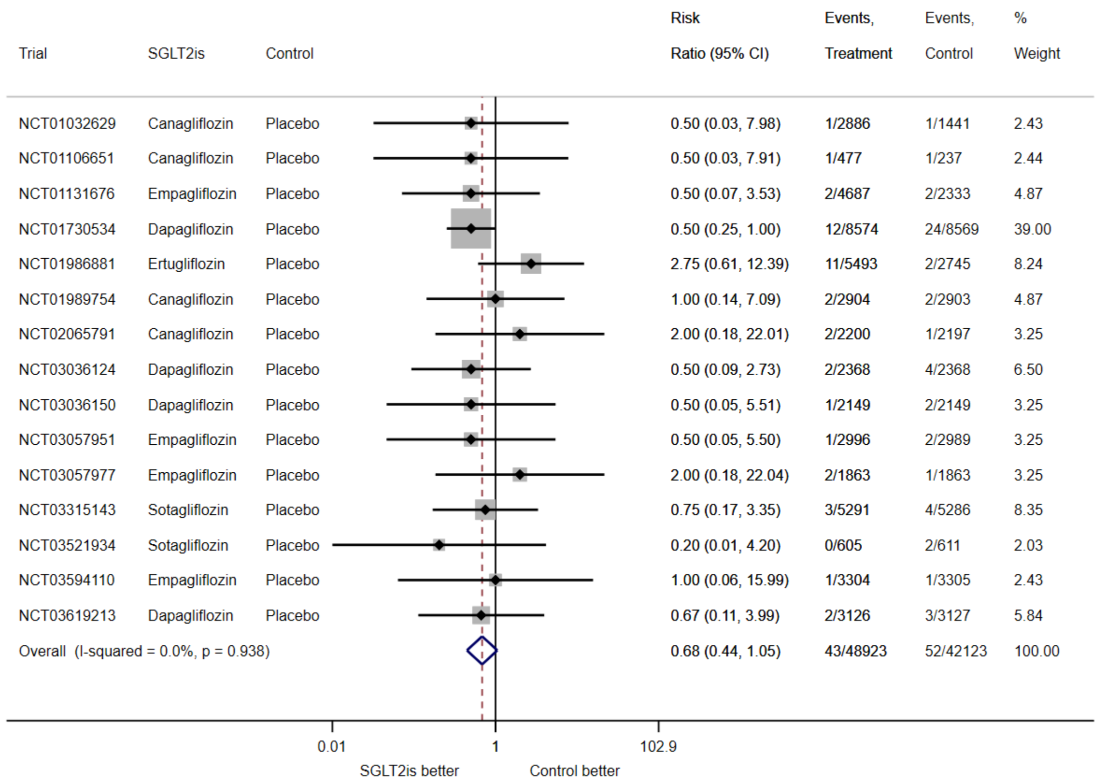

System 15-Musculoskeletal And Connective Tissue Disorders-Figure S21 Meta-analysis of SGLT2is and Musculoskeletal chest pain

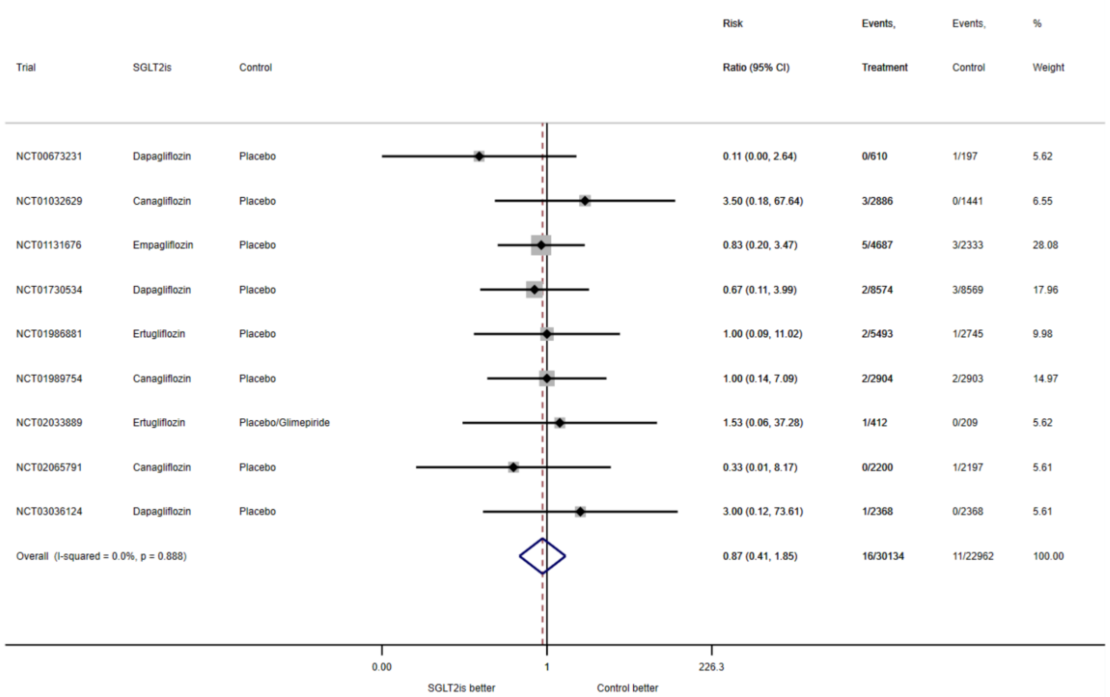

System 15-Musculoskeletal And Connective Tissue Disorders-Figure S22 Meta-analysis of SGLT2is and Musculoskeletal pain

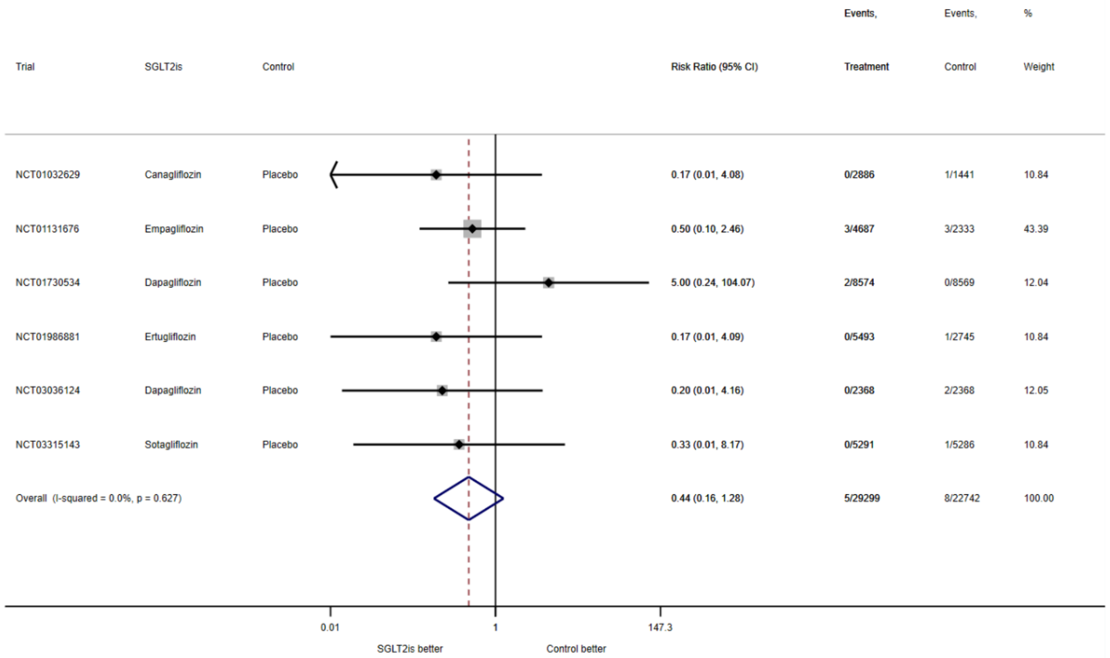

System 15-Musculoskeletal And Connective Tissue Disorders-Figure S23 Meta-analysis of SGLT2is and Myalgia

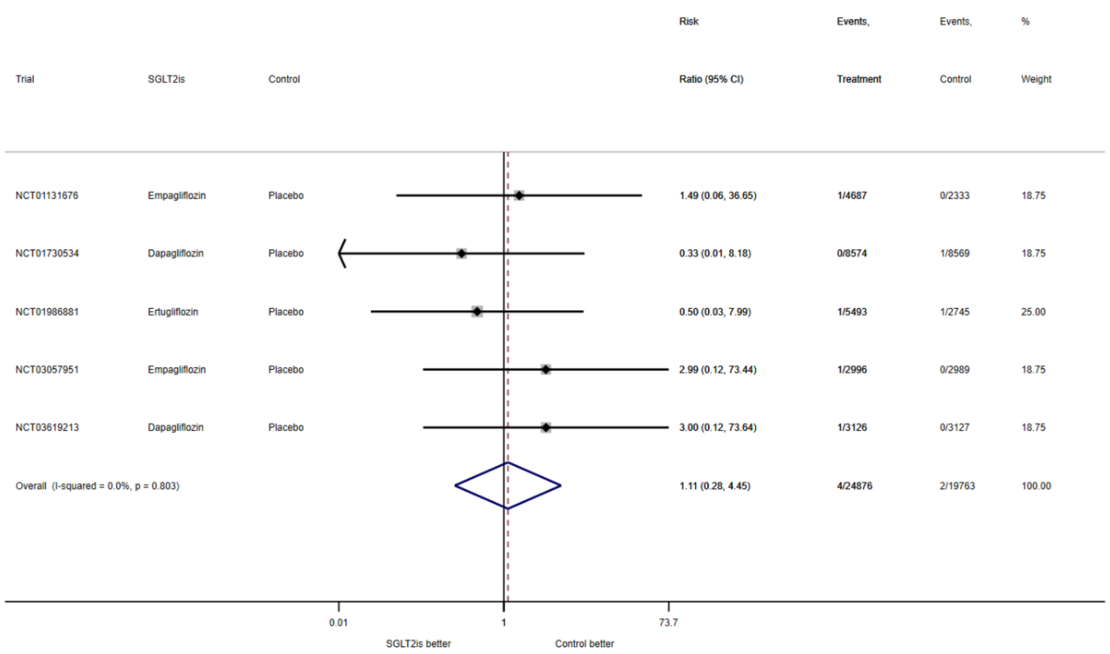

System 15-Musculoskeletal And Connective Tissue Disorders-Figure S24 Meta-analysis of SGLT2is and Myositis

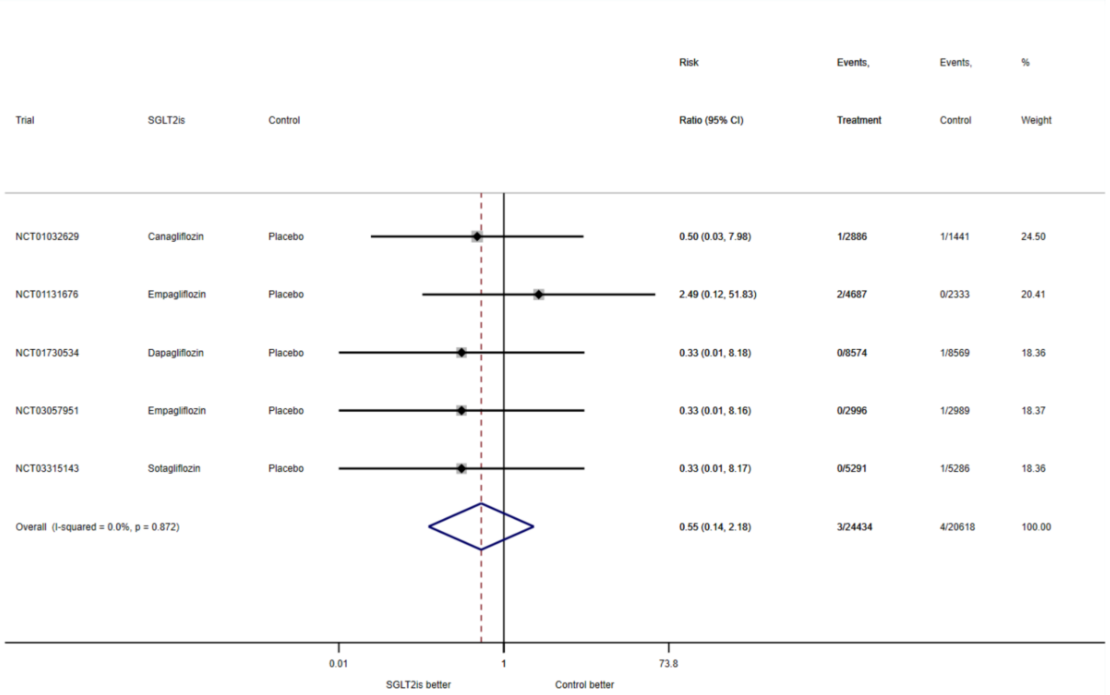

System 15-Musculoskeletal And Connective Tissue Disorders-Figure S25 Meta-analysis of SGLT2is and Neck pain

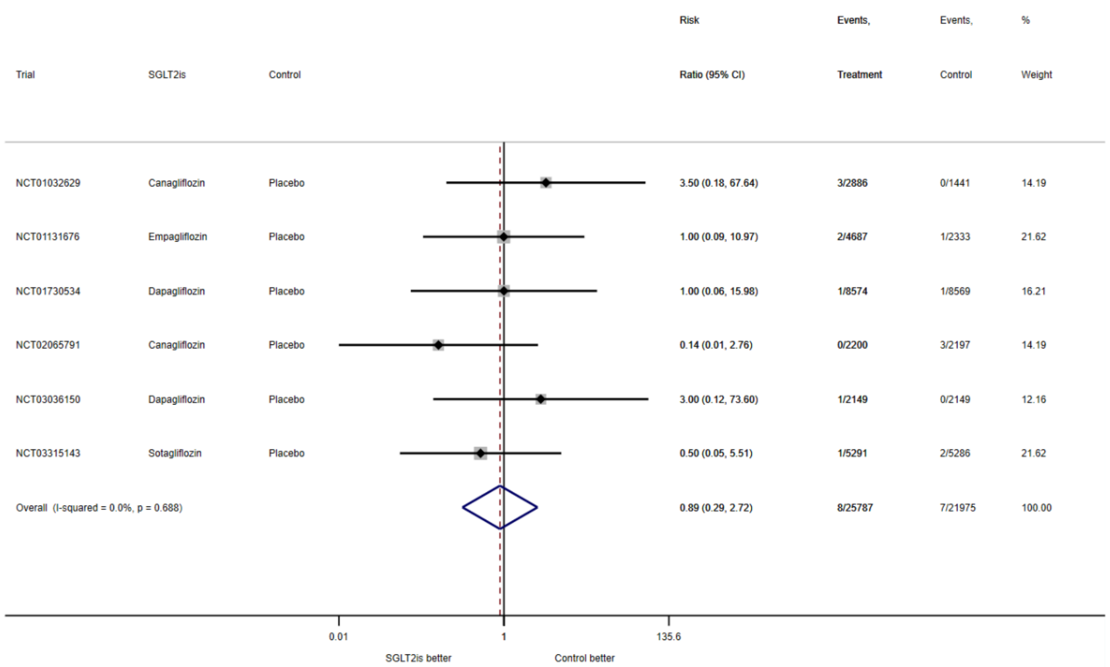

System 15-Musculoskeletal And Connective Tissue Disorders-Figure S26 Meta-analysis of SGLT2is and Neuropathic arthropathy

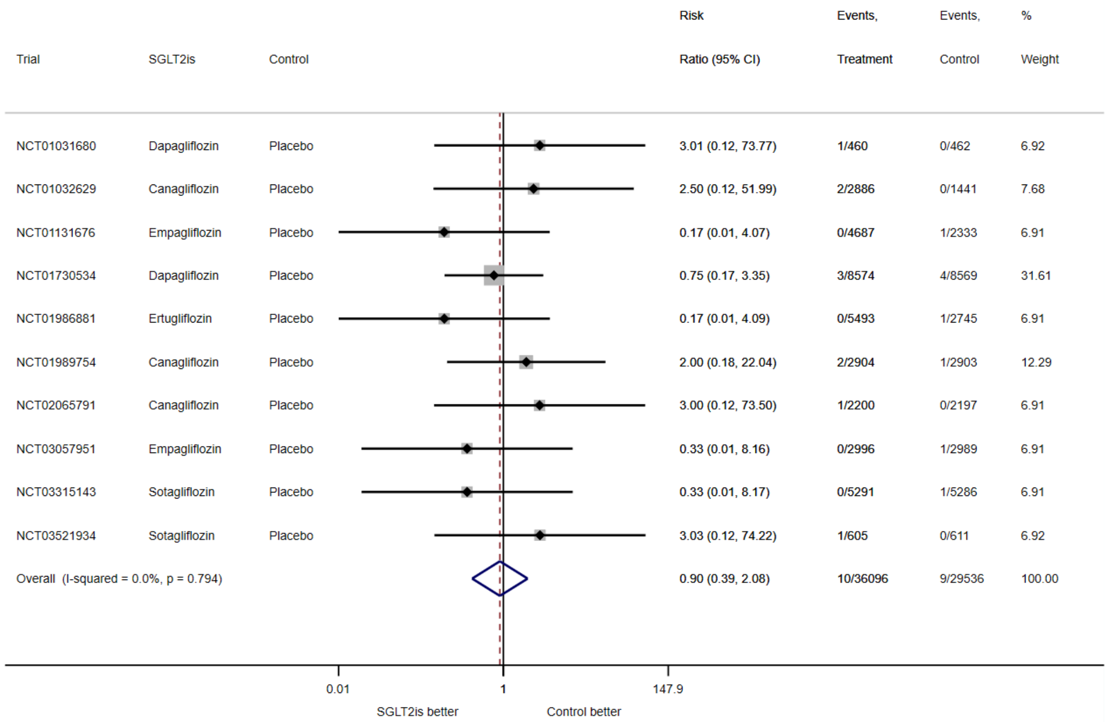

System 15-Musculoskeletal And Connective Tissue Disorders-Figure S27 Meta-analysis of SGLT2is and Osteitis

Trial	SGLT2is	Control	Risk Ratio (95% CI)	Events, Treatment	Events, Control	% Weight
NCT00528879	Dapagliflozin	Placebo	1.01 (0.04, 24.64)	1/409	0/137	0.28
NCT01031680	Dapagliflozin	Placebo	0.33 (0.01, 8.20)	0/460	1/462	0.28
NCT01032629	Canagliflozin	Placebo	0.98 (0.62, 1.55)	53/2886	27/1441	13.49
NCT01042977	Dapagliflozin	Placebo	3.01 (0.12, 73.61)	1/482	0/483	0.28
NCT01106651	Canagliflozin	Placebo	1.49 (0.16, 14.25)	3/477	1/237	0.56
NCT01131676	Empagliflozin	Placebo	1.06 (0.58, 1.96)	32/4687	15/2333	7.61
NCT01164501	Empagliflozin	Placebo	3.81 (0.18, 79.07)	2/419	0/319	0.31
NCT01730534	Dapagliflozin	Placebo	1.12 (0.85, 1.48)	103/8574	92/8569	36.39
NCT01986881	Ertugliflozin	Placebo	0.90 (0.53, 1.54)	38/5493	21/2745	10.09
NCT01989754	Canagliflozin	Placebo	0.81 (0.39, 1.69)	13/2904	16/2903	5.34
NCT02033889	Ertugliflozin	Placebo/Glimepiride	0.51 (0.03, 8.07)	1/412	1/209	0.37
NCT02065791	Canagliflozin	Placebo	0.91 (0.39, 2.13)	10/2200	11/2197	3.90
NCT03036124	Dapagliflozin	Placebo	0.71 (0.23, 2.25)	5/2368	7/2368	2.16
NCT03036150	Dapagliflozin	Placebo	1.17 (0.39, 3.47)	7/2149	6/2149	2.40
NCT03057951	Empagliflozin	Placebo	0.69 (0.32, 1.48)	11/2996	16/2989	4.85
NCT03057977	Empagliflozin	Placebo	0.43 (0.11, 1.65)	3/1863	7/1863	1.56
NCT03242252	Sotagliflozin	Placebo	0.49 (0.07, 3.48)	2/527	2/260	0.74
NCT03315143	Sotagliflozin	Placebo	0.75 (0.32, 1.78)	9/5291	12/5286	3.81
NCT03521934	Sotagliflozin	Placebo	7.07 (0.37, 136.57)	3/605	0/611	0.32
NCT03594110	Empagliflozin	Placebo	0.40 (0.13, 1.27)	4/3304	10/3305	2.12
NCT03619213	Dapagliflozin	Placebo	1.13 (0.43, 2.91)	9/3126	8/3127	3.14
Overall (I-squared = 0.0%, p = 0.949)			0.96 (0.81, 1.14)	310/51632	253/43993	100.00

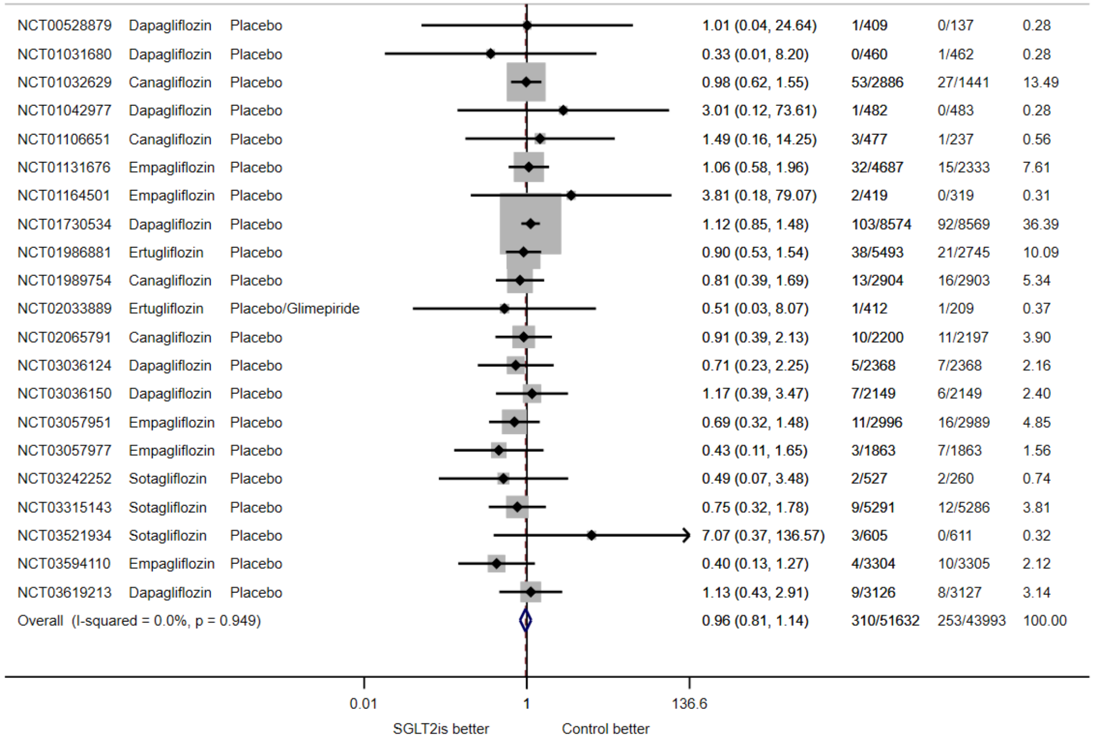

System 15-Musculoskeletal And Connective Tissue Disorders-Figure S28 Meta-analysis of SGLT2is and Osteoarthritis

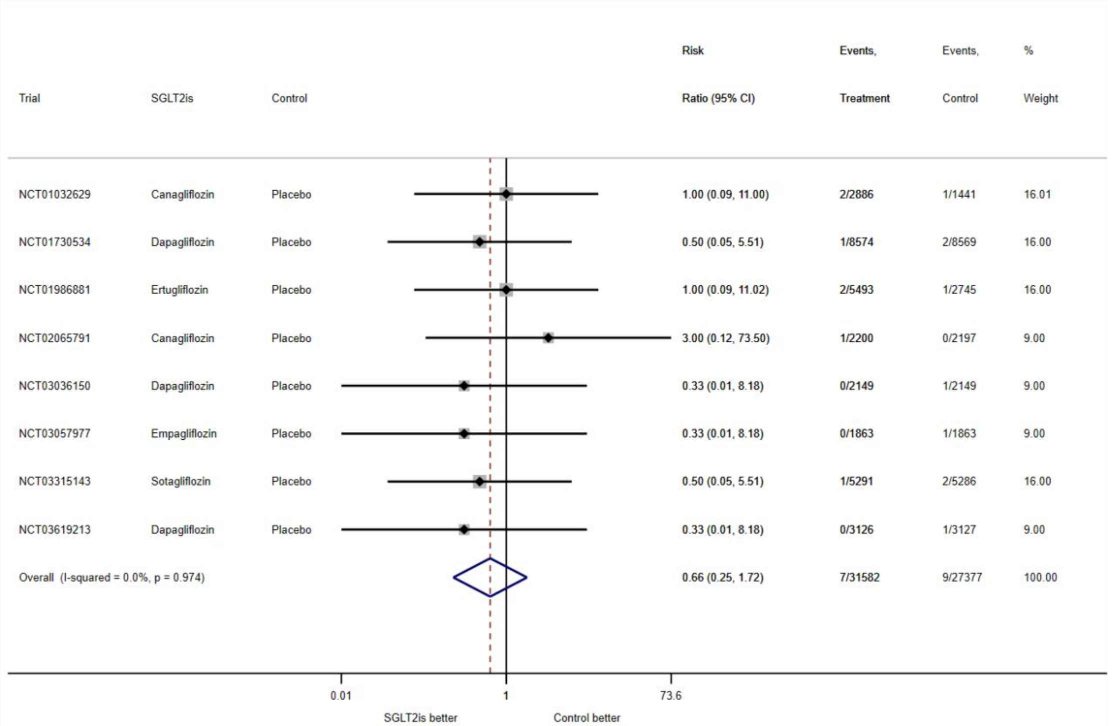

System 15-Musculoskeletal And Connective Tissue Disorders-Figure S29 Meta-analysis of SGLT2is and Osteochondrosis

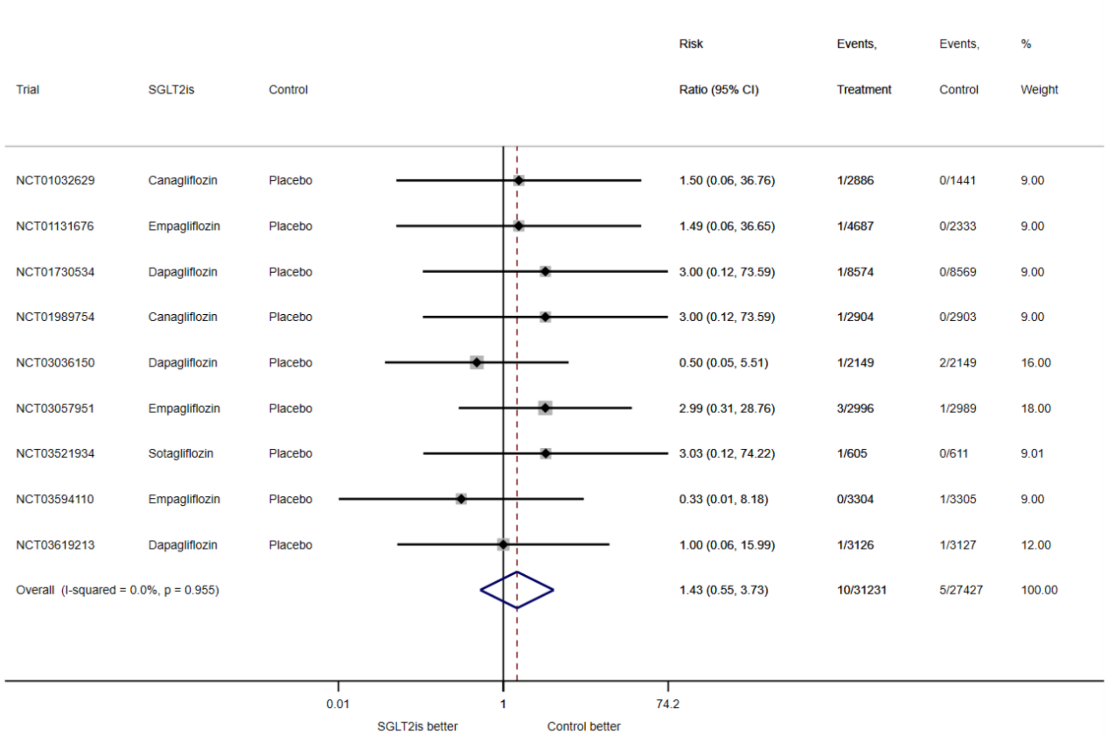

System 15-Musculoskeletal And Connective Tissue Disorders-Figure S30 Meta-analysis of SGLT2is and Osteonecrosis

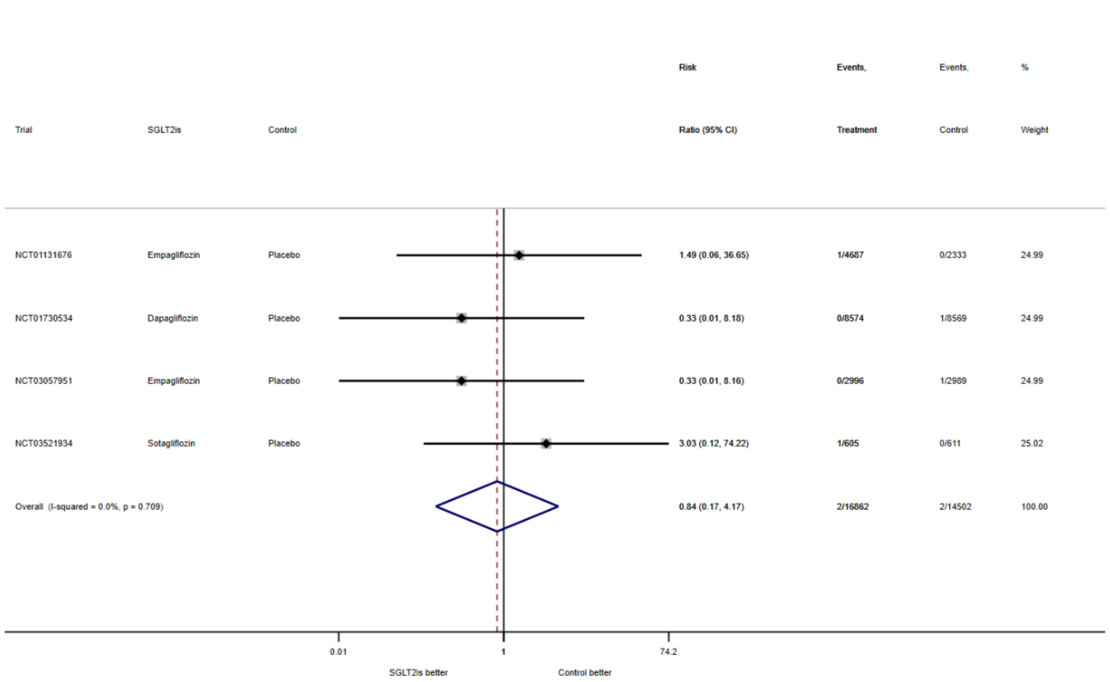

System 15-Musculoskeletal And Connective Tissue Disorders-Figure S31 Meta-analysis of SGLT2is and Osteoporosis

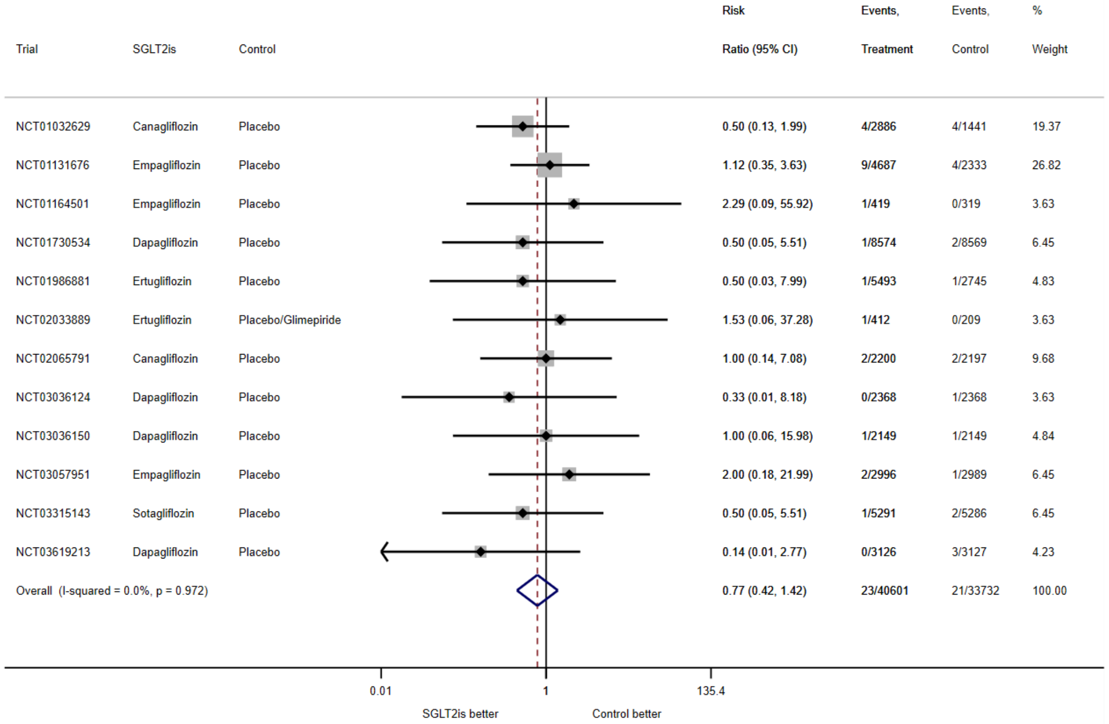

System 15-Musculoskeletal And Connective Tissue Disorders-Figure S32 Meta-analysis of SGLT2is and Pain in extremity

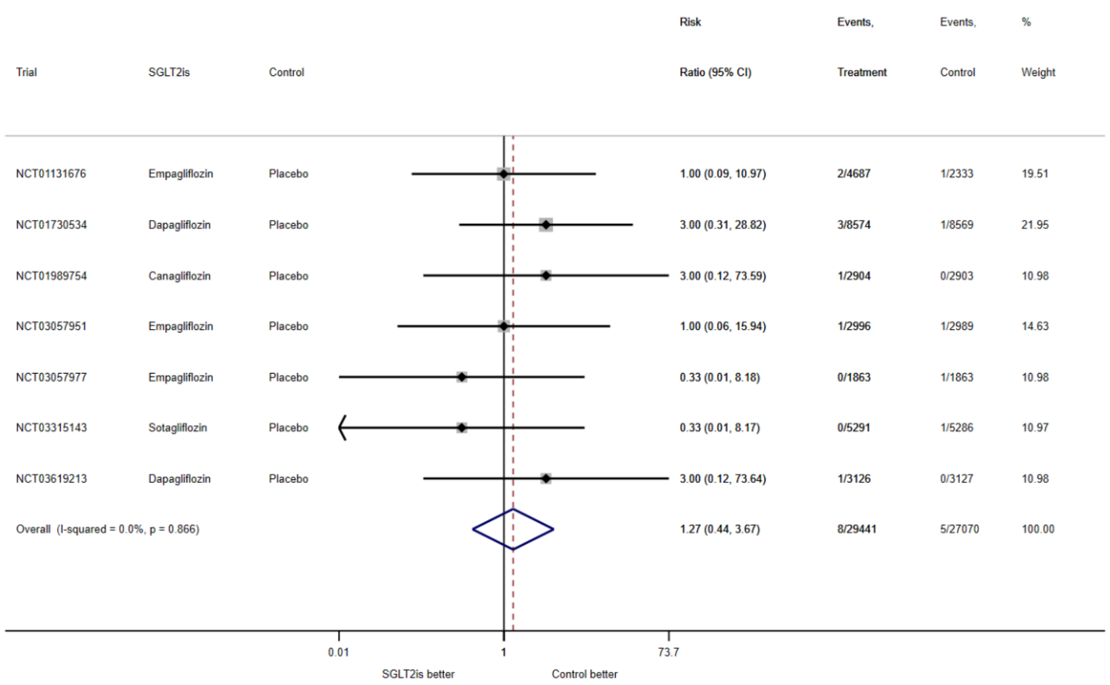

System 15-Musculoskeletal And Connective Tissue Disorders-Figure S33 Meta-analysis of SGLT2is and Pathological fracture

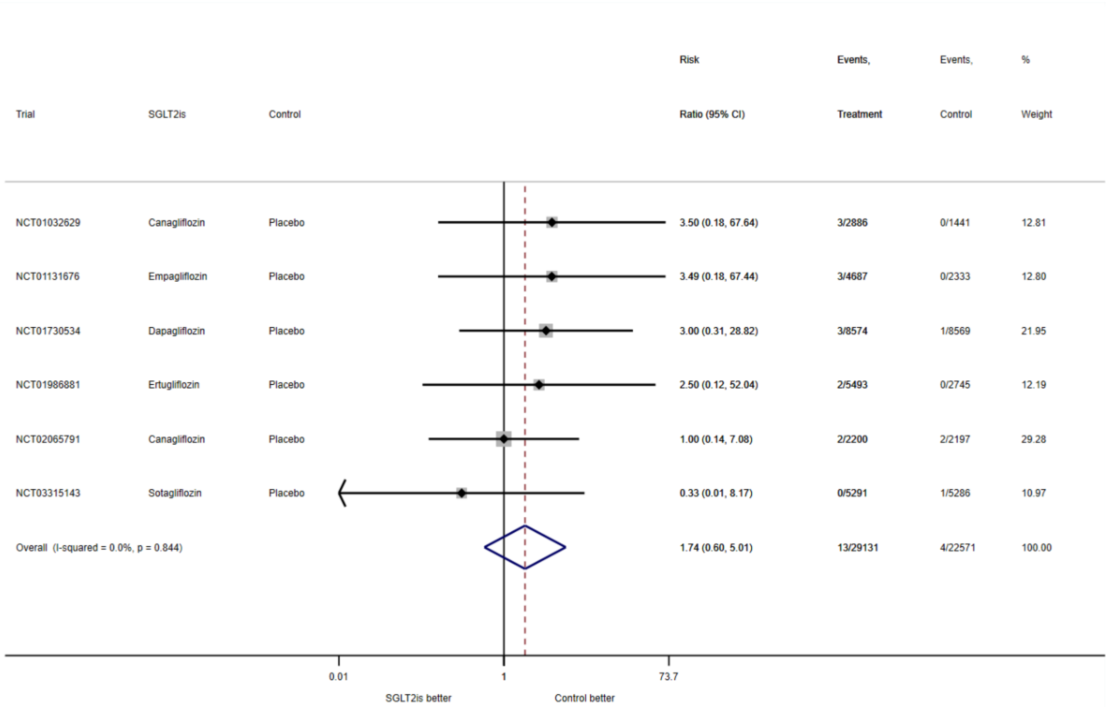

System 15-Musculoskeletal And Connective Tissue Disorders-Figure S34 Meta-analysis of SGLT2is and Periarthritis

Trial	SGLT2is	Control	Risk Ratio (95% CI)	Events, Treatment	Events, Control	% Weight
NCT01032629	Canagliflozin	Placebo	0.17 (0.01, 4.08)	0/2886	1/1441	13.63
NCT01042977	Dapagliflozin	Placebo	3.01 (0.12, 73.61)	1/482	0/483	13.65
NCT01730534	Dapagliflozin	Placebo	3.00 (0.12, 73.59)	1/8574	0/8569	13.63
NCT01986881	Ertugliflozin	Placebo	1.50 (0.06, 36.80)	1/5493	0/2745	13.63
NCT03315143	Sotagliflozin	Placebo	0.33 (0.01, 8.17)	0/5291	1/5286	13.63
NCT03521934	Sotagliflozin	Placebo	1.01 (0.06, 16.11)	1/605	1/611	18.20
NCT03619213	Dapagliflozin	Placebo	0.33 (0.01, 8.18)	0/3126	1/3127	13.63
Overall (I-squared = 0.0%, p = 0.811)			0.83 (0.25, 2.70)	4/26457	4/22262	100.00

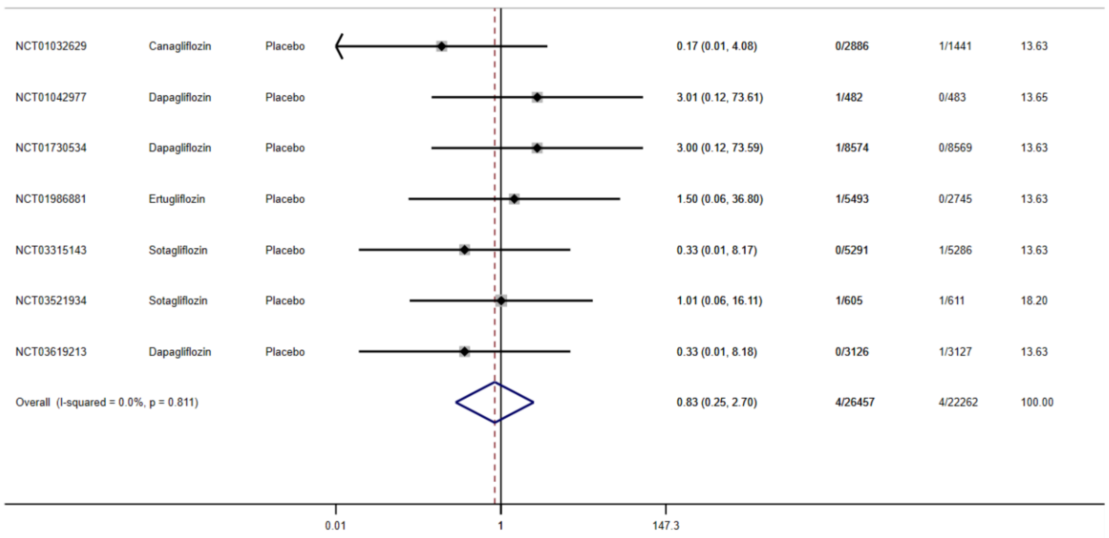

System 15-Musculoskeletal And Connective Tissue Disorders-Figure S35 Meta-analysis of SGLT2is and Polyarthritis

Trial	SGLT2is	Control	Risk Ratio (95% CI)	Events, Treatment	Events, Control	% Weight
NCT01032629	Canagliflozin	Placebo	0.25 (0.02, 2.75)	1/2886	2/1441	8.60
NCT01730534	Dapagliflozin	Placebo	0.33 (0.03, 3.20)	1/8574	3/8569	9.67
NCT01986881	Ertugliflozin	Placebo	1.50 (0.06, 36.80)	1/5493	0/2745	4.84
NCT01989754	Canagliflozin	Placebo	3.00 (0.12, 73.59)	1/2904	0/2903	4.84
NCT03036124	Dapagliflozin	Placebo	0.33 (0.01, 8.18)	0/2368	1/2368	4.84
NCT03036150	Dapagliflozin	Placebo	3.00 (0.12, 73.60)	1/2149	0/2149	4.84
NCT03057951	Empagliflozin	Placebo	0.33 (0.09, 1.23)	3/2996	9/2989	29.05
NCT03057977	Empagliflozin	Placebo	0.33 (0.01, 8.18)	0/1863	1/1863	4.84
NCT03315143	Sotagliflozin	Placebo	0.33 (0.01, 8.17)	0/5291	1/5286	4.84
NCT03594110	Empagliflozin	Placebo	2.00 (0.37, 10.92)	4/3304	2/3305	17.21
NCT03619213	Dapagliflozin	Placebo	1.00 (0.06, 15.99)	1/3126	1/3127	6.45
Overall (I-squared = 0.0%, p = 0.794)			0.63 (0.31, 1.28)	13/40954	20/36745	100.00

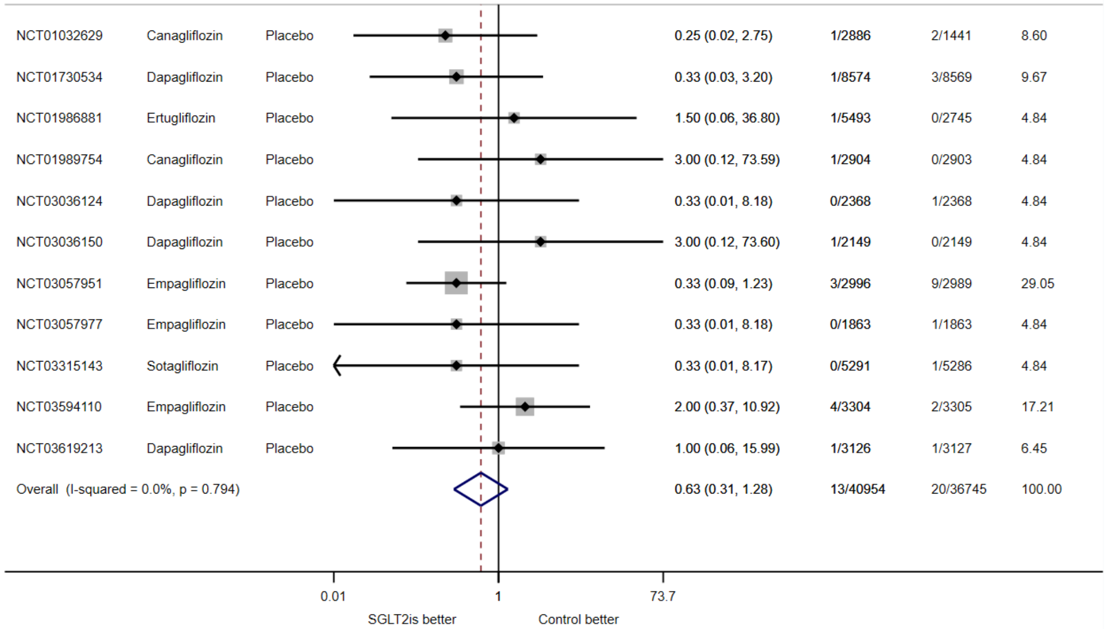

System 15-Musculoskeletal And Connective Tissue Disorders-Figure S36 Meta-analysis of SGLT2is and Polymyalgia rheumatica

Trial	SGLT2is	Control	Risk Ratio (95% CI)	Events, Treatment	Events, Control	% Weight
NCT01131676	Empagliflozin	Placebo	1.49 (0.30, 7.39)	6/4687	2/2333	18.25
NCT01730534	Dapagliflozin	Placebo	0.29 (0.06, 1.37)	2/8574	7/8569	18.91
NCT01986881	Ertugliflozin	Placebo	0.50 (0.07, 3.55)	2/5493	2/2745	12.16
NCT01989754	Canagliflozin	Placebo	0.33 (0.01, 8.18)	0/2904	1/2903	4.56
NCT03036150	Dapagliflozin	Placebo	3.00 (0.31, 28.82)	3/2149	1/2149	9.12
NCT03057951	Empagliflozin	Placebo	0.09 (0.01, 1.64)	0/2996	5/2989	5.57
NCT03057977	Empagliflozin	Placebo	1.00 (0.14, 7.09)	2/1863	2/1863	12.17
NCT03315143	Sotagliflozin	Placebo	0.50 (0.05, 5.51)	1/5291	2/5286	8.11
NCT03594110	Empagliflozin	Placebo	1.00 (0.06, 15.99)	1/3304	1/3305	6.08
NCT03619213	Dapagliflozin	Placebo	0.20 (0.01, 4.17)	0/3126	2/3127	5.07
Overall (I-squared = 0.0%, p = 0.663)			0.63 (0.32, 1.24)	17/40387	25/35269	100.00

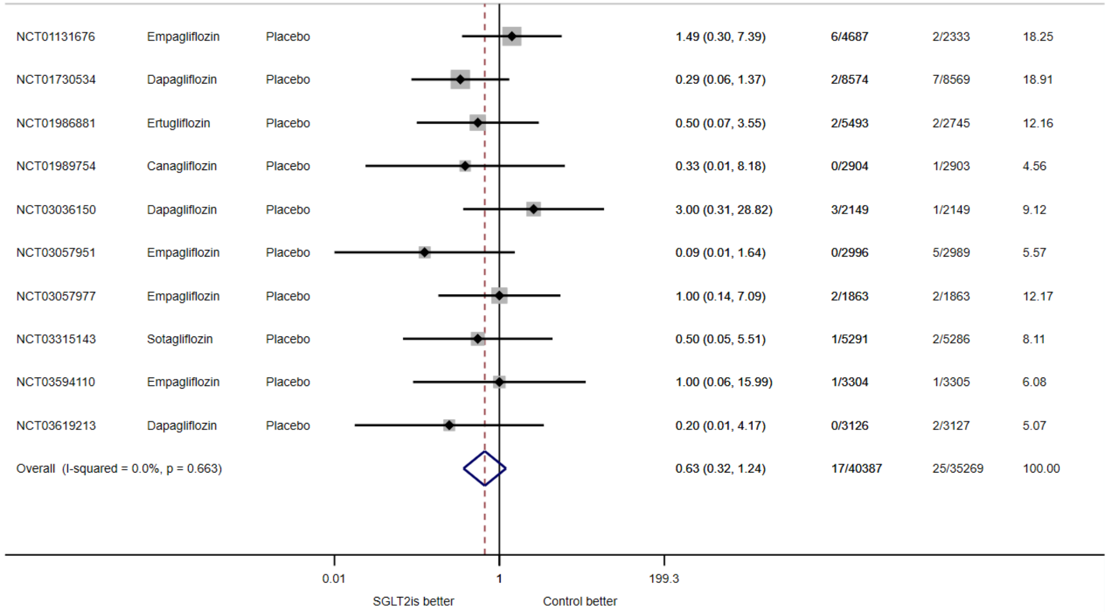

System 15-Musculoskeletal And Connective Tissue Disorders-Figure S37 Meta-analysis of SGLT2is and Rhabdomyolysis

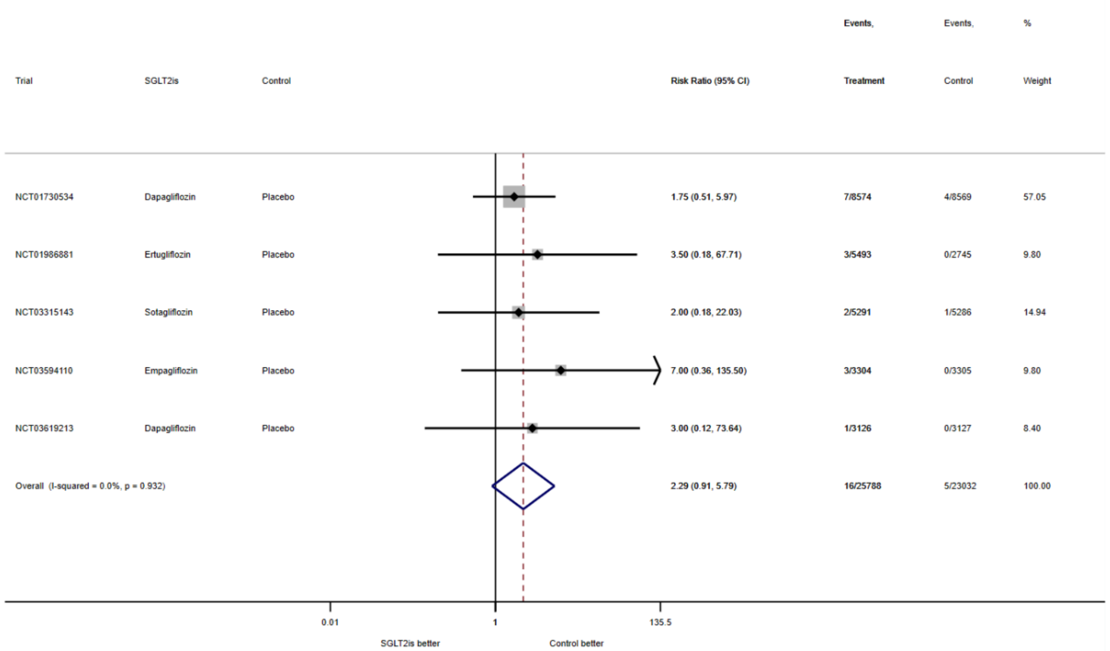

System 15-Musculoskeletal And Connective Tissue Disorders-Figure S38 Meta-analysis of SGLT2is and Rheumatoid arthritis

Trial	SGLT2is	Control	Risk Ratio (95% CI)	Events, Treatment	Events, Control	% Weight
NCT00528879	Dapagliflozin	Placebo	1.01 (0.04, 24.64)	1/409	0/137	3.59
NCT00673231	Dapagliflozin	Placebo	0.97 (0.04, 23.77)	1/610	0/197	3.58
NCT01032629	Canagliflozin	Placebo	1.00 (0.18, 5.45)	4/2886	2/1441	12.73
NCT01131676	Empagliflozin	Placebo	3.48 (0.43, 28.30)	7/4687	1/2333	8.35
NCT01730534	Dapagliflozin	Placebo	0.25 (0.08, 0.75)	4/8574	16/8569	30.53
NCT01986881	Ertugliflozin	Placebo	3.50 (0.43, 28.42)	7/5493	1/2745	8.35
NCT01989754	Canagliflozin	Placebo	4.00 (0.45, 35.75)	4/2904	1/2903	7.63
NCT02065791	Canagliflozin	Placebo	1.00 (0.06, 15.96)	1/2200	1/2197	4.77
NCT03036150	Dapagliflozin	Placebo	0.14 (0.01, 2.76)	0/2149	3/2149	4.17
NCT03057951	Empagliflozin	Placebo	0.33 (0.01, 8.16)	0/2996	1/2989	3.58
NCT03057977	Empagliflozin	Placebo	1.00 (0.06, 15.98)	1/1863	1/1863	4.77
NCT03315143	Sotagliflozin	Placebo	0.20 (0.01, 4.16)	0/5291	2/5286	3.97
NCT03619213	Dapagliflozin	Placebo	0.20 (0.01, 4.17)	0/3126	2/3127	3.97
Overall (I-squared = 10.2%, p = 0.343)			0.70 (0.38, 1.28)	30/43188	31/35936	100.00

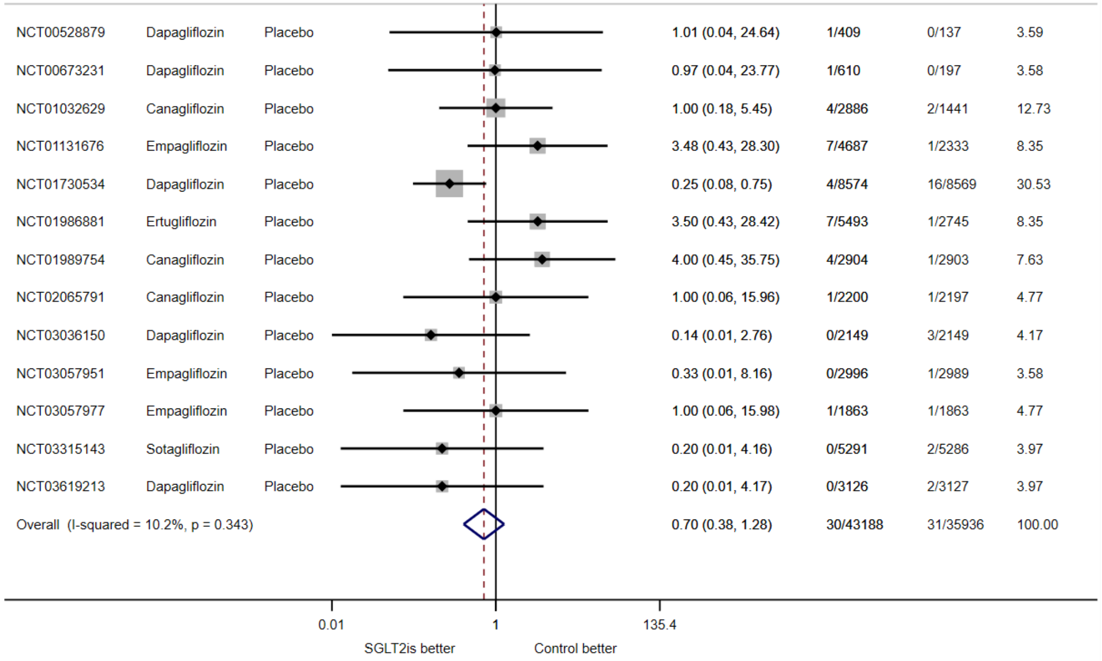

System 15-Musculoskeletal And Connective Tissue Disorders-Figure S39 Meta-analysis of SGLT2is and Rotator cuff syndrome

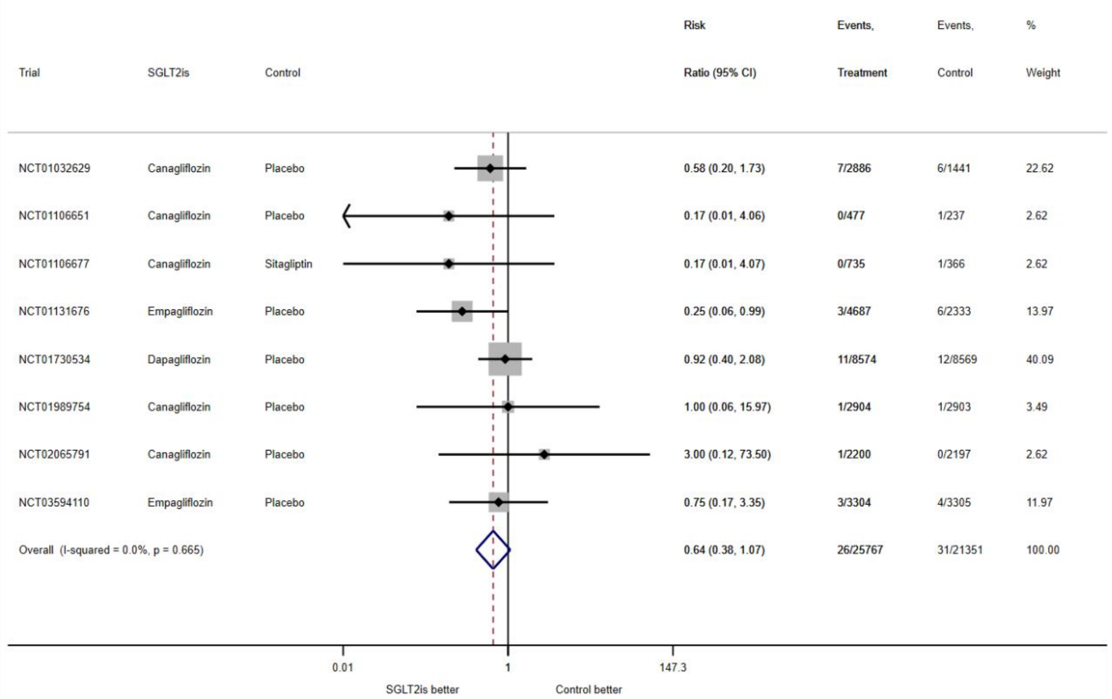

System 15-Musculoskeletal And Connective Tissue Disorders-Figure S40 Meta-analysis of SGLT2is and Spinal column stenosis

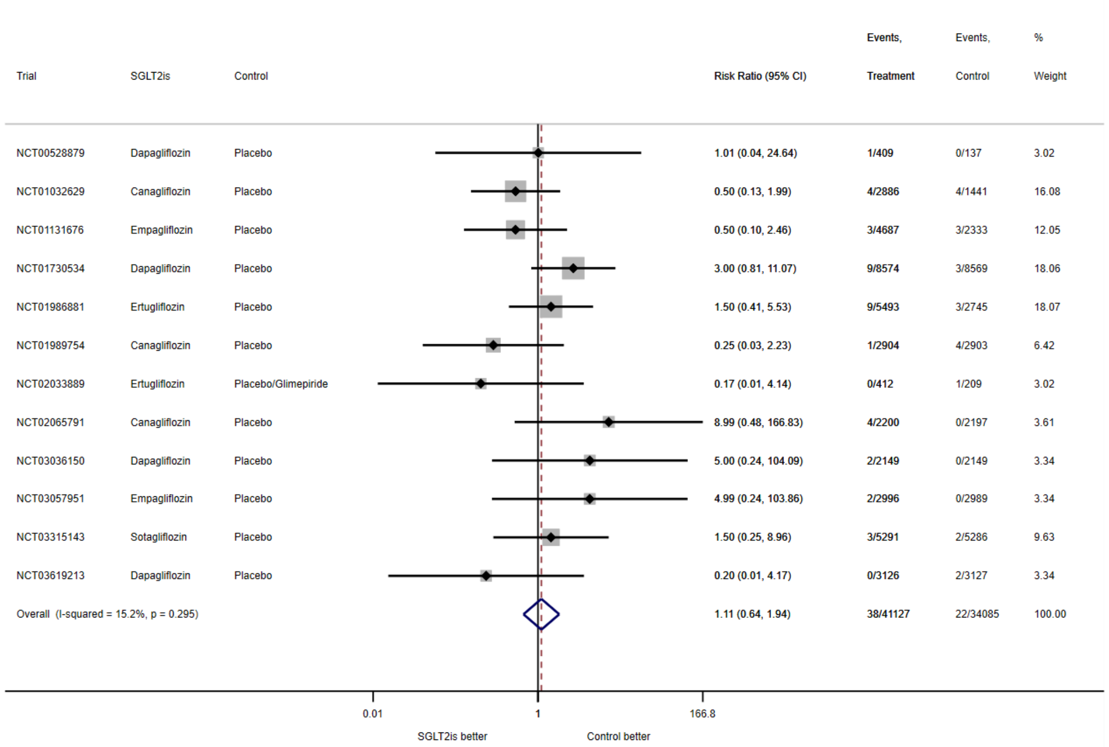

System 15-Musculoskeletal And Connective Tissue Disorders-Figure S41 Meta-analysis of SGLT2is and Spinal osteoarthritis

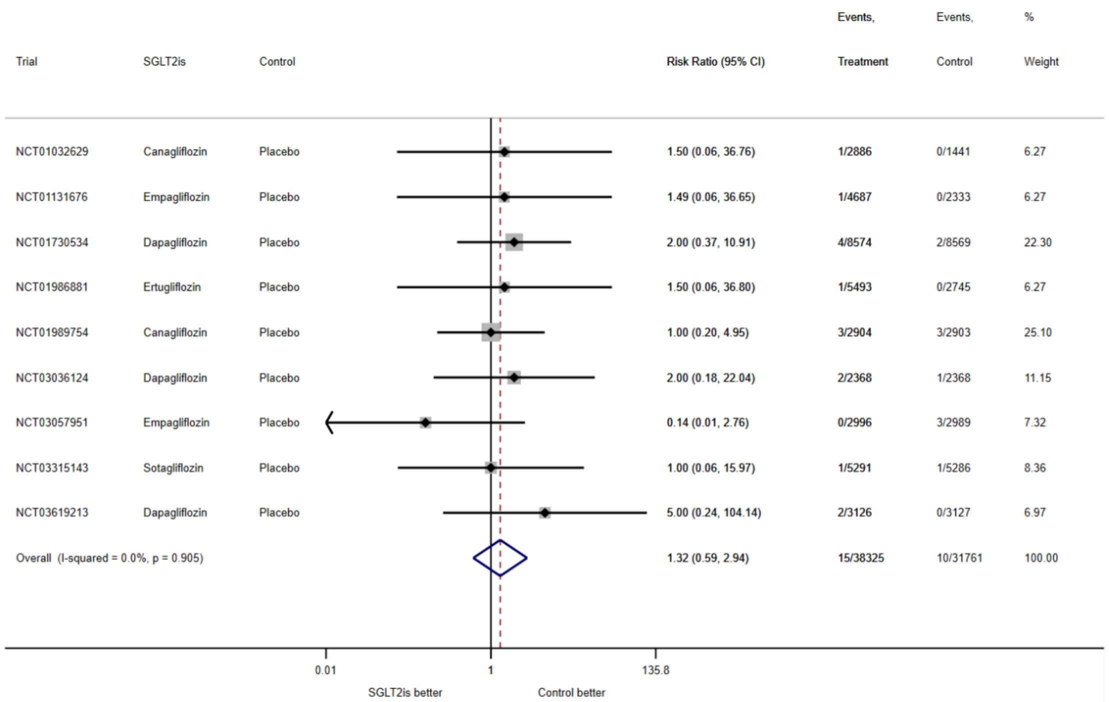

System 15-Musculoskeletal And Connective Tissue Disorders-Figure S42 Meta-analysis of SGLT2is and Spinal pain

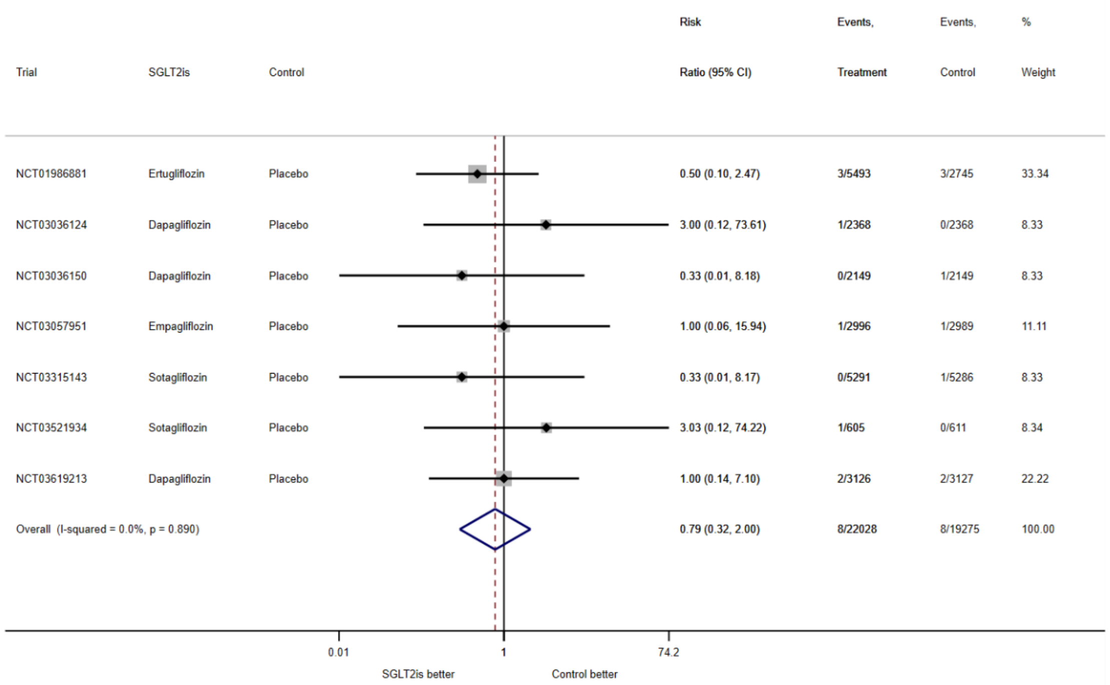

System 15-Musculoskeletal And Connective Tissue Disorders-Figure S43 Meta-analysis of SGLT2is and Spinal stenosis

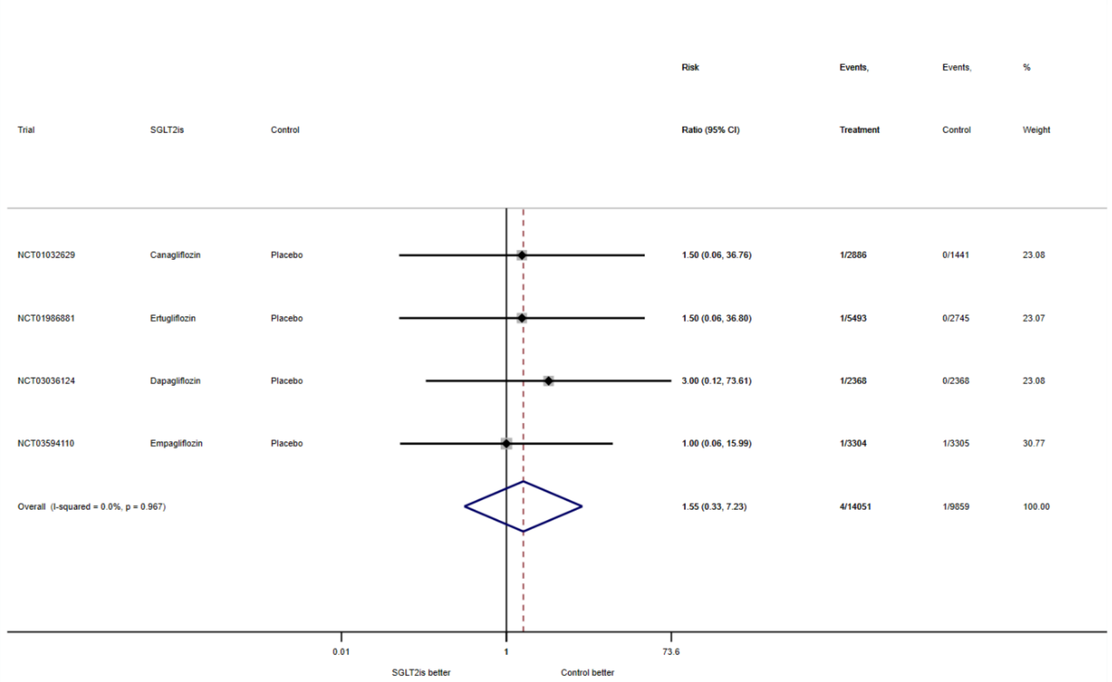

System 15-Musculoskeletal And Connective Tissue Disorders-Figure S44 Meta-analysis of SGLT2is and Spondyloarthropathy

Trial	SGLT2is	Control	Risk Ratio (95% CI)	Events, Treatment	Events, Control	% Weight
NCT01032629	Canagliflozin	Placebo	0.50 (0.07, 3.54)	2/2886	2/1441	18.19
NCT01131676	Empagliflozin	Placebo	2.49 (0.12, 51.83)	2/4687	0/2333	7.57
NCT01730534	Dapagliflozin	Placebo	0.60 (0.14, 2.51)	3/8574	5/8569	34.09
NCT01986881	Ertugliflozin	Placebo	2.50 (0.12, 52.04)	2/5493	0/2745	7.57
NCT01989754	Canagliflozin	Placebo	3.00 (0.12, 73.59)	1/2904	0/2903	6.82
NCT02065791	Canagliflozin	Placebo	0.33 (0.01, 8.17)	0/2200	1/2197	6.82
NCT03036124	Dapagliflozin	Placebo	0.33 (0.01, 8.18)	0/2368	1/2368	6.82
NCT03315143	Sotagliflozin	Placebo	2.00 (0.18, 22.03)	2/5291	1/5286	12.12
Overall (I-squared = 0.0%, p = 0.864)			0.86 (0.37, 1.98)	12/34403	10/27842	100.00

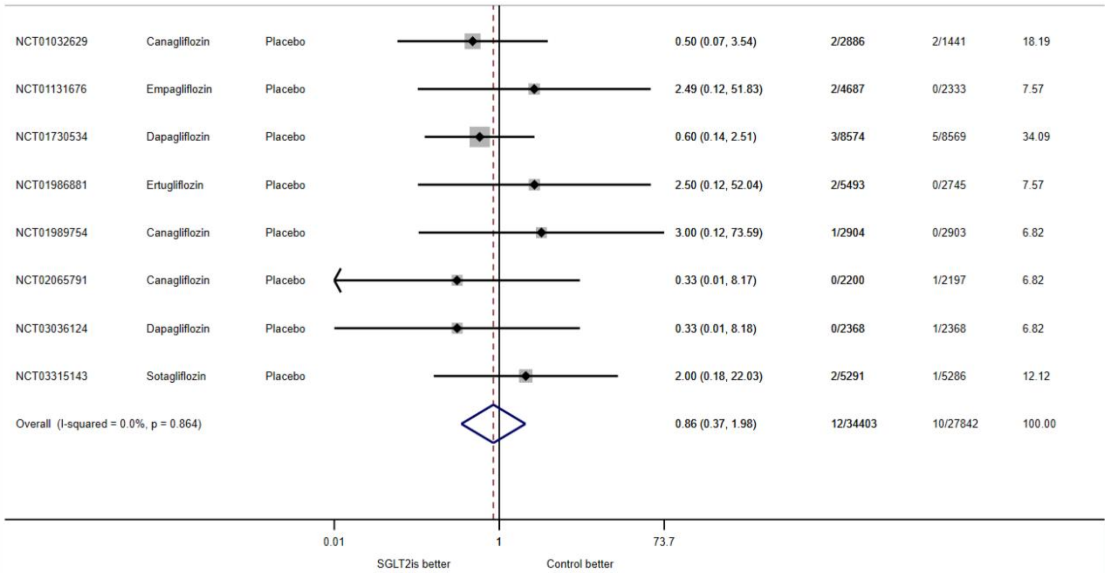

System 15-Musculoskeletal And Connective Tissue Disorders-Figure S45 Meta-analysis of SGLT2is and Spondylolisthesis

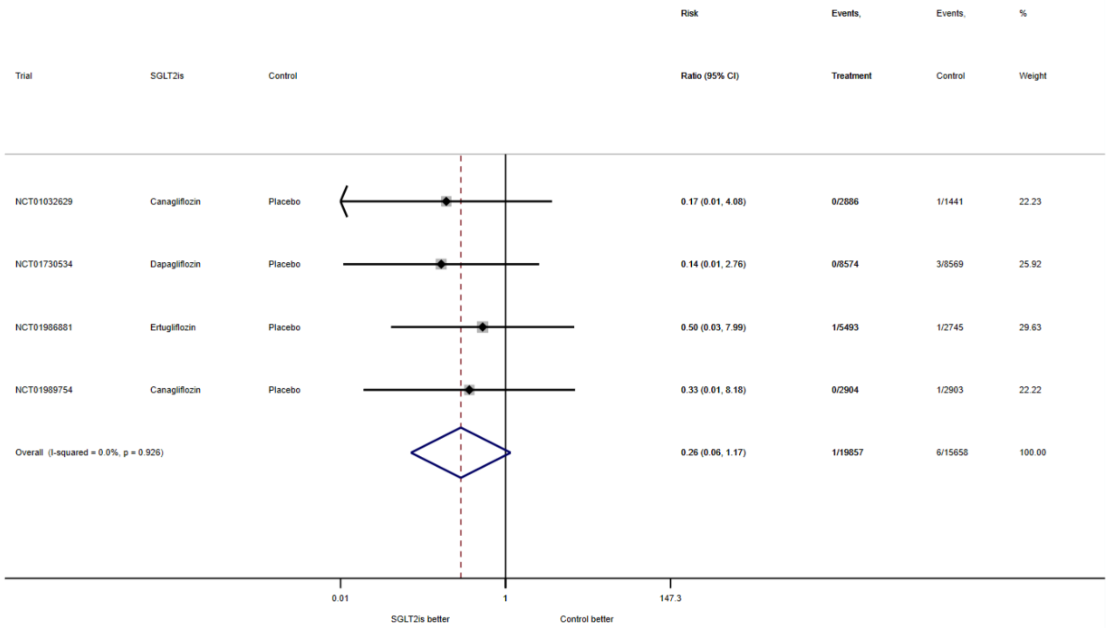

System 15-Musculoskeletal And Connective Tissue Disorders-Figure S46 Meta-analysis of SGLT2is and Synovial cyst

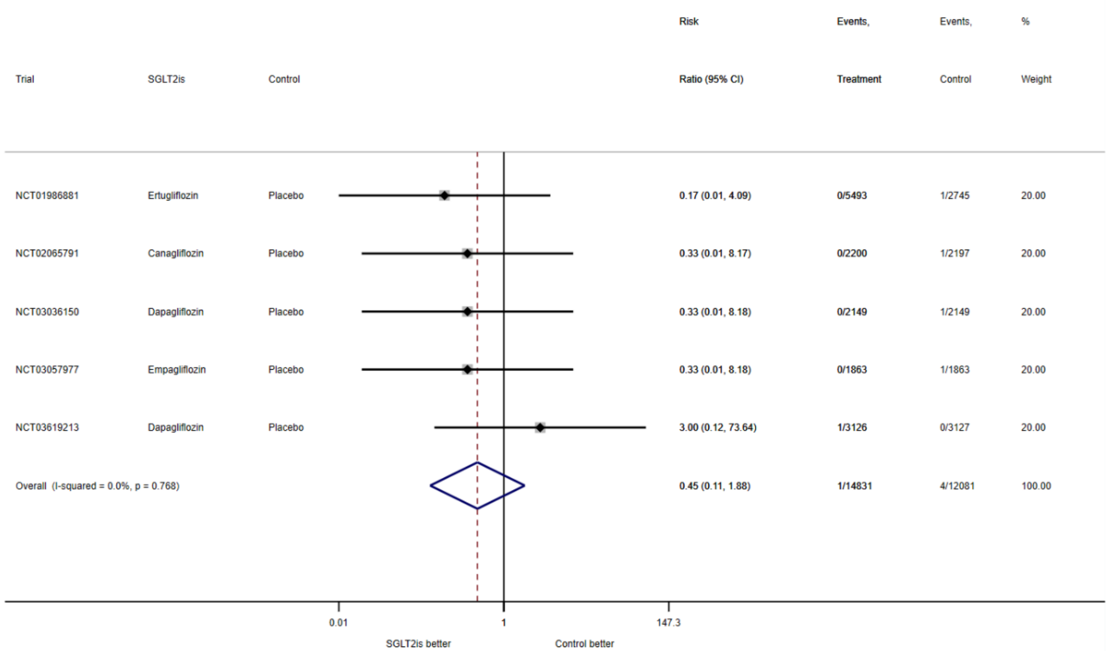

System 15-Musculoskeletal And Connective Tissue Disorders-Figure S47 Meta-analysis of SGLT2is and Systemic lupus erythematosus

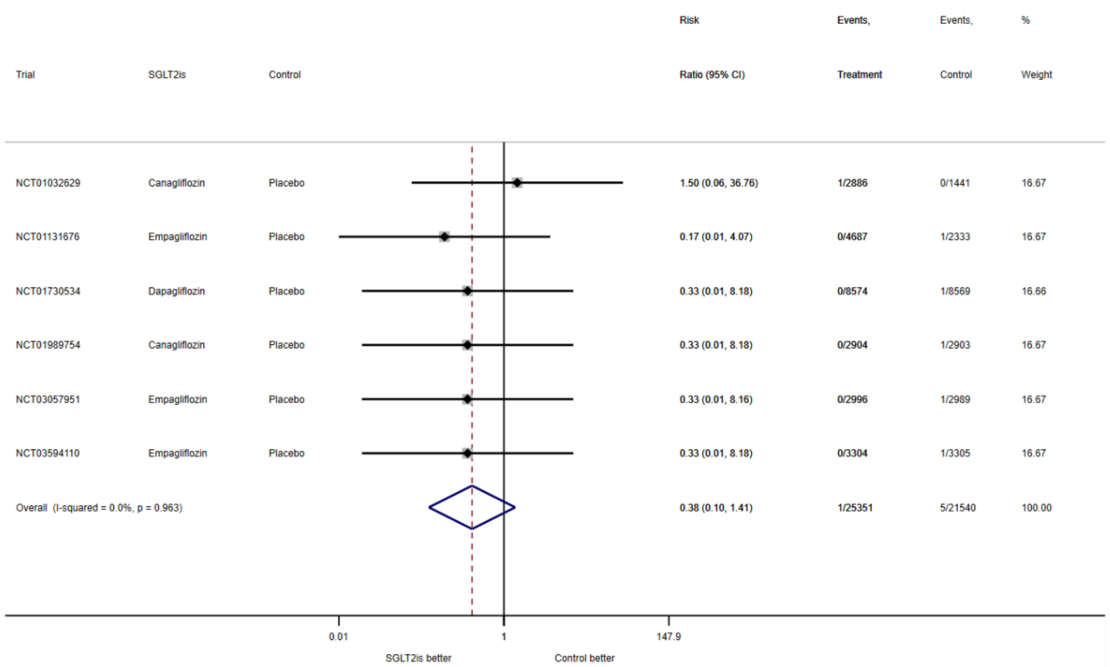

System 15-Musculoskeletal And Connective Tissue Disorders-Figure S48 Meta-analysis of SGLT2is and Tendonitis

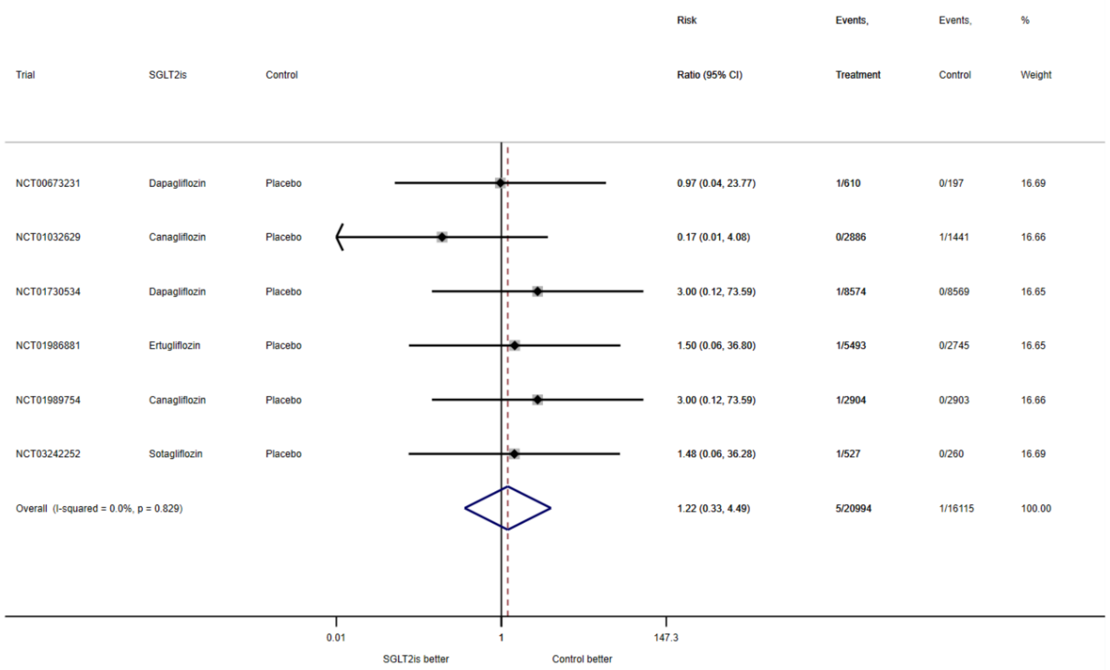

System 15-Musculoskeletal And Connective Tissue Disorders-Figure S49 Meta-analysis of SGLT2is and Tenosynovitis

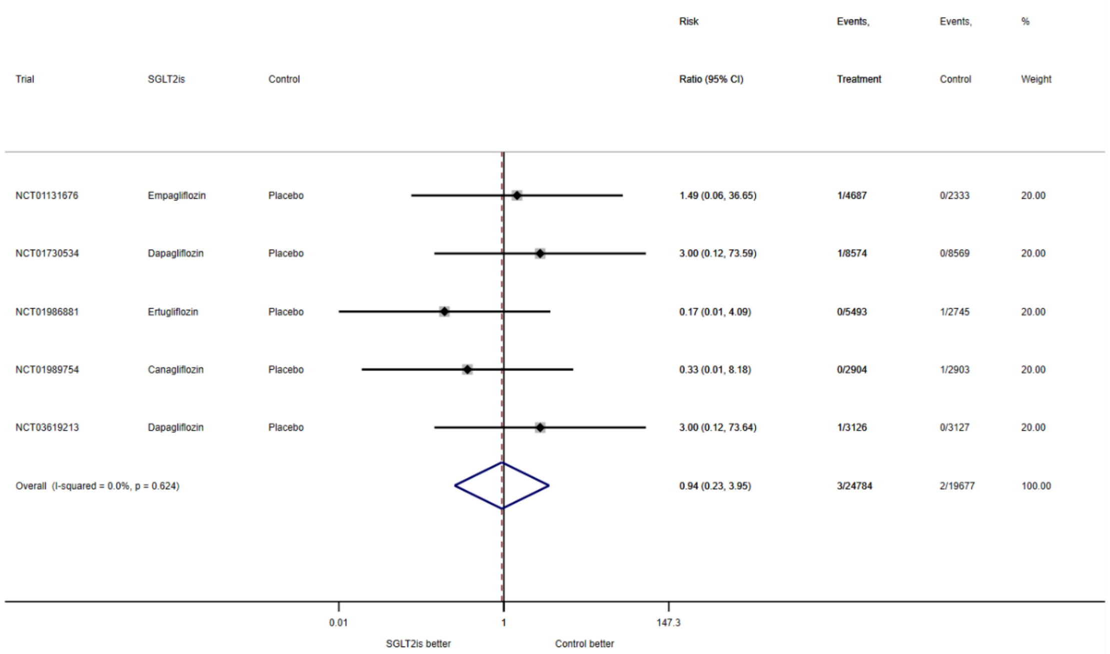

System 15-Musculoskeletal And Connective Tissue Disorders-Figure S50 Meta-analysis of SGLT2is and Tenosynovitis stenosans

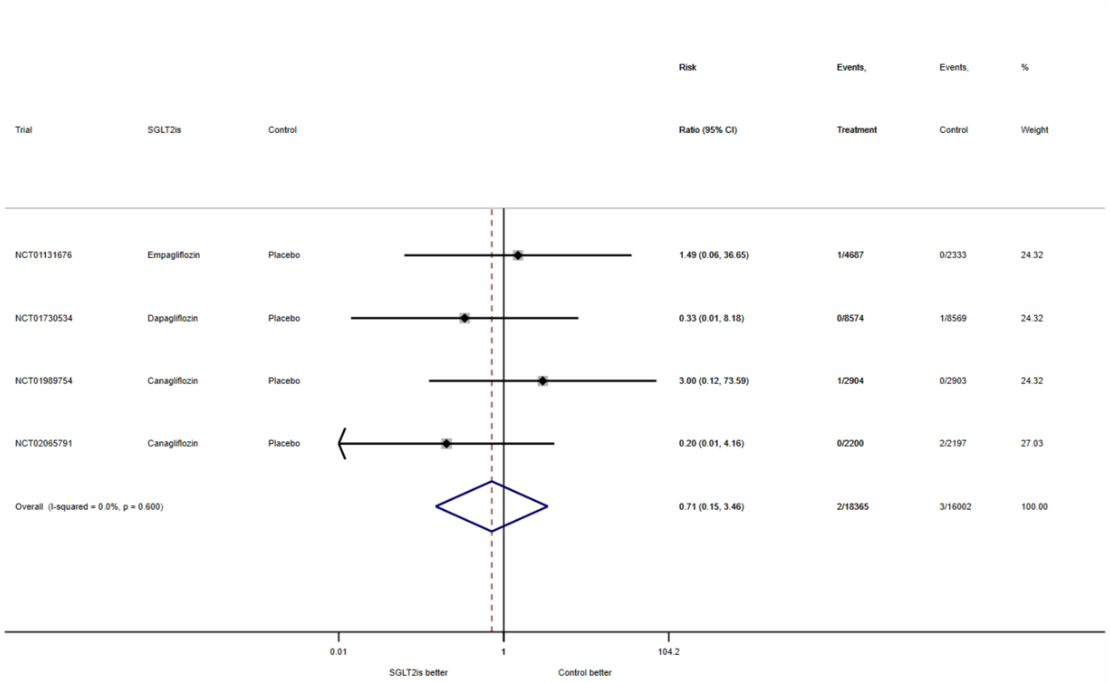

System 15-Musculoskeletal And Connective Tissue Disorders-Figure S51 Meta-analysis of SGLT2is and Trigger finger

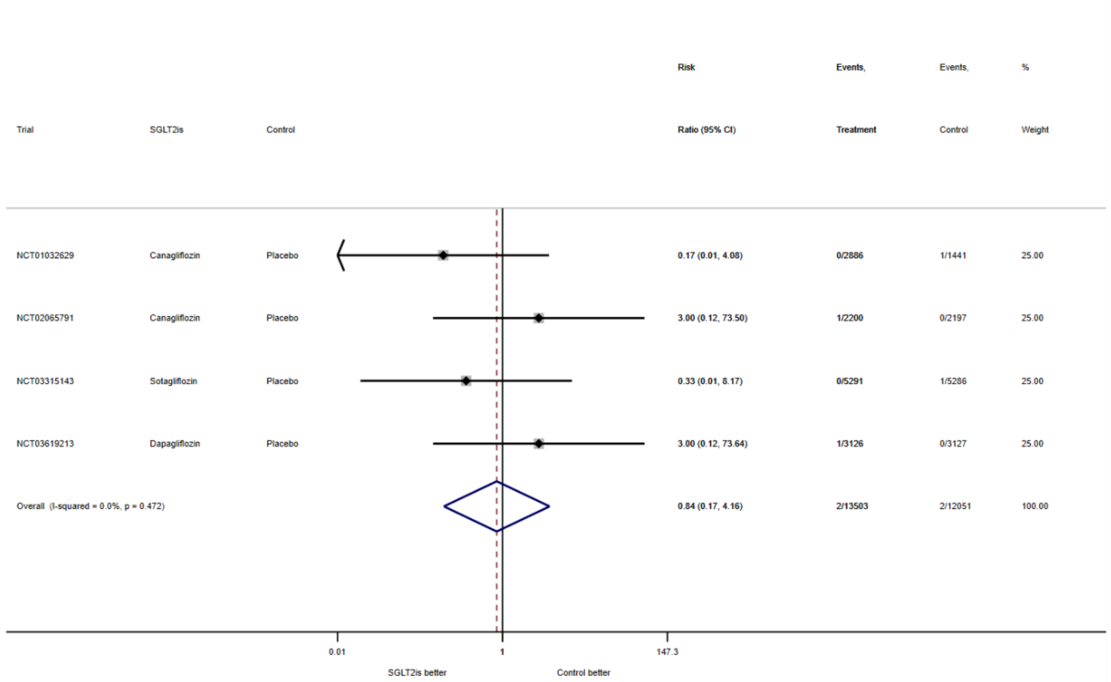

System 16-Neoplasms Benign, Malignant And Unspecified-Figure S1 Meta-analysis of SGLT2is and Acute leukaemia

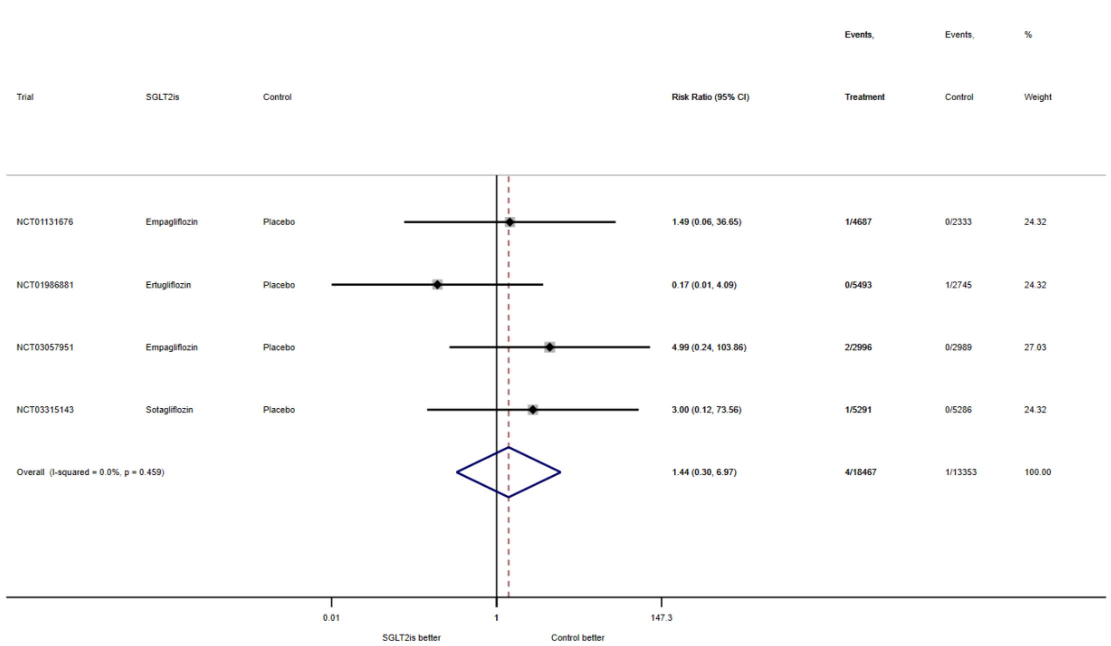

System 16-Neoplasms Benign, Malignant And Unspecified-Figure S2 Meta-analysis of SGLT2is and Acute lymphocytic leukaemia

Trial	SGLT2is	Control	Risk Ratio (95% CI)	Events, Treatment	Events, Control	% Weight
NCT01131676	Empagliflozin	Placebo	1.49 (0.06, 36.65)	1/4687	0/2333	9.18
NCT01730534	Dapagliflozin	Placebo	2.00 (0.37, 10.91)	4/8574	2/8569	32.65
NCT01986881	Ertugliflozin	Placebo	1.50 (0.06, 36.80)	1/5493	0/2745	9.18
NCT01989754	Canagliflozin	Placebo	0.33 (0.01, 8.18)	0/2904	1/2903	9.18
NCT03036124	Dapagliflozin	Placebo	5.00 (0.24, 104.09)	2/2368	0/2368	10.20
NCT03036150	Dapagliflozin	Placebo	0.20 (0.01, 4.16)	0/2149	2/2149	10.21
NCT03594110	Empagliflozin	Placebo	3.00 (0.12, 73.64)	1/3304	0/3305	9.18
NCT03619213	Dapagliflozin	Placebo	0.20 (0.01, 4.17)	0/3126	2/3127	10.20
Overall (I-squared = 0.0%, p = 0.681)			1.15 (0.43, 3.02)	9/32605	7/27499	100.00

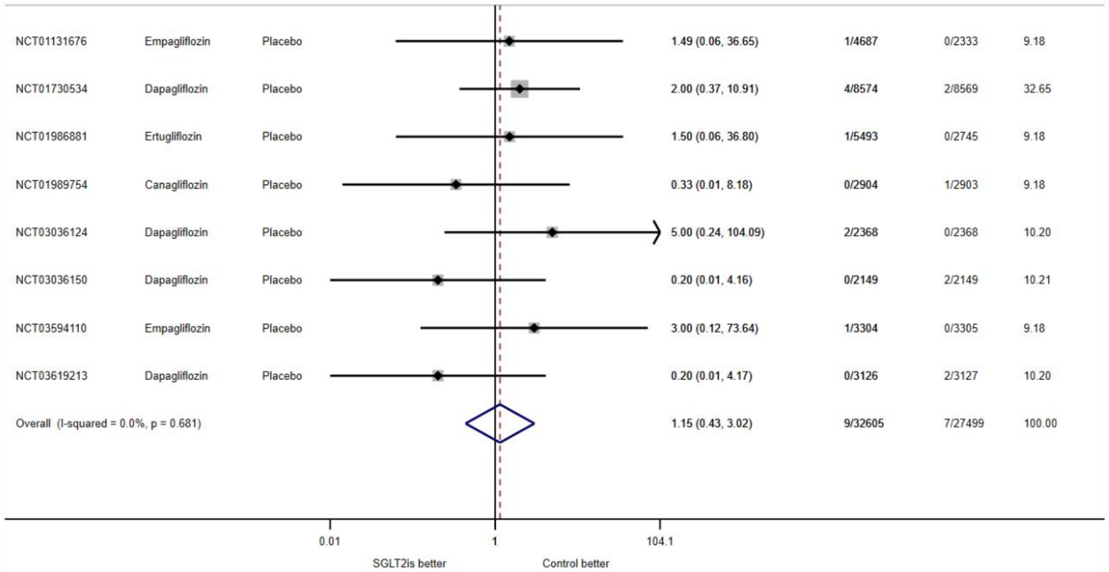

System 16-Neoplasms Benign, Malignant And Unspecified-Figure S3 Meta-analysis of SGLT2is and Acute myeloid leukaemia

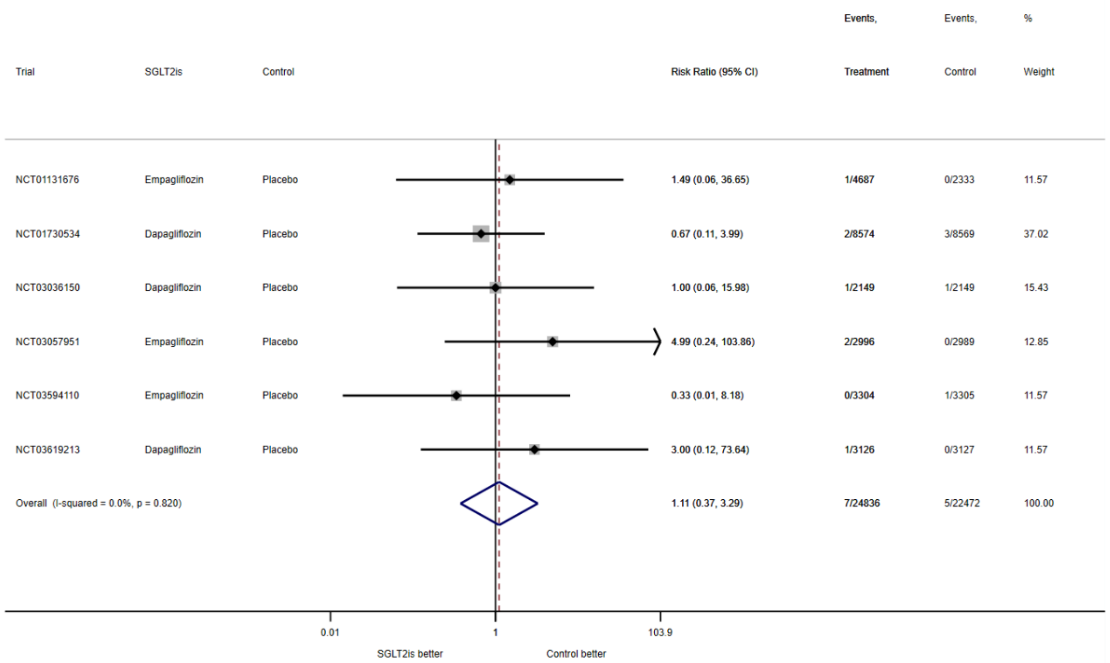

System 16-Neoplasms Benign, Malignant And Unspecified-Figure S4 Meta-analysis of SGLT2is and Adenocarcinoma

Trial	SGLT2is	Control	Risk Ratio (95% CI)	Events, Treatment	Events, Control	% Weight
NCT01032629	Canagliflozin	Placebo	4.50 (0.24, 83.44)	4/2886	0/1441	7.37
NCT01131676	Empagliflozin	Placebo	0.25 (0.02, 2.74)	1/4687	2/2333	10.92
NCT01730534	Dapagliflozin	Placebo	1.00 (0.20, 4.95)	3/8574	3/8569	24.56
NCT01986881	Ertugliflozin	Placebo	4.50 (0.24, 83.52)	4/5493	0/2745	7.37
NCT01989754	Canagliflozin	Placebo	3.00 (0.12, 73.59)	1/2904	0/2903	6.14
NCT02065791	Canagliflozin	Placebo	0.33 (0.01, 8.17)	0/2200	1/2197	6.14
NCT03036150	Dapagliflozin	Placebo	0.33 (0.01, 8.18)	0/2149	1/2149	6.14
NCT03057951	Empagliflozin	Placebo	2.99 (0.12, 73.44)	1/2996	0/2989	6.14
NCT03057977	Empagliflozin	Placebo	0.33 (0.01, 8.18)	0/1863	1/1863	6.14
NCT03315143	Sotagliflozin	Placebo	0.50 (0.05, 5.51)	1/5291	2/5286	10.91
NCT03619213	Dapagliflozin	Placebo	1.00 (0.06, 15.99)	1/3126	1/3127	8.19
Overall (I-squared = 0.0%, p = 0.826)			0.93 (0.42, 2.05)	16/42169	11/35602	100.00

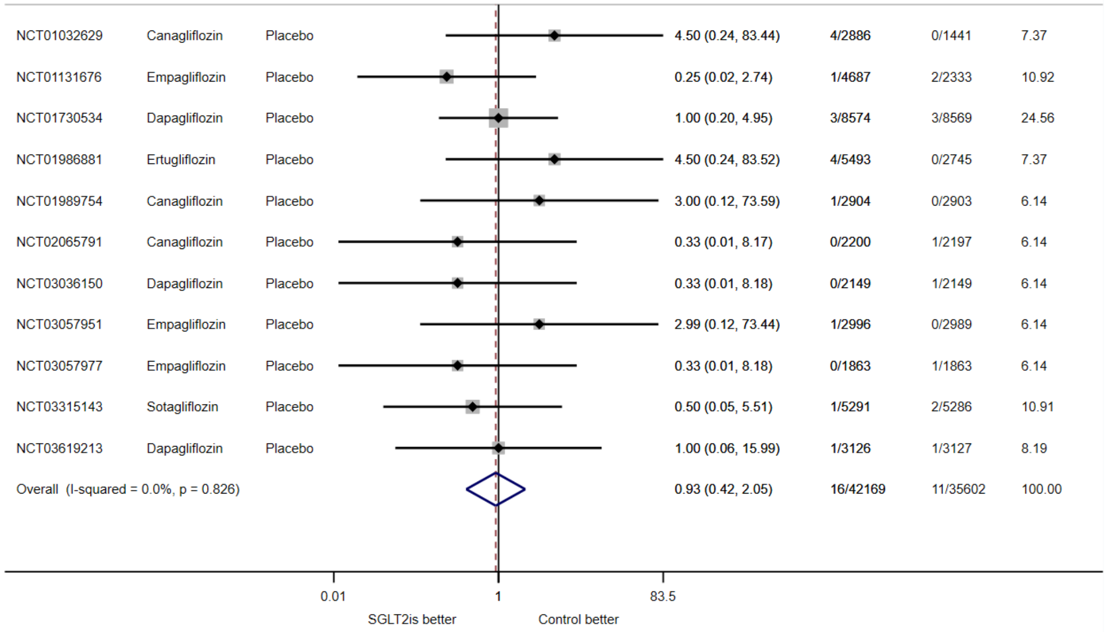

System 16-Neoplasms Benign, Malignant And Unspecified-Figure S5 Meta-analysis of SGLT2is and Adenocarcinoma gastric

Trial	SGLT2is	Control	Risk Ratio (95% CI)	Events, Treatment	Events, Control	% Weight
NCT01032629	Canagliflozin	Placebo	1.00 (0.18, 5.45)	4/2886	2/1441	5.36
NCT01131676	Empagliflozin	Placebo	1.00 (0.18, 5.43)	4/4687	2/2333	5.36
NCT01730534	Dapagliflozin	Placebo	1.11 (0.59, 2.10)	20/8574	18/8569	38.12
NCT01986881	Ertugliflozin	Placebo	0.50 (0.14, 1.72)	5/5493	5/2745	10.05
NCT01989754	Canagliflozin	Placebo	0.33 (0.03, 3.20)	1/2904	3/2903	3.01
NCT02065791	Canagliflozin	Placebo	2.00 (0.18, 22.01)	2/2200	1/2197	2.68
NCT03036124	Dapagliflozin	Placebo	1.00 (0.14, 7.09)	2/2368	2/2368	4.02
NCT03036150	Dapagliflozin	Placebo	1.50 (0.25, 8.97)	3/2149	2/2149	4.82
NCT03057951	Empagliflozin	Placebo	1.00 (0.20, 4.94)	3/2996	3/2989	6.03
NCT03057977	Empagliflozin	Placebo	1.00 (0.14, 7.09)	2/1863	2/1863	4.02
NCT03315143	Sotagliflozin	Placebo	0.75 (0.17, 3.35)	3/5291	4/5286	6.89
NCT03619213	Dapagliflozin	Placebo	1.50 (0.42, 5.31)	6/3126	4/3127	9.65
Overall (I-squared = 0.0%, p = 0.987)			0.99 (0.67, 1.47)	55/44537	48/37970	100.00

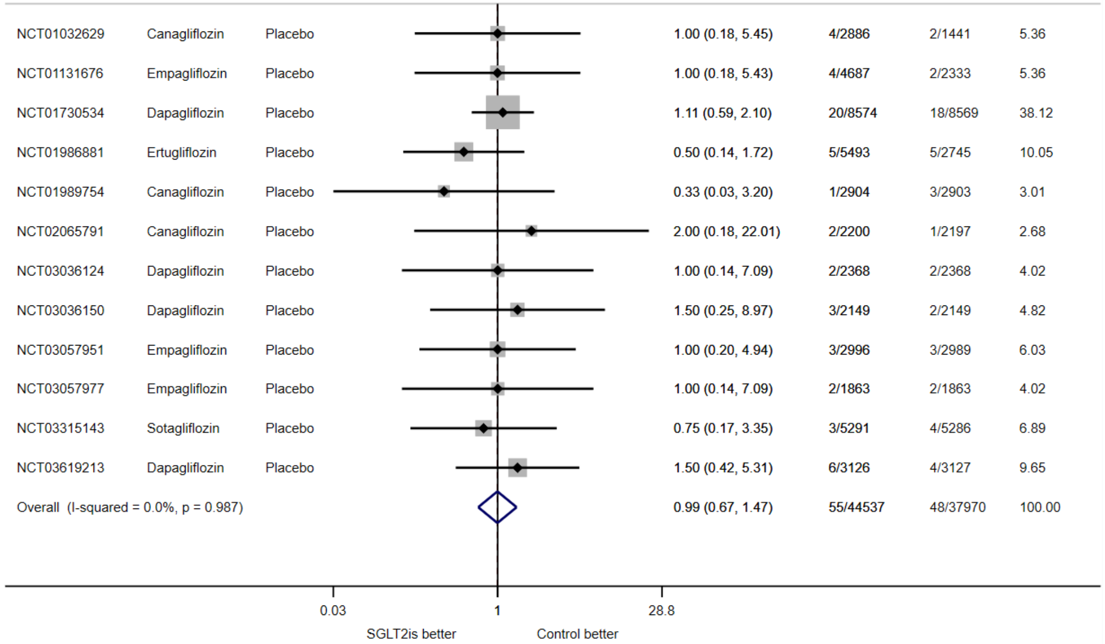

System 16-Neoplasms Benign, Malignant And Unspecified-Figure S6 Meta-analysis of SGLT2is and Adenocarcinoma of colon

Trial	SGLT2is	Control	Risk Ratio (95% CI)	Events, Treatment	Events, Control	% Weight
NCT01131676	Empagliflozin	Placebo	1.49 (0.30, 7.39)	6/4687	2/2333	22.19
NCT01730534	Dapagliflozin	Placebo	0.80 (0.21, 2.98)	4/8574	5/8569	32.85
NCT01986881	Ertugliflozin	Placebo	1.00 (0.09, 11.02)	2/5493	1/2745	9.85
NCT03036150	Dapagliflozin	Placebo	1.00 (0.06, 15.98)	1/2149	1/2149	7.39
NCT03057951	Empagliflozin	Placebo	2.99 (0.12, 73.44)	1/2996	0/2989	5.54
NCT03057977	Empagliflozin	Placebo	0.20 (0.01, 4.16)	0/1863	2/1863	6.16
NCT03594110	Empagliflozin	Placebo	5.00 (0.24, 104.14)	2/3304	0/3305	6.16
NCT03619213	Dapagliflozin	Placebo	0.50 (0.05, 5.51)	1/3126	2/3127	9.86
Overall (I-squared = 0.0%, p = 0.858)			1.01 (0.47, 2.14)	17/32192	13/27080	100.00

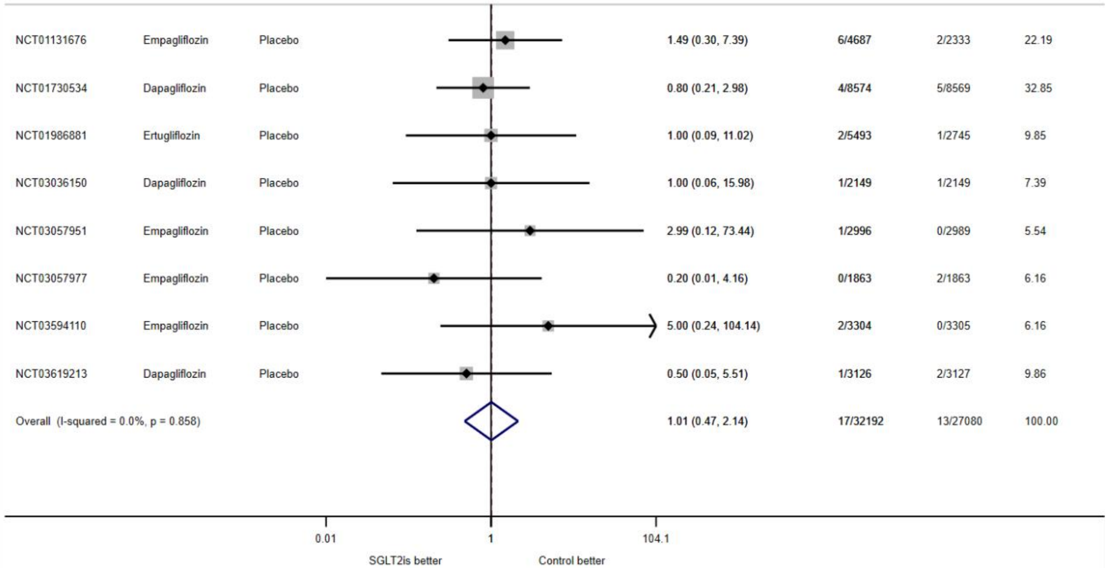

System 16-Neoplasms Benign, Malignant And Unspecified-Figure S7 Meta-analysis of SGLT2is and Adenocarcinoma pancreas

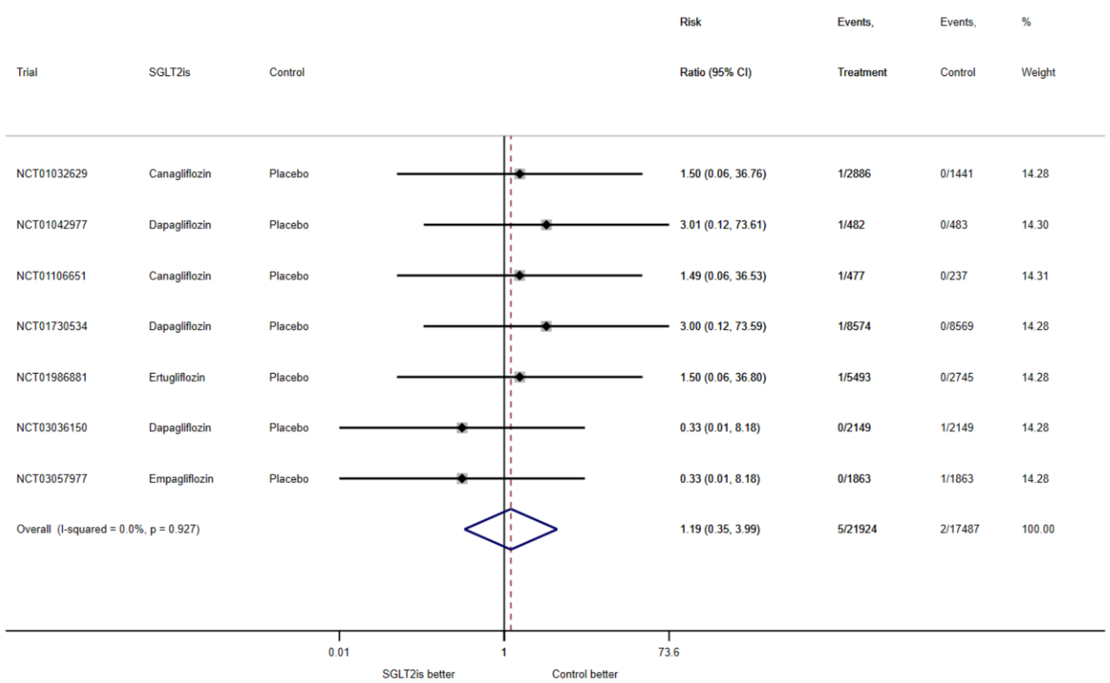

System 16-Neoplasms Benign, Malignant And Unspecified-Figure S8 Meta-analysis of SGLT2is and Adrenal adenoma

Trial	SGLT2is	Control	Risk Ratio (95% CI)	Events, Treatment	Events, Control	% Weight
NCT01131676	Empagliflozin	Placebo	0.17 (0.01, 4.07)	0/4687	1/2333	10.00
NCT01730534	Dapagliflozin	Placebo	5.00 (0.24, 104.07)	2/8574	0/8569	11.11
NCT01986881	Ertugliflozin	Placebo	1.00 (0.09, 11.02)	2/5493	1/2745	17.78
NCT01989754	Canagliflozin	Placebo	0.20 (0.01, 4.16)	0/2904	2/2903	11.11
NCT02065791	Canagliflozin	Placebo	3.00 (0.12, 73.50)	1/2200	0/2197	10.00
NCT03036150	Dapagliflozin	Placebo	0.33 (0.01, 8.18)	0/2149	1/2149	10.00
NCT03242252	Sotagliflozin	Placebo	1.48 (0.06, 36.28)	1/527	0/260	10.02
NCT03315143	Sotagliflozin	Placebo	3.00 (0.12, 73.56)	1/5291	0/5286	10.00
NCT03619213	Dapagliflozin	Placebo	3.00 (0.12, 73.64)	1/3126	0/3127	10.00
Overall (I-squared = 0.0%, p = 0.735)			1.08 (0.39, 2.98)	8/34951	5/29569	100.00

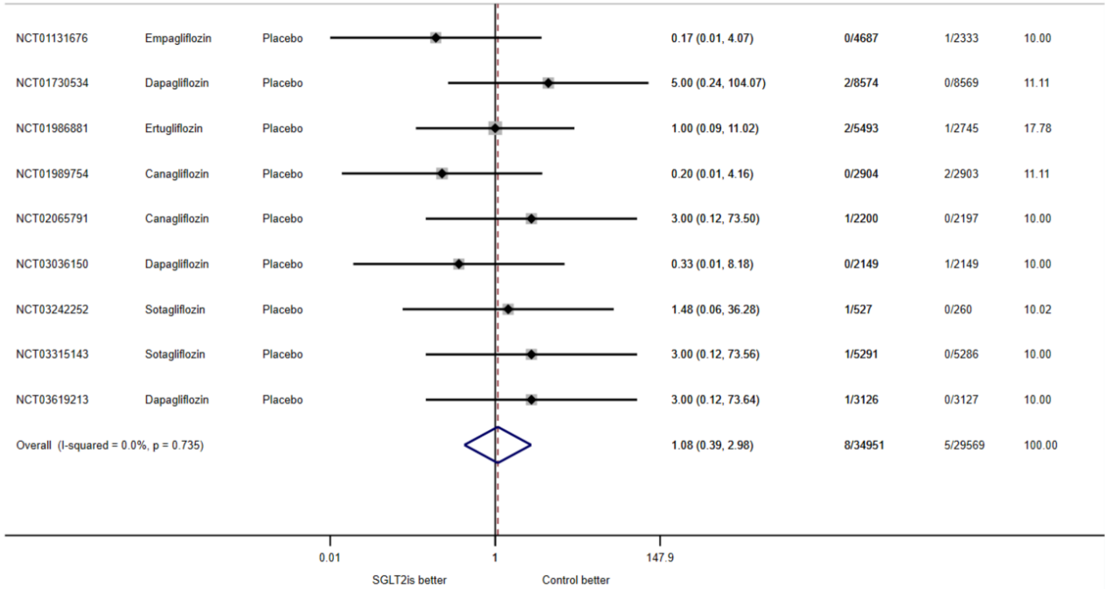

System 16-Neoplasms Benign, Malignant And Unspecified-Figure S9 Meta-analysis of SGLT2is and B-cell lymphoma

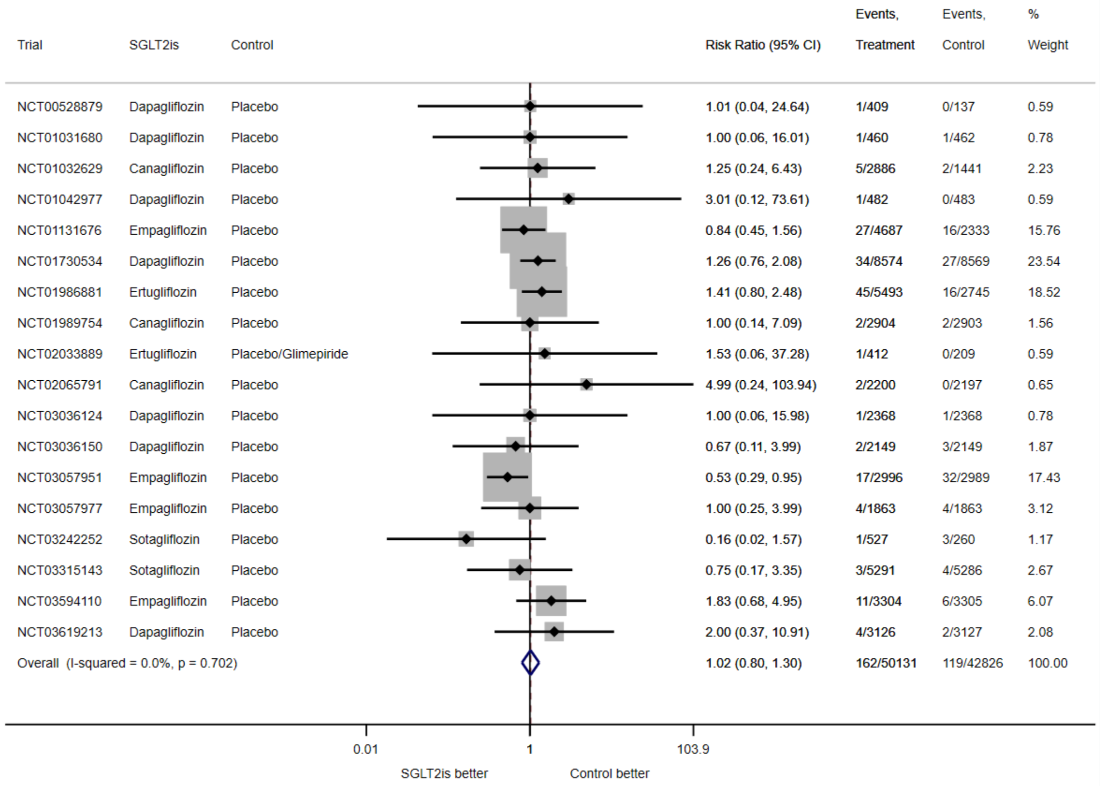

System 16-Neoplasms Benign, Malignant And Unspecified-Figure S10 Meta-analysis of SGLT2is and Basal cell carcinoma

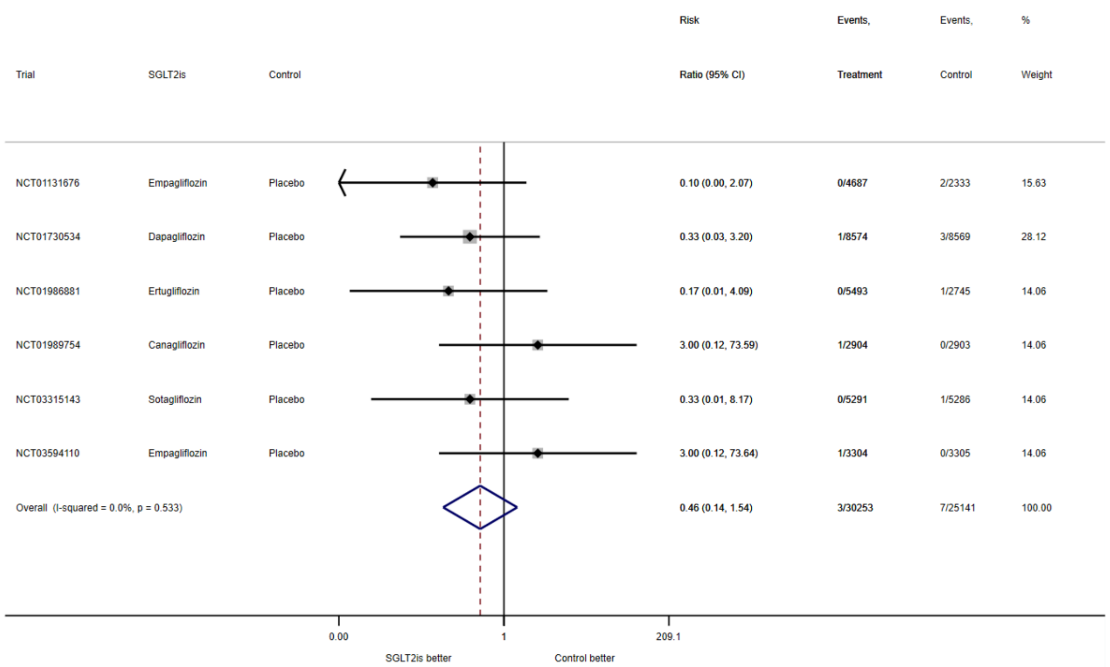

System 16-Neoplasms Benign, Malignant And Unspecified-Figure S11 Meta-analysis of SGLT2is and Benign lung neoplasm

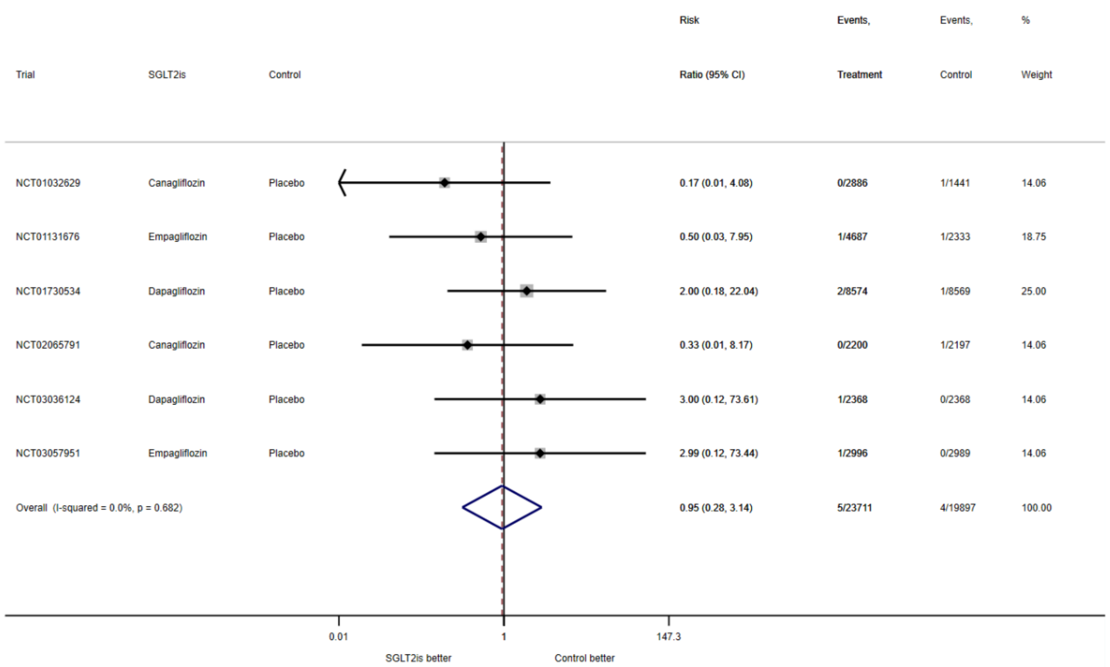

System 16-Neoplasms Benign, Malignant And Unspecified-Figure S12 Meta-analysis of SGLT2is and Benign neoplasm of bladder

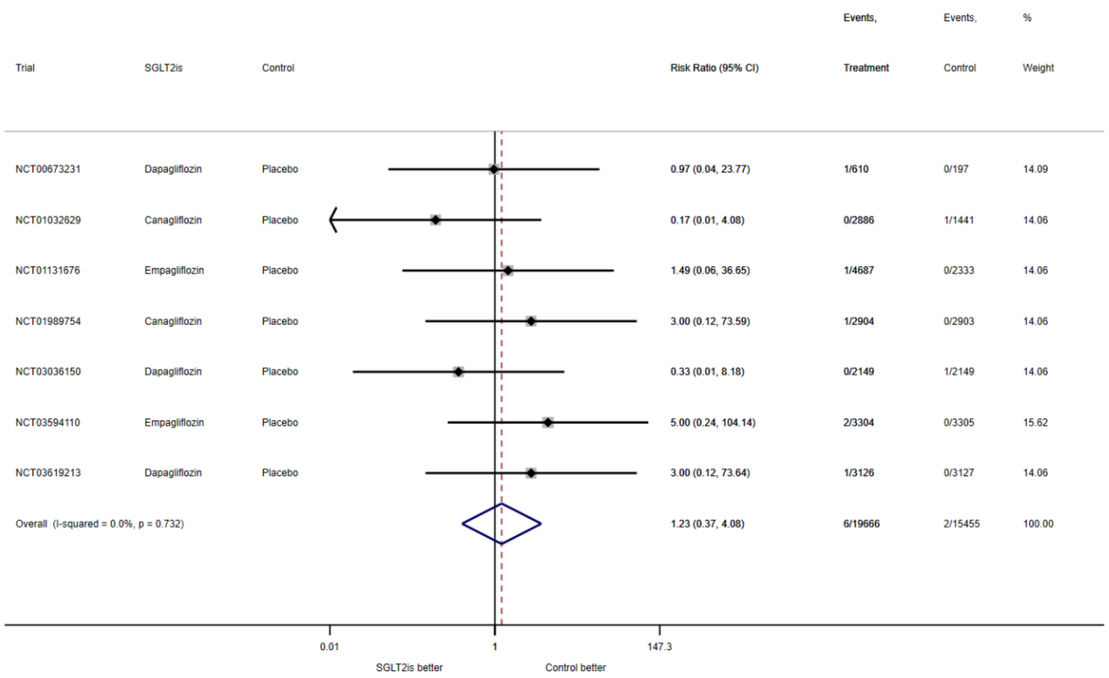

System 16-Neoplasms Benign, Malignant And Unspecified-Figure S13 Meta-analysis of SGLT2is and Bile duct cancer

Trial	SGLT2is	Control	Risk Ratio (95% CI)	Events, Treatment	Events, Control	% Weight
NCT01032629	Canagliflozin	Placebo	0.40 (0.11, 1.49)	4/2886	5/1441	8.86
NCT01042977	Dapagliflozin	Placebo	0.33 (0.01, 8.18)	0/482	1/483	1.49
NCT01131676	Empagliflozin	Placebo	6.47 (0.36, 114.84)	6/4687	0/2333	1.85
NCT01730534	Dapagliflozin	Placebo	0.48 (0.24, 0.95)	12/8574	25/8569	32.30
NCT01986881	Ertugliflozin	Placebo	2.25 (0.49, 10.40)	9/5493	2/2745	6.51
NCT01989754	Canagliflozin	Placebo	1.00 (0.25, 3.99)	4/2904	4/2903	7.96
NCT02065791	Canagliflozin	Placebo	1.25 (0.34, 4.64)	5/2200	4/2197	8.85
NCT03036124	Dapagliflozin	Placebo	0.50 (0.05, 5.51)	1/2368	2/2368	2.65
NCT03036150	Dapagliflozin	Placebo	0.33 (0.03, 3.20)	1/2149	3/2149	2.98
NCT03057951	Empagliflozin	Placebo	1.33 (0.30, 5.94)	4/2996	3/2989	6.82
NCT03057977	Empagliflozin	Placebo	0.33 (0.03, 3.20)	1/1863	3/1863	2.98
NCT03315143	Sotagliflozin	Placebo	1.00 (0.14, 7.09)	2/5291	2/5286	3.98
NCT03594110	Empagliflozin	Placebo	1.50 (0.42, 5.31)	6/3304	4/3305	9.56
NCT03619213	Dapagliflozin	Placebo	0.25 (0.03, 2.24)	1/3126	4/3127	3.18
Overall (I-squared = 0.0%, p = 0.570)			0.74 (0.50, 1.10)	56/48323	62/41758	100.00

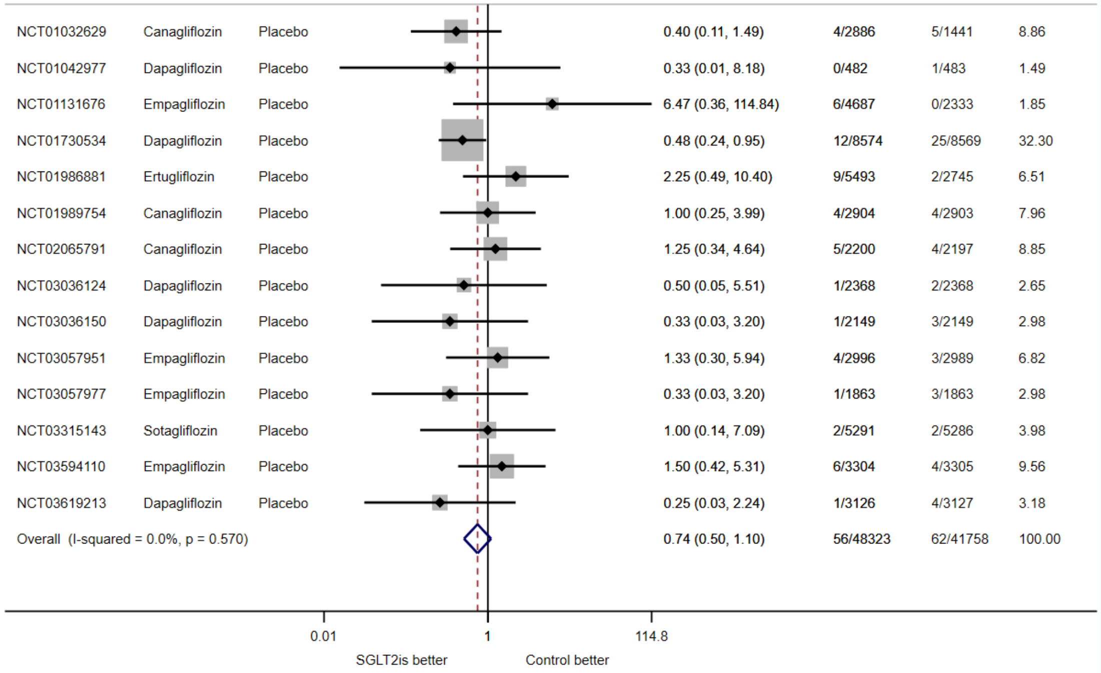

System 16-Neoplasms Benign, Malignant And Unspecified-Figure S14 Meta-analysis of SGLT2is and Bladder cancer

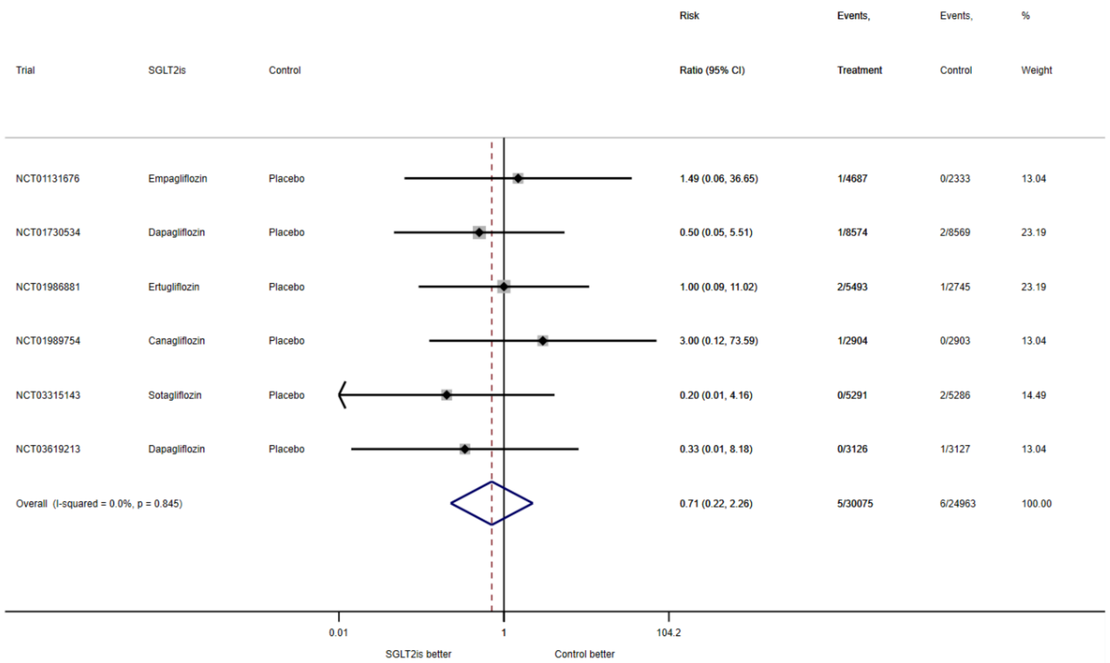

System 16-Neoplasms Benign, Malignant And Unspecified-Figure S15 Meta-analysis of SGLT2is and Bladder cancer recurrent

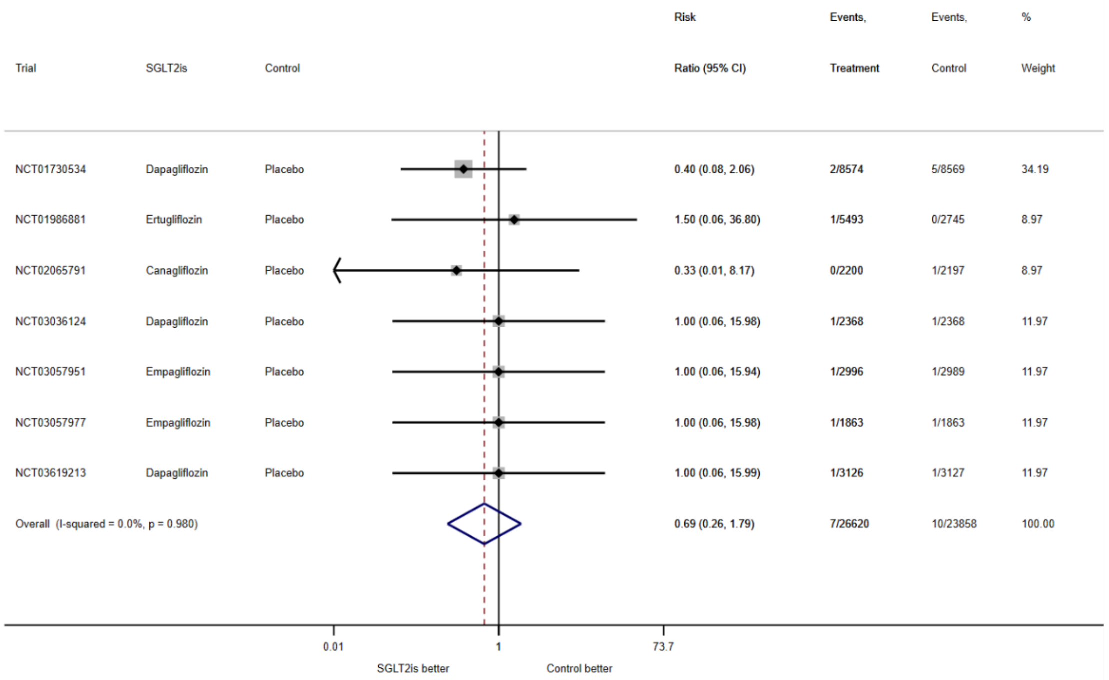

System 16-Neoplasms Benign, Malignant And Unspecified-Figure S16 Meta-analysis of SGLT2is and Bladder neoplasm

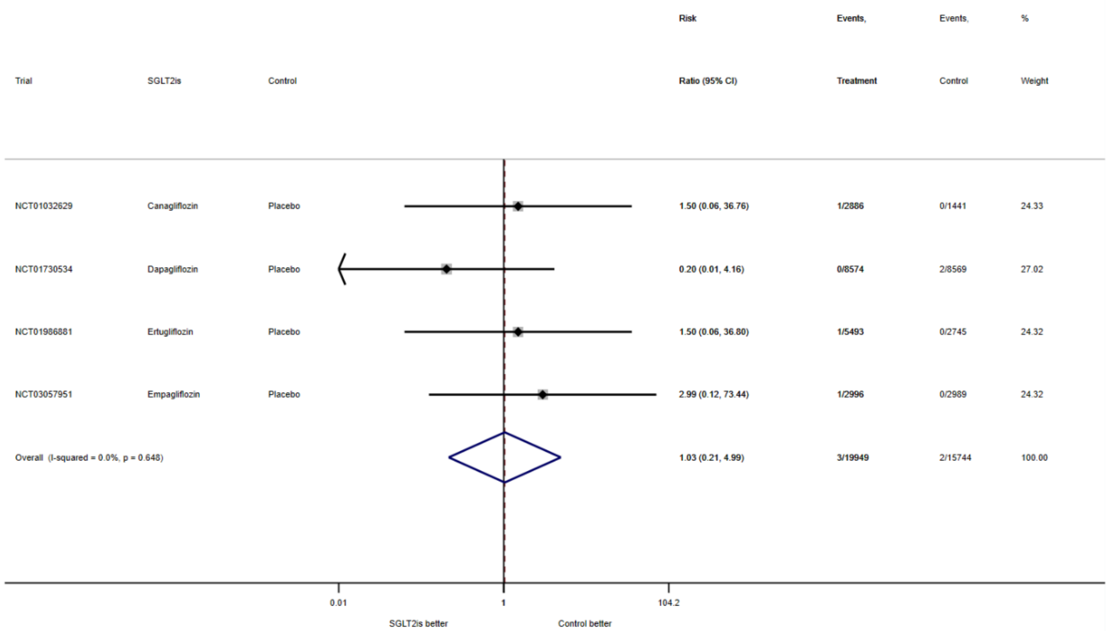

System 16-Neoplasms Benign, Malignant And Unspecified-Figure S17 Meta-analysis of SGLT2is and Bladder papilloma

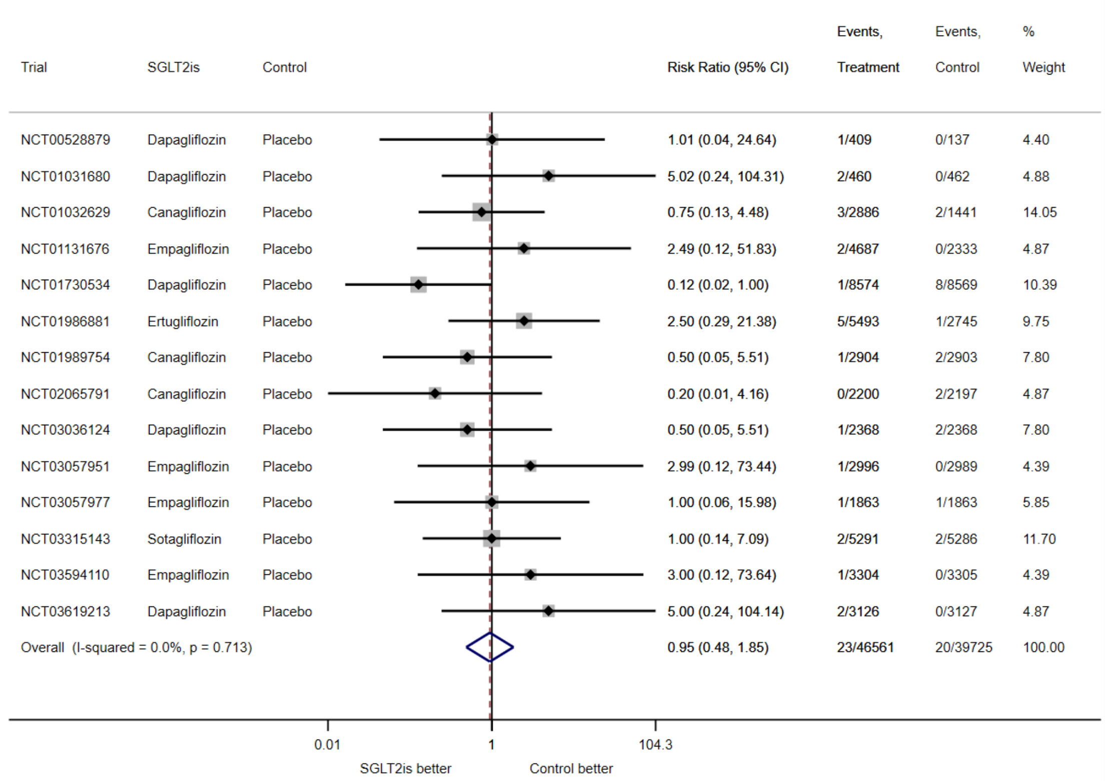

System 16-Neoplasms Benign, Malignant And Unspecified-Figure S18 Meta-analysis of SGLT2is and Bladder transitional cell carcinoma

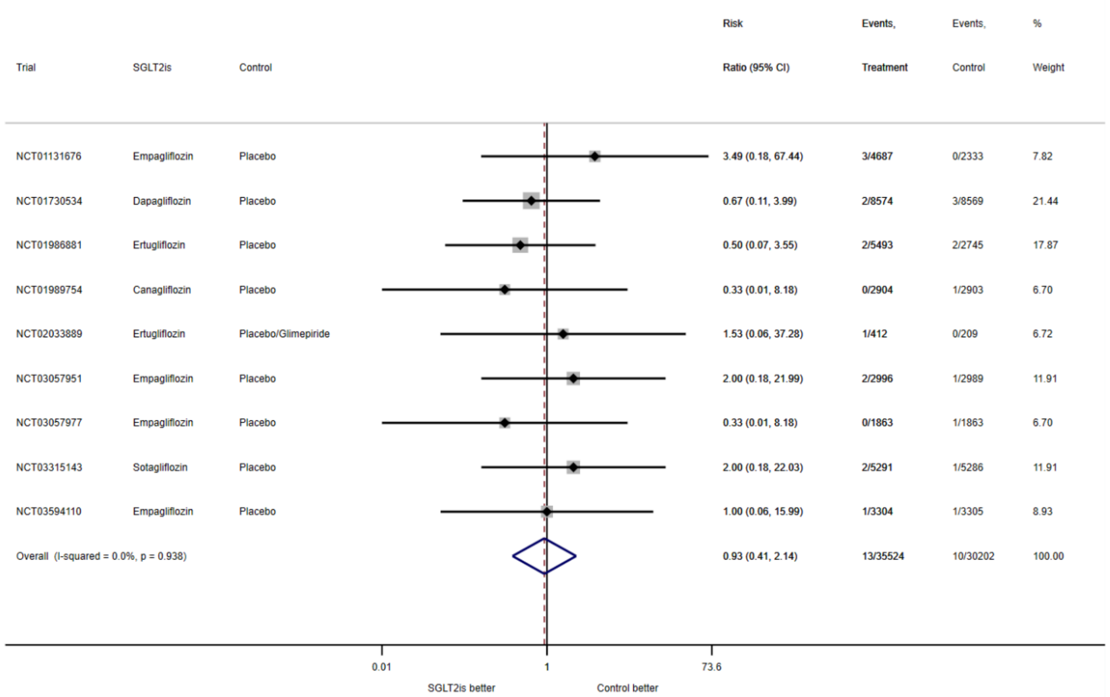

System 16-Neoplasms Benign, Malignant And Unspecified-Figure S19 Meta-analysis of SGLT2is and Bowen's disease

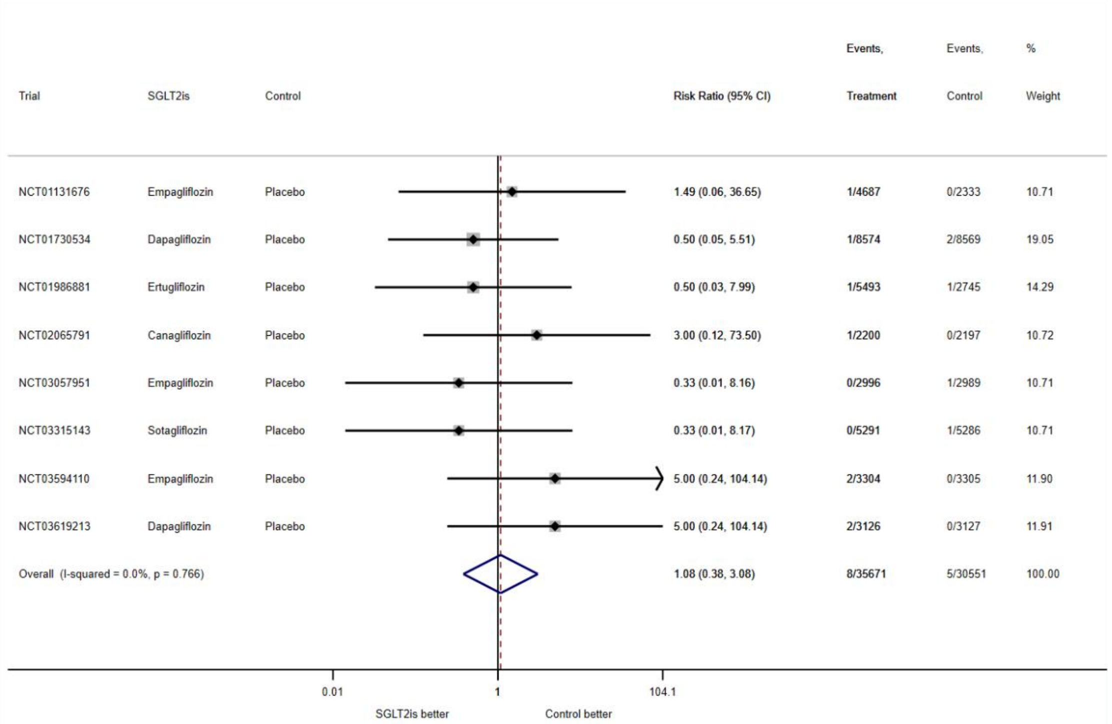

System 16-Neoplasms Benign, Malignant And Unspecified-Figure S20 Meta-analysis of SGLT2is and Brain neoplasm

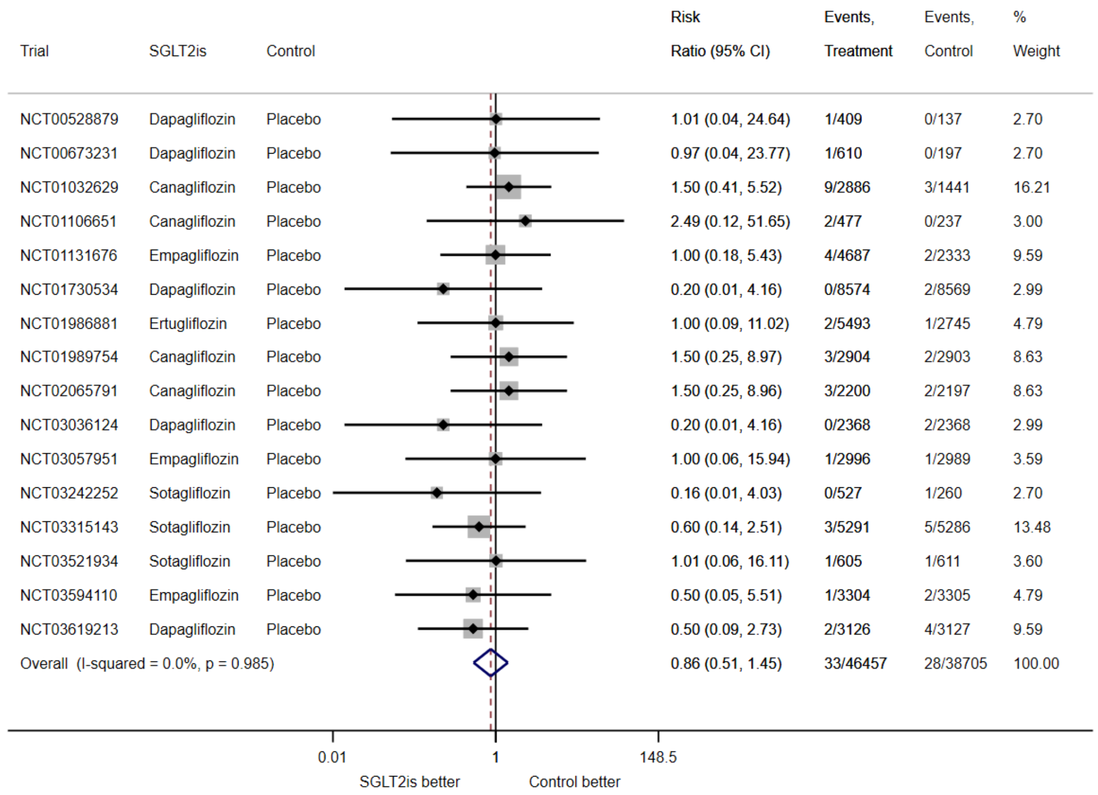

System 16-Neoplasms Benign, Malignant And Unspecified-Figure S21 Meta-analysis of SGLT2is and Breast cancer

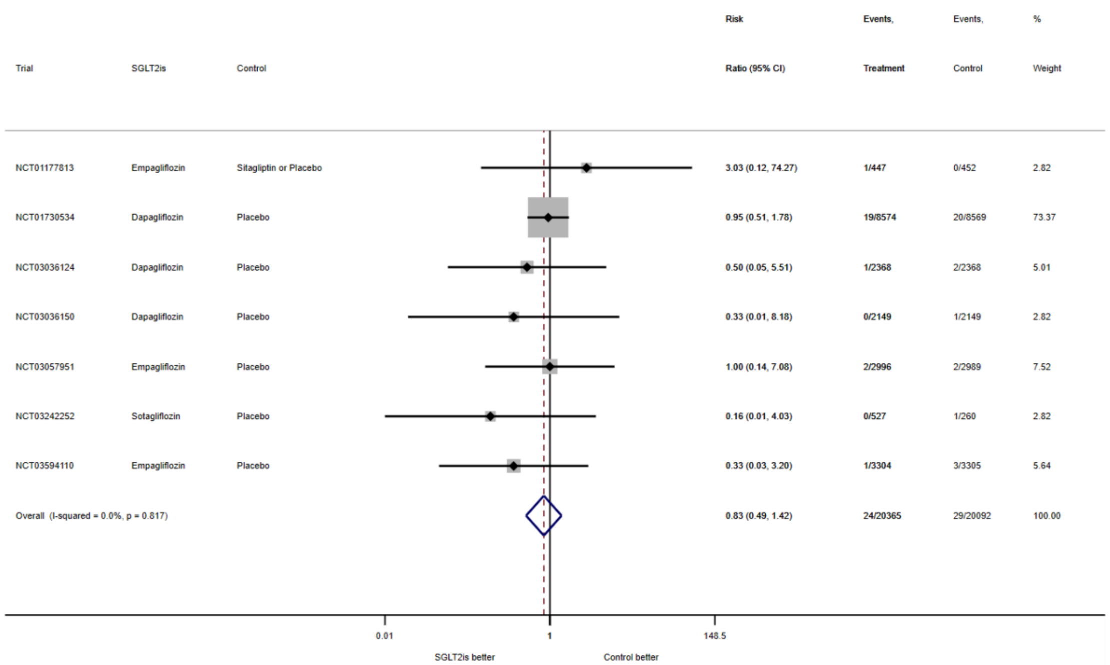

System 16-Neoplasms Benign, Malignant And Unspecified-Figure S22 Meta-analysis of SGLT2is and Breast cancer female

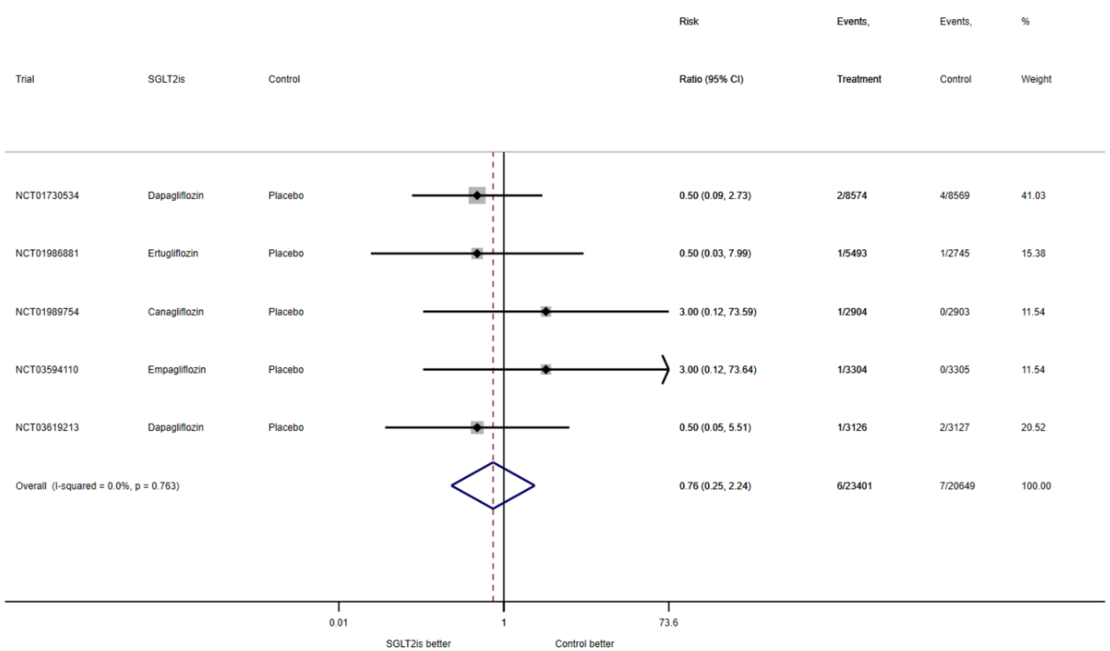

System 16-Neoplasms Benign, Malignant And Unspecified-Figure S23 Meta-analysis of SGLT2is and Breast cancer metastatic

Trial	SGLT2is	Control	Risk Ratio (95% CI)	Events, Treatment	Events, Control	% Weight
NCT01032629	Canagliflozin	Placebo	0.10 (0.00, 2.08)	0/2886	2/1441	10.42
NCT01106677	Canagliflozin	Sitagliptin	1.50 (0.06, 36.63)	1/735	0/366	9.39
NCT01131676	Empagliflozin	Placebo	1.49 (0.06, 36.65)	1/4687	0/2333	9.37
NCT01730534	Dapagliflozin	Placebo	0.50 (0.05, 5.51)	1/8574	2/8569	16.66
NCT01986881	Ertugliflozin	Placebo	1.50 (0.06, 36.80)	1/5493	0/2745	9.37
NCT03036150	Dapagliflozin	Placebo	3.00 (0.12, 73.60)	1/2149	0/2149	9.37
NCT03057951	Empagliflozin	Placebo	2.00 (0.18, 21.99)	2/2996	1/2989	16.67
NCT03594110	Empagliflozin	Placebo	3.00 (0.12, 73.64)	1/3304	0/3305	9.37
NCT03619213	Dapagliflozin	Placebo	0.33 (0.01, 8.18)	0/3126	1/3127	9.37
Overall (I-squared = 0.0%, p = 0.820)			0.98 (0.37, 2.60)	8/33950	6/27024	100.00

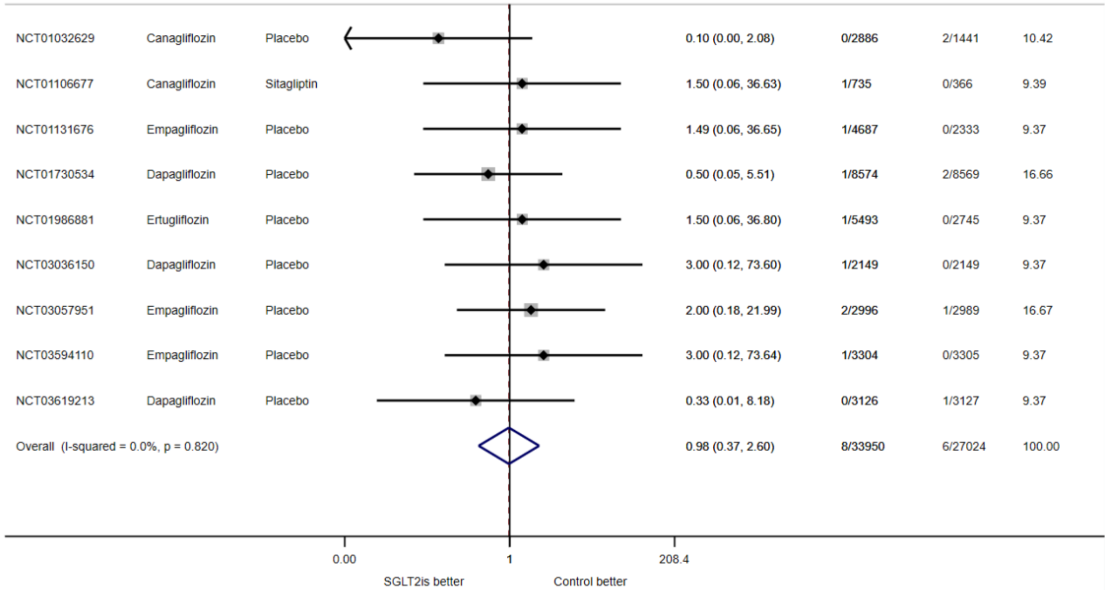

System 16-Neoplasms Benign, Malignant And Unspecified-Figure S24 Meta-analysis of SGLT2is and Bronchial carcinoma

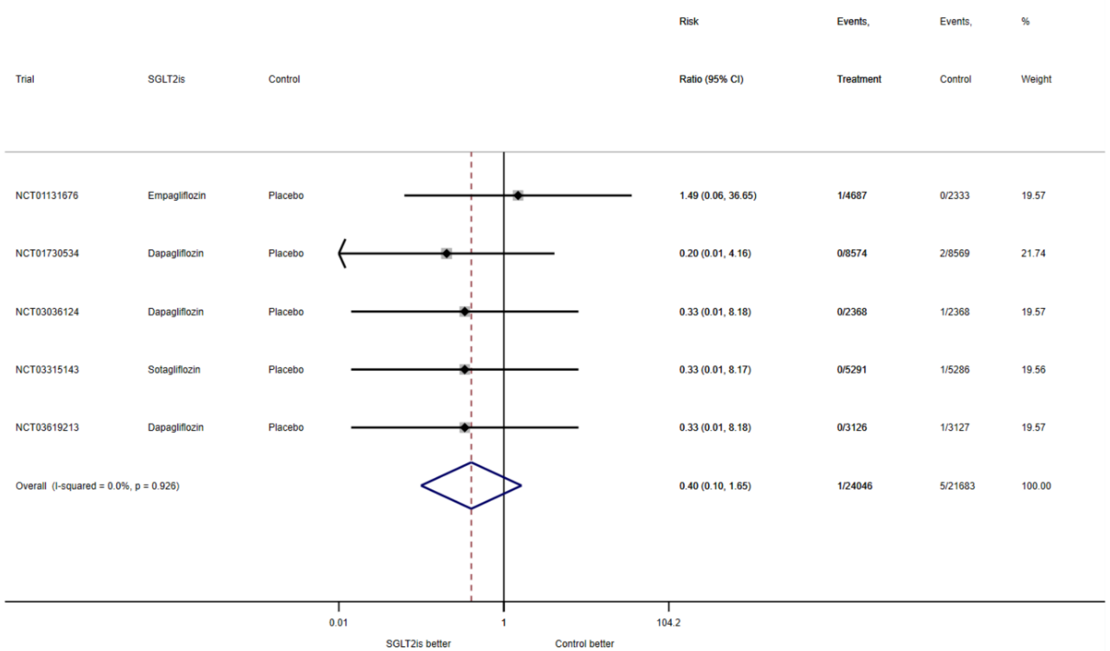

System 16-Neoplasms Benign, Malignant And Unspecified-Figure S25 Meta-analysis of SGLT2is and Cervix carcinoma

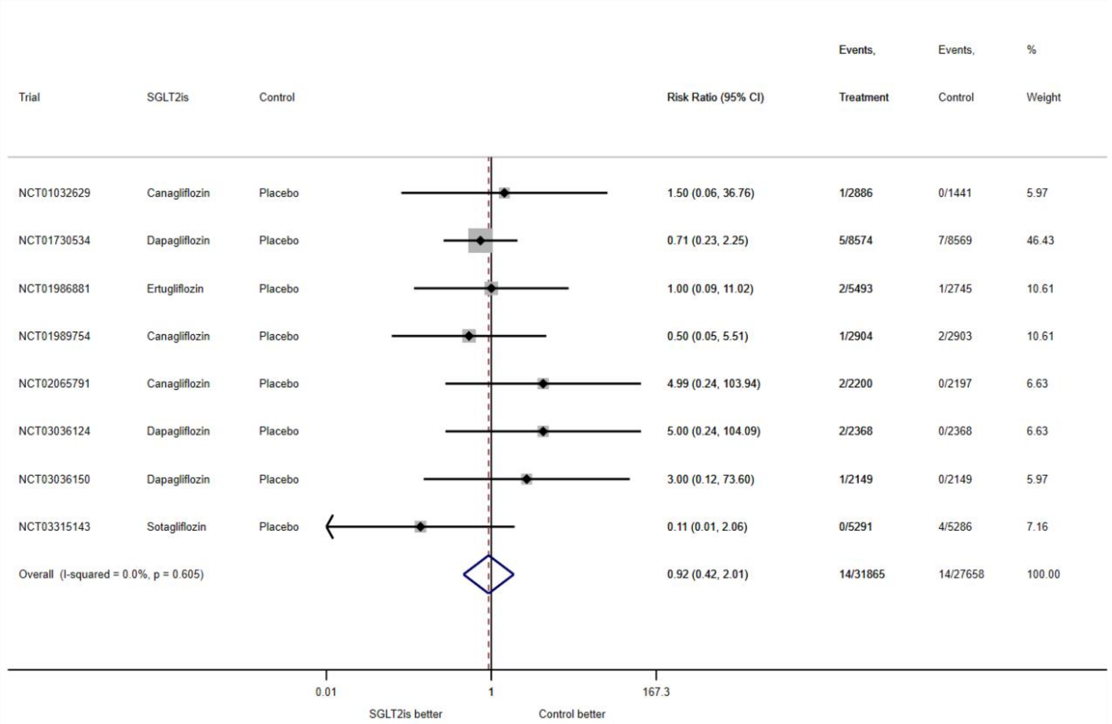

System 16-Neoplasms Benign, Malignant And Unspecified-Figure S26 Meta-analysis of SGLT2is and Cholangiocarcinoma

Trial	SGLT2is	Control	Risk Ratio (95% CI)	Events, Treatment	Events, Control	% Weight
NCT00673231	Dapagliflozin	Placebo	0.11 (0.00, 2.64)	0/610	1/197	5.37
NCT01131676	Empagliflozin	Placebo	3.49 (0.18, 67.44)	3/4687	0/2333	6.25
NCT01730534	Dapagliflozin	Placebo	1.17 (0.39, 3.47)	7/8574	6/8569	46.15
NCT01986881	Ertugliflozin	Placebo	1.50 (0.06, 36.80)	1/5493	0/2745	5.35
NCT02065791	Canagliflozin	Placebo	0.33 (0.01, 8.17)	0/2200	1/2197	5.35
NCT03036124	Dapagliflozin	Placebo	5.00 (0.24, 104.09)	2/2368	0/2368	5.95
NCT03057977	Empagliflozin	Placebo	3.00 (0.12, 73.59)	1/1863	0/1863	5.35
NCT03315143	Sotagliflozin	Placebo	0.33 (0.01, 8.17)	0/5291	1/5286	5.35
NCT03594110	Empagliflozin	Placebo	0.33 (0.01, 8.18)	0/3304	1/3305	5.35
NCT03619213	Dapagliflozin	Placebo	0.50 (0.05, 5.51)	1/3126	2/3127	9.52
Overall (I-squared = 0.0%, p = 0.751)			0.96 (0.46, 2.02)	15/37516	12/31990	100.00

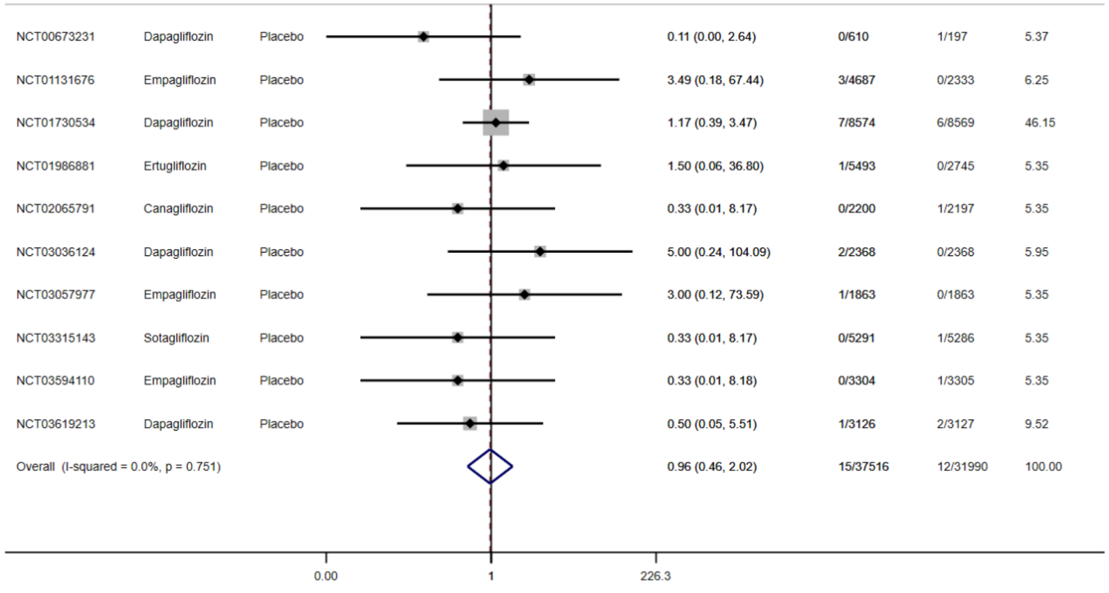

System 16-Neoplasms Benign, Malignant And Unspecified-Figure S27 Meta-analysis of SGLT2is and Chronic lymphocytic leukaemia

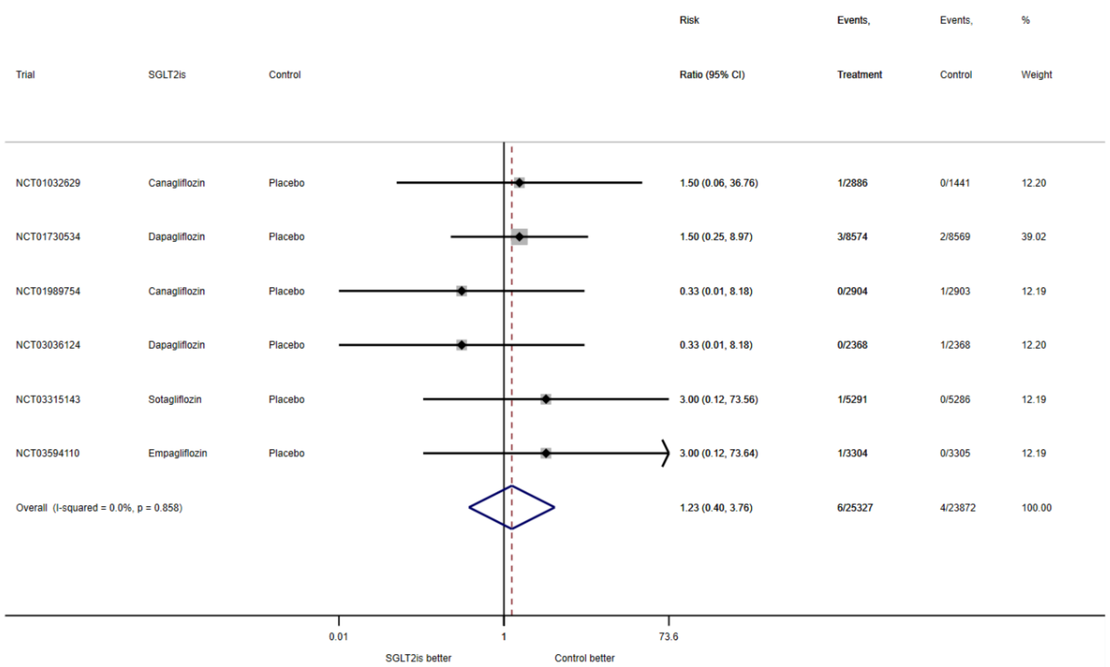

System 16-Neoplasms Benign, Malignant And Unspecified-Figure S28 Meta-analysis of SGLT2is and Chronic myeloid leukaemia

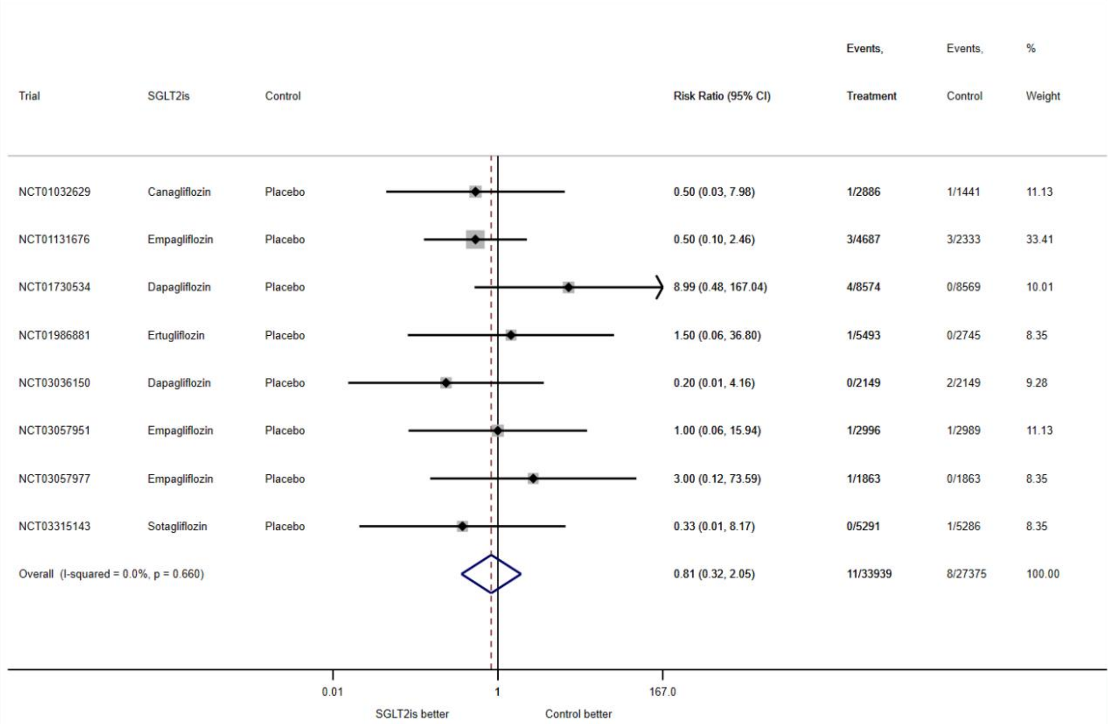

System 16-Neoplasms Benign, Malignant And Unspecified-Figure S29 Meta-analysis of SGLT2is and Clear cell renal cell carcinoma

Trial	SGLT2is	Control	Risk Ratio (95% CI)	Events, Treatment	Events, Control	% Weight
NCT01032629	Canagliflozin	Placebo	2.50 (0.12, 51.99)	2/2886	0/1441	4.51
NCT01131676	Empagliflozin	Placebo	0.17 (0.01, 4.07)	0/4687	1/2333	4.06
NCT01730534	Dapagliflozin	Placebo	1.14 (0.41, 3.15)	8/8574	7/8569	40.44
NCT01986881	Ertugliflozin	Placebo	0.50 (0.03, 7.99)	1/5493	1/2745	5.41
NCT01989754	Canagliflozin	Placebo	3.00 (0.12, 73.59)	1/2904	0/2903	4.06
NCT02065791	Canagliflozin	Placebo	3.00 (0.12, 73.50)	1/2200	0/2197	4.06
NCT03036150	Dapagliflozin	Placebo	0.20 (0.01, 4.16)	0/2149	2/2149	4.51
NCT03057951	Empagliflozin	Placebo	4.99 (0.58, 42.67)	5/2996	1/2989	9.02
NCT03057977	Empagliflozin	Placebo	1.00 (0.06, 15.98)	1/1863	1/1863	5.41
NCT03521934	Sotagliflozin	Placebo	3.03 (0.12, 74.22)	1/605	0/611	4.06
NCT03619213	Dapagliflozin	Placebo	2.00 (0.37, 10.91)	4/3126	2/3127	14.44
Overall (I-squared = 0.0%, p = 0.788)			1.34 (0.70, 2.55)	24/37483	15/30927	100.00

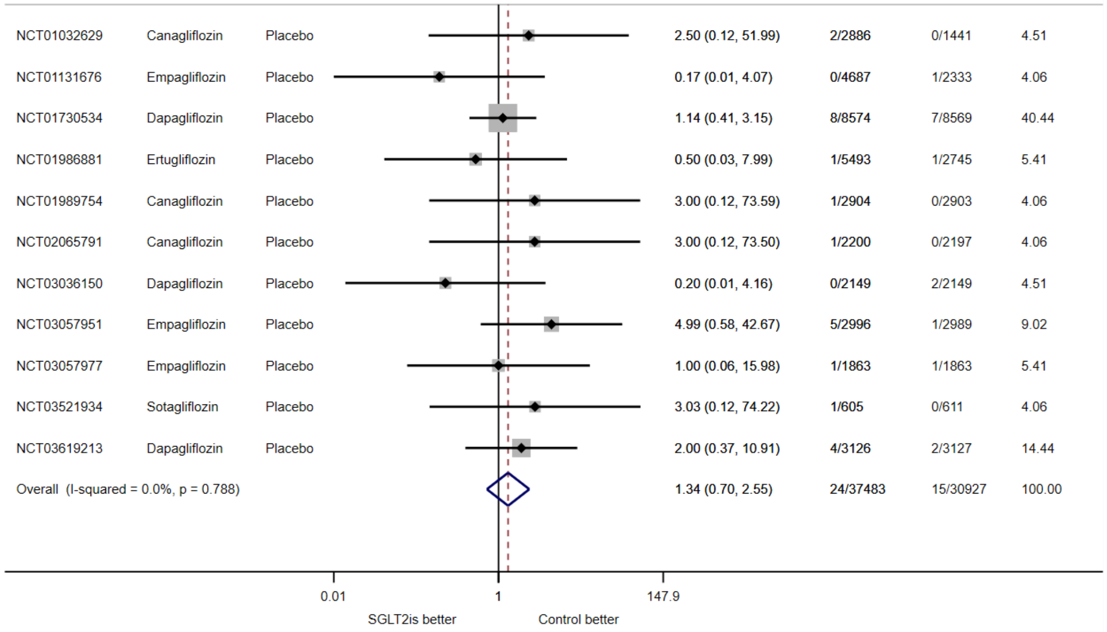

System 16-Neoplasms Benign, Malignant And Unspecified-Figure S30 Meta-analysis of SGLT2is and Colon adenoma

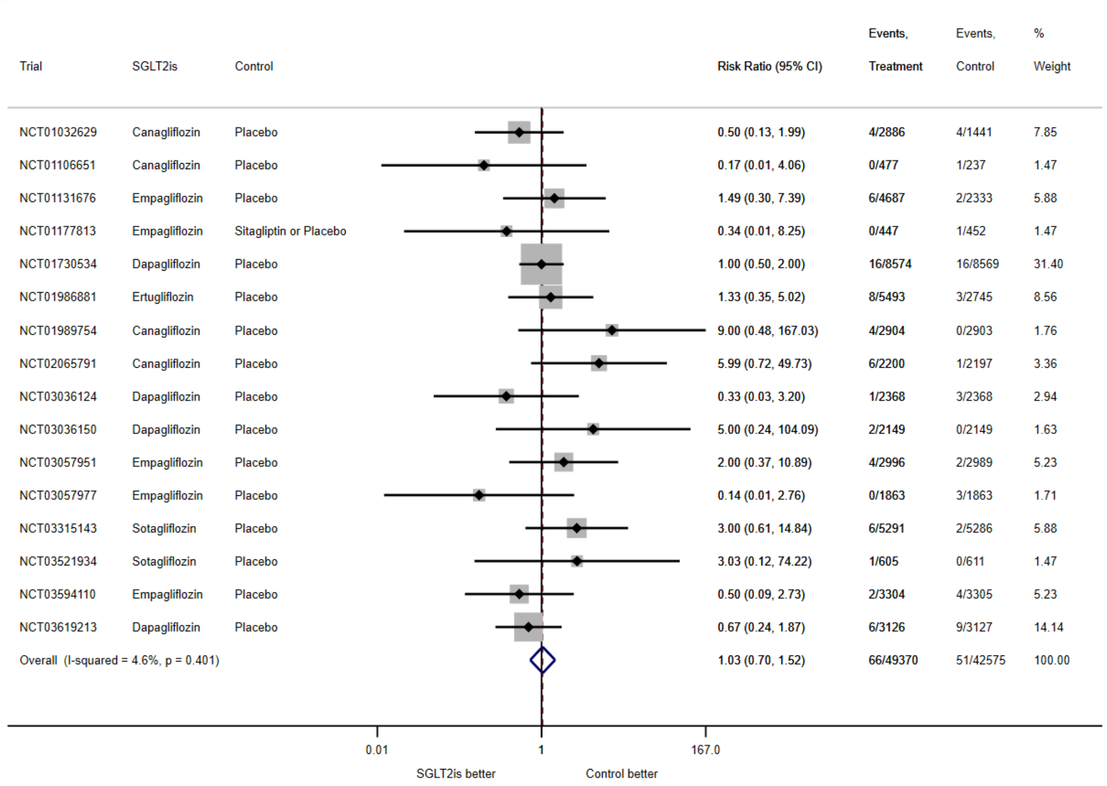

System 16-Neoplasms Benign, Malignant And Unspecified-Figure S31 Meta-analysis of SGLT2is and Colon cancer

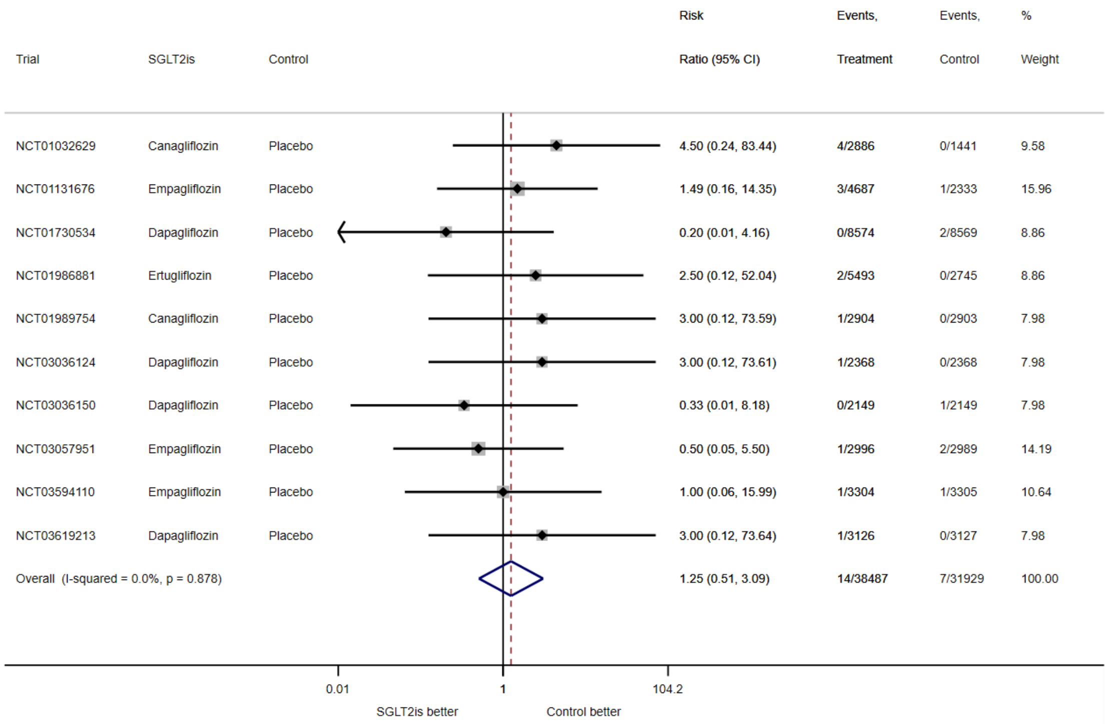

System 16-Neoplasms Benign, Malignant And Unspecified-Figure S32 Meta-analysis of SGLT2is and Colon cancer metastatic

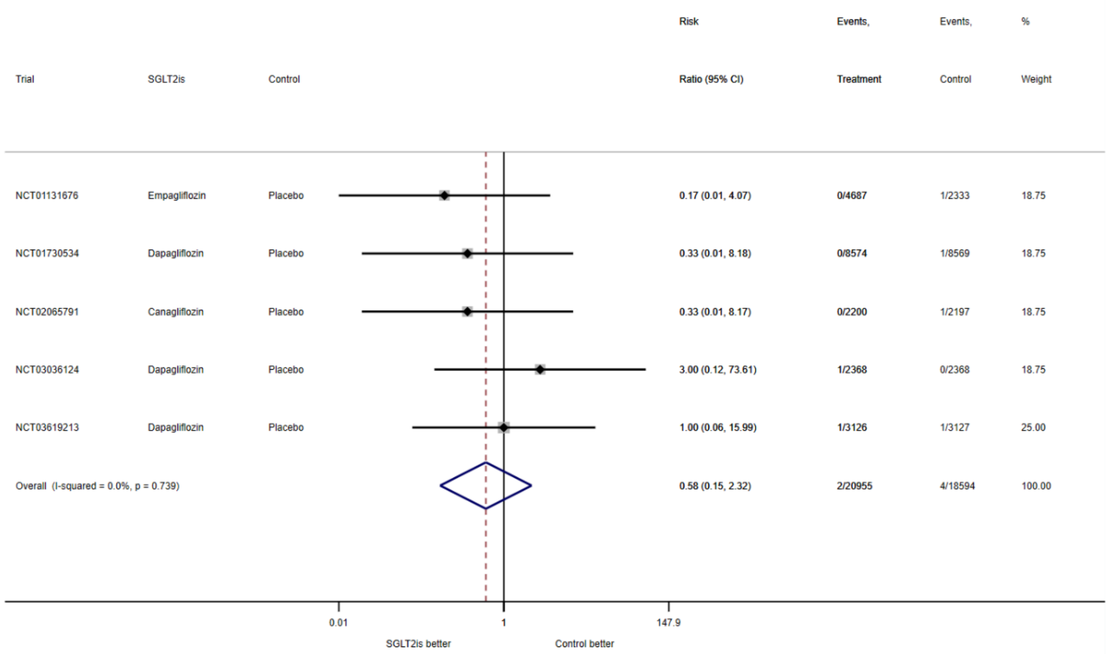

System 16-Neoplasms Benign, Malignant And Unspecified-Figure S33 Meta-analysis of SGLT2is and Colon neoplasm

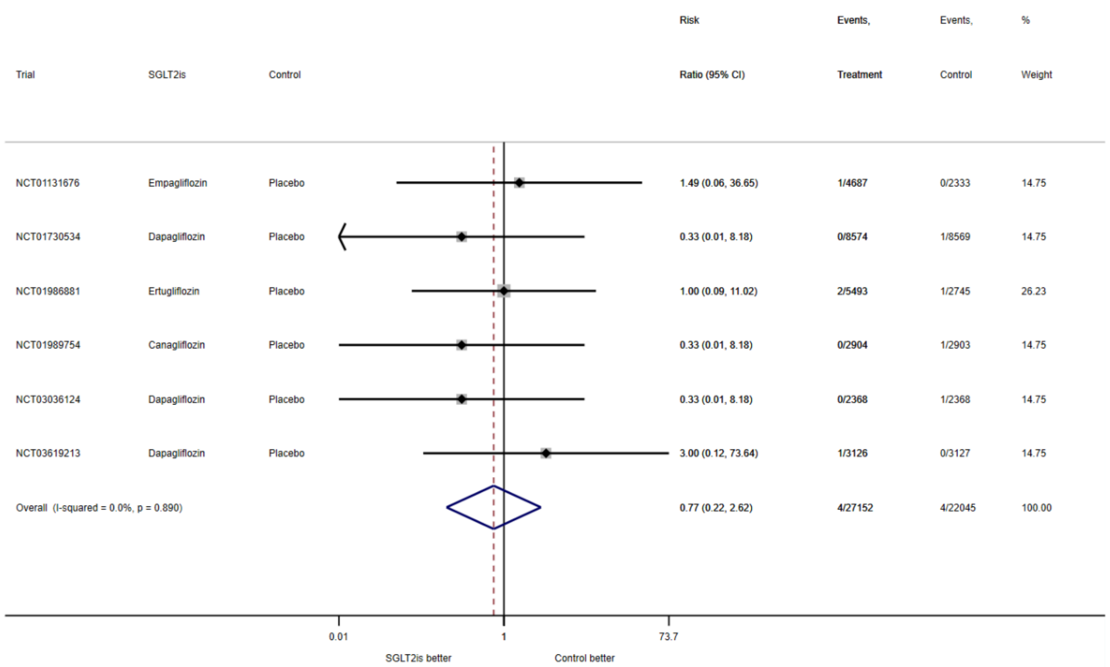

System 16-Neoplasms Benign, Malignant And Unspecified-Figure S34 Meta-analysis of SGLT2is and Colorectal adenocarcinoma

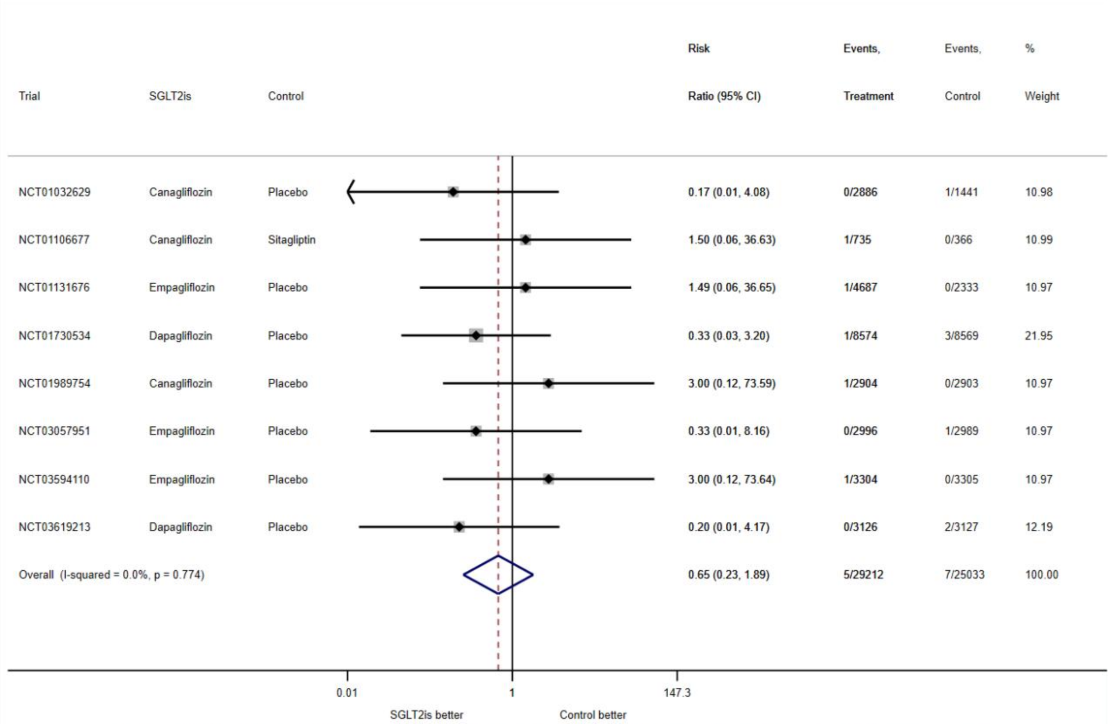

System 16-Neoplasms Benign, Malignant And Unspecified-Figure S35 Meta-analysis of SGLT2is and Colorectal cancer

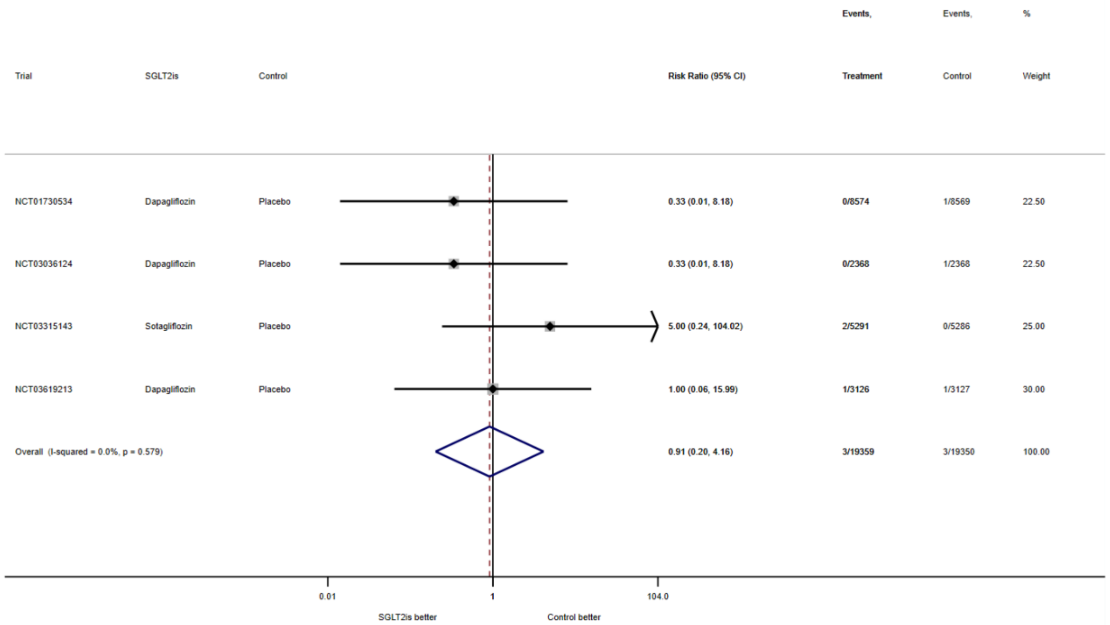

System 16-Neoplasms Benign, Malignant And Unspecified-Figure S36 Meta-analysis of SGLT2is and Colorectal cancer metastatic

Trial	SGLT2is	Control	Risk Ratio (95% CI)	Events, Treatment	Events, Control	% Weight
NCT01032629	Canagliflozin	Placebo	2.50 (0.12, 51.99)	2/2886	0/1441	10.99
NCT01730534	Dapagliflozin	Placebo	1.00 (0.14, 7.09)	2/8574	2/8569	26.37
NCT01989754	Canagliflozin	Placebo	0.33 (0.01, 8.18)	0/2904	1/2903	9.89
NCT02065791	Canagliflozin	Placebo	0.33 (0.01, 8.17)	0/2200	1/2197	9.89
NCT03036150	Dapagliflozin	Placebo	3.00 (0.12, 73.60)	1/2149	0/2149	9.89
NCT03057951	Empagliflozin	Placebo	2.99 (0.12, 73.44)	1/2996	0/2989	9.89
NCT03057977	Empagliflozin	Placebo	3.00 (0.12, 73.59)	1/1863	0/1863	9.89
NCT03619213	Dapagliflozin	Placebo	1.00 (0.06, 15.99)	1/3126	1/3127	13.19
Overall (I-squared = 0.0%, p = 0.931)			1.23 (0.45, 3.37)	8/26698	5/25238	100.00

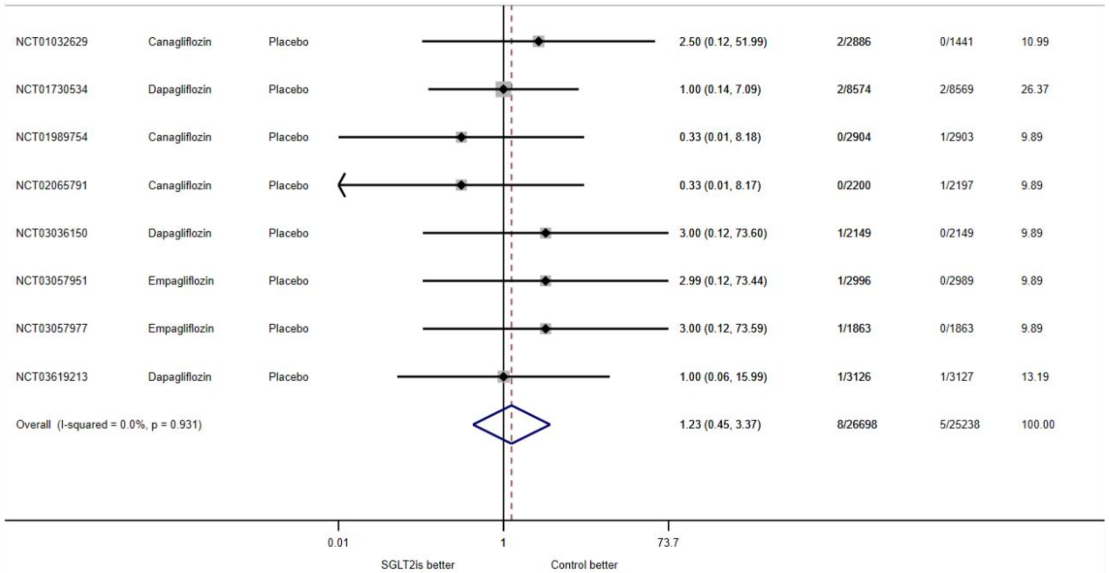

System 16-Neoplasms Benign, Malignant And Unspecified-Figure S37 Meta-analysis of SGLT2is and Diffuse large b-cell lymphoma

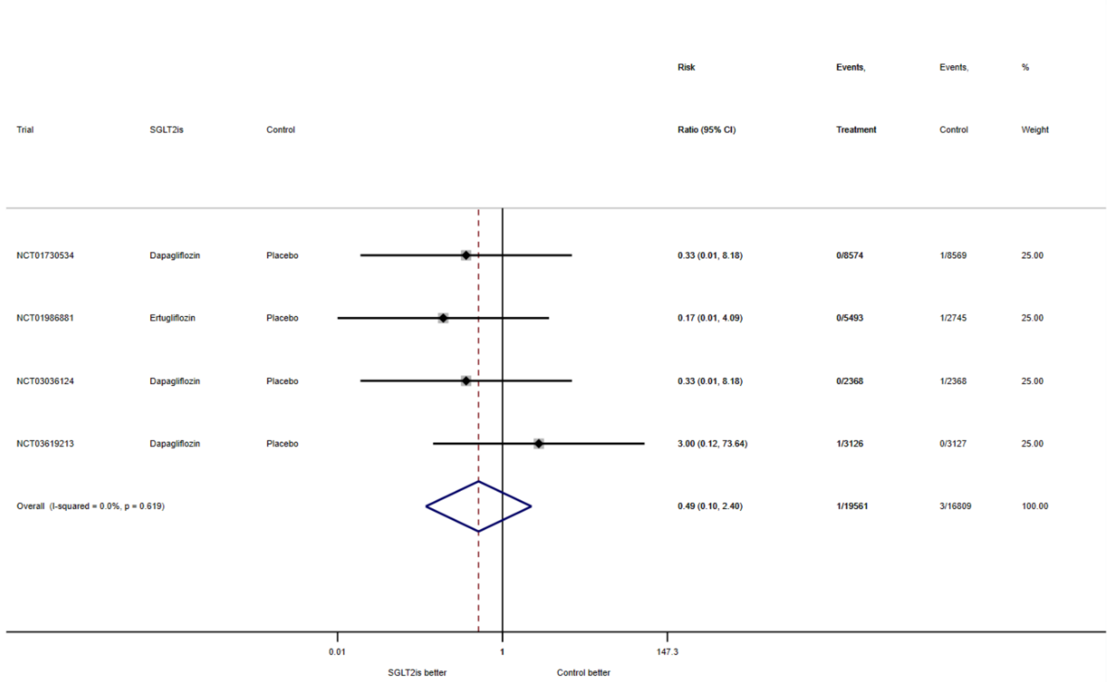

System 16-Neoplasms Benign, Malignant And Unspecified-Figure S38 Meta-analysis of SGLT2is and Ductal adenocarcinoma of pancreas

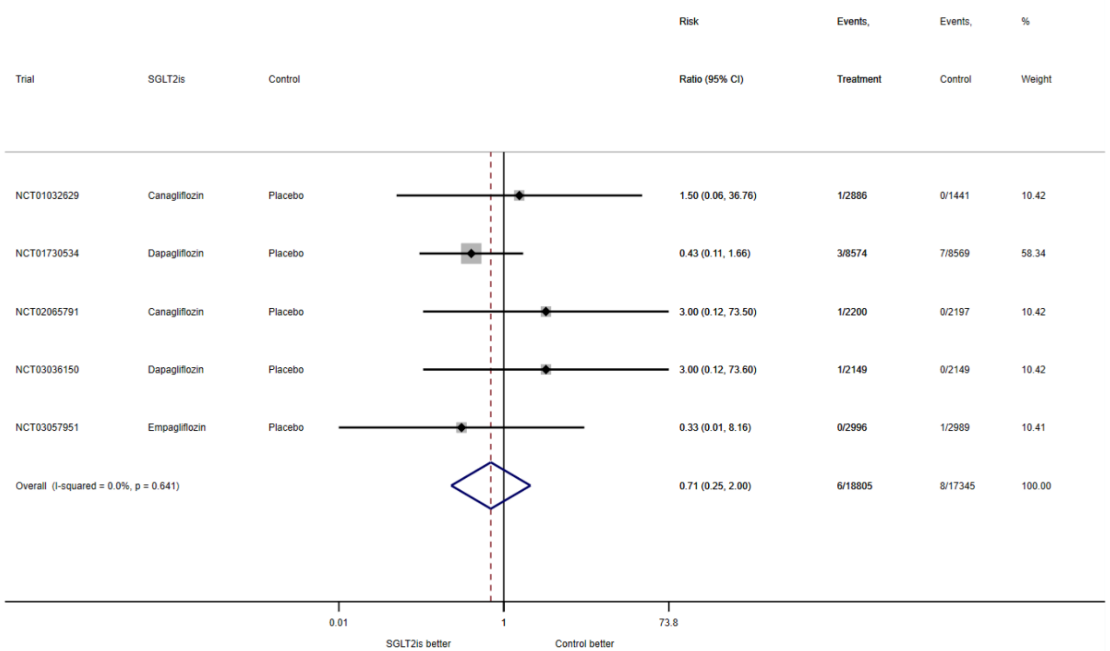

System 16-Neoplasms Benign, Malignant And Unspecified-Figure S39 Meta-analysis of SGLT2is and Endometrial adenocarcinoma

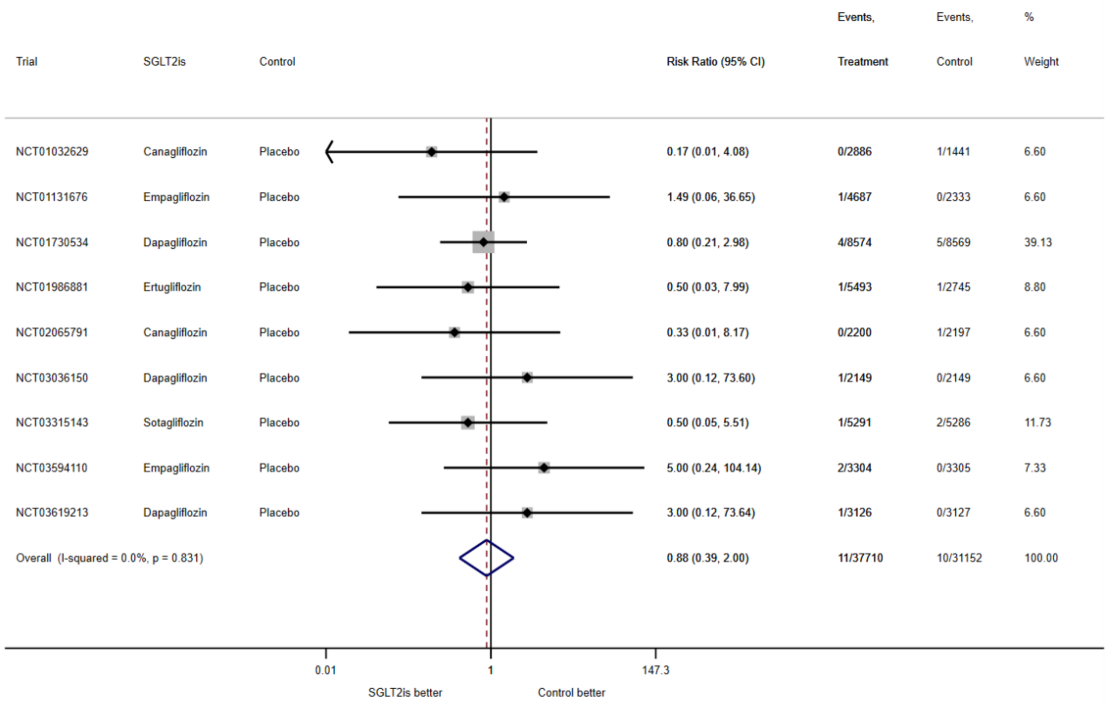

System 16-Neoplasms Benign, Malignant And Unspecified-Figure S40 Meta-analysis of SGLT2is and Endometrial cancer

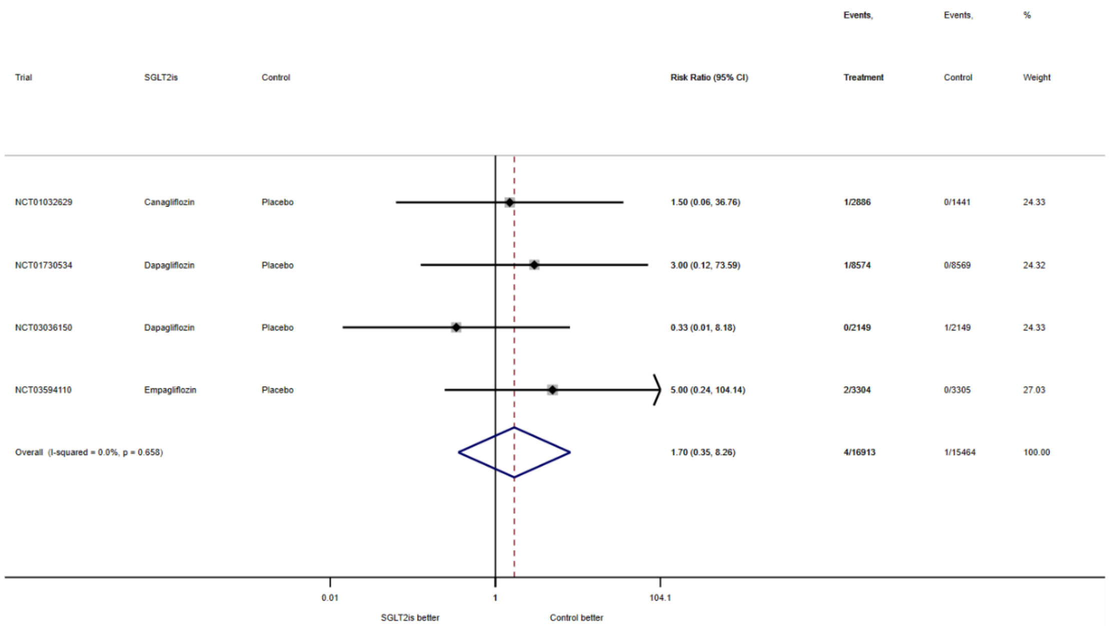

System 16-Neoplasms Benign, Malignant And Unspecified-Figure S41 Meta-analysis of SGLT2is and Gallbladder cancer metastatic

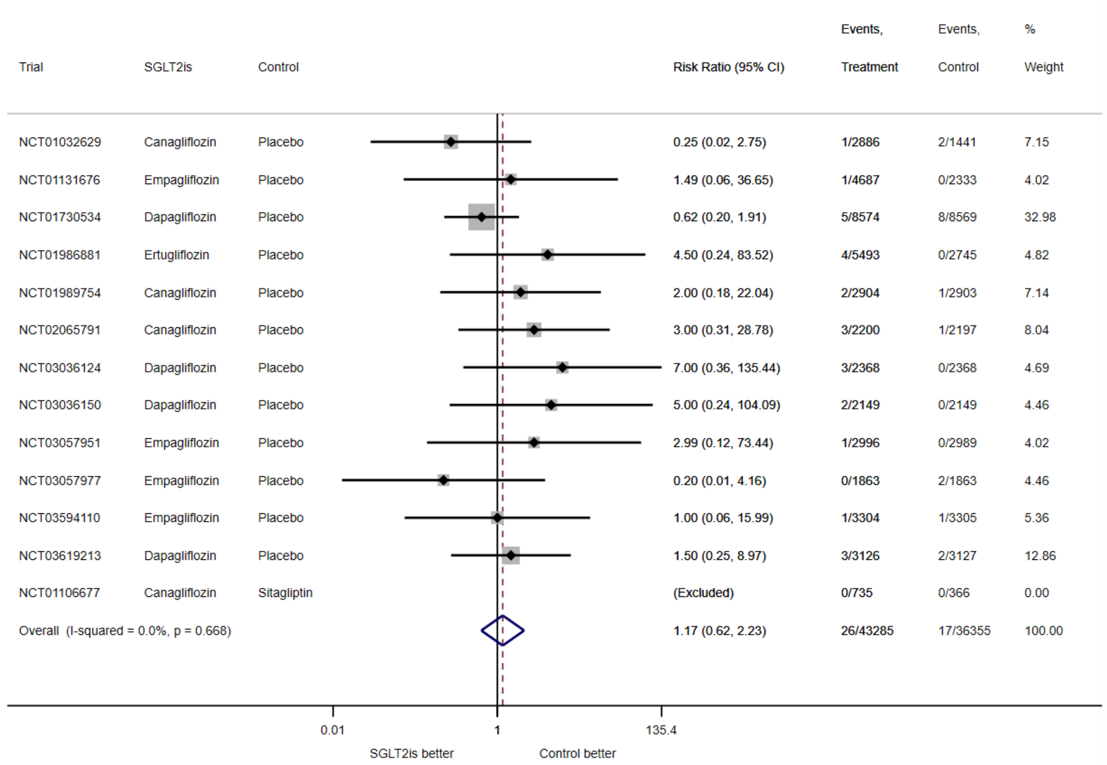

System 16-Neoplasms Benign, Malignant And Unspecified-Figure S42 Meta-analysis of SGLT2is and Gastric cancer

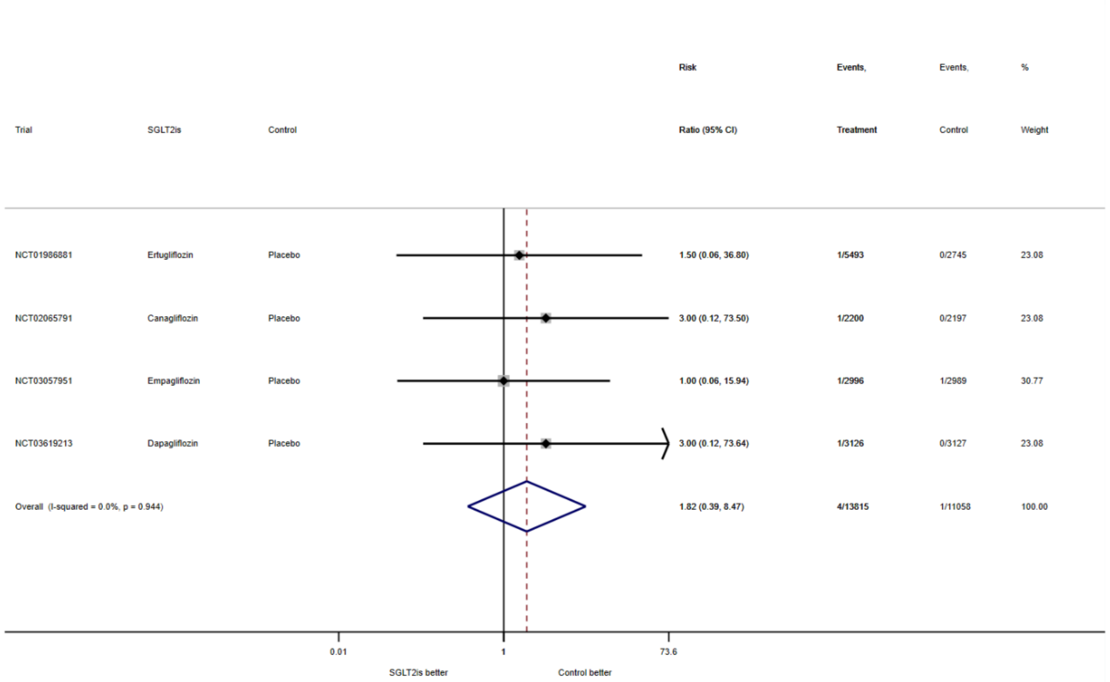

System 16-Neoplasms Benign, Malignant And Unspecified-Figure S43 Meta-analysis of SGLT2is and Gastric neoplasm

Trial	SGLT2is	Control	Risk Ratio (95% CI)	Events, Treatment	Events, Control	% Weight
NCT01730534	Dapagliflozin	Placebo	0.20 (0.01, 4.16)	0/8574	2/8569	18.18
NCT01986881	Ertugliflozin	Placebo	1.50 (0.06, 36.80)	1/5493	0/2745	16.36
NCT01989754	Canagliflozin	Placebo	3.00 (0.12, 73.59)	1/2904	0/2903	16.36
NCT03036124	Dapagliflozin	Placebo	3.00 (0.12, 73.61)	1/2368	0/2368	16.37
NCT03315143	Sotagliflozin	Placebo	3.00 (0.12, 73.56)	1/5291	0/5286	16.36
NCT03619213	Dapagliflozin	Placebo	3.00 (0.12, 73.64)	1/3126	0/3127	16.36
Overall (I-squared = 0.0%, p = 0.792)			1.64 (0.45, 5.97)	5/27756	2/24998	100.00

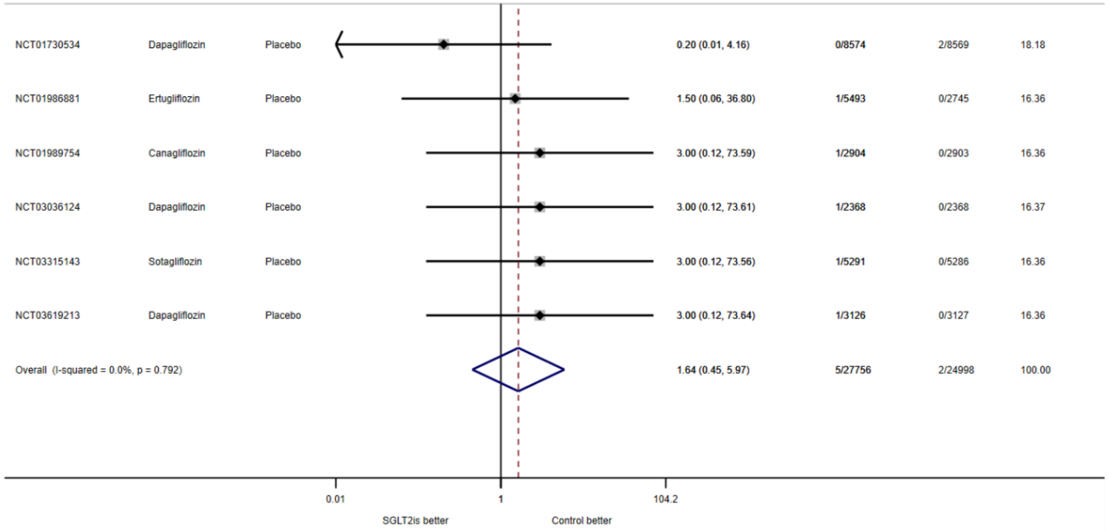

System 16-Neoplasms Benign, Malignant And Unspecified-Figure S44 Meta-analysis of SGLT2is and Glioblastoma

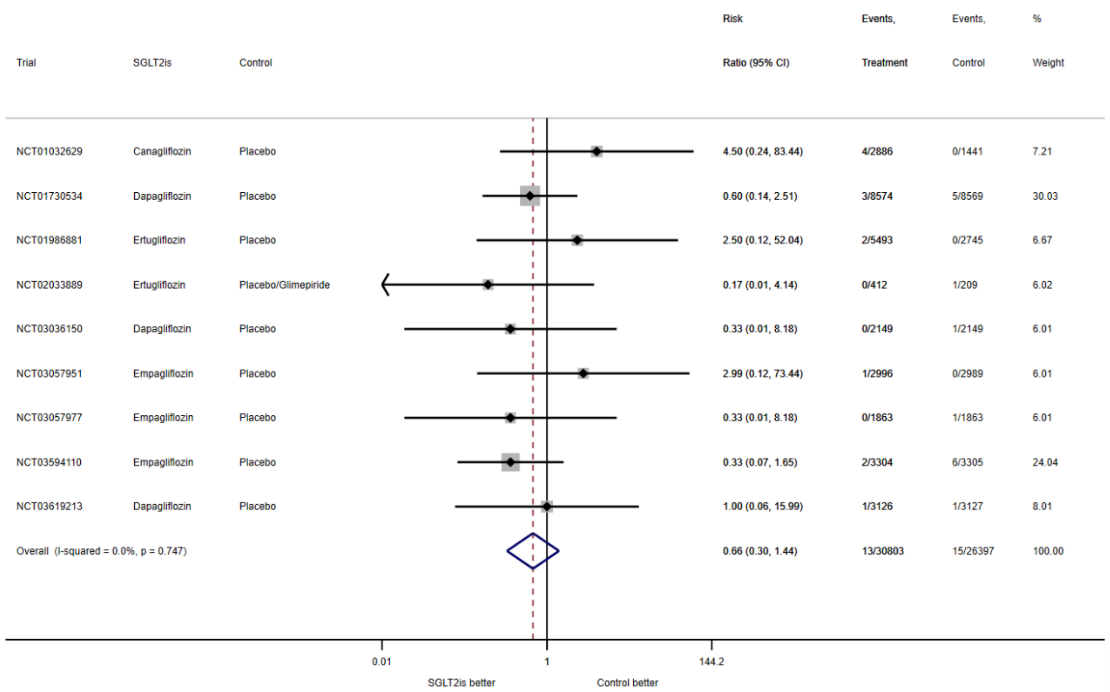

System 16-Neoplasms Benign, Malignant And Unspecified-Figure S45 Meta-analysis of SGLT2is and Hepatic cancer

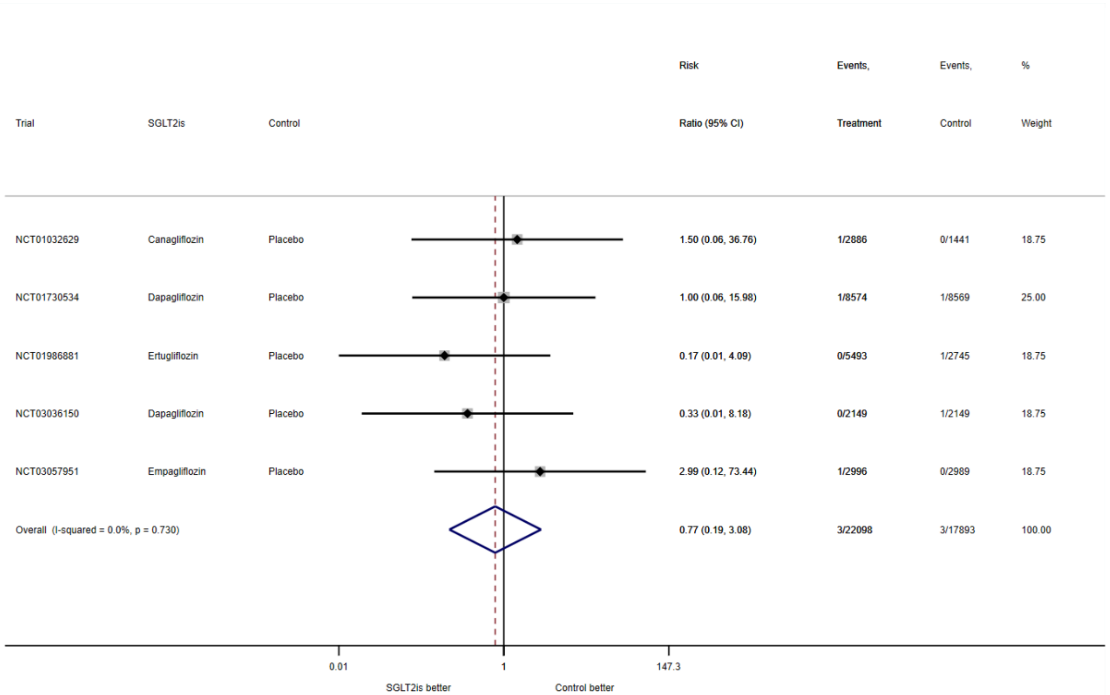

System 16-Neoplasms Benign, Malignant And Unspecified-Figure S46 Meta-analysis of SGLT2is and Hepatic cancer metastatic

Trial	SGLT2is	Control	Risk Ratio (95% CI)	Events, Treatment	Events, Control	% Weight
NCT01032629	Canagliflozin	Placebo	0.17 (0.02, 1.60)	1/2886	3/1441	9.70
NCT01131676	Empagliflozin	Placebo	1.99 (0.22, 17.80)	4/4687	1/2333	10.34
NCT01730534	Dapagliflozin	Placebo	2.00 (0.60, 6.64)	8/8574	4/8569	34.47
NCT01986881	Ertugliflozin	Placebo	0.50 (0.03, 7.99)	1/5493	1/2745	6.46
NCT02065791	Canagliflozin	Placebo	0.14 (0.01, 2.76)	0/2200	3/2197	5.65
NCT03036124	Dapagliflozin	Placebo	3.00 (0.12, 73.61)	1/2368	0/2368	4.85
NCT03036150	Dapagliflozin	Placebo	0.50 (0.05, 5.51)	1/2149	2/2149	8.62
NCT03057951	Empagliflozin	Placebo	1.00 (0.06, 15.94)	1/2996	1/2989	6.46
NCT03315143	Sotagliflozin	Placebo	3.00 (0.12, 73.56)	1/5291	0/5286	4.84
NCT03619213	Dapagliflozin	Placebo	2.00 (0.18, 22.05)	2/3126	1/3127	8.62
Overall (I-squared = 0.0%, p = 0.589)			1.09 (0.54, 2.21)	20/39770	16/33204	100.00

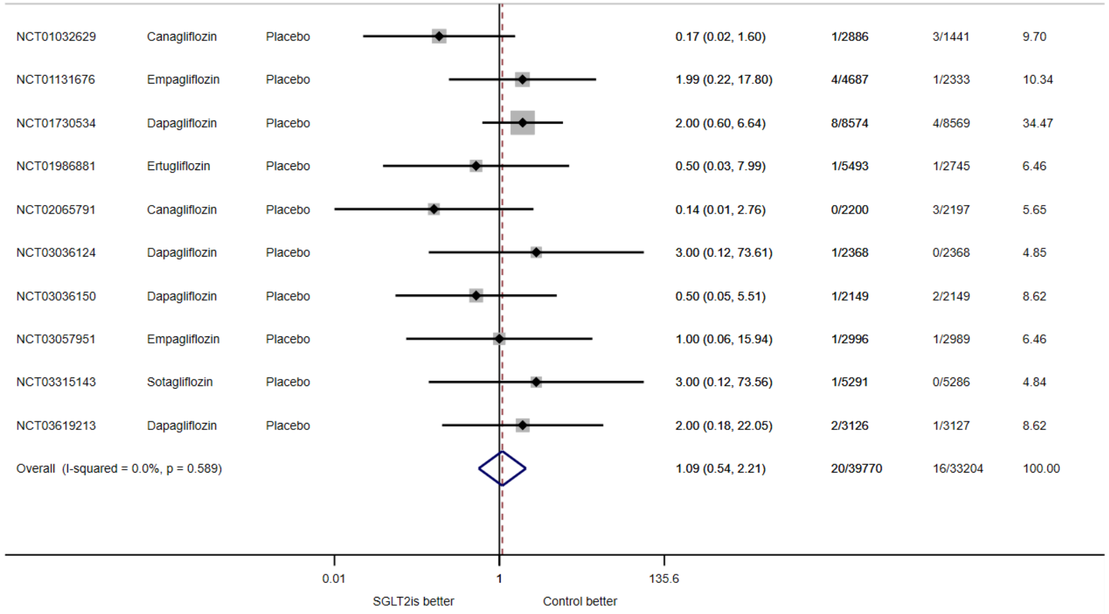

System 16-Neoplasms Benign, Malignant And Unspecified-Figure S47 Meta-analysis of SGLT2is and Hepatocellular carcinoma

Trial	SGLT2is	Control	Risk Ratio (95% CI)	Events, Treatment	Events, Control	% Weight
NCT01032629	Canagliflozin	Placebo	0.50 (0.03, 7.98)	1/2886	1/1441	23.53
NCT01106651	Canagliflozin	Placebo	1.49 (0.06, 36.53)	1/477	0/237	17.68
NCT01730534	Dapagliflozin	Placebo	1.00 (0.06, 15.98)	1/8574	1/8569	23.52
NCT01989754	Canagliflozin	Placebo	0.33 (0.01, 8.18)	0/2904	1/2903	17.64
NCT03315143	Sotagliflozin	Placebo	0.33 (0.01, 8.17)	0/5291	1/5286	17.64
Overall (I-squared = 0.0%, p = 0.949)			0.62 (0.16, 2.37)	3/20132	4/18436	100.00

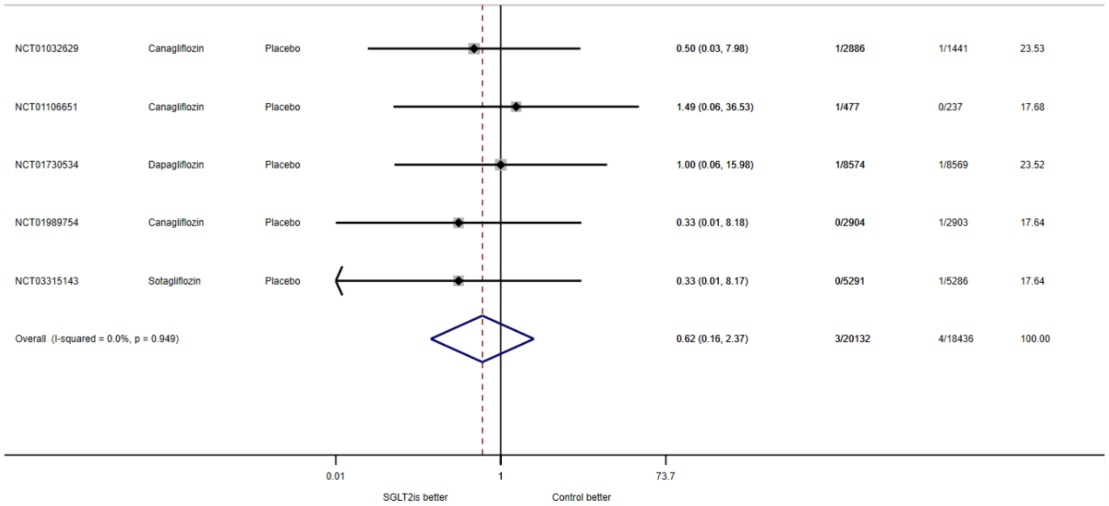

System 16-Neoplasms Benign, Malignant And Unspecified-Figure S48 Meta-analysis of SGLT2is and Hodgkin's disease

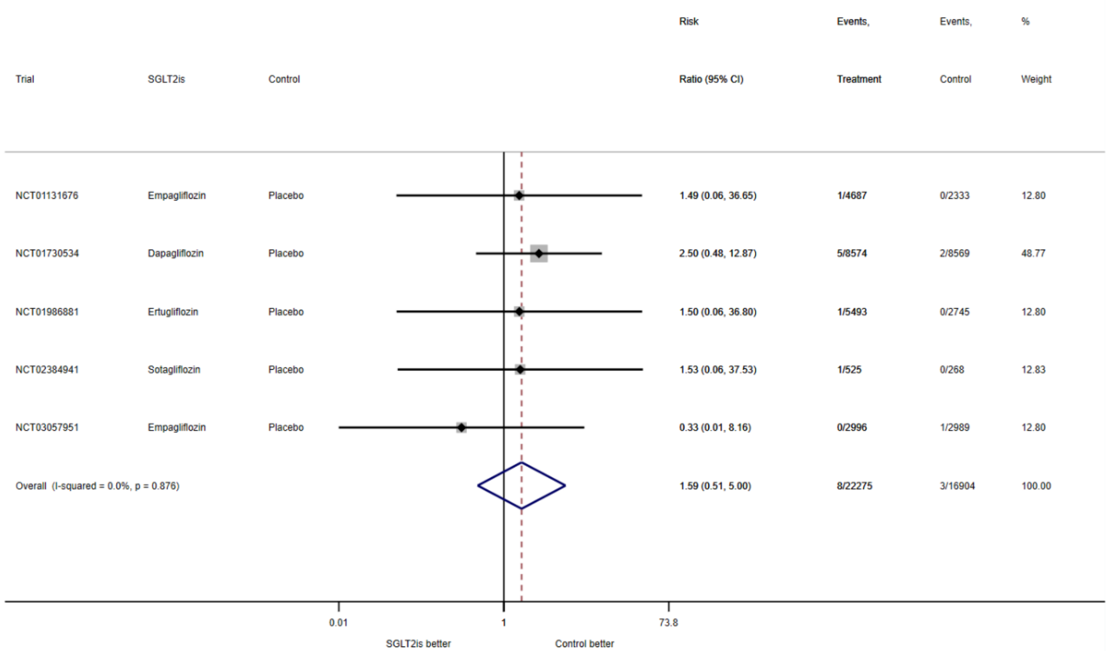

System 16-Neoplasms Benign, Malignant And Unspecified-Figure S49 Meta-analysis of SGLT2is and Intraductal proliferative breast lesion

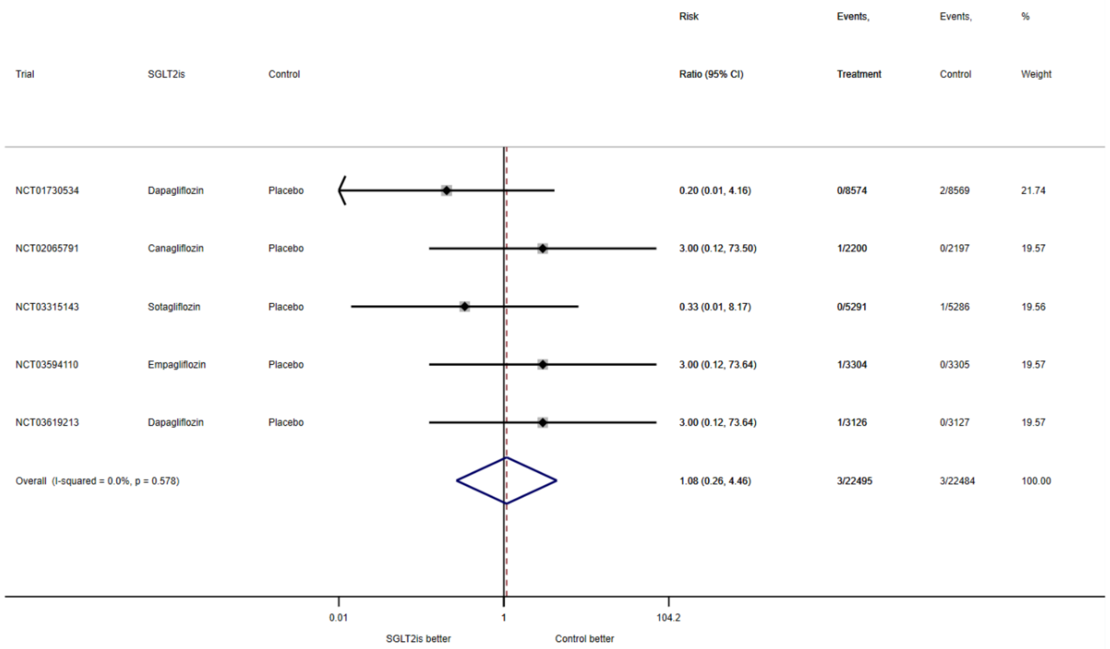

System 16-Neoplasms Benign, Malignant And Unspecified-Figure S50 Meta-analysis of SGLT2is and Invasive breast carcinoma

Trial	SGLT2is	Control	Risk Ratio (95% CI)	Events, Treatment	Events, Control	% Weight
NCT01032629	Canagliflozin	Placebo	0.25 (0.02, 2.75)	1/2886	2/1441	8.33
NCT01131676	Empagliflozin	Placebo	1.49 (0.06, 36.65)	1/4687	0/2333	4.68
NCT01730534	Dapagliflozin	Placebo	1.80 (0.60, 5.37)	9/8574	5/8569	40.15
NCT01986881	Ertugliflozin	Placebo	1.50 (0.06, 36.80)	1/5493	0/2745	4.68
NCT01989754	Canagliflozin	Placebo	0.20 (0.01, 4.16)	0/2904	2/2903	5.20
NCT02065791	Canagliflozin	Placebo	2.00 (0.18, 22.01)	2/2200	1/2197	8.33
NCT02384941	Sotagliflozin	Placebo	1.53 (0.06, 37.53)	1/525	0/268	4.69
NCT03057951	Empagliflozin	Placebo	0.33 (0.01, 8.16)	0/2996	1/2989	4.68
NCT03057977	Empagliflozin	Placebo	1.00 (0.06, 15.98)	1/1863	1/1863	6.24
NCT03315143	Sotagliflozin	Placebo	3.00 (0.12, 73.56)	1/5291	0/5286	4.68
NCT03619213	Dapagliflozin	Placebo	0.50 (0.05, 5.51)	1/3126	2/3127	8.33
Overall (I-squared = 0.0%, p = 0.882)			1.10 (0.55, 2.20)	18/40545	14/33721	100.00

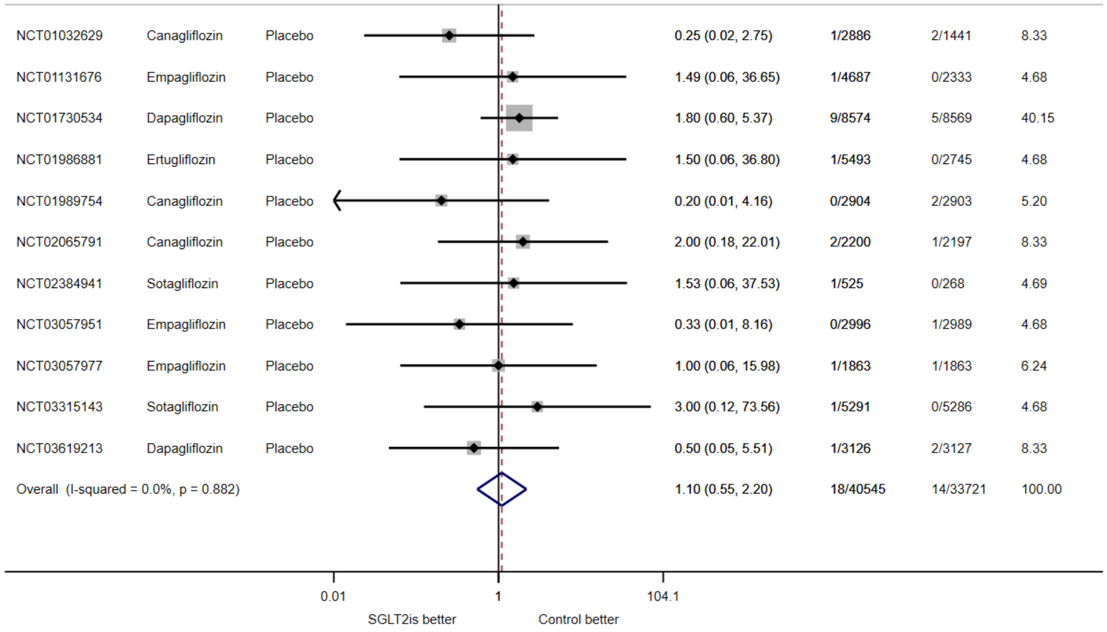

System 16-Neoplasms Benign, Malignant And Unspecified-Figure S51 Meta-analysis of SGLT2is and Invasive ductal breast carcinoma

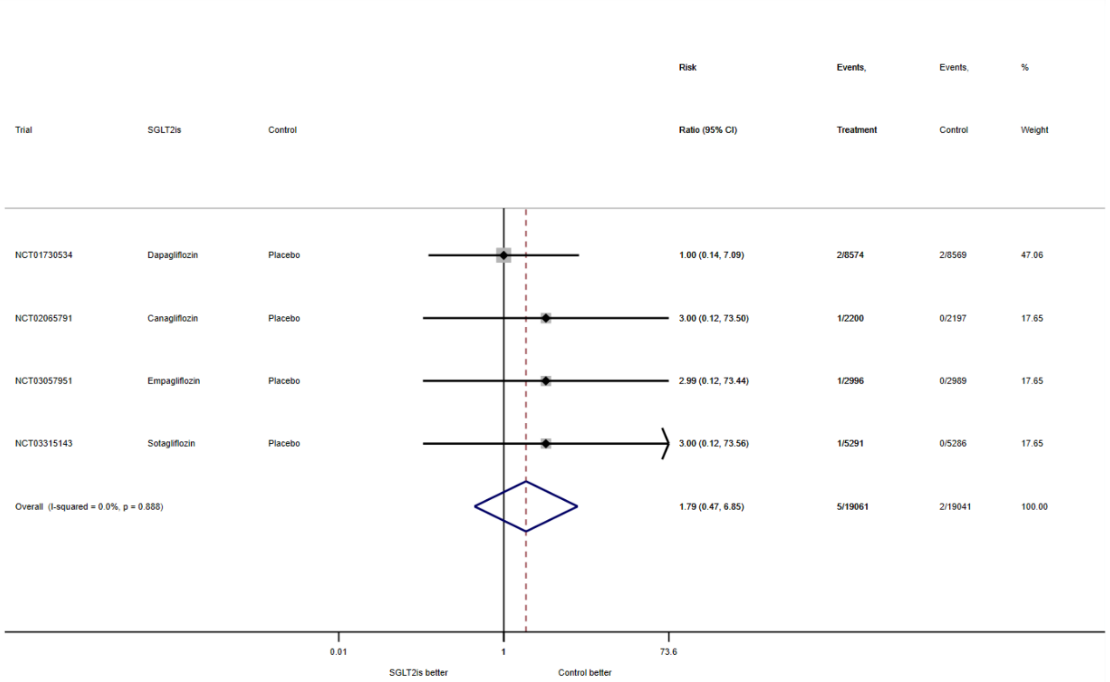

System 16-Neoplasms Benign, Malignant And Unspecified-Figure S52 Meta-analysis of SGLT2is and Invasive lobular breast carcinoma

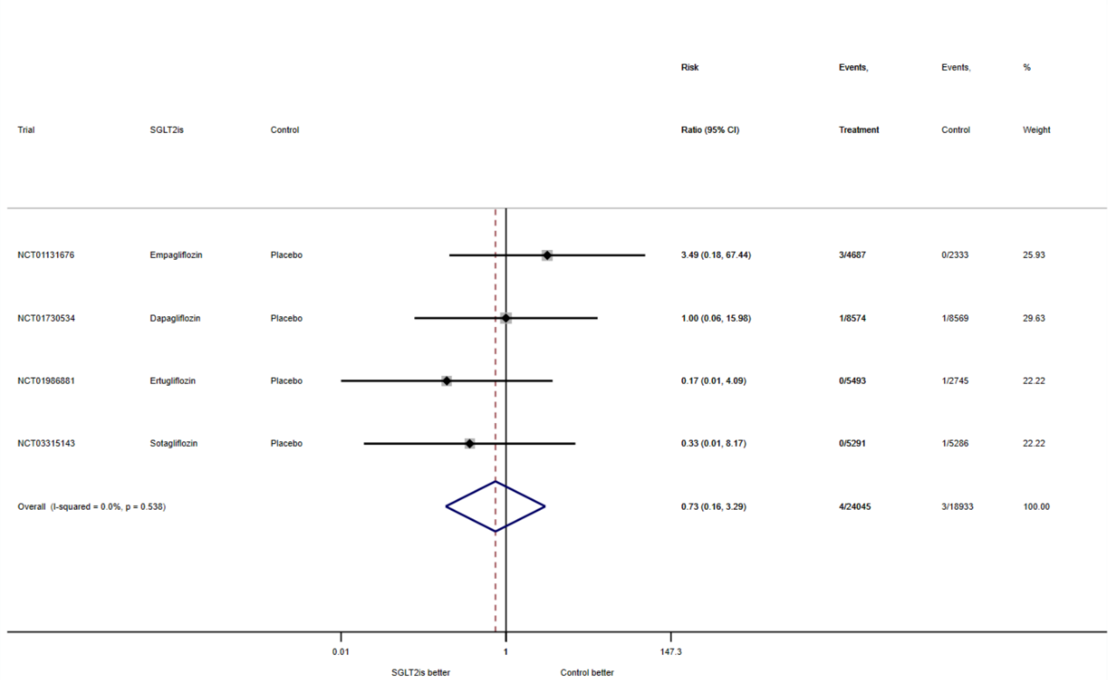

System 16-Neoplasms Benign, Malignant And Unspecified-Figure S53 Meta-analysis of SGLT2is and Keratoacanthoma

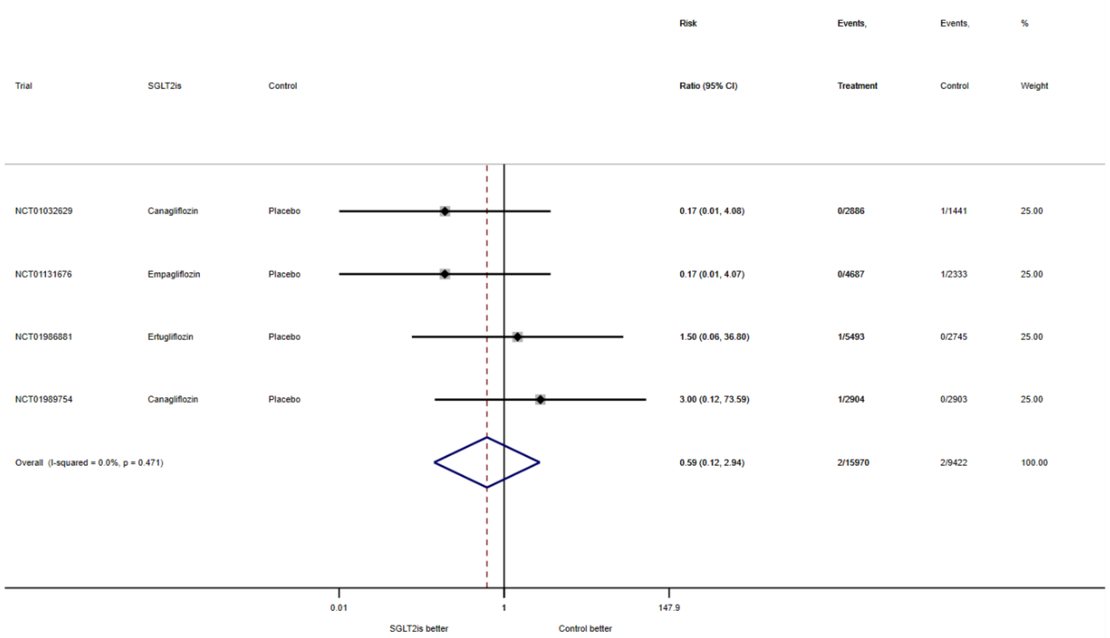

System 16-Neoplasms Benign, Malignant And Unspecified-Figure S54 Meta-analysis of SGLT2is and Large cell lung cancer

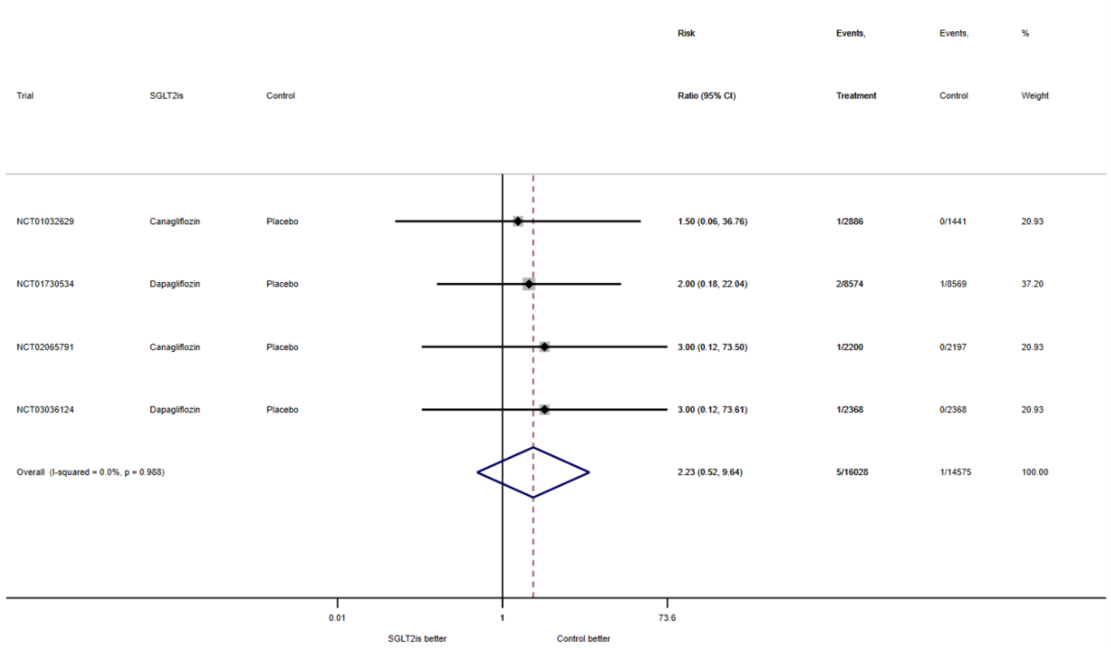

System 16-Neoplasms Benign, Malignant And Unspecified-Figure S55 Meta-analysis of SGLT2is and Large intestine benign neoplasm

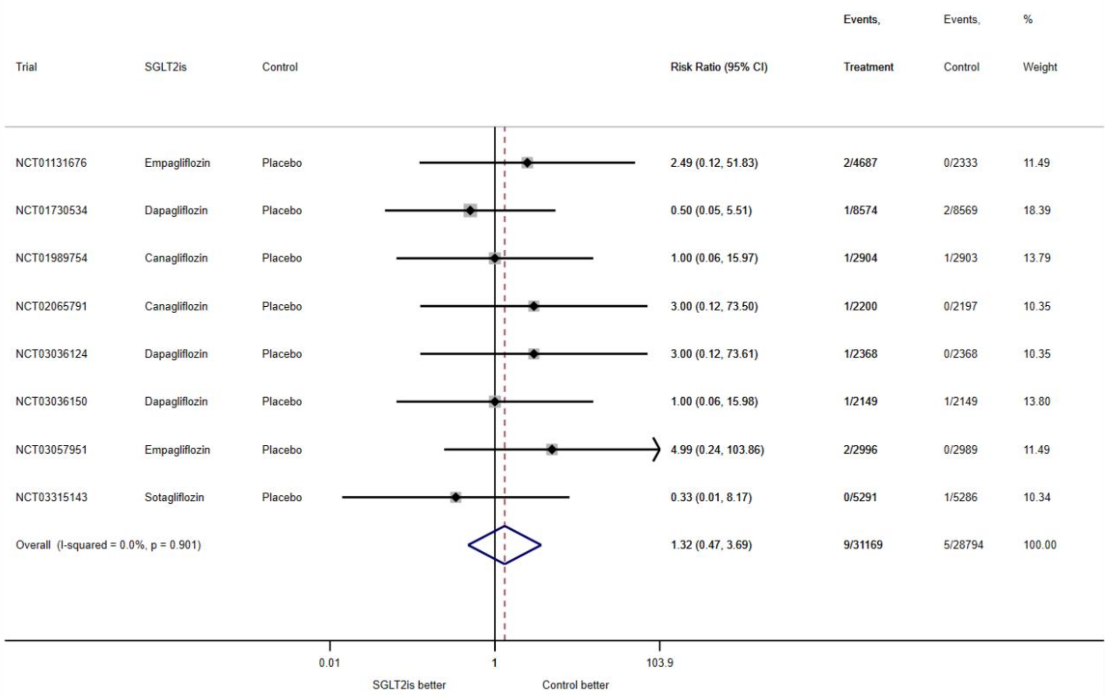

System 16-Neoplasms Benign, Malignant And Unspecified-Figure S56 Meta-analysis of SGLT2is and Laryngeal cancer

Trial	SGLT2is	Control	Risk Ratio (95% CI)	Events, Treatment	Events, Control	% Weight
NCT01032629	Canagliflozin	Placebo	0.17 (0.01, 4.08)	0/2886	1/1441	15.93
NCT01131676	Empagliflozin	Placebo	2.49 (0.12, 51.83)	2/4687	0/2333	17.70
NCT01730534	Dapagliflozin	Placebo	0.14 (0.01, 2.76)	0/8574	3/8569	18.58
NCT03057951	Empagliflozin	Placebo	0.33 (0.01, 8.16)	0/2996	1/2989	15.93
NCT03315143	Sotagliflozin	Placebo	3.00 (0.12, 73.56)	1/5291	0/5286	15.93
NCT03619213	Dapagliflozin	Placebo	3.00 (0.12, 73.64)	1/3126	0/3127	15.93
Overall (I-squared = 0.0%, p = 0.501)			0.73 (0.20, 2.63)	4/27560	5/23745	100.00

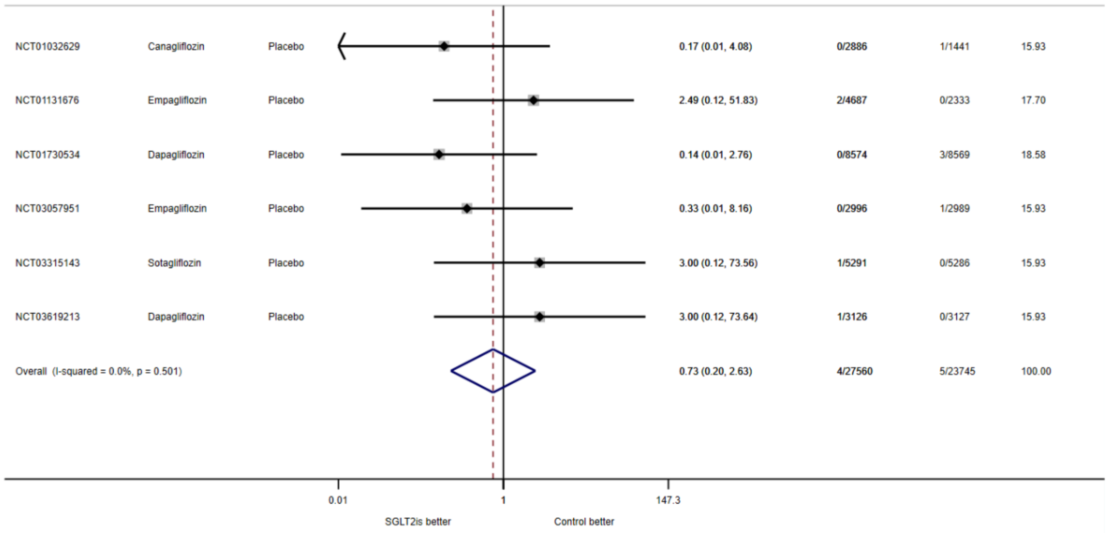

System 16-Neoplasms Benign, Malignant And Unspecified-Figure S57 Meta-analysis of SGLT2is and Laryngeal squamous cell carcinoma

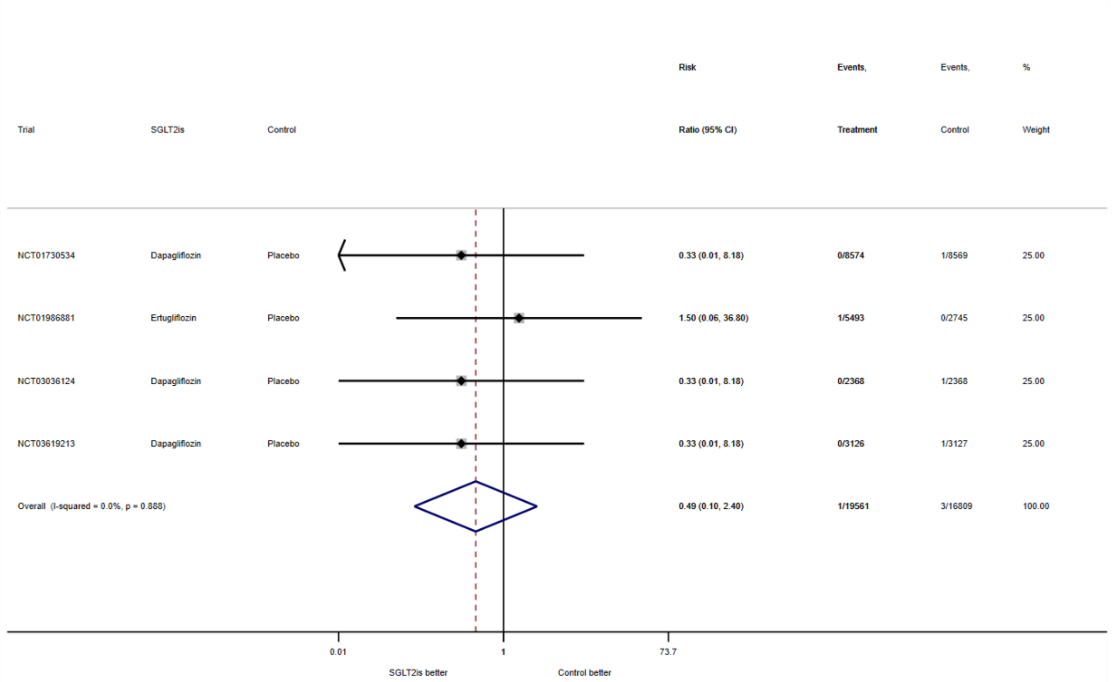

System 16-Neoplasms Benign, Malignant And Unspecified-Figure S58 Meta-analysis of SGLT2is and Leukaemia

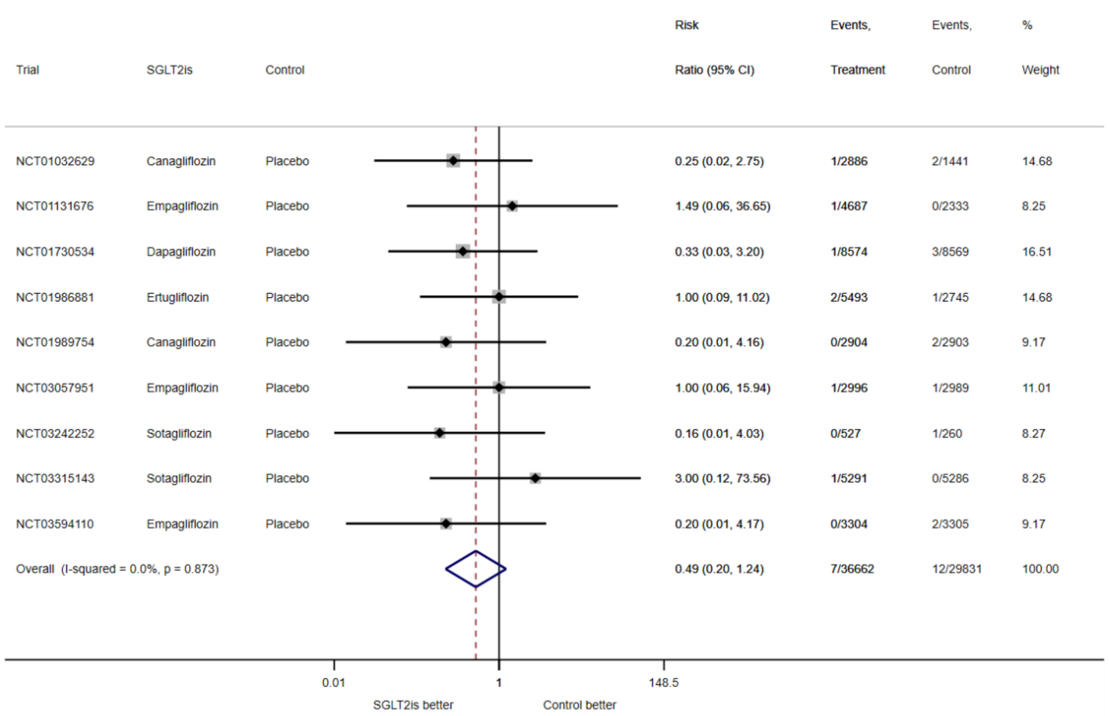

System 16-Neoplasms Benign, Malignant And Unspecified-Figure S59 Meta-analysis of SGLT2is and Lipoma

Trial	SGLT2is	Control	Risk Ratio (95% CI)	Events, Treatment	Events, Control	% Weight
NCT01032629	Canagliflozin	Placebo	0.50 (0.03, 7.98)	1/2886	1/1441	3.58
NCT01131676	Empagliflozin	Placebo	1.99 (0.42, 9.37)	8/4687	2/2333	11.47
NCT01730534	Dapagliflozin	Placebo	0.83 (0.25, 2.73)	5/8574	6/8569	19.54
NCT01986881	Ertugliflozin	Placebo	1.50 (0.16, 14.41)	3/5493	1/2745	5.37
NCT01989754	Canagliflozin	Placebo	2.00 (0.37, 10.91)	4/2904	2/2903	9.56
NCT02065791	Canagliflozin	Placebo	0.33 (0.01, 8.17)	0/2200	1/2197	2.69
NCT03036124	Dapagliflozin	Placebo	0.50 (0.05, 5.51)	1/2368	2/2368	4.78
NCT03036150	Dapagliflozin	Placebo	1.00 (0.06, 15.98)	1/2149	1/2149	3.58
NCT03057951	Empagliflozin	Placebo	1.00 (0.25, 3.99)	4/2996	4/2989	14.34
NCT03315143	Sotagliflozin	Placebo	3.00 (0.31, 28.80)	3/5291	1/5286	5.37
NCT03521934	Sotagliflozin	Placebo	0.34 (0.01, 8.25)	0/605	1/611	2.69
NCT03594110	Empagliflozin	Placebo	1.00 (0.06, 15.99)	1/3304	1/3305	3.58
NCT03619213	Dapagliflozin	Placebo	1.00 (0.20, 4.95)	3/3126	3/3127	10.75
NCT04157751	Empagliflozin	Placebo	3.05 (0.12, 74.43)	1/260	0/264	2.69
Overall (I-squared = 0.0%, p = 0.988)			1.11 (0.66, 1.87)	35/46843	26/40287	100.00

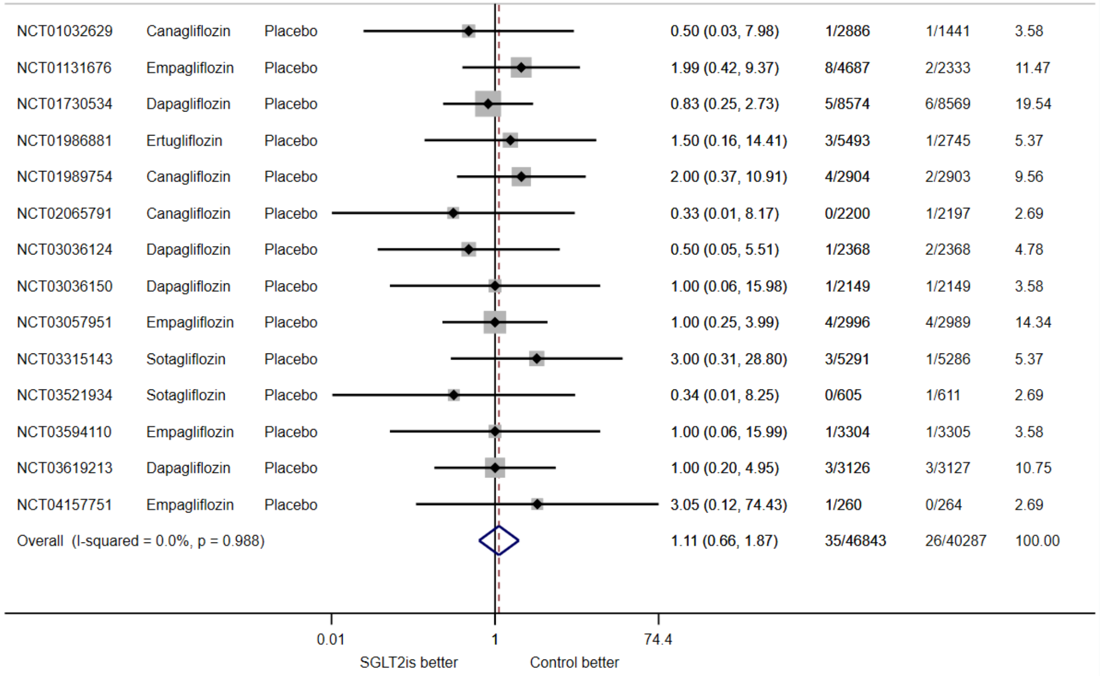

System 16-Neoplasms Benign, Malignant And Unspecified-Figure S60 Meta-analysis of SGLT2is and Lung adenocarcinoma

Trial	SGLT2is	Control	Risk Ratio (95% CI)	Events, Treatment	Events, Control	% Weight
NCT01032629	Canagliflozin	Placebo	0.75 (0.13, 4.48)	3/2886	2/1441	9.15
NCT01131676	Empagliflozin	Placebo	1.49 (0.06, 36.65)	1/4687	0/2333	2.86
NCT01730534	Dapagliflozin	Placebo	0.79 (0.36, 1.73)	11/8574	14/8569	46.96
NCT01986881	Ertugliflozin	Placebo	0.50 (0.13, 2.00)	4/5493	4/2745	15.24
NCT01989754	Canagliflozin	Placebo	1.00 (0.06, 15.97)	1/2904	1/2903	3.81
NCT02065791	Canagliflozin	Placebo	4.99 (0.24, 103.94)	2/2200	0/2197	3.17
NCT03036124	Dapagliflozin	Placebo	7.00 (0.36, 135.44)	3/2368	0/2368	3.33
NCT03057951	Empagliflozin	Placebo	2.99 (0.12, 73.44)	1/2996	0/2989	2.86
NCT03594110	Empagliflozin	Placebo	0.09 (0.01, 1.64)	0/3304	5/3305	3.49
NCT03619213	Dapagliflozin	Placebo	0.67 (0.11, 3.99)	2/3126	3/3127	9.14
Overall (I-squared = 0.0%, p = 0.644)			0.81 (0.47, 1.40)	28/38538	29/31977	100.00

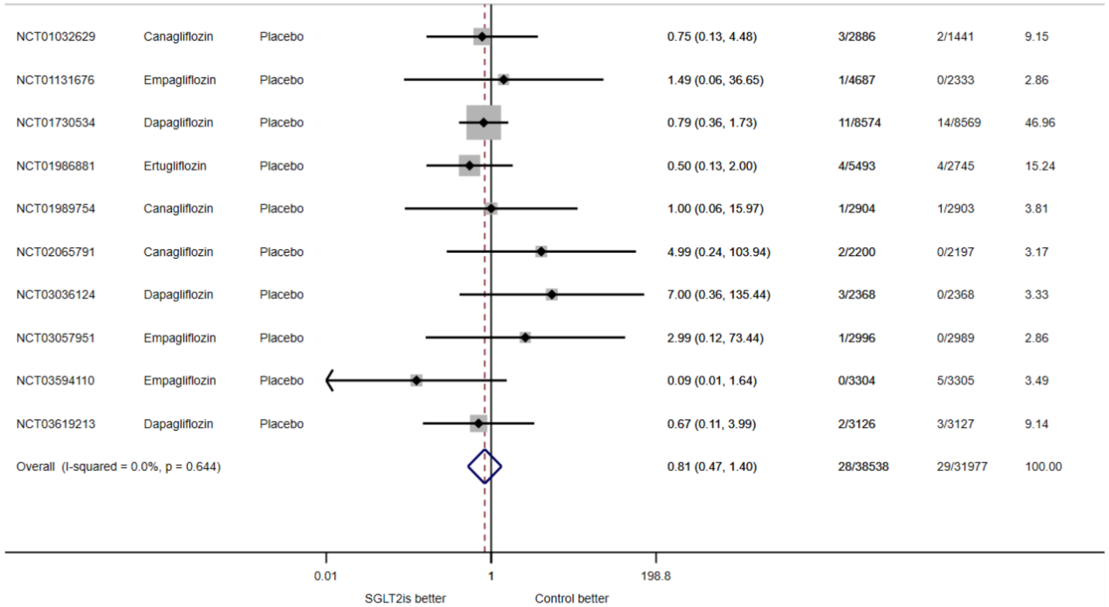

System 16-Neoplasms Benign, Malignant And Unspecified-Figure S61 Meta-analysis of SGLT2is and Lung cancer metastatic

Trial	SGLT2is	Control	Risk Ratio (95% CI)	Events, Treatment	Events, Control	% Weight
NCT01730534	Dapagliflozin	Placebo	1.00 (0.20, 4.95)	3/8574	3/8569	28.45
NCT01986881	Ertugliflozin	Placebo	1.50 (0.06, 36.80)	1/5493	0/2745	7.11
NCT02065791	Canagliflozin	Placebo	2.00 (0.18, 22.01)	2/2200	1/2197	12.65
NCT03036150	Dapagliflozin	Placebo	3.00 (0.12, 73.60)	1/2149	0/2149	7.11
NCT03057951	Empagliflozin	Placebo	0.14 (0.01, 2.76)	0/2996	3/2989	8.30
NCT03057977	Empagliflozin	Placebo	3.00 (0.12, 73.59)	1/1863	0/1863	7.11
NCT03315143	Sotagliflozin	Placebo	0.33 (0.01, 8.17)	0/5291	1/5286	7.11
NCT03521934	Sotagliflozin	Placebo	1.01 (0.06, 16.11)	1/605	1/611	9.50
NCT03619213	Dapagliflozin	Placebo	0.50 (0.05, 5.51)	1/3126	2/3127	12.65
Overall (I-squared = 0.0%, p = 0.883)			0.95 (0.40, 2.22)	10/32297	11/29536	100.00

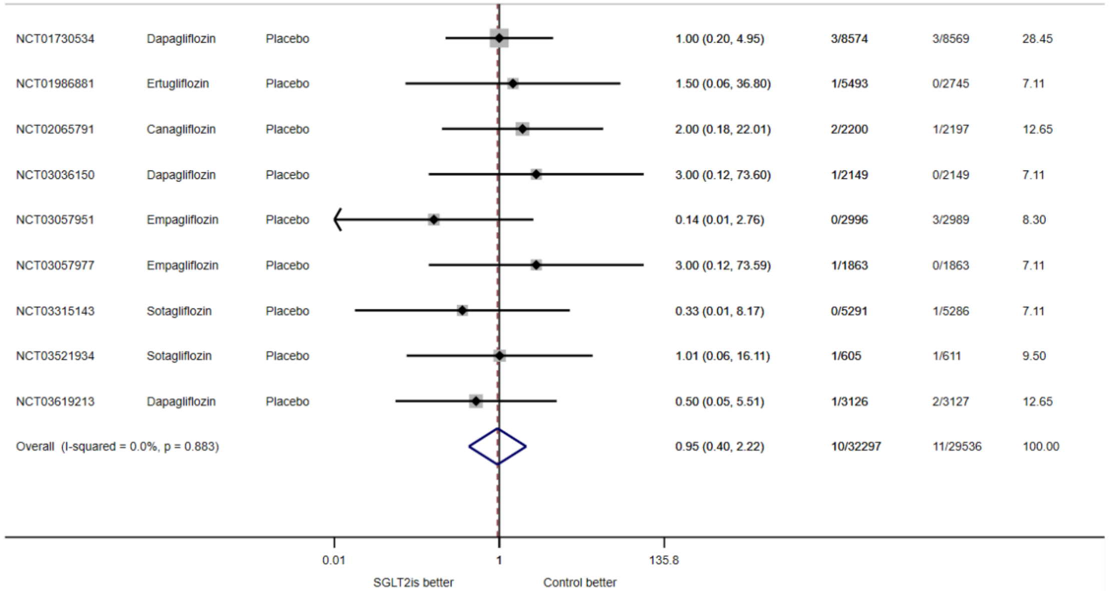

System 16-Neoplasms Benign, Malignant And Unspecified-Figure S62 Meta-analysis of SGLT2is and Lung neoplasm

Trial	SGLT2is	Control	Risk Ratio (95% CI)	Events, Treatment	Events, Control	% Weight
NCT00528879	Dapagliflozin	Placebo	0.11 (0.00, 2.74)	0/409	1/137	1.16
NCT01032629	Canagliflozin	Placebo	0.87 (0.26, 2.98)	7/2886	4/1441	7.85
NCT01131676	Empagliflozin	Placebo	1.33 (0.35, 5.00)	8/4687	3/2333	6.72
NCT01177813	Empagliflozin	Sitagliptin or Placebo	3.03 (0.12, 74.27)	1/447	0/452	1.16
NCT01730534	Dapagliflozin	Placebo	0.57 (0.28, 1.16)	12/8574	21/8569	23.54
NCT01986881	Ertugliflozin	Placebo	0.80 (0.26, 2.44)	8/5493	5/2745	9.48
NCT01989754	Canagliflozin	Placebo	1.00 (0.32, 3.10)	6/2904	6/2903	9.25
NCT02065791	Canagliflozin	Placebo	0.67 (0.11, 3.98)	2/2200	3/2197	3.70
NCT03036124	Dapagliflozin	Placebo	1.50 (0.42, 5.31)	6/2368	4/2368	7.40
NCT03036150	Dapagliflozin	Placebo	0.33 (0.07, 1.65)	2/2149	6/2149	4.62
NCT03057951	Empagliflozin	Placebo	1.66 (0.61, 4.57)	10/2996	6/2989	11.57
NCT03057977	Empagliflozin	Placebo	0.20 (0.01, 4.16)	0/1863	2/1863	1.28
NCT03315143	Sotagliflozin	Placebo	1.00 (0.14, 7.09)	2/5291	2/5286	3.08
NCT03594110	Empagliflozin	Placebo	7.00 (0.36, 135.50)	3/3304	0/3305	1.35
NCT03619213	Dapagliflozin	Placebo	0.57 (0.17, 1.95)	4/3126	7/3127	7.85
Overall (I-squared = 0.0%, p = 0.687)			0.84 (0.60, 1.19)	71/48697	70/41864	100.00

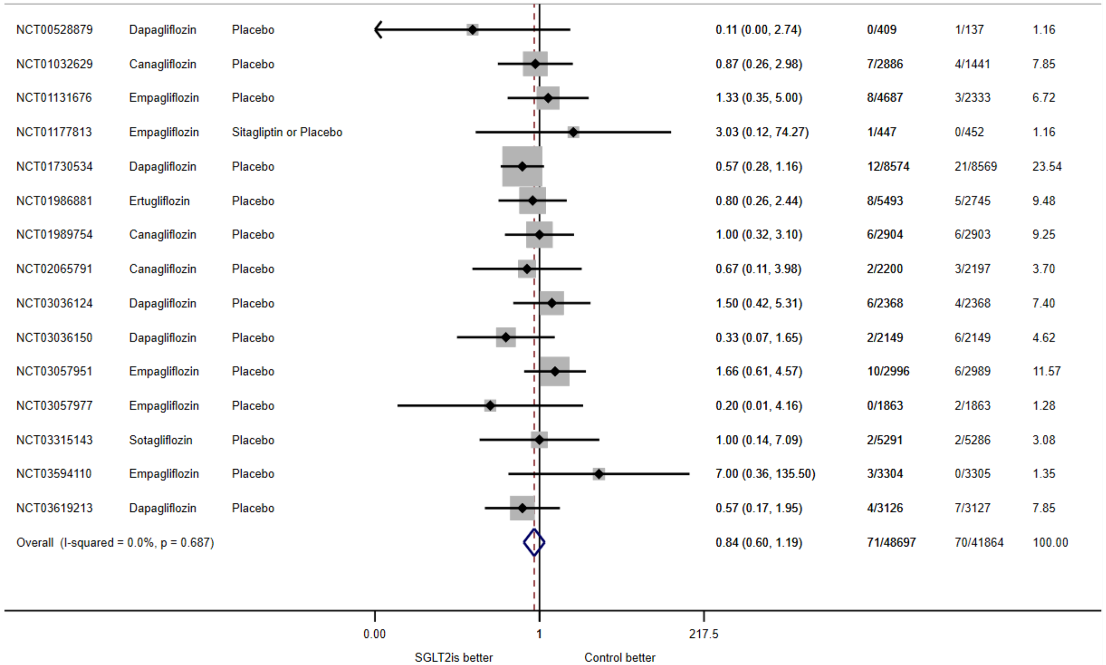

System 16-Neoplasms Benign, Malignant And Unspecified-Figure S63 Meta-analysis of SGLT2is and Lung neoplasm malignant

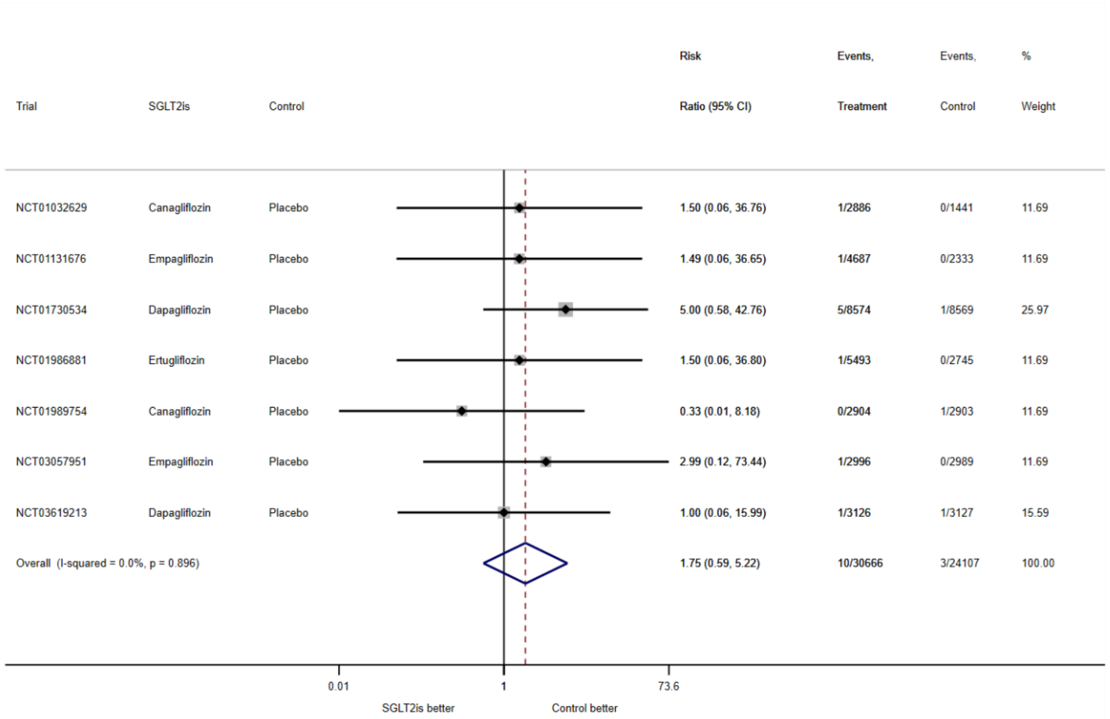

System 16-Neoplasms Benign, Malignant And Unspecified-Figure S64 Meta-analysis of SGLT2is and Lymphoma

Trial	SGLT2is	Control	Risk Ratio (95% CI)	Events, Treatment	Events, Control	% Weight
NCT00528879	Dapagliflozin	Placebo	1.01 (0.04, 24.64)	1/409	0/137	2.29
NCT01032629	Canagliflozin	Placebo	0.17 (0.01, 4.08)	0/2886	1/1441	2.29
NCT01131676	Empagliflozin	Placebo	2.99 (0.36, 24.79)	6/4687	1/2333	5.23
NCT01164501	Empagliflozin	Placebo	2.29 (0.09, 55.92)	1/419	0/319	2.29
NCT01730534	Dapagliflozin	Placebo	1.08 (0.49, 2.37)	13/8574	12/8569	38.10
NCT01986881	Ertugliflozin	Placebo	0.60 (0.18, 1.96)	6/5493	5/2745	16.65
NCT01989754	Canagliflozin	Placebo	7.00 (0.36, 135.41)	3/2904	0/2903	2.67
NCT02065791	Canagliflozin	Placebo	0.33 (0.01, 8.17)	0/2200	1/2197	2.29
NCT03036124	Dapagliflozin	Placebo	0.20 (0.01, 4.16)	0/2368	2/2368	2.54
NCT03057951	Empagliflozin	Placebo	1.33 (0.30, 5.94)	4/2996	3/2989	10.46
NCT03057977	Empagliflozin	Placebo	0.33 (0.01, 8.18)	0/1863	1/1863	2.29
NCT03315143	Sotagliflozin	Placebo	1.00 (0.14, 7.09)	2/5291	2/5286	6.10
NCT03594110	Empagliflozin	Placebo	2.00 (0.18, 22.05)	2/3304	1/3305	4.07
NCT03619213	Dapagliflozin	Placebo	9.00 (0.48, 167.15)	4/3126	0/3127	2.74
Overall (I-squared = 0.0%, p = 0.737)			1.06 (0.65, 1.72)	42/46520	29/39582	100.00

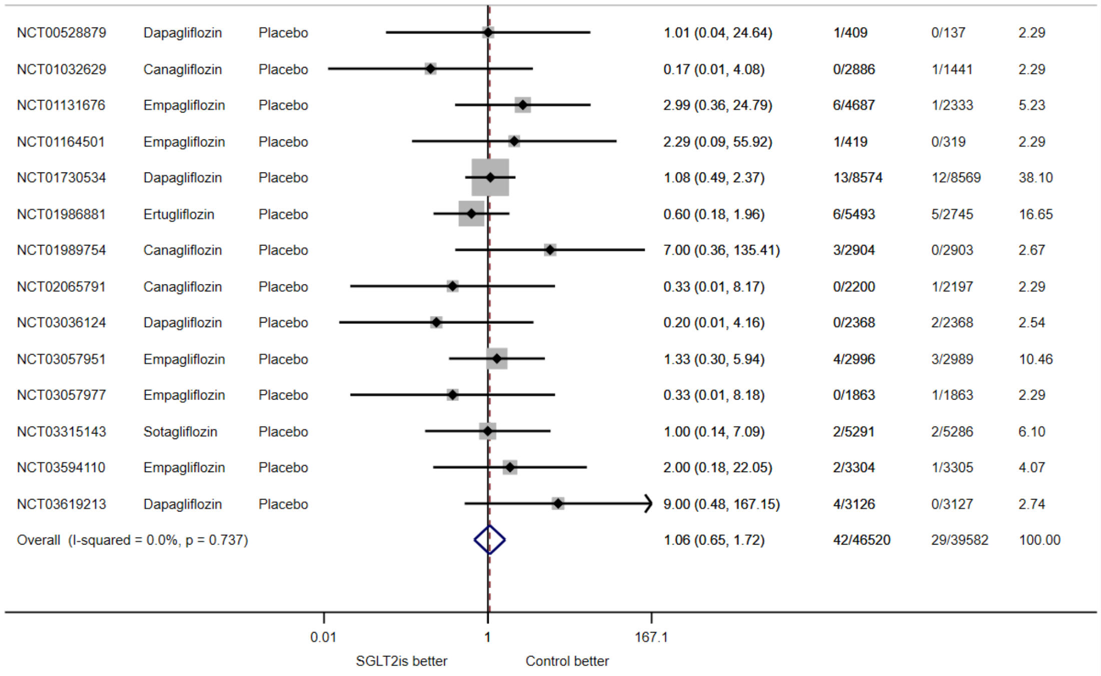

System 16-Neoplasms Benign, Malignant And Unspecified-Figure S65 Meta-analysis of SGLT2is and Malignant melanoma

Trial	SGLT2is	Control	Risk Ratio (95% CI)	Events, Treatment	Events, Control	% Weight
NCT01032629	Canagliflozin	Placebo	2.50 (0.12, 51.99)	2/2886	0/1441	7.96
NCT01131676	Empagliflozin	Placebo	2.49 (0.12, 51.83)	2/4687	0/2333	7.96
NCT01730534	Dapagliflozin	Placebo	1.50 (0.42, 5.31)	6/8574	4/8569	45.87
NCT01986881	Ertugliflozin	Placebo	0.50 (0.03, 7.99)	1/5493	1/2745	9.55
NCT01989754	Canagliflozin	Placebo	3.00 (0.12, 73.59)	1/2904	0/2903	7.16
NCT03057951	Empagliflozin	Placebo	0.33 (0.01, 8.16)	0/2996	1/2989	7.16
NCT03315143	Sotagliflozin	Placebo	0.33 (0.01, 8.17)	0/5291	1/5286	7.16
NCT03594110	Empagliflozin	Placebo	0.33 (0.01, 8.18)	0/3304	1/3305	7.16
Overall (I-squared = 0.0%, p = 0.877)			1.11 (0.47, 2.62)	12/36135	8/29571	100.00

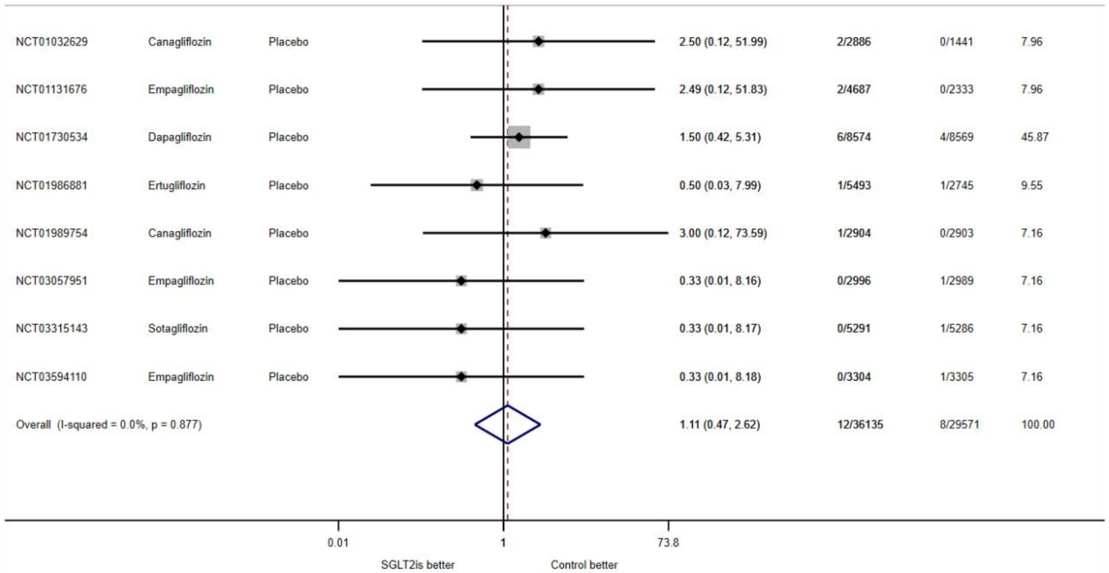

System 16-Neoplasms Benign, Malignant And Unspecified-Figure S66 Meta-analysis of SGLT2is and Malignant melanoma in situ

Trial	SGLT2is	Control	Risk Ratio (95% CI)	Events, Treatment	Events, Control	% Weight
NCT01730534	Dapagliflozin	Placebo	3.00 (0.12, 73.59)	1/8574	0/8569	25.00
NCT01986881	Ertugliflozin	Placebo	1.50 (0.06, 36.80)	1/5493	0/2745	25.00
NCT03057951	Empagliflozin	Placebo	2.99 (0.12, 73.44)	1/2996	0/2989	25.00
NCT03594110	Empagliflozin	Placebo	0.33 (0.01, 8.18)	0/3304	1/3305	25.00
Overall (I-squared = 0.0%, p = 0.752)			1.46 (0.29, 7.21)	3/20367	1/17608	100.00

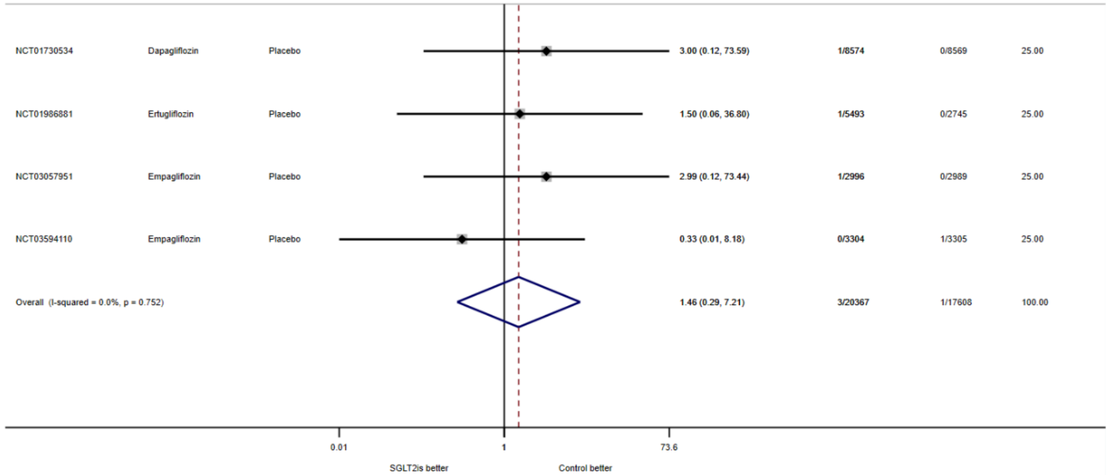

System 16-Neoplasms Benign, Malignant And Unspecified-Figure S67 Meta-analysis of SGLT2is and Malignant neoplasm of renal pelvis

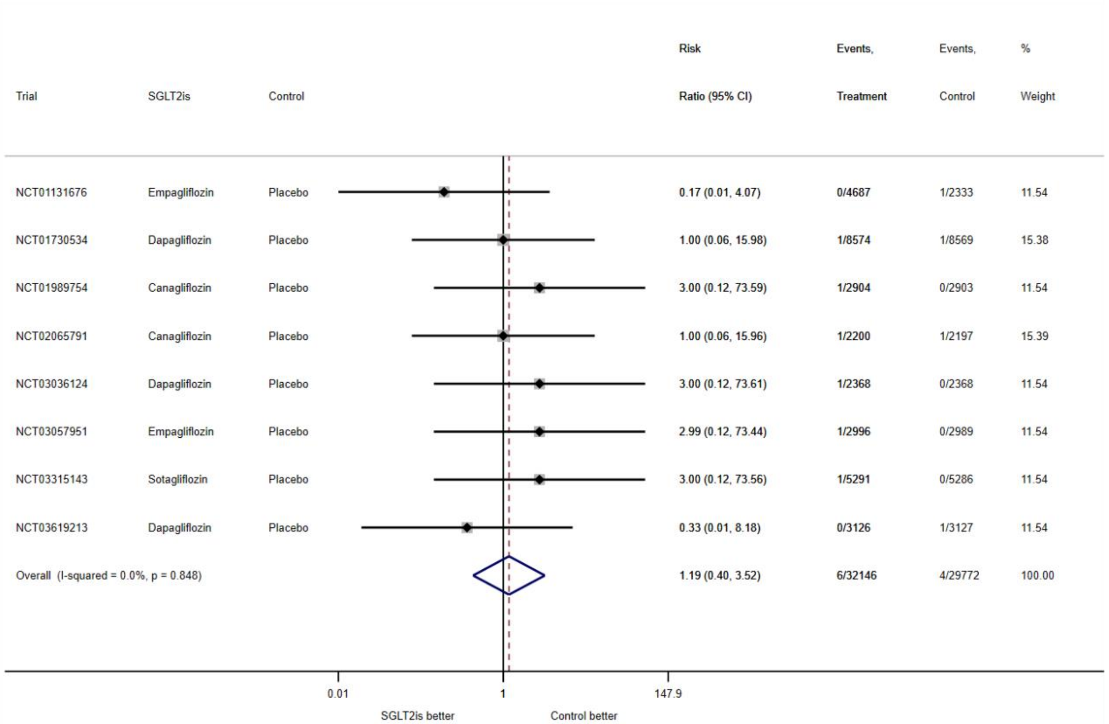

System 16-Neoplasms Benign, Malignant And Unspecified-Figure S68 Meta-analysis of SGLT2is and Malignant neoplasm of unknown primary site

Trial	SGLT2is	Control	Risk Ratio (95% CI)	Events, Treatment	Events, Control	% Weight
NCT01032629	Canagliflozin	Placebo	3.50 (0.18, 67.64)	3/2886	0/1441	9.48
NCT01042977	Dapagliflozin	Placebo	3.01 (0.12, 73.61)	1/482	0/483	8.14
NCT01106677	Canagliflozin	Sitagliptin	1.50 (0.06, 36.63)	1/735	0/366	8.14
NCT01131676	Empagliflozin	Placebo	2.49 (0.12, 51.83)	2/4687	0/2333	9.03
NCT01730534	Dapagliflozin	Placebo	4.00 (0.45, 35.76)	4/8574	1/8569	17.34
NCT01986881	Ertugliflozin	Placebo	2.50 (0.12, 52.04)	2/5493	0/2745	9.03
NCT03036124	Dapagliflozin	Placebo	0.33 (0.01, 8.18)	0/2368	1/2368	8.13
NCT03057951	Empagliflozin	Placebo	0.50 (0.05, 5.50)	1/2996	2/2989	14.45
NCT03619213	Dapagliflozin	Placebo	3.00 (0.12, 73.64)	1/3126	0/3127	8.13
NCT04157751	Empagliflozin	Placebo	3.05 (0.12, 74.43)	1/260	0/264	8.15
Overall (I-squared = 0.0%, p = 0.953)			1.89 (0.76, 4.71)	16/31607	4/24685	100.00

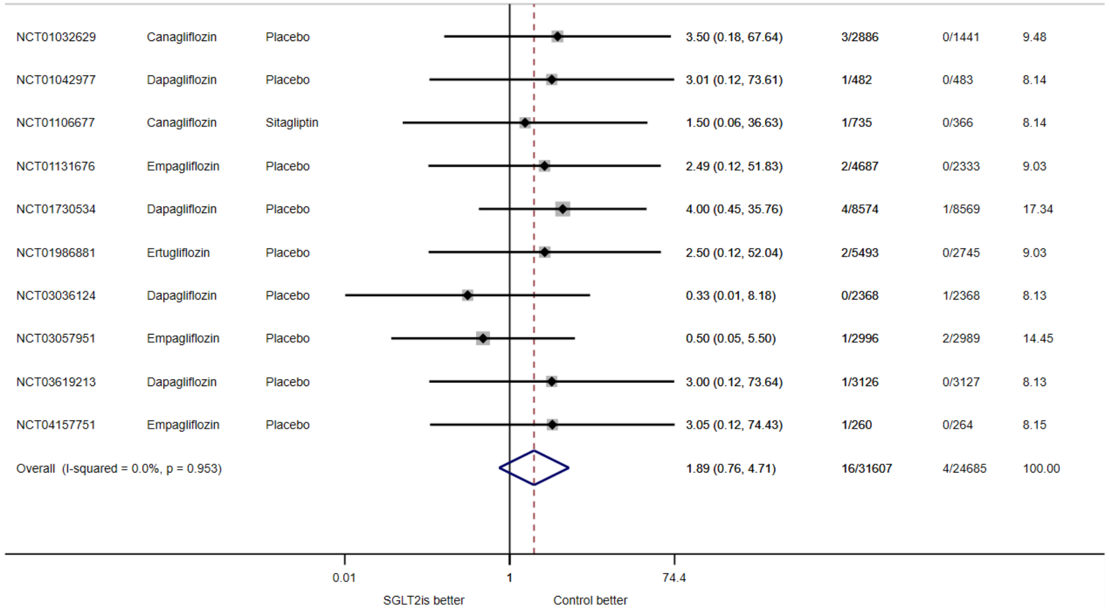

System 16-Neoplasms Benign, Malignant And Unspecified-Figure S69 Meta-analysis of SGLT2is and Meningioma

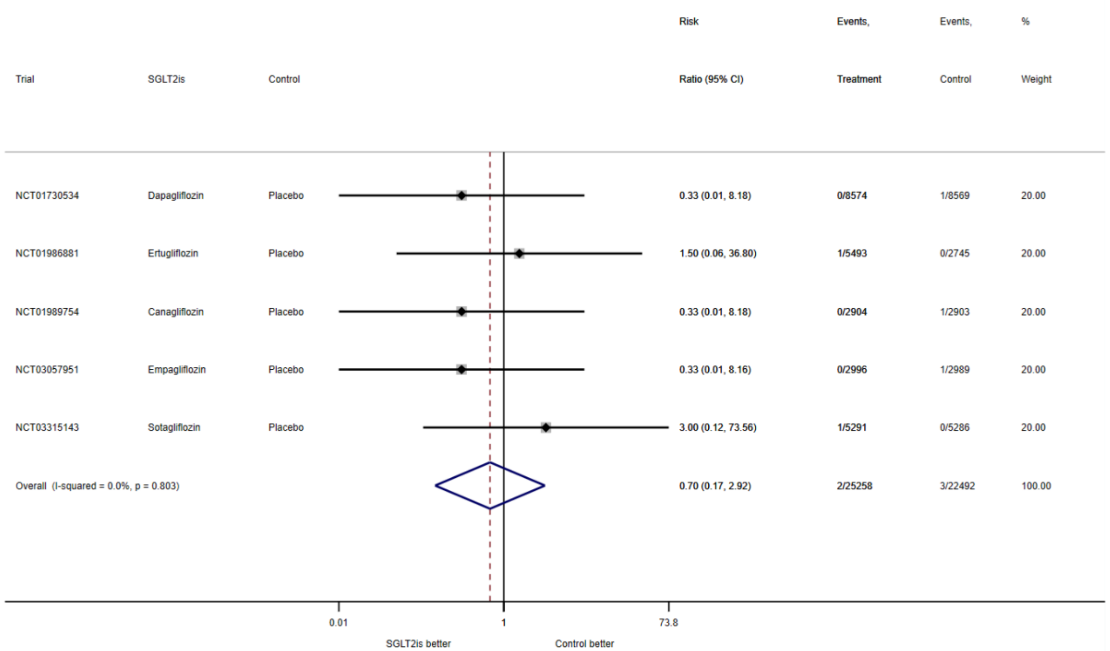

System 16-Neoplasms Benign, Malignant And Unspecified-Figure S70 Meta-analysis of SGLT2is and Meningioma benign

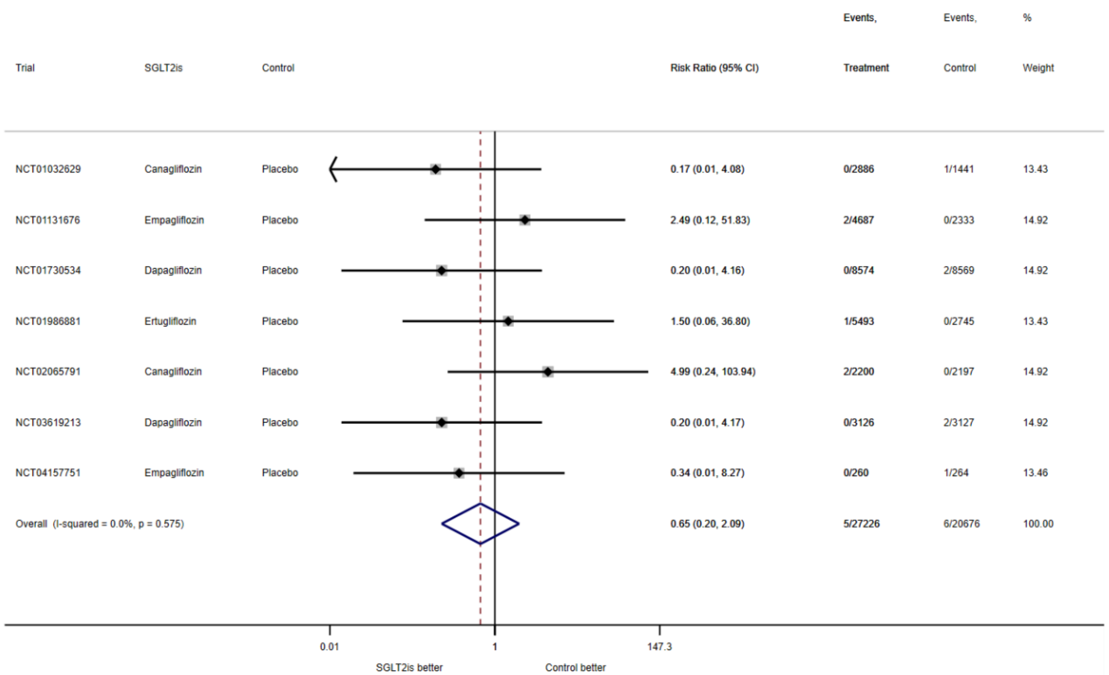

System 16-Neoplasms Benign, Malignant And Unspecified-Figure S71 Meta-analysis of SGLT2is and Metastases to bone

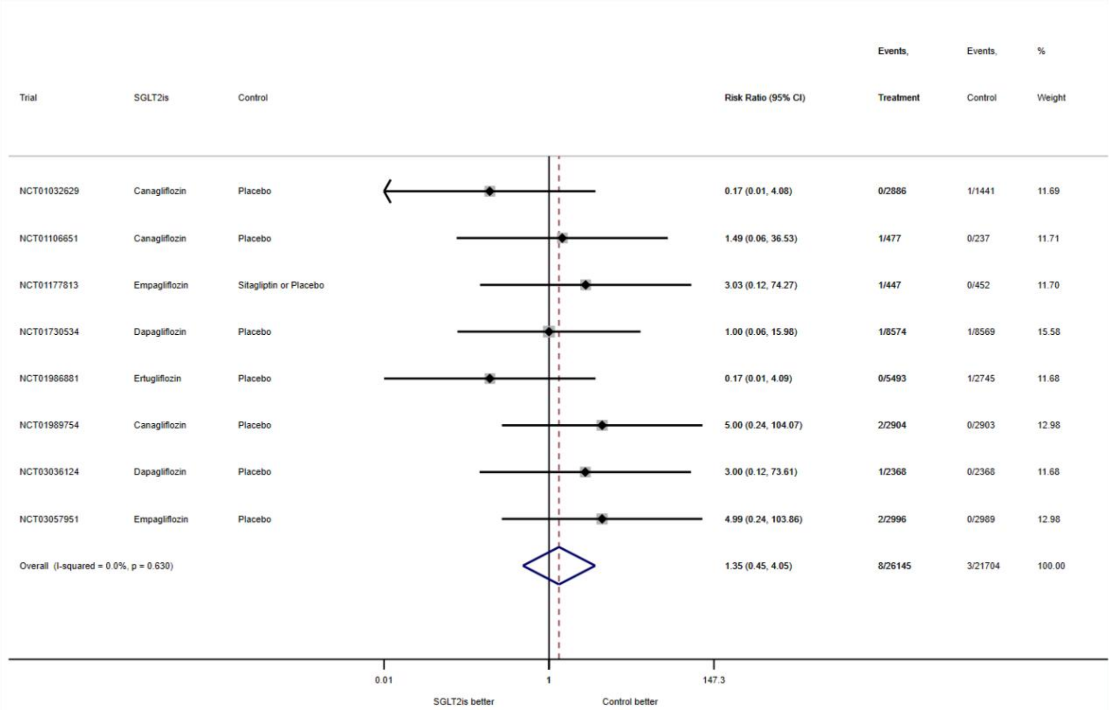

System 16-Neoplasms Benign, Malignant And Unspecified-Figure S72 Meta-analysis of SGLT2is and Metastases to central nervous system

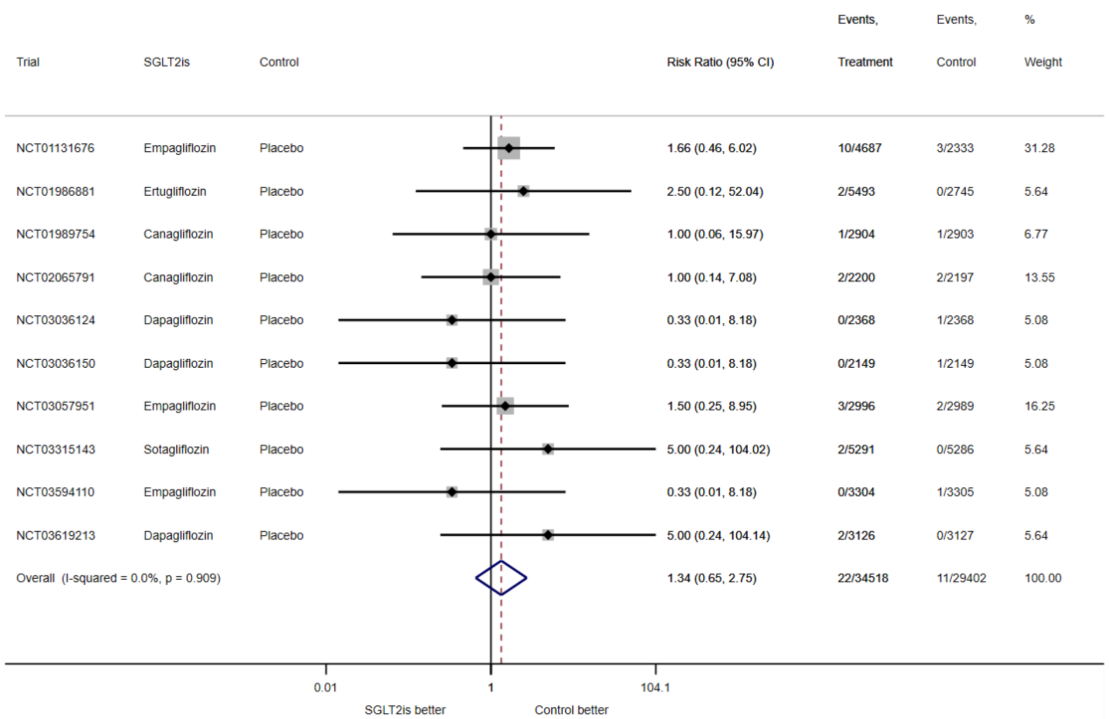

System 16-Neoplasms Benign, Malignant And Unspecified-Figure S73 Meta-analysis of SGLT2is and Metastases to liver

Trial	SGLT2is	Control	Risk Ratio (95% CI)	Events, Treatment	Events, Control	% Weight
NCT01131676	Empagliflozin	Placebo	1.99 (0.22, 17.80)	4/4687	1/2333	30.39
NCT01730534	Dapagliflozin	Placebo	1.00 (0.06, 15.98)	1/8574	1/8569	18.98
NCT03057951	Empagliflozin	Placebo	1.00 (0.06, 15.94)	1/2996	1/2989	18.99
NCT03315143	Sotagliflozin	Placebo	5.00 (0.24, 104.02)	2/5291	0/5286	15.82
NCT03594110	Empagliflozin	Placebo	5.00 (0.24, 104.14)	2/3304	0/3305	15.82
Overall (I-squared = 0.0%, p = 0.881)			2.05 (0.61, 6.86)	10/24852	3/22482	100.00

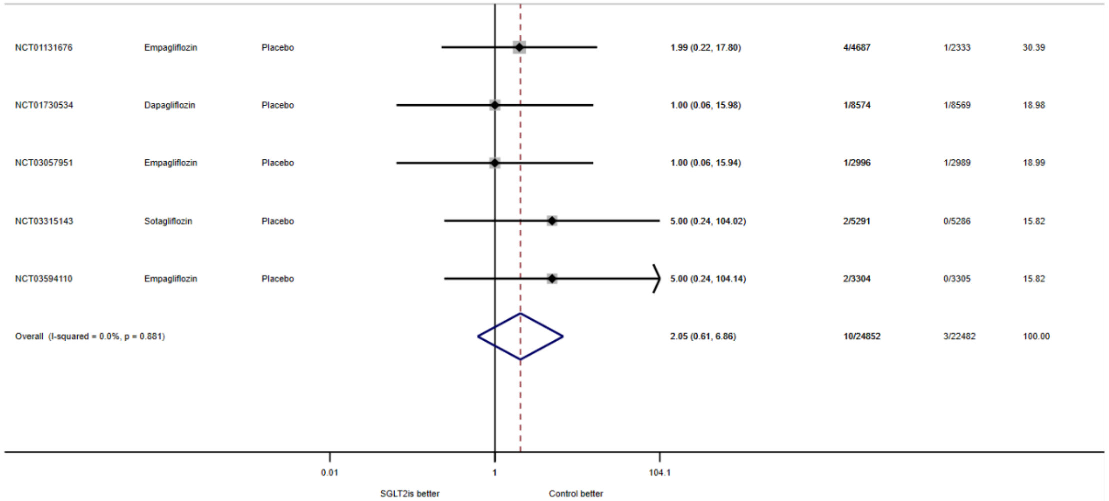

System 16-Neoplasms Benign, Malignant And Unspecified-Figure S74 Meta-analysis of SGLT2is and Metastases to lung

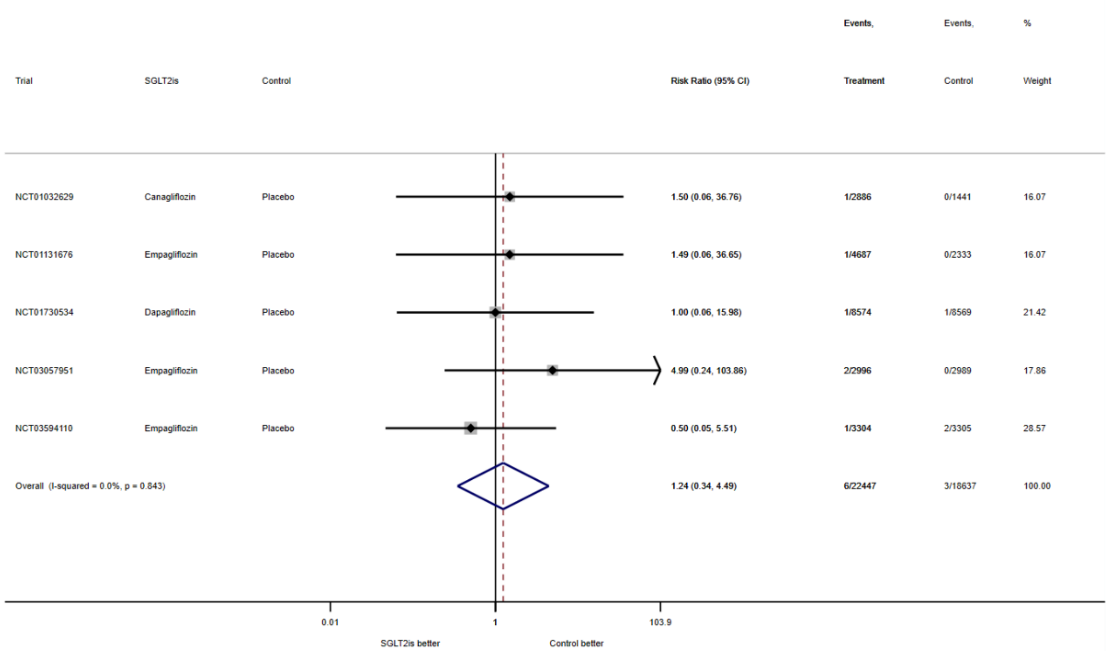

System 16-Neoplasms Benign, Malignant And Unspecified-Figure S75 Meta-analysis of SGLT2is and Metastases to lymph nodes

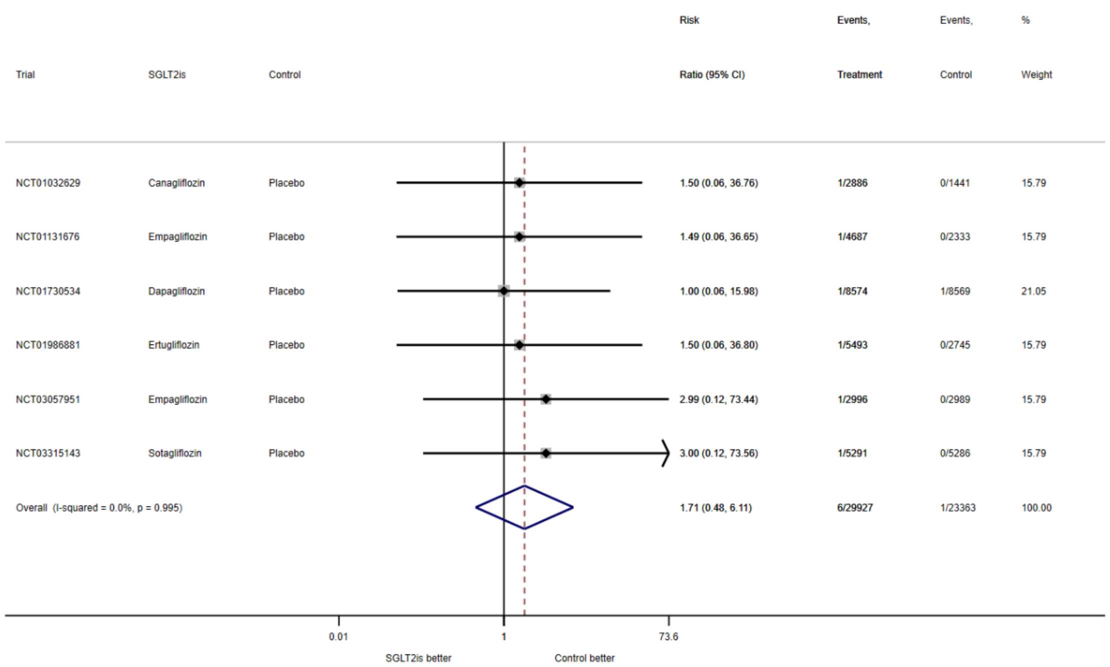

System 16-Neoplasms Benign, Malignant And Unspecified-Figure S76 Meta-analysis of SGLT2is and Metastases to peritoneum

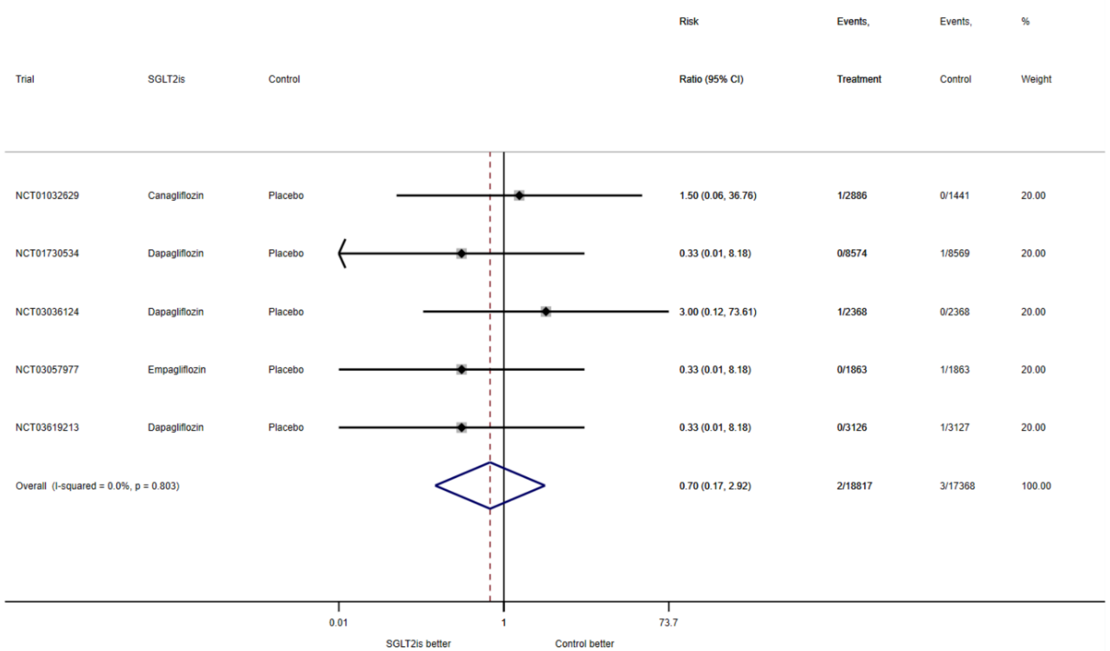

System 16-Neoplasms Benign, Malignant And Unspecified-Figure S77 Meta-analysis of SGLT2is and Metastatic carcinoma of the bladder

Trial	SGLT2is	Control	Risk Ratio (95% CI)	Events, Treatment	Events, Control	% Weight
NCT01032629	Canagliflozin	Placebo	1.50 (0.06, 36.76)	1/2886	0/1441	8.33
NCT01131676	Empagliflozin	Placebo	0.17 (0.01, 4.07)	0/4687	1/2333	8.33
NCT01730534	Dapagliflozin	Placebo	0.60 (0.14, 2.51)	3/8574	5/8569	41.67
NCT01986881	Ertugliflozin	Placebo	1.50 (0.06, 36.80)	1/5493	0/2745	8.33
NCT03036124	Dapagliflozin	Placebo	3.00 (0.12, 73.61)	1/2368	0/2368	8.33
NCT03057951	Empagliflozin	Placebo	2.99 (0.12, 73.44)	1/2996	0/2989	8.33
NCT03315143	Sotagliflozin	Placebo	0.33 (0.01, 8.17)	0/5291	1/5286	8.33
NCT03594110	Empagliflozin	Placebo	0.33 (0.01, 8.18)	0/3304	1/3305	8.33
Overall (I-squared = 0.0%, p = 0.862)			0.74 (0.30, 1.87)	7/35599	8/29036	100.00

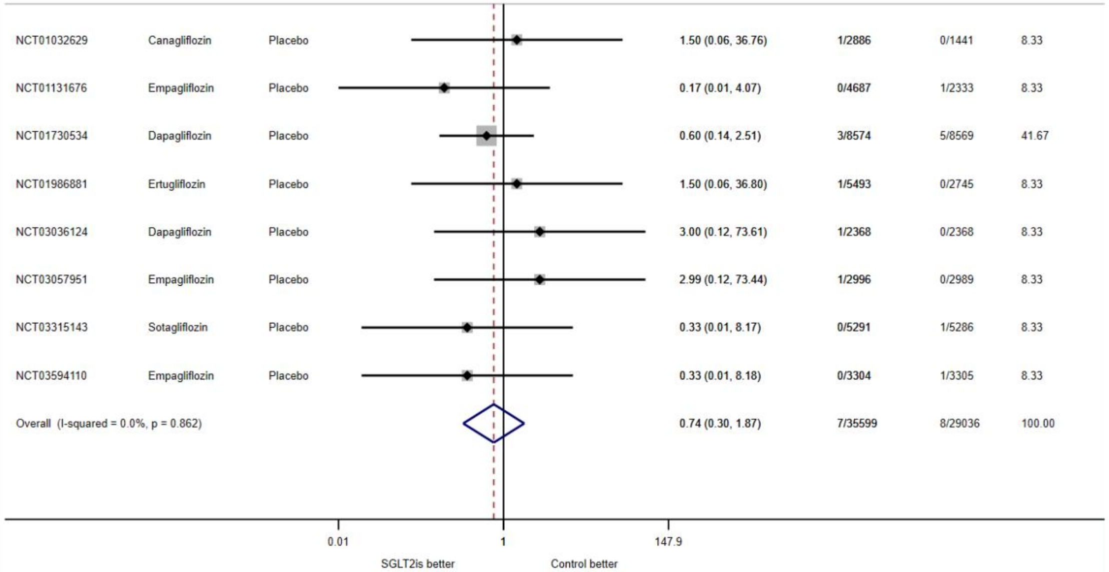

System 16-Neoplasms Benign, Malignant And Unspecified-Figure S78 Meta-analysis of SGLT2is and Metastatic malignant melanoma

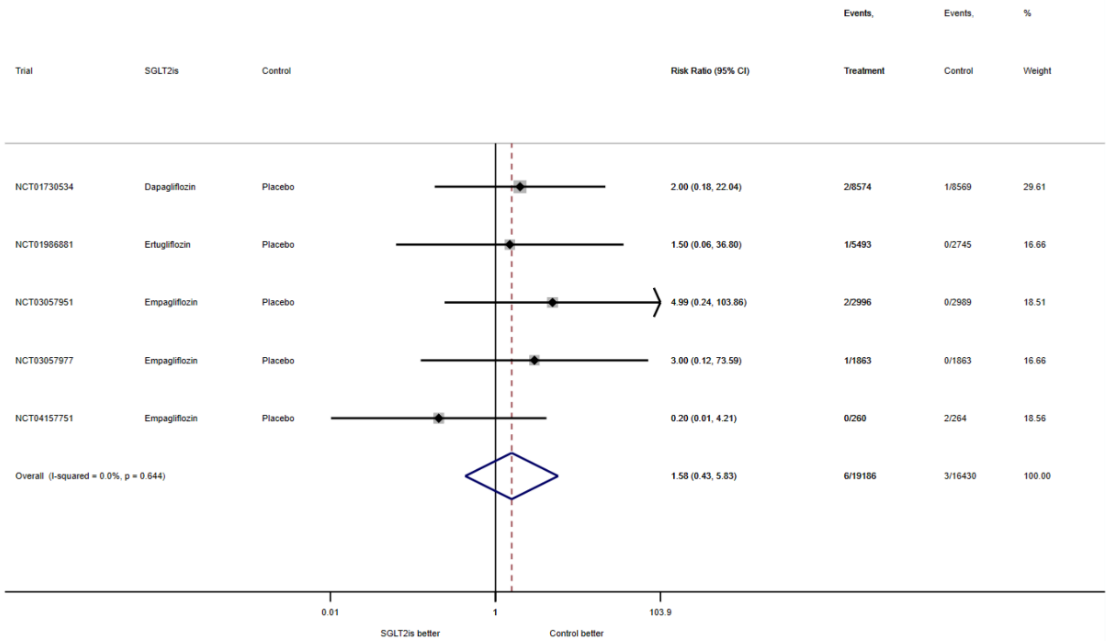

System 16-Neoplasms Benign, Malignant And Unspecified-Figure S79 Meta-analysis of SGLT2is and Metastatic neoplasm

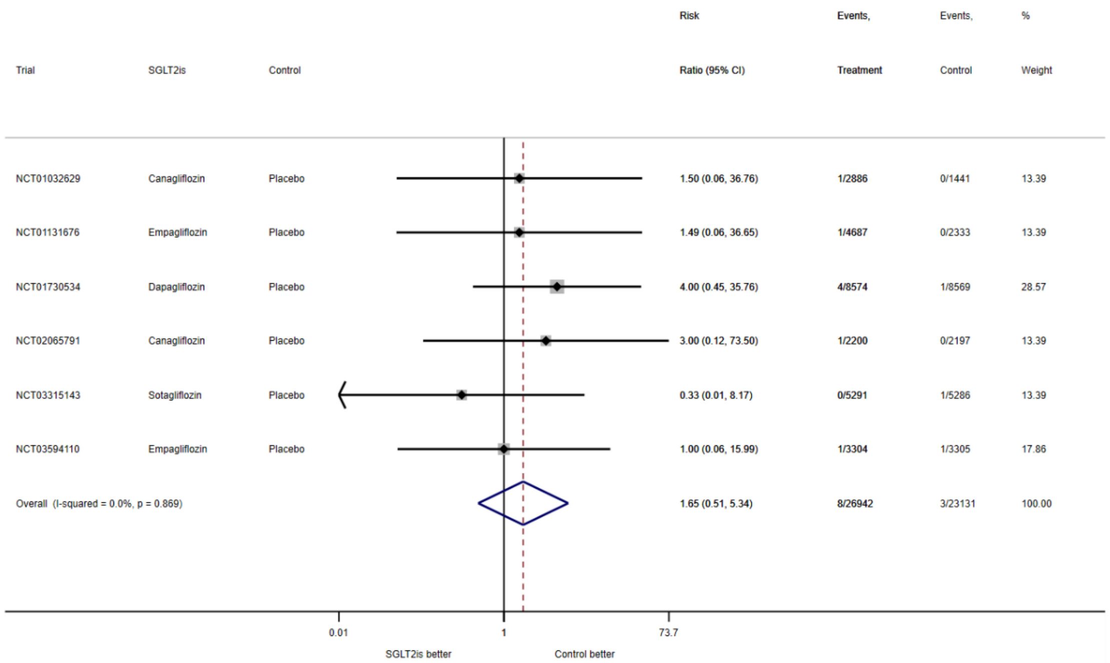

System 16-Neoplasms Benign, Malignant And Unspecified-Figure S80 Meta-analysis of SGLT2is and Metastatic renal cell carcinoma

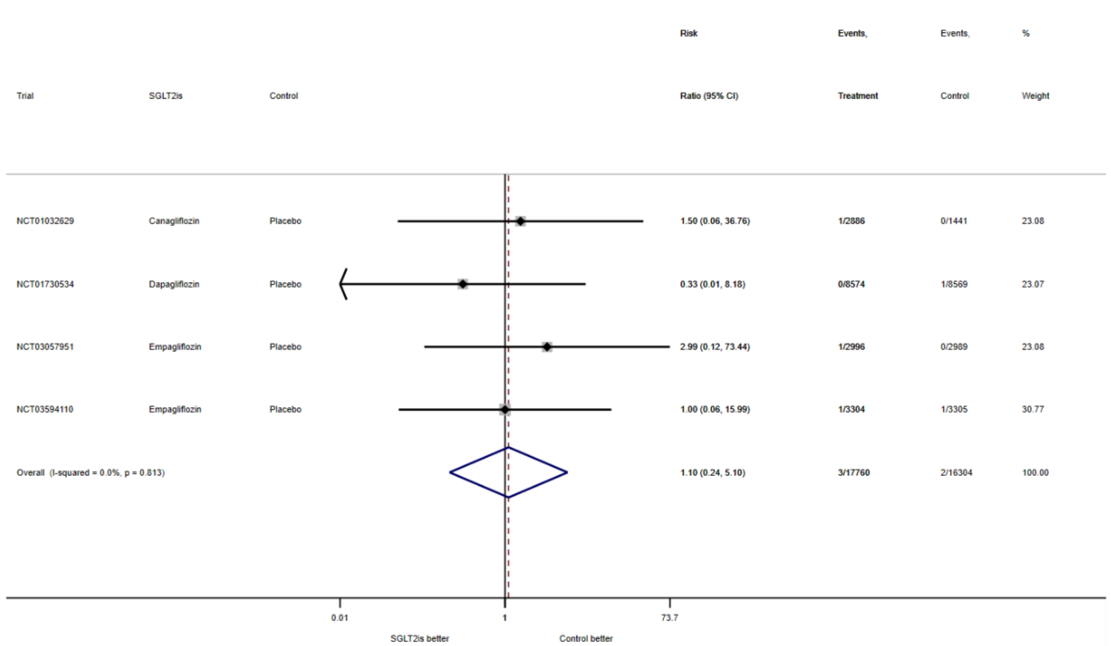

System 16-Neoplasms Benign, Malignant And Unspecified-Figure S81 Meta-analysis of SGLT2is and Monoclonal gammopathy

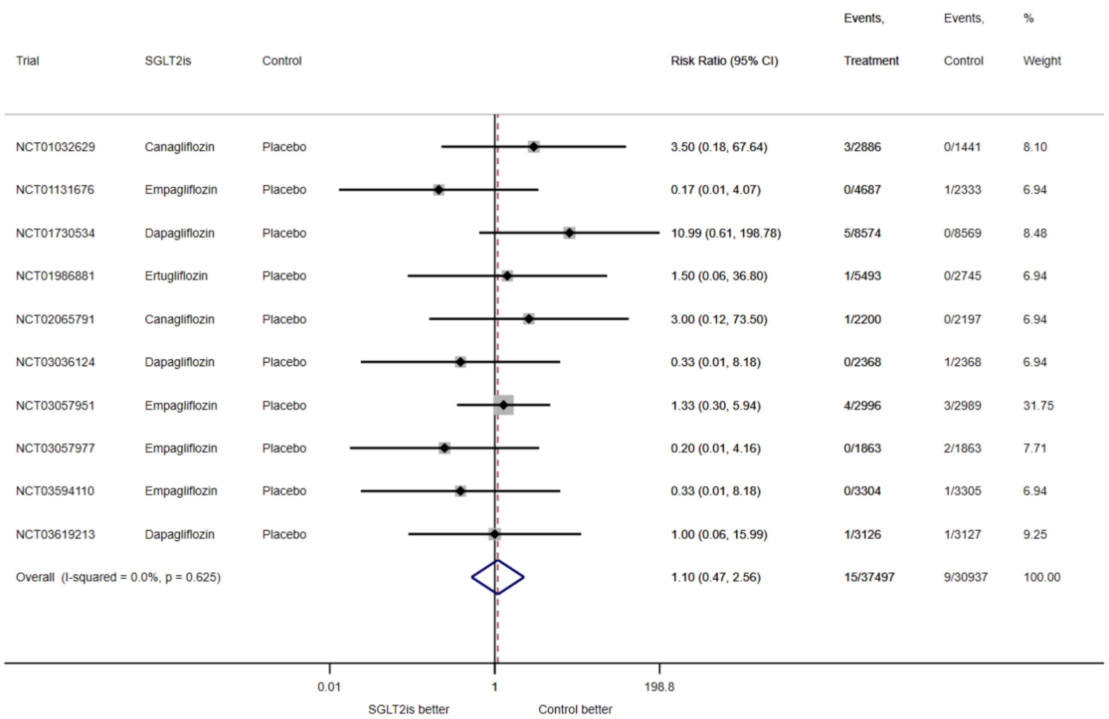

System 16-Neoplasms Benign, Malignant And Unspecified-Figure S82 Meta-analysis of SGLT2is and Myelodysplastic syndrome

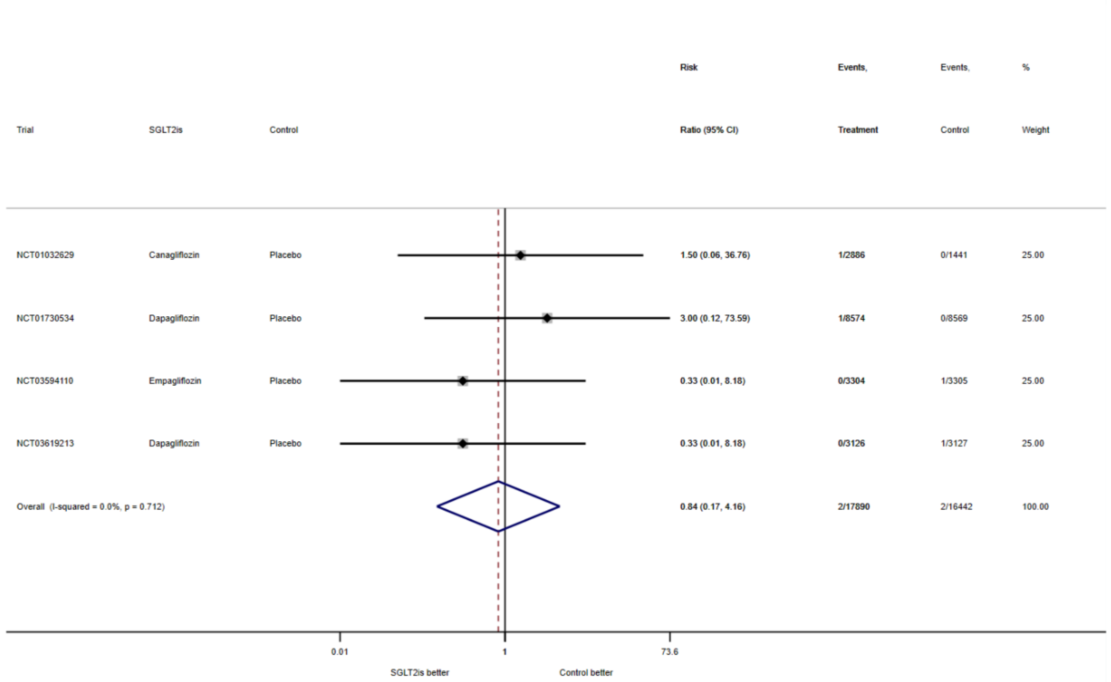

System 16-Neoplasms Benign, Malignant And Unspecified-Figure S83 Meta-analysis of SGLT2is and Myeloproliferative neoplasm

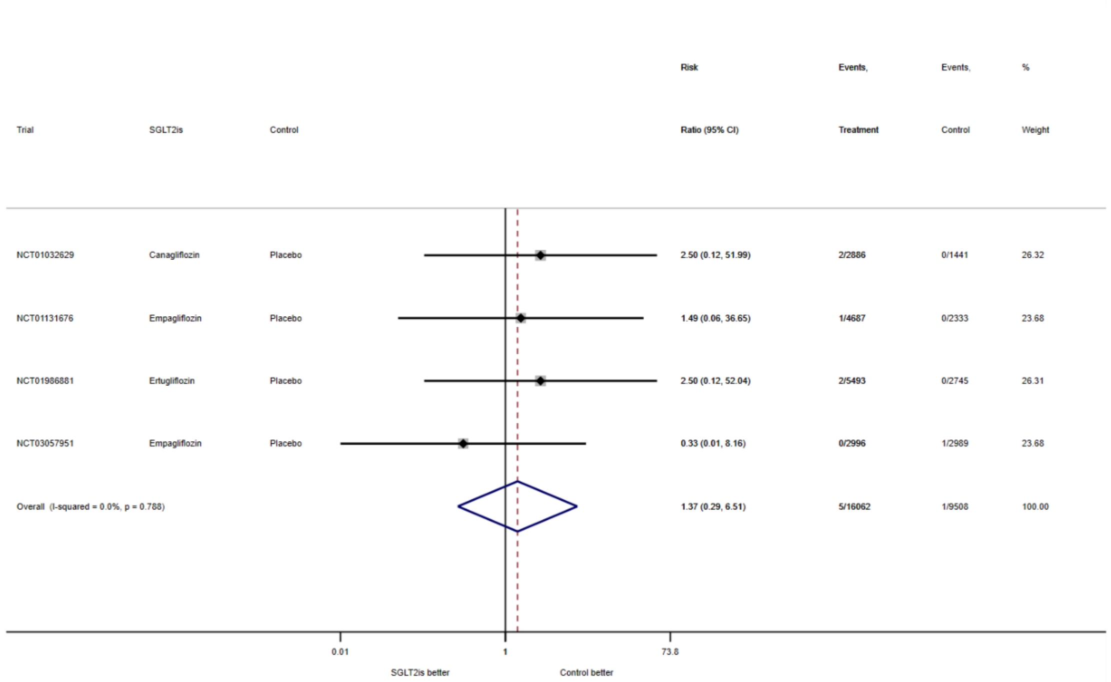

System 16-Neoplasms Benign, Malignant And Unspecified-Figure S84 Meta-analysis of SGLT2is and Neuroendocrine carcinoma of the skin

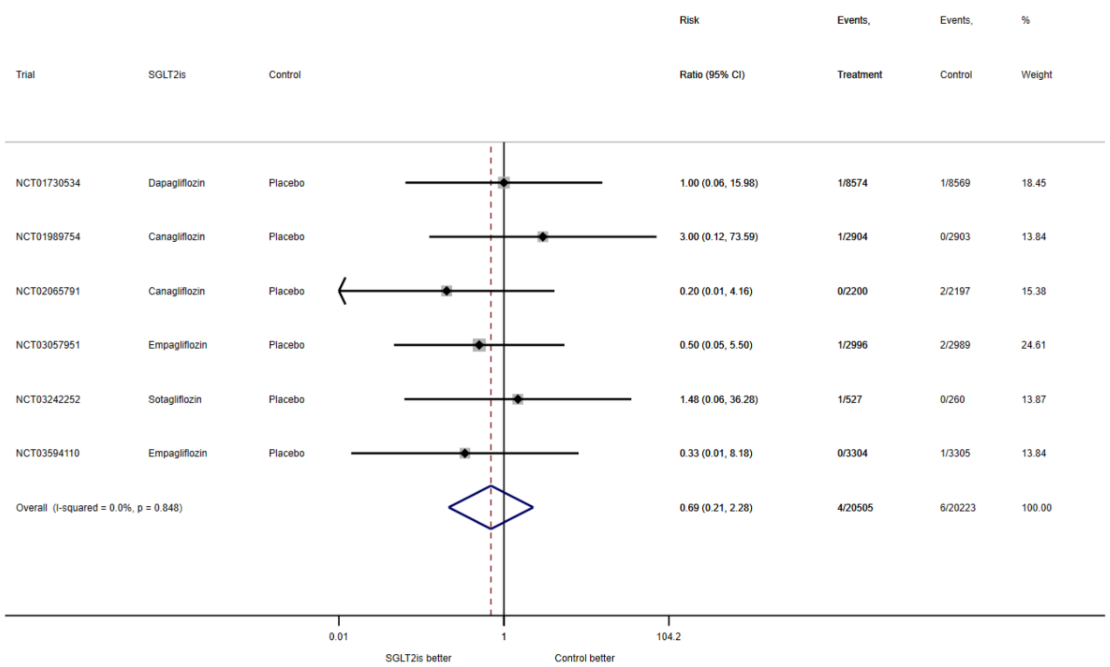

System 16-Neoplasms Benign, Malignant And Unspecified-Figure S85 Meta-analysis of SGLT2is and Neuroendocrine tumour

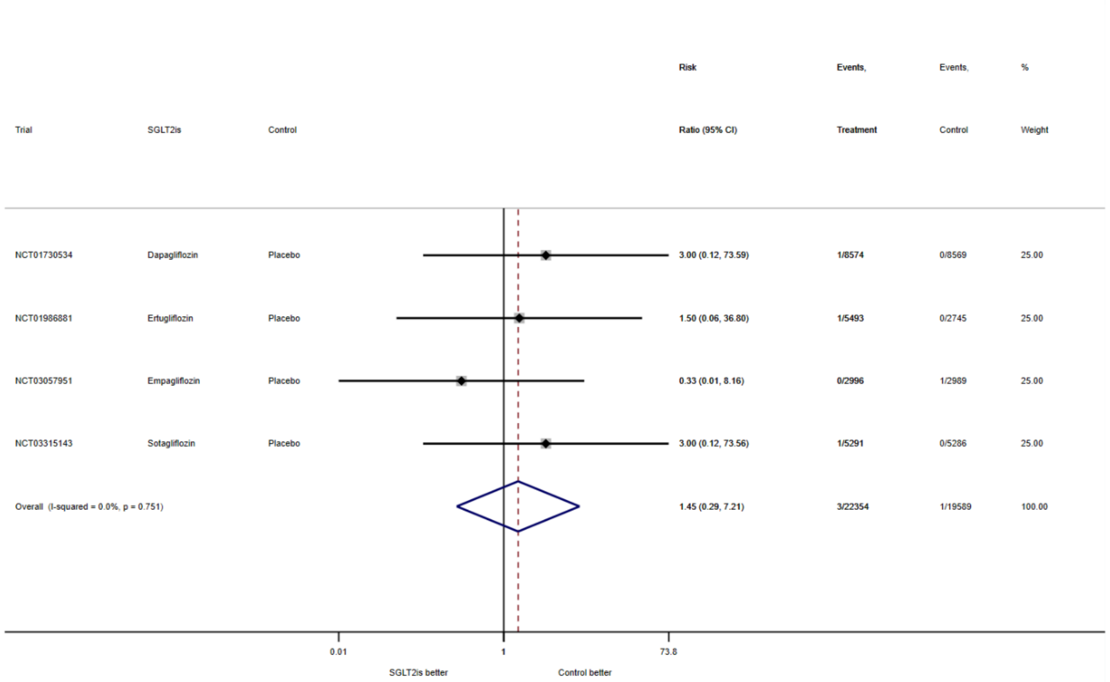

System 16-Neoplasms Benign, Malignant And Unspecified-Figure S86 Meta-analysis of SGLT2is and Neuroendocrine tumour of the lung metastatic

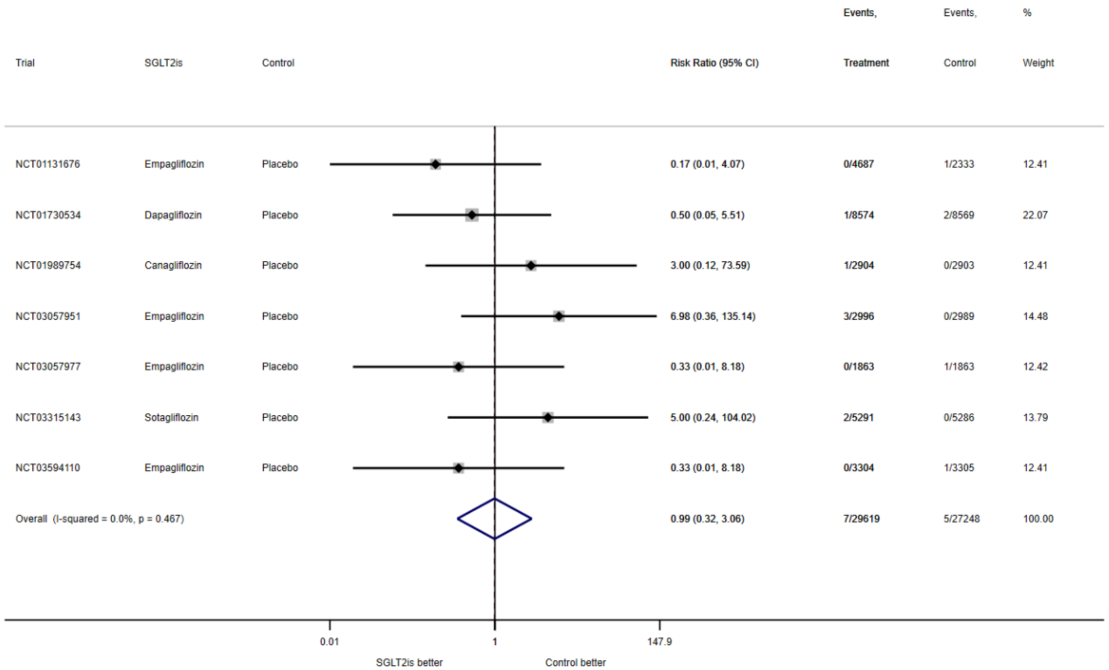

System 16-Neoplasms Benign, Malignant And Unspecified-Figure S87 Meta-analysis of SGLT2is and Non-hodgkin's lymphoma

Trial	SGLT2is	Control	Risk Ratio (95% CI)	Events, Treatment	Events, Control	% Weight
NCT01032629	Canagliflozin	Placebo	0.17 (0.01, 4.08)	0/2886	1/1441	10.00
NCT01730534	Dapagliflozin	Placebo	0.33 (0.03, 3.20)	1/8574	3/8569	20.00
NCT01989754	Canagliflozin	Placebo	0.33 (0.01, 8.18)	0/2904	1/2903	10.00
NCT03036124	Dapagliflozin	Placebo	0.33 (0.01, 8.18)	0/2368	1/2368	10.00
NCT03036150	Dapagliflozin	Placebo	0.20 (0.01, 4.16)	0/2149	2/2149	11.11
NCT03057951	Empagliflozin	Placebo	0.33 (0.01, 8.16)	0/2996	1/2989	10.00
NCT03594110	Empagliflozin	Placebo	0.20 (0.01, 4.17)	0/3304	2/3305	11.11
NCT03619213	Dapagliflozin	Placebo	0.50 (0.05, 5.51)	1/3126	2/3127	17.78
Overall (I-squared = 0.0%, p = 1.000)			0.30 (0.11, 0.82)	2/28307	13/26851	100.00

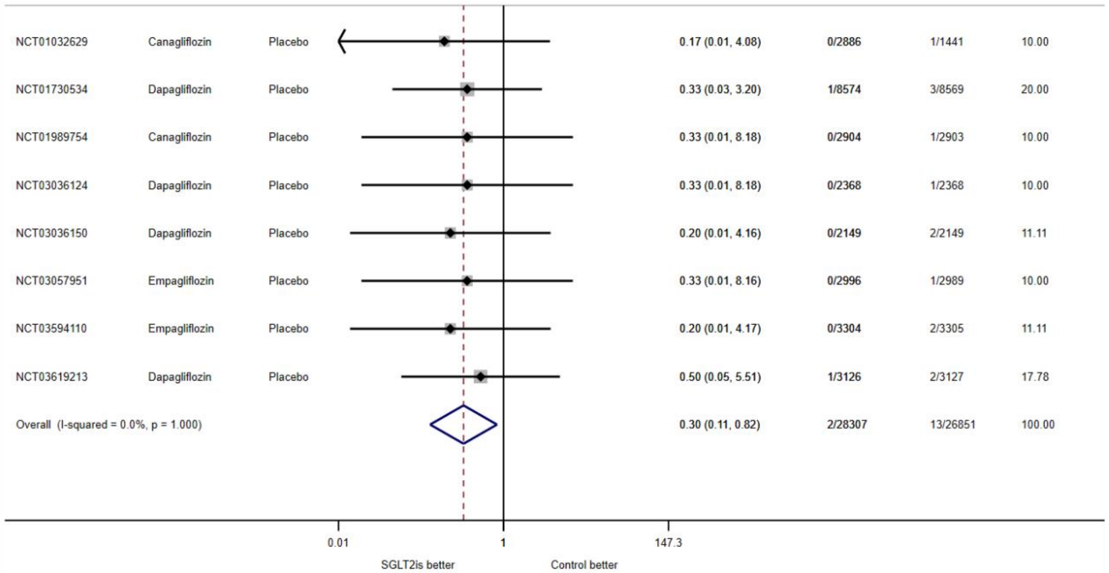

System 16-Neoplasms Benign, Malignant And Unspecified-Figure S88 Meta-analysis of SGLT2is and Non-small cell lung cancer

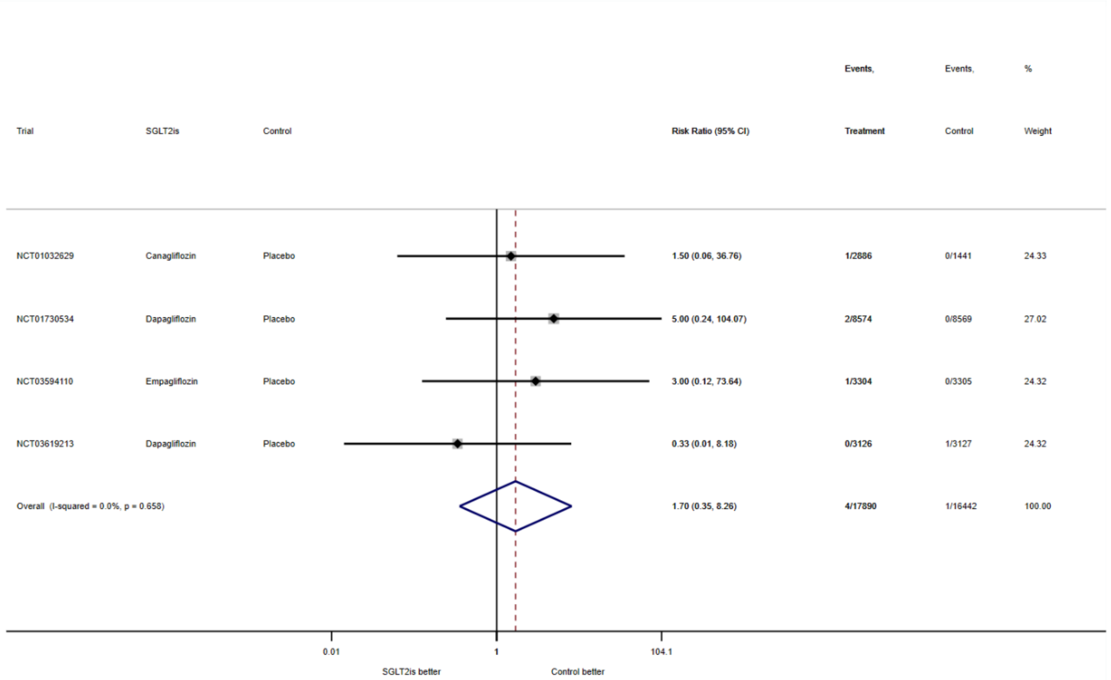

System 16-Neoplasms Benign, Malignant And Unspecified-Figure S89 Meta-analysis of SGLT2is and Non-small cell lung cancer stage iv

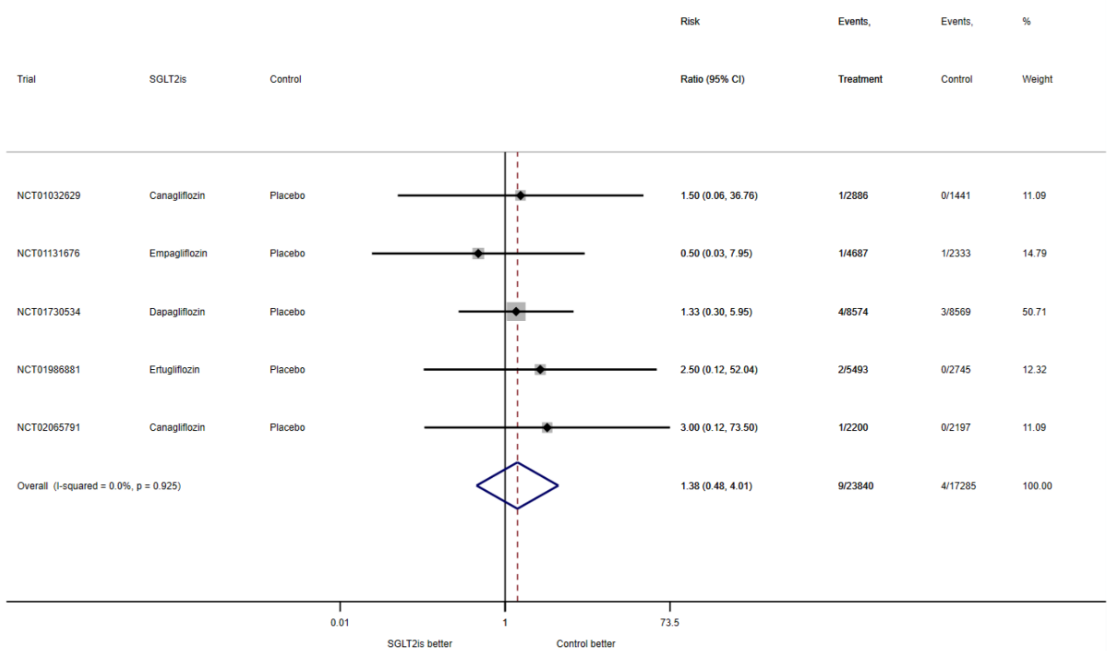

System 16-Neoplasms Benign, Malignant And Unspecified-Figure S90 Meta-analysis of SGLT2is and Oesophageal adenocarcinoma

Trial	SGLT2is	Control	Risk Ratio (95% CI)	Events, Treatment	Events, Control	% Weight
NCT01131676	Empagliflozin	Placebo	0.10 (0.00, 2.07)	0/4687	2/2333	7.20
NCT01164501	Empagliflozin	Placebo	2.29 (0.09, 55.92)	1/419	0/319	6.49
NCT01730534	Dapagliflozin	Placebo	0.25 (0.03, 2.23)	1/8574	4/8569	13.82
NCT01986881	Ertugliflozin	Placebo	3.50 (0.18, 67.71)	3/5493	0/2745	7.56
NCT03036124	Dapagliflozin	Placebo	0.33 (0.01, 8.18)	0/2368	1/2368	6.48
NCT03057951	Empagliflozin	Placebo	1.00 (0.20, 4.94)	3/2996	3/2989	25.93
NCT03057977	Empagliflozin	Placebo	0.20 (0.01, 4.16)	0/1863	2/1863	7.20
NCT03315143	Sotagliflozin	Placebo	4.00 (0.45, 35.74)	4/5291	1/5286	13.82
NCT03619213	Dapagliflozin	Placebo	2.00 (0.18, 22.05)	2/3126	1/3127	11.52
Overall (I-squared = 0.0%, p = 0.434)			0.88 (0.39, 1.99)	14/34817	14/29599	100.00

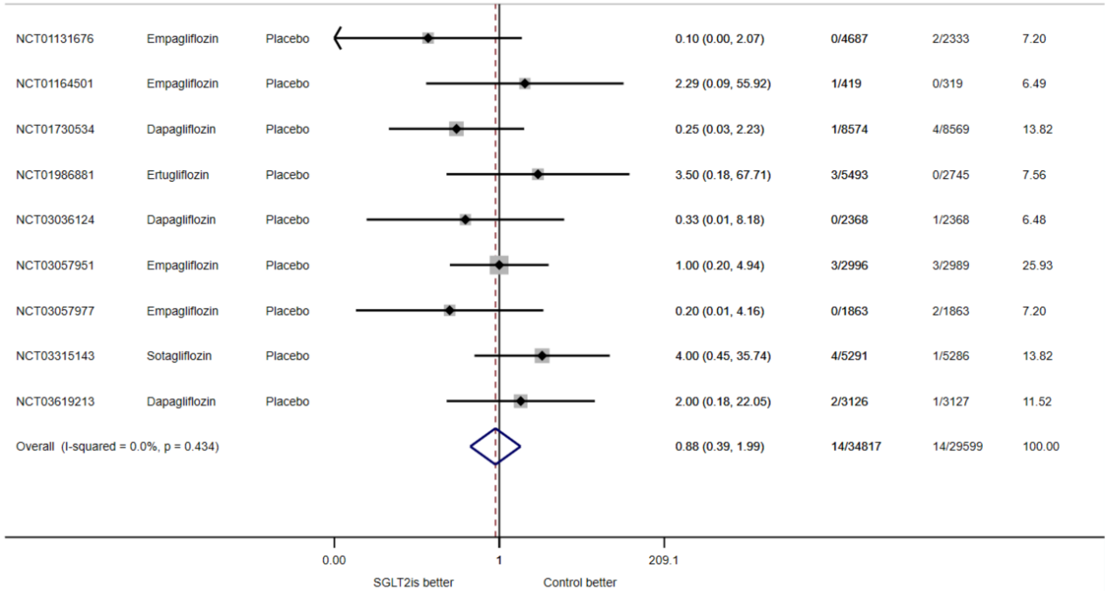

System 16-Neoplasms Benign, Malignant And Unspecified-Figure S91 Meta-analysis of SGLT2is and Oesophageal carcinoma

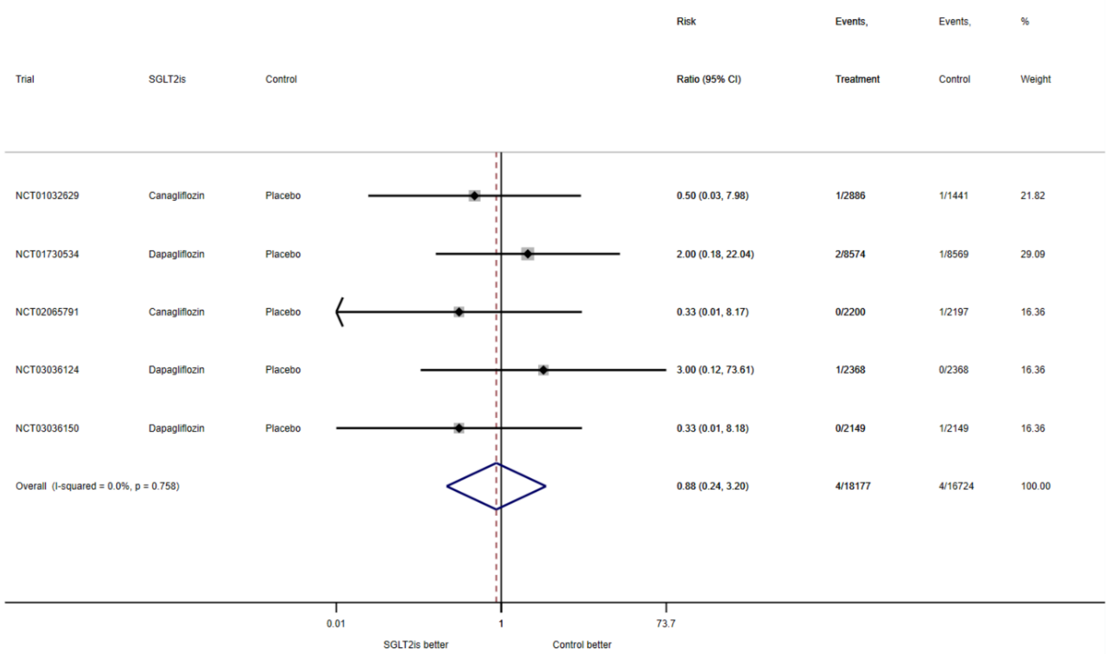

System 16-Neoplasms Benign, Malignant And Unspecified-Figure S92 Meta-analysis of SGLT2is and Ovarian cancer

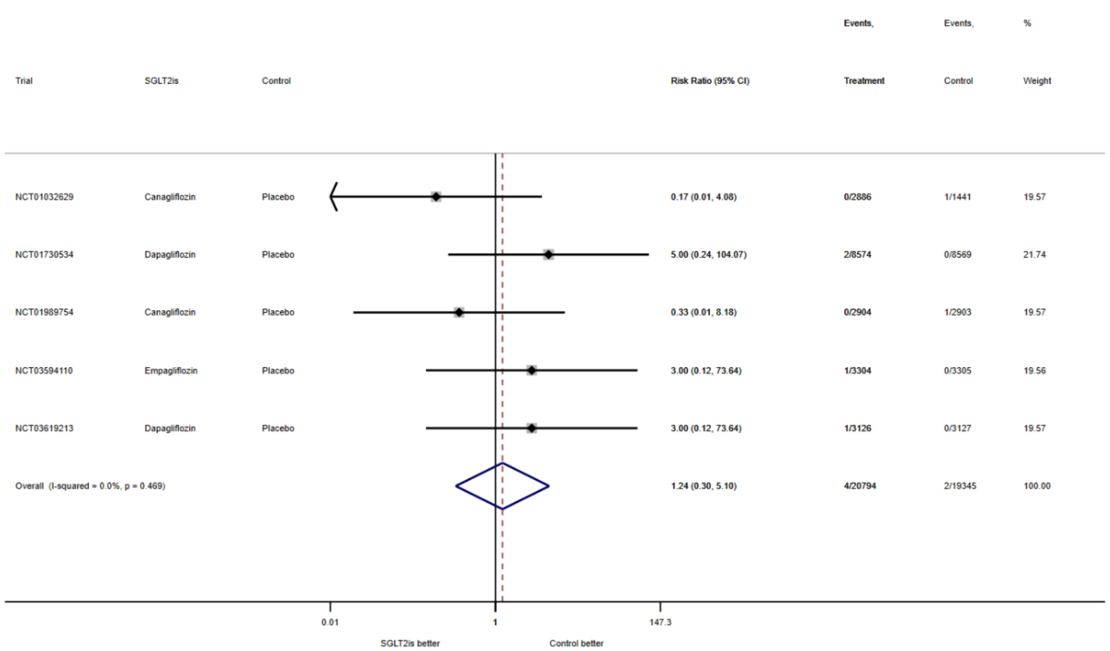

System 16-Neoplasms Benign, Malignant And Unspecified-Figure S93 Meta-analysis of SGLT2is and Ovarian cancer metastatic

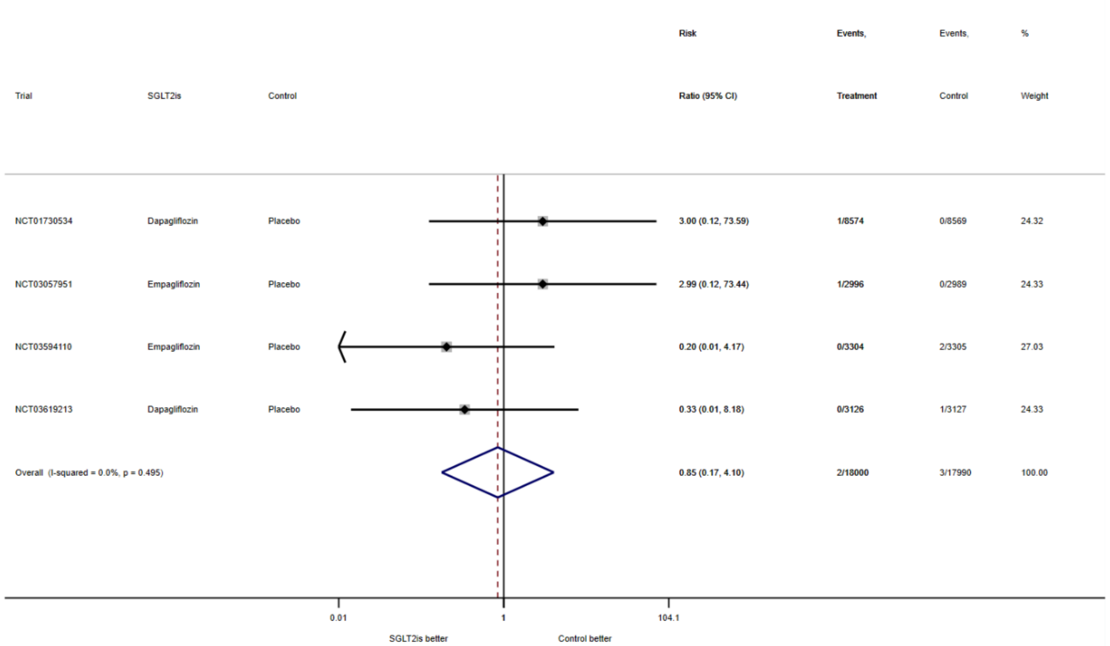

System 16-Neoplasms Benign, Malignant And Unspecified-Figure S94 Meta-analysis of SGLT2is and Ovarian neoplasm

Trial	SGLT2is	Control	Risk Ratio (95% CI)	Events, Treatment	Events, Control	% Weight
NCT00673231	Dapagliflozin	Placebo	0.97 (0.04, 23.77)	1/610	0/197	2.21
NCT01032629	Canagliflozin	Placebo	0.17 (0.02, 1.60)	1/2886	3/1441	4.42
NCT01131676	Empagliflozin	Placebo	4.48 (0.24, 83.19)	4/4687	0/2333	2.65
NCT01164501	Empagliflozin	Placebo	0.25 (0.01, 6.21)	0/419	1/319	2.21
NCT01730534	Dapagliflozin	Placebo	1.12 (0.43, 2.91)	9/8574	8/8569	24.97
NCT01986881	Ertugliflozin	Placebo	0.75 (0.21, 2.65)	6/5493	4/2745	14.15
NCT01989754	Canagliflozin	Placebo	0.17 (0.02, 1.38)	1/2904	6/2903	5.05
NCT02065791	Canagliflozin	Placebo	1.00 (0.06, 15.96)	1/2200	1/2197	2.95
NCT03036124	Dapagliflozin	Placebo	0.14 (0.01, 2.76)	0/2368	3/2368	2.58
NCT03036150	Dapagliflozin	Placebo	1.00 (0.06, 15.98)	1/2149	1/2149	2.95
NCT03057951	Empagliflozin	Placebo	2.00 (0.18, 21.99)	2/2996	1/2989	3.93
NCT03057977	Empagliflozin	Placebo	0.14 (0.01, 2.76)	0/1863	3/1863	2.58
NCT03315143	Sotagliflozin	Placebo	0.80 (0.21, 2.97)	4/5291	5/5286	13.10
NCT03521934	Sotagliflozin	Placebo	0.34 (0.01, 8.25)	0/605	1/611	2.21
NCT03594110	Empagliflozin	Placebo	0.50 (0.05, 5.51)	1/3304	2/3305	3.93
NCT03619213	Dapagliflozin	Placebo	0.75 (0.17, 3.35)	3/3126	4/3127	10.11
Overall (I-squared = 0.0%, p = 0.841)			0.70 (0.44, 1.13)	34/49475	43/42402	100.00

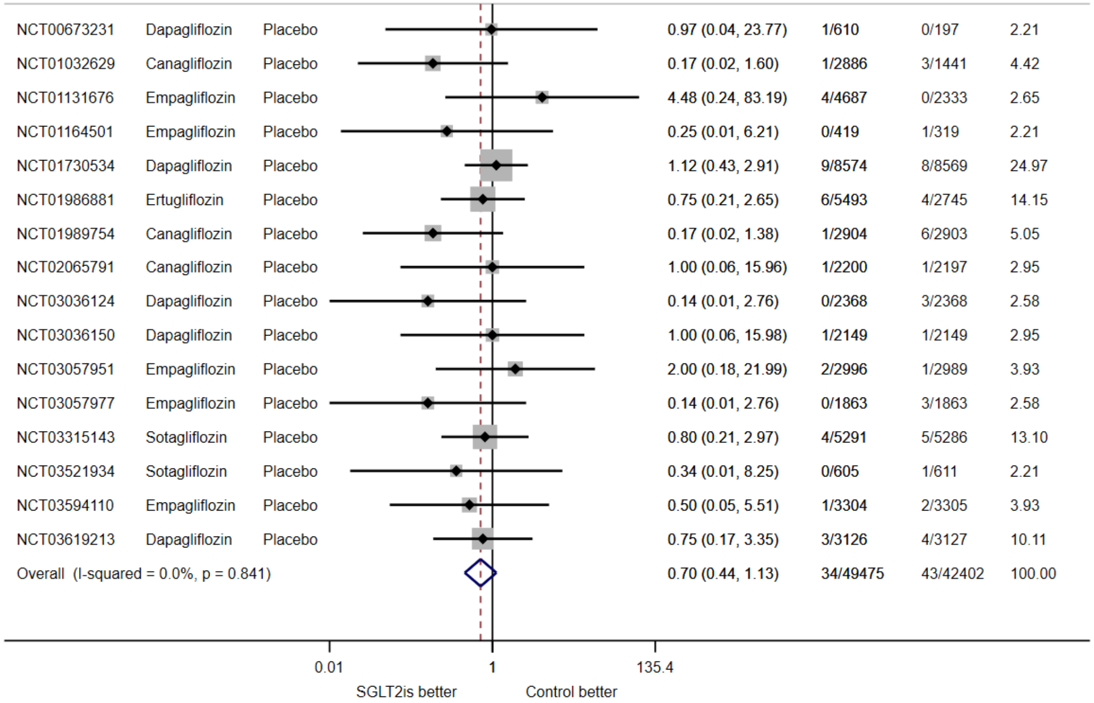

System 16-Neoplasms Benign, Malignant And Unspecified-Figure S95 Meta-analysis of SGLT2is and Pancreatic carcinoma

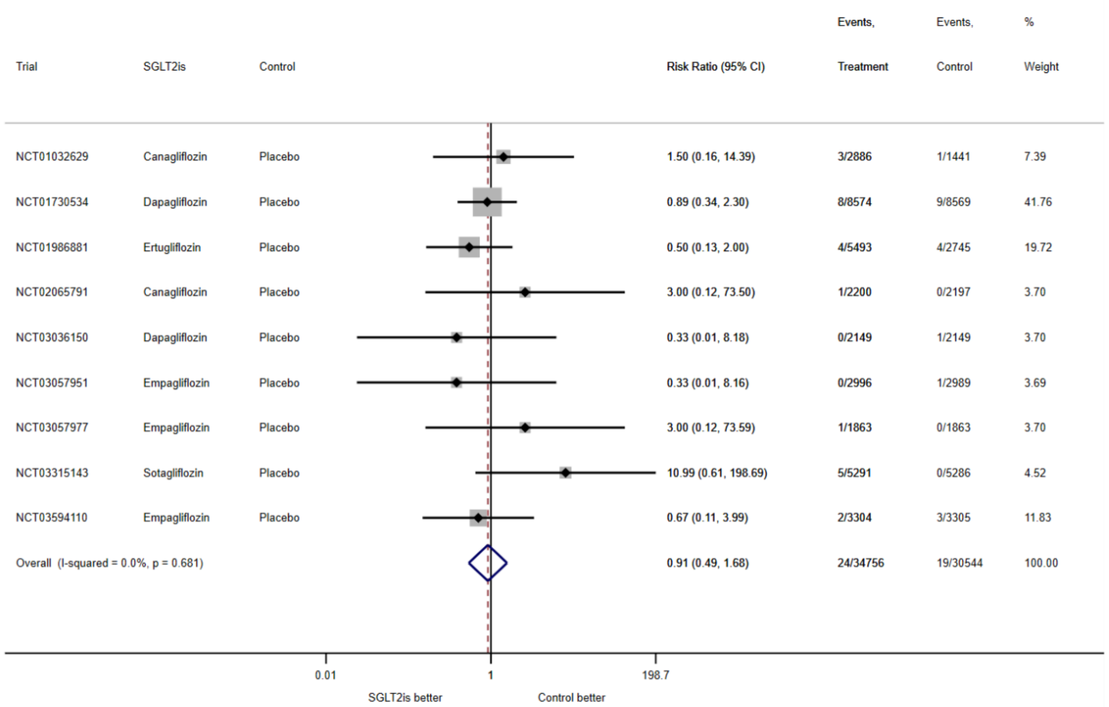

System 16-Neoplasms Benign, Malignant And Unspecified-Figure S96 Meta-analysis of SGLT2is and Pancreatic carcinoma metastatic

Trial	SGLT2is	Control	Risk Ratio (95% CI)	Events, Treatment	Events, Control	% Weight
NCT00673231	Dapagliflozin	Placebo	0.97 (0.04, 23.77)	1/610	0/197	10.16
NCT01032629	Canagliflozin	Placebo	1.50 (0.06, 36.76)	1/2886	0/1441	10.13
NCT01730534	Dapagliflozin	Placebo	8.99 (0.48, 167.04)	4/8574	0/8569	12.16
NCT02065791	Canagliflozin	Placebo	3.00 (0.12, 73.50)	1/2200	0/2197	10.13
NCT03036124	Dapagliflozin	Placebo	1.00 (0.06, 15.98)	1/2368	1/2368	13.51
NCT03036150	Dapagliflozin	Placebo	3.00 (0.12, 73.60)	1/2149	0/2149	10.13
NCT03057951	Empagliflozin	Placebo	2.99 (0.12, 73.44)	1/2996	0/2989	10.13
NCT03315143	Sotagliflozin	Placebo	3.00 (0.12, 73.56)	1/5291	0/5286	10.13
NCT03619213	Dapagliflozin	Placebo	1.00 (0.06, 15.99)	1/3126	1/3127	13.51
Overall (I-squared = 0.0%, p = 0.982)			2.12 (0.76, 5.86)	12/30200	2/28323	100.00

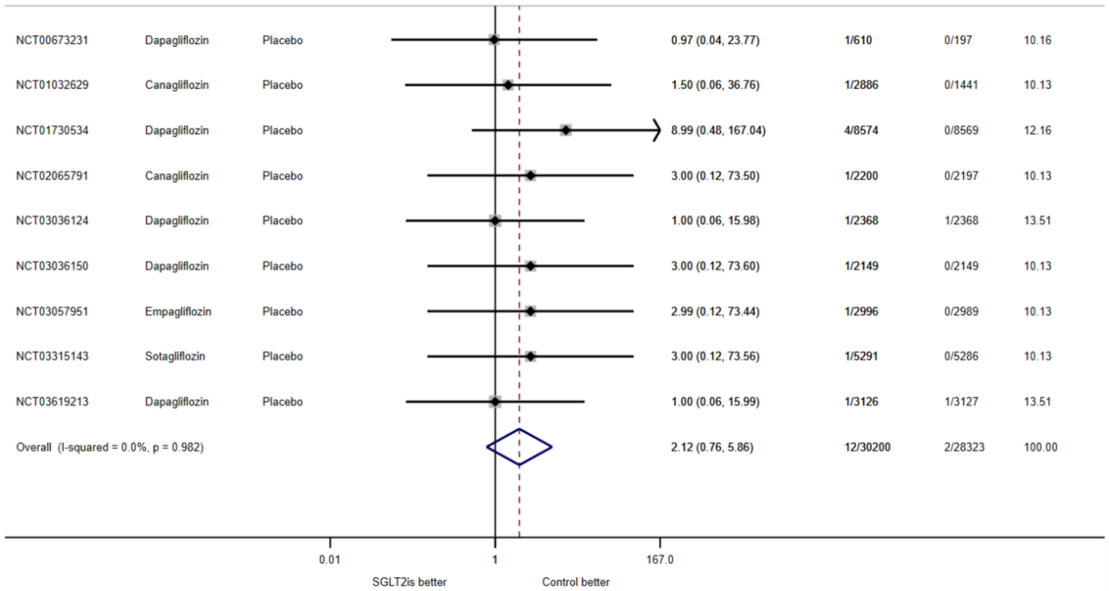

System 16-Neoplasms Benign, Malignant And Unspecified-Figure S97 Meta-analysis of SGLT2is and Pancreatic neoplasm

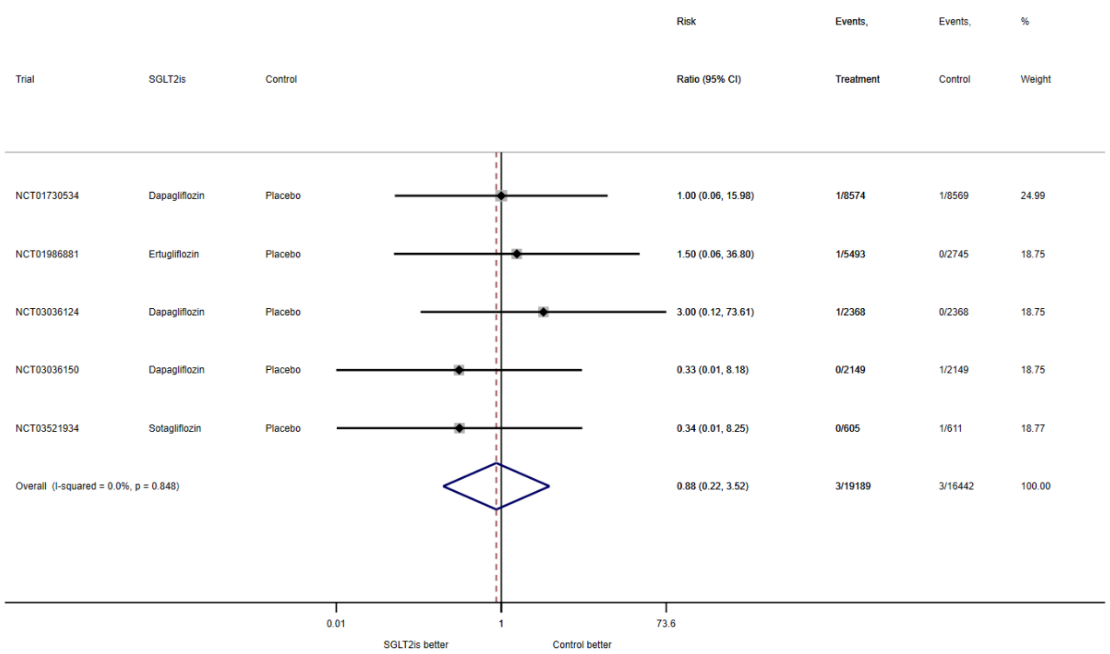

System 16-Neoplasms Benign, Malignant And Unspecified-Figure S98 Meta-analysis of SGLT2is and Papillary renal cell carcinoma

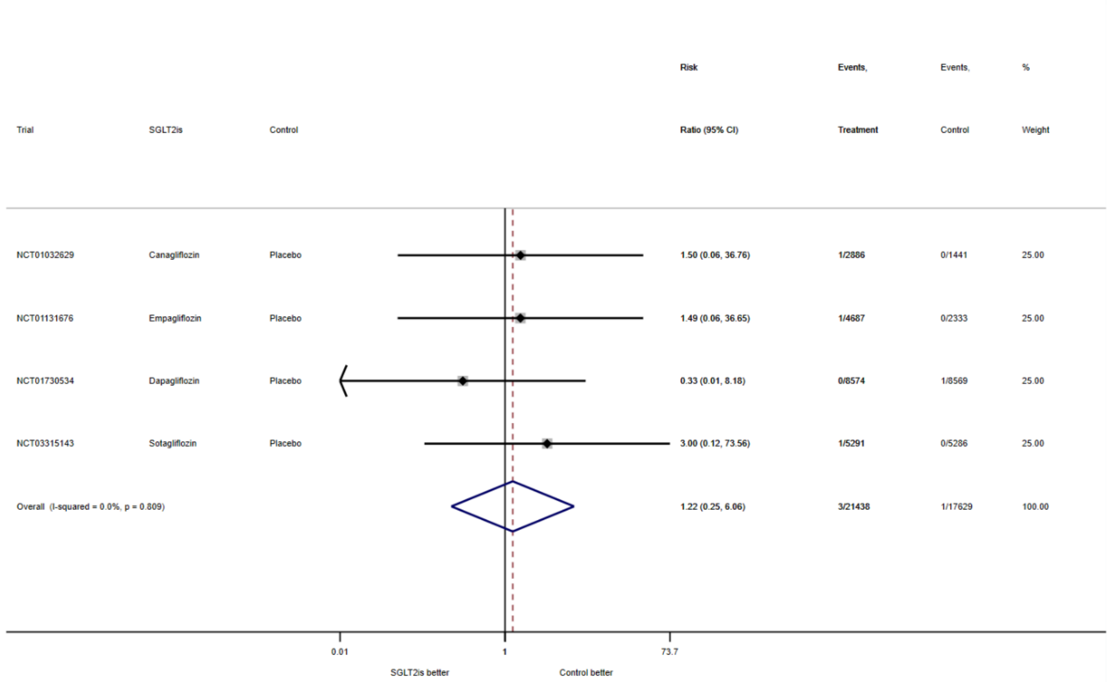

System 16-Neoplasms Benign, Malignant And Unspecified-Figure S99 Meta-analysis of SGLT2is and Papillary serous endometrial carcinoma

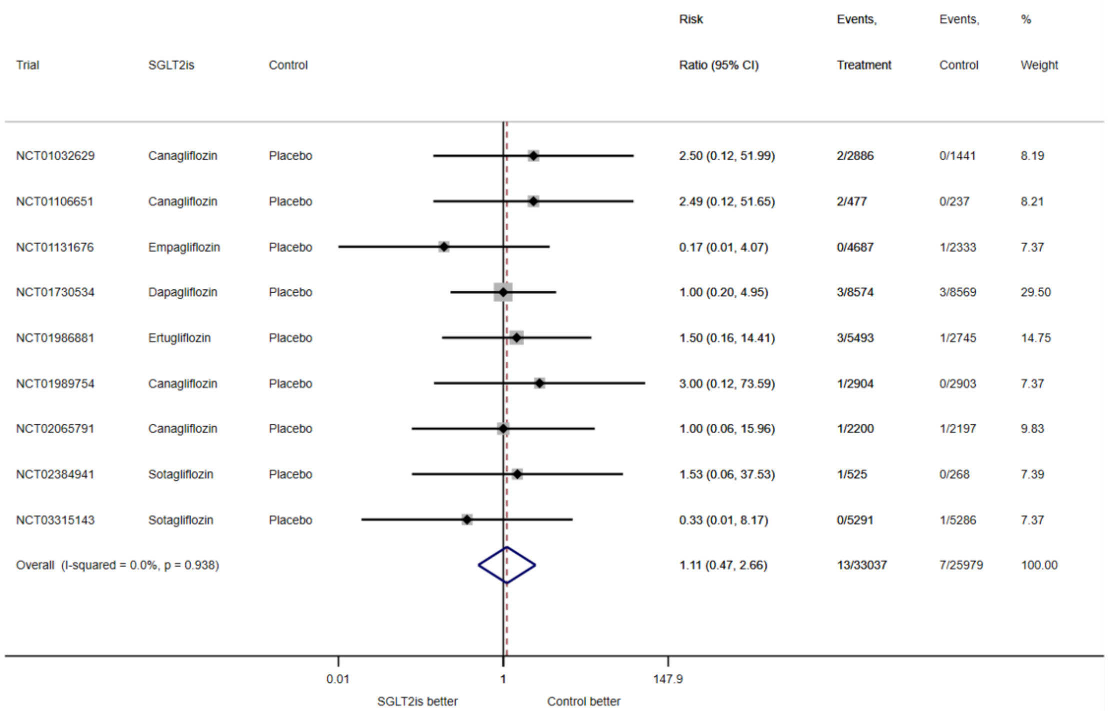

System 16-Neoplasms Benign, Malignant And Unspecified-Figure S100 Meta-analysis of SGLT2is and Papillary thyroid cancer

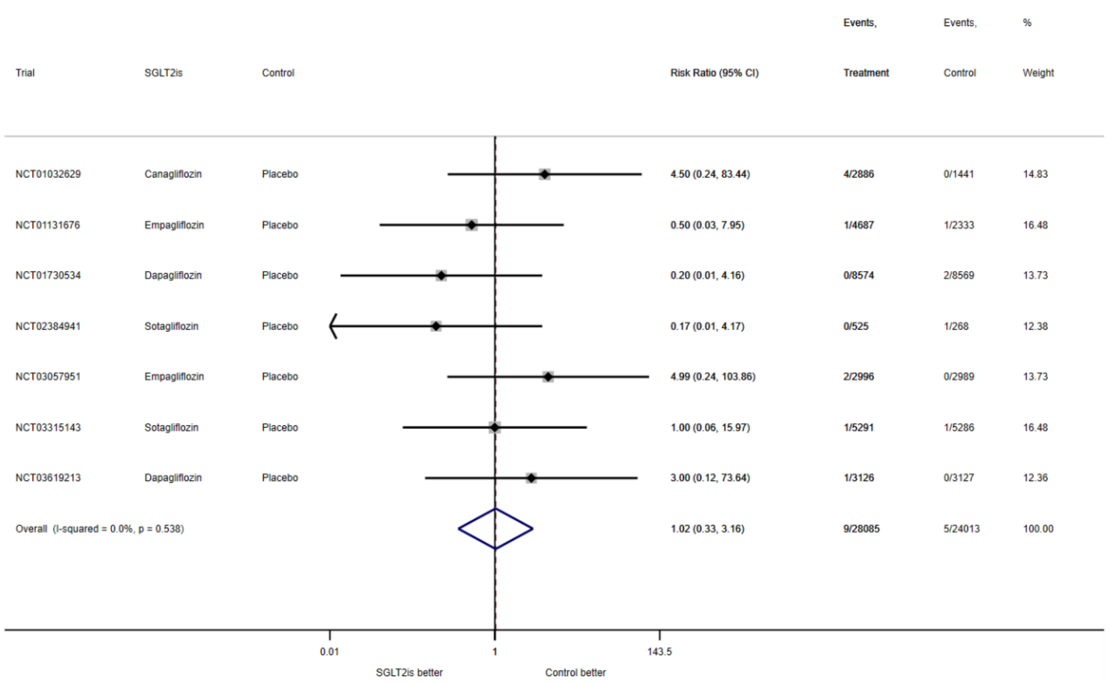

System 16-Neoplasms Benign, Malignant And Unspecified-Figure S101 Meta-analysis of SGLT2is and Parathyroid tumour benign

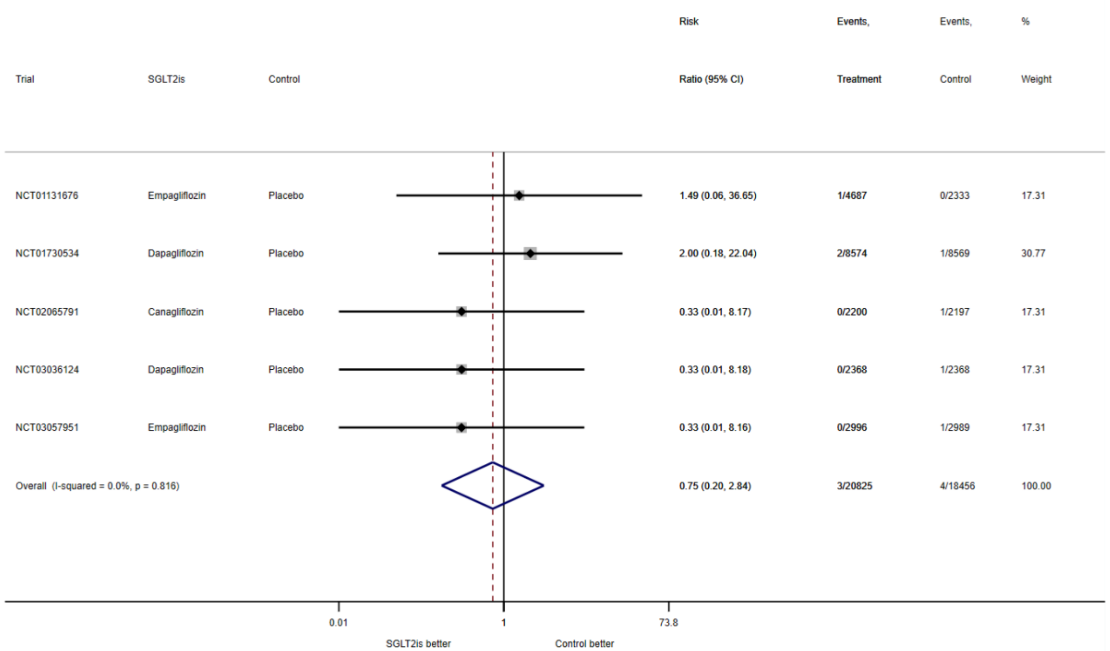

System 16-Neoplasms Benign, Malignant And Unspecified-Figure S102 Meta-analysis of SGLT2is and Pituitary tumour benign

Trial	SGLT2is	Control	Risk Ratio (95% CI)	Events, Treatment	Events, Control	% Weight
NCT01131676	Empagliflozin	Placebo	1.49 (0.16, 14.35)	3/4687	1/2333	9.94
NCT01730534	Dapagliflozin	Placebo	0.33 (0.07, 1.65)	2/8574	6/8569	19.88
NCT01986881	Ertugliflozin	Placebo	0.17 (0.01, 4.09)	0/5493	1/2745	4.97
NCT01989754	Canagliflozin	Placebo	0.33 (0.01, 8.18)	0/2904	1/2903	4.97
NCT02033889	Ertugliflozin	Placebo/Glimepiride	1.53 (0.06, 37.28)	1/412	0/209	4.98
NCT02065791	Canagliflozin	Placebo	0.33 (0.01, 8.17)	0/2200	1/2197	4.97
NCT03036124	Dapagliflozin	Placebo	3.00 (0.12, 73.61)	1/2368	0/2368	4.97
NCT03036150	Dapagliflozin	Placebo	1.00 (0.06, 15.98)	1/2149	1/2149	6.63
NCT03057951	Empagliflozin	Placebo	1.00 (0.06, 15.94)	1/2996	1/2989	6.63
NCT03057977	Empagliflozin	Placebo	0.33 (0.01, 8.18)	0/1863	1/1863	4.97
NCT03242252	Sotagliflozin	Placebo	0.49 (0.03, 7.86)	1/527	1/260	6.64
NCT03315143	Sotagliflozin	Placebo	3.00 (0.12, 73.56)	1/5291	0/5286	4.97
NCT03594110	Empagliflozin	Placebo	5.00 (0.24, 104.14)	2/3304	0/3305	5.52
NCT03619213	Dapagliflozin	Placebo	0.33 (0.03, 3.20)	1/3126	3/3127	9.94
Overall (I-squared = 0.0%, p = 0.918)			0.69 (0.34, 1.41)	14/45894	17/40303	100.00

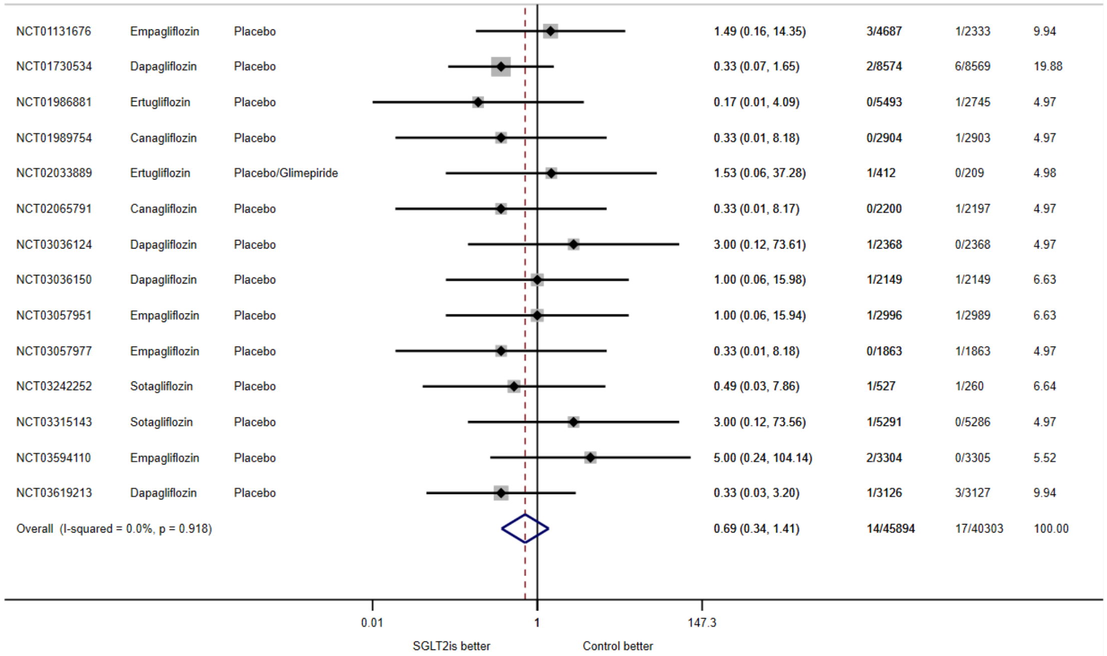

System 16-Neoplasms Benign, Malignant And Unspecified-Figure S103 Meta-analysis of SGLT2is and Plasma cell myeloma

Trial	SGLT2is	Control	Risk Ratio (95% CI)	Events, Treatment	Events, Control	% Weight
NCT01032629	Canagliflozin	Placebo	1.00 (0.40, 2.47)	14/2886	7/1441	5.71
NCT01042977	Dapagliflozin	Placebo	0.33 (0.01, 8.18)	0/482	1/483	0.46
NCT01106651	Canagliflozin	Placebo	2.49 (0.12, 51.65)	2/477	0/237	0.51
NCT01106677	Canagliflozin	Sitagliptin	1.50 (0.06, 36.63)	1/735	0/366	0.46
NCT01131676	Empagliflozin	Placebo	1.11 (0.50, 2.43)	20/4687	9/2333	7.58
NCT01730534	Dapagliflozin	Placebo	1.25 (0.88, 1.78)	69/8574	55/8569	37.51
NCT01986881	Ertugliflozin	Placebo	1.18 (0.58, 2.39)	26/5493	11/2745	9.45
NCT01989754	Canagliflozin	Placebo	0.06 (0.00, 1.02)	0/2904	8/2903	0.57
NCT02033889	Ertugliflozin	Placebo/Glimepiride	1.53 (0.06, 37.28)	1/412	0/209	0.46
NCT02065791	Canagliflozin	Placebo	2.00 (0.60, 6.62)	8/2200	4/2197	3.25
NCT03036124	Dapagliflozin	Placebo	6.00 (0.72, 49.80)	6/2368	1/2368	1.04
NCT03036150	Dapagliflozin	Placebo	1.67 (0.61, 4.58)	10/2149	6/2149	4.58
NCT03057951	Empagliflozin	Placebo	1.00 (0.43, 2.30)	11/2996	11/2989	6.72
NCT03057977	Empagliflozin	Placebo	0.86 (0.29, 2.55)	6/1863	7/1863	3.95
NCT03242252	Sotagliflozin	Placebo	1.48 (0.06, 36.28)	1/527	0/260	0.46
NCT03315143	Sotagliflozin	Placebo	0.77 (0.34, 1.75)	10/5291	13/5286	6.89
NCT03521934	Sotagliflozin	Placebo	5.05 (0.24, 104.96)	2/605	0/611	0.51
NCT03594110	Empagliflozin	Placebo	0.80 (0.32, 2.03)	8/3304	10/3305	5.42
NCT03619213	Dapagliflozin	Placebo	0.71 (0.23, 2.25)	5/3126	7/3127	3.56
NCT04157751	Empagliflozin	Placebo	0.34 (0.01, 8.27)	0/260	1/264	0.46
NCT04350593	Dapagliflozin	Placebo	0.33 (0.01, 8.21)	0/613	1/616	0.46
Overall (I-squared = 0.0%, p = 0.849)			1.12 (0.90, 1.39)	200/51952	152/44321	100.00

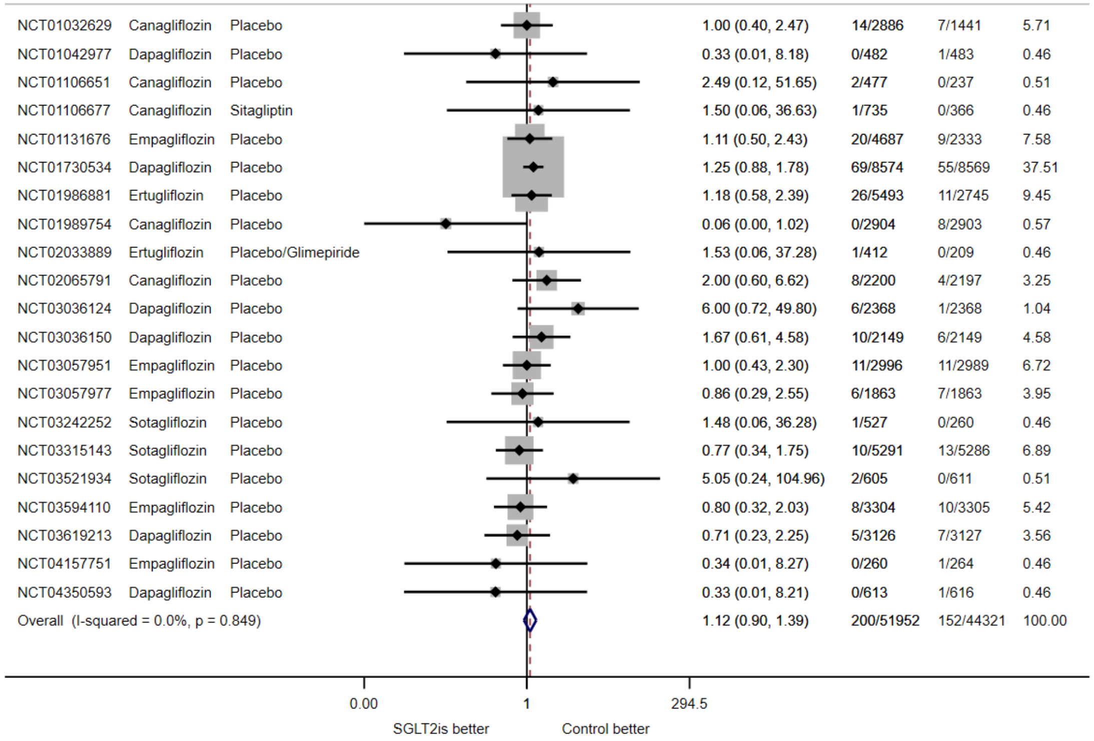

System 16-Neoplasms Benign, Malignant And Unspecified-Figure S104 Meta-analysis of SGLT2is and Prostate cancer

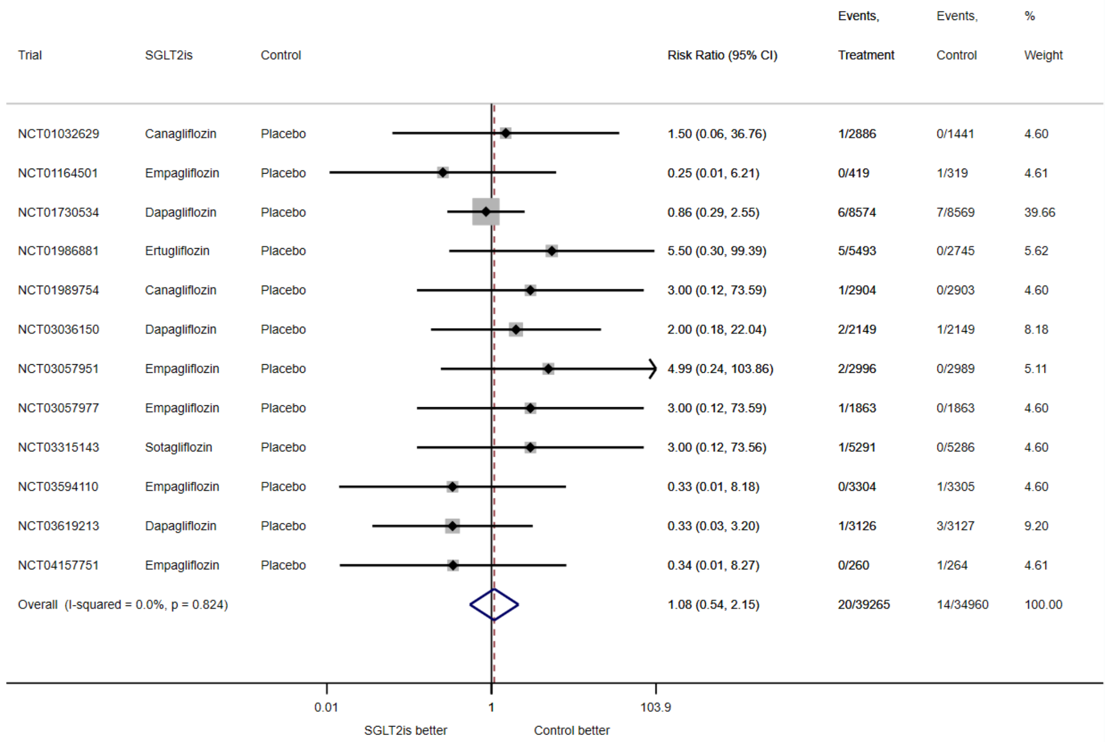

System 16-Neoplasms Benign, Malignant And Unspecified-Figure S105 Meta-analysis of SGLT2is and Prostate cancer metastatic

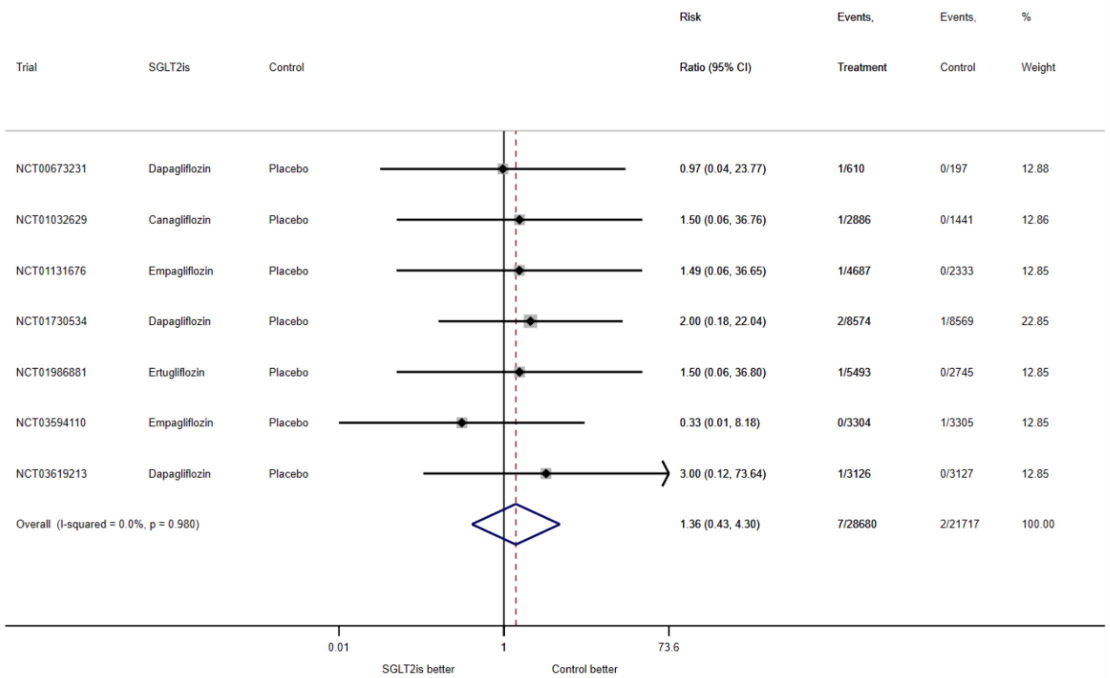

System 16-Neoplasms Benign, Malignant And Unspecified-Figure S106 Meta-analysis of SGLT2is and Prostate cancer recurrent

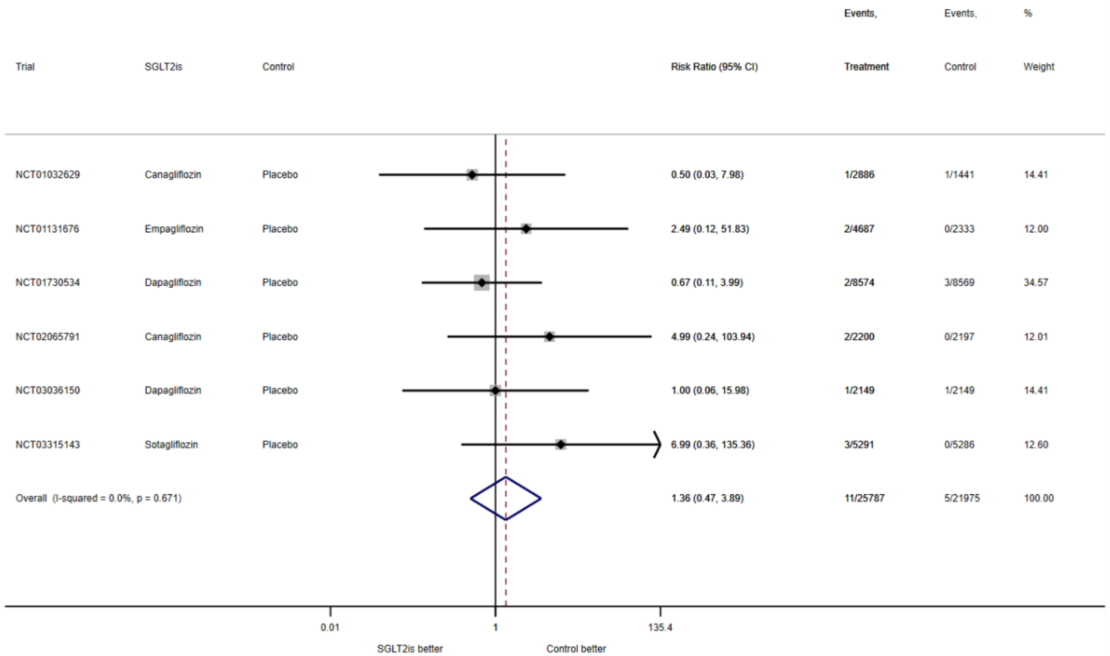

System 16-Neoplasms Benign, Malignant And Unspecified-Figure S107 Meta-analysis of SGLT2is and Prostatic adenoma

Trial	SGLT2is	Control	Risk Ratio (95% CI)	Events, Treatment	Events, Control	% Weight
NCT01032629	Canagliflozin	Placebo	2.50 (0.29, 21.35)	5/2886	1/1441	11.40
NCT01131676	Empagliflozin	Placebo	7.47 (0.43, 130.70)	7/4687	0/2333	6.41
NCT01730534	Dapagliflozin	Placebo	0.25 (0.03, 2.23)	1/8574	4/8569	10.94
NCT01986881	Ertugliflozin	Placebo	0.37 (0.08, 1.67)	3/5493	4/2745	23.46
NCT02065791	Canagliflozin	Placebo	1.00 (0.06, 15.96)	1/2200	1/2197	6.84
NCT03036150	Dapagliflozin	Placebo	3.00 (0.12, 73.60)	1/2149	0/2149	5.13
NCT03057951	Empagliflozin	Placebo	2.00 (0.18, 21.99)	2/2996	1/2989	9.12
NCT03057977	Empagliflozin	Placebo	3.00 (0.12, 73.59)	1/1863	0/1863	5.13
NCT03242252	Sotagliflozin	Placebo	1.48 (0.06, 36.28)	1/527	0/260	5.14
NCT03619213	Dapagliflozin	Placebo	1.50 (0.25, 8.97)	3/3126	2/3127	16.42
Overall (I-squared = 0.0%, p = 0.619)			1.12 (0.54, 2.31)	25/34501	13/27673	100.00

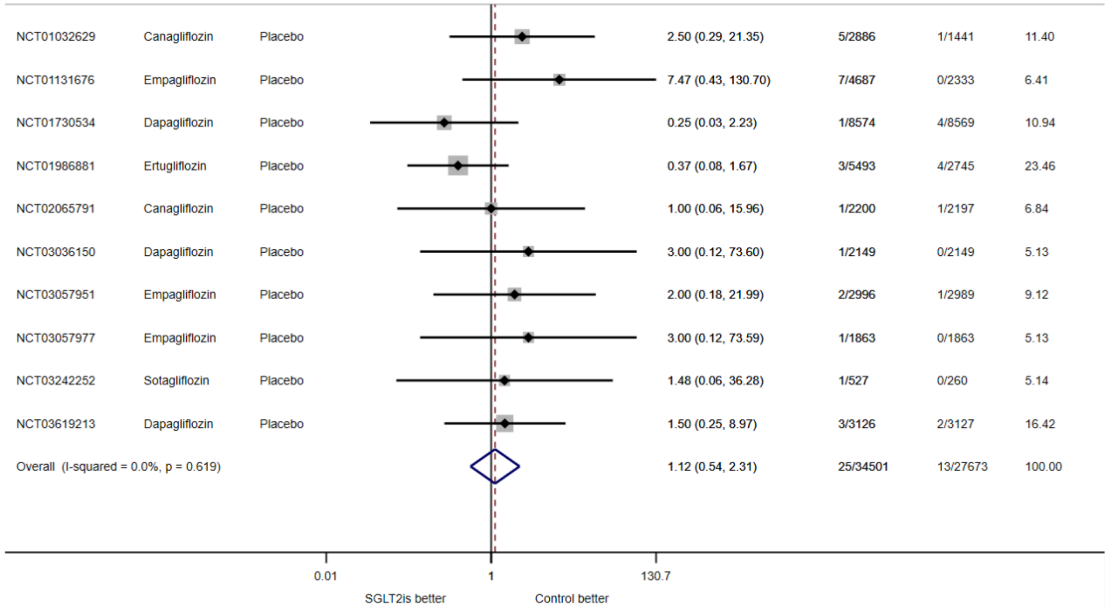

System 16-Neoplasms Benign, Malignant And Unspecified-Figure S108 Meta-analysis of SGLT2is and Rectal adenocarcinoma

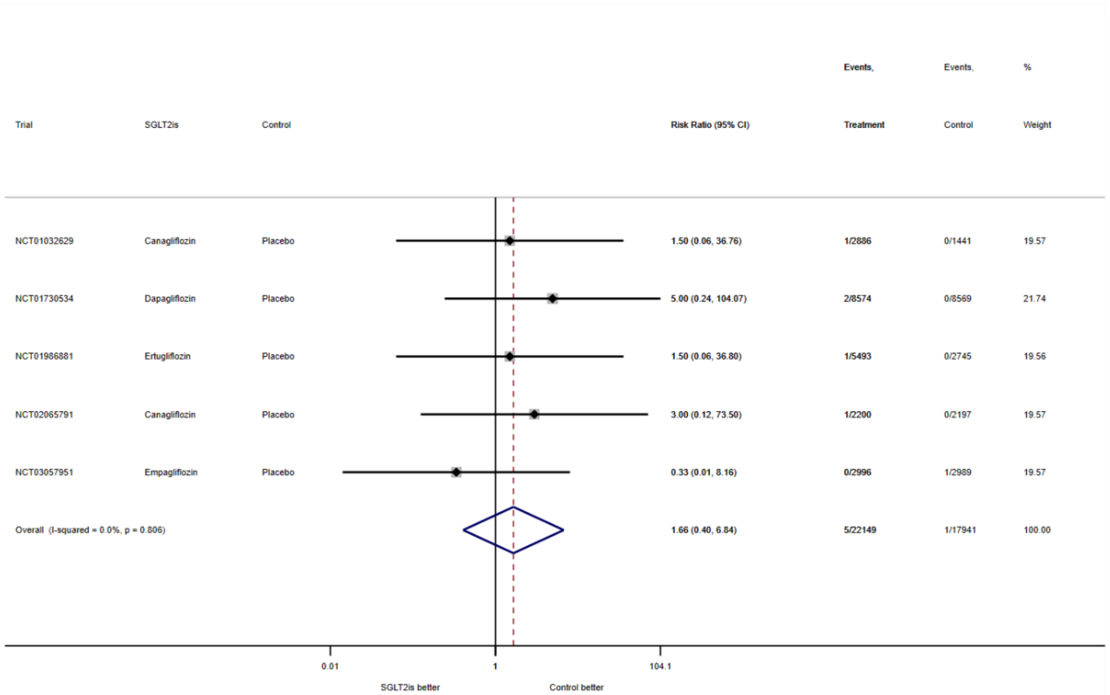

System 16-Neoplasms Benign, Malignant And Unspecified-Figure S109 Meta-analysis of SGLT2is and Rectal adenoma

Trial	SGLT2is	Control	Risk Ratio (95% CI)	Events, Treatment	Events, Control	% Weight
NCT01032629	Canagliflozin	Placebo	1.50 (0.16, 14.39)	3/2886	1/1441	11.43
NCT01131676	Empagliflozin	Placebo	3.49 (0.18, 67.44)	3/4687	0/2333	6.67
NCT01730534	Dapagliflozin	Placebo	1.00 (0.25, 3.99)	4/8574	4/8569	30.48
NCT01986881	Ertugliflozin	Placebo	3.50 (0.18, 67.71)	3/5493	0/2745	6.67
NCT01989754	Canagliflozin	Placebo	0.14 (0.01, 2.76)	0/2904	3/2903	6.67
NCT02065791	Canagliflozin	Placebo	1.00 (0.06, 15.96)	1/2200	1/2197	7.62
NCT03036124	Dapagliflozin	Placebo	0.33 (0.01, 8.18)	0/2368	1/2368	5.71
NCT03036150	Dapagliflozin	Placebo	0.33 (0.01, 8.18)	0/2149	1/2149	5.71
NCT03057951	Empagliflozin	Placebo	2.99 (0.12, 73.44)	1/2996	0/2989	5.71
NCT03594110	Empagliflozin	Placebo	3.00 (0.12, 73.64)	1/3304	0/3305	5.71
NCT03619213	Dapagliflozin	Placebo	1.00 (0.06, 15.99)	1/3126	1/3127	7.62
Overall (I-squared = 0.0%, p = 0.897)			1.09 (0.51, 2.33)	17/40687	12/34126	100.00

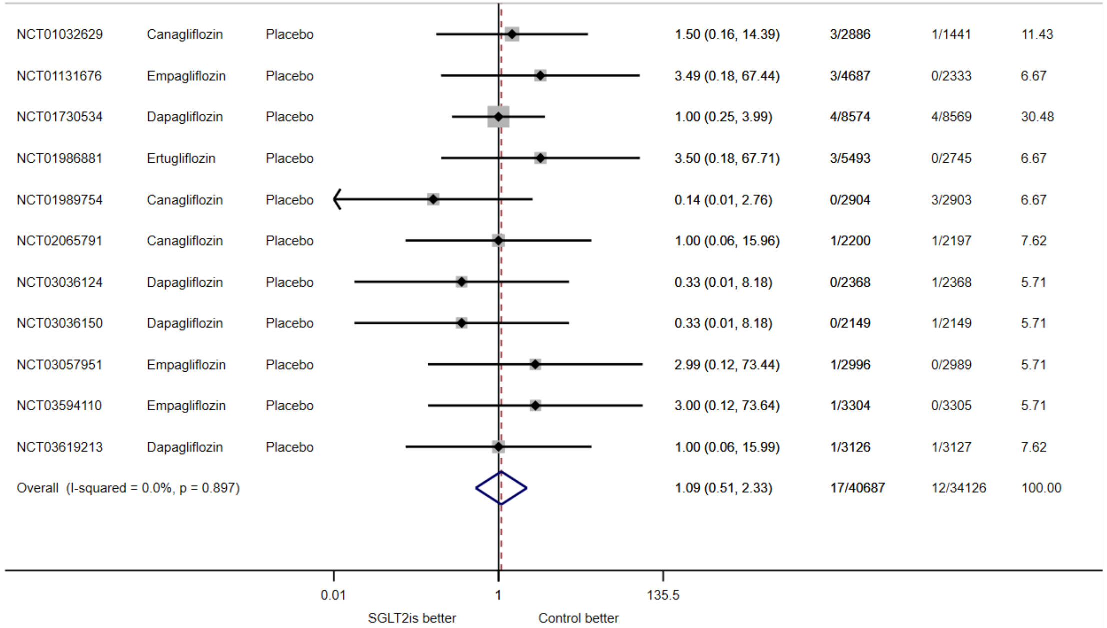

System 16-Neoplasms Benign, Malignant And Unspecified-Figure S110 Meta-analysis of SGLT2is and Rectal cancer

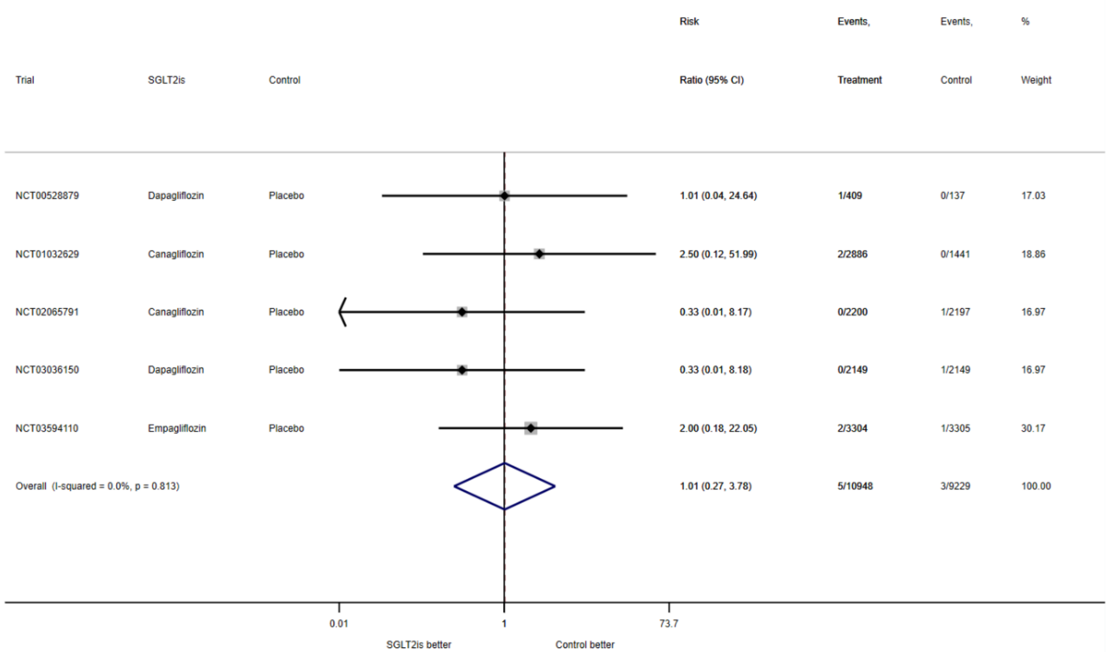

System 16-Neoplasms Benign, Malignant And Unspecified-Figure S111 Meta-analysis of SGLT2is and Rectosigmoid cancer

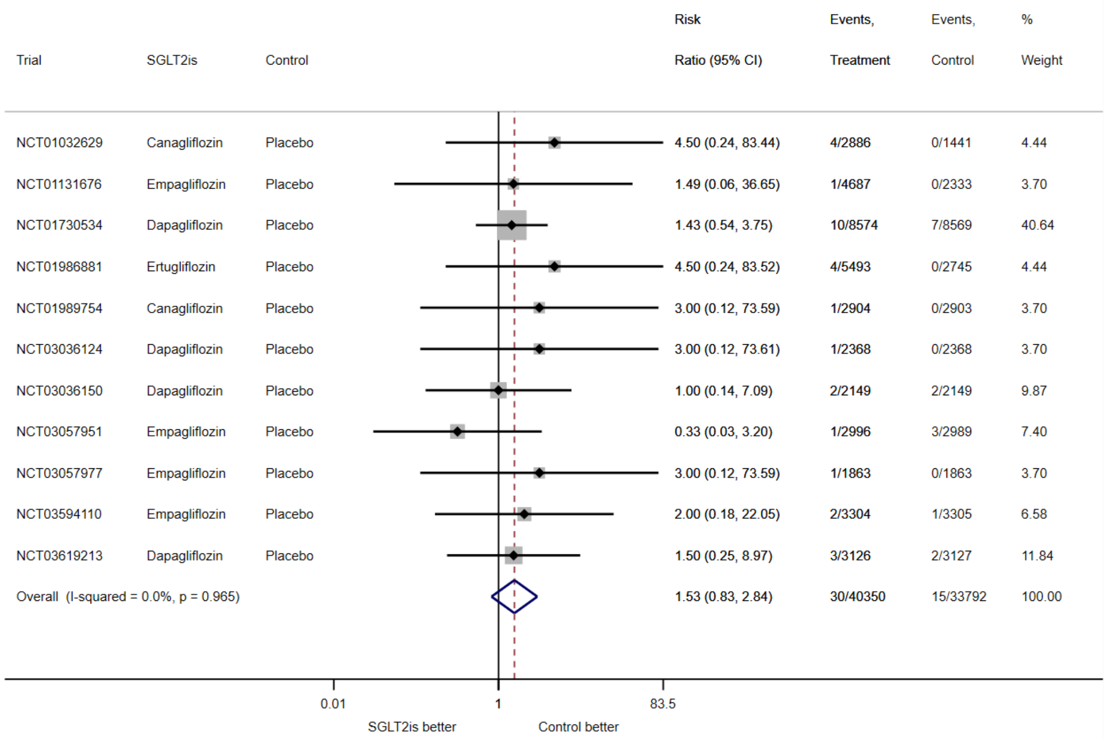

System 16-Neoplasms Benign, Malignant And Unspecified-Figure S112 Meta-analysis of SGLT2is and Renal cancer

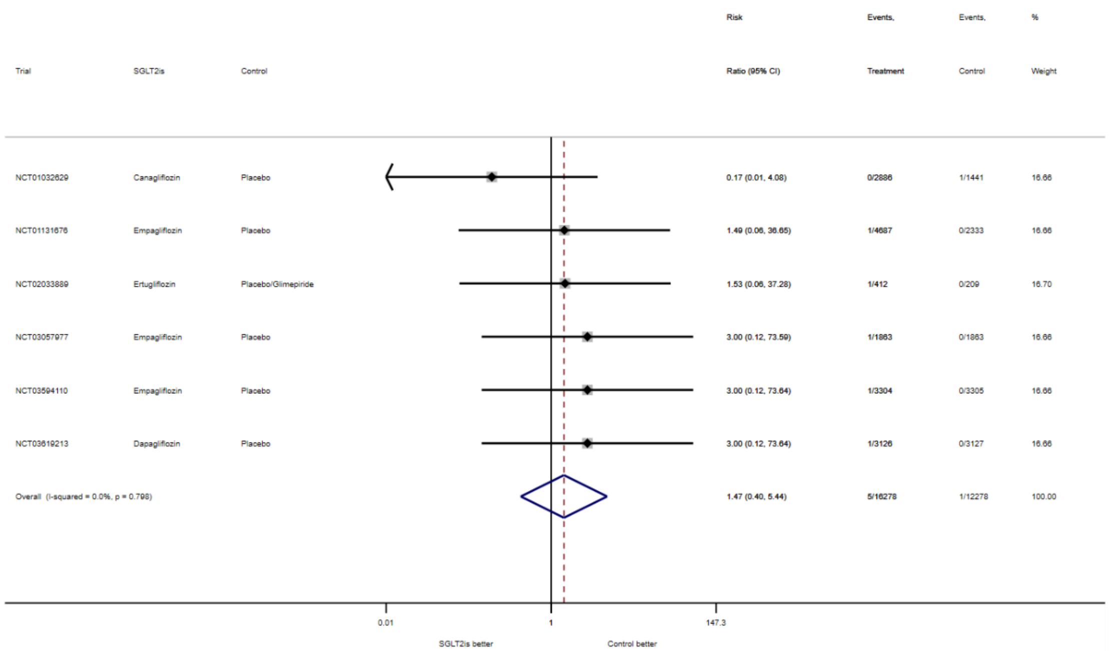

System 16-Neoplasms Benign, Malignant And Unspecified-Figure S113 Meta-analysis of SGLT2is and Renal cancer metastatic

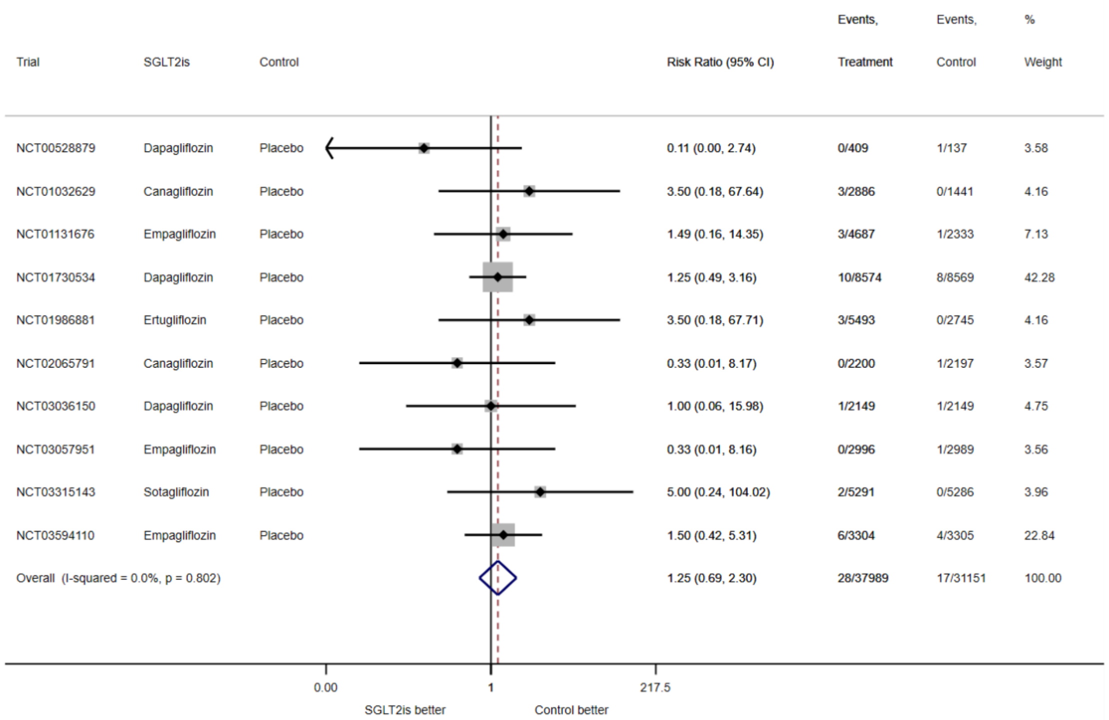

System 16-Neoplasms Benign, Malignant And Unspecified-Figure S114 Meta-analysis of SGLT2is and Renal cell carcinoma

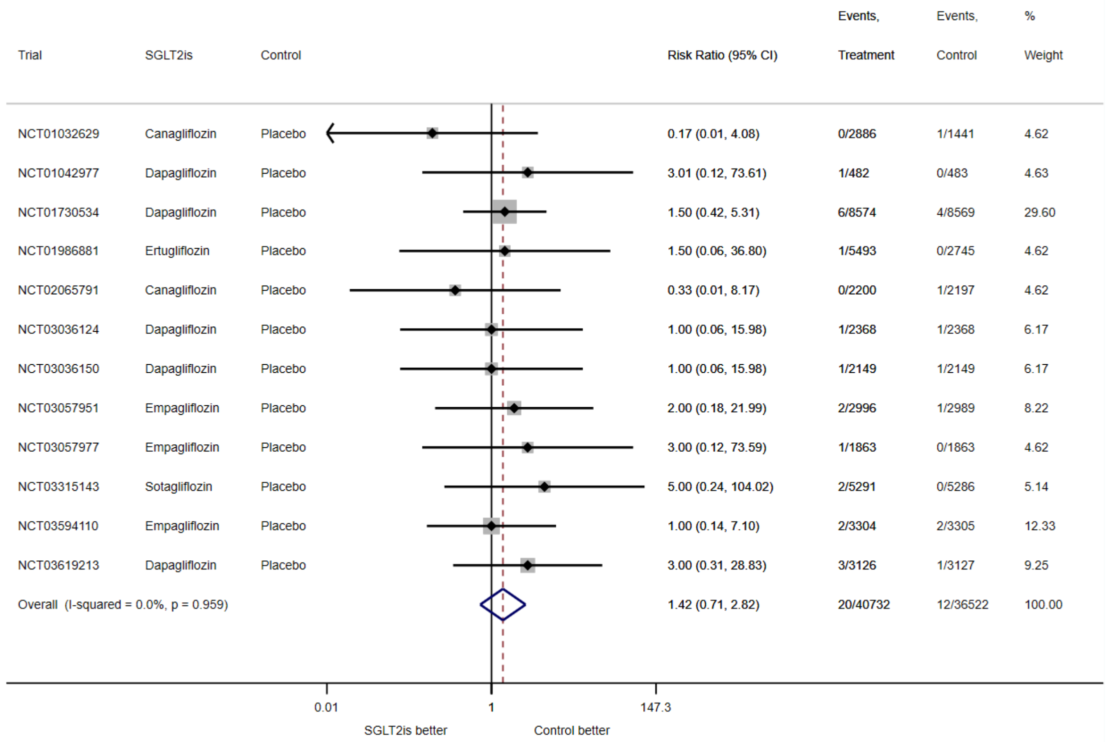

System 16-Neoplasms Benign, Malignant And Unspecified-Figure S115 Meta-analysis of SGLT2is and Renal neoplasm

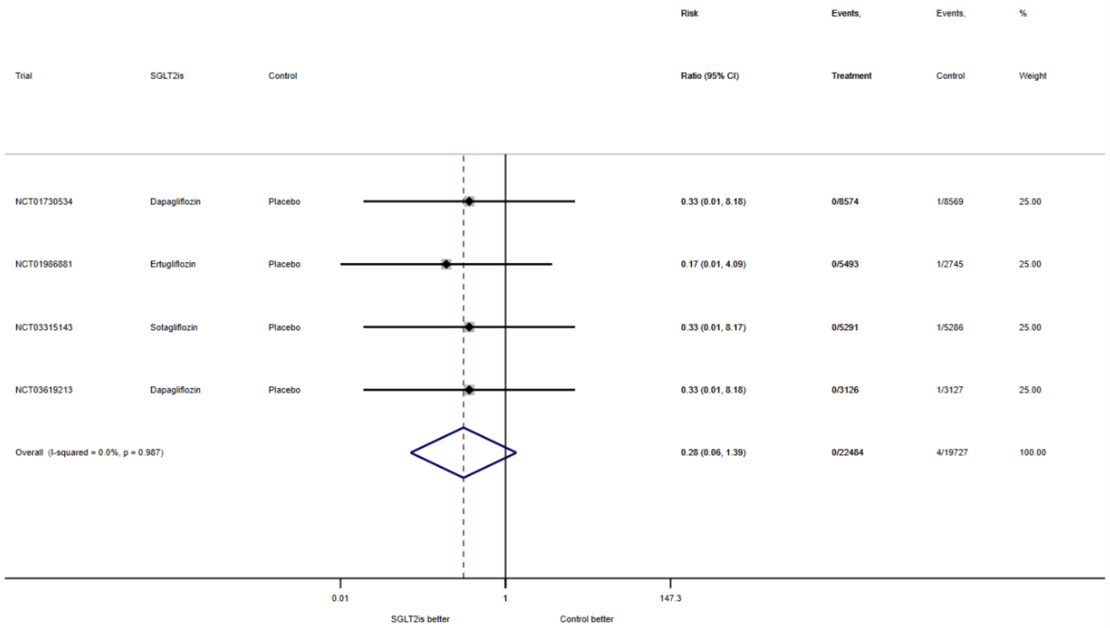

System 16-Neoplasms Benign, Malignant And Unspecified-Figure S116 Meta-analysis of SGLT2is and Seborrhoeic keratosis

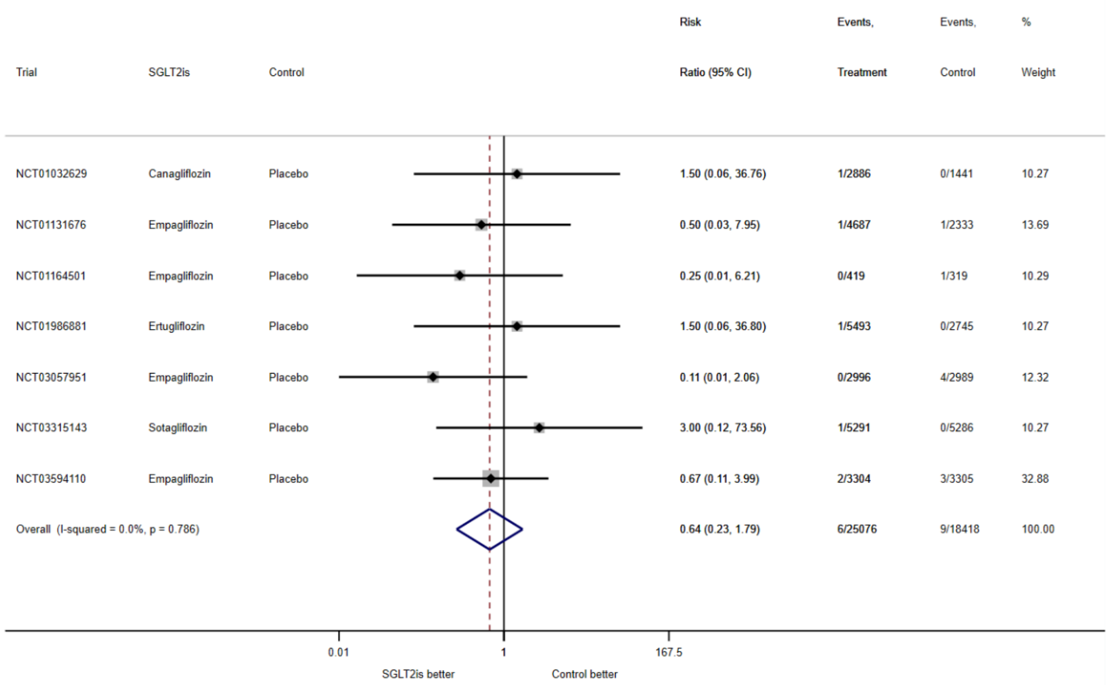

System 16-Neoplasms Benign, Malignant And Unspecified-Figure S117 Meta-analysis of SGLT2is and Skin cancer

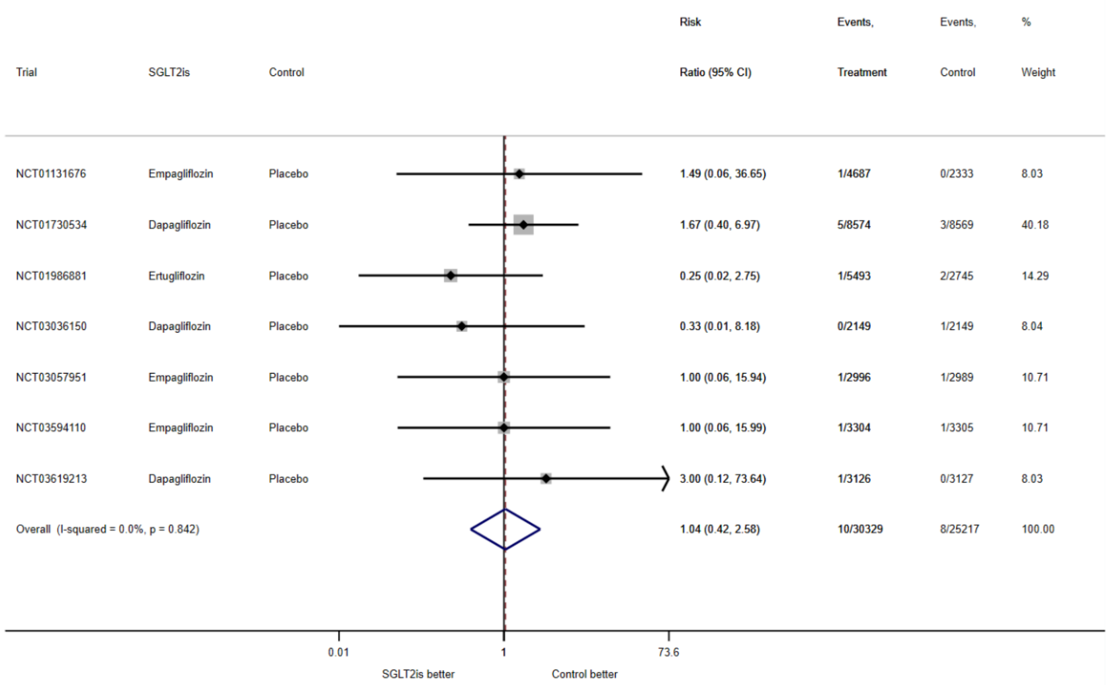

System 16-Neoplasms Benign, Malignant And Unspecified-Figure S118 Meta-analysis of SGLT2is and Small cell lung cancer

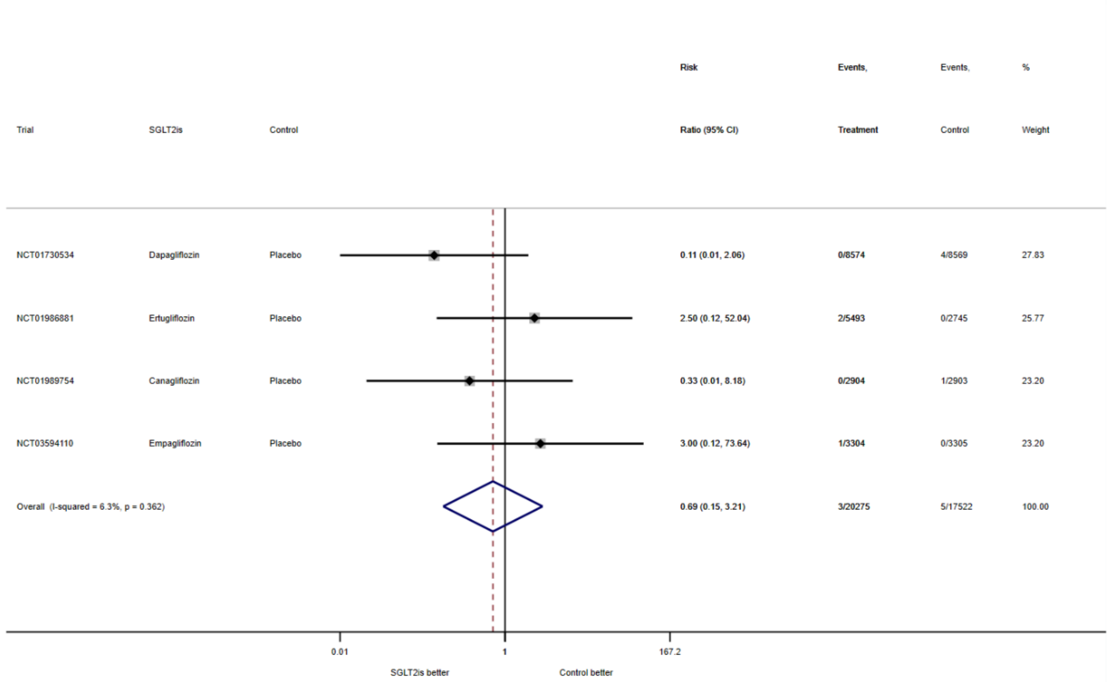

System 16-Neoplasms Benign, Malignant And Unspecified-Figure S119 Meta-analysis of SGLT2is and Small cell lung cancer metastatic

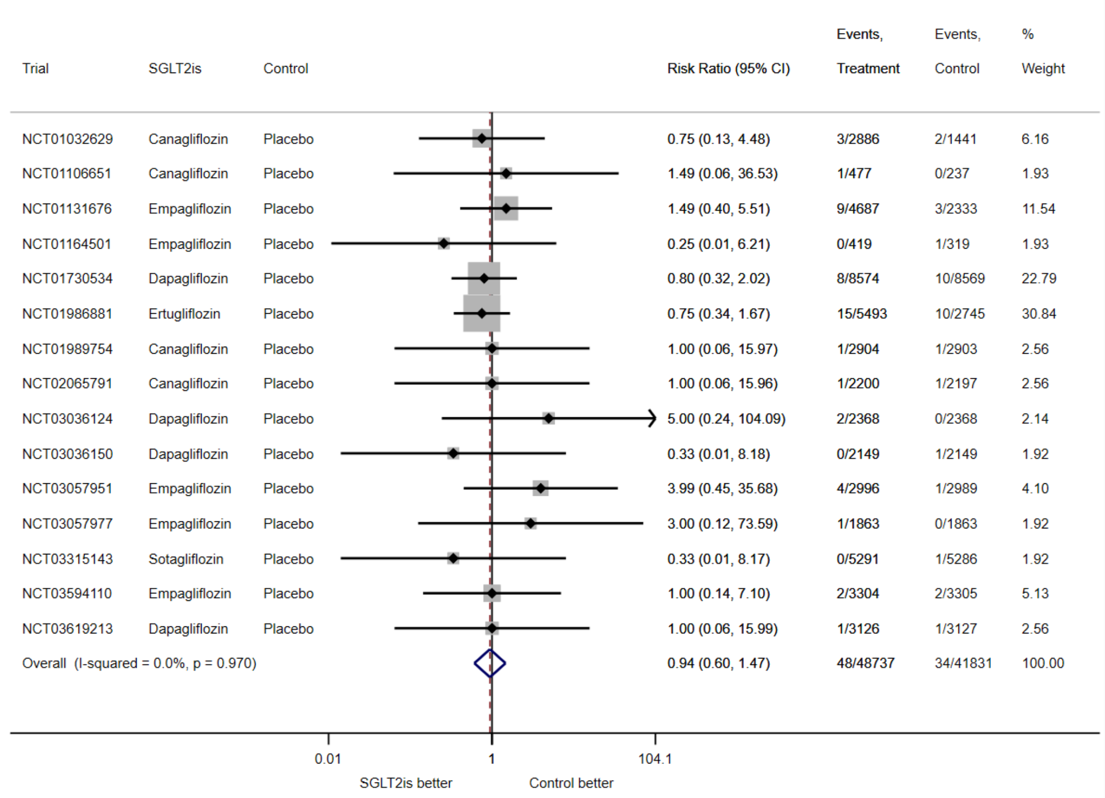

System 16-Neoplasms Benign, Malignant And Unspecified-Figure S120 Meta-analysis of SGLT2is and Squamous cell carcinoma

Trial	SGLT2is	Control	Risk Ratio (95% CI)	Events, Treatment	Events, Control	% Weight
NCT01131676	Empagliflozin	Placebo	1.49 (0.16, 14.35)	3/4687	1/2333	11.53
NCT01730534	Dapagliflozin	Placebo	1.33 (0.30, 5.95)	4/8574	3/8569	26.35
NCT01986881	Ertugliflozin	Placebo	0.50 (0.03, 7.99)	1/5493	1/2745	7.68
NCT01989754	Canagliflozin	Placebo	0.33 (0.01, 8.18)	0/2904	1/2903	5.76
NCT02065791	Canagliflozin	Placebo	1.00 (0.06, 15.96)	1/2200	1/2197	7.69
NCT03036124	Dapagliflozin	Placebo	0.14 (0.01, 2.76)	0/2368	3/2368	6.72
NCT03057951	Empagliflozin	Placebo	0.14 (0.01, 2.76)	0/2996	3/2989	6.72
NCT03057977	Empagliflozin	Placebo	0.20 (0.01, 4.16)	0/1863	2/1863	6.40
NCT03315143	Sotagliflozin	Placebo	0.33 (0.01, 8.17)	0/5291	1/5286	5.76
NCT03594110	Empagliflozin	Placebo	1.00 (0.06, 15.99)	1/3304	1/3305	7.68
NCT03619213	Dapagliflozin	Placebo	1.00 (0.06, 15.99)	1/3126	1/3127	7.68
Overall (I-squared = 0.0%, p = 0.914)			0.65 (0.30, 1.41)	11/42806	18/37685	100.00

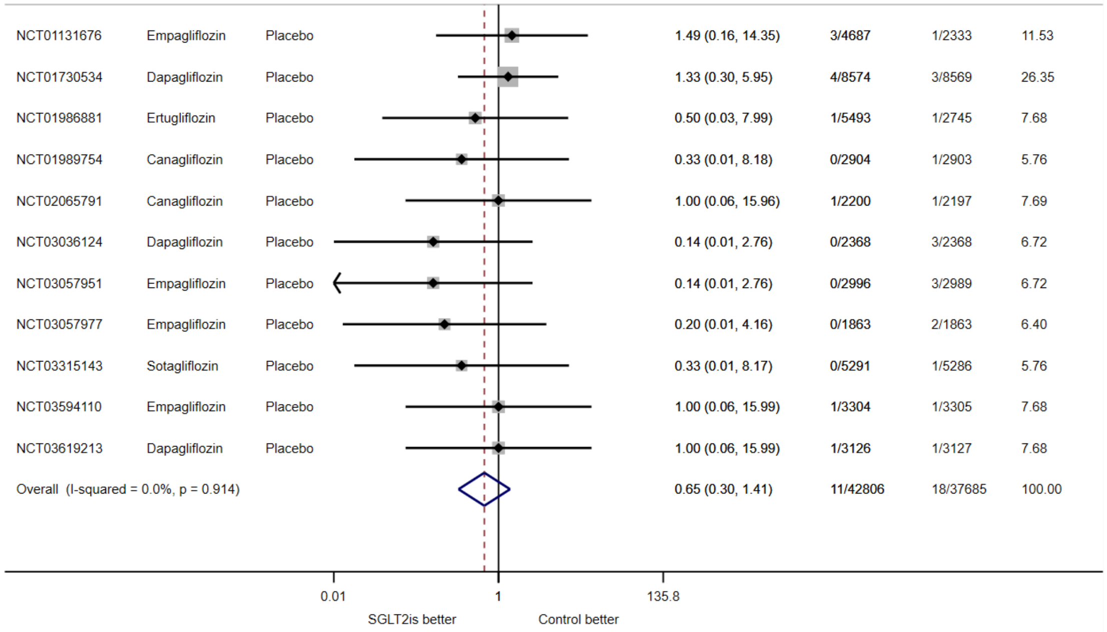

System 16-Neoplasms Benign, Malignant And Unspecified-Figure S121 Meta-analysis of SGLT2is and Squamous cell carcinoma of lung

Trial	SGLT2is	Control	Risk Ratio (95% CI)	Events, Treatment	Events, Control	% Weight
NCT01032629	Canagliflozin	Placebo	0.50 (0.03, 7.98)	1/2886	1/1441	2.90
NCT01131676	Empagliflozin	Placebo	0.50 (0.17, 1.42)	7/4687	7/2333	20.33
NCT01164501	Empagliflozin	Placebo	0.25 (0.01, 6.21)	0/419	1/319	2.18
NCT01730534	Dapagliflozin	Placebo	0.44 (0.14, 1.44)	4/8574	9/8569	16.06
NCT01986881	Ertugliflozin	Placebo	2.50 (0.29, 21.38)	5/5493	1/2745	4.83
NCT01989754	Canagliflozin	Placebo	3.00 (0.12, 73.59)	1/2904	0/2903	2.17
NCT02065791	Canagliflozin	Placebo	4.99 (0.24, 103.94)	2/2200	0/2197	2.42
NCT03036150	Dapagliflozin	Placebo	3.00 (0.12, 73.60)	1/2149	0/2149	2.17
NCT03057951	Empagliflozin	Placebo	0.78 (0.29, 2.08)	7/2996	9/2989	22.88
NCT03057977	Empagliflozin	Placebo	6.00 (0.72, 49.79)	6/1863	1/1863	4.97
NCT03242252	Sotagliflozin	Placebo	0.16 (0.01, 4.03)	0/527	1/260	2.18
NCT03315143	Sotagliflozin	Placebo	3.00 (0.31, 28.80)	3/5291	1/5286	4.35
NCT03594110	Empagliflozin	Placebo	1.00 (0.20, 4.95)	3/3304	3/3305	8.70
NCT03619213	Dapagliflozin	Placebo	2.00 (0.18, 22.05)	2/3126	1/3127	3.87
Overall (I-squared = 0.0%, p = 0.499)			0.88 (0.55, 1.41)	42/46419	35/39486	100.00

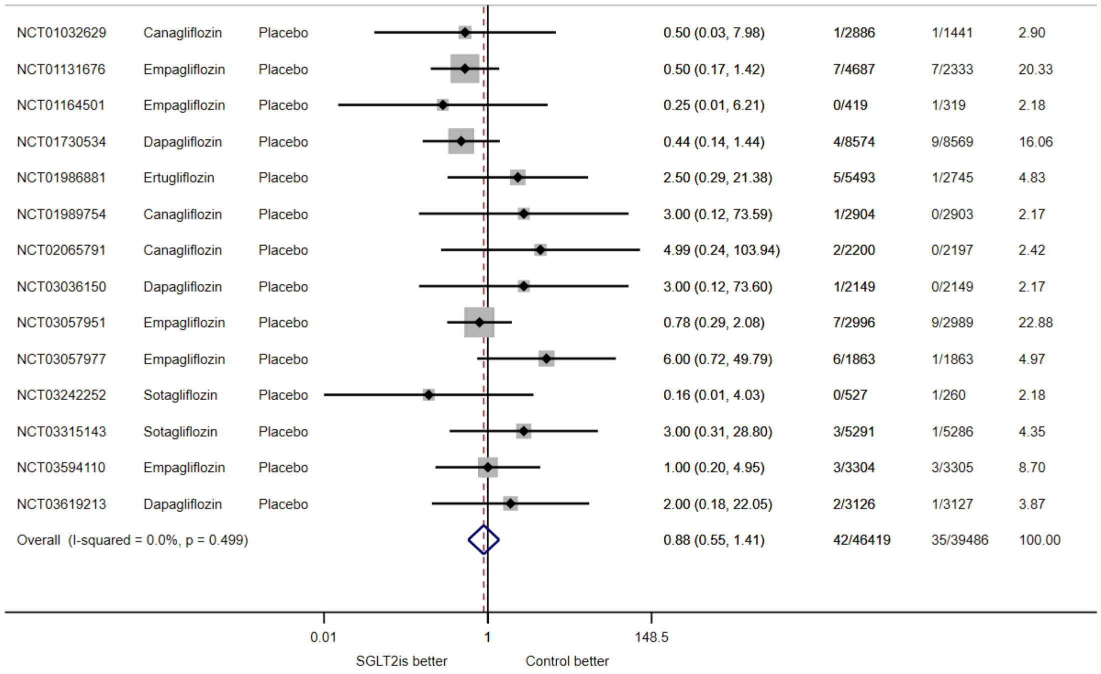

System 16-Neoplasms Benign, Malignant And Unspecified-Figure S122 Meta-analysis of SGLT2is and Squamous cell carcinoma of skin

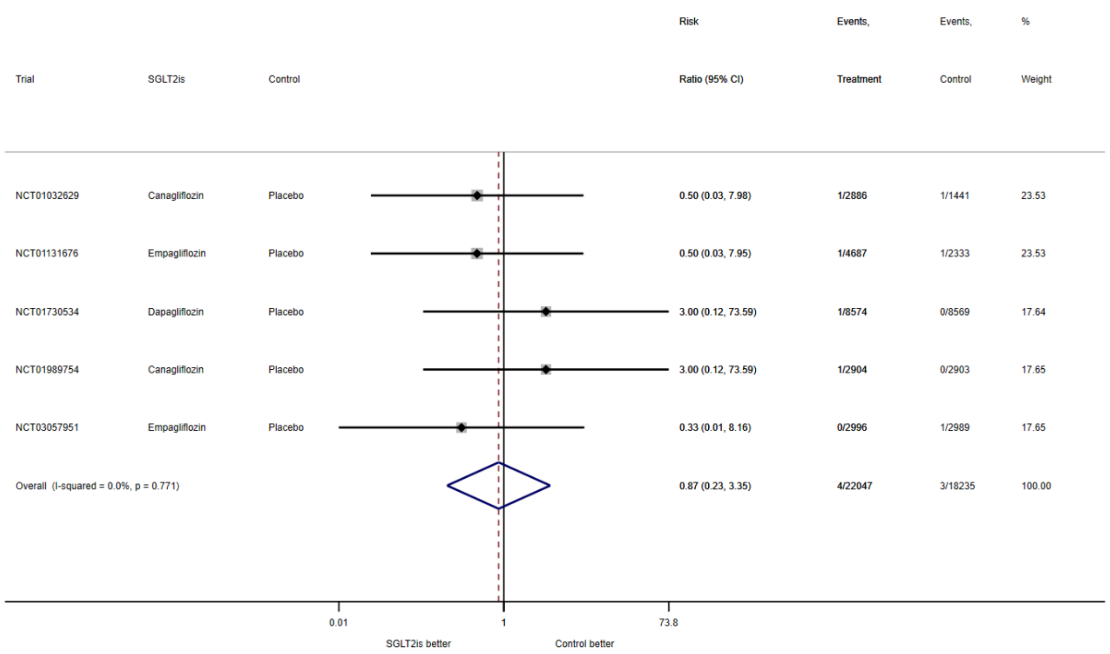

System 16-Neoplasms Benign, Malignant And Unspecified-Figure S123 Meta-analysis of SGLT2is and Squamous cell carcinoma of the oral cavity

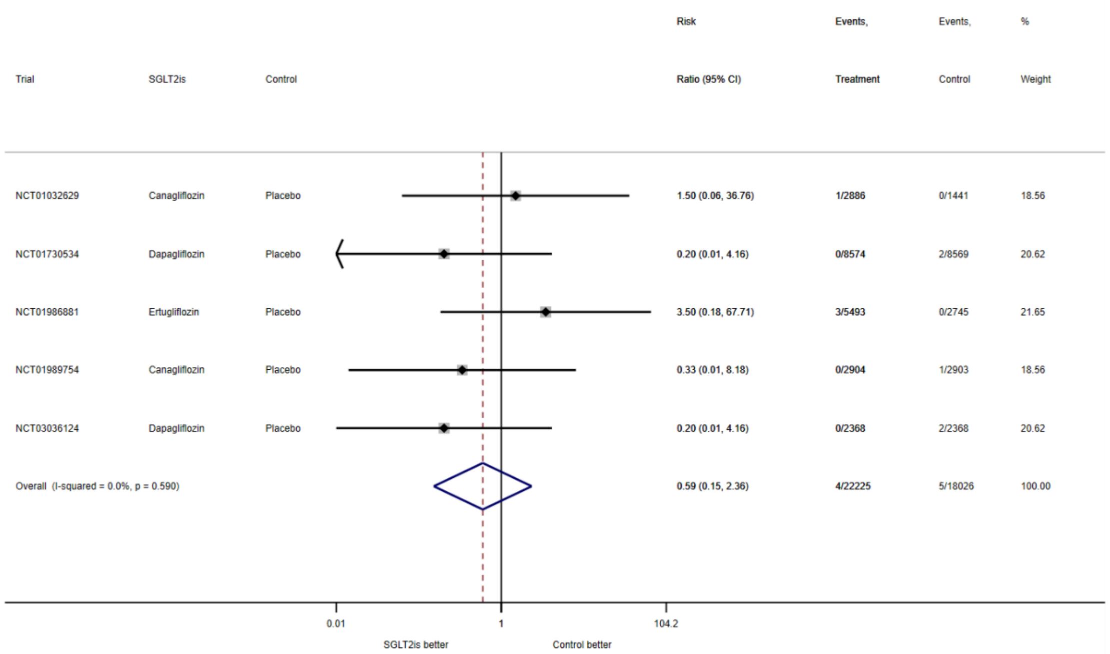

System 16-Neoplasms Benign, Malignant And Unspecified-Figure S124 Meta-analysis of SGLT2is and Squamous cell carcinoma of the tongue

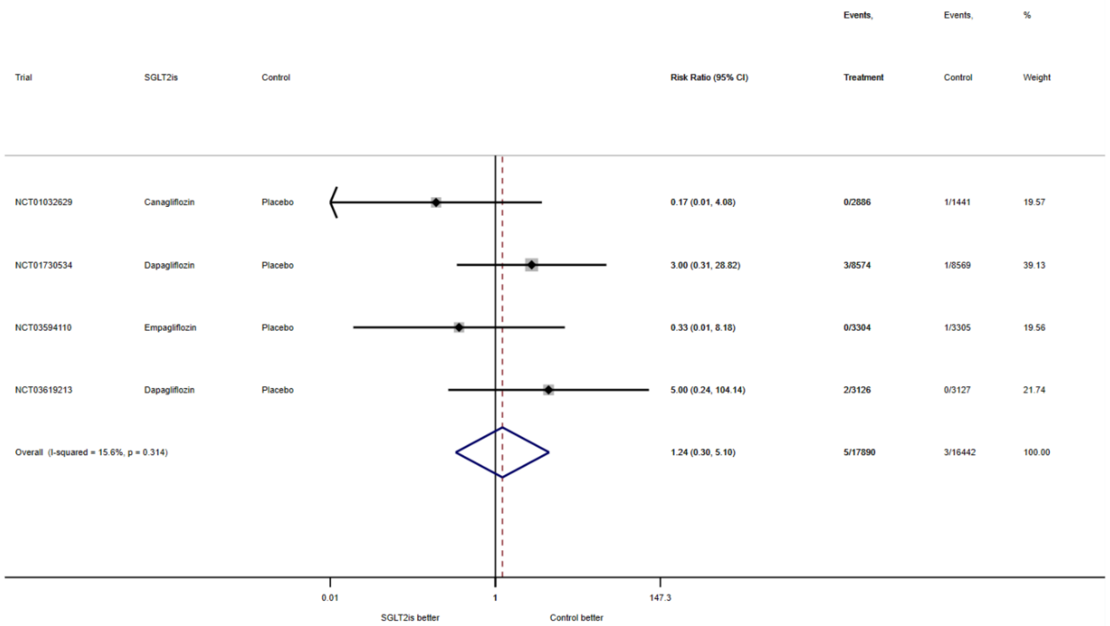

System 16-Neoplasms Benign, Malignant And Unspecified-Figure S125 Meta-analysis of SGLT2is and Thyroid cancer

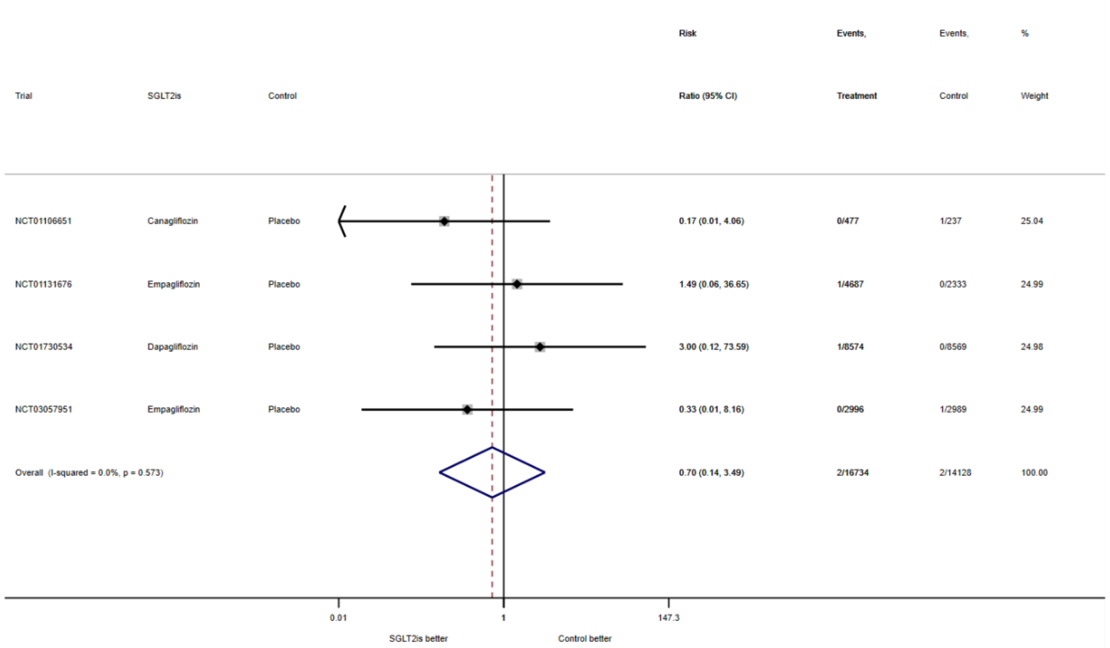

System 16-Neoplasms Benign, Malignant And Unspecified-Figure S126 Meta-analysis of SGLT2is and Thyroid neoplasm

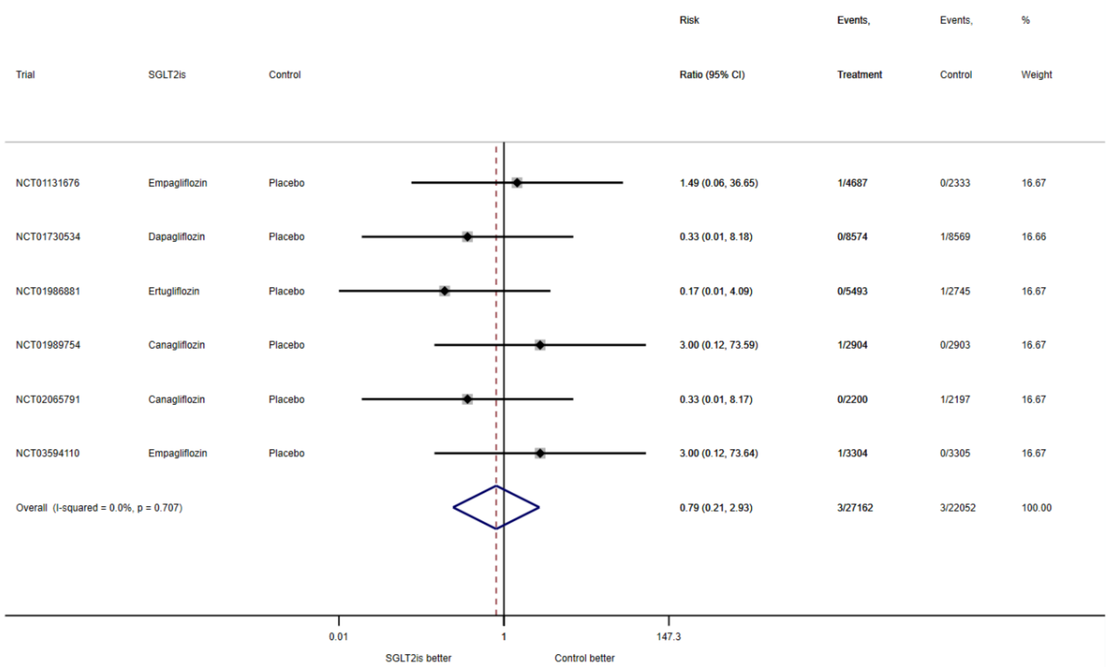

System 16-Neoplasms Benign, Malignant And Unspecified-Figure S127 Meta-analysis of SGLT2is and Tongue neoplasm malignant stage unspecified

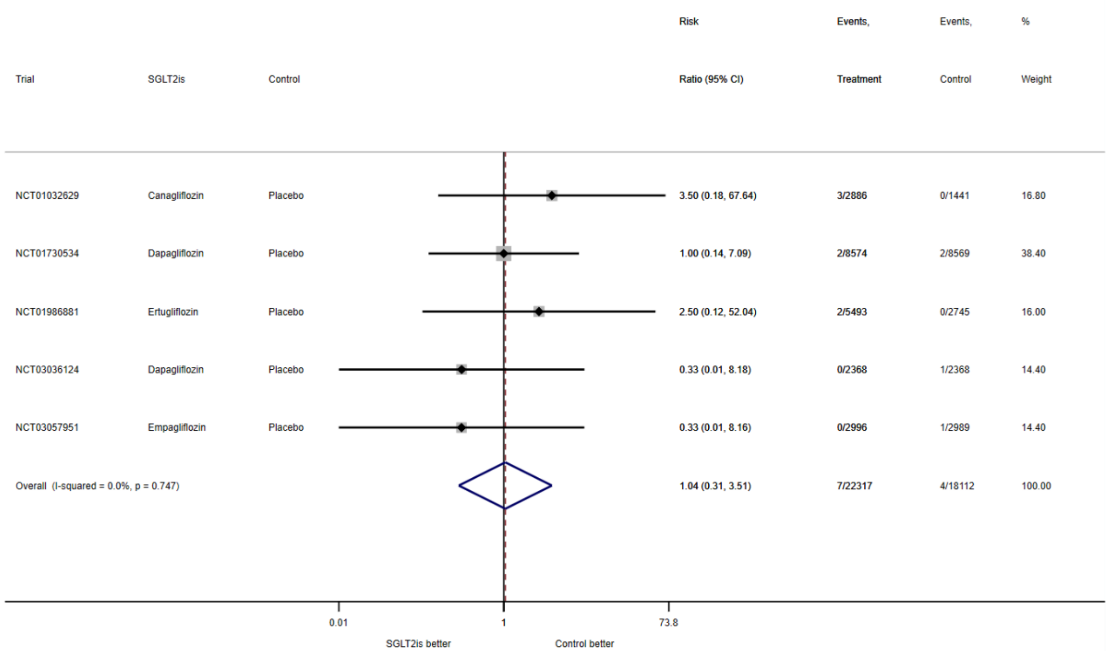

System 16-Neoplasms Benign, Malignant And Unspecified-Figure S128 Meta-analysis of SGLT2is and Tonsil cancer

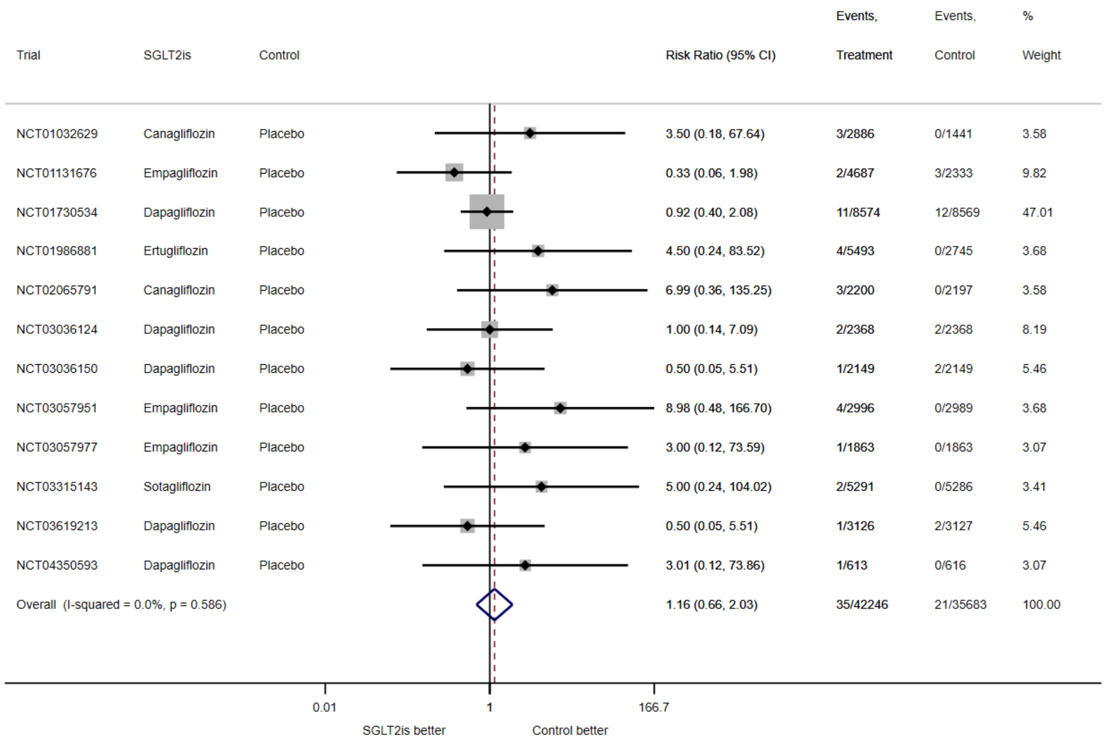

System 16-Neoplasms Benign, Malignant And Unspecified-Figure S129 Meta-analysis of SGLT2is and Transitional cell carcinoma

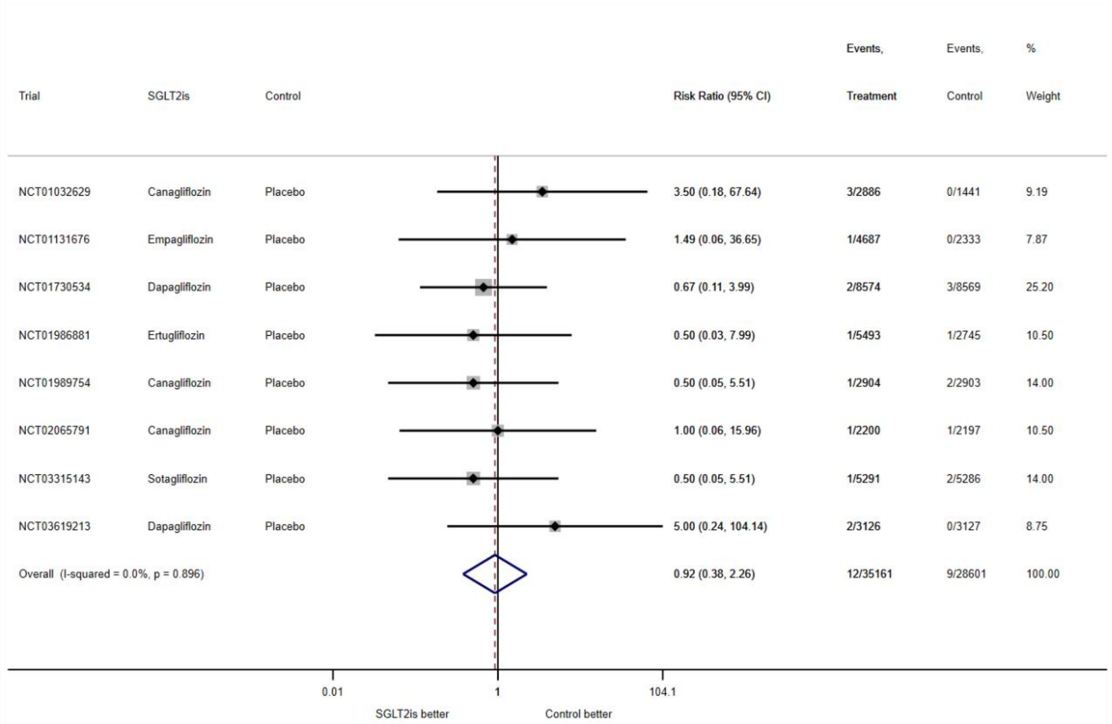

System 16-Neoplasms Benign, Malignant And Unspecified-Figure S130 Meta-analysis of SGLT2is and Uterine cancer

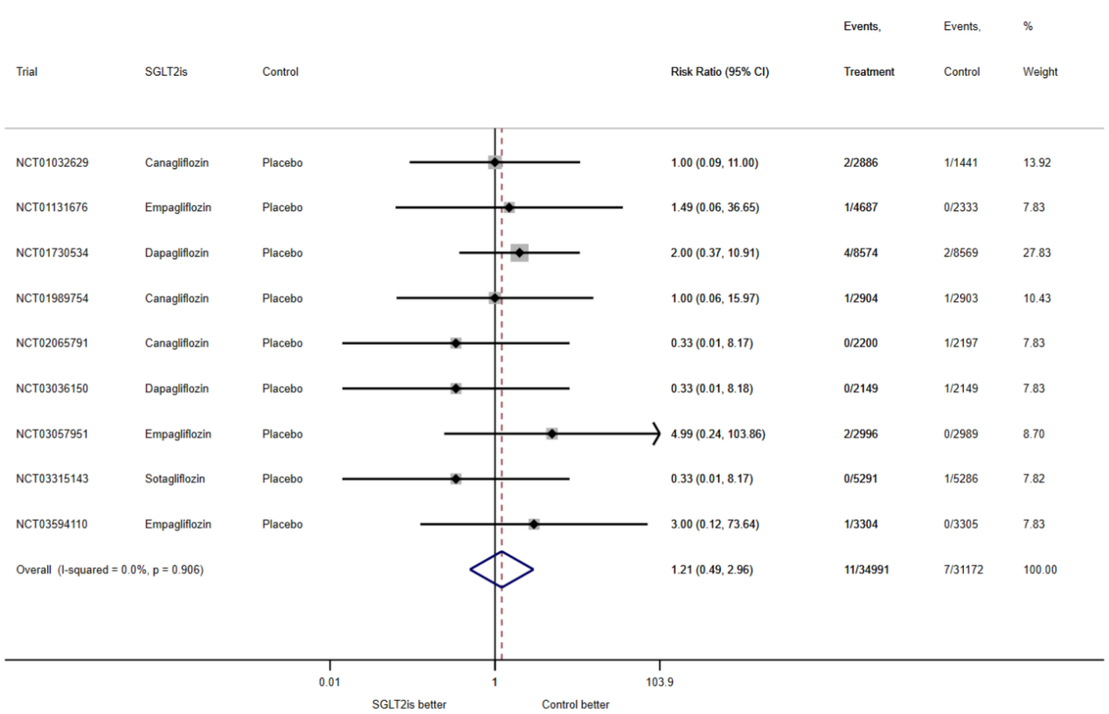

System 16-Neoplasms Benign, Malignant And Unspecified-Figure S131 Meta-analysis of SGLT2is and Uterine leiomyoma

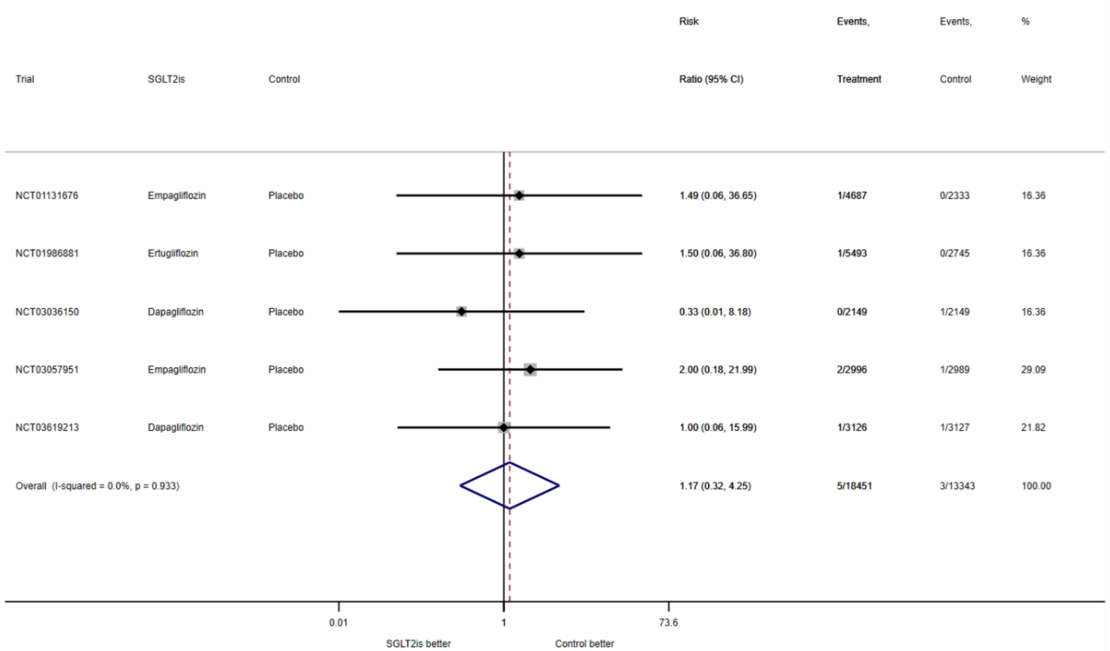

System 17-Nervous System Disorders-Figure S1 Meta-analysis of SGLT2is and Altered state of consciousness

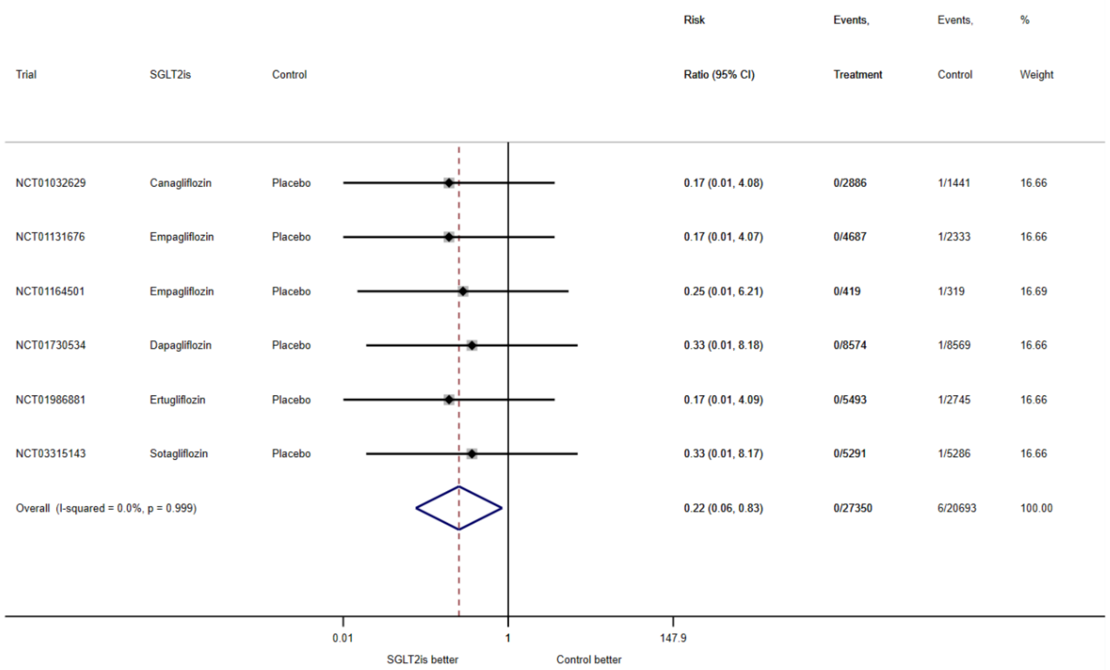

System 17-Nervous System Disorders-Figure S2 Meta-analysis of SGLT2is and Aphasia

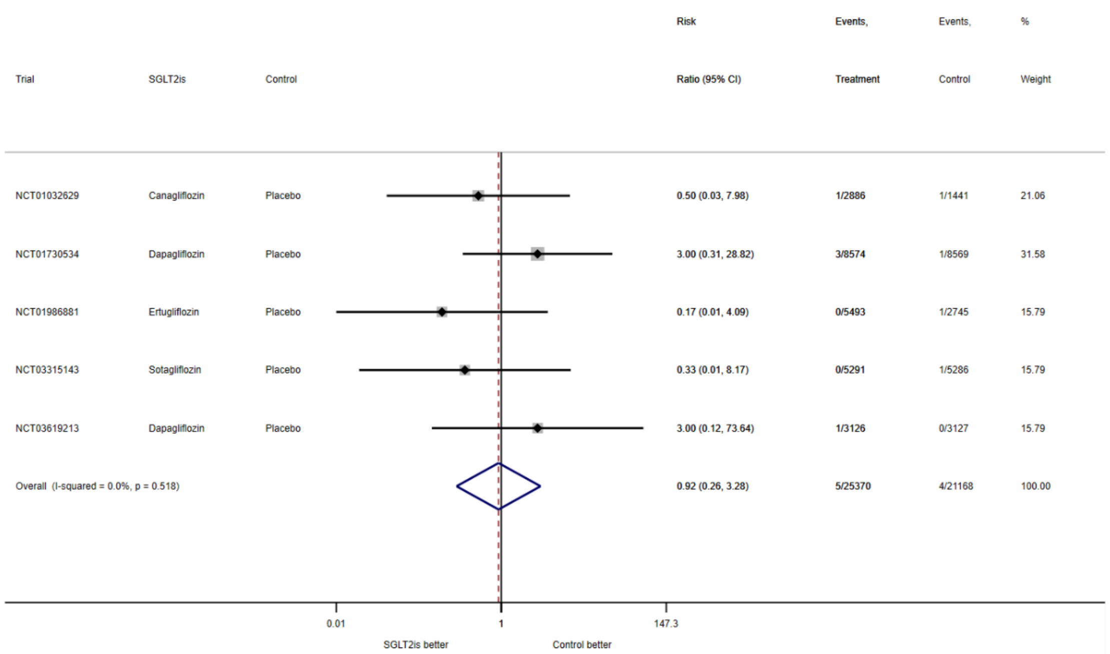

System 17-Nervous System Disorders-Figure S3 Meta-analysis of SGLT2is and Ataxia

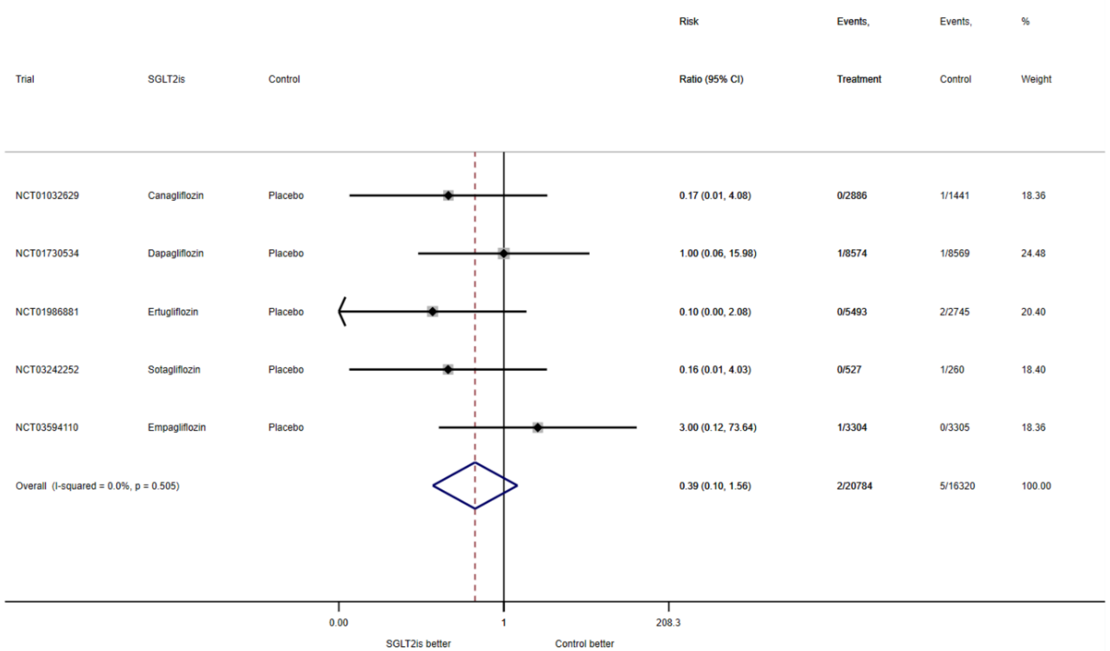

System 17-Nervous System Disorders-Figure S4 Meta-analysis of SGLT2is and Basal ganglia infarction

Trial	SGLT2is	Control	Risk Ratio (95% CI)	Events, Treatment	Events, Control	% Weight
NCT01032629	Canagliflozin	Placebo	1.50 (0.06, 36.76)	1/2886	0/1441	5.87
NCT01730534	Dapagliflozin	Placebo	0.50 (0.13, 2.00)	3/8574	6/8569	31.31
NCT01986881	Ertugliflozin	Placebo	4.00 (0.50, 31.95)	8/5493	1/2745	13.91
NCT01989754	Canagliflozin	Placebo	3.00 (0.12, 73.59)	1/2904	0/2903	5.87
NCT02065791	Canagliflozin	Placebo	3.00 (0.12, 73.50)	1/2200	0/2197	5.87
NCT03036124	Dapagliflozin	Placebo	0.33 (0.01, 8.18)	0/2368	1/2368	5.87
NCT03036150	Dapagliflozin	Placebo	0.33 (0.01, 8.18)	0/2149	1/2149	5.87
NCT03057951	Empagliflozin	Placebo	0.33 (0.03, 3.20)	1/2996	3/2989	11.74
NCT03057977	Empagliflozin	Placebo	3.00 (0.12, 73.59)	1/1863	0/1863	5.87
NCT03315143	Sotagliflozin	Placebo	1.00 (0.06, 15.97)	1/5291	1/5286	7.82
Overall (I-squared = 0.0%, p = 0.752)			0.94 (0.43, 2.03)	17/36724	13/32510	100.00

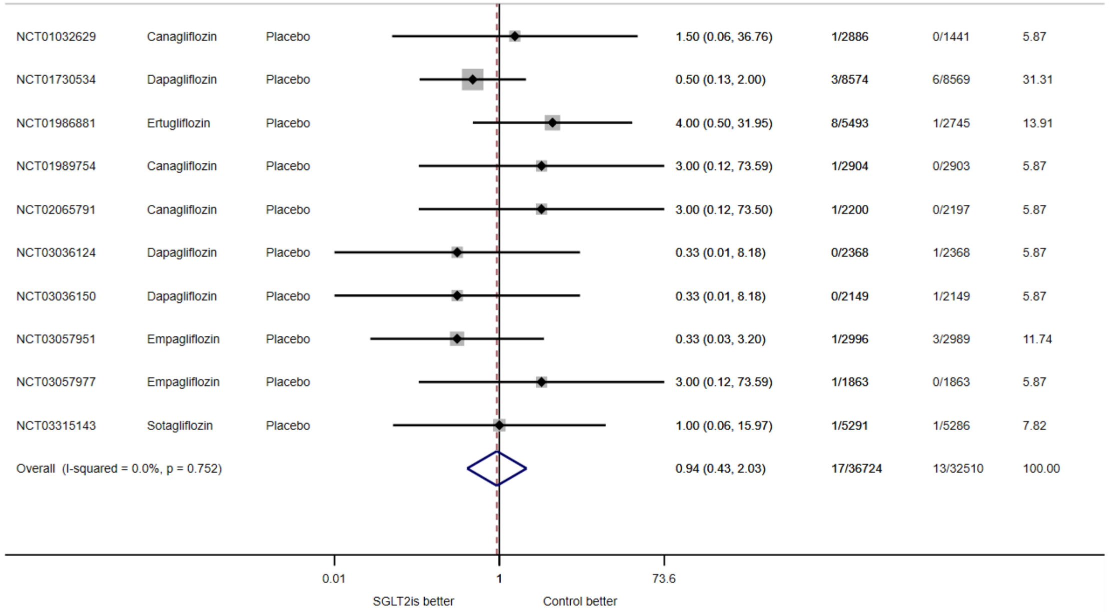

System 17-Nervous System Disorders-Figure S5 Meta-analysis of SGLT2is and Brain injury

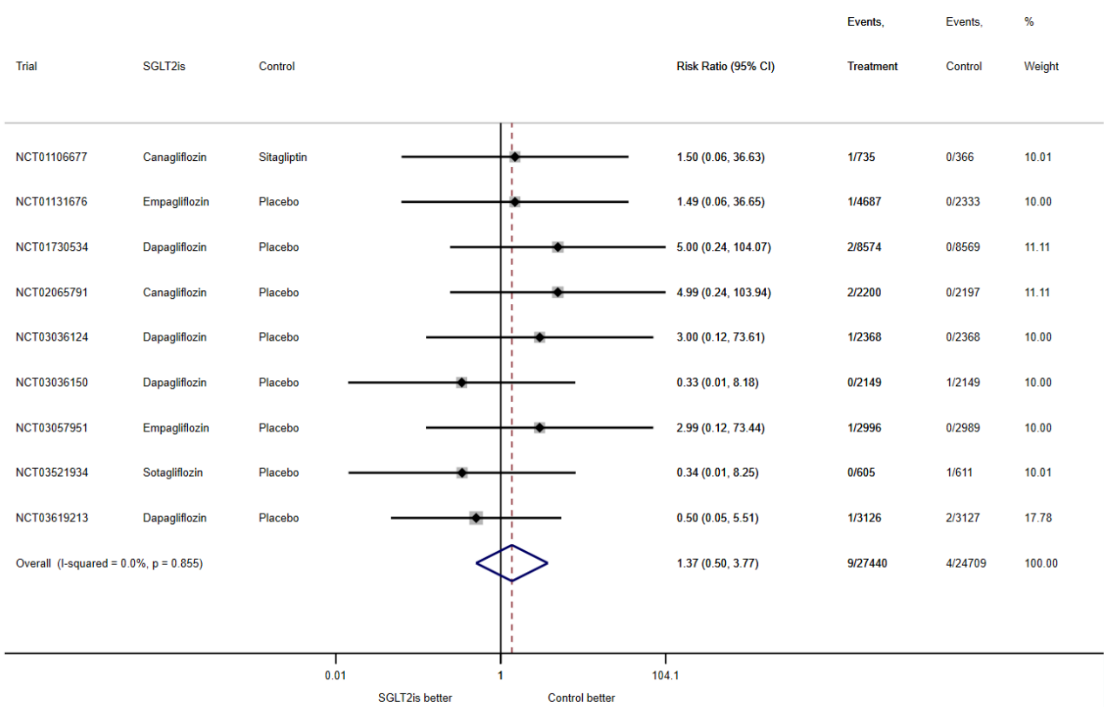

System 17-Nervous System Disorders-Figure S6 Meta-analysis of SGLT2is and Brain oedema

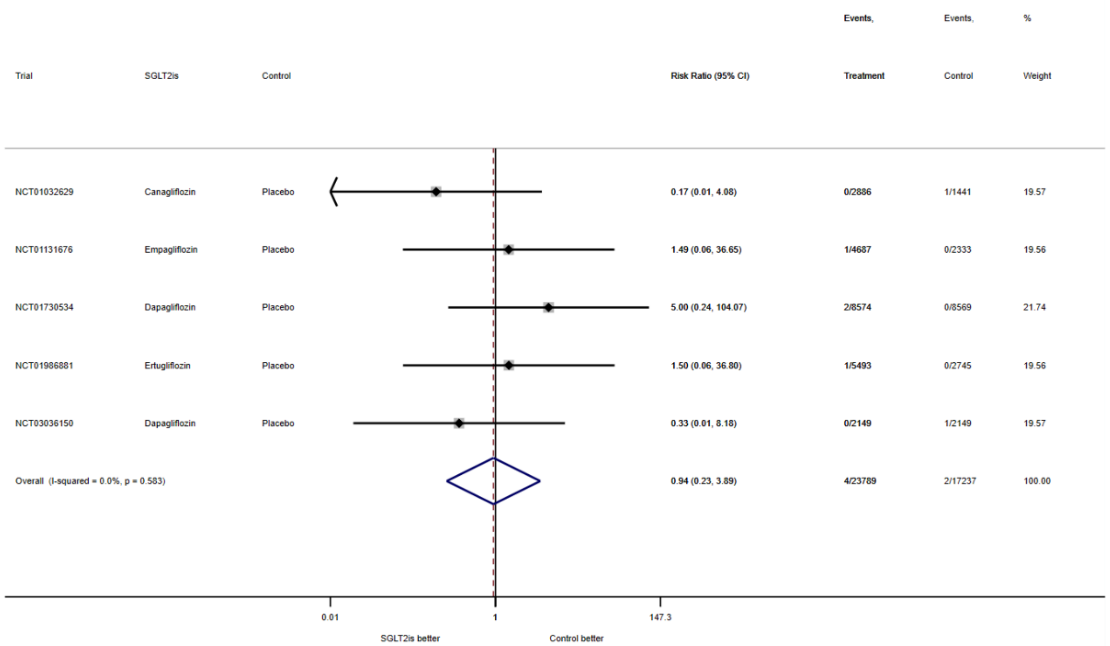

System 17-Nervous System Disorders-Figure S7 Meta-analysis of SGLT2is and Brain stem haemorrhage

Trial	SGLT2is	Control	Risk Ratio (95% CI)	Events, Treatment	Events, Control	% Weight
NCT01032629	Canagliflozin	Placebo	2.50 (0.12, 51.99)	2/2886	0/1441	7.02
NCT01131676	Empagliflozin	Placebo	3.49 (0.18, 67.44)	3/4687	0/2333	7.37
NCT01730534	Dapagliflozin	Placebo	2.00 (0.50, 7.99)	6/8574	3/8569	33.69
NCT01986881	Ertugliflozin	Placebo	1.50 (0.06, 36.80)	1/5493	0/2745	6.31
NCT01989754	Canagliflozin	Placebo	3.00 (0.12, 73.59)	1/2904	0/2903	6.32
NCT03036124	Dapagliflozin	Placebo	3.00 (0.12, 73.61)	1/2368	0/2368	6.32
NCT03036150	Dapagliflozin	Placebo	0.33 (0.01, 8.18)	0/2149	1/2149	6.32
NCT03315143	Sotagliflozin	Placebo	0.33 (0.01, 8.17)	0/5291	1/5286	6.31
NCT03594110	Empagliflozin	Placebo	3.00 (0.12, 73.64)	1/3304	0/3305	6.31
NCT03619213	Dapagliflozin	Placebo	5.00 (0.58, 42.79)	5/3126	1/3127	14.04
Overall (I-squared = 0.0%, p = 0.942)			2.03 (0.91, 4.55)	20/40782	6/34226	100.00

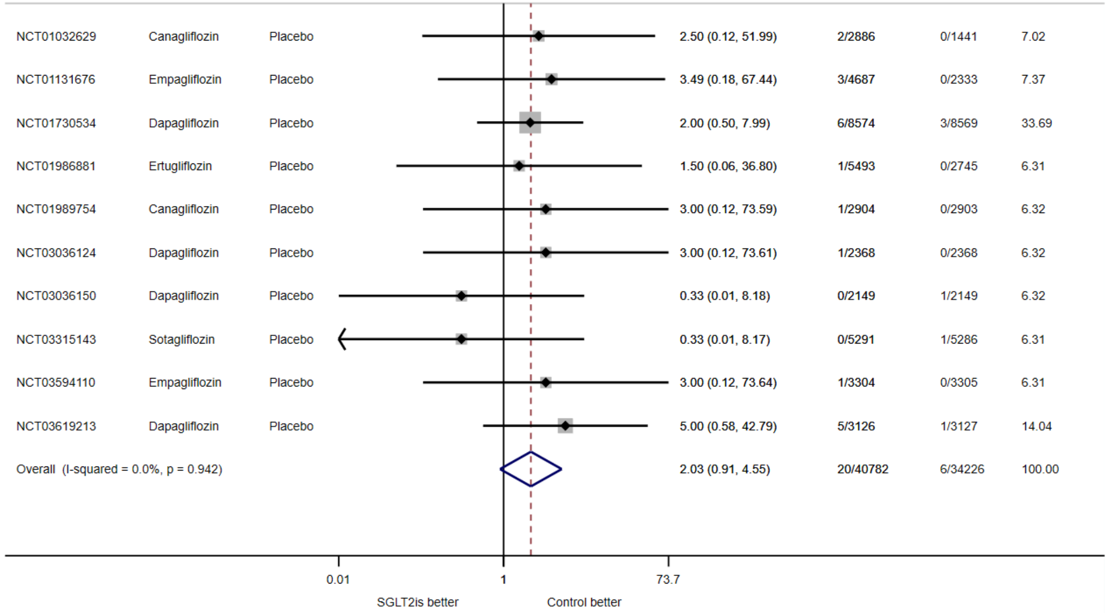

System 17-Nervous System Disorders-Figure S8 Meta-analysis of SGLT2is and Brain stem infarction

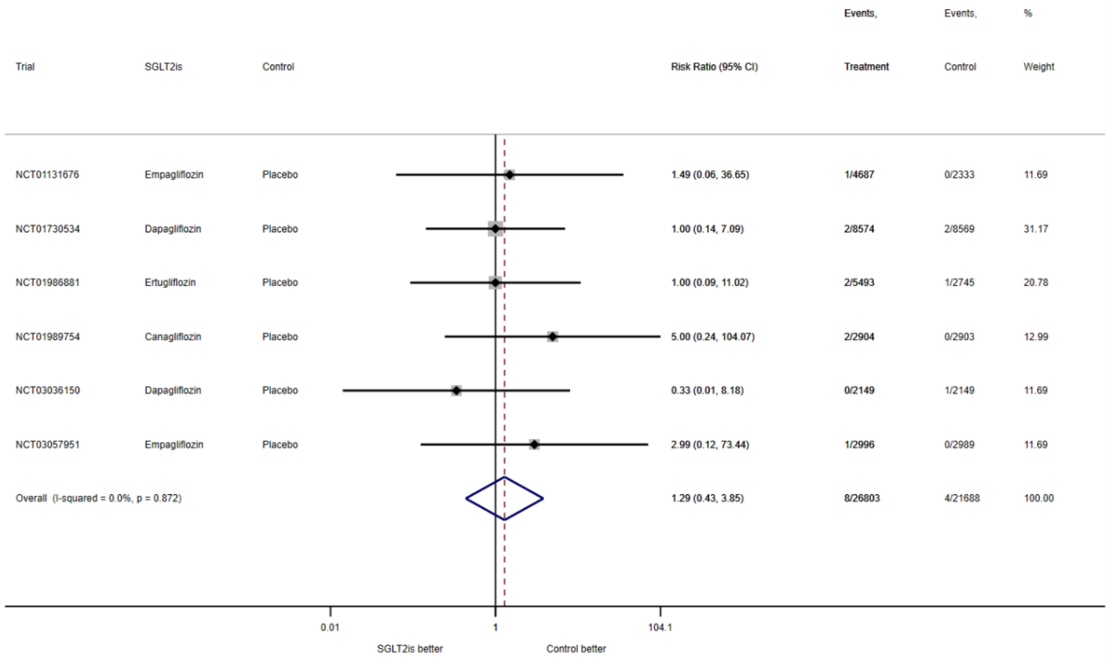

System 17-Nervous System Disorders-Figure S9 Meta-analysis of SGLT2is and Brain stem stroke

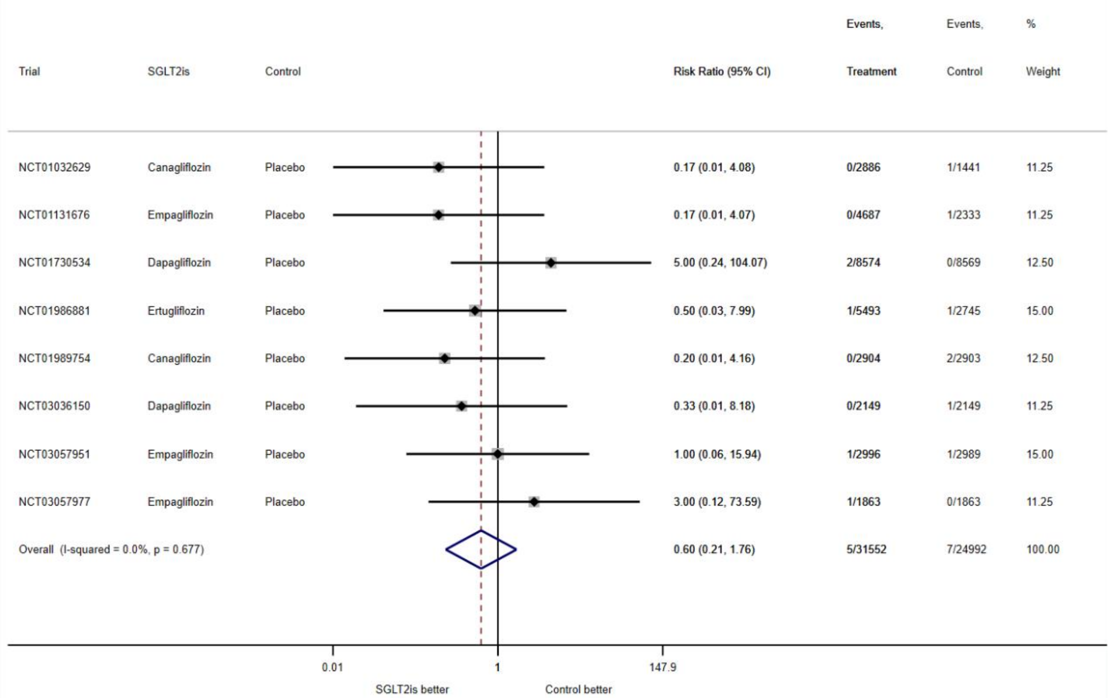

System 17-Nervous System Disorders-Figure S10 Meta-analysis of SGLT2is and Carotid arteriosclerosis

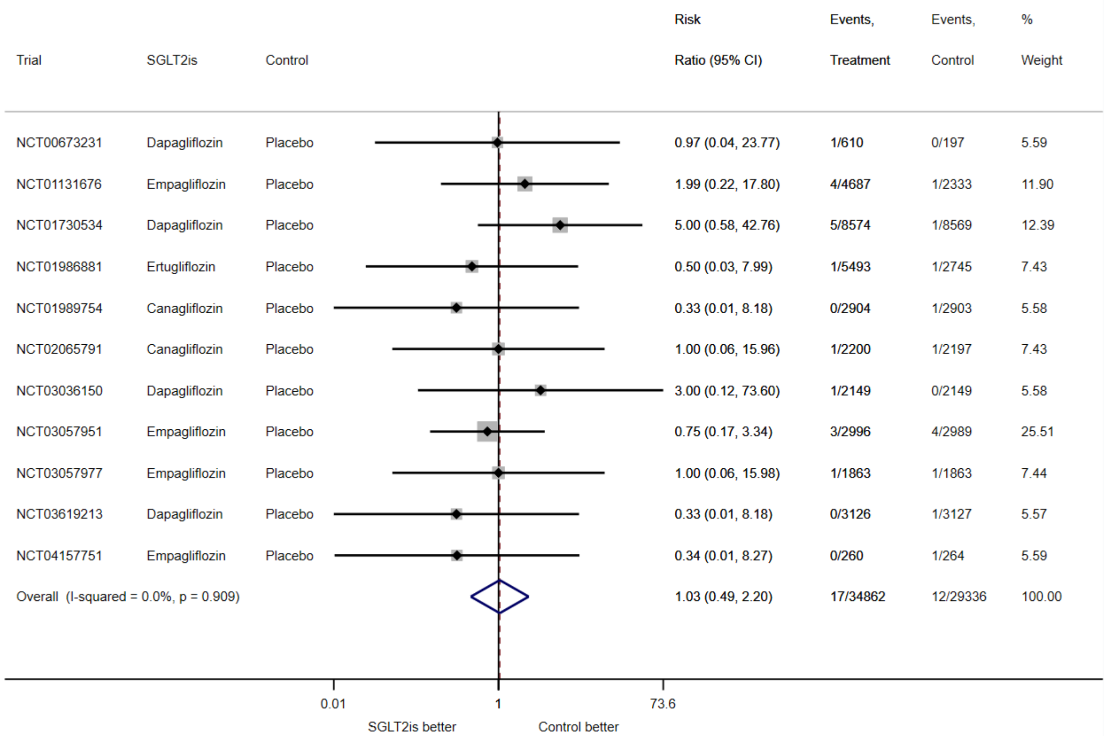

System 17-Nervous System Disorders-Figure S11 Meta-analysis of SGLT2is and Carotid artery occlusion

Trial	SGLT2is	Control	Risk Ratio (95% CI)	Events, Treatment	Events, Control	% Weight
NCT01032629	Canagliflozin	Placebo	1.60 (0.59, 4.35)	16/2886	5/1441	8.50
NCT01042977	Dapagliflozin	Placebo	5.01 (0.24, 104.09)	2/482	0/483	0.93
NCT01106651	Canagliflozin	Placebo	1.49 (0.06, 36.53)	1/477	0/237	0.84
NCT01131676	Empagliflozin	Placebo	0.95 (0.44, 2.03)	19/4687	10/2333	14.62
NCT01730534	Dapagliflozin	Placebo	1.28 (0.73, 2.27)	27/8574	21/8569	26.33
NCT01986881	Ertugliflozin	Placebo	1.00 (0.50, 2.00)	24/5493	12/2745	17.86
NCT01989754	Canagliflozin	Placebo	1.29 (0.48, 3.45)	9/2904	7/2903	8.78
NCT02065791	Canagliflozin	Placebo	0.50 (0.15, 1.66)	4/2200	8/2197	5.94
NCT03036124	Dapagliflozin	Placebo	1.50 (0.25, 8.97)	3/2368	2/2368	2.67
NCT03036150	Dapagliflozin	Placebo	3.00 (0.61, 14.85)	6/2149	2/2149	3.34
NCT03057951	Empagliflozin	Placebo	0.50 (0.09, 2.72)	2/2996	4/2989	2.97
NCT03057977	Empagliflozin	Placebo	3.00 (0.12, 73.59)	1/1863	0/1863	0.83
NCT03315143	Sotagliflozin	Placebo	2.00 (0.37, 10.90)	4/5291	2/5286	2.97
NCT03521934	Sotagliflozin	Placebo	3.03 (0.12, 74.22)	1/605	0/611	0.83
NCT03594110	Empagliflozin	Placebo	0.20 (0.01, 4.17)	0/3304	2/3305	0.93
NCT03619213	Dapagliflozin	Placebo	3.00 (0.31, 28.83)	3/3126	1/3127	1.67
Overall (I-squared = 0.0%, p = 0.864)			1.18 (0.88, 1.58)	122/49405	76/42606	100.00

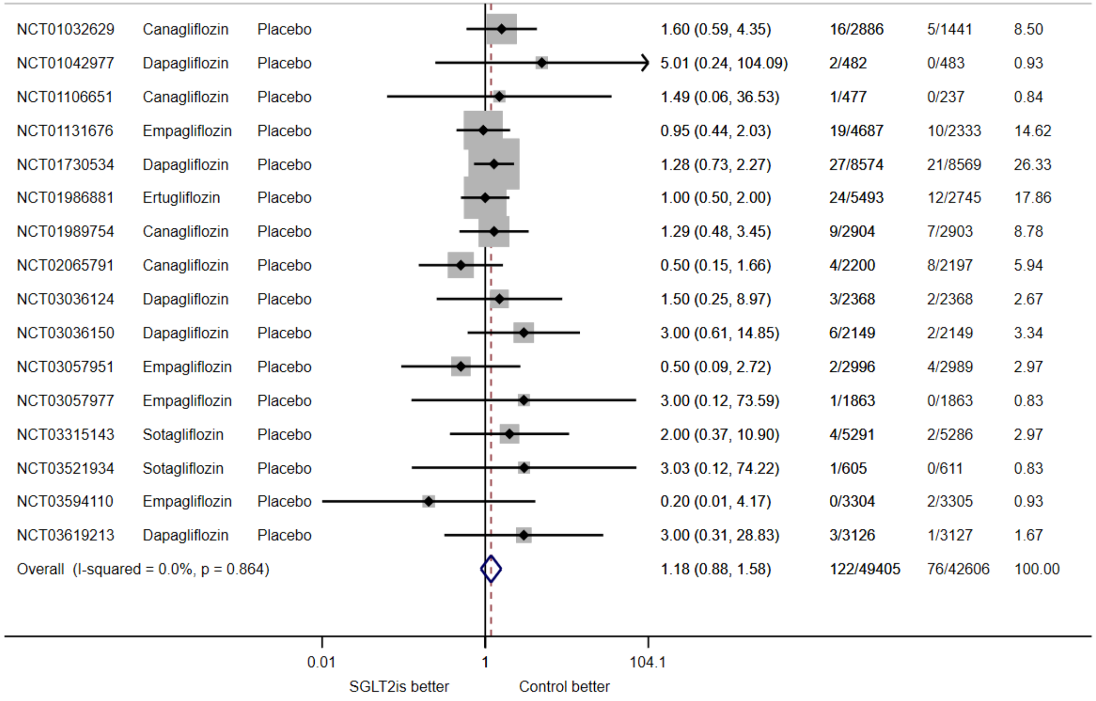

System 17-Nervous System Disorders-Figure S12 Meta-analysis of SGLT2is and Carotid artery stenosis

Trial	SGLT2is	Control	Risk Ratio (95% CI)	Events, Treatment	Events, Control	% Weight
NCT01032629	Canagliflozin	Placebo	1.00 (0.18, 5.45)	4/2886	2/1441	18.13
NCT01131676	Empagliflozin	Placebo	0.33 (0.06, 1.98)	2/4687	3/2333	16.31
NCT01730534	Dapagliflozin	Placebo	0.33 (0.03, 3.20)	1/8574	3/8569	10.19
NCT01986881	Ertugliflozin	Placebo	0.75 (0.13, 4.48)	3/5493	2/2745	16.31
NCT01989754	Canagliflozin	Placebo	1.00 (0.06, 15.97)	1/2904	1/2903	6.79
NCT02065791	Canagliflozin	Placebo	4.99 (0.24, 103.94)	2/2200	0/2197	5.66
NCT03036150	Dapagliflozin	Placebo	1.00 (0.06, 15.98)	1/2149	1/2149	6.79
NCT03057951	Empagliflozin	Placebo	4.99 (0.24, 103.86)	2/2996	0/2989	5.66
NCT03315143	Sotagliflozin	Placebo	2.00 (0.18, 22.03)	2/5291	1/5286	9.06
NCT03619213	Dapagliflozin	Placebo	0.33 (0.01, 8.18)	0/3126	1/3127	5.09
Overall (I-squared = 0.0%, p = 0.814)			0.86 (0.42, 1.77)	18/40306	14/33739	100.00

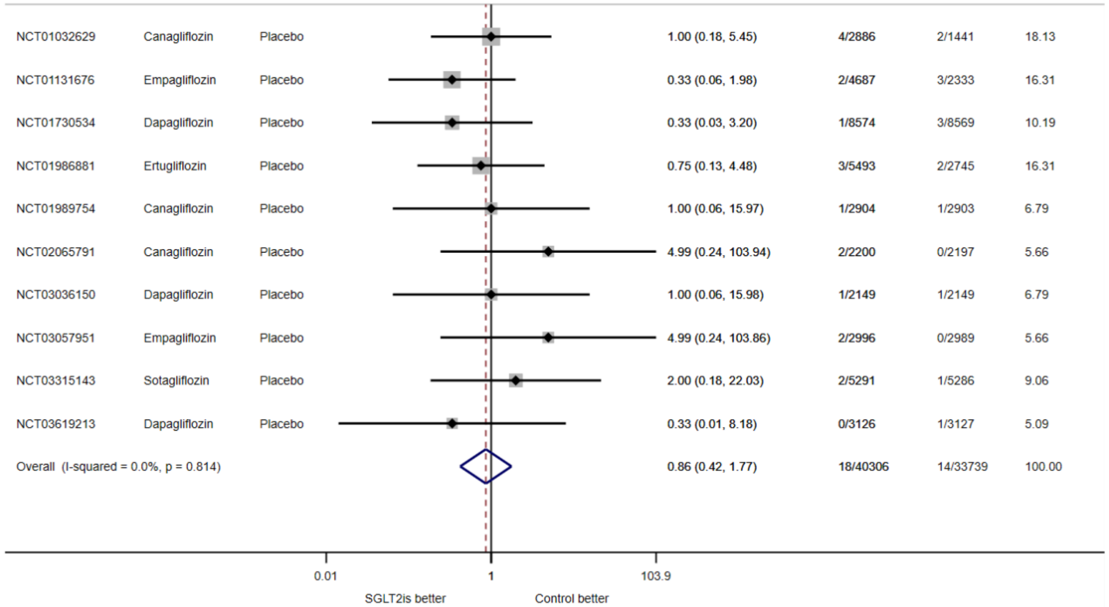

System 17-Nervous System Disorders-Figure S13 Meta-analysis of SGLT2is and Carpal tunnel syndrome

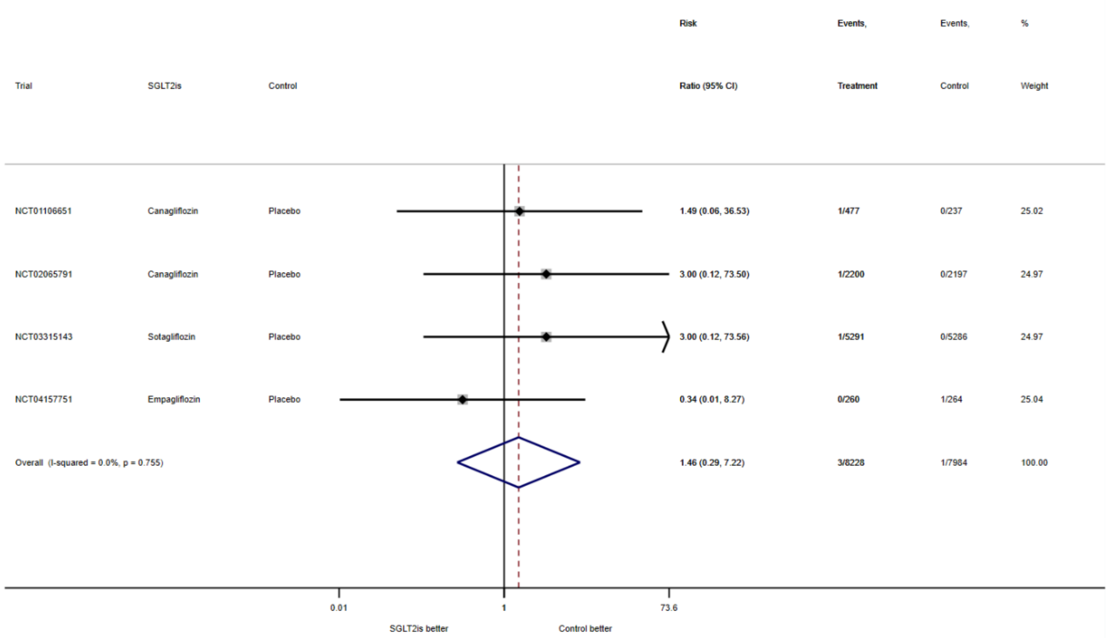

System 17-Nervous System Disorders-Figure S14 Meta-analysis of SGLT2is and Cauda equina syndrome

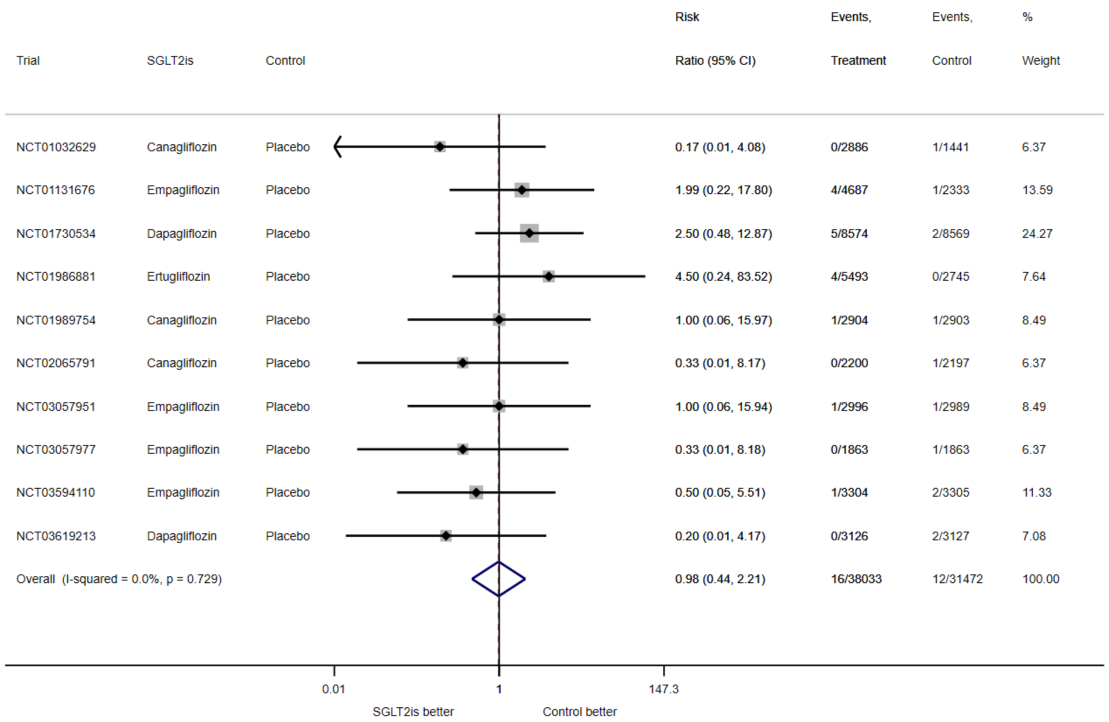

System 17-Nervous System Disorders-Figure S15 Meta-analysis of SGLT2is and Cerebellar infarction

Trial	SGLT2is	Control	Risk Ratio (95% CI)	Events, Treatment	Events, Control	% Weight
NCT01730534	Dapagliflozin	Placebo	0.33 (0.01, 8.18)	0/8574	1/8569	14.06
NCT01986881	Ertugliflozin	Placebo	2.50 (0.12, 52.04)	2/5493	0/2745	15.62
NCT02065791	Canagliflozin	Placebo	3.00 (0.12, 73.50)	1/2200	0/2197	14.06
NCT03036124	Dapagliflozin	Placebo	0.33 (0.01, 8.18)	0/2368	1/2368	14.06
NCT03036150	Dapagliflozin	Placebo	0.33 (0.01, 8.18)	0/2149	1/2149	14.06
NCT03057977	Empagliflozin	Placebo	0.33 (0.01, 8.18)	0/1863	1/1863	14.06
NCT03315143	Sotagliflozin	Placebo	3.00 (0.12, 73.56)	1/5291	0/5286	14.06
Overall (I-squared = 0.0%, p = 0.810)			0.85 (0.26, 2.81)	4/27938	4/25177	100.00

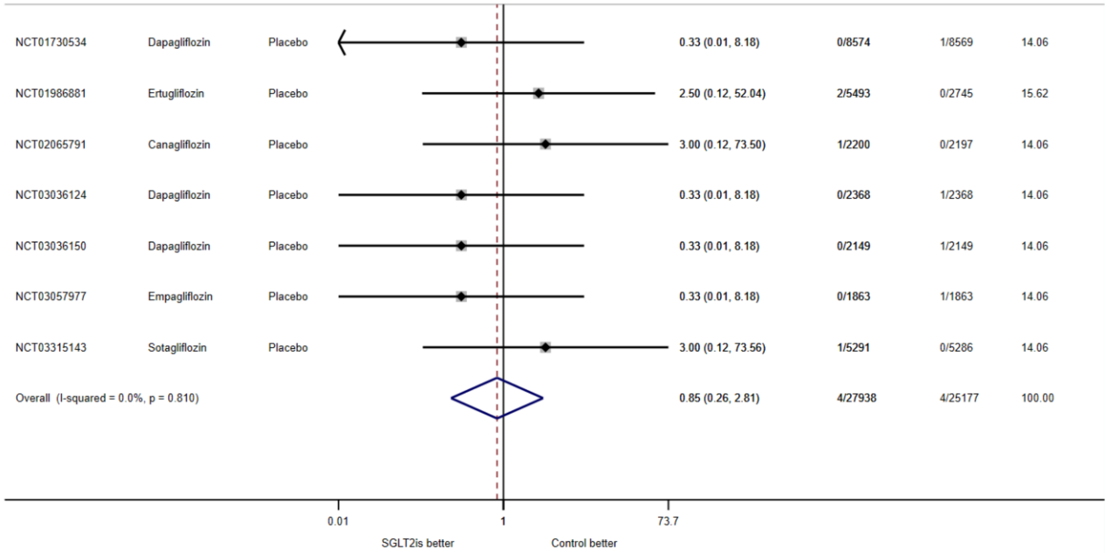

System 17-Nervous System Disorders-Figure S16 Meta-analysis of SGLT2is and Cerebellar stroke

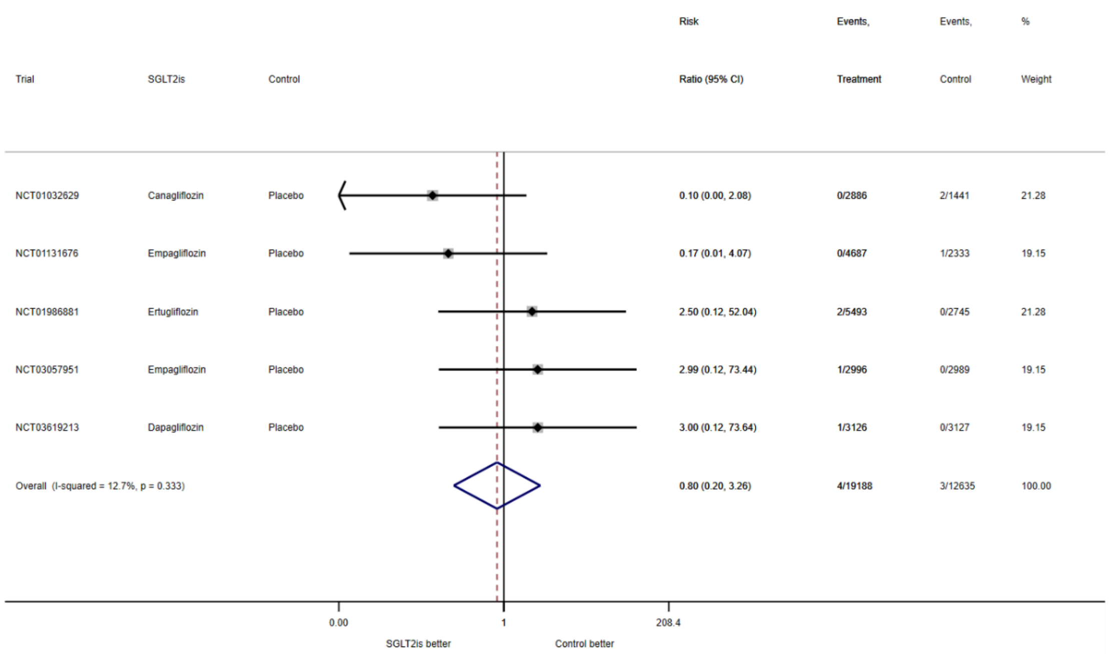

System 17-Nervous System Disorders-Figure S17 Meta-analysis of SGLT2is and Cerebral arteriosclerosis

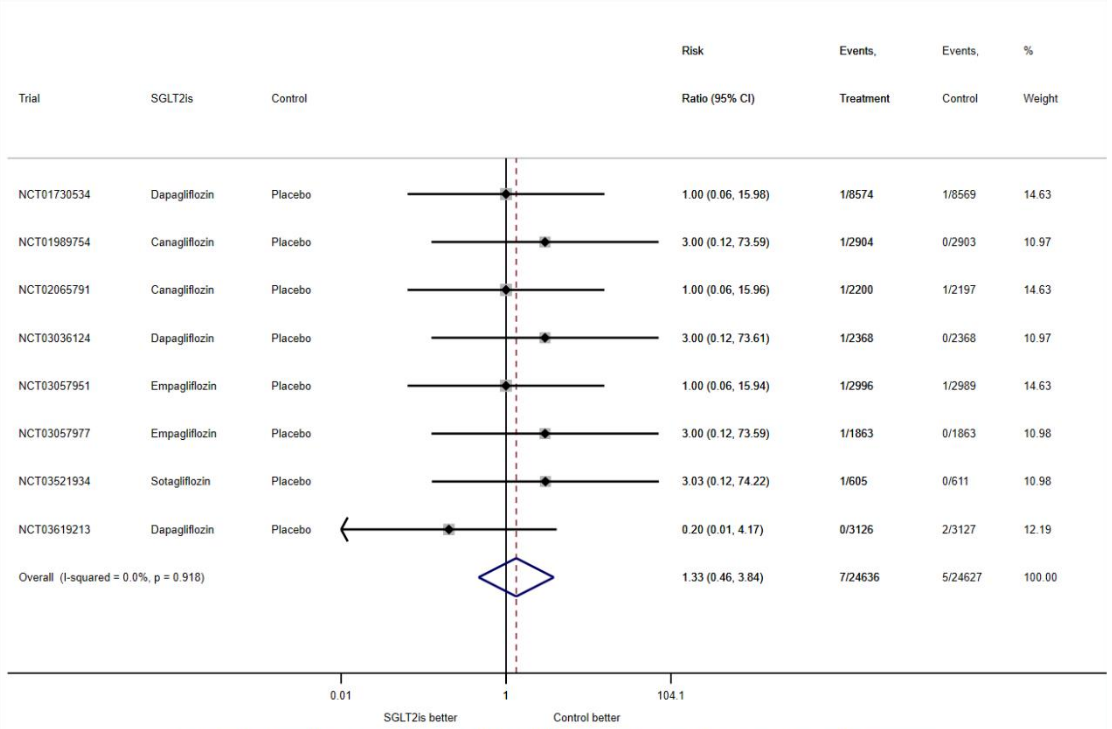

System 17-Nervous System Disorders-Figure S18 Meta-analysis of SGLT2is and Cerebral haematoma

Trial	SGLT2is	Control	Risk Ratio (95% CI)	Events, Treatment	Events, Control	% Weight
NCT01032629	Canagliflozin	Placebo	1.00 (0.09, 11.00)	2/2886	1/1441	6.00
NCT01730534	Dapagliflozin	Placebo	1.17 (0.39, 3.47)	7/8574	6/8569	29.09
NCT01986881	Ertugliflozin	Placebo	2.50 (0.12, 52.04)	2/5493	0/2745	3.75
NCT01989754	Canagliflozin	Placebo	3.00 (0.31, 28.81)	3/2904	1/2903	6.75
NCT02065791	Canagliflozin	Placebo	0.25 (0.03, 2.23)	1/2200	4/2197	7.20
NCT03036124	Dapagliflozin	Placebo	0.20 (0.01, 4.16)	0/2368	2/2368	3.75
NCT03036150	Dapagliflozin	Placebo	0.33 (0.01, 8.18)	0/2149	1/2149	3.38
NCT03057951	Empagliflozin	Placebo	1.50 (0.25, 8.95)	3/2996	2/2989	10.81
NCT03057977	Empagliflozin	Placebo	2.00 (0.18, 22.04)	2/1863	1/1863	6.00
NCT03242252	Sotagliflozin	Placebo	1.48 (0.06, 36.28)	1/527	0/260	3.38
NCT03315143	Sotagliflozin	Placebo	1.00 (0.06, 15.97)	1/5291	1/5286	4.50
NCT03521934	Sotagliflozin	Placebo	0.34 (0.01, 8.25)	0/605	1/611	3.38
NCT03619213	Dapagliflozin	Placebo	0.50 (0.09, 2.73)	2/3126	4/3127	12.01
Overall (I-squared = 0.0%, p = 0.915)			0.94 (0.52, 1.69)	24/40982	24/36508	100.00

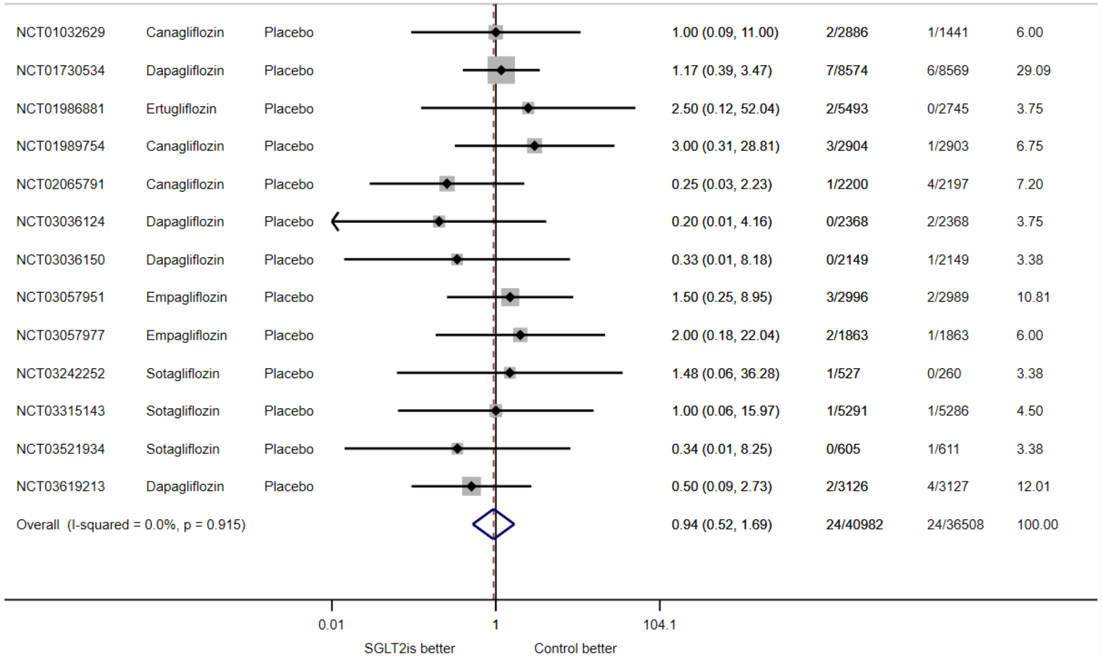

System 17-Nervous System Disorders-Figure S19 Meta-analysis of SGLT2is and Cerebral haemorrhage

Trial	SGLT2is	Control	Risk Ratio (95% CI)	Events, Treatment	Events, Control	% Weight
NCT01032629	Canagliflozin	Placebo	0.67 (0.15, 2.97)	4/2886	3/1441	3.19
NCT01131676	Empagliflozin	Placebo	1.28 (0.54, 3.06)	18/4687	7/2333	9.39
NCT01164501	Empagliflozin	Placebo	2.29 (0.09, 55.92)	1/419	0/319	0.70
NCT01730534	Dapagliflozin	Placebo	0.75 (0.44, 1.27)	24/8574	32/8569	25.54
NCT01986881	Ertugliflozin	Placebo	1.90 (0.71, 5.08)	19/5493	5/2745	7.36
NCT01989754	Canagliflozin	Placebo	0.80 (0.21, 2.98)	4/2904	5/2903	4.13
NCT02065791	Canagliflozin	Placebo	0.86 (0.29, 2.54)	6/2200	7/2197	6.02
NCT03036124	Dapagliflozin	Placebo	0.73 (0.29, 1.80)	8/2368	11/2368	8.63
NCT03036150	Dapagliflozin	Placebo	2.67 (0.71, 10.04)	8/2149	3/2149	4.06
NCT03057951	Empagliflozin	Placebo	1.40 (0.62, 3.14)	14/2996	10/2989	10.87
NCT03057977	Empagliflozin	Placebo	2.00 (0.37, 10.91)	4/1863	2/1863	2.48
NCT03315143	Sotagliflozin	Placebo	0.67 (0.19, 2.36)	4/5291	6/5286	4.46
NCT03521934	Sotagliflozin	Placebo	0.34 (0.01, 8.25)	0/605	1/611	0.70
NCT03594110	Empagliflozin	Placebo	0.67 (0.11, 3.99)	2/3304	3/3305	2.23
NCT03619213	Dapagliflozin	Placebo	1.00 (0.43, 2.30)	11/3126	11/3127	10.25
Overall (I-squared = 0.0%, p = 0.848)			1.00 (0.77, 1.31)	127/48865	106/42205	100.00

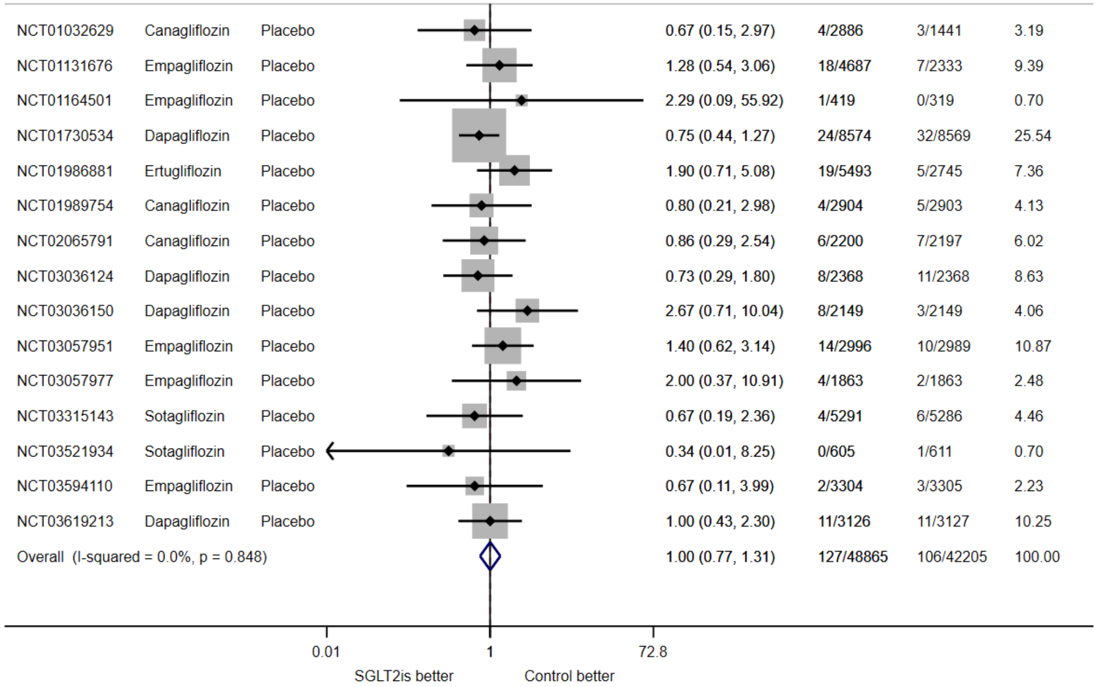

System 17-Nervous System Disorders-Figure S20 Meta-analysis of SGLT2is and Cerebral infarction

Trial	SGLT2is	Control	Risk Ratio (95% CI)	Events, Treatment	Events, Control	% Weight
NCT01131676	Empagliflozin	Placebo	1.99 (0.22, 17.80)	4/4687	1/2333	7.87
NCT01730534	Dapagliflozin	Placebo	0.67 (0.24, 1.87)	6/8574	9/8569	35.45
NCT01986881	Ertugliflozin	Placebo	0.50 (0.07, 3.55)	2/5493	2/2745	9.84
NCT01989754	Canagliflozin	Placebo	1.00 (0.06, 15.97)	1/2904	1/2903	4.92
NCT02065791	Canagliflozin	Placebo	1.00 (0.14, 7.08)	2/2200	2/2197	9.85
NCT03036124	Dapagliflozin	Placebo	1.00 (0.06, 15.98)	1/2368	1/2368	4.92
NCT03036150	Dapagliflozin	Placebo	0.20 (0.01, 4.16)	0/2149	2/2149	4.10
NCT03057951	Empagliflozin	Placebo	4.99 (0.24, 103.86)	2/2996	0/2989	4.10
NCT03057977	Empagliflozin	Placebo	3.00 (0.12, 73.59)	1/1863	0/1863	3.69
NCT03315143	Sotagliflozin	Placebo	0.25 (0.03, 2.23)	1/5291	4/5286	7.87
NCT03521934	Sotagliflozin	Placebo	3.03 (0.12, 74.22)	1/605	0/611	3.69
NCT03594110	Empagliflozin	Placebo	0.33 (0.01, 8.18)	0/3304	1/3305	3.69
Overall (I-squared = 0.0%, p = 0.874)			0.80 (0.43, 1.47)	21/42434	23/37318	100.00

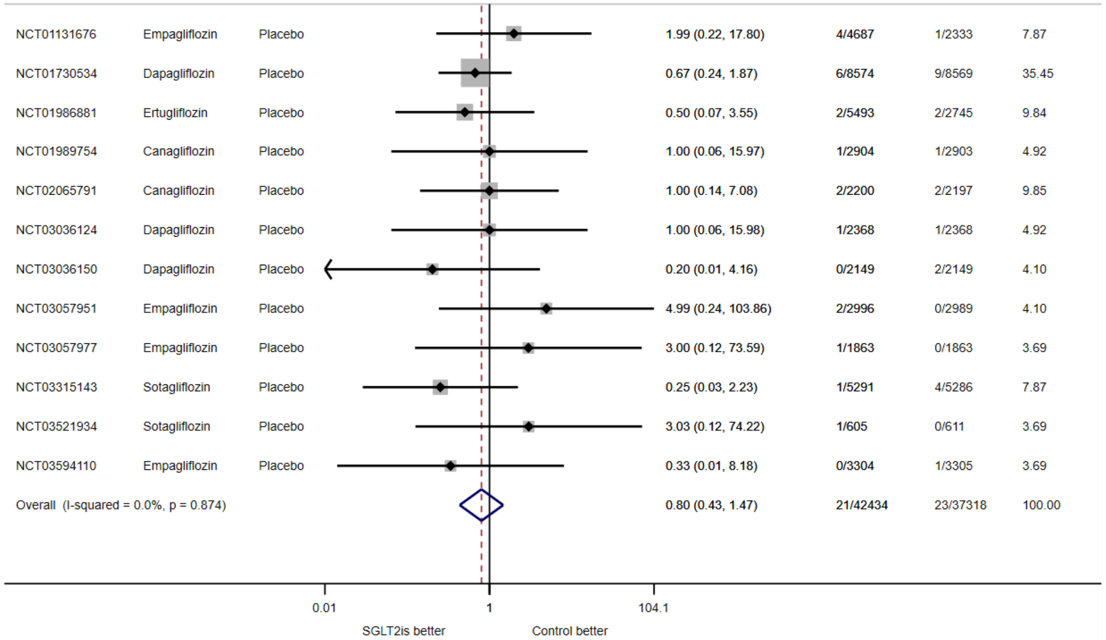

System 17-Nervous System Disorders-Figure S21 Meta-analysis of SGLT2is and Cerebral ischaemia

Trial	SGLT2is	Control	Risk Ratio (95% CI)	Events, Treatment	Events, Control	% Weight
NCT00528879	Dapagliflozin	Placebo	1.01 (0.04, 24.64)	1/409	0/137	0.23
NCT00673231	Dapagliflozin	Placebo	0.65 (0.06, 7.08)	2/610	1/197	0.41
NCT01031680	Dapagliflozin	Placebo	1.00 (0.06, 16.01)	1/460	1/462	0.31
NCT01032629	Canagliflozin	Placebo	1.30 (0.63, 2.68)	26/2886	10/1441	4.46
NCT01042977	Dapagliflozin	Placebo	0.20 (0.01, 4.16)	0/482	2/483	0.26
NCT01106651	Canagliflozin	Placebo	4.48 (0.24, 82.89)	4/477	0/237	0.28
NCT01106677	Canagliflozin	Sitagliptin	0.17 (0.01, 4.07)	0/735	1/366	0.23
NCT01131676	Empagliflozin	Placebo	1.33 (0.88, 2.01)	83/4687	31/2333	14.03
NCT01164501	Empagliflozin	Placebo	1.52 (0.14, 16.72)	2/419	1/319	0.41
NCT01177813	Empagliflozin	Sitagliptin or Placebo	3.03 (0.12, 74.27)	1/447	0/452	0.23
NCT01730534	Dapagliflozin	Placebo	1.16 (0.87, 1.54)	102/8574	88/8569	29.27
NCT01986881	Ertugliflozin	Placebo	1.18 (0.72, 1.94)	52/5493	22/2745	9.55
NCT01989754	Canagliflozin	Placebo	0.41 (0.22, 0.74)	15/2904	37/2903	6.59
NCT02065791	Canagliflozin	Placebo	0.83 (0.51, 1.35)	30/2200	36/2197	10.18
NCT03036124	Dapagliflozin	Placebo	1.40 (0.44, 4.40)	7/2368	5/2368	1.79
NCT03036150	Dapagliflozin	Placebo	1.25 (0.49, 3.16)	10/2149	8/2149	2.73
NCT03057951	Empagliflozin	Placebo	1.05 (0.57, 1.93)	21/2996	20/2989	6.32
NCT03057977	Empagliflozin	Placebo	1.42 (0.68, 2.96)	17/1863	12/1863	4.34
NCT03242252	Sotagliflozin	Placebo	1.97 (0.22, 17.57)	4/527	1/260	0.49
NCT03315143	Sotagliflozin	Placebo	0.81 (0.39, 1.69)	13/5291	16/5286	4.41
NCT03521934	Sotagliflozin	Placebo	1.68 (0.40, 7.01)	5/605	3/611	1.16
NCT03619213	Dapagliflozin	Placebo	1.67 (0.40, 6.97)	5/3126	3/3127	1.15
NCT04157751	Empagliflozin	Placebo	1.02 (0.21, 4.98)	3/260	3/264	0.93
NCT04350593	Dapagliflozin	Placebo	0.33 (0.01, 8.21)	0/613	1/616	0.23
Overall (I-squared = 0.0%, p = 0.637)			1.07 (0.92, 1.24)	404/50581	302/42374	100.00

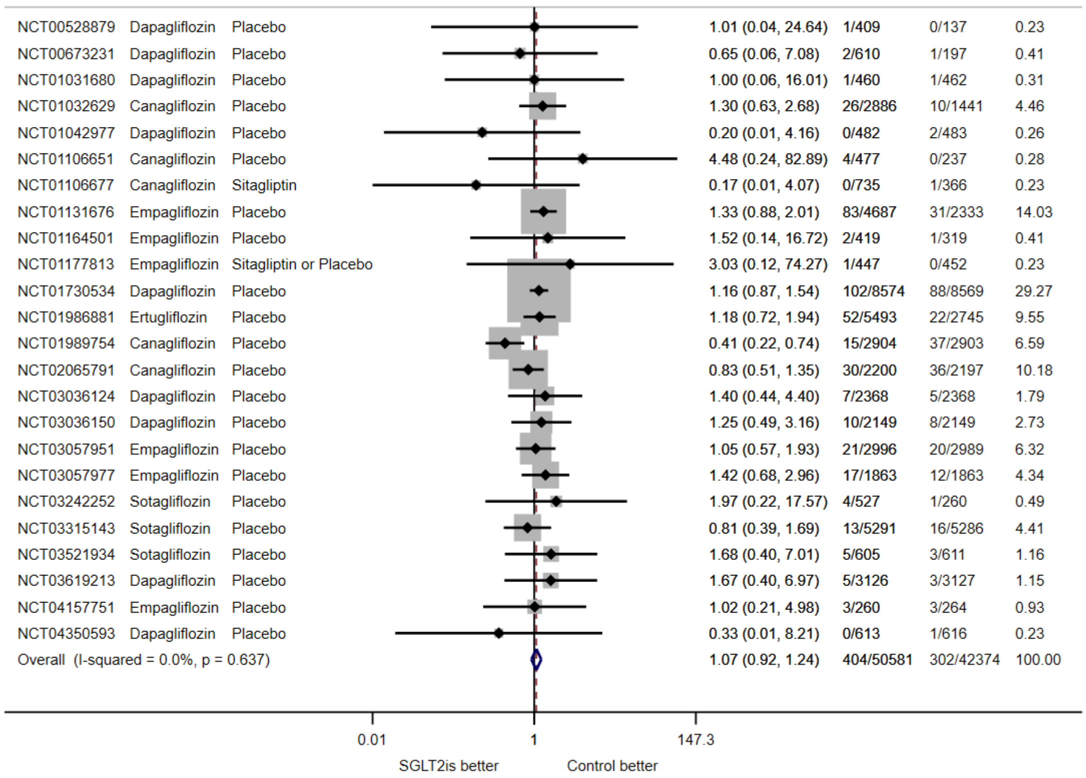

System 17-Nervous System Disorders-Figure S22 Meta-analysis of SGLT2is and Cerebrovascular accident

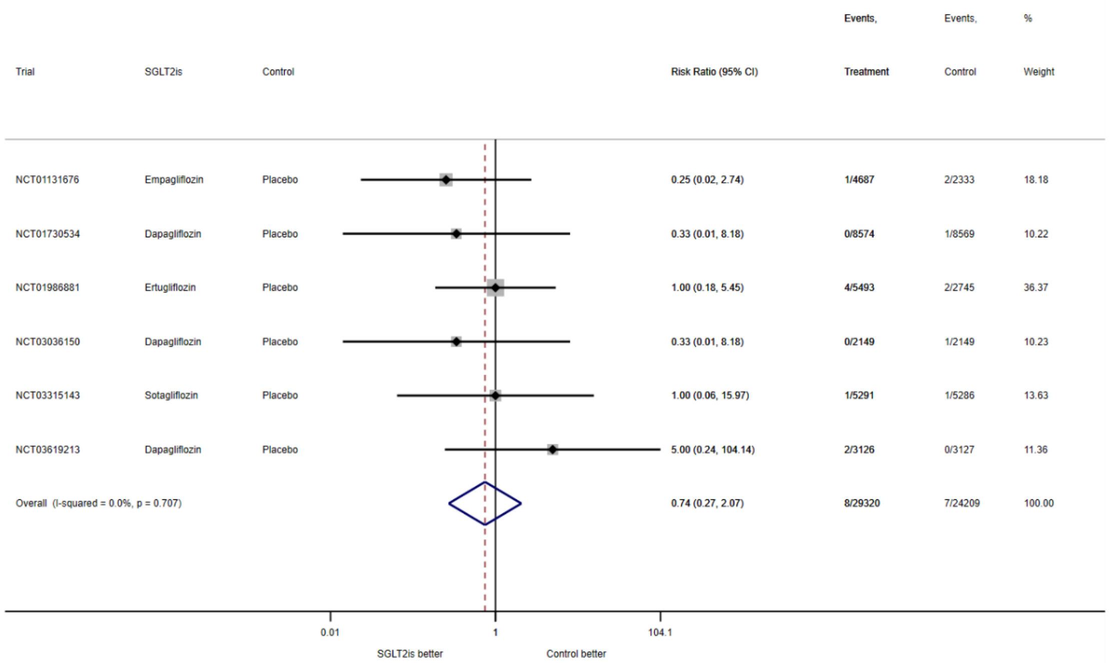

System 17-Nervous System Disorders-Figure S23 Meta-analysis of SGLT2is and Cerebrovascular disorder

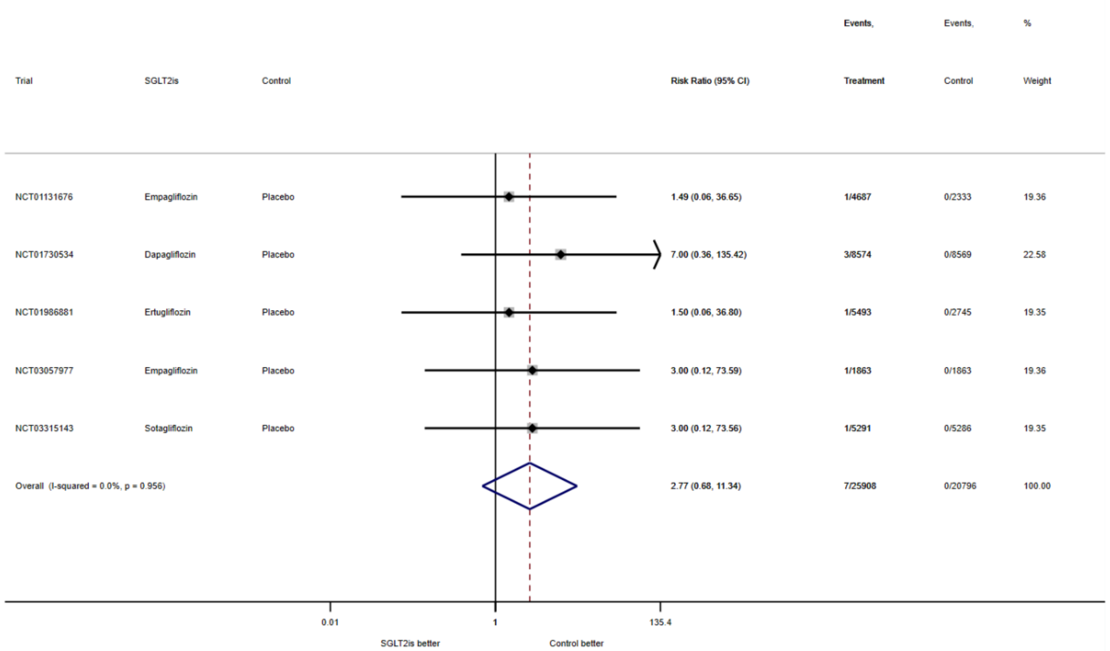

System 17-Nervous System Disorders-Figure S24 Meta-analysis of SGLT2is and Cerebrovascular insufficiency

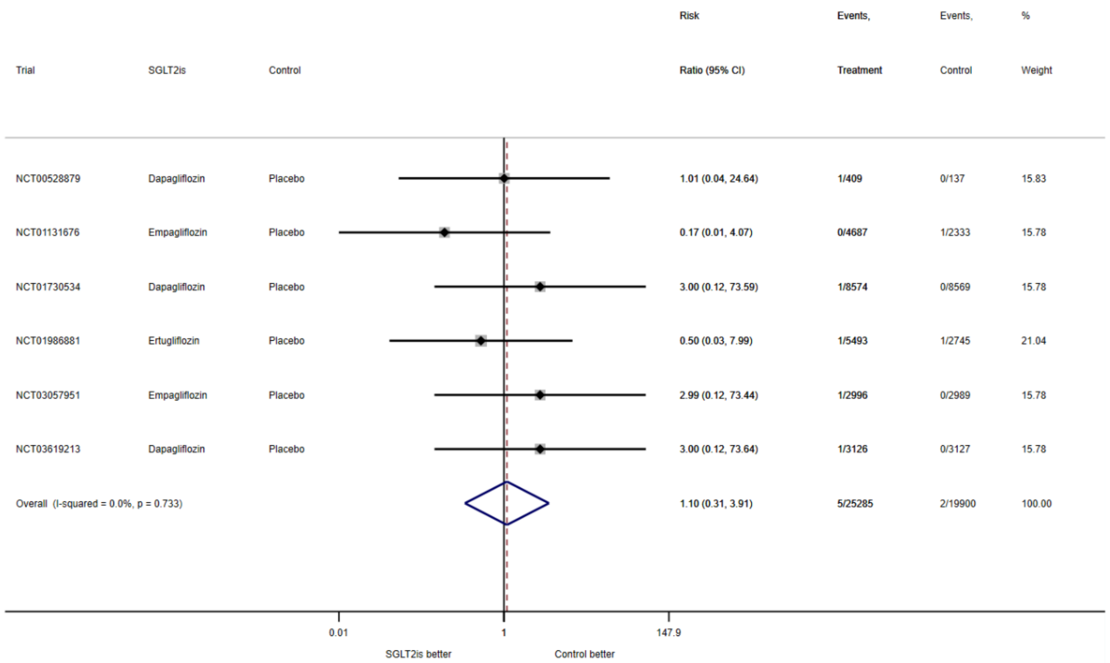

System 17-Nervous System Disorders-Figure S25 Meta-analysis of SGLT2is and Cervical cord compression

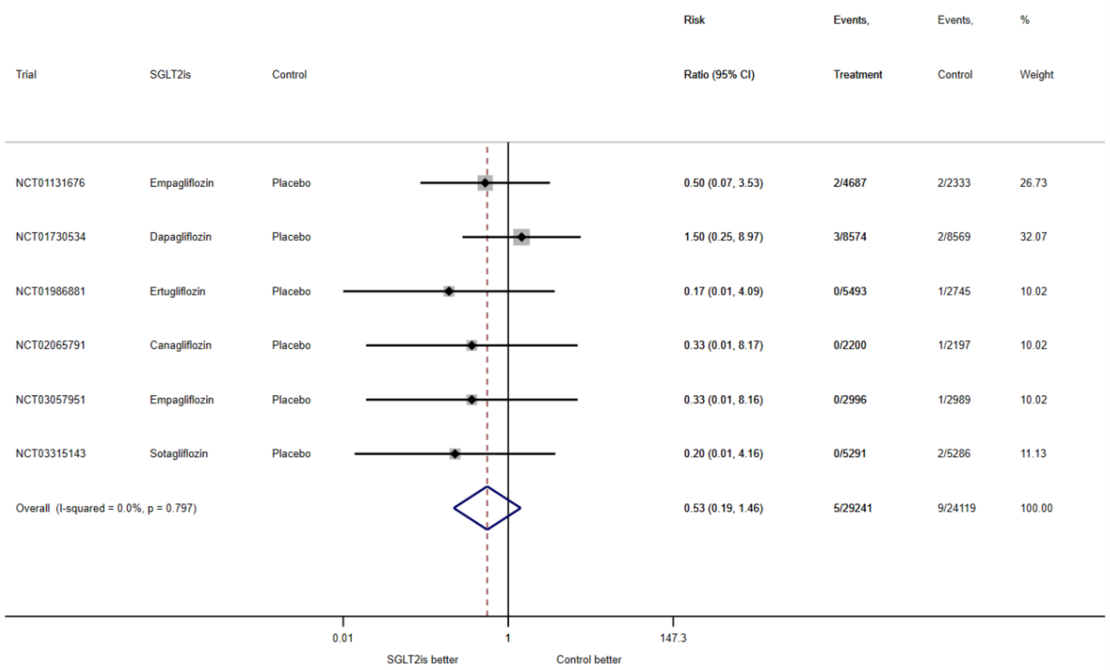

System 17-Nervous System Disorders-Figure S26 Meta-analysis of SGLT2is and Cervical radiculopathy

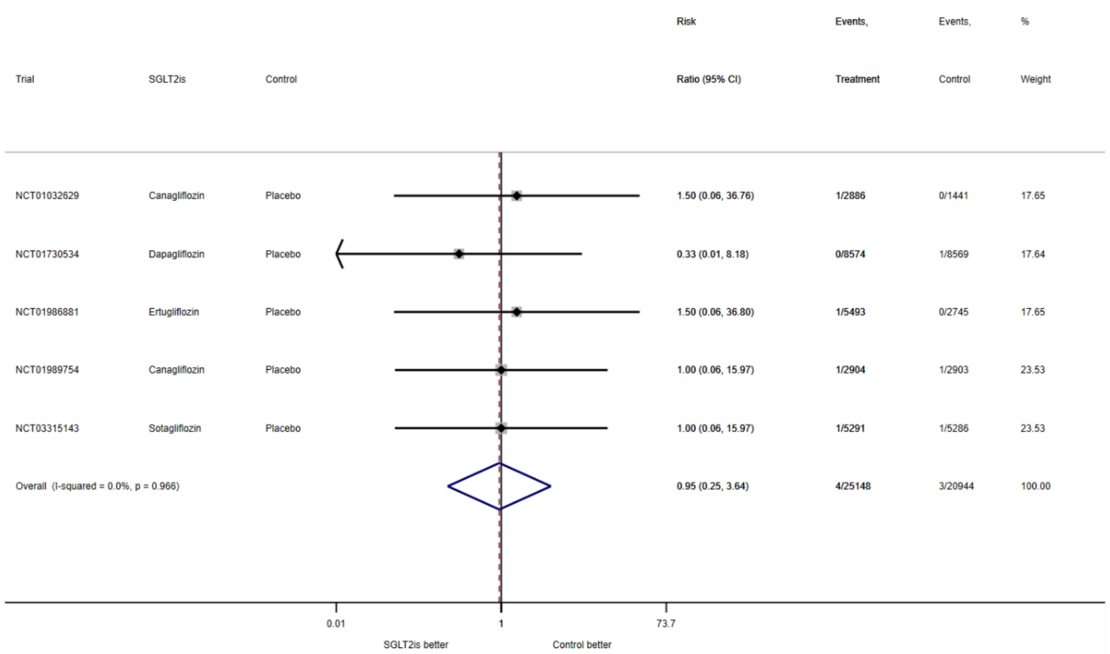

System 17-Nervous System Disorders-Figure S27 Meta-analysis of SGLT2is and Cervicobrachial syndrome

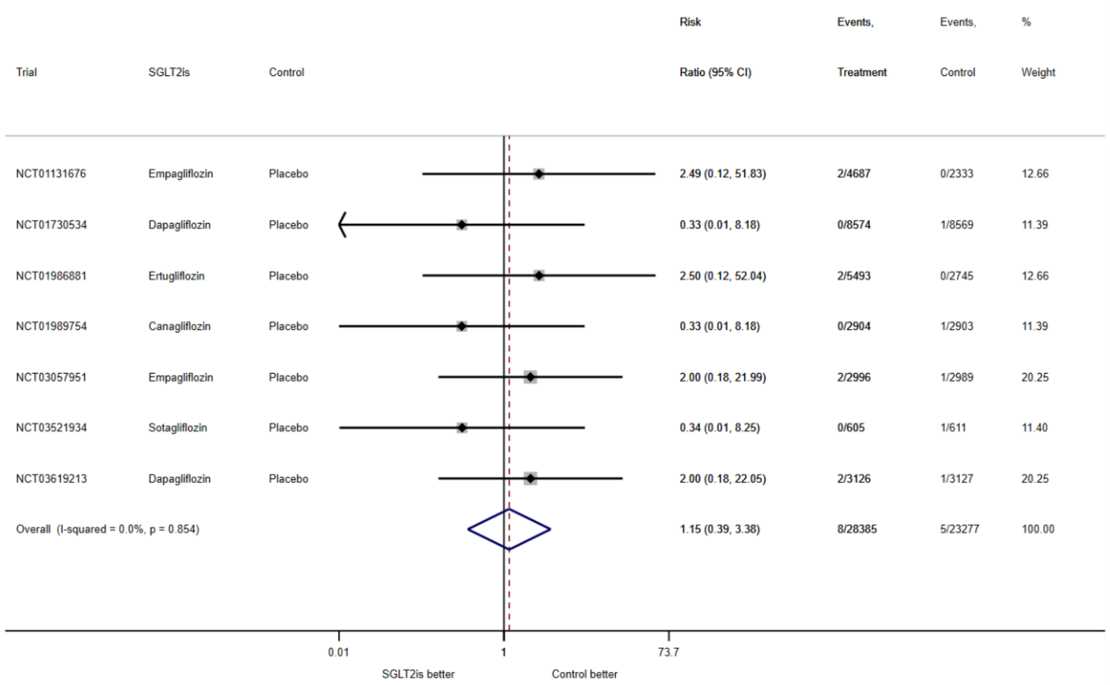

System 17-Nervous System Disorders-Figure S28 Meta-analysis of SGLT2is and Cognitive disorder

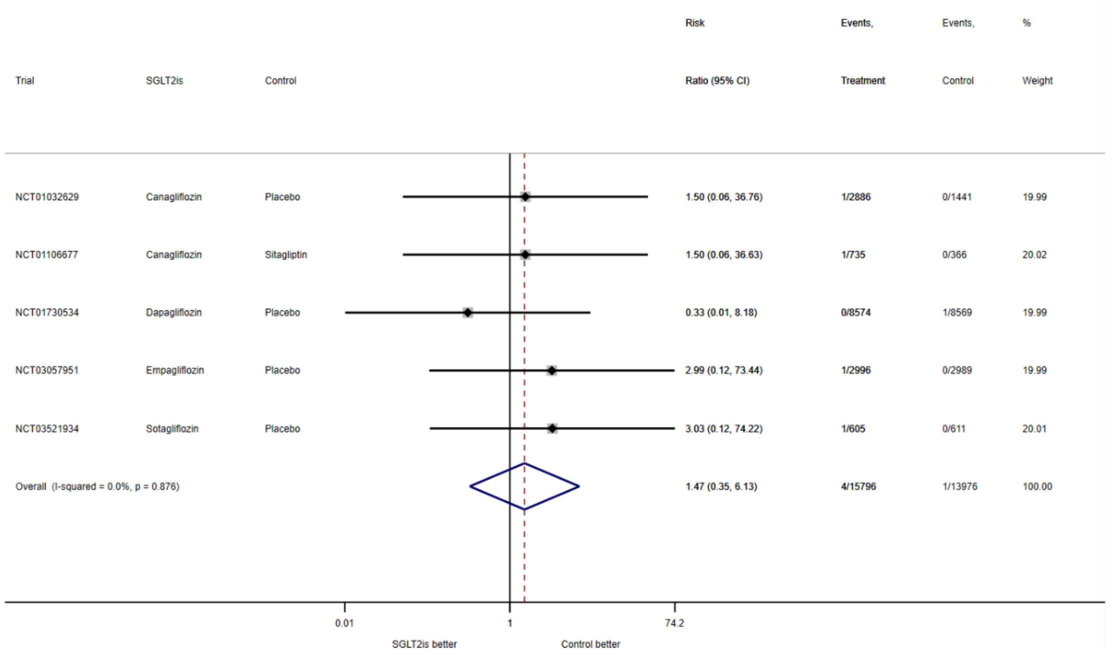

System 17-Nervous System Disorders-Figure S29 Meta-analysis of SGLT2is and Coma

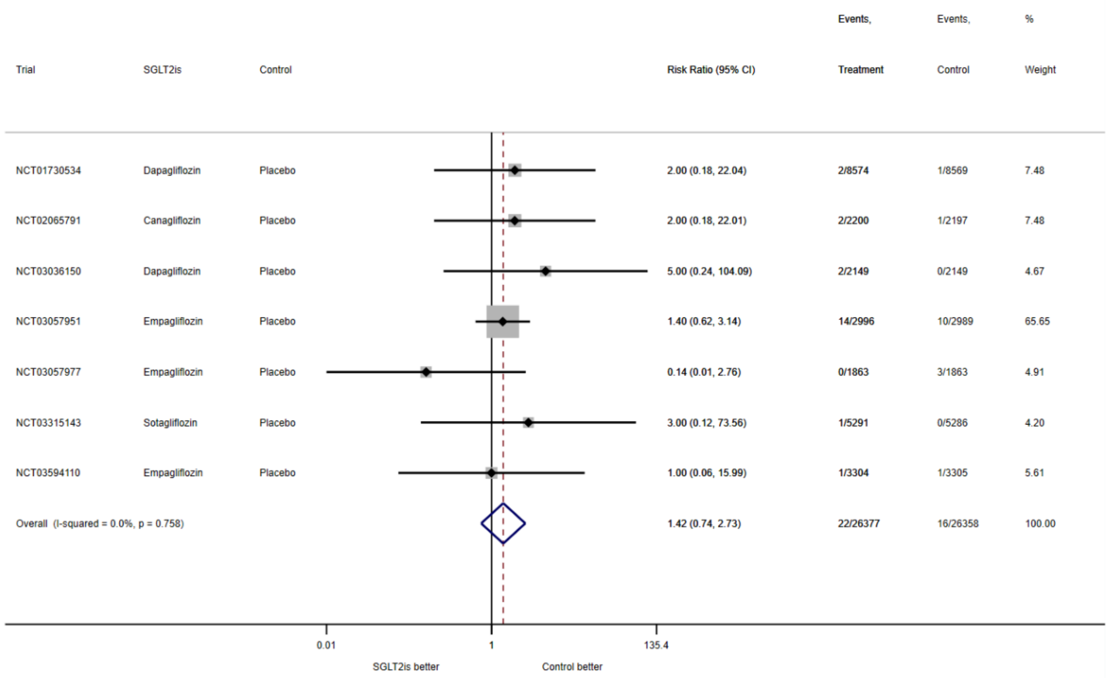

System 17-Nervous System Disorders-Figure S30 Meta-analysis of SGLT2is and Dementia

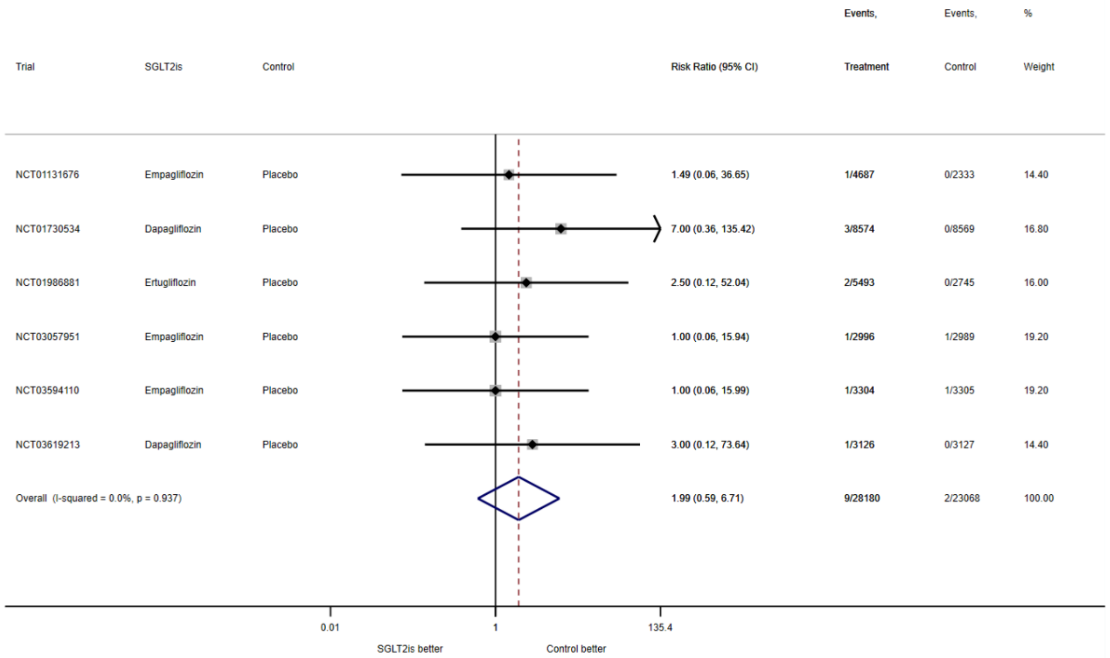

System 17-Nervous System Disorders-Figure S31 Meta-analysis of SGLT2is and Dementia alzheimer's type

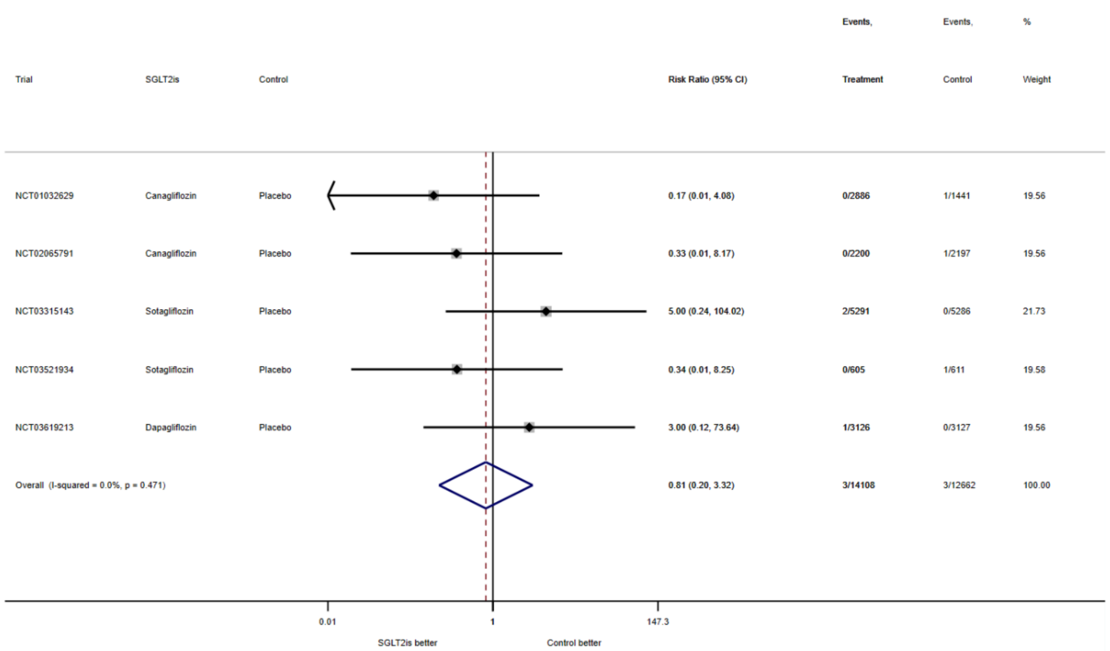

System 17-Nervous System Disorders-Figure S32 Meta-analysis of SGLT2is and Depressed level of consciousness

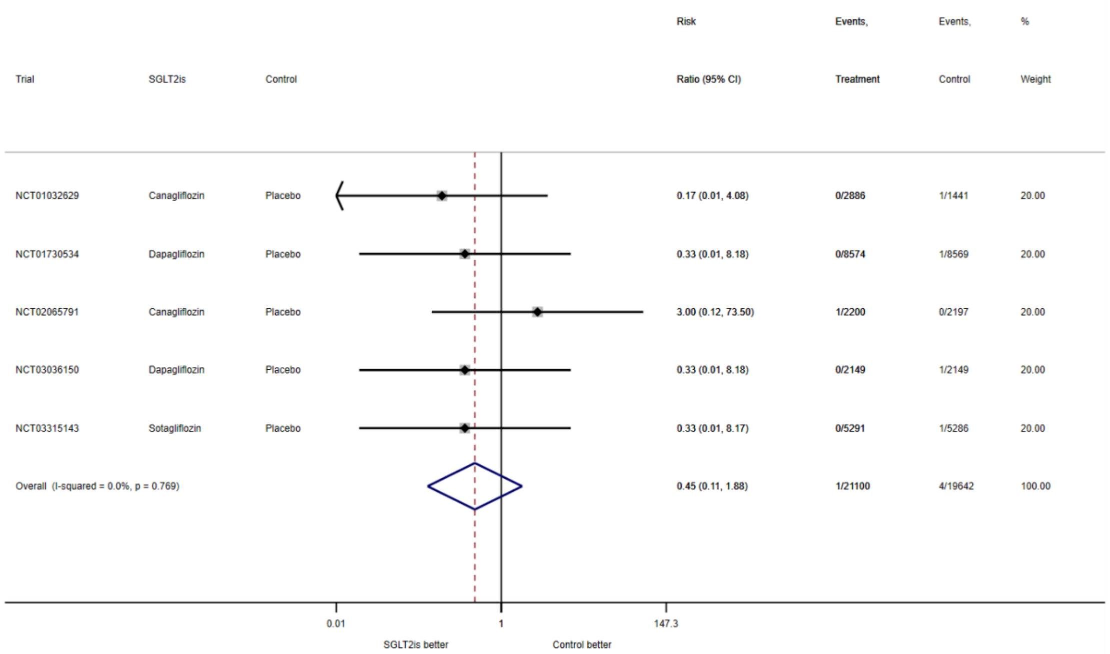

System 17-Nervous System Disorders-Figure S33 Meta-analysis of SGLT2is and Diabetic hyperosmolar coma

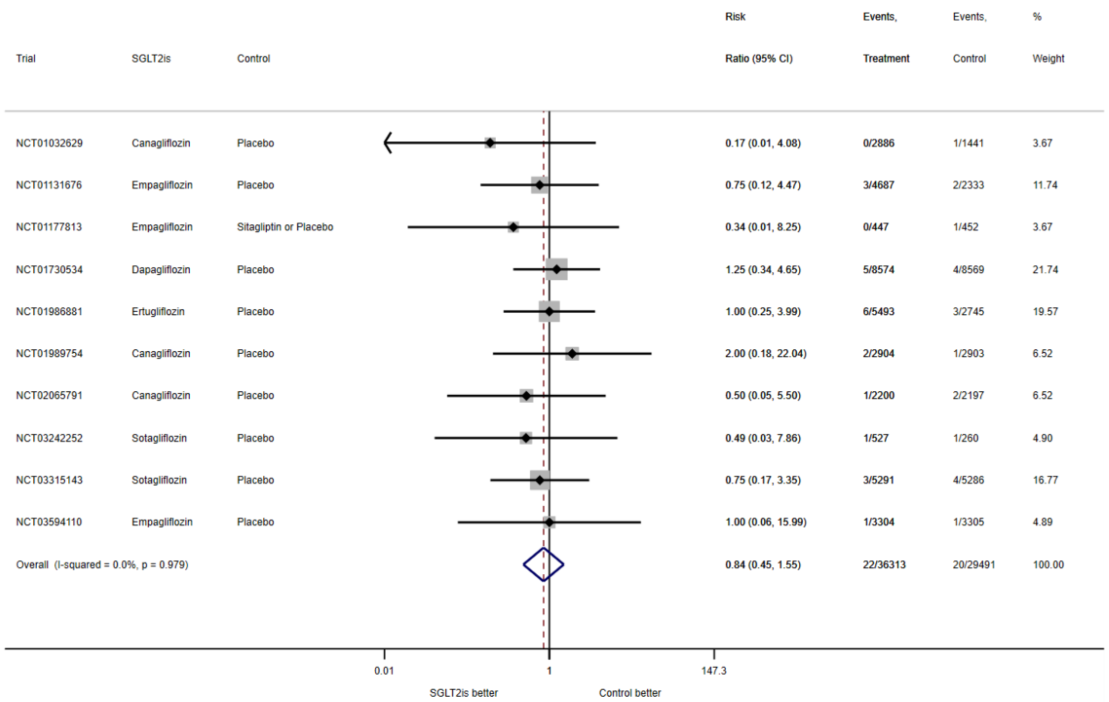

System 17-Nervous System Disorders-Figure S34 Meta-analysis of SGLT2is and Diabetic neuropathy

Trial	SGLT2is	Control	Risk Ratio (95% CI)	Events, Treatment	Events, Control	% Weight
NCT01032629	Canagliflozin	Placebo	2.50 (0.12, 51.99)	2/2886	0/1441	2.02
NCT01042977	Dapagliflozin	Placebo	1.00 (0.06, 15.97)	1/482	1/483	2.43
NCT01131676	Empagliflozin	Placebo	1.29 (0.46, 3.63)	13/4687	5/2333	17.57
NCT01164501	Empagliflozin	Placebo	0.25 (0.01, 6.21)	0/419	1/319	1.82
NCT01730534	Dapagliflozin	Placebo	1.55 (0.67, 3.59)	14/8574	9/8569	26.63
NCT01986881	Ertugliflozin	Placebo	0.50 (0.13, 2.00)	4/5493	4/2745	9.72
NCT02065791	Canagliflozin	Placebo	1.00 (0.06, 15.96)	1/2200	1/2197	2.43
NCT03036124	Dapagliflozin	Placebo	0.14 (0.01, 2.76)	0/2368	3/2368	2.12
NCT03057951	Empagliflozin	Placebo	0.50 (0.12, 1.99)	3/2996	6/2989	9.72
NCT03057977	Empagliflozin	Placebo	3.00 (0.12, 73.59)	1/1863	0/1863	1.82
NCT03315143	Sotagliflozin	Placebo	0.33 (0.03, 3.20)	1/5291	3/5286	3.64
NCT03521934	Sotagliflozin	Placebo	3.03 (0.12, 74.22)	1/605	0/611	1.82
NCT03594110	Empagliflozin	Placebo	0.60 (0.14, 2.51)	3/3304	5/3305	9.11
NCT03619213	Dapagliflozin	Placebo	1.67 (0.40, 6.97)	5/3126	3/3127	9.11
Overall (I-squared = 0.0%, p = 0.792)			0.98 (0.64, 1.51)	49/44294	41/37636	100.00

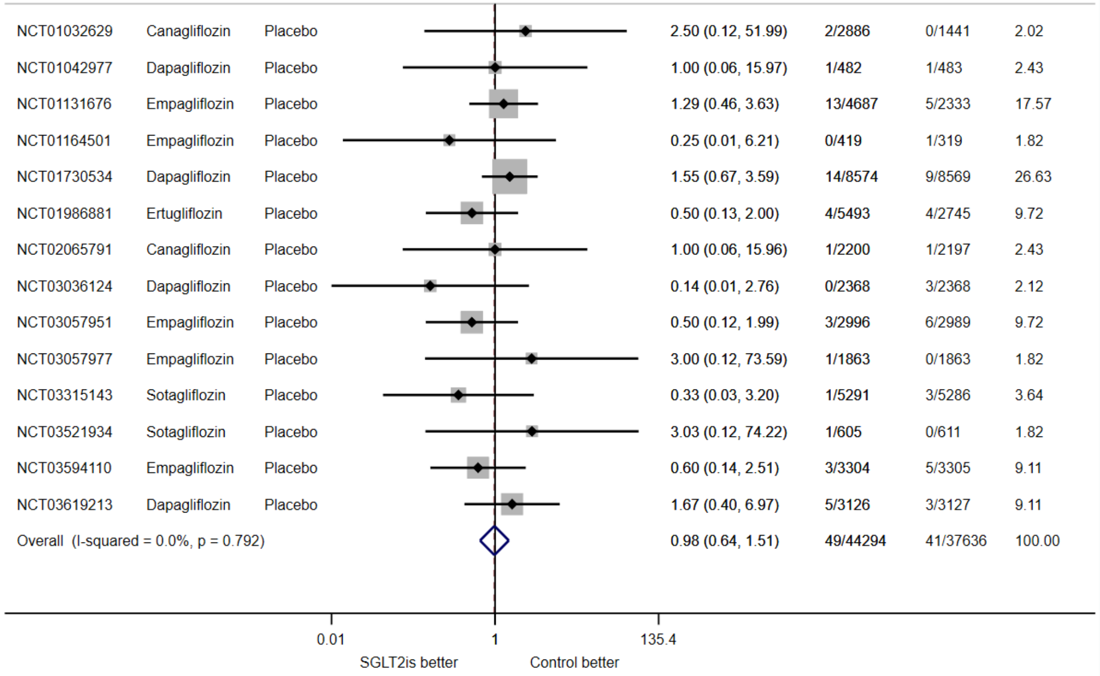

System 17-Nervous System Disorders-Figure S35 Meta-analysis of SGLT2is and Dizziness

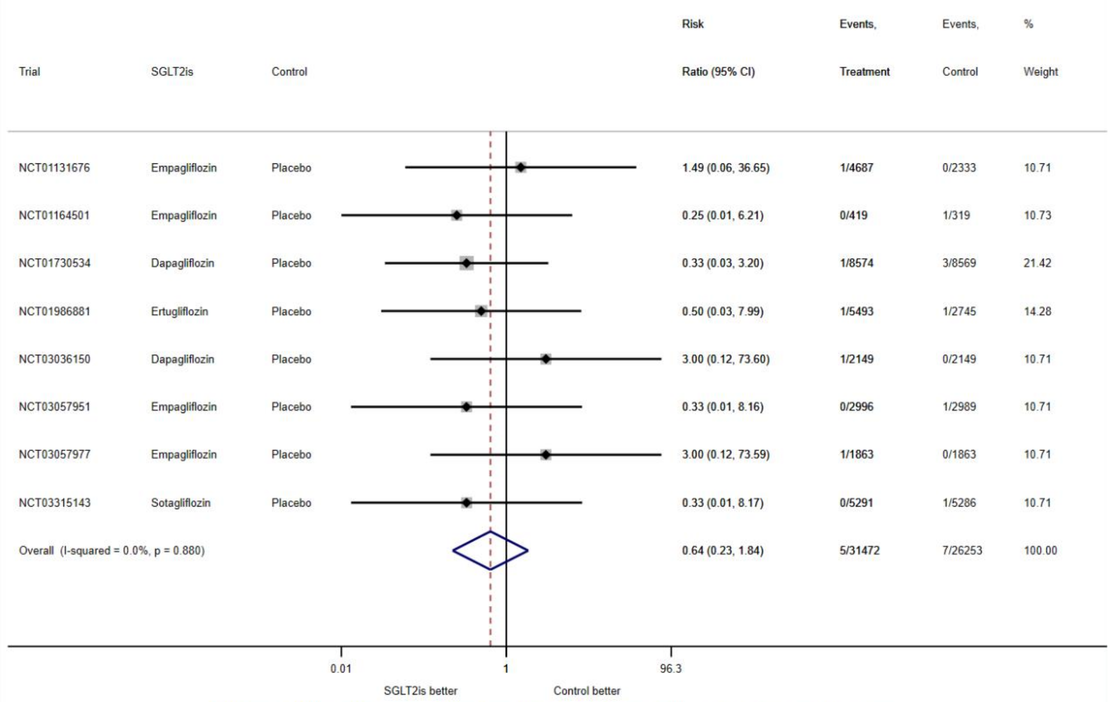

System 17-Nervous System Disorders-Figure S36 Meta-analysis of SGLT2is and Dysarthria

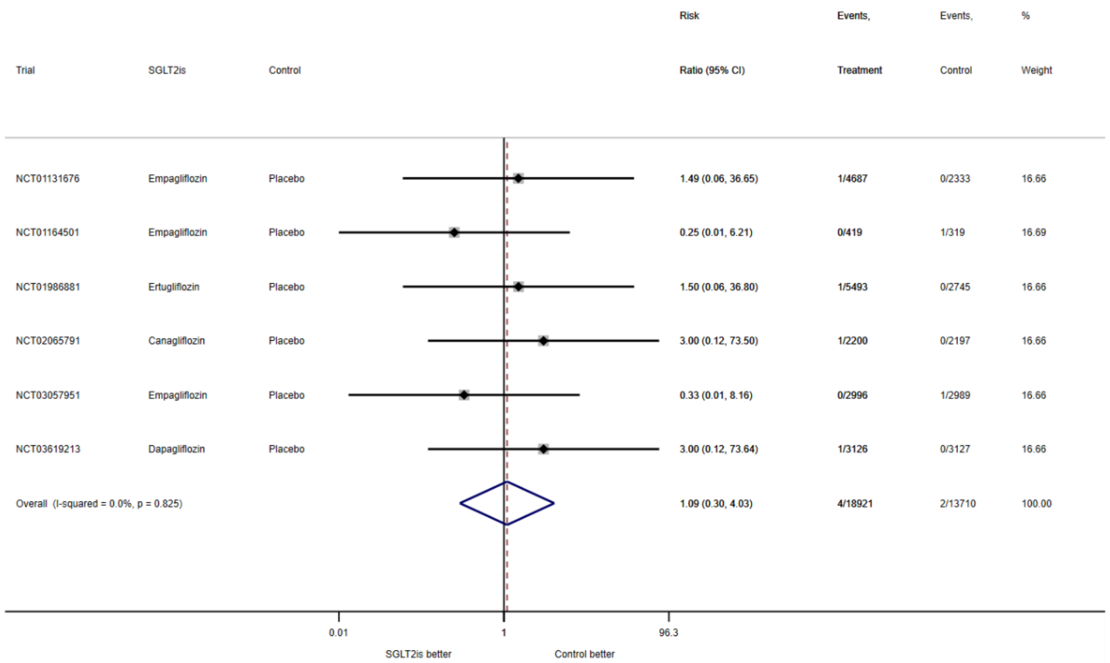

System 17-Nervous System Disorders-Figure S37 Meta-analysis of SGLT2is and Embolic cerebral infarction

Trial	SGLT2is	Control	Risk Ratio (95% CI)	Events, Treatment	Events, Control	% Weight
NCT01131676	Empagliflozin	Placebo	0.17 (0.02, 1.59)	1/4687	3/2333	8.12
NCT01730534	Dapagliflozin	Placebo	0.50 (0.09, 2.73)	2/8574	4/8569	14.42
NCT01986881	Ertugliflozin	Placebo	3.50 (0.18, 67.71)	3/5493	0/2745	4.73
NCT01989754	Canagliflozin	Placebo	0.33 (0.01, 8.18)	0/2904	1/2903	4.06
NCT02065791	Canagliflozin	Placebo	0.33 (0.01, 8.17)	0/2200	1/2197	4.06
NCT03036124	Dapagliflozin	Placebo	0.33 (0.03, 3.20)	1/2368	3/2368	8.12
NCT03036150	Dapagliflozin	Placebo	0.33 (0.01, 8.18)	0/2149	1/2149	4.06
NCT03057951	Empagliflozin	Placebo	3.99 (0.45, 35.68)	4/2996	1/2989	8.66
NCT03057977	Empagliflozin	Placebo	3.00 (0.12, 73.59)	1/1863	0/1863	4.06
NCT03242252	Sotagliflozin	Placebo	0.16 (0.01, 4.03)	0/527	1/260	4.06
NCT03521934	Sotagliflozin	Placebo	0.34 (0.01, 8.25)	0/605	1/611	4.06
NCT03619213	Dapagliflozin	Placebo	1.40 (0.44, 4.41)	7/3126	5/3127	31.60
Overall (I-squared = 0.0%, p = 0.586)			0.77 (0.41, 1.47)	19/37492	21/32114	100.00

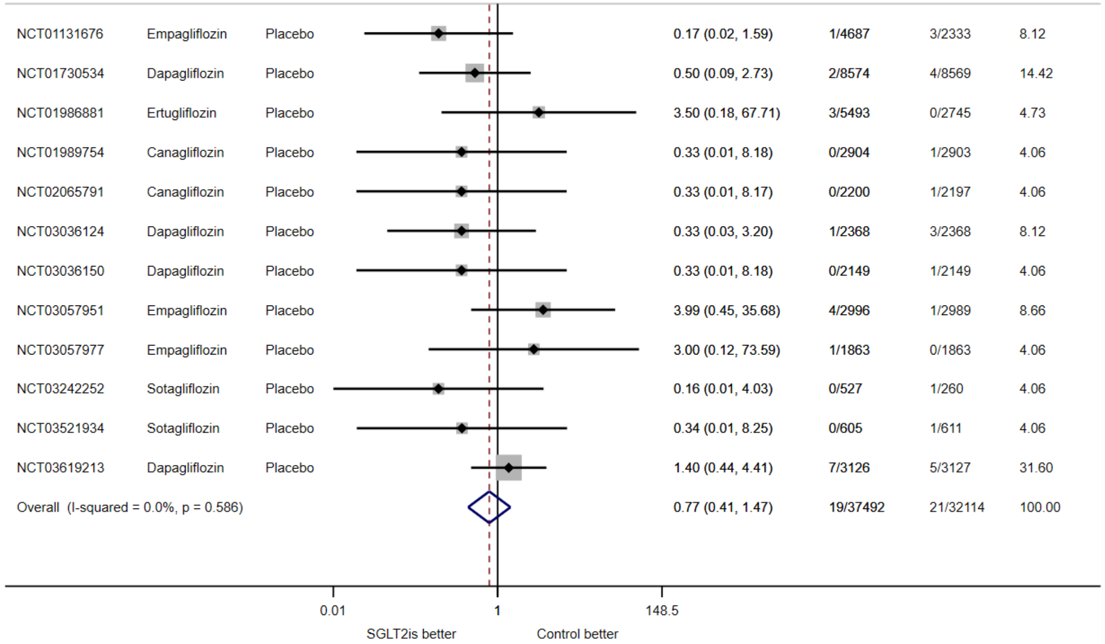

System 17-Nervous System Disorders-Figure S38 Meta-analysis of SGLT2is and Embolic stroke

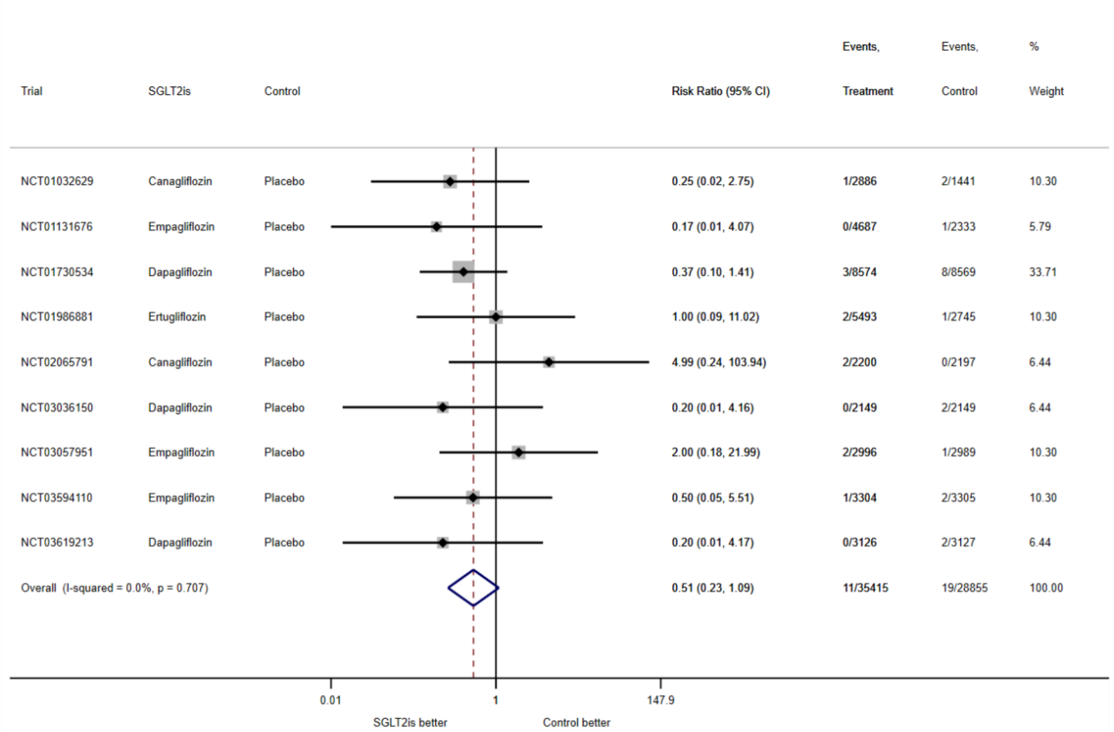

System 17-Nervous System Disorders-Figure S39 Meta-analysis of SGLT2is and Encephalopathy

Trial	SGLT2is	Control	Risk Ratio (95% CI)	Events, Treatment	Events, Control	% Weight
NCT01032629	Canagliflozin	Placebo	0.50 (0.03, 7.98)	1/2886	1/1441	5.34
NCT01131676	Empagliflozin	Placebo	4.48 (0.24, 83.19)	4/4687	0/2333	4.81
NCT01730534	Dapagliflozin	Placebo	0.60 (0.14, 2.51)	3/8574	5/8569	20.03
NCT01986881	Ertugliflozin	Placebo	2.50 (0.12, 52.04)	2/5493	0/2745	4.45
NCT01989754	Canagliflozin	Placebo	2.00 (0.18, 22.04)	2/2904	1/2903	7.12
NCT02065791	Canagliflozin	Placebo	0.33 (0.01, 8.17)	0/2200	1/2197	4.01
NCT03036124	Dapagliflozin	Placebo	0.33 (0.01, 8.18)	0/2368	1/2368	4.01
NCT03036150	Dapagliflozin	Placebo	0.25 (0.03, 2.23)	1/2149	4/2149	8.55
NCT03057951	Empagliflozin	Placebo	0.33 (0.03, 3.20)	1/2996	3/2989	8.01
NCT03057977	Empagliflozin	Placebo	0.67 (0.11, 3.99)	2/1863	3/1863	12.83
NCT03315143	Sotagliflozin	Placebo	0.33 (0.01, 8.17)	0/5291	1/5286	4.01
NCT03594110	Empagliflozin	Placebo	3.00 (0.12, 73.64)	1/3304	0/3305	4.01
NCT03619213	Dapagliflozin	Placebo	0.67 (0.11, 3.99)	2/3126	3/3127	12.83
Overall (I-squared = 0.0%, p = 0.928)			0.69 (0.36, 1.30)	19/47841	23/41275	100.00

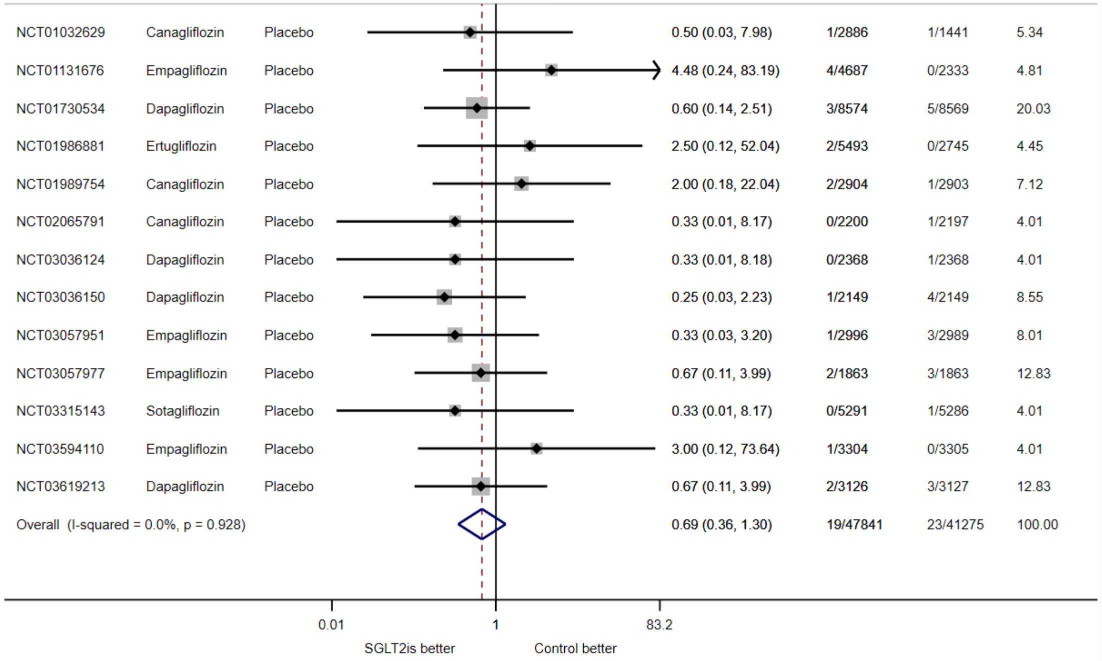

System 17-Nervous System Disorders-Figure S40 Meta-analysis of SGLT2is and Epilepsy

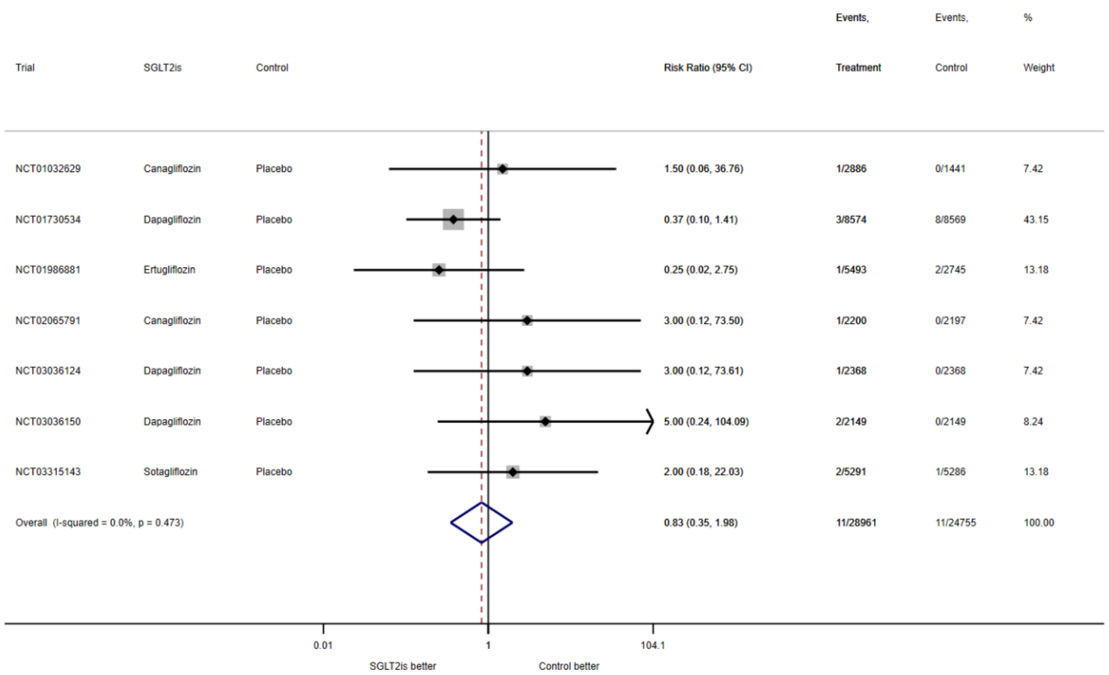

System 17-Nervous System Disorders-Figure S41 Meta-analysis of SGLT2is and Facial paralysis

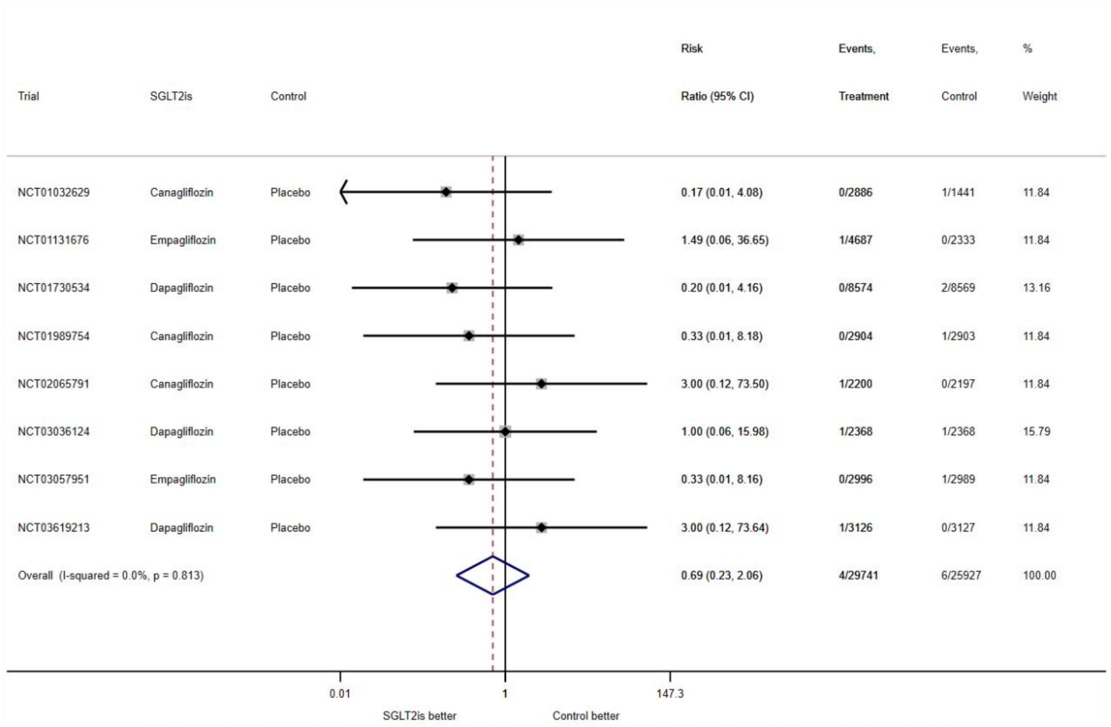

System 17-Nervous System Disorders-Figure S42 Meta-analysis of SGLT2is and Generalised tonic-clonic seizure

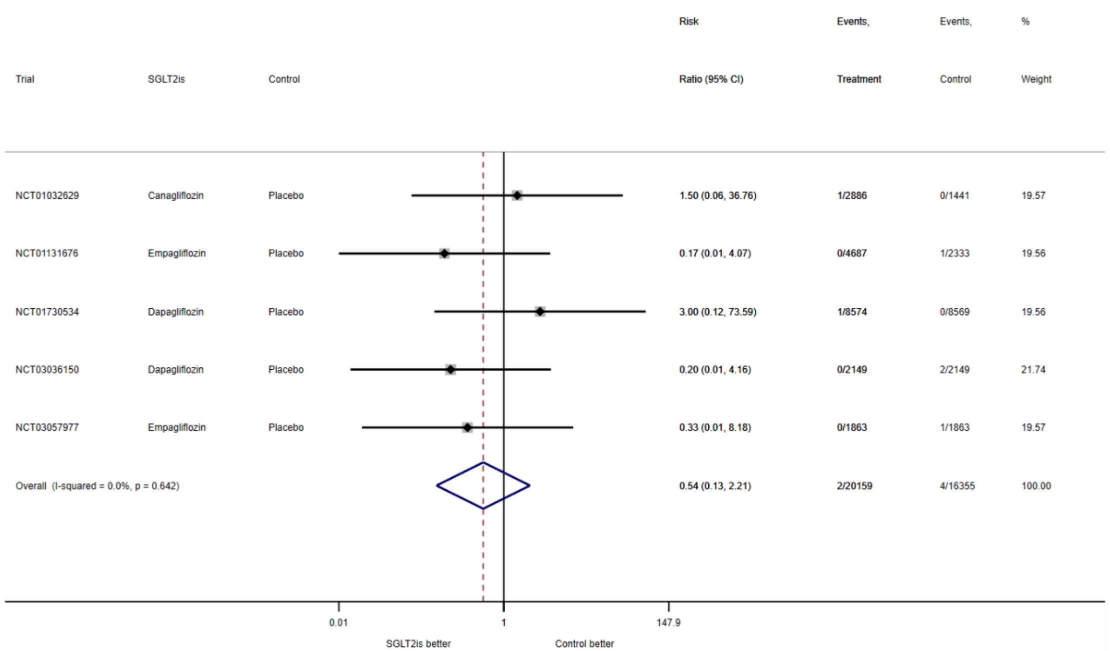

System 17-Nervous System Disorders-Figure S43 Meta-analysis of SGLT2is and Guillain-barre syndrome

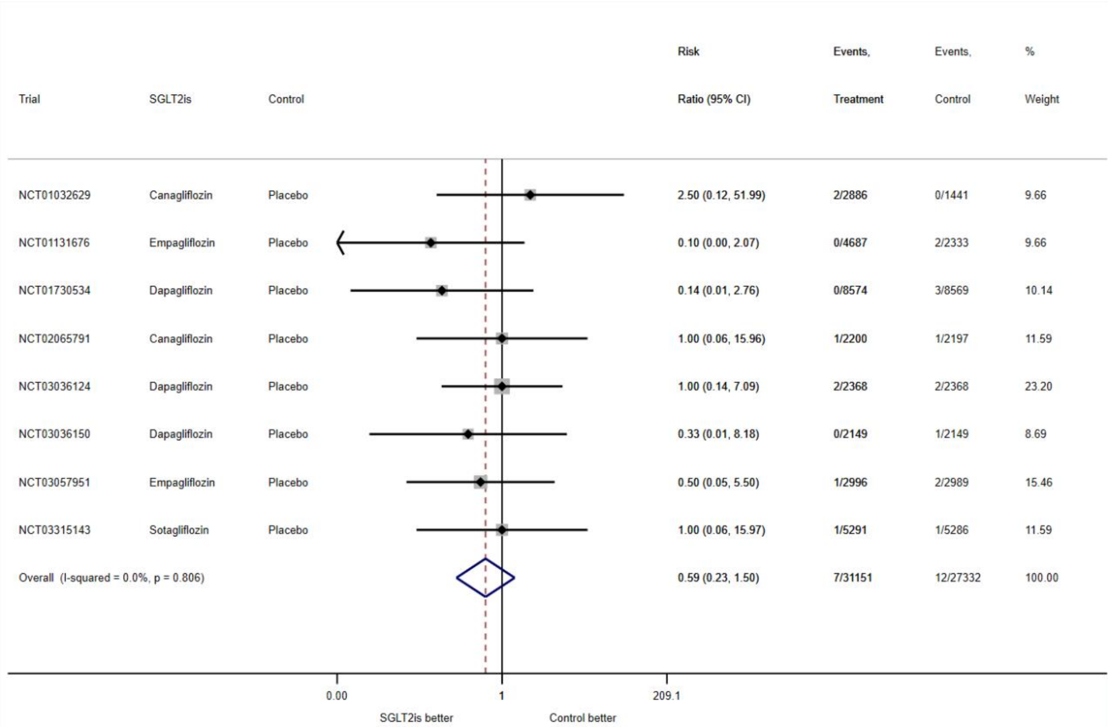

System 17-Nervous System Disorders-Figure S44 Meta-analysis of SGLT2is and Haemorrhage intracranial

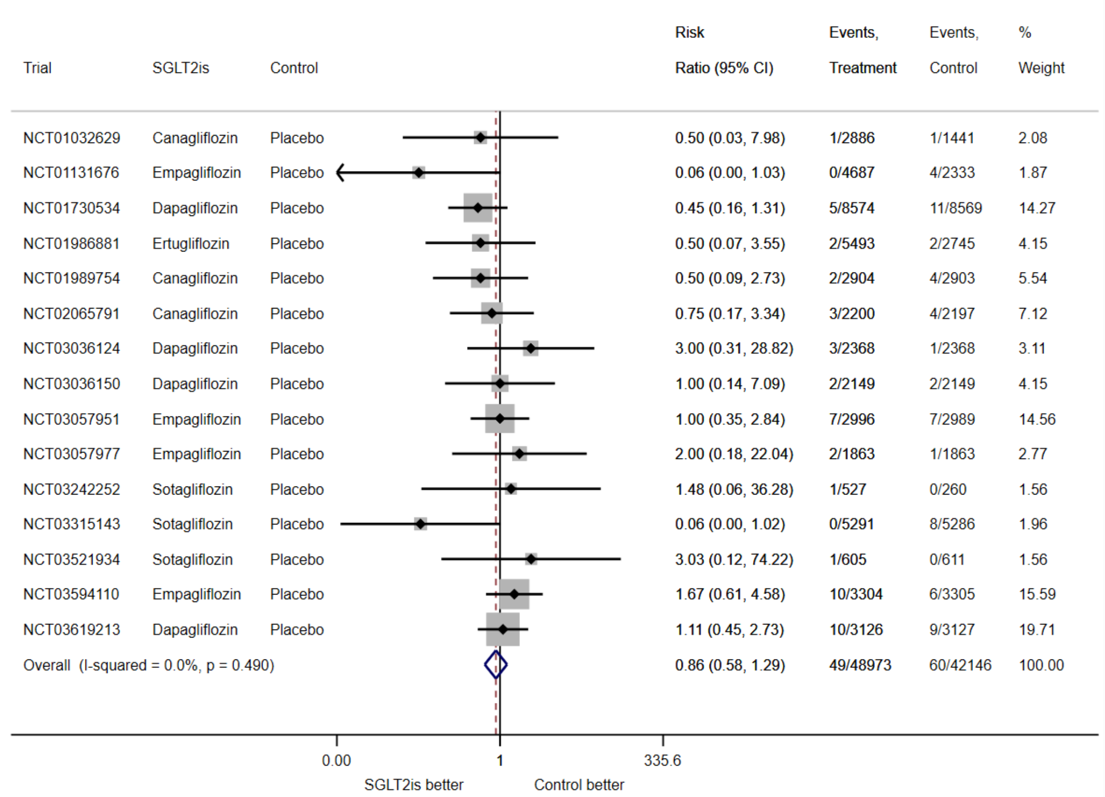

System 17-Nervous System Disorders-Figure S45 Meta-analysis of SGLT2is and Haemorrhagic stroke

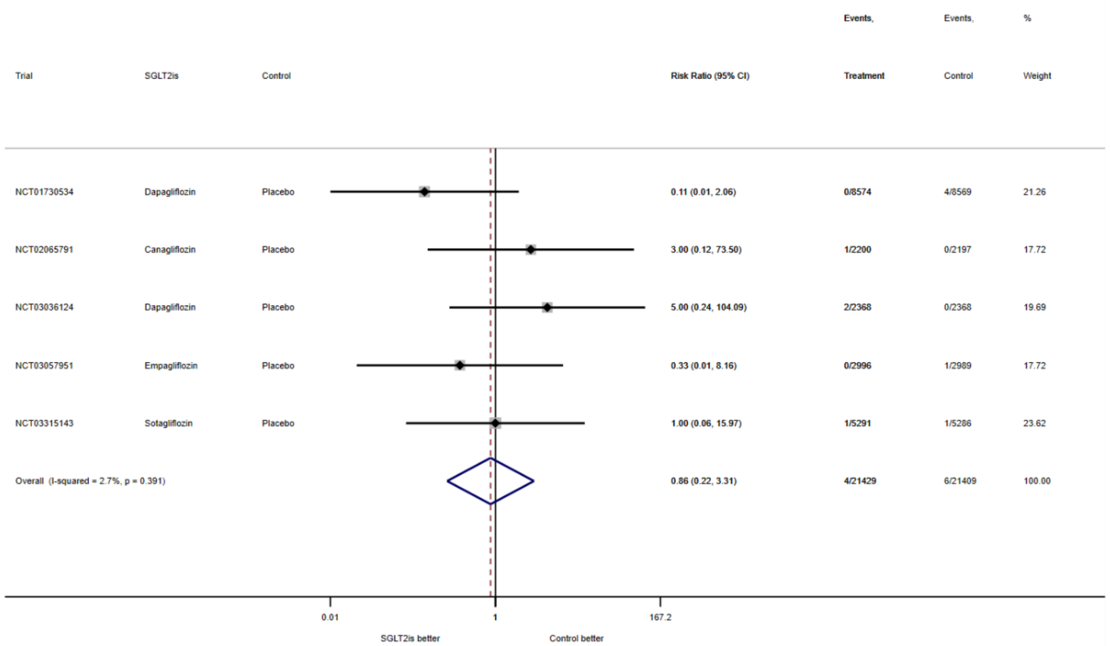

System 17-Nervous System Disorders-Figure S46 Meta-analysis of SGLT2is and Haemorrhagic transformation stroke

Trial	SGLT2is	Control	Risk Ratio (95% CI)	Events, Treatment	Events, Control	% Weight
NCT01032629	Canagliflozin	Placebo	1.00 (0.09, 11.00)	2/2886	1/1441	7.11
NCT01131676	Empagliflozin	Placebo	0.25 (0.02, 2.74)	1/4687	2/2333	7.11
NCT01730534	Dapagliflozin	Placebo	1.00 (0.20, 4.95)	3/8574	3/8569	16.00
NCT01986881	Ertugliflozin	Placebo	1.00 (0.18, 5.45)	4/5493	2/2745	14.22
NCT01989754	Canagliflozin	Placebo	2.00 (0.18, 22.04)	2/2904	1/2903	7.11
NCT02065791	Canagliflozin	Placebo	1.00 (0.06, 15.96)	1/2200	1/2197	5.33
NCT03036124	Dapagliflozin	Placebo	0.50 (0.05, 5.51)	1/2368	2/2368	7.11
NCT03036150	Dapagliflozin	Placebo	0.33 (0.01, 8.18)	0/2149	1/2149	4.00
NCT03057951	Empagliflozin	Placebo	2.99 (0.12, 73.44)	1/2996	0/2989	4.00
NCT03057977	Empagliflozin	Placebo	3.00 (0.12, 73.59)	1/1863	0/1863	4.00
NCT03315143	Sotagliflozin	Placebo	3.00 (0.31, 28.80)	3/5291	1/5286	8.00
NCT03594110	Empagliflozin	Placebo	3.00 (0.61, 14.86)	6/3304	2/3305	16.01
Overall (I-squared = 0.0%, p = 0.899)			1.23 (0.65, 2.34)	25/44715	16/38148	100.00

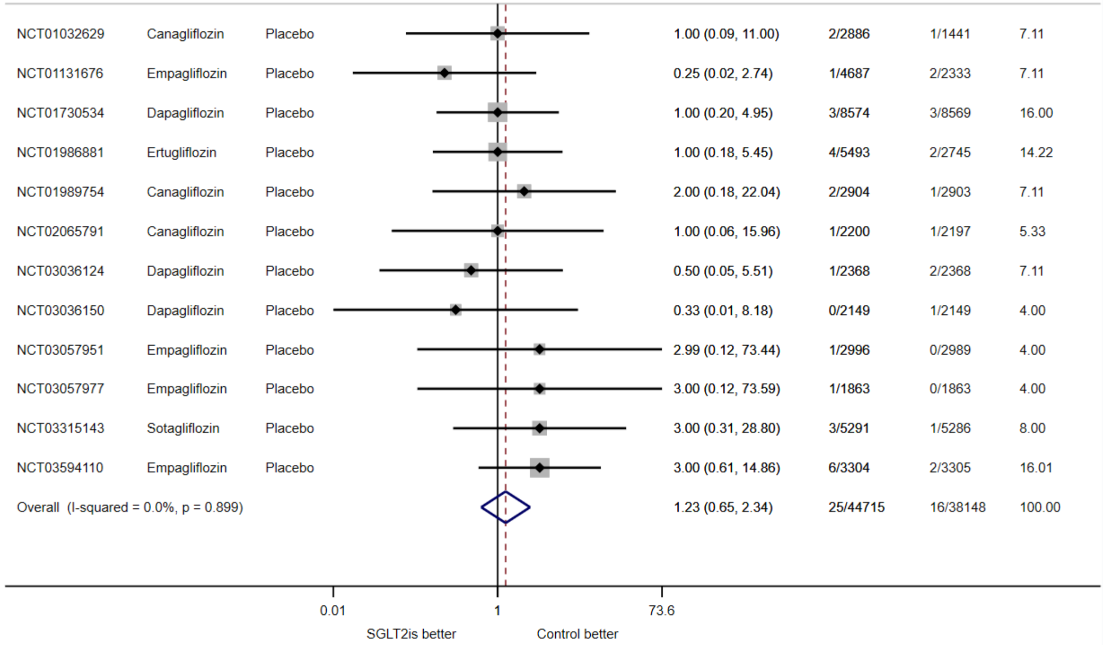

System 17-Nervous System Disorders-Figure S47 Meta-analysis of SGLT2is and Headache

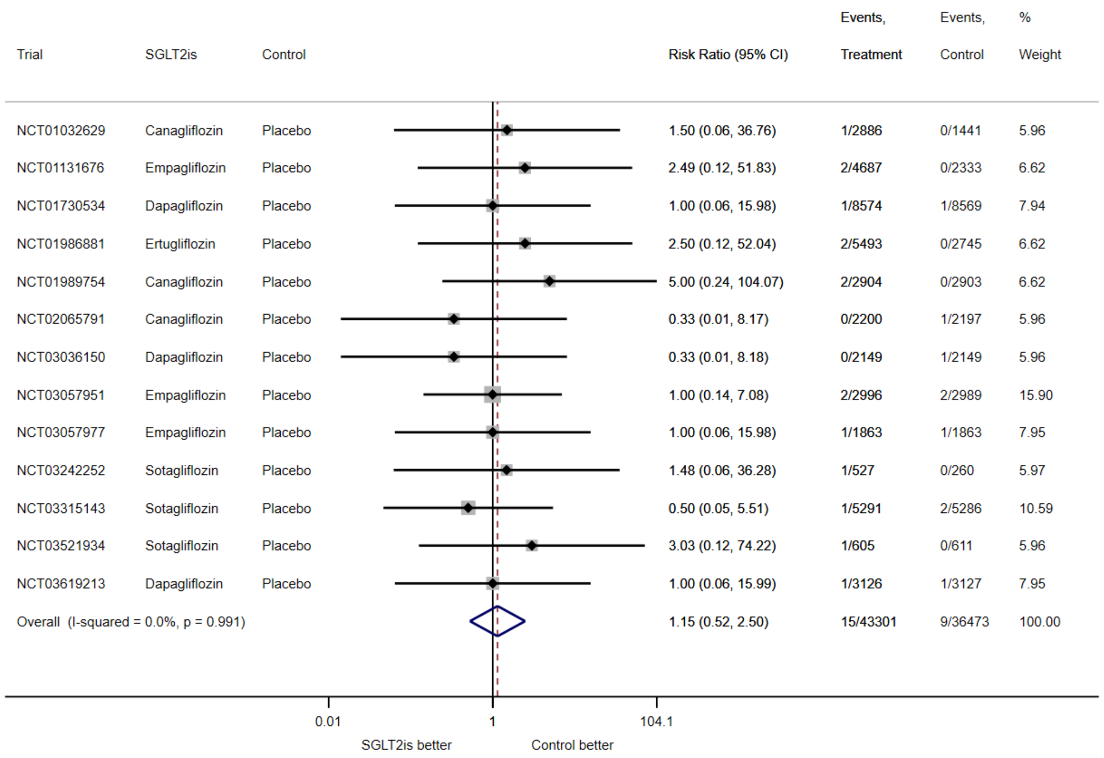

System 17-Nervous System Disorders-Figure S48 Meta-analysis of SGLT2is and Hemiparesis

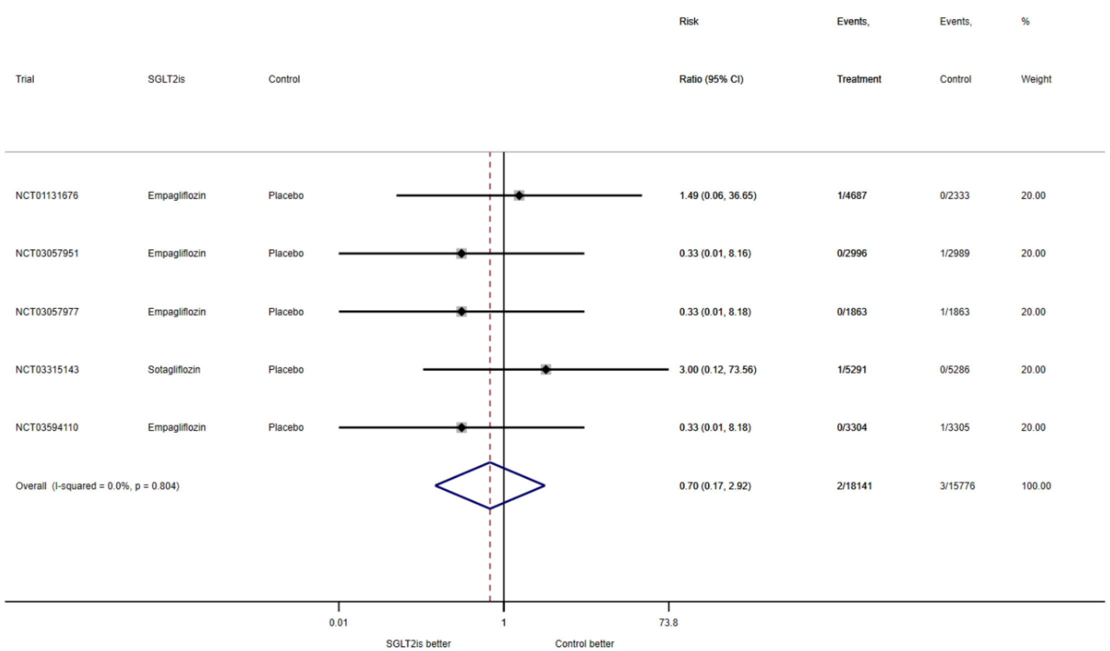

System 17-Nervous System Disorders-Figure S49 Meta-analysis of SGLT2is and Hemiplegia

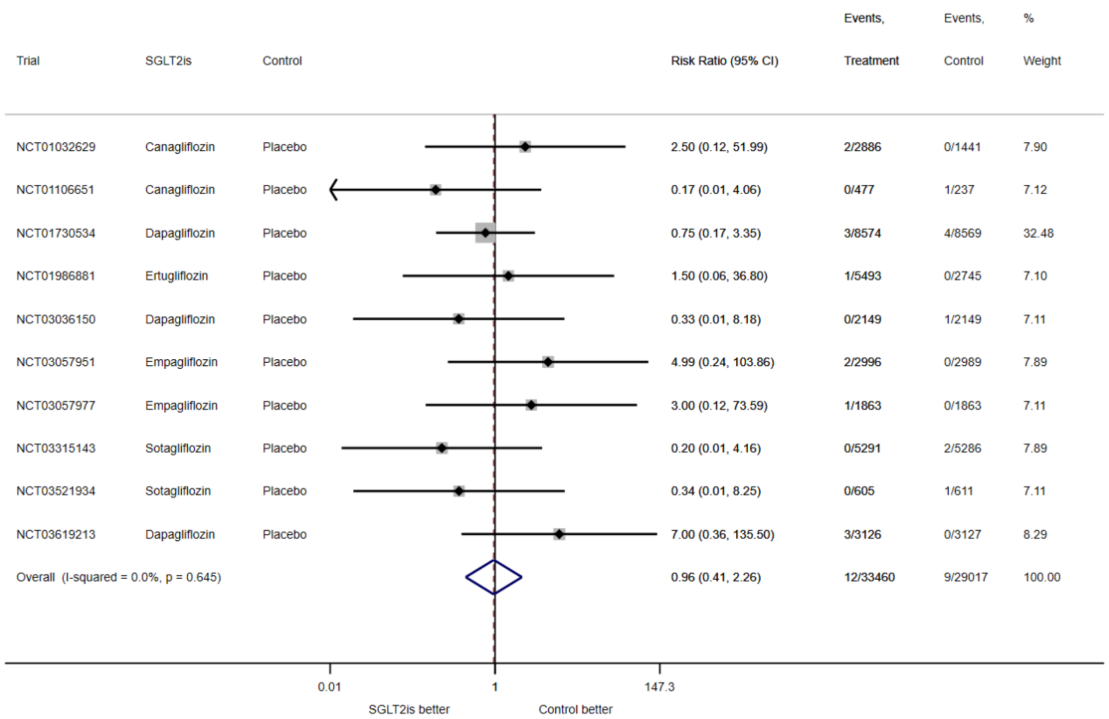

System 17-Nervous System Disorders-Figure S50 Meta-analysis of SGLT2is and Hepatic encephalopathy

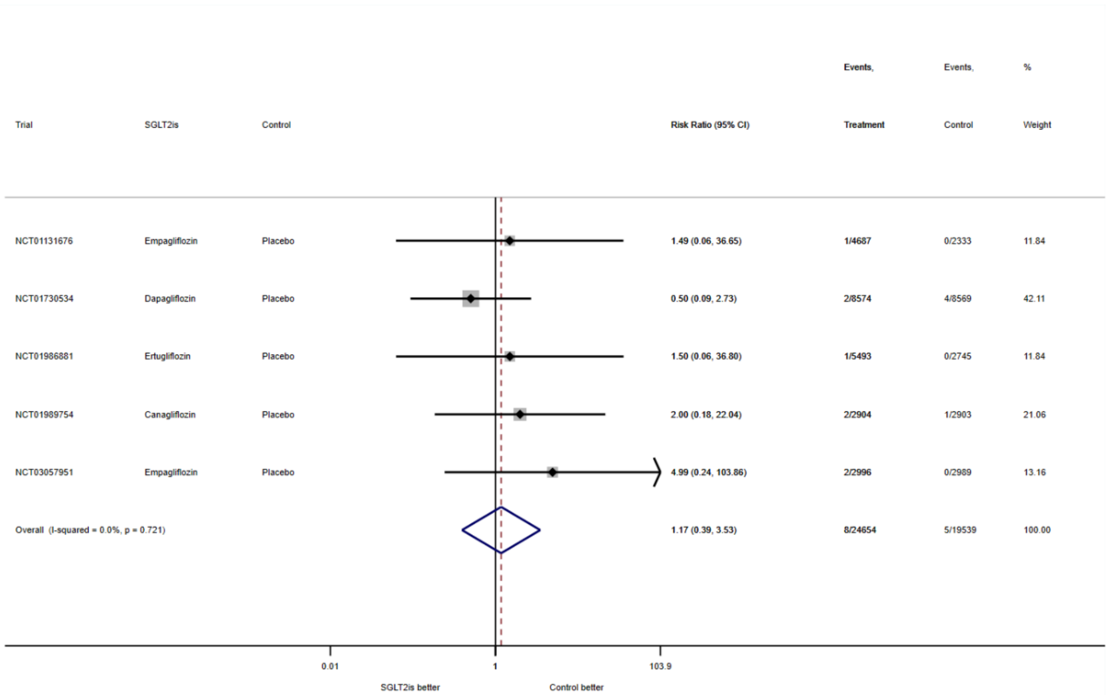

System 17-Nervous System Disorders-Figure S51 Meta-analysis of SGLT2is and Hydrocephalus
