# Supplementary material for: A comprehensive meta-analysis on safety outcomes reveals the novel potentials of SGLT2is, especially preventing respiratory diseases
Source: Front Endocrinol (Lausanne). 2024 Apr 29;15:1376446. doi: 10.3389/fendo.2024.1376446 (PMC11089104; doi:10.3389/fendo.2024.1376446)
Supplement: Supplementary file 5 [file DataSheet_5.pdf]

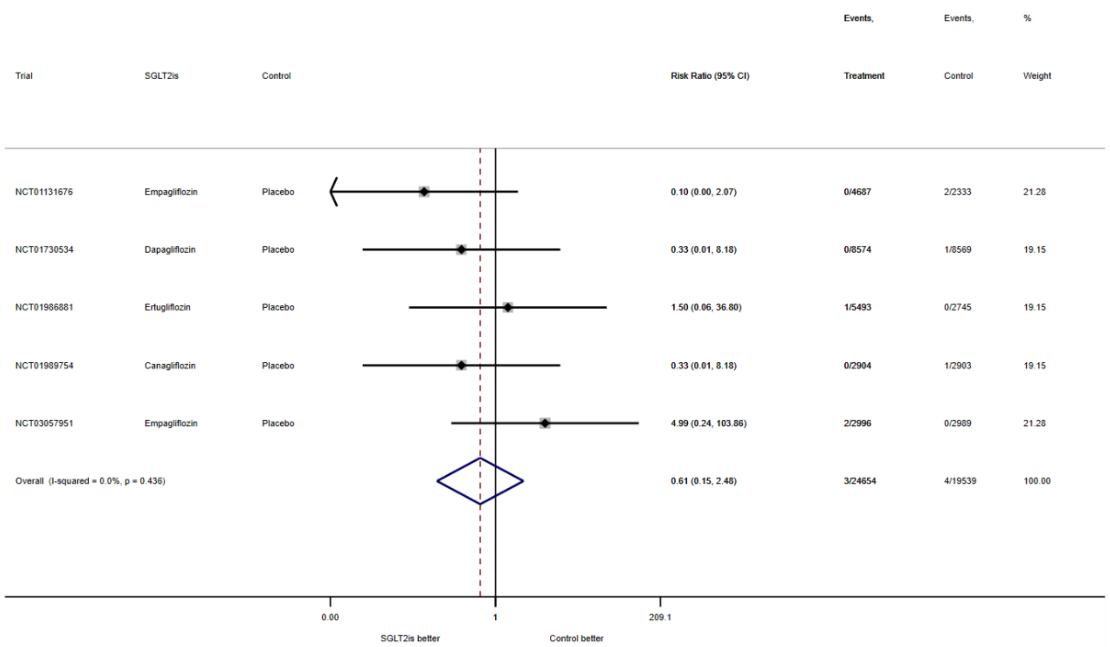

System 17-Nervous System Disorders-Figure S52 Meta-analysis of SGLT2is and Hypertensive encephalopathy

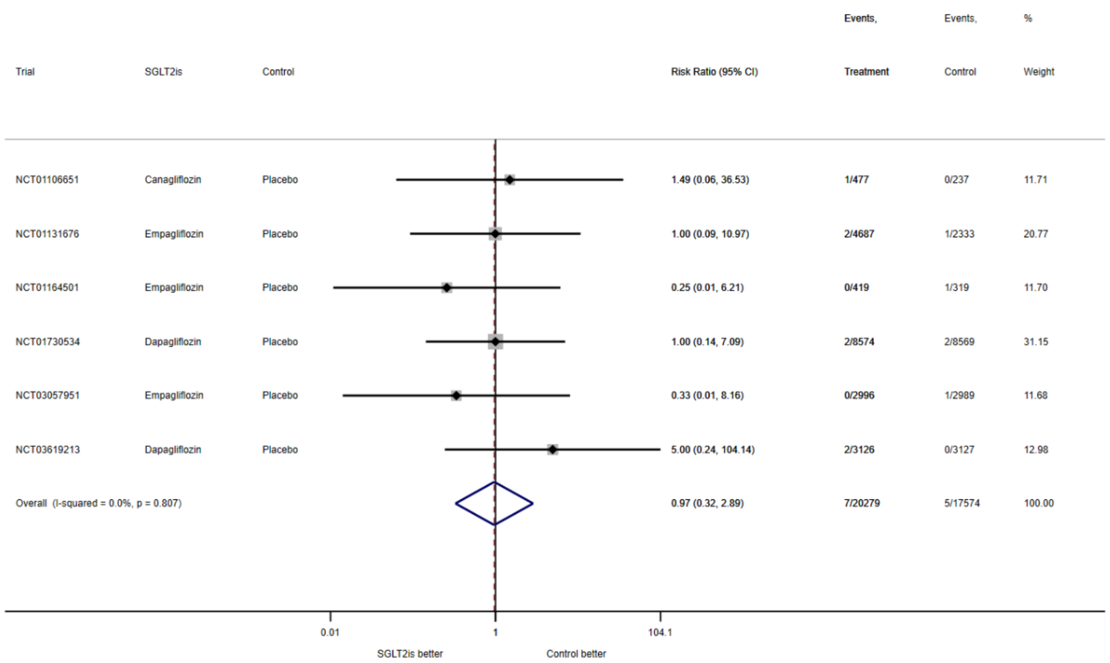

System 17-Nervous System Disorders-Figure S53 Meta-analysis of SGLT2is and Hypoaesthesia

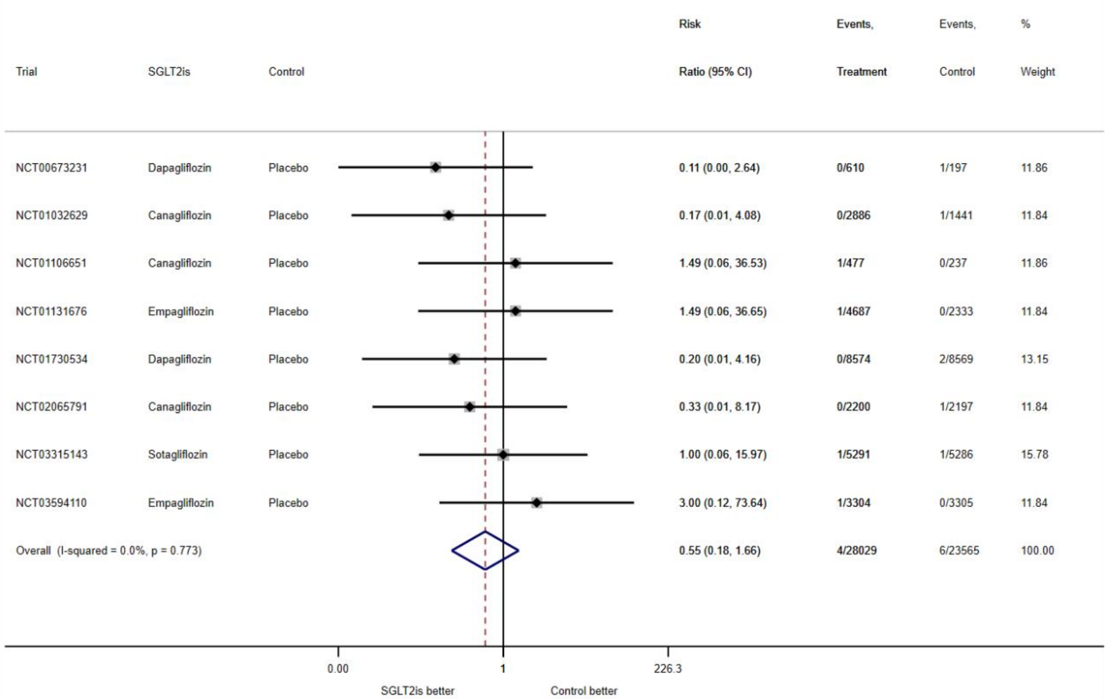

System 17-Nervous System Disorders-Figure S54 Meta-analysis of SGLT2is and Hypoglycaemic coma

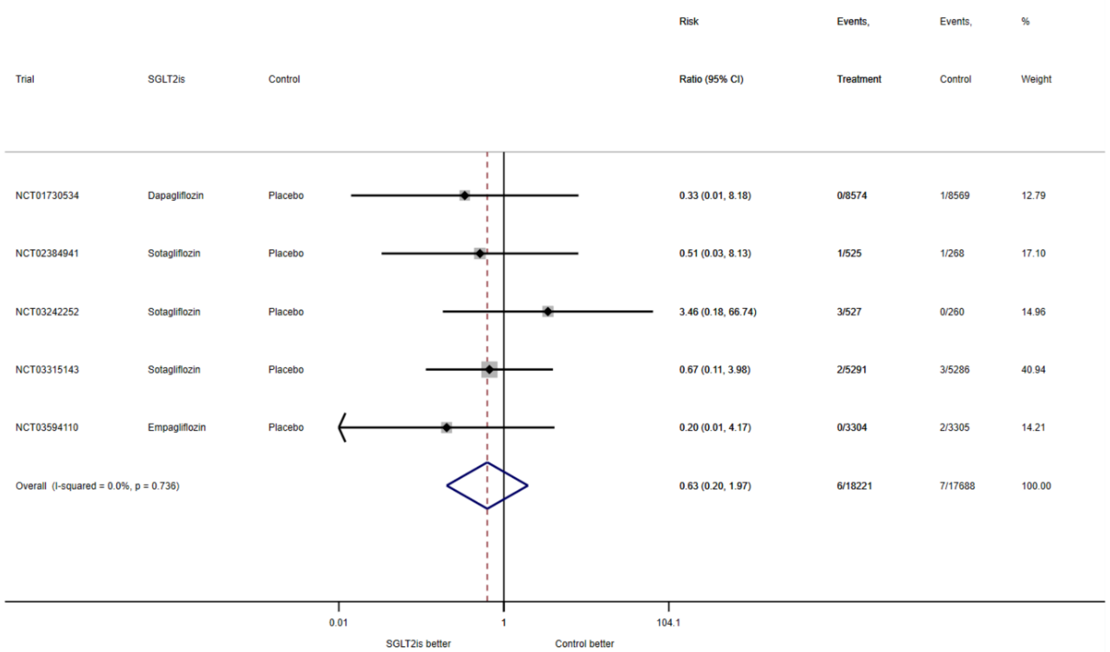

System 17-Nervous System Disorders-Figure S55 Meta-analysis of SGLT2is and Hypoglycaemic unconsciousness

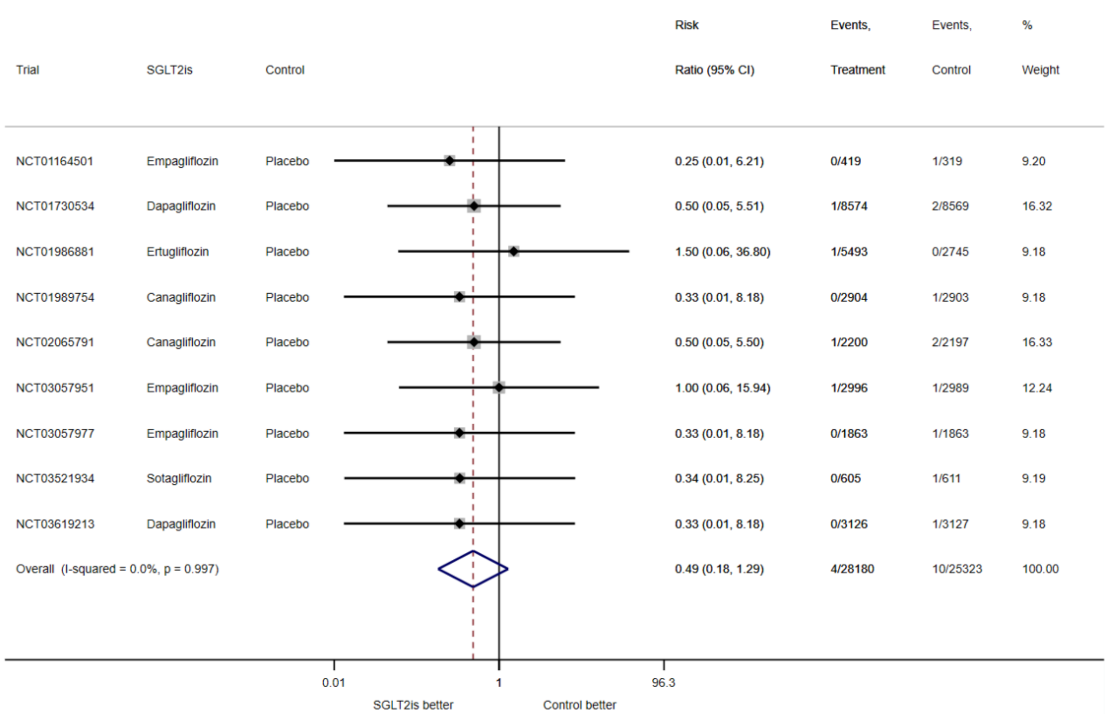

System 17-Nervous System Disorders-Figure S56 Meta-analysis of SGLT2is and Hypoxic-ischaemic encephalopathy

Trial	SGLT2is	Control	Risk Ratio (95% CI)	Events, Treatment	Events, Control	% Weight
NCT01730534	Dapagliflozin	Placebo	0.20 (0.01, 4.16)	0/8574	2/8569	12.19
NCT02384941	Sotagliflozin	Placebo	0.17 (0.01, 4.17)	0/525	1/268	10.99
NCT03036150	Dapagliflozin	Placebo	0.33 (0.01, 8.18)	0/2149	1/2149	10.97
NCT03057951	Empagliflozin	Placebo	1.00 (0.20, 4.94)	3/2996	3/2989	43.91
NCT03057977	Empagliflozin	Placebo	3.00 (0.12, 73.59)	1/1863	0/1863	10.97
NCT03594110	Empagliflozin	Placebo	0.33 (0.01, 8.18)	0/3304	1/3305	10.97
Overall (I-squared = 0.0%, p = 0.743)			0.60 (0.21, 1.73)	4/19411	8/19143	100.00

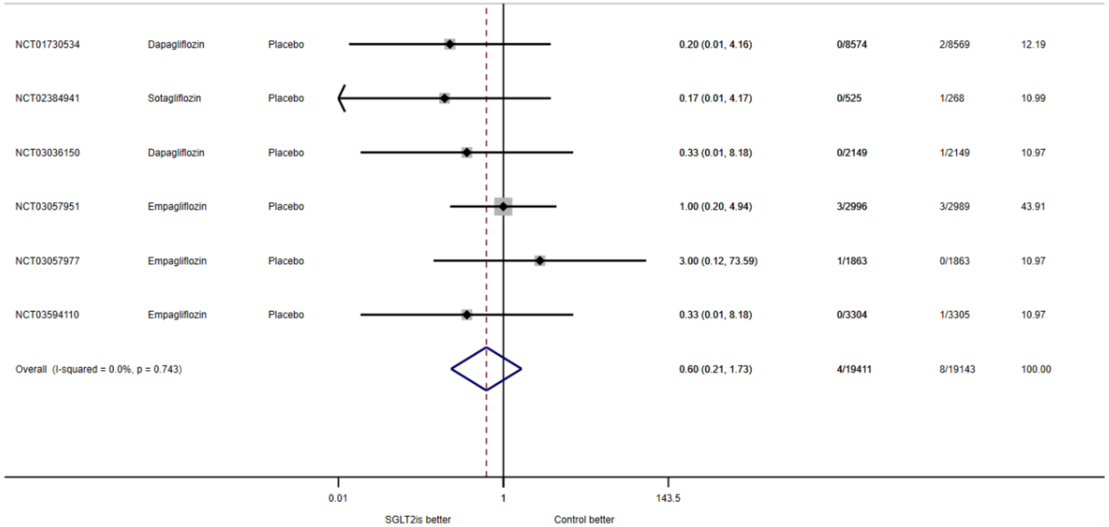

System 17-Nervous System Disorders-Figure S57 Meta-analysis of SGLT2is and Intracranial aneurysm

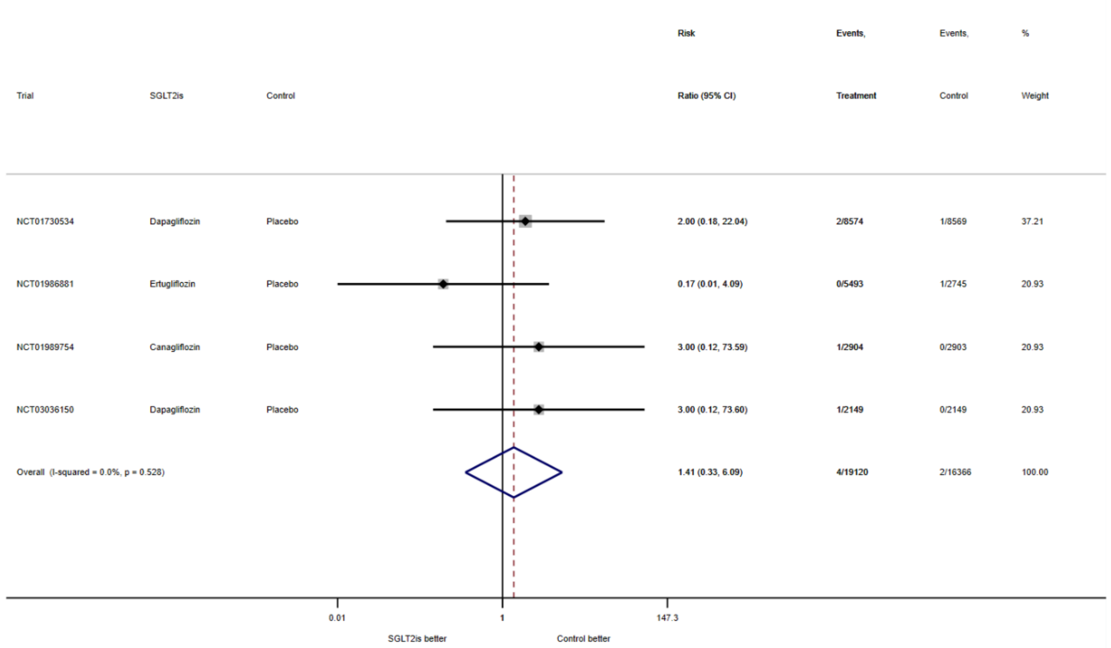

System 17-Nervous System Disorders-Figure S58 Meta-analysis of SGLT2is and Intraventricular haemorrhage

Trial	SGLT2is	Control	Risk Ratio (95% CI)	Events, Treatment	Events, Control	% Weight
NCT01131676	Empagliflozin	Placebo	0.50 (0.03, 7.95)	1/4687	1/2333	11.76
NCT01730534	Dapagliflozin	Placebo	1.00 (0.06, 15.98)	1/8574	1/8569	11.76
NCT01986881	Ertugliflozin	Placebo	0.50 (0.03, 7.99)	1/5493	1/2745	11.76
NCT01989754	Canagliflozin	Placebo	3.00 (0.12, 73.59)	1/2904	0/2903	8.82
NCT02065791	Canagliflozin	Placebo	0.33 (0.01, 8.17)	0/2200	1/2197	8.82
NCT03036124	Dapagliflozin	Placebo	3.00 (0.12, 73.61)	1/2368	0/2368	8.82
NCT03036150	Dapagliflozin	Placebo	3.00 (0.31, 28.82)	3/2149	1/2149	17.65
NCT03057951	Empagliflozin	Placebo	0.33 (0.01, 8.16)	0/2996	1/2989	8.82
NCT03619213	Dapagliflozin	Placebo	1.00 (0.06, 15.99)	1/3126	1/3127	11.76
Overall (I-squared = 0.0%, p = 0.921)			1.03 (0.40, 2.67)	9/34497	7/29380	100.00

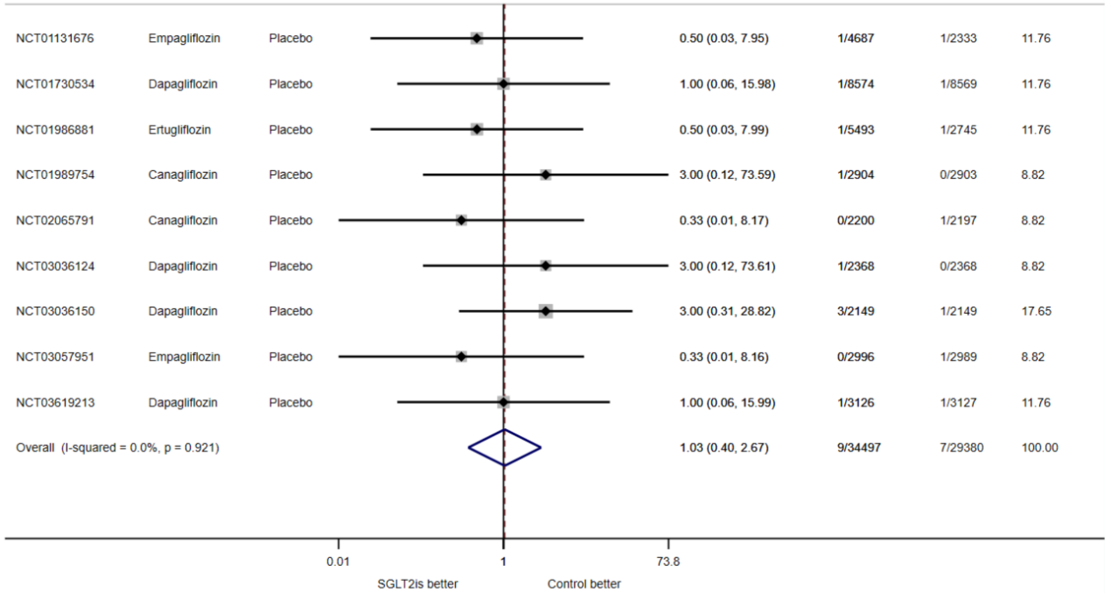

System 17-Nervous System Disorders-Figure S59 Meta-analysis of SGLT2is and Ischaemic cerebral infarction

Trial	SGLT2is	Control	Risk Ratio (95% CI)	Events, Treatment	Events, Control	% Weight
NCT00528879	Dapagliflozin	Placebo	0.11 (0.00, 2.74)	0/409	1/137	0.16
NCT01031680	Dapagliflozin	Placebo	1.00 (0.06, 16.01)	1/460	1/462	0.21
NCT01032629	Canagliflozin	Placebo	0.62 (0.17, 2.32)	5/2886	4/1441	0.95
NCT01042977	Dapagliflozin	Placebo	1.00 (0.06, 15.97)	1/482	1/483	0.21
NCT01106677	Canagliflozin	Sitagliptin	1.50 (0.06, 36.63)	1/735	0/366	0.16
NCT01131676	Empagliflozin	Placebo	0.86 (0.51, 1.45)	38/4687	22/2333	6.00
NCT01177813	Empagliflozin	Sitagliptin or Placebo	0.34 (0.01, 8.25)	0/447	1/452	0.16
NCT01730534	Dapagliflozin	Placebo	1.08 (0.81, 1.43)	97/8574	90/8569	20.14
NCT01986881	Ertugliflozin	Placebo	0.84 (0.57, 1.23)	69/5493	41/2745	11.13
NCT01989754	Canagliflozin	Placebo	1.67 (0.82, 3.40)	20/2904	12/2903	3.22
NCT02033889	Ertugliflozin	Placebo/Glimepiride	0.51 (0.03, 8.07)	1/412	1/209	0.21
NCT02065791	Canagliflozin	Placebo	0.82 (0.41, 1.66)	14/2200	17/2197	3.30
NCT02384941	Sotagliflozin	Placebo	1.53 (0.06, 37.53)	1/525	0/268	0.16
NCT03036124	Dapagliflozin	Placebo	0.92 (0.53, 1.60)	24/2368	26/2368	5.38
NCT03036150	Dapagliflozin	Placebo	0.79 (0.45, 1.37)	22/2149	28/2149	5.32
NCT03057951	Empagliflozin	Placebo	1.20 (0.77, 1.87)	42/2996	35/2989	8.25
NCT03057977	Empagliflozin	Placebo	0.95 (0.51, 1.77)	19/1863	20/1863	4.20
NCT03242252	Sotagliflozin	Placebo	0.49 (0.07, 3.48)	2/527	2/260	0.43
NCT03315143	Sotagliflozin	Placebo	0.80 (0.49, 1.31)	29/5291	36/5286	6.89
NCT03521934	Sotagliflozin	Placebo	1.01 (0.40, 2.53)	9/605	9/611	1.95
NCT03594110	Empagliflozin	Placebo	0.88 (0.54, 1.44)	30/3304	34/3305	6.87
NCT03619213	Dapagliflozin	Placebo	1.08 (0.77, 1.50)	70/3126	65/3127	14.70
Overall (I-squared = 0.0%, p = 0.983)			0.97 (0.85, 1.10)	495/52443	446/44523	100.00

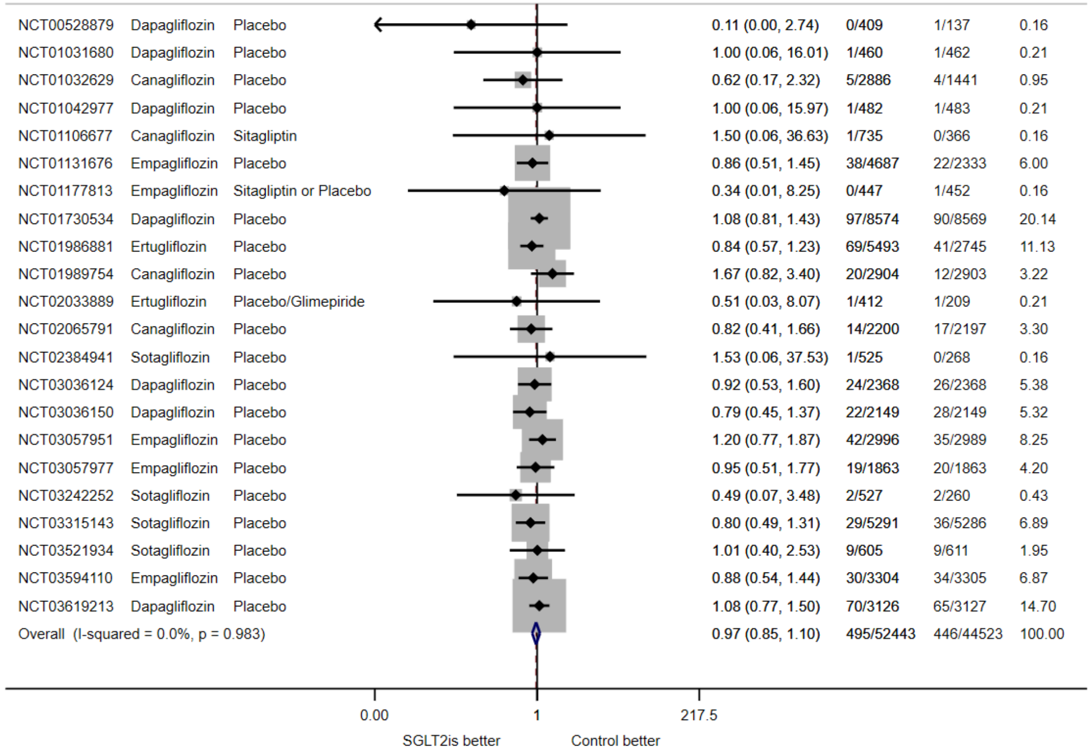

System 17-Nervous System Disorders-Figure S60 Meta-analysis of SGLT2is and Ischaemic stroke

Trial	SGLT2is	Control	Risk Ratio (95% CI)	Events, Treatment	Events, Control	% Weight
NCT01032629	Canagliflozin	Placebo	2.50 (0.12, 51.99)	2/2886	0/1441	3.01
NCT01131676	Empagliflozin	Placebo	0.75 (0.12, 4.47)	3/4687	2/2333	8.68
NCT01164501	Empagliflozin	Placebo	2.29 (0.09, 55.92)	1/419	0/319	2.72
NCT01730534	Dapagliflozin	Placebo	0.57 (0.24, 1.36)	8/8574	14/8569	36.83
NCT01986881	Ertugliflozin	Placebo	0.25 (0.02, 2.75)	1/5493	2/2745	4.82
NCT01989754	Canagliflozin	Placebo	1.00 (0.14, 7.09)	2/2904	2/2903	7.23
NCT02065791	Canagliflozin	Placebo	1.00 (0.25, 3.99)	4/2200	4/2197	14.48
NCT03057951	Empagliflozin	Placebo	0.67 (0.11, 3.98)	2/2996	3/2989	8.68
NCT03057977	Empagliflozin	Placebo	3.00 (0.12, 73.59)	1/1863	0/1863	2.71
NCT03315143	Sotagliflozin	Placebo	3.00 (0.12, 73.56)	1/5291	0/5286	2.71
NCT03594110	Empagliflozin	Placebo	0.33 (0.01, 8.18)	0/3304	1/3305	2.71
NCT03619213	Dapagliflozin	Placebo	0.33 (0.03, 3.20)	1/3126	3/3127	5.42
Overall (I-squared = 0.0%, p = 0.945)			0.73 (0.43, 1.24)	26/43743	31/37077	100.00

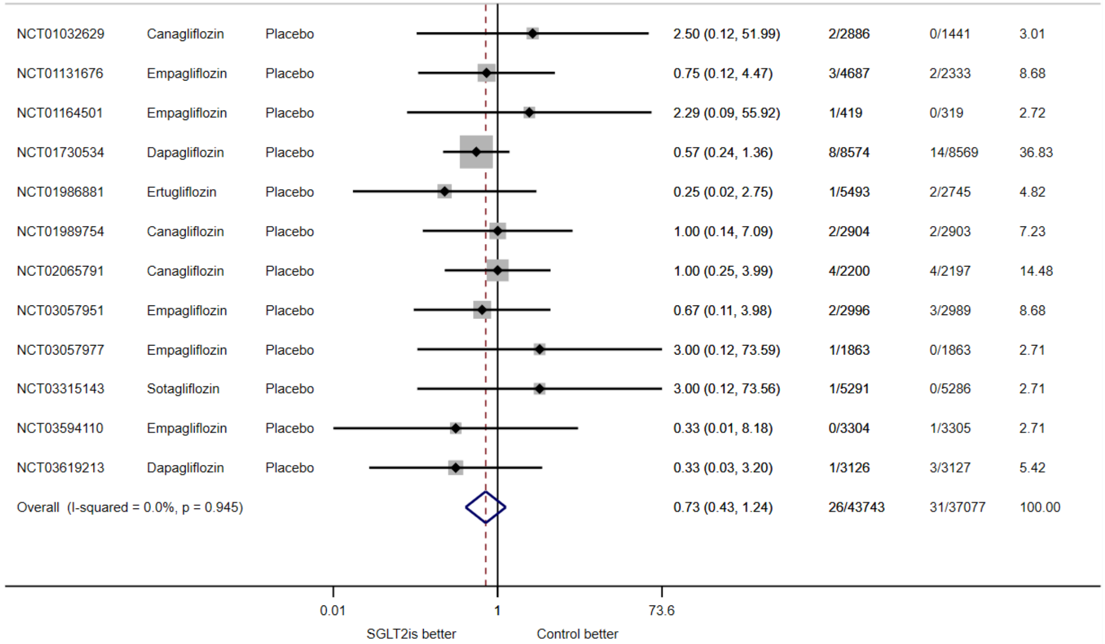

System 17-Nervous System Disorders-Figure S61 Meta-analysis of SGLT2is and Lacunar infarction

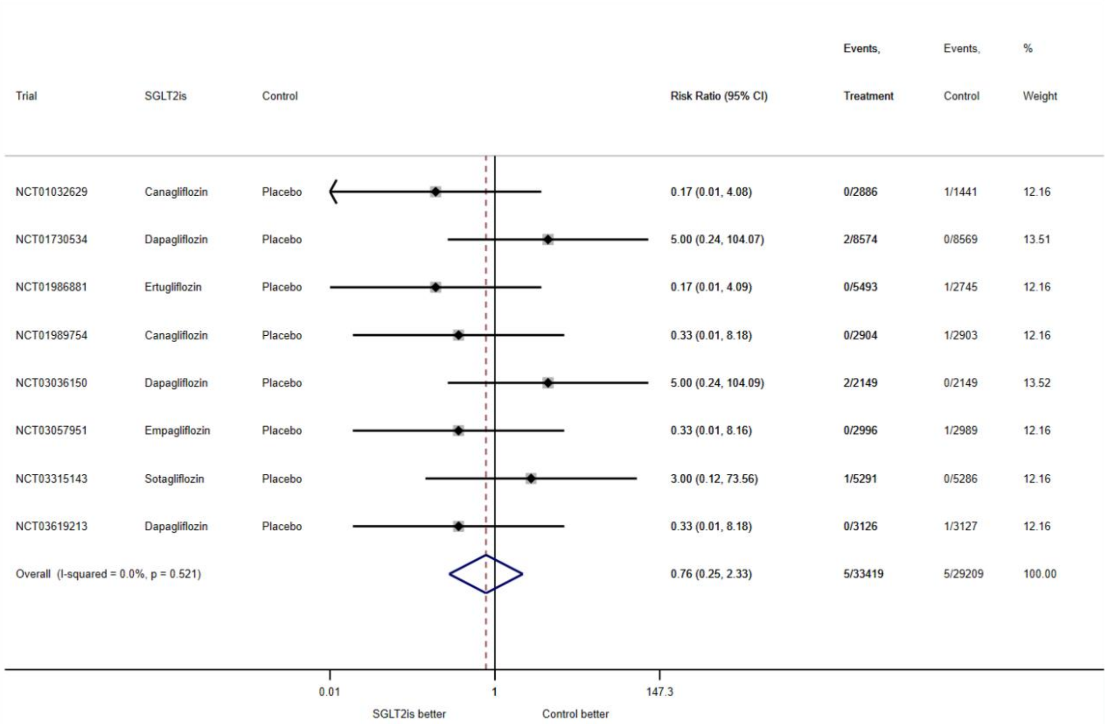

System 17-Nervous System Disorders-Figure S62 Meta-analysis of SGLT2is and Lacunar stroke

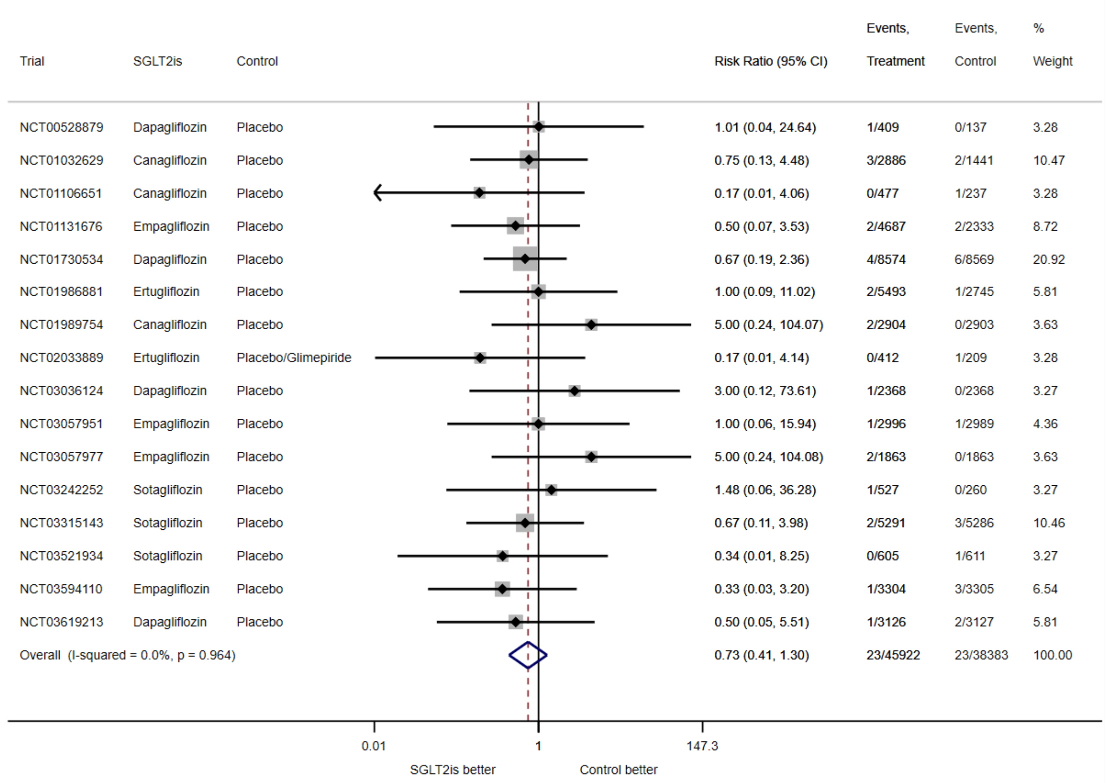

System 17-Nervous System Disorders-Figure S63 Meta-analysis of SGLT2is and Loss of consciousness

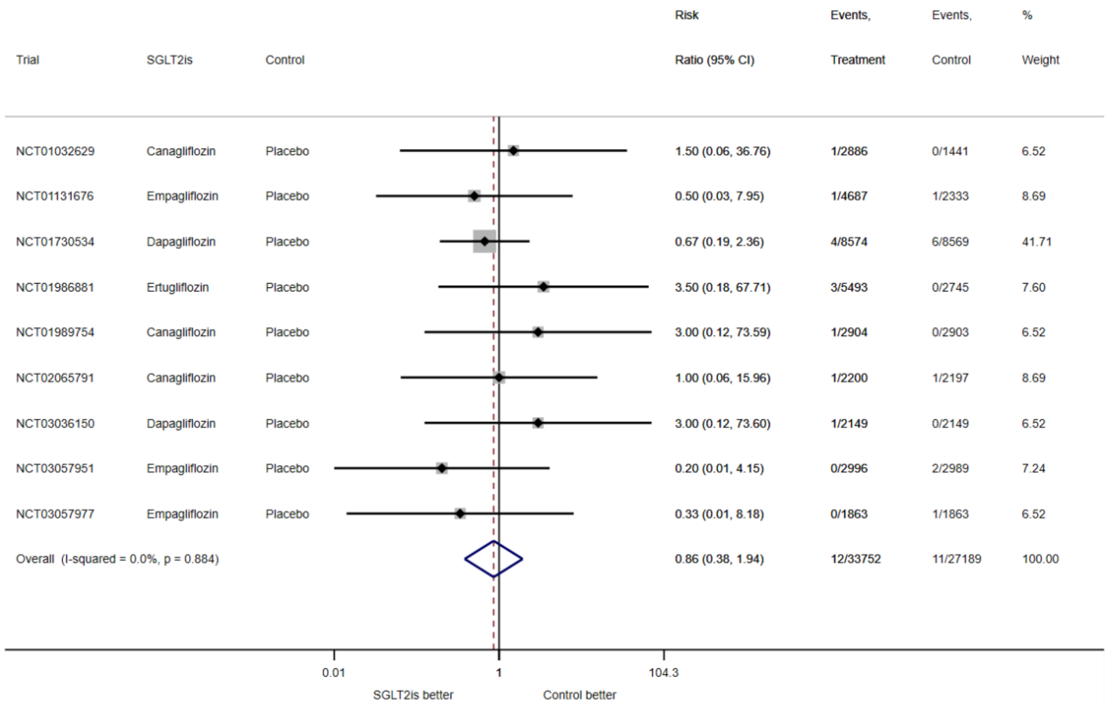

System 17-Nervous System Disorders-Figure S64 Meta-analysis of SGLT2is and Lumbar radiculopathy

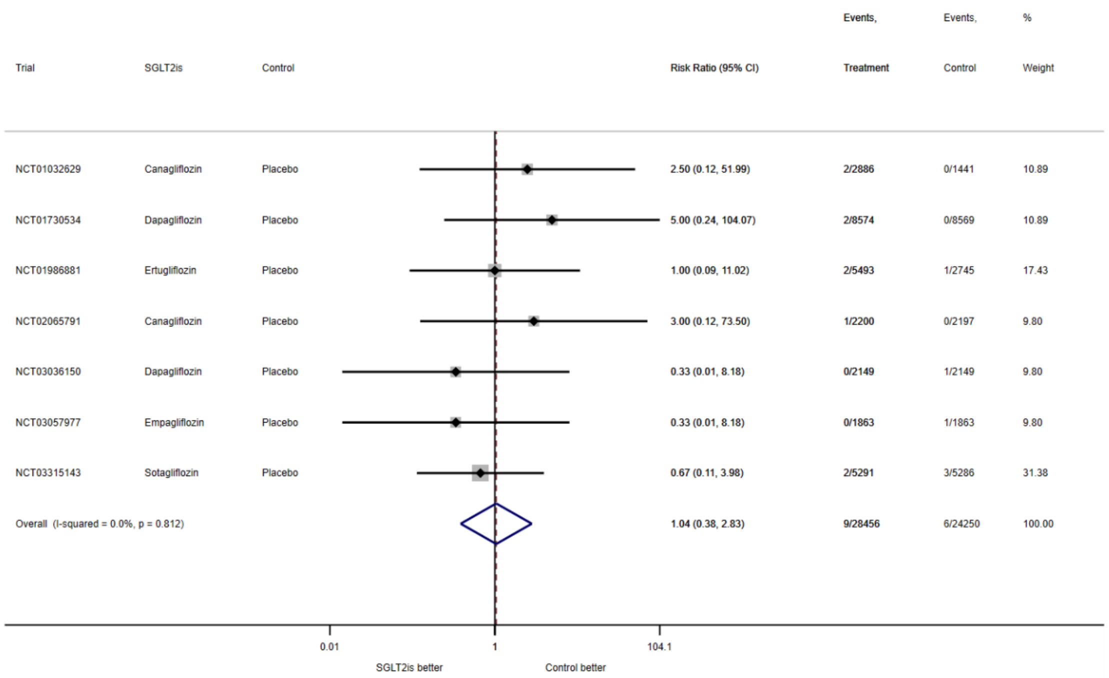

System 17-Nervous System Disorders-Figure S65 Meta-analysis of SGLT2is and Lumbosacral radiculopathy

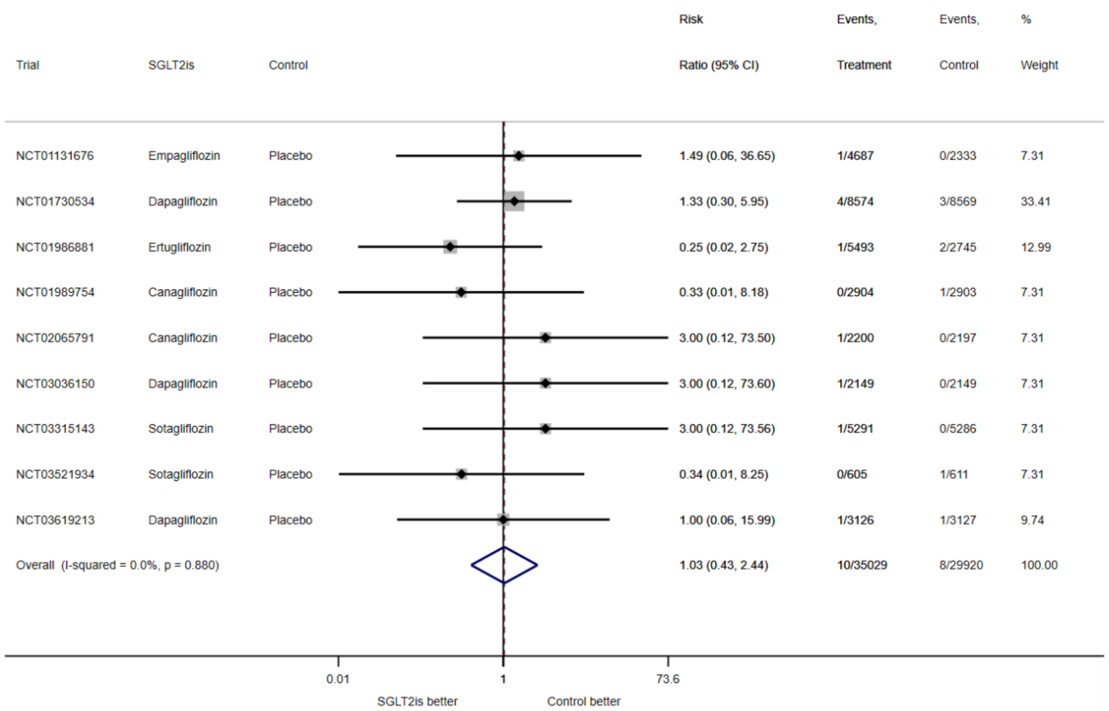

System 17-Nervous System Disorders-Figure S66 Meta-analysis of SGLT2is and Metabolic encephalopathy

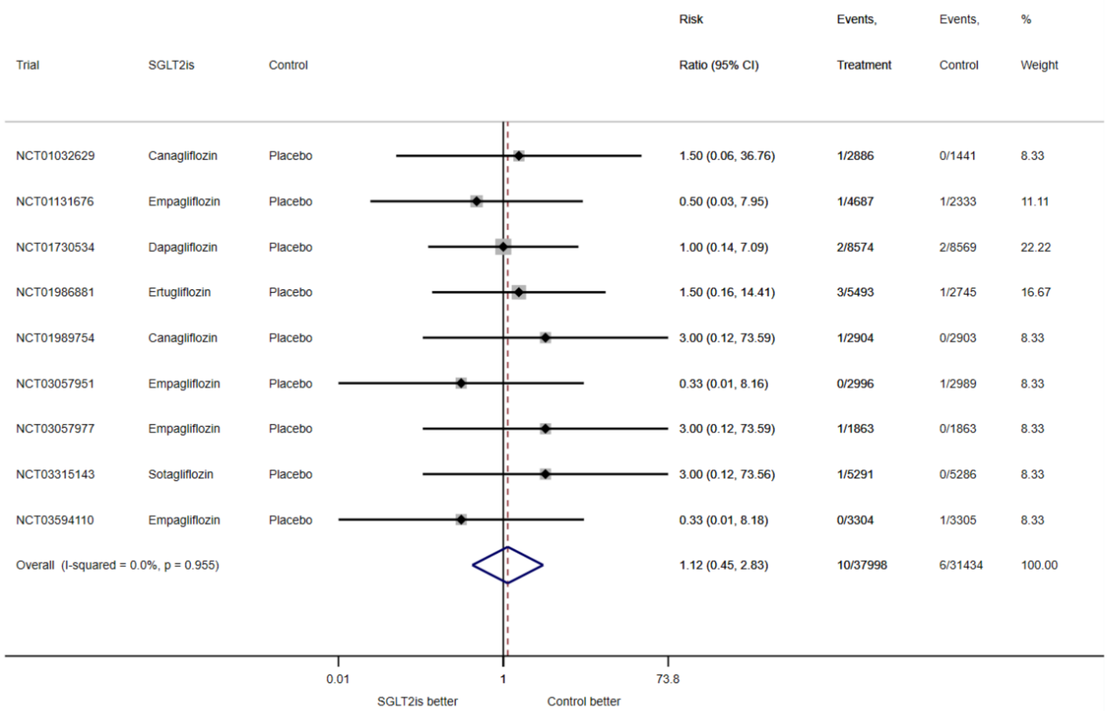

System 17-Nervous System Disorders-Figure S67 Meta-analysis of SGLT2is and Migraine

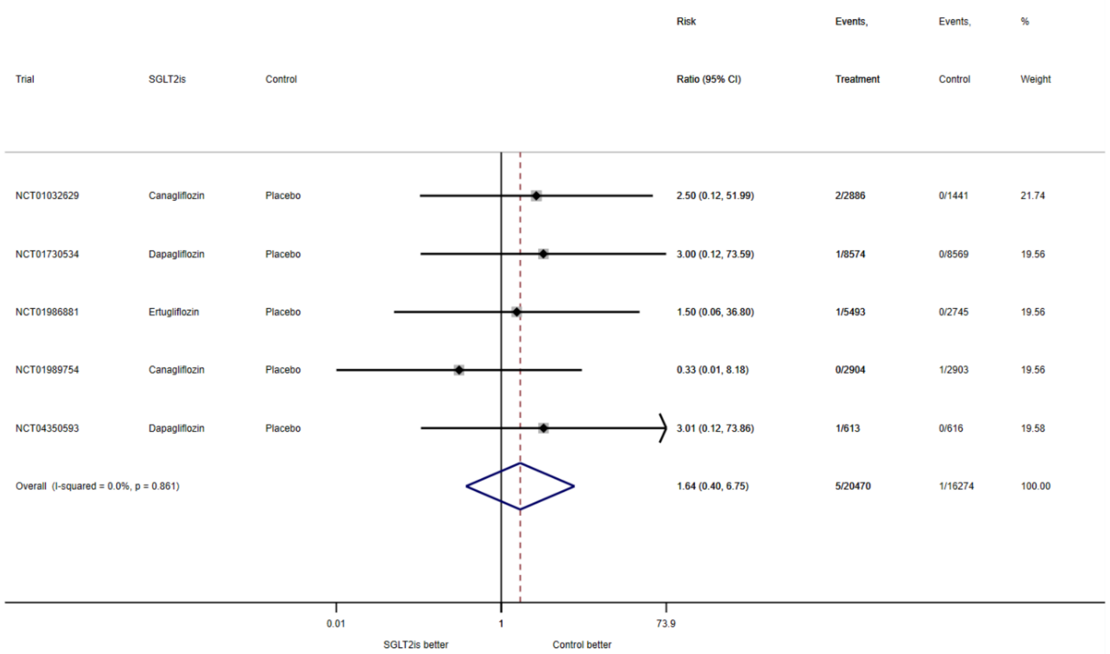

System 17-Nervous System Disorders-Figure S68 Meta-analysis of SGLT2is and Myasthenia gravis

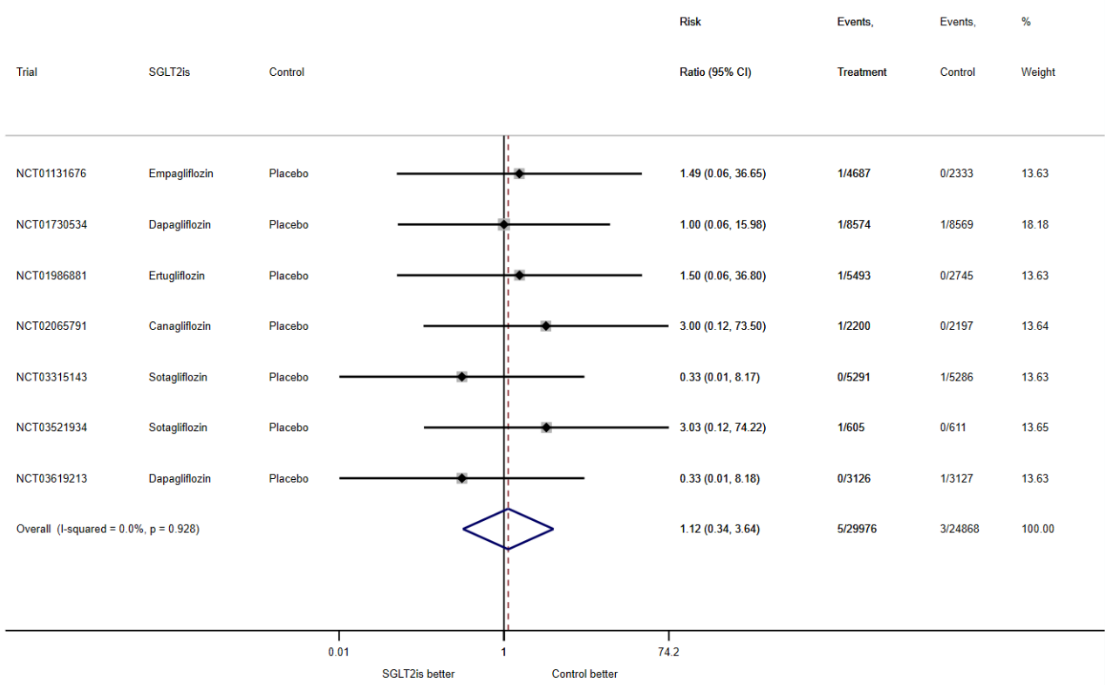

System 17-Nervous System Disorders-Figure S69 Meta-analysis of SGLT2is and Myelopathy

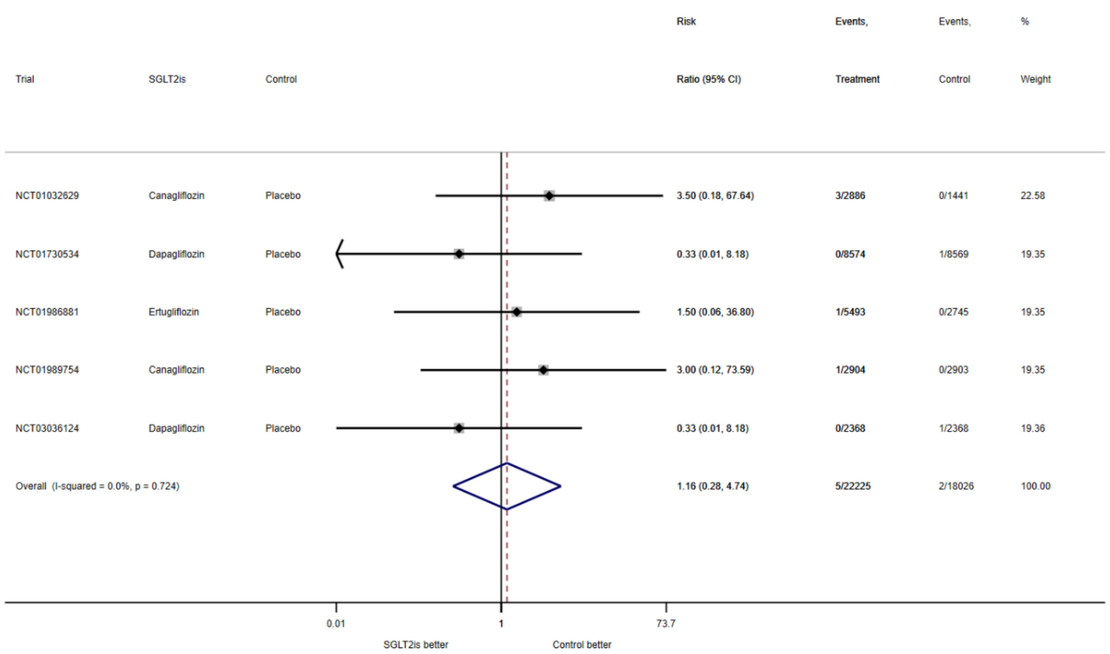

System 17-Nervous System Disorders-Figure S70 Meta-analysis of SGLT2is and Neuralgia

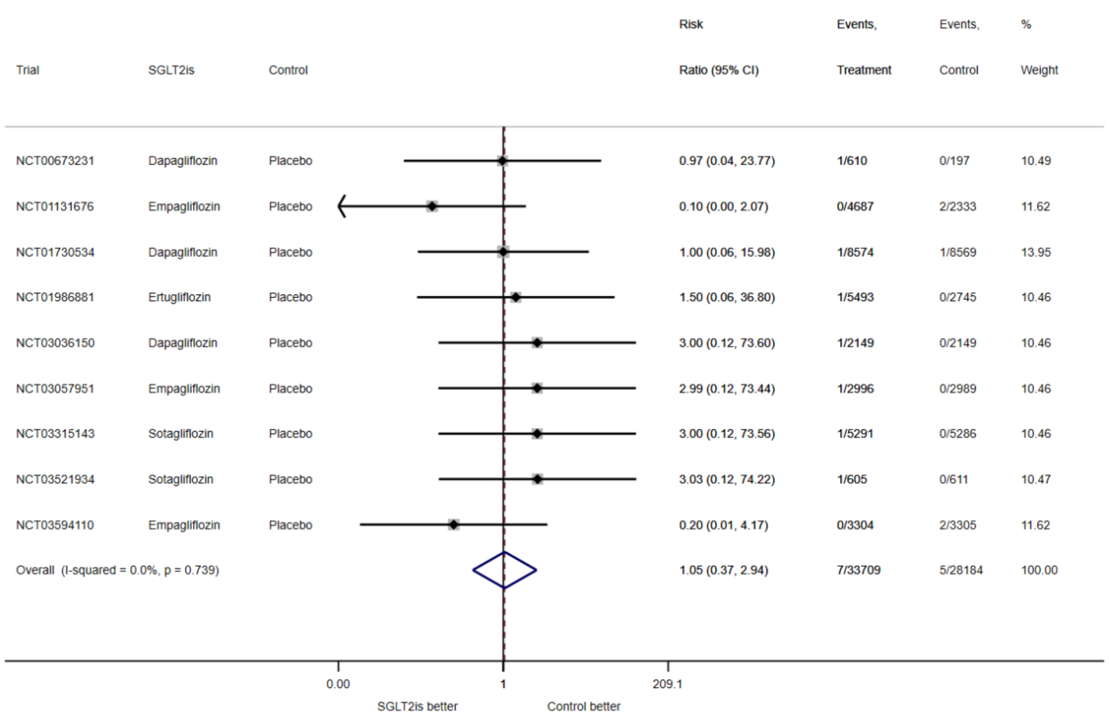

System 17-Nervous System Disorders-Figure S71 Meta-analysis of SGLT2is and Neuropathy peripheral

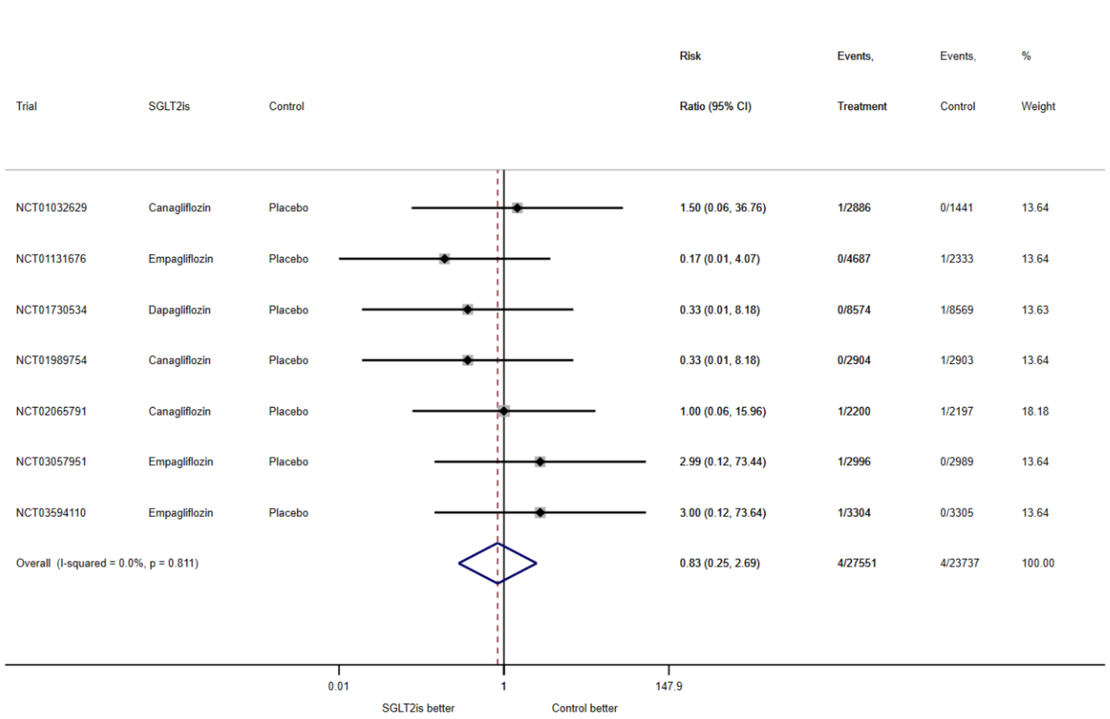

System 17-Nervous System Disorders-Figure S72 Meta-analysis of SGLT2is and Paraesthesia

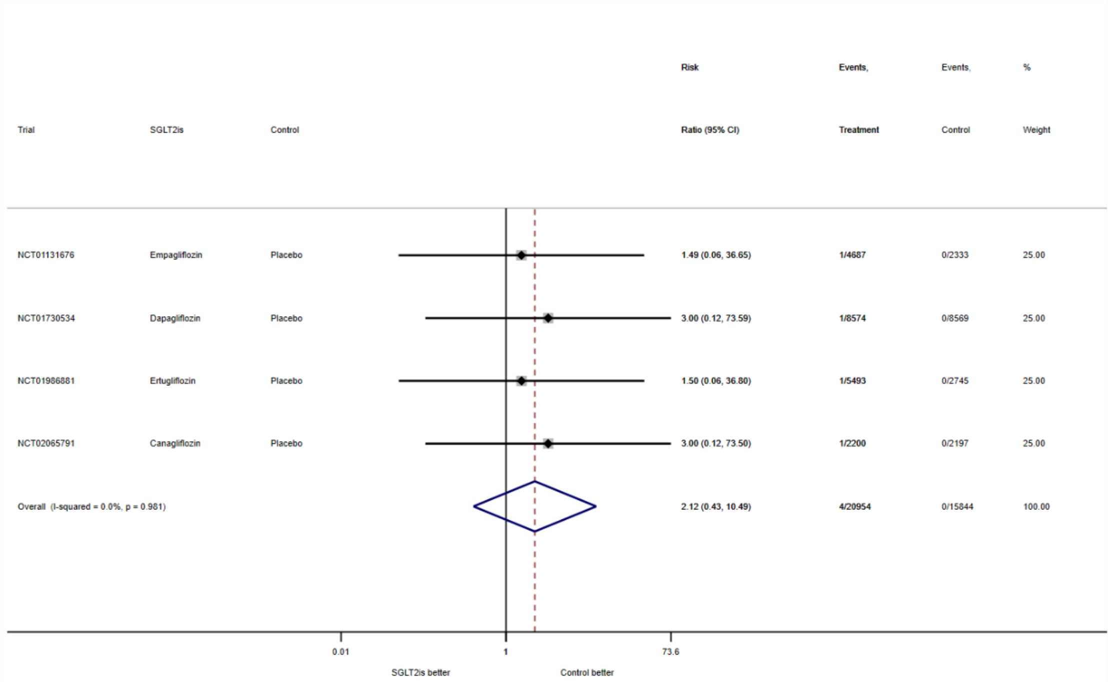

System 17-Nervous System Disorders-Figure S73 Meta-analysis of SGLT2is and Paraparesis

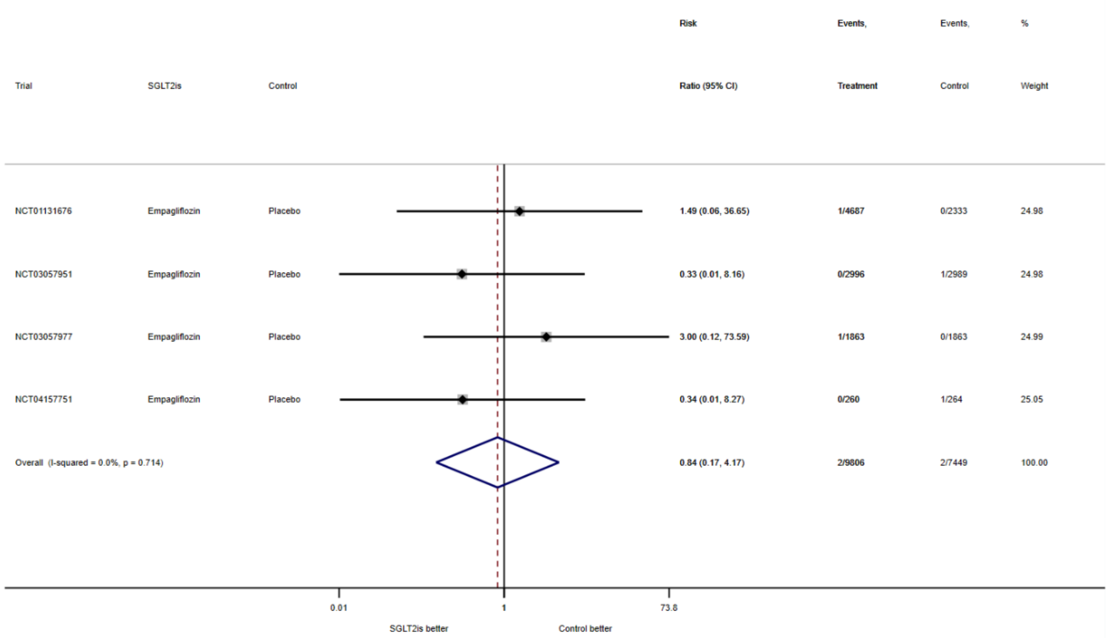

System 17-Nervous System Disorders-Figure S74 Meta-analysis of SGLT2is and Paraplegia

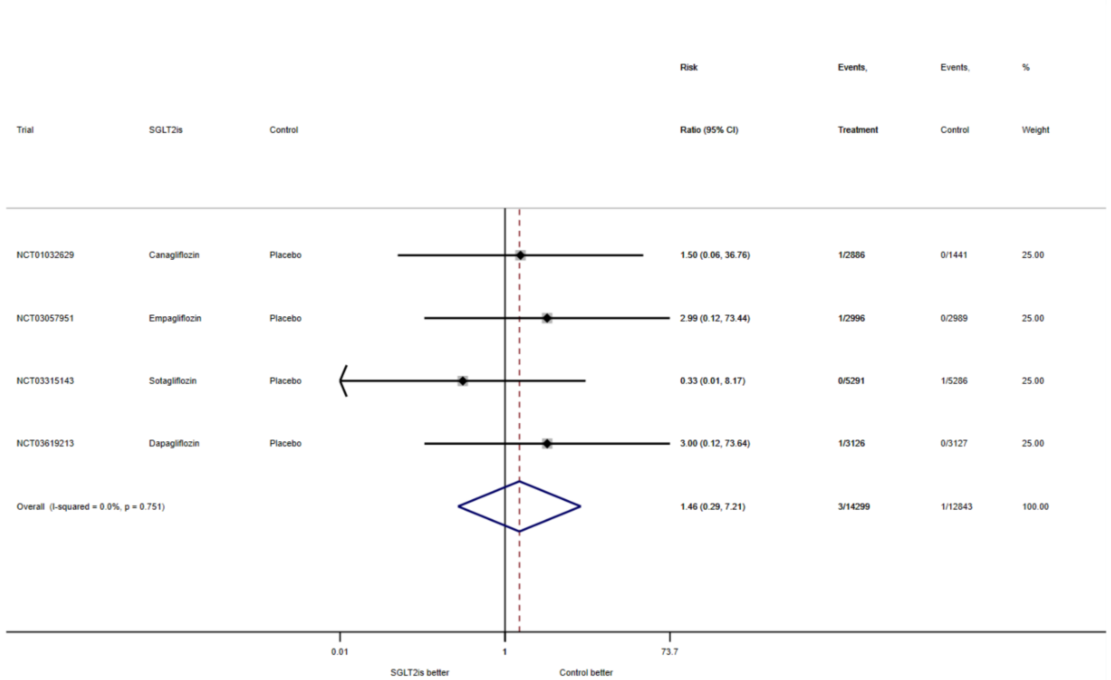

System 17-Nervous System Disorders-Figure S75 Meta-analysis of SGLT2is and Parkinsonism

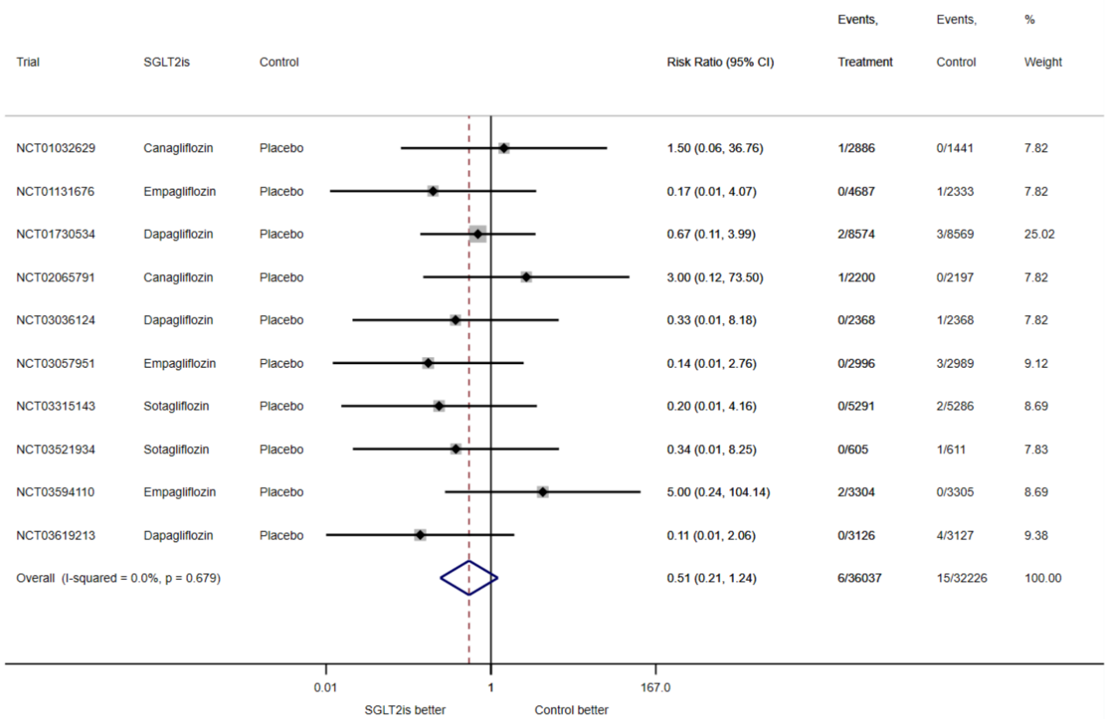

System 17-Nervous System Disorders-Figure S76 Meta-analysis of SGLT2is and Parkinson's disease

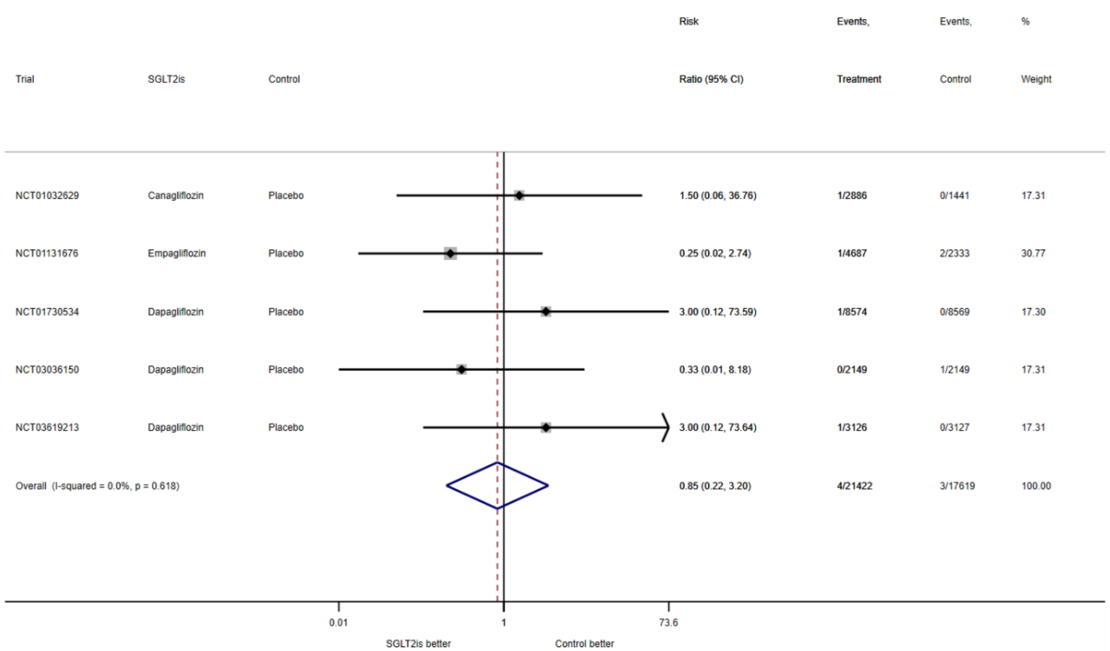

System 17-Nervous System Disorders-Figure S77 Meta-analysis of SGLT2is and Partial seizures

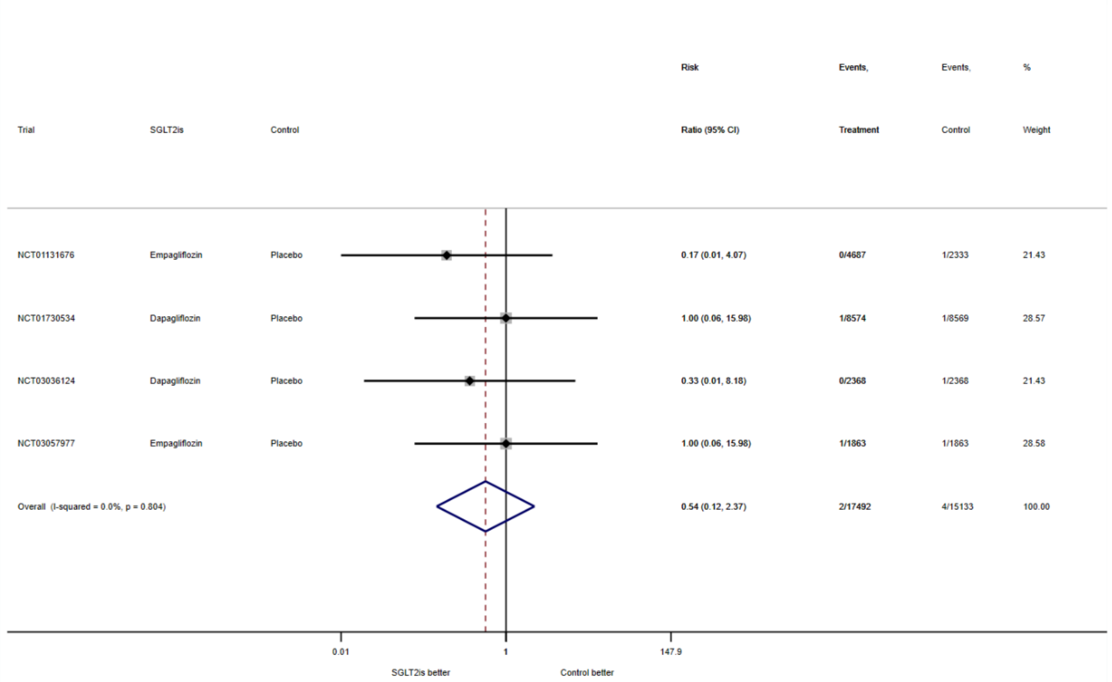

System 17-Nervous System Disorders-Figure S78 Meta-analysis of SGLT2is and Peroneal nerve palsy

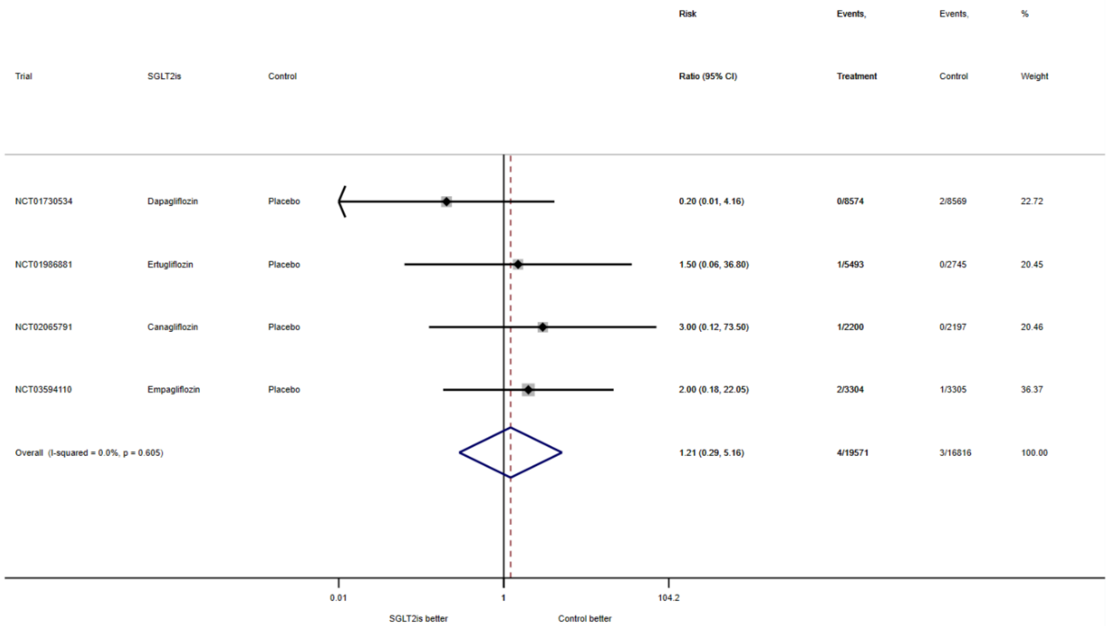

System 17-Nervous System Disorders-Figure S79 Meta-analysis of SGLT2is and Polyneuropathy

Trial	SGLT2is	Control	Risk Ratio (95% CI)	Events, Treatment	Events, Control	% Weight
NCT01032629	Canagliflozin	Placebo	2.50 (0.29, 21.35)	5/2886	1/1441	7.17
NCT01106651	Canagliflozin	Placebo	1.49 (0.06, 36.53)	1/477	0/237	3.23
NCT01131676	Empagliflozin	Placebo	2.49 (0.12, 51.83)	2/4687	0/2333	3.58
NCT01730534	Dapagliflozin	Placebo	1.00 (0.20, 4.95)	3/8574	3/8569	12.89
NCT01986881	Ertugliflozin	Placebo	0.60 (0.18, 1.96)	6/5493	5/2745	23.47
NCT01989754	Canagliflozin	Placebo	3.00 (0.31, 28.81)	3/2904	1/2903	6.45
NCT02065791	Canagliflozin	Placebo	1.00 (0.06, 15.96)	1/2200	1/2197	4.30
NCT03036124	Dapagliflozin	Placebo	0.14 (0.01, 2.76)	0/2368	3/2368	3.76
NCT03036150	Dapagliflozin	Placebo	1.00 (0.06, 15.98)	1/2149	1/2149	4.30
NCT03057951	Empagliflozin	Placebo	2.99 (0.31, 28.76)	3/2996	1/2989	6.45
NCT03057977	Empagliflozin	Placebo	9.00 (0.48, 167.04)	4/1863	0/1863	3.87
NCT03315143	Sotagliflozin	Placebo	3.00 (0.31, 28.80)	3/5291	1/5286	6.45
NCT03521934	Sotagliflozin	Placebo	0.34 (0.01, 8.25)	0/605	1/611	3.23
NCT03594110	Empagliflozin	Placebo	0.08 (0.00, 1.37)	0/3304	6/3305	3.99
NCT03619213	Dapagliflozin	Placebo	4.00 (0.45, 35.78)	4/3126	1/3127	6.88
Overall (I-squared = 0.0%, p = 0.522)			1.19 (0.67, 2.11)	36/48923	25/42123	100.00

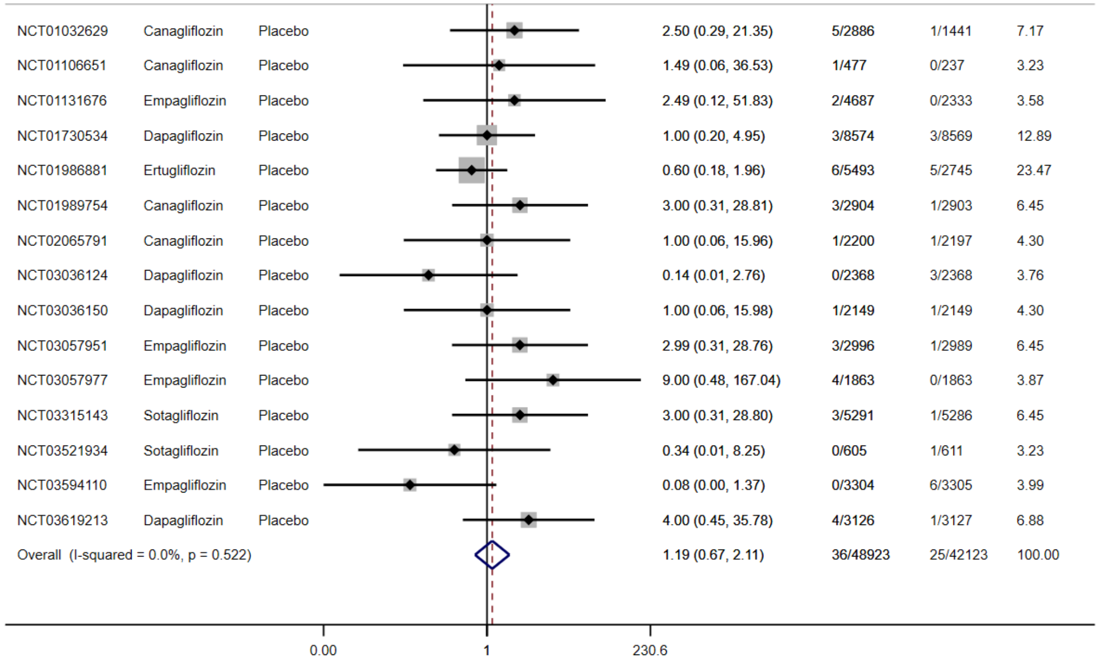

System 17-Nervous System Disorders-Figure S80 Meta-analysis of SGLT2is and Presyncope

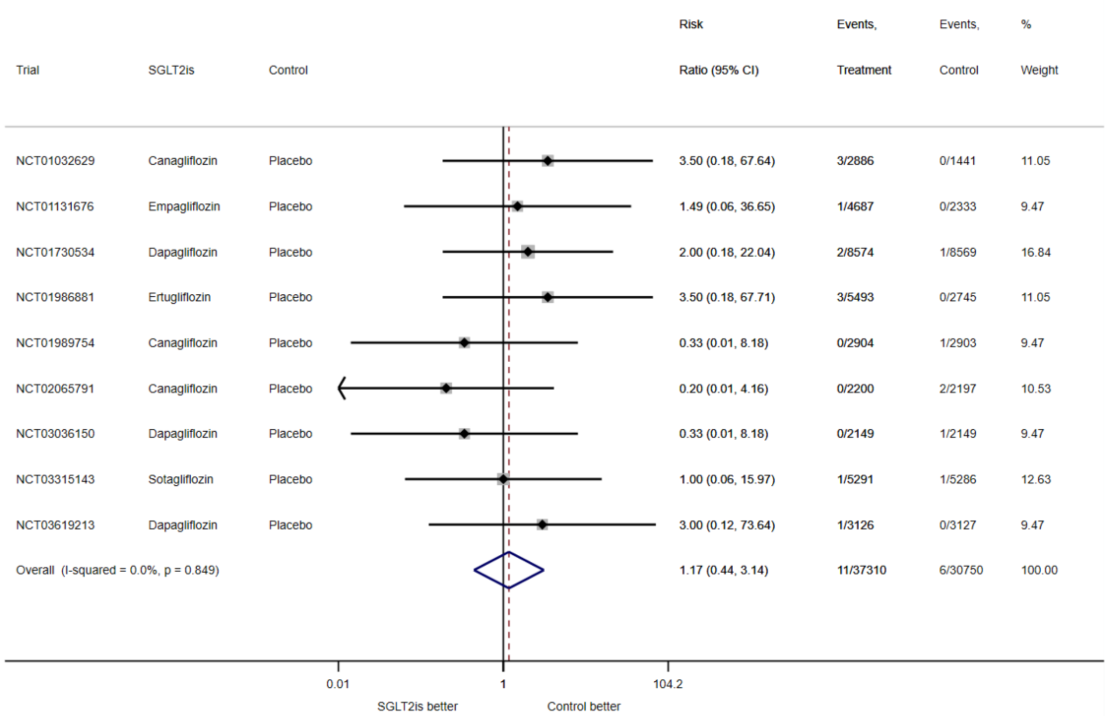

System 17-Nervous System Disorders-Figure S81 Meta-analysis of SGLT2is and Radiculopathy

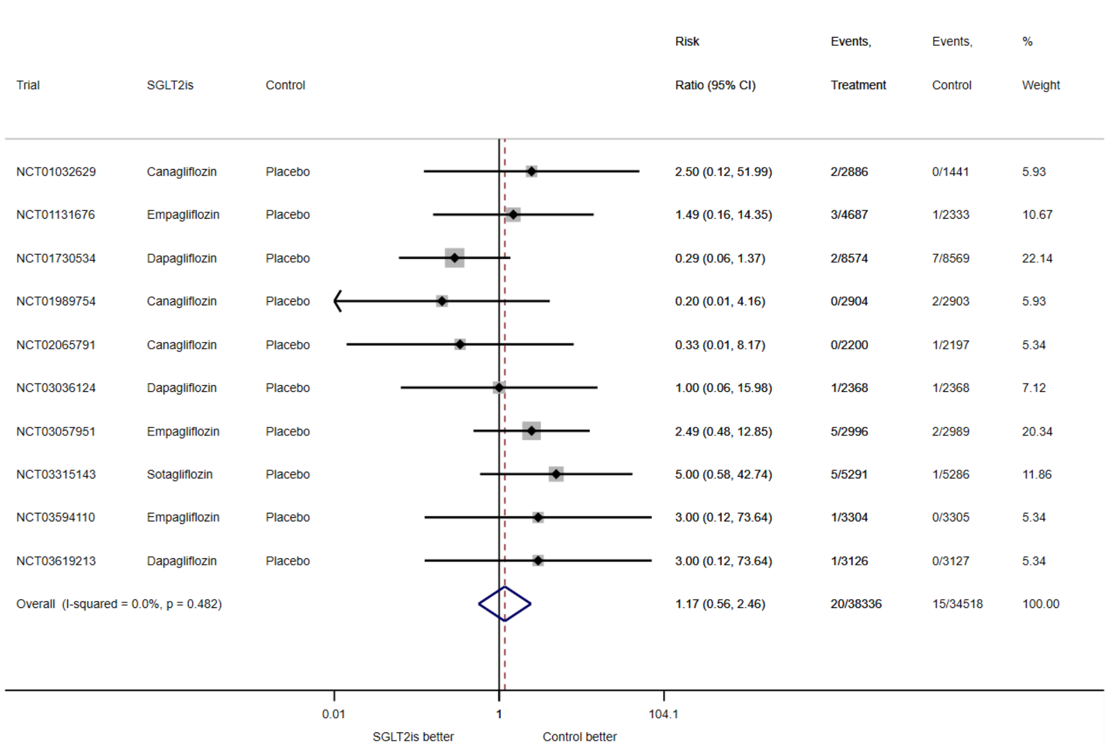

System 17-Nervous System Disorders-Figure S82 Meta-analysis of SGLT2is and Sciatica

Trial	SGLT2is	Control	Risk Ratio (95% CI)	Events, Treatment	Events, Control	% Weight
NCT01032629	Canagliflozin	Placebo	0.75 (0.13, 4.48)	3/2886	2/1441	7.11
NCT01131676	Empagliflozin	Placebo	1.49 (0.48, 4.62)	12/4687	4/2333	17.79
NCT01730534	Dapagliflozin	Placebo	0.50 (0.17, 1.46)	5/8574	10/8569	19.74
NCT01986881	Ertugliflozin	Placebo	4.50 (0.24, 83.52)	4/5493	0/2745	2.66
NCT01989754	Canagliflozin	Placebo	0.20 (0.01, 4.16)	0/2904	2/2903	2.47
NCT02065791	Canagliflozin	Placebo	2.00 (0.18, 22.01)	2/2200	1/2197	3.95
NCT03036150	Dapagliflozin	Placebo	0.33 (0.01, 8.18)	0/2149	1/2149	2.22
NCT03057951	Empagliflozin	Placebo	0.43 (0.11, 1.65)	3/2996	7/2989	12.45
NCT03057977	Empagliflozin	Placebo	0.67 (0.11, 3.99)	2/1863	3/1863	7.11
NCT03315143	Sotagliflozin	Placebo	1.00 (0.14, 7.09)	2/5291	2/5286	5.92
NCT03521934	Sotagliflozin	Placebo	0.34 (0.01, 8.25)	0/605	1/611	2.22
NCT03594110	Empagliflozin	Placebo	2.00 (0.37, 10.92)	4/3304	2/3305	7.90
NCT03619213	Dapagliflozin	Placebo	2.50 (0.49, 12.88)	5/3126	2/3127	8.46
Overall (I-squared = 0.0%, p = 0.715)			0.90 (0.56, 1.44)	42/46078	37/39518	100.00

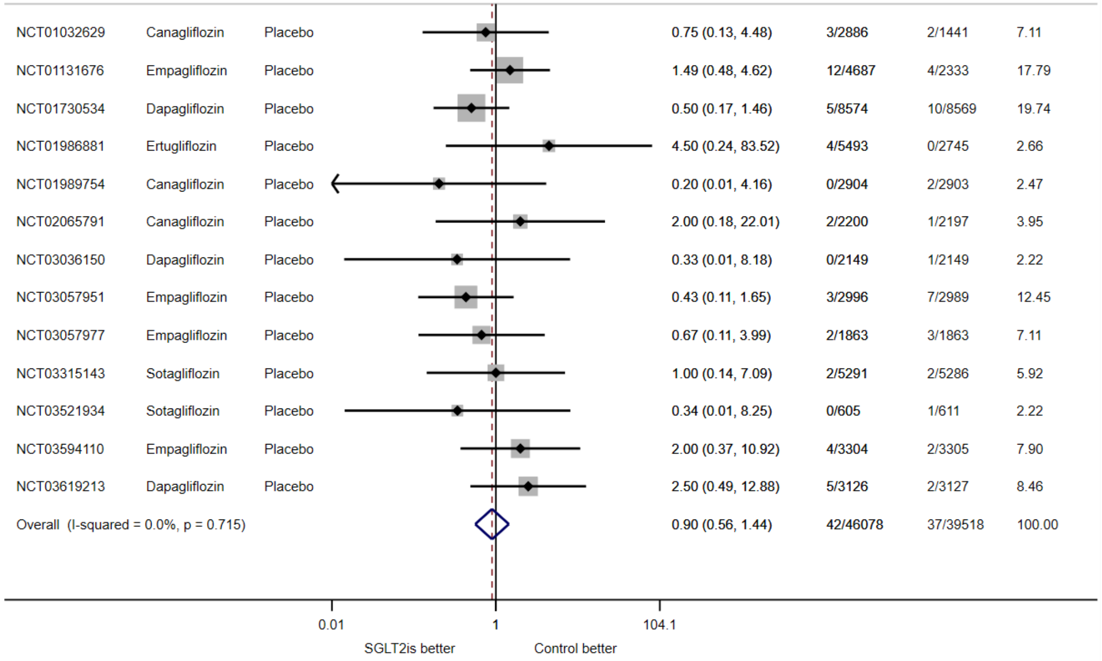

System 17-Nervous System Disorders-Figure S83 Meta-analysis of SGLT2is and Seizure

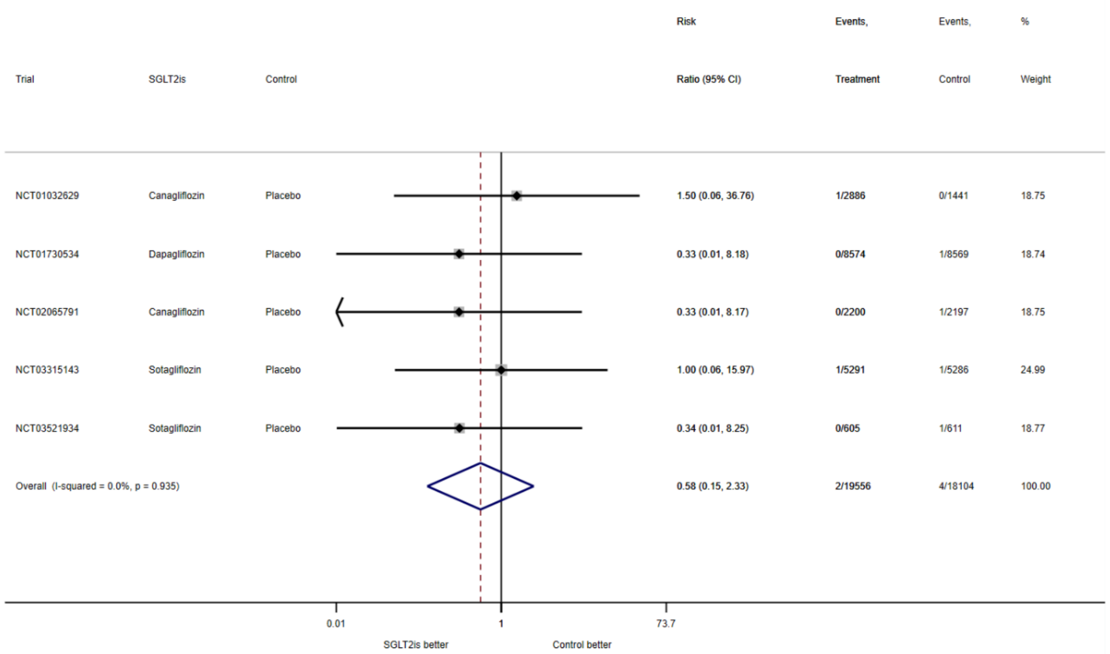

System 17-Nervous System Disorders-Figure S84 Meta-analysis of SGLT2is and Somnolence

Trial	SGLT2is	Control	Risk Ratio (95% CI)	Events, Treatment	Events, Control	% Weight
NCT01032629	Canagliflozin	Placebo	1.50 (0.06, 36.76)	1/2886	0/1441	14.28
NCT01042977	Dapagliflozin	Placebo	3.01 (0.12, 73.61)	1/482	0/483	14.30
NCT01131676	Empagliflozin	Placebo	1.49 (0.06, 36.65)	1/4687	0/2333	14.28
NCT01989754	Canagliflozin	Placebo	0.33 (0.01, 8.18)	0/2904	1/2903	14.28
NCT02065791	Canagliflozin	Placebo	3.00 (0.12, 73.50)	1/2200	0/2197	14.28
NCT03057977	Empagliflozin	Placebo	3.00 (0.12, 73.59)	1/1863	0/1863	14.28
NCT04157751	Empagliflozin	Placebo	0.34 (0.01, 8.27)	0/260	1/264	14.31
Overall (I-squared = 0.0%, p = 0.903)			1.32 (0.39, 4.41)	5/15282	2/11484	100.00

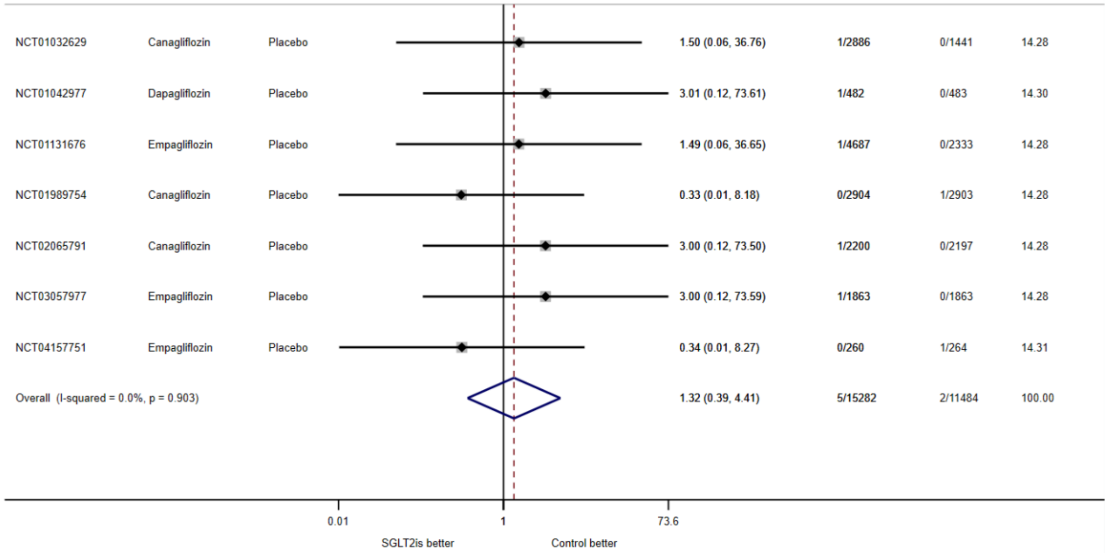

System 17-Nervous System Disorders-Figure S85 Meta-analysis of SGLT2is and Spinal cord compression

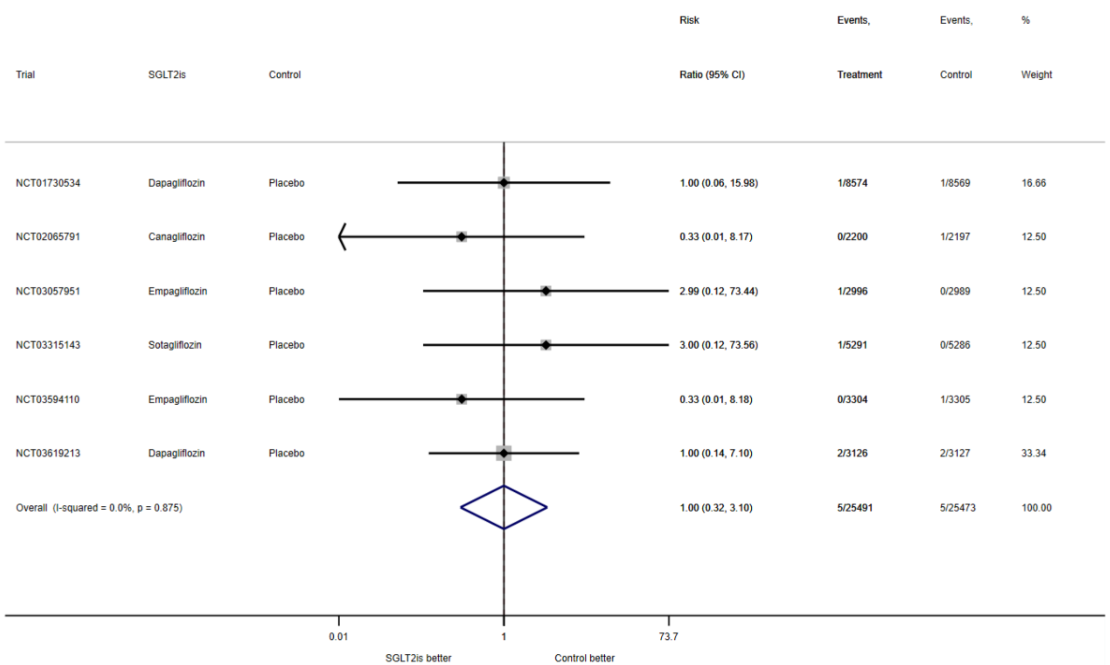

System 17-Nervous System Disorders-Figure S86 Meta-analysis of SGLT2is and Status epilepticus

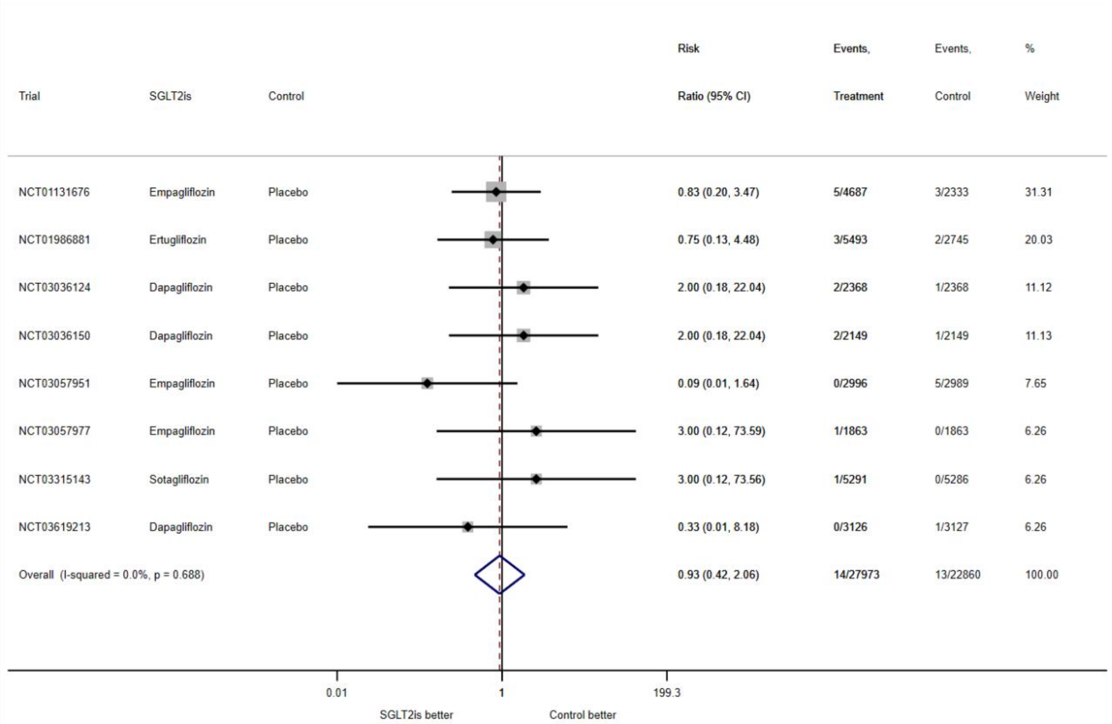

System 17-Nervous System Disorders-Figure S87 Meta-analysis of SGLT2is and Subarachnoid haemorrhage

Trial	SGLT2is	Control	Risk Ratio (95% CI)	Events, Treatment	Events, Control	% Weight
NCT00528879	Dapagliflozin	Placebo	0.33 (0.02, 5.32)	1/409	1/137	0.52
NCT01032629	Canagliflozin	Placebo	1.00 (0.40, 2.47)	14/2886	7/1441	4.84
NCT01042977	Dapagliflozin	Placebo	1.00 (0.06, 15.97)	1/482	1/483	0.52
NCT01106651	Canagliflozin	Placebo	1.49 (0.06, 36.53)	1/477	0/237	0.39
NCT01131676	Empagliflozin	Placebo	1.68 (0.76, 3.69)	27/4687	8/2333	6.39
NCT01164501	Empagliflozin	Placebo	2.29 (0.09, 55.92)	1/419	0/319	0.39
NCT01730534	Dapagliflozin	Placebo	0.90 (0.58, 1.41)	37/8574	41/8569	20.16
NCT01986881	Ertugliflozin	Placebo	1.12 (0.49, 2.58)	18/5493	8/2745	5.73
NCT01989754	Canagliflozin	Placebo	1.25 (0.49, 3.16)	10/2904	8/2903	4.60
NCT02065791	Canagliflozin	Placebo	1.33 (0.30, 5.94)	4/2200	3/2197	1.77
NCT03036124	Dapagliflozin	Placebo	0.54 (0.22, 1.35)	7/2368	13/2368	4.71
NCT03036150	Dapagliflozin	Placebo	1.40 (0.45, 4.40)	7/2149	5/2149	3.02
NCT03057951	Empagliflozin	Placebo	1.21 (0.66, 2.21)	23/2996	19/2989	10.81
NCT03057977	Empagliflozin	Placebo	2.13 (0.92, 4.91)	17/1863	8/1863	5.65
NCT03242252	Sotagliflozin	Placebo	0.16 (0.01, 4.03)	0/527	1/260	0.39
NCT03315143	Sotagliflozin	Placebo	0.96 (0.56, 1.65)	26/5291	27/5286	13.74
NCT03594110	Empagliflozin	Placebo	2.00 (0.86, 4.67)	16/3304	8/3305	5.52
NCT03619213	Dapagliflozin	Placebo	0.86 (0.46, 1.61)	18/3126	21/3127	10.07
NCT04157751	Empagliflozin	Placebo	3.05 (0.12, 74.43)	1/260	0/264	0.39
NCT04350593	Dapagliflozin	Placebo	0.33 (0.01, 8.21)	0/613	1/616	0.39
Overall (I-squared = 0.0%, p = 0.838)			1.09 (0.89, 1.33)	229/51028	180/43591	100.00

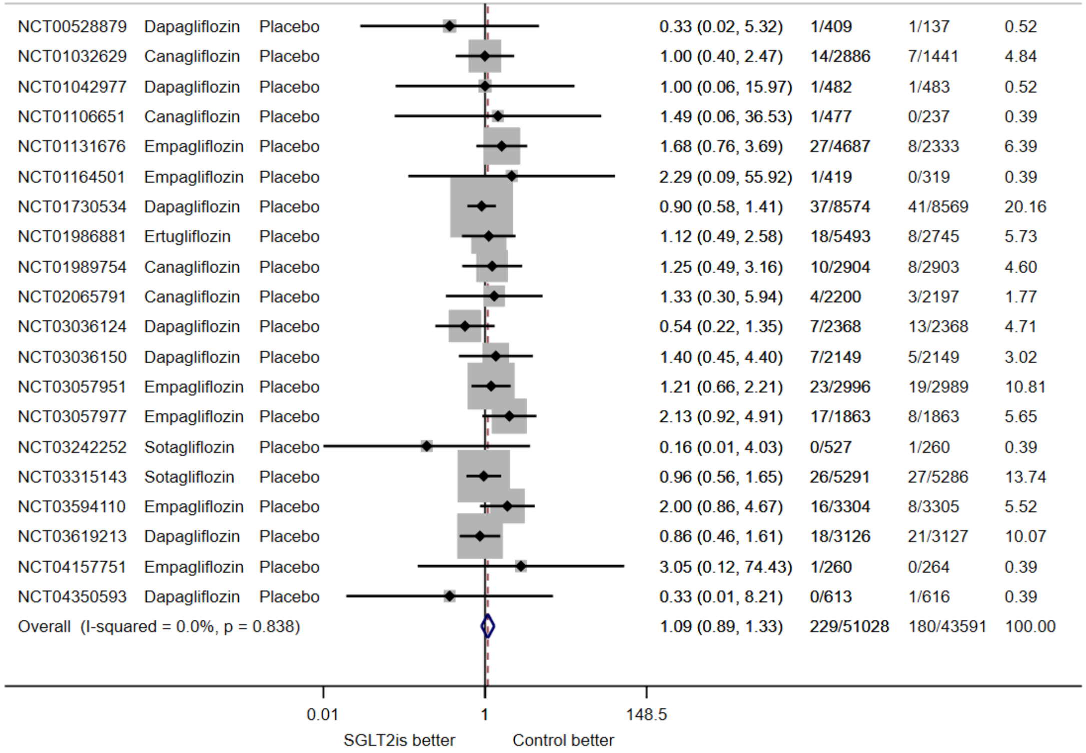

System 17-Nervous System Disorders-Figure S88 Meta-analysis of SGLT2is and Syncope

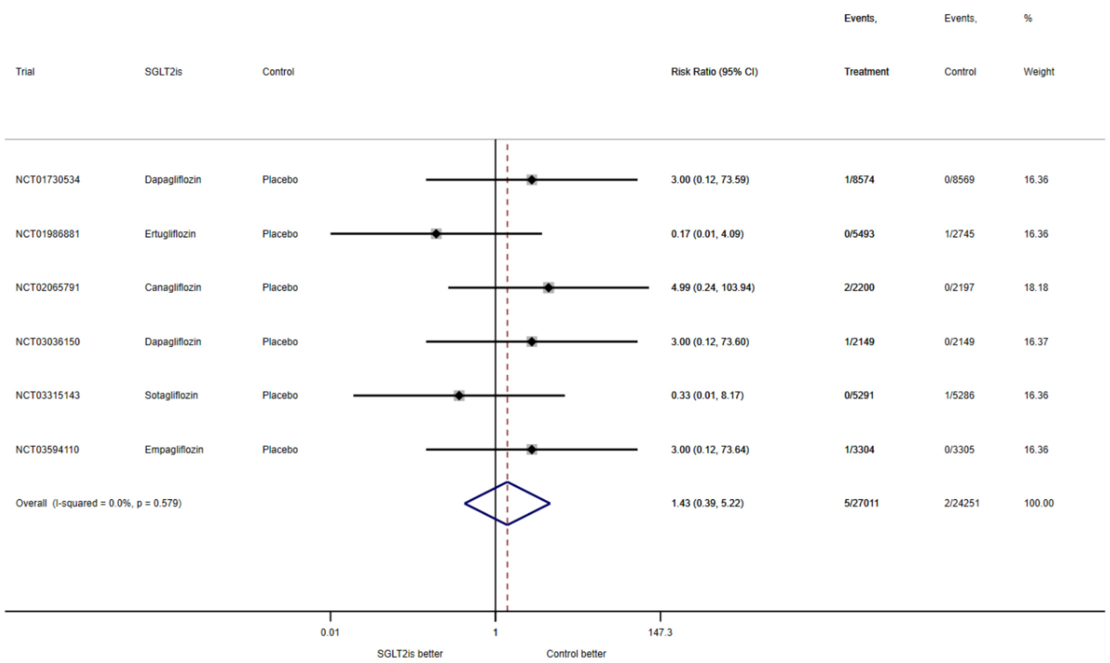

System 17-Nervous System Disorders-Figure S89 Meta-analysis of SGLT2is and Tension headache

Trial	SGLT2is	Control	Risk Ratio (95% CI)	Events, Treatment	Events, Control	% Weight
NCT01131676	Empagliflozin	Placebo	0.50 (0.03, 7.95)	1/4687	1/2333	14.82
NCT01730534	Dapagliflozin	Placebo	1.00 (0.14, 7.09)	2/8574	2/8569	29.63
NCT01986881	Ertugliflozin	Placebo	1.50 (0.06, 36.80)	1/5493	0/2745	11.11
NCT01989754	Canagliflozin	Placebo	3.00 (0.12, 73.59)	1/2904	0/2903	11.11
NCT02065791	Canagliflozin	Placebo	0.33 (0.01, 8.17)	0/2200	1/2197	11.11
NCT03036150	Dapagliflozin	Placebo	3.00 (0.12, 73.60)	1/2149	0/2149	11.11
NCT03619213	Dapagliflozin	Placebo	0.33 (0.01, 8.18)	0/3126	1/3127	11.11
Overall (I-squared = 0.0%, p = 0.910)			0.94 (0.32, 2.74)	6/29133	5/24023	100.00

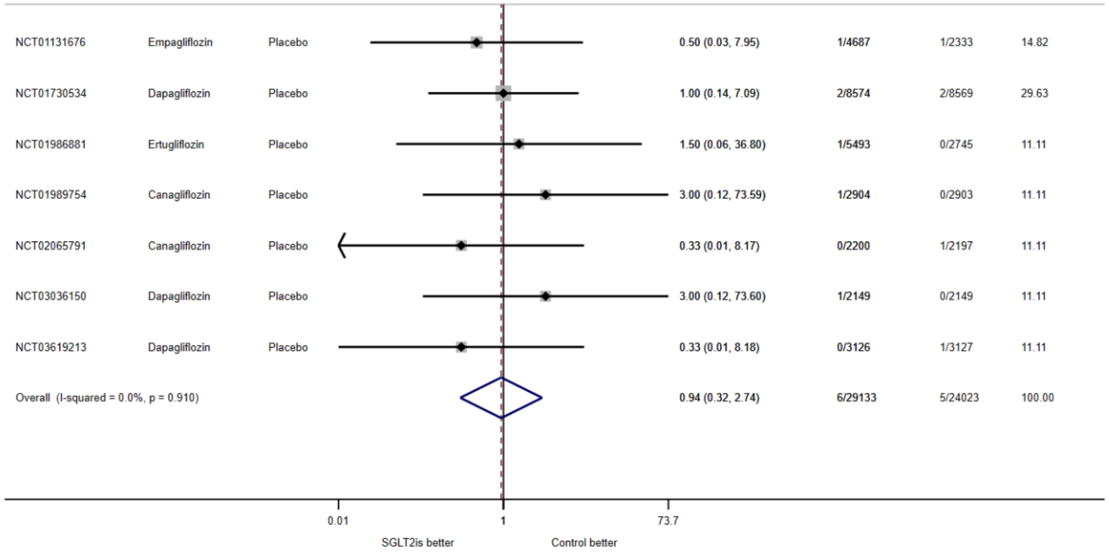

System 17-Nervous System Disorders-Figure S90 Meta-analysis of SGLT2is and Thalamic infarction

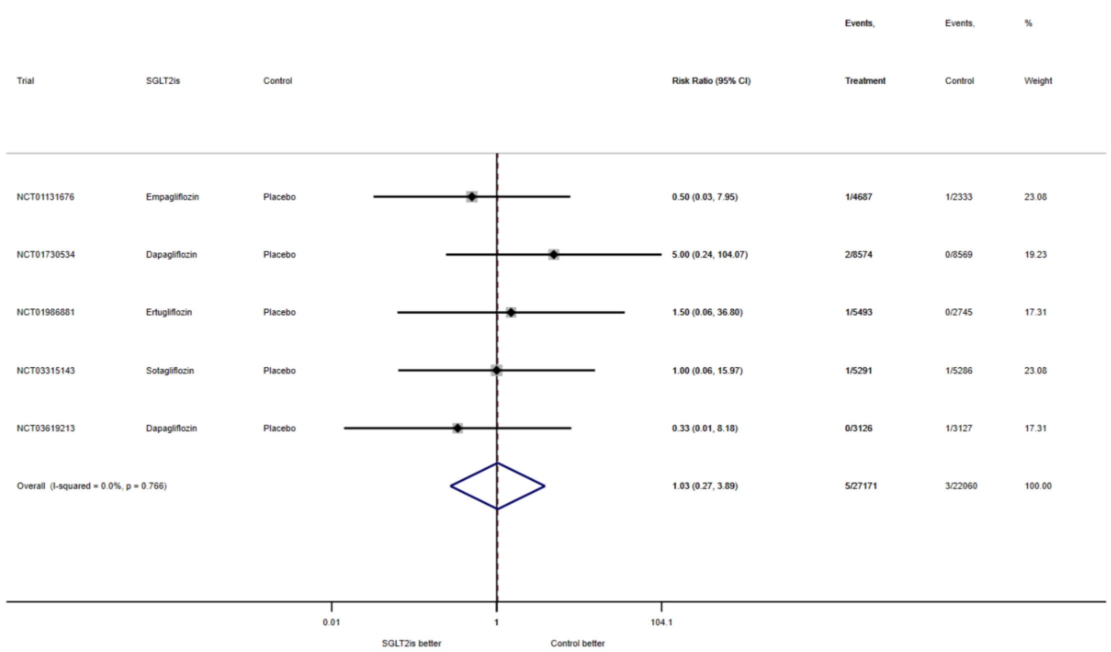

System 17-Nervous System Disorders-Figure S91 Meta-analysis of SGLT2is and Thrombotic cerebral infarction

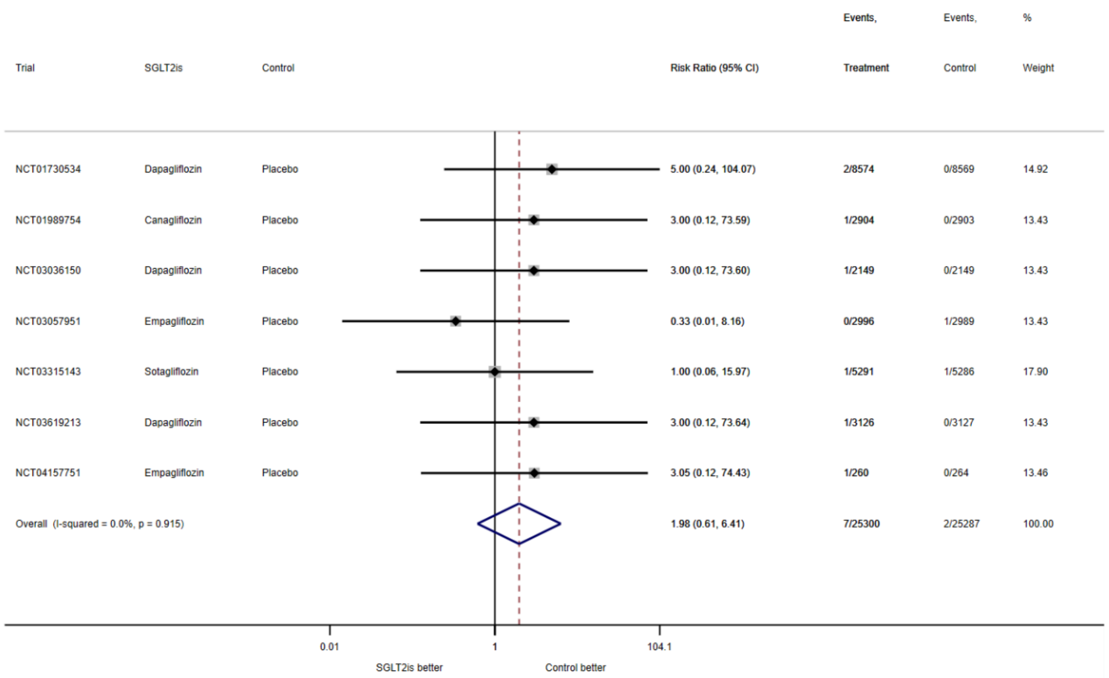

System 17-Nervous System Disorders-Figure S92 Meta-analysis of SGLT2is and Toxic encephalopathy

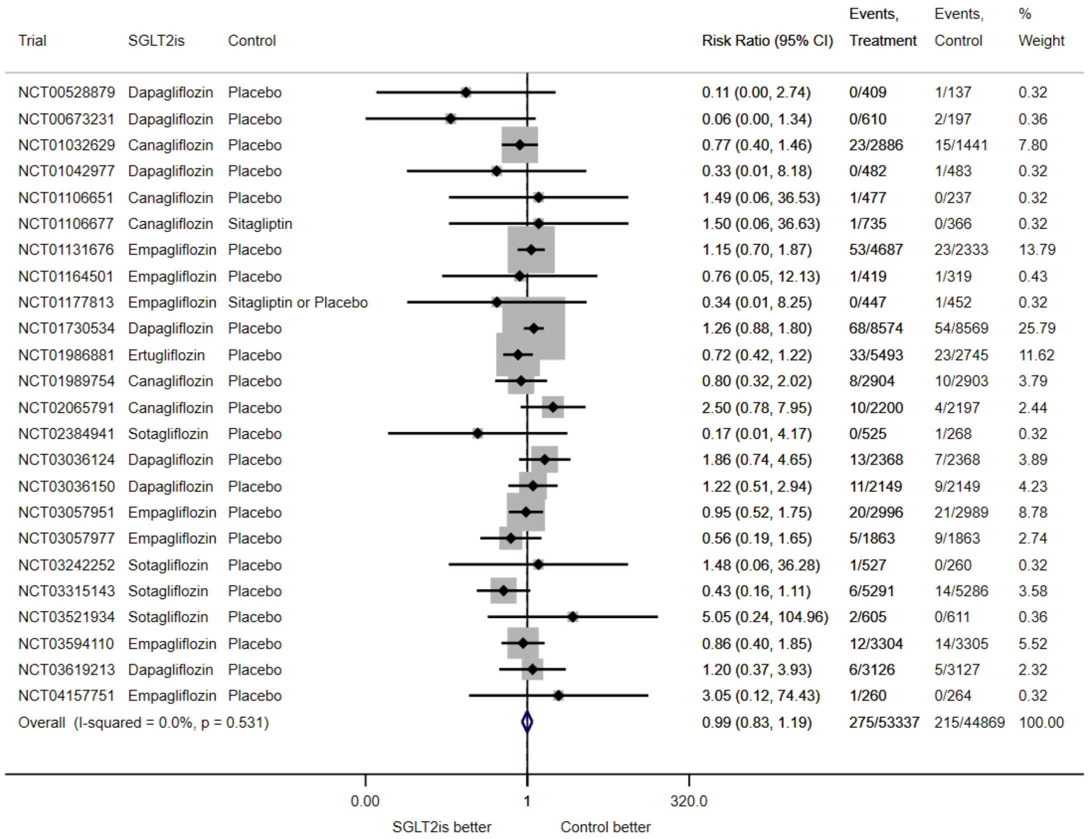

System 17-Nervous System Disorders-Figure S93 Meta-analysis of SGLT2is and Transient ischaemic attack

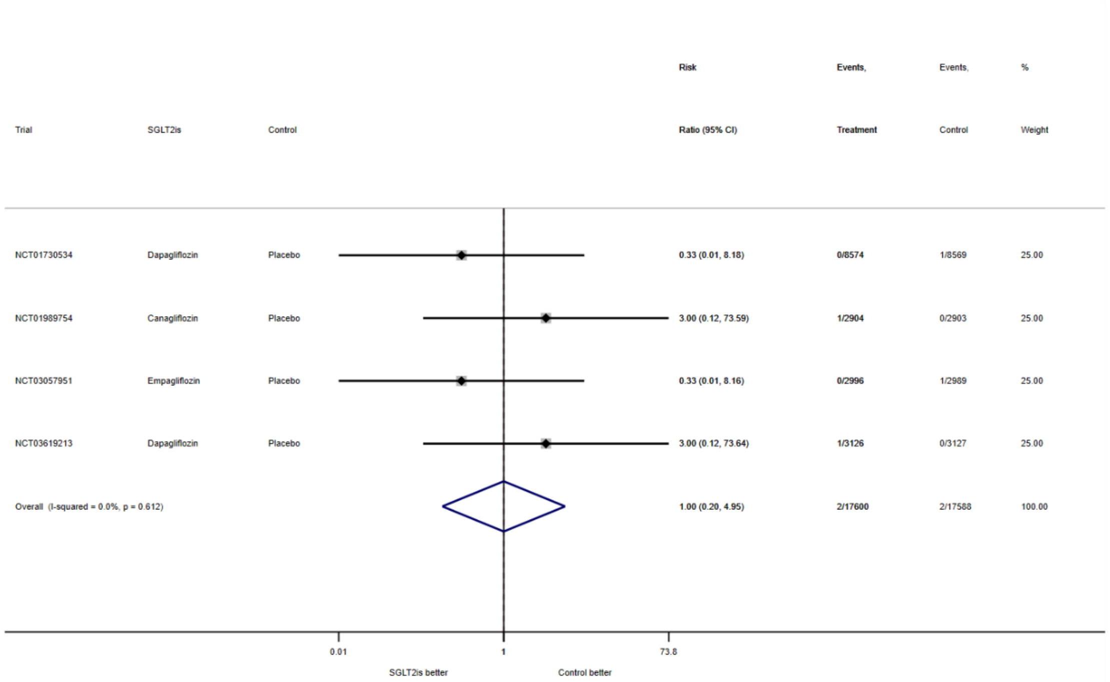

System 17-Nervous System Disorders-Figure S94 Meta-analysis of SGLT2is and Tremor

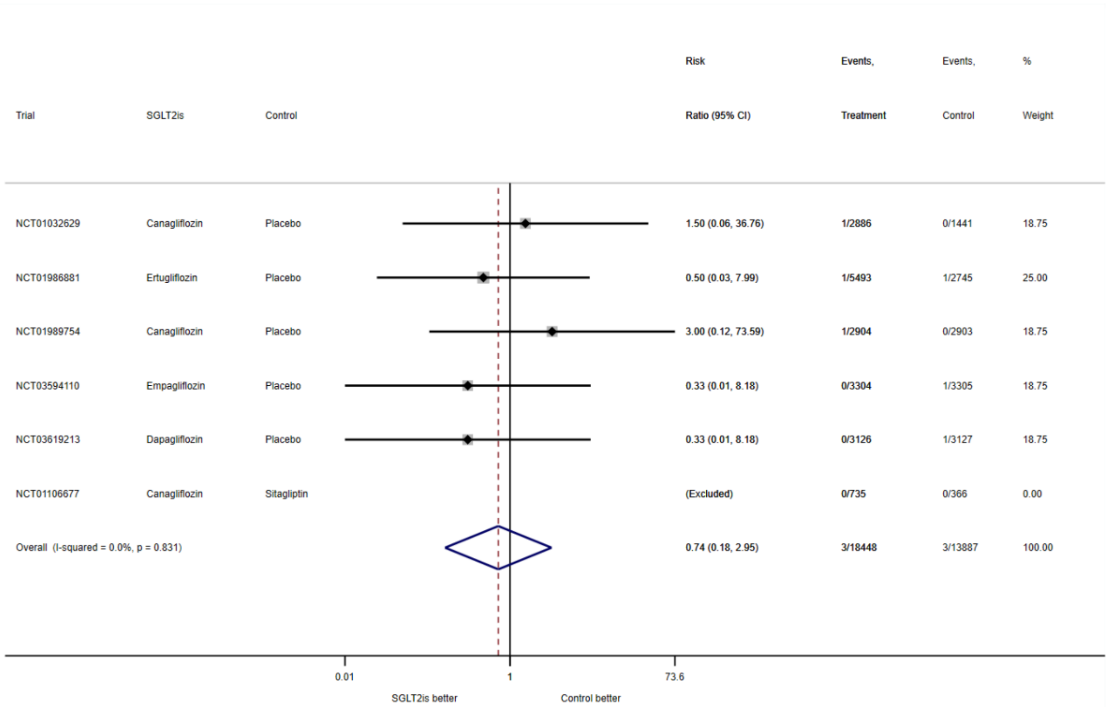

System 17-Nervous System Disorders-Figure S95 Meta-analysis of SGLT2is and Trigeminal neuralgia

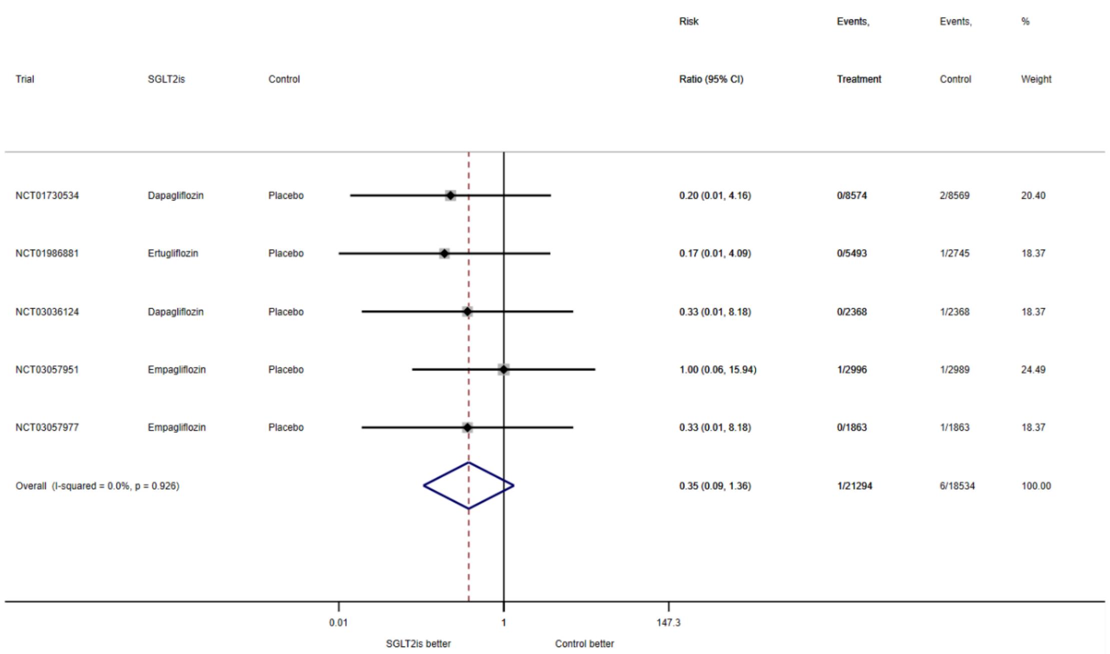

System 17-Nervous System Disorders-Figure S96 Meta-analysis of SGLT2is and Vascular dementia

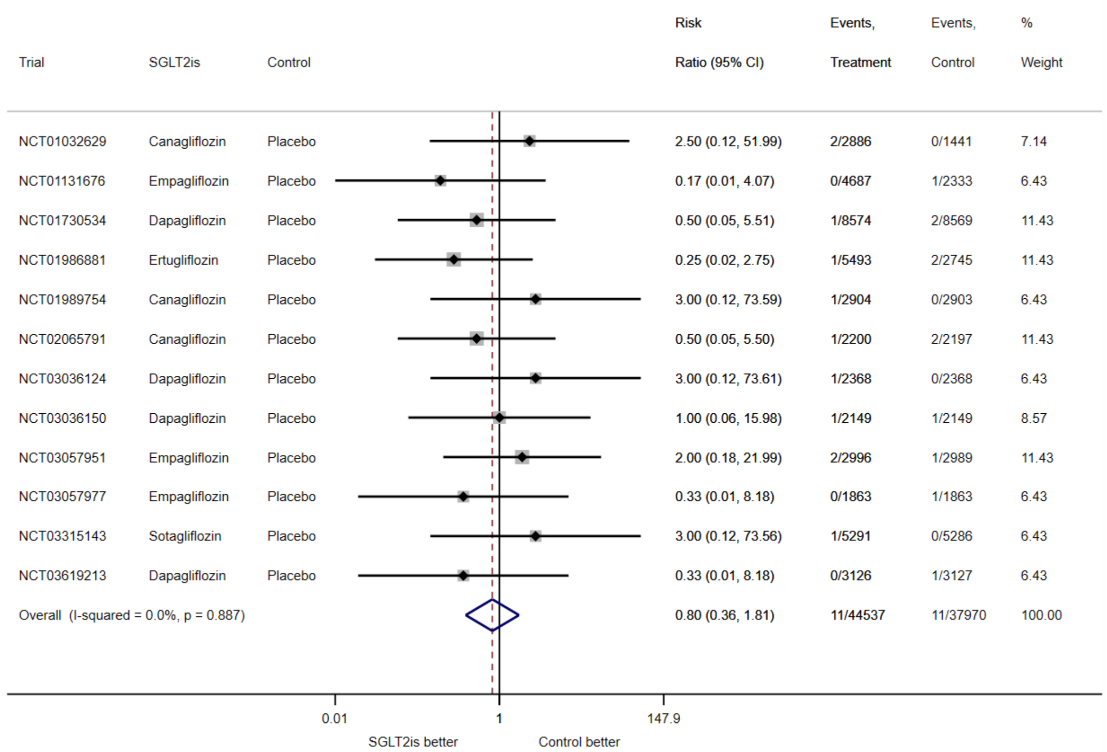

System 17-Nervous System Disorders-Figure S97 Meta-analysis of SGLT2is and Vascular encephalopathy

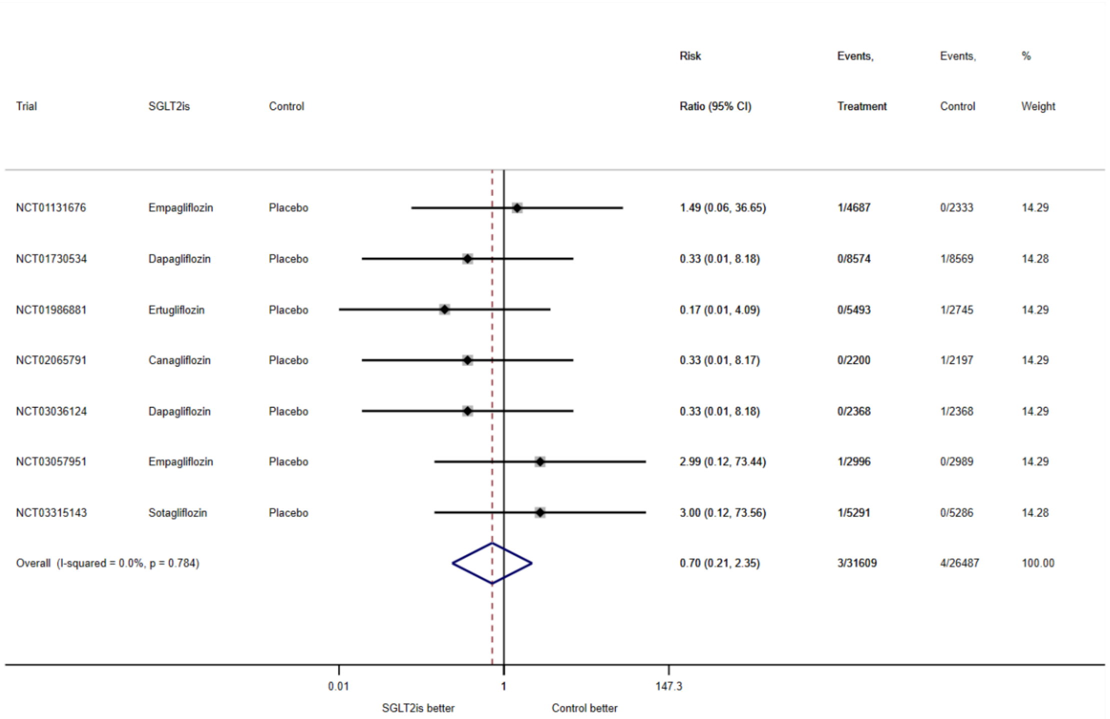

System 17-Nervous System Disorders-Figure S98 Meta-analysis of SGLT2is and Vertebral artery stenosis

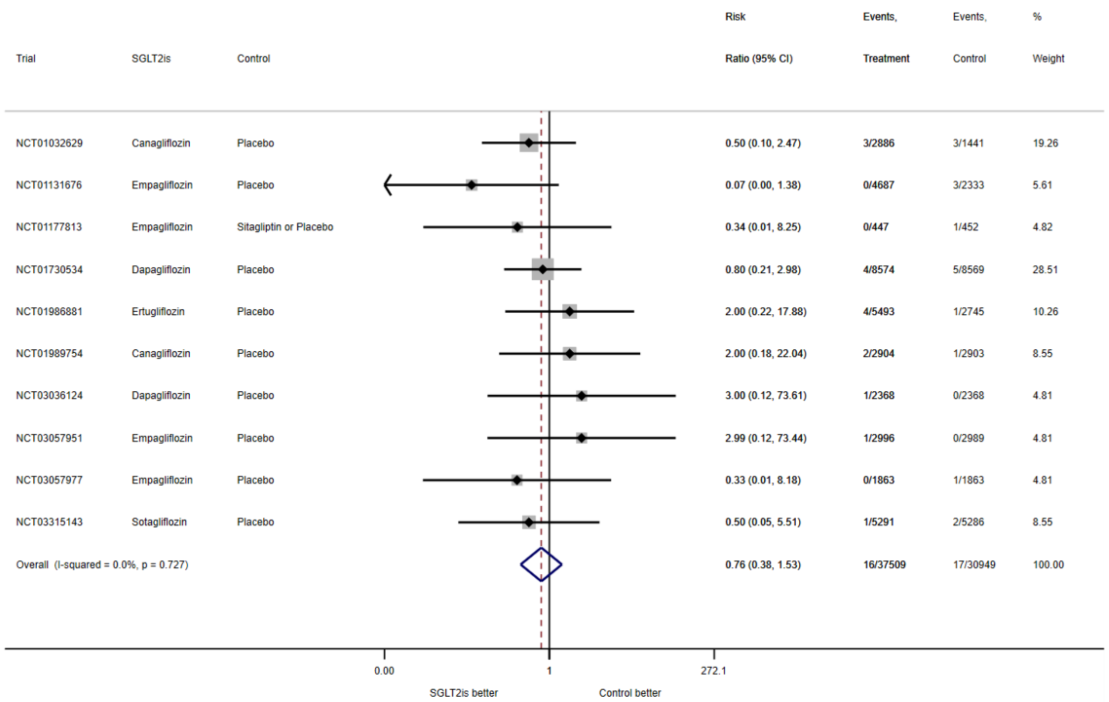

System 17-Nervous System Disorders-Figure S99 Meta-analysis of SGLT2is and Vertebrobasilar insufficiency

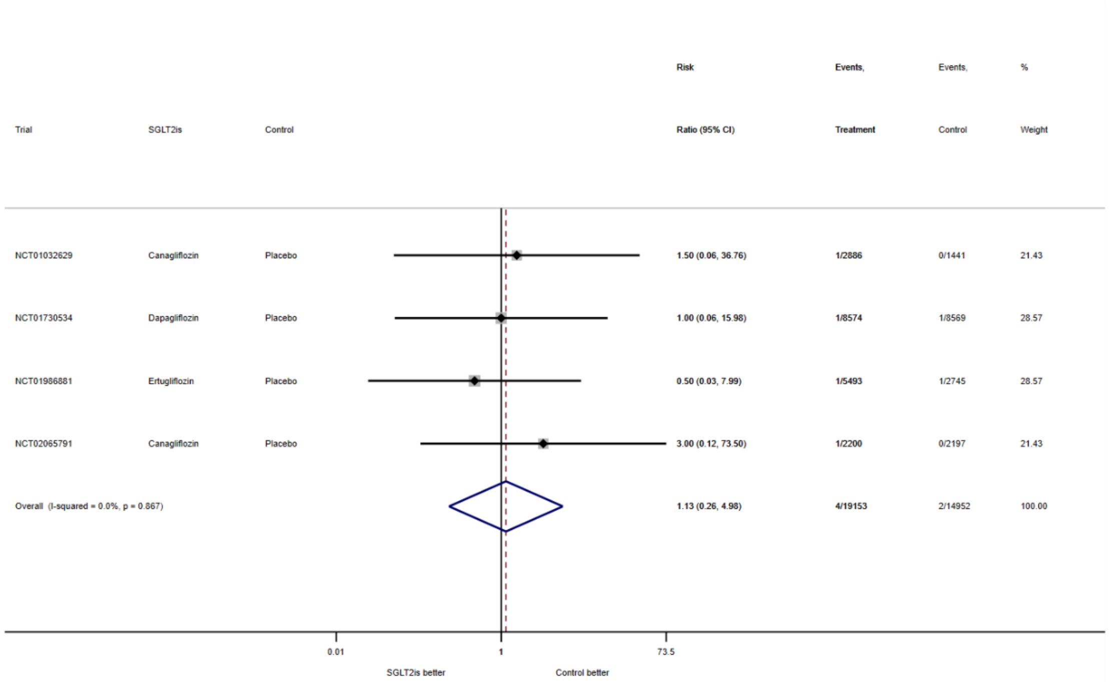

System 17-Nervous System Disorders-Figure S100 Meta-analysis of SGLT2is and Vith nerve paralysis

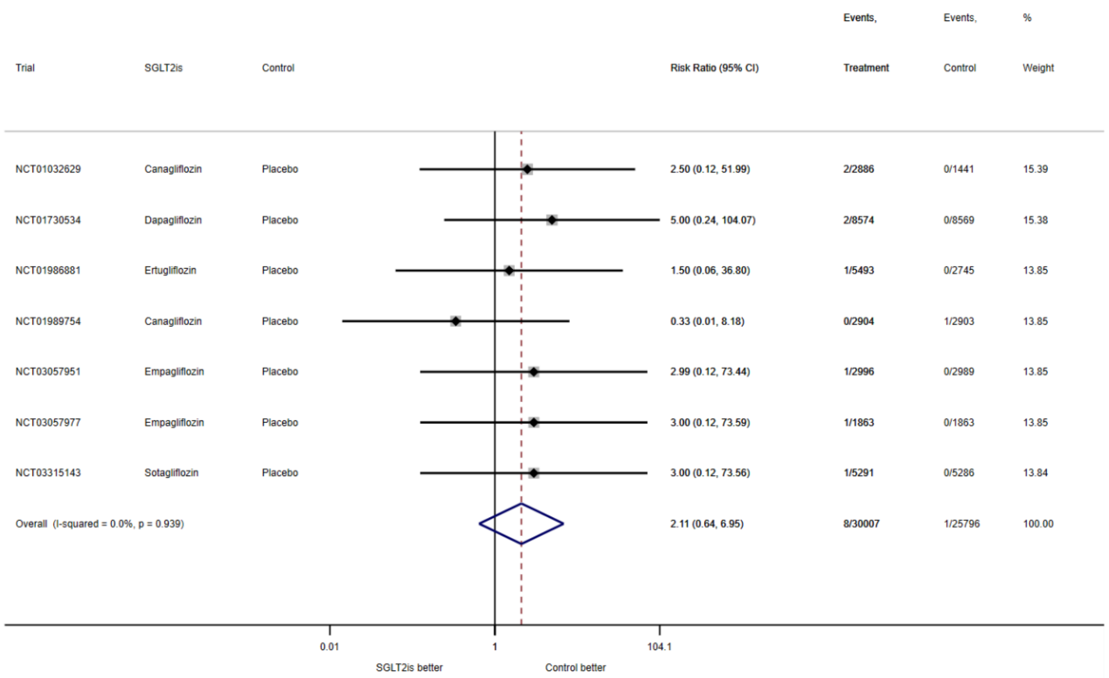

System 18-Product Issues-Figure S1 Meta-analysis of SGLT2is and Device breakage

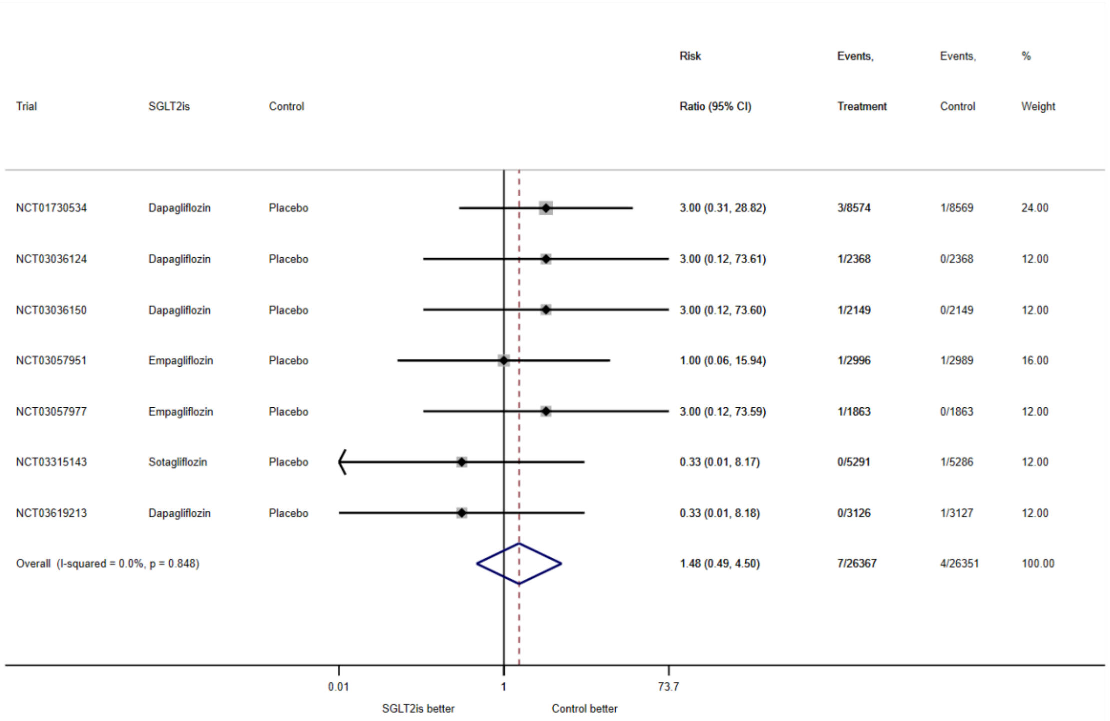

System 18-Product Issues-Figure S2 Meta-analysis of SGLT2is and Device dislocation

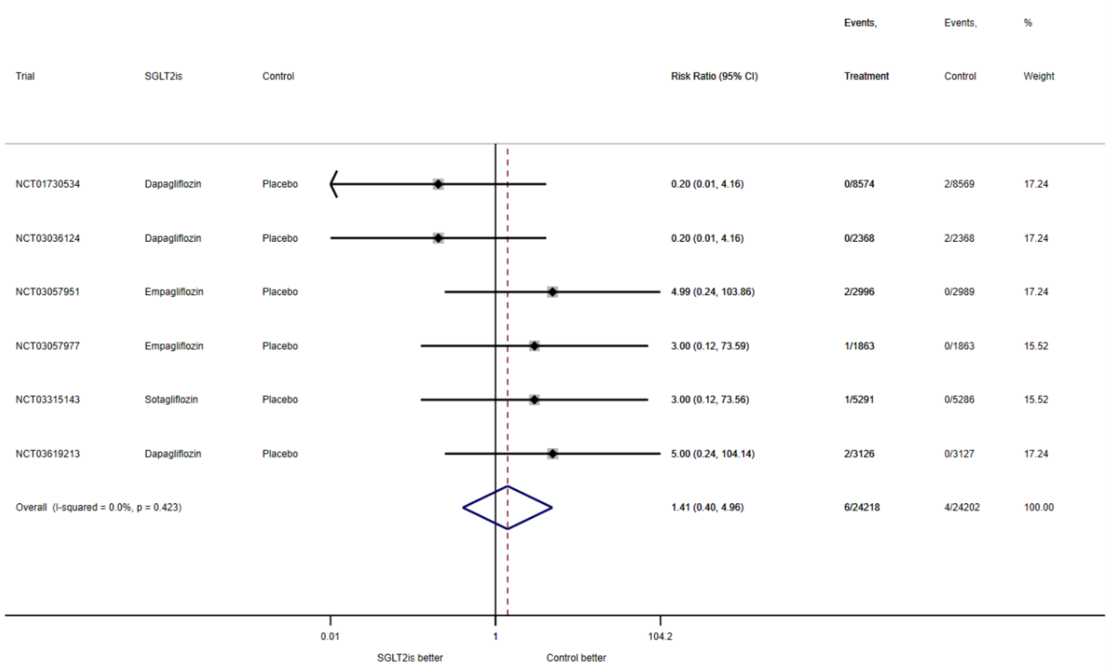

System 18-Product Issues-Figure S3 Meta-analysis of SGLT2is and Device failure

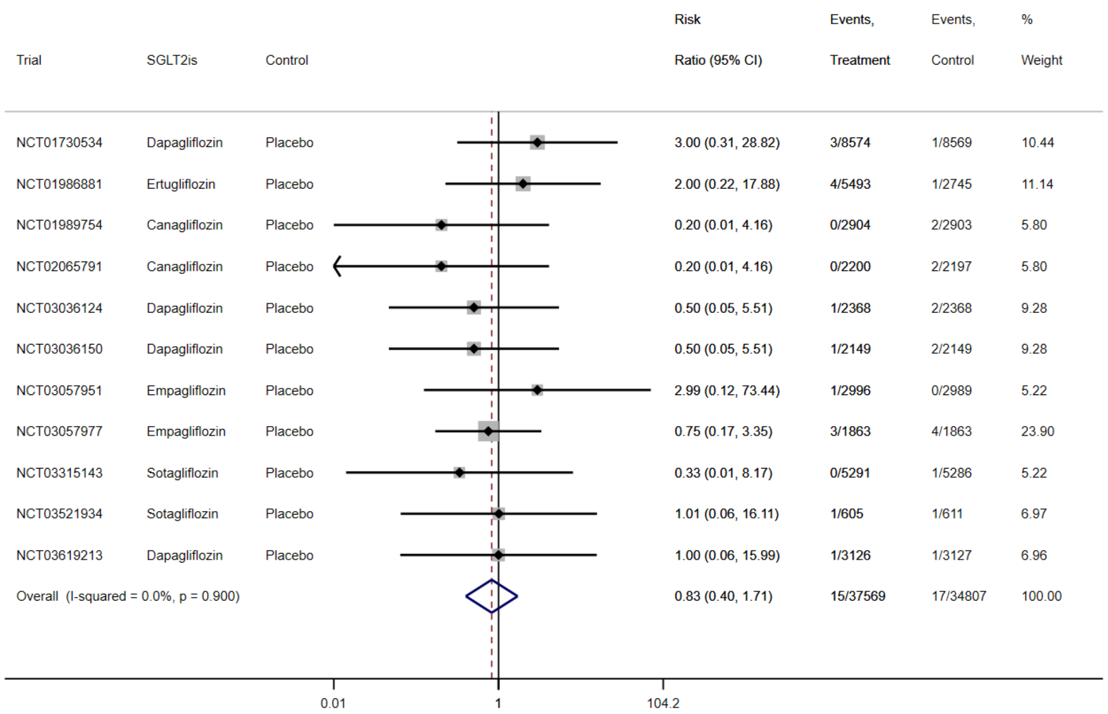

System 18-Product Issues-Figure S4 Meta-analysis of SGLT2is and Device malfunction

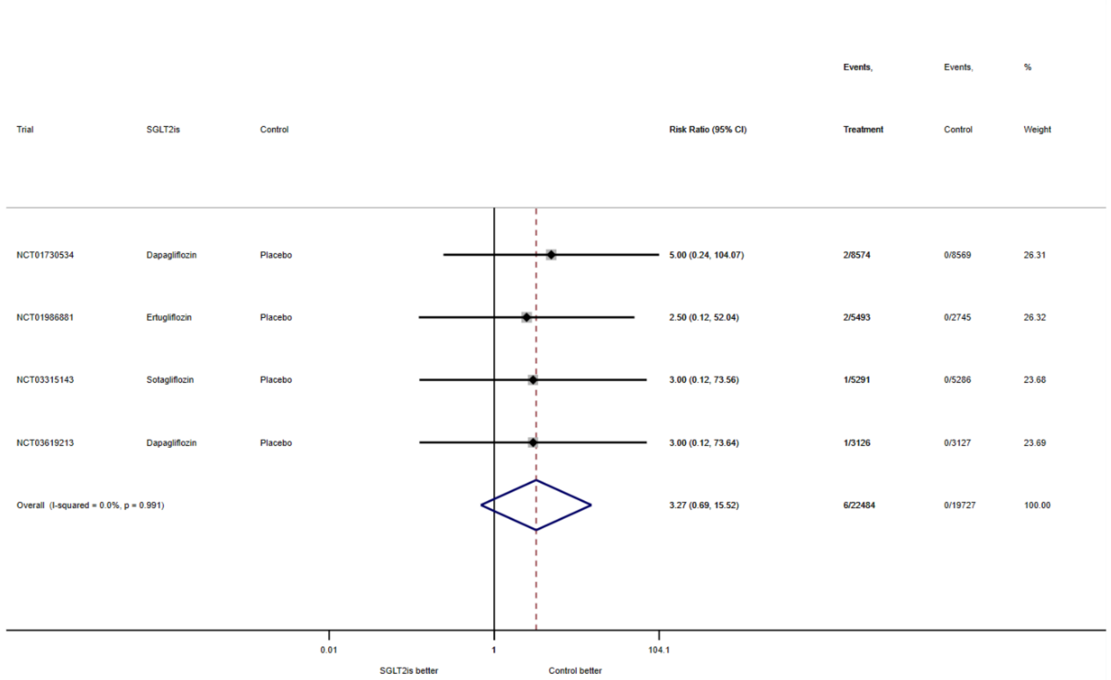

System 18-Product Issues-Figure S5 Meta-analysis of SGLT2is and Device occlusion

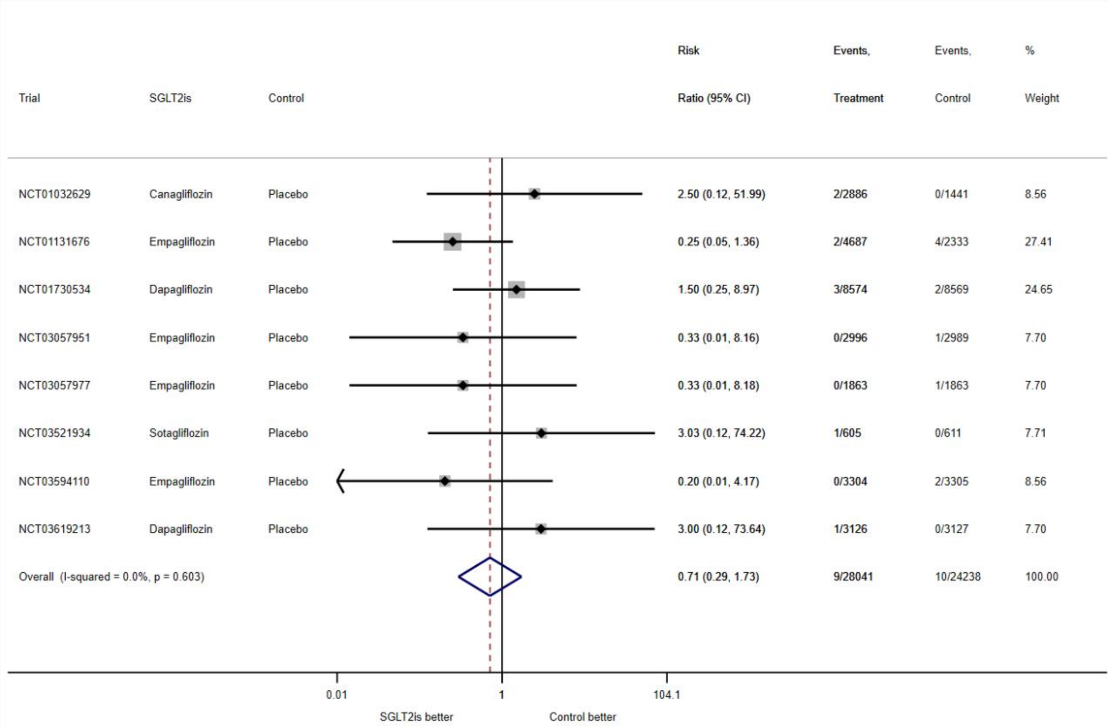

System 19-Psychiatric Disorders-Figure S1 Meta-analysis of SGLT2is and Anxiety

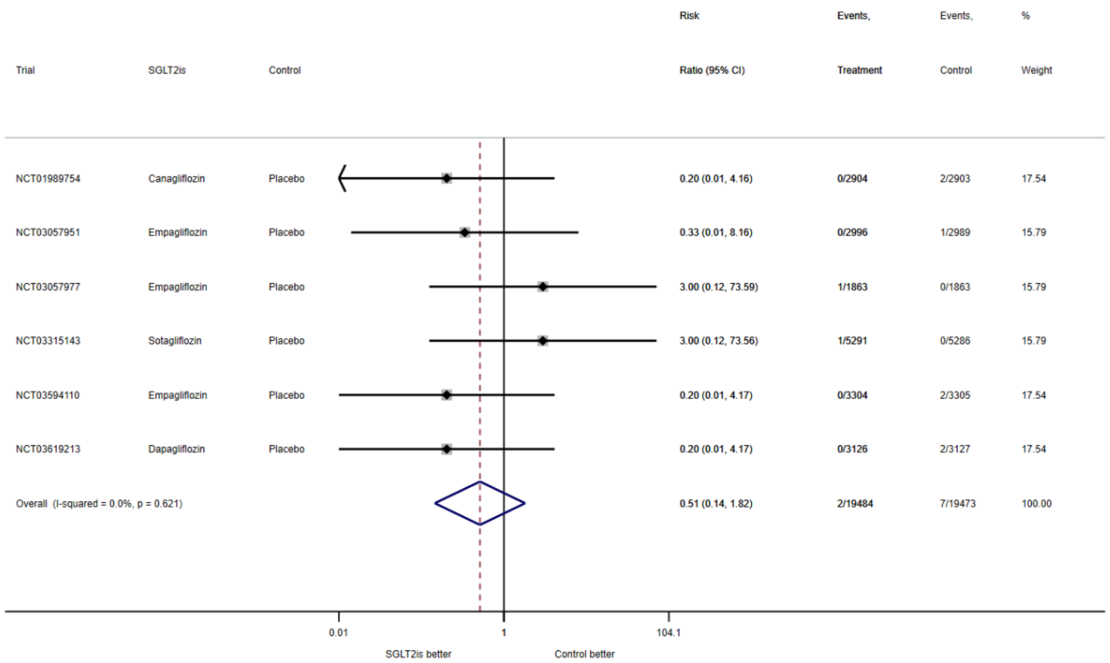

System 19-Psychiatric Disorders-Figure S2 Meta-analysis of SGLT2is and Bipolar disorder

Trial	SGLT2is	Control	Risk Ratio (95% CI)	Events, Treatment	Events, Control	% Weight
NCT01032629	Canagliflozin	Placebo	1.50 (0.06, 36.76)	1/2886	0/1441	7.29
NCT01131676	Empagliflozin	Placebo	1.49 (0.06, 36.65)	1/4687	0/2333	7.29
NCT01730534	Dapagliflozin	Placebo	1.00 (0.14, 7.09)	2/8574	2/8569	19.43
NCT01986881	Ertugliflozin	Placebo	0.10 (0.00, 2.08)	0/5493	2/2745	8.10
NCT01989754	Canagliflozin	Placebo	3.00 (0.12, 73.59)	1/2904	0/2903	7.29
NCT02065791	Canagliflozin	Placebo	1.00 (0.06, 15.96)	1/2200	1/2197	9.72
NCT03036124	Dapagliflozin	Placebo	0.20 (0.01, 4.16)	0/2368	2/2368	8.10
NCT03036150	Dapagliflozin	Placebo	0.33 (0.01, 8.18)	0/2149	1/2149	7.29
NCT03057951	Empagliflozin	Placebo	0.14 (0.01, 2.76)	0/2996	3/2989	8.50
NCT03057977	Empagliflozin	Placebo	3.00 (0.12, 73.59)	1/1863	0/1863	7.29
NCT03619213	Dapagliflozin	Placebo	1.00 (0.06, 15.99)	1/3126	1/3127	9.72
Overall (I-squared = 0.0%, p = 0.830)			0.71 (0.30, 1.68)	8/39246	12/32684	100.00

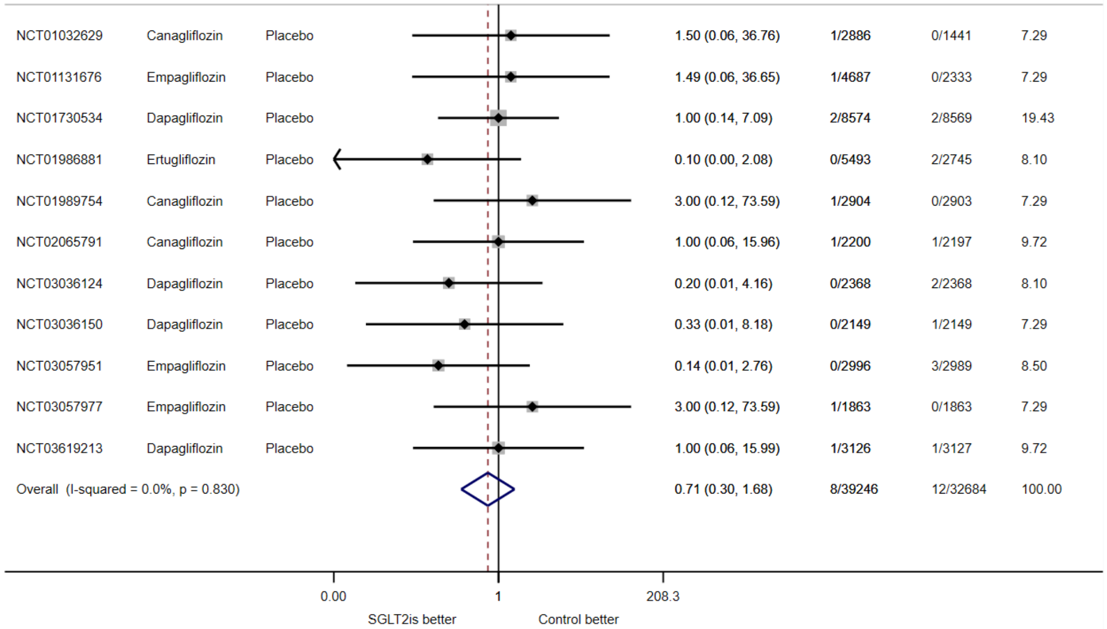

System 19-Psychiatric Disorders-Figure S3 Meta-analysis of SGLT2is and Completed suicide

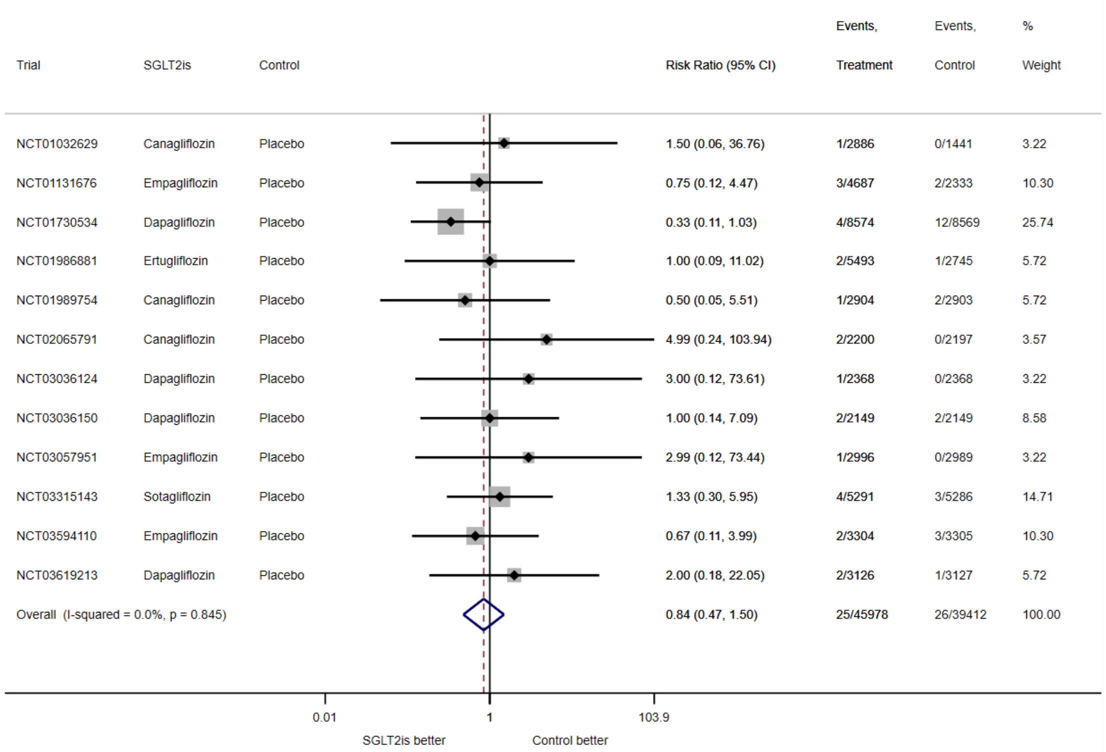

System 19-Psychiatric Disorders-Figure S4 Meta-analysis of SGLT2is and Confusional state

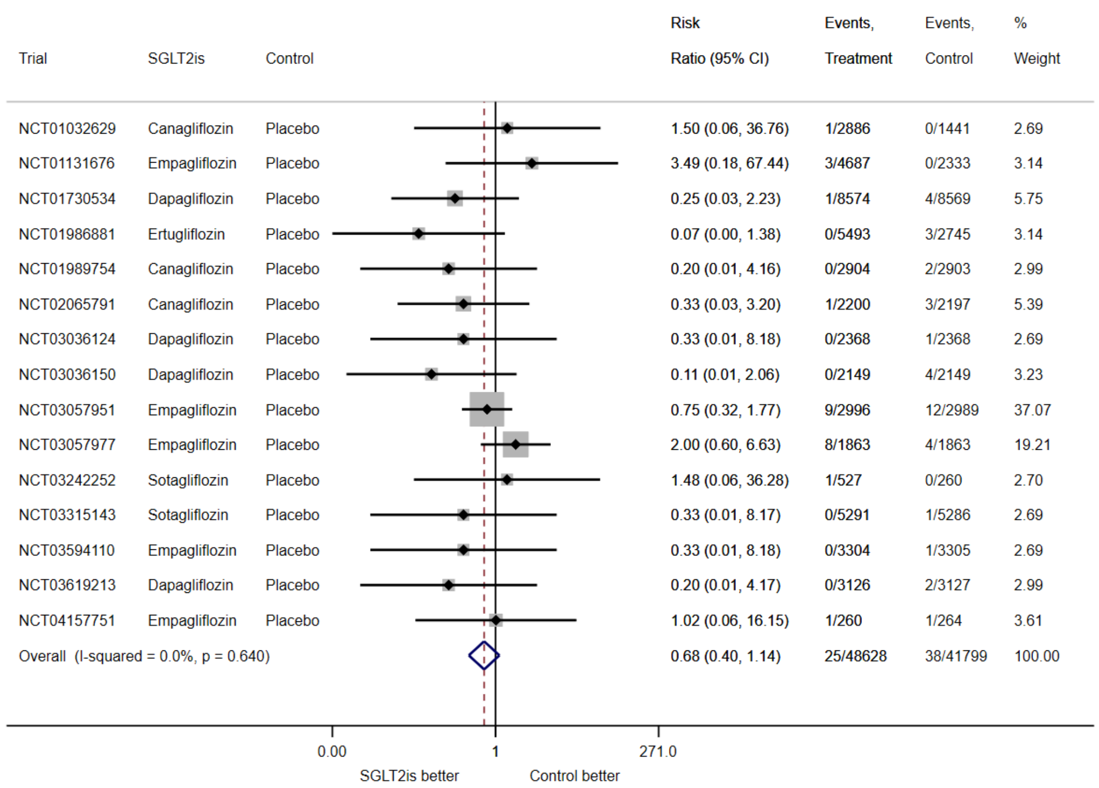

System 19-Psychiatric Disorders-Figure S5 Meta-analysis of SGLT2is and Delirium

Trial	SGLT2is	Control	Risk Ratio (95% CI)	Events, Treatment	Events, Control	% Weight
NCT01031680	Dapagliflozin	Placebo	3.01 (0.12, 73.77)	1/460	0/462	4.47
NCT01032629	Canagliflozin	Placebo	1.50 (0.16, 14.39)	3/2886	1/1441	8.94
NCT01131676	Empagliflozin	Placebo	0.20 (0.04, 1.03)	2/4687	5/2333	17.03
NCT01730534	Dapagliflozin	Placebo	0.15 (0.03, 0.68)	2/8574	13/8569	20.65
NCT01986881	Ertugliflozin	Placebo	2.50 (0.12, 52.04)	2/5493	0/2745	4.96
NCT01989754	Canagliflozin	Placebo	0.33 (0.01, 8.18)	0/2904	1/2903	4.47
NCT03036124	Dapagliflozin	Placebo	3.00 (0.12, 73.61)	1/2368	0/2368	4.47
NCT03036150	Dapagliflozin	Placebo	3.00 (0.12, 73.60)	1/2149	0/2149	4.47
NCT03057951	Empagliflozin	Placebo	1.00 (0.06, 15.94)	1/2996	1/2989	5.96
NCT03057977	Empagliflozin	Placebo	0.33 (0.01, 8.18)	0/1863	1/1863	4.47
NCT03315143	Sotagliflozin	Placebo	1.00 (0.06, 15.97)	1/5291	1/5286	5.96
NCT03521934	Sotagliflozin	Placebo	0.14 (0.01, 2.79)	0/605	3/611	5.22
NCT03594110	Empagliflozin	Placebo	3.00 (0.12, 73.64)	1/3304	0/3305	4.47
NCT03619213	Dapagliflozin	Placebo	0.33 (0.01, 8.18)	0/3126	1/3127	4.47
Overall (I-squared = 0.0%, p = 0.549)			0.53 (0.27, 1.05)	15/46706	27/40151	100.00

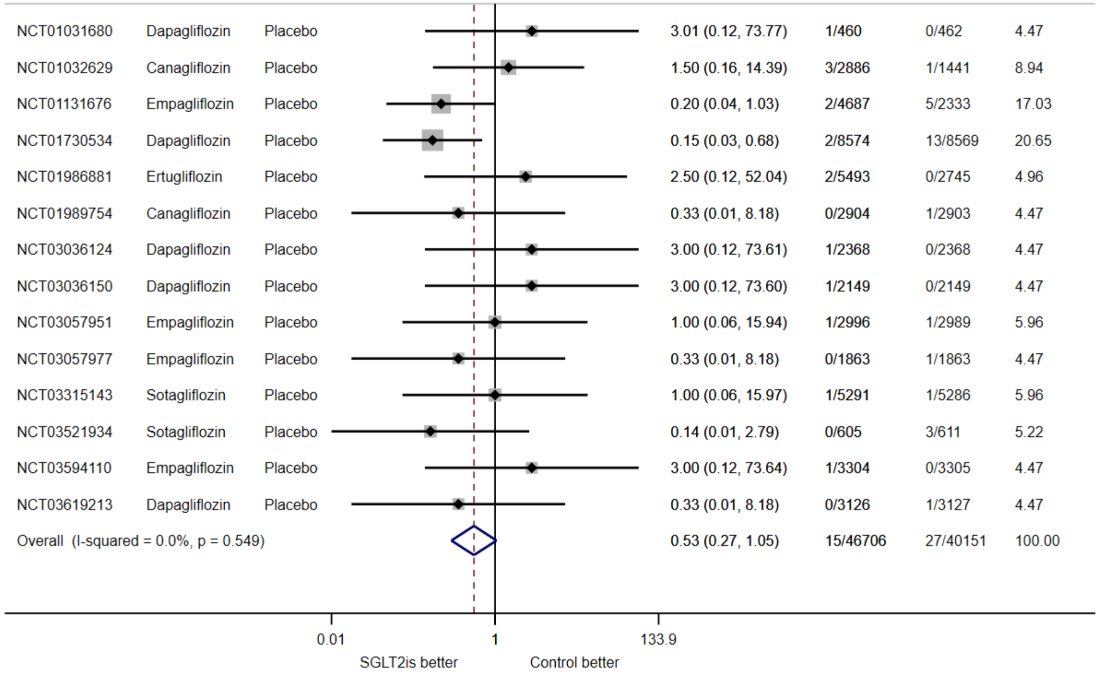

System 19-Psychiatric Disorders-Figure S6 Meta-analysis of SGLT2is and Depression

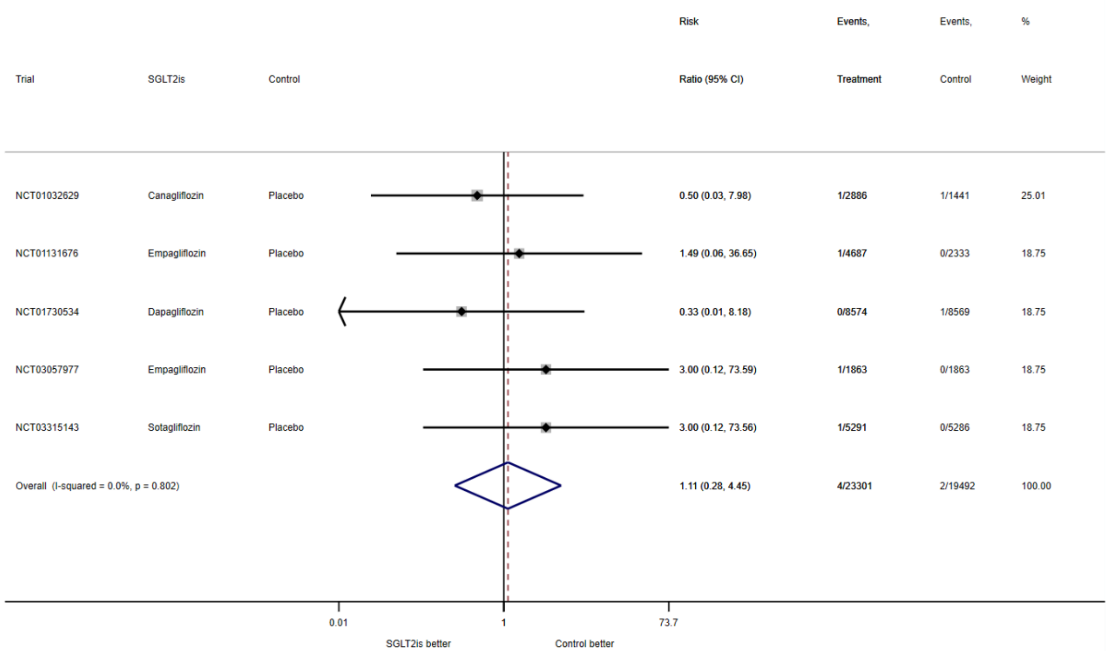

System 19-Psychiatric Disorders-Figure S7 Meta-analysis of SGLT2is and Hallucination

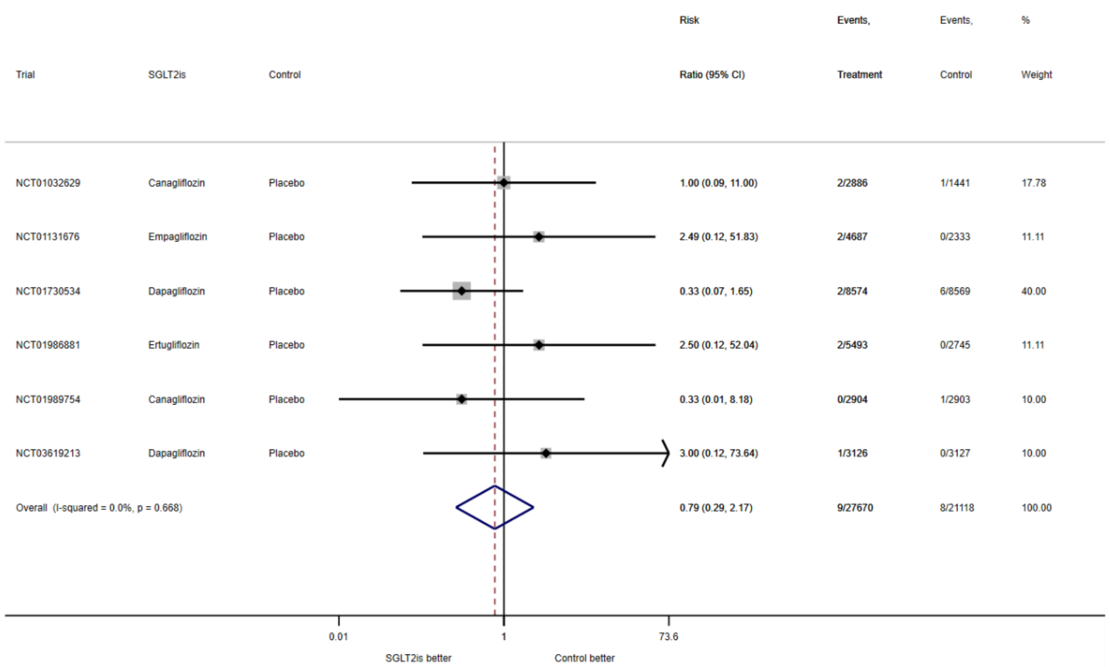

System 19-Psychiatric Disorders-Figure S8 Meta-analysis of SGLT2is and Major depression

Trial	SGLT2is	Control	Risk Ratio (95% CI)	Events, Treatment	Events, Control	% Weight
NCT01032629	Canagliflozin	Placebo	0.37 (0.08, 1.67)	3/2886	4/1441	16.57
NCT01131676	Empagliflozin	Placebo	0.25 (0.05, 1.36)	2/4687	4/2333	12.88
NCT01730534	Dapagliflozin	Placebo	0.71 (0.23, 2.25)	5/8574	7/8569	28.16
NCT01986881	Ertugliflozin	Placebo	0.50 (0.03, 7.99)	1/5493	1/2745	4.83
NCT02065791	Canagliflozin	Placebo	0.20 (0.01, 4.16)	0/2200	2/2197	4.02
NCT02384941	Sotagliflozin	Placebo	1.53 (0.06, 37.53)	1/525	0/268	3.63
NCT03036124	Dapagliflozin	Placebo	0.50 (0.05, 5.51)	1/2368	2/2368	6.44
NCT03315143	Sotagliflozin	Placebo	0.33 (0.01, 8.17)	0/5291	1/5286	3.62
NCT03619213	Dapagliflozin	Placebo	0.29 (0.06, 1.37)	2/3126	7/3127	15.02
NCT04157751	Empagliflozin	Placebo	1.02 (0.06, 16.15)	1/260	1/264	4.84
Overall (I-squared = 0.0%, p = 0.975)			0.45 (0.25, 0.83)	16/35410	29/28598	100.00

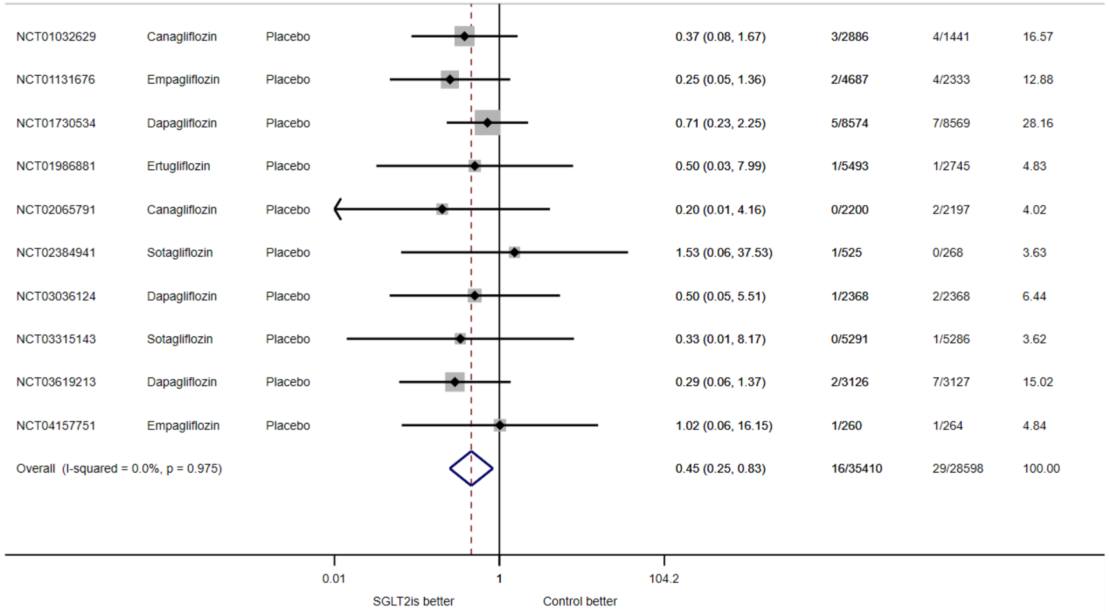

System 19-Psychiatric Disorders-Figure S9 Meta-analysis of SGLT2is and Mental status changes

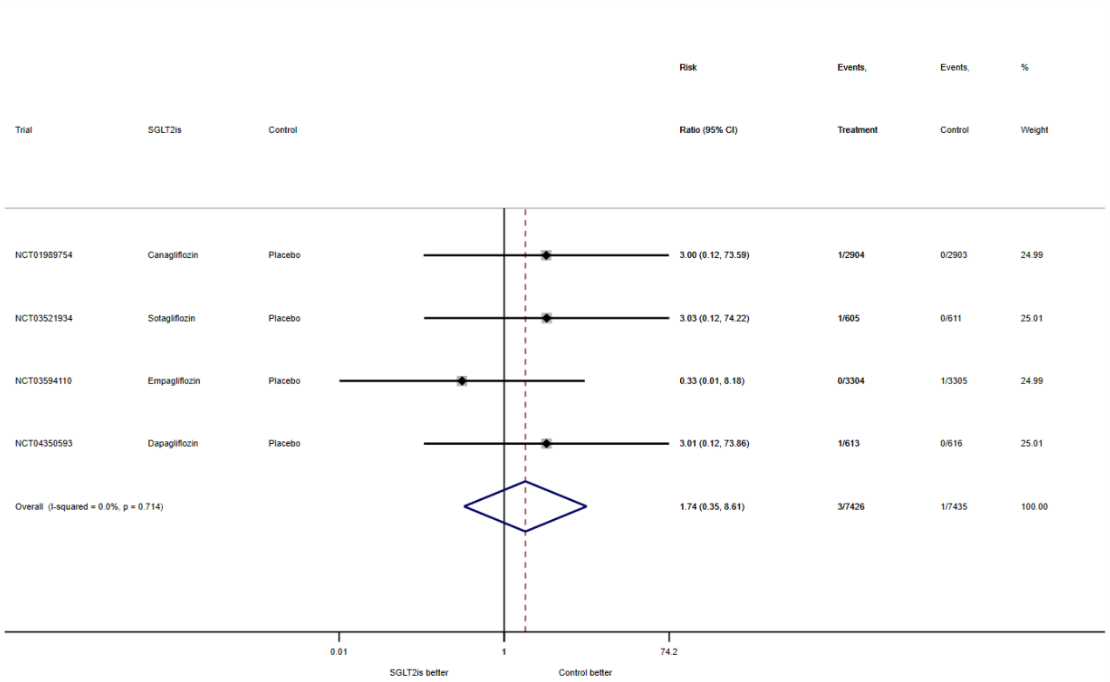

System 19-Psychiatric Disorders-Figure S10 Meta-analysis of SGLT2is and Psychotic disorder

Trial	SGLT2is	Control	Risk Ratio (95% CI)	Events, Treatment	Events, Control	% Weight
NCT01131676	Empagliflozin	Placebo	1.49 (0.06, 36.65)	1/4687	0/2333	11.61
NCT01730534	Dapagliflozin	Placebo	0.20 (0.01, 4.16)	0/8574	2/8569	12.90
NCT01986881	Ertugliflozin	Placebo	0.50 (0.03, 7.99)	1/5493	1/2745	15.48
NCT01989754	Canagliflozin	Placebo	0.33 (0.01, 8.18)	0/2904	1/2903	11.61
NCT03036150	Dapagliflozin	Placebo	7.00 (0.36, 135.44)	3/2149	0/2149	13.55
NCT03315143	Sotagliflozin	Placebo	0.33 (0.01, 8.17)	0/5291	1/5286	11.61
NCT03521934	Sotagliflozin	Placebo	3.03 (0.12, 74.22)	1/605	0/611	11.62
NCT03619213	Dapagliflozin	Placebo	3.00 (0.12, 73.64)	1/3126	0/3127	11.61
Overall (I-squared = 0.0%, p = 0.677)			1.00 (0.33, 2.96)	7/32829	5/27723	100.00

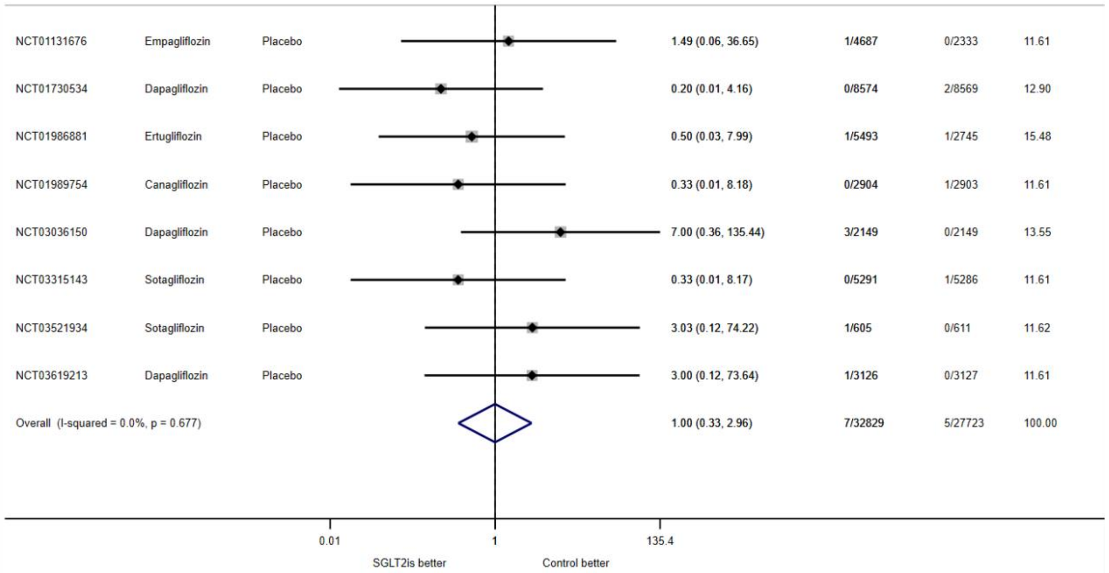

System 19-Psychiatric Disorders-Figure S11 Meta-analysis of SGLT2is and Suicidal ideation

Trial	SGLT2is	Control	Risk Ratio (95% CI)	Events, Treatment	Events, Control	% Weight
NCT01032629	Canagliflozin	Placebo	0.50 (0.03, 7.98)	1/2886	1/1441	9.60
NCT01131676	Empagliflozin	Placebo	1.00 (0.09, 10.97)	2/4687	1/2333	12.80
NCT01730534	Dapagliflozin	Placebo	0.50 (0.05, 5.51)	1/8574	2/8569	12.80
NCT01986881	Ertugliflozin	Placebo	0.17 (0.01, 4.09)	0/5493	1/2745	7.20
NCT01989754	Canagliflozin	Placebo	3.00 (0.12, 73.59)	1/2904	0/2903	7.20
NCT02065791	Canagliflozin	Placebo	3.00 (0.12, 73.50)	1/2200	0/2197	7.20
NCT03036124	Dapagliflozin	Placebo	3.00 (0.12, 73.61)	1/2368	0/2368	7.20
NCT03036150	Dapagliflozin	Placebo	3.00 (0.12, 73.60)	1/2149	0/2149	7.20
NCT03057951	Empagliflozin	Placebo	2.99 (0.12, 73.44)	1/2996	0/2989	7.20
NCT03594110	Empagliflozin	Placebo	0.33 (0.01, 8.18)	0/3304	1/3305	7.20
NCT03619213	Dapagliflozin	Placebo	0.33 (0.01, 8.18)	0/3126	1/3127	7.20
NCT04157751	Empagliflozin	Placebo	0.34 (0.01, 8.27)	0/260	1/264	7.22
Overall (I-squared = 0.0%, p = 0.916)			0.88 (0.37, 2.08)	9/40947	8/34390	100.00

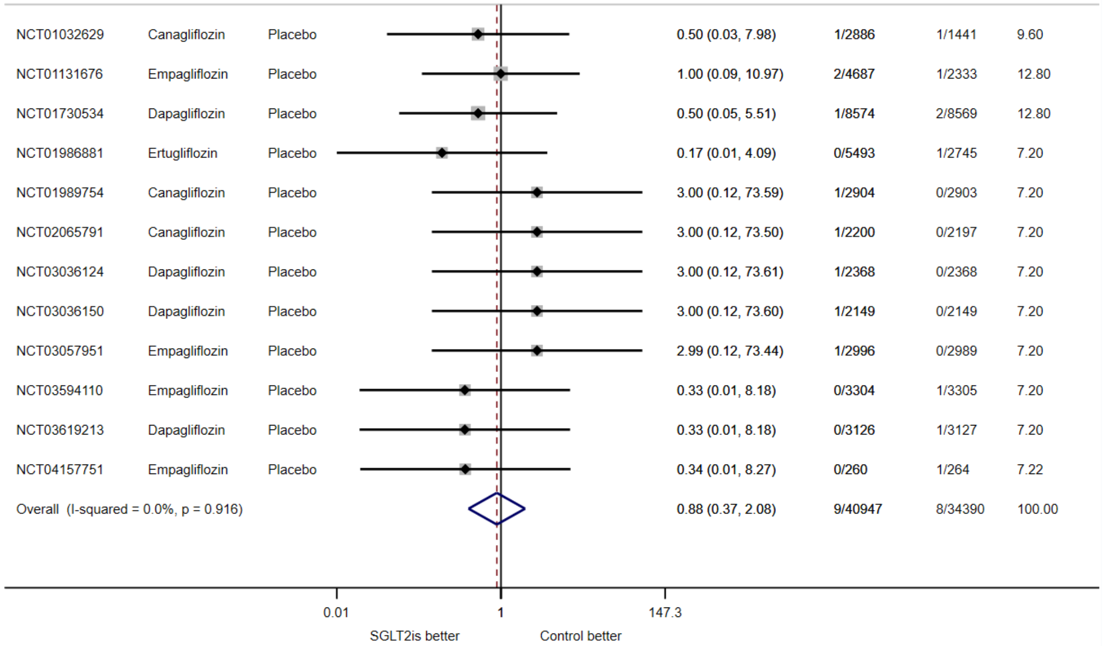

System 19-Psychiatric Disorders-Figure S12 Meta-analysis of SGLT2is and Suicide attempt

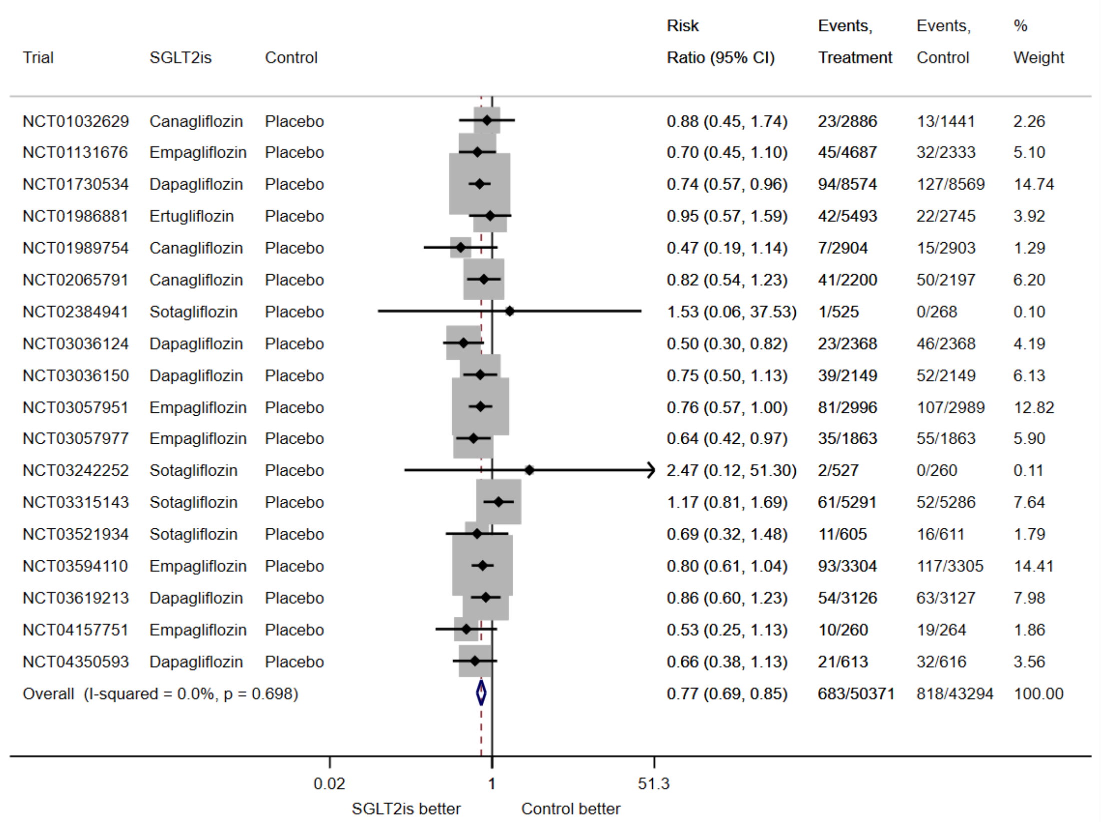

System 20-Renal And Urinary Disorders-Figure S1 Meta-analysis of SGLT2is and Acute kidney injury

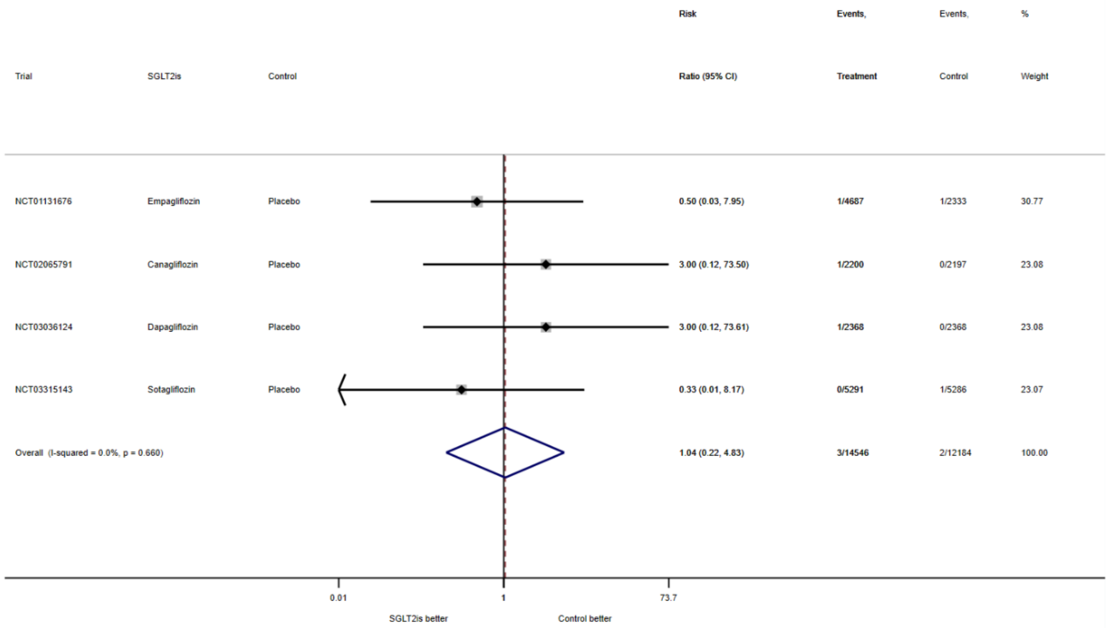

System 20-Renal And Urinary Disorders-Figure S2 Meta-analysis of SGLT2is and Anuria

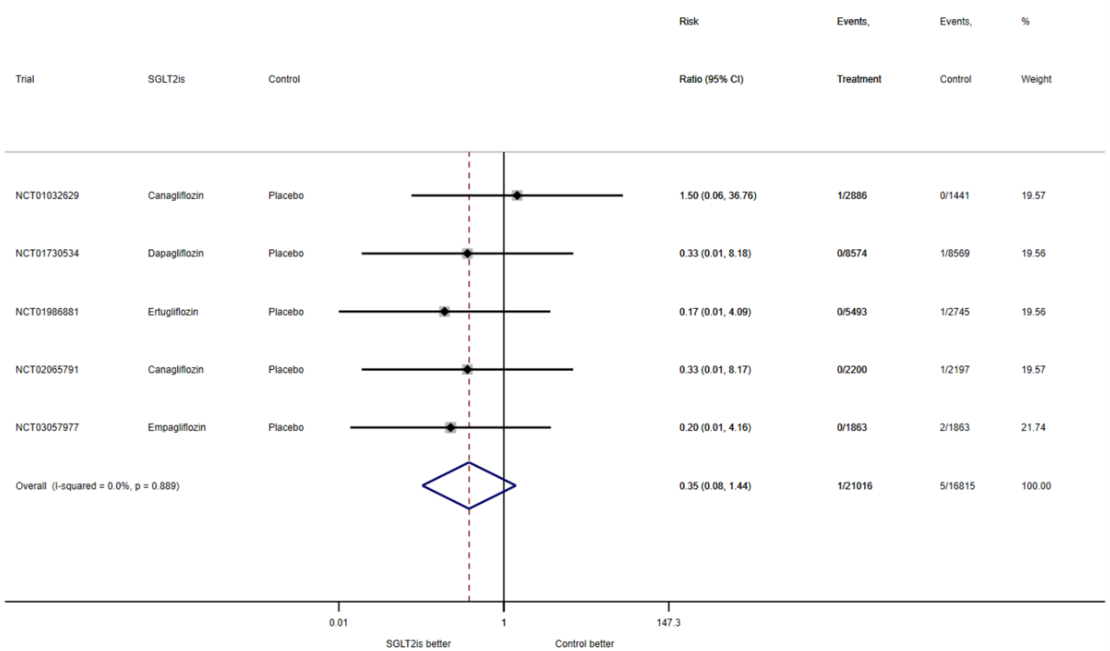

System 20-Renal And Urinary Disorders-Figure S3 Meta-analysis of SGLT2is and Azotaemia

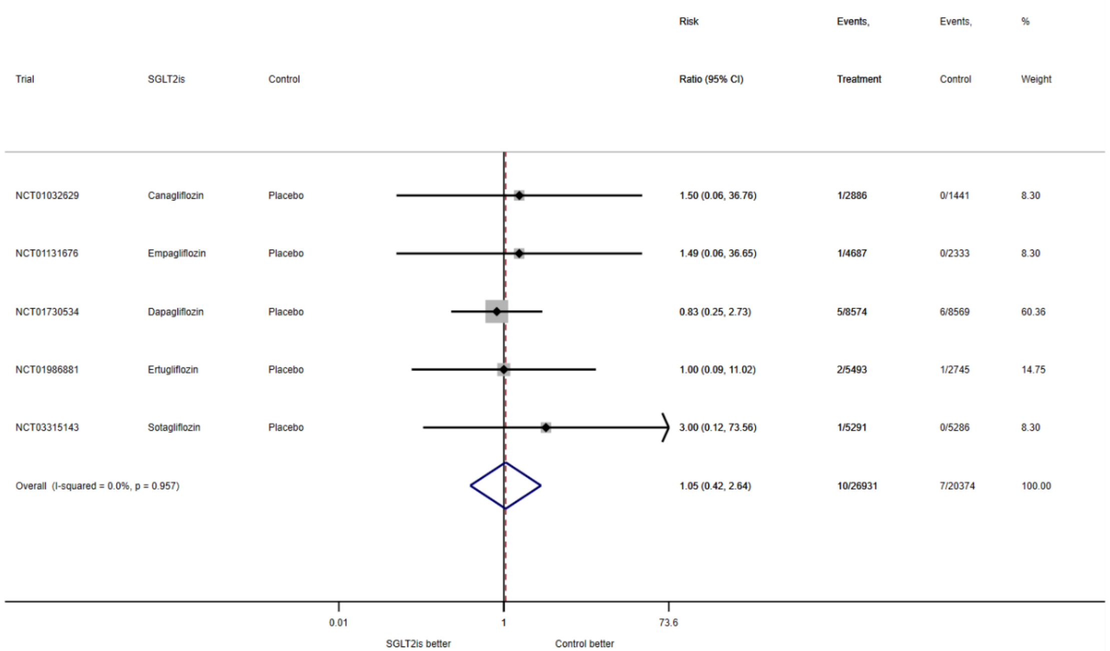

System 20-Renal And Urinary Disorders-Figure S4 Meta-analysis of SGLT2is and Calculus bladder

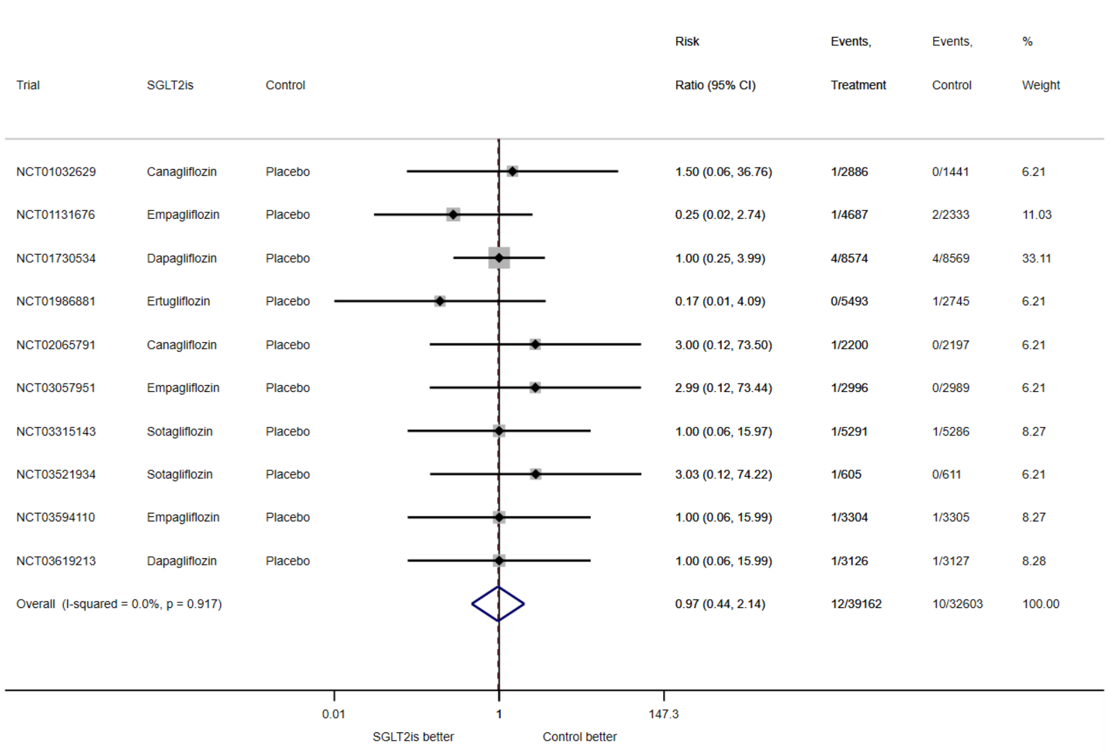

System 20-Renal And Urinary Disorders-Figure S5 Meta-analysis of SGLT2is and Calculus urinary

Trial	SGLT2is	Control	Risk Ratio (95% CI)	Events, Treatment	Events, Control	% Weight
NCT01032629	Canagliflozin	Placebo	0.50 (0.07, 3.54)	2/2886	2/1441	1.63
NCT01131676	Empagliflozin	Placebo	1.00 (0.25, 3.98)	6/4687	3/2333	3.26
NCT01730534	Dapagliflozin	Placebo	0.69 (0.30, 1.62)	9/8574	13/8569	8.67
NCT01986881	Ertugliflozin	Placebo	0.87 (0.26, 2.98)	7/5493	4/2745	4.15
NCT01989754	Canagliflozin	Placebo	1.50 (0.25, 8.97)	3/2904	2/2903	1.96
NCT02065791	Canagliflozin	Placebo	1.00 (0.45, 2.22)	12/2200	12/2197	9.83
NCT03036124	Dapagliflozin	Placebo	0.50 (0.15, 1.66)	4/2368	8/2368	4.35
NCT03036150	Dapagliflozin	Placebo	0.65 (0.37, 1.13)	20/2149	31/2149	20.03
NCT03057951	Empagliflozin	Placebo	0.64 (0.34, 1.19)	16/2996	25/2989	15.99
NCT03057977	Empagliflozin	Placebo	0.50 (0.13, 2.00)	3/1863	6/1863	3.26
NCT03242252	Sotagliflozin	Placebo	0.99 (0.09, 10.83)	2/527	1/260	1.09
NCT03315143	Sotagliflozin	Placebo	0.78 (0.39, 1.56)	14/5291	18/5286	12.86
NCT03521934	Sotagliflozin	Placebo	0.50 (0.13, 2.01)	3/605	6/611	3.28
NCT03619213	Dapagliflozin	Placebo	1.20 (0.52, 2.77)	12/3126	10/3127	8.92
NCT04157751	Empagliflozin	Placebo	0.15 (0.01, 2.79)	0/260	3/264	0.71
Overall (I-squared = 0.0%, p = 0.977)			0.74 (0.57, 0.95)	113/45929	144/39105	100.00

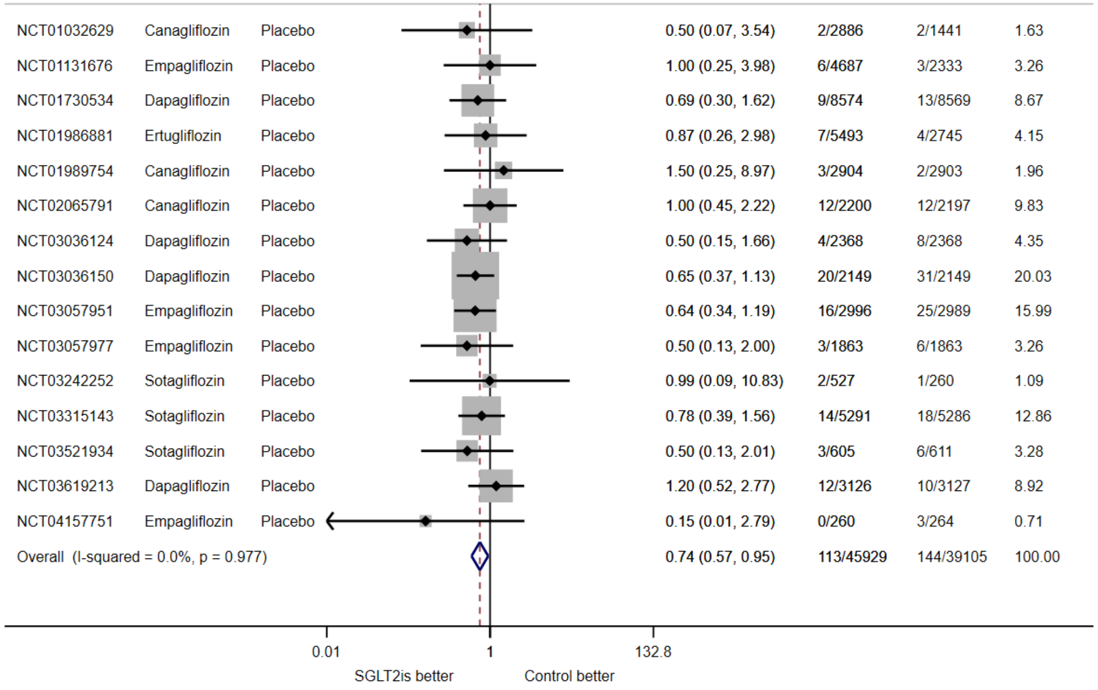

System 20-Renal And Urinary Disorders-Figure S6 Meta-analysis of SGLT2is and Chronic kidney disease

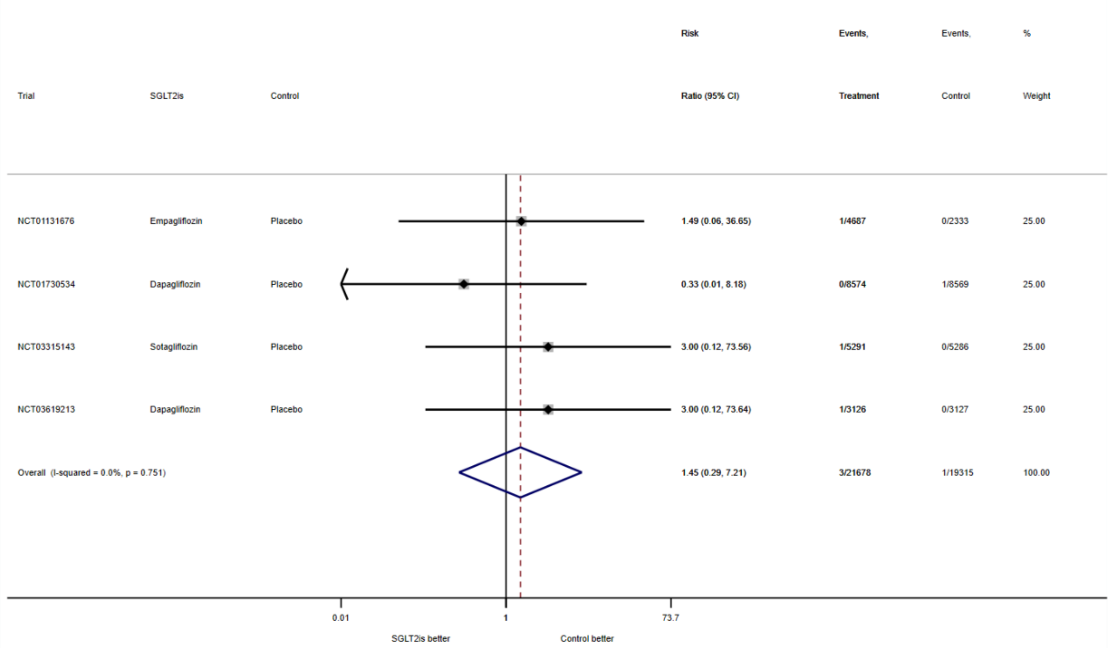

System 20-Renal And Urinary Disorders-Figure S7 Meta-analysis of SGLT2is and Cystitis glandularis

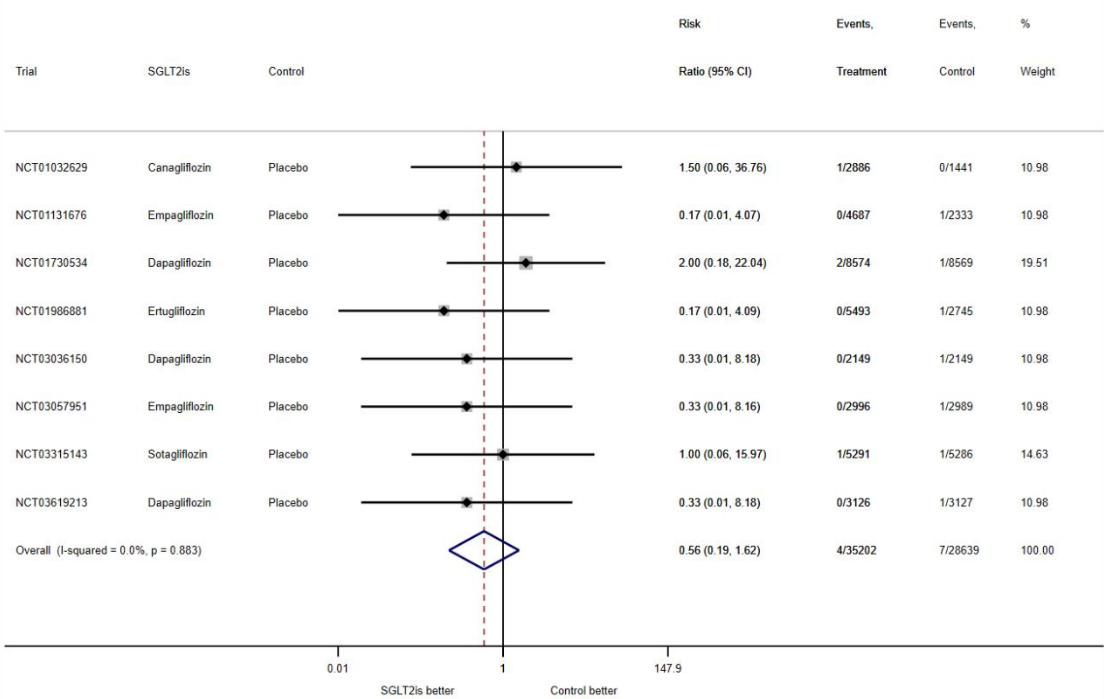

System 20-Renal And Urinary Disorders-Figure S8 Meta-analysis of SGLT2is and Cystitis haemorrhagic

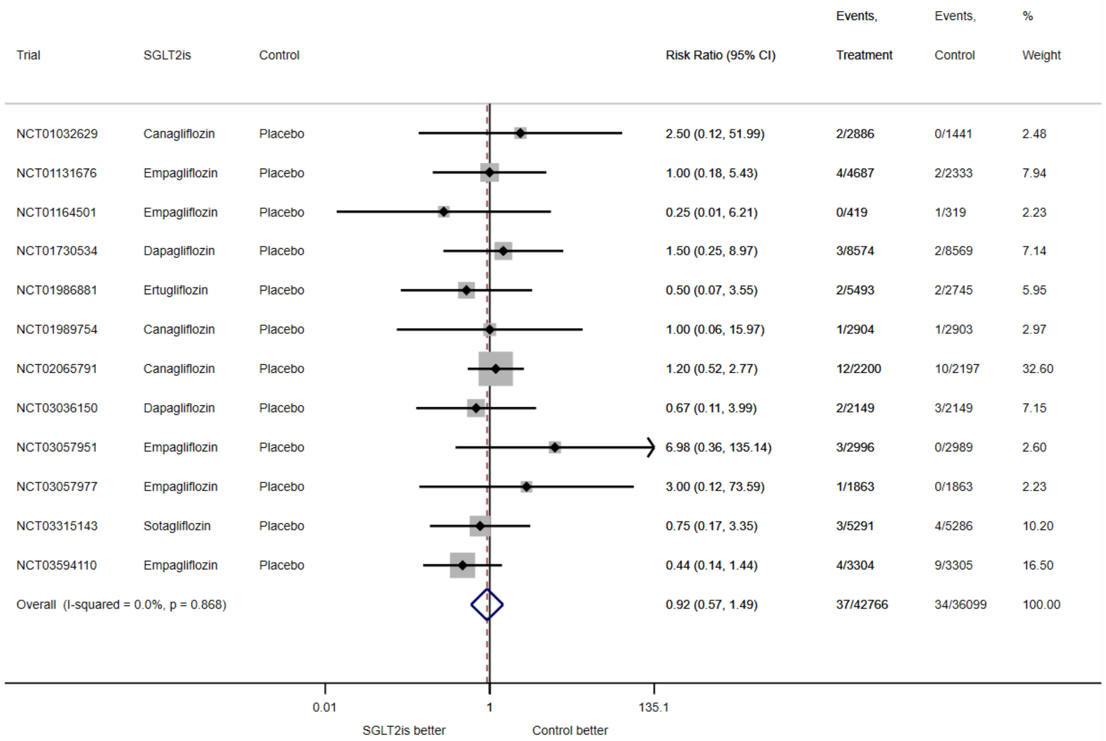

System 20-Renal And Urinary Disorders-Figure S9 Meta-analysis of SGLT2is and Diabetic nephropathy

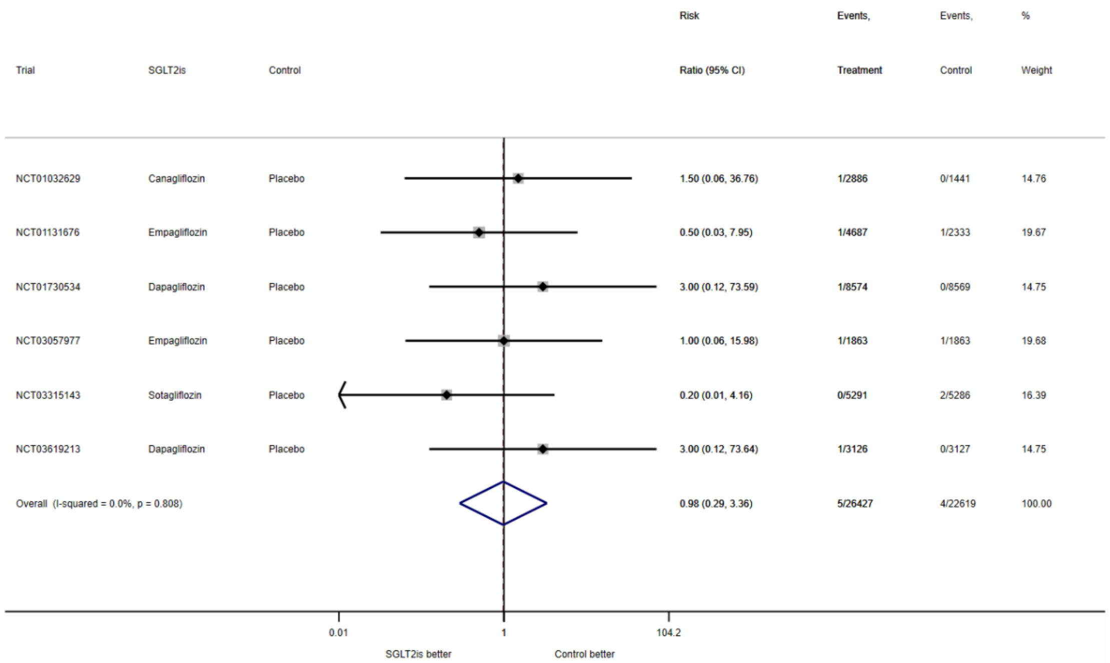

System 20-Renal And Urinary Disorders-Figure S10 Meta-analysis of SGLT2is and Dysuria

Trial	SGLT2is	Control	Risk Ratio (95% CI)	Events, Treatment	Events, Control	% Weight
NCT01730534	Dapagliflozin	Placebo	0.43 (0.11, 1.66)	3/8574	7/8569	4.47
NCT01986881	Ertugliflozin	Placebo	0.17 (0.01, 4.09)	0/5493	1/2745	0.80
NCT02065791	Canagliflozin	Placebo	0.69 (0.37, 1.31)	16/2200	23/2197	20.26
NCT03036124	Dapagliflozin	Placebo	2.00 (0.18, 22.04)	2/2368	1/2368	1.42
NCT03036150	Dapagliflozin	Placebo	0.72 (0.44, 1.19)	26/2149	36/2149	32.59
NCT03057951	Empagliflozin	Placebo	1.00 (0.06, 15.94)	1/2996	1/2989	1.06
NCT03057977	Empagliflozin	Placebo	0.33 (0.01, 8.18)	0/1863	1/1863	0.80
NCT03242252	Sotagliflozin	Placebo	0.16 (0.01, 4.03)	0/527	1/260	0.80
NCT03315143	Sotagliflozin	Placebo	0.33 (0.01, 8.17)	0/5291	1/5286	0.80
NCT03594110	Empagliflozin	Placebo	1.30 (0.79, 2.14)	35/3304	27/3305	32.74
NCT03619213	Dapagliflozin	Placebo	1.00 (0.25, 4.00)	4/3126	4/3127	4.26
Overall (I-squared = 0.0%, p = 0.654)			0.84 (0.63, 1.12)	87/37891	103/34858	100.00

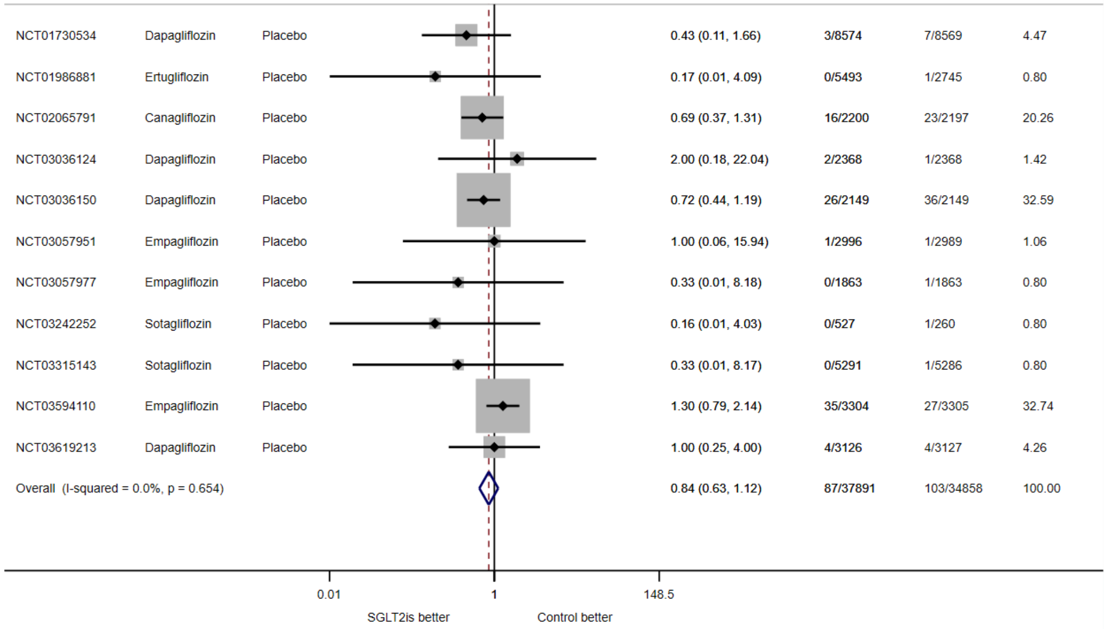

System 20-Renal And Urinary Disorders-Figure S11 Meta-analysis of SGLT2is and End stage renal disease

Trial	SGLT2is	Control	Risk Ratio (95% CI)	Events, Treatment	Events, Control	% Weight
NCT01032629	Canagliflozin	Placebo	1.50 (0.30, 7.41)	6/2886	2/1441	8.91
NCT01131676	Empagliflozin	Placebo	0.37 (0.08, 1.67)	3/4687	4/2333	10.18
NCT01730534	Dapagliflozin	Placebo	0.25 (0.08, 0.75)	4/8574	16/8569	18.99
NCT01986881	Ertugliflozin	Placebo	1.50 (0.06, 36.80)	1/5493	0/2745	2.22
NCT01989754	Canagliflozin	Placebo	3.00 (0.31, 28.81)	3/2904	1/2903	4.45
NCT02065791	Canagliflozin	Placebo	0.33 (0.03, 3.20)	1/2200	3/2197	4.45
NCT03036124	Dapagliflozin	Placebo	2.00 (0.18, 22.04)	2/2368	1/2368	3.96
NCT03036150	Dapagliflozin	Placebo	1.50 (0.25, 8.97)	3/2149	2/2149	7.13
NCT03057951	Empagliflozin	Placebo	1.33 (0.30, 5.94)	4/2996	3/2989	10.18
NCT03057977	Empagliflozin	Placebo	0.50 (0.05, 5.51)	1/1863	2/1863	3.96
NCT03315143	Sotagliflozin	Placebo	8.99 (0.48, 166.96)	4/5291	0/5286	2.67
NCT03521934	Sotagliflozin	Placebo	1.01 (0.06, 16.11)	1/605	1/611	2.97
NCT03594110	Empagliflozin	Placebo	7.00 (0.36, 135.50)	3/3304	0/3305	2.60
NCT03619213	Dapagliflozin	Placebo	0.71 (0.23, 2.25)	5/3126	7/3127	17.33
Overall (I-squared = 9.4%, p = 0.350)			0.81 (0.50, 1.31)	41/48446	42/41886	100.00

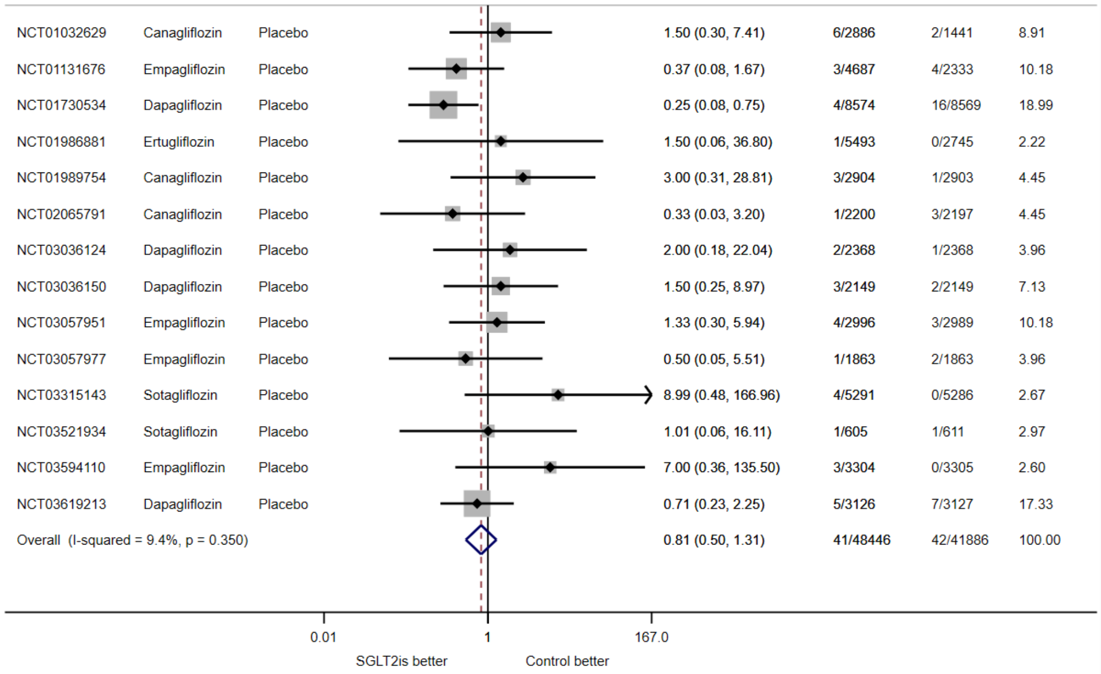

System 20-Renal And Urinary Disorders-Figure S12 Meta-analysis of SGLT2is and Haematuria

Trial	SGLT2is	Control	Risk Ratio (95% CI)	Events, Treatment	Events, Control	% Weight
NCT01032629	Canagliflozin	Placebo	2.00 (0.22, 17.85)	4/2886	1/1441	9.67
NCT01131676	Empagliflozin	Placebo	0.50 (0.07, 3.53)	2/4687	2/2333	12.08
NCT01730534	Dapagliflozin	Placebo	0.33 (0.07, 1.65)	2/8574	6/8569	18.11
NCT01986881	Ertugliflozin	Placebo	0.10 (0.00, 2.08)	0/5493	2/2745	5.03
NCT01989754	Canagliflozin	Placebo	0.33 (0.01, 8.18)	0/2904	1/2903	4.53
NCT02065791	Canagliflozin	Placebo	0.20 (0.01, 4.16)	0/2200	2/2197	5.03
NCT03036124	Dapagliflozin	Placebo	5.00 (0.24, 104.09)	2/2368	0/2368	5.03
NCT03036150	Dapagliflozin	Placebo	1.00 (0.06, 15.98)	1/2149	1/2149	6.04
NCT03057951	Empagliflozin	Placebo	8.98 (0.48, 166.70)	4/2996	0/2989	5.43
NCT03057977	Empagliflozin	Placebo	0.20 (0.01, 4.16)	0/1863	2/1863	5.03
NCT03315143	Sotagliflozin	Placebo	6.99 (0.36, 135.36)	3/5291	0/5286	5.28
NCT03594110	Empagliflozin	Placebo	4.00 (0.45, 35.78)	4/3304	1/3305	9.66
NCT03619213	Dapagliflozin	Placebo	0.33 (0.01, 8.18)	0/3126	1/3127	4.53
NCT04157751	Empagliflozin	Placebo	0.34 (0.01, 8.27)	0/260	1/264	4.54
Overall (I-squared = 10.3%, p = 0.340)			0.81 (0.41, 1.61)	22/48101	20/41539	100.00

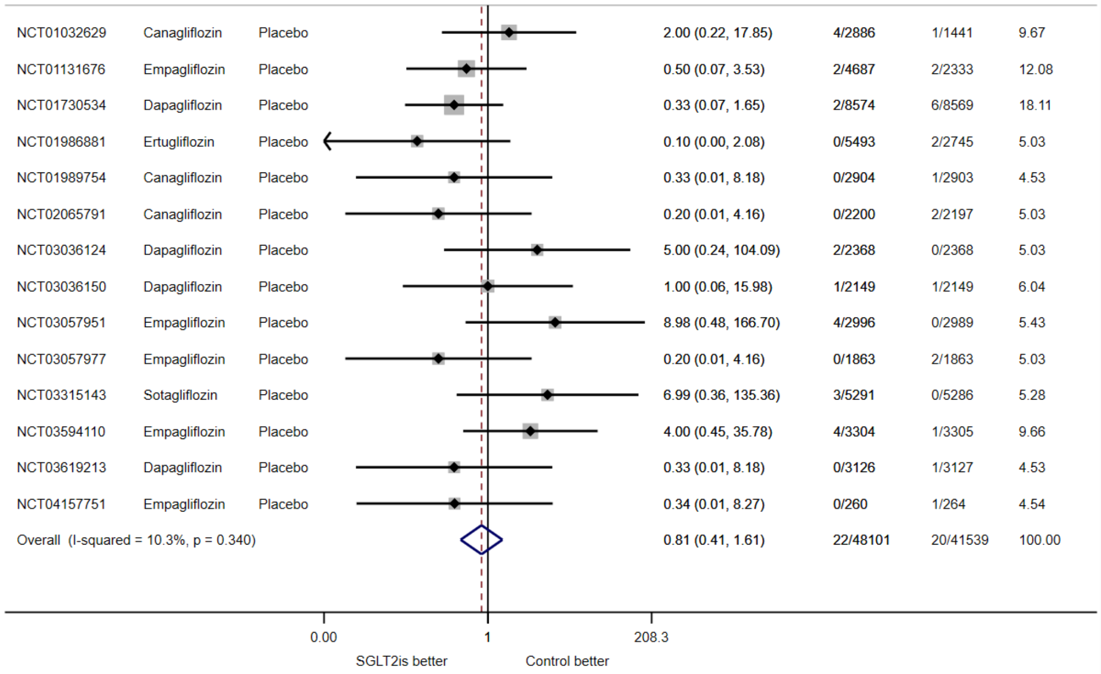

System 20-Renal And Urinary Disorders-Figure S13 Meta-analysis of SGLT2is and Hydronephrosis

Trial	SGLT2is	Control	Risk Ratio (95% CI)	Events, Treatment	Events, Control	% Weight
NCT01032629	Canagliflozin	Placebo	0.29 (0.08, 0.97)	4/2886	7/1441	10.28
NCT01042977	Dapagliflozin	Placebo	0.33 (0.01, 8.18)	0/482	1/483	1.51
NCT01106651	Canagliflozin	Placebo	4.48 (0.24, 82.89)	4/477	0/237	1.82
NCT01131676	Empagliflozin	Placebo	1.66 (0.46, 6.02)	10/4687	3/2333	9.31
NCT01730534	Dapagliflozin	Placebo	0.78 (0.39, 1.56)	14/8574	18/8569	31.77
NCT01986881	Ertugliflozin	Placebo	0.50 (0.16, 1.55)	6/5493	6/2745	12.10
NCT01989754	Canagliflozin	Placebo	4.00 (0.85, 18.81)	8/2904	2/2903	6.45
NCT02065791	Canagliflozin	Placebo	3.00 (0.12, 73.50)	1/2200	0/2197	1.51
NCT03036124	Dapagliflozin	Placebo	1.00 (0.06, 15.98)	1/2368	1/2368	2.01
NCT03036150	Dapagliflozin	Placebo	0.25 (0.03, 2.23)	1/2149	4/2149	3.22
NCT03057951	Empagliflozin	Placebo	1.00 (0.14, 7.08)	2/2996	2/2989	4.03
NCT03057977	Empagliflozin	Placebo	3.00 (0.12, 73.59)	1/1863	0/1863	1.51
NCT03242252	Sotagliflozin	Placebo	0.16 (0.01, 4.03)	0/527	1/260	1.51
NCT03315143	Sotagliflozin	Placebo	0.25 (0.05, 1.18)	2/5291	8/5286	6.45
NCT03594110	Empagliflozin	Placebo	5.00 (0.24, 104.14)	2/3304	0/3305	1.68
NCT03619213	Dapagliflozin	Placebo	0.67 (0.11, 3.99)	2/3126	3/3127	4.84
Overall (I-squared = 13.6%, p = 0.298)			0.77 (0.52, 1.14)	58/49327	56/42255	100.00

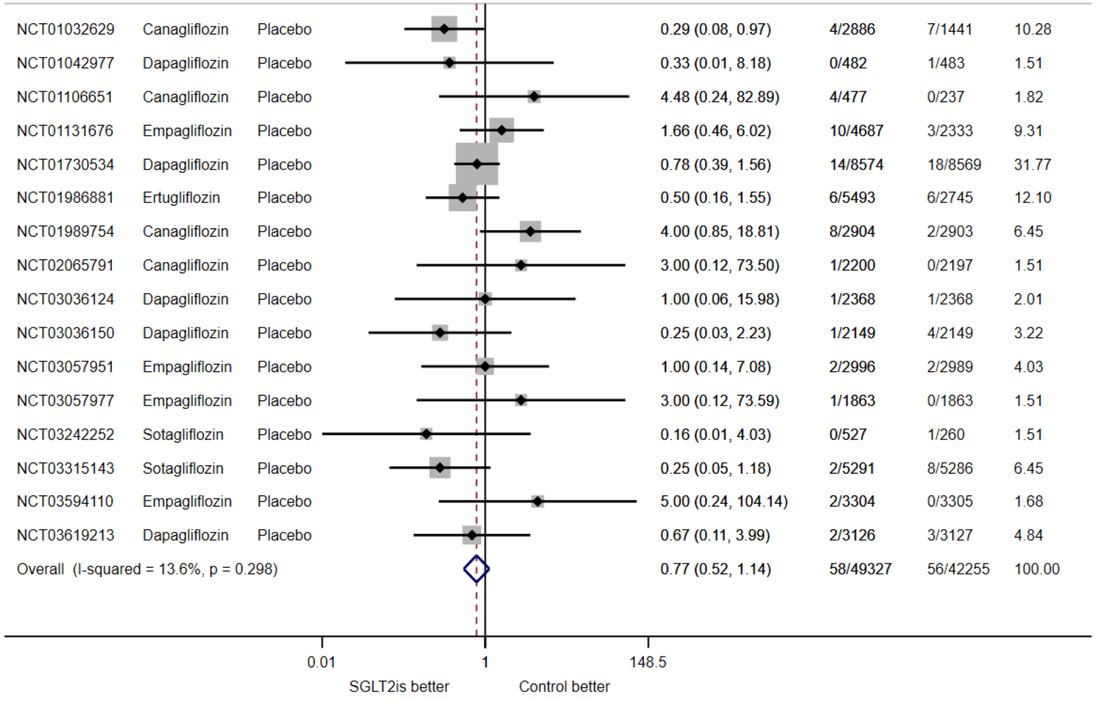

System 20-Renal And Urinary Disorders-Figure S14 Meta-analysis of SGLT2is and Nephrolithiasis

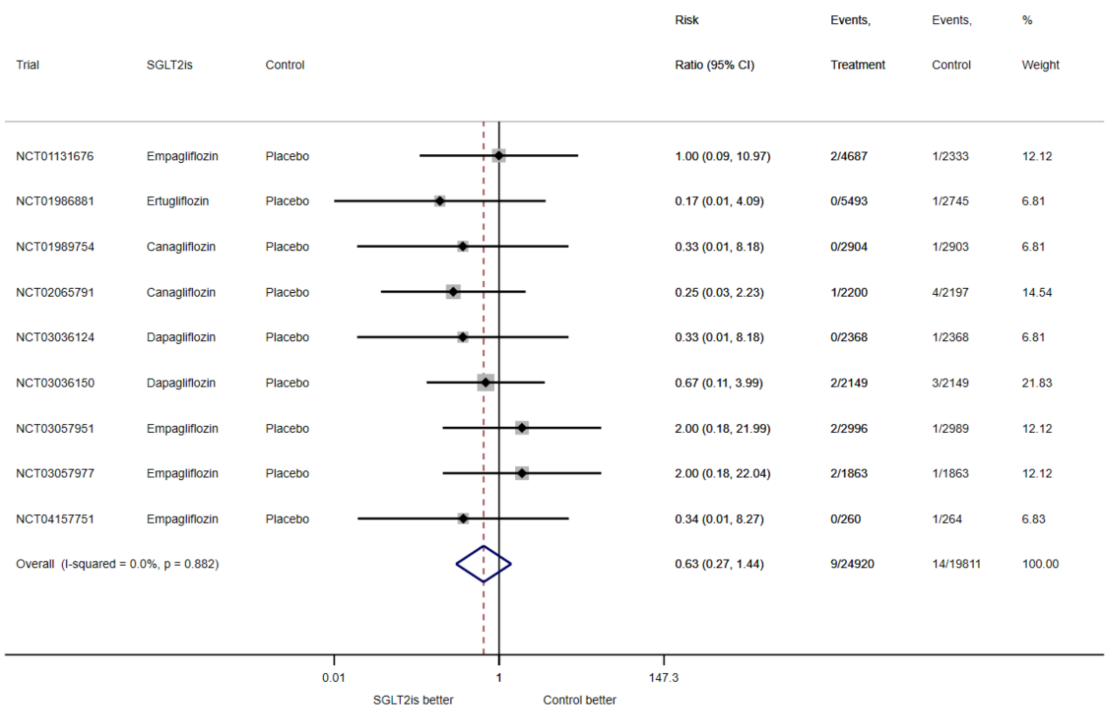

System 20-Renal And Urinary Disorders-Figure S15 Meta-analysis of SGLT2is and Nephropathy

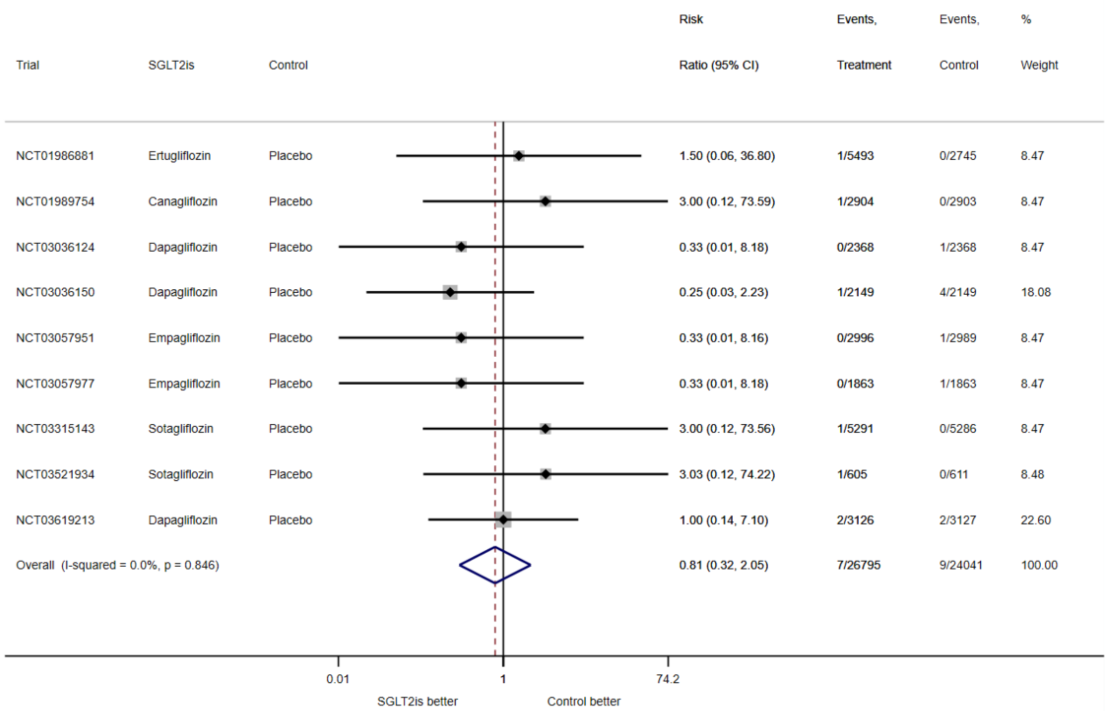

System 20-Renal And Urinary Disorders-Figure S16 Meta-analysis of SGLT2is and Nephropathy toxic

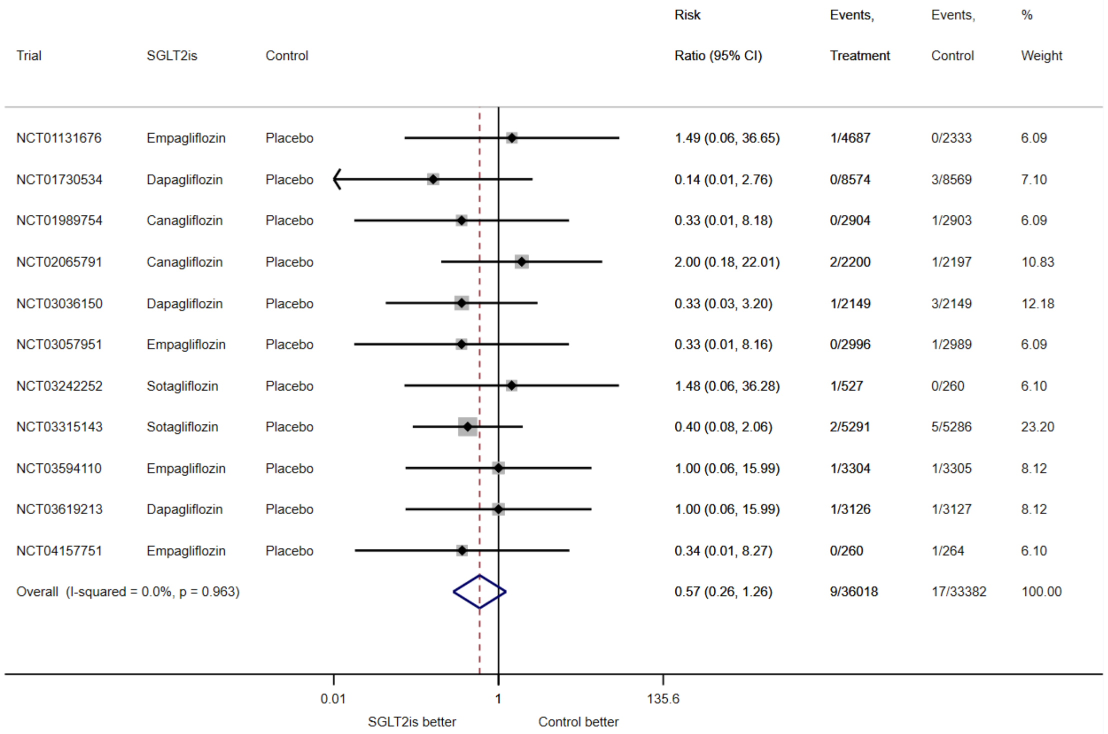

System 20-Renal And Urinary Disorders-Figure S17 Meta-analysis of SGLT2is and Nephrotic syndrome

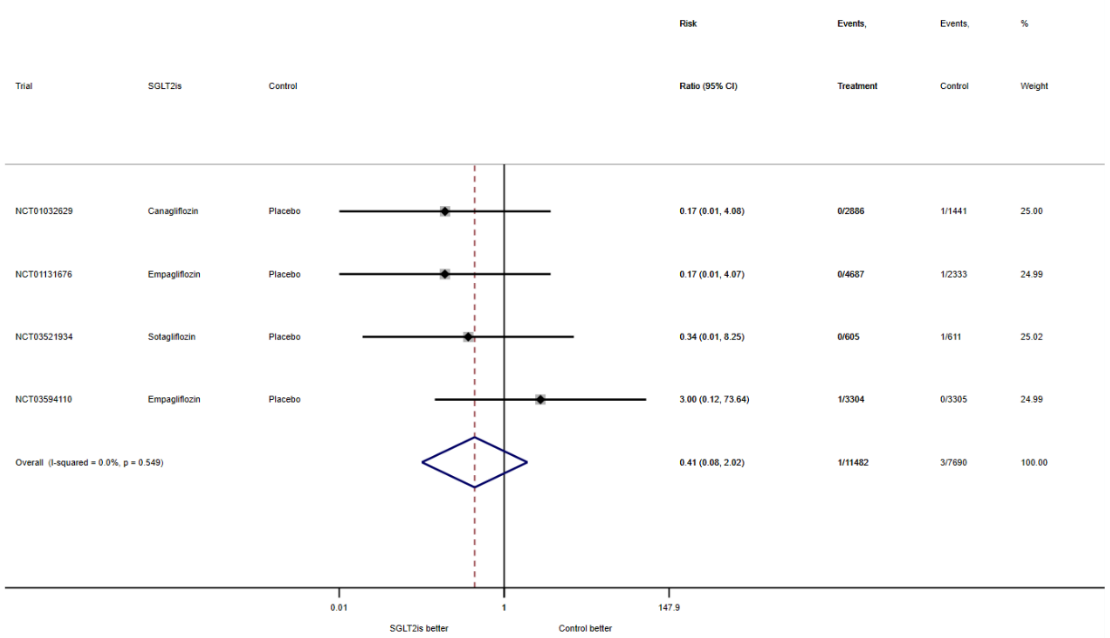

System 20-Renal And Urinary Disorders-Figure S18 Meta-analysis of SGLT2is and Oliguria

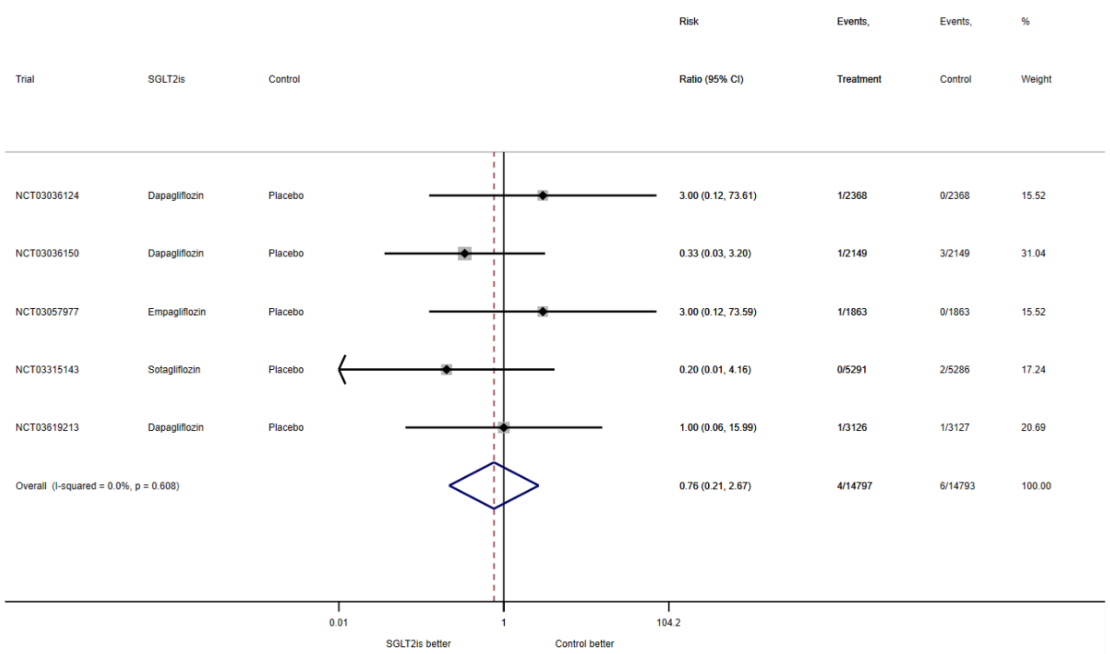

System 20-Renal And Urinary Disorders-Figure S19 Meta-analysis of SGLT2is and Prerenal failure

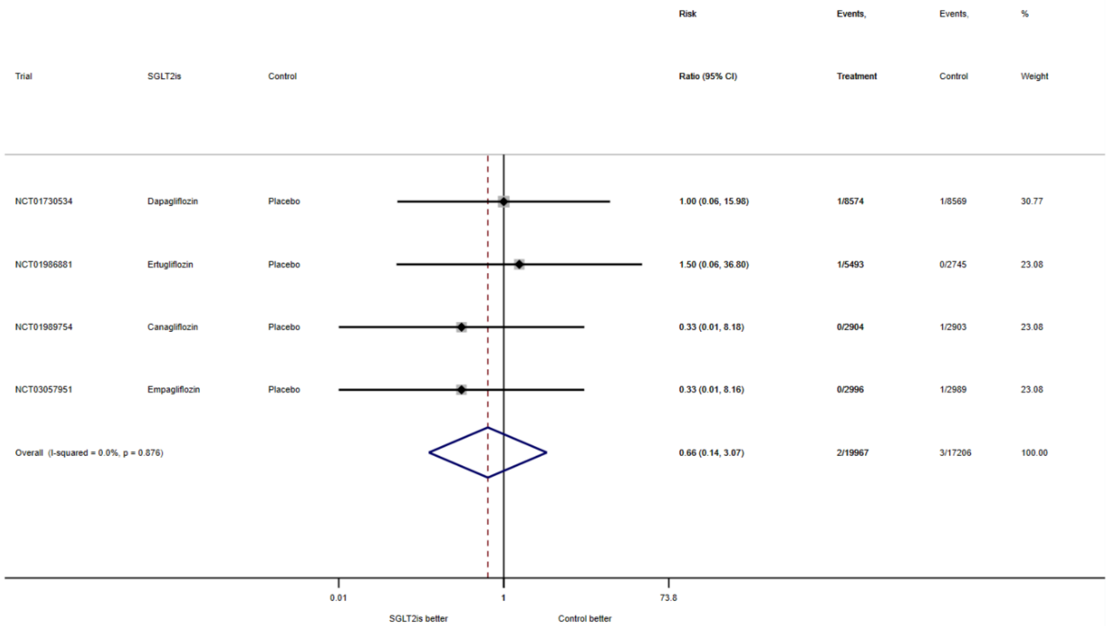

System 20-Renal And Urinary Disorders-Figure S20 Meta-analysis of SGLT2is and Proteinuria

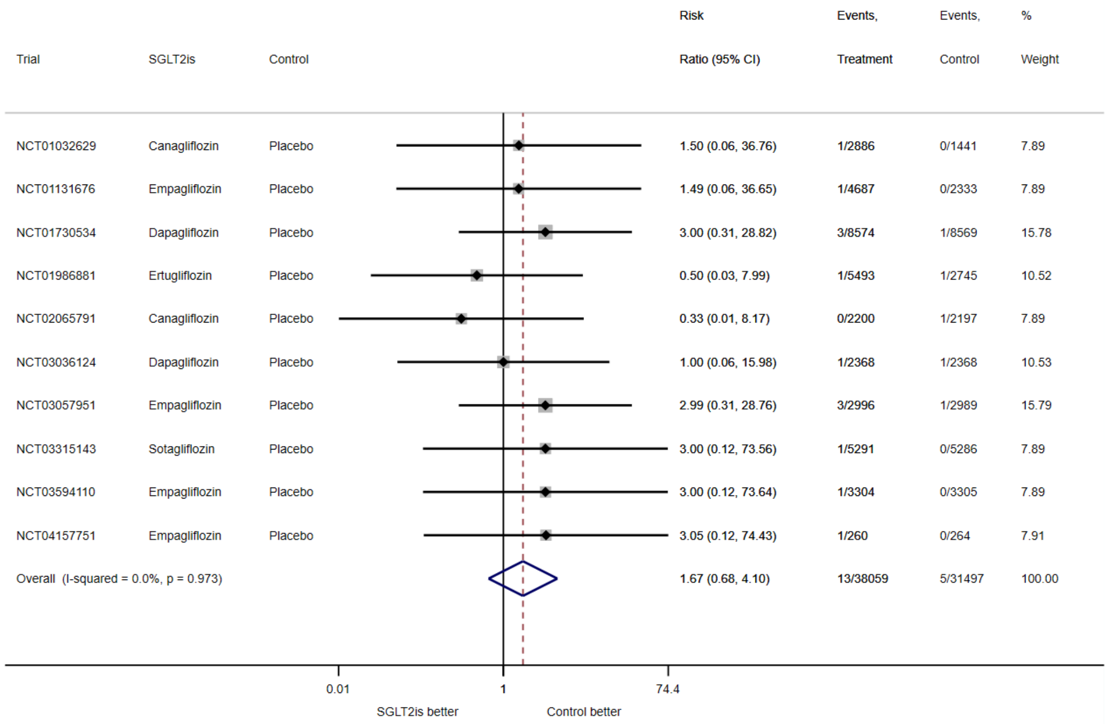

System 20-Renal And Urinary Disorders-Figure S21 Meta-analysis of SGLT2is and Renal artery stenosis

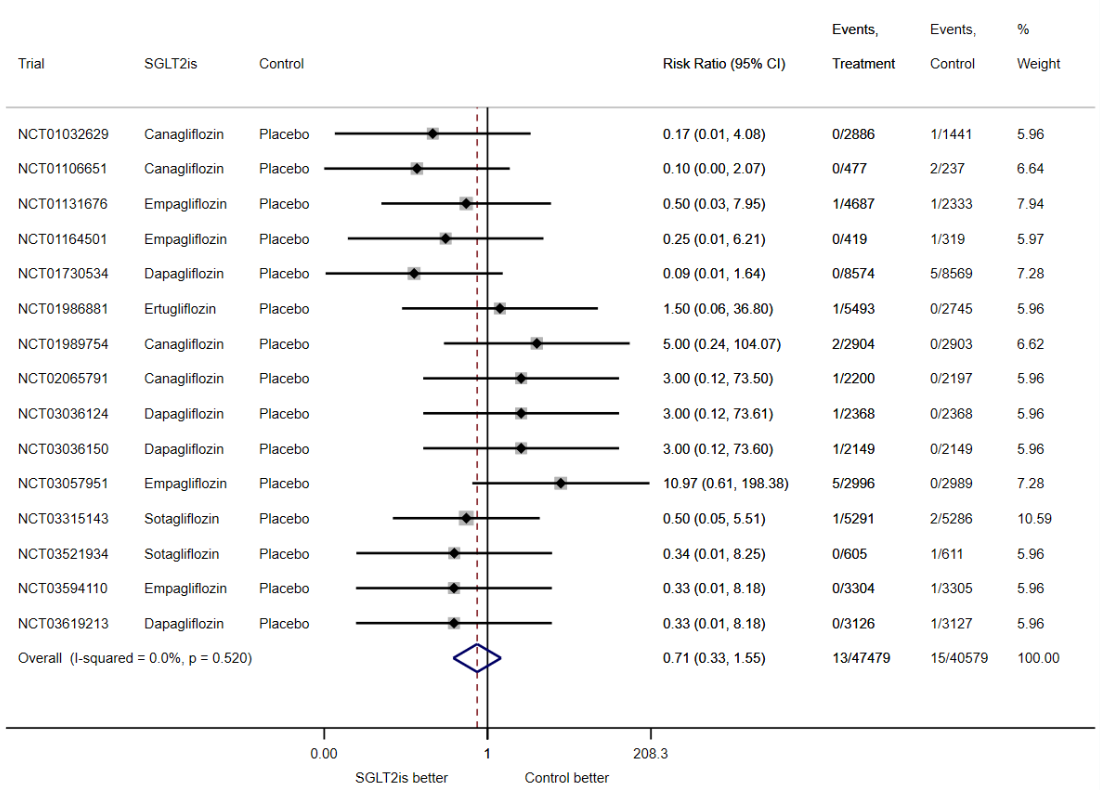

System 20-Renal And Urinary Disorders-Figure S22 Meta-analysis of SGLT2is and Renal colic

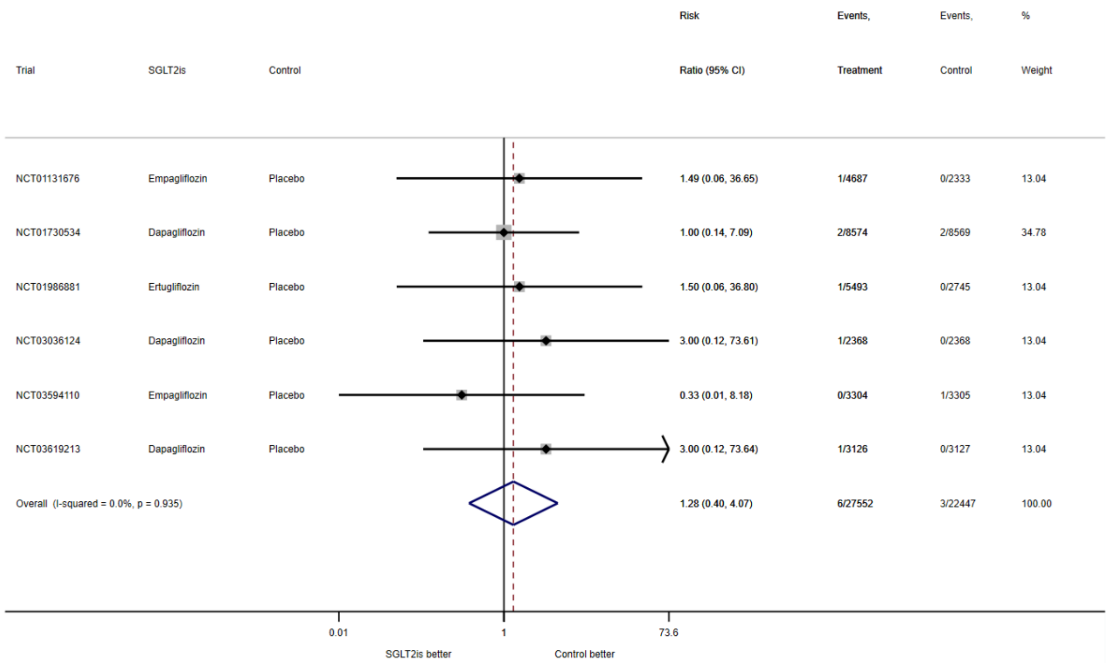

System 20-Renal And Urinary Disorders-Figure S23 Meta-analysis of SGLT2is and Renal cyst

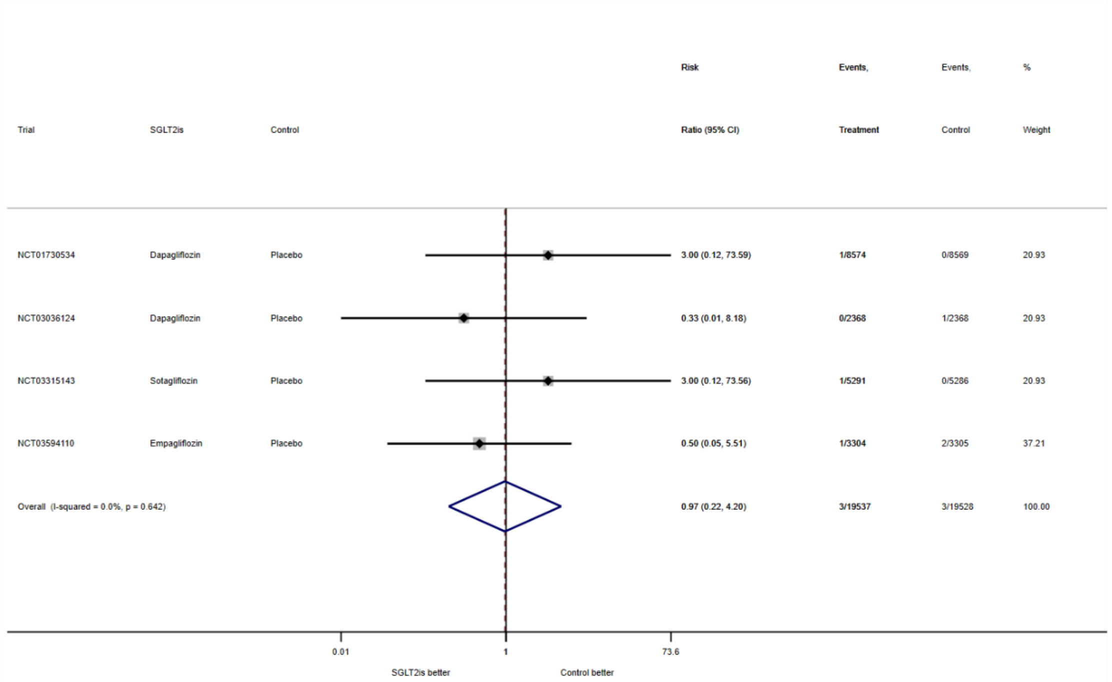

System 20-Renal And Urinary Disorders-Figure S24 Meta-analysis of SGLT2is and Renal disorder

Trial	SGLT2is	Control	Risk Ratio (95% CI)	Events, Treatment	Events, Control	% Weight
NCT01032629	Canagliflozin	Placebo	0.50 (0.07, 3.54)	2/2886	2/1441	1.97
NCT01042977	Dapagliflozin	Placebo	3.01 (0.12, 73.61)	1/482	0/483	0.74
NCT01131676	Empagliflozin	Placebo	0.30 (0.11, 0.82)	6/4687	10/2333	7.41
NCT01164501	Empagliflozin	Placebo	0.76 (0.05, 12.13)	1/419	1/319	0.99
NCT01730534	Dapagliflozin	Placebo	0.59 (0.27, 1.28)	10/8574	17/8569	12.43
NCT01986881	Ertugliflozin	Placebo	1.25 (0.24, 6.44)	5/5493	2/2745	2.82
NCT01989754	Canagliflozin	Placebo	1.00 (0.14, 7.09)	2/2904	2/2903	1.97
NCT02065791	Canagliflozin	Placebo	0.55 (0.19, 1.65)	5/2200	9/2197	6.36
NCT03036124	Dapagliflozin	Placebo	1.00 (0.38, 2.66)	8/2368	8/2368	7.91
NCT03036150	Dapagliflozin	Placebo	0.60 (0.22, 1.65)	6/2149	10/2149	7.42
NCT03057951	Empagliflozin	Placebo	0.43 (0.20, 0.93)	9/2996	21/2989	12.47
NCT03057977	Empagliflozin	Placebo	0.64 (0.28, 1.48)	9/1863	14/1863	10.86
NCT03315143	Sotagliflozin	Placebo	0.69 (0.32, 1.48)	11/5291	16/5286	12.88
NCT03521934	Sotagliflozin	Placebo	0.50 (0.13, 2.01)	3/605	6/611	3.97
NCT03619213	Dapagliflozin	Placebo	0.82 (0.34, 1.97)	9/3126	11/3127	9.79
Overall (I-squared = 0.0%, p = 0.957)			0.62 (0.47, 0.81)	87/46043	129/39383	100.00

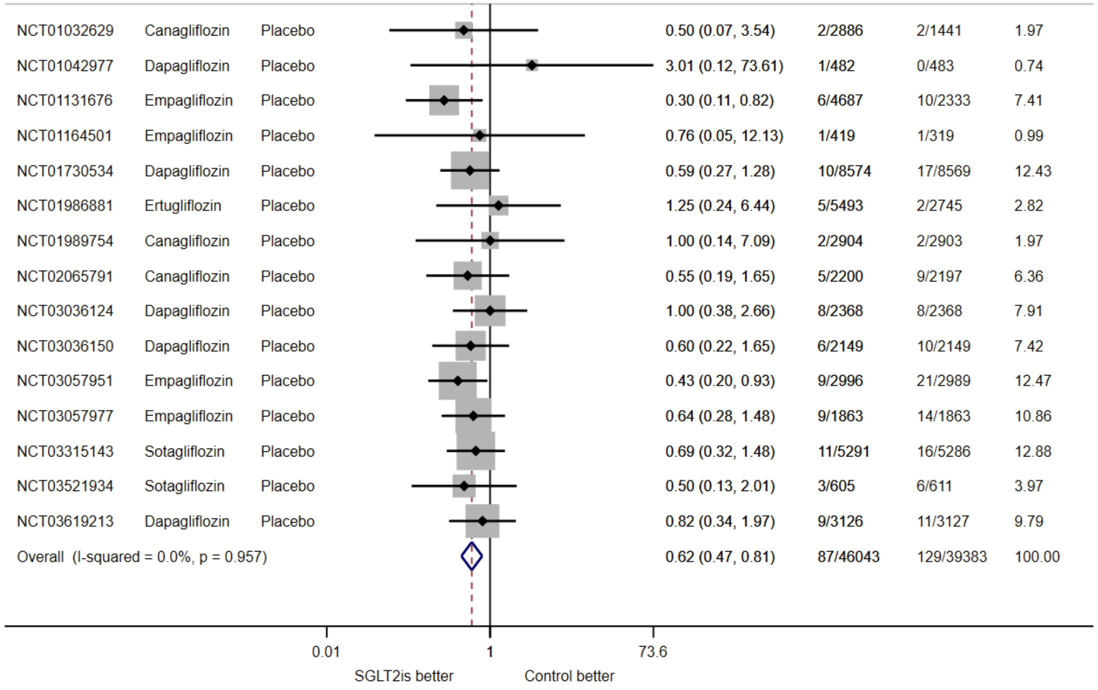

System 20-Renal And Urinary Disorders-Figure S25 Meta-analysis of SGLT2is and Renal failure

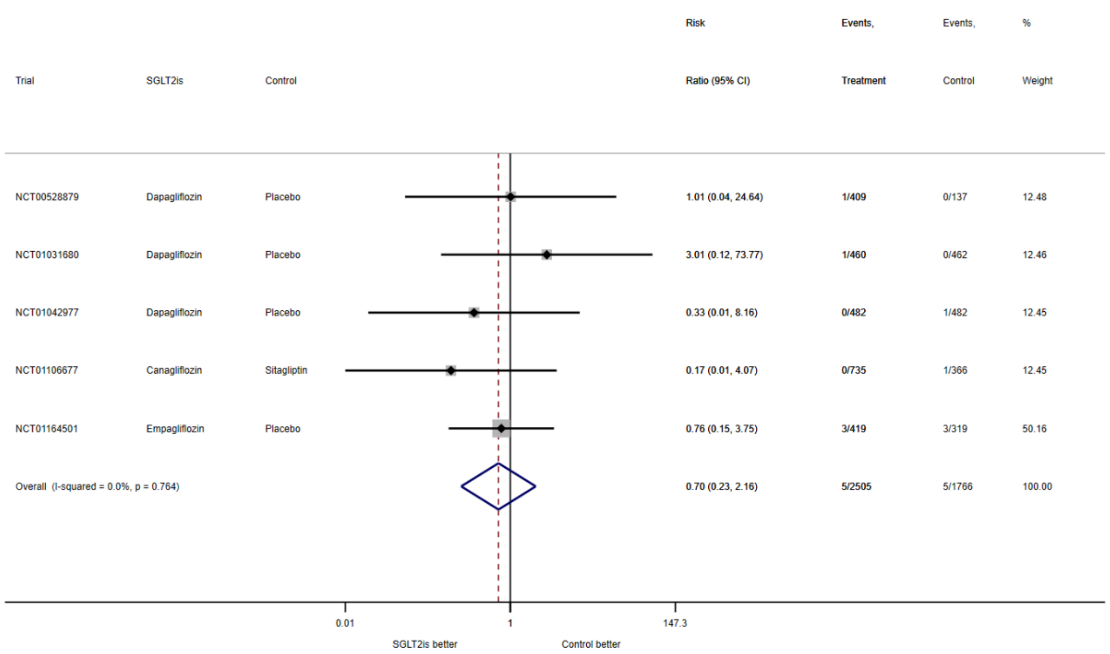

System 20-Renal And Urinary Disorders-Figure S26 Meta-analysis of SGLT2is and Renal failure acute

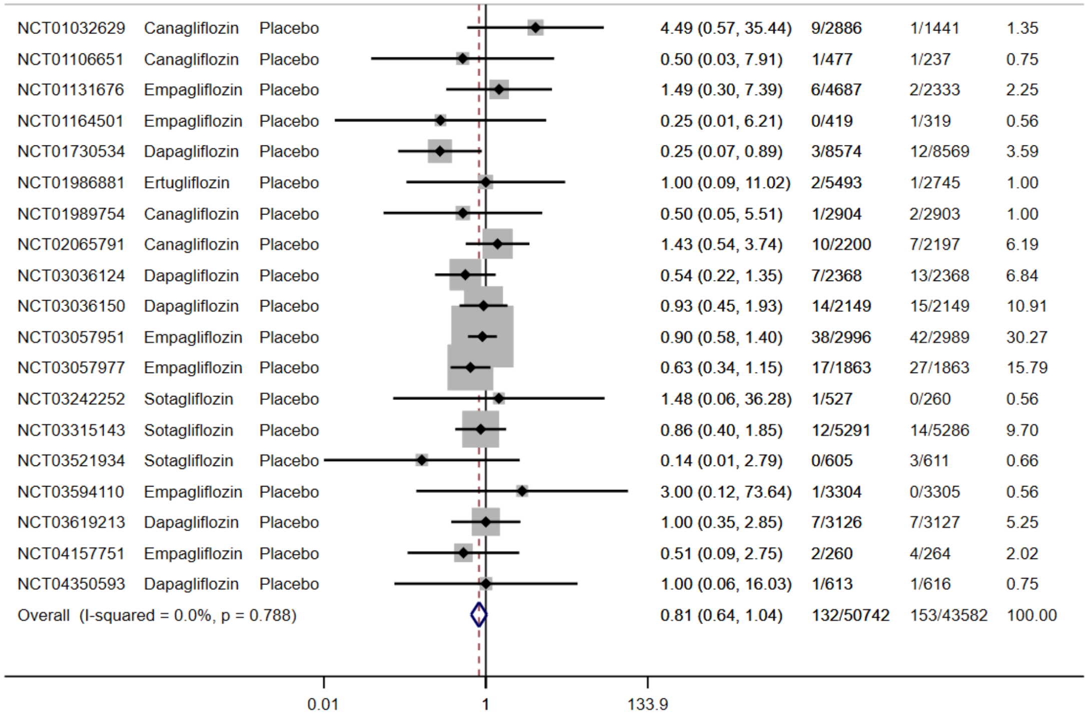

Trial	SGLT2is	Control	Risk Ratio (95% CI)	Events, Treatment	Events, Control	% Weight
NCT01032629	Canagliflozin	Placebo	4.49 (0.57, 35.44)	9/2886	1/1441	1.35
NCT01106651	Canagliflozin	Placebo	0.50 (0.03, 7.91)	1/477	1/237	0.75
NCT01131676	Empagliflozin	Placebo	1.49 (0.30, 7.39)	6/4687	2/2333	2.25
NCT01164501	Empagliflozin	Placebo	0.25 (0.01, 6.21)	0/419	1/319	0.56
NCT01730534	Dapagliflozin	Placebo	0.25 (0.07, 0.89)	3/8574	12/8569	3.59
NCT01986881	Ertugliflozin	Placebo	1.00 (0.09, 11.02)	2/5493	1/2745	1.00
NCT01989754	Canagliflozin	Placebo	0.50 (0.05, 5.51)	1/2904	2/2903	1.00
NCT02065791	Canagliflozin	Placebo	1.43 (0.54, 3.74)	10/2200	7/2197	6.19
NCT03036124	Dapagliflozin	Placebo	0.54 (0.22, 1.35)	7/2368	13/2368	6.84
NCT03036150	Dapagliflozin	Placebo	0.93 (0.45, 1.93)	14/2149	15/2149	10.91
NCT03057951	Empagliflozin	Placebo	0.90 (0.58, 1.40)	38/2996	42/2989	30.27
NCT03057977	Empagliflozin	Placebo	0.63 (0.34, 1.15)	17/1863	27/1863	15.79
NCT03242252	Sotagliflozin	Placebo	1.48 (0.06, 36.28)	1/527	0/260	0.56
NCT03315143	Sotagliflozin	Placebo	0.86 (0.40, 1.85)	12/5291	14/5286	9.70
NCT03521934	Sotagliflozin	Placebo	0.14 (0.01, 2.79)	0/605	3/611	0.66
NCT03594110	Empagliflozin	Placebo	3.00 (0.12, 73.64)	1/3304	0/3305	0.56
NCT03619213	Dapagliflozin	Placebo	1.00 (0.35, 2.85)	7/3126	7/3127	5.25
NCT04157751	Empagliflozin	Placebo	0.51 (0.09, 2.75)	2/260	4/264	2.02
NCT04350593	Dapagliflozin	Placebo	1.00 (0.06, 16.03)	1/613	1/616	0.75
Overall (I-squared = 0.0%, p = 0.788)			0.81 (0.64, 1.04)	132/50742	153/43582	100.00

System 20-Renal And Urinary Disorders-Figure S27 Meta-analysis of SGLT2is and Renal impairment

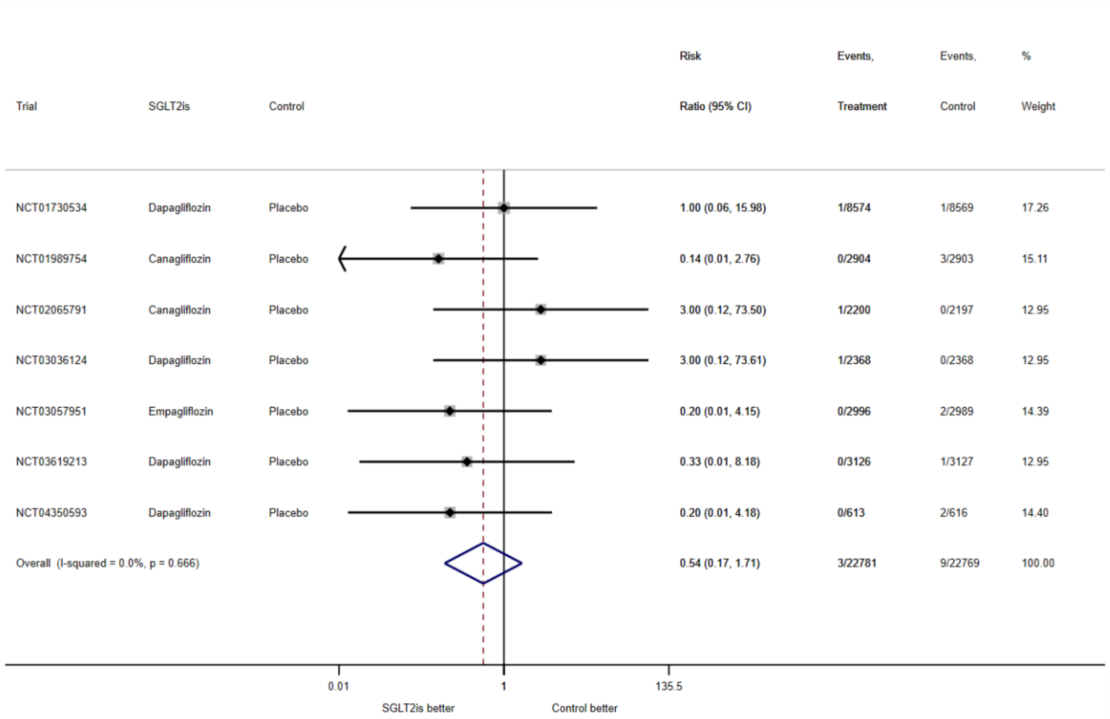

System 20-Renal And Urinary Disorders-Figure S28 Meta-analysis of SGLT2is and Renal injury

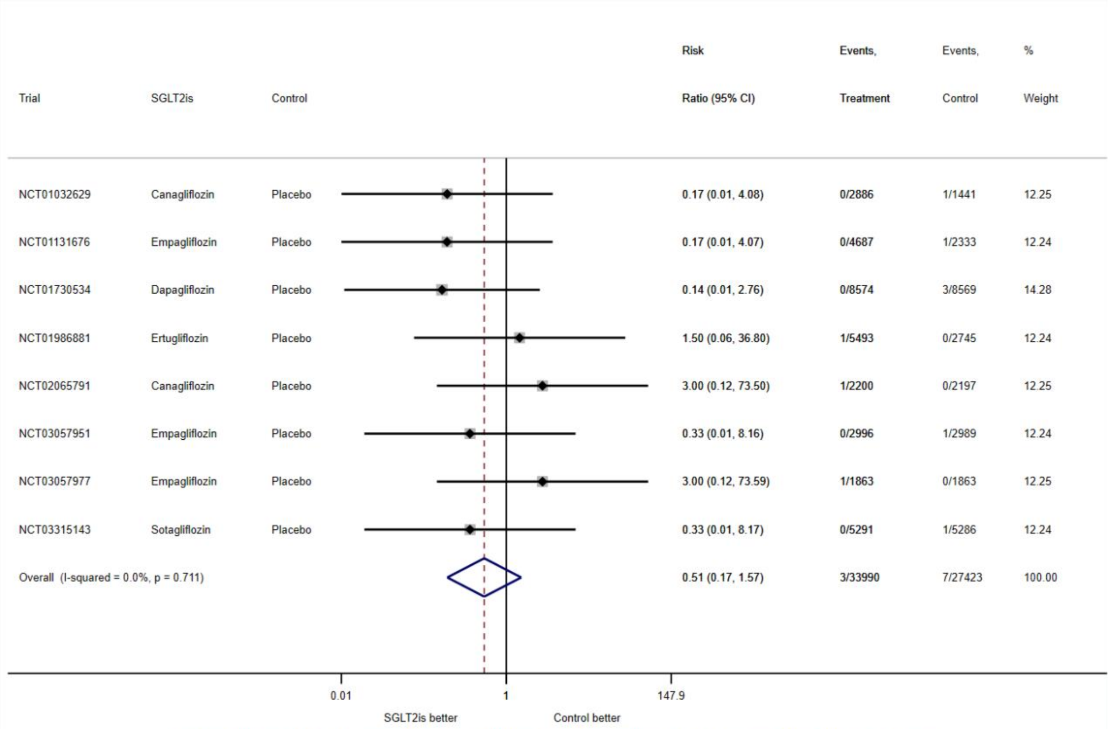

System 20-Renal And Urinary Disorders-Figure S29 Meta-analysis of SGLT2is and Renal tubular necrosis

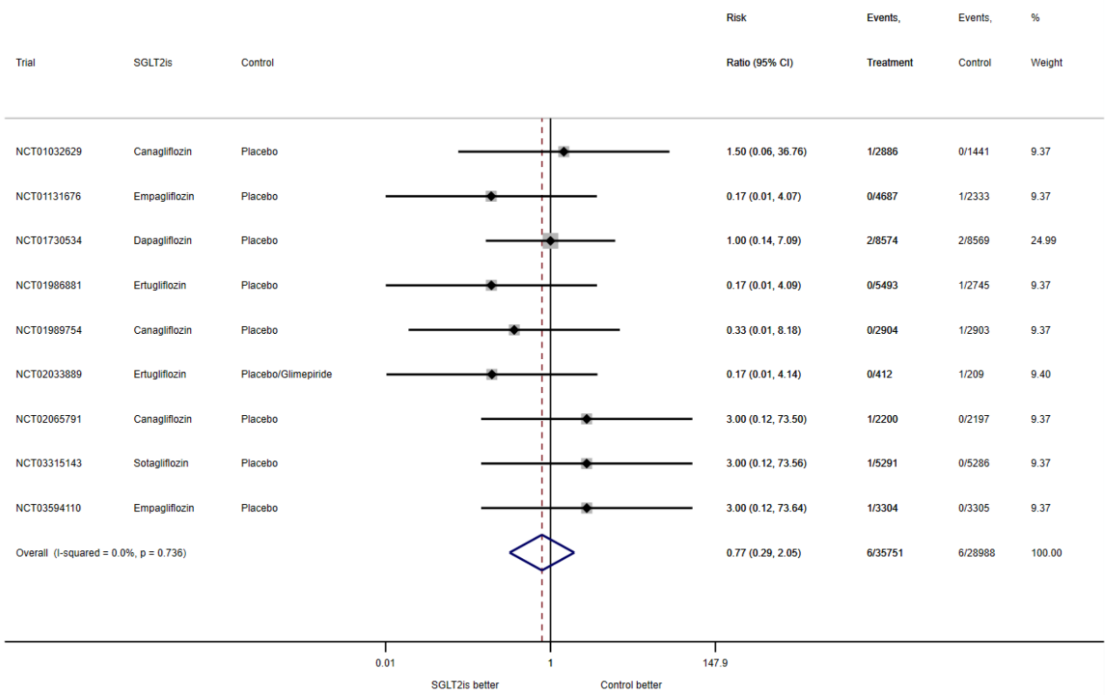

System 20-Renal And Urinary Disorders-Figure S30 Meta-analysis of SGLT2is and Stress urinary incontinence

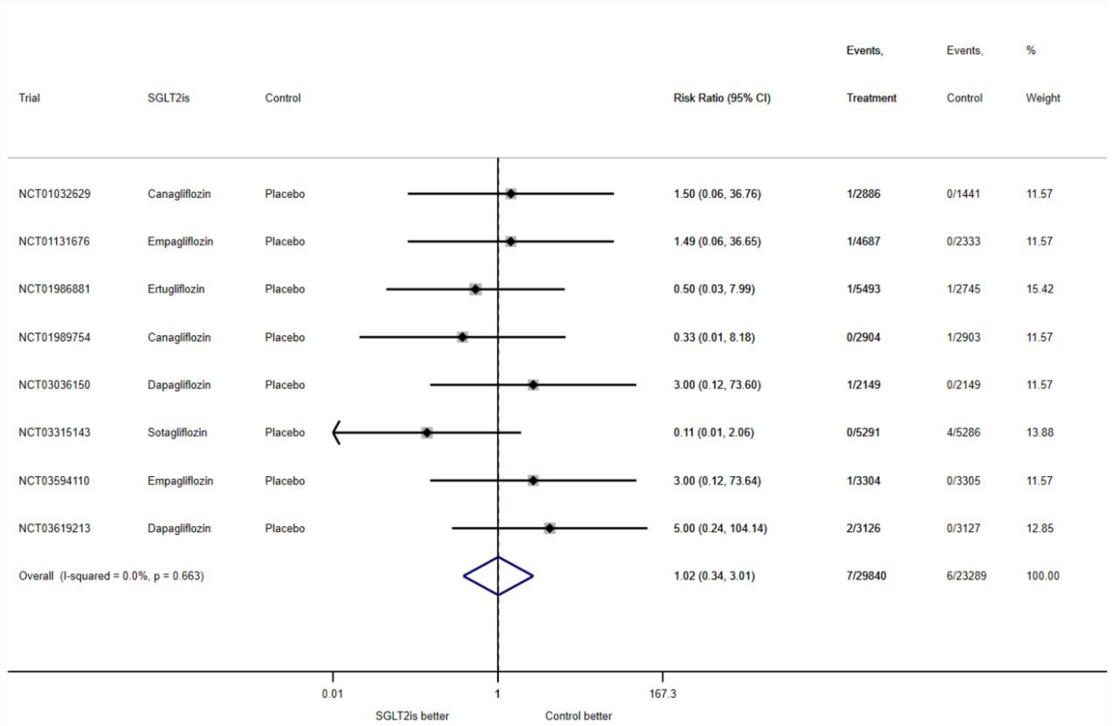

System 20-Renal And Urinary Disorders-Figure S31 Meta-analysis of SGLT2is and Tubulointerstitial nephritis

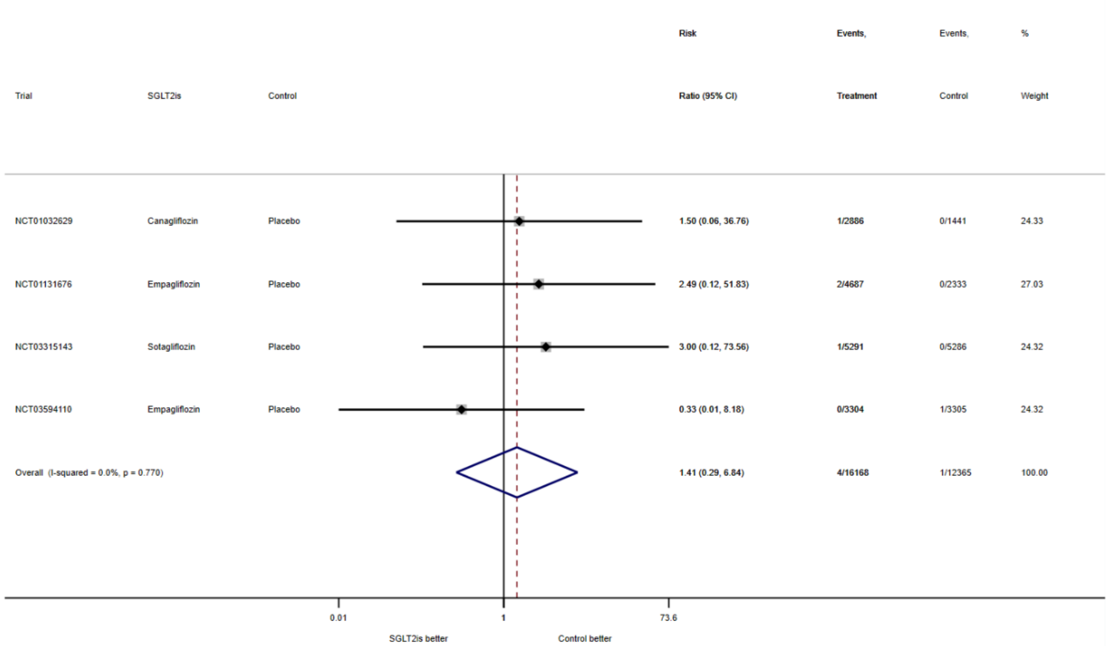

System 20-Renal And Urinary Disorders-Figure S32 Meta-analysis of SGLT2is and Ureteric obstruction

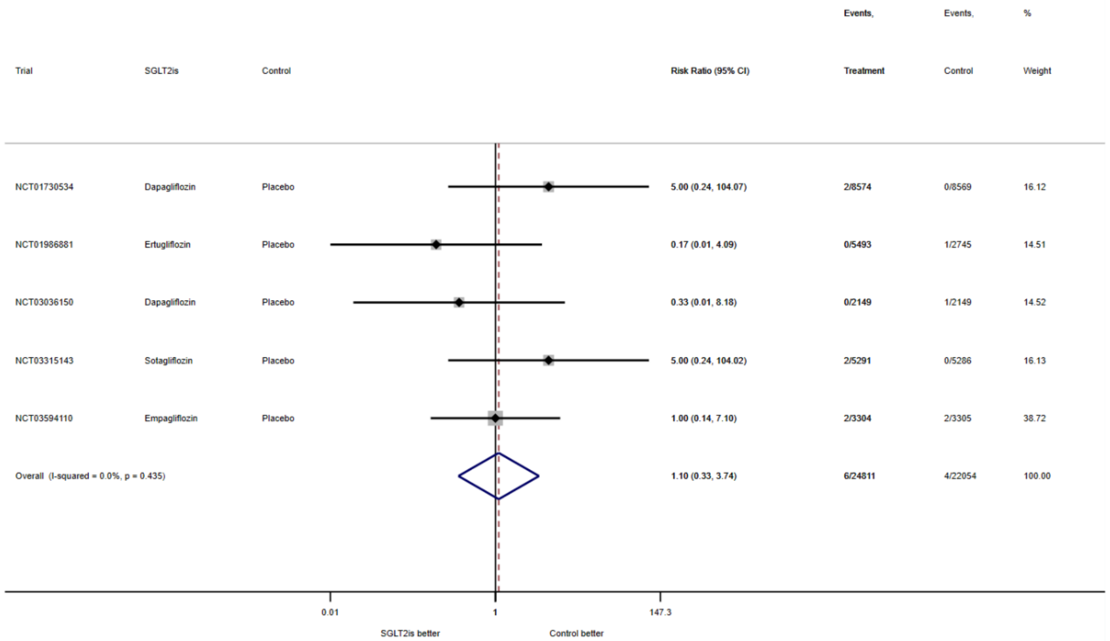

System 20-Renal And Urinary Disorders-Figure S33 Meta-analysis of SGLT2is and Ureteric stenosis

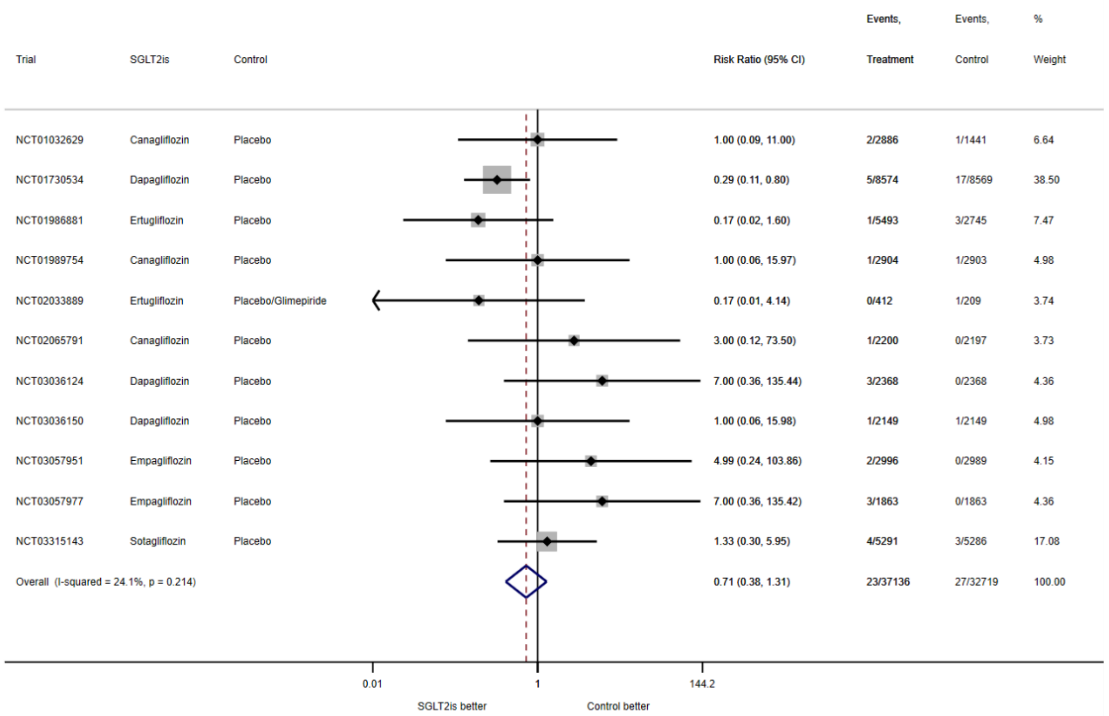

System 20-Renal And Urinary Disorders-Figure S34 Meta-analysis of SGLT2is and Ureterolithiasis

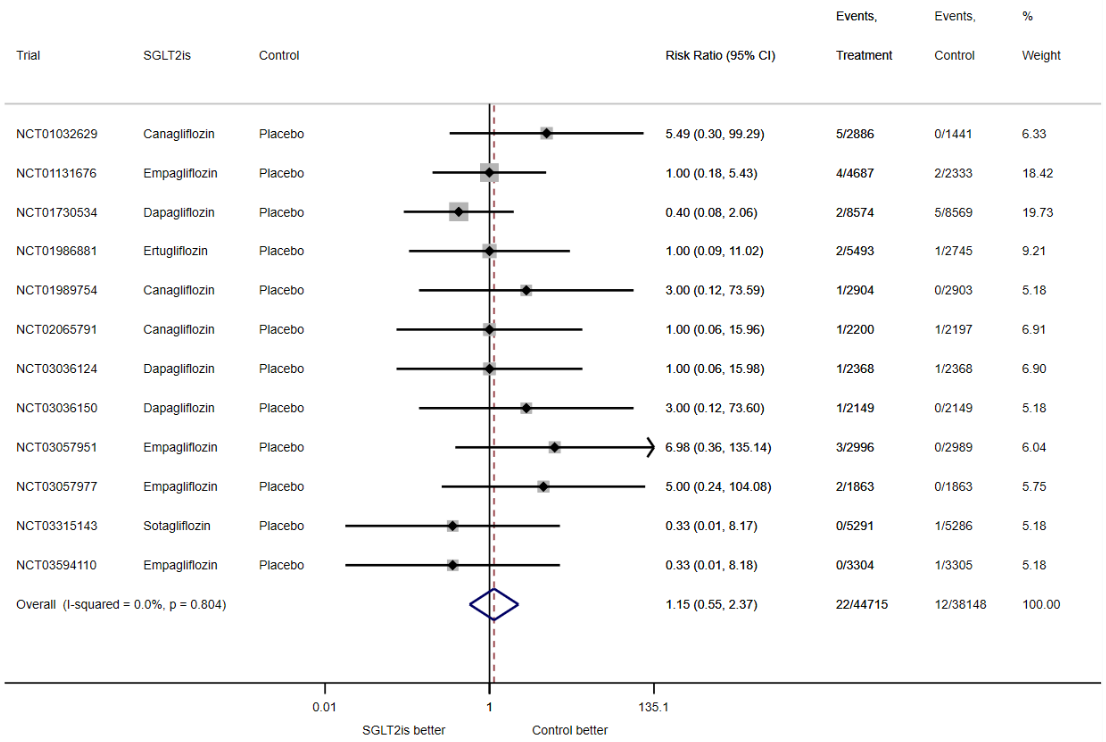

System 20-Renal And Urinary Disorders-Figure S35 Meta-analysis of SGLT2is and Urethral stenosis

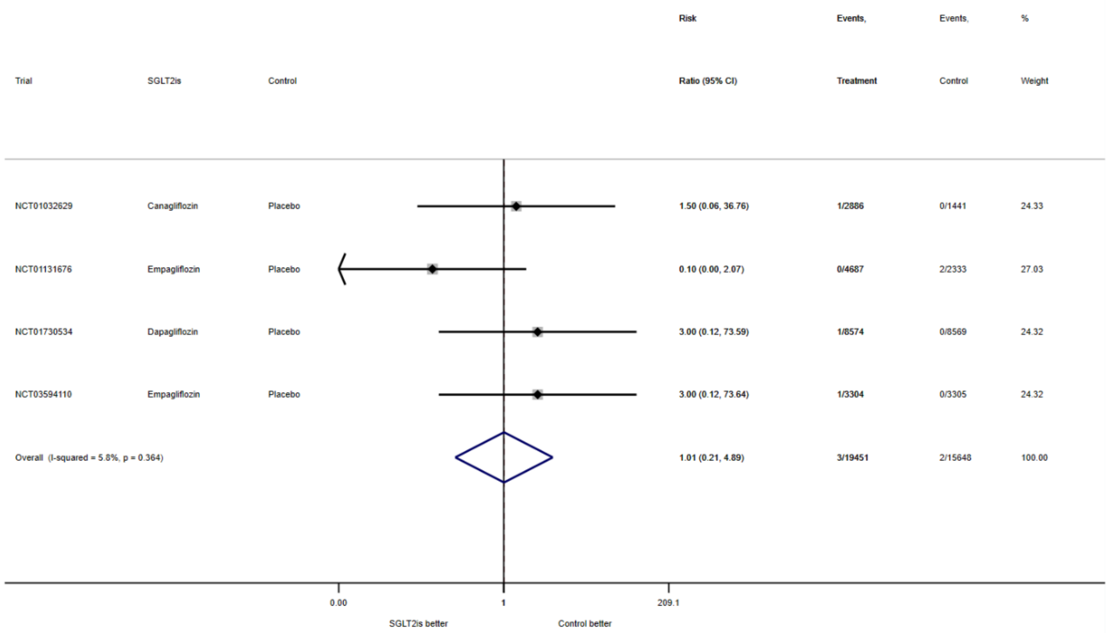

System 20-Renal And Urinary Disorders-Figure S36 Meta-analysis of SGLT2is and Urinary bladder polyp

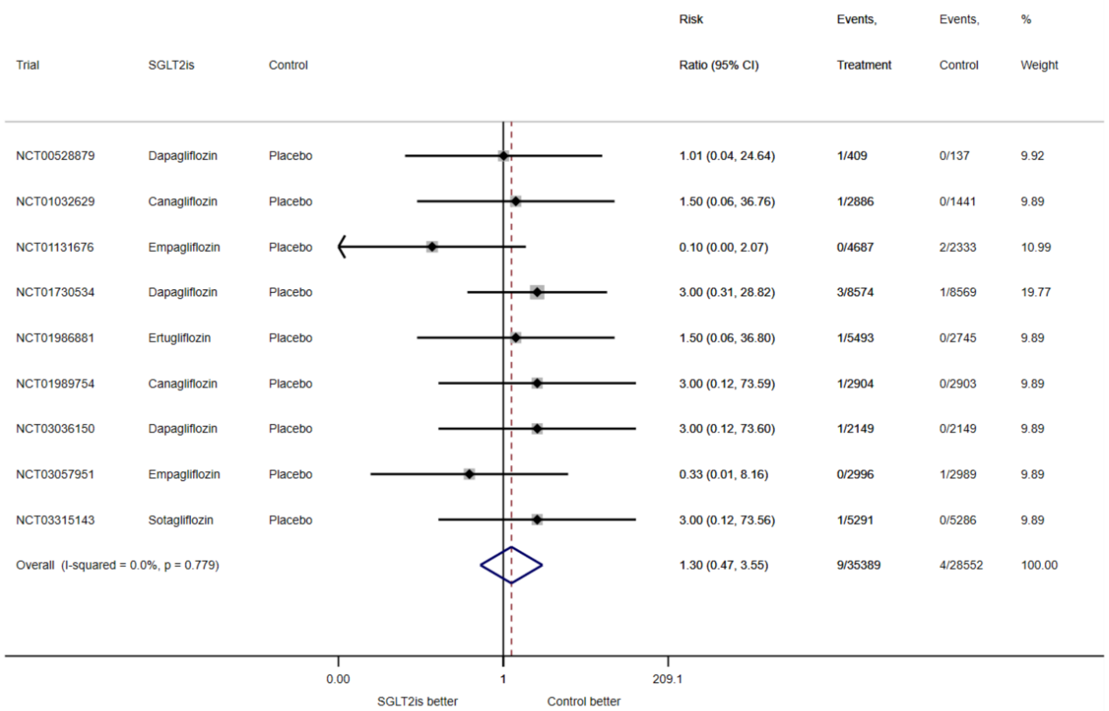

System 20-Renal And Urinary Disorders-Figure S37 Meta-analysis of SGLT2is and Urinary incontinence

Trial	SGLT2is	Control	Risk Ratio (95% CI)	Events, Treatment	Events, Control	% Weight
NCT01032629	Canagliflozin	Placebo	0.75 (0.13, 4.48)	3/2886	2/1441	7.14
NCT01042977	Dapagliflozin	Placebo	3.01 (0.12, 73.61)	1/482	0/483	2.23
NCT01131676	Empagliflozin	Placebo	1.49 (0.40, 5.51)	9/4687	3/2333	13.38
NCT01730534	Dapagliflozin	Placebo	0.80 (0.32, 2.02)	8/8574	10/8569	26.43
NCT01986881	Ertugliflozin	Placebo	1.50 (0.30, 7.42)	6/5493	2/2745	8.92
NCT01989754	Canagliflozin	Placebo	0.50 (0.05, 5.51)	1/2904	2/2903	3.96
NCT02065791	Canagliflozin	Placebo	2.00 (0.18, 22.01)	2/2200	1/2197	3.96
NCT03036124	Dapagliflozin	Placebo	0.20 (0.01, 4.16)	0/2368	2/2368	2.48
NCT03036150	Dapagliflozin	Placebo	0.25 (0.03, 2.23)	1/2149	4/2149	4.76
NCT03057951	Empagliflozin	Placebo	0.20 (0.02, 1.71)	1/2996	5/2989	4.95
NCT03057977	Empagliflozin	Placebo	1.00 (0.06, 15.98)	1/1863	1/1863	2.97
NCT03242252	Sotagliflozin	Placebo	1.48 (0.06, 36.28)	1/527	0/260	2.23
NCT03315143	Sotagliflozin	Placebo	1.00 (0.06, 15.97)	1/5291	1/5286	2.97
NCT03594110	Empagliflozin	Placebo	1.00 (0.20, 4.95)	3/3304	3/3305	8.92
NCT03619213	Dapagliflozin	Placebo	5.00 (0.24, 104.14)	2/3126	0/3127	2.48
NCT04157751	Empagliflozin	Placebo	3.05 (0.12, 74.43)	1/260	0/264	2.23
Overall (I-squared = 0.0%, p = 0.911)			0.93 (0.57, 1.49)	41/49110	36/42282	100.00

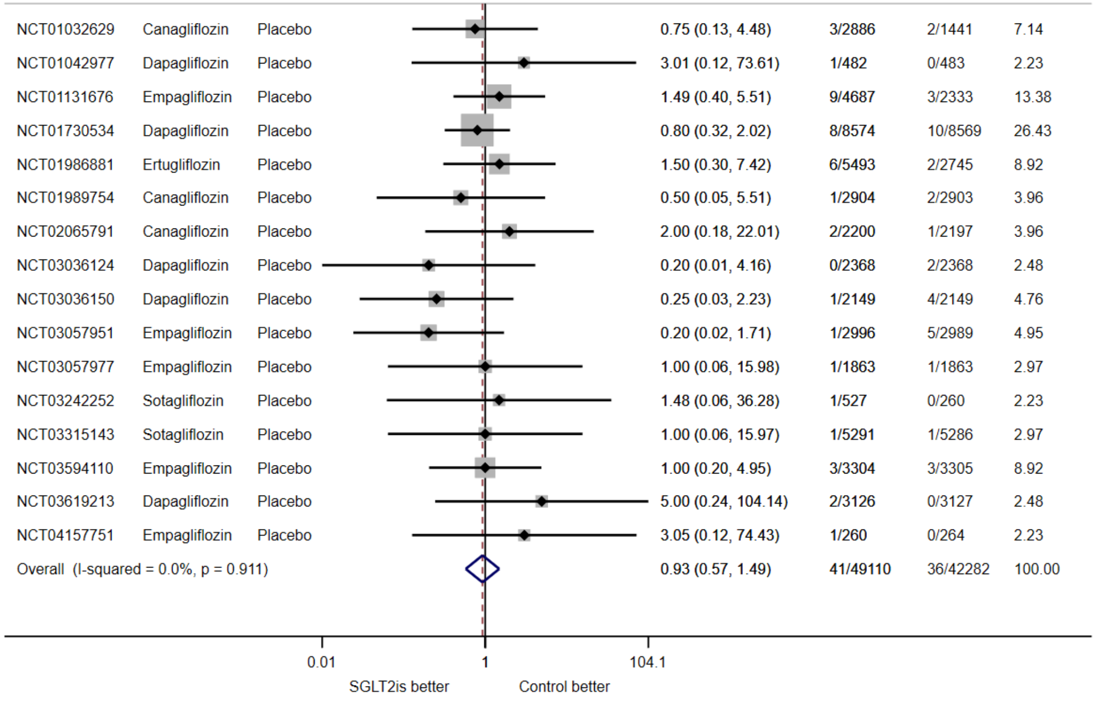

System 20-Renal And Urinary Disorders-Figure S38 Meta-analysis of SGLT2is and Urinary retention

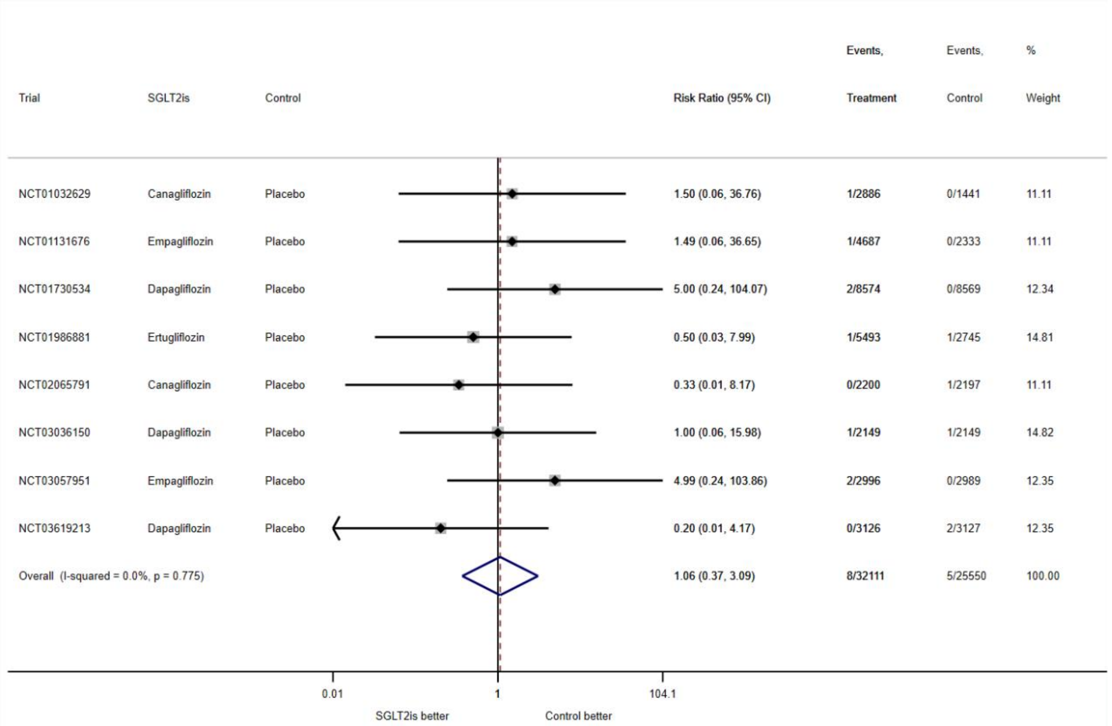

System 20-Renal And Urinary Disorders-Figure S39 Meta-analysis of SGLT2is and Urinary tract obstruction

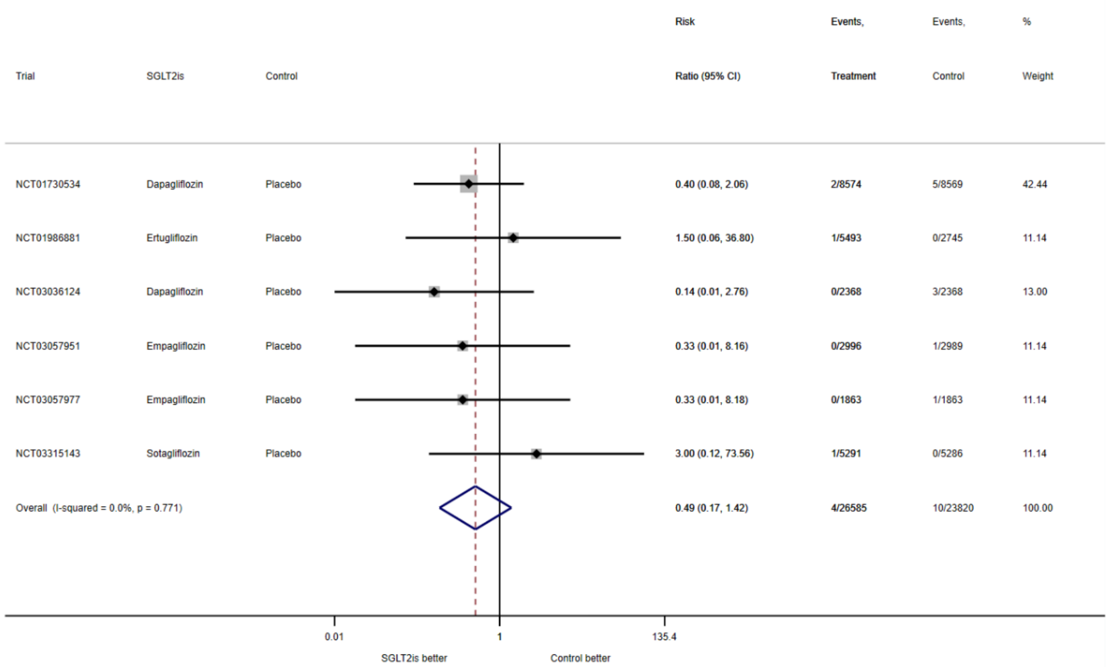

System 21-Reproductive System And Breast Disorders-Figure S1 Meta-analysis of SGLT2is and Acquired hydrocele

Trial	SGLT2is	Control	Risk Ratio (95% CI)	Events, Treatment	Events, Control	% Weight
NCT01032629	Canagliflozin	Placebo	1.50 (0.06, 36.76)	1/2886	0/1441	12.68
NCT01730534	Dapagliflozin	Placebo	10.99 (1.42, 85.13)	11/8574	1/8569	30.98
NCT01986881	Ertugliflozin	Placebo	2.50 (0.12, 52.04)	2/5493	0/2745	14.08
NCT02065791	Canagliflozin	Placebo	0.33 (0.01, 8.17)	0/2200	1/2197	12.68
NCT03057951	Empagliflozin	Placebo	2.99 (0.12, 73.44)	1/2996	0/2989	12.68
NCT03619213	Dapagliflozin	Placebo	1.00 (0.06, 15.99)	1/3126	1/3127	16.90
Overall (I-squared = 0.0%, p = 0.540)			2.52 (0.81, 7.86)	16/25275	3/21068	100.00

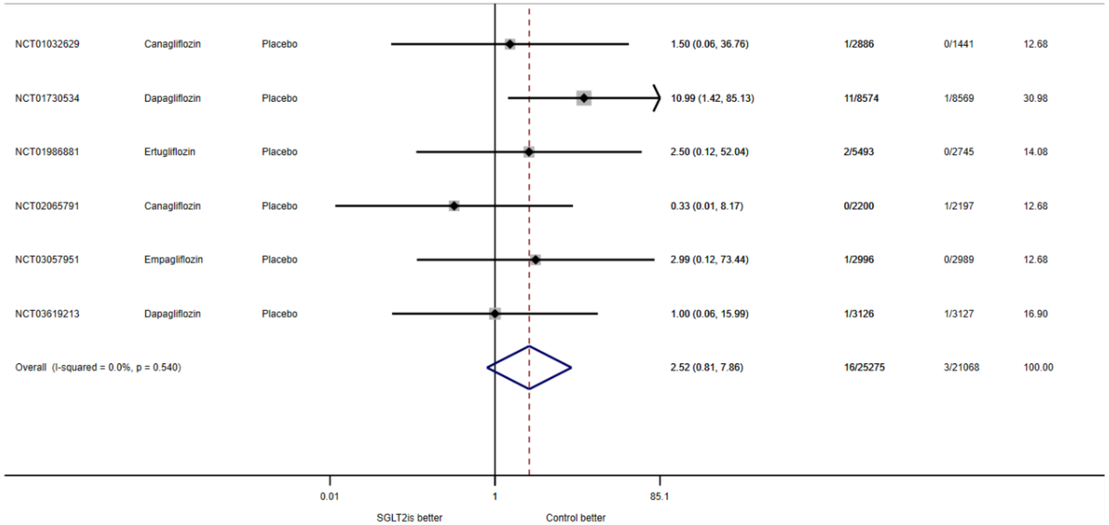

System 21-Reproductive System And Breast Disorders-Figure S2 Meta-analysis of SGLT2is and Acquired phimosis

Trial	SGLT2is	Control	Risk Ratio (95% CI)	Events, Treatment	Events, Control	% Weight
NCT01106651	Canagliflozin	Placebo	1.49 (0.06, 36.53)	1/477	0/237	12.70
NCT01730534	Dapagliflozin	Placebo	0.50 (0.05, 5.51)	1/8574	2/8569	22.53
NCT01989754	Canagliflozin	Placebo	3.00 (0.12, 73.59)	1/2904	0/2903	12.67
NCT03036124	Dapagliflozin	Placebo	0.33 (0.01, 8.18)	0/2368	1/2368	12.67
NCT03036150	Dapagliflozin	Placebo	3.00 (0.12, 73.60)	1/2149	0/2149	12.67
NCT03057977	Empagliflozin	Placebo	3.00 (0.12, 73.59)	1/1863	0/1863	12.67
NCT03315143	Sotagliflozin	Placebo	5.00 (0.24, 104.02)	2/5291	0/5286	14.08
Overall (I-squared = 0.0%, p = 0.834)			1.49 (0.48, 4.66)	7/23626	3/23375	100.00

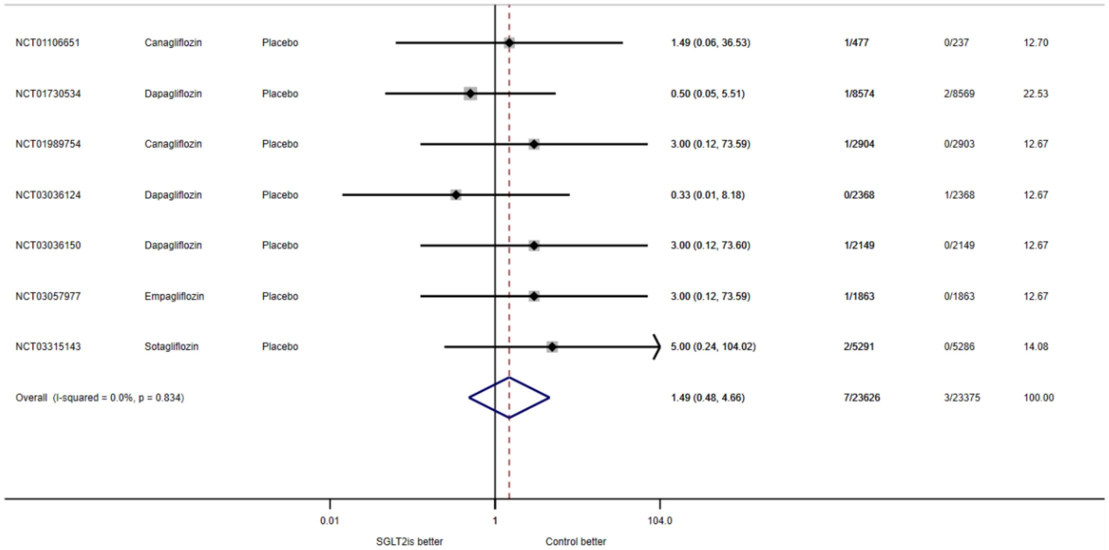

System 21-Reproductive System And Breast Disorders-Figure S3 Meta-analysis of SGLT2is and Balanoposthitis

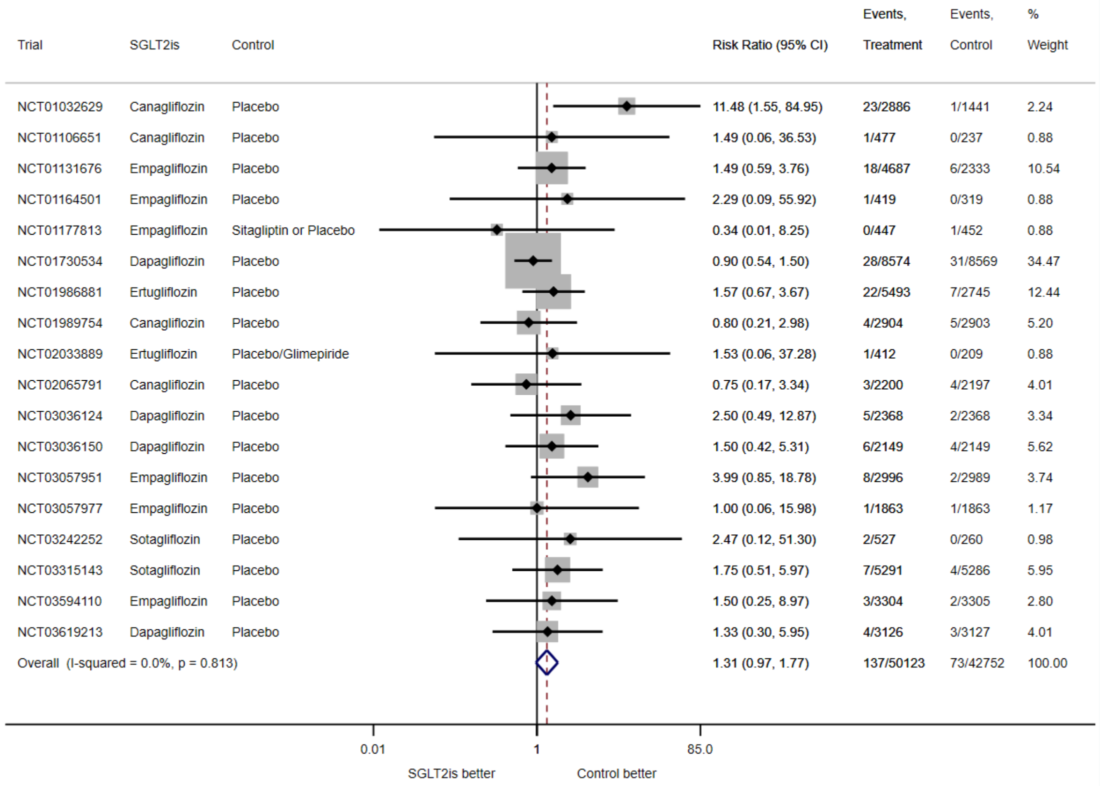

System 21-Reproductive System And Breast Disorders-Figure S4 Meta-analysis of SGLT2is and Benign prostatic hyperplasia

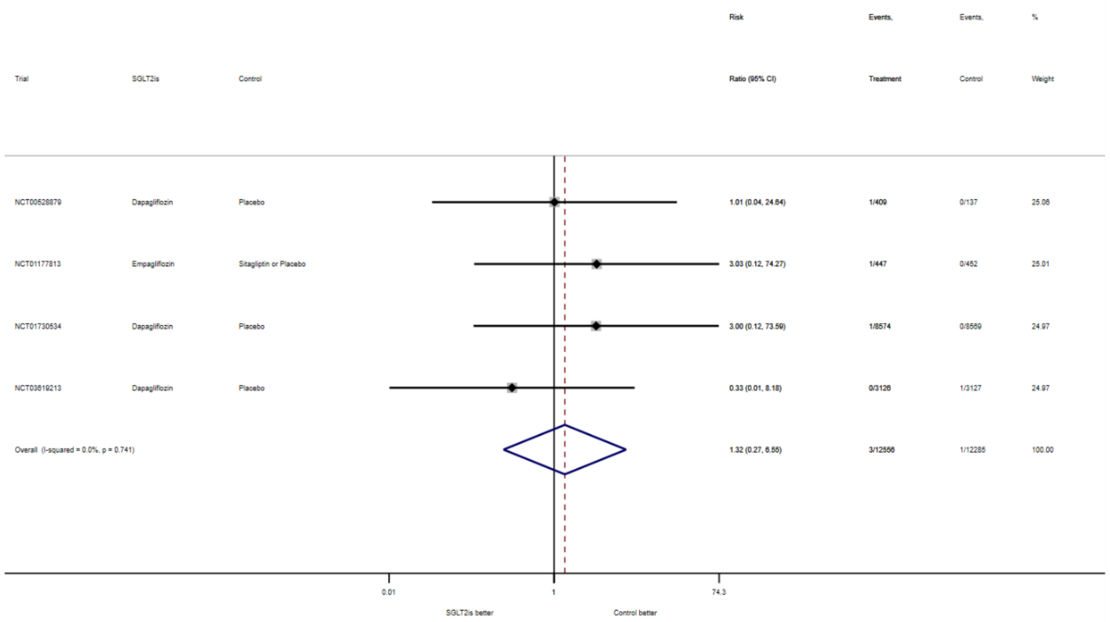

System 21-Reproductive System And Breast Disorders-Figure S5 Meta-analysis of SGLT2is and Breast mass

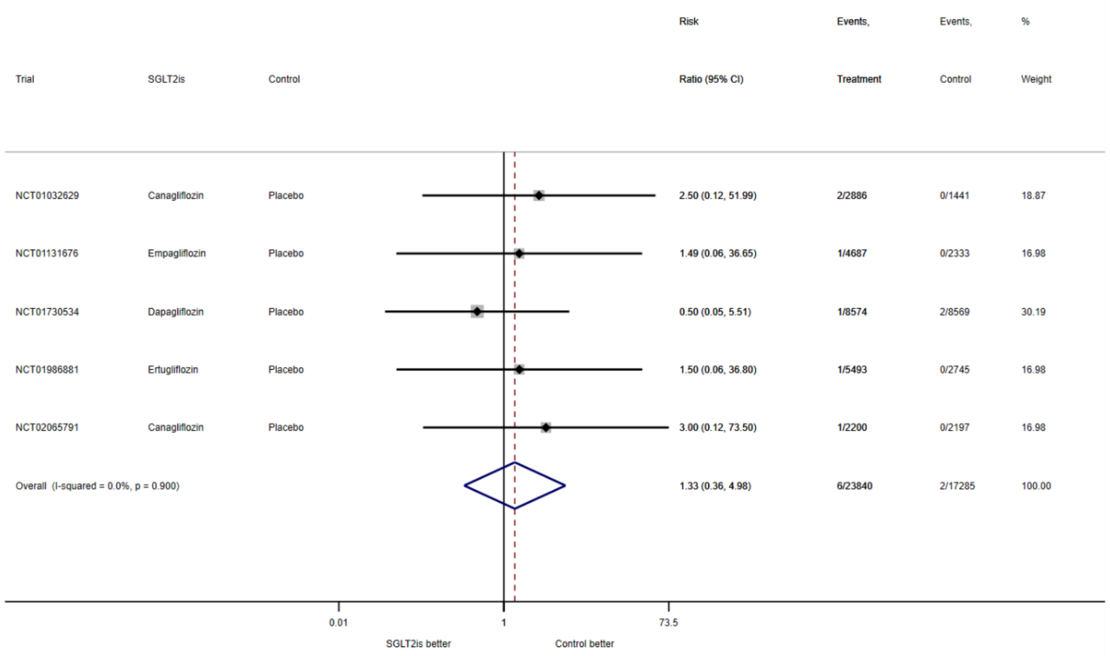

System 21-Reproductive System And Breast Disorders-Figure S6 Meta-analysis of SGLT2is and Cystocele

Trial	SGLT2is	Control	Risk Ratio (95% CI)	Events, Treatment	Events, Control	% Weight
NCT01032629	Canagliflozin	Placebo	0.50 (0.03, 7.98)	1/2886	1/1441	18.19
NCT01131676	Empagliflozin	Placebo	0.17 (0.01, 4.07)	0/4687	1/2333	13.64
NCT01730534	Dapagliflozin	Placebo	0.33 (0.03, 3.20)	1/8574	3/8569	27.27
NCT01989754	Canagliflozin	Placebo	3.00 (0.12, 73.59)	1/2904	0/2903	13.64
NCT03036124	Dapagliflozin	Placebo	0.33 (0.01, 8.18)	0/2368	1/2368	13.64
NCT03315143	Sotagliflozin	Placebo	0.33 (0.01, 8.17)	0/5291	1/5286	13.63
Overall (I-squared = 0.0%, p = 0.868)			0.44 (0.13, 1.43)	3/26710	7/22900	100.00

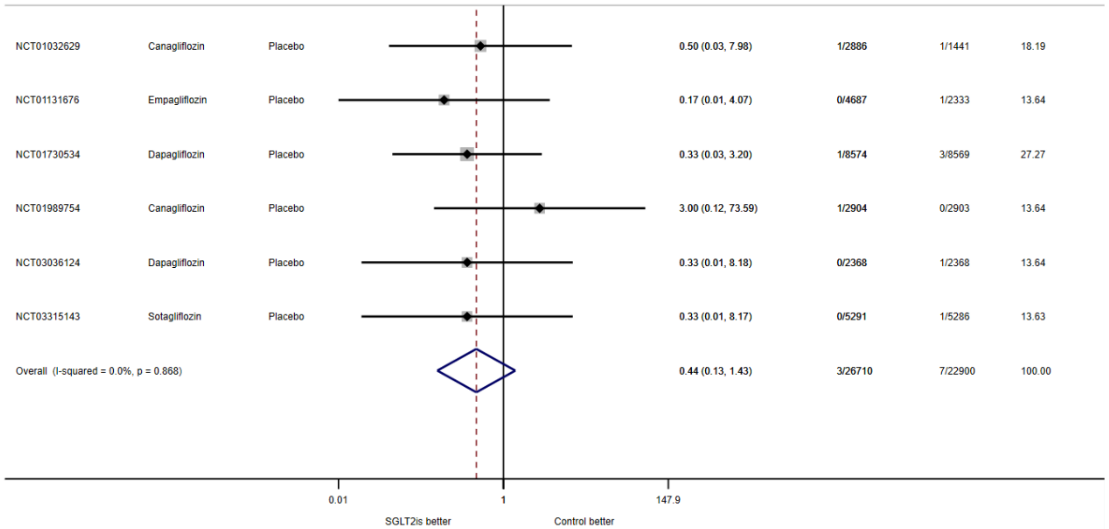

System 21-Reproductive System And Breast Disorders-Figure S7 Meta-analysis of SGLT2is and Endometrial hyperplasia

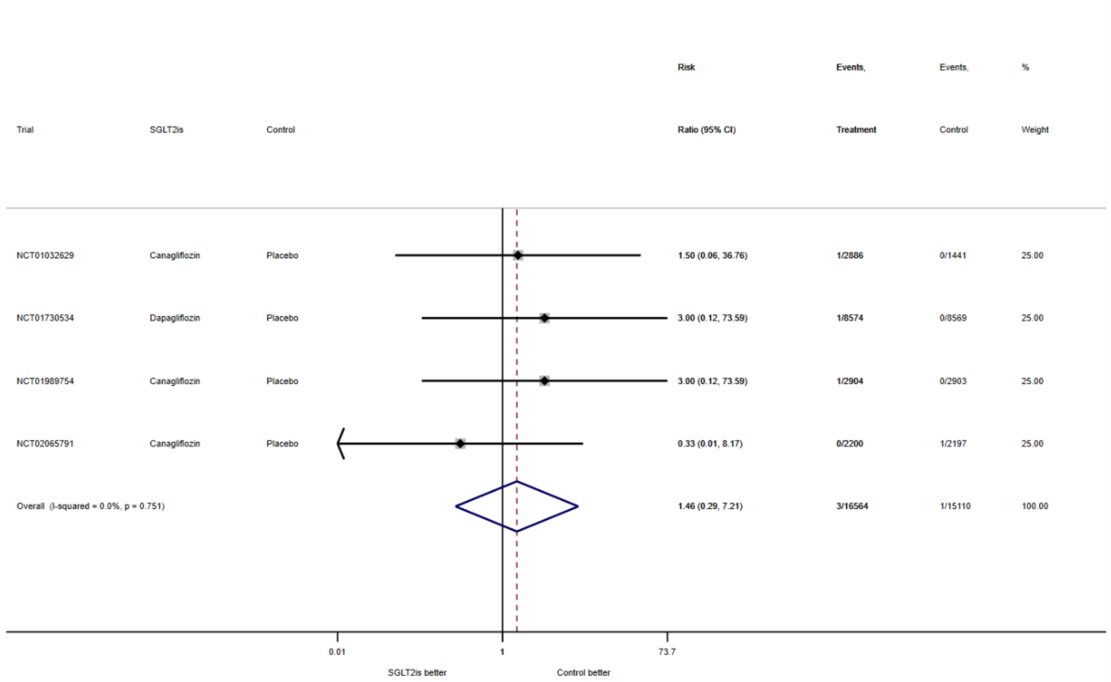

System 21-Reproductive System And Breast Disorders-Figure S8 Meta-analysis of SGLT2is and Erectile dysfunction

Trial	SGLT2is	Control	Risk Ratio (95% CI)	Events, Treatment	Events, Control	% Weight
NCT01032629	Canagliflozin	Placebo	2.50 (0.12, 51.99)	2/2886	0/1441	17.86
NCT01131676	Empagliflozin	Placebo	2.49 (0.12, 51.83)	2/4687	0/2333	17.86
NCT01730534	Dapagliflozin	Placebo	0.33 (0.01, 8.18)	0/8574	1/8569	16.07
NCT01986881	Ertugliflozin	Placebo	0.17 (0.01, 4.09)	0/5493	1/2745	16.07
NCT02065791	Canagliflozin	Placebo	3.00 (0.12, 73.50)	1/2200	0/2197	16.07
NCT03036124	Dapagliflozin	Placebo	0.33 (0.01, 8.18)	0/2368	1/2368	16.07
Overall (I-squared = 0.0%, p = 0.667)			0.87 (0.24, 3.14)	5/26208	3/19653	100.00

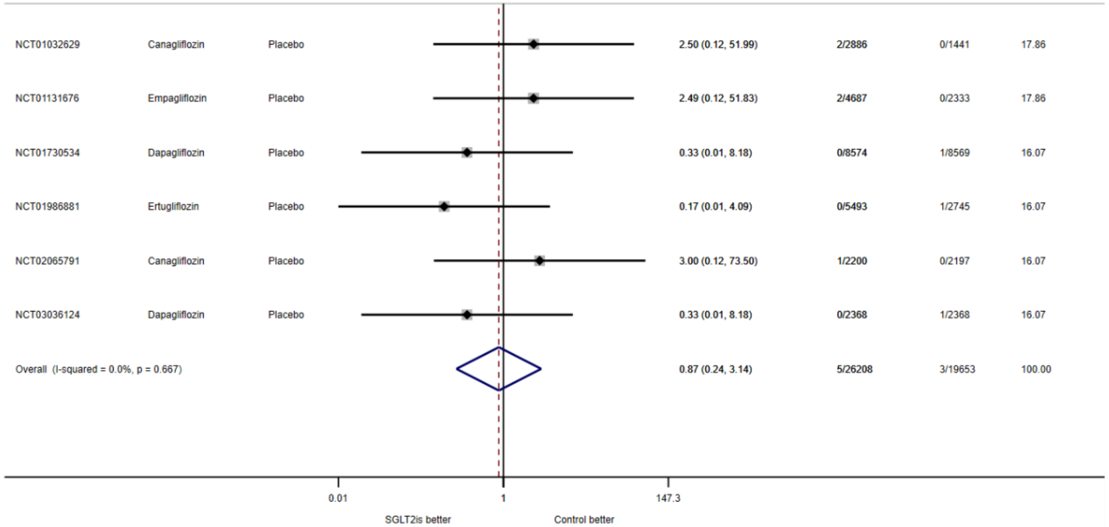

System 21-Reproductive System And Breast Disorders-Figure S9 Meta-analysis of SGLT2is and Ovarian cyst

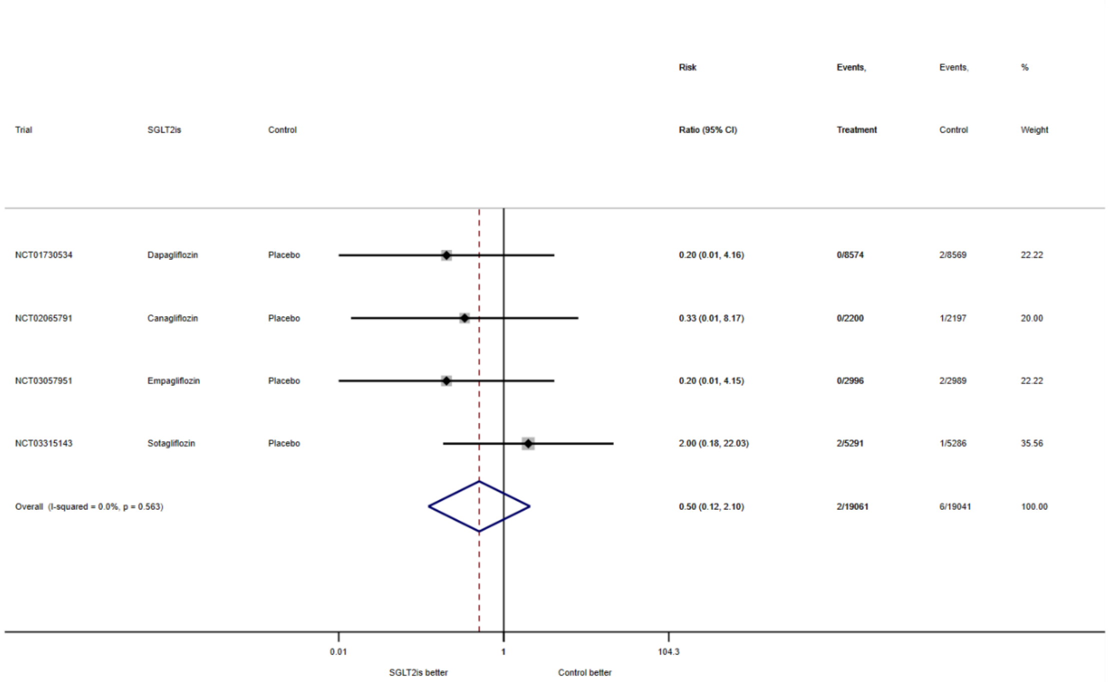

System 21-Reproductive System And Breast Disorders-Figure S10 Meta-analysis of SGLT2is and Postmenopausal haemorrhage

Trial	SGLT2is	Control	Risk Ratio (95% CI)	Events, Treatment	Events, Control	% Weight
NCT01032629	Canagliflozin	Placebo	1.50 (0.16, 14.39)	3/2886	1/1441	10.41
NCT01131676	Empagliflozin	Placebo	1.00 (0.18, 5.43)	4/4687	2/2333	18.50
NCT01730534	Dapagliflozin	Placebo	0.50 (0.15, 1.66)	4/8574	8/8569	37.00
NCT01986881	Ertugliflozin	Placebo	2.50 (0.12, 52.04)	2/5493	0/2745	5.78
NCT01989754	Canagliflozin	Placebo	0.33 (0.03, 3.20)	1/2904	3/2903	10.40
NCT02065791	Canagliflozin	Placebo	1.00 (0.06, 15.96)	1/2200	1/2197	6.94
NCT03057951	Empagliflozin	Placebo	0.20 (0.01, 4.15)	0/2996	2/2989	5.78
NCT03315143	Sotagliflozin	Placebo	3.00 (0.12, 73.56)	1/5291	0/5286	5.20
Overall (I-squared = 0.0%, p = 0.836)			0.73 (0.35, 1.52)	16/35031	17/28463	100.00

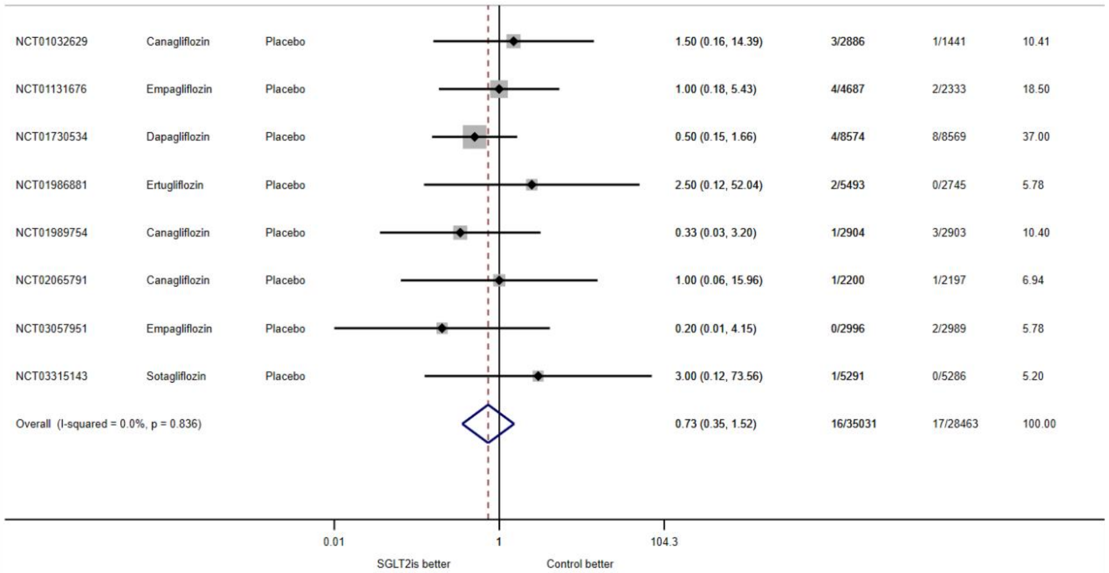

System 21-Reproductive System And Breast Disorders-Figure S11 Meta-analysis of SGLT2is and Prostatitis

Trial	SGLT2is	Control	Risk Ratio (95% CI)	Events, Treatment	Events, Control	% Weight
NCT01032629	Canagliflozin	Placebo	3.50 (0.18, 67.64)	3/2886	0/1441	13.91
NCT01730534	Dapagliflozin	Placebo	0.20 (0.02, 1.71)	1/8574	5/8569	26.49
NCT01986881	Ertugliflozin	Placebo	0.17 (0.01, 4.09)	0/5493	1/2745	11.92
NCT01989754	Canagliflozin	Placebo	3.00 (0.12, 73.59)	1/2904	0/2903	11.92
NCT03036150	Dapagliflozin	Placebo	3.00 (0.12, 73.60)	1/2149	0/2149	11.92
NCT03315143	Sotagliflozin	Placebo	3.00 (0.12, 73.56)	1/5291	0/5286	11.92
NCT03619213	Dapagliflozin	Placebo	3.00 (0.12, 73.64)	1/3126	0/3127	11.92
Overall (I-squared = 0.0%, p = 0.440)			1.06 (0.35, 3.20)	8/30423	6/26220	100.00

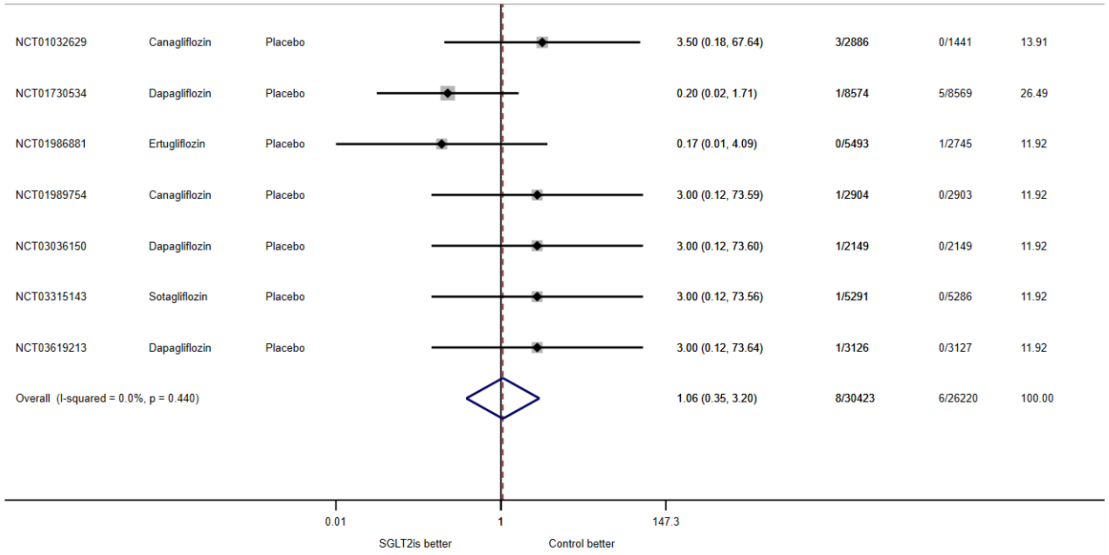

System 21-Reproductive System And Breast Disorders-Figure S12 Meta-analysis of SGLT2is and Prostatomegaly

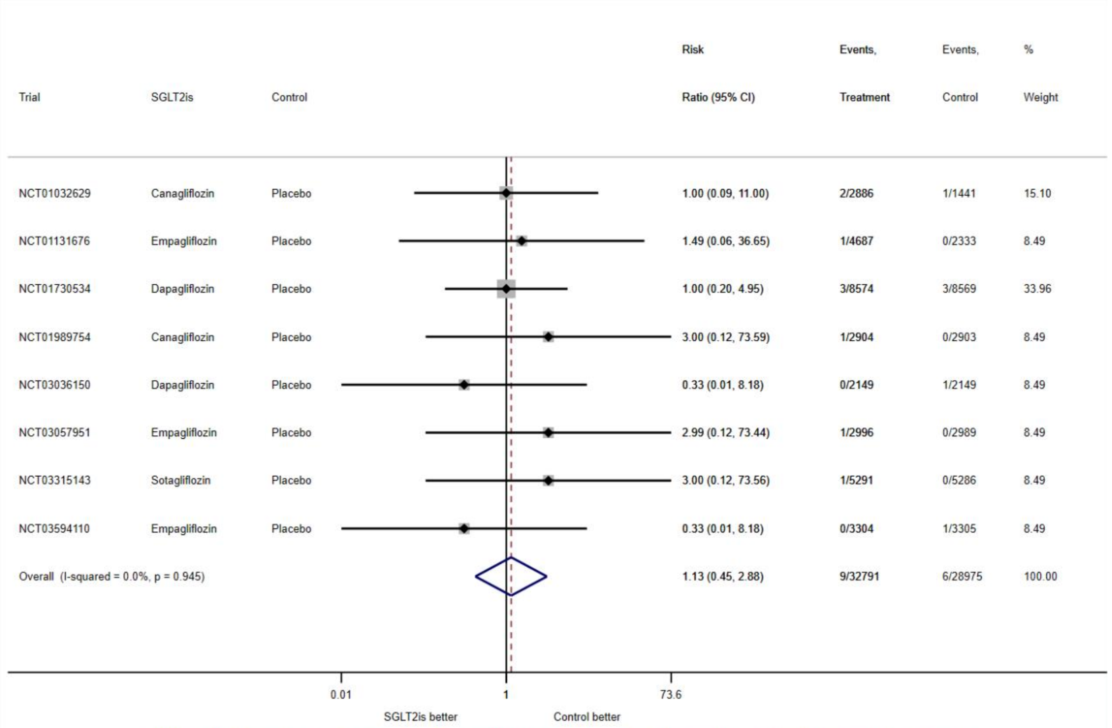

System 21-Reproductive System And Breast Disorders-Figure S13 Meta-analysis of SGLT2is and Uterine polyp

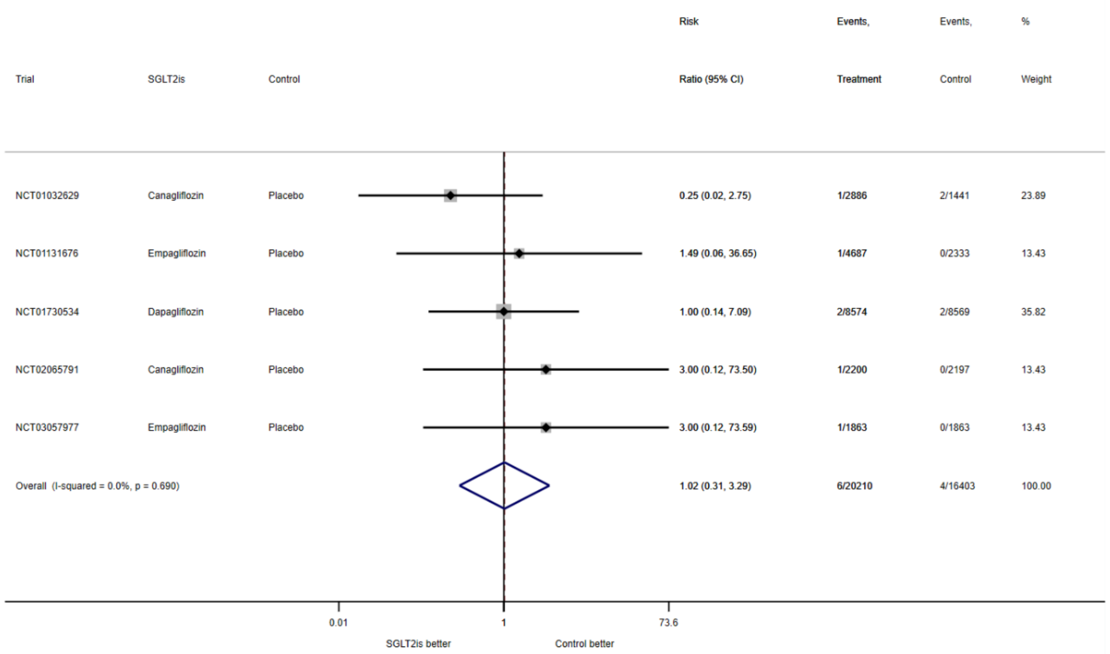

System 21-Reproductive System And Breast Disorders-Figure S14 Meta-analysis of SGLT2is and Uterovaginal prolapse

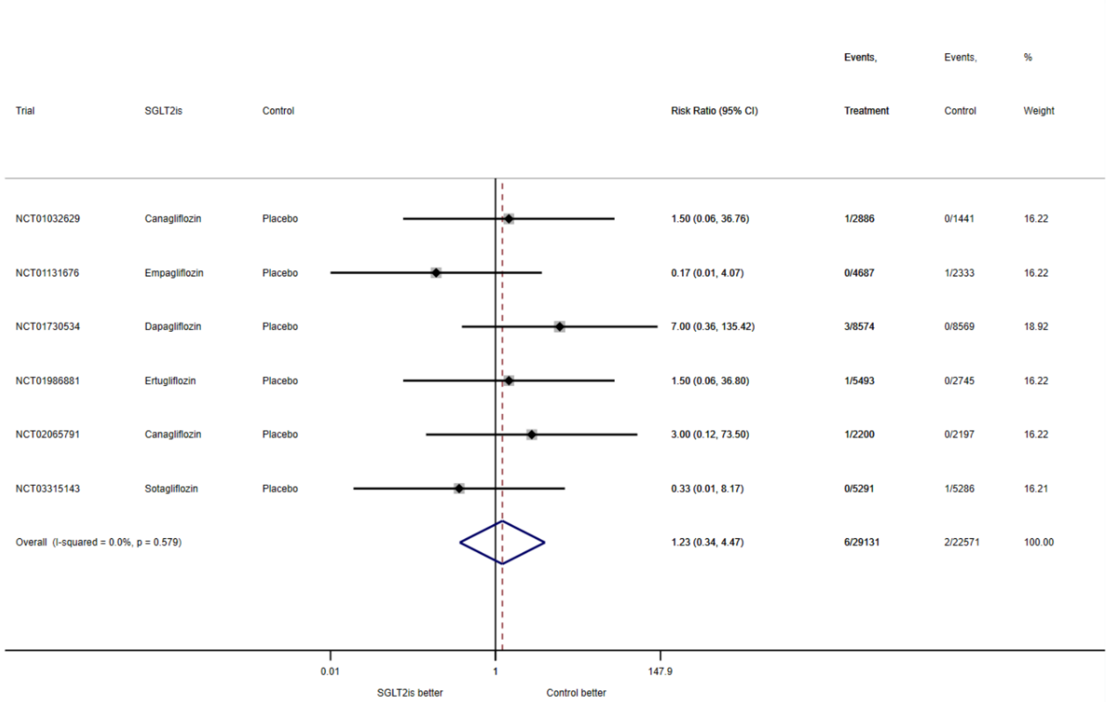

System 21-Reproductive System And Breast Disorders-Figure S15 Meta-analysis of SGLT2is and Vaginal prolapse

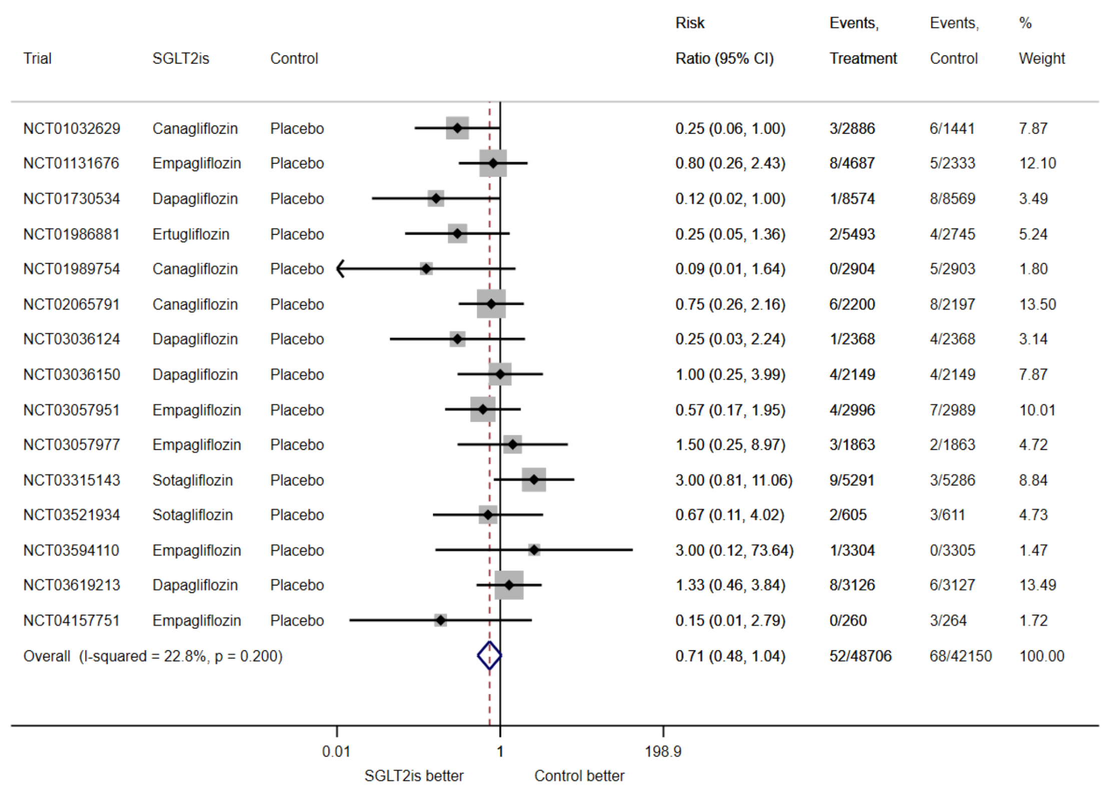

System 22-Respiratory, Thoracic And Mediastinal Disorders-Figure S1 Meta-analysis of SGLT2is and Acute pulmonary oedema

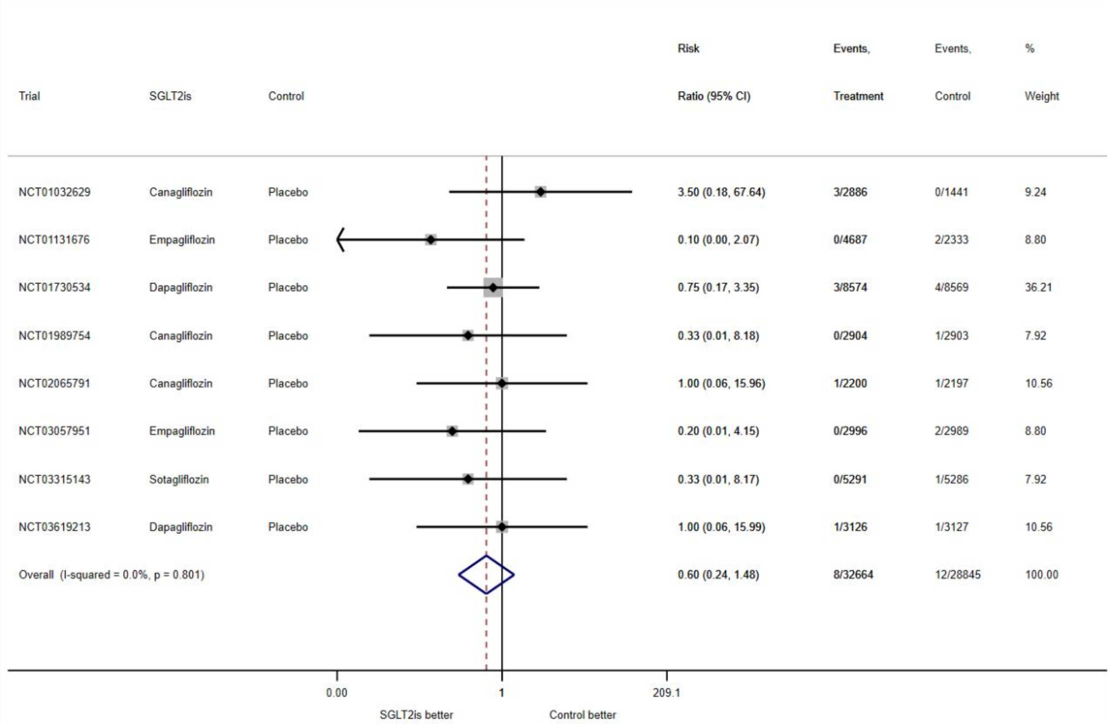

System 22-Respiratory, Thoracic And Mediastinal Disorders-Figure S2 Meta-analysis of SGLT2is and Acute respiratory distress syndrome

Trial	SGLT2is	Control	Risk Ratio (95% CI)	Events, Treatment	Events, Control	% Weight
NCT01032629	Canagliflozin	Placebo	2.50 (0.12, 51.99)	2/2886	0/1441	0.60
NCT01131676	Empagliflozin	Placebo	1.00 (0.30, 3.30)	8/4687	4/2333	3.87
NCT01730534	Dapagliflozin	Placebo	0.86 (0.55, 1.33)	37/8574	43/8569	28.96
NCT01986881	Ertugliflozin	Placebo	0.83 (0.36, 1.90)	15/5493	9/2745	8.18
NCT01989754	Canagliflozin	Placebo	0.33 (0.03, 3.20)	1/2904	3/2903	1.09
NCT02065791	Canagliflozin	Placebo	0.83 (0.25, 2.72)	5/2200	6/2197	3.96
NCT03036124	Dapagliflozin	Placebo	0.50 (0.20, 1.24)	7/2368	14/2368	6.79
NCT03036150	Dapagliflozin	Placebo	1.09 (0.48, 2.47)	12/2149	11/2149	8.36
NCT03057951	Empagliflozin	Placebo	0.40 (0.18, 0.90)	8/2996	20/2989	8.31
NCT03057977	Empagliflozin	Placebo	0.60 (0.14, 2.51)	3/1863	5/1863	2.72
NCT03315143	Sotagliflozin	Placebo	0.92 (0.40, 2.07)	11/5291	12/5286	8.34
NCT03521934	Sotagliflozin	Placebo	1.01 (0.06, 16.11)	1/605	1/611	0.73
NCT03594110	Empagliflozin	Placebo	0.40 (0.08, 2.06)	2/3304	5/3305	2.07
NCT03619213	Dapagliflozin	Placebo	0.94 (0.48, 1.86)	16/3126	17/3127	12.01
NCT04157751	Empagliflozin	Placebo	0.34 (0.04, 3.23)	1/260	3/264	1.09
NCT04350593	Dapagliflozin	Placebo	1.00 (0.25, 4.00)	4/613	4/616	2.92
Overall (I-squared = 0.0%, p = 0.941)			0.78 (0.62, 0.99)	133/49319	157/42766	100.00

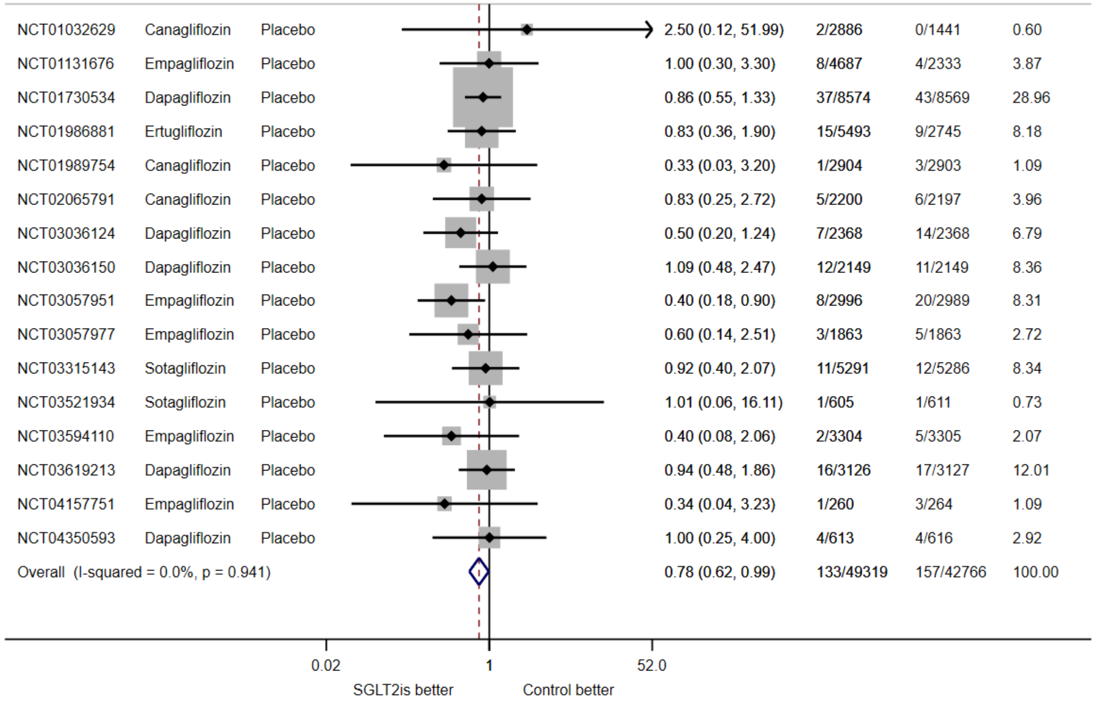

System 22-Respiratory, Thoracic And Mediastinal Disorders-Figure S3 Meta-analysis of SGLT2is and Acute respiratory failure

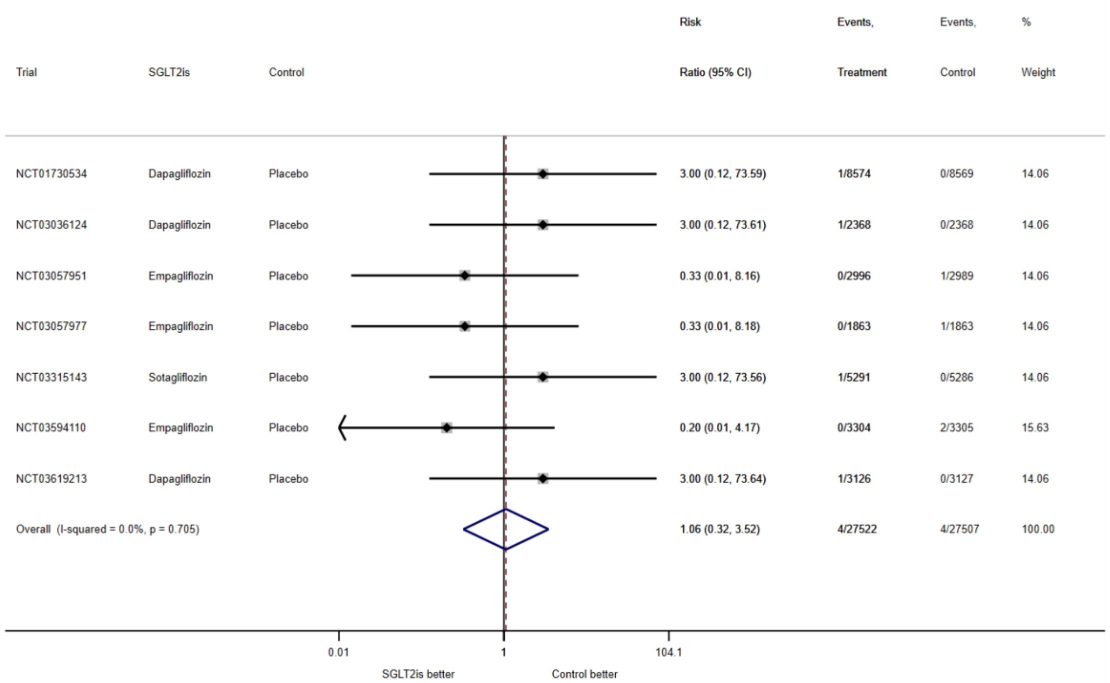

System 22-Respiratory, Thoracic And Mediastinal Disorders-Figure S4 Meta-analysis of SGLT2is and Aspiration

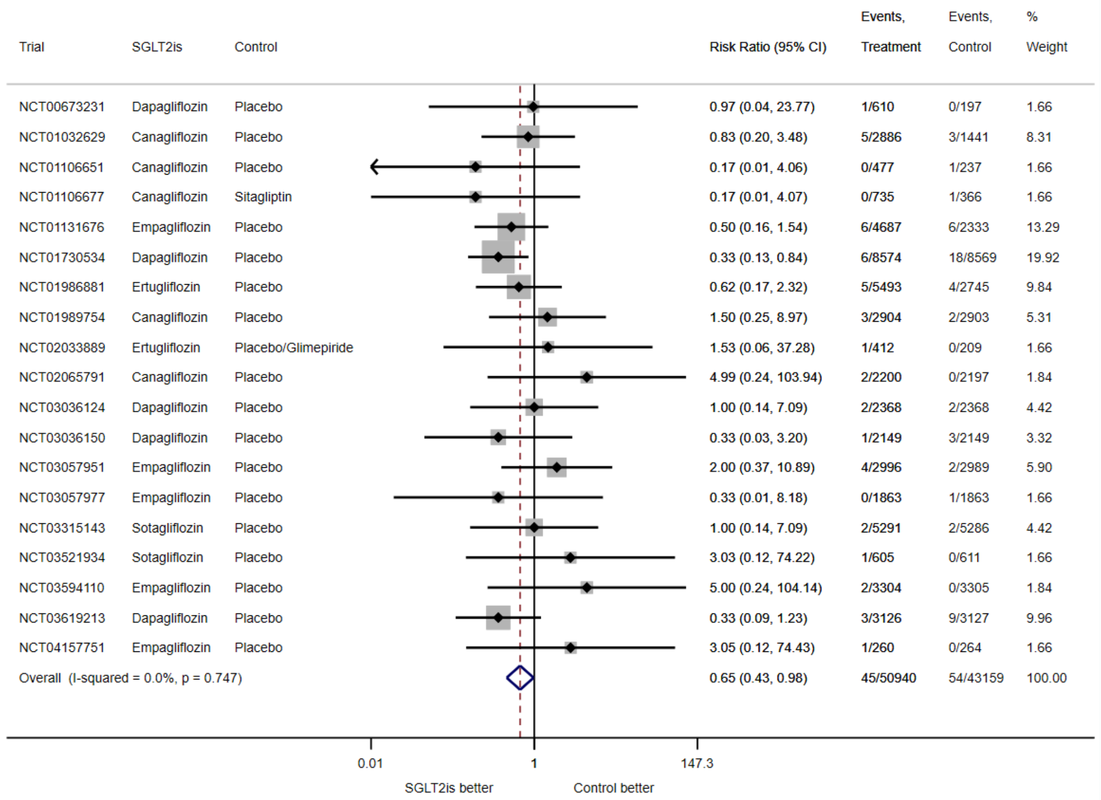

System 22-Respiratory, Thoracic And Mediastinal Disorders-Figure S5 Meta-analysis of SGLT2is and Asthma

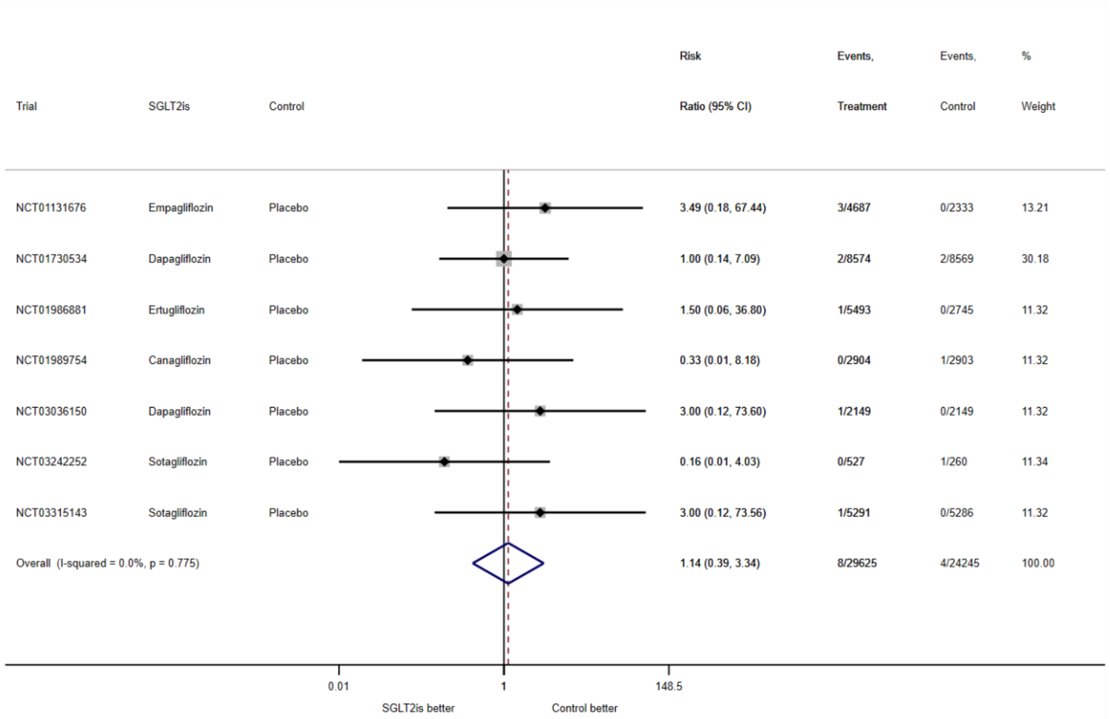

System 22-Respiratory, Thoracic And Mediastinal Disorders-Figure S6 Meta-analysis of SGLT2is and Atelectasis

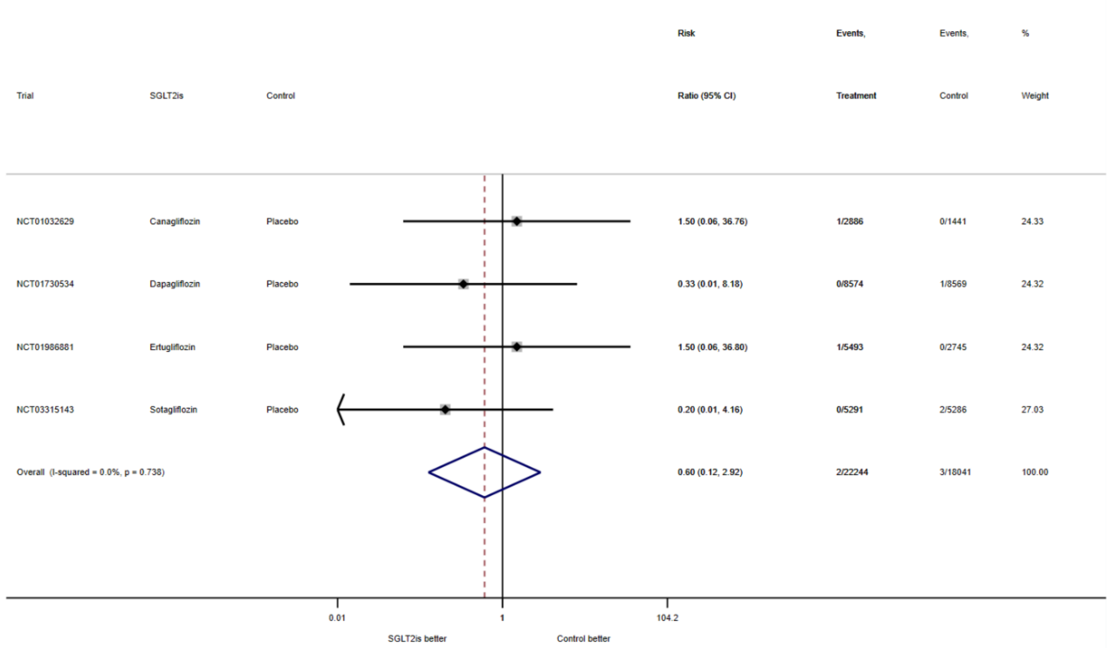

System 22-Respiratory, Thoracic And Mediastinal Disorders-Figure S7 Meta-analysis of SGLT2is and Bronchial hyperreactivity

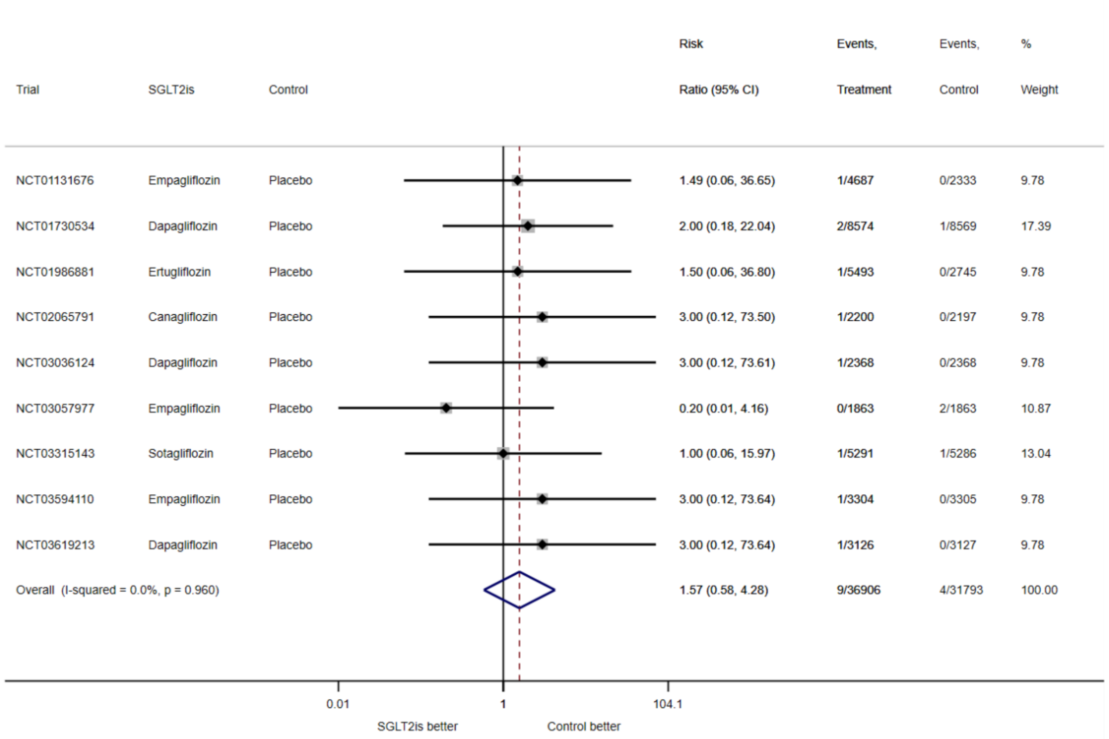

System 22-Respiratory, Thoracic And Mediastinal Disorders-Figure S8 Meta-analysis of SGLT2is and Bronchiectasis

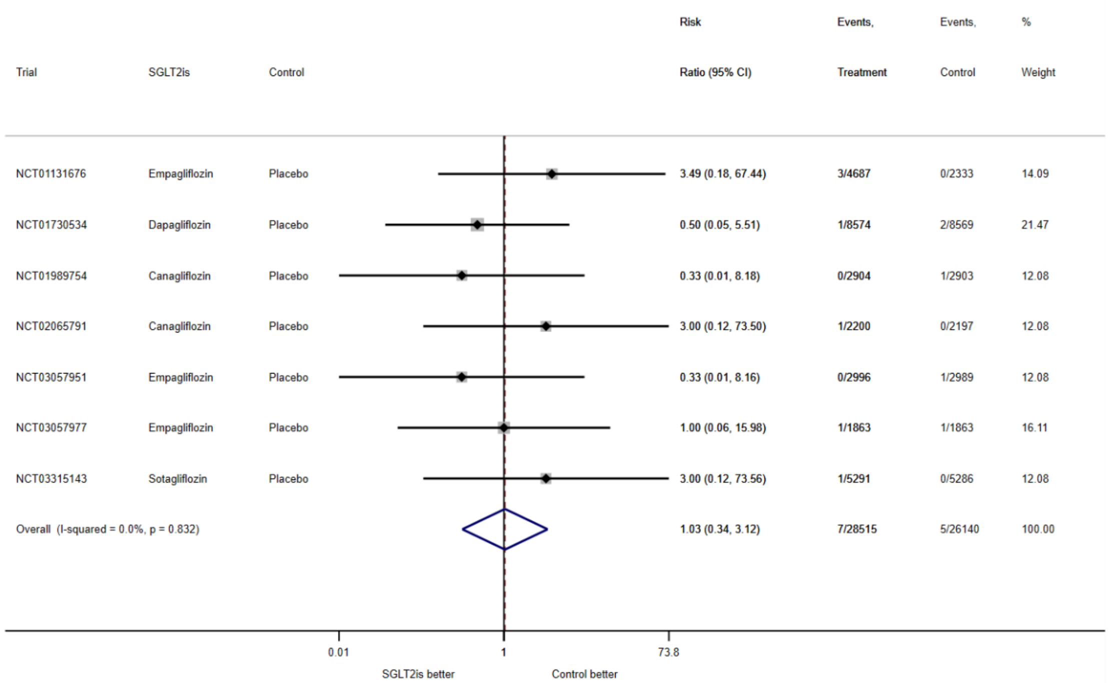

System 22-Respiratory, Thoracic And Mediastinal Disorders-Figure S9 Meta-analysis of SGLT2is and Bronchitis chronic

Trial	SGLT2is	Control	Risk Ratio (95% CI)	Events, Treatment	Events, Control	% Weight
NCT01131676	Empagliflozin	Placebo	0.10 (0.00, 2.07)	0/4687	2/2333	14.49
NCT01730534	Dapagliflozin	Placebo	0.33 (0.01, 8.18)	0/8574	1/8569	13.04
NCT01986881	Ertugliflozin	Placebo	1.50 (0.06, 36.80)	1/5493	0/2745	13.04
NCT01989754	Canagliflozin	Placebo	0.50 (0.05, 5.51)	1/2904	2/2903	23.19
NCT02065791	Canagliflozin	Placebo	3.00 (0.12, 73.50)	1/2200	0/2197	13.04
NCT03057951	Empagliflozin	Placebo	2.00 (0.18, 21.99)	2/2996	1/2989	23.19
Overall (I-squared = 0.0%, p = 0.609)			0.75 (0.24, 2.40)	5/26854	6/21736	100.00

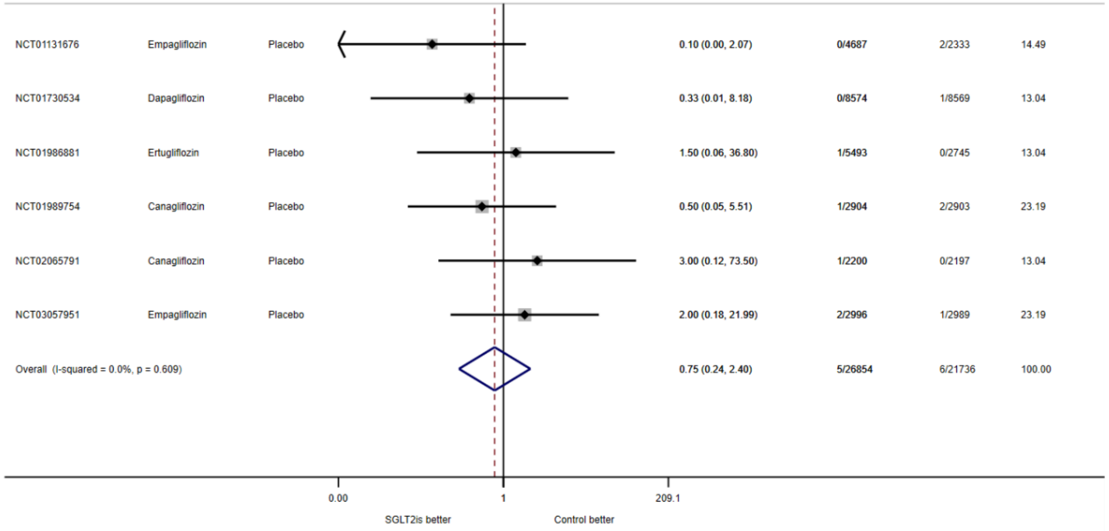

System 22-Respiratory, Thoracic And Mediastinal Disorders-Figure S10 Meta-analysis of SGLT2is and Bronchospasm

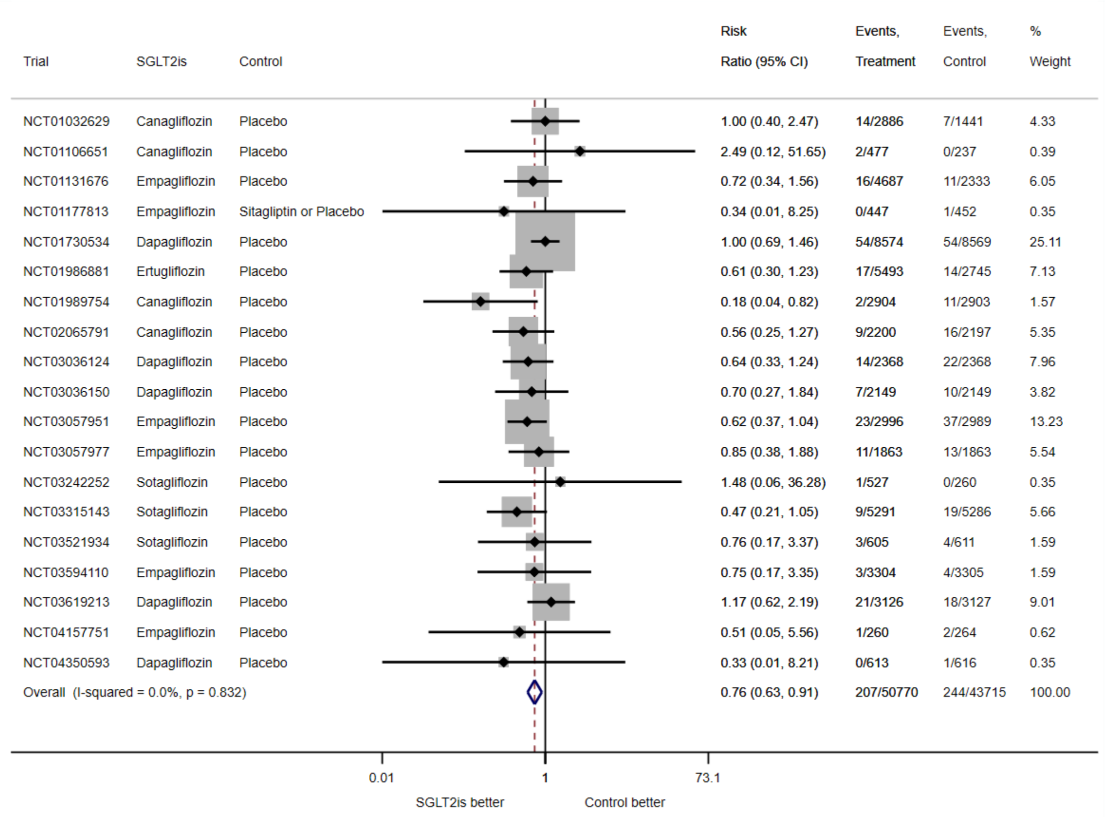

stem 22-Respiratory, Thoracic And Mediastinal Disorders-Figure S11 Meta-analysis of SGLT2is and Chronic obstructive pulmonary dise

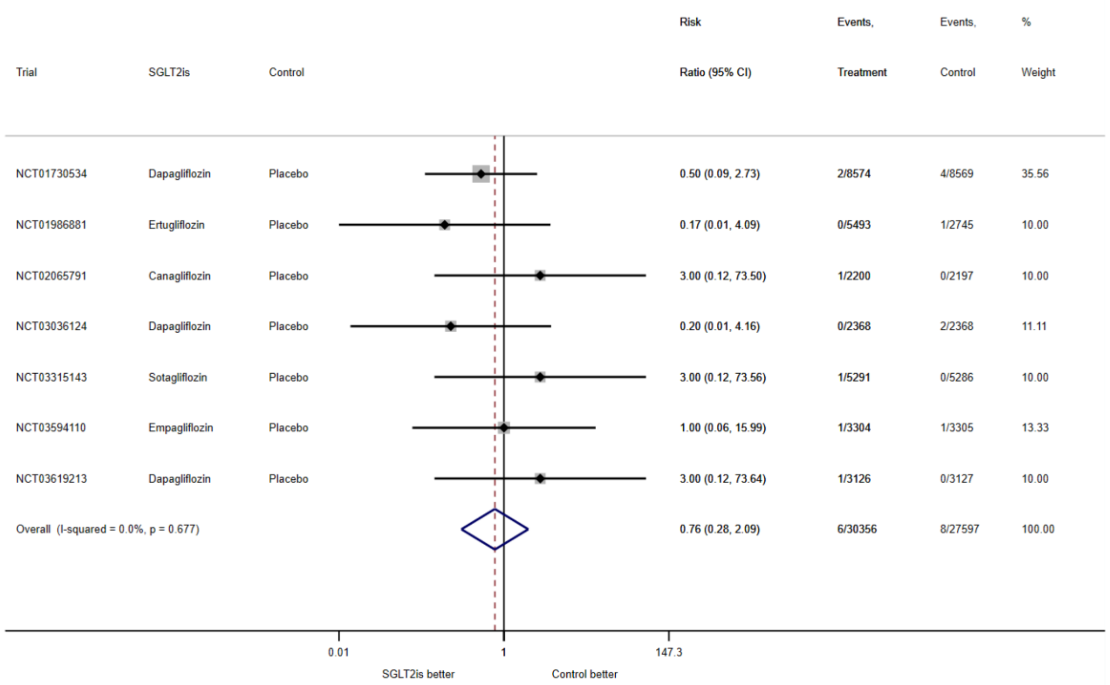

System 22-Respiratory, Thoracic And Mediastinal Disorders-Figure S12 Meta-analysis of SGLT2is and Chronic respiratory failure

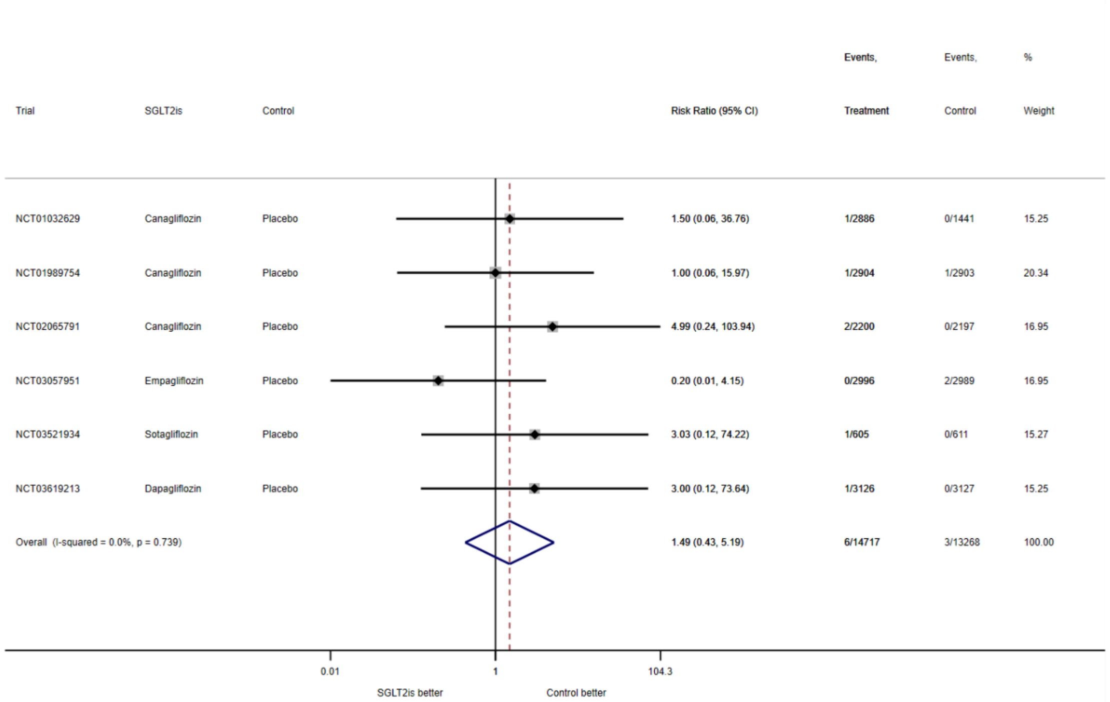

System 22-Respiratory, Thoracic And Mediastinal Disorders-Figure S13 Meta-analysis of SGLT2is and Cough

Trial	SGLT2is	Control	Risk Ratio (95% CI)	Events, Treatment	Events, Control	% Weight
NCT00528879	Dapagliflozin	Placebo	1.01 (0.04, 24.64)	1/409	0/137	0.85
NCT01032629	Canagliflozin	Placebo	1.12 (0.49, 2.58)	18/2886	8/1441	12.60
NCT01131676	Empagliflozin	Placebo	0.53 (0.26, 1.10)	15/4687	14/2333	16.45
NCT01164501	Empagliflozin	Placebo	0.15 (0.01, 3.16)	0/419	2/319	0.94
NCT01730534	Dapagliflozin	Placebo	0.19 (0.07, 0.55)	4/8574	21/8569	7.61
NCT01986881	Ertugliflozin	Placebo	0.83 (0.20, 3.48)	5/5493	3/2745	4.25
NCT01989754	Canagliflozin	Placebo	1.00 (0.38, 2.66)	8/2904	8/2903	9.07
NCT02065791	Canagliflozin	Placebo	1.00 (0.20, 4.94)	3/2200	3/2197	3.40
NCT03036124	Dapagliflozin	Placebo	0.67 (0.19, 2.36)	4/2368	6/2368	5.44
NCT03036150	Dapagliflozin	Placebo	0.50 (0.05, 5.51)	1/2149	2/2149	1.51
NCT03057951	Empagliflozin	Placebo	2.99 (0.81, 11.04)	9/2996	3/2989	5.10
NCT03057977	Empagliflozin	Placebo	1.75 (0.51, 5.97)	7/1863	4/1863	5.77
NCT03242252	Sotagliflozin	Placebo	1.48 (0.06, 36.28)	1/527	0/260	0.85
NCT03315143	Sotagliflozin	Placebo	0.90 (0.37, 2.21)	9/5291	10/5286	10.73
NCT03521934	Sotagliflozin	Placebo	0.67 (0.11, 4.02)	2/605	3/611	2.72
NCT03594110	Empagliflozin	Placebo	1.20 (0.37, 3.93)	6/3304	5/3305	6.18
NCT03619213	Dapagliflozin	Placebo	0.60 (0.14, 2.51)	3/3126	5/3127	4.25
NCT04157751	Empagliflozin	Placebo	1.02 (0.14, 7.15)	2/260	2/264	2.28
Overall (I-squared = 0.0%, p = 0.466)			0.81 (0.60, 1.08)	98/50061	99/42866	100.00

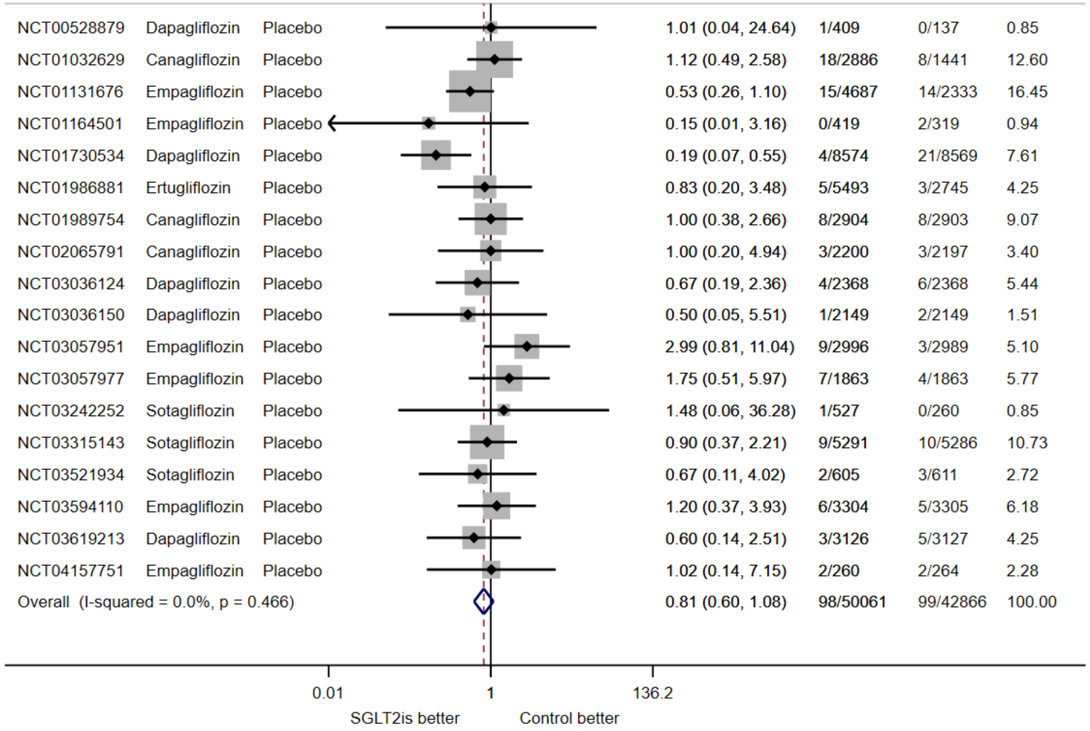

System 22-Respiratory, Thoracic And Mediastinal Disorders-Figure S14 Meta-analysis of SGLT2is and Dyspnoea

Trial	SGLT2is	Control	Risk Ratio (95% CI)	Events, Treatment	Events, Control	% Weight
NCT01032629	Canagliflozin	Placebo	0.67 (0.15, 2.97)	4/2886	3/1441	20.49
NCT01131676	Empagliflozin	Placebo	0.25 (0.02, 2.74)	1/4687	2/2333	7.96
NCT01730534	Dapagliflozin	Placebo	0.80 (0.21, 2.98)	4/8574	5/8569	26.53
NCT01986881	Ertugliflozin	Placebo	3.50 (0.18, 67.71)	3/5493	0/2745	5.22
NCT02384941	Sotagliflozin	Placebo	1.53 (0.06, 37.53)	1/525	0/268	4.48
NCT03036150	Dapagliflozin	Placebo	1.00 (0.06, 15.98)	1/2149	1/2149	5.97
NCT03057977	Empagliflozin	Placebo	0.20 (0.01, 4.16)	0/1863	2/1863	4.97
NCT03315143	Sotagliflozin	Placebo	0.50 (0.05, 5.51)	1/5291	2/5286	7.96
NCT03521934	Sotagliflozin	Placebo	0.34 (0.01, 8.25)	0/605	1/611	4.48
NCT03619213	Dapagliflozin	Placebo	1.00 (0.14, 7.10)	2/3126	2/3127	11.94
Overall (I-squared = 0.0%, p = 0.954)			0.70 (0.36, 1.38)	17/35199	18/28392	100.00

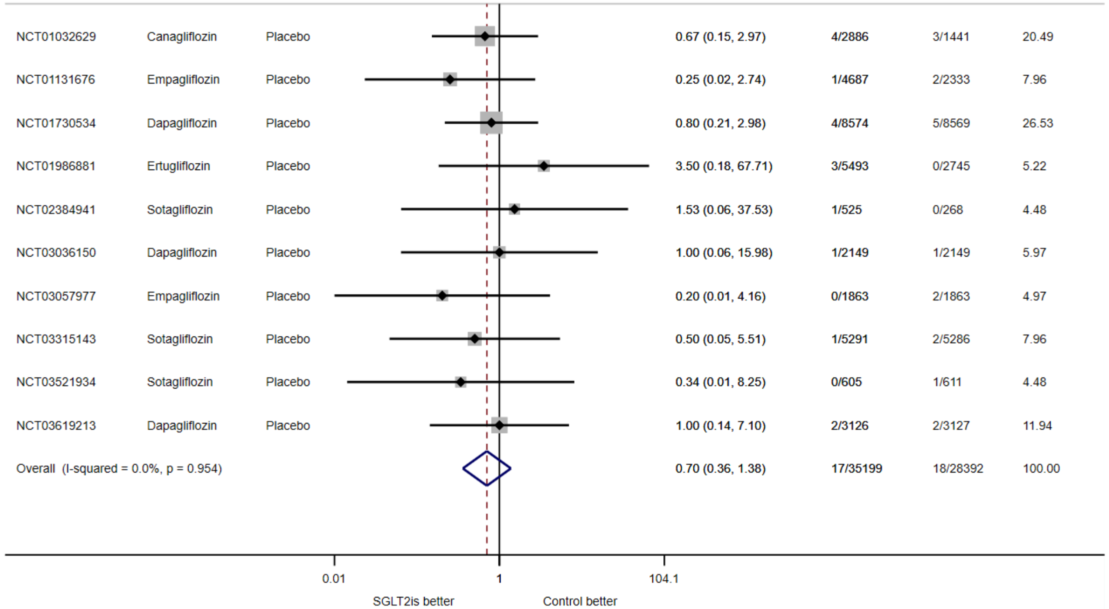

System 22-Respiratory, Thoracic And Mediastinal Disorders-Figure S15 Meta-analysis of SGLT2is and Dyspnoea exertional

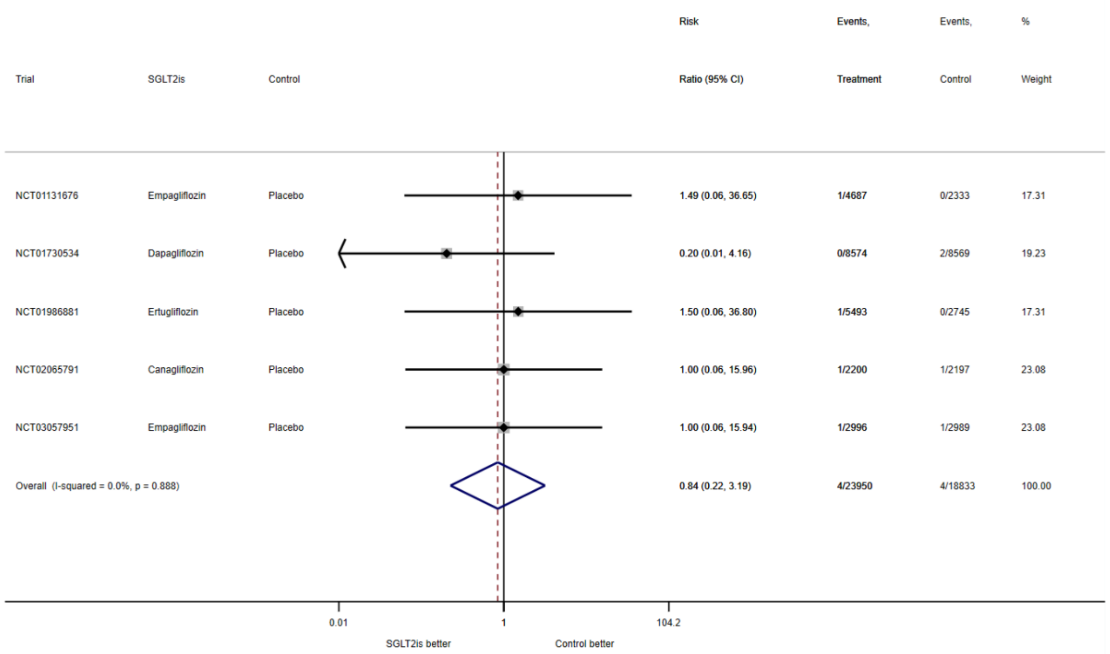

System 22-Respiratory, Thoracic And Mediastinal Disorders-Figure S16 Meta-analysis of SGLT2is and Emphysema

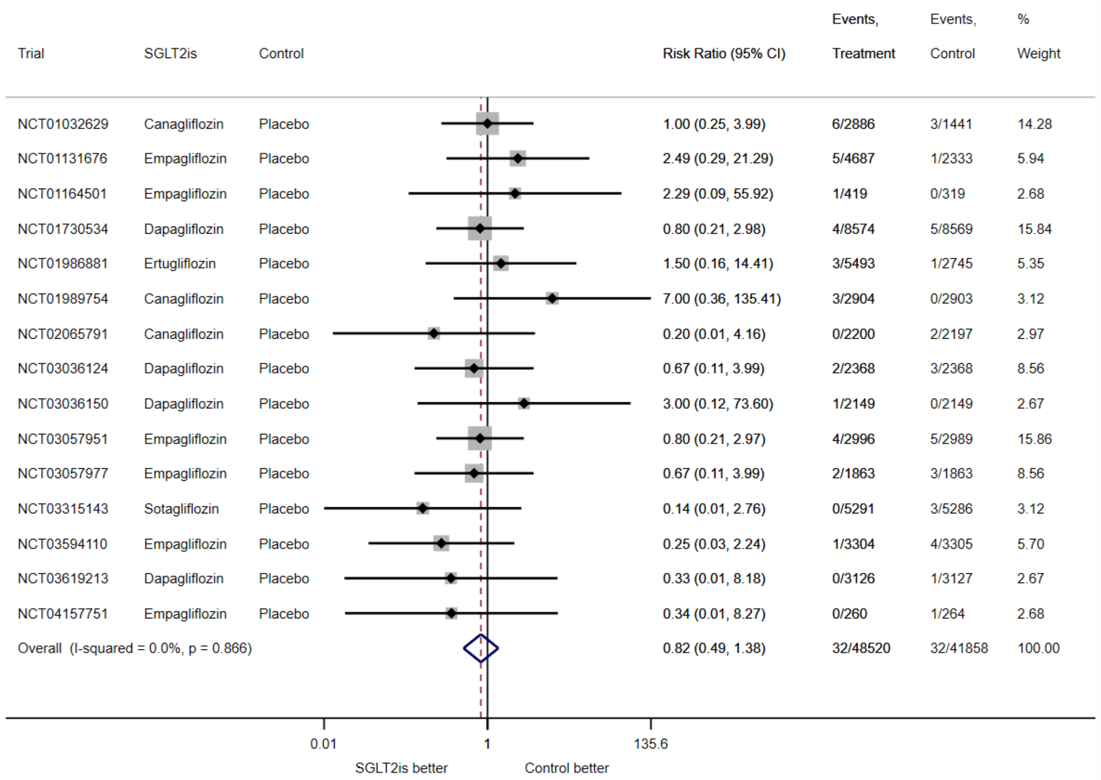

System 22-Respiratory, Thoracic And Mediastinal Disorders-Figure S17 Meta-analysis of SGLT2is and Epistaxis

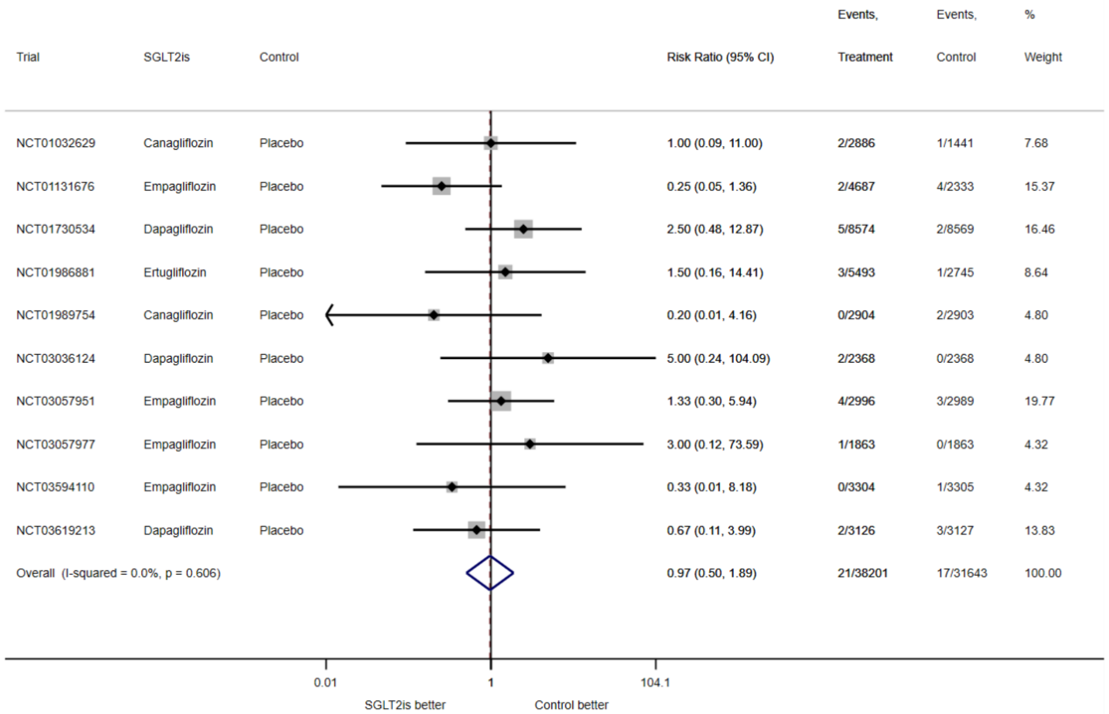

System 22-Respiratory, Thoracic And Mediastinal Disorders-Figure S18 Meta-analysis of SGLT2is and Haemoptysis

Trial	SGLT2is	Control	Risk Ratio (95% CI)	Events, Treatment	Events, Control	% Weight
NCT01131676	Empagliflozin	Placebo	0.17 (0.01, 4.07)	0/4687	1/2333	8.67
NCT01730534	Dapagliflozin	Placebo	1.50 (0.25, 8.97)	3/8574	2/8569	27.74
NCT01989754	Canagliflozin	Placebo	0.33 (0.01, 8.18)	0/2904	1/2903	8.67
NCT03036150	Dapagliflozin	Placebo	0.33 (0.01, 8.18)	0/2149	1/2149	8.67
NCT03057951	Empagliflozin	Placebo	1.00 (0.06, 15.94)	1/2996	1/2989	11.56
NCT03057977	Empagliflozin	Placebo	0.33 (0.01, 8.18)	0/1863	1/1863	8.67
NCT03594110	Empagliflozin	Placebo	3.00 (0.12, 73.64)	1/3304	0/3305	8.67
NCT03619213	Dapagliflozin	Placebo	0.33 (0.01, 8.18)	0/3126	1/3127	8.67
NCT04157751	Empagliflozin	Placebo	0.34 (0.01, 8.27)	0/260	1/264	8.69
Overall (I-squared = 0.0%, p = 0.912)			0.65 (0.26, 1.68)	5/29863	9/27502	100.00

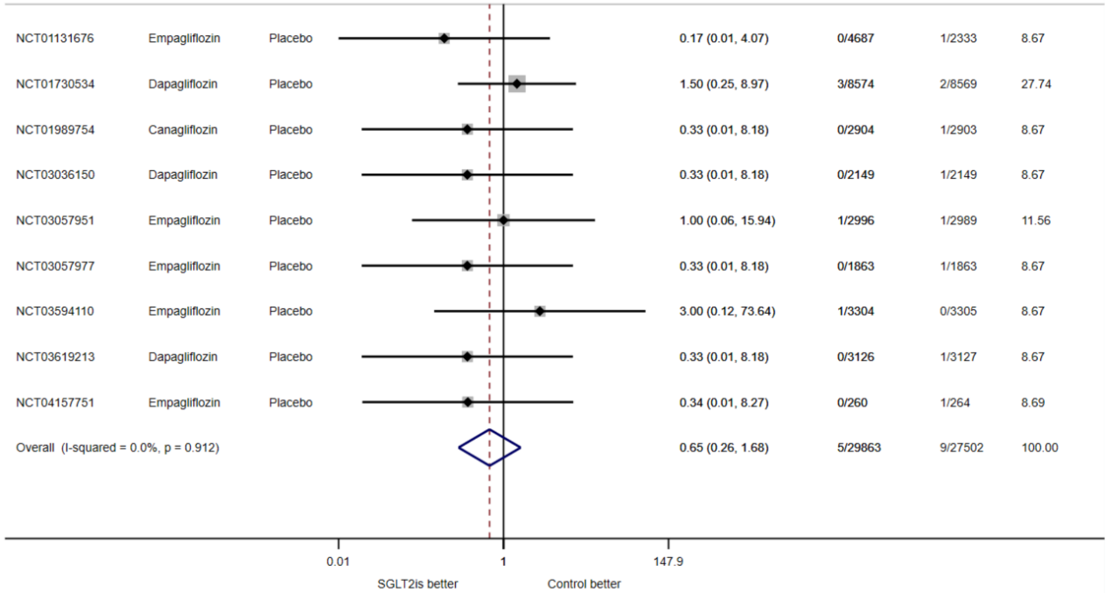

System 22-Respiratory, Thoracic And Mediastinal Disorders-Figure S19 Meta-analysis of SGLT2is and Haemothorax

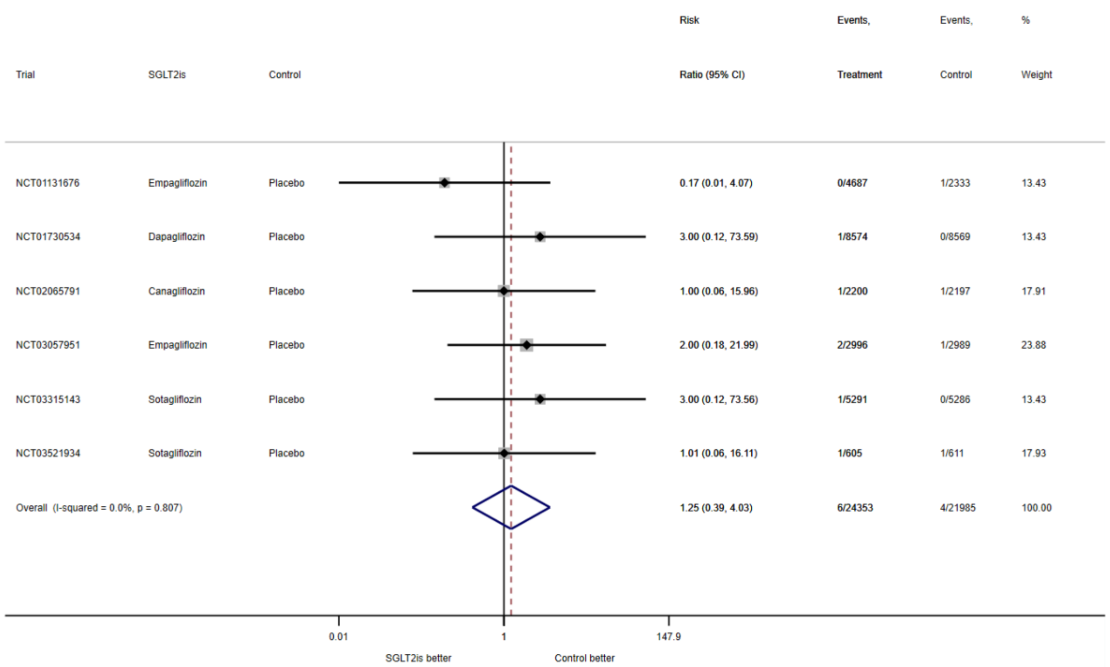

System 22-Respiratory, Thoracic And Mediastinal Disorders-Figure S20 Meta-analysis of SGLT2is and Hydrothorax

Trial	SGLT2is	Control	Risk Ratio (95% CI)	Events, Treatment	Events, Control	% Weight
NCT01032629	Canagliflozin	Placebo	1.00 (0.09, 11.00)	2/2886	1/1441	7.96
NCT01131676	Empagliflozin	Placebo	1.49 (0.16, 14.35)	3/4687	1/2333	8.96
NCT01164501	Empagliflozin	Placebo	0.76 (0.05, 12.13)	1/419	1/319	5.99
NCT01730534	Dapagliflozin	Placebo	1.00 (0.20, 4.95)	3/8574	3/8569	17.92
NCT01986881	Ertugliflozin	Placebo	0.25 (0.02, 2.75)	1/5493	2/2745	7.96
NCT02065791	Canagliflozin	Placebo	3.00 (0.12, 73.50)	1/2200	0/2197	4.48
NCT03036124	Dapagliflozin	Placebo	1.00 (0.06, 15.98)	1/2368	1/2368	5.97
NCT03036150	Dapagliflozin	Placebo	0.33 (0.01, 8.18)	0/2149	1/2149	4.48
NCT03057951	Empagliflozin	Placebo	0.50 (0.05, 5.50)	1/2996	2/2989	7.96
NCT03521934	Sotagliflozin	Placebo	3.03 (0.12, 74.22)	1/605	0/611	4.48
NCT03594110	Empagliflozin	Placebo	0.33 (0.01, 8.18)	0/3304	1/3305	4.48
NCT03619213	Dapagliflozin	Placebo	0.20 (0.01, 4.17)	0/3126	2/3127	4.98
NCT04350593	Dapagliflozin	Placebo	1.51 (0.25, 8.99)	3/613	2/616	14.38
Overall (I-squared = 0.0%, p = 0.969)			0.85 (0.43, 1.67)	17/39420	17/32769	100.00

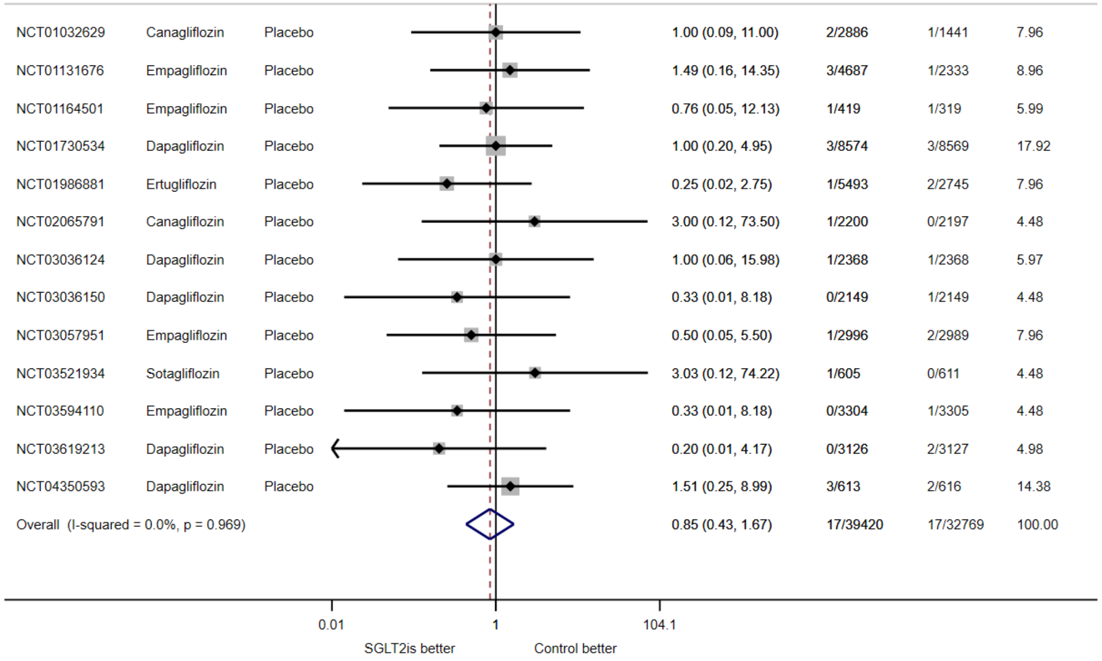

System 22-Respiratory, Thoracic And Mediastinal Disorders-Figure S21 Meta-analysis of SGLT2is and Hypoxia

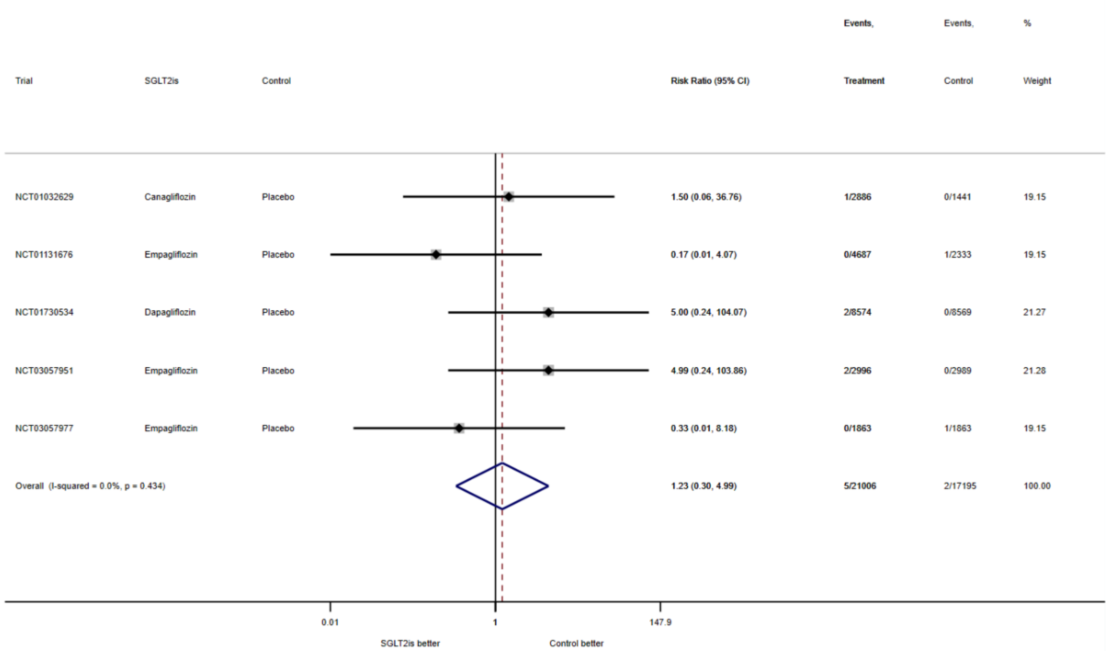

System 22-Respiratory, Thoracic And Mediastinal Disorders-Figure S22 Meta-analysis of SGLT2is and Idiopathic pulmonary fibrosis

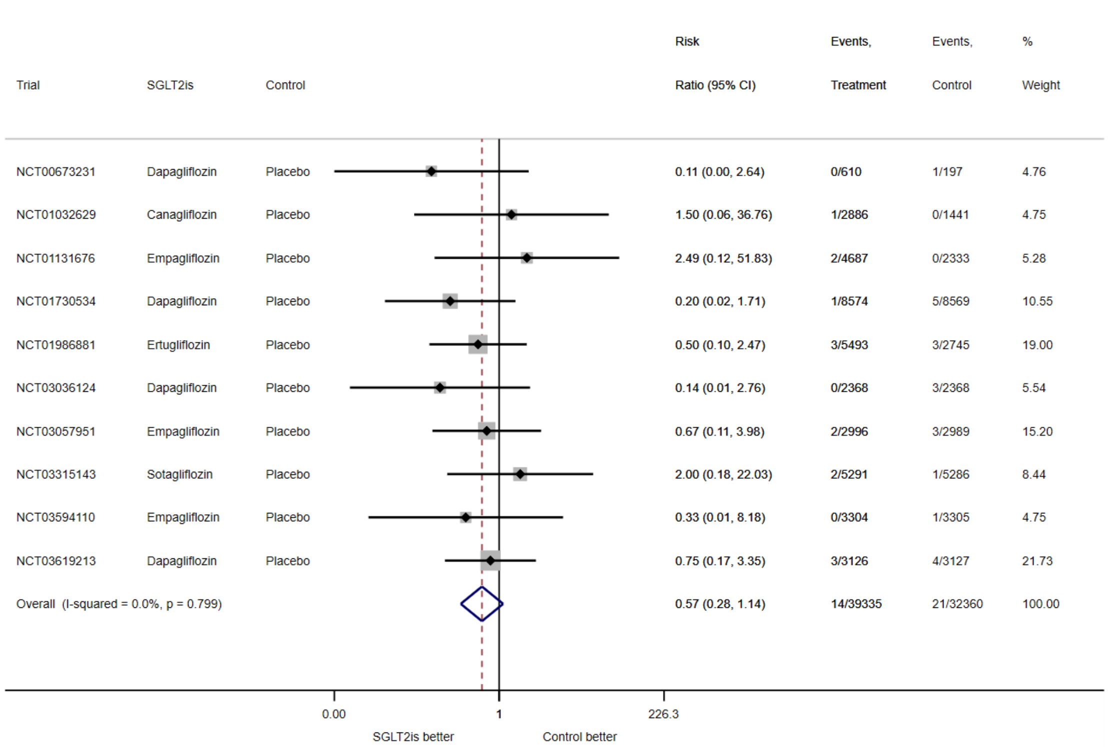

System 22-Respiratory, Thoracic And Mediastinal Disorders-Figure S23 Meta-analysis of SGLT2is and Interstitial lung disease

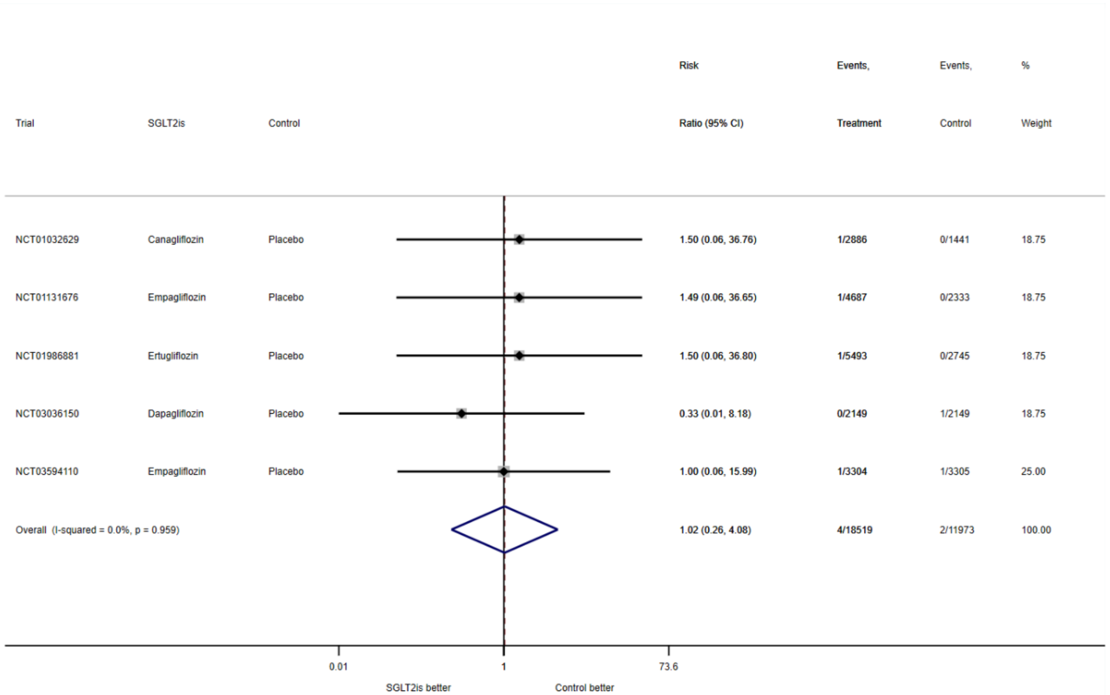

System 22-Respiratory, Thoracic And Mediastinal Disorders-Figure S24 Meta-analysis of SGLT2is and Laryngeal oedema

Trial	SGLT2is	Control	Risk Ratio (95% CI)	Events, Treatment	Events, Control	% Weight
NCT01131676	Empagliflozin	Placebo	0.50 (0.03, 7.95)	1/4687	1/2333	21.05
NCT01730534	Dapagliflozin	Placebo	0.50 (0.05, 5.51)	1/8574	2/8569	28.06
NCT03057977	Empagliflozin	Placebo	5.00 (0.24, 104.08)	2/1863	0/1863	17.54
NCT03521934	Sotagliflozin	Placebo	3.03 (0.12, 74.22)	1/605	0/611	15.80
NCT03619213	Dapagliflozin	Placebo	0.20 (0.01, 4.17)	0/3126	2/3127	17.54
Overall (I-squared = 0.0%, p = 0.538)			0.85 (0.24, 3.02)	5/18855	5/16503	100.00

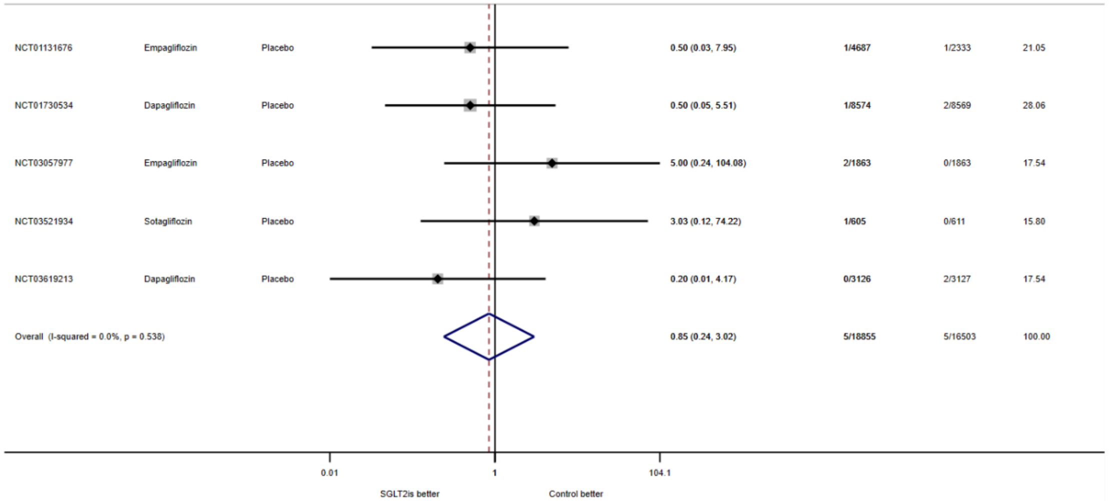

System 22-Respiratory, Thoracic And Mediastinal Disorders-Figure S25 Meta-analysis of SGLT2is and Lung disorder

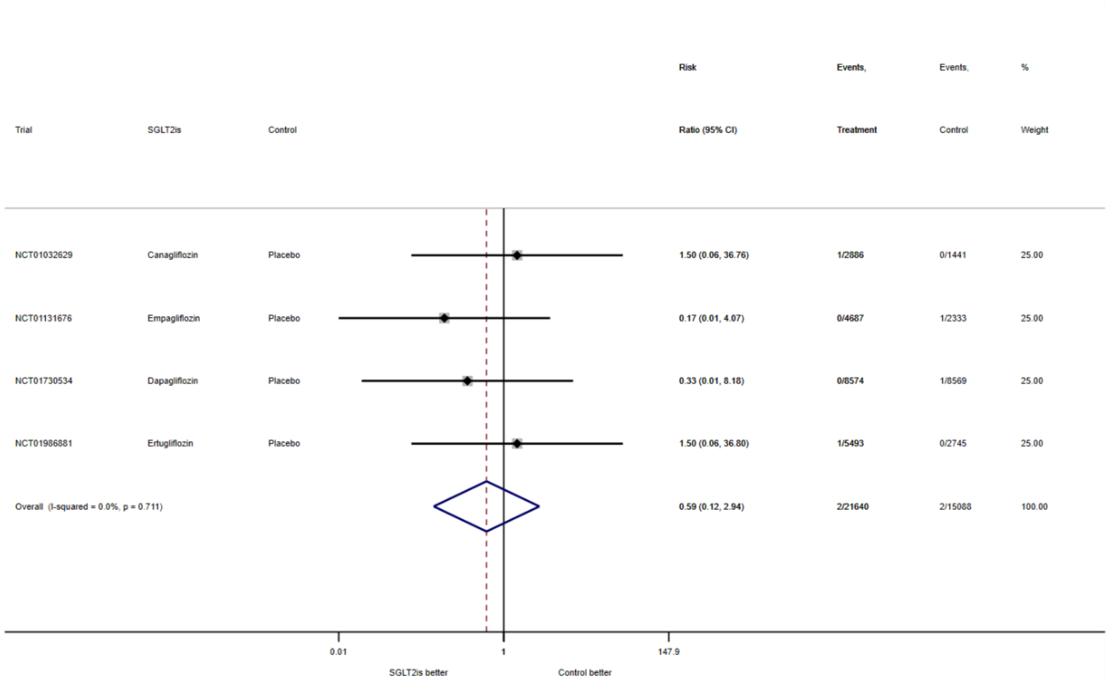

System 22-Respiratory, Thoracic And Mediastinal Disorders-Figure S26 Meta-analysis of SGLT2is and Lung infiltration

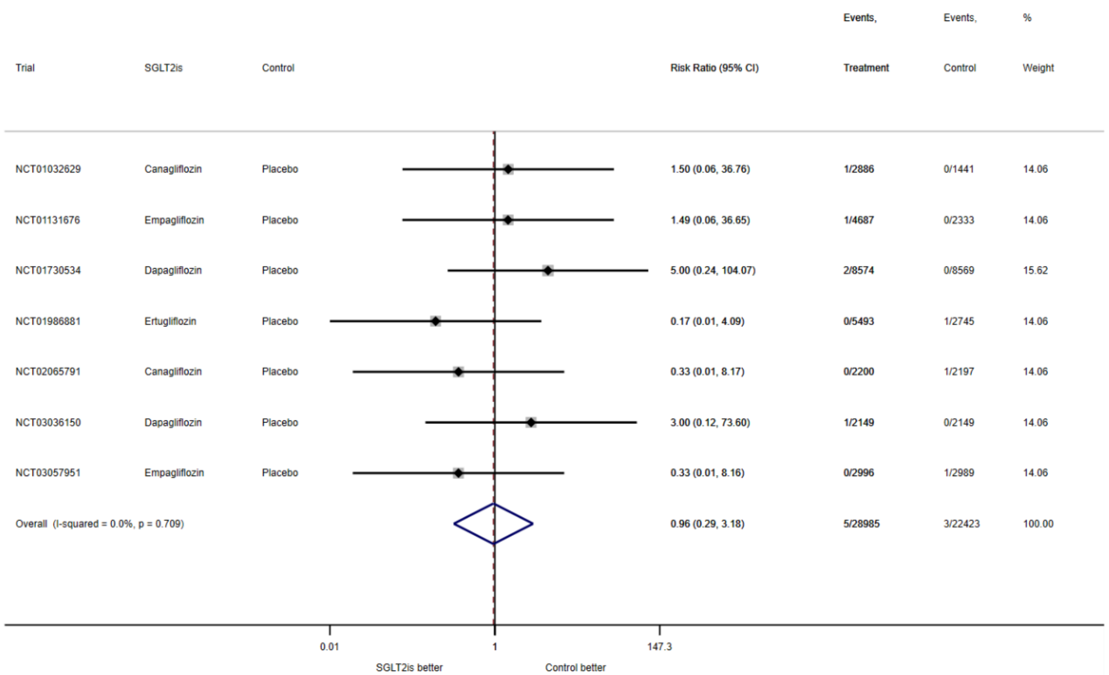

System 22-Respiratory, Thoracic And Mediastinal Disorders-Figure S27 Meta-analysis of SGLT2is and Nasal polyps

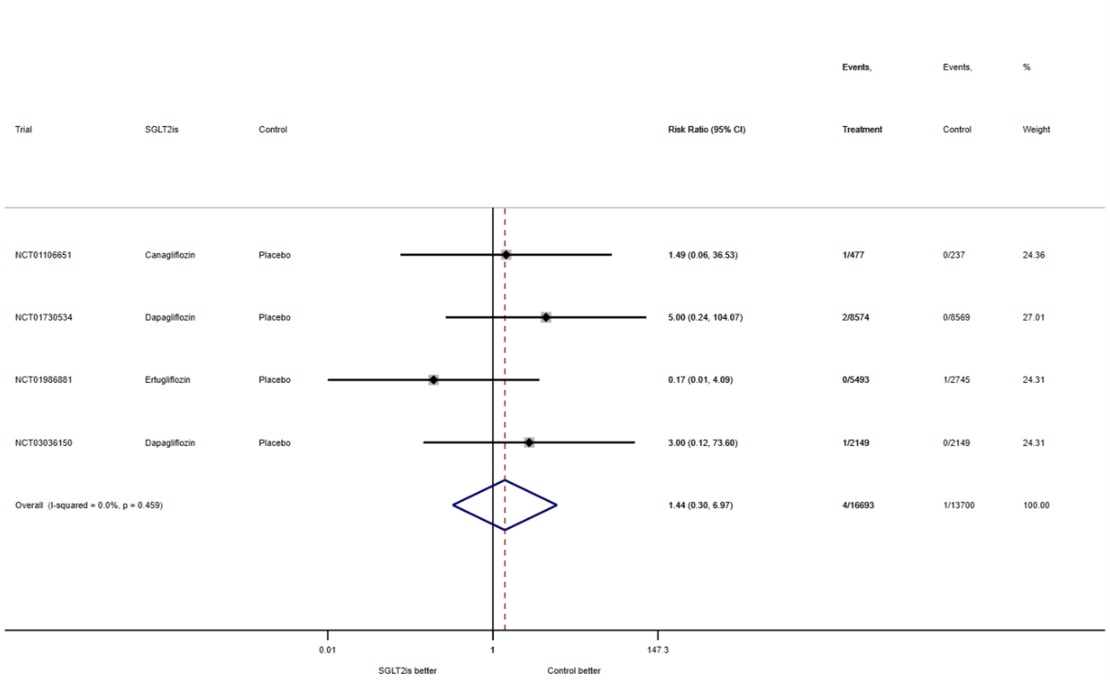

System 22-Respiratory, Thoracic And Mediastinal Disorders-Figure S28 Meta-analysis of SGLT2is and Nasal septum deviation

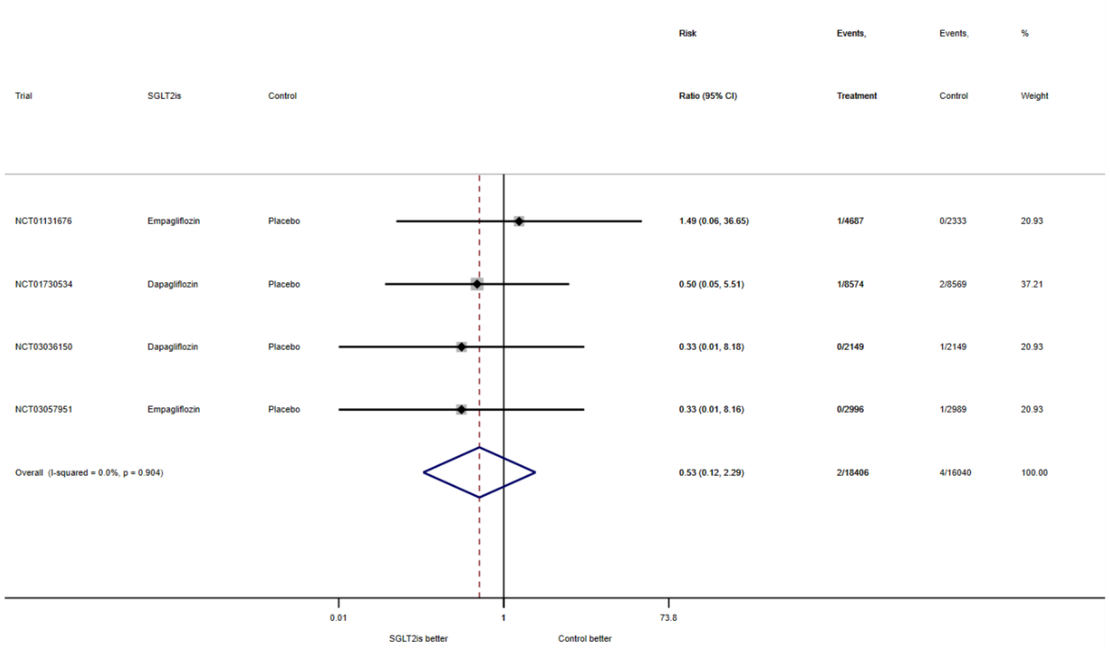

System 22-Respiratory, Thoracic And Mediastinal Disorders-Figure S29 Meta-analysis of SGLT2is and Obstructive airways disorder

Trial	SGLT2is	Control	Risk Ratio (95% CI)	Events, Treatment	Events, Control	% Weight
NCT01032629	Canagliflozin	Placebo	2.50 (0.12, 51.99)	2/2886	0/1441	1.66
NCT01131676	Empagliflozin	Placebo	0.62 (0.17, 2.31)	5/4687	4/2333	8.84
NCT01730534	Dapagliflozin	Placebo	0.46 (0.18, 1.21)	6/8574	13/8569	16.33
NCT01986881	Ertugliflozin	Placebo	1.50 (0.30, 7.42)	6/5493	2/2745	5.97
NCT01989754	Canagliflozin	Placebo	0.40 (0.08, 2.06)	2/2904	5/2903	5.68
NCT02065791	Canagliflozin	Placebo	0.50 (0.13, 1.99)	3/2200	6/2197	7.96
NCT03036124	Dapagliflozin	Placebo	0.67 (0.11, 3.99)	2/2368	3/2368	4.77
NCT03036150	Dapagliflozin	Placebo	0.25 (0.03, 2.23)	1/2149	4/2149	3.18
NCT03057951	Empagliflozin	Placebo	2.66 (0.71, 10.02)	8/2996	3/2989	8.68
NCT03057977	Empagliflozin	Placebo	0.40 (0.08, 2.06)	2/1863	5/1863	5.69
NCT03242252	Sotagliflozin	Placebo	1.48 (0.06, 36.28)	1/527	0/260	1.49
NCT03315143	Sotagliflozin	Placebo	0.25 (0.03, 2.23)	1/5291	4/5286	3.18
NCT03521934	Sotagliflozin	Placebo	7.07 (0.37, 136.57)	3/605	0/611	1.74
NCT03594110	Empagliflozin	Placebo	2.00 (0.50, 7.99)	6/3304	3/3305	7.96
NCT03619213	Dapagliflozin	Placebo	1.14 (0.42, 3.15)	8/3126	7/3127	14.87
NCT04157751	Empagliflozin	Placebo	1.02 (0.06, 16.15)	1/260	1/264	1.99
Overall (I-squared = 0.0%, p = 0.518)			0.82 (0.56, 1.22)	57/49233	60/42410	100.00

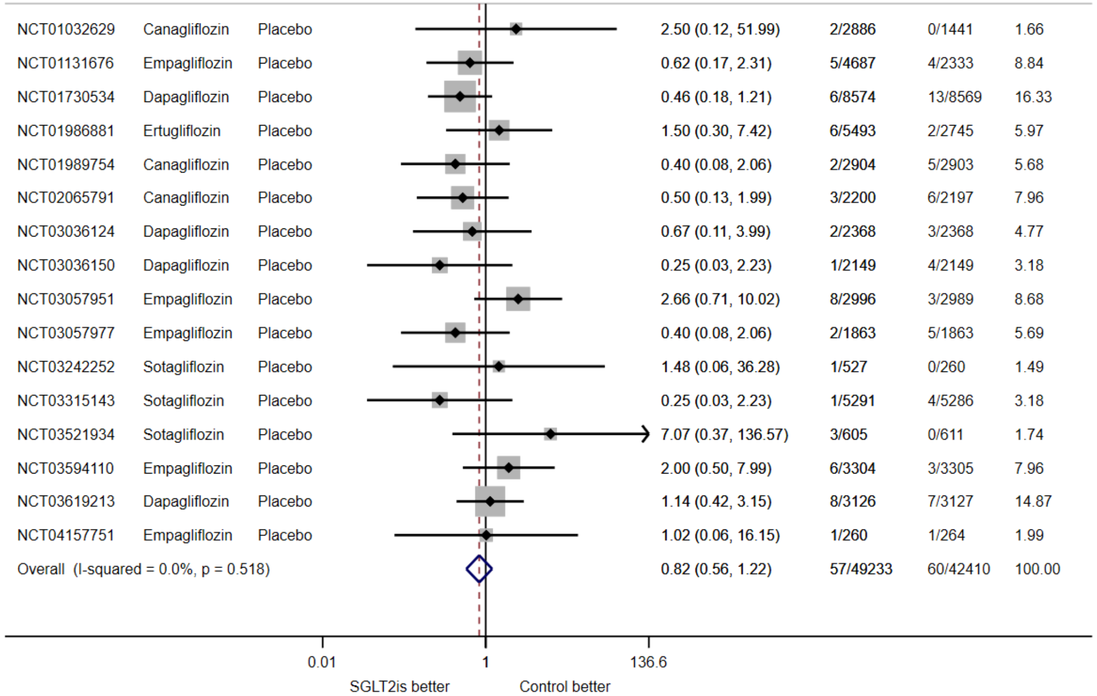

System 22-Respiratory, Thoracic And Mediastinal Disorders-Figure S30 Meta-analysis of SGLT2is and Pleural effusion

Trial	SGLT2is	Control	Risk Ratio (95% CI)	Events, Treatment	Events, Control	% Weight
NCT01131676	Empagliflozin	Placebo	1.49 (0.06, 36.65)	1/4687	0/2333	6.99
NCT01730534	Dapagliflozin	Placebo	0.67 (0.11, 3.99)	2/8574	3/8569	22.36
NCT01986881	Ertugliflozin	Placebo	0.50 (0.03, 7.99)	1/5493	1/2745	9.32
NCT03036124	Dapagliflozin	Placebo	0.50 (0.05, 5.51)	1/2368	2/2368	12.43
NCT03036150	Dapagliflozin	Placebo	3.00 (0.12, 73.60)	1/2149	0/2149	6.99
NCT03057951	Empagliflozin	Placebo	0.33 (0.01, 8.16)	0/2996	1/2989	6.99
NCT03315143	Sotagliflozin	Placebo	1.00 (0.14, 7.09)	2/5291	2/5286	18.63
NCT03594110	Empagliflozin	Placebo	3.00 (0.12, 73.64)	1/3304	0/3305	6.99
NCT03619213	Dapagliflozin	Placebo	1.00 (0.06, 15.99)	1/3126	1/3127	9.32
Overall (I-squared = 0.0%, p = 0.978)			0.87 (0.37, 2.03)	10/37988	10/32871	100.00

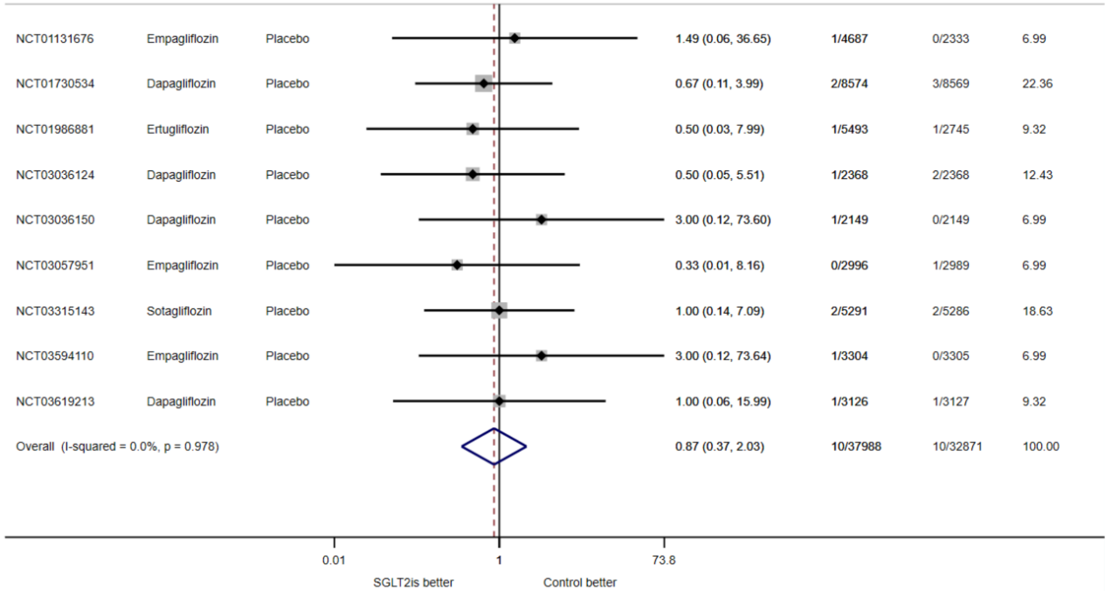

System 22-Respiratory, Thoracic And Mediastinal Disorders-Figure S31 Meta-analysis of SGLT2is and Pleurisy

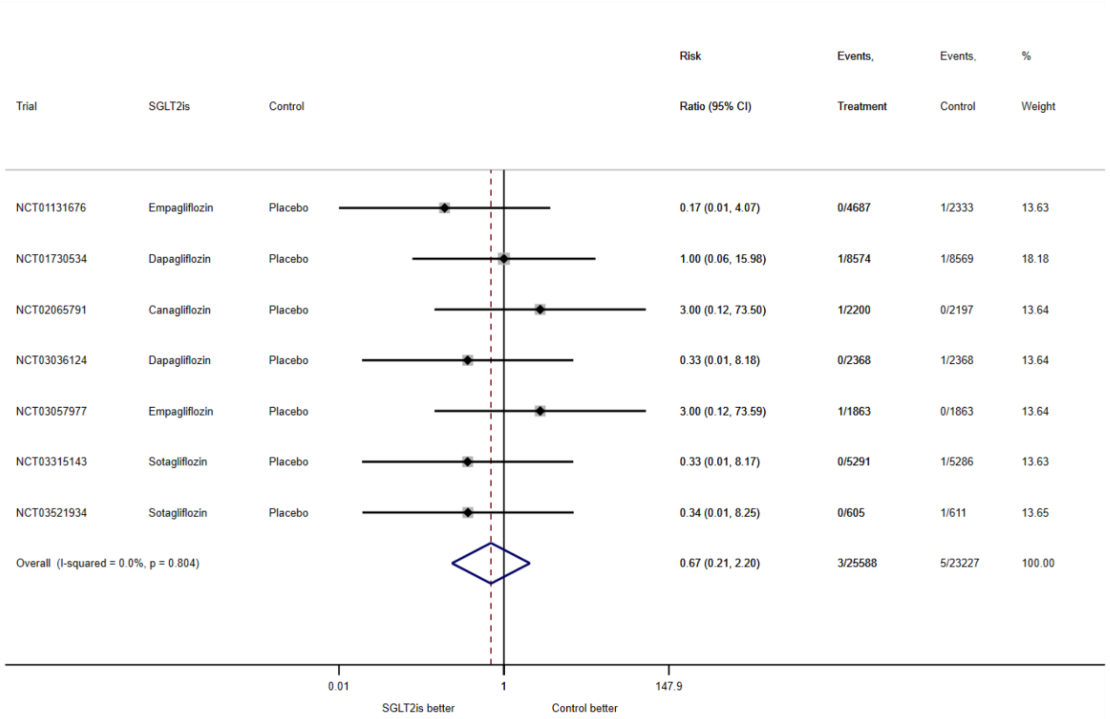

System 22-Respiratory, Thoracic And Mediastinal Disorders-Figure S32 Meta-analysis of SGLT2is and Pleuritic pain

Trial	SGLT2is	Control	Risk Ratio (95% CI)	Events, Treatment	Events, Control	% Weight
NCT00528879	Dapagliflozin	Placebo	0.11 (0.00, 2.74)	0/409	1/137	2.13
NCT01032629	Canagliflozin	Placebo	1.50 (0.06, 36.76)	1/2886	0/1441	2.13
NCT01131676	Empagliflozin	Placebo	0.25 (0.02, 2.74)	1/4687	2/2333	3.78
NCT01730534	Dapagliflozin	Placebo	1.80 (0.83, 3.89)	18/8574	10/8569	36.48
NCT01986881	Ertugliflozin	Placebo	0.33 (0.06, 1.99)	2/5493	3/2745	6.80
NCT01989754	Canagliflozin	Placebo	0.20 (0.01, 4.16)	0/2904	2/2903	2.36
NCT02065791	Canagliflozin	Placebo	0.20 (0.01, 4.16)	0/2200	2/2197	2.36
NCT03036124	Dapagliflozin	Placebo	0.75 (0.17, 3.35)	3/2368	4/2368	9.73
NCT03036150	Dapagliflozin	Placebo	1.00 (0.14, 7.09)	2/2149	2/2149	5.67
NCT03057951	Empagliflozin	Placebo	0.60 (0.14, 2.50)	3/2996	5/2989	10.64
NCT03057977	Empagliflozin	Placebo	0.67 (0.11, 3.99)	2/1863	3/1863	6.81
NCT03315143	Sotagliflozin	Placebo	3.00 (0.31, 28.80)	3/5291	1/5286	4.25
NCT03521934	Sotagliflozin	Placebo	3.03 (0.12, 74.22)	1/605	0/611	2.13
NCT03594110	Empagliflozin	Placebo	0.20 (0.02, 1.71)	1/3304	5/3305	4.72
Overall (I-squared = 0.0%, p = 0.449)			0.87 (0.54, 1.38)	37/45729	40/38896	100.00

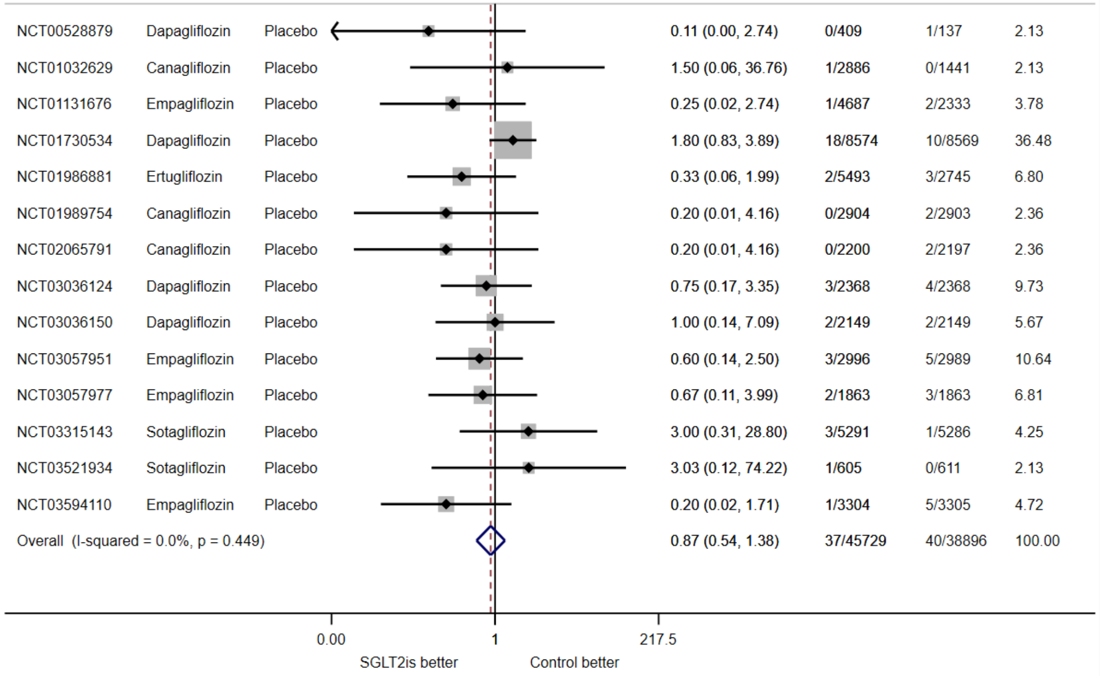

System 22-Respiratory, Thoracic And Mediastinal Disorders-Figure S33 Meta-analysis of SGLT2is and Pneumonia aspiration

Trial	SGLT2is	Control	Risk Ratio (95% CI)	Events, Treatment	Events, Control	% Weight
NCT01032629	Canagliflozin	Placebo	1.50 (0.06, 36.76)	1/2886	0/1441	8.39
NCT01131676	Empagliflozin	Placebo	0.17 (0.01, 4.07)	0/4687	1/2333	8.39
NCT01730534	Dapagliflozin	Placebo	0.40 (0.08, 2.06)	2/8574	5/8569	31.96
NCT01986881	Ertugliflozin	Placebo	0.17 (0.01, 4.09)	0/5493	1/2745	8.39
NCT03036124	Dapagliflozin	Placebo	3.00 (0.12, 73.61)	1/2368	0/2368	8.39
NCT03057951	Empagliflozin	Placebo	0.20 (0.01, 4.15)	0/2996	2/2989	9.32
NCT03057977	Empagliflozin	Placebo	0.33 (0.01, 8.18)	0/1863	1/1863	8.39
NCT03315143	Sotagliflozin	Placebo	3.00 (0.12, 73.56)	1/5291	0/5286	8.39
NCT03594110	Empagliflozin	Placebo	3.00 (0.12, 73.64)	1/3304	0/3305	8.39
Overall (I-squared = 0.0%, p = 0.721)			0.59 (0.23, 1.49)	6/37462	10/30899	100.00

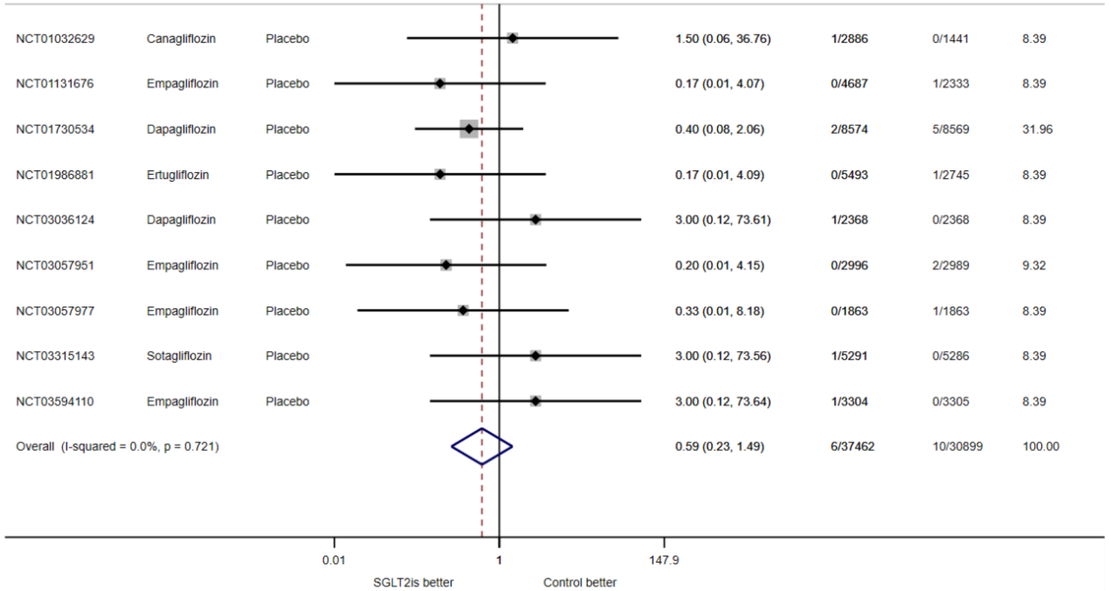

System 22-Respiratory, Thoracic And Mediastinal Disorders-Figure S34 Meta-analysis of SGLT2is and Pneumonitis

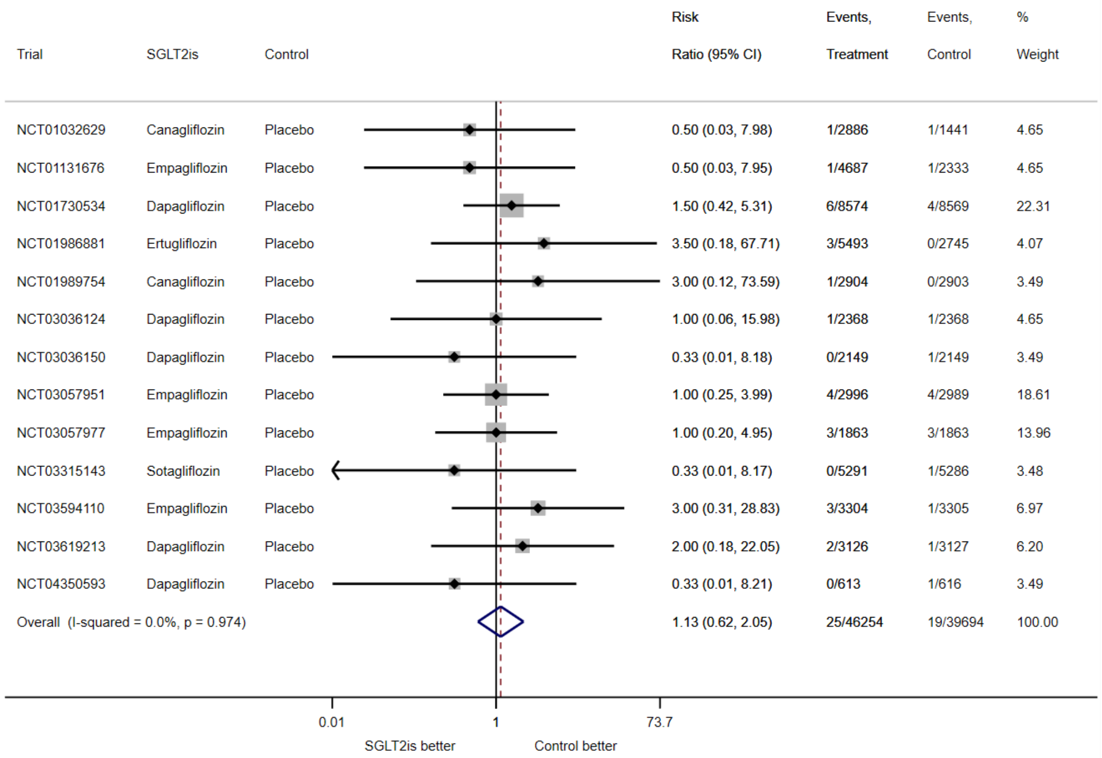

System 22-Respiratory, Thoracic And Mediastinal Disorders-Figure S35 Meta-analysis of SGLT2is and Pneumothorax

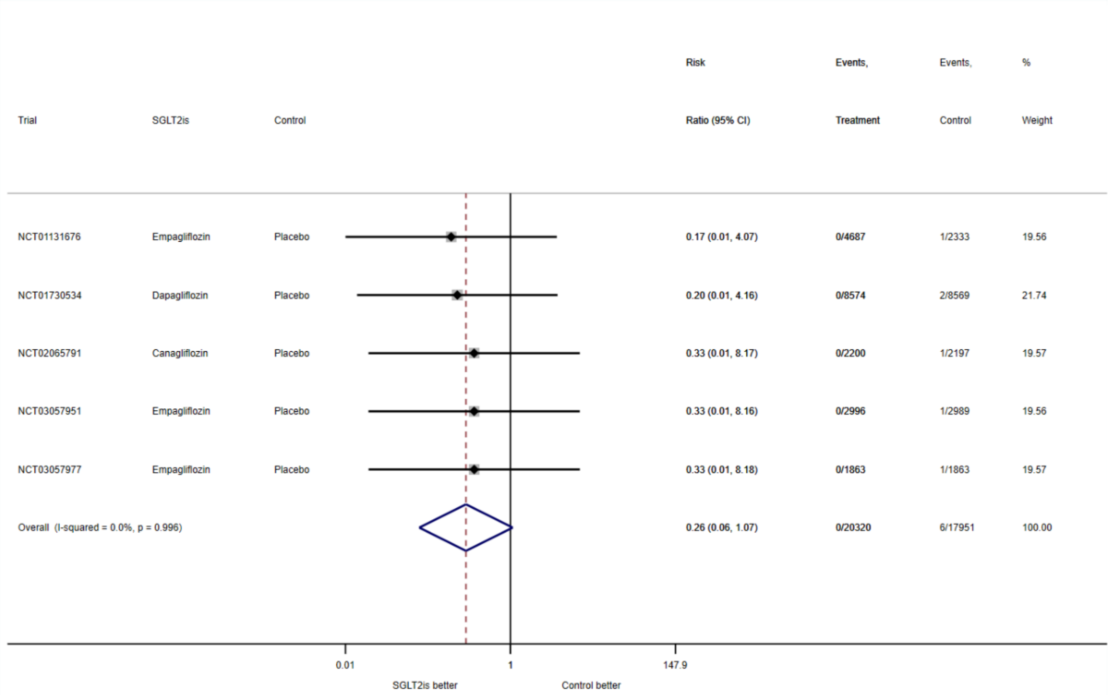

System 22-Respiratory, Thoracic And Mediastinal Disorders-Figure S36 Meta-analysis of SGLT2is and Pulmonary arterial hypertension

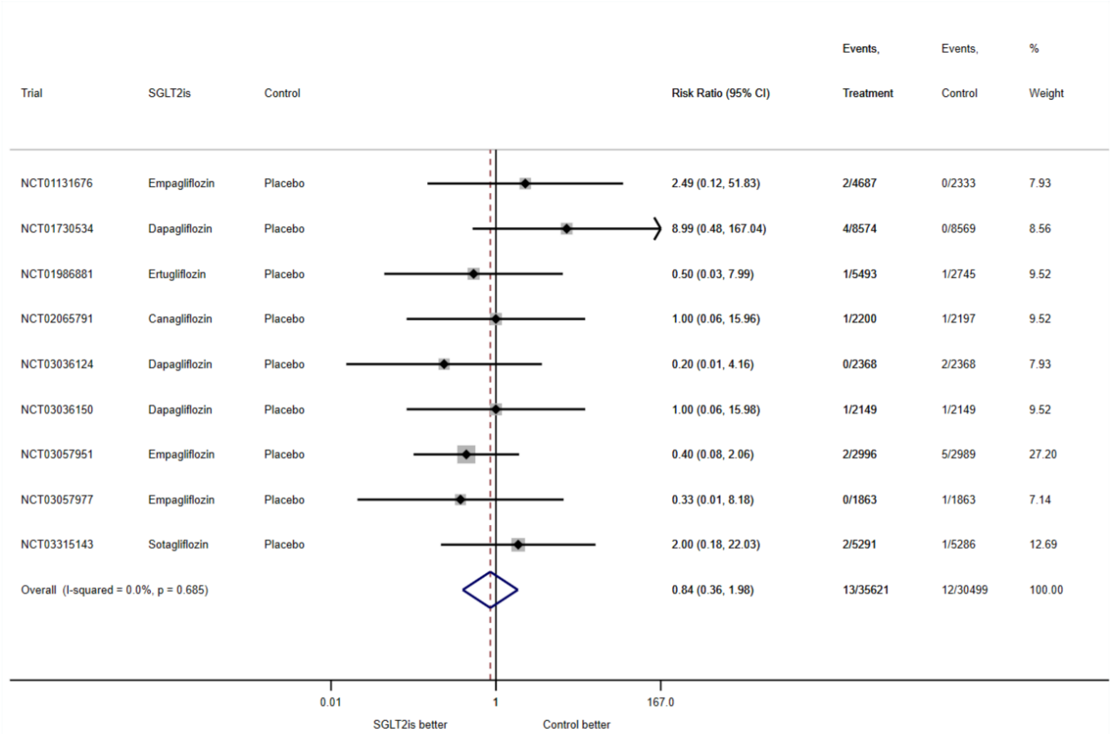

System 22-Respiratory, Thoracic And Mediastinal Disorders-Figure S37 Meta-analysis of SGLT2is and Pulmonary congestion

Trial	SGLT2is	Control	Risk Ratio (95% CI)	Events, Treatment	Events, Control	% Weight
NCT00673231	Dapagliflozin	Placebo	0.11 (0.00, 2.64)	0/610	1/197	0.62
NCT01032629	Canagliflozin	Placebo	1.00 (0.40, 2.47)	14/2886	7/1441	7.76
NCT01042977	Dapagliflozin	Placebo	1.00 (0.06, 15.94)	1/482	1/482	0.83
NCT01106651	Canagliflozin	Placebo	0.17 (0.01, 4.06)	0/477	1/237	0.62
NCT01131676	Empagliflozin	Placebo	0.75 (0.21, 2.64)	6/4687	4/2333	3.98
NCT01164501	Empagliflozin	Placebo	0.25 (0.01, 6.21)	0/419	1/319	0.62
NCT01730534	Dapagliflozin	Placebo	0.96 (0.56, 1.65)	26/8574	27/8569	21.99
NCT01986881	Ertugliflozin	Placebo	1.25 (0.49, 3.22)	15/5493	6/2745	7.11
NCT01989754	Canagliflozin	Placebo	1.20 (0.37, 3.93)	6/2904	5/2903	4.52
NCT02033889	Ertugliflozin	Placebo/Glimepiride	1.53 (0.06, 37.28)	1/412	0/209	0.62
NCT02065791	Canagliflozin	Placebo	2.33 (0.60, 9.00)	7/2200	3/2197	3.48
NCT03036124	Dapagliflozin	Placebo	0.50 (0.20, 1.24)	7/2368	14/2368	7.76
NCT03036150	Dapagliflozin	Placebo	1.14 (0.42, 3.15)	8/2149	7/2149	6.20
NCT03057951	Empagliflozin	Placebo	0.45 (0.16, 1.30)	5/2996	11/2989	5.70
NCT03057977	Empagliflozin	Placebo	1.25 (0.34, 4.65)	5/1863	4/1863	3.69
NCT03242252	Sotagliflozin	Placebo	1.48 (0.06, 36.28)	1/527	0/260	0.62
NCT03315143	Sotagliflozin	Placebo	0.54 (0.21, 1.35)	7/5291	13/5286	7.54
NCT03521934	Sotagliflozin	Placebo	0.08 (0.00, 1.38)	0/605	6/611	0.77
NCT03594110	Empagliflozin	Placebo	0.50 (0.15, 1.66)	4/3304	8/3305	4.42
NCT03619213	Dapagliflozin	Placebo	1.22 (0.51, 2.95)	11/3126	9/3127	8.22
NCT04157751	Empagliflozin	Placebo	0.34 (0.04, 3.23)	1/260	3/264	1.25
NCT04350593	Dapagliflozin	Placebo	1.00 (0.14, 7.11)	2/613	2/616	1.66
Overall (I-squared = 0.0%, p = 0.775)			0.84 (0.65, 1.08)	127/52246	133/44470	100.00

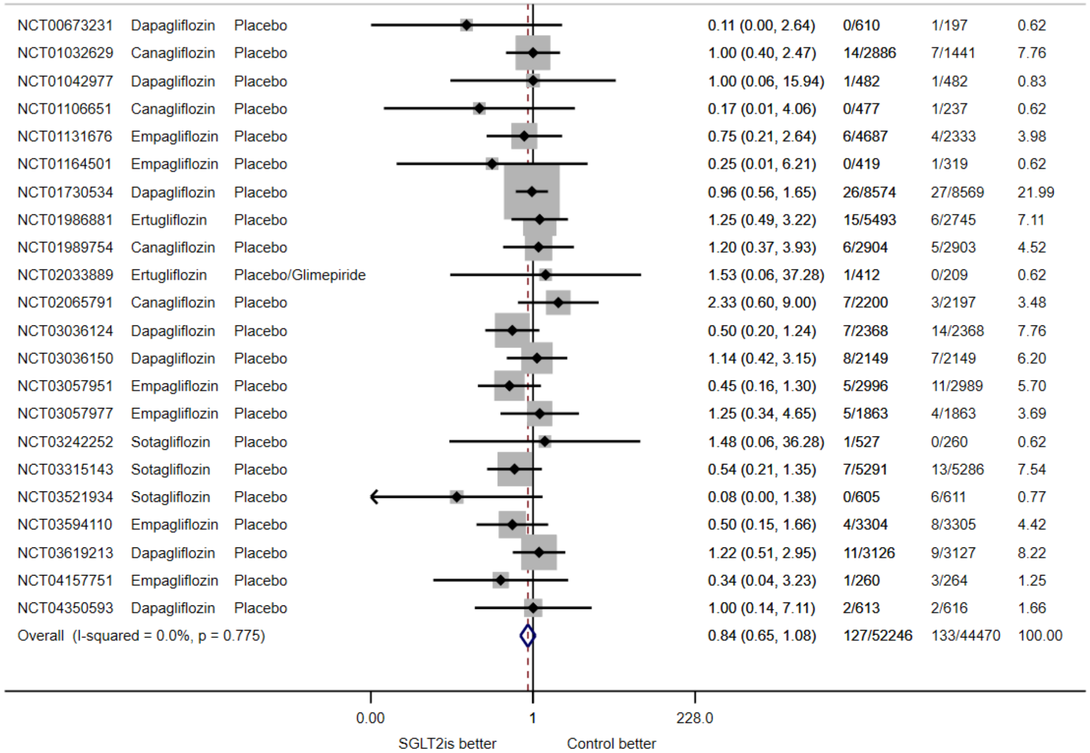

System 22-Respiratory, Thoracic And Mediastinal Disorders-Figure S38 Meta-analysis of SGLT2is and Pulmonary embolism

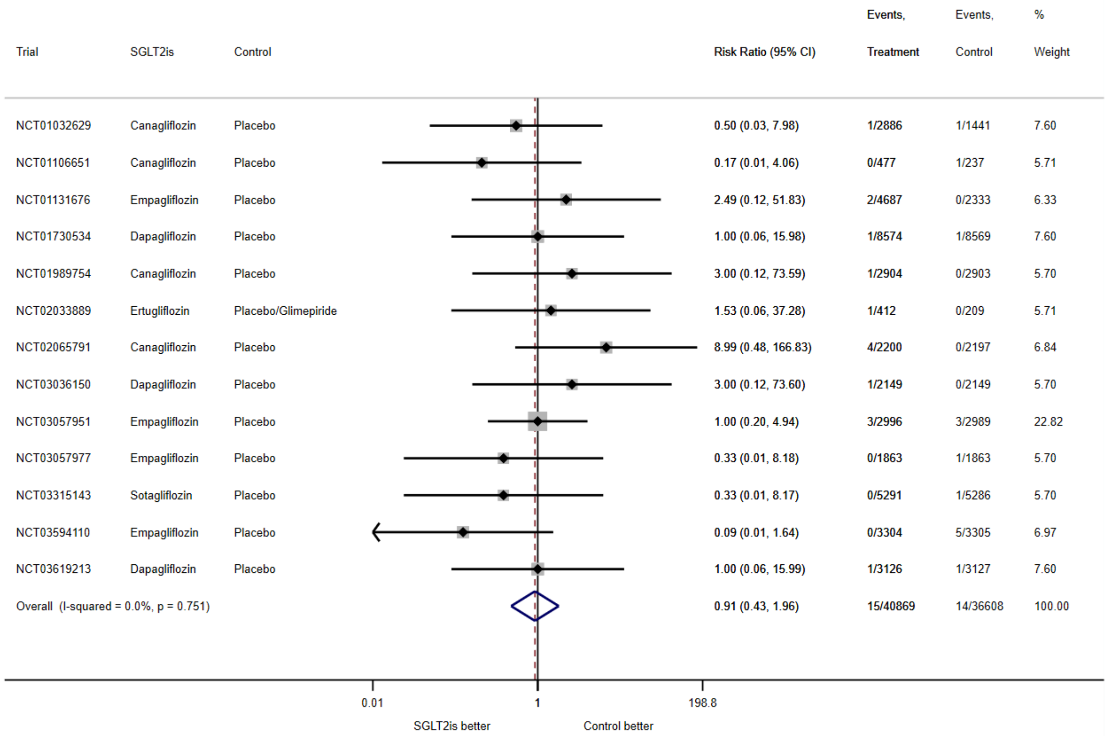

System 22-Respiratory, Thoracic And Mediastinal Disorders-Figure S39 Meta-analysis of SGLT2is and Pulmonary fibrosis

Trial	SGLT2is	Control	Risk Ratio (95% CI)	Events, Treatment	Events, Control	% Weight
NCT01032629	Canagliflozin	Placebo	1.50 (0.06, 36.76)	1/2886	0/1441	2.90
NCT01131676	Empagliflozin	Placebo	0.30 (0.07, 1.25)	3/4687	5/2333	14.52
NCT01730534	Dapagliflozin	Placebo	0.25 (0.03, 2.23)	1/8574	4/8569	6.19
NCT01986881	Ertugliflozin	Placebo	4.50 (0.24, 83.52)	4/5493	0/2745	3.48
NCT03036124	Dapagliflozin	Placebo	3.00 (0.12, 73.61)	1/2368	0/2368	2.90
NCT03057951	Empagliflozin	Placebo	0.61 (0.25, 1.48)	8/2996	13/2989	38.44
NCT03057977	Empagliflozin	Placebo	0.67 (0.11, 3.99)	2/1863	3/1863	9.30
NCT03315143	Sotagliflozin	Placebo	0.20 (0.01, 4.16)	0/5291	2/5286	3.22
NCT03594110	Empagliflozin	Placebo	0.50 (0.09, 2.73)	2/3304	4/3305	10.32
NCT03619213	Dapagliflozin	Placebo	3.00 (0.31, 28.83)	3/3126	1/3127	5.81
NCT04157751	Empagliflozin	Placebo	0.34 (0.01, 8.27)	0/260	1/264	2.91
Overall (I-squared = 0.0%, p = 0.701)			0.62 (0.36, 1.07)	25/40848	33/34290	100.00

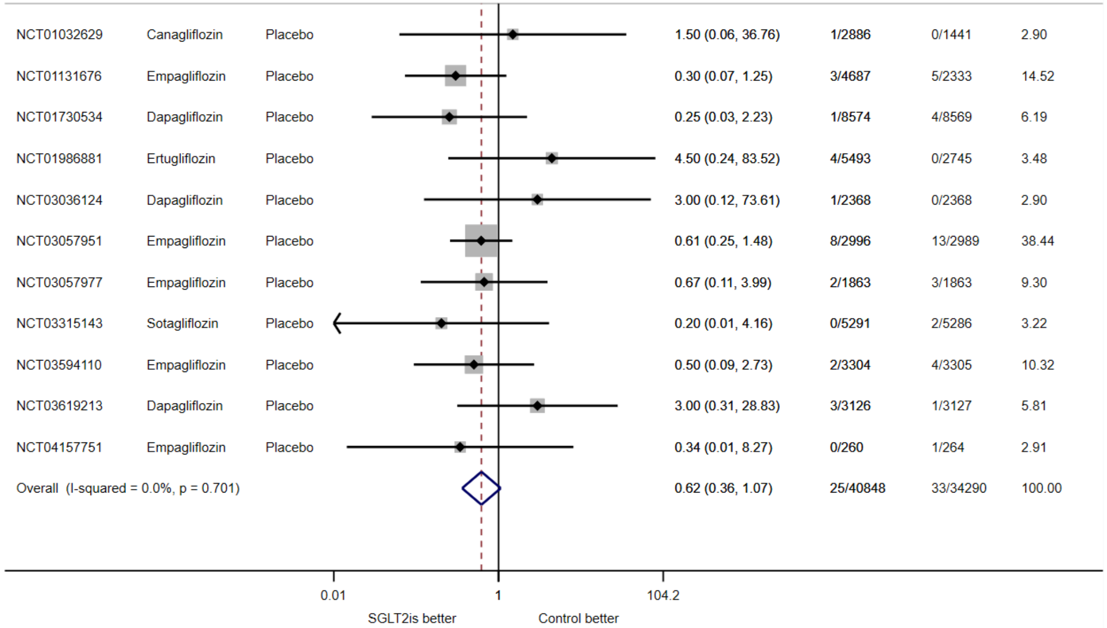

System 22-Respiratory, Thoracic And Mediastinal Disorders-Figure S40 Meta-analysis of SGLT2is and Pulmonary hypertension

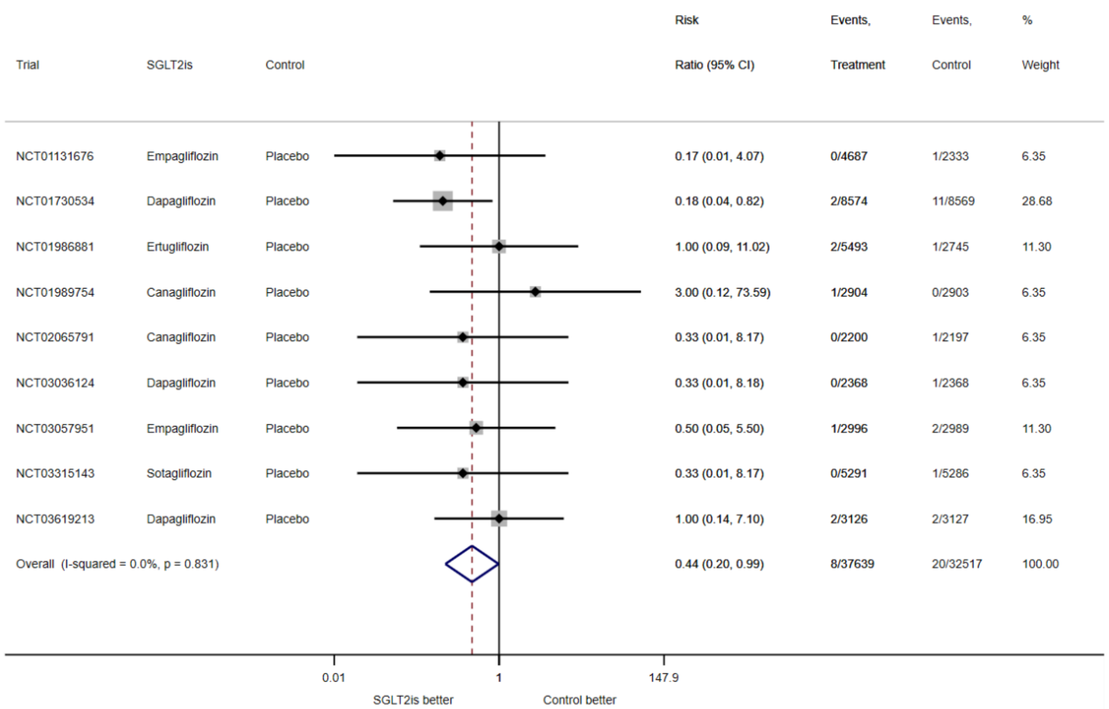

System 22-Respiratory, Thoracic And Mediastinal Disorders-Figure S41 Meta-analysis of SGLT2is and Pulmonary mass

Trial	SGLT2is	Control	Risk Ratio (95% CI)	Events, Treatment	Events, Control	% Weight
NCT01032629	Canagliflozin	Placebo	0.50 (0.10, 2.47)	3/2886	3/1441	6.48
NCT01131676	Empagliflozin	Placebo	0.30 (0.07, 1.25)	3/4687	5/2333	8.10
NCT01164501	Empagliflozin	Placebo	0.15 (0.01, 3.16)	0/419	2/319	1.80
NCT01730534	Dapagliflozin	Placebo	0.27 (0.09, 0.80)	4/8574	15/8569	13.64
NCT01986881	Ertugliflozin	Placebo	0.33 (0.09, 1.18)	4/5493	6/2745	10.37
NCT01989754	Canagliflozin	Placebo	0.08 (0.00, 1.36)	0/2904	6/2903	2.00
NCT02065791	Canagliflozin	Placebo	0.43 (0.11, 1.65)	3/2200	7/2197	9.08
NCT03036124	Dapagliflozin	Placebo	0.60 (0.14, 2.51)	3/2368	5/2368	8.11
NCT03036150	Dapagliflozin	Placebo	0.63 (0.20, 1.91)	5/2149	8/2149	13.32
NCT03057951	Empagliflozin	Placebo	0.17 (0.02, 1.38)	1/2996	6/2989	3.70
NCT03057977	Empagliflozin	Placebo	1.00 (0.06, 15.98)	1/1863	1/1863	2.16
NCT03315143	Sotagliflozin	Placebo	0.06 (0.00, 1.02)	0/5291	8/5286	2.04
NCT03521934	Sotagliflozin	Placebo	1.01 (0.14, 7.15)	2/605	2/611	4.33
NCT03594110	Empagliflozin	Placebo	1.00 (0.06, 15.99)	1/3304	1/3305	2.16
NCT03619213	Dapagliflozin	Placebo	1.00 (0.29, 3.45)	5/3126	5/3127	10.81
NCT04157751	Empagliflozin	Placebo	0.15 (0.01, 2.79)	0/260	3/264	1.89
Overall (I-squared = 0.0%, p = 0.819)			0.42 (0.28, 0.63)	35/49125	83/42469	100.00

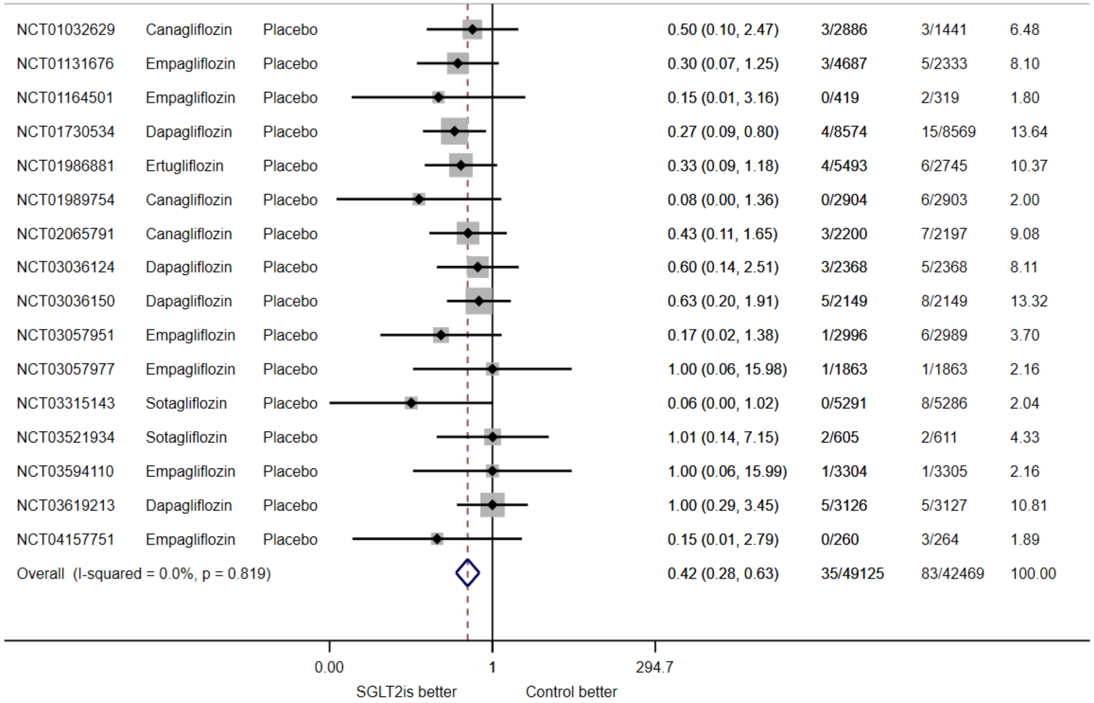

System 22-Respiratory, Thoracic And Mediastinal Disorders-Figure S42 Meta-analysis of SGLT2is and Pulmonary oedema

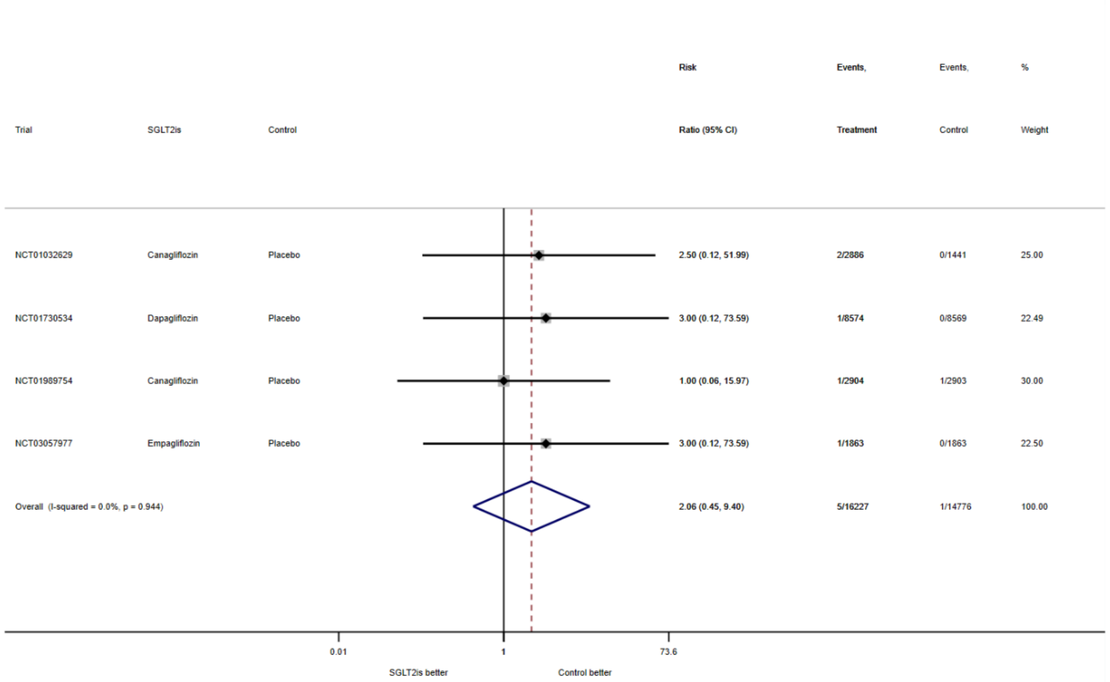

System 22-Respiratory, Thoracic And Mediastinal Disorders-Figure S43 Meta-analysis of SGLT2is and Respiratory acidosis

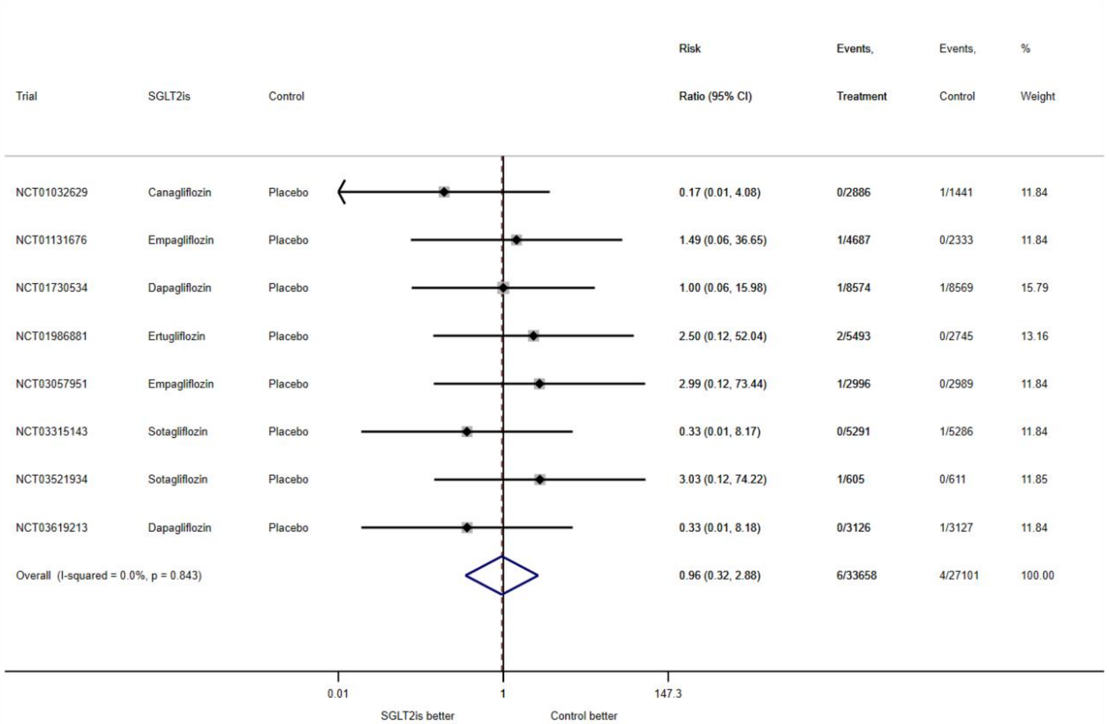

System 22-Respiratory, Thoracic And Mediastinal Disorders-Figure S44 Meta-analysis of SGLT2is and Respiratory arrest

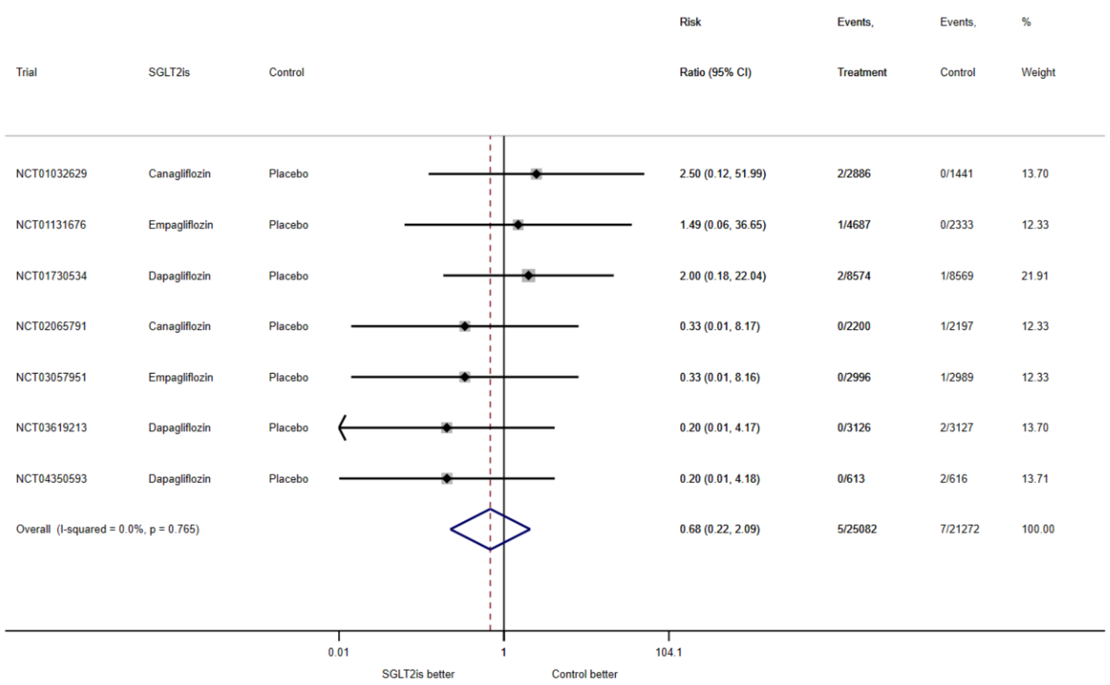

System 22-Respiratory, Thoracic And Mediastinal Disorders-Figure S45 Meta-analysis of SGLT2is and Respiratory distress

Trial	SGLT2is	Control	Risk Ratio (95% CI)	Events, Treatment	Events, Control	% Weight
NCT01031680	Dapagliflozin	Placebo	3.01 (0.12, 73.77)	1/460	0/462	0.58
NCT01032629	Canagliflozin	Placebo	0.50 (0.16, 1.55)	6/2886	6/1441	4.68
NCT01106651	Canagliflozin	Placebo	1.49 (0.06, 36.53)	1/477	0/237	0.58
NCT01106677	Canagliflozin	Sitagliptin	0.17 (0.01, 4.07)	0/735	1/366	0.58
NCT01131676	Empagliflozin	Placebo	0.33 (0.12, 0.93)	6/4687	9/2333	5.61
NCT01730534	Dapagliflozin	Placebo	1.21 (0.70, 2.07)	29/8574	24/8569	20.47
NCT01986881	Ertugliflozin	Placebo	0.68 (0.31, 1.48)	15/5493	11/2745	9.90
NCT01989754	Canagliflozin	Placebo	0.33 (0.03, 3.20)	1/2904	3/2903	1.17
NCT02065791	Canagliflozin	Placebo	1.00 (0.42, 2.39)	10/2200	10/2197	7.81
NCT03036124	Dapagliflozin	Placebo	0.43 (0.11, 1.66)	3/2368	7/2368	3.27
NCT03036150	Dapagliflozin	Placebo	0.33 (0.07, 1.65)	2/2149	6/2149	2.33
NCT03057951	Empagliflozin	Placebo	0.93 (0.45, 1.93)	14/2996	15/2989	11.31
NCT03057977	Empagliflozin	Placebo	0.40 (0.08, 2.06)	2/1863	5/1863	2.22
NCT03242252	Sotagliflozin	Placebo	0.16 (0.01, 4.03)	0/527	1/260	0.58
NCT03315143	Sotagliflozin	Placebo	1.00 (0.38, 2.66)	8/5291	8/5286	6.23
NCT03521934	Sotagliflozin	Placebo	1.01 (0.20, 4.98)	3/605	3/611	2.34
NCT03594110	Empagliflozin	Placebo	2.00 (0.18, 22.05)	2/3304	1/3305	1.04
NCT03619213	Dapagliflozin	Placebo	0.59 (0.27, 1.28)	10/3126	17/3127	9.82
NCT04157751	Empagliflozin	Placebo	1.02 (0.06, 16.15)	1/260	1/264	0.78
NCT04350593	Dapagliflozin	Placebo	1.00 (0.44, 2.30)	11/613	11/616	8.70
Overall (I-squared = 0.0%, p = 0.789)			0.78 (0.61, 1.00)	125/51518	139/44091	100.00

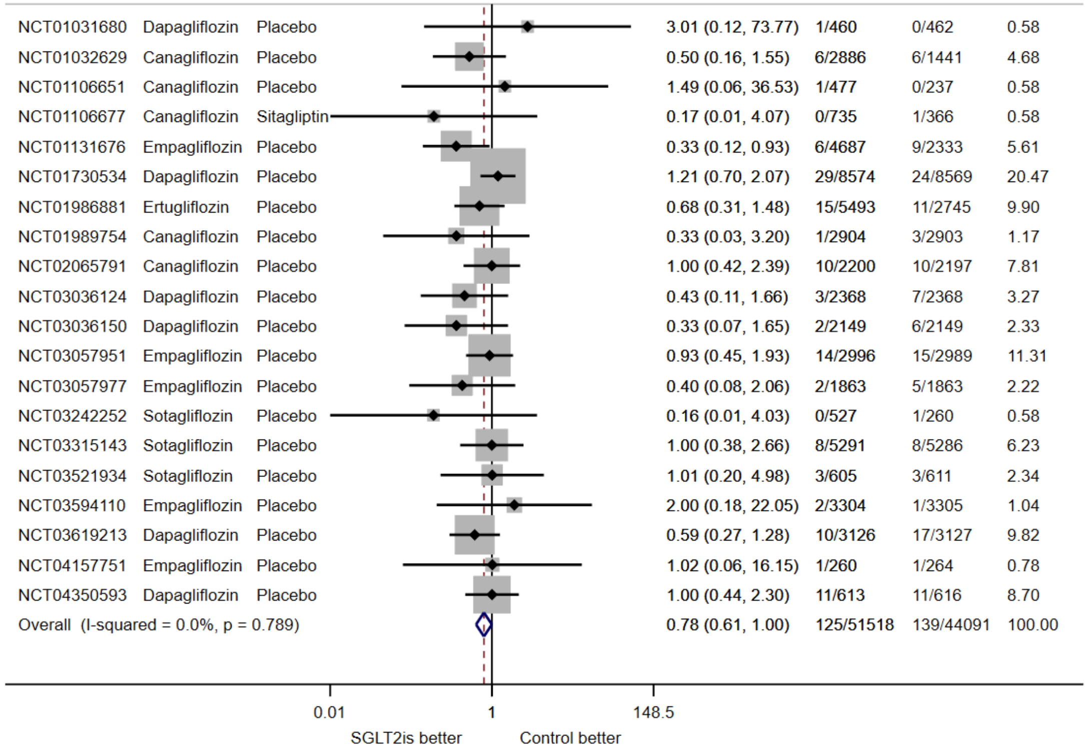

System 22-Respiratory, Thoracic And Mediastinal Disorders-Figure S46 Meta-analysis of SGLT2is and Respiratory failure

Trial	SGLT2is	Control	Risk Ratio (95% CI)	Events, Treatment	Events, Control	% Weight
NCT01032629	Canagliflozin	Placebo	0.12 (0.01, 1.12)	1/2886	4/1441	10.53
NCT01131676	Empagliflozin	Placebo	0.12 (0.01, 1.11)	1/4687	4/2333	10.53
NCT01730534	Dapagliflozin	Placebo	2.00 (0.18, 22.04)	2/8574	1/8569	8.77
NCT01986881	Ertugliflozin	Placebo	0.50 (0.07, 3.55)	2/5493	2/2745	13.16
NCT01989754	Canagliflozin	Placebo	1.00 (0.06, 15.97)	1/2904	1/2903	6.58
NCT02033889	Ertugliflozin	Placebo/Glimepiride	0.17 (0.01, 4.14)	0/412	1/209	4.94
NCT02065791	Canagliflozin	Placebo	0.33 (0.01, 8.17)	0/2200	1/2197	4.93
NCT03036150	Dapagliflozin	Placebo	0.33 (0.01, 8.18)	0/2149	1/2149	4.93
NCT03057951	Empagliflozin	Placebo	2.99 (0.31, 28.76)	3/2996	1/2989	9.87
NCT03057977	Empagliflozin	Placebo	0.20 (0.01, 4.16)	0/1863	2/1863	5.48
NCT03315143	Sotagliflozin	Placebo	2.00 (0.18, 22.03)	2/5291	1/5286	8.77
NCT03594110	Empagliflozin	Placebo	1.00 (0.06, 15.99)	1/3304	1/3305	6.58
NCT03619213	Dapagliflozin	Placebo	3.00 (0.12, 73.64)	1/3126	0/3127	4.93
Overall (I-squared = 0.0%, p = 0.577)			0.59 (0.29, 1.20)	14/45885	20/39116	100.00

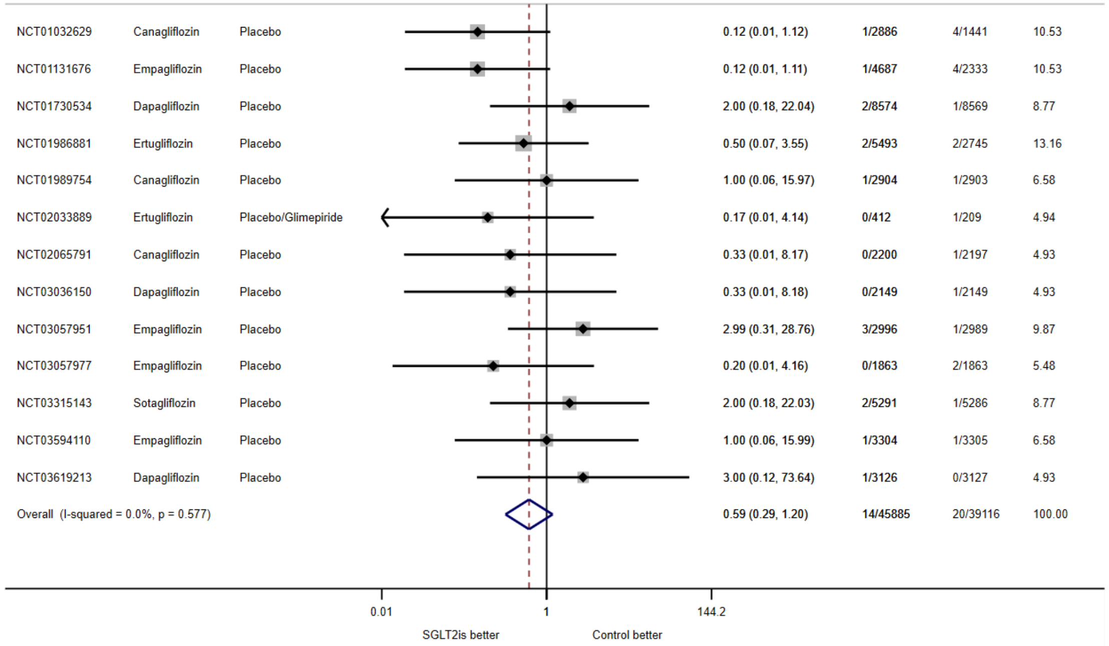

System 22-Respiratory, Thoracic And Mediastinal Disorders-Figure S47 Meta-analysis of SGLT2is and Sleep apnoea syndrome

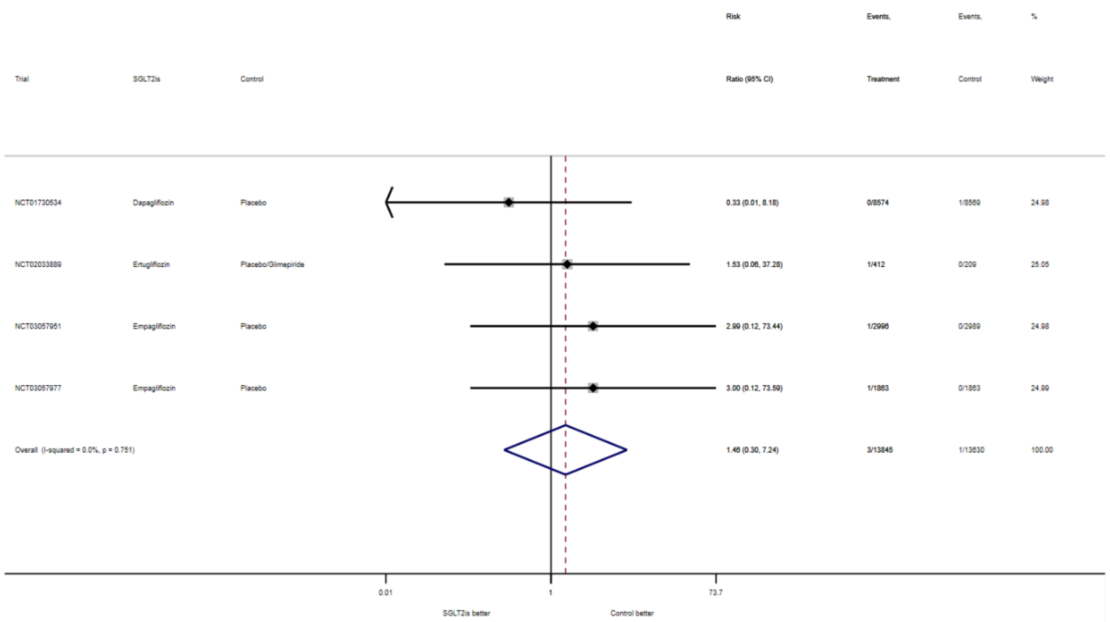

System 23-Skin And Subcutaneous Tissue Disorders-Figure S1 Meta-analysis of SGLT2is and Actinic keratosis

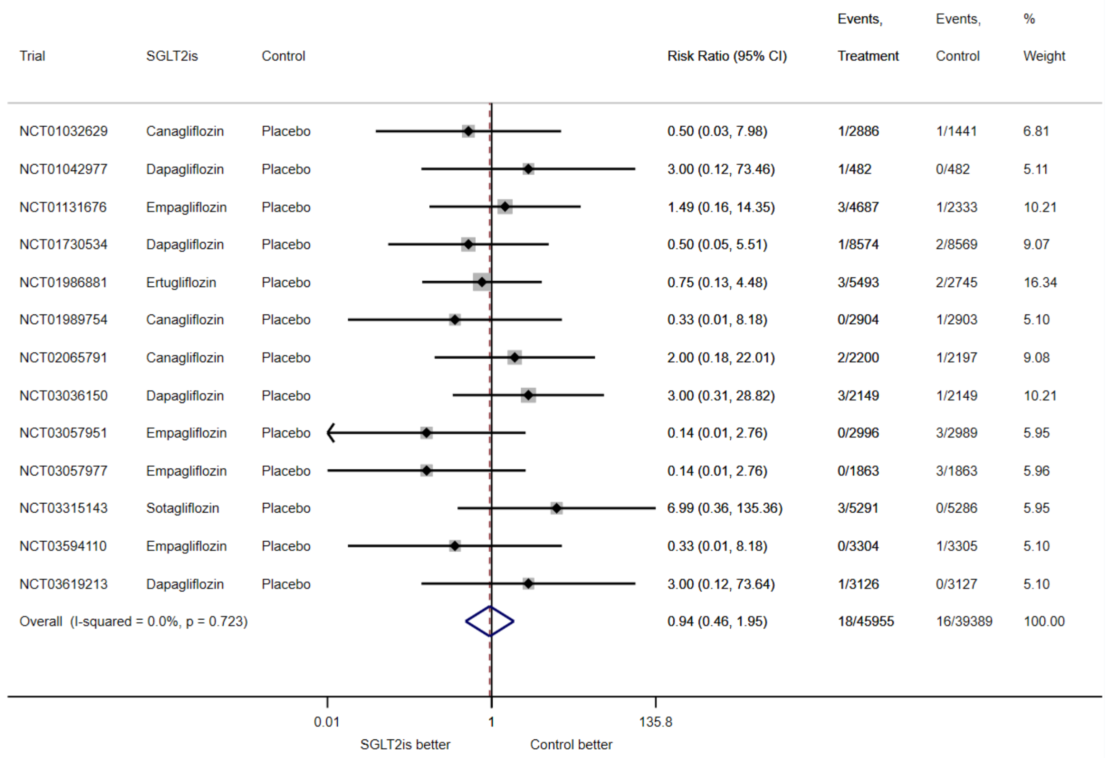

System 23-Skin And Subcutaneous Tissue Disorders-Figure S2 Meta-analysis of SGLT2is and Angioedema

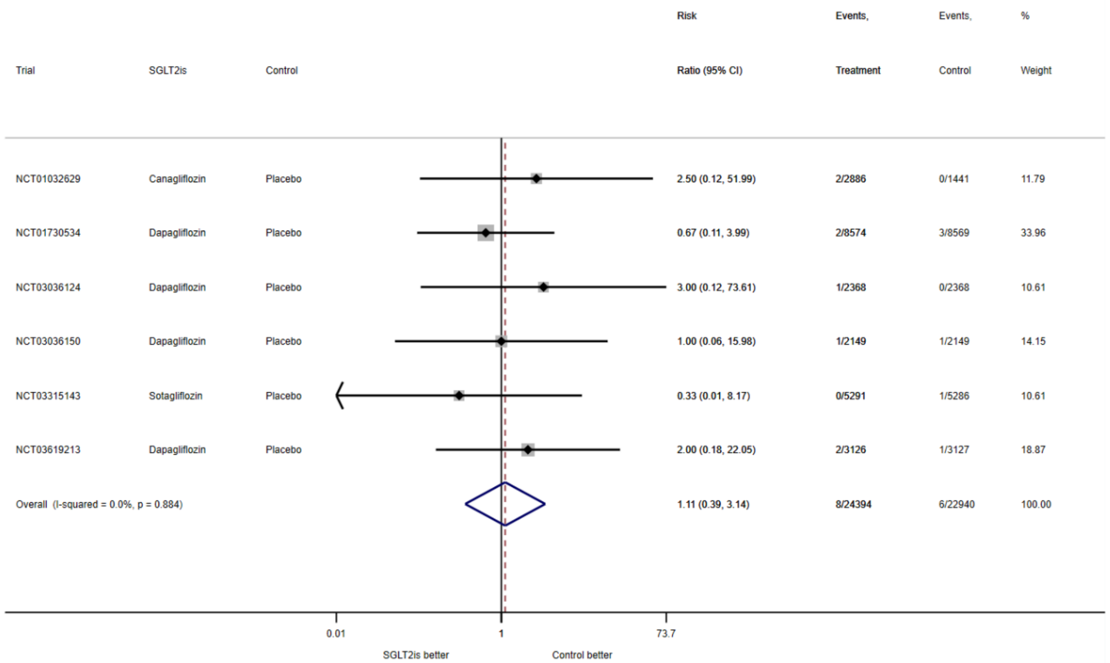

System 23-Skin And Subcutaneous Tissue Disorders-Figure S3 Meta-analysis of SGLT2is and Decubitus ulcer

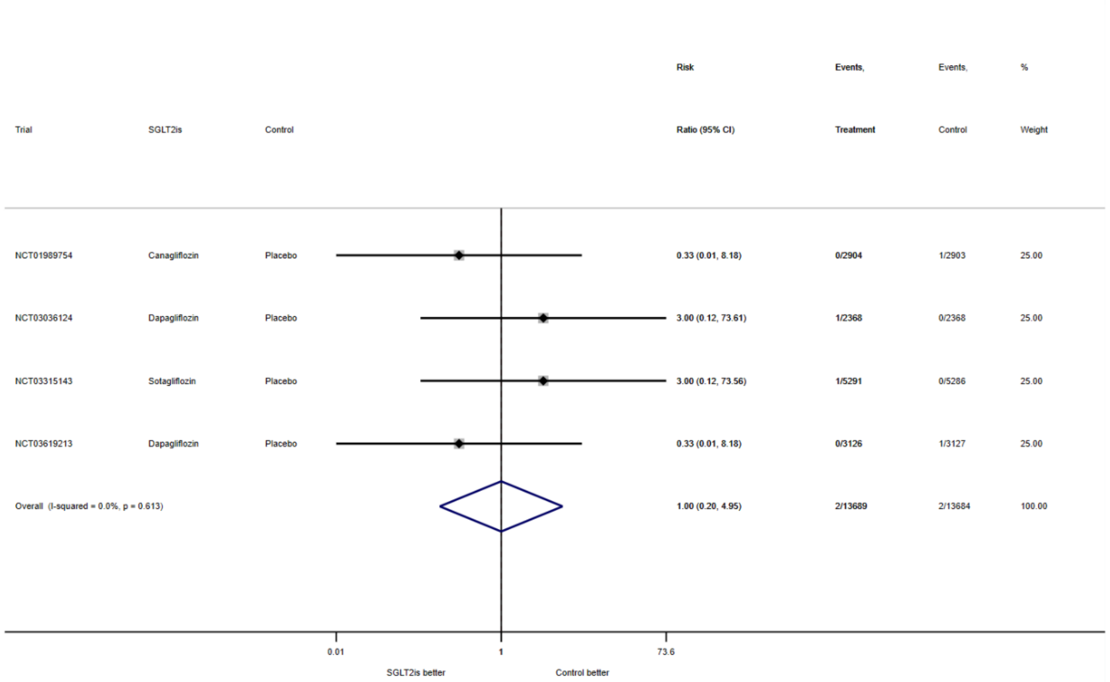

System 23-Skin And Subcutaneous Tissue Disorders-Figure S4 Meta-analysis of SGLT2is and Dermatitis allergic

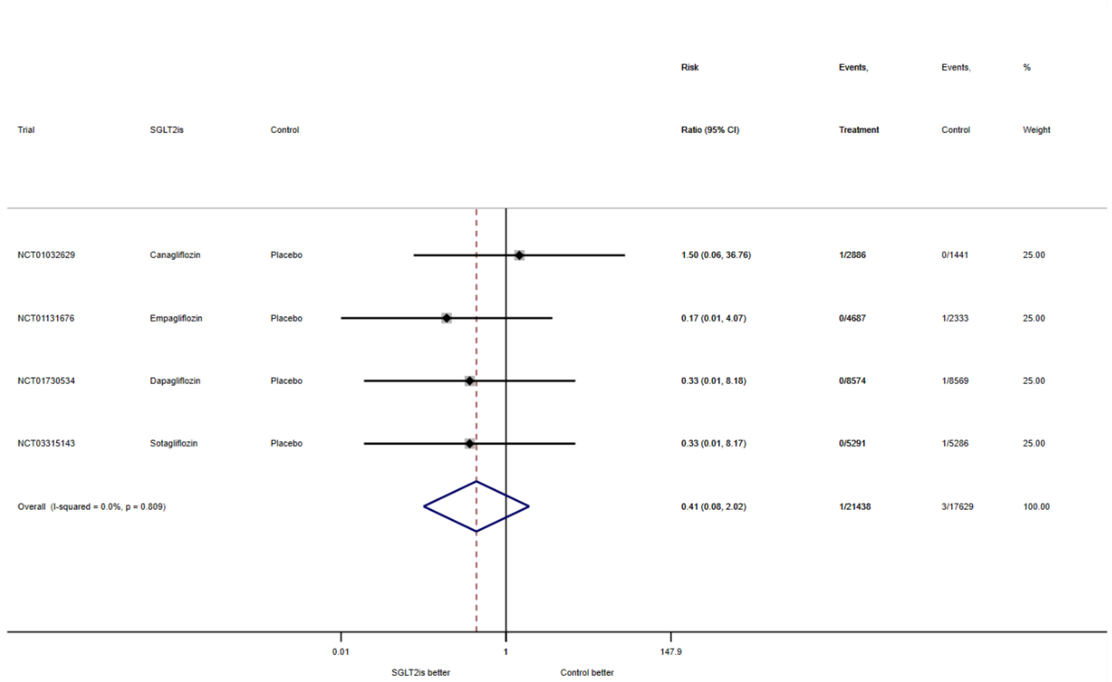

System 23-Skin And Subcutaneous Tissue Disorders-Figure S5 Meta-analysis of SGLT2is and Dermatitis contact

Trial	SGLT2is	Control	Risk Ratio (95% CI)	Events, Treatment	Events, Control	% Weight
NCT01032629	Canagliflozin	Placebo	2.37 (0.81, 6.96)	19/2886	4/1441	5.23
NCT01131676	Empagliflozin	Placebo	0.87 (0.43, 1.77)	21/4687	12/2333	12.10
NCT01164501	Empagliflozin	Placebo	2.29 (0.09, 55.92)	1/419	0/319	0.59
NCT01730534	Dapagliflozin	Placebo	1.28 (0.78, 2.07)	37/8574	29/8569	25.73
NCT01986881	Ertugliflozin	Placebo	0.73 (0.36, 1.48)	19/5493	13/2745	12.22
NCT01989754	Canagliflozin	Placebo	1.11 (0.45, 2.73)	10/2904	9/2903	7.49
NCT02065791	Canagliflozin	Placebo	1.50 (0.67, 3.33)	15/2200	10/2197	9.51
NCT03036124	Dapagliflozin	Placebo	1.00 (0.14, 7.09)	2/2368	2/2368	1.58
NCT03036150	Dapagliflozin	Placebo	0.86 (0.29, 2.55)	6/2149	7/2149	5.11
NCT03057951	Empagliflozin	Placebo	0.80 (0.21, 2.97)	4/2996	5/2989	3.51
NCT03057977	Empagliflozin	Placebo	3.00 (0.61, 14.84)	6/1863	2/1863	2.37
NCT03242252	Sotagliflozin	Placebo	0.16 (0.01, 4.03)	0/527	1/260	0.59
NCT03315143	Sotagliflozin	Placebo	1.55 (0.67, 3.59)	14/5291	9/5286	8.65
NCT03521934	Sotagliflozin	Placebo	1.01 (0.14, 7.15)	2/605	2/611	1.58
NCT03594110	Empagliflozin	Placebo	3.00 (0.12, 73.64)	1/3304	0/3305	0.59
NCT03619213	Dapagliflozin	Placebo	0.50 (0.13, 2.00)	3/3126	6/3127	3.16
Overall (I-squared = 0.0%, p = 0.798)			1.13 (0.89, 1.45)	160/49392	111/42465	100.00

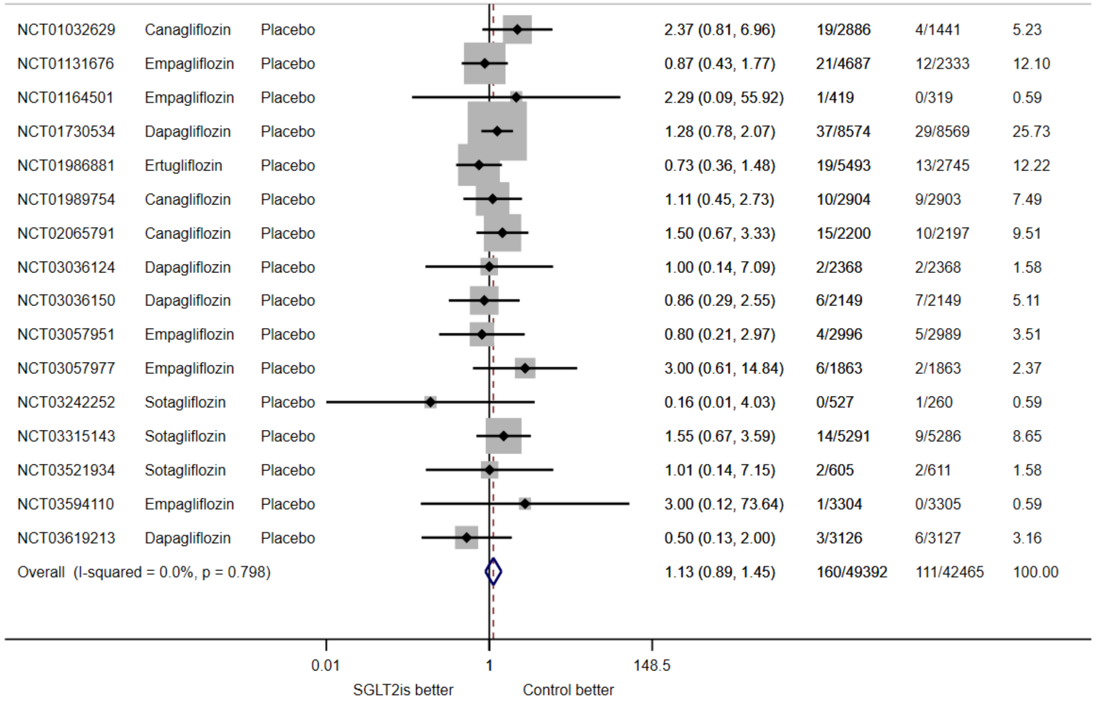

System 23-Skin And Subcutaneous Tissue Disorders-Figure S6 Meta-analysis of SGLT2is and Diabetic foot

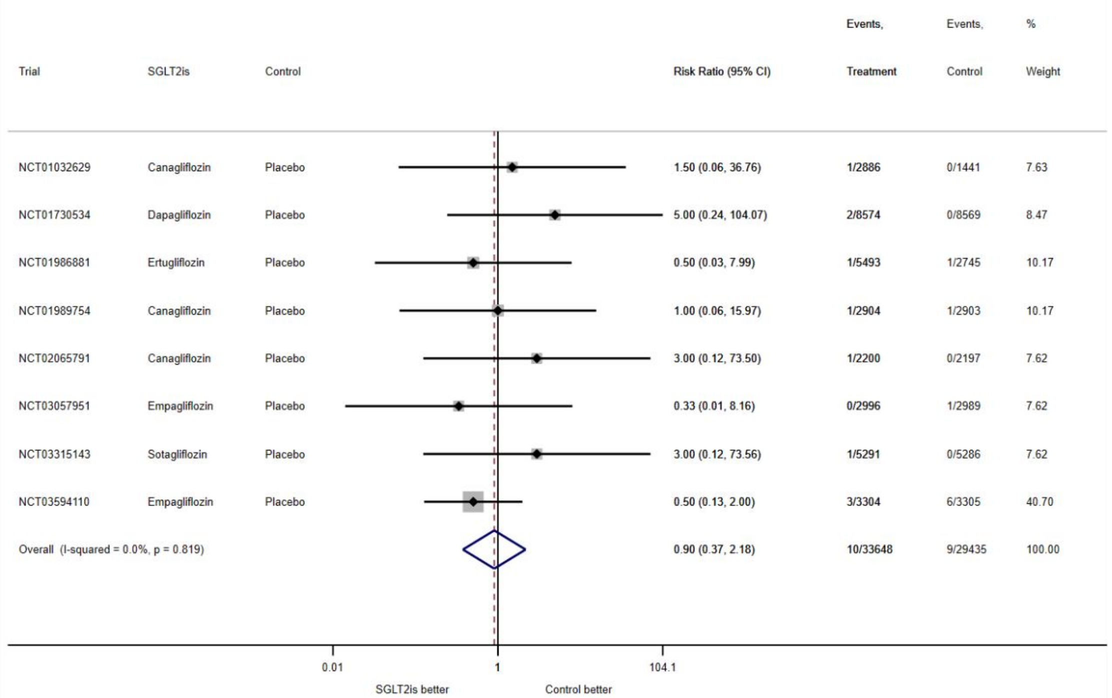

System 23-Skin And Subcutaneous Tissue Disorders-Figure S7 Meta-analysis of SGLT2is and Diabetic ulcer

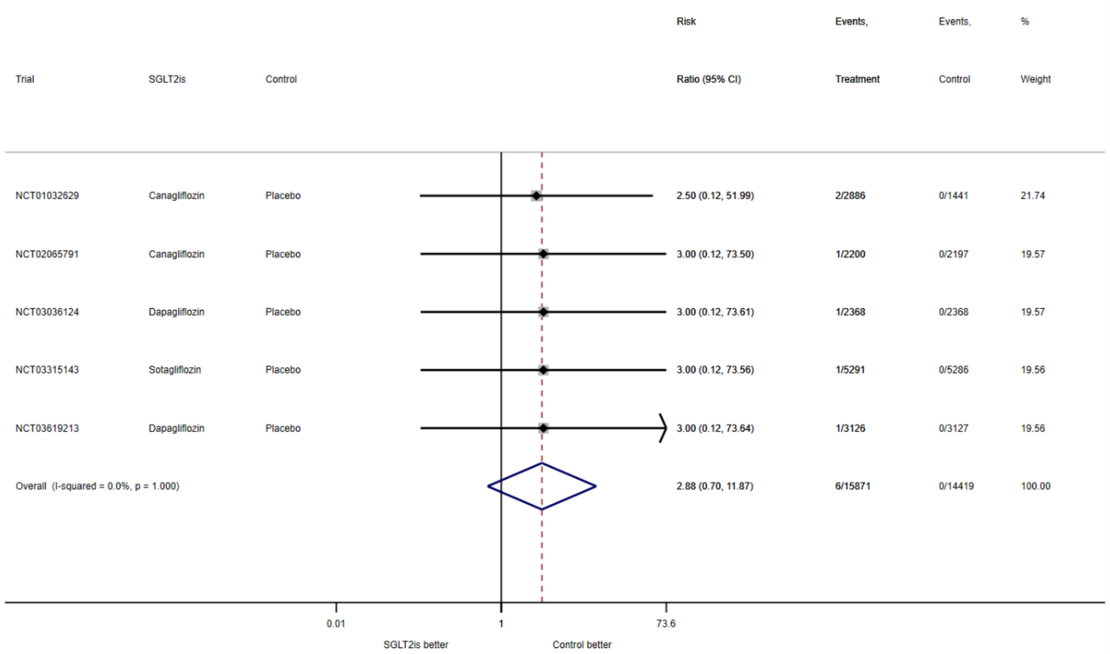

System 23-Skin And Subcutaneous Tissue Disorders-Figure S8 Meta-analysis of SGLT2is and Drug eruption

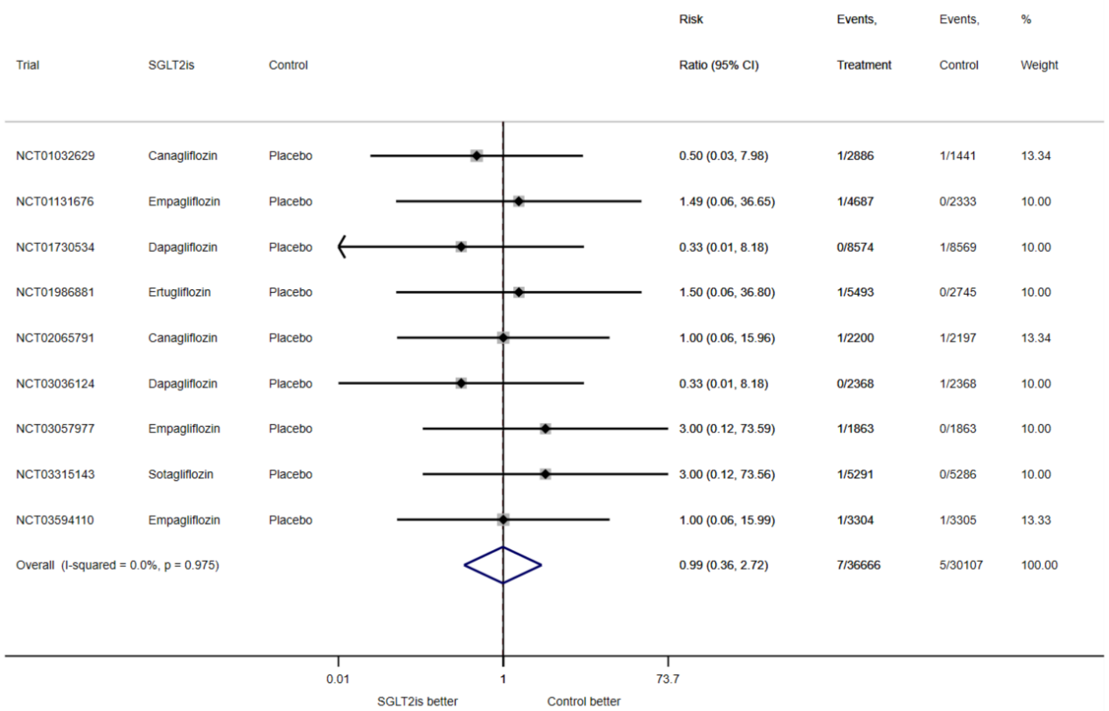

System 23-Skin And Subcutaneous Tissue Disorders-Figure S9 Meta-analysis of SGLT2is and Eczema

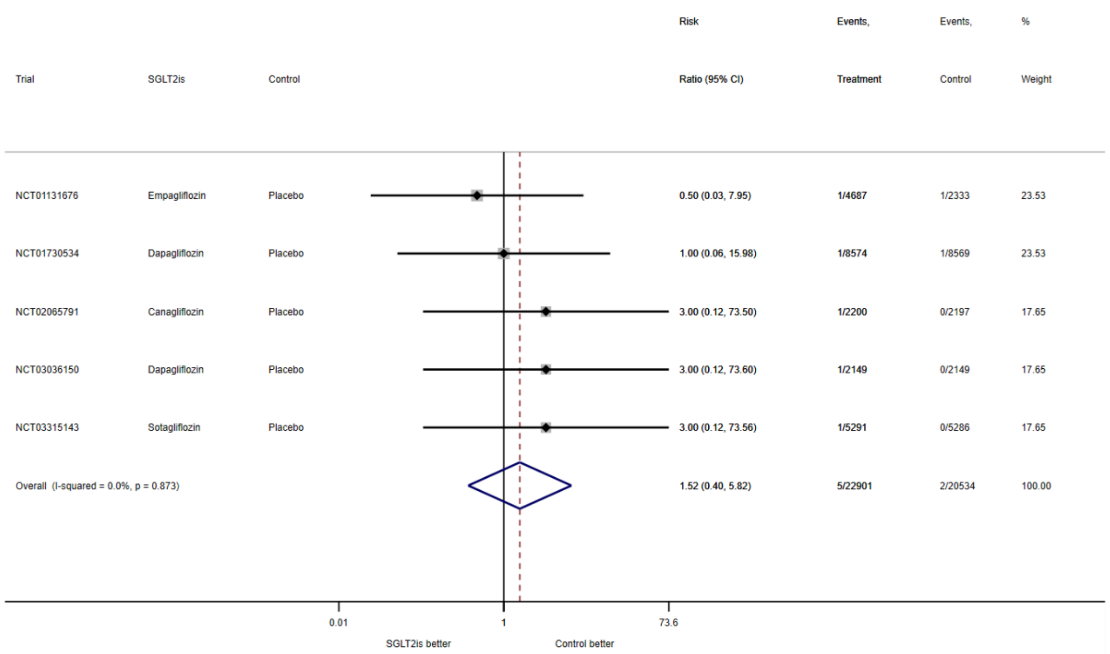

System 23-Skin And Subcutaneous Tissue Disorders-Figure S10 Meta-analysis of SGLT2is and Neuropathic ulcer

Trial	SGLT2is	Control	Risk Ratio (95% CI)	Events, Treatment	Events, Control	% Weight
NCT01106651	Canagliflozin	Placebo	1.49 (0.06, 36.53)	1/477	0/237	15.82
NCT01986881	Ertugliflozin	Placebo	1.50 (0.06, 36.80)	1/5493	0/2745	15.78
NCT02065791	Canagliflozin	Placebo	1.00 (0.06, 15.96)	1/2200	1/2197	21.05
NCT03036124	Dapagliflozin	Placebo	3.00 (0.12, 73.61)	1/2368	0/2368	15.78
NCT03057951	Empagliflozin	Placebo	2.99 (0.12, 73.44)	1/2996	0/2989	15.78
NCT03315143	Sotagliflozin	Placebo	3.00 (0.12, 73.56)	1/5291	0/5286	15.78
Overall (I-squared = 0.0%, p = 0.993)			1.91 (0.54, 6.81)	6/18825	1/15822	100.00

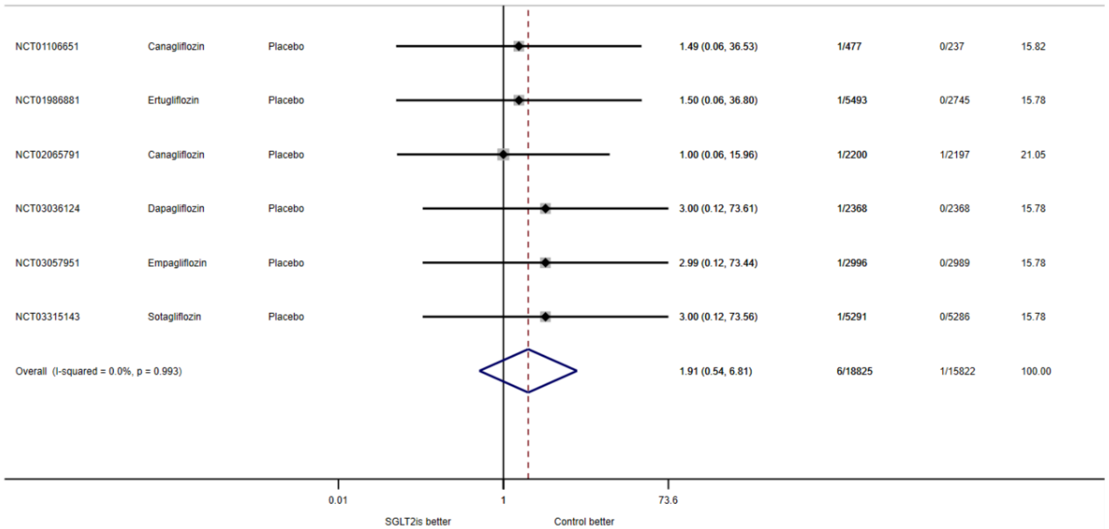

System 23-Skin And Subcutaneous Tissue Disorders-Figure S11 Meta-analysis of SGLT2is and Psoriasis

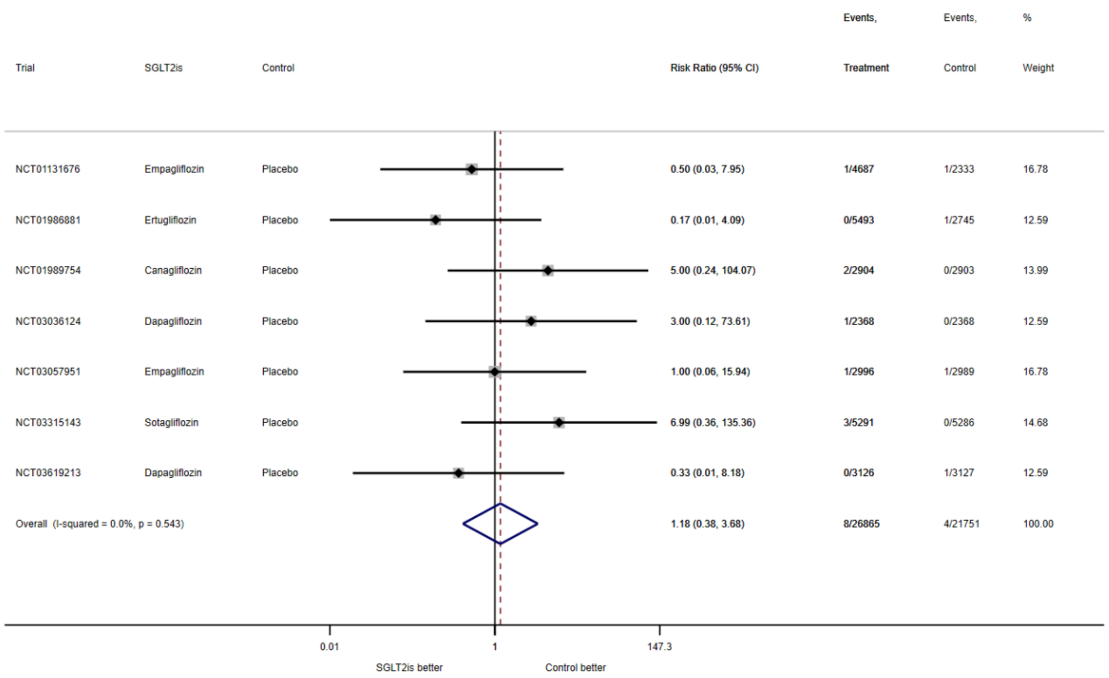

System 23-Skin And Subcutaneous Tissue Disorders-Figure S12 Meta-analysis of SGLT2is and Skin necrosis

Trial	SGLT2is	Control	Risk Ratio (95% CI)	Events, Treatment	Events, Control	% Weight
NCT01032629	Canagliflozin	Placebo	5.99 (1.42, 25.32)	24/2886	2/1441	3.95
NCT01042977	Dapagliflozin	Placebo	3.00 (0.12, 73.46)	1/482	0/482	0.80
NCT01131676	Empagliflozin	Placebo	0.85 (0.39, 1.85)	17/4687	10/2333	13.49
NCT01730534	Dapagliflozin	Placebo	1.04 (0.59, 1.85)	24/8574	23/8569	25.13
NCT01986881	Ertugliflozin	Placebo	1.50 (0.48, 4.64)	12/5493	4/2745	6.41
NCT01989754	Canagliflozin	Placebo	1.40 (0.44, 4.40)	7/2904	5/2903	6.24
NCT02065791	Canagliflozin	Placebo	2.25 (0.98, 5.16)	18/2200	8/2197	11.88
NCT02384941	Sotagliflozin	Placebo	1.53 (0.06, 37.53)	1/525	0/268	0.80
NCT03036124	Dapagliflozin	Placebo	2.00 (0.18, 22.04)	2/2368	1/2368	1.42
NCT03036150	Dapagliflozin	Placebo	0.29 (0.06, 1.37)	2/2149	7/2149	3.32
NCT03057951	Empagliflozin	Placebo	0.54 (0.20, 1.47)	6/2996	11/2989	8.30
NCT03057977	Empagliflozin	Placebo	1.33 (0.30, 5.95)	4/1863	3/1863	3.66
NCT03242252	Sotagliflozin	Placebo	1.48 (0.06, 36.28)	1/527	0/260	0.80
NCT03315143	Sotagliflozin	Placebo	3.00 (0.97, 9.29)	12/5291	4/5286	6.41
NCT03521934	Sotagliflozin	Placebo	5.05 (0.24, 104.96)	2/605	0/611	0.89
NCT03594110	Empagliflozin	Placebo	2.00 (0.18, 22.05)	2/3304	1/3305	1.42
NCT03619213	Dapagliflozin	Placebo	1.00 (0.25, 4.00)	4/3126	4/3127	4.27
NCT04157751	Empagliflozin	Placebo	3.05 (0.12, 74.43)	1/260	0/264	0.80
Overall (I-squared = 6.2%, p = 0.381)			1.28 (0.96, 1.71)	140/50240	83/43160	100.00

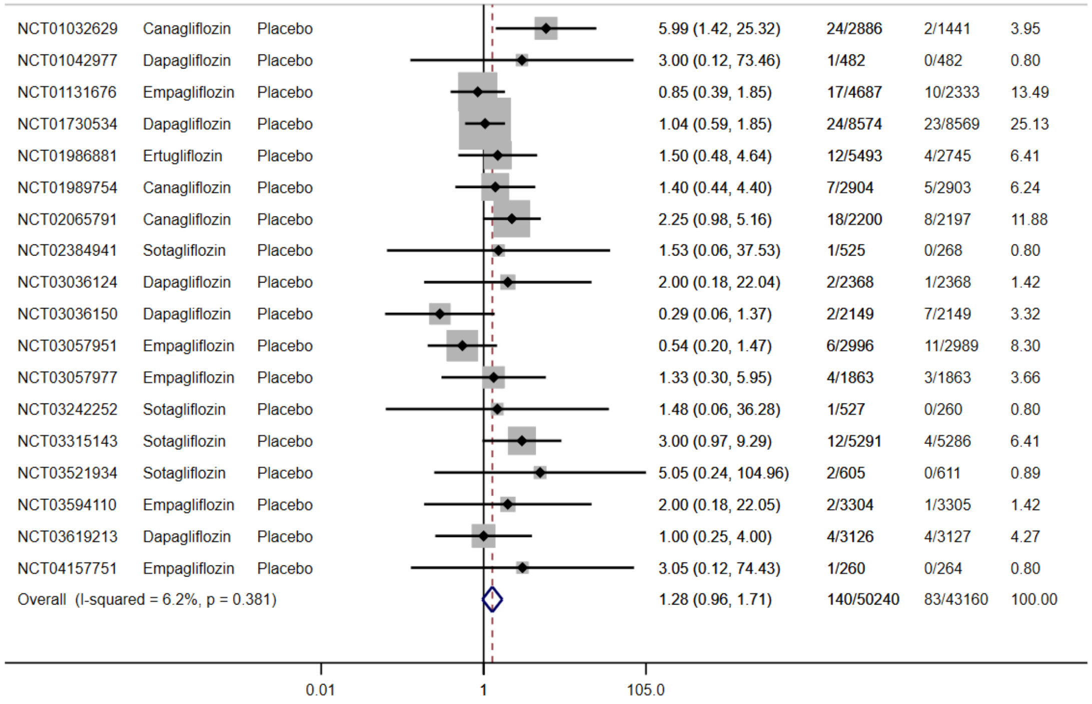

System 23-Skin And Subcutaneous Tissue Disorders-Figure S13 Meta-analysis of SGLT2is and Skin ulcer

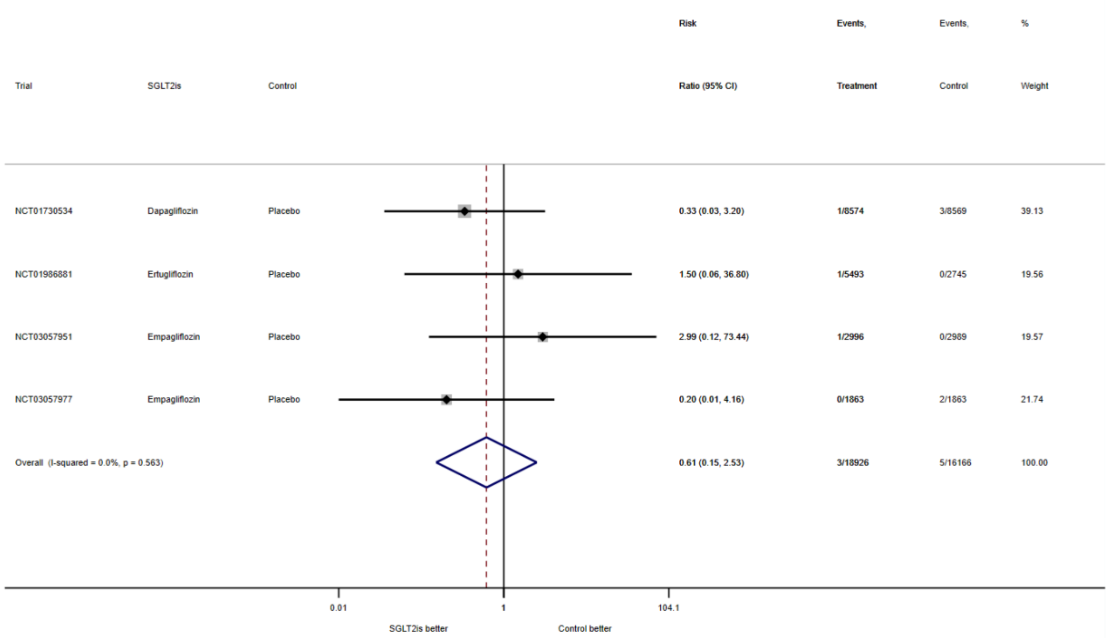

System 23-Skin And Subcutaneous Tissue Disorders-Figure S14 Meta-analysis of SGLT2is and Stasis dermatitis

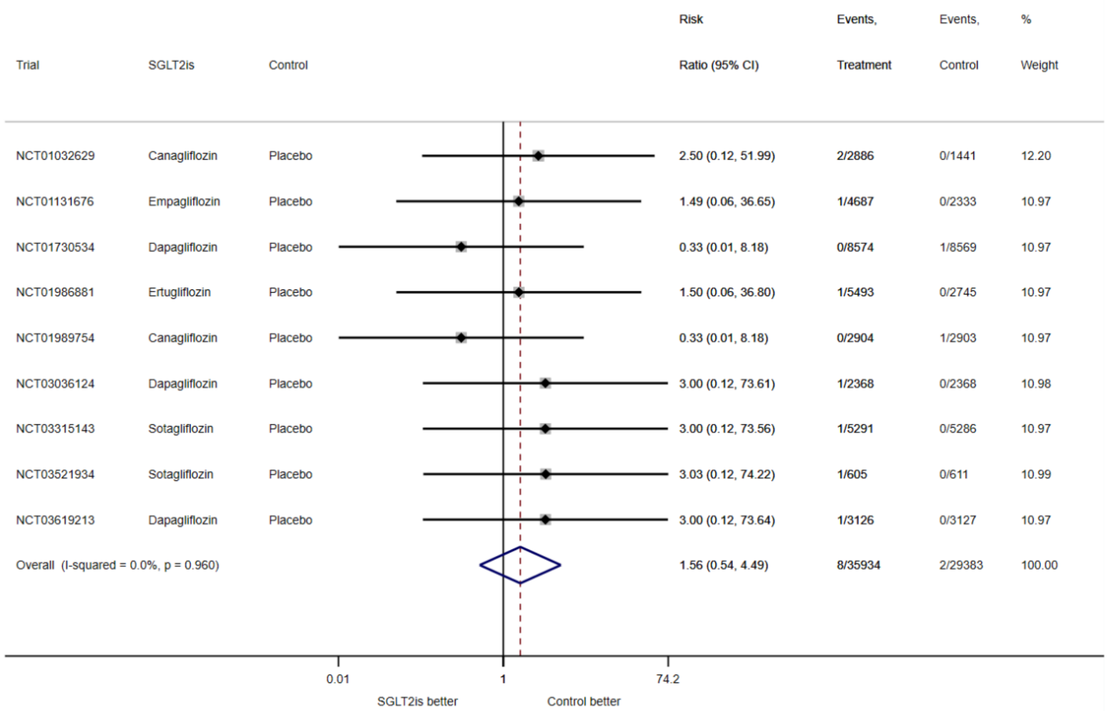

System 23-Skin And Subcutaneous Tissue Disorders-Figure S15 Meta-analysis of SGLT2is and Urticaria

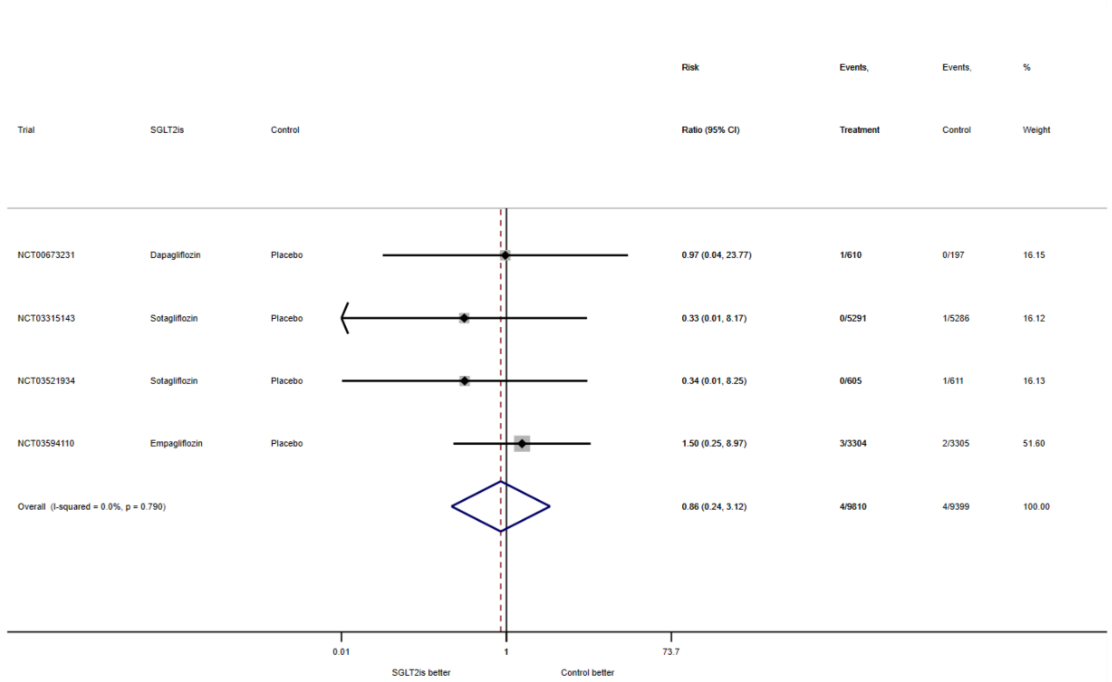

System 24-Surgical And Medical Procedures-Figure S1 Meta-analysis of SGLT2is and Aortic valve replacement

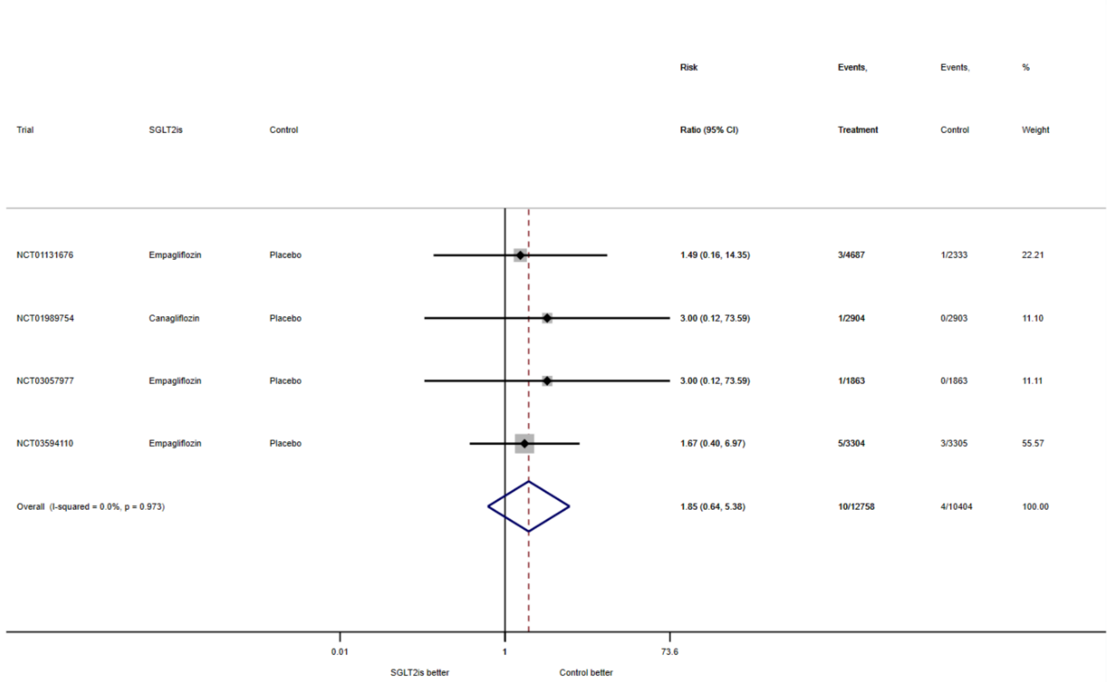

System 24-Surgical And Medical Procedures-Figure S2 Meta-analysis of SGLT2is and Coronary arterial stent insertion

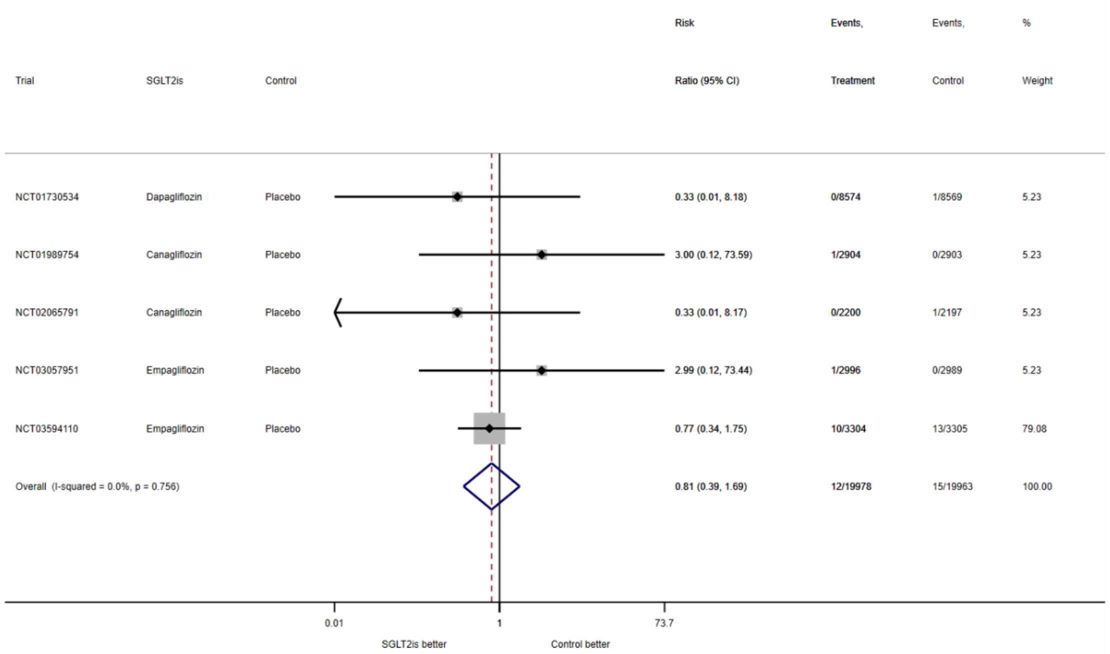

System 24-Surgical And Medical Procedures-Figure S3 Meta-analysis of SGLT2is and Hospitalisation

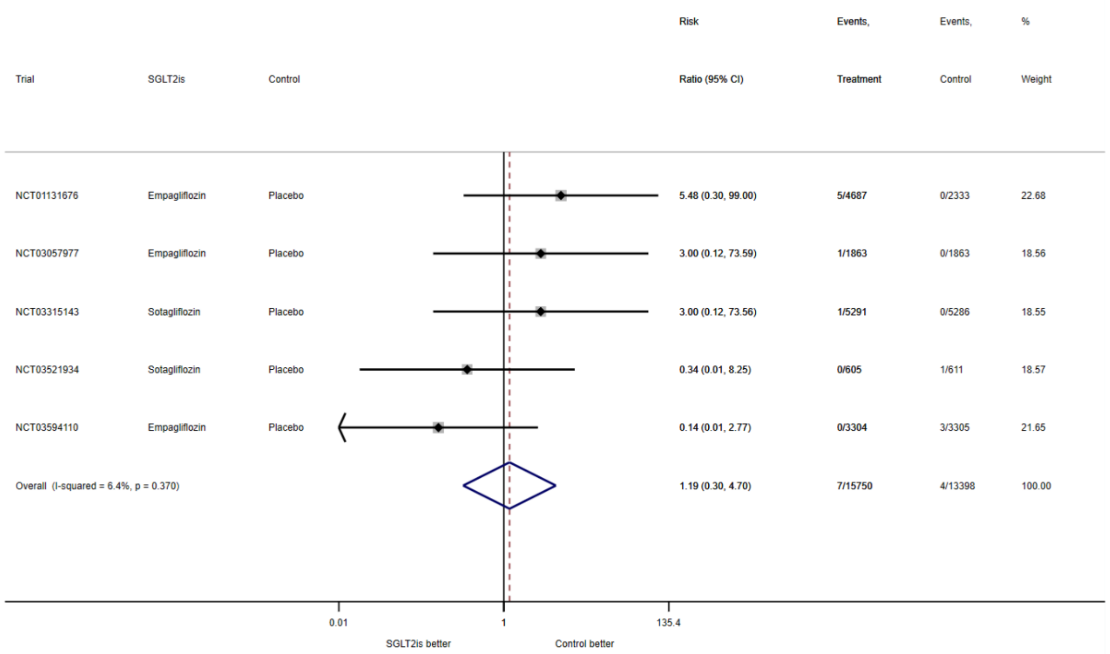

System 24-Surgical And Medical Procedures-Figure S4 Meta-analysis of SGLT2is and Percutaneous coronary intervention

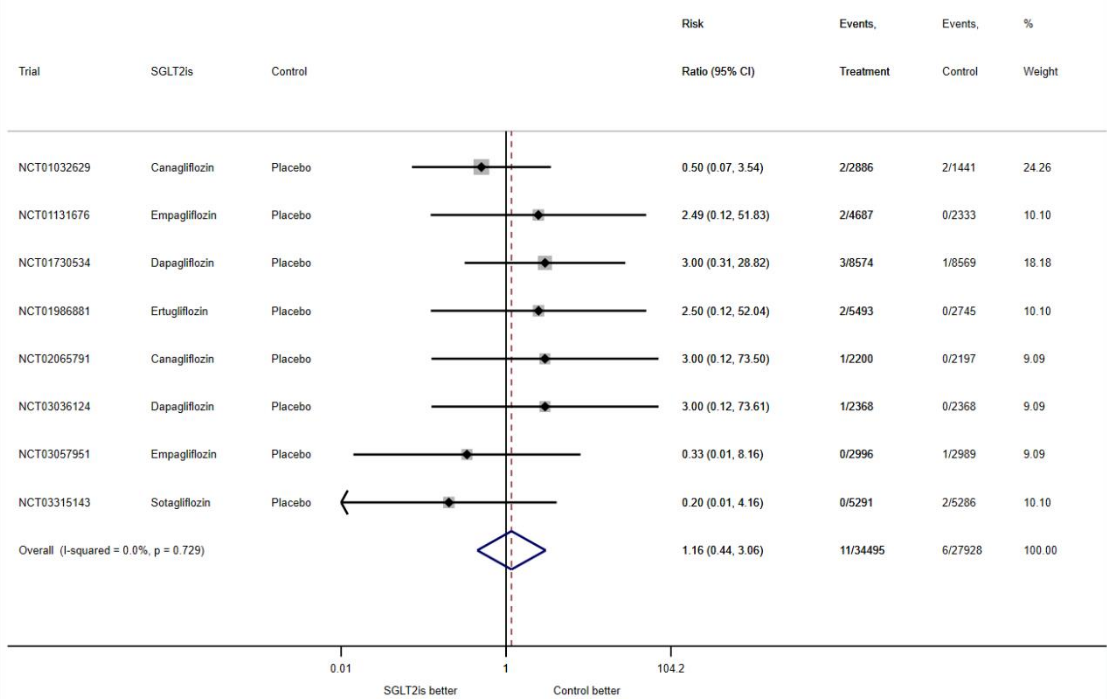

System 25-Vascular Disorders-Figure S1 Meta-analysis of SGLT2is and Accelerated hypertension

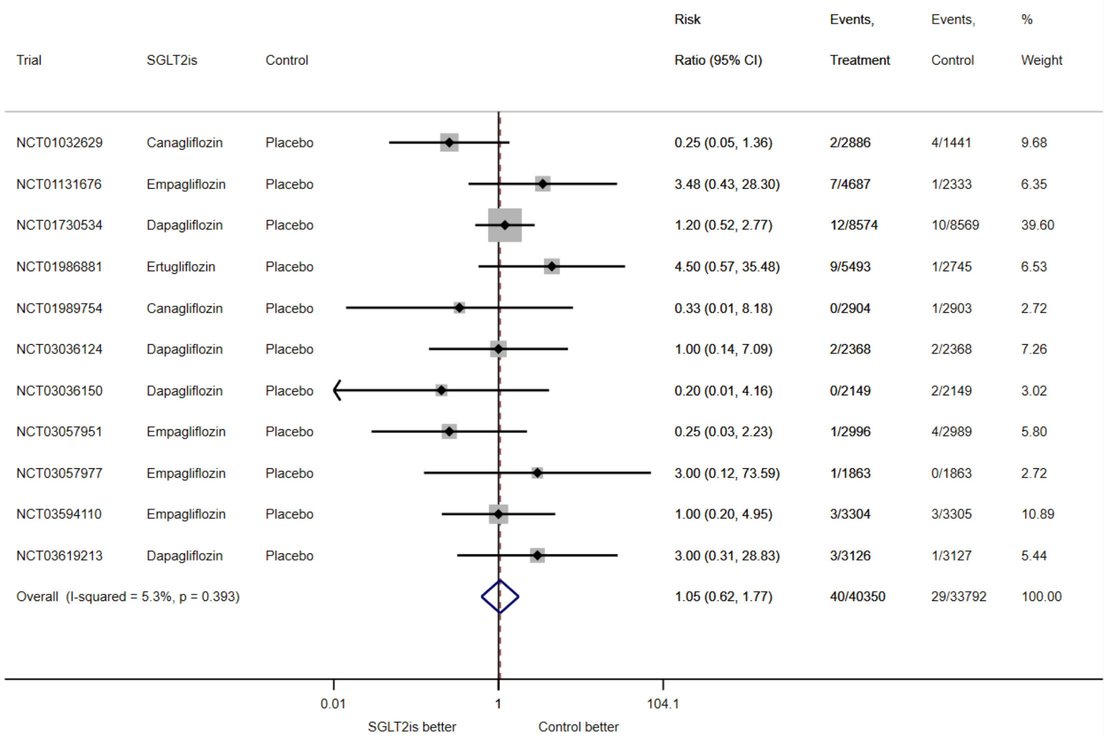

System 25-Vascular Disorders-Figure S2 Meta-analysis of SGLT2is and Aortic aneurysm

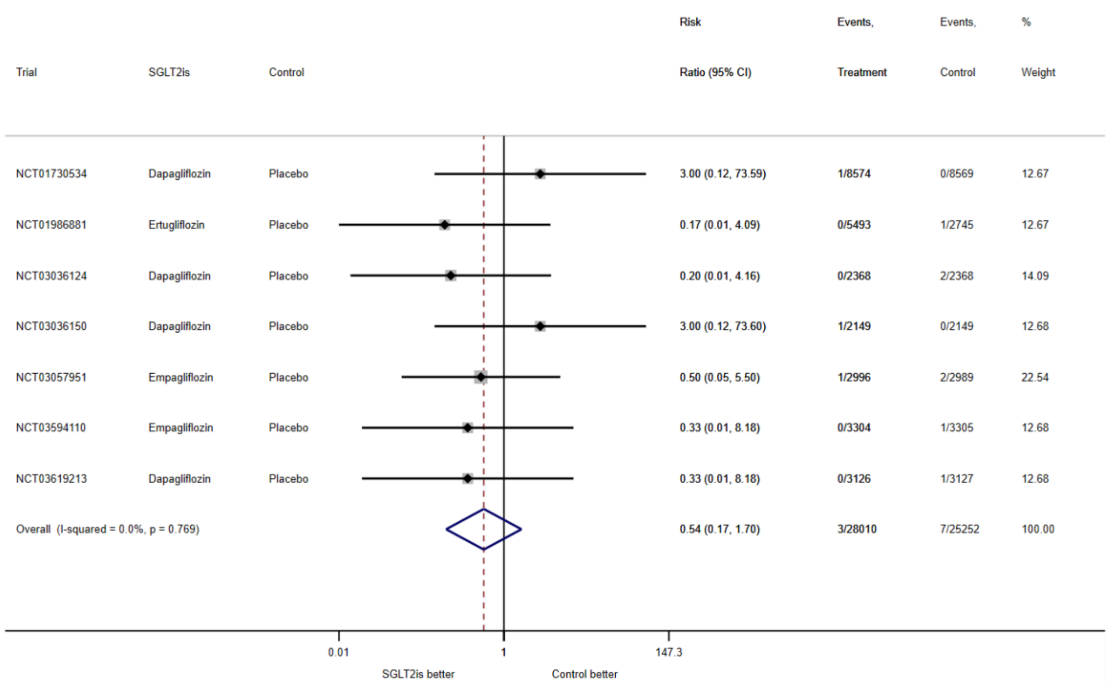

System 25-Vascular Disorders-Figure S3 Meta-analysis of SGLT2is and Aortic aneurysm rupture

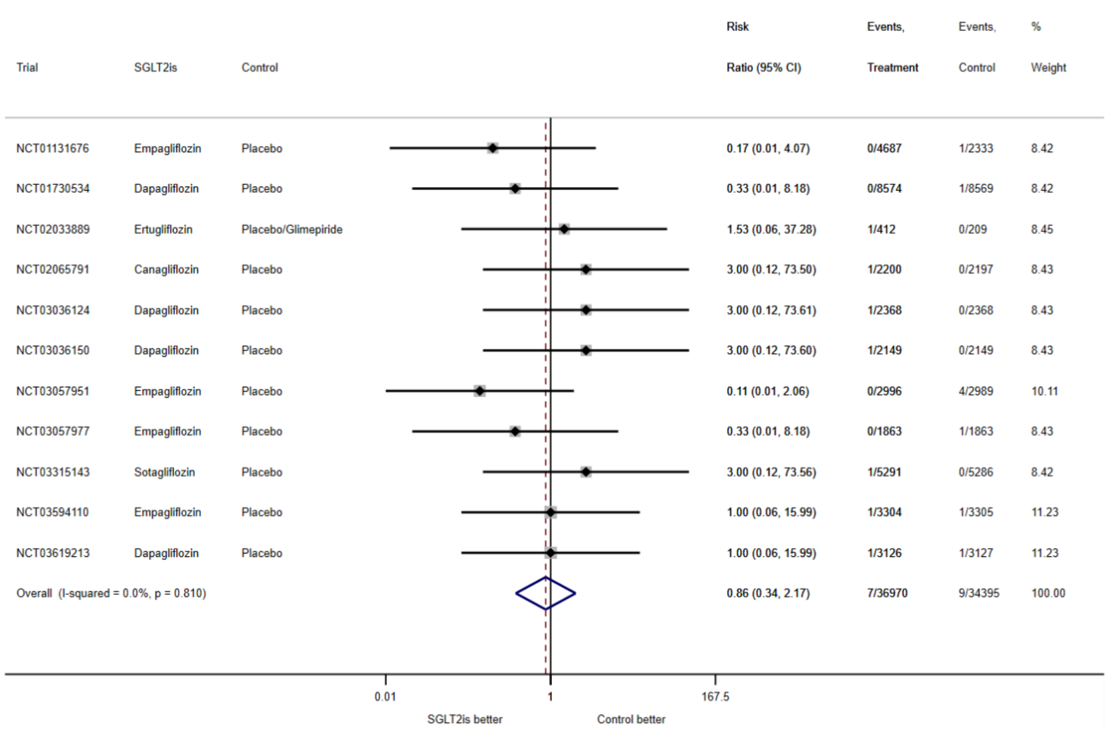

System 25-Vascular Disorders-Figure S4 Meta-analysis of SGLT2is and Aortic dissection

Trial	SGLT2is	Control	Risk Ratio (95% CI)	Events, Treatment	Events, Control	% Weight
NCT01032629	Canagliflozin	Placebo	3.00 (0.36, 24.86)	6/2886	1/1441	3.14
NCT01131676	Empagliflozin	Placebo	0.62 (0.17, 2.31)	5/4687	4/2333	8.14
NCT01164501	Empagliflozin	Placebo	2.29 (0.09, 55.92)	1/419	0/319	1.37
NCT01730534	Dapagliflozin	Placebo	1.29 (0.69, 2.43)	22/8574	17/8569	35.15
NCT01986881	Ertugliflozin	Placebo	0.42 (0.13, 1.36)	5/5493	6/2745	9.99
NCT01989754	Canagliflozin	Placebo	1.25 (0.34, 4.65)	5/2904	4/2903	8.14
NCT02065791	Canagliflozin	Placebo	0.60 (0.14, 2.50)	3/2200	5/2197	6.87
NCT03036124	Dapagliflozin	Placebo	0.33 (0.03, 3.20)	1/2368	3/2368	2.74
NCT03036150	Dapagliflozin	Placebo	0.50 (0.05, 5.51)	1/2149	2/2149	2.44
NCT03057951	Empagliflozin	Placebo	1.40 (0.44, 4.40)	7/2996	5/2989	10.69
NCT03057977	Empagliflozin	Placebo	0.33 (0.01, 8.18)	0/1863	1/1863	1.37
NCT03315143	Sotagliflozin	Placebo	1.00 (0.06, 15.97)	1/5291	1/5286	1.83
NCT03619213	Dapagliflozin	Placebo	0.80 (0.22, 2.98)	4/3126	5/3127	8.14
Overall (I-squared = 0.0%, p = 0.846)			0.95 (0.65, 1.38)	61/44956	54/38289	100.00

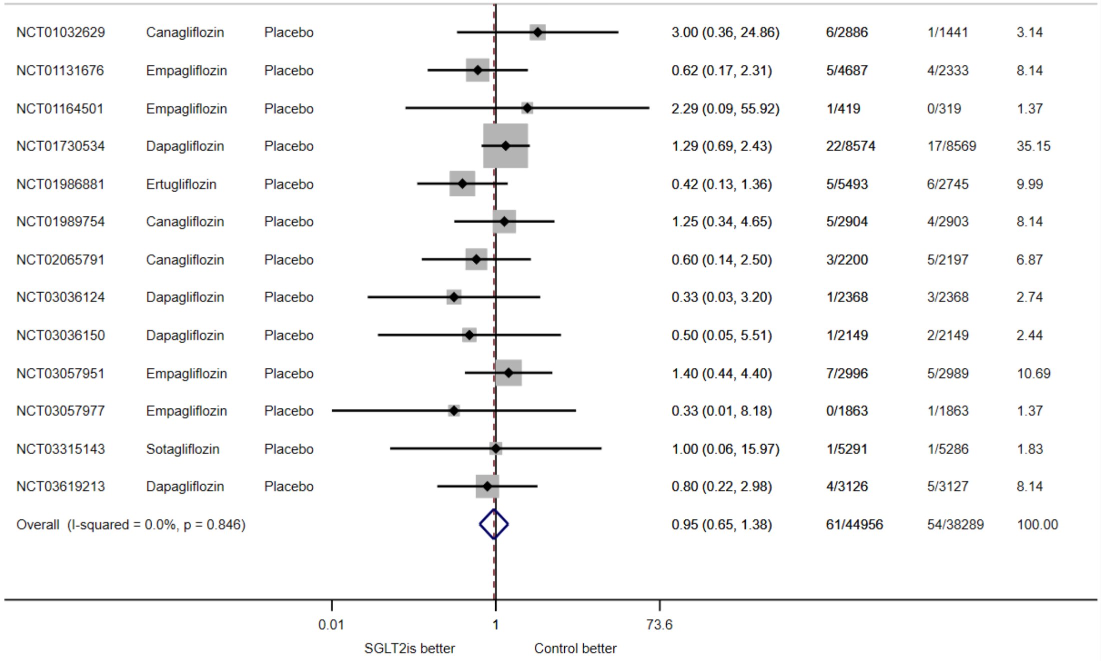

System 25-Vascular Disorders-Figure S5 Meta-analysis of SGLT2is and Aortic stenosis

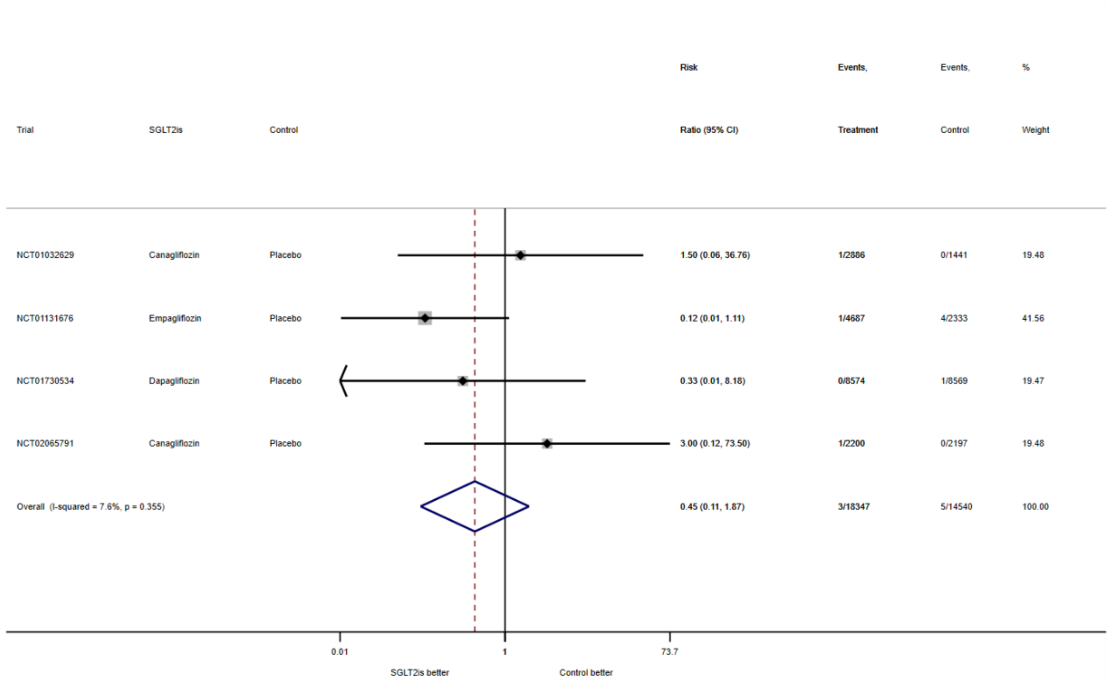

System 25-Vascular Disorders-Figure S6 Meta-analysis of SGLT2is and Arterial disorder

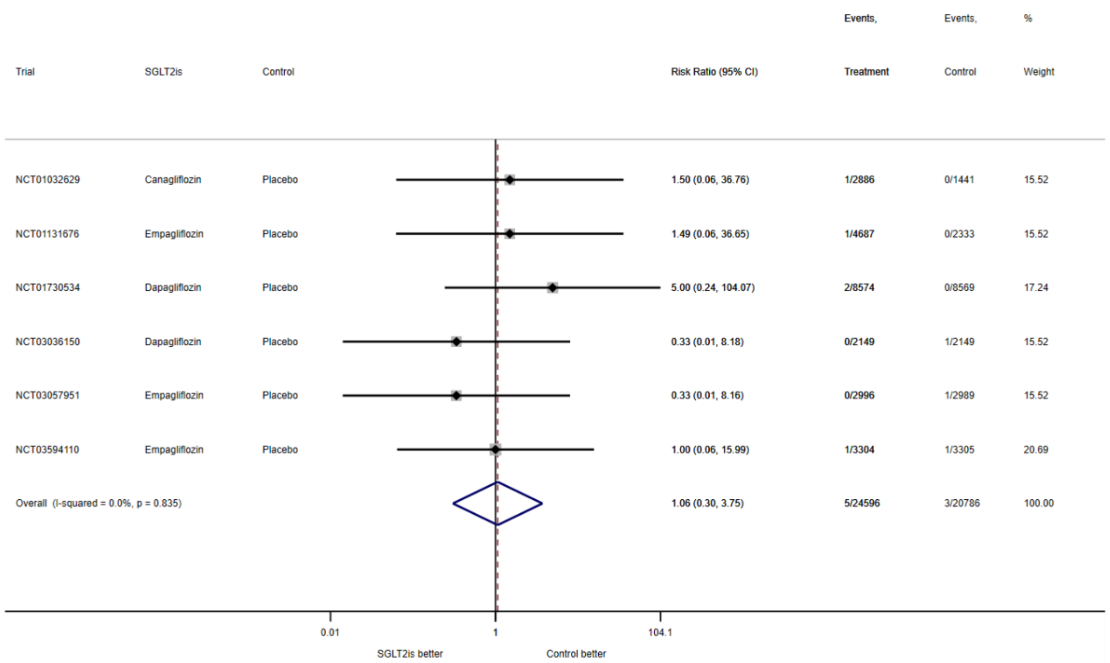

System 25-Vascular Disorders-Figure S7 Meta-analysis of SGLT2is and Arterial occlusive disease

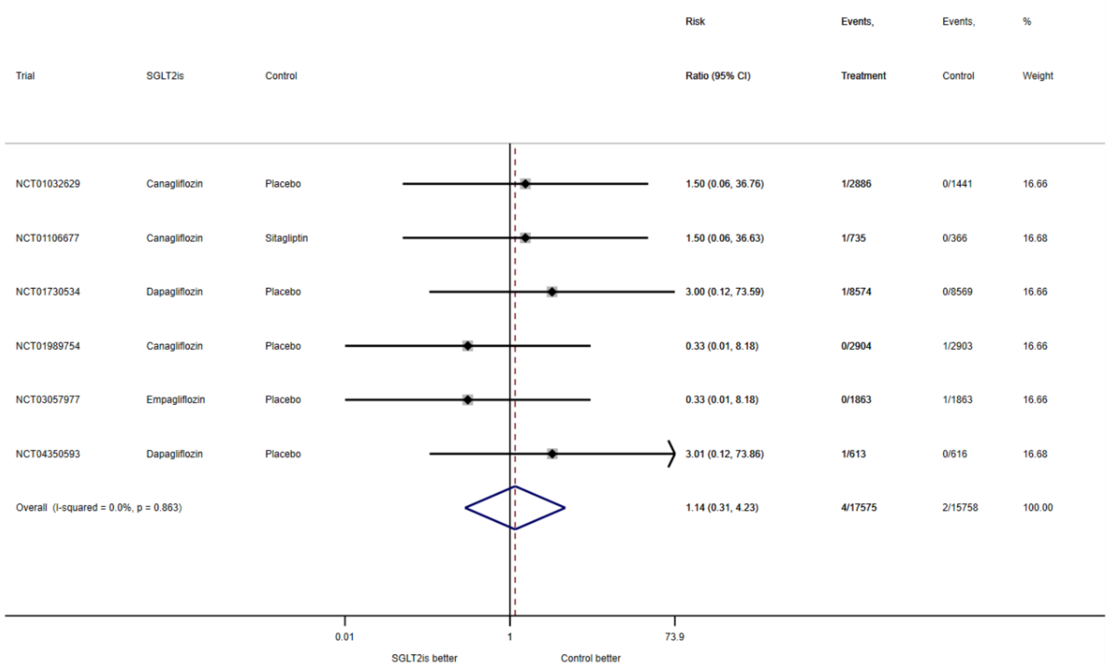

System 25-Vascular Disorders-Figure S8 Meta-analysis of SGLT2is and Arterial thrombosis

Trial	SGLT2is	Control	Risk Ratio (95% CI)	Events, Treatment	Events, Control	% Weight
NCT01032629	Canagliflozin	Placebo	1.50 (0.16, 14.39)	3/2886	1/1441	9.66
NCT01131676	Empagliflozin	Placebo	0.50 (0.07, 3.53)	2/4687	2/2333	12.88
NCT01730534	Dapagliflozin	Placebo	0.80 (0.21, 2.98)	4/8574	5/8569	28.62
NCT01986881	Ertugliflozin	Placebo	0.17 (0.01, 4.09)	0/5493	1/2745	4.83
NCT01989754	Canagliflozin	Placebo	1.00 (0.14, 7.09)	2/2904	2/2903	12.88
NCT02065791	Canagliflozin	Placebo	0.33 (0.03, 3.20)	1/2200	3/2197	9.66
NCT03036150	Dapagliflozin	Placebo	1.00 (0.06, 15.98)	1/2149	1/2149	6.44
NCT03057951	Empagliflozin	Placebo	2.99 (0.12, 73.44)	1/2996	0/2989	4.83
NCT03057977	Empagliflozin	Placebo	0.20 (0.01, 4.16)	0/1863	2/1863	5.37
NCT03521934	Sotagliflozin	Placebo	3.03 (0.12, 74.22)	1/605	0/611	4.83
Overall (I-squared = 0.0%, p = 0.899)			0.75 (0.37, 1.52)	15/34357	17/27800	100.00

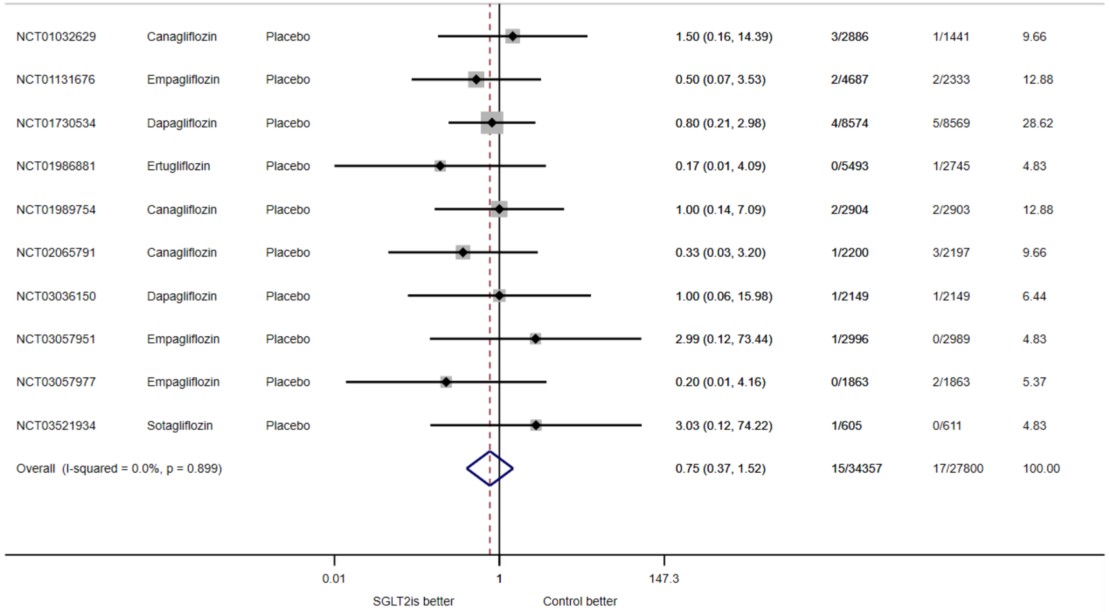

System 25-Vascular Disorders-Figure S9 Meta-analysis of SGLT2is and Arteriosclerosis

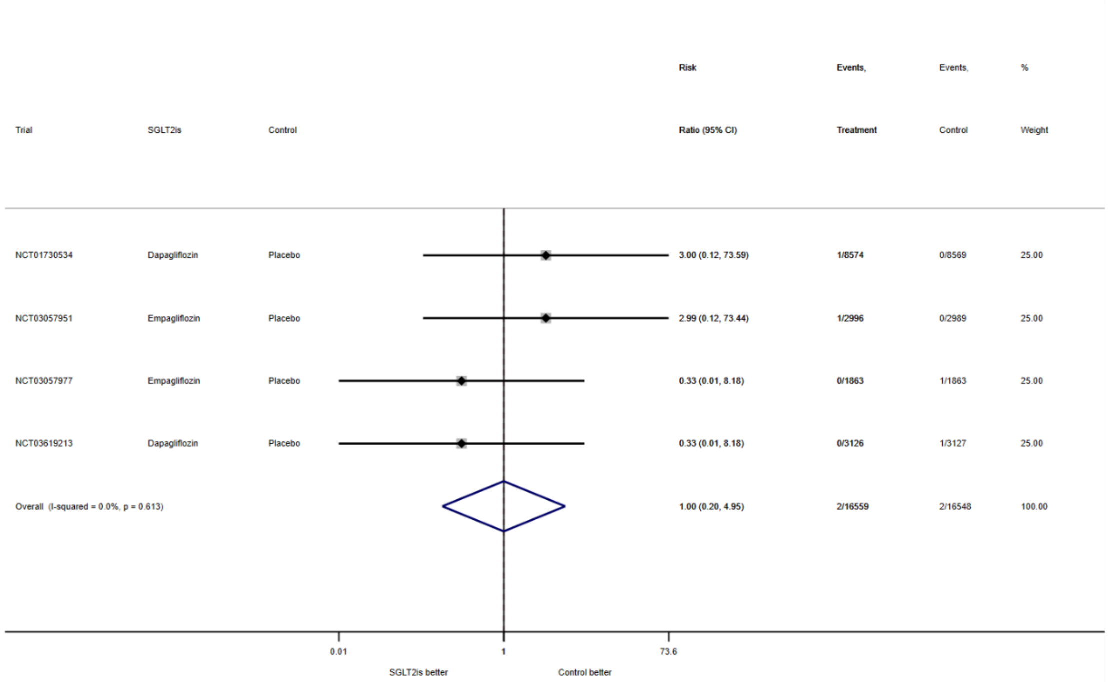

System 25-Vascular Disorders-Figure S10 Meta-analysis of SGLT2is and Bleeding varicose vein

Trial	SGLT2is	Control	Risk Ratio (95% CI)	Events, Treatment	Events, Control	% Weight
NCT01032629	Canagliflozin	Placebo	0.50 (0.03, 7.98)	1/2886	1/1441	5.78
NCT01042977	Dapagliflozin	Placebo	0.33 (0.01, 8.16)	0/482	1/482	4.34
NCT01164501	Empagliflozin	Placebo	0.15 (0.01, 3.16)	0/419	2/319	4.82
NCT01730534	Dapagliflozin	Placebo	0.67 (0.11, 3.99)	2/8574	3/8569	13.86
NCT01986881	Ertugliflozin	Placebo	4.50 (0.24, 83.52)	4/5493	0/2745	5.20
NCT01989754	Canagliflozin	Placebo	1.00 (0.06, 15.97)	1/2904	1/2903	5.77
NCT02065791	Canagliflozin	Placebo	3.00 (0.12, 73.50)	1/2200	0/2197	4.33
NCT03036124	Dapagliflozin	Placebo	0.33 (0.03, 3.20)	1/2368	3/2368	8.66
NCT03057951	Empagliflozin	Placebo	0.75 (0.17, 3.34)	3/2996	4/2989	19.81
NCT03057977	Empagliflozin	Placebo	0.09 (0.01, 1.64)	0/1863	5/1863	5.29
NCT03315143	Sotagliflozin	Placebo	0.50 (0.05, 5.51)	1/5291	2/5286	7.70
NCT03521934	Sotagliflozin	Placebo	3.03 (0.12, 74.22)	1/605	0/611	4.33
NCT03594110	Empagliflozin	Placebo	1.00 (0.06, 15.99)	1/3304	1/3305	5.77
NCT03619213	Dapagliflozin	Placebo	3.00 (0.12, 73.64)	1/3126	0/3127	4.33
Overall (I-squared = 0.0%, p = 0.861)			0.71 (0.36, 1.38)	17/42511	23/38205	100.00

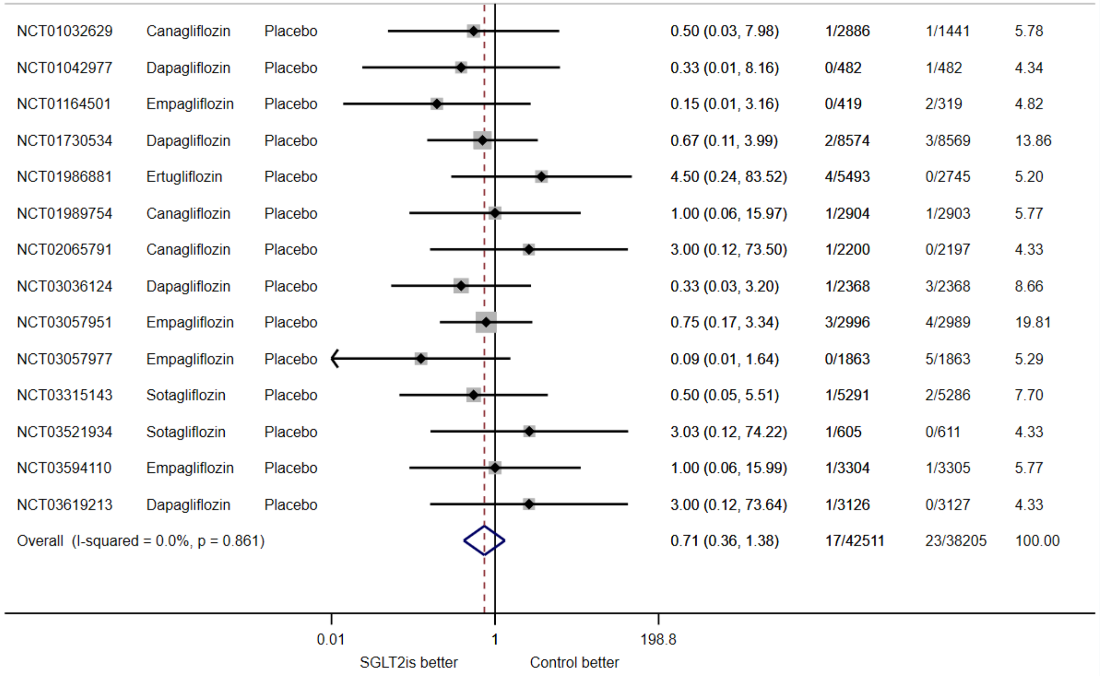

System 25-Vascular Disorders-Figure S11 Meta-analysis of SGLT2is and Circulatory collapse

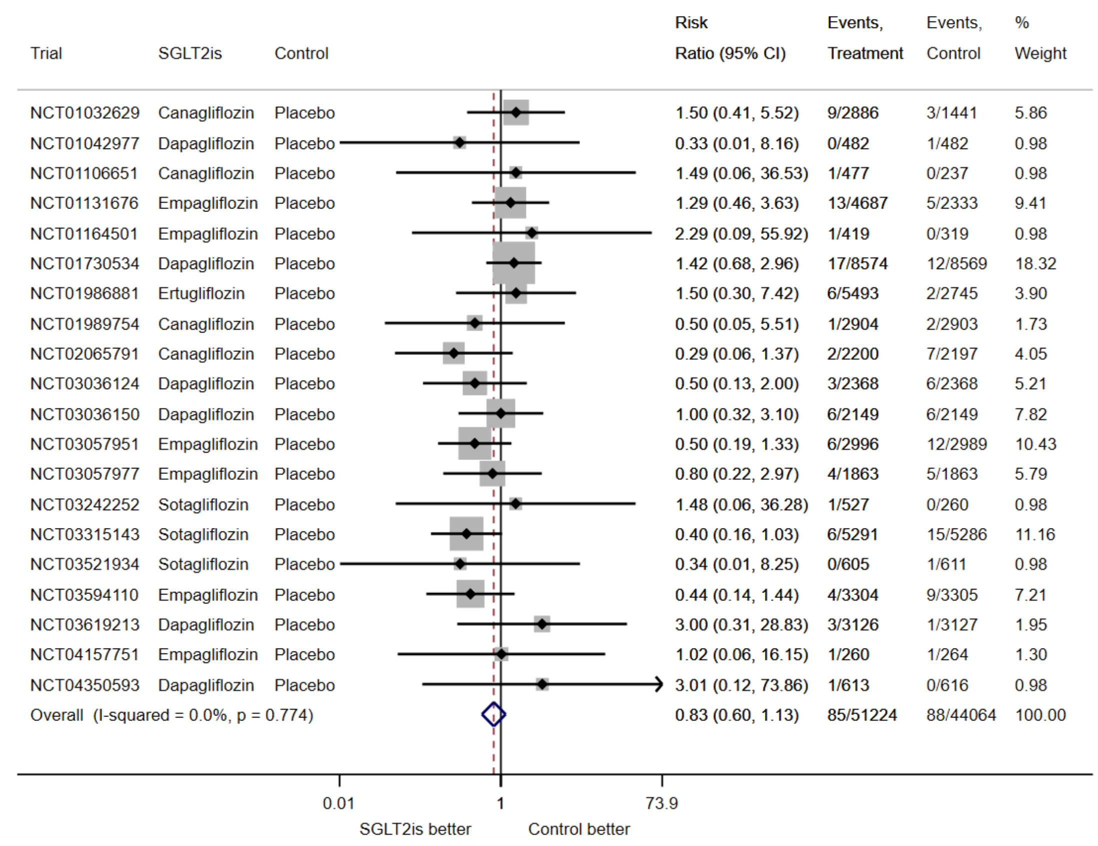

System 25-Vascular Disorders-Figure S12 Meta-analysis of SGLT2is and Deep vein thrombosis

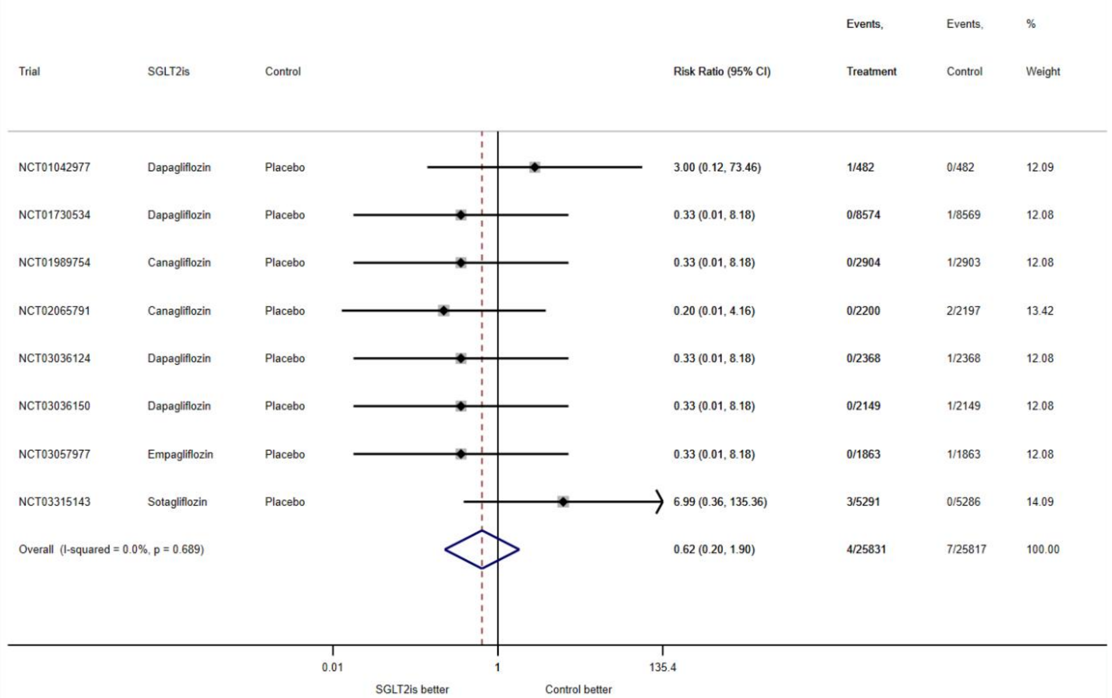

System 25-Vascular Disorders-Figure S13 Meta-analysis of SGLT2is and Diabetic vascular disorder

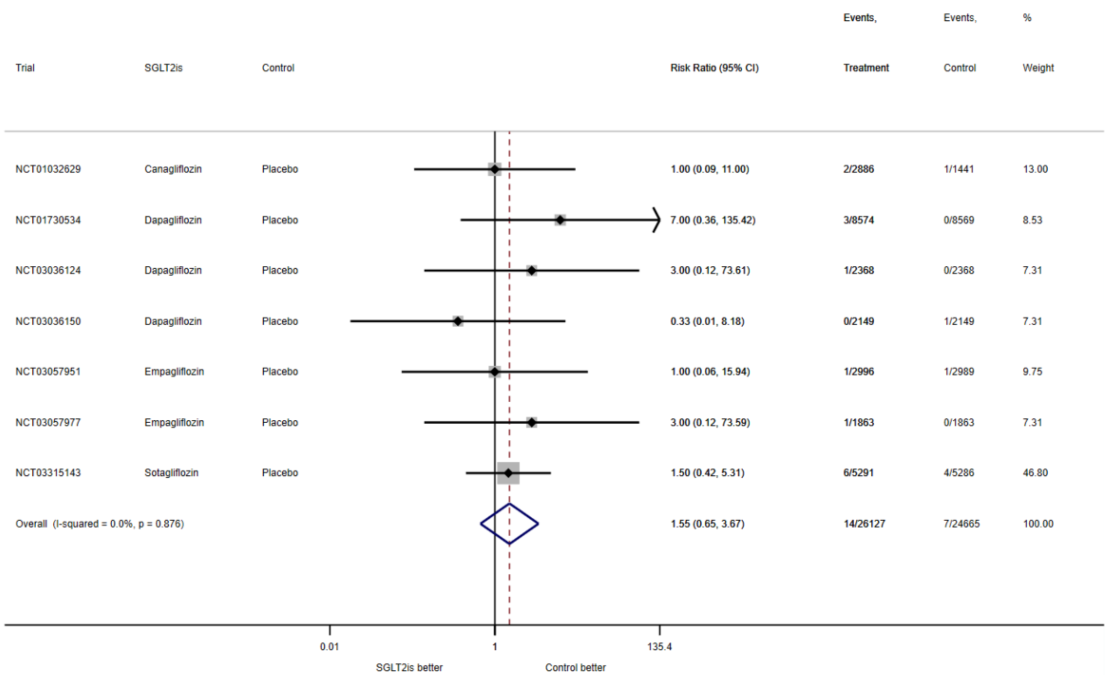

System 25-Vascular Disorders-Figure S14 Meta-analysis of SGLT2is and Dry gangrene

Trial	SGLT2is	Control	Risk Ratio (95% CI)	Events, Treatment	Events, Control	% Weight
NCT01131676	Empagliflozin	Placebo	0.50 (0.03, 7.95)	1/4687	1/2333	24.49
NCT01730534	Dapagliflozin	Placebo	0.20 (0.01, 4.16)	0/8574	2/8569	20.40
NCT02065791	Canagliflozin	Placebo	0.33 (0.01, 8.17)	0/2200	1/2197	18.37
NCT03057951	Empagliflozin	Placebo	2.99 (0.12, 73.44)	1/2996	0/2989	18.37
NCT03057977	Empagliflozin	Placebo	3.00 (0.12, 73.59)	1/1863	0/1863	18.37
Overall (I-squared = 0.0%, p = 0.645)			0.74 (0.19, 2.92)	3/20320	4/17951	100.00

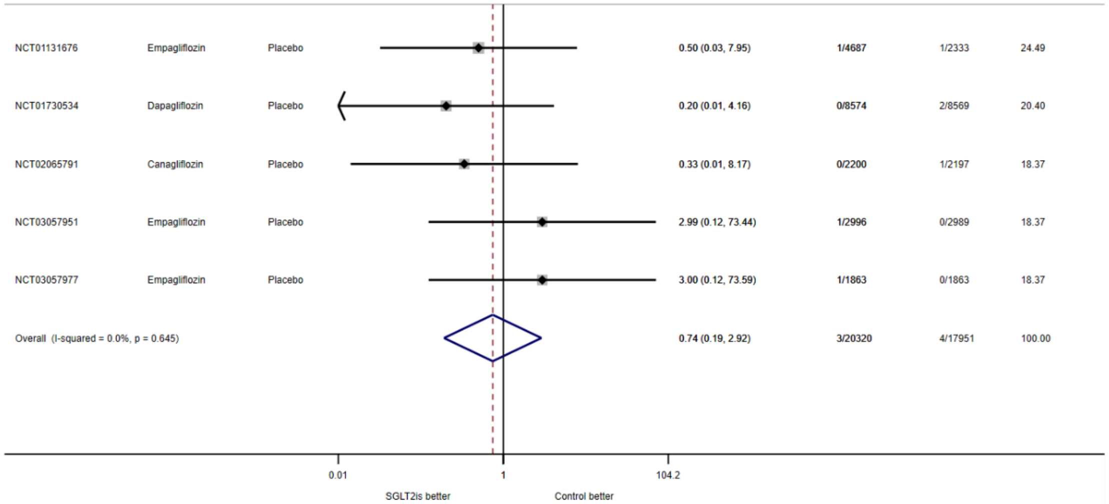

System 25-Vascular Disorders-Figure S15 Meta-analysis of SGLT2is and Embolism

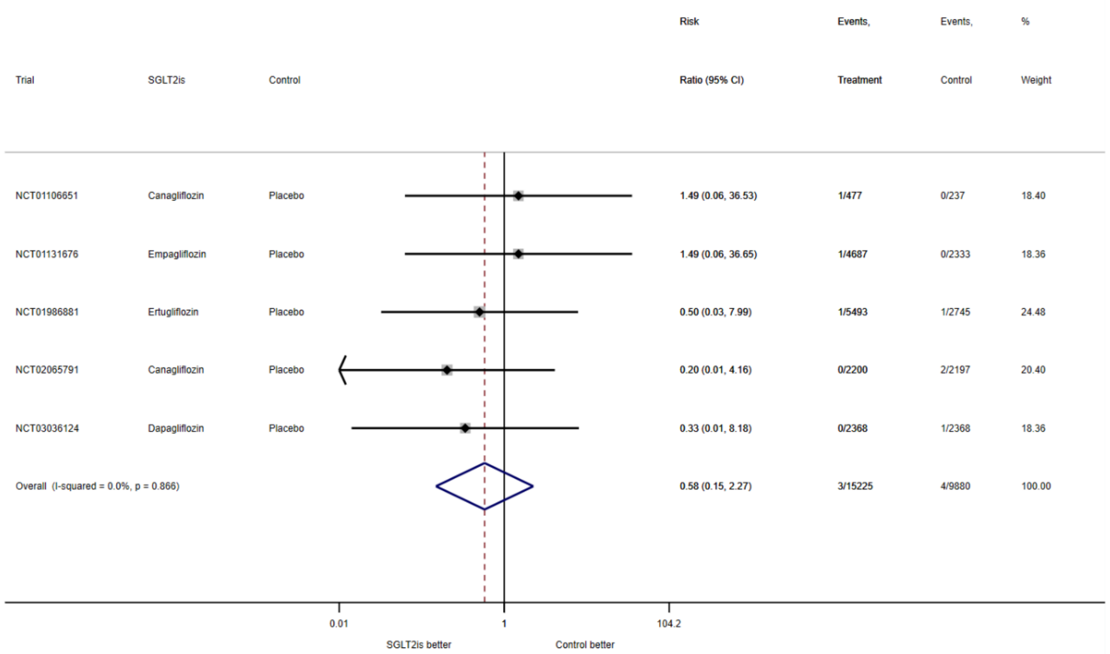

System 25-Vascular Disorders-Figure S16 Meta-analysis of SGLT2is and Embolism arterial

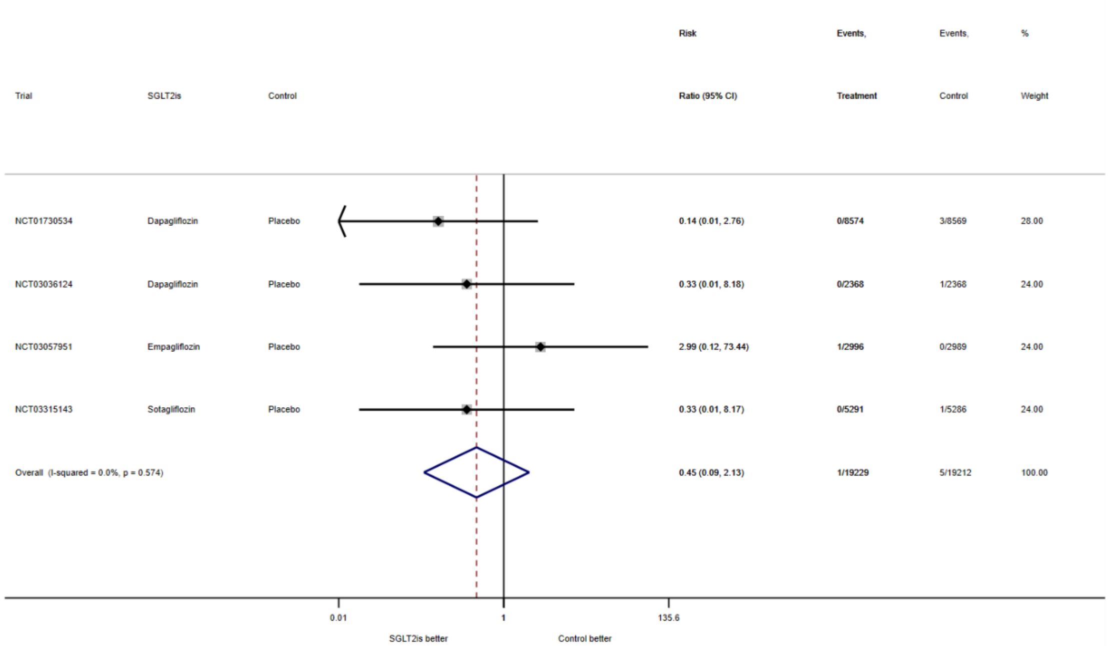

System 25-Vascular Disorders-Figure S17 Meta-analysis of SGLT2is and Essential hypertension

Trial	SGLT2is	Control	Risk Ratio (95% CI)	Events, Treatment	Events, Control	% Weight
NCT01032629	Canagliflozin	Placebo	5.49 (0.30, 99.29)	5/2886	0/1441	2.24
NCT01042977	Dapagliflozin	Placebo	0.33 (0.01, 8.16)	0/482	1/482	1.84
NCT01131676	Empagliflozin	Placebo	1.19 (0.42, 3.39)	12/4687	5/2333	17.30
NCT01164501	Empagliflozin	Placebo	2.29 (0.09, 55.92)	1/419	0/319	1.84
NCT01730534	Dapagliflozin	Placebo	1.75 (0.51, 5.97)	7/8574	4/8569	12.45
NCT01986881	Ertugliflozin	Placebo	1.00 (0.30, 3.32)	8/5493	4/2745	13.06
NCT01989754	Canagliflozin	Placebo	0.60 (0.14, 2.51)	3/2904	5/2903	9.18
NCT02065791	Canagliflozin	Placebo	1.12 (0.43, 2.91)	9/2200	8/2197	20.79
NCT03036124	Dapagliflozin	Placebo	3.00 (0.12, 73.61)	1/2368	0/2368	1.83
NCT03036150	Dapagliflozin	Placebo	7.00 (0.36, 135.44)	3/2149	0/2149	2.14
NCT03057951	Empagliflozin	Placebo	0.50 (0.09, 2.72)	2/2996	4/2989	6.53
NCT03057977	Empagliflozin	Placebo	2.00 (0.18, 22.04)	2/1863	1/1863	3.26
NCT03242252	Sotagliflozin	Placebo	1.48 (0.06, 36.28)	1/527	0/260	1.84
NCT03315143	Sotagliflozin	Placebo	0.33 (0.01, 8.17)	0/5291	1/5286	1.83
NCT03619213	Dapagliflozin	Placebo	0.20 (0.01, 4.17)	0/3126	2/3127	2.04
NCT04157751	Empagliflozin	Placebo	3.05 (0.12, 74.43)	1/260	0/264	1.84
Overall (I-squared = 0.0%, p = 0.911)			1.13 (0.73, 1.75)	55/46225	35/39295	100.00

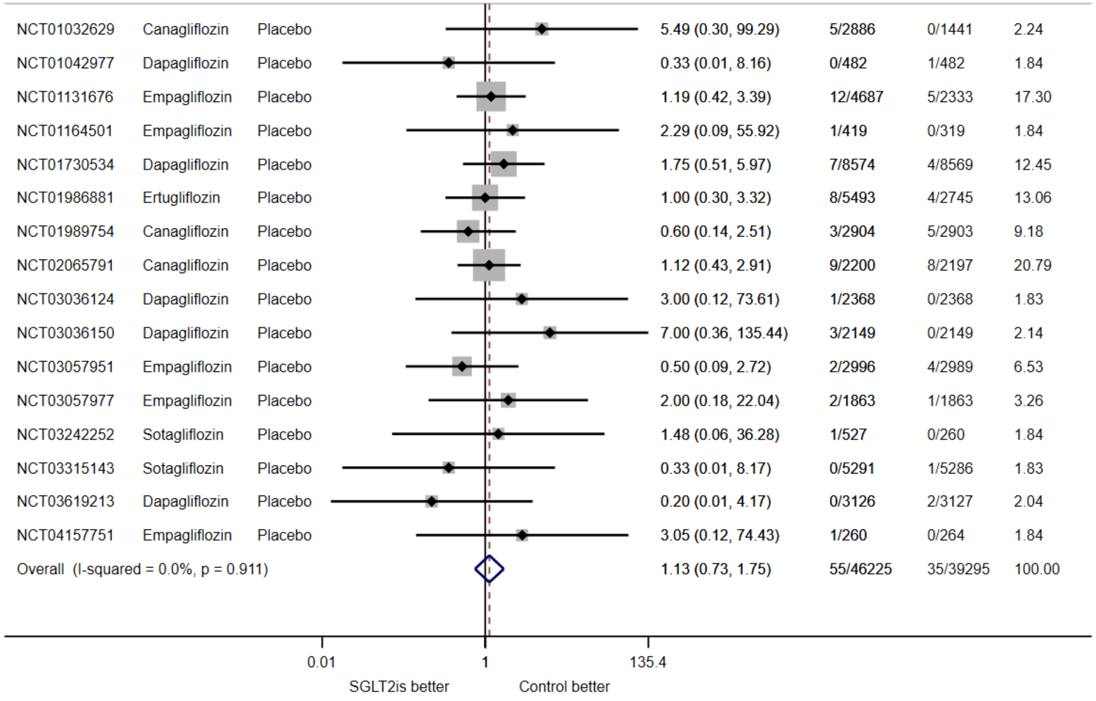

System 25-Vascular Disorders-Figure S18 Meta-analysis of SGLT2is and Extremity necrosis

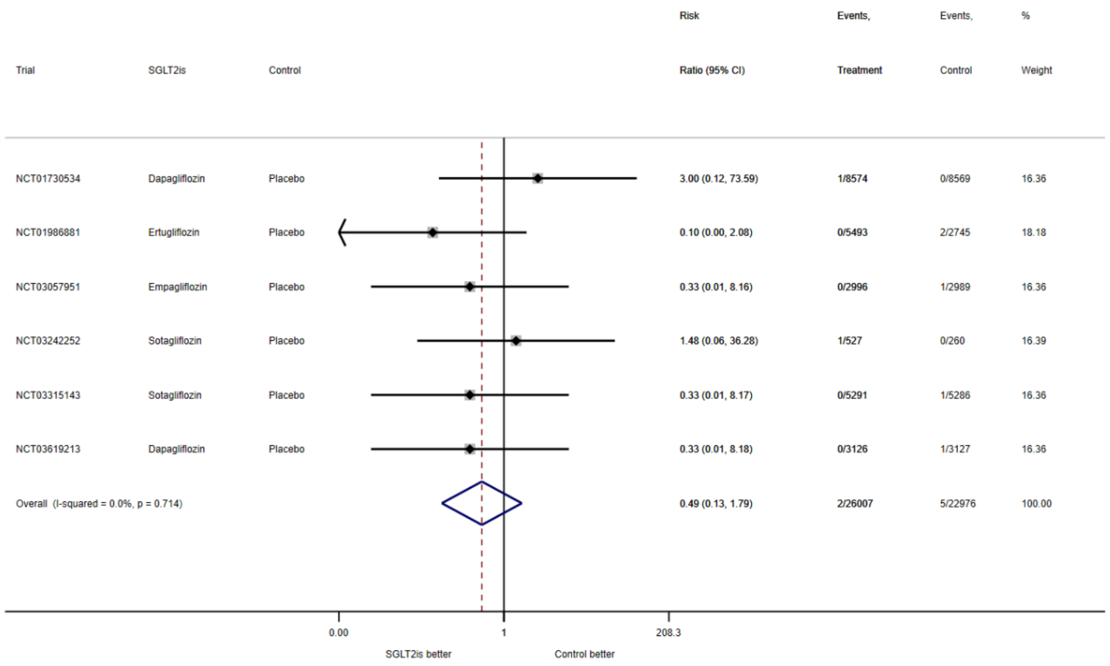

System 25-Vascular Disorders-Figure S19 Meta-analysis of SGLT2is and Femoral artery aneurysm

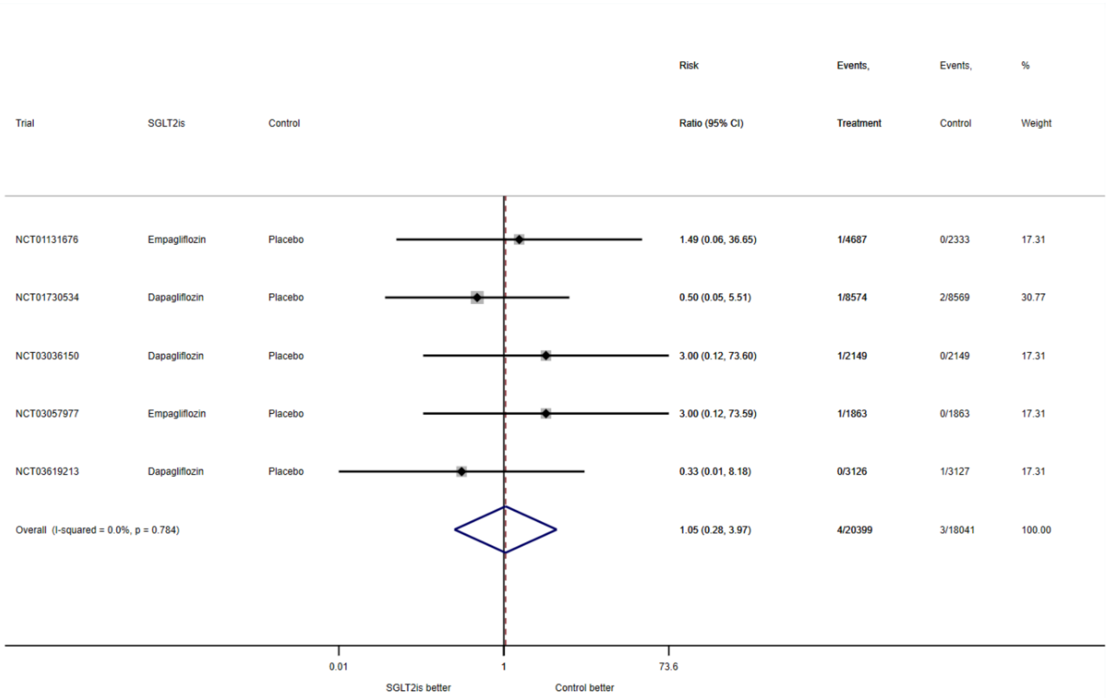

System 25-Vascular Disorders-Figure S20 Meta-analysis of SGLT2is and Femoral artery embolism

Trial	SGLT2is	Control	Risk Ratio (95% CI)	Events, Treatment	Events, Control	% Weight
NCT01032629	Canagliflozin	Placebo	1.00 (0.09, 11.00)	2/2886	1/1441	6.61
NCT01106651	Canagliflozin	Placebo	1.49 (0.06, 36.53)	1/477	0/237	3.72
NCT01131676	Empagliflozin	Placebo	3.49 (0.18, 67.44)	3/4687	0/2333	4.33
NCT01730534	Dapagliflozin	Placebo	0.33 (0.07, 1.65)	2/8574	6/8569	14.86
NCT01986881	Ertugliflozin	Placebo	4.50 (0.24, 83.52)	4/5493	0/2745	4.46
NCT01989754	Canagliflozin	Placebo	1.00 (0.06, 15.97)	1/2904	1/2903	4.95
NCT02065791	Canagliflozin	Placebo	2.00 (0.18, 22.01)	2/2200	1/2197	6.61
NCT03036124	Dapagliflozin	Placebo	0.20 (0.01, 4.16)	0/2368	2/2368	4.13
NCT03036150	Dapagliflozin	Placebo	0.33 (0.01, 8.18)	0/2149	1/2149	3.72
NCT03057951	Empagliflozin	Placebo	2.00 (0.50, 7.97)	6/2996	3/2989	19.84
NCT03594110	Empagliflozin	Placebo	1.00 (0.20, 4.95)	3/3304	3/3305	14.87
NCT03619213	Dapagliflozin	Placebo	0.67 (0.11, 3.99)	2/3126	3/3127	11.90
Overall (I-squared = 0.0%, p = 0.832)			1.00 (0.54, 1.85)	26/41164	21/34363	100.00

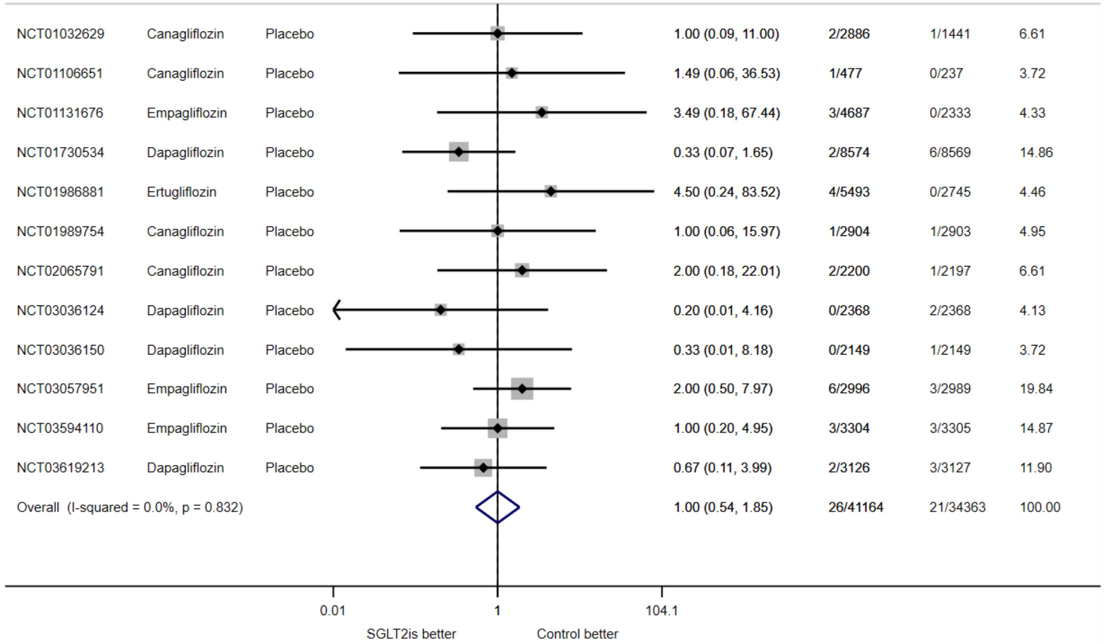

System 25-Vascular Disorders-Figure S21 Meta-analysis of SGLT2is and Haematoma

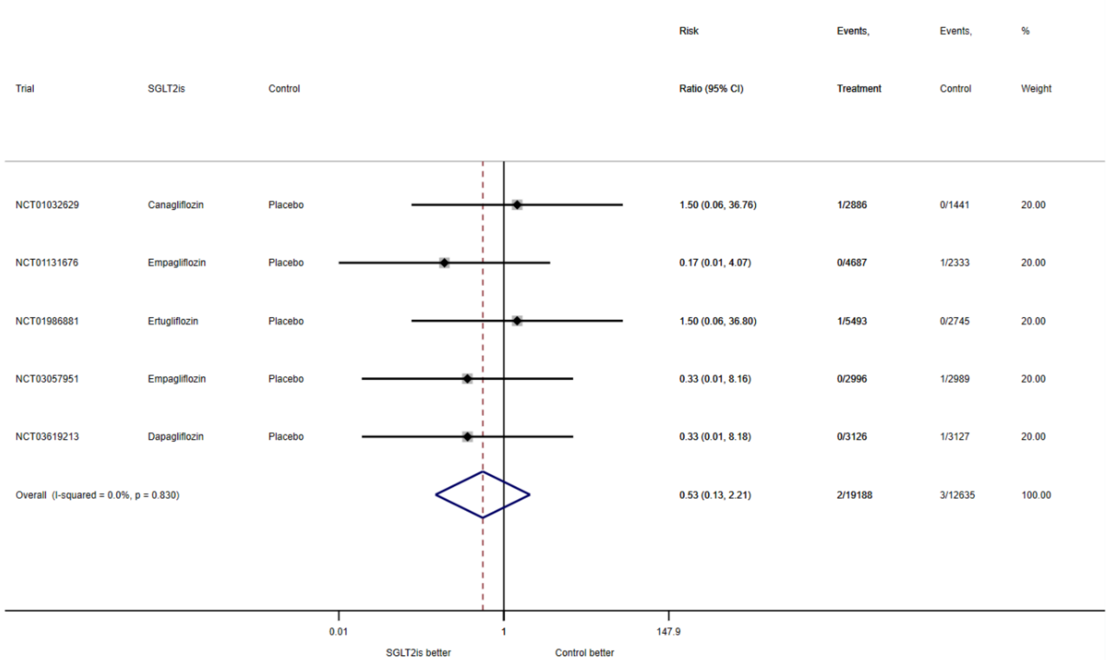

System 25-Vascular Disorders-Figure S22 Meta-analysis of SGLT2is and Haemorrhage

Trial	SGLT2is	Control	Risk Ratio (95% CI)	Events, Treatment	Events, Control	% Weight
NCT00673231	Dapagliflozin	Placebo	0.97 (0.04, 23.77)	1/610	0/197	0.67
NCT01031680	Dapagliflozin	Placebo	0.33 (0.01, 8.20)	0/460	1/462	0.67
NCT01032629	Canagliflozin	Placebo	0.25 (0.09, 0.73)	5/2886	10/1441	5.94
NCT01131676	Empagliflozin	Placebo	0.55 (0.23, 1.29)	11/4687	10/2333	9.34
NCT01164501	Empagliflozin	Placebo	0.15 (0.01, 3.16)	0/419	2/319	0.74
NCT01730534	Dapagliflozin	Placebo	1.00 (0.59, 1.70)	27/8574	27/8569	24.06
NCT01986881	Ertugliflozin	Placebo	0.32 (0.12, 0.82)	7/5493	11/2745	7.62
NCT01989754	Canagliflozin	Placebo	0.25 (0.03, 2.23)	1/2904	4/2903	1.42
NCT02033889	Ertugliflozin	Placebo/Glimepiride	0.17 (0.01, 4.14)	0/412	1/209	0.67
NCT02065791	Canagliflozin	Placebo	1.00 (0.35, 2.84)	7/2200	7/2197	6.24
NCT03036124	Dapagliflozin	Placebo	1.00 (0.14, 7.09)	2/2368	2/2368	1.78
NCT03036150	Dapagliflozin	Placebo	0.90 (0.37, 2.21)	9/2149	10/2149	8.45
NCT03057951	Empagliflozin	Placebo	0.67 (0.27, 1.62)	8/2996	12/2989	8.55
NCT03057977	Empagliflozin	Placebo	0.33 (0.01, 8.18)	0/1863	1/1863	0.67
NCT03315143	Sotagliflozin	Placebo	0.61 (0.26, 1.48)	8/5291	13/5286	8.81
NCT03521934	Sotagliflozin	Placebo	0.20 (0.01, 4.20)	0/605	2/611	0.74
NCT03594110	Empagliflozin	Placebo	0.38 (0.10, 1.41)	3/3304	8/3305	3.88
NCT03619213	Dapagliflozin	Placebo	1.56 (0.67, 3.59)	14/3126	9/3127	9.76
Overall (I-squared = 1.3%, p = 0.439)			0.68 (0.53, 0.89)	103/50347	130/43073	100.00

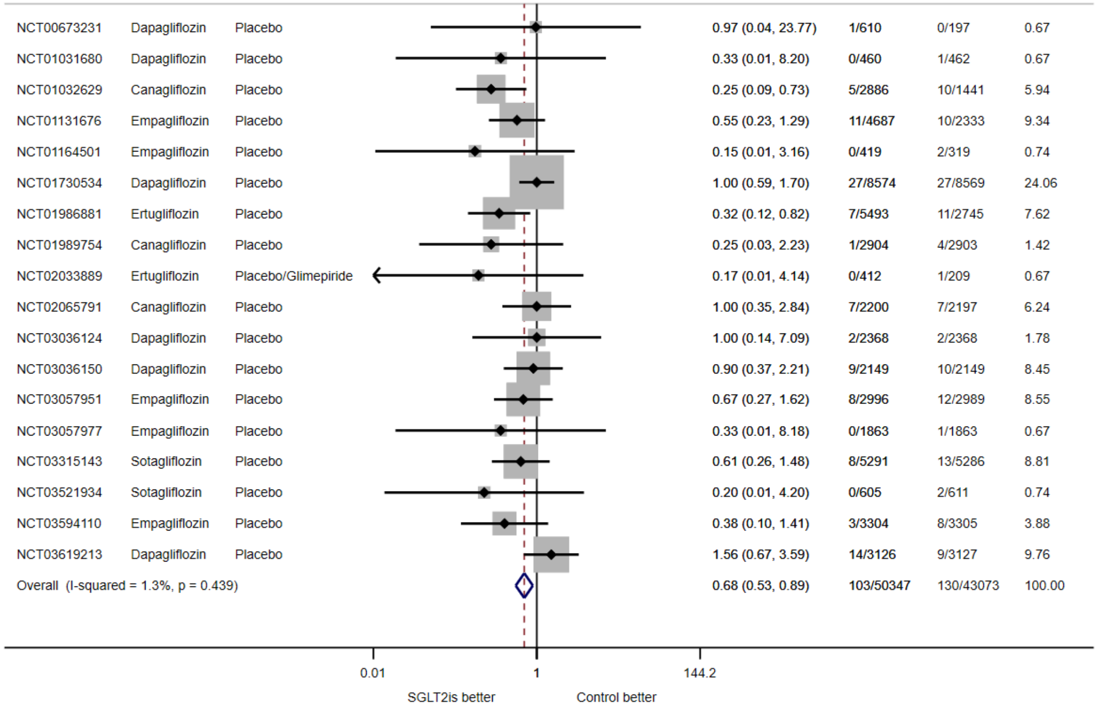

System 25-Vascular Disorders-Figure S23 Meta-analysis of SGLT2is and Hypertension

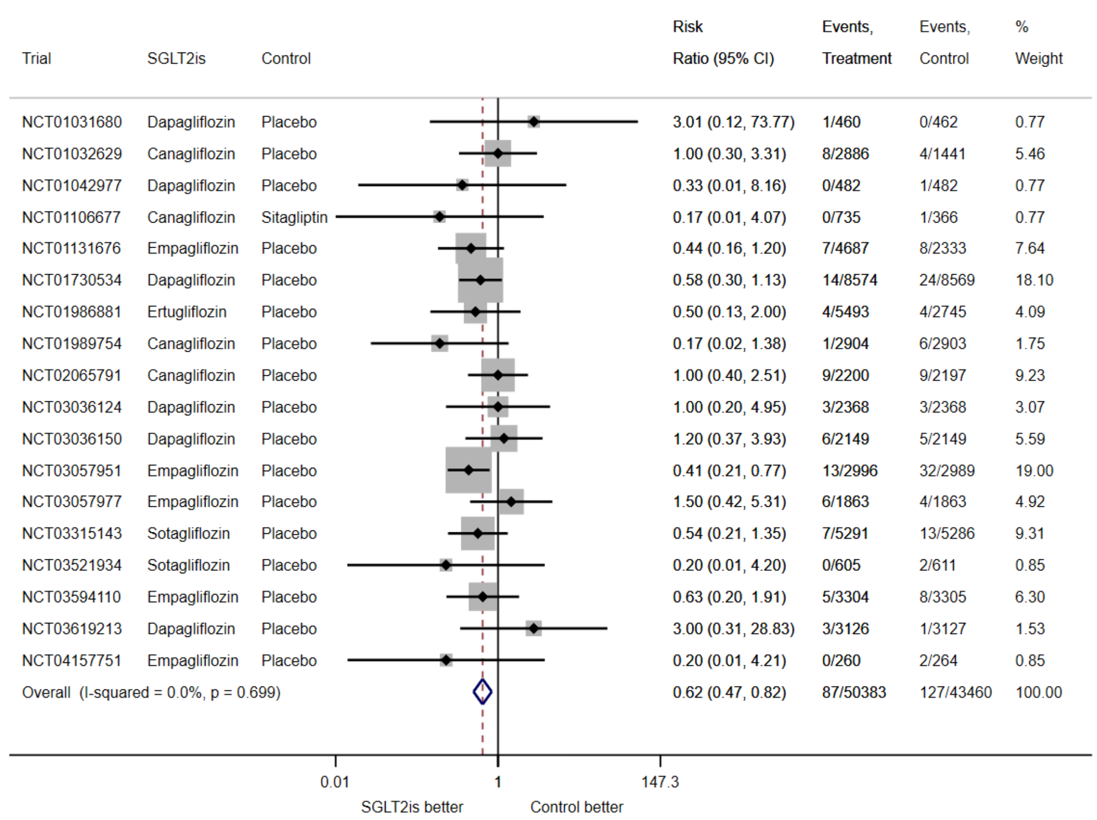

System 25-Vascular Disorders-Figure S24 Meta-analysis of SGLT2is and Hypertensive crisis

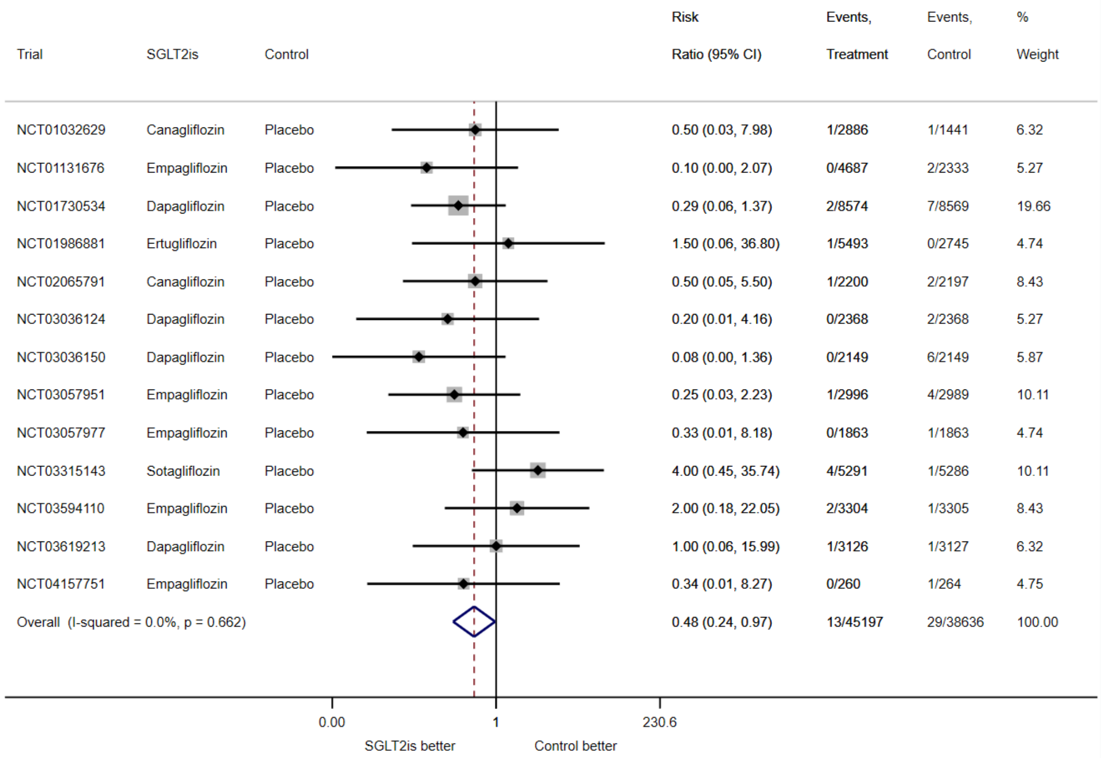

System 25-Vascular Disorders-Figure S25 Meta-analysis of SGLT2is and Hypertensive emergency

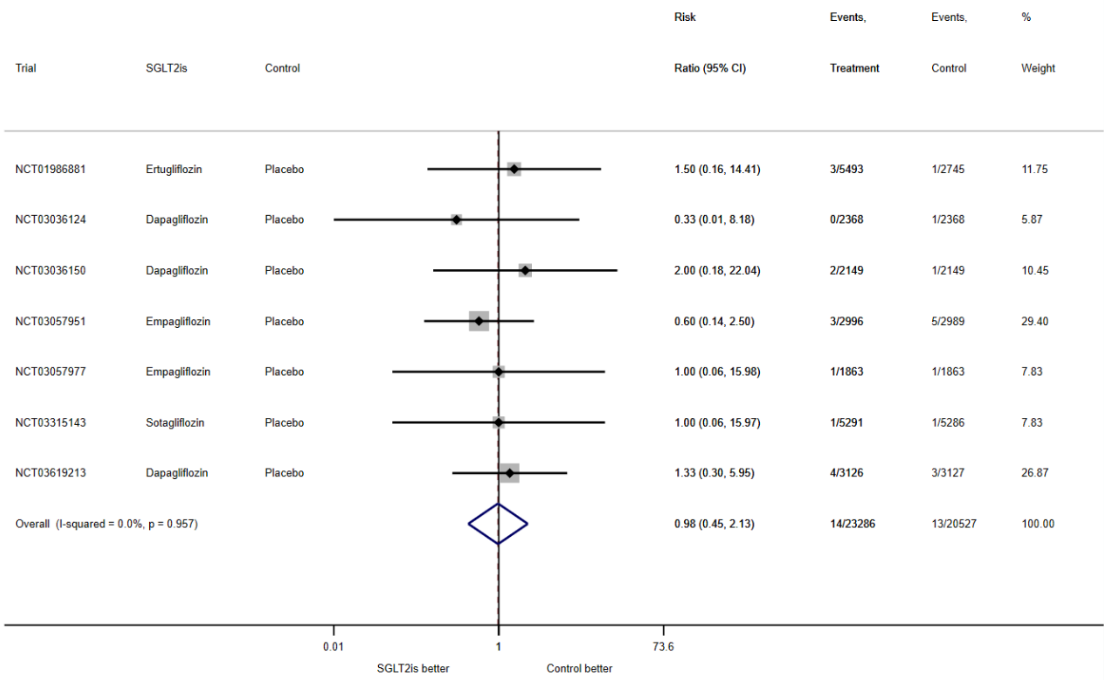

System 25-Vascular Disorders-Figure S26 Meta-analysis of SGLT2is and Hypertensive urgency

Trial	SGLT2is	Control	Risk Ratio (95% CI)	Events, Treatment	Events, Control	% Weight
NCT01032629	Canagliflozin	Placebo	1.33 (0.35, 5.01)	8/2886	3/1441	3.90
NCT01131676	Empagliflozin	Placebo	0.50 (0.20, 1.25)	9/4687	9/2333	8.05
NCT01164501	Empagliflozin	Placebo	0.25 (0.01, 6.21)	0/419	1/319	0.67
NCT01730534	Dapagliflozin	Placebo	2.00 (1.05, 3.79)	28/8574	14/8569	16.69
NCT01986881	Ertugliflozin	Placebo	0.30 (0.11, 0.82)	6/5493	10/2745	6.71
NCT01989754	Canagliflozin	Placebo	0.14 (0.01, 2.76)	0/2904	3/2903	0.78
NCT02065791	Canagliflozin	Placebo	1.33 (0.30, 5.94)	4/2200	3/2197	3.06
NCT03036124	Dapagliflozin	Placebo	0.64 (0.25, 1.64)	7/2368	11/2368	7.66
NCT03036150	Dapagliflozin	Placebo	2.67 (0.71, 10.04)	8/2149	3/2149	3.90
NCT03057951	Empagliflozin	Placebo	1.37 (0.76, 2.46)	26/2996	19/2989	19.74
NCT03057977	Empagliflozin	Placebo	1.08 (0.51, 2.28)	14/1863	13/1863	12.12
NCT03242252	Sotagliflozin	Placebo	1.48 (0.06, 36.28)	1/527	0/260	0.67
NCT03315143	Sotagliflozin	Placebo	0.71 (0.23, 2.25)	5/5291	7/5286	5.21
NCT03521934	Sotagliflozin	Placebo	6.06 (0.73, 50.18)	6/605	1/611	1.53
NCT03619213	Dapagliflozin	Placebo	1.67 (0.61, 4.58)	10/3126	6/3127	6.71
NCT04157751	Empagliflozin	Placebo	0.41 (0.08, 2.07)	2/260	5/264	2.58
Overall (I-squared = 37.4%, p = 0.066)			1.07 (0.83, 1.40)	134/46348	108/39424	100.00

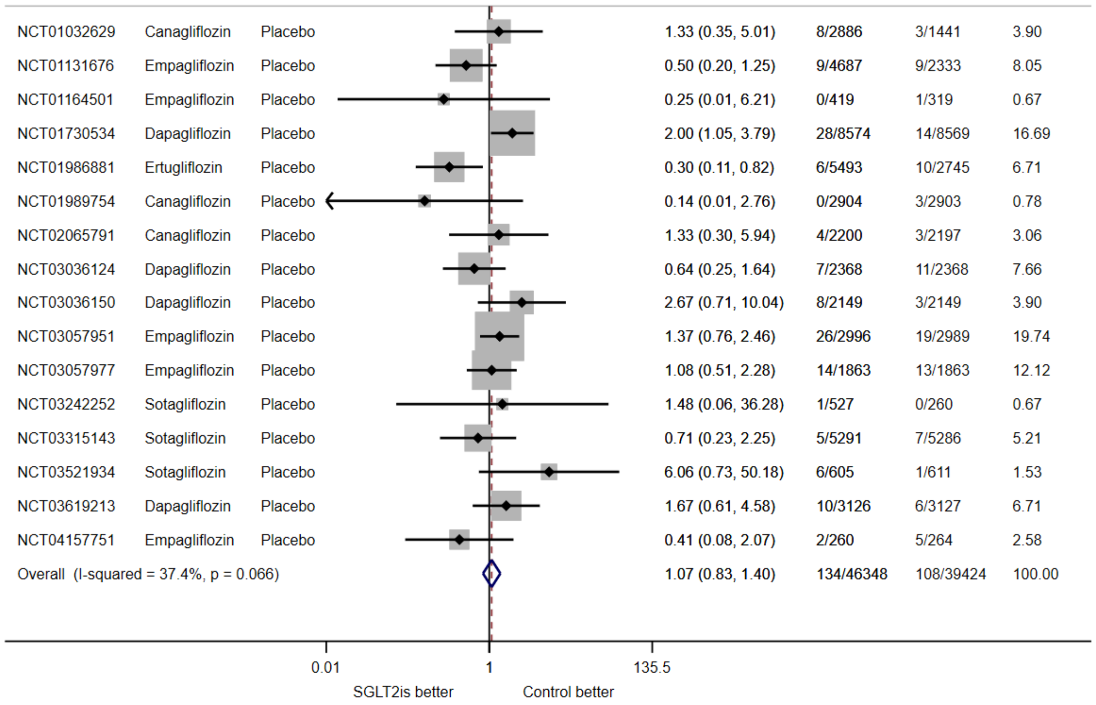

System 25-Vascular Disorders-Figure S27 Meta-analysis of SGLT2is and Hypotension

Trial	SGLT2is	Control	Risk Ratio (95% CI)	Events, Treatment	Events, Control	% Weight
NCT01131676	Empagliflozin	Placebo	0.25 (0.02, 2.74)	1/4687	2/2333	9.34
NCT01730534	Dapagliflozin	Placebo	0.50 (0.05, 5.51)	1/8574	2/8569	9.34
NCT01986881	Ertugliflozin	Placebo	2.00 (0.22, 17.88)	4/5493	1/2745	11.21
NCT01989754	Canagliflozin	Placebo	3.00 (0.12, 73.59)	1/2904	0/2903	5.25
NCT03036124	Dapagliflozin	Placebo	0.33 (0.01, 8.18)	0/2368	1/2368	5.26
NCT03036150	Dapagliflozin	Placebo	0.33 (0.01, 8.18)	0/2149	1/2149	5.26
NCT03057951	Empagliflozin	Placebo	0.33 (0.07, 1.65)	2/2996	6/2989	21.04
NCT03057977	Empagliflozin	Placebo	0.33 (0.01, 8.18)	0/1863	1/1863	5.26
NCT03315143	Sotagliflozin	Placebo	3.00 (0.12, 73.56)	1/5291	0/5286	5.25
NCT03521934	Sotagliflozin	Placebo	0.34 (0.01, 8.25)	0/605	1/611	5.26
NCT03594110	Empagliflozin	Placebo	1.00 (0.06, 15.99)	1/3304	1/3305	7.01
NCT03619213	Dapagliflozin	Placebo	0.33 (0.01, 8.18)	0/3126	1/3127	5.25
NCT04157751	Empagliflozin	Placebo	0.34 (0.01, 8.27)	0/260	1/264	5.27
Overall (I-squared = 0.0%, p = 0.957)			0.56 (0.27, 1.17)	11/43620	18/38512	100.00

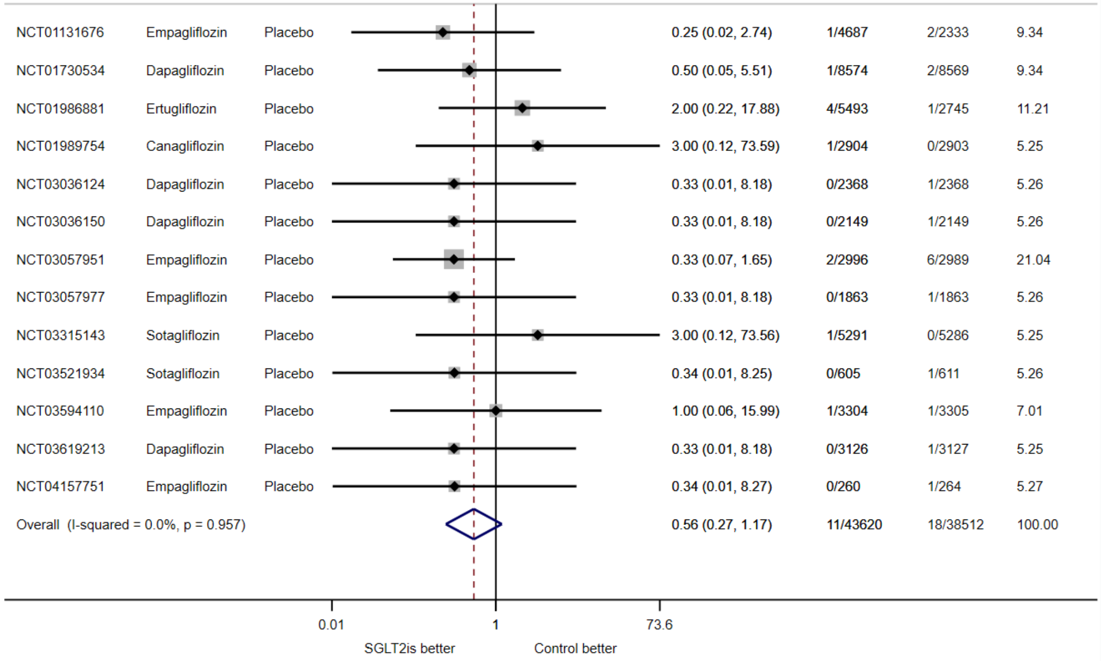

System 25-Vascular Disorders-Figure S28 Meta-analysis of SGLT2is and Hypovolaemic shock

Trial	SGLT2is	Control	Risk Ratio (95% CI)	Events, Treatment	Events, Control	% Weight
NCT01032629	Canagliflozin	Placebo	1.50 (0.06, 36.76)	1/2886	0/1441	9.02
NCT01131676	Empagliflozin	Placebo	1.00 (0.09, 10.97)	2/4687	1/2333	16.03
NCT01730534	Dapagliflozin	Placebo	1.50 (0.25, 8.97)	3/8574	2/8569	28.86
NCT01986881	Ertugliflozin	Placebo	0.10 (0.00, 2.08)	0/5493	2/2745	10.02
NCT01989754	Canagliflozin	Placebo	3.00 (0.12, 73.59)	1/2904	0/2903	9.02
NCT03036124	Dapagliflozin	Placebo	0.33 (0.01, 8.18)	0/2368	1/2368	9.02
NCT03057951	Empagliflozin	Placebo	0.33 (0.01, 8.16)	0/2996	1/2989	9.02
NCT03315143	Sotagliflozin	Placebo	0.33 (0.01, 8.17)	0/5291	1/5286	9.02
Overall (I-squared = 0.0%, p = 0.784)			0.76 (0.29, 1.98)	7/35199	8/28634	100.00

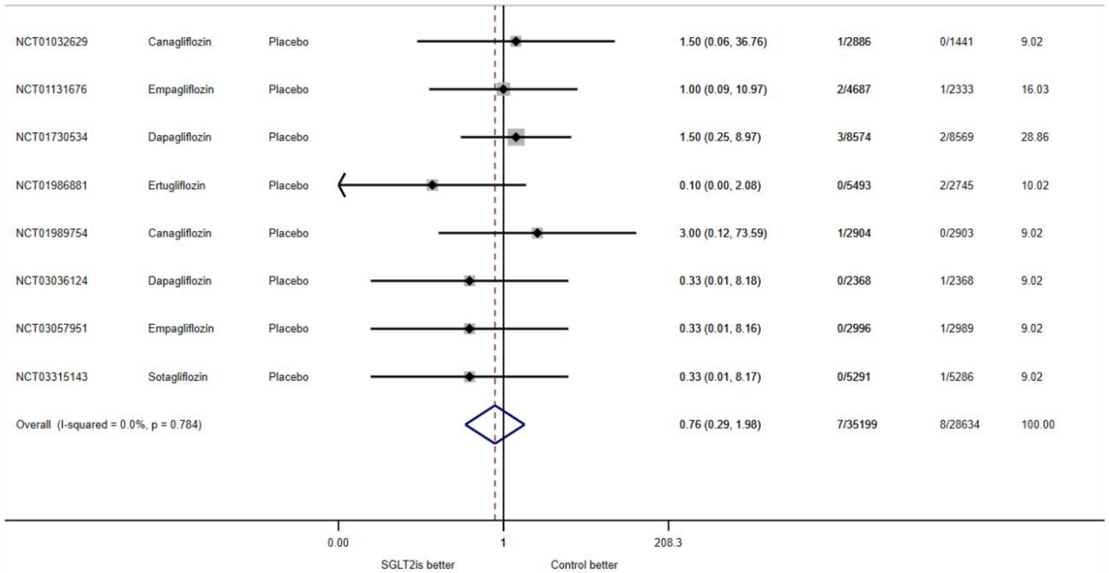

System 25-Vascular Disorders-Figure S29 Meta-analysis of SGLT2is and Iliac artery occlusion

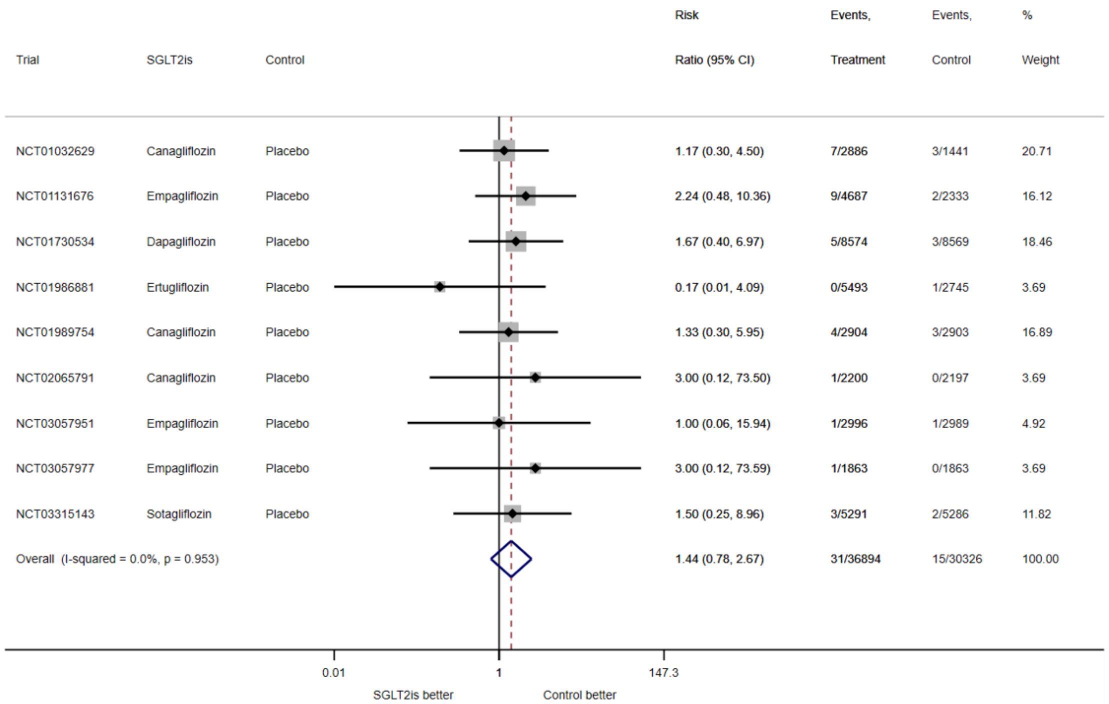

System 25-Vascular Disorders-Figure S30 Meta-analysis of SGLT2is and Intermittent claudication

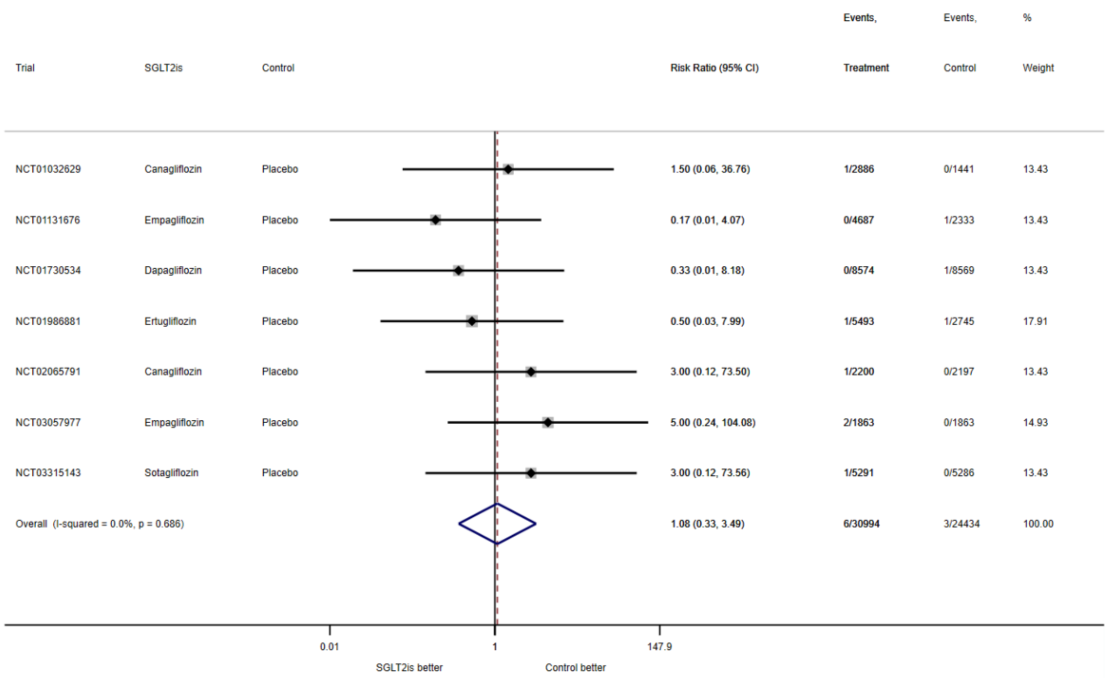

System 25-Vascular Disorders-Figure S31 Meta-analysis of SGLT2is and Leriche syndrome

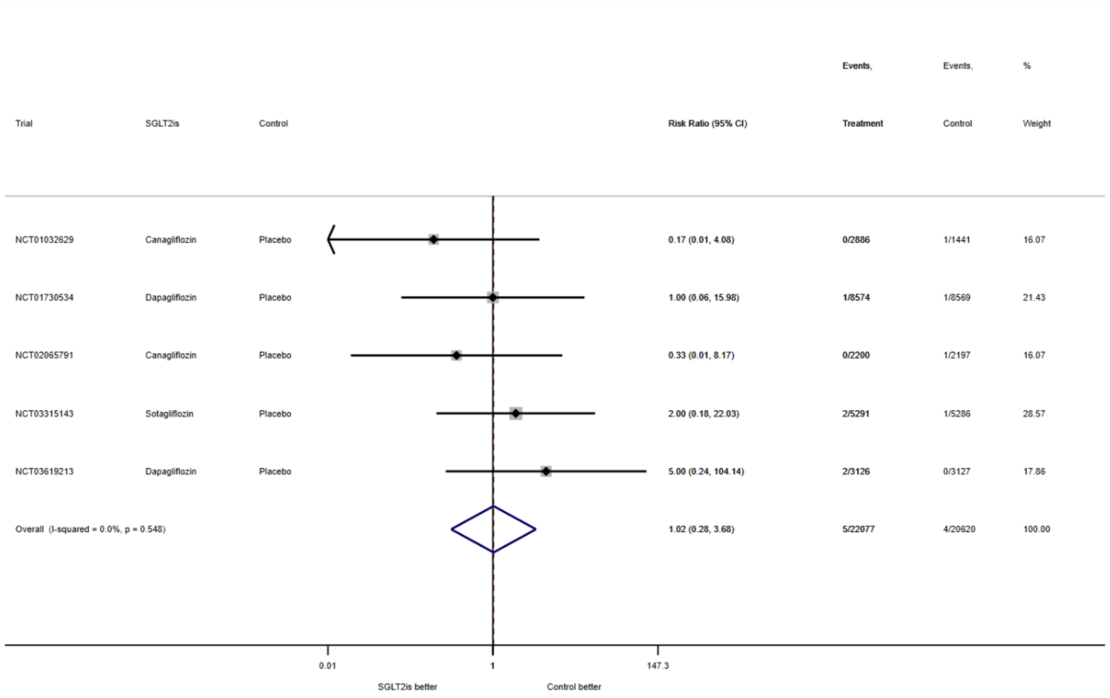

System 25-Vascular Disorders-Figure S32 Meta-analysis of SGLT2is and Lymphoedema

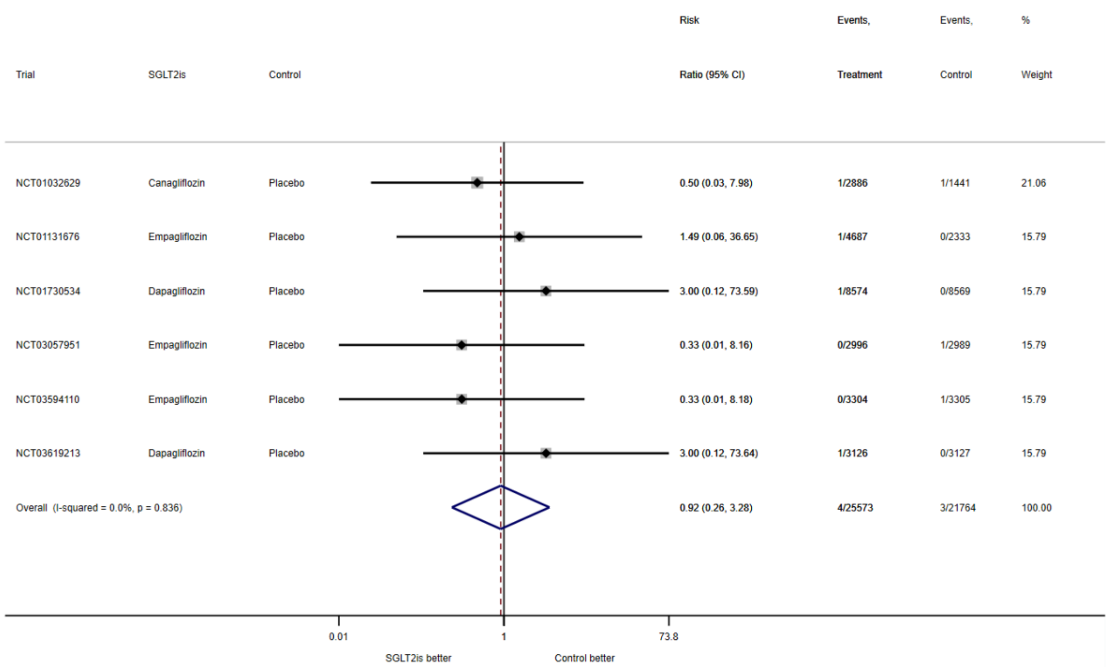

System 25-Vascular Disorders-Figure S33 Meta-analysis of SGLT2is and Malignant hypertension

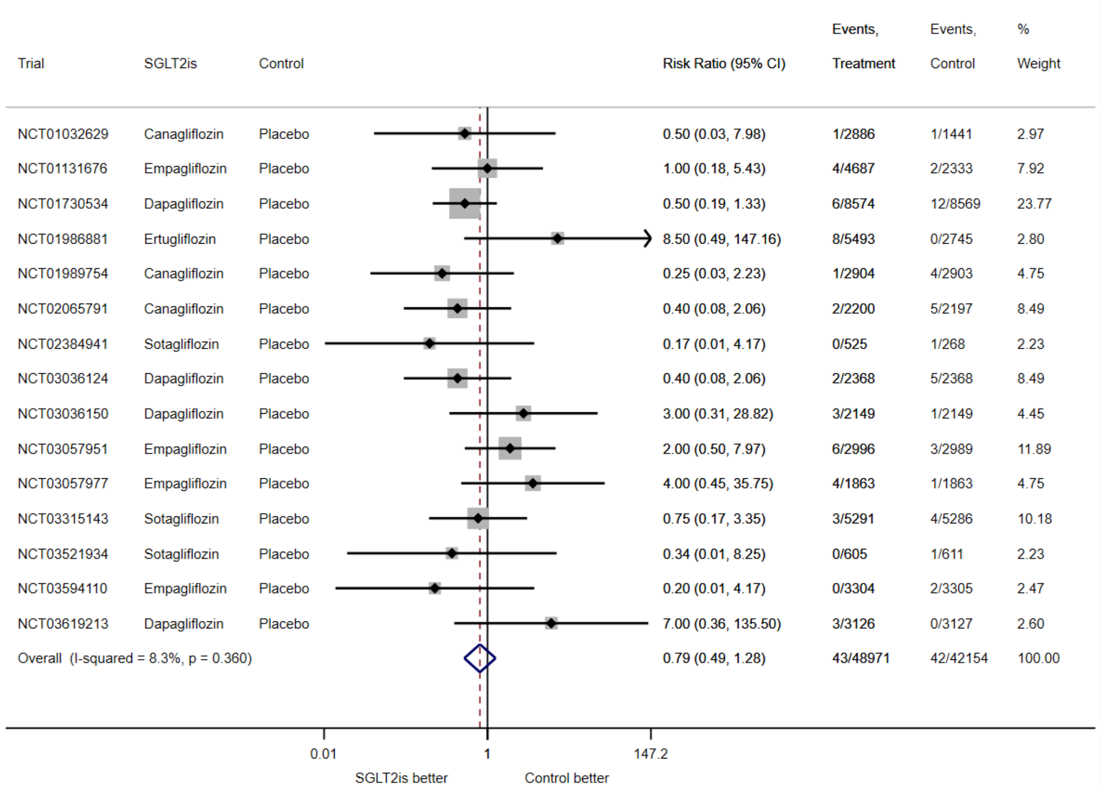

System 25-Vascular Disorders-Figure S34 Meta-analysis of SGLT2is and Orthostatic hypotension

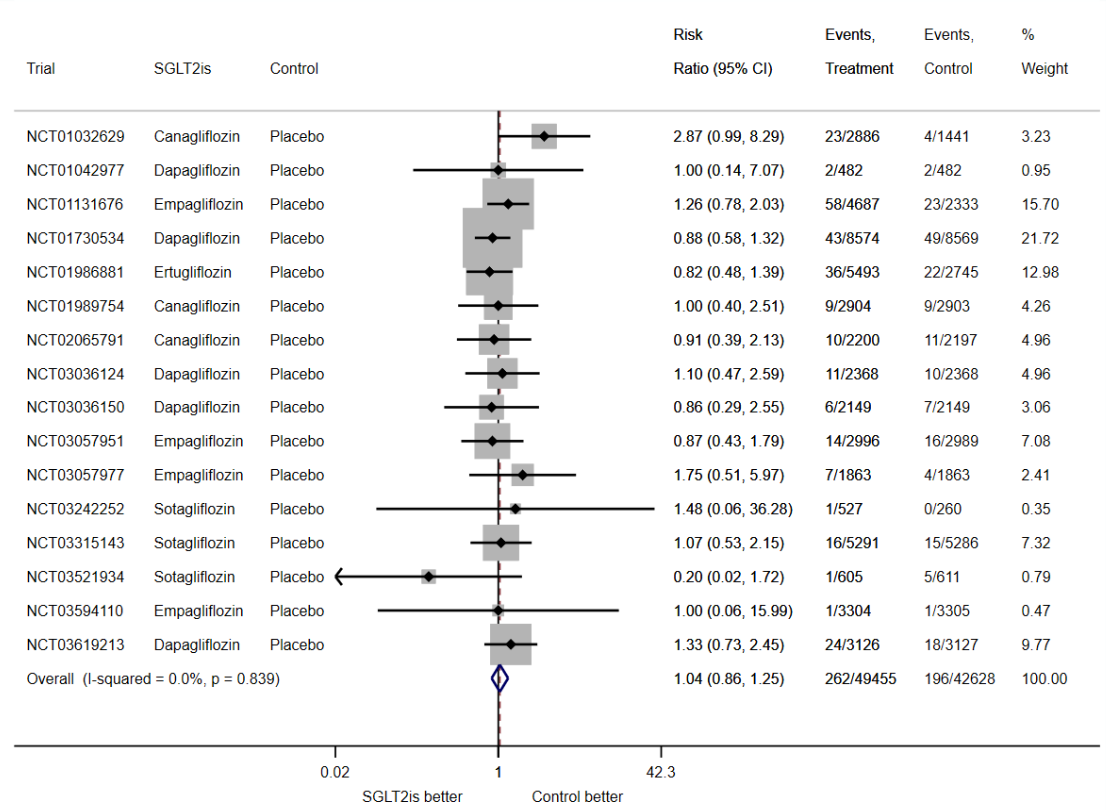

System 25-Vascular Disorders-Figure S35 Meta-analysis of SGLT2is and Peripheral arterial occlusive disease

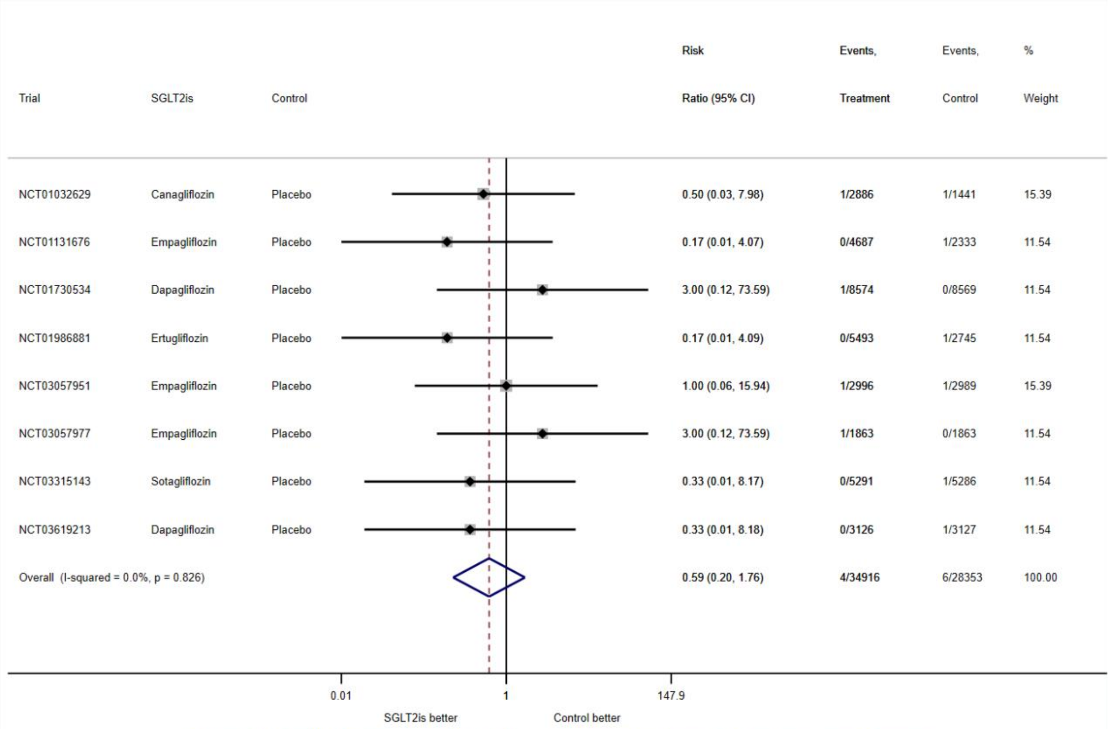

System 25-Vascular Disorders-Figure S36 Meta-analysis of SGLT2is and Peripheral artery aneurysm

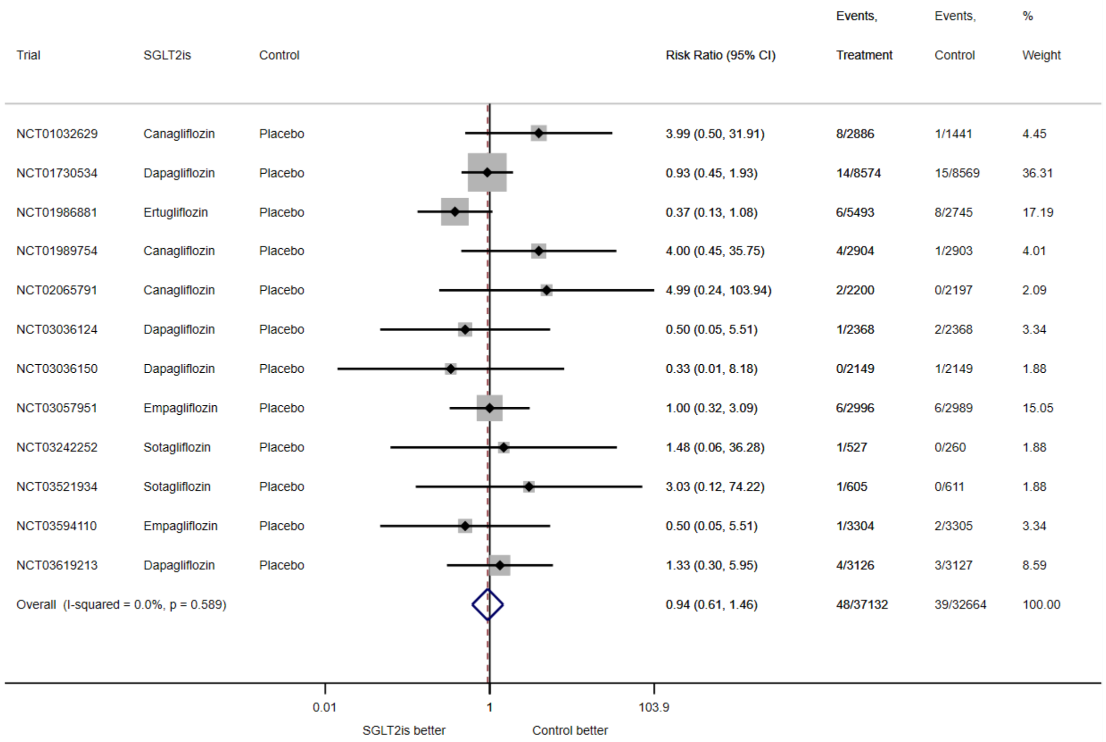

System 25-Vascular Disorders-Figure S37 Meta-analysis of SGLT2is and Peripheral artery occlusion

Trial	SGLT2is	Control	Risk Ratio (95% CI)	Events, Treatment	Events, Control	% Weight
NCT01032629	Canagliflozin	Placebo	1.50 (0.48, 4.64)	12/2886	4/1441	10.83
NCT01131676	Empagliflozin	Placebo	1.00 (0.37, 2.65)	12/4687	6/2333	14.43
NCT01730534	Dapagliflozin	Placebo	1.15 (0.63, 2.09)	23/8574	20/8569	38.59
NCT01986881	Ertugliflozin	Placebo	0.60 (0.18, 1.96)	6/5493	5/2745	9.83
NCT01989754	Canagliflozin	Placebo	4.00 (0.85, 18.81)	8/2904	2/2903	5.76
NCT03036124	Dapagliflozin	Placebo	3.00 (0.12, 73.61)	1/2368	0/2368	1.35
NCT03036150	Dapagliflozin	Placebo	3.00 (0.31, 28.82)	3/2149	1/2149	2.70
NCT03057951	Empagliflozin	Placebo	0.67 (0.11, 3.98)	2/2996	3/2989	4.32
NCT03057977	Empagliflozin	Placebo	1.00 (0.06, 15.98)	1/1863	1/1863	1.80
NCT03242252	Sotagliflozin	Placebo	2.47 (0.12, 51.30)	2/527	0/260	1.50
NCT03315143	Sotagliflozin	Placebo	0.25 (0.03, 2.23)	1/5291	4/5286	2.88
NCT03594110	Empagliflozin	Placebo	0.50 (0.05, 5.51)	1/3304	2/3305	2.40
NCT03619213	Dapagliflozin	Placebo	1.00 (0.14, 7.10)	2/3126	2/3127	3.60
Overall (I-squared = 0.0%, p = 0.792)			1.12 (0.77, 1.62)	74/46168	50/39338	100.00

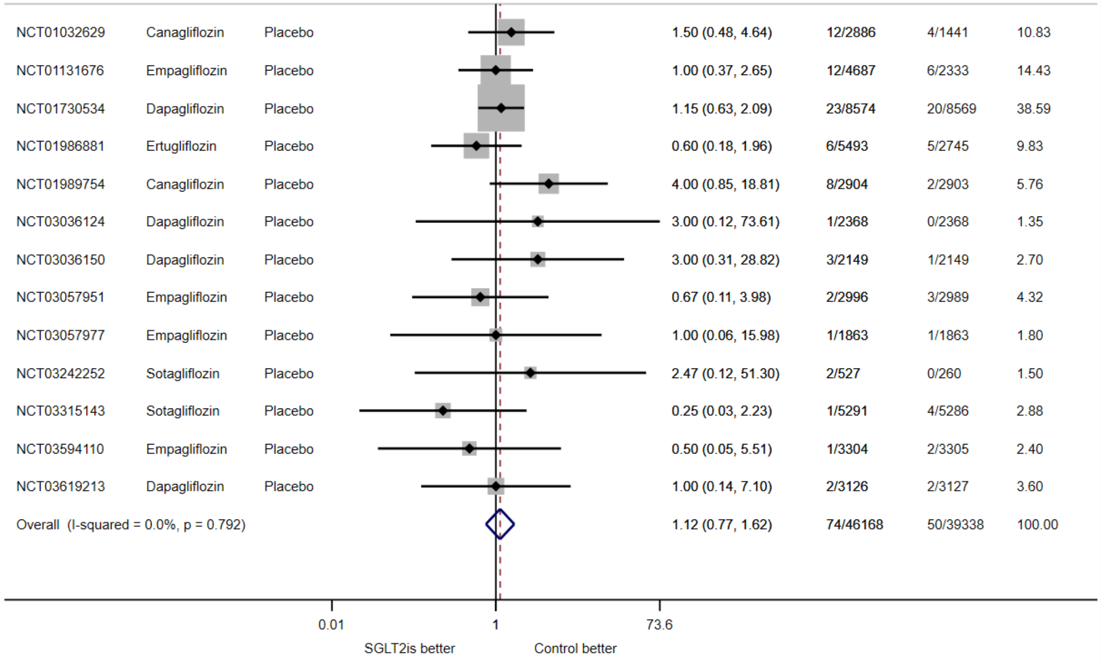

System 25-Vascular Disorders-Figure S38 Meta-analysis of SGLT2is and Peripheral artery stenosis

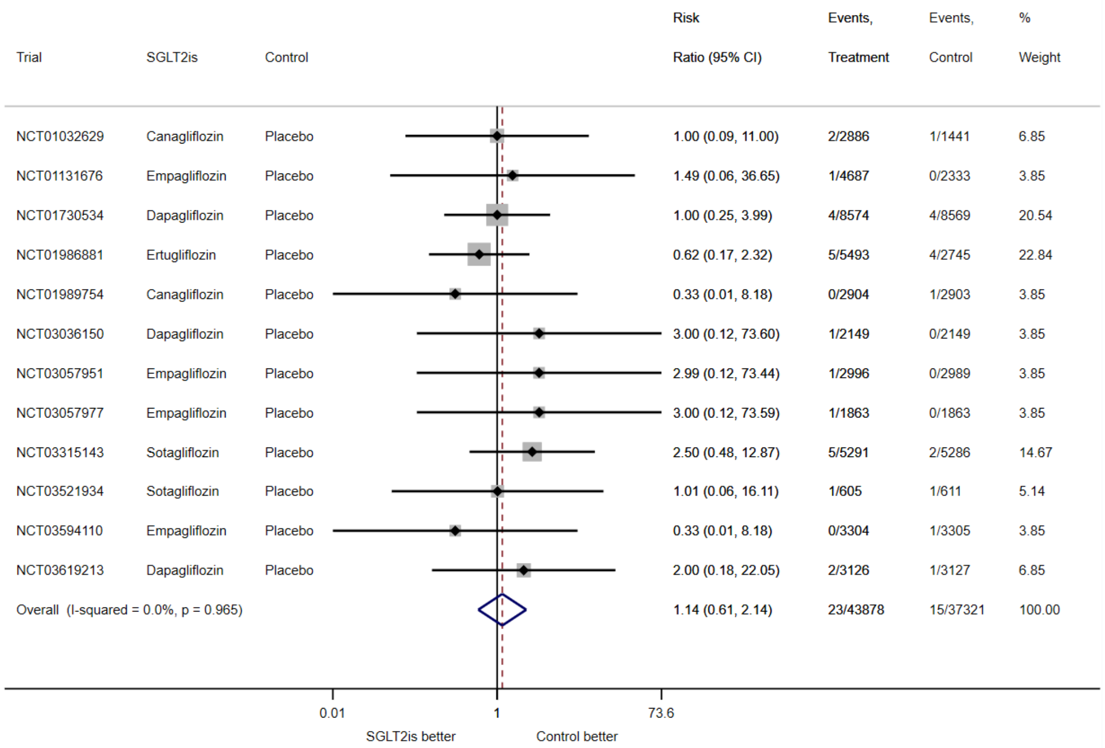

System 25-Vascular Disorders-Figure S39 Meta-analysis of SGLT2is and Peripheral artery thrombosis

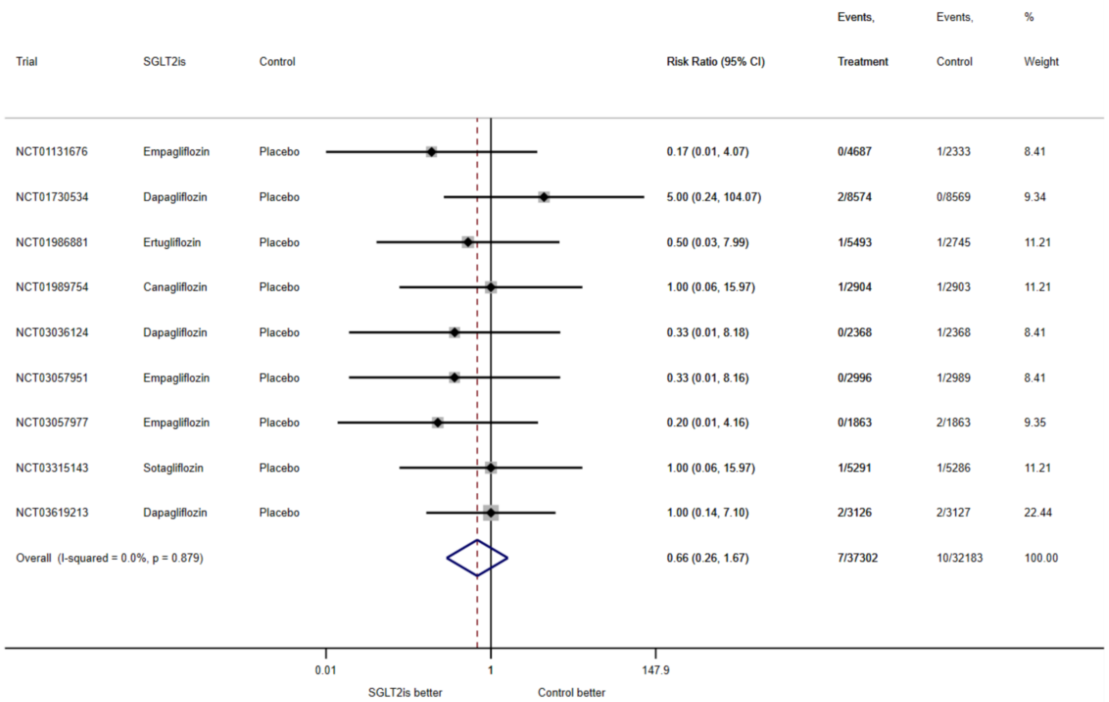

System 25-Vascular Disorders-Figure S40 Meta-analysis of SGLT2is and Peripheral embolism

Trial	SGLT2is	Control	Risk Ratio (95% CI)	Events, Treatment	Events, Control	% Weight
NCT01032629	Canagliflozin	Placebo	0.83 (0.37, 1.90)	15/2886	9/1441	9.45
NCT01042977	Dapagliflozin	Placebo	0.33 (0.01, 8.16)	0/482	1/482	0.63
NCT01131676	Empagliflozin	Placebo	0.83 (0.36, 1.89)	15/4687	9/2333	9.43
NCT01164501	Empagliflozin	Placebo	2.29 (0.09, 55.92)	1/419	0/319	0.63
NCT01730534	Dapagliflozin	Placebo	0.86 (0.53, 1.39)	30/8574	35/8569	27.08
NCT01986881	Ertugliflozin	Placebo	1.50 (0.73, 3.06)	30/5493	10/2745	12.58
NCT01989754	Canagliflozin	Placebo	1.40 (0.44, 4.40)	7/2904	5/2903	4.88
NCT02033889	Ertugliflozin	Placebo/Glimepiride	2.54 (0.12, 52.72)	2/412	0/209	0.70
NCT02065791	Canagliflozin	Placebo	0.60 (0.14, 2.50)	3/2200	5/2197	3.14
NCT03036124	Dapagliflozin	Placebo	1.80 (0.60, 5.36)	9/2368	5/2368	5.38
NCT03036150	Dapagliflozin	Placebo	0.50 (0.15, 1.66)	4/2149	8/2149	4.47
NCT03057951	Empagliflozin	Placebo	1.40 (0.44, 4.40)	7/2996	5/2989	4.88
NCT03057977	Empagliflozin	Placebo	1.25 (0.34, 4.65)	5/1863	4/1863	3.72
NCT03242252	Sotagliflozin	Placebo	2.47 (0.12, 51.30)	2/527	0/260	0.70
NCT03315143	Sotagliflozin	Placebo	1.60 (0.52, 4.88)	8/5291	5/5286	5.15
NCT03521934	Sotagliflozin	Placebo	5.05 (0.24, 104.96)	2/605	0/611	0.70
NCT03594110	Empagliflozin	Placebo	0.50 (0.05, 5.51)	1/3304	2/3305	1.11
NCT03619213	Dapagliflozin	Placebo	0.56 (0.19, 1.66)	5/3126	9/3127	5.38
Overall (I-squared = 0.0%, p = 0.891)			1.01 (0.78, 1.30)	146/50286	112/43156	100.00

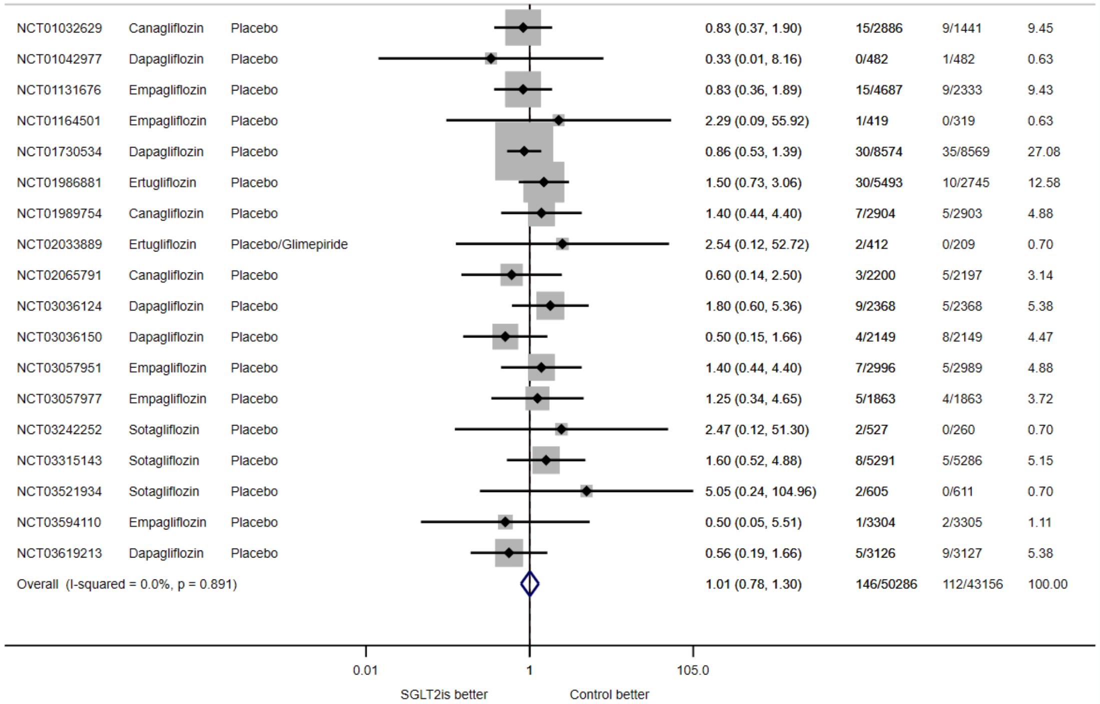

System 25-Vascular Disorders-Figure S41 Meta-analysis of SGLT2is and Peripheral ischaemia

Trial	SGLT2is	Control	Risk Ratio (95% CI)	Events, Treatment	Events, Control	% Weight
NCT01032629	Canagliflozin	Placebo	1.28 (0.54, 3.07)	18/2886	7/1441	15.74
NCT01106651	Canagliflozin	Placebo	1.49 (0.06, 36.53)	1/477	0/237	1.17
NCT01131676	Empagliflozin	Placebo	0.56 (0.22, 1.45)	9/4687	8/2333	13.19
NCT01730534	Dapagliflozin	Placebo	1.79 (1.02, 3.13)	34/8574	19/8569	37.96
NCT01986881	Ertugliflozin	Placebo	0.87 (0.26, 2.98)	7/5493	4/2745	7.92
NCT01989754	Canagliflozin	Placebo	2.25 (0.69, 7.30)	9/2904	4/2903	8.62
NCT02065791	Canagliflozin	Placebo	4.99 (0.58, 42.70)	5/2200	1/2197	2.59
NCT03036124	Dapagliflozin	Placebo	3.00 (0.12, 73.61)	1/2368	0/2368	1.17
NCT03036150	Dapagliflozin	Placebo	1.00 (0.06, 15.98)	1/2149	1/2149	1.55
NCT03057951	Empagliflozin	Placebo	2.00 (0.18, 21.99)	2/2996	1/2989	2.07
NCT03057977	Empagliflozin	Placebo	3.00 (0.12, 73.59)	1/1863	0/1863	1.17
NCT03315143	Sotagliflozin	Placebo	1.00 (0.06, 15.97)	1/5291	1/5286	1.55
NCT03594110	Empagliflozin	Placebo	2.00 (0.37, 10.92)	4/3304	2/3305	4.14
NCT03619213	Dapagliflozin	Placebo	0.33 (0.01, 8.18)	0/3126	1/3127	1.16
Overall (I-squared = 0.0%, p = 0.813)			1.41 (1.00, 1.99)	93/48318	49/41512	100.00

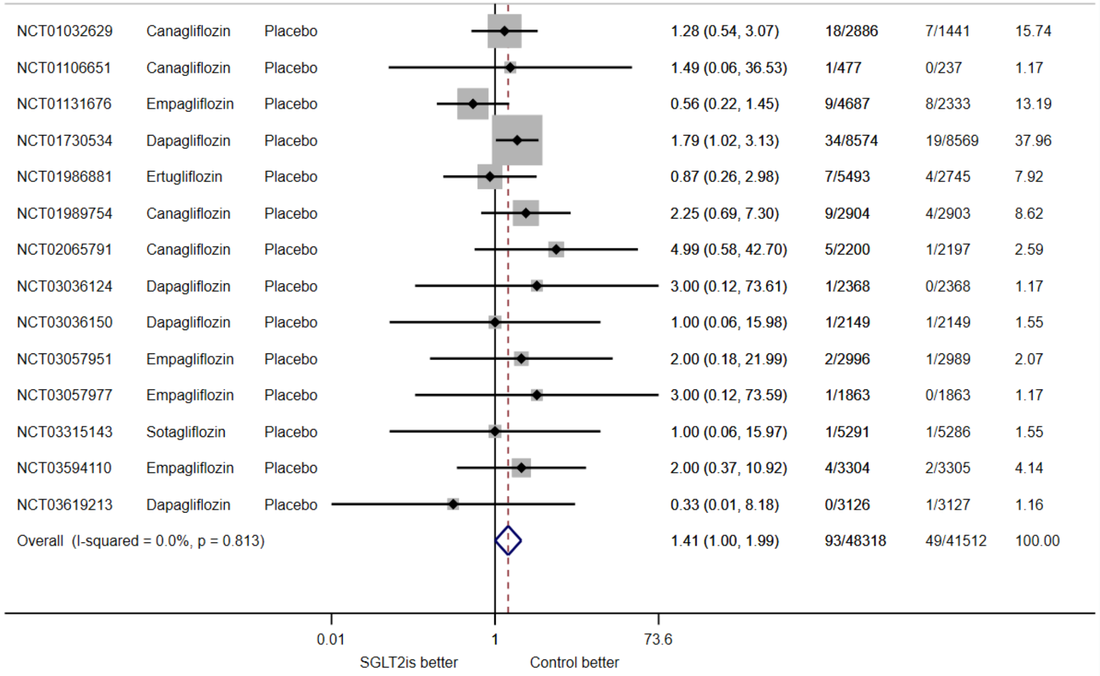

System 25-Vascular Disorders-Figure S42 Meta-analysis of SGLT2is and Peripheral vascular disorder

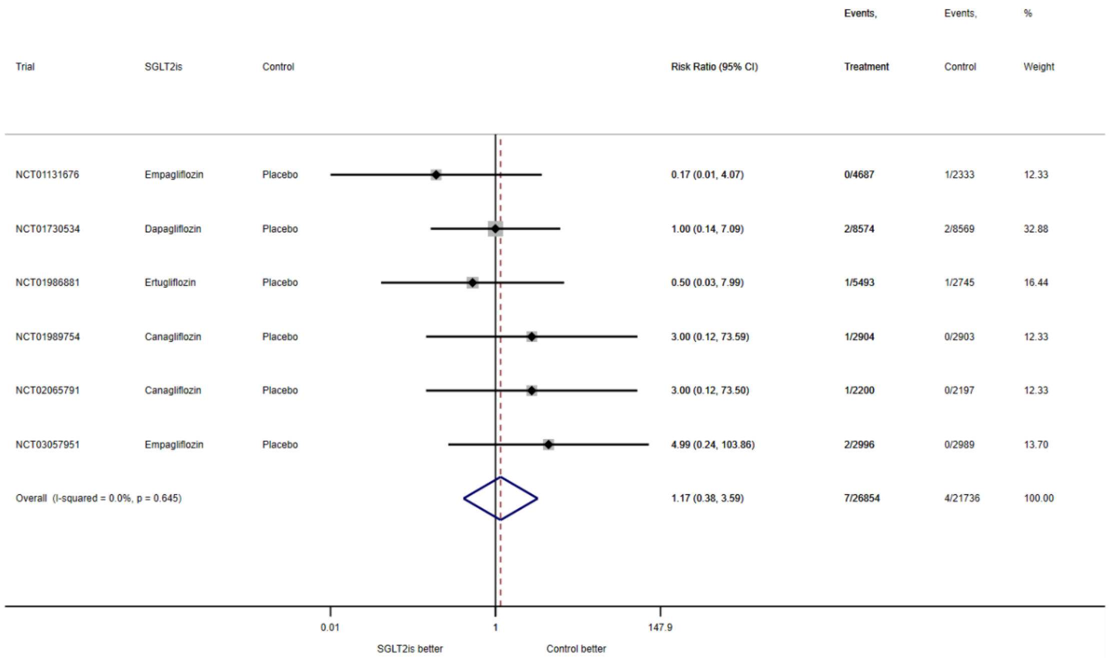

System 25-Vascular Disorders-Figure S43 Meta-analysis of SGLT2is and Peripheral venous disease

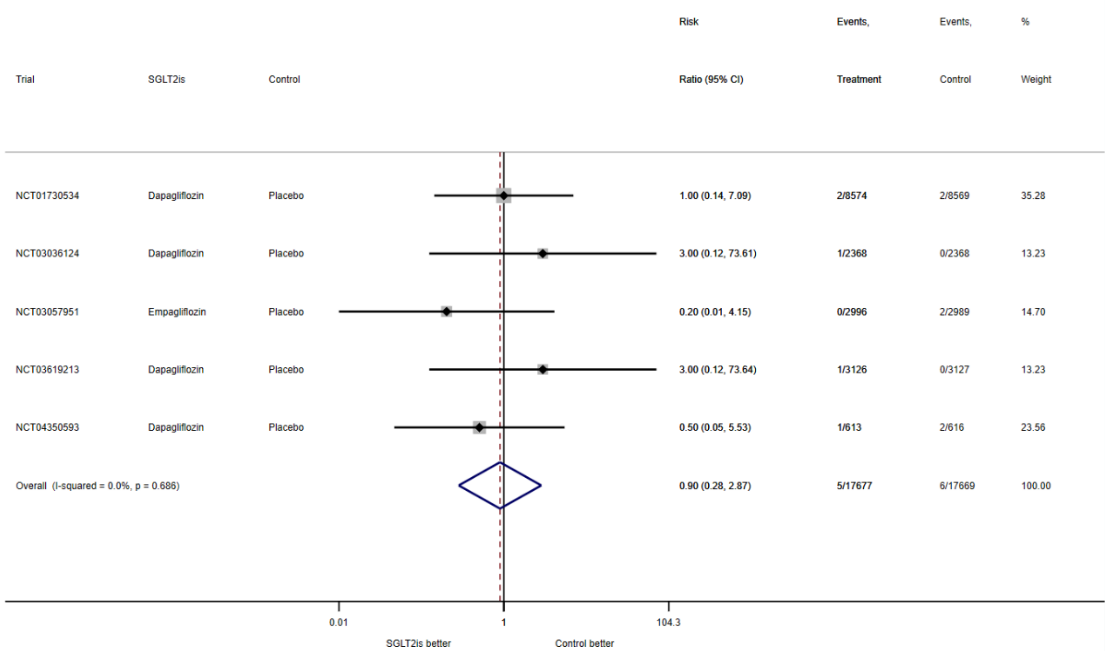

System 25-Vascular Disorders-Figure S44 Meta-analysis of SGLT2is and Shock

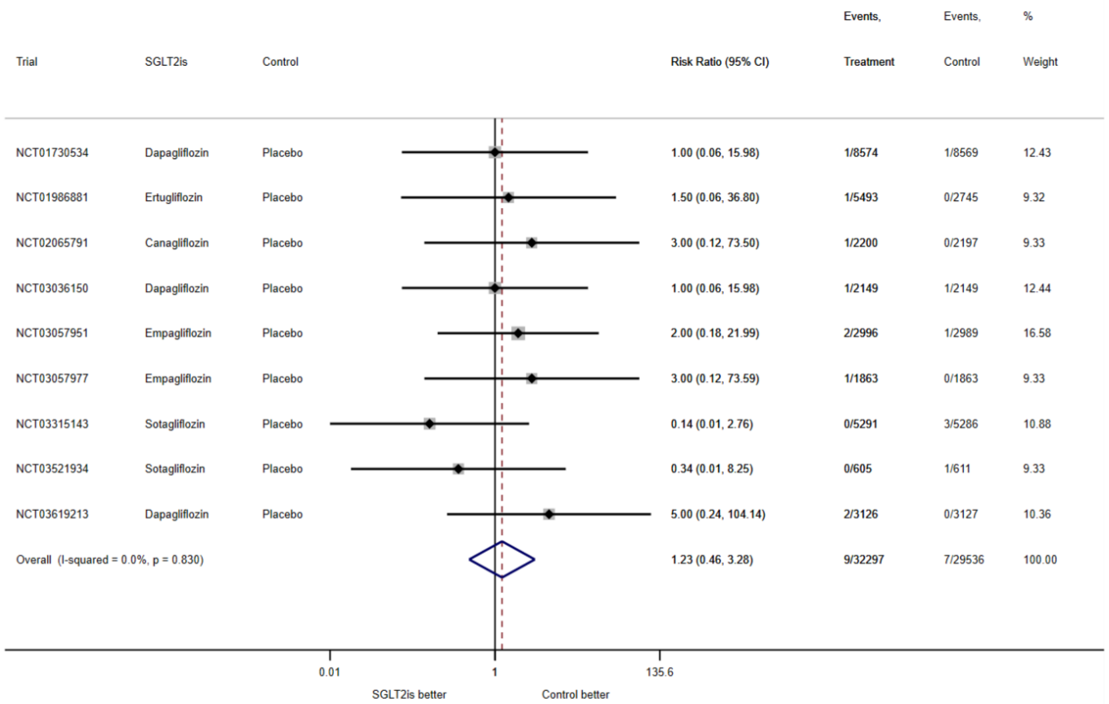

System 25-Vascular Disorders-Figure S45 Meta-analysis of SGLT2is and Shock haemorrhagic

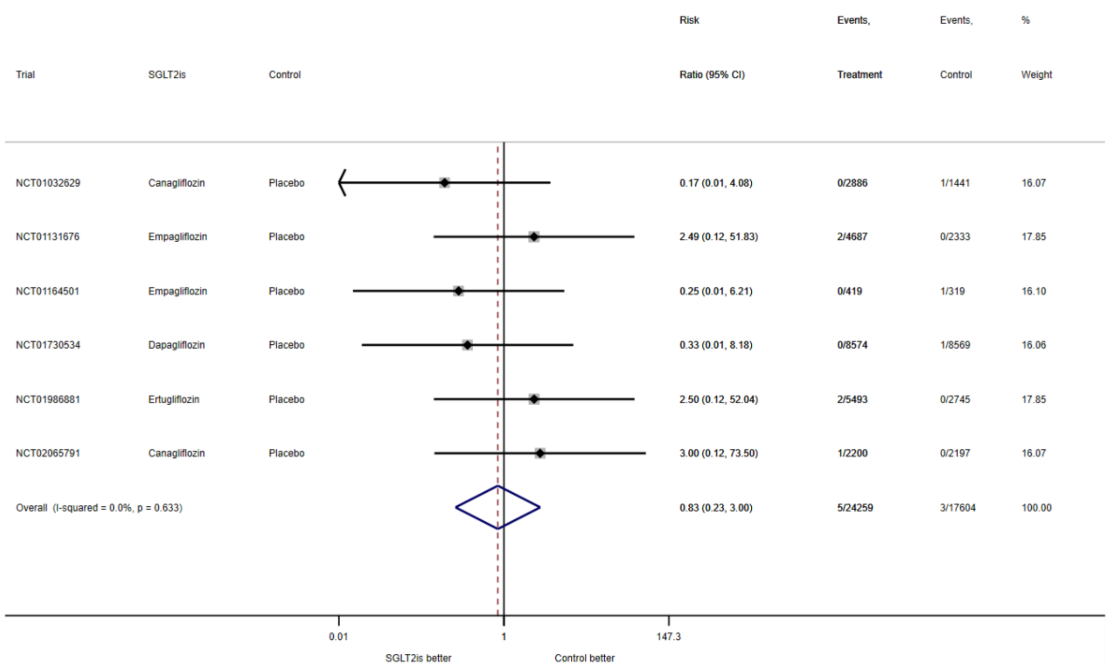

System 25-Vascular Disorders-Figure S46 Meta-analysis of SGLT2is and Subclavian artery stenosis

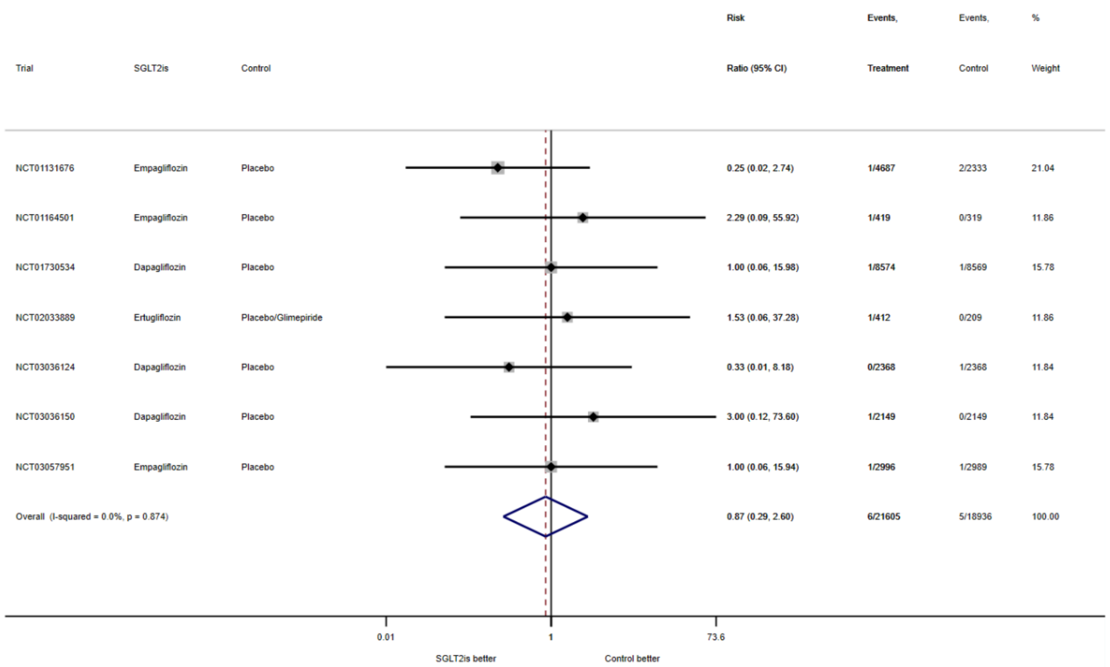

System 25-Vascular Disorders-Figure S47 Meta-analysis of SGLT2is and Thrombophlebitis

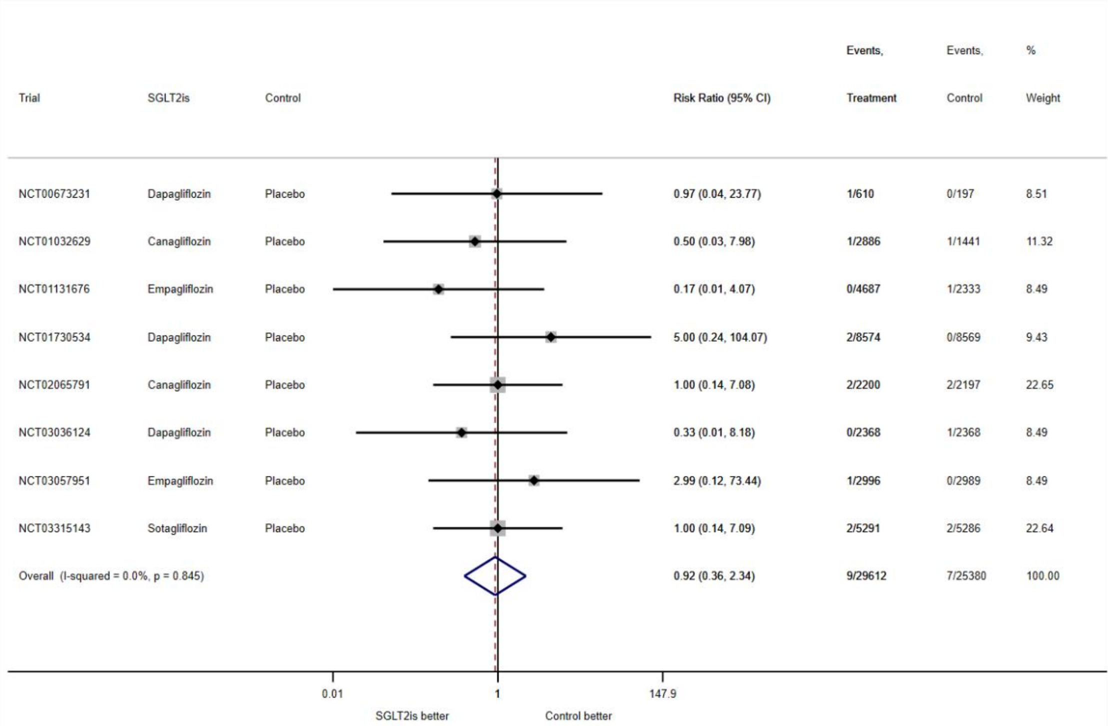

System 25-Vascular Disorders-Figure S48 Meta-analysis of SGLT2is and Thrombophlebitis superficial

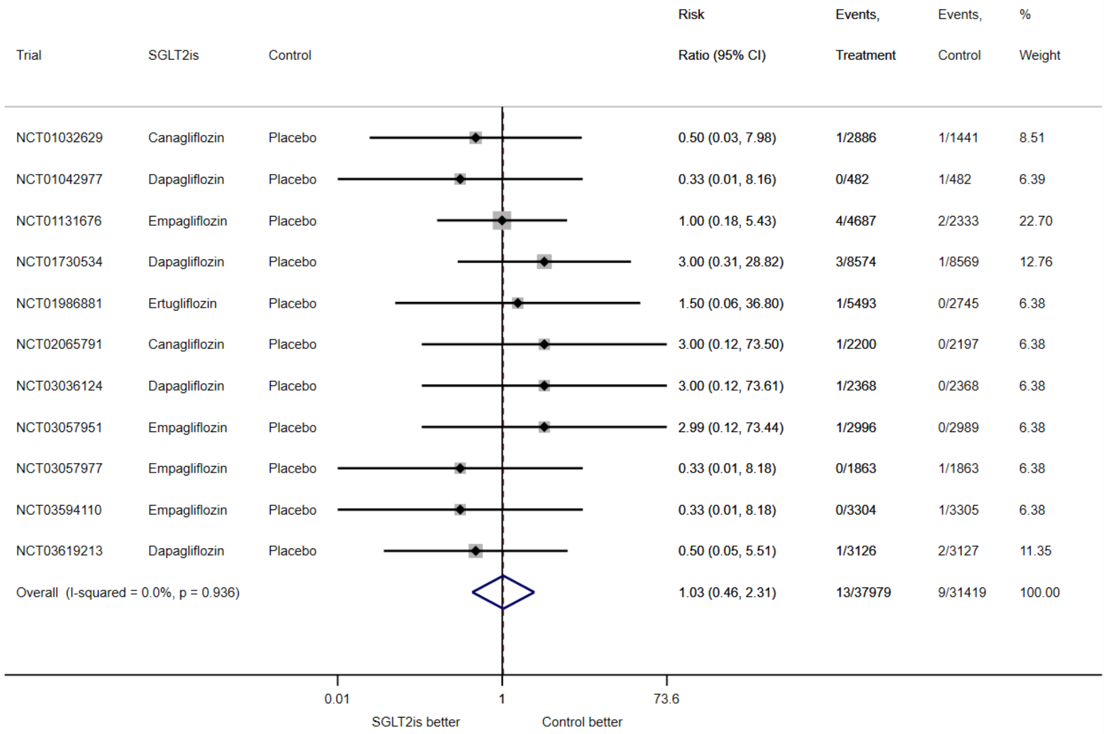

System 25-Vascular Disorders-Figure S49 Meta-analysis of SGLT2is and Thrombosis

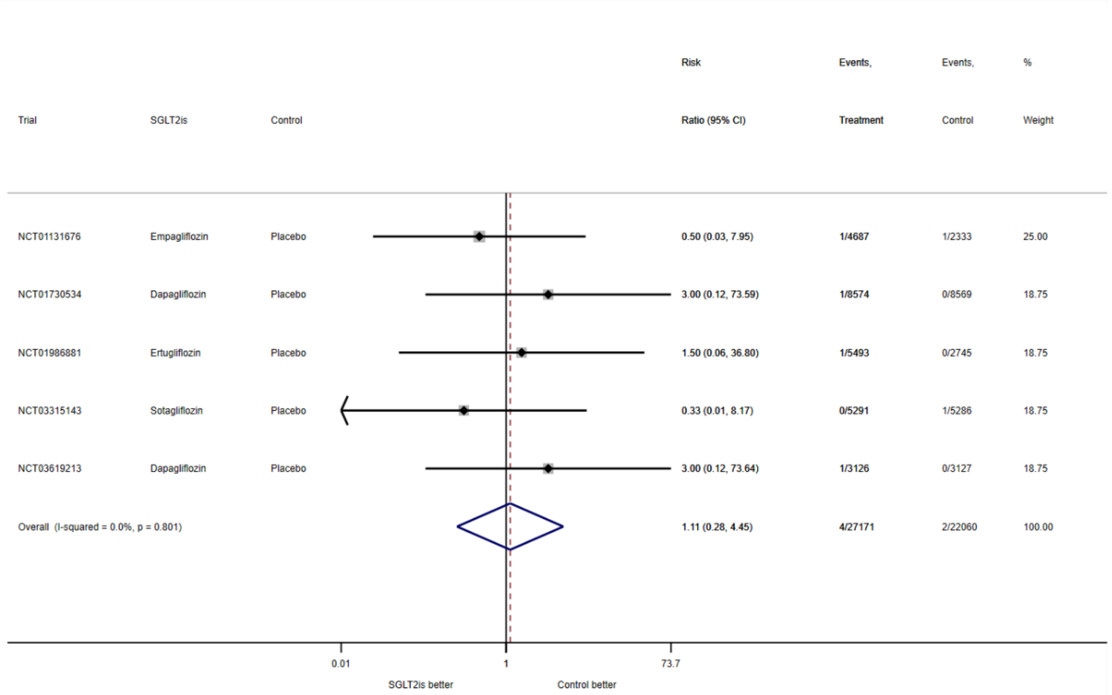

System 25-Vascular Disorders-Figure S50 Meta-analysis of SGLT2is and Varicose ulceration

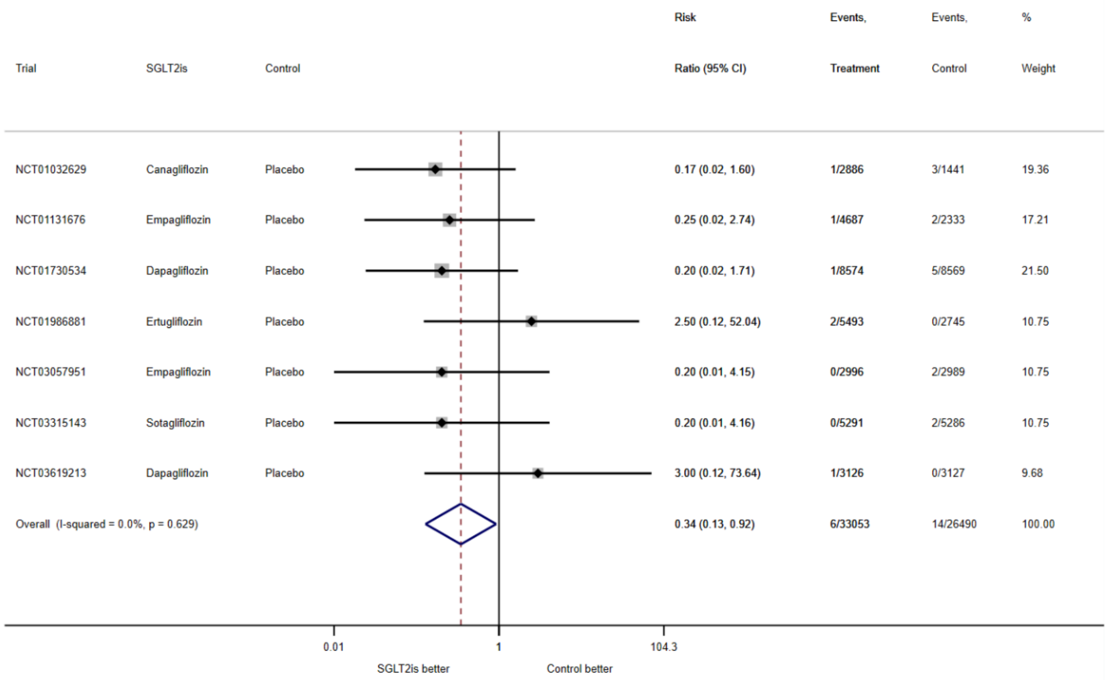

System 25-Vascular Disorders-Figure S51 Meta-analysis of SGLT2is and Varicose vein

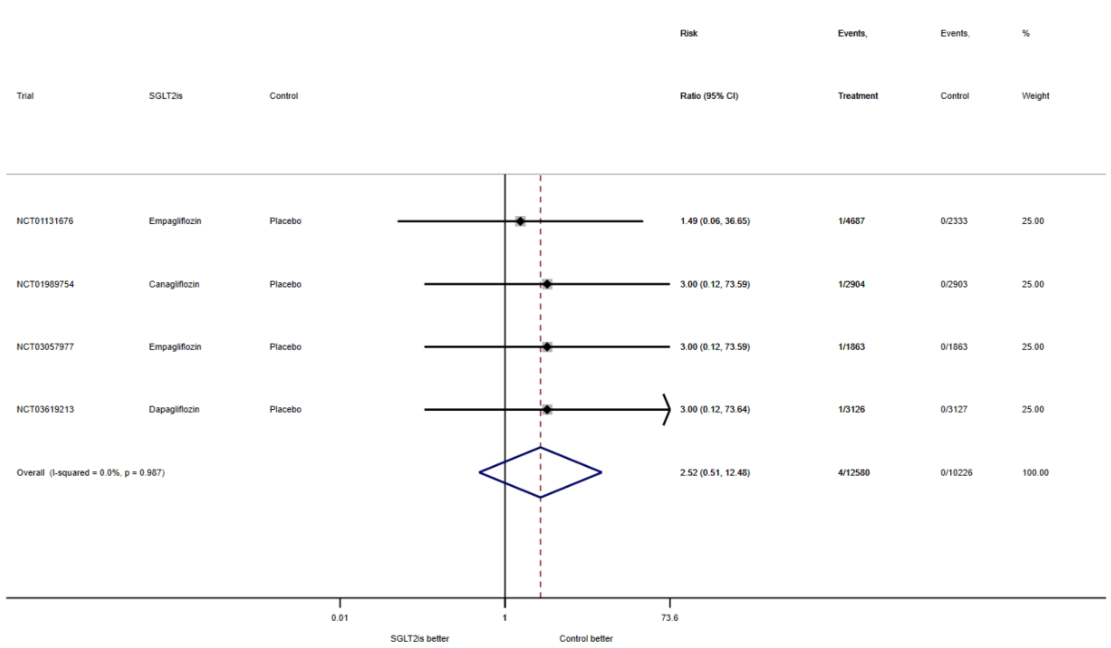

System 25-Vascular Disorders-Figure S52 Meta-analysis of SGLT2is and Vascular insufficiency

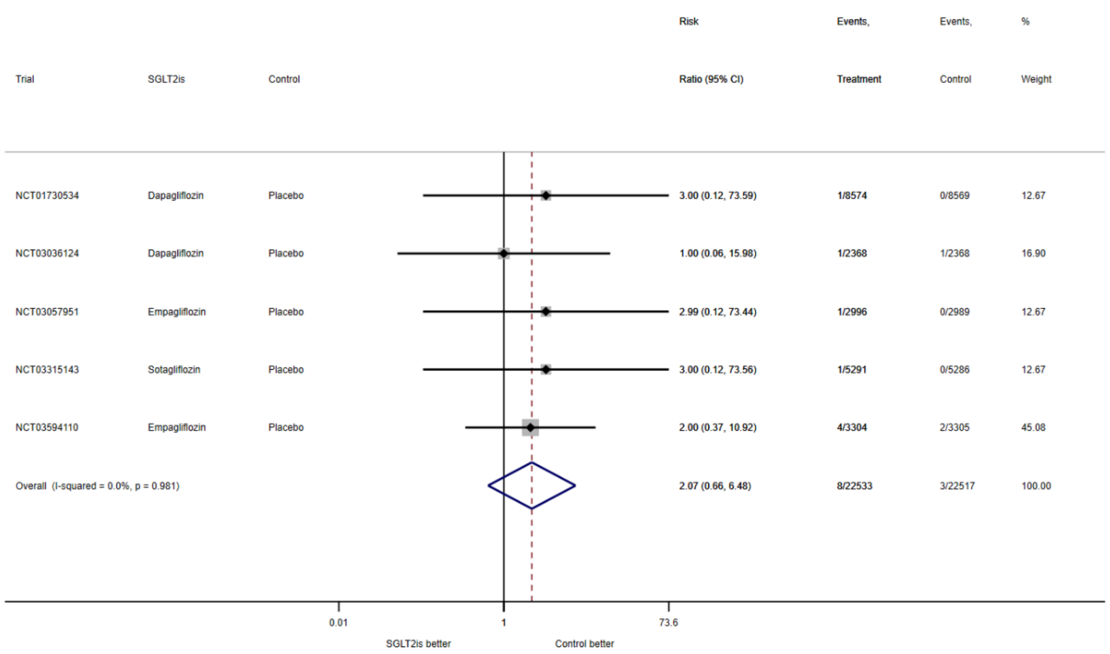

System 25-Vascular Disorders-Figure S53 Meta-analysis of SGLT2is and Vasculitis

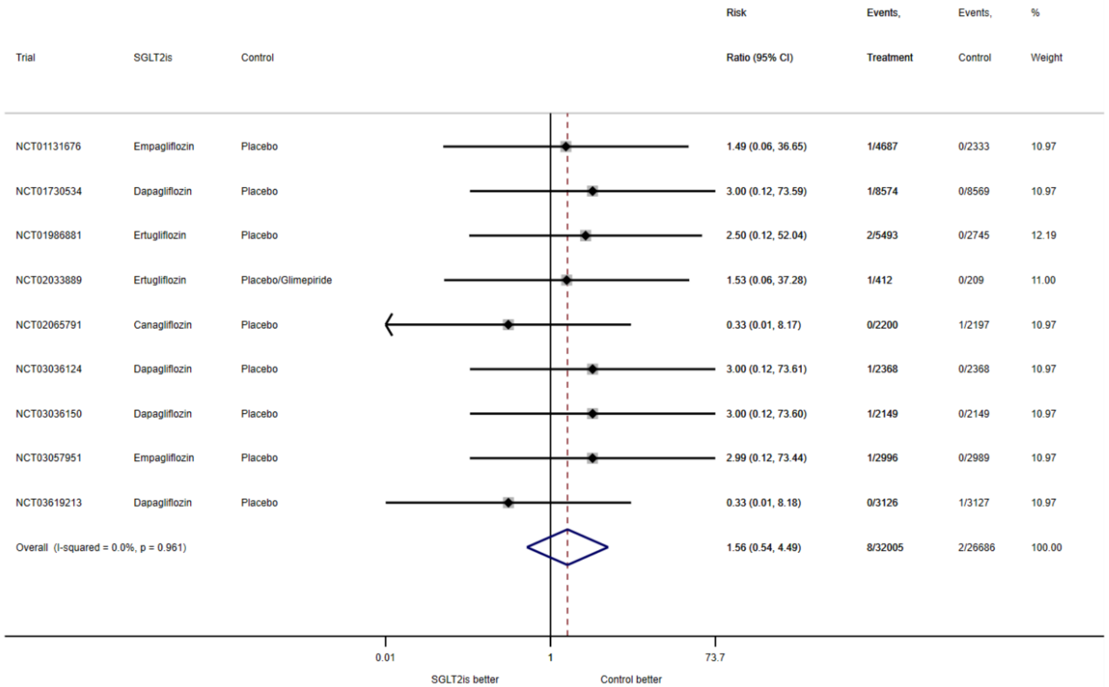

System 25-Vascular Disorders-Figure S54 Meta-analysis of SGLT2is and Venous thrombosis limb
