# Supplementary material for: A comprehensive meta-analysis on safety outcomes reveals the novel potentials of SGLT2is, especially preventing respiratory diseases
Source: Front Endocrinol (Lausanne). 2024 Apr 29;15:1376446. doi: 10.3389/fendo.2024.1376446 (PMC11089104; doi:10.3389/fendo.2024.1376446)
Supplement: Supplementary file 6 [file Table_1.docx]

**Table S1** The characteristics of included trials

| NCT number | Name of trial | First author | Year | Treatment | Control | N (Treatment) | N (Control) | N (Total) | Population | Study duration |
| --- | --- | --- | --- | --- | --- | --- | --- | --- | --- | --- |
| NCT03594110  [7] | EMPA-KIDNEY | Colin Baigent | 2023 | Empagliflozin 10 mg | Placebo | 3304 | 3305 | 6609 | CKD | 2.0 years |
| NCT03619213  [8] | DELIVER | Scott D Solomon | 2022 | Dapagliflozin 10mg | Placebo | 3131 | 3132 | 6263 | HF | 2.3 years |
| NCT03057951  [9] | EMPEROR-Preserved | Stefan D Anker | 2021 | Empagliflozin 10 mg | Placebo | 2996 | 2989 | 5985 | HF | 26.2 months |
| NCT03521934  [10] | SOLOIST-WHF | Deepak L Bhatt | 2021 | Sotagliflozin 400 mg | Placebo | 605 | 611 | 1216 | T2D and HF | 21.2 months |
| NCT03315143  [11] | SCORED | Deepak L Bhatt | 2021 | Sotagliflozin 400 mg | Placebo | 5292 | 5292 | 10584 | T2D and CKD | 28.9 months |
| NCT03242252  [13] | SOTA-CKD3 | David Z I Cherney | 2023 | Sotagliflozin 200 mg, 400 mg | Placebo | 527 | 260 | 787 | T2D and CKD | 60 weeks |
| NCT02033889  [14] | VERTIS MET | Silvina Gallo | 2022 | Ertugliflozin 5 mg, 15 mg | Placebo/Glimepiride | 412 | 209 | 621 | T2D | 104 weeks |
| NCT04157751  [15] | EMPULSE | Adriaan A Voors | 2022 | Empagliflozin 10 mg | Placebo | 260 | 264 | 524 | Acute HF | 90 days |
| NCT02384941  [16] | inTandem1 | John B Buse | 2018 | Sotagliflozin 200 mg, 400 mg | Placebo | 525 | 268 | 793 | T1D | 52 weeks |
| NCT01042977  [17] | - | L A Leiter | 2016 | Dapagliflozin 10 mg | Placebo | 482 | 483 | 965 | T2D and CVD | 24 weeks |
| NCT01031680  [18] | - | William T Cefalu | 2015 | Dapagliflozin 10 mg | Placebo | 460 | 462 | 922 | T2D and CVD | 52 weeks |
| NCT01164501  [19] | EMPA-REG RENAL | Anthony H Barnett | 2014 | Empagliflozin 10 mg, 25 mg | Placebo | 419 | 319 | 738 | T2D and CKD | 52 weeks |
| NCT01106677  [20] | CANTATA-D | F J Lavalle-González | 2013 | Canagliflozin 100 mg, 300 mg | Sitagliptin 100mg | 735 | 366 | 1101 | T2D | 52 weeks |
| NCT01177813  [21] | EMPA-REG MONO | Michael Roden | 2013 | Empagliflozin 10 mg, 25 mg | Sitagliptin or Placebo | 447 | 452 | 899 | T2D | 24 Weeks |
| NCT01106651  [22] | - | James L Januzzi Jr | 2017 | Canagliflozin 100 mg, 300 mg | Placebo | 477 | 237 | 714 | T2D | 104 weeks |
| NCT00528879  [23] | - | Clifford J Bailey | 2010 | Dapagliflozin 2.5 mg, 5.0 mg,, 10 mg | Placebo | 409 | 137 | 546 | T2D | 24 weeks |
| NCT04350593  [24] | DARE-19 | Mikhail N Kosiborod | 2021 | Dapagliflozin 10mg | Placebo | 613 | 616 | 1229 | COVID-19 | 30 days |
| NCT00673231  [25] | - | John P H Wilding | 2012 | Dapagliflozin 2.5 mg, 5.0 mg,, 10 mg | Placebo | 610 | 197 | 807 | T2D | 24 weeks |
| NCT02065791  [26] | CREDENCE | Vlado Perkovic | 2019 | Canagliflozin 100 mg | Placebo | 2200 | 2197 | 4397 | T2D and Nephropathy | 2.62 years |
| NCT01032629  [27] | CANVAS | Bruce Neal | 2017 | Canagliflozin 100 mg, 300 mg | Placebo | 2886 | 1441 | 4327 | T2D | 338 weeks |
| NCT01989754  [27] | CANVAS-R | Bruce Neal | 2017 | Canagliflozin 300 mg | Placebo | 2904 | 2903 | 5807 | T2D | 3 years |
| NCT03057977  [28] | EMPEROR-Reduced | Milton Packer | 2020 | Empagliflozin 10 mg | Placebo | 1863 | 1863 | 3726 | HF | 16 months |
| NCT03036150  [29] | DAPA-CKD | Hiddo J L Heerspink | 2020 | Dapagliflozin 10mg | Placebo | 2149 | 2149 | 4298 | CKD | 2.4 years |
| NCT01986881  [30] | VERTIS CV | Christopher P Cannon | 2020 | Ertugliflozin 5 mg, 15 mg | Placebo | 5493 | 2745 | 8238 | T2D | 3.5 years |
| NCT01131676  [31] | EMPA-REG OUTCOME | Bernard Zinman | 2015 | Empagliflozin 10 mg, 25 mg | Placebo | 4687 | 2333 | 7020 | T2D | 3.1 years |
| NCT03036124  [32] | DAPA-HF | John J V McMurray | 2019 | Dapagliflozin 10mg | Placebo | 2368 | 2368 | 4736 | HF | 18.2 months |
| NCT01730534  [33] | DECLARE-TIMI 58 | Stephen D Wiviott | 2019 | Dapagliflozin 10mg | Placebo | 8574 | 8569 | 17143 | T2D | 4.2 years |

"-" indicates Not Reported. T2D = type 2 diabetes. HF = heart failure. CKD = chronic kidney disease. CVD = cardiovascular disease.
